# The RXFP3 receptor is functionally associated with cellular responses to oxidative stress and DNA damage

**DOI:** 10.18632/aging.102528

**Published:** 2019-12-03

**Authors:** Jaana van Gastel, Hanne Leysen, Paula Santos-Otte, Jhana O. Hendrickx, Abdelkrim Azmi, Bronwen Martin, Stuart Maudsley

**Affiliations:** 1 Receptor Biology Lab, Department of Biomedical Sciences, University of Antwerp, Antwerp, Belgium; 2 Translational Neurobiology Group, Centre for Molecular Neuroscience, VIB, Antwerp, Belgium; 3 Center for Molecular and Cellular Bioengineering (CMCB), Technische Universität Dresden, Dresden, Germany; 4 Faculty of Pharmaceutical, Veterinary and Biomedical Science, University of Antwerp, Antwerp, Belgium

**Keywords:** relaxin family peptide 3 receptor, relaxin 3, GPCR, DNA damage, aging

## Abstract

DNA damage response (DDR) processes, often caused by oxidative stress, are important in aging and -related disorders. We recently showed that G protein-coupled receptor (GPCR) kinase interacting protein 2 (GIT2) plays a key role in both DNA damage and oxidative stress. Multiple tissue analyses in GIT2KO mice demonstrated that GIT2 expression affects the GPCR relaxin family peptide 3 receptor (RXFP3), and is thus a therapeutically-targetable system. RXFP3 and GIT2 play similar roles in metabolic aging processes. Gaining a detailed understanding of the RXFP3-GIT2 functional relationship could aid the development of novel anti-aging therapies. We determined the connection between RXFP3 and GIT2 by investigating the role of RXFP3 in oxidative stress and DDR. Analyzing the effects of oxidizing (H_2_O_2_) and DNA-damaging (camptothecin) stressors on the interacting partners of RXFP3 using Affinity Purification-Mass Spectrometry, we found multiple proteins linked to DDR and cell cycle control. RXFP3 expression increased in response to DNA damage, overexpression, and Relaxin 3-mediated stimulation of RXFP3 reduced phosphorylation of DNA damage marker H2AX, and repair protein BRCA1, moderating DNA damage. Our data suggests an RXFP3-GIT2 system that could regulate cellular degradation after DNA damage, and could be a novel mechanism for mitigating the rate of age-related damage accumulation.

## INTRODUCTION

Disruption of the glucose metabolic system is nearly universal in aging and as this primary metabolic process falters, a negative energy balance occurs, as ATP levels reduce while reactive oxygen species (ROS) levels increase. This simultaneous loss of the capacity to maintain energetic processes in the face of increasing oxidative stress will eventually overwhelm cellular antioxidant capabilities, resulting in oxidative damage of DNA, one of the most well-known and recognized hallmarks of aging [1]. Many age-related disorders that affect our population, such as neurodegeneration, type 2 diabetes mellitus (T2DM) and cardiovascular disorders, are caused by these hallmarks [2].

Stress-induced DNA damage is arguably the most prominent underlying cause of aging and in turn may be one of the primary players in almost all age-related disorders [3]. This hypothesis has been supported by the investigation of accelerated aging disorders, *i.e.* Hutchinson–Gilford progeria syndrome, Ataxia Telangiectasia and Werner syndrome, which have one commonality, they are a direct effect of DNA damage response (DDR) and repair disruption [4–8]. As such, therapeutic amelioration of this stress-induced DNA damage could be very effective for treating age-related disorders. We recently identified the G protein-coupled receptor (GPCR) associated protein, GIT2, as a potential keystone in aging [9]. Further work demonstrated that this receptor scaffolding protein also plays a role in oxidative stress responses [10], and is crucial for integrating several components of the DDR [11]. GIT2 knockout (GIT2KO) mice showed an increased vulnerability to DNA damage [12], displayed symptoms of T2DM [13], showed signs of ‘inflammaging’ [14, 15], and most importantly, showed accelerated aging compared to their wild-type littermates [12]. While this makes GIT2 an interesting target for treating multiple age-related disorders, GIT2 is a scaffolding protein and is therefore difficult to target directly. Typically, drugs are designed to be directed at enzymes, ion channels or receptors. However, as GIT2 is a GPCR interacting protein, it is highly likely that we can identify a receptor that is strongly associated with the GIT2 system. Our recent work has shown that GPCRs possess a potent capacity to control transcriptional and translational efficacies, often via non-G protein signaling activities [16, 17]. This likely contributes to their ability to generate and control the integrity and coherency of cellular signaling pathways, via the coordinated regulation of cascade proteins [18–20]. As there are likely to be strong transcriptional co-relationships between proteins linked via a common signaling function, it is possible that there are dedicated GPCRs that possess a profound link to specific signaling proteins via correlated expression. This ability to link an important target signaling protein to a tractable drug target, such as a GPCR, holds tremendous promise for the generation of intelligently-targeted therapeutics for age-related disorders. In this study, we investigated a receptor that shows an expressional and functional relationship with GIT2, the Relaxin Family Peptide 3 Receptor (RXFP3).

RXFP3 has been implicated in stress response [21], anxiety [22], depression [22, 23], feeding [24–27], arousal [24] and alcohol addiction [28] using RXFP3/RLN3 deficient mouse models. The first indications linking the RXFP3/RLN3 system to stress and metabolic control, was through its presence in the hypothalamic regions involved in the hypothalamic-pituitary-adrenal axis [27, 29–31] and the paraventricular nucleus [26, 27]. The relationship to stress has further been supported by the activation of RLN3 containing neurons in the nucleus incertus after administration of corticotropin releasing factor. The association to anxiety and depression was discovered as RLN3 expressing neurons also express inhibitory serotonin type 1A (5-HT_1A_) receptors, suggesting functional interactions between these two systems [32]. There is currently emerging evidence linking anxiety to accelerated aging, as several accelerated aging mouse models display anxiety [12, 13]. In addition, we have previously shown that RXFP3 expression is significantly affected when an aging-associated alteration occurs in the affect-modulating dopaminergic functionality in mice [33]. Thus, RXFP3 may be associated with controlling aging-related functions in addition to its anxiolytic and anti-depressant effects. Furthermore, RXFP3 may represent an important neurochemical markers of depression in Alzheimer's disease (AD), where Lee et al. demonstrated an increase in immunoreactivity in depressed AD patients [22]. Given our previous work concerning the role of GIT2 in the regulation of the aging process, the determination of a therapeutically targetable GIT2-RXFP3 synergistic signaling system may provide a basis for the design of novel GIT2-RXFP3 based therapeutics for the treatment of aging-related disorders.

## RESULTS

### Coordinated mRNA and protein expression profiles between GIT2 and RXFP3

**Figure 1:**
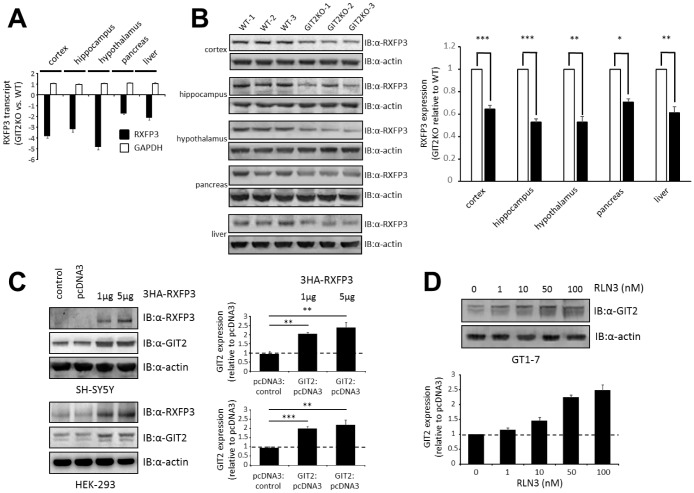
**Discovery of RXFP3 as a possible controller for GIT2, identified through an expressional relationship.** (**A**) Using transcriptome profiling, we investigated the expression levels of RXFP3 in GIT2-knock out (GIT2KO) mice (n=4). Both in the central nervous system (CNS) (cortex, hippocampus, and hypothalamus) and in peripheral tissues (pancreas, and liver) RXFP3 expression level was decreased compared to Wild-type (WT) littermates (n=4), GAPDH expression was shown to be stable as a control. (**B**) These results were replicated using western blotting, actin loading control was used. (**C**) Through transient transfection, RXFP3 was over expressed in SH-SY5Y and HEK293 cells at 1 and 2 μg, as control cells were also overexpressed with an empty vector (pcDNA3). Actin was used as a loading control (n=3). A specific expression increase for GIT2 was seen after overexpression of RXFP3 in both cell types. (**D**) Stimulation of GT1-7 cells using the endogenous ligand Relaxin 3 (RLN3), showed a dose dependent increase in GIT2 expression, with increasing levels of RLN3 (n=3). Data represent the means ± SEM (standard error of the mean). Statistical analyses (Student’s t-test) were performed using GraphPad Prism version 7.0 (GraphPad Software, San Diego, CA, USA). Significance level is indicated in each figure as *p ≤ 0.05; **p ≤ 0.01; ***p ≤ 0.001.

In this study, multiple tissues, from the central nervous system (cortex, hippocampus and hypothalamus) and the periphery (pancreas and liver) were collected from male GIT2KO mice for mRNA expression profiling (Figure 1A), where we found a strong connection between GIT2 and RXFP3 expression, replicating what we have previously seen for murine GIT2 heterozygous KO hypothalamic extracts [34]. To validate these mRNA expression findings, we performed western blots to assess RXFP3 protein expression patterns in multiple GIT2KO mice (Figure 1B). Again, we found that in response to a diminution of GIT2 expression, there was a significant decrease in the levels of RXFP3 in all the assessed tissues. Additionally, upon introduction of a cDNA clone for human RXFP3 to either human neuronal SH-SY5Y or classical HEK293 cells, we found a significant increase in the expression of human GIT2 (Figure 1C).

Previously, we showed that hypothalamic functionality was altered in GIT2KO mice [13]. Thus, we investigated whether functional stimulation of endogenous RXFP3 at endogenous expression levels in murine hypothalamic neuronal GT1-7 cells with its cognate ligand relaxin 3 (RLN3, 1-100 nM, 6hrs of stimulation) would affect GIT2 expression. We found a significant dose-dependent elevation of GIT2 expression (Figure 1D). Hence, both in response to enhanced constitutive receptor activity (induced by augmented ectopic expression – Figure 1C) and ligand stimulation (Figure 1D), the active RXFP3 state seems to be functionally associated with GIT2 expression levels. In a previous study, we showed that GIT2 expression levels are associated with oxidative stress, diabetic pathologies, advancing age and DNA damage [9–11, 13]. These data suggest that the GIT2-RXFP3 relationship may represent a molecular axis important for regulation of age-related damage. Thus, we next investigated further functional links between these two proteins with relation to oxidative stress and DNA damage.

### RXFP3 constitutive activity regulates the expression of DNA damage response proteins

Recent research has demonstrated that in addition to short-term intermediary cell metabolism events mediated by G protein activation, GPCRs such as RXFP3 can regulate the expression profiles of multiple downstream signaling proteins. This occurs via the creation of more stable signaling entities, such as intricate G protein-independent multi-protein ‘receptorsome’ complexes [17, 35]. The human (and murine) RXFP3 is a relatively unique receptor with respect to its basal activity status. It bears a pro-activating natural mutation in its ultra-conserved Asp-Arg-Tyr (DRY) amino acid triple motif, an Asp to Thr alteration, found at the juxtamembrane region of the second intracellular loop (Supplementary Figure 1A, 1B). This natural mutation increases its ligand independent activity [36]. Therefore, RXFP3 likely possesses a range of diverse signaling functions in the absence of its cognate ligand.

**Figure 2:**
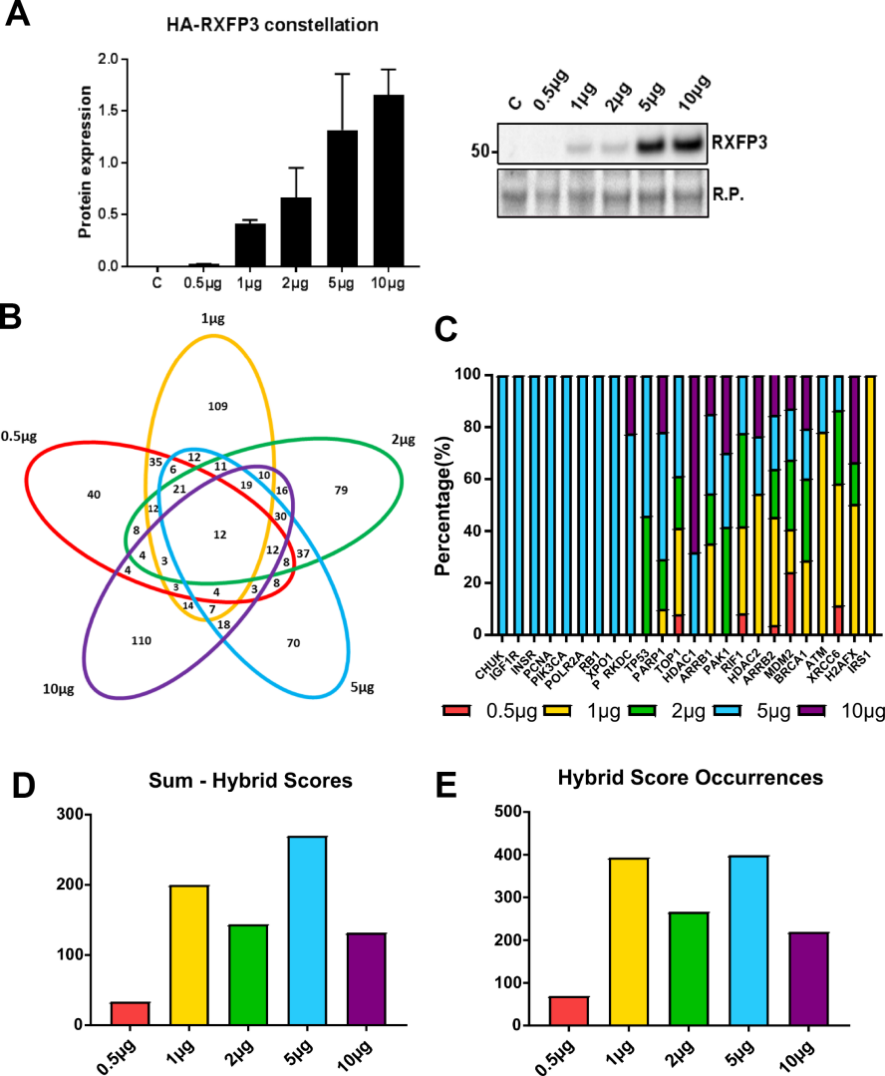
**5 μg of RXFP3 overexpression indicates a role in the DNA damage response.** (**A**) Western blot validation of the differential overexpression curve for RXFP3-HA (n=3). We see a clear increase of RXFP3-HA signal with an increased level of transfections. (**B**) We then selected the proteins unique to each overexpression level using a InteractiVenn for further investigation, obtaining the following percentages of uniquely-regulated proteins: 0.5 μg RXFP3 21.8% unique; 1 μg RXFP3 – 37.2% unique; 2 μg RXFP3 26.6% unique; 5 μg RXFP3 25.2% unique; 10 μg RXFP3 40.8% unique. (**C**) Applying protein-protein interaction (PPI) pattern analysis through Enrichr, we were able to show a strong representation for DNA damage repair and energy metabolism-related proteins across the different RXFP3 expression ranges. We also observed a strongest PPI dataset enrichment for DNA damage response proteins for the 5 μg RXFP3 expression level. (**D**–**E**) We next calculated the number of input dataset proteins associated with the target PPI database protein multiplied by the negative log_10_ of the enrichment probability, or hybrid score. We see that for the (**D**) sum and the (**E**) total sum of hybrid score occurrences, the enrichment for DDR-associated factors was shown to be most profound for the 5 μg RXFP3 level.

To assess these functions, likely generated by an ensemble of stably reinforced receptorsome structures, we used a stepwise ectopic expression process for generating the broadest possible range of ‘active’ receptorsome complexes, which we termed a ‘*constellation curve*’ [37–39]. Without an unprecedented knowledge of receptorsome composition, or range of sub-state-selective or biased ligands, the ability to selectively induce distinct-signaling receptorsomes can only be achieved using such an expression-level variation protocol. By increasing expression levels, the range of receptorsomes will likely increase the diversity of distinct RXFP3 receptorsomes incrementally, revealing expression-specific function actions, until a saturation level is reached. As a gestalt readout of these distinct receptor forms, we employed a proteomic screening assessment of cellular alterations in response to the different RXFP3 receptorsomes, analogous (although less detailed) to a perturbagen ‘constellation’ process with the GIT2 scaffolding protein to assess which signaling pathways it was associated with [11]. In this current study, we performed a ‘constellation’ experiment in HEK293 cells, where we ectopically expressed an ascending level of an N-terminally 3xHA-tagged human RXFP3. Selective western-blot analyses specific for HA epitope tag (RXFP3) confirmed the effective constellation expression variance of the human RXFP3 receptor clone (Figure 2A). Using both anti-native RXFP3 antibody measurements and our quantitative mass spectrometric data we found that even a higher expression level of RXFP3 (5 μg) only engendered a relatively modest increase in total cellular RXFP3 content (1.5-2-fold increase). It is likely that such modest alterations are a) relatively within normal physiological expression ranges and b) more akin to the potentially elevated levels of RXFP3 that may be engineered by the cells after protracted periods of stressor exposure as is likely in the aging process.

We next assessed, in an unbiased manner, the proteomic ‘constellation’ perturbagen response to these ascending expression levels of RXFP3. Protein extracts were investigated as a multiplex using quantitative proteomics through iTRAQ labelling, with each RXFP3 expression level compared ratiometrically to the proteomic response effects to empty vector (E.V.) ectopic expression (Supplementary Table 1). We found that the introduction of various expression levels of RXFP3 (Figure 2A) caused significant and selective alterations in protein expression across the whole RXFP3 level range. The number of significantly altered proteins (compared to empty vector (E.V) transfected controls) responding to the specific RXFP3 expression levels were the following: 0.5 μg RXFP3 – 183; 1 μg RXFP3 – 293; 2 μg RXFP3 297; 5 μg RXFP3 – 278; 10 μg RXFP3 – 269. We next separated the common and distinctive RXFP3 perturbagen responsive proteins using InteractiVenn (Figure 2B). For each expression level, the percentage of uniquely-regulated proteins was relatively similar, *i.e.* 0.5 μg RXFP3 21.8% unique; 1 μg RXFP3 – 37.2% unique; 2 μg RXFP3 26.6% unique; 5 μg RXFP3 25.2% unique; 10 μg RXFP3 40.8% unique (Figure 2B). In contrast to these unique expression events, 12 proteins were significantly altered across all five RXFP3 expression levels (Supplementary Figure 3: PRR14L, SCYL1, CCDC9, NEK7, HIST1H3A, ATPIF1, CDCA2, PAGR1, POTEKP, MTMR1, ZCCHC3, MEPCE). Many of these commonly regulated proteins are known to be associated with energy balance regulation (PRR14L [40]), inflammaging (NEK7 [41]; ATPIF1 [42]), aging associated DNA damage (SCYL1 [43]; CCDC9 [44]; HIST1H3A [45]; ATPIF1 [46]; PAGR1 [47]; ZCCHC3 [48]) or cell senescence (CDCA2 [49]). As such it appears that in addition to expression level effects, a core functionality of the RXFP3 was also evident, which was tightly linked to age-related pathologies.

Given the impact on protein complex formation and the regulation of cellular signaling paradigms, we next analyzed, using protein-protein interaction (PPI) pattern analysis, each distinct RXFP3 expression level dataset using Enrichr (Supplementary Figure 2A) [50], to discover the most DDR-related RXFP3 overexpression level. We observed a strong representation of multiple DNA damage repair and energy metabolism-related proteins across the RXFP3 expression ranges used (Figure 2C; Supplementary Tables 2–6). The strongest PPI dataset enrichment for DDR proteins such as PRKDC, p53, PARP1 and TOP1 was observed for proteins influenced by the 5 μg RXFP3 expression level (Figure 2C). This was also indicated by calculating a hybrid score, *i.e.* the number of input dataset proteins associating with the target PPI database protein multiplied by the negative log_10_ of the enrichment probability, evidenced by the greater numbers of DDR-associated PPI proteins found (Figure 2D) as well as the total sum of the hybrid scores for DDR-associated PPI proteins associated with the different RXFP3 protein datasets (Figure 2E).

**Figure 3:**
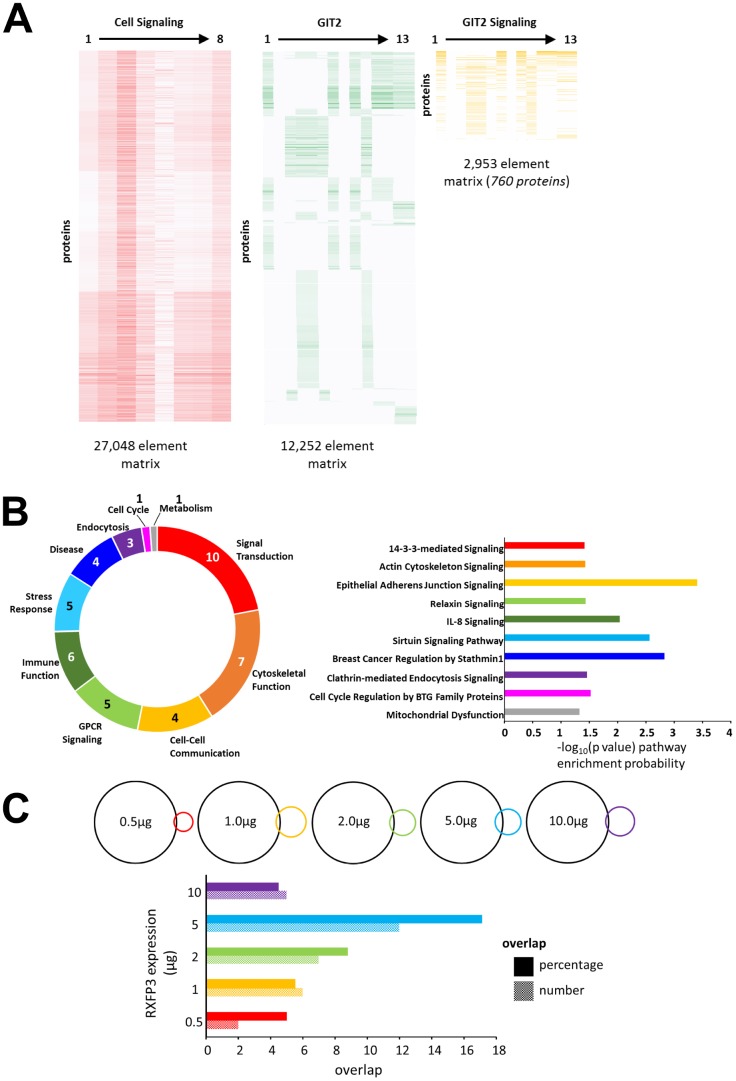
**RXFP3 constellation comparison to a theoretical GIT2-Cell signaling dataset.** (**A**) To further investigate the role for RXFP3 as a controller for GIT2, we created a GIT2-specific signaling set, comprised out of 760 proteins, by investigating the intersection dataset between ‘Cellular Signaling’ and ‘GIT2’ associated text matrices. (**B**) Analysis of this theoretical GIT2-signaling set using canonical signaling pathway analysis shows a strong recollection of the known GIT2 signaling capacities. But interestingly some other novel functionalities were also apparent such as Sirtuin Signaling and Relaxin Signaling. (**C**) Lastly, we investigated which overexpression level of the RXFP3 constellation showed a potential GIT2-associated bias. Calculating both the numerical and percentage overlap, we found that the strongest intersection with the theoretical GIT2-signaling set was clear with the 5 μg RXFP3 level.

In a previous study, we demonstrated that when investigating novel GPCR-based signaling paradigms it is possible to assess selective signaling specificity using comparisons of empirical data with mass analysis-based ‘theoretical’ signaling datasets [17]. To this end, we created a GIT2-specific signaling set in an analogous manner to our previously created arrestin-signaling datasets [17], by isolating the intersection dataset between ‘Cellular Signaling’ and ‘GIT2’ associated text matrices (Figure 3A: Supplementary Table 7–9). This concatenated Latent Semantic Analysis (LSA)-based dataset comprised 760 GIT2-Signaling proteins. Initially we found that with canonical signaling pathway analysis (Supplementary Table 10) of this ‘theoretical’ dataset, a strong signaling pathway phenotype reminiscent of known GIT2 signaling capacities was apparent, *i.e.* cytoskeletal control, GPCR signaling, immune function, stress responses as well as cell cycle and metabolism regulation [13, 51, 52] (Figure 3B). This GIT2-reminiscent mechanistic interpretation of our ‘theoretical’ GIT2 signaling dataset, strongly reinforces our unbiased informatics approach to aid novel signaling paradigm investigation. Furthermore, upon inspection of some exemplars of the significantly enriched pathways (Ingenuity Pathway Analysis-based) within this theoretical dataset, we not only demonstrate specific GIT2-associated activities, *e.g. ‘Actin Cytoskeleton Signaling*’ [53], ‘*Breast Cancer Regulation by Stathmin 1*’ [11] and ‘*Mitochondrial dysfunction*’ [13], but also novel functions, *e.g.* ‘*Sirtuin Signaling*’ and ‘*Relaxin Signaling’* (Figure 3B). We then compared the overlap between this theoretical GIT2 dataset and the diverse RXFP3 expression levels, to help identify any potential biases towards GIT2-associated signaling functionality (Supplementary Figure 2B). Calculating both the numerical and percentage overlap of distinct RXFP3 expression range, we found that the strongest intersection with the theoretical GIT2-signaling set occurred with the 5 μg RXFP3 expression level (Figure 3C).

**Figure 4:**
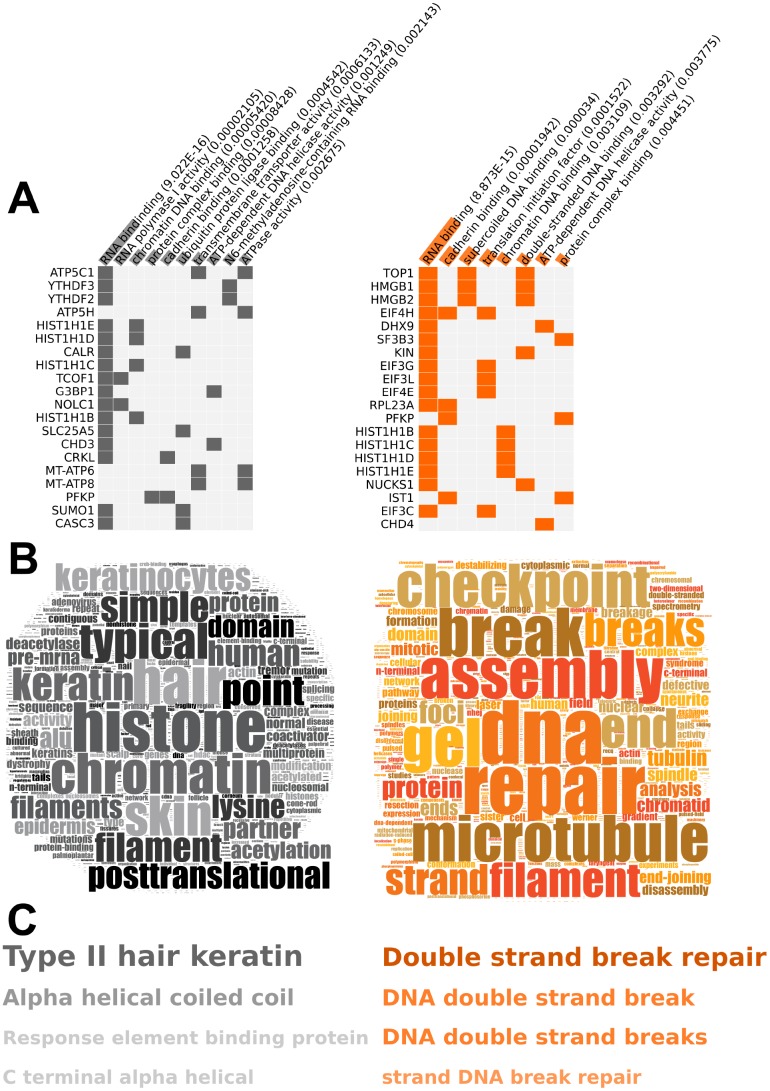
**RXFP3 constellation, differential overexpression of RXFP3 indicates a role in DNA damage response**. (**A**–**C**) The results from the bioinformatic analysis of 0.5 μg RXFP3-HA (Left, grey), and 5 μg RXFP3-HA overexpression (Right, orange) using (**B**) Gene Ontology through Enrichr [50]; (**C**) Wordcloud generation and (**D**) phrase frequency counting (WriteWords) of the words and noun-phrases extracted from Textrous! [54]. Here we see that a different overexpression level of RXFP3, indicates a different role of RXFP3, where with 0.5 μg RXFP3 we see a role in translation, and chromatin structure, while for 5 μg RXFP3 overexpression a role for DNA damage response and repair.

From our unbiased multidimensional ‘constellation’ analysis it was clear that a strong phenotype related to the protection of nucleic acids was observed. To further investigate this, we then focused on two levels of RXFP3 expression – one relatively innocuous 0.5 μg (allowing ectopic expression to occur without the introduction of a profound cellular phenotype), and the second being the 5 μg level that was strongly associated with DNA protection activity. Protein expression patterns significantly regulated in response to 0.5 μg and 5 μg of RXFP3 overexpression were co-analyzed using Gene Ontology (GO) term enrichment analysis (Supplementary Figure 2C), demonstrating the presence of common (RNA binding, cadherin binding) as well as distinct (transmembrane transport activity – 0.5 μg; Double stranded DNA binding – 5 μg) significantly enriched GO term groups (Figure 4A). The strongest clustering proteins at the 0.5 μg RXFP3 level were associated with mitochondrial activity (ATP5C1) and RNA methylation (YTHDF3, YTHDF2) – while in response to the 5 μg RXFP3 expression level, strongly clustering proteins were associated with DNA damage/repair (TOP1), stress responsiveness (HMGB1) and cell senescence (HMGB2). Using the natural language processing informatic platform Textrous! [54] (Supplementary Figure 2D), we also found a strong functional divergence between these two datasets, *i.e*. the lower level of expression was associated with histone and microtubular function while the 5 μg RXFP3 expression level again was strongly associated with DNA repair activities. The WriteWords phrase frequency counter was used (Supplementary Figure 2D) to extract the top 4 highest frequency phrases from Textrous! visualized in Figure 4B, 4C. Given these results and our PPI enrichment analysis results (Figure 2) we next decided to further investigate the physical interactome of the human RXFP3 receptor in control and aging stress-associated conditions.

### Aging-related cellular stress alters the physical RXFP3 interactome

As complex signal transduction occurs through the dynamic modulation of PPIs [55], especially with respect to receptors [37, 56, 57], we next analyzed the nature of the proteins which physically associate with RXFP3, and how these interactions are modulated in response to oxidative stress (peroxide-induced) and DNA damage (CPT-induced). Using western blotting and confocal microscopy we observed the presence of DNA damage foci in the nucleus, specifically demonstrated by γ-H2AX as well as the increase of phospho-ATM – both these factors indicate DNA damage and DDR (Supplementary Figure 4A) [58, 59].

**Figure 5:**
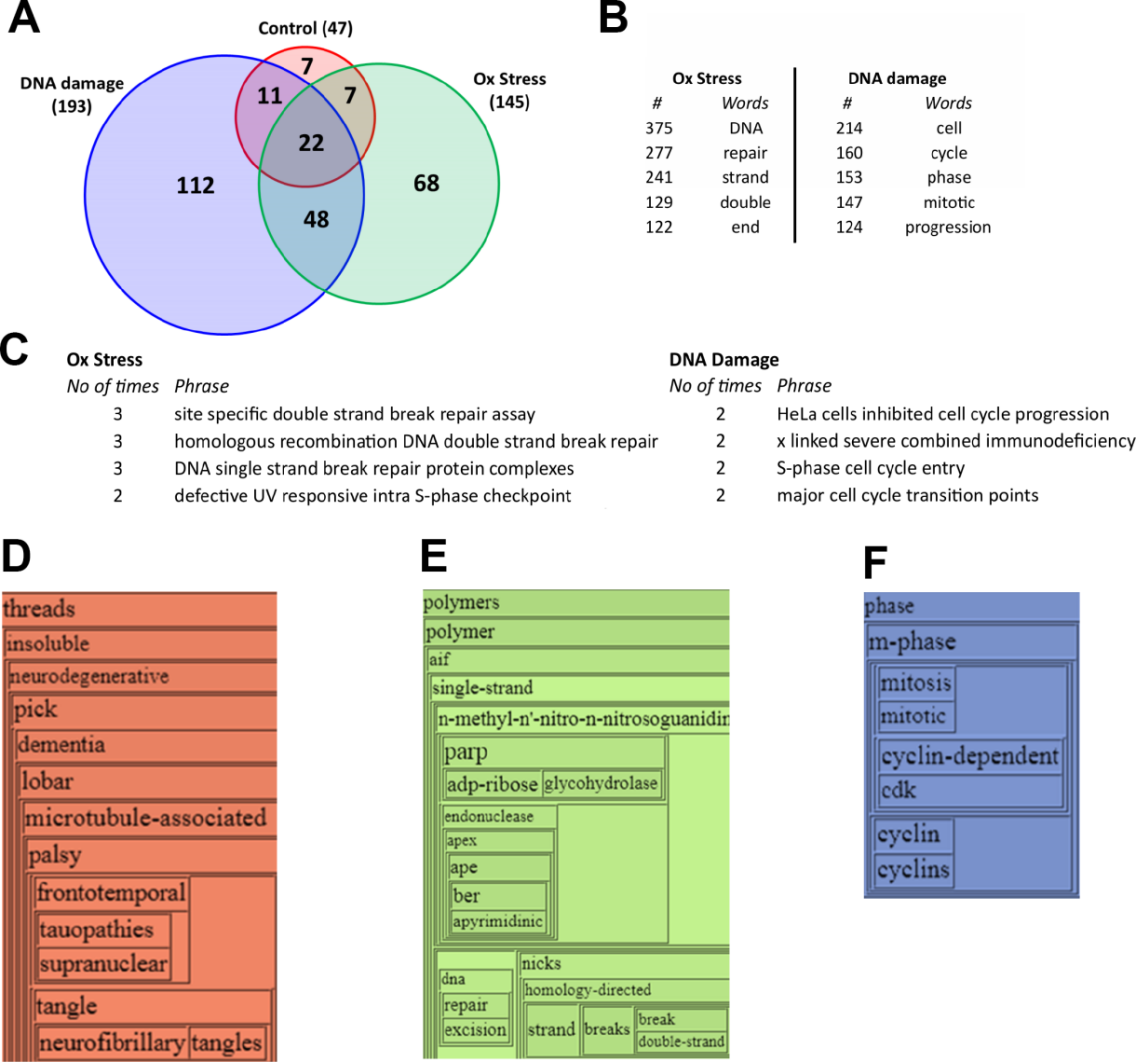
**Initial analysis of the unique RXFP3 interactomes under stress conditions indicates a role in DNA damage response and cell cycle control.** (**A**) VennPlex analysis of the different RXFP3 interactomes, RXFP3 without stress (control; orange) (n=3), RXFP3 in response to Oxidative stress using 100 nM hydrogen peroxide for 90 minutes (Ox Stress; Green) (n=3), and DNA damage using 1 μM of camptothecin for 3 hours (DNA damage; blue) (n=3). For further analysis, the proteins unique to Oxidative stress and DNA damage were used for further analysis using *Textrous!* (textrous.irp.nia.nih.gov), which employs latent semantic indexing to achieve an easy and unbiased appreciation of our data, supplying a list of words and noun-phrases related to our dataset. Further analysis of these wordlists using WriteWords (http://www.writewords.org.uk), enabled us to count the most prominently present (**B**) words, and (**C**) noun-phrases associated to the specific datasets, from this it becomes clear that RXFP3 interacts with proteins involved in DNA damage response (Oxidative Stress) and Cell cycle control (DNA damage) in response to stress. Next, we extracted the hierarchical wordcloud from Textrous!, where we see the specific words for (**D**) Control, indicating a role in control of neurodegenerative disorders such as Alzheimer’s disease and frontotemporal dementia (FTD), (**E**) Oxidative Stress, again indicating an association with DNA damage repair; and (**F**) DNA damage, indicating a connection to cell cycle control.

Employing a cellular SILAC (Stable Isotope Labeling with Amino Acids in Cell Culture)-based affinity purification-mass spectrometry (AP-MS) approach, we were able to identify and quantify the potential interacting proteins that may represent components of the RXFP3 interactome. Three AP-MS RXFP3 interactomes were extracted, *i)* control interactome ‘*Control*’: E.V. *vs.* RXFP3 no stress (n=3); *ii)* oxidative stress interactome ‘*Ox Stress’*: E.V. *vs.* RXFP3 stressed with 100 nM H_2_O_2_ for 90 minutes (n=3); and *iii)* DNA damage interactome ‘*DNA damage*’: E.V. *vs.* RXFP3 stressed with 1 μM of CPT for 3 hours (n=3). We then compared the different protein compositions of these interactomes and investigated the proteins unique to each condition (Figure 5A, proteins are listed in Supplementary Table 2). In control conditions we reliably observed 47 distinct RXFP3 interacting partner proteins (Figure 5A – Supplementary Table 11). Among these control condition interacting proteins, we found several that were strongly associated with DNA stability management, *e.g*. EIF4A1 [60], RPS27A [61], MAP4 [62] and PHB [63]. Upon the introduction of the two age-related perturbagens, H_2_O_2_ and CPT, we found a profound increase – potentially due to a functional stabilization of the interactome complexes [64] – in the size of the stress-associated RXFP3 interactomes (Figure 5A). In response to peroxide exposure, multiple factors linking oxidative stress to DNA damage management and senescence were found to physically associate with RXFP3 receptorsomes including PRDX6 [65, 66], PCNA [67, 68], FUS [69], RBMX [70], PARP1 [71], PRDX1 [72–74], SOD1 [75, 76], LDHB [77] and YBX1 [78]. In response to a DNA damaging perturbagen, multiple factors linking cellular stress to DNA damage management, senescence and organismal longevity were also found to be physically associated with RXFP3 receptorsomes, including PHB2 [79–82], TRAP1 [83, 84], AIMP1 [85, 86], KPNA2 [87, 88], TUFM [89], EMD [90, 91], MAT2A [92, 93], NONO [94-100], IGFBP2 [101–103], SNRPA1 [104], HNRNPF [105], FAM98A [106] and ELAVL1 [107–110]. It is likely that during pathological aging, the generation of oxygen radicals, *e.g.* ROS, precedes the eventual DNA damage that is induced by this ongoing oxidative stress [111], which is what our results demonstrate. These data indicate that, with respect to PPI-associated signaling events, RXFP3 can be ‘activated’ or stabilized into distinct states by aging-associated stress and thus dynamically modifies its stable interactome profile.

To further investigate this dynamic receptorsome aspect of RXFP3 biology (Supplementary Figure 5), we performed unbiased natural language processing analytics to generate a gestalt appreciation of the distinctive phenotypes of the stress-associated RXFP3 interactomes (Supplementary Figure 5A). Applying the collective processing mode of Textrous! to the specific RXFP3 interactomes we found that the extracted words with the strongest association frequency to the entire peroxide-induced interactome dataset (measured using WriteWords) were all linked to DNA damage repair processes (Figure 5B), while the CPT-induced RXFP3 interactome, was linked to cell cycle regulation – indicative of potential senescent related behavior (Figure 5B). Using noun-phrase chunking platform of Textrous! [54], allowing more natural syntactic interpretations of the interactome datasets, we again demonstrated a DNA damage versus cell cycle regulation distinction between peroxide- or CPT-induced interactomes (Figure 5C). Inspecting the specific agglomerative wordcloud structures for the control (Figure 5D) RXFP3 interactome bore a strong association with neurodegenerative conditions such as dementia, and more specifically frontotemporal dementia, that are strongly associated with age-related damage accumulation [112] suggesting a basic and fundamental role of RXFP3 systems in age-related disease. The RXFP3 interactomes stabilized by peroxide (Figure 5E) or CPT (Figure 5F) treatment again revealed a strong bias towards DNA damage repair and cell cycle regulatory behavior respectively. Further adding to the connection between RXFP3 biology and its ability to interact with cell cycle machinery, we indeed found that the RXFP3 can physically interact with mitotic spindle structures (Supplementary Figure 4B), thus potentially revealing an additional role of the RXFP3 in anti-aging and Senescence Associated Secretory Phenotype (SASP)-associated mechanisms [113, 114] that have also been linked previously to GIT2 functionality [13, 15].

### Stress sensitive RXFP3 network analysis

**Figure 6:**
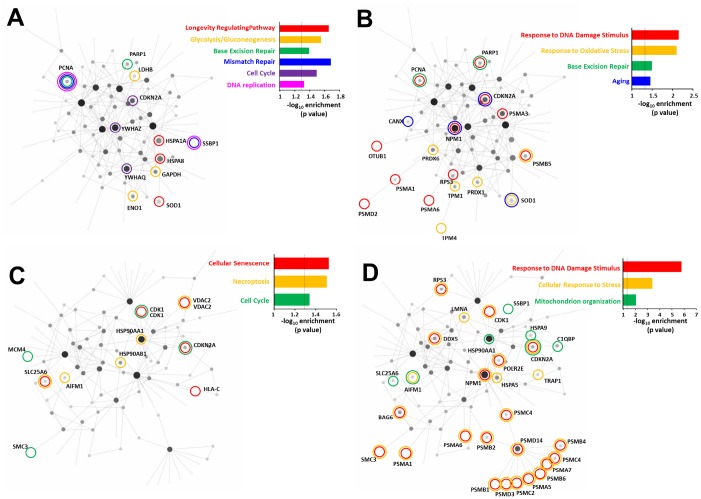
**Comparison of the oxidative stress and DNA damage RXFP3 interactome indicates roles in age-related processes.** Using the specific and consistent peroxide (**A**, **B**) and CPT-induced (**C**, **D**) RXFP3 interactome datasets we investigated the potential of these proteins to form a network using NetworkAnalyst. To generate statistically-significant interaction network KEGG enrichment (**A** and **C**) and Gene Ontology – Biological process (**B** and **D**) analysis were performed. Using both generic and hypothalamus-specific backgrounds resulted in highly similar enriched GO terms and KEGG pathways. Using the peroxide-induced RXFP3 interactome we found that the interacting protein network was (**A**) strongly associated with DNA protection management, cell cycle control, energy metabolism, and lifespan at the KEGG pathways level, (**B**) while at the GO biological annotation, we also see a strong connection to DNA integrity management, and additional age-associated oxidative damage. (**C**) Using the same KEGG pathways analysis on the CPT-induced interactome we again found a strong network phenotype associated with aging, *i.e.* cell cycle control, and age-related tissue pathology. (**D**) GO biological process analysis showed a strong association with pathological aging responses, such as energy diversification and stress resilience.

Using the specific and consistent peroxide- (Figure 6A, 6B) or CPT-induced (Figure 6C, 6D) RXFP3 interactome protein datasets, we investigated the potential dynamic physical networks of these proteins using the Network Analyst platform (Supplementary Figure 5C) [115]. We employed both generic (non-tissue specific) and hypothalamic-specific datasets (given the initial identification of GIT2 as an aging keystone in this tissue) as background databases to generate statistically-significant interaction networks for KEGG pathway enrichment (Figure 6A, 6C) and Gene Ontology (GO; biological process; Figure 6B, 6D). At the level of both GO and KEGG pathway enrichment, the use of either background databases resulted in highly similar enriched GO terms and KEGG pathways. For GO analysis the percentage overlap of hypothalamic (Supplementary Table 12 (Peroxide) –Supplementary Table 13 (CPT)) *vs.* generic tissue (Supplementary Table 14 (Peroxide) – Supplementary Table 15 (CPT)) was 72.1 and 87.1% for peroxide- and CPT-induced interactomes respectively. At the KEGG pathway level, these same analytical values were 56.5 and 45.5% respectively for KEGG analysis of peroxide or CPT effects.

Using the peroxide-induced RXFP3 interactome we found that at the KEGG pathway level (Supplementary Table 16 (Generic) – 17 (Hypothalamic)) the interacting protein network was strongly associated (indicated by significant pathway enrichment values) with DNA protection management (Base Excision Repair p=0.0403; Mismatch Repair p=0.0206), cell cycle control (Cell Cycle p=0.0319), energy management diversity (Glycolysis/Gluconeogenesis p=0.028) and lifespan (Longevity Regulating Pathway p=0.022: Figure 6A). Using GO biological annotation of the peroxide-induced RXFP3 interactome network we found that this protein cluster was strongly linked to DNA integrity management (Response to DNA Damage Stimulus p=0.00702; Base Excision Repair p=0.0319) and age-associated oxidative damage (Response to Oxidative Stress p=0.0079; Aging p=0.0346) (Figure 6B). Performing similar KEGG pathway analysis of the CPT-induced RXFP3 interactome (Supplementary Table 18 (generic) – 19 (hypothalamic)) we again found a strong aging-associated network phenotype of proteins, *i.e*. a strong representation (indicated by significant pathway enrichment values) of KEGG pathways associated with cell cycle control (Cell Cycle p=0.0455) and age-associated tissue pathology (Cellular Senescence p=0.0297; Necroptosis p=0.0311) (Figure 6C). GO biological process analysis of the CPT-induced RXFP3 interactome supported these results, *i.e.* energy diversification (Mitochondrion organization p=0.00854) and stress resilience (Response to DNA Damage Stimulus p=0.00000159; Cellular Response to Stress p=0.000377) (Figure 6D). Upon meta-analysis of these GO/KEGG annotations we found that for the oxidative stress RXFP3 interactome, the most dominant interacting protein factor was proliferating cell nuclear antigen (PCNA), through multiple (6 independent associations) links to different annotations (both GO and KEGG). In a similar manner, in response to CPT the most multidimensional factor (5 independent associations) linked to multiple significant annotations was cyclin-dependent kinase Inhibitor 2A (CDKN2A). Recent evidence has shown that one of the most specific functions of PCNA is to control the oxidative stress sensitivity of cells in concert with DNA damage repair mechanisms [116]. CDKN2A is an active connector between oxidative and DNA damage [117] with cellular senescence/pathological aging programs [118, 119]. The dynamic physical interaction of RXFP3 with these key factors in stress-related aging mechanisms underpins the potential importance of RXFP3 system in pathological aging.

### Comparative interactome analysis

**Figure 7:**
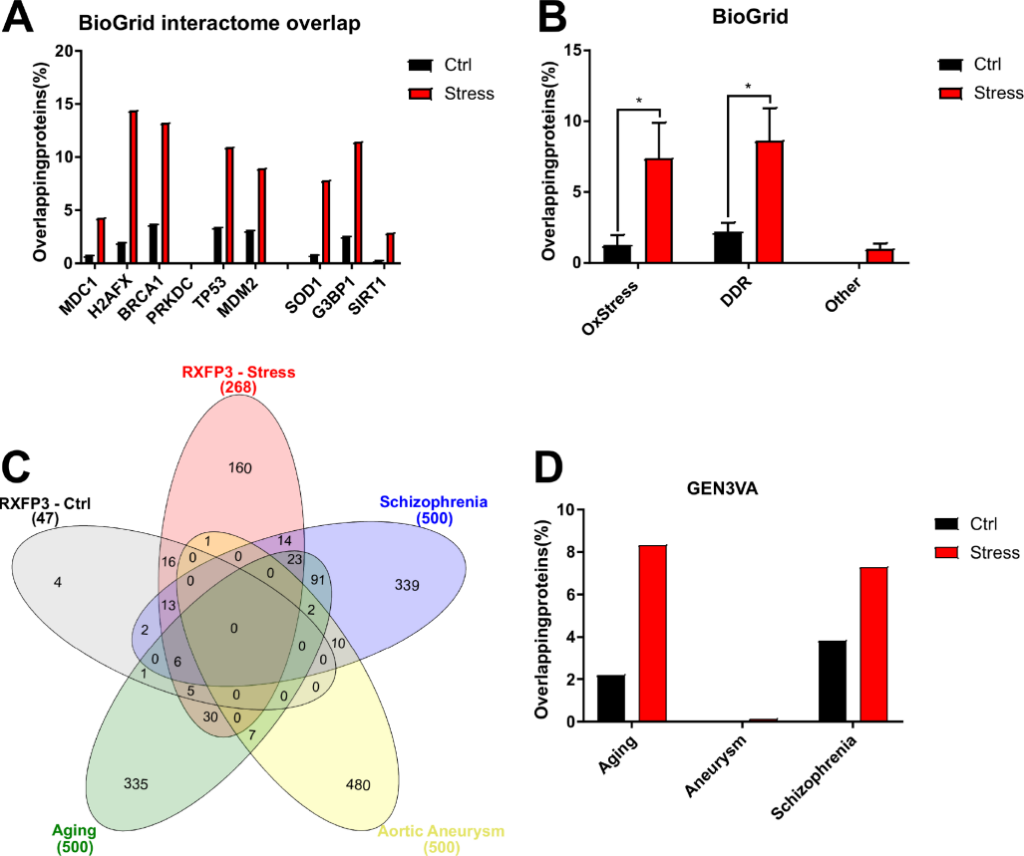
**RXFP3 interactome analysis using BioGRID extracted interactomes and GEN3VA extracted signatures, a first indication of a role in Aging.** Using the BioGRID database (http://www.thebiogrid.org) the interacting proteins of several known oxidative stress and DNA damage proteins were extracted, and assembled into specific interactomes. (**A**) Here we show the number of overlapping proteins between the unstressed RXFP3 interactome (Ctrl; black) versus the stressed interactomes (combined both the response to oxidative stress and DNA damage, Stress; Red) and the interacting proteins of DNA damage response/repair proteins (MDC1, H2AFX, BRCA1, PRKDC, TP53, MDM2) and oxidative stress proteins (SOD1, G3BP1, SIRT1). We see that while the RXFP3 interactome in control conditions already shows overlap, this overlap is considerably increased under stress. (**B**) When we assemble the interactomes of the oxidative stress and DNA damage proteins separately, Ox Stress and DDR, respectively, we again see this greater overlap for the RXFP3 “stress” interactome. When this was repeated for three proteins unrelated to oxidative stress, DNA damage or aging (other), we did not see this overlap. (**C**) Next, GEN3VA (http://amp.pharm.mssm.edu/gen3va/) was used to extract protein signatures pertaining to aging, which is of most interest to us, schizophrenia, of which we hypothesize RXPF3 might be a controlling factor, and as a negative control we extracted protein signatures for aortic aneurysm, where we suspect RXFP3 is not associated with at all. The overlap was visualized using InteractiVenn (http://www.interactivenn.net). (**D**) The overlapping proteins were then visualized in a bar chart, showing the large differences between “ctrl” and the “stress” interactomes of RXFP3. In addition, we see nearly no overlap with the signature for aneurysm, a decent overlap for schizophrenia, and a large overlap with aging. This data not only indicates the large differences between RXFP3 in control versus RXFP3 in stress conditions, but that RXFP3 possible plays an important role in oxidative stress, DNA damage response and aging.

To simplify the following analysis, we combined the peroxide- and CPT-induced RXFP3 interactomes to make one generic *‘stress’* interactome (Supplementary Figure 5B). We then compared the RXFP3 *‘control’* and combined ‘*stress*’ datasets to each BioGRID-derived protein interactomes. We extracted specific curated physical interactome database entries from BioGRID version 3.5, for well-known oxidative stress (G3BP1 – 299 proteins, SIRT1 – 251 proteins, SOD1 – 294 proteins) and DDR (PRKDC – 283 proteins, H2AFX – 300 proteins, MDM2 – 299 proteins, MDC1 – 198 proteins, TP53 – 300 proteins, BRCA1 – 301 proteins) proteins (Supplementary Table 20), to assess the potential PPI-based functionality of RXFP3 receptorsomes (Supplementary Figure 5D). To control for the differences in numerical size between the ‘control’ (47 proteins) and ‘stress’ RXFP3 interactome (268 proteins) we also employed multiple (n=3) randomly generated (http://www.molbiotools.com/randomgenesetgenerator.html) protein datasets (47 for ‘control’ RXFP3 and 268 for the ‘stress’ RXFP3 interactome) for comparative interactome analyses. We found that with both the ‘control’ and ‘stress’ interactomes, versus the random datasets, there were considerable functional overlaps with many of the aging/metabolism BioGRID interactome datasets (Figure 7A, 7B). The largest overlap was seen for G3BP1 (G3BP Stress Granule Assembly Factor 1), H2AFX (H2A Histone Family Member X), and BRCA1 (BRCA1, DNA repair associated) (Figure 7A). As an additional workflow control for this comparative interactome experiment we created an extra list of proteins relatively unrelated to aging and DNA damage (CNTRL, CRP, LONP2: ‘*Non-stress’*), where we clearly see a minimal overlap compared to the oxidative stress and DNA damage proteins (Figure 7B). The large overlap seen for all these proteins indicates an important regulatory role for RXFP3 in stress response. How this correlates to aging and age-related disorders was analyzed next.

We extracted available meta-data for several age-related disorders and non-age-related disorders from GEN3VA [120] and compared these to our interactome datasets (Figure 7C, 7D; Supplementary Figure 5E). We extracted datasets for classical Aging, Schizophrenia, in fact a potential aging-associated disorder [121] and non-specifically age-associated condition, *i.e.* aortic aneurysm as a control to assess whether the protein overlap is specific (Supplementary Table 21). As can be seen in Figure 7C and 7D, the numerical protein overlap with ‘Aging’ is larger than the other two datasets, where 64 of the combined RXFP3 ‘*stress*’ dataset show overlap, which is 23.88% of the total RXFP3 ‘*stress*’ set. Perhaps not completely unsurprising [121], we also see a strong overlap with ‘schizophrenia’, *i.e.* 54 proteins, or 20.90%. We see only one protein overlapping with ‘aortic aneurysm’ for the RXFP3 ‘*stress*’ dataset, namely PHGDH (Phosphoglycerate Dehydrogenase) and none for the RXFP3 ‘control’ set. These results gained from human curated dataset collections further support our hypothesis that RXFP3 may play a role in aging and the related disorders.

**Figure 8:**
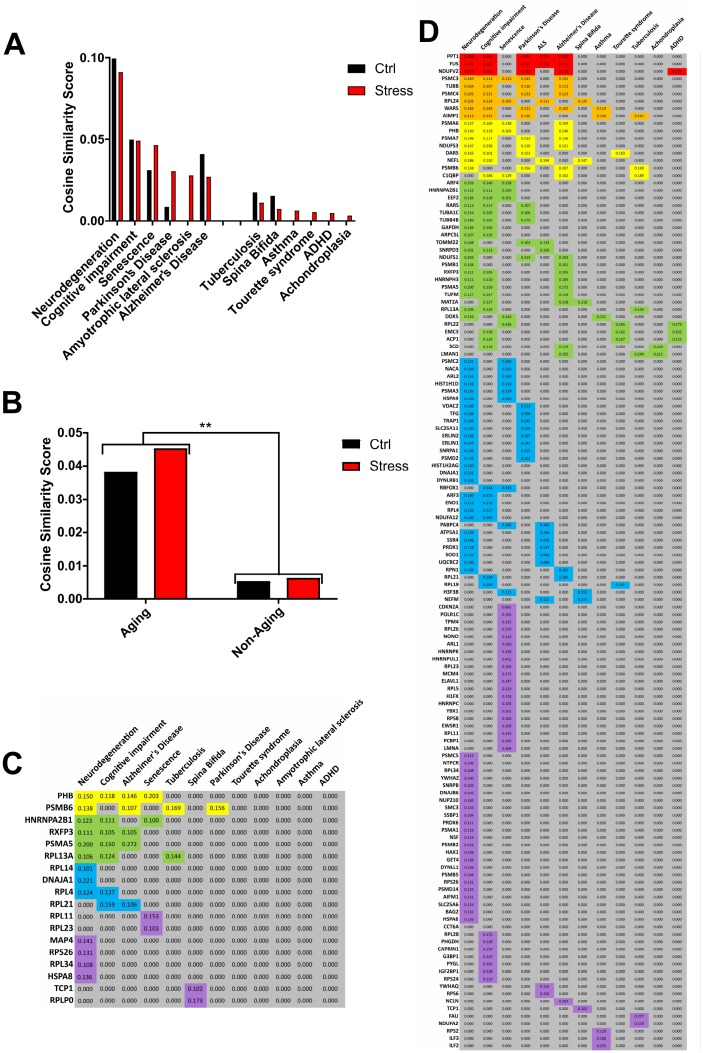
**Investigation of the RXFP3 interactome using Latent Semantic Indexing program, GeneIndexer.** Using specific age-related or -unrelated interrogator terms, the Latent Semantic Indexing program, GeneIndexer is able to show a relationship between our dataset and aging. (**A**) Using *Neurodegeneration, cognitive impairment, senescence, Parkinson’s disease, amyotrophic lateral sclerosis, and Alzheimer’s disease* as the Age-related interrogation terms (“aging”), and *Tuberculosis, spina bifida, asthma, Tourette syndrome, ADHD, achondroplasia,* as the unrelated aging terms (“Non-aging”), we were able to support our relationship of RXFP3 with aging (Figure 5). Both for the Control (ctrl) and Stress interactome a stronger correlation was seen with the aging interrogation terms. (**B**) When the results of the interrogation terms were added together, the difference between the aging and non-aging terms was significant. (**C**) GeneIndexer output with the cosine similarity score for the proteins with the interrogation terms of the control interactome for RXFP3, and (**D**) for the stress interactome for RXFP3.

Lastly, we used the Latent Semantic Indexing (LSI)-based informatic platform GeneIndexer [122], to interrogate our RXFP3 ‘control’ and ‘*stress*’ datasets with the following age-related syntactic concepts (Aging): *Neurodegeneration, Cognitive impairment, Senescence, Parkinson's Disease, Amyotrophic lateral sclerosis, Alzheimer's Disease*; and non-age-related terms (non-Aging): *Tuberculosis, Spina Bifida, Asthma, Tourette syndrome, ADHD, Achondroplasia* (Figure 8A, 8B; Supplementary Figure 5F). Using this, we were able to assess the (RXFP3 ‘control’ (Supplementary Table 22) or ‘*stress*’ (Supplementary Table 23)) strength of correlation with specific aging- and non-aging related interrogator concepts and the RXFP3 interactome datasets (Figure 8C, 8D), by averaging the different extracted individual protein cosine similarity scores. In this instance we see a larger average cosine similarity score for both RXFP3 datasets with the age-related terms compared to the non-aging interrogation paradigm.

### RXFP3 is a possible protective factor against DNA damage

**Figure 9:**
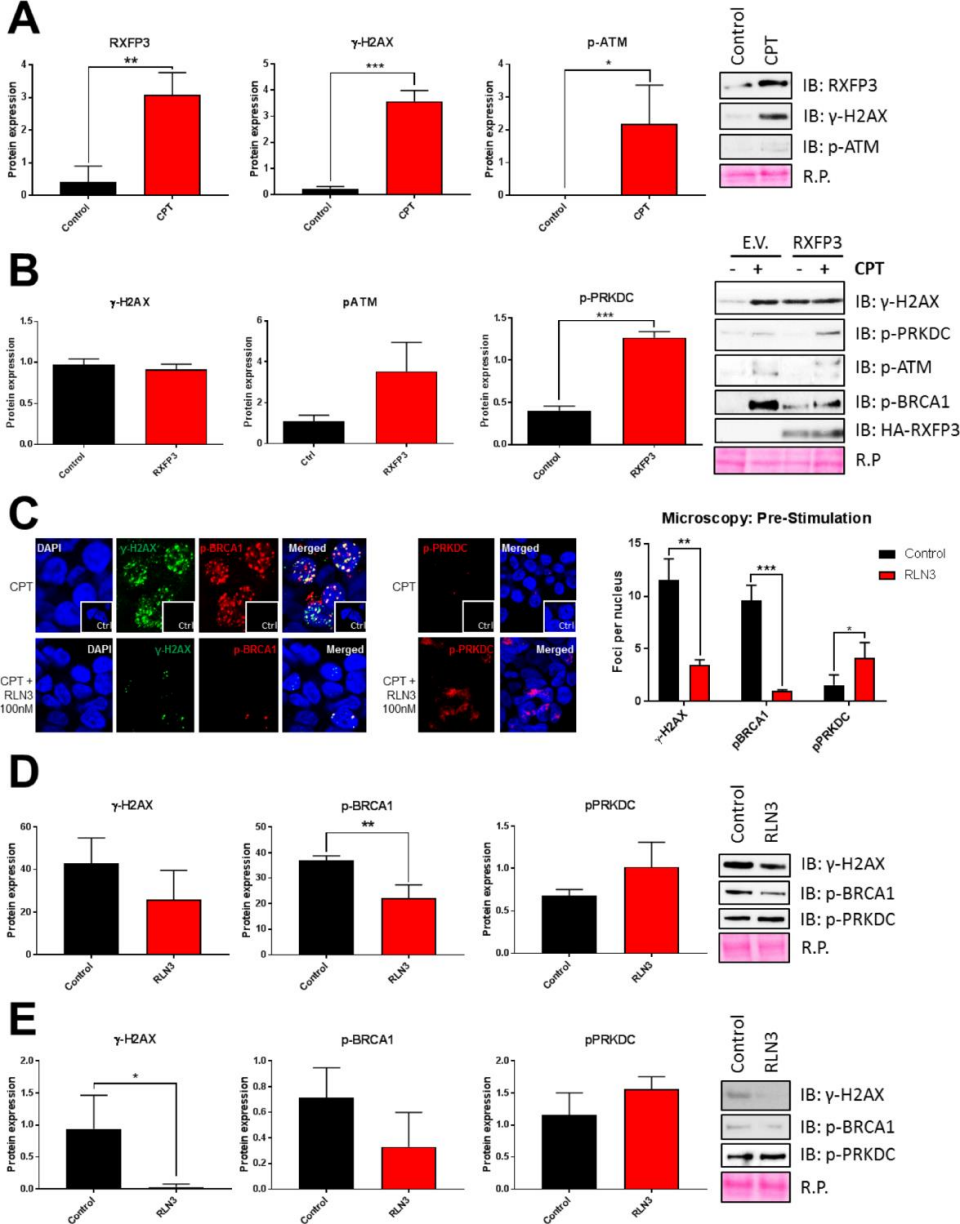
**RXFP3 acts as a protective factor of DNA damage.** (**A**) RXFP3 expression increases significantly with DNA damage caused by 10 μM CPT for 24 hours (n=3). Induction of DNA damage was validated using γ-H2AX and p-ATM. (**B**) Overexpression of RXFP3 compared to control (E.V.) elicits a specific response of DNA damage-associated proteins in unstressed stressed (CPT-treated) cells. Overexpression of the RXFP3, in the absence of CPT exposure, appears to prepare the cell for stress responsivity as this results in the activation of BRCA1 (p-BRCA1), PRKDC (p-PRKDC), ATM (p-ATM) and H2AX (γ-H2AX). However, we also see a decrease in activation compared to control (E.V.) transfected cells after stress of these proteins, indicating that the RXFP3 potentially facilitates DNA damage repair (n=3). (**C**) RXFP3 stimulation using RLN3 (100 nM, 1 and 2 hours prior to stress induction using 10 μM CPT), directly affects the number of γ-H2AX, pBRCA1 and pPRKDC foci (n=30) using confocal microscopy, where we see a specific decrease in γ-H2AX, and p-BRCA1 foci, and an increase in PRKDC activation. (**D**) These results were also shown using immunoblotting (n=6). (**E**) Stimulating RXFP3 after stress induction (2 shots, 1 and 2 hours after 3h 10 μM CPT), here called post-stimulation, induced similar effects (n=6).

If RXFP3 acts as a regulatory factor for aging-related stress management we hypothesized that, like GIT2 [10, 11], its expression may be directly altered by aging-associated stress perturbations. We compared 24 hours of vehicle to 24 hours of CPT stimulation and saw a significant increase in RXFP3 expression with CPT-induced DNA damage, which was indicated by an increase in γ-H2AX and phospho-ATM (Figure 9A). To assess the potential mechanistic role of RXFP3 in DNA damage repair, we overexpressed RXFP3 in cells prior to stressing the cells using CPT (Figure 9B). We investigated how this RXFP3 overexpression affected the phosphorylation status of DNA damage repair proteins, such as BRCA1, H2AX, ATM and PRKDC. The phosphorylation of BRCA1 is indicative of the cells’ attempt to repair and recover from the damage caused [123–125]. The phosphorylation of H2AX, generating the γ-H2AX form, is the first sign of DNA damage, which is dephosphorylated immediately after DNA damage has been repaired. ATM activation mediates DDR through homologous recombination (HR), while PRKDC (also known as DNA-PKc) activates non-homologous end-joining (NHEJ). With the introduction of ectopic RXFP3 expression we observed a decrease in ATM and H2AX phosphorylation, while we observed a concomitant increase in BRCA1, PRKDC phosphorylation (Figure 9B), suggesting that the cellular DNA-reparative process is likely induced with increased RXFP3 expression. When stress is applied to the cells, we observed a clear increase in ATM and PRKDC phosphorylation, while BRCA1 activation was diminished compared to control levels. This data suggests that RXFP3 overexpression likely enhances the cellular stress responsivity to potential DNA damage – in a similar manner previously observed with GIT2 expression potentiation and protection against DNA-damaging insults [11].

In addition to this passive functionality of RXFP3, we tested the DNA-protective ability of RLN3-mediated stabilization of RXFP3. In this experimental paradigm, cell cultures were strongly stimulated with RLN3 (100 nM) for 1 and 2 hours before the introduction of CPT-induced DNA damage. We observed an effective reduction in ATM, BRCA1, and H2AX phosphorylation, compared to our control conditions. These results indicate that the activation of RXFP3 with its endogenous ligand RLN3 attenuated the potential impact of DNA damage stress and thus may be associated with an augmented cellular protection from stress and thus aid in cellular recovery via a more effective stress resilience mechanism. This relationship between ligand-stimulated RXFP3 and DNA damage recovery proteins was next investigated using confocal microscopy (Figure 9C) and western blotting (Figure 9D). Here we observed a strong decrease in the number of nuclear foci identified using γ-H2AX and phospho-BRCA1 antibodies in conditions where the cells were pre-stimulated with RLN3 compared to the control cells (Figure 9C, D). In addition to this, we investigated the dynamic phosphorylation status of the DNA damage regulating kinase, PRKDC. Here we saw the opposite, PRKDC phosphorylation is higher in RLN3 compared to vehicle control conditions (Figure 9C, D). To assess the potential therapeutic (as opposed to prophylactic) DNA-protecting capacity of RXFP3 activity, we first stressed the cells with CPT and then stimulated them with two doses of RLN3. Similar to our findings in Figure 9D, we see that when administered after the DNA-stressing insult we again revealed the ability of RLN3 to reduce the extent of eventual DNA stress, suggesting that, in addition to a prophylactic DNA-protective capacity, RLN3 can also induce an efficient, post-stress, DNA-damage attenuation therapeutic action (Figure 9E).

## DISCUSSION

We investigated the potential connectivity of RXFP3 with the DDR signaling domain. Our previous studies demonstrated that the GPCR scaffolding protein GIT2 likely plays a potent and trophic level regulatory role in the aging process [52]. We have found that GIT2 expression is sensitive to oxidative perturbations [126], normal and pathological aging [9, 13, 127], alterations of dietary energy intake [13], age-related neurodegeneration [33], somatic glycemic and metabolic status [13] and DNA-damaging insults [11]. GIT2 genomic deletion has been shown to disrupt pancreatic beta cell development [13], adipose deposition [127], immune cell migration [53], control cellular senescence as well as attenuate overall lifespan [15]. These multidimensional findings suggest that GIT2 could indeed represent a novel therapeutic target for aging related disorders. One mechanism that is currently being investigated is the synthetic chemical mediated regulation of ADP ribosylation factor GTPase Activating Protein (ARF GAP) enzymatic activity, with the experimental agent QS11 [128]. While small molecules are a simple mechanism to regulate ARF GAP activity, the GIT molecules (both GIT1 and GIT2) are highly interconnected proteomic factors and simple indiscriminate inhibition of enzymatic activity will not likely elicit specific signaling effects. In addition, as GIT2 is a scaffolding protein, as well as an ARF GAP, it is also possible that simple chemical inhibition/modulation of ARF GAP activity, will not control all the additional protein-protein binding functionalities of GIT2. Our work over recent years has demonstrated that in addition to the simple regulation of intermediary cell metabolism events such as calcium mobilization or activation of protein kinases, GPCRs possess a functional efficacy profile that exists at the transcriptional and protein translational level [16, 17, 129-131]. Thus, GPCRs likely possess a translational relationship with signaling factors that they then employ as components in their diverse signaling outputs – hence each signaling molecule possesses an intrinsic priority relationship to their associated GPCRs. In this study, we investigated this potential for linking a cell surface GPCR to intracellular signaling protein GIT2, and to modulate its age-controlling functionality. Thus, we present a novel paradigm for therapeutic drug development and prioritization. Using a genomic deletion model for GIT2 analysis we found that the RXFP3 was reflexively altered in its expression in a coordinated manner with GIT2 (Figure 1). In addition to this expression-based relationship, we also demonstrated that both ectopic expression and ligand stimulation (using RLN3) of the RXFP3 receptor was able to exert an expression level effect (in multiple cell types) upon GIT2, suggesting a close functional synergy between GIT2 and the RXFP3 receptor. The cognate ligand of RXFP3, RLN3, is also termed insulin-like peptide 7 and like insulin, is composed of an A- and B-chain connected by two disulfide bonds [132]. While RXFP3 responds to the binding of its ligand, its amino acid structure (Supplementary Figure 1A, 1B) reveals the presence of a natural modification of the canonical third transmembrane helix (TM3) activity-regulating ‘DRY’ motif - replaced by a ‘TRY’ motif, where the aspartic acid is replaced by a threonine in humans and an alanine in mice. DRY motif disruption typically results in enhanced constitutive active levels in the absence of the cognate orthosteric ligand [36]. In typical class A GPCRs, the DRY motif Asp is bound to a Gln/Glu in TM6 which allows the formation of a salt bridge, creating an ionic lock which is further stabilized by the interaction between the Asp and Arg in the DRY motif itself [133, 134]. This ionic lock, which typically constrains the receptor in an ‘inactive’ state until a ligand is bound, is absent in RXFP3 allowing it to exist in the active conformation [36, 133, 134]. While considerable research has been conducted into how structural alterations can affect constitutive activity levels, these studies were conducted initially in the context of a monodimensional mode of GPCR signaling, *i.e.* through heterotrimeric G proteins. Subsequent discoveries concerning first β-arrestin [135] and then further multi- [16] and pluridimensional [136] GPCR signaling modes necessitates the application of a nuanced understanding of which multiple output efficacies are constitutively active, and which are not. Within the pluridimensional signaling context, it is likely that a broad receptorsome ensemble [137] is responsible for the multiple modes of receptor signaling. Therefore, the measurement of a single, often soluble second messenger index, is unlikely to reveal a diverse range of efficacy profiles.

Using our ‘constellation’ curve analysis, we correlated such a divergent signaling profile with a ‘theoretical’ GIT2 dataset approach we have pioneered recently to investigate unchartered signaling paradigms [17]. Employing this approach (Figure 3) we found that the RXFP3 expression level most associated with DDR functionalities was also most strongly intersecting (at the protein expression level) with the GIT2-signaling ‘theoretical’ dataset. It is interesting to note that the natural DRY motif mutation in the RXFP3 has been associated with preferences for enhanced receptor internalization. It may be possible therefore that this internalized RXFP3 receptor pool could initiate distinct signaling modalities independent from that emanating from the plasma membrane [138, 139]. In this context, it is unsurprising that such a functional idiosyncrasy may be associated with a protein strongly linked to internalization behavior and microvesicular movement, *i.e*. GIT2 [51]. This theory coincides with the possibility that RXFP3 might be a stress sensor within the cellular interior.

With our RXFP3 constellation expression data we saw a clear suggestion of a role in DNA damage response at 5 μg overexpression level compared to 0.5 μg (Figures 2 and 4; top 15 up and down-regulated proteins in Supplementary Table 24). In addition to these most strongly regulated proteins, we observed proteins that specifically relocate to another cellular fraction, *e.g.* Thioredoxin (TXN2) involved in oxidative stress response [140], translocated from the nucleus to the plasma membrane. Using LSI (Textrous!) we revealed a strong connection between DDR activity and increased RXFP3 expression (Figure 4B). While this may suggest that RXFP3 causes DNA damage, we hypothesize, that increased expression of RXFP3 prepares the cells for damage. Therefore, RXFP3 activity may enhance innate cellular resilience and thus engender an augmented, more sensitive response mechanism to DNA damage, which was supported by the elevation in RXFP3 expression after DNA damage (Figure 9A), which may be a response of the cell to prepare for the presence of incipient damage and thus responds by enhancing its coterie of interacting DDR proteins. In turn, our data also demonstrates that RXFP3 interacts with many proteins involved in the DNA damage response and repair process (Figure 5).

As signaling proteins are unlikely to exist as discrete unitary entities, it is now widely accepted that the formation of coherent and co-functional protein complexes lies at the basis of nearly each physiological and pathophysiological process [141–143]. Our AP-MS investigation into the dynamic RXFP3 interactome was interrogated using multiple unbiased approaches (Figs. 5-8) to generate an accurate gestalt appreciation of this signaling entity. From a receptor-based point of view our current research was primarily focused on the augmentation of our understanding of molecular interactomes in a high-dimensionality manner, *i.e.* focusing on proteins as a collective functional group rather than focusing down specifically on one protein in a more reductionist manner [144–146]. Hence, while not focusing in-depth on specific protein-protein interactions, albeit an entirely valid scientific endeavor, here we chose to appreciate molecular signaling at a more collective, gestalt, level [147–149]. Our follow-up manuscript on this topic, addresses in a combinatorial manner both a low-dimensionality (*i.e.* specific protein-protein interaction analysis) and high-dimensionality data analysis of DNA-repair complexes linked with this receptor system. Hence, the most effective appreciation of GPCR associated signaling dynamics will likely emerge from such synergistic approaches.

We found a potent link between RXFP3 and a role in DNA damage response and repair, cell cycle control and potentially even cell senescence regulation. We identified multiple proteins related to senescence, *e.g.* CDKN2A [150, 151], LMNA [152], PARP1 [153], PSMA5 [154], and PHB [155]. Senescence occurs naturally during embryonic development and recently it has received considerable attention through its potential role in the development of age-related disorders. Senescent cells arise due to replicative telomeric attrition or stress-associated cellular damage and can have either an unfavorable or beneficial impact on tissues and organs depending on the cell type and metabolic state. As senescent cells amass in tissues with progressing age, they have been connected to many aging declines and diseases [150]. CDKN2A (p16)/RB (Retinoblastoma-associated protein) directs one of the two pathways which is responsible for the initiating and maintaining cellular senescence programs, where CDKN1A or p21 together with p53, a consistent GIT2-interacting protein [11] directs the other [150].

BioGRID was used to compare the interactome of RXFP3 to known oxidative stress and DDR proteins (Figure 7). A large interactome overlap was found for several canonical DDR proteins, (MDC1 [156]; H2AFX - [157, 158]; BRCA1 [159, 160]; PRKDC [161], TP53 [162], MDM2 [160]) and anti-oxidative stress associated proteins (SOD1 [163]; SIRT1 [164, 165]; G3BP1 [166]). MDC1 is recruited to the double strand break (DSB) sites [156] after the initiation of the signaling cascade caused by these lesions, which starts with the phosphorylation of H2AX, generating the γ-H2AX species. The interaction between MDC1 and γ-H2AX allow for binding and retaining of additional DDR factors at DNA damage sites including GIT2 and BRCA1 [12, 167]. With DNA damage, p53 expression elevates significantly in cells and functions as a transcription factor to control the expression of proteins that coordinate the DDR related post-translational modification of the E3 ubiquitin ligase MDM2, which may act as the master regulator of p53 [160]. BRCA1 is a tumor suppressor gene, which contributes to DNA repair and transcriptional regulation in response to DNA damage, and protects the genome from damage. In addition, this repair protein regulates the transcription of proteins involved in the repair of DNA. Lastly, PRKDC is recruited to DSBs and aids in the repair of DNA via NHEJ, interacts with the GIT2-interactor p53 and appears to play a pivotal role in DNA repair in non-proliferating cells [168].

SOD1 scavenges ROS such as superoxide radicals produced in the body, thus playing a vital role in oxidative stress and lifespan management [169–171]. SIRT1, similar to SOD1, is an oxidative stress-sensitivity [172] and longevity regulator that has stress attenuation functions in vascular endothelial and neuronal cells, indicating a role in cardiovascular and neurodegenerative disorders. G3BP1, while not directly related to oxidative stress, is important in stress granule formation in response to this oxidative stress. Cells have two methods following exposure to environmental stress, *i)* induce apoptosis, *ii)* inhibit apoptosis and repair the damage induced by stress. These two options minimize cell loss, while preventing the damaged cells, with abnormal DNA and protein changes [173–175]. Stress granules control these two alternatives and this antioxidant activity is partially regulated by G3BP1 [176, 177].

The overlap over RXFP3 with these proteins related to oxidative stress and subsequent stress granule (SG) formation – which is also associated with senescence mechanisms [166] piqued our interest. SGs are formed by RNA binding proteins which consolidate important transcripts required to maintain cell viability during the presence and after the alleviation of stress [178]. In this context in our constellation data we found that SOD1, ATXN2 and G3BP1 were seen to be specifically translocated from the nucleus to the cytoplasm in response to RXFP3 overexpression. In addition, we have shown that RXFP3 interacts with SOD1 and G3BP1 proteins in response to oxidative stress and DNA damage, respectively, using AP-MS. This in combination with the considerable overlap of the RXFP3 and the curated SOD1 and G3BP1 interactomes, supports our hypothesis that RXFP3 acts as a stress sensor and then controls translation of DDR proteins and interacts with these SGs in order to protect the cell from potential harm. However, further investigation into this role of RXFP3 as an oxidative stress sensor and responder will be the subject of further studies.

Using GEN3VA-based curated data we were able to establish independently that there may be a role for RXFP3 in aging, and a new potential role was found for RXFP3 in the development of schizophrenia (Figure 7), a mental disorder characterized by delusions and hallucinations [179] that is being increasingly associated with pro-aging mechanisms [121]. One of the plausible underlying mechanisms for schizophrenia is a disruption in dopaminergic neurotransmission, possibly due to oxidative stress [180]. While this connection between RXFP3 and schizophrenia has previously not yet been made, as RXFP3 plays a potential role in dopamine transmission [181] and protection against oxidative stress, shown in this paper, this association with schizophrenia is not entirely surprising. Reinforcing this posit we have also recently shown that in pro-aging murine models areas of the central nervous system whose dopaminergic connectivity is affected also show deficits in both GIT2 and RXFP3 expression [33].

Following our molecular investigation of RXFP3 at the dynamic interactome level we have demonstrated that stimulation of the RLN3/RXFP3 system in cells, appears to be protective against DNA damage. Both prior to stress or following stressor exposure, RLN3 application decreased the phosphorylation of H2AX and BRCA1, while increasing PRKDC phosphorylation. This shows that RLN3 is a protective factor prior to damage, indicating it could possibly be used as a DNA-damage protecting agent.

Furthermore, BRCA1 is bound and phosphorylated by ATM kinase [125, 182], as well as G2/M control kinase, CHK2, and potentially many other kinases [183]. Further investigation has shown that BRCA1 is involved in complexes that promote and activate the repair of DSB and initiate HR. This is supported by the co-localization and interaction with important DDR proteins such as Rad51 [184], Rad50, MRE11 [185, 186], ATM [125, 182], H2AX [187, 188], and p53 [183]. Lastly, BRCA1 and PARP, whilst involved in completely different response and repair mechanisms, appear to cooperate to repair DNA damage [189]. In addition, and underlining the role of DDR processes in neurodegenerative aging, BRCA1 has also been recently implicated in AD [190]. Many of these stress repair proteins, *i.e.* H2AX, GIT2, PARP, BRCA1, and ATM, have been shown to be of importance in both here with the RXFP3 paradigm and our previous GIT2 signaling findings, *i.e.* GIT2 is phosphorylated by ATM upon DNA damage and form complexes with DDR-associated factors such as p53, ATM, PARP1 and γ-H2AX [11].

**Figure 10:**
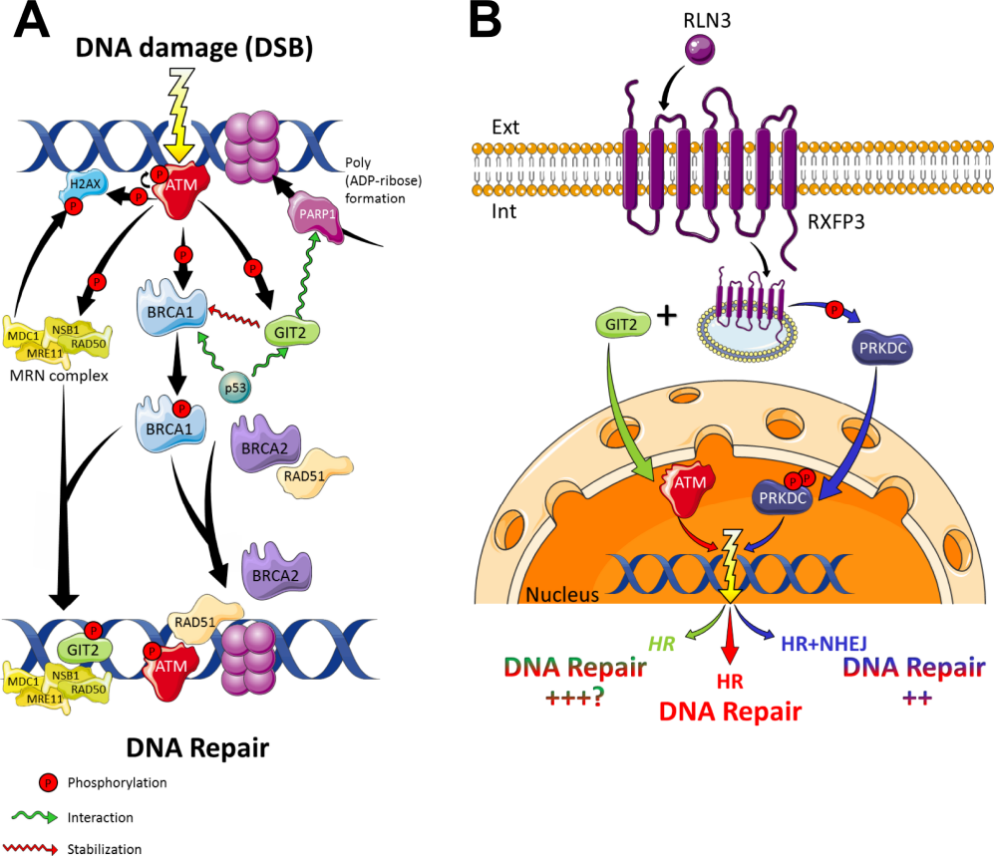
**RXFP3 in DNA damage response and repair.** (**A**) With DNA damage in the form of a double strand break (DSB), the cell has several mechanisms to respond and thus repair itself. The damage is recognized by the MRE11-RAD50-NBS1 complex (MRN complex), which recruits and activates ATM (Ataxia Telangiectasia Mutated), which is autophosphorylated. ATM, in turn phosphorylates multiple damage-associated proteins. H2AX (a variant of the histone H2A family) phosphorylation (generating γ-H2AX) allows for the recruitment of MDC1 (Mediator of Damage Checkpoint protein 1), also a phosphorylation substrate of ATM. Phosphorylated MDC1 serves as a scaffold for the recruitment of other proteins required for the activation of BRCA1 by ATM, thus promoting cell cycle arrest and DNA repair. BRCA1, in turn, then interacts with multiple proteins, *i.e.* p53, RAD50, RAD51, ATM, NSB1 and BRCA2, to modulate DNA repair, transcription, and the cell cycle. Phosphorylation of BRCA1 by ATM activates DNA repair through homologous recombination, in cooperation with BRCA2 and RAD51. Additionally, BRCA1 recruits the MRN-complex to the sites of DNA damage. ATM furthermore phosphorylates the aging keystone GIT2 promotes the repair of DNA damage via the stabilization of repair factor BRCA1, in the repair complex. GIT2 then allows the formation of a nuclear DSB focus dependent on H2AX, ATM, and MRE11. Also GIT2 is a strong interactor of the cell cycle checkpoint protein p53 and PARP1. PARPs play a pivotal role in DNA damage detection and repair, by the formation of ADP-ribose ribosylation complexes, allowing the recruitment of DDR proteins to the damaged DNA. After the DNA has been repaired, γ-H2AX is dephosphorylated by PP2A, a phosphatase. (**B**) Cells exploit two major DSB repair pathways, *i)* ATM-dependent homologous recombination (HR), and *ii)* PRKDC-mediated Non-homologous end joining (NHEJ). In the absence of a functional ATM, it seems cells are able to rely on functional PRKDC signaling for their survival, thus using the NHEJ pathway as a backup pathway for DSB repair [191].

Using our current findings we have attempted to situate RXFP3 and its signaling functionality within the known DDR domain (Figure 10A, 10B). We propose that RXFP3 participates in PRKDC activation following RLN3 stimulation. This RXFP3-entrained process allows the cell to employ PRKDC-dependent NHEJ, in addition to typically ATM-associated HR. RXFP3 can potentially enhance DNA repair, thus explaining our observed accelerated decrease in ATM, H2AX and BRCA1 phosphorylation, while PRKDC phosphorylation is increased. This proposal is supported by the research of Riabinska, et al. [191] which demonstrated that in ATM-defective cells, PRKDC offers a backup mechanism for failed DSB repair through HR, using NHEJ. Their research was further reinforced by the early embryonic lethality of ATM+PRKDC-KO mice [192], while animals only lacking one of the two proteins are viable [193, 194]. This direct effect of RXFP3 stimulation and expression on the activation of PRKDC and the NHEJ DNA repair is supported by minimal PRKDC phosphorylation alteration in response to DNA damage caused by CPT (Figure 9B, 9C). While the overall effect of RXFP3 system activation upon PRKDC expression and phosphorylation, also supports our acquired information displayed in Figure 9, using co-IP we further demonstrated that activated PRKDC (pPRKDC) interacts with RXFP3 (Supplementary Figure 4C). This interaction supports our theory depicted in Figure 10B, where we suggested that RXFP3 interacts with PRKDC and allows its activation, in addition to the activation of ATM thus facilitating DNA damage repair. As can also be seen, the RXFP3 sequence (Supplementary Figure 1) contains three potential phosphorylation sites, two for ATM and one for PRKDC. Interestingly, however, the phosphorylation sites mentioned in Supplementary Figure 1 are located at the extracellular region of the receptor. This indicates that this GPCR is potentially available in the cell in a usual ‘outside-out’ conformation, but also in a novel ‘outside-in’ conformation. It may thus be very important to explore further, through receptor mutation, as performed previously for GIT2 [11].

**Figure 11:**
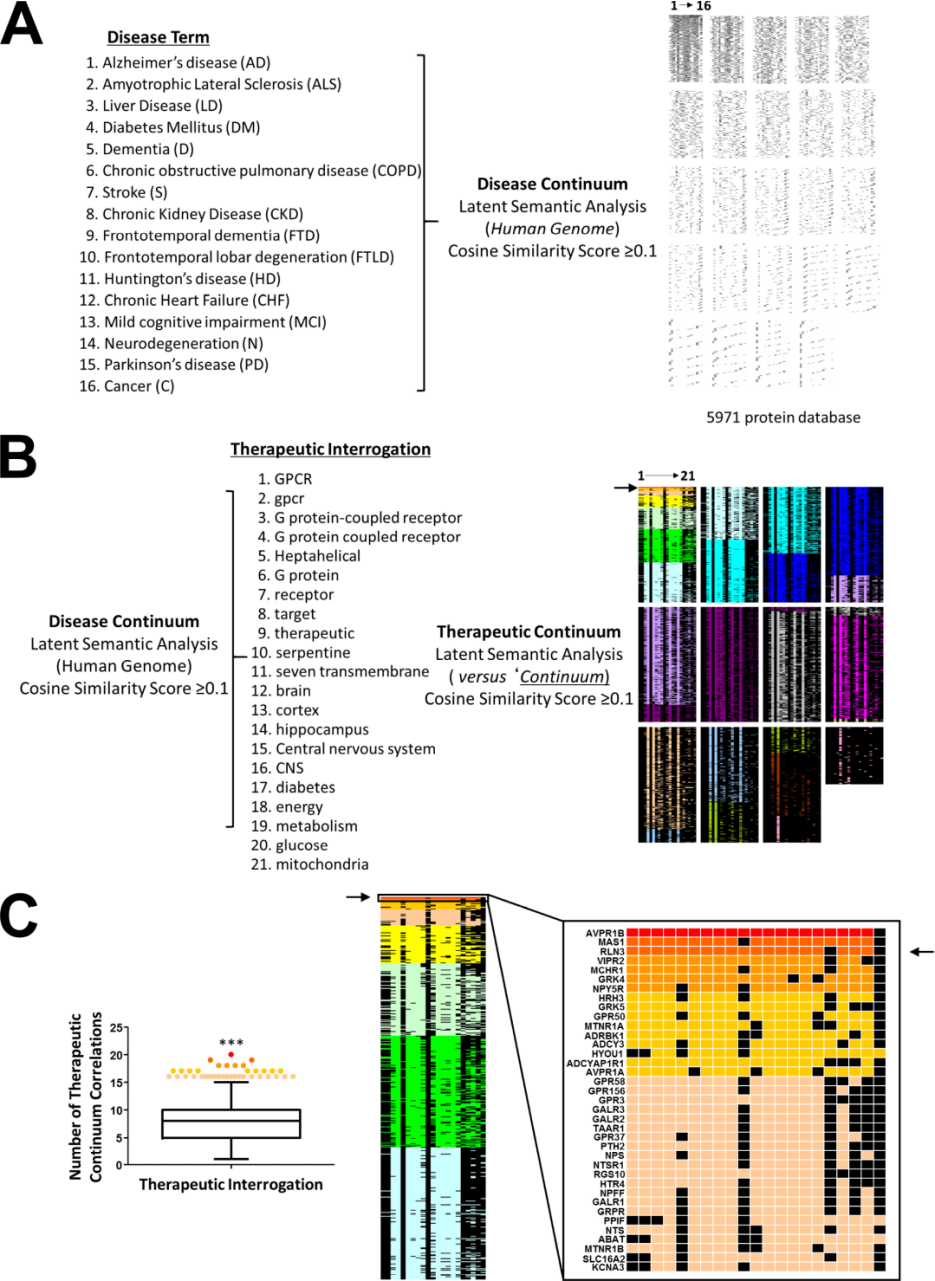
**Unbiased analysis of the potential role for RXFP3 in controlling age-related disorders.** To test the validity of our hypothesis in an unbiased manner, we used reverse-database analysis using latent semantic indexing platform GeneIndexer. (**A**) We started by creating a database of proteins, associated with input interrogator terms of the majority of age-related disorders, which we termed the ‘*Disease Continuum*’. (**B**) We next tried to identify a potential therapeutic target for these diseases, a GPCR in particular. To do so we investigated the Disease continuum protein dataset with GPCR-related terms, to create the ‘*Therapeutic continuum*’. (**C**) The proteins were ranked according to the cosine similarity scores, and based on a correlation ranking probability score (p<0.001, ***) 37 specific proteins were identified demonstrating a large amount of correlation within the therapeutic interrogators greater than the 99^th^ percentile. The top three ranked multidimensional proteins that could represent effective age-related disease targets were, AVPR1B, MAS1 and RLN3, the cognate ligand for the RXFP3.

Given our demonstration of a potential role for RXFP3 in controlling aging-associated disease we further tested the potential validity of our contention at an unbiased, mass analytical level. To this end we employed a recently demonstrated technique of reverse-database analysis using LSI [195]. As such, we first created a database of proteins, extracted from the entire human genome (via PubMed Central text mining using GeneIndexer), explicitly and implicitly associated with input interrogator terms covering the majority of age-associated disease conditions (Supplementary Table 25), termed the ‘*Disease Continuum*’ (Figure 11A). To identify potential therapeutic targets that could interact and potentially control this ‘*Disease Continuum*’ in a multidimensional manner we isolated the proteins within this continuum most strongly associated with a battery of terms linked to central nervous system function, energy metabolism and GPCR-focused signaling (Supplementary Table 26). Ranking the Cosine Similarity scores of the resultant factors that were prominent in the disease continuum and the ‘Therapeutic Interrogators’ (Figure 11B) we found that, based on a correlation ranking probability score (p<0.001, ***) there were 37 specific protein targets demonstrating a number of correlations within the therapeutic interrogators that was greater than the 99% (representing only 0.67% of the input ‘*Disease Continuum*’ dataset). The top three ranked multidimensional proteins that could represent effective age-related disease interdiction targets were, arginine vasopressin receptor 1B (AVPR1B), MAS1 proto-oncogene, GPCR (MAS1) and RLN3, the cognate ligand for the RXFP3 (Figure 11C). It is interesting to note that the MAS1 [196] and arginine vasopressin receptors [197] have recently been implicated in age-related disorder amelioration. In this context, using an unbiased multidimensional approach it is tantalizing to anticipate that therapeutic modulation of the RXFP3 may also hold significant promise for the attenuation of multiple age-related diseases.

Taken together, our findings suggest that RXFP3, may be involved in oxidative stress and DNA damage stress response, in addition to its role in corticotropin stress response [21, 198]. This involvement of GPCRs in DDR has only recently been observed [195], and it appears that the receptor systems have long been developed alongside DDR systems to act as stress sensors, as appears to be the case for RXFP3, *i.e.* oxidative stress, DNA damage and potentially age-related stress. When we combine this information with its expressional and functional relationship and association with GIT2 [9, 10, 12, 13], it appears that we may be able to target RXFP3, and as such control GIT2 and potentially treat or even prevent age-related disorders such as neurodegeneration. Future research should focus on further unraveling the relationship between GIT2 and RXFP3 so that RXFP3-targeted therapeutics can be designed.

## MATERIALS AND METHODS

### Cell culture, transfection and treatment

Human HEK293 (CRL 1573) were obtained from ECACC and propagated at 37^o^C with 5% CO_2_ ambient tension, according to the approved culture protocols defined for these cell lines. Human neuronal SH-SY5Y (CRL-2266) were obtained from ATCC and again propagated according to the published protocols of the disseminating organization. Murine hypothalamic neuronal cells, GT1-7 cells were obtained from Pamela Mellon (San Diego, California, USA [199]). All cell lines were maintained in Dulbecco’s Modified Eagle Medium (DMEM; Sigma-Aldrich) with 10% fetal bovine serum (FBS)-containing propagation media, supplemented with 1% Penicillin/Streptomycin antibiotics as previously described [200].

For the interactomic experiments, we used SILAC (Stable isotope labeling of amino acids in cell culture), in order to label our proteins/peptides prior to experimentation. As such this technique allows us to analyze a very small amount of peptides using mass spectrometry. Customized DMEM media, without arginine and lysine were purchased from AthenaES. This medium was supplemented with *‘medium’* (L-lysine-U-^13^C_6_ (K6), L-arginine-U-^13^C_6_ (R6)) and *‘heavy’* (L-lysine-U-^13^C_6_-^15^N_2_ (K8), L-arginine-U-^13^C_6_-^15^N_4_ (R10)), respectively (Cambridge Isotope Laboratories [201], and with dialyzed FBS and 1% antibiotics. To engender at least 90% of protein labeling, cells were cultured in SILAC medium for at least five passages. The ‘medium’ and ‘heavy’ conditions were compared to avoid non-labeled peptide errors [201]. Dependent on prevailing growth rates, the cells were passaged on a regular basis.

One day prior to transfection, 3×10^6^ cells were seeded into 10cm plates to obtain a 50–80% cell confluence the day of the transfection. Cells were counted using a Luna II Automated Cell Counter (Invitrogen-Life Technologies). The cDNAs for a hemagglutinin (3xHA)-tagged human RXFP3 receptor (obtained from the Missouri S&T cDNA Resource Center: https://www.cdna.org) and an empty plasmid (pcDNA3.1+: Invitrogen-Life Technologies) were transfected into the cells with Lipofectamine® 3000, using the manufacturers’ instructions. To investigate the effect of differential receptor overexpression on downstream proteins, we transfected the cells with a range of cDNA concentrations (0.5, 1, 2, 5, and 10 μg). To induce oxidative stress, cells were treated with 100 nM hydrogen peroxide (H_2_O_2_/peroxide) for 90 minutes. DNA damage was caused using 1 μM Camptothecin (CPT) for 3, and 24 hours, dependent on the experiment.

Investigation of the ability of RXFP3 to accelerate DDR, we initially overexpressed RXFP3 compared to E.V. (5 μg) after which we stressed the cells with 10 μM of CT, cells were extracted using RIPA 1% SDS (150mM NaCl, 50mM Tris, 0.5% Sodium deoxycholate, 1% NP-40) 1% SDS (sodium dodecyl sulphate). To assess whether stimulation of RXFP3 using its endogenous ligand RLN3 might have a possible protective ability, we pre-stimulated the cells with 100 nM of RLN3. We administered two stimulation points, 1 and 2 hours prior to stressing the cells with 10 μM CPT for 3 hours. As a control, an equal amount of TFA was used, which we used to dissolve RLN3. This experiment was repeated in reverse, where cells were stressed prior to RLN3 stimulation.

### Cellular protein extraction

For generic low-definition cellular protein extraction, following a described cellular treatment, cells were washed three times with ice-cold PBS and scraped from dishes in the presence of either RIPA 0.1% or 1% SDS supplemented with phosphatase Inhibitor Cocktail (PhosSTOP, Roche Diagnostics) and protease inhibitor cocktail (complete mini, Roche Diagnostics), dependent on the experiment. To generate differential cell fraction protein extracts, cells were first washed as monolayers with ice-cold PBS and then subjected to a detergent dependent fractionation process using a Q proteome extraction kit (Qiagen) according to the manufacturers’ instructions. Before eventual analytical use, protein quantification of generated cellular lysates was performed using a standard colorimetric protein assay, *i.e.* the Bio-Rad RC DC^TM^ assay (Bio-Rad).

### iTRAQ sample preparation

The protein concentrations were assessed a second time after trichloroacetic acid (TCA) precipitation overnight at 4°C using 1/3 volume of TCA, to ensure correct protein levels. For each sample 100 μg of protein was reduced using 5mM Tris-2-(carboxyethyl)-phosphine hydrochloride solution (TCEP; Pierce Biotechnology) and cysteine blocking was performed with 2mM methyl methanethiosulphonate (MMTS) solution (Sigma-Aldrich). Each sample containing 100 μg proteins, was digested using 10 μg trypsin (Promega) at 37°C overnight. Samples were then labelled using iTRAQ reagents (ABSciex) before being pooled into one mixture. The following iTRAQ labels were used for the transfected cells: iTRAQ labels 113 was used for the transfected cells with the pcDNA3.1+ empty plasmid; iTRAQ labels from 114 to 118 were used to label the differentially transfected cells with RXFP3 (114-0.5 μg_RXFP3-HA, 115-1 μg_RXFP3-HA, 116-2 μg_RXFP3-HA1, 117-5 μg_RXFP3-HA, 118-10 μg_RXFP3-HA). After labelling, the samples were pooled, dried down and dissolved with SCX buffer prior to SCX chromatography.

### SILAC sample preparation

RXFP3 interactomes, extracted using RIPA 0.1% SDS, were isolated by immunoprecipitation using anti-HA bound beads (Sigma-Aldrich) overnight on an end-over-end shaker, after which the bound proteins were extracted using 100μl of 150mM Glycine-HCl buffer (pH 2.5). The samples were continuously vortexed for 5 minutes, then centrifuged. The supernatants, containing the proteins, were immediately transferred to a new tube containing 40μl neutralizing buffer (1M Tris, pH 8). Proteins were then precipitated overnight at 4°C using TCA, after which the pellet was washed with acetone and resuspended in 60μl resolubilization buffer (6M Urea, 2M ThioUrea, 10% SDS in 50mM TEAB). Equal amounts of proteins from ‘*medium*’ and ‘*heavy*’ conditions were mixed to prepare the SILAC mix. The sample was then reduced (TCEP), cysteine blocking was performed (MMTS), and the proteins were digested with trypsin (see section iTRAQ sample preparation).

### Strong cation exchange chromatography separation (SCX)

The pooled samples were diluted 10-fold with HPRP buffer A (5mM KH_2_PO_4_ in 5% acetonitrile at pH 3.0), to reduce sample complexity during the LC-MS/MS analysis. These were then separated using a 1mm x 150mm polysulfoethyl aspartamide column (Dionex). The column was eluted with a gradient: 0 to 25 minutes 60% HPRP buffer B (5mM KH_2_PO_4_ at pH 3.0 in 5% acetonitrile containing 0.5M NaCl), 25 to 45 minutes 100% HPRP buffer B and 25 to 45 minutes 100% HPRP buffer A. For each sample, 40 fractions were eluted and then pooled into 10 fractions. These fractions were then diluted before being loaded into the C18 column.

### Nano-LC-MS/MS analysis

The mass spectrometric analysis of the iTRAQ samples was performed using a nano-LC column (Dionex ULTIMATE 3000) coupled online to a Q Exactive™-Plus Orbitrap (ThermoScientific), the SILAC samples were analyzed using our Orbitrap Fusion™ Tribrid™ (ThermoScientific). Peptides were loaded onto a 75 μM × 150mm, 2 μM fused silica c18 capillary column, and mobile phase elution was performed using buffer A (0.05% formic acid, 99.5% Milli-Q water) and buffer B (0.05% formic acid in 80% acetonitrile/ Milli-Q water). The peptides were eluted using a gradient from 4% buffer B to 90% buffer B over 45 min at a flow rate of 0.3μl/min. The LC eluent was directed to an ESI source for Orbitrap analysis. The mass spectrometer was set to perform data dependent acquisition in the positive ion mode for a selected mass range of 350-1800 m/z for quantitative expression difference at the MS1 (70000 resolution) level followed by peptide backbone fragmentation with normalized collision energy (NCE) of 32, and identification at the MS2 level (17500 resolution). The raw data was analyzed using Thermo Fisher Proteome Discoverer 2.0, the software was connected to a Sequest HT search engine (Thermo Fisher Scientific) using UNIPROT/SWISSPROT annotated database. Each protein was assigned a confidence score (0% to 100%) based on the confidence scores of its constituent peptides based on unique spectral patterns. Proteins were only identified from the recovery and measurement of one peptide (from MS2) that is identified with a 99% confidence.

### Bioinformatic analyses

We applied a multidimensional informatic approach to the analysis of our proteomic and interactomic data. To facilitate the specific separation of complex datasets, we employed the Venn diagram platforms, VennPlex, VENNTURE [202, 203] and interactivenn (http://www.interactivenn.net/). To generate unbiased outputs, we employed the latent semantic indexing (LSI)-based informatic platform *Textrous!* [17] to create *de novo* signaling descriptions from selected datasets that facilitate a more nuanced appreciation of high-dimensionality receptor signaling paradigms [17]. From *Textrous!-*based natural language processing analyses wordclouds were generated with WordCloud (https://www.wordclouds.com/). To extract both word and phrase frequencies from our *Textrous!* output we employed the Frequency Counter application from WriteWords (http://www.writewords.org.uk/word_count.asp). Furthermore, Enrichr (http://amp.pharm.mssm.edu/Enrichr/#) was employed to analyze the significantly relevant related pathways and diseases/ontologies, identified using Gene Ontology (GO).

In addition, we created interactomes of several important players in oxidative stress (G3BP1, SIRT1, and SOD1) and DNA damage (PRKDC, H2AFX, MDM2, MDC1, TP53, BRCA1) response *in silico*, using the freely available BioGRID (https://thebiogrid.org/). As a control for this experiment we made an additional list of proteins unrelated to these stress responses (Non-stress; *i.e.* CNTRL, CRP, LONP2).

Next, we used GEN3VA (GENE Expression and Enrichment Vector Analyzer; http://amp.pharm.mssm.edu/gen3va/), which is based on GEO (gene expression omnibus; https://www.ncbi.nlm.nih.gov/geo/), a web-based system enabling the integrative analysis of amassed collections of gene expression signatures identified and extracted from GEO. Allowing us to extract specific disease signatures, which we can compare to our own data. We have extracted the following signatures: *i)* Aging (found in GEN3VA under the name “Model of cerebral aging and Alzheimer's disease: temporal cortex”), *ii)* schizophrenia (in GEN3VA “Schizophrenia: postmortem superior temporal cortex”) and *iii)* aortic aneurysm (“Abdominal aortic aneurysm”). The latter was used as a control to assess whether the protein overlap is specific and this does not occur with non-aging-related datasets (Data sets found in Supplementary Table 4).

Lastly, LSI platform GeneIndexer (https://geneindexer.com/) was used to investigate the association of our protein set with our own input of interrogator terms. Here we used age-related and age-unrelated terms, *i.e.* Aging: *Neurodegeneration, Cognitive impairment, Senescence, Parkinson's Disease, Amyotrophic lateral sclerosis, Alzheimer's Disease* (Figure 8A); and non-Aging: *Tuberculosis, Spina Bifida, Asthma, Tourette syndrome, ADHD, Achondroplasia* (Figure 8B). The use of this LSI allows us to use natural language processing for data extraction, identifying hidden connections between the proteins and the interrogation terms. The GeneIndexer database holds more than 1.5 million Medline abstracts, which correspond to over 21.000 mammalian genes. This program extracts all gene-to-word relationships from the literature using LSI. Here a cosine similarity score larger than 0.2 typically specifies an explicit association, while a score lower than 0.2 indicates an implied relationship, a cutoff score was set at 0.1.

For advanced network-based analysis we employed the NetworkAnalyst (https://www.networkanalyst.ca/) application that is designed to serve as a visual analytics platform for comprehensive gene expression profiling and meta-analysis. NetworkAnalyst allows for the creation, and eventual informatics interrogation, of multiple network types. Multiple types of protein interaction database are available for interactome enrichment analysis including the IMEx (International Molecular Exchange Consortium) consortium (http://www.imexconsortium.org/), STRING (https://string-db.org) and the CCSB-associated Rolland Interactome (http://interactome.dfci.harvard.edu/H_sapiens/: [204]).

### Immunoblot, immunoprecipitation, and immunocytochemistry

To validate the proteomic data, the experiments were replicated and analyzed using immunoblotting with a standard protocol (Supplementary Figure 4C, 4D). In short, all samples separated on 4%–12% SDS-PAGE (Life Technologies), transferred to PVDF membrane (Amersham) and blocked using 5% BLOTTO milk. Primary antibodies for immunoblots: GIT2 (Bethyl), RXFP3 (LSBio), HA-tag, phospho-BRCA1 (ThermoScientific), G3BP1 (Santa Cruz Biotechnology), Actin (Sigma Aldrich), PARP1, EIF4a1, γ-H2AX, PHB (GeneTex), XPOI (Atlas Antibodies), phospho-ATM (Rockland), DDB1, phospho-PRKDC OSSA (Abcam), Src (Cell Signaling technology). The membrane was then incubated with species appropriate secondary antibodies conjugated to horseradish peroxidase (HRP), immune complexes were then identified using enhanced chemiluminescence (ECL, GE Healthcare) and an Amersham imager 680 system. WB quantification was performed with GE-ImageQuant TL and Image J software, using red ponceau staining as a loading control.

SILAC data was validated using a standard co-immunoprecipitation protocol coupled to immunoblotting, where the proteins were extracted from the anti-HA affinity beads through resuspension in Laemmli buffer (DTT and LDS 2x), after which the samples were analyzed using immunoblotting (see above). Immunostaining was performed according to a standard protocol. Briefly, cells were fixed in 4% paraformaldehyde, for at least 20 min and no longer than 24h at room temperature. Non-specific labeling was blocked with 5% goat serum (1:500 dilution; Dako, Heverlee, Belgium) for 1 h, primary antibodies were incubated for 1 h (1:500 dilution) and secondary antibodies for 30 min at room temperature. Nucleus staining was performed with DAPI (Invitrogen), after which cells were mounted with Dako fluorescent mounting medium (Dako, Heverlee, Belgium) and examined with confocal microscopy LSM 700 (Zeiss).

### Murine tissue RT-PCR

GIT2KO gene-trap animals [205] based on a standard C57BL/6 background, initially obtained from Duke University (Richard Premont, Durham, NC) were bred at the National Institute on Aging under NIH protocol numbers, 432-LCI-2015 and 433-LCI-2015, according to approval of the Institutional Review Board. All animal studies performed were approved according to the guidelines of the NIA Animal Care and Use Committee. Mice were maintained in a 12h light/dark cycle on an *ad libitum* regular diet. The RNeasy Mini kit (Qiagen) was used for cellular mRNA extraction from multiple tissues derived from wild type (C57Bl6) and GIT2KO mice. Reverse transcription was performed using proprietary kits (Life Technologies, Carlsbad CA). Genes were normalized to GAPDH. RT-PCR was performed using the ABI Prism 7300 Sequence Detector (Applied Biosystems, Carlsbad CA).

### Statistical analyses

In each histogram or figure, data represent the means ± SEM (standard error of the mean). Statistical analyses (Student’s t-test) were performed using GraphPad Prism version 7.0 (GraphPad Software, San Diego, CA, USA). Significance level is indicated in each figure as *p ≤ 0.05; **p ≤ 0.01; ***p ≤ 0.001.

## Supplementary Material

Supplementary Figures

Supplementary Table 1

Supplementary Table 2

Supplementary Table 3

Supplementary Table 4

Supplementary Table 5

Supplementary Table 6

Supplementary Table 7

Supplementary Table 8

Supplementary Table 9

Supplementary Table 10

Supplementary Table 11

Supplementary Table 12

Supplementary Table 13

Supplementary Table 14

Supplementary Table 15

Supplementary Table 16

Supplementary Table 17

Supplementary Table 18

Supplementary Table 19

Supplementary Table 20

Supplementary Table 21

Supplementary Table 22

Supplementary Table 23

Supplementary Table 24

Supplementary Table 25

Supplementary Table 26
