# Supplementary material for: The RXFP3 receptor is functionally associated with cellular responses to oxidative stress and DNA damage
Source: Aging (Albany NY). 2019 Dec 3;11(23):11268–313. doi: 10.18632/aging.102528 (PMC6932917; doi:10.18632/aging.102528)
Supplement: Supplementary Figures [file aging-11-102528-s014..pdf]

SUPPLEMENTARY FIGURES

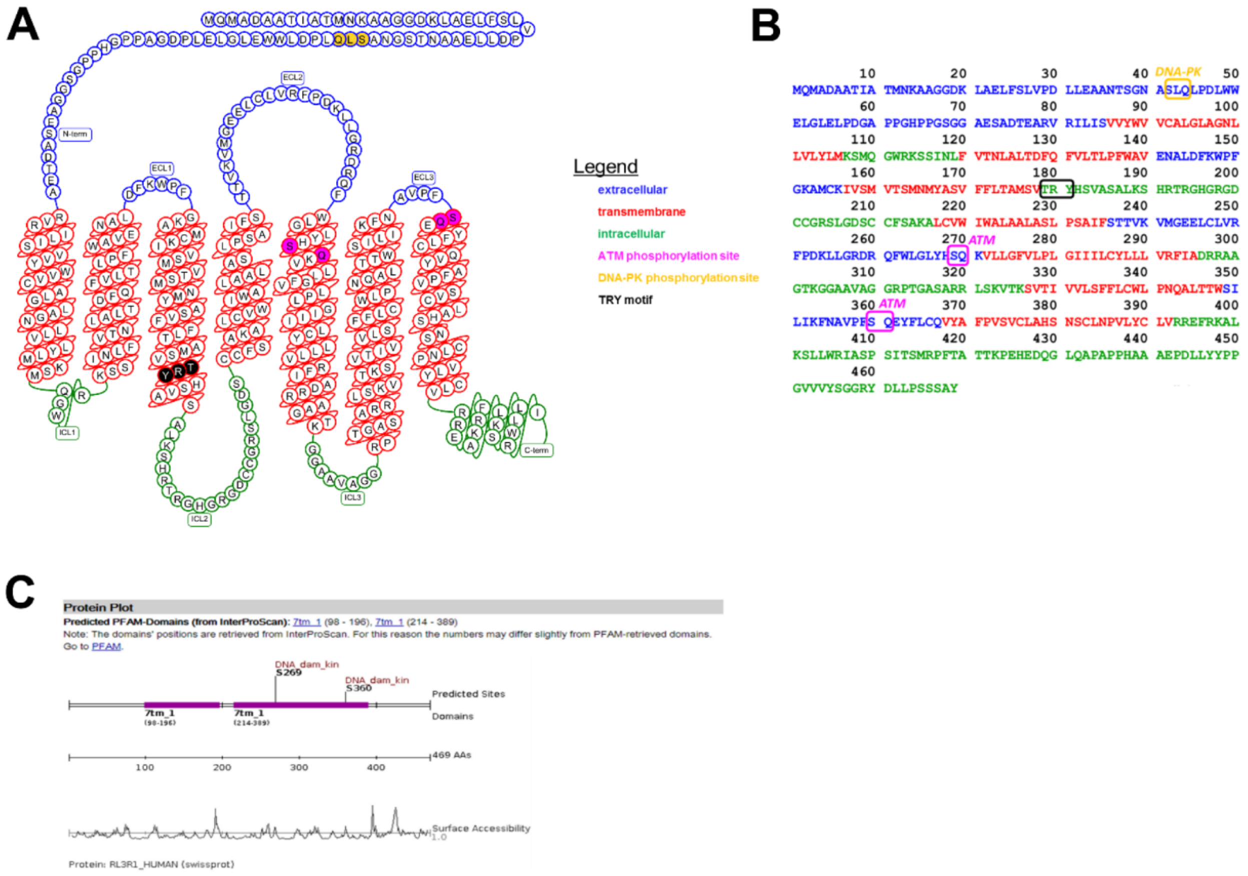

Supplementary Figure 1. Analysis of the RXFP3 amino acid structure indicates a potential role in the DNA damage response. (**A**, **B**) Amino acid structure of RXFP3. (**A**) RXFP3 represented as a heptahelical snake extracted from GPCRdb (www.GPCRdb.org), and (**B**) as an amino acid sequence. Visualized in both A and B are the predicted extracellular (blue), transmembrane (red), and intracellular (green) domains. Further investigation into the different motifs in the sequence indicates the absence of the typical "DRY" or Asp-Arg-Tyr motif responsible for receptor conformation change after ligand activation, instead a "TRY" or Thr-Arg-Tyr motif (Black) is present. In addition, we identified two phosphorylation sites for Ataxia Telangiectasia Mutated (ATM), with typical motif: Sx, in this case SQ or Ser-Gln (Pink), and one phosphorylation site for DNA protein kinase C (DNA-PK also known as PRKDC); with typical SxQ motif, in this case SLQ or Ser-Leu-Gln (Yellow). Potential phosphorylation sites were identified using Scansite (https://scansite4.mit.edu) and InterProScan (http://www.ebi.ac.uk/interpro/interproscan.html).

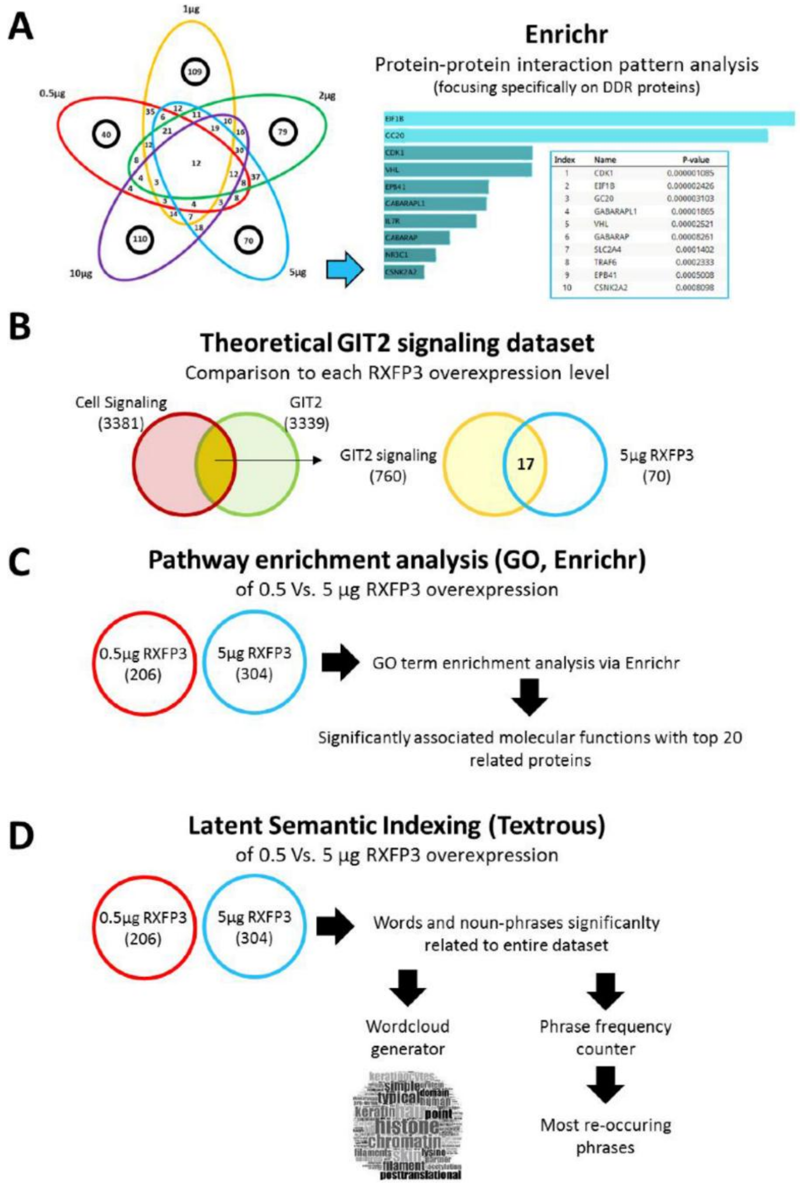

Supplementary Figure 2. Flowchart of the quantitative proteome Bioinformatic pipeline. For the bioinformatics analysis of our quantitative proteomics data of the different receptorsomes of RXFP3 obtained through our constellation experiment, we have used the following investigative techniques organized in a flowchart. (**A**) First we isolated the proteins unique to each overexpression level of RXFP3 (0.5 μg, Red; 1 μg, yellow; 2μg, green; 5 μg, blue; 10 μg, purple), and analyzed each level individually using the PPI-hub function of Enrichr. As such we performed a protein-protein interaction pattern analysis of the constellation perturbagens, where we focused specifically on DNA damage response (DDR) proteins in order to investigate which overexpression level shows the clearest relationship to DNA damage repair. (**B**) Next, we used latent semantic indexing to create a theoretical dataset for 'GIT2 signaling' (760 proteins, yellow), by overlapping the genes extracted from GeneIndexer significantly correlated to interrogator terms related to 'Cell Signaling' (3381 proteins, Red) and 'GIT2' (3339 proteins, green). This theoretical GIT2 signaling dataset was then compared to the different overexpression levels of RXFP3, where we found that the 5 μg dataset was the most reminiscent of GIT2 signaling. To further interrogate the role of this 5 μg dataset, we compared the full datasets of 5 μg and 0.5 μg overexpression with (**C**) Gene Ontology, which allowed us to extract the significantly associated molecular functions, associated with the dataset, and (**D**) latent sematic indexing tool Textrous! which allows us to extract the words and noun-phrases significantly associated to the entire gene list of each dataset. These words and noun-phrases were then organized in a word cloud which correlates word frequency with word size and writewords, which allows us to identify the most re-occurring phrases.

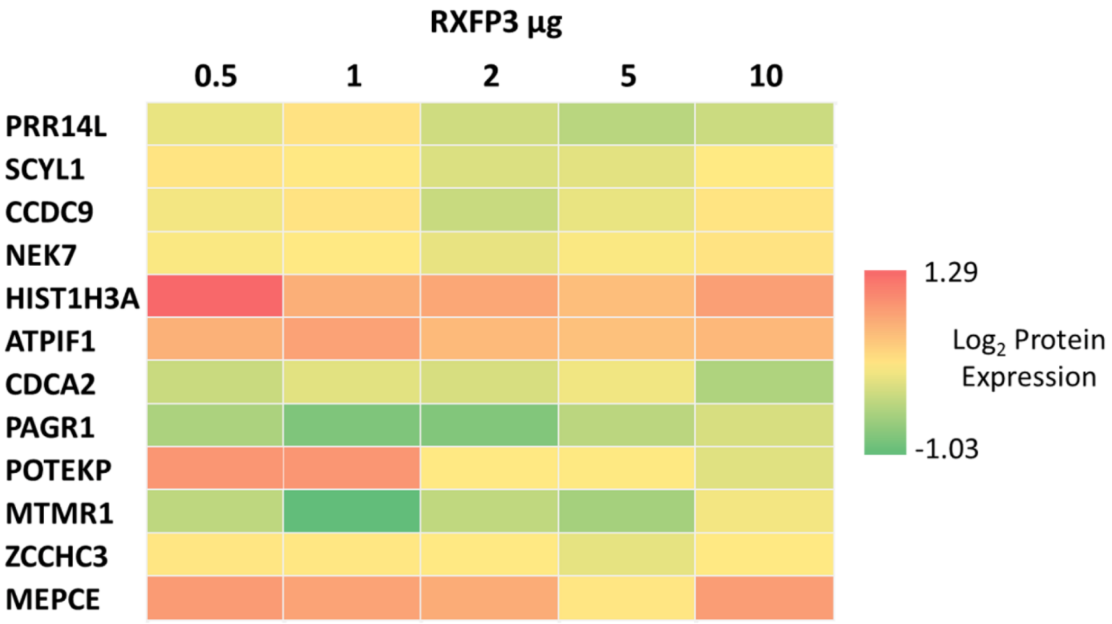

Supplementary Figure 3. Expression level-independent RXFP3 associated proteins. Through InteractiVenn (www.interactivenn.net), we were able to investigate the unique and overlapping proteins for the RXFP3 constellation experiment. 12 proteins were significantly altered across each of the RXFP3 overexpression levels, *i.e.* PRR14L, SCYL1, CCDC9, NEK7, HIST1H3A, ATPIF1, CDCA2, PAGR1, POTEKP, MTMR1, ZCCHC3, and MEPCE. These proteins show involvement in energy metabolism regulation, DNA damage associated with aging, and cell senescence. Using a heatmap we are able to show the protein expression of each protein, where green is downregulation and red is upregulation. Certain proteins were contra-regulated in the different overexpression levels and cellular fractions, and are therefore neither upregulated nor downregulated (yellow).

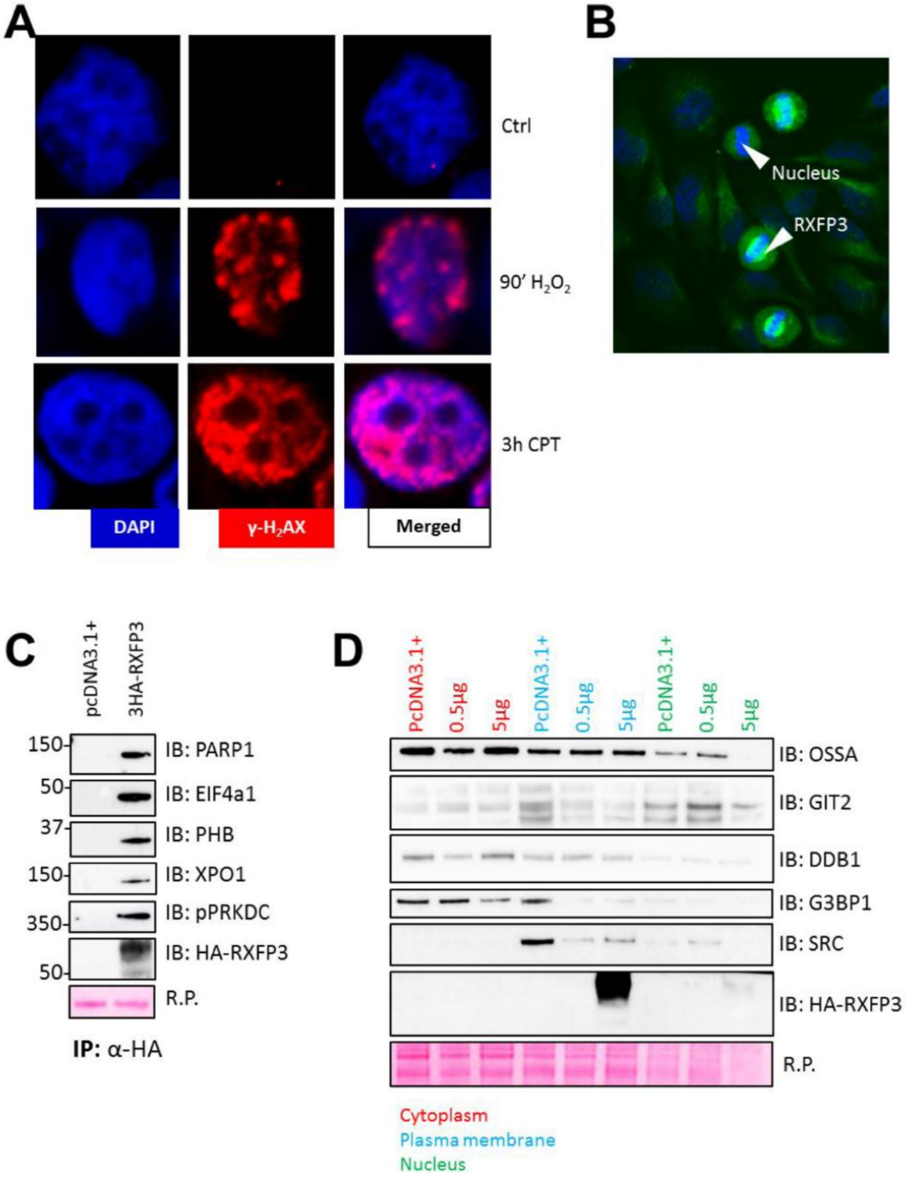

Supplementary Figure 4. Molecular Analyses of stress-associated damage and protein-protein interaction. (**A**) Validation of mass spectrometry data (RXFP3 0.5 and 5 µg overexpression) through immunoblotting, testing: SRC, G3BP1, OSSA, DDB1, and HA (RXFP3), with Red Ponceau as a loading control. (**B**) Validation of interactomics experiment using co-immunoprecipitation. (**C**) DNA damage validation after oxidative stress and camptothecin, using the DNA damage responder γ-H2AX as a marker, indicating the 90 minute exposure of 100 nM H_2O_2 can cause a possibly survivable amount of DNA damage, while 3 h of 1 µM camptothecin elicits a larger amount of DNA damage. (**D**) Confocal microscopy was used to investigate the expression of RXFP3 (green), where we see that RXFP3 expression can overlap with the mitotic spindle (blue) during mitosis, indicating a role in cell cycle control.

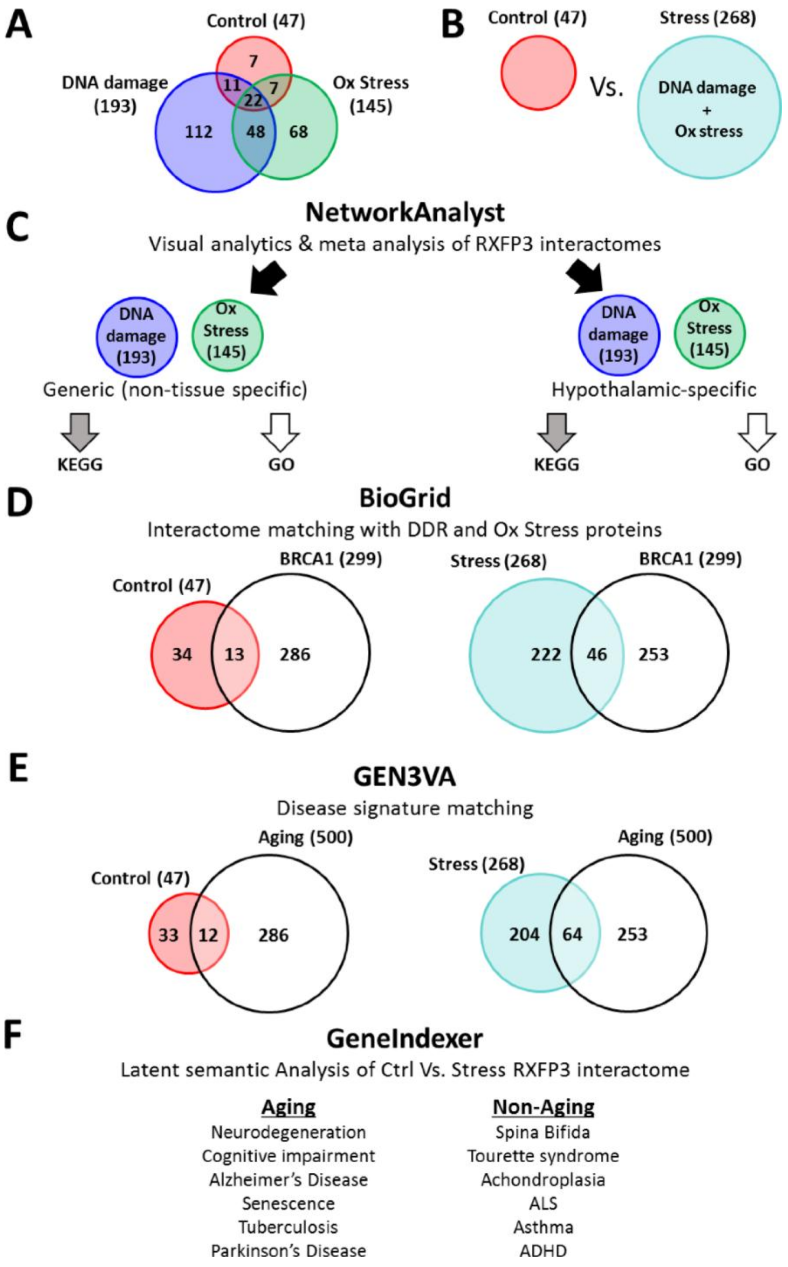

Supplementary Figure 5. Flowchart of the interactome Bioinformatic pipeline. To further elucidate the role of RXFP3 we have continued by investigating it interacting proteins in three different conditions, (**A**) *i)* Control (Red, 47 proteins), *ii)* Oxidative stress (Ox stress, Green; 145 proteins), and *iii)* DNA damage (Blue, 193 protein). Initially, we started the interactome analysis by isolating the unique proteins to each condition, and analyzing them with textrous, word clouds and write words, similar to what we have described in Supplementary Figure 2D. (**B**) For further analysis we also created a *'stress'* dataset (turquoise) where we have combined the oxidative stress and DNA damage interactomes. (**C**) The network analyst platform was used to assess the potential of these proteins to create a dynamic physical network. In order to do so, we employed both non-tissue specific (generic) and hypothalamic-specific datasets, to generate interaction networks for both KEGG pathway enrichment (grey arrow) and Gene Ontology (GO; white arrow). These datasets we then overlapped with our experimentally generated RXFP3 interactome after DNA damage and Oxidative stress. (**D**) We then performed a comparative interactome analysis, by investigating the overlap of the interacting proteins of several DNA damage-related (PRKDC, H2AFX, MDC1, TP53, BRCA1), and oxidative stress-related (G3BP1, SIRT1, SOD1) proteins, with our control and *'stress'* interactome dataset (as described in B). As a control for the specificity of the overlap, we also extracted the interacting proteins of several proteins which are unrelated to DNA damage response and oxidative stress (other; CNTRL, CRP, LONP2). (**E**) to further elaborate a role for RXFP3 in aging, we compared our datasets (Control and Stress) to several aging related disease signatures extracted from GEN3VA, to establish the specificity of this overlap we also used several control signatures such as aortic aneurysm, which are not age-related. (**F**) Lastly, we used latent semantic indexing tool GeneIndexer to interrogate our control and stress dataset with age-related (Aging) and –unrelated terms (Non-Aging). GeneIndexer associates our dataset with these interrogation terms and expresses significance with a cosine similarity score.
