# Supplementary material for: The RXFP3 receptor is functionally associated with cellular responses to oxidative stress and DNA damage
Source: Aging (Albany NY). 2019 Dec 3;11(23):11268–313. doi: 10.18632/aging.102528 (PMC6932917; doi:10.18632/aging.102528)
Supplement: Supplementary Table 1 [file aging-11-102528-s023..pdf]

Table S1: Proteins selectively affected by the RXFP3 constellation. In an unbiased manner we discovered the proteomic 'constellation' perturbagen response to ascending expression levels of RXFP3 expression (0.5, 1, 2, 5, 10μg of cDNA expressed). Protein extracts were investigated as a multiplex using quantitative proteomics through iTRAQ labelling with each RXFP3 expression level compared ratiometrically to the proteomic response effects to empty vector ectopic expression. The ratios were then normalized using a Log_2 transformation, allowing us to identify the significantly up- and downregulated proteins.

0.5μg		1μg		2μg		5μg		10μg	
Gene Symbol	Log_2	Gene Symbol	Log_2	Gene Symbol	Log_2	Gene Symbol	Log_2	Gene Symbol	Log_2
AASDHPPT	0.294177644	AASDHPPT	0.325364834	ACLY	-0.292542289	ACTC1	0.271539975	ABLIM1	-0.332220572
ABCF1	-0.238729249	ACADM	0.25560635	ACOT7	-0.34518901	AGTRAP	0.296490926	ACTA2	-0.194868466
ABLIM1	-0.295446062	ACAT1	0.335100621	ACTC1	0.743016546	AHCYL1	0.558913446	ACY1	0.286055465
ACOX1	0.352519881	ACOT7	-0.220086212	AHCYL1	0.561512025	ALB	0.37685384	AHCYL1	0.293053616
ACTC1	0.344455414	ACTC1	0.303334806	AIF1L	-0.257611393	ALDH5A1	0.297748322	AHSA1	-0.297855957
AHCYL1	0.445385857	AFF4	-0.289851897	AK6	0.217915408	ANP32A	0.18341314	AIF1L	0.207420555
AIDA	0.286140339	AHSA1	0.30836933	ALDH5A1	0.24464642	AP3B1	-0.303101928	AK6	0.279852669
AIF1L	-0.241909298	AKR1B1	0.381214273	ANKRD11	-0.474910075	ARHGEF18	0.194974413	AK6	0.279852669
ALG9	0.676016115	ALDH5A1	0.389317218	ANP32A	-0.370269523	ARL8B	-0.260314503	ALCAM	0.30834821
ANKRD11	-0.277508735	ALG9	0.461573364	AP2S1	-0.338298764	ASMTL	-0.285562062	ALG1	-0.288540758
ANKZF1	0.461782475	ANKRD11	-0.27255319	AP3B1	-0.31080733	ASPSCR1	0.219918851	ANKRD11	-0.544017002
AP3B1	-0.266769456	APRT	0.270950252	ARL14EP	-0.360886717	ATL3	-0.323081679	ANP32A	-0.295605932
AP3D1	0.236183718	ARHGEF2	0.262897667	ATPIF1	0.272809073	ATOX1	0.172625738	AP3B1	-0.316018475
ARHGAP11A	-0.244503482	ARL14EP	-0.253013246	ATXN2	-0.319331327	ATPIF1	0.179550436	ARHGEF18	0.323345208
ARHGEF18	0.331523071	ASMTL	-0.290995803	B4GAT1	-0.242105958	ATXN10	-0.258671403	ASMTL	-0.213930426
ASMTL	-0.246149736	ATOX1	0.305780778	BANF1	0.418023533	ATXN2	-0.527003055	ATL3	-0.298298994
ATP5C1	0.281798429	ATP5A1	0.427904167	BCLAF1	0.248102454	BANF1	0.481931315	ATPIF1	0.296369478
ATP5G3	0.313705542	ATP5B	0.353870651	BIRC5	0.269041449	BUB1B	-0.32397814	BANF1	0.281928005
ATP5H	0.231590348	ATP5C1	0.318823125	BRIX1	-0.37599491	C15orf38-AP3S2	0.18224626	BIRC5	0.315412626
ATP5J2-PTCD1	0.267766929	ATP5D	0.486532741	BUB1B	-0.536689395	C4orf32	-0.31813144	BRIX1	-0.226328127
ATPIF1	0.380469739	ATP5F1	0.264529972	C3orf17	0.26894941	CA3	0.755350473	BUB1B	-0.302505258
ATXN10	0.233277691	ATP5G3	0.290018208	C4orf27	-0.314680018	CADM4	0.27705221	BZW2	-0.320733202
ATXN2	-0.347991086	ATP5J2-PTCD1	0.317527757	CA3	0.484425101	CALM2	0.16197064	C19orf53	0.353942375
BPHL	0.239156379	ATP5O	0.279135144	CADM4	0.24725512	CAPN2	-0.261307045	CA3	0.320322102
C12orf75	-0.26276642	ATPIF1	0.571191271	CAND1	-0.289280338	CAPNS1	-0.280158025	CAPNS1	-0.372370845
C1orf174	-0.296784531	ATXN10	0.28656706	CAPN2	-0.399885598	CARHSP1	0.228804686	CCDC9	-0.256711568
C4orf32	-0.275806353	ATXN2	-0.287773094	CAPNS1	-0.262976931	CCDC9	-0.428989344	CCNB2	0.43402735
CA3	0.230159127	BANF1	-0.274326516	CAPRIN1	-0.278219555	CCK	0.266538434	CDC73	-0.487217952
CALD1	-0.296450134	BUB1B	-0.317152923	CASC3	-0.375490378	CD9	-0.276226967	CDCA2	-0.689431775

CALR	0.303753439	C1QBP	0.409888308	CBX1	0.383572052	CDC73	-0.629365293	CENPV	-0.239963147
CALR	0.303753439	CALD1	0.264472481	CCDC9	-0.578875534	CDCA2	-0.397730738	CEP55	0.335023026
CAPRIN1	-0.264199716	CALR	0.395967777	CCSMST1	-0.320174516	CDK9	-0.323685731	CHD4	-0.207524868
CASC3	-0.282913504	CALU	0.243698799	CDC73	-0.579476554	CDKN2AIP	-0.26468516	CHMP1B	0.316760088
CBS	0.235942	CAPRIN1	-0.306362449	CDCA2	-0.511550591	CENPL	-0.260478511	CHMP2A	0.332582381
CBX1	0.36885386	CARHSP1	0.239417475	CDCA3	-0.257580737	CENPV	-0.366501814	CHMP3	0.195991044
CCDC9	-0.388787162	CASC3	-0.383487457	CDK9	-0.304091678	CEP55	0.295130228	CNBP	-0.351551
CCNK	-0.237592104	CBS	0.291650494	CDKN2AIP	0.238110339	CFL1	0.167814159	COX14	-0.329007055
CDCA2	-0.572116462	CBX1	0.508390076	CENPV	-0.383792415	CGN	-0.308861258	COX5A	-0.348554912
CDCA3	-0.261997204	CBX3	0.233103937	CEP55	0.272959887	CHCHD2	0.307058273	COX6B1	0.364165967
CENPV	-0.225732366	CCDC86	-0.264969312	CHD4	-0.33937399	CHD4	-0.344665129	CPNE1	-0.29041289
CEP131	-0.262749403	CCDC9	-0.245948622	CHMP2A	0.243896053	CHMP2B	-0.265388936	CRYGD	-0.203053378
CGN	-0.239029904	CCNYL1	0.351811888	CHMP2B	-0.236806789	CLN6	0.277907188	CTSV	0.288762911
CHCHD2	0.329967647	CDCA2	-0.46083529	CLTC	-0.290597455	CNOT10	-0.335306466	DCP1B	0.222271291
CHD3	-0.242526248	CDKN2AIP	-0.443149178	COPB1	-0.34121033	COPZ1	0.26549606	DCTD	-0.289756164
CLASP1	0.2767584	CGN	-0.293039742	COQ6	-0.343958495	COQ6	-0.318255202	DDOST	-0.251846942
COX6B1	0.458417583	CHMP2A	0.277702375	COX5A	-0.358928386	COX5A	-0.3032919	DDT	-0.261879173
CRKL	0.239327479	CHTOP	0.240936101	COX6B1	0.188392706	CRYGD	-0.316191917	DDX20	-0.272358731
CSNK1E	-0.235936687	CNBP	0.26664066	CSRP2	-0.289933808	CTSV	-0.445650241	DERL1	0.289706186
CTSV	-0.271203329	CNBP	0.26664066	CTBP2	-0.348798869	CTTN	-0.2663929	DFFA	-0.294341045
DCD	-0.374078821	COX6B1	0.418721482	DBNL	-0.237204185	DCD	-0.300457641	DIABLO	0.339601057
DCTD	-0.2296447	CS	0.245635151	DCTN4	-0.289320074	DCTD	-0.279388727	DPY30	0.391422375
DDX19A	0.388126862	CTBP2	-0.224893341	DDB1	-0.301785644	DDB1	-0.276528287	DSG1	0.449467007
EIF4E	-0.26289304	CTSV	-0.385866974	DDT	-0.27081301	DDOST	-0.277366119	EEA1	0.188921055
EMC6	-0.23421476	CUL4A	-0.240577425	DDX19A	0.446185275	DDX19A	0.548467894	EEF2	-0.195089207
FMR1	-0.255940172	CXXC1	-0.278607791	DERL1	0.242086827	DHX57	-0.255072238	EIF3G	-0.344445116
FOXK2	-0.228125007	DCTD	-0.263876698	DHRS4	-0.268271193	DHX9	-0.257144797	EIF3L	-0.309263192
FTSJ3	-0.597216129	DDB1	-0.237353595	DHX15	-0.298659061	DSG1	0.330684414	ENAH	-0.434365204
FXR1	-0.257151169	DDRGK1	0.309620788	DHX57	-0.287704667	EIF1AX	0.175679571	EPC1	0.353386897
G3BP1	-0.324607388	DDT	0.267851991	DHX9	-0.287806677	EIF3C	-0.328265683	EPN1	-0.343263538
G3BP2	-0.443212682	DDX19A	0.43404105	DNM1L	-0.30137597	EIF3G	-0.303679795	ESD	-0.352285626
GABARAP	0.337986989	DDX6	-0.266779733	DYNC1H1	-0.305430525	EIF3L	-0.290165289	ESRRA	0.325267477
GNA11	0.239166625	DERL1	0.37281578	EEF2	-0.332343321	EIF4E	-0.26778028	FAM107B	0.190093556
GNB4	-0.273055798	DHX37	-0.226898727	EHD1	-0.301038526	EIF4H	-0.288571798	FAM208B	-0.318468293
GPATCH4	-0.242402923	DLD	0.430238535	EIF3G	-0.236291435	ENAH	-0.523609312	FAM76A	0.293884184
GPBP1	0.316796	DLD	0.430238535	EIF3L	-0.308463073	EPN1	-0.476698301	FDPS	-0.291130113
GPS1	-0.284378992	DSG1	0.426800505	EIF4E	-0.373691748	EPN2	-0.517168956	FKBP15	0.209263078
GTF2A2	-0.211736949	EEF2	-0.22551217	EIF4H	-0.296376124	ESD	-0.362075255	FLG2	0.337704811
GYG1	0.433178399	ELP2	-0.271719205	EIF5B	-0.321863616	FAM103A1	0.220299086	FTSJ3	-0.617704717
H2AFZ	0.362155736	EML4	-0.220614257	ELP2	-0.312629542	FAU	0.216384855	FTSJ3	-0.617704717
HBA1	-0.33977776	ENY2	0.270679714	ENAH	-0.376196558	FKBP7	0.615105832	FUNDC2	0.312458338

HGS	0.496436792	EXOC4	0.265737055	ESD	-0.248403758	FTSJ3	-0.611563656	GANAB	-0.373088929
HIST1H1B	0.65287147	G3BP1	-0.297178916	FAM64A	-0.282979229	G3BP2	-0.302499676	GAR1	0.335758509
HIST1H1D	1.695376646	G3BP2	-0.511404915	FLOT2	0.302428518	GALNT1	0.259033937	GCFC2	-0.233711923
HIST1H2BA	0.985021672	GABARAP	0.257092904	FMR1	-0.269059309	GALNT7	-0.349768784	GINS3	-0.199388725
HIST1H3A	1.296878006	GATAD2A	-0.285028511	FTSJ3	-0.596293715	GCN1L1	-0.319788	GIT2	0.31935434
HSD17B11	0.328687887	GCN1L1	-0.271468672	G3BP2	-0.395895915	GMPS	-0.264398526	GMPS	-0.190171848
HSPA6	0.306894912	GDE1	-0.264369467	GALNT7	-0.323612264	GNA11	0.277022844	GNB2L1	-0.228360994
IKBIP	-0.232878983	GDI1	0.266618846	GAPVD1	-0.351619395	GNL1	-0.275215968	GNL1	-0.437841584
INPP5K	-0.261369812	GMPS	-0.233022649	GCFC2	-0.303105131	GOLIM4	-0.414749648	GNL2	-0.193102383
INTS10	-0.235283693	GNA11	0.32473566	GCN1L1	-0.310150916	GPALPP1	0.188590515	GORASP2	-0.316855189
IST1	0.248809927	GOLIM4	0.284156787	GDE1	-0.277850603	GPBP1	0.18040124	GPALPP1	0.254378711
KAT5	0.296410549	GPALPP1	-0.285202752	GGA2	-0.34557823	GRB10	-0.250578343	GSPT1	-0.354765906
KIN	-0.223379574	GRB10	-0.319535209	GIT2	0.19671156	GSTO1	-0.310612796	GTPBP3	-0.295240749
KRT1	-0.227011698	GRPEL1	0.367547231	GMPS	-0.352708682	H3F3A	-0.289726298	H1F0	0.402194778
KRT10	-0.332392279	GTF3C2	-0.226898727	GNB2L1	-0.353988796	HACD3	-0.270408769	H1FX	0.386885714
KRT2	-0.314826862	GYG1	0.381484859	GNL1	-0.279620782	HADHB	0.174725733	H2AFX	0.404632594
KRT2	-0.27831157	H2AFY	0.343643482	GOLIM4	-0.438297209	HBA1	0.366690792	H2AFZ	0.433160354
KRT9	-0.212483973	H2AFZ	0.772739503	GORASP2	-0.382812138	HBB	0.589252257	H3F3A	-0.35625838
LAMTOR2	0.303209351	H3F3A	-0.305293771	GPALPP1	-0.261938523	HDDC2	0.162352969	HARS	-0.307486908
LDB1	0.331143433	HINT1	0.263183319	GPBP1	0.228004349	HDGFRP3	0.29422599	HBB	0.861101917
LIG4	-0.268060206	HIST1H1B	0.719495803	GPS1	-0.235118279	HEATR3	-0.271892174	HDGFRP3	0.336514044
LRRC1	0.373920795	HIST1H1E	0.391808731	GSTO1	-0.264279496	HGS	0.214586707	HEATR3	-0.188131875
LSM12	-0.277368045	HIST1H2BA	0.725680343	H3F3A	-0.343005918	HGS	0.214586707	HEXIM1	0.223836251
LYAR	-0.249959647	HIST1H2BJ	0.370357936	HACD3	-0.306234528	HINT1	-0.325213863	HGS	0.224229136
MAGI1	-0.306430634	HIST1H2BL	0.47860332	HADHB	0.232032248	HIST1H1C	0.2508332	HIST1H1C	0.380380473
MAGI3	-0.255519998	HIST1H3A	0.40598354	HAGH	-0.263844491	HIST1H1D	0.665861176	HIST1H1D	0.734135217
MEPCE	0.66262566	HIST2H2AB	0.59814944	HARS	-0.2675812	HIST1H3A	0.217401531	HIST1H1E	0.710057979
METAP2	-0.265478657	HIST3H2BB	0.520173478	HBB	0.381890114	HK1	-0.261938898	HIST1H2AB	0.363465557
MGAT2	-0.250821894	HK1	-0.223991811	HEATR3	-0.334638998	HM13	-0.266744039	HIST1H2BK	0.452290288
MIF	0.426783883	HMGA1	0.298404214	HGS	0.338163344	HMGB1	0.200608955	HIST1H3A	0.60745177
MPP7	-0.259065956	HMGN2	0.29489128	HIST1H1C	0.353483835	HMGB2	0.167922719	HIST1H4A	-0.277273704
MRPL28	0.260104406	HMGN5	0.27606369	HIST1H1D	1.04834744	HNRNPD	0.270000704	HIST2H2AB	0.358533007
MRPS17	0.296374466	HNRNPA2B1	0.238005971	HIST1H3A	0.513162211	HNRNPM	-0.250298289	HIST2H2AC	0.649704717
MT-ATP6	0.320491414	HNRNPC	0.237173163	HIST1H4A	-0.385897884	HSPE1	0.166213591	HIST2H3PS2	0.312141124
MT-ATP8	0.243017244	HNRNPDL	0.263204306	HIST2H2AC	0.387184449	HUWE1	-0.449659965	HIST3H2BB	0.210243836
MTMR1	-0.621246409	HPRT1	0.321677766	HK1	-0.361732958	INA	0.318944783	HM13	-0.221316316
NBN	-0.27380469	HSD17B10	0.317771694	HLA-C	0.291162711	INPP5K	-0.319484305	HMGA1	0.345414976
NCKAP1	0.362999721	HSD17B12	0.277894772	HN1L	-0.282857874	INTS6	0.475437723	HMGA2	0.521071709
NDUFA4	0.348403454	HSPA9	0.273997563	HNRNPA1L2	0.26204341	IRF2BP1	-0.272459007	HMGB2	0.353506823
NEDD4	-0.264900045	HSPD1	0.441124661	HNRNPC	0.235023281	ISCA1	-0.26762552	HMGN1	0.472286199
NEK7	-0.356300459	HSPE1	0.290345746	HSD17B11	0.253036318	ISCA2	0.182178241	HMGN2	0.423436644

NFXL1	0.45266145	HUWE1	0.28127957	HSPE1	0.249074142	IST1	0.333981423	HMGN3	0.43004497
NOLC1	0.291768502	IDH3A	0.247929581	HSPH1	-0.291779478	JAGN1	-0.264693431	HMGN4	0.378835564
NOLC1	-0.252577233	INTS4	-0.274289571	HZGJ	-0.323722907	JMJD6	-0.292029837	HN1L	-0.406048196
NPC1	0.822075641	IPO4	0.316817663	IARS	-0.350401699	KAT5	0.349668339	HNRNPC	0.370567093
NRDE2	0.710485678	IRF2BP1	0.265710806	ILKAP	-0.292374717	KCMF1	0.175737339	HNRNPD	-0.20995832
NTPCR	0.376307366	IRF2BP1	-0.303579979	INA	0.36297199	KIAA1211	0.263236731	HNRNPM	-0.241581154
OGFOD3	-0.438018407	ISCA1	-0.27346846	INTS6	0.357710933	KIF2C	0.260991804	HSPE1	0.213669644
OPA3	0.363978484	ISG20L2	0.258644927	IPO4	-0.300362092	KIN	-0.341478433	HUWE1	-0.471365495
ORC1	-0.233126486	ITPK1	-0.451631266	ISCA1	-0.338659784	KPNA2	-0.316688919	IDE	-0.311814383
PAGR1	-0.699972463	KIAA0101	-0.405707371	IST1	0.291846673	KPRP	0.258826853	INA	0.289905853
PALLD	-0.299893602	KPNA2	-0.253508369	ITPK1	0.239839426	KRT10	-0.337016947	INTS10	-0.290810933
PAPD5	-0.251213884	KRT10	-0.505846927	JMJD6	-0.434028495	KRT2	-0.27051424	INTS6	0.399092741
PCCA	0.270951963	KRT2	-0.571130788	JMJD6	-0.434028495	LAMTOR3	-0.272541025	ISCA1	-0.631264704
PDE7B	0.732153179	LBR	0.304225494	KAT5	0.301983271	LARP4	-0.267602059	ITPK1	0.251086859
PEX16	-0.542415816	LDB1	0.277862711	KCMF1	0.247177504	LIG4	-0.291278377	JADE3	-0.340068403
PFKFB2	-0.34331902	LMNB1	0.307486462	KIAA0101	-0.262658237	LSM7	0.179194973	JMJD6	-0.213418266
PFKL	0.36261375	LRRC1	0.375761296	KIAA0415	-0.288156297	LYRM2	0.333898972	KAT5	0.585683559
PFKP	-0.933801233	LRRC59	-0.259663836	KIN	-0.340457074	MANBAL	-0.277196252	KCMF1	0.242644502
PGM1	0.253764465	LSM7	0.230221912	KNSTRN	0.264876589	MAPRE2	0.196463537	KIAA0415	-0.196783706
POLR1E	-0.298391118	LYAR	-0.295894807	KPNA2	-0.327704235	MARCKS	0.203311961	KIF18B	-0.777383072
POLR2G	-0.274414203	MAGI3	-0.288195999	KRT10	-0.436654749	MARCKSL1	0.166862138	KIN	-0.265423885
POLR2G	-0.274414203	MAPRE2	0.235494712	KRT2	-0.499771989	MAZ	0.274551869	KPNA2	-0.243902071
POLR2K	-0.334293116	MDH2	0.565411432	LAMP2	-0.256908694	MB	0.268792804	KRT10	-0.200021903
POP1	-0.480293841	MED4	-0.364741794	LAMTOR3	-0.246558826	MCFD2	-0.361677197	KRT10	-0.200021903
POTEKP	0.722163395	MEPCE	0.556926861	LDB1	0.269764507	MCM7	-0.271698507	KRT2	-0.194565943
PPP1R14B	0.260681259	METAP2	-0.381440352	LIG4	-0.260407541	MED15	-0.302379316	LIG1	0.220874669
PPP2CB	-0.229162592	MIF	0.624630213	LYRM2	0.358050221	MEPCE	-0.281876657	LYPLA1	0.327415774
PPP6R3	0.44182277	MMACHC	-0.350892115	MAGI1	-0.255653543	MMACHC	-0.416485789	MAGI1	-0.292138509
PRKAR2B	-0.214305901	MORF4L1	-0.235430435	MAP1LC3B2	-0.368250626	MPP7	-0.296750329	MAP1LC3B2	0.295726177
PRKCSH	0.345806011	MRPS2	0.242235041	MAP4K4	0.269742061	MPST	-0.325213863	MARCKSL1	0.213659916
PRR14L	-0.430807924	MRPS24	-0.261560602	MAPRE2	0.202478987	MRPL23	0.260659529	MED8	-0.204140564
PRSS1	0.230798917	MT-ATP6	0.299122441	MARCKSL1	0.218360817	MTMR1	-0.730178296	MEPCE	0.631385217
RAB18	0.590458164	MT-ATP8	0.316346883	MCFD2	-0.340889608	MYDGF	0.162060855	MIS12	0.295540552
RAB35	-0.483292704	MT-CO3	-0.281546649	MCM7	-0.316313468	MYL12A	-0.283133344	MMACHC	-0.386293538
RDH14	-0.255595774	MTMR1	-1.030854449	MED22	-0.280330071	NARS	-0.301769883	MMS22L	-0.315419354
RHOT2	0.47823089	MYDGF	0.237432046	MED4	-0.525194529	NCL	0.182428199	MPST	-0.380907602
RNF40	-0.229769076	NAA25	-0.227490125	MEPCE	0.446444427	NDUFA8	0.287186726	MPV17	-0.489894452
RPLP1	0.371943536	NDUFA4	0.398376823	METAP2	-0.268181013	NDUFS6	0.259114059	MRPL23	0.326726691
RRBP1	0.405231334	NEDD4	-0.265369479	MIS12	0.234832725	NEDD4	-0.509910996	MRPS14	-0.292369223
RRBP1	-0.289908274	NEK7	-0.327239935	MLXIP	-0.243381617	NEFM	0.341532439	MRPS2	-0.193985137
SCYL1	-0.263308476	NENF	0.321936007	MMACHC	-0.319117108	NEK7	-0.359347582	MTERF3	0.310976667

SDHC	0.696110174	NHP2L1	0.264724468	MRPL23	0.301988834	NENF	0.202472442	MTMR1	-0.389546598
SEC24B	-0.24934264	NKX2-5	-0.305289084	MRPS21	0.235002644	NFXL1	0.370200403	NAA40	0.320773086
SLC12A4	-0.258730479	NOLC1	0.449328652	MT-ATP6	0.313816042	NIPSNAP1	0.278662723	NARS	-0.248130711
SLC25A3	0.243782123	NOLC1	-0.325320639	MT-CO3	-0.315968396	NR2C2AP	0.201274431	NCL	0.336121314
SLC25A4	0.228159549	NPC1	1.284694053	MTMR1	-0.613499941	NUCKS1	0.219333411	NDUFB1	-0.393995449
SLC25A5	0.268138883	NPM1	0.232101459	MXRA7	-0.241142093	NUF2	-0.280767133	NECAP1	0.229762954
SLC25A6	0.311686561	NSMCE1	0.286740891	MYH9	-0.31107568	NUP98	-0.275598991	NEK7	-0.241449854
SLC39A7	0.248649064	NT5C2	-0.262866807	MYL12A	-0.289801979	ORC1	-0.30252977	NEK9	0.20279355
SMARCA2	0.251712116	NTPCR	0.312498578	NAE1	-0.326780154	OSBP	-0.411835469	NENF	0.250400574
SNTB2	0.233325473	NUCB1	-0.343680533	NARS	-0.491178533	PAFAH1B1	-0.265298454	NFKBIB	0.231311422
SPCS1	0.273666399	NUP98	-0.237005586	NCKAP1	0.36306266	PAFAH1B2	-0.277577086	NFYA	0.286723516
SPTY2D1	-0.282763761	OAT	0.229058357	NDE1	0.242629984	PAFAH1B3	-0.286052047	NHP2L1	0.295879549
SUB1	-0.2537187	OGFOD3	-0.336075491	NEFM	0.341935424	PAGR1	-0.633955817	NME3	0.363214261
SUMO1	-0.309873695	OR1M1	0.245106299	NEK7	-0.43613965	PALLD	-0.266317459	NUCB1	-0.298173401
TAF12	-0.244093663	ORC1	-0.386602299	NOC3L	0.258644975	PDE7B	-0.261527872	NUP98	-0.210698314
TCEB3	-0.300096738	OSBP	-0.260363141	NT5C2	-0.353806158	PEX16	-0.62561786	OMA1	-0.340822428
TCOF1	-0.582391813	OSTC	0.353526173	NTPCR	0.249867221	PFKFB2	-0.278408867	ORC1	-0.312745454
TP53I11	0.24337319	P4HB	0.287704719	NUF2	-0.323597974	PFKP	-1.230178296	OSBP	-0.224894387
TRIP6	-0.367218785	PAF1	0.245794345	NXF1	-0.290915556	PHPT1	0.244380139	PAGR1	-0.511592794
TUBA1B	0.362507855	PAGR1	-0.902370725	OGFOD3	-0.360483478	PLCG1	-0.308275649	PALLD	-0.211137624
TUBA1C	0.356290656	PAIP2	0.233698589	OSBP	-0.408847496	PLD3	-0.304045924	PALM	0.462942701
TWISTNB	-0.276773608	PAPD5	-0.280297955	OSTC	0.236088335	PLOD2	-0.318947388	PAPD5	-0.341795452
UAP1	0.317689442	PBX1	0.316852562	PAFAH1B1	-0.293895571	PNISR	0.191167821	PCGF6	0.316250516
UBE2A	-0.839495701	PDE7B	-0.374846657	PAGR1	-0.886030979	PODXL2	0.263533868	PCM1	-0.314917969
UBE2E1	-0.310424338	PDHB	0.341621686	PDCD5	-0.259408228	POLR2K	-0.438444014	PEX3	0.391162413
UNC45A	0.268841344	PEX16	-0.259649747	PDE7B	-0.335327919	POP1	-0.463568129	PHF20L1	0.391547024
VPS26B	0.297528861	PFAS	-0.226618252	PEX16	-0.419401723	POTEKP	-0.343763157	PJA1	0.196515248
VPS4A	0.295095984	PFKFB2	-0.319003557	PFKFB2	-0.405799538	PPIC	-0.324956011	PKN2	-0.321919142
WDHD1	-0.360503667	PFKL	0.298529111	PFKM	-0.310851328	PPP1R13B	-0.393295953	PLAA	-0.23122803
YBX1	-0.284928326	PFKP	-1.12894572	PFKP	-0.777850603	PPP1R9A	-0.286159181	PLS3	-0.331392324
YTHDF2	-0.3157826	PGM1	0.350395895	PLAA	-0.299063135	PPP2CB	-0.354273633	PNISR	0.235759622
YTHDF3	-0.345485934	PHB	0.524222193	PLCG1	-0.332209926	PPP4R2	0.162753913	POLE3	0.352248521
ZC3H4	-0.303256648	PHB2	0.367943816	PLOD2	-0.289075948	PPP5C	-0.368011128	POTEKP	-0.468783627
ZCCHC3	-0.279740086	PLAA	-0.262888804	POLR2K	-0.353565903	PPP6R3	0.408300184	PPAT	-0.200032684
ZFHX4	0.246935407	POLR1A	-0.258880114	POP1	-0.348011969	PQBP1	0.260832761	PPHLN1	0.344516585
ZNF503	-0.258264013	POLR1C	-0.29313799	POTEKP	-0.316462724	PROSER2	0.375119583	PPP2CB	-0.256092231
ZNF770	0.497310683	POLR1E	-0.303422569	PPP1R13B	-0.266014188	PRPF39	0.195508547	PPP3CA	-0.454328729
ZRANB2	-0.310009016	POLR2F	0.274582424	PPP1R9A	-0.27696714	PRR14L	-0.645649407	PPP5C	-0.408701465
		POLR2F	-0.263914048	PPP6R3	0.366154933	PSAP	-0.286233969	PRC1	0.340267361
		POLR2K	-0.446183615	PQBP1	0.299071878	PSMB7	-0.328265683	PROSER2	0.408360073
		POLR2L	-0.280718245	PRKDC	-0.36182495	PSMC4	-0.310410632	PRR14L	-0.565406217

		POP1	-0.400697063	PROCR	-0.359878152	PSMD12	0.339632732	PRSS1	0.513636754
		POTEKP	0.724123032	PROSER2	0.358887678	PSMD7	-0.269179552	PSIP1	0.320295876
		PPA2	0.247929581	PRR14L	-0.544866854	PTGES3	-0.334970252	PSMD12	0.313266873
		PPAT	-0.219622028	PRSS1	0.423864461	PTMA	0.284582309	PSMD6	-0.293658736
		PPIC	0.2967391	PSMB2	-0.274628975	PTMS	0.293317198	PSMG1	-0.406048196
		PPIF	0.309755155	PSMC4	-0.236619523	PWP1	-0.268519286	PSMG3	0.194765011
		PPP1R14B	0.269623333	PSMD13	-0.36413763	PYURF	-0.313713957	PTGES3	-0.436513501
		PPP6R3	0.413093838	PSMD14	-0.361751033	RAB10	-0.273965803	PTRH2	-0.332401114
		PRCC	-0.264025246	PSMD7	-0.293940806	RAB18	0.364109621	PTRH2	-0.332401114
		PRDX3	0.325831527	PTGES3	-0.331765911	RAB5B	-0.321977974	RAB10	-0.212275932
		PRDX6	0.298576641	PTMS	-0.279547393	RANBP1	0.20224131	RAB35	-0.193127411
		PRKAR2B	-0.25324239	PTPN18	0.218450641	RAP1GDS1	-0.335875421	RAI1	-0.328559346
		PRKCSH	0.426105562	PYURF	-0.290552412	RAPGEF3	-0.289479999	RAP1GDS1	-0.194321481
		PRR14L	-0.227527284	RAB18	0.393644252	RDH14	-0.40579057	RAPGEF3	-0.193753429
		PRSS1	0.282055657	RAB35	-0.286394283	RHOT2	0.430380388	RBMX	0.302624957
		PSAP	0.29038553	RABL3	0.303687989	ROMO1	-0.262014305	RCN2	-0.252510891
		PSIP1	0.270410468	RABL6	-0.302967081	RPP38	0.277329561	RCOR3	0.323430632
		PSMD12	0.357519266	RAP1GDS1	-0.329252954	RRM1	-0.258873791	RDH14	-0.327248996
		PSMD13	-0.223663317	RAPGEF3	-0.349225864	RXFP3	1.161003313	RECQL4	-0.292228752
		PTGES3	0.22745667	RDH14	-0.293018688	SAPCD2	-0.484252573	REEP5	-0.194414383
		RAB18	0.931693147	RHOT2	0.255273391	SCYL1	-0.456282278	RIC8A	-0.292138509
		RABL3	0.381974067	RMDN1	-0.233088838	SDF4	0.187127839	RNF114	0.249348253
		RAP1GDS1	-0.223456446	RPL26L1	0.294988125	SDHC	0.319133814	RPL26	0.355459096
		RAP2C	0.272894707	RPL39	-0.347246007	SEC61B	-0.406889442	RPL26L1	0.501164862
		RAPGEF3	-0.352631921	RPLP1	0.406276582	SF3B3	-0.268333853	RPL27	0.296288302
		RHOT2	0.41491212	RPN1	-0.374766022	SIGMAR1	0.314761295	RPL32	0.296842484
		RNF40	-0.289219122	RPS15A	0.323438284	SIPA1L3	0.277329561	RPS27A	0.433563692
		RNMTL1	0.305780778	RPS29	0.219399704	SLC12A4	-0.260314503	RPS29	0.216586466
		ROMO1	-0.287405341	RRM1	-0.28861352	SLC25A10	0.391207117	RSL1D1	-0.222766262
		RPL11	0.247149903	RSAD1	-0.274323224	SLC25A3	0.313638596	RSRC1	0.293548133
		RPLP1	0.2657226	RXFP3	0.764157225	SLC25A5	0.291013238	RXFP3	1.02710619
		RPS15	0.231670062	SAFB	-0.375975387	SLC25A6	0.345630116	SAPCD2	-0.361847958
		RPS29	0.295645218	SAPCD2	-0.365472634	SLC39A7	0.381822541	SBF1	-0.210698314
		RRBP1	-0.377975515	SCYL1	-0.491774253	SMAP	0.298609489	SCYL1	-0.336625609
		RSAD1	-0.269073471	SDF4	0.210366611	SMIM12	-0.306626776	SEC11A	-0.212127052
		RXFP3	0.332539234	SDHC	0.507188371	SNAP29	-0.275438806	SEC24B	-0.327542014
		SAE1	0.274420481	SEC24C	-0.34849231	SNX32	-0.281146701	SEC61B	-0.382865568
		SAPCD2	-0.227535116	SENP1	0.34324141	SOD1	0.241425765	SETMAR	-0.312549744
		SBF1	-0.230725513	SERF2	0.254973119	SPTLC1	0.286351043	SH3GLB1	0.470449981
		SCP2	0.254175489	SF3B3	-0.355907492	SRC	0.323849012	SLBP	0.194013607
		SCYL1	-0.303595689	SIX4	-0.269750526	SRRM1	0.168246824	SNRPF	0.330069042

		SDHC	0.503253997	SLC12A4	-0.349878479	SS18	0.263533868	SNX18	0.192131666
		SEC61B	0.252307588	SLC25A10	0.293123688	STAT5B	0.171827587	SNX32	-0.341948944
		SF3B3	-0.28289445	SLC25A3	0.232901238	STMN1	0.22712901	SRM	-0.404918359
		SF3B4	0.233423838	SLC35F6	-0.259993262	STX18	0.275553045	SS18	0.286614614
		SHMT2	0.325394028	SMAP	0.273881889	SUB1	0.172843263	SSBP1	0.316786529
		SLC25A3	0.400649567	SMARCA2	0.238666526	SURF4	-0.307823846	SSR2	0.321169619
		SLC25A5	0.63341201	SMN1	-0.290282959	TAF12	-0.282400719	SURF4	-0.261767519
		SLC25A6	0.308575662	SNRPF	0.215381044	TCEB3	-0.269918683	SYPL1	0.43447506
		SLC5A6	0.280192385	SNX32	-0.454265883	TEX10	-0.266530718	TACO1	0.304595423
		SLIRP	0.293587164	SPAG9	-0.270474226	TFCP2	0.261802291	TAF7	0.393611813
		SMAP	0.242512344	SPAST	-0.24182774	THOP1	-0.276764547	TANC1	0.213666027
		SNRPC	0.238638004	SPCS1	0.235028598	TIMM23B	0.31448747	THOP1	-0.218940958
		SNRPF	0.485901548	SPTLC1	0.258114045	TMEM97	0.269815747	TLE4	-0.278916246
		SNX32	-0.405406163	SSBP1	0.234271774	TMSB10	0.296292755	TMOD2	0.285702026
		SOD1	0.262857893	STAU2	0.254758676	TOP1	-0.266891162	TMSB10	0.596894905
		SP3	-0.25761403	SUB1	-0.274426534	TPP1	0.282205659	TNPO1	-0.24151057
		SPCS1	0.258816093	SUMO1	-0.243908992	TRIP6	-0.378068353	TRUB1	0.348103669
		SRP9	0.226891728	SURF4	-0.418655697	TRMT1	-0.294583748	TXN2	-0.54321532
		SSBP1	0.250213098	TAF12	-0.267705652	TRUB1	0.246521159	UBA52	0.204647915
		SSBP3	-0.265497688	TARS	-0.335840107	TSNAX	0.326473458	UBE2A	-0.824959839
		STARD7	-0.270753846	TCEB3	-0.303176188	TSR1	-0.267347678	UBE2D3	-0.235646007
		SUB1	-0.294724151	TCOF1	-0.296797933	TUBA1C	0.315782949	UBQLN2	-0.206008651
		SUMF2	0.32372617	TFCP2	0.237522123	TUBB2A	-0.250716593	UBXN1	-0.304996298
		SUMO2	0.294173095	THOP1	-0.384116197	TWISTNB	-0.465514395	UMPS	0.406546816
		TAF15	0.227736067	TIMM23B	0.276149061	TXN2	-0.515007866	UPRT	0.311199245
		TARBP1	0.247526931	TMEM192	0.243328084	UBE2A	-0.892186016	VDAC1	-0.203075968
		TBC1D22A	-0.237435771	TRABD	0.269746753	UBE2Q1	0.352671912	VDAC3	-0.353606114
		TBL1X	-0.31221521	TRIP6	-0.372678099	UBQLN2	-0.486822208	VIM	0.509646957
		TCEB3	-0.355949751	TRMT1	-0.332269444	UBXN1	-0.303402151	VPS26B	0.446219236
		TCOF1	-0.344333319	TRUB1	0.320402465	UMPS	-0.261758593	VPS4B	-0.25895361
		TFG	0.27496231	TSPO	0.276014529	UNC45A	-0.254807182	VPS52	0.229762954
		THOP1	-0.262709858	TSR1	-0.340591252	UNC45A	0.41764516	VTN	0.601159672
		TIMM23B	0.259423732	TTC1	-0.251806347	UQCR10	0.528803227	WARS2	0.320965195
		TMSB10	0.28265838	TUBA1C	0.307700993	VDAC3	-0.326791003	WDR18	-0.194414383
		TNIK	0.531770542	TUBB2A	-0.345043927	VIM	0.413561921	XPOT	-0.218310365
		TRAP1	0.319894477	TWISTNB	-0.293398643	VKORC1L1	-0.282036171	XRCC1	-0.219378357
		TRIP6	-0.386335947	TXN2	-0.407522873	VPS4A	0.285761947	YWHAB	-0.261109976
		TRMT1	-0.295944519	UBAP2L	-0.239026733	VPS4B	-0.275188547	ZCCHC3	-0.316855189
		TSPYL1	0.291178656	UBE2A	-0.849961025	VTN	0.801930718	ZFHX4	0.548190039
		TUBA1B	0.39546636	UBE2E1	-0.338544375	WLS	-0.365833819	ZNF503	0.357190649
		TUBA1C	0.471246419	UBQLN2	-0.533395195	XPOT	-0.27881383	ZNF770	0.317782013

		TUBB	0.312926441	UBXN1	-0.297552402	XRCC1	-0.269213737		
		TUBB4A	0.293671533	UCHL1	-0.37154306	YWHAB	-0.351949839		
		TUBB4B	0.4437489	UGDH	-0.33201802	ZC3H4	-0.373634222		
		TUFM	0.267032784	UNC45A	-0.344025899	ZCCHC3	-0.4459702		
		TWISTNB	-0.510116131	UPRT	0.328623828	ZNF687	0.271599153		
		UAP1	0.362792125	VARS	-0.300088406	ZNF770	0.377211323		
		UBE2Q1	0.328502496	VDAC3	-0.3044461	ZNF787	-0.256020733		
		UNC45A	0.281457635	VIM	0.567393962				
		UQCRC1	0.566006472	VKORC1L1	-0.24555434				
		USMG5	0.260163341	VPS4A	0.233847304				
		VAPA	0.240333618	VPS4B	-0.289591132				
		VARS	-0.224041817	VTN	0.463820195				
		VDAC1	0.500569412	WASL	-0.29162164				
		VDAC2	0.309128037	WDHD1	-0.258315821				
		VIM	0.337014238	WDR1	-0.342584339				
		VPS26B	0.281157136	XPO1	-0.288833023				
		VPS4B	-0.264477595	XPOT	-0.434711182				
		WARS	0.286364406	YKT6	-0.355732125				
		WDHD1	-0.350371352	YWHAQ	-0.263782379				
		WDR74	-0.281001088	ZC3H4	-0.315802816				
		YBX1	-0.262427682	ZC3HAV1	-0.333918517				
		YTHDF2	-0.332185987	ZCCHC3	-0.319705956				
		YTHDF3	-0.283151122	ZFHX4	0.353352324				
		ZCCHC3	-0.294115599	ZNF770	0.384808739				
		ZRANB2	-0.261714359	ZYX	-0.474780634				
