# Supplementary material for: The RXFP3 receptor is functionally associated with cellular responses to oxidative stress and DNA damage
Source: Aging (Albany NY). 2019 Dec 3;11(23):11268–313. doi: 10.18632/aging.102528 (PMC6932917; doi:10.18632/aging.102528)
Supplement: Supplementary Table 2 [file aging-11-102528-s012..pdf]

Table S2. Enrichr-based PPI Hub Protein enrichment analysis (0.5µg RXFP3). Hub Protein-Protein Interaction enrichment analysis was performed using the Enrichr (http://amp.pharm.mssm.edu/Enrichr/) functional annotation suite with the 0.5µg perturbagen level of RXFP3 expression. For each enriched target PPI hub protein the overlap protein identity from the input dataset with the Enrichr-curated hub data (Overlap), the probability of PPI hub enrichment (P-value), cumulated Z-score (Z-score), Combined ranking score (Combined Score) and the protein identities from the input dataset that overlap with the Enrichr-curated PPI Hub dataset (Proteins) are detailed.

Term	Overlap	P-value	Z-score	Combined Score	Proteins
GABARAP	10/479	6.76E-08	-1.33564	22.05072	NEDD4;KRT2;KRT1;HSPA6;CALR;KRT10;GYG1;GABARAP;KRT9;FTSJ3
GABARAPL1	10/499	9.9E-08	-1.28259	20.68569	NEDD4;KRT2;KRT1;HSPA6;CALR;KRT10;GYG1;GABARAP;KRT9;FTSJ3
GABARAPL2	10/539	2.02E-07	-1.25339	19.31853	PRKCSH;NEDD4;KRT2;KRT1;HSPA6;CALR;KRT10;GABARAP;KRT9;FTSJ3
MAP1LC3B	7/322	6.04E-06	-1.35199	16.24769	NEDD4;KRT2;KRT1;HSPA6;KRT10;GABARAP;KRT9
MAP1LC3A	7/383	1.86E-05	-1.38571	15.09327	NEDD4;KRT2;KRT1;HSPA6;KRT10;GABARAP;KRT9
ALB	5/164	2.92E-05	-1.57998	16.49446	KRT1;KRT10;HBA1;GABARAP;KRT9
MDM2	5/197	7.01E-05	-1.56364	14.9581	KRT2;KRT1;UBE2A;KRT10;KRT9
RPS6KA3	6/375	0.000161	-1.79306	15.65603	PFKFB2;HIST1H3A;PRKAR2B;NEDD4;HIST1H1D;NOLC1
NEDD4	4/141	0.000258	-1.67209	13.81472	HGS;NEDD4;UBE2E1;GABARAP
PRKCZ	4/156	0.000379	-1.16772	9.198523	PPP2CB;PRKCSH;KRT10;GABARAP
YWHAQ	4/188	0.000765	-1.67716	12.03429	PFKFB2;KRT1;GABARAP;KRT9
HIST1H3A	3/122	0.002444	-1.49499	8.991068	HIST1H3A;CBX1;CHD3
CHD3	3/132	0.003054	-1.67741	9.714177	HIST1H3A;ATPIF1;CHD3
APC	3/150	0.004373	-1.53853	8.35783	HGS;KRT1;KRT9
SMAD3	4/328	0.005754	-1.1972	6.175001	HGS;NEDD4;KRT2;KRT10
PRKCB	4/338	0.00639	-1.1559	5.840841	PFKFB2;PPP2CB;HIST1H1D;NOLC1
MAPK14	5/552	0.007032	-1.10582	5.481799	HIST1H3A;NOLC1;RRBP1;UBE2A;CHD3
ESR2	4/361	0.008026	-1.14015	5.501261	HSPA6;NOLC1;RRBP1;FTSJ3
PRKCE	3/193	0.008764	-0.45225	2.142379	PRKAR2B;KRT1;HIST1H1D
CDK2	5/675	0.015814	-0.90944	3.771282	HIST1H1D;NOLC1;RRBP1;UBE2A;CHD3
APP	3/247	0.016984	-1.40734	5.735579	HGS;NEDD4;CALR
YWHAZ	4/500	0.023826	-1.0341	3.864411	PFKFB2;NOLC1;CALR;GABARAP
CLTC	2/120	0.028666	-1.24766	4.431718	HGS;GABARAP
C1ORF103	2/122	0.029552	-1.16796	4.113067	CBX1;CHD3
UBC	4/540	0.030466	-0.85906	2.9991	HGS;NEDD4;UBE2E1;UBE2A
TUBB	2/125	0.030902	-1.23014	4.277123	HGS;GABARAP
CDH1	2/127	0.031815	-1.28861	4.44291	KRT1;KRT9
IL7R	2/127	0.031815	-1.19995	4.137224	HSPA6;CALR
MED19	2/127	0.031815	-1.1011	3.79638	NOLC1;POLR2G
BTK	2/128	0.032275	1.706575	-5.85944	H2AFZ;CBX1
TRAF6	4/550	0.032281	-1.01787	3.494609	PPP2CB;PRKCSH;UBE2E1;CALR
HNRNPA1	2/130	0.033204	-1.16396	3.963383	CBX1;GABARAP

ARRB2	3/323	0.033964	-0.92202	3.118695	NEDD4;HSPA6;NOLC1
UBB	2/135	0.035573	-1.09521	3.653822	HGS;NEDD4
TOP1	2/136	0.036054	-1.41127	4.68927	HIST1H1D;GABARAP
TCP1	2/139	0.037513	-1.73702	5.702743	PPP2CB;GABARAP
XRCC6	2/141	0.038499	-1.02116	3.326057	NEDD4;GABARAP
UBA1	2/144	0.039995	-1.74521	5.617823	HGS;NEDD4
NPM1	2/144	0.039995	-1.14444	3.683938	HIST1H3A;GABARAP
PPP2R1A	2/146	0.041005	-0.98917	3.159461	PPP2CB;GABARAP
AKT1	3/355	0.043012	0.483723	-1.52192	PFKFB2;PALLD;KRT10
EP300	3/357	0.043614	-0.81789	2.561937	HIST1H3A;GPBP1;HIST1H1D
RIF1	2/157	0.046729	-1.09916	3.367133	CBX1;CHD3
RPA2	2/162	0.049423	-1.74402	5.244836	KIN;CBX1
