# Supplementary material for: The RXFP3 receptor is functionally associated with cellular responses to oxidative stress and DNA damage
Source: Aging (Albany NY). 2019 Dec 3;11(23):11268–313. doi: 10.18632/aging.102528 (PMC6932917; doi:10.18632/aging.102528)
Supplement: Supplementary Table 3 [file aging-11-102528-s016..pdf]

Table S3. Enrichr-based PPI Hub Protein enrichment analysis (1.0µg RXFP3). Hub Protein-Protein Interaction enrichment analysis was performed using the Enrichr (http://amp.pharm.mssm.edu/Enrichr/) functional annotation suite with the 1.0µg perturbagen level of RXFP3 expression. For each enriched target PPI hub protein the overlap protein identity from the input dataset with the Enrichr-curated hub data (Overlap), the probability of PPI hub enrichment (P-value), cumulated Z-score (Z-score), Combined ranking score (Combined Score) and the protein identities from the input dataset that overlap with the Enrichr-curated PPI Hub dataset (Proteins) are detailed.

Term	Overlap	P-value	Z-score	Combined Score	Proteins
GABARAPL2	37/539	9.87E-25	-1.28536	71.0488	SF3B4;SLC25A3;PRKCSH;HIST1H2BL;RPL11;ATP5A1;PHB;PHB2;HSD17B10;GABARAP;HSPD1;ACAT1;PRDX3;ATP5B;C1QBP;PAIP2;HSPA9;TRAP1;NPM1;CHTOP;TAF15;MDH2;KRT2;KRT10;HSPE1;EEF2;TUFM;NEDD4;HNRNPA2B1;VDAC1;VIM;SSBP1;CALR;SNRPF;HNRNPC;SLC25A5;SNRPC
MAP1LC3A	31/383	7.47E-23	-1.43472	73.09616	SLC25A3;OAT;SF3B3;RPL11;ATP5A1;GMPS;GABARAP;PRDX3;ATP5B;C1QBP;KPNA2;HSPA9;TRAP1;NPM1;CHTOP;TAF15;MDH2;KRT2;KRT10;EEF2;SRP9;TUFM;NEDD4;HNRNPA2B1;CALU;VIM;SSBP1;HNRNPC;SLC25A5;SNRPC;DLD
GABARAPL1	34/499	1.29E-22	-1.26686	63.85361	SLC25A3;SF3B3;HIST1H2BL;RPL11;ATP5A1;PHB;HSD17B10;GABARAP;HSPD1;PRDX3;ATP5B;C1QBP;ACADM;GYG1;HSPA9;TRAP1;NPM1;TAF15;MDH2;KRT2;KRT10;HSPE1;EEF2;DDB1;NEDD4;HNRNPA2B1;VDAC2;VIM;SSBP1;CALR;HNRNPC;SLC25A5;SNRPC;DLD
MAP1LC3B	28/322	1.59E-21	-1.35199	64.75072	VARS;RPL11;ATP5A1;GMPS;PHB;PDHB;PHB2;HSD17B10;GABARAP;HSPD1;PRDX3;ATP5B;C1QBP;HSPA9;TRAP1;NPM1;TAF15;KRT2;KRT10;EEF2;TUFM;NEDD4;HNRNPA2B1;VIM;SSBP1;HNRNPC;SLC25A5;SNRPC
GABARAP	32/479	4.9E-21	-1.27295	59.53037	SLC25A3;SF3B3;HIST1H2BL;RPL11;ATP5A1;PDHB;HSD17B10;GABARAP;HSPD1;PRDX3;ATP5B;C1QBP;GYG1;HSPA9;TRAP1;NPM1;TAF15;MDH2;KRT2;KRT10;HSPE1;EEF2;TUFM;DDB1;NEDD4;HNRNPA2B1;VIM;SSBP1;CALR;HNRNPC;SLC25A5;SNRPC
SLC2A4	33/635	2.36E-18	-1.13288	45.98103	SLC25A3;OAT;VARS;SHMT2;RPL11;ATP5A1;GOLIM4;PHB;PDHB;PHB2;HSD17B10;HSPD1;ACAT1;RPS15;PRDX3;ATP5B;C1QBP;ATP5D;ACADM;IDH3A;HSPA9;MDH2;HSPE1;EEF2;CS;UQCRC1;VDAC2;VDAC1;VIM;SSBP1;CALR;SLC25A5;DLD
DLG4	22/409	9.26E-13	-1.31001	36.29752	HSPA9;SLC25A3;ACOT7;SF3B3;ATP5A1;PDHB;PHB2;HSPD1;TUFM;ACAT1;SOD1;CS;PRDX3;ATP5B;PRKAR2B;ATP5D;UQCRC1;VDAC2;VDAC1;SLC25A5;DLD;IDH3A
231403	19/288	1.05E-12	-1.52438	42.05041	TRAP1;NPM1;OAT;PSMD13;SHMT2;GMPS;HSPE1;PFAS;TUFM;ACAT1;LMNB1;SMAP;CS;HINT1;GRPEL1;VAPA;DDT;VDAC1;DLD
HNRNPK	14/188	2.35E-10	-1.54974	34.35685	HSPA9;RPL11;ATP5A1;GABARAP;HSPD1;ACAT1;PRDX3;DDB1;MORF4L1;ATP5B;NEDD4;C1QBP;SUMO2;GCN1L1
EIF2C2	13/156	2.63E-10	-1.53924	33.9548	HSPA9;SLC25A3;NPM1;SF3B3;RPL11;PHB;PHB2;HSPD1;DDB1;C1QBP;GCN1L1;HNRNPC;SLC25A5
YWHAB	26/812	7.39E-10	-0.98941	20.80325	SLC25A3;ATP5A1;PHB;PDHB;PHB2;HSPD1;ACAT1;HK1;ATP5B;PRKAR2B;C1QBP;SBF1;LBR;IDH3A;HSPA9;MDH2;EEF2;CS;VAPA;NEDD4;UQCRC1;VDAC2;VDAC1;SLC25A5;DLD;MAPRE2
TRAF6	21/550	1.76E-09	-1.31038	26.41176	HSPA9;NPM1;OAT;VARS;PSMD13;PRKCSH;PTGES3;HSPE1;PHB2;PFAS;TUFM;CS;DDB1;CARHSP1;NHP2L1;GCN1L1;VDAC2;VDAC1;CALR;HNRNPC;SLIRP
MCC	15/292	8.88E-09	-1.38768	25.7261	NPM1;OAT;VARS;PSMD13;CNBP;PTGES3;PDHB;PHB2;PFAS;TUFM;ACAT1;SMAP;CS;RNMTL1;VDAC1
YWHAZ	19/500	1.21E-08	-1.16041	21.15859	PFKFB2;HSPA9;TRAP1;NPM1;SF3B3;VARS;ATP5A1;NOLC1;EEF2;GABARAP;HSPD1;LMNB1;DDB1;ATP5B;GCN1L1;VIM;SSBP1;CALR;HNRNPC
PRKCE	12/193	3.61E-08	-0.73396	12.57855	ATP5B;PRKAR2B;HNRNPA2B1;ATP5A1;VDAC1;PHB;VIM;PDHB;SLC25A5;HSPD1;TUFM;IDH3A
TNFRSF1B	10/124	4.47E-08	-1.46163	24.73473	HSPA9;DDB1;SLC25A3;C1QBP;ATP5A1;GCN1L1;VIM;HNRNPC;SLC25A5;TUFM
IKBKE	17/454	9.21E-08	0.920136	-14.9065	HSPA9;NPM1;PRKCSH;SHMT2;PTGES3;GMPS;PDHB;HSPE1;PHB2;PFAS;SMAP;DDT;GCN1L1;VDAC1;HNRNPC;THOP1;DLD
EPB41	11/172	1.02E-07	-1.46924	23.64568	SCP2;NHP2L1;VARS;CALR;SLIRP;HSPE1;KPNA2;PHB2;PFAS;SRP9;TUFM
H2AFX	11/172	1.02E-07	-1.37814	22.17959	HSPA9;PRDX3;TRAP1;NPM1;PRKCSH;HNRNPA2B1;SSBP1;CALR;HNRNPC;HSD17B10;SUMF2
EIF2C1	11/174	1.15E-07	-1.38075	22.05908	DDB1;SLC25A3;NPM1;SF3B3;RPL11;MRPS2;PHB;HNRNPC;SLC25A5;PHB2;HSPD1
MED19	9/127	6.5E-07	-1.305	18.59178	HSPA9;DDB1;NPM1;GCN1L1;POLR2F;NOLC1;KPNA2;MED4;HSPD1
SRRM2	10/166	6.9E-07	-1.414	20.06024	SF3B4;SF3B3;HNRNPA2B1;VIM;SNRPF;PDHB;HNRNPC;SNRPC;GABARAP;HSPD1
SGK1	9/135	1.09E-06	-0.27475	3.772836	CS;CARHSP1;VARS;NEDD4;ATP5D;KPNA2;LBR;PFAS;TUFM
TNFRSF1A	9/173	8.39E-06	-1.29764	15.16738	HSPA9;TRAP1;SLC25A3;VAPA;C1QBP;ATP5A1;GCN1L1;SEC61B;SLC25A5
SNCA	12/328	9.9E-06	-1.22009	14.05957	HSPA9;SLC25A3;VAPA;HNRNPA2B1;HIST2H2AB;UQCRC1;VDAC2;VDAC1;PHB;PHB2;COX6B1;HK1
GRB2	18/767	2.87E-05	-0.73902	7.729985	SF3B4;SLC25A3;NPM1;SHMT2;ATP5A1;ISG20L2;EEF2;SRP9;HSPD1;TUFM;HIST1H3A;CALD1;HNRNPA2B1;GRB10;SSBP1;HNRNPC;HMGN2;DLD
EIF1B	8/159	3.42E-05	-1.18293	12.16296	OAT;DDT;PSMD13;SHMT2;RPS29;ATP5D;CALR;ACAT1
GC20	8/165	4.46E-05	-1.42819	14.30779	OAT;DDT;PSMD13;SHMT2;RPS29;ATP5D;CALR;ACAT1
ARRB2	11/323	4.51E-05	-0.98739	9.880413	NPM1;SF3B3;NEDD4;C1QBP;RPL11;HIST2H2AB;NOLC1;VIM;HNRNPC;EEF2;LBR
YWHAE	9/215	4.7E-05	-1.16462	11.60644	ATP5B;NPM1;HNRNPA2B1;ATP5A1;GRB10;PHB;VIM;HNRNPC;GABARAP
CDK1	16/659	5.49E-05	-0.19992	1.961283	NPM1;GMPS;HMGA1;BUB1B;NOLC1;EEF2;LMNB1;CARHSP1;HMGN5;CALD1;GRB10;HIST1H1E;NUP98;VIM;SSBP1;LBR
IKBKG	11/332	5.78E-05	-0.91935	8.971986	HSPA9;DDB1;ATP5B;VARS;PSMD13;RPL11;GCN1L1;VIM;SLC25A5;HSPD1;IDH3A

ACTB	11/339	6.96E-05	-0.92049	8.811216	MORF4L1;NPM1;RPL11;HNRNPA2B1;ATP5A1;VDAC2;VDAC1;PHB;VIM;GABARAP;HSPD1
HNRNPA1	7/130	6.98E-05	-1.1127	10.64819	HSPA9;NHP2L1;HNRNPA2B1;CBX1;HMGA1;HNRNPC;GABARAP
YWHAG	12/428	0.00013	-1.1246	10.05882	PFKFB2;HSPA9;ATP5B;SLC25A3;SF3B3;TAF15;C1QBP;ATP5A1;NOLC1;VIM;LBR;HSPD1
MYC	13/498	0.000139	-0.87867	7.804427	GTF3C2;OAT;SF3B3;HIST1H2BL;RPL11;HSPD1;C1QBP;GCN1L1;DHX37;HNRNPC;KPNA2;LBR;IDH3A
VHL	10/314	0.000174	-0.98523	8.528519	HINT1;CARHSP1;GRPEL1;VARS;DDT;PSMD13;PTGES3;HNRNPA2B1;HSPE1;PFAS
ESR1	14/591	0.00021	-0.67278	5.696299	HSPA9;NPM1;PTGES3;RPL11;MRPS2;PSIP1;PHB;MED4;PHB2;HSPD1;ATP5B;HNRNPA2B1;SEC61B;SLC25A5
ARRB1	9/263	0.000217	-0.91457	7.714603	HSPA9;ATP5B;NPM1;CALD1;NEDD4;ATP5A1;HIST2H2AB;NOLC1;VIM
MAP3K14	7/166	0.000318	9.519152	-76.6675	HSPA9;NPM1;C1QBP;RPL11;ATP5A1;GRB10;HIST1H1E
RELA	9/283	0.000372	-0.83413	6.586596	HSPA9;NPM1;ATP5A1;GCN1L1;PHB;VIM;SLC25A5;KPNA2;PHB2
HDAC1	10/346	0.000377	-0.77406	6.101682	MORF4L1;NPM1;HIST1H3A;H2AFY;SUMO2;BUB1B;PHB;NUP98;PHB2;HSPD1
HIST1H3A	6/122	0.000379	-0.63277	4.984163	CUL4A;DDB1;NPM1;HIST1H3A;CBX3;CBX1
CASP3	7/193	0.000782	-0.93344	6.678033	NEDD4;PTGES3;PSIP1;VIM;HSPE1;HSPD1;LMNB1
CDK2	14/675	0.000789	0.664398	-4.74655	NPM1;RPL11;GMPS;HMGA1;NOLC1;EEF2;LMNB1;HMGN5;CALD1;NAA25;HIST1H1E;NUP98;VIM;LBR
NPM1	6/144	0.00091	-0.96195	6.735332	HIST1H3A;CNBP;SUMO2;HMGA1;NUP98;GABARAP
GRIN2B	7/219	0.001624	-0.84513	5.428116	PRKAR2B;ATP5A1;VDAC2;VDAC1;SLC25A5;PHB2;LMNB1
ATM	7/222	0.001755	0.988745	-6.27406	CUL4A;DDB1;HINT1;C1QBP;HMGA1;VIM;LMNB1
PRKAB1	7/223	0.0018	-0.98057	6.197148	DDB1;OAT;GRPEL1;RNMTL1;SHMT2;GMPS;GCN1L1
MEPCE	6/165	0.001829	-0.82899	5.225862	LSM7;NHP2L1;HNRNPA2B1;SNRPF;HNRNPC;KPNA2
RPS6KA3	9/375	0.002656	1.164299	-6.9055	PFKFB2;CARHSP1;HIST1H3A;PRKAR2B;NEDD4;NOLC1;VIM;HMGN2;LBR
IL7R	5/127	0.003122	-0.56736	3.273334	PRDX3;ATP5B;CALR;HSPD1;SOD1
NFKB2	6/184	0.003151	-0.79747	4.593341	HSPA9;PSMD13;RPL11;KRT10;HNRNPC;LMNB1
PHLDA3	5/129	0.003338	-0.70939	4.045178	CUL4A;GCN1L1;SNRPF;LBR;MAPRE2
EGFR	10/467	0.003544	2.042726	-11.526	HSPA9;ATP5B;SLC25A3;HIST1H3A;VAPA;RAP1GDS1;GRB10;HIST1H1E;SLC25A5;HSPD1
CHD3	5/132	0.003683	-0.60514	3.391258	HIST1H3A;ATPIF1;SUMO2;VIM;KPNA2
TOP1	5/136	0.004181	-0.85136	4.663	NPM1;H2AFY;HNRNPA2B1;HNRNPC;GABARAP
CSNK1E	6/195	0.004187	1.649408	-9.03198	NPM1;C1QBP;OSBP;PSIP1;HNRNPC;EEF2
MDM2	6/197	0.004399	-0.68472	3.715589	NPM1;CTBP2;KRT2;RPL11;VIM;KRT10
MAPK13	5/139	0.004586	5.868867	-31.6027	TAF15;MDH2;ATP5A1;HIST1H1E;EEF2
XRCC6	5/141	0.00487	-0.48735	2.59496	NHP2L1;NEDD4;SUMO2;HMGA1;GABARAP
SMARCA4	5/144	0.00532	-0.42806	2.241449	NPM1;PHB;HNRNPC;KPNA2;LMNB1
HSPA1A	5/145	0.005477	-0.46582	2.425643	CUL4A;PTGES3;SUMO2;HMGA1;GABARAP
RUVBL1	5/147	0.005799	-0.55687	2.867919	MORF4L1;HINT1;NHP2L1;NEDD4;SUMO2
HDAC2	6/214	0.006538	-0.57457	2.890178	MORF4L1;NPM1;HIST1H3A;CTBP2;SUMO2;PHB2
AKT1	8/355	0.00657	2.126199	-10.6847	PFKFB2;NPM1;CARHSP1;GRB10;VIM;KRT10;LBR;PHB2
EP300	8/357	0.006787	-0.40965	2.045283	GTF3C2;NPM1;HIST1H3A;CTBP2;HIST2H2AB;NUP98;HMGN2;KPNA2
ESR2	8/361	0.007238	-0.32658	1.609532	PRDX3;GTF3C2;SF3B3;TAF15;MRPS2;NOLC1;VIM;MED4
RIF1	5/157	0.007613	-0.55936	2.728511	GTF3C2;NHP2L1;CBX1;VIM;HSPE1
MAP3K3	6/227	0.008621	17.84139	-84.8097	HSPA9;DDB1;C1QBP;ATP5A1;VIM;SLC25A5
ATG12	5/165	0.009324	-1.08853	5.089035	CHTOP;RNMTL1;RPS29;GABARAP;LMNB1
POLR2A	5/165	0.009324	-0.40202	1.879497	CBX3;NEDD4;PAF1;POLR2F;MED4
PRKACB	5/174	0.011546	2.677364	-11.9447	CALD1;CBX3;PRKAR2B;VIM;LMNB1

MAP3K1	5/184	0.014411	7.165928	-30.3818	HSPA9;DDB1;VIM;SNRPF;SLC25A5
C1ORF103	4/122	0.015054	0.240897	-1.01083	NHP2L1;CBX1;VIM;HSPE1
BTK	4/128	0.01765	7.025622	-28.3626	ATP5B;H2AFZ;CBX1;ATP5A1
PRKACA	8/440	0.021566	2.863102	-10.9847	PFKFB2;CBX3;PRKAR2B;NOLC1;VIM;HMGN2;THOP1;HSPD1
HSP90AB1	4/138	0.022563	-0.06679	0.253234	SUMO2;PHB;EEF2;GABARAP
RB1	5/209	0.023565	-0.36082	1.352341	TRAP1;GTF3C2;MORF4L1;CBX1;PHB
PARP1	4/140	0.023636	-0.05826	0.218186	NPM1;H2AFY;HMGA1;BUB1B
EWSR1	5/215	0.026214	-0.45485	1.656331	TAF15;HMGA1;SNRPC;PHB2;GABARAP
BRCA1	5/216	0.026673	-0.36129	1.309348	NPM1;H2AFY;PSAP;SUMO2;KPNA2
PRKCA	9/547	0.026926	3.328533	-12.0315	PFKFB2;NPM1;C1QBP;HMGA1;PLAA;VIM;HMGN2;EEF2;LMNB1
PPP2R1A	4/146	0.027039	-0.03423	0.123601	EEF2;PHB2;GABARAP;HSPD1
COPS6	4/147	0.027633	-0.07127	0.255753	CUL4A;DDB1;PSAP;VIM
MAPK14	9/552	0.028318	3.362418	-11.9845	HIST1H3A;CALD1;GRB10;PLAA;PSIP1;NOLC1;VDAC1;NUP98;LBR
APC	4/150	0.029462	-0.00871	0.0307	DDB1;BUB1B;NUP98;MAPRE2
CSNK2A1	9/564	0.031863	3.42335	-11.7979	NPM1;PTGES3;HNRNPA2B1;CBX1;OSBP;HMGA1;PSIP1;NOLC1;HNRNPC
PRKCZ	4/156	0.033332	4.543463	-15.4535	PRKCSH;C1QBP;KRT10;GABARAP
IRS1	4/157	0.034004	0.039556	-0.13375	NPM1;GRB10;PHB;VIM
HSP90AA1	5/231	0.034185	-0.13752	0.464254	CUL4A;PRR14L;CALD1;PTGES3;GABARAP
RUVBL2	4/161	0.036772	0.006092	-0.02012	MORF4L1;HINT1;NHP2L1;UQCRC1
SUMO2	4/162	0.037484	0.030432	-0.09993	HSPA9;NPM1;SUMO2;VIM
ARF6	4/163	0.038204	-0.04926	0.160837	CS;OAT;RPS29;DLD
ALB	4/164	0.038932	0.112281	-0.36446	DDB1;GCN1L1;KRT10;GABARAP
RAF1	4/166	0.040411	16.88481	-54.1776	GRB10;VDAC1;PHB;HSPD1
IKBKB	4/169	0.042689	7.810461	-24.6328	HSPA9;PRDX3;C1QBP;HSPD1
APP	5/247	0.043517	-0.20453	0.641107	OAT;NEDD4;CALU;CALR;HSD17B10
PRKCB	6/338	0.048282	4.287613	-12.9944	PFKFB2;HMGA1;NOLC1;VIM;LBR;LMNB1
