# Supplementary material for: The RXFP3 receptor is functionally associated with cellular responses to oxidative stress and DNA damage
Source: Aging (Albany NY). 2019 Dec 3;11(23):11268–313. doi: 10.18632/aging.102528 (PMC6932917; doi:10.18632/aging.102528)
Supplement: Supplementary Table 4 [file aging-11-102528-s002..pdf]

Table S4. Enrichr-based PPI Hub Protein enrichment analysis (2.0µg RXFP3). Hub Protein-Protein Interaction enrichment analysis was performed using the Enrichr (http://amp.pharm.mssm.edu/Enrichr/) functional annotation suite with the 2.0µg perturbagen level of RXFP3 expression. For each enriched target PPI hub protein the overlap protein identity from the input dataset with the Enrichr-curated hub data (Overlap), the probability of PPI hub enrichment (P-value), cumulated Z-score (Z-score), Combined ranking score (Combined Score) and the protein identities from the input dataset that overlap with the Enrichr-curated PPI Hub dataset (Proteins) are detailed.

Term	Overlap	P-value	Z-score	Combined Score	Proteins
GABARAP	23/479	4.22E-13	-1.33564	38.05886	SF3B3;DHX9;PRKDC;KRT2;CLTC;CHD4;KRT10;HSPE1;EEF2;FTSJ3;HADHB;DDB1;NXF1;HIST1H4A;HSPH1;TUBB2A;DHX57;DHX15;BRIX1;SSBP1;IARS;SEC24C;HIST1H1C
GABARAPL1	22/499	7.71E-12	-1.28259	32.81979	SF3B3;DHX9;PRKDC;KRT2;RPN1;CLTC;KRT10;HSPE1;EEF2;FTSJ3;HADHB;DDB1;NXF1;HIST1H4A;HSPH1;CSRP2;DHX15;MYH9;BRIX1;SSBP1;SEC24C;HIST1H1C
MAP1LC3B	18/322	1.51E-11	-1.36816	34.08513	DYNC1H1;VARS;DHX9;PRKDC;KRT2;CLTC;GMPS;MAP1LC3B2;KRT10;EEF2;SAFB;HADHB;HSPH1;TUBB2A;SSBP1;IARS;SEC24C;HIST1H1C
GABARAPL2	20/539	1.53E-09	-1.2374	25.11788	DYNC1H1;MCM7;DHX9;PRKDC;KRT2;RPN1;CLTC;KRT10;HSPE1;EEF2;FTSJ3;HADHB;HIST1H4A;HSPH1;DHX15;SSBP1;SNRPF;IARS;SEC24C;HIST1H1C
MAP1LC3A	17/383	2E-09	-1.38571	27.75425	DYNC1H1;SF3B3;DHX9;PRKDC;COPB1;KRT2;CLTC;GMPS;KRT10;EEF2;SAFB;HADHB;HSPH1;TUBB2A;DHX15;SSBP1;KPNA2
IKBKE	18/454	3.86E-09	-0.91764	17.7779	RRM1;PSMD14;PTGES3;RPN1;CLTC;GMPS;TARS;HSPE1;SMAP;NARS;ACLY;UCHL1;TUBB2A;GCN1L1;VDAC3;MYH9;IARS;THOP1
MYC	18/498	1.62E-08	-1.33004	23.86196	UNC45A;DYNC1H1;SF3B3;MCM7;PRKDC;RPN1;CHD4;IPO4;CDK9;HADHB;XPO1;HSPH1;XPOT;DHX15;GCN1L1;AP2S1;HEATR3;KPNA2
YWHAZ	17/500	1.01E-07	-1.25513	20.22389	PFKFB2;DYNC1H1;SF3B3;VARS;DHX9;PRKDC;CLTC;EEF2;DDB1;HIST1H4A;XPO1;HSPH1;CAND1;GCN1L1;MYH9;SSBP1;IARS
IKBKG	14/332	1.09E-07	-1.29757	20.8052	DYNC1H1;MCM7;VARS;PSMD13;PRKDC;RPN1;CLTC;GNB2L1;DDB1;XPO1;GCN1L1;MYH9;IARS;MAP4K4
MCC	13/292	1.7E-07	-1.43473	22.36383	RRM1;PSMD14;MCM7;VARS;PSMD13;PTGES3;CLTC;TARS;SMAP;UCHL1;AP2S1;IARS;PFKM
231403	12/288	1.04E-06	-1.47742	20.35018	RRM1;MCM7;DHX9;PSMD13;RPN1;GMPS;VDAC3;TARS;BANF1;IARS;HSPE1;SMAP
TRAF6	16/550	1.91E-06	-1.31038	17.25256	DYNC1H1;PSMD14;MCM7;VARS;DHX9;PSMD13;PTGES3;UBE2E1;TARS;HSPE1;DDB1;UCHL1;GCN1L1;HEATR3;IARS;RPL39
VHL	12/314	2.56E-06	-1.35949	17.5025	ACLY;UCHL1;MCM7;VARS;PSMD13;PTGES3;RAB35;TARS;IARS;HSPE1;COX5A;GNB2L1
GSTK1	8/122	2.58E-06	-1.68027	21.62071	UNC45A;DDB1;MCM7;RPN1;VDAC3;HEATR3;IARS;IPO4
SLC2A4	17/635	2.76E-06	-0.98659	12.62999	DYNC1H1;VARS;COPB1;DCTN4;RPN1;CLTC;HSPE1;EEF2;COX5A;GNB2L1;HADHB;ACLY;CSRP2;CAND1;SSBP1;YKT6;PAFAH1B1
TNFRSF1B	8/124	2.92E-06	-1.46163	18.629	DDB1;XPO1;XPOT;PRKDC;COPB1;CLTC;GCN1L1;MYH9
PRKAB1	10/223	4.47E-06	-1.49126	18.36838	DDB1;ACLY;RRM1;PSMD14;MCM7;PRKDC;CLTC;GMPS;GCN1L1;TARS
NFKB2	9/184	6.78E-06	-1.4143	16.83198	HADHB;DYNC1H1;DHX9;PSMD13;PRKDC;CLTC;MYH9;KRT10;IARS
YWHAB	18/812	1.84E-05	-0.85919	9.367861	DYNC1H1;PSMD14;PRKDC;DCTN4;RPN1;CLTC;GAPVD1;EEF2;HK1;HADHB;ACLY;KIAA0415;HSPH1;DHX15;VDAC3;MYH9;PFKM;MAPRE2
POLR2A	8/165	2.38E-05	-1.41845	15.10036	CDK9;DHX9;PRKDC;SMN1;MED4;SAFB;CDC73;PQBP1
HGS	7/122	2.66E-05	-1.29839	13.67782	GGA2;ACLY;SF3B3;TUBB2A;CLTC;PFKM;ILKAP
CHD3	7/132	4.41E-05	-1.45298	14.57094	HSPH1;HIST1H3A;ATPIF1;CHD4;KPNA2;SAFB;SERF2
MAP3K1	8/184	5.18E-05	0.969168	-9.56426	HADHB;DDB1;HIST1H4A;HSPH1;DHX9;UBE2E1;SNRPF;MAP4K4
PTP4A3	7/137	5.59E-05	-1.4677	14.37087	UNC45A;MCM7;SURF4;GCN1L1;AP2S1;HEATR3;IPO4
HSP90AB1	7/138	5.86E-05	-1.31601	12.82488	CDK9;XPO1;HSPH1;PRKDC;MYH9;WASL;EEF2
ARF6	7/163	0.000166	-1.38785	12.07798	ACLY;DYNC1H1;DHX9;RPS29;RPN1;TARS;IARS
MEPCE	7/165	0.000179	-1.27137	10.96869	CDK9;XPO1;CAND1;DHX9;SNRPF;KPNA2;CDC73
MAP3K3	8/227	0.000222	5.948641	-50.0508	DDB1;HIST1H4A;TUBB2A;XPOT;PRKDC;CLTC;MYH9;IARS
UBQLN4	7/171	0.000223	-1.19497	10.04734	NXF1;HGS;COPB1;ATPIF1;RPN1;NAE1;UBQLN2
EPB41	7/172	0.000231	-1.2471	10.44112	DYNC1H1;VARS;DHX9;MYH9;IARS;HSPE1;KPNA2

TNFRSF1A	7/173	0.00024	-1.16857	9.742274	HIST1H4A;XPOT;PRKDC;CLTC;GCN1L1;MYH9;GNB2L1
MED19	6/127	0.000295	-1.02463	8.330392	DDB1;GCN1L1;SMN1;KPNA2;MED4;IPO4
ESR1	13/591	0.000307	-0.75695	6.122018	UNC45A;DHX9;PRKDC;PTGES3;RPN1;CLTC;CHD4;ZC3HAV1;MED4;SAFB;FTSJ3;DHX15;MYH9
PHLDA3	6/129	0.00032	-1.23255	9.917216	UNC45A;DYNC1H1;GCN1L1;SNRPF;MAPRE2;IPO4
EEF1A1	7/185	0.00036	-1.185	9.396789	CSRP2;XPOT;VARS;PLCG1;HSPE1;AK6;PQBP1
HNRNPK	7/188	0.000396	-1.07519	8.421843	CDK9;DDB1;XPO1;H3F3A;GCN1L1;SAFB;HIST1H1C
MDM2	7/197	0.000525	-1.05657	7.980342	TUBB2A;CTBP2;PRKDC;KRT2;UBE2A;KRT10;MAP4K4
EIF2C2	6/156	0.000875	-0.96999	6.829817	DDB1;SF3B3;DHX9;DHX15;GCN1L1;IPO4
CHUK	6/157	0.000905	2.123883	-14.8839	TUBB2A;PRKDC;H3F3A;CLTC;MYH9;KRT10
RELA	8/283	0.000953	-0.8502	5.914103	CDK9;HSPH1;DHX9;PRKDC;CLTC;GCN1L1;KPNA2;HIST1H1C
ESR2	9/361	0.001121	-0.76591	5.203138	HIST1H4A;SF3B3;PRKDC;ZC3HAV1;SMN1;MED4;GNB2L1;FTSJ3;HIST1H1C
GRB2	14/767	0.001138	-0.4744	3.215629	DYNC1H1;WDR1;DHX9;PRKDC;WASL;EEF2;HADHB;UGDH;HIST1H4A;HIST1H3A;AP2S1;MYH9;PLCG1;SSBP1
MAP3K7	6/168	0.001283	6.223485	-41.4396	HIST1H4A;TUBB2A;HGS;CLTC;IARS;MAP4K4
HSP90AA1	7/231	0.001332	-0.89671	5.93748	UNC45A;DYNC1H1;UCHL1;PRR14L;PRKDC;PTGES3;WASL
PAK1	6/176	0.001626	1.361711	-8.74406	GIT2;HIST1H4A;HIST1H3A;HGS;H3F3A;PLCG1
C1ORF103	5/122	0.001792	-0.56022	3.542941	RRM1;CBX1;SMN1;HSPE1;PQBP1
HIST1H3A	5/122	0.001792	-0.52152	3.298227	DDB1;HIST1H4A;HIST1H3A;CBX1;IPO4
ARRB2	8/323	0.002194	-0.67691	4.144026	DYNC1H1;SF3B3;TUBB2A;CLTC;DHX15;MYH9;EEF2;HIST1H1C
CSNK2A1	11/564	0.002331	0.947456	-5.74277	EIF5B;MARCKSL1;HSPH1;PTGES3;CBX1;OSBP;MYH9;CHD4;SMN1;DNM1L;PAFAH1B1
PRKCE	6/193	0.002584	1.730998	-10.3137	HADHB;COPB1;HIST1H1D;MYH9;GNB2L1;MAP4K4
CDK1	12/659	0.00262	1.101908	-6.55023	UGDH;ACLY;DYNC1H1;DHX9;GMPS;BUB1B;SSBP1;UBE2A;DNM1L;GAPVD1;EEF2;MAP4K4
DLG4	9/409	0.002624	-0.6038	3.588415	DYNC1H1;ACOT7;SF3B3;TUBB2A;HGS;PRKDC;CLTC;VDAC3;PFKM
CSNK1E	6/195	0.00272	1.337064	-7.8981	EIF5B;PRKDC;OSBP;SMN1;GAPVD1;EEF2
ARRB1	7/263	0.002771	-0.68017	4.005294	HIST1H4A;TUBB2A;H3F3A;CLTC;AP2S1;MYH9;HIST1H1C
SGK1	5/135	0.002786	2.052779	-12.0767	EIF5B;VARS;IARS;KPNA2;IPO4
TSGA14	5/137	0.002968	-0.69361	4.036633	VARS;WASL;IARS;DNM1L;PAFAH1B1
MAPK13	5/139	0.003159	5.486699	-31.5892	EIF5B;DHX9;CLTC;EEF2;HIST1H1C
CDK2	12/675	0.003182	1.482792	-8.52652	EIF5B;ACLY;MARCKSL1;MCM7;GMPS;HIST1H1D;CHD4;ZC3HAV1;UBE2A;DNM1L;EEF2;UBQLN2
PARP1	5/140	0.003258	-0.52675	3.016537	PRKDC;H3F3A;BUB1B;BANF1;HIST1H1C
PRKACA	9/440	0.004241	1.737049	-9.48937	PFKFB2;EIF5B;ACLY;H3F3A;CLTC;CAPN2;PLCG1;DNM1L;THOP1
APC	5/150	0.00437	-0.55358	3.007579	DDB1;XPO1;HGS;BUB1B;MAPRE2
RIF1	5/157	0.005294	-0.70469	3.693416	RRM1;CBX1;SMN1;HSPE1;PQBP1
MAPK14	10/552	0.006101	1.873006	-9.55099	EIF5B;ACLY;HIST1H3A;DHX9;COPB1;CLTC;PLAA;CHD4;UBE2A;UBQLN2
SNCA	7/328	0.009058	-0.60085	2.826464	UCHL1;CLTC;VDAC3;IARS;COX5A;COX6B1;HK1
PRKCB	7/338	0.010569	2.057739	-9.36239	PFKFB2;MARCKSL1;PRKDC;HIST1H1D;PLCG1;SAFB;GNB2L1
CLTC	4/120	0.010607	0.020312	-0.09234	EHD1;GGA2;HGS;CLTC
VCL	4/121	0.01091	-0.04952	0.223732	HADHB;CLTC;MYH9;GNB2L1
YWHAG	8/428	0.011506	-0.60179	2.686897	PFKFB2;GIT2;DYNC1H1;RRM1;SF3B3;PRKDC;CLTC;DHX15
HNRNPA1	4/130	0.013896	-0.21565	0.922142	CDK9;CBX1;CAPN2;SAFB
EP300	7/357	0.013933	-0.36066	1.541283	HIST1H4A;HIST1H3A;CTBP2;GPBP1;HIST1H1D;CHD4;KPNA2
COPS5	4/132	0.014623	-0.08451	0.357083	DDB1;UCHL1;MYH9;GNB2L1

UBC	9/540	0.015072	-0.105	0.440455	DDB1;UCHL1;XPO1;HGS;UBE2E1;PLCG1;UBE2A;SMN1;DNM1L
TOP1	4/136	0.016149	-0.44416	1.832566	DHX9;PRKDC;HIST1H1D;HIST1H1C
NR3C1	5/209	0.016826	-0.44872	1.832948	EIF5B;PRKDC;PTGES3;CLTC;KPNA2
RPS6KA3	7/375	0.017772	2.814916	-11.3444	PFKFB2;EIF5B;ACLY;XPO1;HIST1H3A;HIST1H1D;PFKM
XRCC6	4/141	0.018192	-0.15372	0.615931	HIST1H4A;DHX9;PRKDC;CLTC
HDAC2	5/214	0.01844	-0.38111	1.521852	HIST1H3A;CTBP2;CHD4;BANF1;SMN1
EGFR	8/467	0.018512	3.705703	-14.7832	EHD1;DYNC1H1;UCHL1;HIST1H3A;XPOT;HGS;RAP1GDS1;PLCG1
NCOR1	4/142	0.018619	-0.07724	0.3077	HIST1H4A;SF3B3;HIST1H3A;SAFB
BRCA1	5/216	0.019113	-0.39377	1.558304	HIST1H4A;DHX9;PRKDC;KPNA2;MAP4K4
NPM1	4/144	0.019491	-0.16357	0.644107	HIST1H4A;XPO1;HIST1H3A;HIST1H1C
PIN1	4/145	0.019937	-0.1157	0.452992	GGA2;CDK9;DDB1;DHX15
GRIN1	4/149	0.021782	-0.07659	0.293079	CLTC;MYH9;PLCG1;GNB2L1
TP53	8/502	0.027036	-0.06701	0.241947	CDK9;UCHL1;XPO1;PRKDC;UBE2A;SMN1;EEF2;KPNA2
RPA2	4/162	0.028484	-0.76123	2.70877	KIN;TUBB2A;PRKDC;CBX1
APP	5/247	0.031666	-0.39198	1.353303	HADHB;UCHL1;HGS;MYH9;NAE1
IKBKB	4/169	0.032547	6.808929	-23.321	TUBB2A;PRKDC;CLTC;GNB2L1
H2AFX	4/172	0.034388	-0.10489	0.353477	HIST1H4A;DHX9;PRKDC;SSBP1
PRKCD	4/173	0.035014	3.973329	-13.3186	HIST1H3A;PRKDC;DNM1L;GNB2L1
EIF2C1	4/174	0.035648	-0.11208	0.373697	DDB1;SF3B3;DHX9;DHX15
ABL1	6/351	0.039446	4.21983	-13.6419	DDB1;PTPN18;PRKDC;PLCG1;WASL;GNB2L1
HSPA8	4/183	0.041646	-0.15294	0.486134	UCHL1;HSPH1;HGS;CLTC
ATXN1	4/194	0.049707	-0.18654	0.559904	NARS;ACOT7;UBE2E1;PQBP1
