# Supplementary material for: The RXFP3 receptor is functionally associated with cellular responses to oxidative stress and DNA damage
Source: Aging (Albany NY). 2019 Dec 3;11(23):11268–313. doi: 10.18632/aging.102528 (PMC6932917; doi:10.18632/aging.102528)
Supplement: Supplementary Table 5 [file aging-11-102528-s005..pdf]

Table S5. Enrichr-based PPI Hub Protein enrichment analysis (5.0µg RXFP3). Hub Protein-Protein Interaction enrichment analysis was performed using the Enrichr (http://amp.pharm.mssm.edu/Enrichr/) functional annotation suite with the 5.0µg perturbagen level of RXFP3 expression. For each enriched target PPI hub protein the overlap protein identity from the input dataset with the Enrichr-curated hub data (Overlap), the probability of PPI hub enrichment (P-value), cumulated Z-score (Z-score), Combined ranking score (Combined Score) and the protein identities from the input dataset that overlap with the Enrichr-curated PPI Hub dataset (Proteins) are detailed.

Term	Overlap	P-value	Z-score	Combined Score	Proteins
GABARAP	18/479	1.26E-09	-1.33564	27.36617	SF3B3;DHX9;TEX10;KRT2;CHD4;KRT10;HSPE1;FTSJ3;HADHB;DDB1;HNRNPM;TUBB2A;NCL;NEDD4;DHX57;TOP1;FAU;HIST1H1C
GABARAPL1	18/499	2.41E-09	-1.28259	25.4517	SF3B3;DHX9;YWHAB;TEX10;KRT2;KRT10;HSPE1;DDOST;FTSJ3;HADHB;DDB1;HNRNPM;PRPF39;NCL;NEDD4;CFL1;TOP1;HIST1H1C
CDK1	18/659	1.68E-07	-2.02247	31.54549	PNISR;DHX9;GMPS;BUB1B;NUCKS1;HMGB1;UBE2A;SRRM1;MARCKS;CARHSP1;PPP4R2;PALLD;NCL;STMN1;GRB10;NUP98;TOP1;PTMA
231403	12/288	2.86E-07	-1.58699	23.91126	PPP2CB;RANBP1;RRM1;MCM7;DHX9;STMN1;GMPS;VDAC3;BANF1;HSPE1;DDOST;SMAP
MAP1LC3A	13/383	9.21E-07	-1.38571	19.25808	SF3B3;DHX9;KRT2;GMPS;KRT10;HADHB;HNRNPM;PRPF39;TUBB2A;NCL;NEDD4;CFL1;KPNA2
GSTK1	8/122	1.05E-06	-1.91184	26.32357	UNC45A;DDB1;HNRNPM;MCM7;VDAC3;HEATR3;ATXN10;DDOST
PTP4A3	8/137	2.51E-06	-1.87532	24.17949	UNC45A;HNRNPM;MCM7;SURF4;GCN1L1;HEATR3;ATXN10;DDOST
CDK2	16/675	5.39E-06	-1.66488	20.19799	STAT5B;MARCKSL1;MCM7;GMPS;XRCC1;NUCKS1;CHD4;UBE2A;SRRM1;MARCKS;NCL;STMN1;HIST1H1D;NUP98;PTMA;UBQLN2
IKBKE	13/454	5.96E-06	-0.41643	5.009802	RANBP1;RRM1;GMPS;HSPE1;SMAP;NARS;PPP2CB;HNRNPM;TUBB2A;GCN1L1;VDAC3;THOP1;CALM2
TRAF6	14/550	9.82E-06	-1.34118	15.46518	RANBP1;MCM7;DHX9;EIF1AX;HMGB2;HSPE1;RAB10;DDB1;PPP2CB;MARCKS;CARHSP1;GCN1L1;HEATR3;PHPT1
CSNK2A1	14/564	1.3E-05	-1.46495	16.47946	PNISR;MARCKSL1;ANP32A;YWHAB;XRCC1;CHD4;SRRM1;MARCKS;NCL;SUB1;STMN1;OSBP;TOP1;PAFAH1B1
MYC	13/498	1.6E-05	-1.25222	13.83142	UNC45A;SF3B3;MCM7;YWHAB;CHD4;CDK9;HADHB;XPOT;NCL;GCN1L1;HEATR3;TOP1;KPNA2
ESR1	14/591	2.19E-05	-1.0936	11.73436	UNC45A;DHX9;HMGB2;CHD4;HMGB1;LARP4;SRRM1;FTSJ3;HNRNPM;NCL;SEC61B;TOP1;PTMA;CALM2
HNRNPK	8/188	2.56E-05	-1.46186	15.45444	CDK9;DDB1;NEDD4;H3F3A;GCN1L1;TOP1;HMGB1;HIST1H1C
PARP1	7/140	2.99E-05	-1.50835	15.71337	NCL;H3F3A;BUB1B;XRCC1;BANF1;TOP1;HIST1H1C
GABARAPL2	13/539	3.64E-05	-1.04558	10.68812	MCM7;DHX9;KRT2;KRT10;HSPE1;FTSJ3;HADHB;HNRNPM;NCL;NEDD4;TOP1;FAU;HIST1H1C
MAP1LC3B	10/322	3.77E-05	-1.14183	11.63054	HADHB;HNRNPM;TUBB2A;DHX9;NCL;NEDD4;KRT2;GMPS;KRT10;HIST1H1C
NR3C1	8/209	5.44E-05	-1.37585	13.50966	STAT5B;ANP32A;NCL;HMGB2;HMGB1;HBA1;KPNA2;PTMS
PRKCB	10/338	5.66E-05	-0.95915	9.379195	PFKFB2;PPP2CB;MARCKSL1;MARCKS;YWHAB;STMN1;HMGB2;HIST1H1D;PLCG1;SRRM1
RELA	9/283	7.78E-05	-1.17168	11.08554	CDK9;PPP2CB;HNRNPM;DHX9;NCL;GCN1L1;HMGB1;KPNA2;HIST1H1C
MAP3K14	7/166	8.82E-05	4.063327	-37.9345	HNRNPM;TUBB2A;DHX9;NCL;GRB10;FAU;CALM2
EP300	10/357	8.92E-05	-1.14448	10.67213	STAT5B;HIST1H3A;SUB1;GPBP1;HIST1H1D;CHD4;NUP98;KPNA2;PTMA;PTMS
MAPK14	12/552	0.000194	-0.71728	6.130367	HIST1H3A;DHX9;YWHAB;NCL;STMN1;GRB10;CHD4;NUP98;UBE2A;LARP4;SRRM1;UBQLN2
SGK1	6/135	0.000215	-0.20202	1.706213	HNRNPM;MARCKS;CARHSP1;NEDD4;KPNA2;SRRM1
MAPK13	6/139	0.000252	1.410231	-11.6878	MARCKS;DHX9;YWHAB;NCL;STMN1;HIST1H1C
MAPK1	10/406	0.000253	-0.48874	4.047585	PPP2CB;STAT5B;MARCKS;HIST1H3A;NEDD4;STMN1;CAPN2;GRB10;CHD4;SRRM1
ARRB1	8/263	0.000265	-1.1021	9.075375	HNRNPM;TUBB2A;YWHAB;NCL;NEDD4;H3F3A;CFL1;HIST1H1C
CREBBP	9/334	0.00027	-1.0003	8.220667	STAT5B;HIST1H3A;H3F3A;HMGB2;NUP98;HMGB1;KPNA2;PTMA;PTMS
NPM1	6/144	0.000304	-1.34973	10.92951	HNRNPM;HIST1H3A;NCL;NUP98;TOP1;HIST1H1C
APC	6/150	0.000379	-1.20322	9.480153	DDB1;HNRNPM;HGS;BUB1B;NUP98;MAPRE2
PRKDC	8/296	0.000582	-0.13094	0.975403	MARCKSL1;DHX9;YWHAB;HMGB2;XRCC1;TOP1;HMGB1;HIST1H1C

RPS6KA3	9/375	0.000624	-0.14244	1.051099	PFKFB2;PNISR;CARHSP1;HIST1H3A;YWHAB;NEDD4;STMN1;HIST1H1D;SRRM1
MEPCE	6/165	0.000627	-1.15597	8.524458	CDK9;LSM7;ANP32A;DHX9;KPNA2;CDC73
EGFR	10/467	0.000755	0.524356	-3.76947	STAT5B;HIST1H3A;XPOT;HGS;RAP1GDS1;CFL1;GRB10;PLCG1;ATXN10;CALM2
EPB41	6/172	0.000779	-1.15454	8.262885	RAB10;RANBP1;DHX9;YWHAB;HSPE1;KPNA2
EIF2C1	6/174	0.000828	-1.09077	7.740857	DDB1;SF3B3;DHX9;NCL;TEX10;DDOST
VHL	8/314	0.000854	-0.98523	6.960679	RANBP1;CARHSP1;MCM7;EIF1AX;STMN1;HSPE1;PTMA;COX5A
ARRB2	8/323	0.001025	-0.84032	5.784175	HNRNPM;SF3B3;TUBB2A;YWHAB;NCL;NEDD4;CFL1;HIST1H1C
MAP3K1	6/184	0.001105	2.91325	-19.832	HADHB;DDB1;HNRNPM;DHX9;NCL;CALM2
EEF1A1	6/185	0.001137	-1.0977	7.44198	XPOT;YWHAB;PLCG1;HSPE1;SRRM1;PQBP1
IL7R	5/127	0.001262	-0.8573	5.722296	STAT5B;TUBB2A;YWHAB;CFL1;SOD1
PHLDA3	5/129	0.001353	-1.02329	6.759223	UNC45A;HNRNPM;GCN1L1;ATXN10;MAPRE2
GSK3B	12/696	0.001499	0.607531	-3.95059	STAT5B;PNISR;MARCKSL1;MARCKS;PPP4R2;NCL;STMN1;GRB10;CHD4;UBE2A;LARP4;SRRM1
MDM2	6/197	0.001567	-0.93825	6.059745	TUBB2A;NCL;KRT2;NUCKS1;UBE2A;KRT10
TOP1	5/136	0.001709	-1.15677	7.370887	DHX9;NCL;HIST1H1D;TOP1;HIST1H1C
PCNA	5/146	0.00233	-0.88109	5.341064	DDB1;DHX9;SUB1;XRCC1;PTMA
CSNK2A2	7/289	0.002394	0.843219	-5.0888	ANP32A;NCL;OSBP;XRCC1;TOP1;PTMA;CALM2
EIF2C2	5/156	0.003102	-0.76669	4.428283	DDB1;SF3B3;DHX9;GCN1L1;DDOST
CAMK2A	5/160	0.003457	1.334075	-7.56083	YWHAB;STMN1;GPBP1;PLCG1;CALM2
POLR2A	5/165	0.00394	-0.83205	4.60663	CDK9;DHX9;NEDD4;CDC73;PQBP1
SRRM2	5/166	0.004043	-0.85381	4.70526	HNRNPM;SF3B3;PPP4R2;YWHAB;SRRM1
PAK1	5/176	0.005172	1.877645	-9.88483	HIST1H3A;HGS;H3F3A;STMN1;PLCG1
HSPA8	5/183	0.006085	-0.85144	4.343977	YWHAB;HGS;STMN1;SEC61B;SOD1
NFKB2	5/184	0.006224	-0.77985	3.961099	HADHB;HNRNPM;DHX9;NCL;KRT10
HDAC1	7/346	0.006375	-0.56089	2.835513	HIST1H3A;YWHAB;BUB1B;CHD4;BANF1;NUP98;PTMA
ABL1	7/351	0.006877	1.820928	-9.06757	DDB1;STAT5B;YWHAB;NEDD4;GRB10;PLCG1;TOP1
AKT1	7/355	0.007298	1.534908	-7.55192	PFKFB2;PNISR;CARHSP1;PALLD;GRB10;PLCG1;KRT10
C1ORF103	4/122	0.007449	-0.22872	1.120668	RRM1;HMGB1;HSPE1;PQBP1
RPL7A	5/193	0.007578	-1.59724	7.798474	DHX9;YWHAB;NCL;HIST1H1D;TOP1
CASP3	5/193	0.007578	-0.67832	3.311873	NEDD4;TOP1;HMGB1;HSPE1;PTMA
ATXN1	5/194	0.00774	-0.72676	3.533004	NARS;ANP32A;CFL1;PHPT1;PQBP1
TNFRSF1B	4/124	0.00788	-0.2065	1.000177	DDB1;XPOT;GCN1L1;DDOST
MED19	4/127	0.008557	-0.23451	1.116487	DDB1;HNRNPM;GCN1L1;KPNA2
INSR	5/202	0.009125	2.071645	-9.7299	STAT5B;YWHAB;GRB10;PLCG1;CALM2
HNRNPA1	4/130	0.009271	-0.31817	1.489313	CDK9;HNRNPM;CAPN2;TOP1
CHD3	4/132	0.009768	-0.35578	1.646783	HIST1H3A;ATPIF1;CHD4;KPNA2
MCC	6/292	0.010539	-0.54069	2.461588	PPP2CB;RANBP1;RRM1;MCM7;DDOST;SMAP
NEDD4	4/141	0.012218	-0.36685	1.615913	YWHAB;HGS;NEDD4;GRB10
PRKAB1	5/223	0.013556	-0.66138	2.844563	DDB1;RRM1;MCM7;GMPS;GCN1L1
YWHAZ	8/500	0.013931	-0.27629	1.180776	PFKFB2;DDB1;SF3B3;DHX9;YWHAB;NCL;CFL1;GCN1L1
TP53	8/502	0.014239	-0.27091	1.151842	CDK9;NCL;HMGB2;XRCC1;TOP1;HMGB1;UBE2A;KPNA2
MAP3K3	5/227	0.014539	18.40771	-77.8818	DDB1;TUBB2A;XPOT;YWHAB;CALM2

DLG4	7/409	0.015043	-0.27424	1.150929	SF3B3;TUBB2A;YWHAB;HGS;CFL1;VDAC3;SOD1
ATM	3/222	0.016488	6.862538	-12.3701	DDB1;MARCKSL1;CHD4
PRKCZ	4/156	0.017115	3.459062	-14.0708	PPP2CB;YWHAB;NCL;KRT10
RIF1	4/157	0.017479	-0.41404	1.675498	RRM1;HMGB1;HSPE1;PQBP1
CHUK	4/157	0.017479	5.806272	-23.4965	TUBB2A;H3F3A;KRT10;CALM2
EIF1B	4/159	0.018222	-0.2119	0.84869	RANBP1;EIF1AX;HMGB2;FAU
JAK2	4/159	0.018222	4.572681	-18.3142	STAT5B;HIST1H3A;GRB10;PLCG1
YWHAG	7/428	0.018809	-0.42752	1.698709	PFKFB2;HNRNPM;RRM1;SF3B3;YWHAB;CFL1;SRRM1
SLC2A4	9/635	0.01914	0.070003	-0.27693	HADHB;RAB10;PPP2CB;YWHAB;NCL;CFL1;HSPE1;COX5A;PAFAH1B1
CTNNB1	6/337	0.020007	-0.13581	0.531266	HNRNPM;ANP32A;DHX9;YWHAB;CDC73;HIST1H1C
ALB	4/164	0.020164	-0.1349	0.526643	DDB1;GCN1L1;KRT10;HBA1
ACTB	6/339	0.020528	-0.09613	0.373563	MARCKS;YWHAB;NCL;CFL1;VDAC3;PLCG1
GC20	4/165	0.020567	-0.32846	1.275776	RANBP1;EIF1AX;HMGB2;FAU
UBC	8/540	0.021075	0.103573	-0.39975	DDB1;YWHAB;HGS;NEDD4;PLCG1;HMGB1;UBE2A;SOD1
PRKACA	7/440	0.021504	3.459248	-13.2818	PFKFB2;MARCKS;H3F3A;STMN1;CAPN2;PLCG1;THOP1
XRCC6	2/141	0.022295	1.736847	-2.60664	DHX9;NEDD4
PRKCA	8/547	0.022553	3.598488	-13.6451	PFKFB2;PPP2CB;MARCKS;YWHAB;NCL;PLCG1;TOP1;HMGB1
UBQLN4	4/171	0.023089	-0.11868	0.447243	HGS;ATPIF1;STMN1;UBQLN2
TNFRSF1A	4/173	0.02397	-0.09912	0.369818	XPOT;GCN1L1;SEC61B;DDOST
PPP1CA	4/184	0.029178	-0.17725	0.626447	YWHAB;CFL1;CALM2;PAFAH1B1
STAT3	4/192	0.033356	-0.13832	0.470368	CDK9;STAT5B;STMN1;PTMA
CCT3	4/202	0.039046	-0.96894	3.142289	CDK9;PPP2CB;YWHAB;CDC73
CDC42	4/205	0.040854	-0.20485	0.655054	KIN;NEDD4;NUF2;RAP1GDS1
HSPA5	3/121	0.041117	0.812726	-2.59368	CDK9;YWHAB;STMN1
HGS	3/122	0.041966	0.750269	-2.37903	SF3B3;TUBB2A;NEDD4
HIST1H3A	3/122	0.041966	0.841334	-2.66778	DDB1;HIST1H3A;ANP32A
RB1	4/209	0.043339	-0.06335	0.198836	CDK9;MCM7;NCL;HMGB1
XPO1	3/124	0.04369	0.769098	-2.40777	RANBP1;ANP32A;GCN1L1
PIK3CA	3/125	0.044565	28.58356	-88.9179	YWHAB;ATPIF1;GRB10
HDAC2	4/214	0.046563	-0.0103	0.031589	HIST1H3A;CHD4;BANF1;PTMA
IGF1R	4/215	0.047224	4.829429	-14.7436	YWHAB;NEDD4;GRB10;PLCG1
BRCA1	4/216	0.047889	-0.03649	0.110876	DHX9;SUB1;XRCC1;KPNA2
DNM1	3/129	0.048153	0.43293	-1.31324	YWHAB;NEDD4;PLCG1
