# Supplementary material for: The RXFP3 receptor is functionally associated with cellular responses to oxidative stress and DNA damage
Source: Aging (Albany NY). 2019 Dec 3;11(23):11268–313. doi: 10.18632/aging.102528 (PMC6932917; doi:10.18632/aging.102528)
Supplement: Supplementary Table 6 [file aging-11-102528-s009..pdf]

Table S6. Enrichr-based PPI Hub Protein enrichment analysis (10.0μg RXFP3). Hub Protein-Protein Interaction enrichment analysis was performed using the Enrichr (http://amp.pharm.mssm.edu/Enrichr/) functional annotation suite with the 10.0μg perturbagen level of RXFP3 expression. For each enriched target PPI hub protein the overlap protein identity from the input dataset with the Enrichr-curated hub data (Overlap), the probability of PPI hub enrichment (P-value), cumulated Z-score (Z-score), Combined ranking score (Combined Score) and the protein identities from the input dataset that overlap with the Enrichr-curated PPI Hub dataset (Proteins) are detailed.

Term	Overlap	P-value	Z-score	Combined Score	Proteins
GABARAPL1	17/499	6.71E-09	-1.29832	24.4347	NEK9;YWHAB;KRT2;KRT10;HSPE1;EEF2;DDOST;FTSJ3;ACTA2;RSL1D1;HNRNPM;HIST1H4A;RCN2;HNRNPD;BRIX1;SSBP1;HIST1H1C
GABARAPL2	16/539	1.29E-07	-1.26937	20.13554	NEK9;KRT2;KRT10;HSPE1;EEF2;FTSJ3;ACTA2;RSL1D1;HNRNPM;HIST1H4A;RCN2;HNRNPD;VDAC1;SSBP1;SNRPF;HIST1H1C
GABARAP	15/479	1.66E-07	-1.3043	20.35867	NEK9;KRT2;CHD4;KRT10;HSPE1;EEF2;FTSJ3;RSL1D1;HNRNPM;HIST1H4A;RCN2;HNRNPD;BRIX1;SSBP1;HIST1H1C
231403	10/288	8.77E-06	-1.58699	18.47965	PPP2CB;GANAB;DDT;UBE2D3;GMPS;VDAC3;VDAC1;BANF1;HSPE1;DDOST
MAP1LC3A	11/383	1.83E-05	-1.38571	15.11824	ACTA2;NEK9;HNRNPM;KRT2;GMPS;DDX20;HNRNPD;SSBP1;KRT10;EEF2;KPNA2
MAP1LC3B	10/322	2.3E-05	-1.31966	14.09477	ACTA2;NEK9;HNRNPM;KRT2;GMPS;HNRNPD;SSBP1;KRT10;EEF2;HIST1H1C
CDK2	14/675	4.89E-05	-1.72783	17.1487	SLBP;MARCKSL1;LIG1;GMPS;DDX20;XRCC1;CHD4;UBE2A;EEF2;TANC1;HNRNPD;HIST1H1D;NUP98;UBQLN2
TNFRSF1A	7/173	7.97E-05	-1.59266	15.03063	HIST1H4A;RCN2;XPOT;SEC61B;UBA52;DDOST;GNB2L1
MAPK14	12/552	0.000113	-1.62387	14.76341	EEA1;RCN2;HIST1H3A;LIG1;YWHAB;HNRNPD;PLAA;CHD4;VDAC1;NUP98;UBE2A;UBQLN2
CDK1	13/659	0.000152	-1.56683	13.77409	TLE4;SLBP;PNISR;LIG1;GMPS;BUB1B;UBE2A;EEF2;RSL1D1;PALLD;HNRNPD;NUP98;SSBP1
HDAC1	9/346	0.000228	-1.28237	10.75446	TLE4;HIST1H4A;HIST1H3A;YWHAB;HNRNPD;BUB1B;CHD4;BANF1;NUP98
IKBKE	10/454	0.000386	0.084782	-0.6663	NARS;PPP2CB;HNRNPM;DDT;PTRH2;GMPS;VDAC3;VDAC1;HSPE1;THOP1
SLC2A4	12/635	0.000406	-1.0191	7.957158	RAB10;ACTA2;PPP2CB;YWHAB;VDAC1;SSBP1;HSPE1;EEF2;SEC11A;UBA52;COX5A;GNB2L1
ARF6	6/163	0.000434	-1.62407	12.5734	HNRNPM;GANAB;RPS29;UBE2D3;SEC11A;DDOST
GSTK1	5/122	0.000816	-1.65133	11.74225	HNRNPM;VDAC3;HEATR3;VDAC1;DDOST
MAP3K1	6/184	0.000821	0.118633	-0.84287	HNRNPM;HIST1H4A;RCN2;UBE2D3;SNRPF;UBA52
MED19	5/127	0.000978	-1.40695	9.750416	RSL1D1;HNRNPM;DDX20;MED8;KPNA2
PTP4A3	5/137	0.001371	-1.61156	10.62395	HNRNPM;SURF4;DDX20;HEATR3;DDOST
ESR2	8/361	0.001438	-1.12388	7.355163	HIST1H4A;WDR18;MRPS2;GNL2;GNB2L1;NFKBIB;FTSJ3;HIST1H1C
PARP1	5/140	0.001509	-1.39681	9.074378	H3F3A;BUB1B;XRCC1;BANF1;HIST1H1C
NPM1	5/144	0.001708	-1.53222	9.763919	HNRNPM;HIST1H4A;HIST1H3A;NUP98;HIST1H1C
ESR1	10/591	0.002803	-0.94211	5.536714	HEXIM1;RSL1D1;HNRNPM;WDR18;HNRNPD;MRPS2;CHD4;SEC61B;GNL2;FTSJ3
MEPCE	5/165	0.003085	-1.34831	7.794788	HEXIM1;HNRNPD;SNRPF;KPNA2;CDC73
ARRB2	7/323	0.003219	-1.06909	6.134998	HNRNPM;YWHAB;MED8;HNRNPD;EEF2;UBA52;HIST1H1C
H2AFX	5/172	0.003684	-1.26742	7.102197	HIST1H4A;GANAB;YWHAB;HNRNPD;SSBP1
PAK1	5/176	0.004062	-0.03868	0.212994	GIT2;HIST1H4A;HIST1H3A;HGS;H3F3A
ACTB	7/339	0.004193	-1.01941	5.58065	TANC1;YWHAB;HNRNPD;VDAC3;VDAC1;TNPO1;GNB2L1
HSPA8	5/183	0.004788	-1.29919	6.93972	YWHAB;HGS;HNRNPD;SEC61B;NFKBIB
ARRB1	6/263	0.00492	-1.07084	5.69088	HNRNPM;HIST1H4A;YWHAB;H3F3A;UBA52;HIST1H1C

EP300	7/357	0.005539	-1.01385	5.267968	ACTA2;HIST1H4A;HIST1H3A;HIST1H1D;CHD4;NUP98;KPNA2
TRAF6	9/550	0.005621	-1.01787	5.273757	RAB10;PPP2CB;GANAB;RCN2;UBE2D3;HEATR3;VDAC1;HSPE1;SEC11A
PRKCE	5/193	0.00598	0.463304	-2.37181	YWHAB;HNRNPD;HIST1H1D;VDAC1;GNB2L1
MDM2	5/197	0.00651	-1.12418	5.659519	RCN2;KRT2;UBE2A;KRT10;PJA1
CSNK2A1	9/564	0.006591	-0.00481	0.024159	SLBP;PNISR;MARCKSL1;LIG1;YWHAB;OSBP;XRCC1;CHD4;NFKBIB
TUBB	4/125	0.006645	-0.98646	4.946049	YWHAB;HGS;GNB2L1;NFKBIB
RELA	6/283	0.006973	-0.9145	4.54116	HEXIM1;PPP2CB;HNRNPM;KPNA2;NFKBIB;HIST1H1C
CHD3	4/132	0.008026	-1.07894	5.205891	HIST1H3A;ATPIF1;CHD4;KPNA2
MAPK13	4/139	0.009585	3.066296	-14.2509	GANAB;YWHAB;EEF2;HIST1H1C
MYC	8/498	0.009875	-0.83198	3.841845	NEK9;GANAB;XPOT;YWHAB;DDX20;HEATR3;CHD4;KPNA2
VHL	6/314	0.011288	-0.93845	4.207972	DDT;UBE2D3;RAB35;HSPE1;COX5A;GNB2L1
MAP3K3	5/227	0.011581	9.629731	-42.9334	ACTA2;HIST1H4A;XPOT;YWHAB;UBA52
APC	4/150	0.01241	-0.95175	4.17746	HNRNPM;HGS;BUB1B;NUP98
SNCA	6/328	0.013753	-0.93428	4.00483	YWHAB;VPS52;VDAC3;VDAC1;COX5A;COX6B1
CHUK	4/157	0.014459	2.635326	-11.1645	ACTA2;H3F3A;KRT10;NFKBIB
CREBBP	6/334	0.014916	-0.71867	3.022231	ACTA2;HIST1H4A;HIST1H3A;H3F3A;NUP98;KPNA2
PRKCB	6/338	0.015729	0.811633	-3.37013	ACTA2;PPP2CB;MARCKSL1;YWHAB;HIST1H1D;GNB2L1
UBQLN4	4/171	0.01917	-0.86661	3.426952	HGS;ATPIF1;UBA52;UBQLN2
EPB41	4/172	0.019538	-0.91389	3.596491	RAB10;YWHAB;HSPE1;KPNA2
PRKCD	4/173	0.019911	1.262944	-4.94628	ACTA2;HIST1H3A;YWHAB;GNB2L1
EIF2C1	4/174	0.020289	-0.83704	3.262514	RSL1D1;WDR18;MRPS2;DDOST
DYNLL1	4/183	0.023885	-0.85144	3.179714	PPP2CB;NEK9;GNB2L1;FAM107B
EEF1A1	4/185	0.024733	-0.88816	3.285859	XPOT;YWHAB;HSPE1;AK6
CSNK1E	4/195	0.029247	1.337064	-4.72248	RSL1D1;YWHAB;OSBP;EEF2
CSNK2A2	5/289	0.029359	1.288361	-4.54552	SLBP;RSL1D1;LIG1;OSBP;XRCC1
MCC	5/292	0.030502	-0.72891	2.543871	PPP2CB;GANAB;VDAC1;DDOST;NFKBIB
PRKDC	5/296	0.032069	1.491463	-5.13041	MARCKSL1;HIST1H4A;YWHAB;XRCC1;HIST1H1C
CLTC	3/120	0.034906	-0.24508	0.822252	YWHAB;HGS;NFKBIB
DLG4	6/409	0.035615	-0.50964	1.699651	TANC1;YWHAB;HGS;VDAC3;VDAC1;UBA52
TNFRSF1B	3/124	0.037901	-0.28836	0.943729	RCN2;XPOT;DDOST
HDAC2	4/214	0.039091	-0.65518	2.124	HIST1H3A;DDX20;CHD4;BANF1
BRCA1	4/216	0.040225	-0.70233	2.25678	HIST1H4A;UBE2D3;XRCC1;KPNA2
HDAC4	3/127	0.040231	-0.28839	0.926638	NARS;HIST1H4A;YWHAB
GRIN2B	4/219	0.041961	-0.58979	1.870252	TANC1;YWHAB;VDAC1;GNB2L1
UBC	7/540	0.042581	-0.23335	0.736527	RNF114;LIG1;YWHAB;HGS;HNRNPD;UBE2A;PJA1
MAPK9	4/222	0.043738	2.073815	-6.49007	SLBP;RSL1D1;PNISR;H3F3A
PRKCA	7/547	0.045096	2.181223	-6.75954	ACTA2;GIT2;PPP2CB;YWHAB;PLAA;EEF2;GNB2L1
SGK1	3/135	0.046789	2.925603	-8.95852	HNRNPM;GANAB;KPNA2

HSP90AB1	3/138	0.049376	-0.30249	0.909986	YWHAB;EEF2;NFKBIB
TCP1	3/139	0.050254	-0.86379	2.583307	PPP2CB;YWHAB;CDC73
