# Supplementary material for: The RXFP3 receptor is functionally associated with cellular responses to oxidative stress and DNA damage
Source: Aging (Albany NY). 2019 Dec 3;11(23):11268–313. doi: 10.18632/aging.102528 (PMC6932917; doi:10.18632/aging.102528)
Supplement: Supplementary Table 7 [file aging-11-102528-s025..pdf]

Table S7. Cellular Signaling theoretical data set. Biomedical text word items, *i.e.* Gene Symbols, associated with generic 'Cellular Signaling' extraction terms are represented. All proteins identified possess a Cosine Similarity score of association with the interrogator term of >0.1.

Gene Symbol	signaling	cell signaling	cellular signaling	signal transduction	intermediary cell metabolism	signalling	cell signalling	cellular signalling
c6orf120	0.154	0.224	0.353	0.224	0.126	0.197	0.207	0.268
pde1c	0.154	0.201	0.273	0.204	0.122	0.132	0.14	0.186
zmat1	0.154	0.238	0.411	0.162	0.149	0.172	0.184	0.265
mad2l2	0.154	0.244	0.392	0.15	0.143	0.167	0.18	0.255
yy1	0.154	0.235	0.389	0.169	0.17	0.119	0.132	0.211
spats1	0.154	0.247	0.357	0.195	0.327	0.152	0.166	0.23
rnvu1-1	0.154	0.248	0.455	0.223	0.175	0.148	0.162	0.257
pex26	0.154	0.215	0.42	0.181	0.101	0.192	0.201	0.285
nfs1	0.154	0.216	0.461	0.189	0.115	0.157	0.166	0.267
mrrf	0.155	0.237	0.413	0.187	0.167	0.154	0.166	0.249
ebf3	0.155	0.235	0.228	0.142	0.112	0.159	0.17	0.196
gtsf1l	0.155	0.27	0.397	0.227	0.155	0.168	0.184	0.257
nfil3	0.155	0.215	0.255	0.152	0.168	0.119	0.129	0.169
zc3h8	0.155	0.227	0.255	0.16	0.25	0.133	0.144	0.182
gcc1	0.155	0.196	0.326	0.243	0.156	0.142	0.149	0.212
slc23a2	0.155	0.221	0.366	0.21	0.114	0.138	0.148	0.221
dync1h1	0.154	0.229	0.392	0.206	0.103	0.174	0.185	0.261
trim60	0.154	0.241	0.371	0.195	0.186	0.164	0.176	0.245
ktn1	0.154	0.217	0.305	0.179	0.102	0.145	0.154	0.207
slc7a10	0.154	0.204	0.295	0.198	0.119	0.143	0.151	0.203
rbm12	0.154	0.233	0.368	0.196	0.179	0.166	0.177	0.246
cercam	0.154	0.215	0.293	0.168	0.121	0.15	0.16	0.208
mageb2	0.153	0.243	0.251	0.163	0.124	0.156	0.17	0.201
tspo	0.153	0.225	0.323	0.215	0.114	0.135	0.146	0.205
fam107a	0.153	0.239	0.366	0.174	0.124	0.161	0.174	0.241
thap5	0.154	0.21	0.366	0.151	0.108	0.188	0.196	0.265
cnnm1	0.154	0.22	0.402	0.233	0.142	0.153	0.163	0.245
rpph1	0.154	0.251	0.505	0.241	0.21	0.16	0.173	0.283
polr2m	0.154	0.239	0.459	0.209	0.237	0.155	0.167	0.264
poteb	0.154	0.214	0.323	0.164	0.152	0.163	0.172	0.229
mir342	0.154	0.216	0.222	0.122	0.121	0.13	0.14	0.169
frg1	0.154	0.245	0.39	0.165	0.144	0.198	0.21	0.281
rchts	0.154	0.166	0.162	0.133	0.195	0.198	0.2	0.21
sqstm1	0.154	0.171	0.275	0.165	0.102	0.165	0.168	0.216
zcchc12	0.155	0.157	0.173	0.12	0.122	0.155	0.156	0.175

mir632	0.155	0.214	0.227	0.144	0.114	0.16	0.169	0.197
anp32a	0.155	0.252	0.416	0.212	0.149	0.163	0.176	0.258
erf	0.155	0.223	0.349	0.212	0.135	0.124	0.135	0.203
akap10	0.156	0.208	0.323	0.237	0.152	0.154	0.163	0.221
zadh2	0.155	0.222	0.358	0.172	0.167	0.156	0.166	0.234
igf2bp1	0.155	0.224	0.37	0.178	0.123	0.157	0.167	0.239
tas2r4	0.155	0.182	0.2	0.168	0.182	0.163	0.167	0.191
nme3	0.155	0.232	0.364	0.201	0.147	0.167	0.178	0.245
rock1p1	0.156	0.232	0.305	0.171	0.15	0.165	0.176	0.225
mir26a1	0.156	0.227	0.309	0.133	0.157	0.129	0.141	0.195
foxred1	0.156	0.232	0.334	0.185	0.142	0.156	0.167	0.226
meis1	0.156	0.208	0.228	0.16	0.103	0.177	0.185	0.212
mak	0.156	0.238	0.308	0.219	0.162	0.102	0.115	0.17
cog7	0.156	0.238	0.406	0.243	0.111	0.169	0.181	0.26
steap1	0.156	0.25	0.272	0.157	0.265	0.117	0.132	0.173
hook1	0.156	0.24	0.386	0.209	0.118	0.169	0.182	0.254
copa	0.156	0.227	0.411	0.276	0.102	0.179	0.189	0.271
styx	0.155	0.227	0.308	0.258	0.158	0.158	0.169	0.22
crebzf	0.155	0.188	0.325	0.122	0.242	0.168	0.173	0.235
ccdc142	0.155	0.204	0.319	0.143	0.176	0.139	0.147	0.207
ervfc1-1	0.155	0.253	0.413	0.185	0.141	0.15	0.164	0.245
ervpablb-1	0.155	0.253	0.413	0.185	0.141	0.15	0.164	0.245
ttc31	0.155	0.204	0.319	0.143	0.176	0.139	0.147	0.207
stau1	0.155	0.209	0.426	0.203	0.11	0.155	0.164	0.254
igsf1	0.155	0.198	0.2	0.193	0.112	0.143	0.15	0.173
znf426	0.155	0.226	0.355	0.161	0.153	0.14	0.151	0.219
ervk-21	0.155	0.253	0.413	0.185	0.141	0.15	0.164	0.245
spz1	0.155	0.23	0.329	0.237	0.126	0.146	0.157	0.216
pygl	0.155	0.214	0.342	0.223	0.177	0.174	0.182	0.245
tusc3	0.155	0.223	0.327	0.143	0.227	0.189	0.198	0.253
gabpa	0.155	0.234	0.385	0.18	0.204	0.147	0.159	0.234
krt37	0.155	0.214	0.287	0.15	0.167	0.167	0.176	0.222
scnm1	0.155	0.189	0.219	0.107	0.1	0.172	0.177	0.205
tlx3	0.155	0.217	0.21	0.109	0.103	0.169	0.178	0.2
c8orf59	0.155	0.224	0.287	0.189	0.131	0.155	0.165	0.211
defb106a	0.153	0.223	0.328	0.129	0.121	0.19	0.2	0.254
mterfd2	0.153	0.23	0.254	0.139	0.229	0.17	0.181	0.214
dynlt1	0.152	0.221	0.357	0.221	0.109	0.169	0.179	0.245
slc30a7	0.152	0.214	0.337	0.199	0.12	0.153	0.163	0.225
cpd	0.152	0.222	0.293	0.258	0.132	0.165	0.175	0.221
naca2	0.152	0.238	0.488	0.206	0.118	0.149	0.162	0.269

bex1	0.152	0.244	0.349	0.206	0.15	0.179	0.191	0.251
arcn1	0.152	0.229	0.398	0.239	0.113	0.171	0.182	0.26
znf181	0.152	0.239	0.411	0.246	0.184	0.158	0.17	0.252
cpne1	0.152	0.228	0.371	0.201	0.126	0.16	0.171	0.241
uggt2	0.152	0.221	0.398	0.209	0.195	0.164	0.174	0.254
lin28a	0.152	0.269	0.338	0.15	0.108	0.147	0.164	0.22
copz2	0.152	0.236	0.46	0.243	0.116	0.172	0.184	0.28
prdm12	0.152	0.227	0.314	0.206	0.11	0.154	0.165	0.219
mia3	0.152	0.208	0.308	0.179	0.108	0.139	0.148	0.203
katnb1	0.152	0.248	0.471	0.215	0.122	0.166	0.179	0.278
ypel5	0.152	0.242	0.371	0.212	0.151	0.162	0.175	0.244
ppp1r42	0.152	0.231	0.42	0.274	0.17	0.15	0.162	0.248
mbnl2	0.152	0.223	0.308	0.177	0.108	0.161	0.172	0.224
pcyt2	0.152	0.225	0.426	0.192	0.136	0.154	0.165	0.254
magoh	0.152	0.218	0.362	0.211	0.127	0.171	0.181	0.249
rabep2	0.152	0.205	0.295	0.244	0.111	0.153	0.161	0.212
krt17	0.152	0.231	0.258	0.137	0.127	0.153	0.165	0.201
trmt112	0.151	0.232	0.327	0.165	0.158	0.168	0.18	0.235
rab11fip3	0.151	0.198	0.321	0.209	0.123	0.18	0.187	0.244
loh1cr1	0.151	0.22	0.299	0.126	0.122	0.178	0.188	0.236
parpbp	0.151	0.205	0.306	0.145	0.106	0.175	0.183	0.235
tbc1d14	0.152	0.213	0.337	0.203	0.138	0.175	0.184	0.245
cog1	0.152	0.231	0.413	0.228	0.114	0.16	0.172	0.255
acrbp	0.152	0.229	0.302	0.203	0.109	0.12	0.132	0.185
akap3	0.152	0.196	0.257	0.237	0.106	0.137	0.144	0.186
pak6	0.152	0.186	0.244	0.139	0.207	0.147	0.153	0.191
arsd	0.152	0.198	0.25	0.158	0.135	0.166	0.173	0.209
golga3	0.152	0.236	0.437	0.256	0.107	0.164	0.177	0.266
liph	0.152	0.203	0.233	0.165	0.101	0.184	0.191	0.22
atp6v1c2	0.152	0.234	0.379	0.201	0.122	0.162	0.173	0.246
prdm11	0.152	0.223	0.285	0.164	0.104	0.133	0.144	0.191
hvbs8	0.153	0.257	0.383	0.223	0.175	0.168	0.182	0.252
rps27p3	0.153	0.233	0.323	0.146	0.184	0.166	0.177	0.232
ifitm8p	0.153	0.233	0.323	0.146	0.184	0.166	0.177	0.232
higd1ap1	0.153	0.233	0.323	0.146	0.184	0.166	0.177	0.232
znf726	0.153	0.233	0.323	0.146	0.184	0.166	0.177	0.232
znf723	0.153	0.233	0.323	0.146	0.184	0.166	0.177	0.232
ppfia2	0.153	0.2	0.233	0.165	0.168	0.148	0.156	0.188
dennd5a	0.153	0.228	0.459	0.256	0.171	0.177	0.188	0.284
higd1c	0.153	0.233	0.323	0.146	0.184	0.166	0.177	0.232
ralgps1	0.153	0.183	0.253	0.268	0.133	0.204	0.208	0.244

trbv6-1	0.153	0.293	0.341	0.212	0.125	0.129	0.149	0.205
flnc	0.153	0.21	0.323	0.213	0.103	0.165	0.174	0.232
acbd3	0.153	0.205	0.324	0.221	0.121	0.136	0.145	0.206
fignl1	0.153	0.243	0.272	0.154	0.168	0.137	0.15	0.19
gtpbp2	0.153	0.249	0.411	0.242	0.12	0.167	0.181	0.26
tmem59l	0.153	0.222	0.382	0.243	0.144	0.16	0.171	0.246
fstl4	0.153	0.2	0.241	0.144	0.103	0.177	0.184	0.216
znf280c	0.153	0.223	0.431	0.202	0.145	0.161	0.171	0.261
klf7	0.153	0.182	0.186	0.11	0.103	0.152	0.157	0.177
ct64	0.153	0.197	0.242	0.101	0.106	0.134	0.141	0.178
ct62	0.153	0.197	0.242	0.101	0.106	0.134	0.141	0.178
ct45a6	0.153	0.197	0.242	0.101	0.106	0.134	0.141	0.178
ct45a3	0.153	0.197	0.242	0.101	0.106	0.134	0.141	0.178
ct60	0.153	0.197	0.242	0.101	0.106	0.134	0.141	0.178
pcgf2	0.153	0.244	0.359	0.165	0.134	0.153	0.166	0.232
hoxa11-as	0.153	0.21	0.258	0.152	0.165	0.174	0.183	0.219
ct45b1p	0.153	0.197	0.242	0.101	0.106	0.134	0.141	0.178
ct45a4	0.153	0.197	0.242	0.101	0.106	0.134	0.141	0.178
dusp12	0.153	0.204	0.298	0.206	0.143	0.142	0.15	0.203
cog5	0.153	0.224	0.37	0.22	0.134	0.162	0.173	0.244
katna1	0.153	0.25	0.468	0.214	0.127	0.167	0.18	0.278
defa1a3	0.153	0.188	0.195	0.181	0.125	0.166	0.172	0.193
ct45a5	0.153	0.197	0.242	0.101	0.106	0.134	0.141	0.178
myo1b	0.153	0.215	0.343	0.231	0.106	0.143	0.153	0.218
c1ql4	0.156	0.232	0.308	0.185	0.152	0.154	0.166	0.217
pelp1	0.156	0.19	0.234	0.157	0.527	0.126	0.132	0.169
eny2	0.159	0.188	0.243	0.135	0.115	0.165	0.17	0.206
capn9	0.159	0.233	0.35	0.229	0.106	0.187	0.197	0.258
tmem259	0.159	0.23	0.413	0.212	0.149	0.158	0.168	0.252
zscan2	0.159	0.217	0.307	0.142	0.204	0.16	0.169	0.221
hlf	0.159	0.233	0.322	0.226	0.141	0.135	0.146	0.204
wars	0.159	0.255	0.472	0.244	0.142	0.162	0.176	0.274
mtfr1	0.159	0.227	0.346	0.272	0.105	0.134	0.145	0.21
tgfa	0.159	0.213	0.206	0.161	0.125	0.169	0.177	0.197
mir499a	0.159	0.222	0.259	0.156	0.123	0.136	0.146	0.185
hotair	0.159	0.276	0.417	0.153	0.156	0.167	0.184	0.262
fbxo32	0.16	0.2	0.299	0.147	0.139	0.158	0.165	0.217
sec23a	0.16	0.227	0.367	0.203	0.111	0.164	0.174	0.243
gle1	0.159	0.223	0.367	0.184	0.11	0.166	0.175	0.245
bcl7c	0.159	0.268	0.404	0.219	0.172	0.148	0.164	0.241
arf4	0.159	0.225	0.398	0.264	0.109	0.183	0.193	0.27

atf2	0.159	0.189	0.285	0.245	0.104	0.141	0.146	0.197
rnase9	0.159	0.234	0.331	0.198	0.243	0.14	0.152	0.211
mycn	0.159	0.239	0.319	0.112	0.135	0.161	0.173	0.226
aifm2	0.158	0.259	0.499	0.198	0.12	0.174	0.189	0.294
ikbkap	0.158	0.202	0.251	0.191	0.128	0.173	0.18	0.215
gpr52	0.159	0.233	0.368	0.22	0.144	0.175	0.186	0.254
mif4gd	0.158	0.249	0.481	0.26	0.37	0.167	0.18	0.281
rsf1	0.158	0.279	0.44	0.163	0.163	0.142	0.159	0.246
ndn	0.158	0.24	0.339	0.168	0.12	0.177	0.188	0.246
nlrp5	0.158	0.259	0.302	0.148	0.115	0.157	0.172	0.217
gpr53p	0.159	0.233	0.368	0.22	0.144	0.175	0.186	0.254
snd1	0.159	0.189	0.22	0.193	0.142	0.152	0.158	0.187
tbc1d17	0.159	0.21	0.234	0.225	0.102	0.181	0.189	0.217
frg2c	0.159	0.24	0.369	0.182	0.227	0.168	0.18	0.248
loc285299	0.159	0.24	0.369	0.182	0.227	0.168	0.18	0.248
cabp7	0.159	0.209	0.349	0.199	0.107	0.158	0.166	0.232
slc1a5	0.159	0.228	0.403	0.202	0.113	0.151	0.161	0.243
chic1	0.159	0.232	0.358	0.226	0.219	0.168	0.179	0.244
pomzp3	0.159	0.232	0.386	0.194	0.157	0.175	0.185	0.259
caskin2	0.16	0.232	0.397	0.221	0.139	0.174	0.185	0.262
nme2	0.16	0.243	0.427	0.234	0.155	0.169	0.181	0.266
plin1	0.161	0.199	0.288	0.194	0.186	0.154	0.161	0.21
dgat1	0.161	0.2	0.292	0.188	0.141	0.158	0.165	0.215
trim59	0.161	0.206	0.248	0.121	0.382	0.14	0.148	0.185
tsc22d2	0.161	0.197	0.211	0.182	0.102	0.185	0.191	0.213
dio3	0.161	0.208	0.252	0.175	0.167	0.165	0.173	0.208
cltcl1	0.161	0.213	0.315	0.205	0.121	0.209	0.216	0.266
dhrs9	0.161	0.211	0.267	0.15	0.15	0.19	0.198	0.235
mcc	0.161	0.212	0.234	0.129	0.196	0.219	0.226	0.25
etv3	0.161	0.201	0.243	0.208	0.113	0.12	0.127	0.166
loc145989	0.161	0.277	0.343	0.212	0.136	0.14	0.157	0.215
smu1	0.161	0.217	0.308	0.206	0.133	0.184	0.192	0.242
nbpf23	0.161	0.205	0.259	0.143	0.131	0.161	0.168	0.206
krt71	0.161	0.229	0.291	0.154	0.113	0.19	0.2	0.243
snora69	0.161	0.243	0.383	0.211	0.139	0.161	0.173	0.245
nucks1	0.161	0.254	0.422	0.209	0.158	0.162	0.175	0.258
tbc1d5	0.161	0.201	0.36	0.214	0.109	0.18	0.186	0.256
mars	0.161	0.217	0.384	0.233	0.165	0.157	0.166	0.242
rab19	0.16	0.25	0.387	0.272	0.283	0.177	0.19	0.261
atad3a	0.16	0.266	0.48	0.173	0.101	0.169	0.184	0.283
clec18b	0.16	0.221	0.276	0.155	0.107	0.175	0.184	0.225

clec18c	0.16	0.221	0.276	0.155	0.107	0.175	0.184	0.225
ets2	0.16	0.238	0.315	0.209	0.185	0.12	0.132	0.188
mid1ip1	0.16	0.21	0.323	0.176	0.19	0.162	0.17	0.228
hdac11	0.16	0.212	0.272	0.179	0.102	0.173	0.181	0.221
peg10	0.16	0.243	0.34	0.168	0.194	0.17	0.182	0.24
cog4	0.16	0.236	0.403	0.24	0.12	0.166	0.177	0.256
mir222	0.16	0.275	0.309	0.164	0.12	0.14	0.157	0.204
eif6	0.16	0.24	0.394	0.177	0.157	0.161	0.173	0.249
edc3	0.16	0.229	0.405	0.227	0.139	0.149	0.16	0.242
fcho1	0.16	0.214	0.284	0.237	0.112	0.205	0.212	0.254
kctd8	0.16	0.207	0.246	0.145	0.134	0.173	0.18	0.213
pdcd2l	0.16	0.223	0.294	0.172	0.166	0.155	0.165	0.212
gsta4	0.16	0.21	0.353	0.18	0.18	0.162	0.17	0.237
hars2	0.158	0.219	0.384	0.2	0.127	0.156	0.165	0.242
gpr83	0.158	0.226	0.27	0.205	0.139	0.152	0.163	0.203
siah2	0.157	0.2	0.37	0.154	0.303	0.191	0.198	0.269
ceacam7	0.157	0.236	0.24	0.143	0.105	0.175	0.186	0.214
tktl1	0.157	0.258	0.371	0.146	0.105	0.156	0.17	0.238
ppp4r1	0.157	0.221	0.354	0.209	0.121	0.173	0.182	0.247
tmem216	0.157	0.192	0.229	0.135	0.186	0.201	0.207	0.234
cnnm3	0.157	0.227	0.363	0.233	0.144	0.15	0.161	0.23
srpx	0.157	0.21	0.295	0.147	0.142	0.172	0.18	0.228
pafah1b1	0.157	0.222	0.302	0.173	0.106	0.136	0.146	0.199
ndufaf6	0.157	0.245	0.395	0.198	0.15	0.151	0.164	0.241
or2ag1	0.157	0.183	0.252	0.188	0.1	0.146	0.151	0.192
emc3	0.157	0.185	0.233	0.199	0.189	0.155	0.16	0.194
aim1	0.157	0.24	0.328	0.179	0.111	0.17	0.182	0.236
znf345	0.157	0.203	0.337	0.195	0.362	0.162	0.169	0.232
tapt1	0.157	0.207	0.247	0.167	0.117	0.201	0.208	0.239
mir10a	0.157	0.214	0.242	0.14	0.126	0.149	0.158	0.192
insl3	0.157	0.205	0.169	0.171	0.223	0.141	0.148	0.161
srrm4	0.157	0.215	0.267	0.129	0.187	0.146	0.155	0.197
ubap2l	0.156	0.19	0.226	0.146	0.272	0.169	0.174	0.204
or1d5	0.156	0.24	0.355	0.252	0.156	0.155	0.167	0.232
ell3	0.156	0.221	0.278	0.176	0.124	0.156	0.166	0.209
rltpr	0.156	0.235	0.376	0.222	0.13	0.166	0.177	0.248
cyp20a1	0.156	0.2	0.269	0.121	0.109	0.149	0.156	0.2
arih2	0.156	0.193	0.3	0.12	0.151	0.16	0.166	0.219
hydin	0.156	0.235	0.325	0.162	0.125	0.17	0.181	0.236
pttg1ip	0.156	0.231	0.329	0.162	0.134	0.149	0.16	0.218
micd	0.156	0.259	0.399	0.234	0.138	0.157	0.172	0.248

micc	0.156	0.259	0.399	0.234	0.138	0.157	0.172	0.248
ewsr1	0.156	0.25	0.309	0.179	0.116	0.153	0.167	0.216
ddx24	0.156	0.238	0.505	0.221	0.205	0.162	0.174	0.285
sgip1	0.156	0.188	0.302	0.219	0.206	0.172	0.177	0.23
dnase2b	0.156	0.227	0.343	0.177	0.105	0.139	0.15	0.214
sh3pxd2a	0.156	0.184	0.225	0.221	0.122	0.161	0.165	0.196
rnf38	0.156	0.231	0.331	0.178	0.147	0.171	0.182	0.238
slc25a23	0.156	0.211	0.399	0.227	0.124	0.15	0.158	0.241
ptma	0.157	0.206	0.333	0.239	0.173	0.142	0.15	0.214
ppp5c	0.157	0.185	0.261	0.212	0.267	0.146	0.151	0.195
mir100	0.158	0.247	0.289	0.139	0.104	0.149	0.162	0.205
mapk15	0.158	0.204	0.296	0.229	0.197	0.138	0.146	0.198
ropn1b	0.158	0.201	0.336	0.298	0.1	0.145	0.152	0.217
bin1	0.158	0.231	0.368	0.235	0.104	0.161	0.172	0.241
mir1825	0.158	0.217	0.289	0.18	0.141	0.179	0.188	0.233
mir320b2	0.158	0.217	0.289	0.18	0.141	0.179	0.188	0.233
mir320c2	0.158	0.217	0.289	0.18	0.141	0.179	0.188	0.233
loc146253	0.158	0.228	0.308	0.189	0.185	0.168	0.178	0.229
coa3	0.158	0.236	0.42	0.205	0.177	0.157	0.168	0.254
fam162b	0.158	0.224	0.358	0.181	0.147	0.158	0.167	0.235
slc6a6	0.158	0.221	0.334	0.209	0.139	0.152	0.161	0.222
larp4	0.158	0.218	0.374	0.186	0.111	0.155	0.164	0.237
ccdc65	0.158	0.212	0.306	0.191	0.132	0.16	0.168	0.221
kiaa1551	0.158	0.208	0.198	0.123	0.148	0.207	0.214	0.229
nr4a2	0.158	0.204	0.223	0.145	0.212	0.171	0.178	0.205
dleu2	0.158	0.252	0.368	0.198	0.124	0.157	0.171	0.238
mir320d1	0.158	0.217	0.289	0.18	0.141	0.179	0.188	0.233
hmgb1	0.158	0.217	0.289	0.18	0.141	0.179	0.188	0.233
pnpla2	0.157	0.188	0.298	0.165	0.173	0.151	0.156	0.21
spam1	0.157	0.259	0.406	0.226	0.145	0.152	0.167	0.245
ruvbl1	0.157	0.225	0.406	0.134	0.13	0.172	0.182	0.263
gtf2i	0.157	0.224	0.293	0.225	0.164	0.14	0.151	0.199
rbm45	0.157	0.236	0.342	0.194	0.143	0.143	0.155	0.217
hdac6	0.157	0.225	0.398	0.175	0.142	0.15	0.16	0.241
mcoln2	0.157	0.207	0.286	0.226	0.136	0.151	0.159	0.207
ncaph2	0.157	0.241	0.313	0.246	0.166	0.149	0.162	0.214
tmem254-as1	0.157	0.268	0.297	0.194	0.158	0.16	0.176	0.218
rfpl4a	0.158	0.241	0.371	0.178	0.122	0.158	0.171	0.24
ubiad1	0.158	0.195	0.252	0.138	0.119	0.139	0.145	0.186
mrpl12	0.158	0.215	0.354	0.208	0.156	0.176	0.185	0.25
abce1	0.158	0.233	0.436	0.206	0.104	0.137	0.149	0.242

gspt1	0.157	0.225	0.418	0.177	0.161	0.171	0.181	0.266
urahp	0.158	0.21	0.245	0.122	0.131	0.187	0.195	0.226
mir133a1	0.151	0.244	0.342	0.161	0.12	0.15	0.164	0.224
epb41l4b	0.151	0.214	0.237	0.155	0.185	0.127	0.137	0.171
dux4	0.145	0.231	0.383	0.164	0.196	0.172	0.184	0.257
atf1	0.145	0.173	0.231	0.197	0.126	0.135	0.14	0.177
fras1	0.145	0.214	0.252	0.153	0.103	0.174	0.184	0.218
helz	0.144	0.211	0.34	0.164	0.137	0.144	0.154	0.219
lpin2	0.144	0.196	0.304	0.14	0.157	0.138	0.146	0.202
cox15	0.144	0.249	0.386	0.203	0.137	0.159	0.174	0.246
stat5b	0.144	0.193	0.239	0.126	0.125	0.118	0.126	0.164
rnf2	0.145	0.235	0.4	0.153	0.166	0.162	0.175	0.254
lrrc8e	0.145	0.183	0.244	0.172	0.116	0.112	0.119	0.161
cpne5	0.145	0.2	0.173	0.116	0.123	0.131	0.139	0.155
arhgap25	0.145	0.186	0.245	0.164	0.102	0.136	0.143	0.182
wdr77	0.145	0.228	0.401	0.215	0.256	0.156	0.167	0.248
capn11	0.145	0.231	0.36	0.197	0.125	0.143	0.155	0.224
tacc2	0.145	0.213	0.265	0.156	0.187	0.133	0.143	0.186
lrrc8d	0.145	0.183	0.244	0.172	0.116	0.112	0.119	0.161
lrrc8b	0.145	0.183	0.244	0.172	0.116	0.112	0.119	0.161
grhl1	0.144	0.214	0.283	0.159	0.139	0.12	0.131	0.18
pttg1	0.144	0.27	0.395	0.187	0.125	0.149	0.166	0.24
znf236	0.144	0.213	0.298	0.213	0.116	0.172	0.182	0.23
sox21	0.144	0.189	0.167	0.102	0.144	0.119	0.127	0.143
bhlha15	0.144	0.222	0.253	0.166	0.108	0.132	0.144	0.181
ppp2r2c	0.144	0.199	0.312	0.19	0.14	0.145	0.153	0.211
atp5j	0.143	0.186	0.258	0.161	0.107	0.14	0.146	0.189
tsg101	0.143	0.21	0.422	0.216	0.116	0.172	0.181	0.269
mina	0.143	0.26	0.43	0.188	0.116	0.145	0.161	0.247
larp4b	0.144	0.215	0.419	0.218	0.164	0.147	0.158	0.246
myrf	0.144	0.22	0.324	0.207	0.107	0.174	0.184	0.24
chiap2	0.144	0.227	0.362	0.188	0.129	0.152	0.164	0.232
nek8	0.144	0.216	0.282	0.137	0.118	0.147	0.158	0.204
tuba1b	0.144	0.23	0.423	0.243	0.105	0.151	0.163	0.25
loc440683	0.144	0.228	0.372	0.254	0.136	0.142	0.155	0.227
papd4	0.144	0.232	0.441	0.201	0.101	0.136	0.149	0.244
psca	0.144	0.249	0.294	0.138	0.114	0.142	0.157	0.203
smtnl1	0.144	0.169	0.227	0.174	0.197	0.158	0.163	0.196
ifnnp1	0.145	0.177	0.183	0.12	0.105	0.168	0.173	0.191
zc3h4	0.145	0.208	0.388	0.182	0.154	0.177	0.186	0.263
c2cd2l	0.146	0.221	0.406	0.188	0.136	0.157	0.168	0.25

tuba4b	0.146	0.199	0.27	0.174	0.131	0.13	0.138	0.184
arf3	0.146	0.223	0.43	0.282	0.126	0.171	0.182	0.27
nav3	0.145	0.246	0.287	0.148	0.119	0.14	0.155	0.199
rpp21	0.145	0.181	0.282	0.14	0.125	0.145	0.151	0.202
rlim	0.145	0.214	0.291	0.168	0.174	0.14	0.15	0.2
cox7c	0.145	0.207	0.298	0.194	0.135	0.134	0.144	0.197
b3gntl1	0.146	0.237	0.38	0.213	0.16	0.164	0.177	0.249
bzrap1	0.146	0.177	0.243	0.194	0.131	0.156	0.161	0.199
dbip1	0.146	0.221	0.386	0.218	0.13	0.17	0.181	0.256
trim63	0.146	0.177	0.285	0.137	0.191	0.138	0.144	0.196
pms2p8	0.146	0.191	0.271	0.187	0.103	0.148	0.155	0.201
btn1a1	0.146	0.22	0.333	0.221	0.115	0.138	0.149	0.211
obp2b	0.146	0.209	0.291	0.167	0.1	0.169	0.178	0.226
cldn20	0.146	0.222	0.267	0.152	0.215	0.155	0.166	0.206
znf354a	0.146	0.242	0.416	0.209	0.263	0.156	0.17	0.253
spata24	0.145	0.226	0.423	0.173	0.237	0.169	0.181	0.267
csde1	0.145	0.241	0.462	0.213	0.139	0.134	0.148	0.248
entpd3	0.145	0.21	0.341	0.199	0.125	0.175	0.184	0.246
rest	0.145	0.224	0.321	0.148	0.167	0.135	0.147	0.205
sh2d3a	0.145	0.2	0.28	0.21	0.104	0.115	0.123	0.174
gpam	0.145	0.185	0.323	0.197	0.157	0.151	0.157	0.219
hmgn3	0.145	0.233	0.408	0.196	0.207	0.142	0.154	0.238
ceacam8	0.145	0.223	0.273	0.248	0.101	0.136	0.148	0.191
prkar2a	0.145	0.177	0.235	0.229	0.1	0.131	0.136	0.174
atf4	0.145	0.188	0.317	0.137	0.101	0.123	0.13	0.193
psg11	0.145	0.209	0.264	0.182	0.149	0.136	0.145	0.188
pnn	0.145	0.238	0.364	0.168	0.257	0.134	0.148	0.217
ankrd35	0.145	0.197	0.327	0.182	0.132	0.129	0.137	0.201
cpsf1	0.145	0.227	0.447	0.23	0.195	0.149	0.161	0.256
pwp1	0.145	0.212	0.274	0.169	0.112	0.197	0.206	0.245
neat1	0.145	0.254	0.466	0.242	0.2	0.137	0.152	0.251
tekt4	0.145	0.222	0.346	0.208	0.17	0.16	0.171	0.234
or1d2	0.145	0.2	0.356	0.235	0.111	0.158	0.166	0.236
dynlt3	0.143	0.224	0.406	0.229	0.129	0.152	0.164	0.247
elp5	0.143	0.221	0.326	0.185	0.173	0.145	0.156	0.215
igkj4	0.141	0.231	0.334	0.198	0.138	0.133	0.147	0.208
igkj1	0.141	0.231	0.334	0.198	0.138	0.133	0.147	0.208
igkj2	0.141	0.231	0.334	0.198	0.138	0.133	0.147	0.208
igkj3	0.141	0.231	0.334	0.198	0.138	0.133	0.147	0.208
etf1p2	0.141	0.219	0.382	0.201	0.122	0.185	0.196	0.268
cstf3	0.141	0.223	0.452	0.224	0.185	0.159	0.171	0.267

etf1p3	0.141	0.219	0.382	0.201	0.122	0.185	0.196	0.268
igkj5	0.141	0.231	0.334	0.198	0.138	0.133	0.147	0.208
slc9c1	0.141	0.182	0.242	0.165	0.101	0.162	0.168	0.204
piwil1	0.142	0.257	0.422	0.16	0.128	0.144	0.161	0.245
spic	0.142	0.22	0.229	0.193	0.11	0.105	0.117	0.15
nbea	0.142	0.206	0.282	0.175	0.122	0.141	0.15	0.198
hsd11b1	0.142	0.184	0.267	0.159	0.246	0.14	0.147	0.193
plekha6	0.142	0.202	0.274	0.138	0.149	0.149	0.158	0.203
nampt	0.141	0.182	0.271	0.123	0.105	0.136	0.142	0.19
tmem161b-as1	0.141	0.191	0.307	0.115	0.127	0.144	0.152	0.209
znf143	0.141	0.201	0.318	0.171	0.117	0.113	0.123	0.185
csn2	0.141	0.207	0.291	0.248	0.13	0.128	0.138	0.19
nlrp9	0.141	0.227	0.301	0.176	0.123	0.168	0.18	0.228
trappc1	0.141	0.228	0.427	0.216	0.166	0.134	0.147	0.237
kdm3a	0.141	0.225	0.351	0.184	0.237	0.124	0.136	0.205
dlat	0.141	0.226	0.384	0.237	0.133	0.132	0.144	0.222
rfx6	0.141	0.189	0.211	0.135	0.178	0.14	0.147	0.175
eif4a3	0.14	0.211	0.433	0.204	0.116	0.14	0.15	0.244
krt2	0.14	0.209	0.269	0.137	0.127	0.161	0.171	0.212
tsnax	0.141	0.221	0.372	0.198	0.187	0.126	0.138	0.213
nr0b2	0.141	0.172	0.234	0.181	0.354	0.129	0.134	0.173
fam114a1	0.141	0.169	0.154	0.183	0.11	0.152	0.156	0.168
ergic1	0.141	0.204	0.254	0.127	0.114	0.137	0.146	0.186
spata21	0.141	0.253	0.4	0.258	0.183	0.15	0.166	0.243
narr	0.141	0.202	0.37	0.166	0.145	0.167	0.176	0.249
aftph	0.141	0.192	0.343	0.213	0.131	0.154	0.161	0.229
dmrtb1	0.141	0.205	0.263	0.156	0.158	0.171	0.18	0.219
yy2	0.141	0.22	0.361	0.168	0.178	0.136	0.148	0.219
dpysl3	0.142	0.217	0.317	0.195	0.116	0.148	0.159	0.216
kdelr1	0.142	0.207	0.323	0.204	0.103	0.136	0.146	0.207
fyco1	0.143	0.207	0.325	0.182	0.129	0.165	0.174	0.233
zc4h2	0.143	0.182	0.278	0.139	0.129	0.136	0.143	0.193
mir210	0.143	0.221	0.332	0.135	0.101	0.129	0.141	0.203
sema4f	0.143	0.208	0.247	0.147	0.105	0.191	0.199	0.231
pcat6	0.143	0.209	0.29	0.117	0.173	0.15	0.159	0.208
adam22	0.143	0.199	0.266	0.196	0.238	0.129	0.138	0.182
or3b1p	0.143	0.16	0.18	0.16	0.103	0.17	0.173	0.192
rnase10	0.143	0.204	0.245	0.178	0.278	0.131	0.14	0.178
mcat	0.143	0.187	0.322	0.158	0.159	0.168	0.174	0.235
mycbpap	0.143	0.213	0.354	0.198	0.186	0.132	0.142	0.212
rnu5a-1	0.143	0.203	0.388	0.214	0.116	0.138	0.147	0.228

sra1	0.143	0.176	0.236	0.138	0.462	0.128	0.134	0.173
mitd1	0.143	0.201	0.264	0.152	0.135	0.17	0.179	0.218
asah1	0.143	0.233	0.386	0.211	0.1	0.127	0.14	0.218
lpar6	0.143	0.211	0.301	0.163	0.104	0.162	0.172	0.223
pcat1	0.143	0.22	0.305	0.116	0.125	0.18	0.191	0.24
trbv11-1	0.143	0.243	0.33	0.231	0.142	0.161	0.175	0.231
fam21a	0.143	0.203	0.408	0.215	0.108	0.139	0.149	0.236
smcr7l	0.142	0.203	0.361	0.137	0.115	0.169	0.177	0.248
sp110	0.142	0.222	0.324	0.173	0.149	0.136	0.147	0.207
brix1	0.142	0.19	0.388	0.158	0.149	0.211	0.217	0.293
dpy19l2p2	0.142	0.19	0.256	0.112	0.111	0.167	0.174	0.213
dpy19l2p4	0.142	0.19	0.256	0.112	0.111	0.167	0.174	0.213
fchsd2	0.142	0.192	0.272	0.151	0.125	0.161	0.168	0.213
prss21	0.142	0.247	0.388	0.217	0.18	0.155	0.17	0.243
sms	0.142	0.224	0.387	0.179	0.141	0.151	0.163	0.24
kcnrg	0.142	0.241	0.387	0.206	0.129	0.136	0.151	0.227
cited4	0.143	0.169	0.261	0.132	0.244	0.125	0.13	0.177
fam21b	0.143	0.203	0.408	0.215	0.108	0.139	0.149	0.236
vcx3a	0.142	0.19	0.275	0.179	0.143	0.198	0.204	0.246
gnmt	0.142	0.198	0.323	0.216	0.156	0.134	0.143	0.205
znf85	0.142	0.244	0.398	0.19	0.278	0.156	0.17	0.248
zpld1	0.142	0.202	0.256	0.14	0.155	0.15	0.159	0.198
pms2p6	0.146	0.191	0.271	0.187	0.103	0.148	0.155	0.201
cpne6	0.146	0.205	0.378	0.302	0.128	0.136	0.145	0.223
mgat3	0.15	0.23	0.3	0.196	0.113	0.141	0.153	0.203
igflr1	0.15	0.223	0.308	0.178	0.157	0.148	0.159	0.211
ube3c	0.15	0.195	0.346	0.158	0.108	0.157	0.164	0.232
epg5	0.149	0.207	0.343	0.127	0.108	0.169	0.177	0.241
gjb5	0.149	0.211	0.242	0.134	0.111	0.182	0.191	0.221
eef1a2	0.149	0.26	0.386	0.24	0.118	0.171	0.186	0.256
ighmbp2	0.149	0.234	0.335	0.192	0.13	0.143	0.156	0.216
capn8	0.15	0.221	0.271	0.172	0.12	0.148	0.158	0.2
cpsf4	0.15	0.223	0.428	0.224	0.143	0.132	0.143	0.235
linc00272	0.15	0.211	0.278	0.192	0.147	0.14	0.15	0.196
nlrp14	0.15	0.214	0.243	0.16	0.204	0.169	0.179	0.21
il1rapl1	0.15	0.185	0.226	0.174	0.118	0.176	0.181	0.211
loc100288079	0.15	0.211	0.278	0.192	0.147	0.14	0.15	0.196
immt	0.15	0.22	0.399	0.191	0.158	0.154	0.164	0.245
slbp	0.15	0.24	0.419	0.23	0.137	0.162	0.175	0.259
brd4	0.15	0.26	0.527	0.205	0.247	0.153	0.169	0.285
gcn1l1	0.149	0.213	0.415	0.171	0.113	0.138	0.147	0.236

pex7	0.149	0.198	0.35	0.197	0.141	0.168	0.175	0.243
mrps15	0.149	0.201	0.387	0.181	0.116	0.162	0.17	0.249
ptcd3	0.149	0.201	0.387	0.181	0.116	0.162	0.17	0.249
meig1	0.149	0.203	0.29	0.144	0.219	0.177	0.185	0.232
rbm5-as1	0.149	0.238	0.354	0.228	0.101	0.163	0.176	0.24
gabarap	0.149	0.188	0.324	0.168	0.128	0.153	0.159	0.221
tfap2b	0.149	0.184	0.235	0.115	0.164	0.154	0.16	0.194
zfyve1	0.149	0.211	0.335	0.184	0.119	0.178	0.186	0.246
kiaa1524	0.149	0.253	0.319	0.136	0.105	0.134	0.149	0.203
pdk4	0.149	0.178	0.272	0.154	0.247	0.154	0.159	0.206
atp5g3	0.149	0.179	0.257	0.189	0.101	0.155	0.16	0.202
bnc2	0.149	0.217	0.279	0.141	0.144	0.166	0.175	0.218
eaf2	0.149	0.216	0.291	0.166	0.175	0.167	0.176	0.223
ints7	0.149	0.191	0.315	0.211	0.214	0.145	0.152	0.211
zbtb18	0.149	0.253	0.329	0.137	0.176	0.143	0.158	0.214
dnali1	0.149	0.22	0.336	0.204	0.133	0.187	0.197	0.255
ppp1r3a	0.149	0.188	0.337	0.196	0.158	0.154	0.16	0.226
sept3	0.15	0.193	0.292	0.177	0.101	0.152	0.159	0.21
umodl1	0.15	0.245	0.316	0.189	0.118	0.174	0.187	0.237
sat1	0.151	0.241	0.412	0.173	0.157	0.174	0.187	0.268
ptbp1	0.151	0.248	0.468	0.216	0.163	0.14	0.155	0.255
hunk	0.151	0.208	0.3	0.142	0.154	0.142	0.151	0.204
mtif3	0.151	0.225	0.386	0.195	0.211	0.163	0.174	0.249
mrpl17	0.151	0.192	0.279	0.22	0.104	0.184	0.19	0.234
ndufs4	0.151	0.211	0.39	0.202	0.176	0.163	0.172	0.25
rpl21	0.151	0.194	0.252	0.132	0.106	0.156	0.163	0.201
znf180	0.151	0.215	0.32	0.239	0.151	0.148	0.158	0.215
shank3	0.151	0.19	0.27	0.174	0.102	0.168	0.174	0.217
ddx51	0.151	0.229	0.456	0.17	0.151	0.153	0.164	0.262
tgif2lx	0.151	0.221	0.294	0.168	0.228	0.185	0.194	0.24
pabpc1	0.151	0.232	0.498	0.221	0.104	0.146	0.158	0.269
dffa	0.151	0.256	0.414	0.217	0.121	0.146	0.161	0.243
tanc1	0.151	0.18	0.211	0.137	0.202	0.155	0.16	0.188
ccnd2	0.151	0.269	0.272	0.155	0.103	0.117	0.135	0.173
fam110b	0.151	0.222	0.278	0.162	0.309	0.127	0.138	0.184
rbm8a	0.151	0.221	0.42	0.257	0.139	0.161	0.171	0.258
immp2l	0.151	0.216	0.281	0.147	0.114	0.161	0.17	0.214
qrfpr	0.15	0.19	0.241	0.173	0.125	0.154	0.16	0.196
znf266	0.15	0.228	0.365	0.218	0.173	0.143	0.155	0.225
camkv	0.15	0.224	0.331	0.184	0.111	0.168	0.178	0.236
txndc2	0.15	0.252	0.441	0.24	0.187	0.175	0.189	0.277

rabggta	0.15	0.228	0.362	0.24	0.116	0.162	0.174	0.241
linc00581	0.15	0.206	0.299	0.152	0.165	0.167	0.175	0.226
anxa6	0.15	0.23	0.386	0.263	0.109	0.149	0.16	0.236
pcbp1	0.15	0.236	0.423	0.249	0.14	0.151	0.164	0.25
spaca7	0.15	0.175	0.247	0.135	0.139	0.153	0.157	0.197
rnf115	0.151	0.181	0.314	0.12	0.117	0.182	0.187	0.244
psmc1	0.151	0.224	0.475	0.229	0.154	0.17	0.181	0.283
gnpda1	0.151	0.212	0.312	0.176	0.133	0.16	0.169	0.224
tmprss2	0.151	0.247	0.308	0.146	0.162	0.145	0.16	0.209
alpk1	0.15	0.199	0.359	0.173	0.11	0.173	0.181	0.25
gabarapl2	0.151	0.199	0.392	0.192	0.125	0.182	0.189	0.268
ldlrad4	0.149	0.225	0.33	0.189	0.172	0.167	0.178	0.235
srpk1	0.149	0.231	0.451	0.242	0.15	0.153	0.165	0.261
tubb7p	0.147	0.201	0.284	0.144	0.115	0.153	0.161	0.209
mir1469	0.147	0.224	0.285	0.166	0.168	0.134	0.146	0.193
mir1471	0.147	0.224	0.285	0.166	0.168	0.134	0.146	0.193
u2af1	0.147	0.228	0.441	0.248	0.177	0.151	0.163	0.256
page5	0.147	0.208	0.273	0.189	0.102	0.139	0.149	0.193
ssx2b	0.147	0.261	0.383	0.224	0.231	0.155	0.171	0.242
rtf1	0.147	0.224	0.389	0.213	0.155	0.144	0.155	0.233
mir1251	0.147	0.224	0.285	0.166	0.168	0.134	0.146	0.193
mir1470	0.147	0.224	0.285	0.166	0.168	0.134	0.146	0.193
trappc5	0.147	0.236	0.429	0.221	0.187	0.159	0.172	0.26
barhl1	0.147	0.191	0.151	0.111	0.123	0.172	0.178	0.184
ppan-p2ry11	0.147	0.212	0.311	0.219	0.102	0.165	0.174	0.228
evi5l	0.147	0.2	0.315	0.177	0.142	0.137	0.145	0.204
jarid2	0.147	0.222	0.254	0.133	0.117	0.128	0.14	0.178
set	0.147	0.237	0.443	0.24	0.2	0.147	0.16	0.253
erv3-1	0.147	0.237	0.385	0.212	0.152	0.135	0.148	0.224
inf2	0.147	0.204	0.33	0.201	0.102	0.172	0.18	0.24
c1orf43	0.147	0.197	0.273	0.196	0.128	0.137	0.145	0.192
trim46	0.146	0.22	0.348	0.203	0.149	0.161	0.172	0.236
taf15	0.146	0.226	0.425	0.208	0.281	0.149	0.161	0.25
trim27	0.146	0.201	0.351	0.162	0.146	0.158	0.167	0.235
anxa11	0.146	0.235	0.438	0.249	0.105	0.176	0.189	0.278
crisp2	0.146	0.233	0.353	0.219	0.14	0.137	0.15	0.217
erap2	0.146	0.225	0.308	0.187	0.145	0.148	0.16	0.212
dach2	0.146	0.194	0.224	0.135	0.155	0.182	0.189	0.216
pcf11	0.146	0.232	0.449	0.192	0.161	0.155	0.168	0.263
tm9sf1	0.146	0.214	0.356	0.189	0.114	0.141	0.151	0.221
lsg1	0.147	0.22	0.32	0.189	0.111	0.152	0.163	0.22

olig3	0.147	0.172	0.13	0.135	0.111	0.184	0.188	0.188
cbx7	0.147	0.255	0.41	0.14	0.147	0.148	0.164	0.244
cnot7	0.147	0.233	0.386	0.196	0.146	0.149	0.162	0.237
ppm1k	0.146	0.178	0.271	0.126	0.168	0.15	0.155	0.202
znf540	0.146	0.186	0.316	0.187	0.174	0.126	0.133	0.195
myb	0.146	0.232	0.311	0.202	0.179	0.135	0.148	0.202
nrip1	0.147	0.173	0.256	0.141	0.652	0.133	0.138	0.183
synrg	0.148	0.201	0.374	0.24	0.131	0.158	0.166	0.241
casp8ap2	0.148	0.189	0.279	0.137	0.234	0.151	0.158	0.206
mau2	0.148	0.218	0.366	0.145	0.166	0.196	0.206	0.273
fli1	0.148	0.218	0.254	0.176	0.11	0.129	0.139	0.178
lpp	0.148	0.213	0.316	0.168	0.16	0.148	0.158	0.215
mir5691	0.148	0.189	0.258	0.155	0.131	0.143	0.15	0.192
mir2110	0.148	0.265	0.353	0.146	0.117	0.138	0.155	0.217
mir1307	0.148	0.265	0.353	0.146	0.117	0.138	0.155	0.217
hist1h3h	0.148	0.239	0.393	0.214	0.165	0.143	0.156	0.234
opcml	0.148	0.235	0.336	0.177	0.102	0.168	0.18	0.238
nelfcd	0.149	0.198	0.389	0.162	0.145	0.176	0.184	0.262
znf235	0.149	0.235	0.389	0.192	0.169	0.166	0.178	0.253
ciz1	0.149	0.256	0.401	0.172	0.205	0.172	0.187	0.262
gpr61	0.148	0.175	0.232	0.189	0.142	0.161	0.165	0.2
btg3	0.148	0.222	0.326	0.166	0.106	0.169	0.179	0.236
znf569	0.148	0.223	0.378	0.225	0.236	0.141	0.152	0.227
tnrc6a	0.148	0.237	0.457	0.191	0.158	0.154	0.166	0.263
mir103a2	0.148	0.265	0.353	0.146	0.117	0.138	0.155	0.217
cln3	0.148	0.227	0.406	0.195	0.135	0.157	0.169	0.251
tssk1b	0.148	0.219	0.336	0.2	0.129	0.162	0.172	0.233
tcf12	0.148	0.22	0.218	0.147	0.112	0.124	0.135	0.163
ptcsc1	0.148	0.205	0.292	0.165	0.143	0.166	0.174	0.223
gpr45	0.148	0.177	0.21	0.159	0.108	0.161	0.166	0.193
aars	0.148	0.208	0.398	0.192	0.163	0.149	0.158	0.241
alms1	0.148	0.208	0.283	0.167	0.124	0.163	0.172	0.217
adam12-ot1	0.148	0.198	0.277	0.148	0.202	0.135	0.143	0.191
tnfaip2	0.148	0.231	0.345	0.196	0.193	0.141	0.153	0.217
ros1	0.148	0.197	0.251	0.218	0.114	0.149	0.157	0.195
tmem26	0.148	0.185	0.162	0.104	0.178	0.186	0.192	0.2
rpl3l	0.148	0.224	0.369	0.189	0.117	0.153	0.164	0.235
tssk2	0.148	0.223	0.319	0.207	0.145	0.137	0.149	0.206
tbc1d25	0.148	0.223	0.408	0.229	0.146	0.186	0.197	0.277
aqpep	0.148	0.227	0.274	0.187	0.113	0.164	0.175	0.215
acsm1	0.148	0.224	0.312	0.197	0.11	0.156	0.167	0.22

malat1	0.161	0.289	0.424	0.199	0.136	0.151	0.17	0.25
krtap15-1	0.161	0.209	0.263	0.16	0.136	0.168	0.175	0.214
rbm3	0.179	0.29	0.445	0.209	0.126	0.164	0.18	0.265
arhgdib	0.179	0.239	0.289	0.242	0.112	0.202	0.211	0.25
gbp6	0.179	0.207	0.254	0.218	0.132	0.168	0.173	0.209
il1rapl2	0.179	0.224	0.214	0.195	0.128	0.177	0.184	0.205
gucy2c	0.179	0.236	0.258	0.192	0.14	0.19	0.199	0.231
inpp5k	0.179	0.21	0.239	0.166	0.16	0.172	0.178	0.209
sctr	0.179	0.246	0.312	0.247	0.101	0.178	0.188	0.237
rrbp1	0.179	0.276	0.427	0.247	0.117	0.19	0.204	0.283
vps35	0.179	0.237	0.455	0.237	0.137	0.191	0.2	0.293
fam65a	0.179	0.248	0.324	0.231	0.13	0.197	0.208	0.258
tfip11	0.179	0.255	0.362	0.202	0.116	0.181	0.192	0.255
znf467	0.179	0.213	0.241	0.25	0.114	0.139	0.145	0.18
zar1	0.179	0.255	0.294	0.156	0.13	0.19	0.201	0.241
cdk2ap1	0.179	0.291	0.372	0.182	0.137	0.194	0.21	0.269
caskin1	0.179	0.239	0.334	0.206	0.152	0.194	0.203	0.257
cog8	0.179	0.264	0.438	0.255	0.147	0.186	0.199	0.283
neurod6	0.178	0.224	0.224	0.138	0.102	0.178	0.185	0.209
gdf6	0.178	0.207	0.168	0.112	0.152	0.154	0.16	0.171
slc27a4	0.178	0.217	0.263	0.135	0.127	0.197	0.203	0.239
taz	0.178	0.247	0.371	0.172	0.162	0.196	0.206	0.271
rab20	0.178	0.255	0.39	0.217	0.139	0.175	0.186	0.258
btn3a1	0.177	0.251	0.264	0.207	0.121	0.167	0.178	0.212
mvd	0.177	0.263	0.433	0.304	0.132	0.188	0.201	0.284
cdc5l	0.177	0.26	0.441	0.249	0.14	0.191	0.203	0.288
fbxl16	0.177	0.245	0.27	0.131	0.102	0.18	0.19	0.225
or7e24	0.178	0.247	0.324	0.203	0.118	0.191	0.202	0.252
clec18a	0.178	0.258	0.296	0.178	0.13	0.169	0.181	0.224
adssl1	0.178	0.267	0.466	0.281	0.192	0.184	0.197	0.29
fgf19	0.178	0.197	0.178	0.138	0.172	0.249	0.252	0.258
gng12	0.178	0.25	0.385	0.265	0.13	0.205	0.216	0.284
inhbb	0.178	0.225	0.193	0.183	0.132	0.181	0.188	0.202
dnah5	0.178	0.238	0.296	0.17	0.1	0.196	0.205	0.247
linc00152	0.178	0.252	0.285	0.117	0.213	0.187	0.198	0.236
scamp3	0.178	0.241	0.351	0.221	0.103	0.209	0.219	0.277
gimap1	0.179	0.268	0.285	0.241	0.213	0.149	0.163	0.203
upf2	0.179	0.254	0.469	0.247	0.146	0.179	0.19	0.286
ano10	0.181	0.243	0.299	0.158	0.126	0.193	0.203	0.246
inpp5f	0.181	0.263	0.358	0.23	0.163	0.217	0.229	0.285
mir221	0.181	0.303	0.326	0.178	0.12	0.161	0.179	0.226

gcm1	0.181	0.248	0.301	0.17	0.142	0.182	0.193	0.237
znf18	0.181	0.26	0.325	0.163	0.174	0.208	0.219	0.267
spa17	0.18	0.299	0.412	0.283	0.154	0.189	0.206	0.278
brms1	0.181	0.277	0.355	0.182	0.107	0.175	0.19	0.248
eif3cl	0.181	0.251	0.428	0.205	0.127	0.175	0.185	0.27
mir129-2	0.181	0.294	0.348	0.175	0.127	0.197	0.213	0.265
mkl1	0.181	0.231	0.336	0.184	0.123	0.17	0.178	0.236
mir365b	0.181	0.287	0.405	0.185	0.132	0.186	0.202	0.273
dclk2	0.181	0.234	0.274	0.169	0.108	0.157	0.166	0.206
eps15	0.181	0.224	0.369	0.244	0.107	0.222	0.228	0.293
cadm4	0.181	0.268	0.307	0.205	0.133	0.194	0.206	0.249
isg20l2	0.181	0.262	0.42	0.22	0.117	0.185	0.197	0.277
tpr	0.181	0.254	0.441	0.277	0.135	0.189	0.2	0.286
actl6b	0.18	0.259	0.349	0.163	0.22	0.195	0.207	0.263
nup210l	0.18	0.278	0.346	0.203	0.186	0.197	0.211	0.264
exosc10	0.18	0.263	0.412	0.177	0.152	0.163	0.175	0.254
timm10b	0.18	0.252	0.41	0.234	0.127	0.171	0.182	0.261
shd	0.18	0.202	0.259	0.327	0.161	0.166	0.17	0.209
rexo1	0.18	0.241	0.377	0.261	0.162	0.189	0.198	0.266
hist3h2ba	0.18	0.268	0.446	0.245	0.222	0.173	0.186	0.274
rpl13a	0.18	0.265	0.415	0.22	0.162	0.159	0.172	0.252
znf649	0.18	0.223	0.284	0.191	0.187	0.157	0.164	0.209
she	0.18	0.202	0.259	0.327	0.161	0.166	0.17	0.209
pa2g4	0.18	0.236	0.339	0.187	0.14	0.146	0.156	0.217
tulp4	0.18	0.257	0.424	0.268	0.199	0.209	0.22	0.299
calml5	0.18	0.252	0.349	0.194	0.12	0.193	0.204	0.261
tdgf1p3	0.18	0.263	0.308	0.192	0.147	0.181	0.193	0.238
cabyr	0.18	0.23	0.281	0.255	0.124	0.179	0.187	0.228
kiaa1328	0.18	0.258	0.46	0.241	0.193	0.208	0.22	0.309
dgcr2	0.18	0.239	0.275	0.163	0.12	0.191	0.2	0.237
mir184	0.18	0.27	0.327	0.165	0.253	0.184	0.197	0.246
armcx1	0.177	0.237	0.266	0.223	0.177	0.182	0.191	0.226
catr1	0.177	0.278	0.42	0.227	0.165	0.201	0.215	0.29
fam5b	0.175	0.217	0.252	0.171	0.135	0.197	0.203	0.235
pex11g	0.175	0.233	0.358	0.221	0.132	0.161	0.17	0.236
mustn1	0.175	0.235	0.254	0.15	0.13	0.161	0.171	0.204
klf15	0.175	0.219	0.276	0.167	0.362	0.175	0.182	0.223
rxfp3	0.175	0.214	0.274	0.231	0.103	0.195	0.201	0.24
ceacam3	0.175	0.262	0.28	0.216	0.122	0.167	0.18	0.217
irak1bp1	0.175	0.186	0.202	0.136	0.168	0.176	0.179	0.201
capn5	0.175	0.255	0.314	0.172	0.112	0.175	0.187	0.235

mos	0.175	0.271	0.417	0.256	0.101	0.172	0.186	0.264
gpr123	0.175	0.236	0.313	0.194	0.132	0.16	0.17	0.221
pclo	0.175	0.23	0.29	0.204	0.106	0.189	0.197	0.24
rpl7	0.175	0.251	0.365	0.223	0.139	0.175	0.186	0.251
defb129	0.175	0.223	0.312	0.157	0.135	0.202	0.209	0.258
rragb	0.175	0.211	0.359	0.174	0.128	0.163	0.169	0.238
rab8b	0.175	0.244	0.373	0.254	0.131	0.184	0.194	0.261
ehd3	0.175	0.241	0.366	0.23	0.111	0.19	0.2	0.265
six1	0.175	0.227	0.203	0.101	0.112	0.228	0.235	0.247
scgb3a1	0.175	0.255	0.287	0.184	0.103	0.169	0.181	0.222
osbpl3	0.174	0.245	0.424	0.221	0.118	0.171	0.181	0.266
sh3pxd2b	0.174	0.209	0.244	0.208	0.149	0.166	0.171	0.205
epb41l3	0.174	0.281	0.401	0.217	0.107	0.183	0.199	0.269
atg9b	0.174	0.235	0.364	0.164	0.106	0.165	0.175	0.242
rnaseh2b	0.174	0.264	0.401	0.158	0.141	0.144	0.157	0.234
chsy1	0.174	0.239	0.321	0.174	0.136	0.169	0.179	0.232
ap2a2	0.174	0.221	0.345	0.28	0.116	0.189	0.196	0.257
akap8	0.174	0.264	0.364	0.242	0.148	0.143	0.157	0.223
tpm4	0.175	0.269	0.408	0.215	0.112	0.165	0.179	0.255
plcz1	0.175	0.242	0.305	0.248	0.132	0.159	0.17	0.218
tmem134	0.175	0.187	0.183	0.24	0.101	0.153	0.156	0.175
tspan16	0.175	0.257	0.321	0.221	0.118	0.167	0.18	0.23
atg3	0.175	0.226	0.428	0.173	0.112	0.181	0.189	0.276
ttc7a	0.175	0.249	0.232	0.162	0.105	0.164	0.176	0.2
nol4	0.175	0.222	0.275	0.177	0.138	0.166	0.173	0.215
eif2b3	0.175	0.257	0.456	0.263	0.105	0.179	0.191	0.283
tubb6	0.176	0.25	0.346	0.202	0.134	0.183	0.194	0.252
ehd2	0.176	0.23	0.339	0.252	0.102	0.196	0.204	0.261
sfxn3	0.177	0.267	0.348	0.212	0.195	0.189	0.203	0.258
ifitm3	0.177	0.252	0.292	0.184	0.145	0.149	0.161	0.205
psmc6	0.177	0.233	0.506	0.263	0.149	0.202	0.21	0.318
neurod4	0.177	0.224	0.171	0.114	0.101	0.208	0.215	0.219
safb2	0.177	0.235	0.332	0.156	0.504	0.147	0.157	0.216
amph	0.176	0.218	0.315	0.292	0.116	0.195	0.201	0.253
pcdh8	0.176	0.224	0.23	0.106	0.109	0.218	0.224	0.246
mir376a1	0.177	0.245	0.287	0.155	0.125	0.183	0.194	0.234
gfm1	0.177	0.251	0.414	0.232	0.148	0.173	0.184	0.264
hsd17b12	0.177	0.234	0.292	0.202	0.155	0.179	0.188	0.232
impact	0.177	0.251	0.37	0.182	0.132	0.176	0.187	0.253
slirp	0.177	0.227	0.356	0.177	0.255	0.182	0.19	0.254
dync1li1	0.177	0.241	0.307	0.209	0.103	0.207	0.217	0.261

mat2a	0.177	0.257	0.368	0.203	0.139	0.165	0.177	0.243
rmnd1	0.177	0.258	0.403	0.211	0.207	0.186	0.198	0.273
ndrg3	0.177	0.252	0.363	0.152	0.108	0.177	0.188	0.251
lsm12	0.176	0.247	0.424	0.208	0.213	0.196	0.206	0.287
zcchc4	0.176	0.247	0.424	0.208	0.213	0.196	0.206	0.287
prlhr	0.176	0.22	0.246	0.195	0.161	0.166	0.173	0.205
rnu1-13p	0.176	0.267	0.442	0.235	0.177	0.168	0.182	0.269
rnu1-15p	0.176	0.267	0.442	0.235	0.177	0.168	0.182	0.269
mir30b	0.176	0.215	0.292	0.167	0.118	0.125	0.132	0.183
cpeb2	0.176	0.277	0.473	0.243	0.174	0.173	0.188	0.283
emilin2	0.176	0.257	0.321	0.193	0.157	0.162	0.174	0.225
nell2	0.176	0.234	0.279	0.205	0.133	0.174	0.183	0.223
mir125b2	0.176	0.267	0.316	0.168	0.108	0.178	0.192	0.238
atp5g2	0.176	0.215	0.293	0.216	0.13	0.185	0.191	0.237
upf1	0.176	0.268	0.5	0.23	0.145	0.184	0.197	0.301
pabpn1l	0.176	0.277	0.434	0.236	0.157	0.191	0.205	0.286
phb2	0.176	0.226	0.284	0.16	0.349	0.134	0.143	0.189
uevld	0.176	0.27	0.537	0.251	0.169	0.202	0.215	0.328
krt12	0.176	0.232	0.239	0.122	0.149	0.198	0.206	0.232
mir141	0.176	0.23	0.3	0.201	0.147	0.201	0.209	0.254
tmf1	0.181	0.247	0.388	0.23	0.236	0.205	0.214	0.284
trex1	0.182	0.248	0.393	0.2	0.109	0.14	0.151	0.228
uck1	0.187	0.246	0.375	0.264	0.124	0.16	0.169	0.239
vps26b	0.187	0.257	0.425	0.26	0.118	0.201	0.211	0.291
mir1301	0.187	0.287	0.38	0.182	0.108	0.176	0.191	0.255
defb105a	0.187	0.27	0.387	0.188	0.136	0.197	0.21	0.276
oaz3	0.187	0.27	0.456	0.213	0.2	0.202	0.214	0.302
creb3l1	0.187	0.244	0.327	0.184	0.12	0.187	0.196	0.249
c2orf68	0.187	0.261	0.291	0.198	0.13	0.176	0.187	0.227
ocrl	0.187	0.238	0.348	0.263	0.129	0.204	0.212	0.27
wwtr1	0.187	0.264	0.33	0.159	0.123	0.199	0.21	0.26
svil	0.187	0.257	0.378	0.279	0.213	0.192	0.202	0.269
tdpx2	0.187	0.29	0.428	0.302	0.12	0.211	0.226	0.301
mir24-2	0.187	0.27	0.36	0.181	0.1	0.18	0.193	0.253
il1rap	0.187	0.186	0.142	0.161	0.112	0.178	0.179	0.183
ccdc67	0.187	0.27	0.386	0.224	0.198	0.198	0.21	0.277
crb2	0.187	0.234	0.261	0.232	0.1	0.2	0.207	0.24
hand2	0.187	0.21	0.161	0.103	0.102	0.219	0.223	0.225
prickle4	0.187	0.286	0.362	0.217	0.135	0.211	0.225	0.281
mir425	0.186	0.27	0.369	0.15	0.154	0.17	0.182	0.246
mir218-1	0.186	0.303	0.356	0.158	0.111	0.198	0.215	0.268

mfn2	0.186	0.257	0.406	0.179	0.113	0.189	0.2	0.275
tmx3	0.186	0.24	0.315	0.196	0.156	0.219	0.227	0.273
med13l	0.186	0.25	0.327	0.178	0.29	0.189	0.199	0.251
vps4a	0.186	0.263	0.478	0.232	0.104	0.211	0.222	0.317
tmem189-ube2v1	0.186	0.258	0.429	0.254	0.116	0.21	0.22	0.301
sowahb	0.186	0.236	0.263	0.183	0.14	0.253	0.26	0.287
naca	0.186	0.272	0.443	0.252	0.19	0.177	0.19	0.276
cirbp	0.186	0.248	0.388	0.248	0.1	0.181	0.191	0.263
pcbp4	0.186	0.296	0.487	0.256	0.138	0.204	0.22	0.314
rnf133	0.186	0.226	0.265	0.121	0.115	0.223	0.229	0.261
stmn2	0.186	0.246	0.277	0.19	0.117	0.211	0.22	0.254
golim4	0.186	0.276	0.532	0.249	0.128	0.207	0.22	0.33
ermp1	0.186	0.237	0.225	0.159	0.131	0.179	0.187	0.21
dsg4	0.186	0.256	0.275	0.143	0.121	0.197	0.207	0.241
tmem14c	0.186	0.271	0.358	0.219	0.123	0.203	0.215	0.272
prap1	0.187	0.266	0.336	0.221	0.219	0.16	0.172	0.227
zmynd19	0.187	0.234	0.351	0.231	0.129	0.192	0.199	0.26
fcho2	0.188	0.225	0.35	0.237	0.104	0.225	0.231	0.289
magec2	0.188	0.316	0.372	0.224	0.167	0.186	0.204	0.261
lsm4	0.188	0.288	0.466	0.258	0.119	0.201	0.216	0.304
allc	0.188	0.25	0.298	0.164	0.14	0.237	0.246	0.284
tacstd2	0.188	0.302	0.321	0.179	0.1	0.189	0.205	0.248
batf3	0.188	0.262	0.243	0.173	0.114	0.123	0.135	0.165
klf3	0.188	0.268	0.361	0.207	0.126	0.152	0.165	0.228
ciita	0.188	0.272	0.31	0.244	0.103	0.142	0.155	0.203
has2-as1	0.189	0.271	0.329	0.213	0.12	0.172	0.185	0.236
eea1	0.189	0.245	0.4	0.274	0.107	0.172	0.181	0.258
spg20	0.189	0.251	0.41	0.194	0.101	0.228	0.237	0.31
phc1	0.189	0.281	0.375	0.224	0.162	0.191	0.205	0.267
scarna15	0.189	0.254	0.374	0.152	0.146	0.159	0.17	0.238
hoxc6	0.189	0.244	0.247	0.143	0.141	0.233	0.241	0.264
ervh-2	0.189	0.277	0.386	0.245	0.161	0.175	0.189	0.256
mospd1	0.189	0.254	0.329	0.196	0.117	0.17	0.181	0.234
hmga2	0.188	0.292	0.397	0.185	0.138	0.183	0.198	0.266
ssx1	0.188	0.303	0.398	0.232	0.189	0.177	0.194	0.261
pdk3	0.187	0.237	0.391	0.211	0.226	0.164	0.172	0.248
alg3p1	0.187	0.273	0.411	0.209	0.139	0.18	0.193	0.268
gadd45a	0.187	0.299	0.423	0.205	0.111	0.196	0.212	0.287
pou2af1	0.187	0.28	0.256	0.241	0.138	0.131	0.146	0.177
chka	0.187	0.27	0.392	0.259	0.147	0.184	0.196	0.266
meis3	0.187	0.24	0.268	0.175	0.105	0.234	0.241	0.272

eftud2	0.187	0.276	0.389	0.23	0.176	0.208	0.221	0.287
rfx1	0.187	0.256	0.353	0.241	0.284	0.158	0.169	0.231
pskh1	0.188	0.277	0.414	0.308	0.114	0.184	0.197	0.273
mir15a	0.188	0.301	0.343	0.198	0.126	0.162	0.179	0.231
n4bp3	0.188	0.25	0.347	0.192	0.115	0.184	0.193	0.252
ankrd30a	0.188	0.314	0.433	0.253	0.133	0.182	0.2	0.277
kiaa0319	0.188	0.23	0.28	0.162	0.117	0.185	0.192	0.232
map1lc3b	0.188	0.266	0.429	0.207	0.132	0.177	0.189	0.271
fabp12	0.188	0.28	0.404	0.207	0.21	0.201	0.215	0.285
fth1p15	0.185	0.268	0.408	0.215	0.182	0.191	0.203	0.277
pom121b	0.185	0.266	0.389	0.265	0.109	0.17	0.182	0.253
or2a7	0.183	0.231	0.186	0.101	0.157	0.219	0.226	0.234
tptep1	0.183	0.222	0.291	0.154	0.107	0.198	0.205	0.248
eif2b4	0.183	0.248	0.411	0.259	0.105	0.181	0.191	0.27
fstl5	0.183	0.251	0.308	0.163	0.148	0.186	0.196	0.242
dync1i1	0.183	0.256	0.384	0.255	0.155	0.191	0.202	0.27
pmepa1	0.183	0.233	0.245	0.142	0.315	0.188	0.196	0.224
lin28b	0.183	0.284	0.35	0.164	0.157	0.164	0.179	0.235
ndufaf1	0.183	0.261	0.456	0.24	0.216	0.182	0.194	0.285
tcea3	0.183	0.262	0.315	0.228	0.17	0.159	0.171	0.22
foxd3	0.183	0.24	0.187	0.114	0.119	0.213	0.222	0.229
pkd1p2	0.183	0.273	0.385	0.207	0.127	0.211	0.224	0.288
pkd1p3	0.183	0.273	0.385	0.207	0.127	0.211	0.224	0.288
papolb	0.183	0.255	0.417	0.228	0.191	0.194	0.204	0.282
c21orf62	0.183	0.256	0.33	0.168	0.127	0.178	0.19	0.242
pwp2	0.183	0.255	0.355	0.228	0.137	0.19	0.201	0.26
cdc73	0.183	0.25	0.285	0.2	0.144	0.166	0.176	0.217
apitd1-cort	0.183	0.268	0.429	0.192	0.144	0.182	0.195	0.277
tyw5	0.182	0.231	0.393	0.237	0.192	0.178	0.186	0.262
wbp1	0.182	0.25	0.414	0.28	0.117	0.189	0.199	0.278
sumf1	0.182	0.251	0.415	0.18	0.109	0.194	0.204	0.282
leo1	0.182	0.247	0.373	0.208	0.16	0.169	0.179	0.247
srpr	0.182	0.209	0.248	0.245	0.118	0.184	0.188	0.221
ist1	0.182	0.263	0.446	0.222	0.137	0.214	0.226	0.31
nkx2-3	0.182	0.24	0.215	0.142	0.107	0.185	0.194	0.213
tincr	0.182	0.28	0.355	0.165	0.118	0.17	0.185	0.243
pop4	0.182	0.257	0.488	0.231	0.132	0.177	0.188	0.29
cyp26b1	0.182	0.227	0.228	0.114	0.116	0.22	0.226	0.247
frg2	0.182	0.265	0.383	0.218	0.238	0.206	0.218	0.283
slc30a10	0.182	0.245	0.371	0.192	0.162	0.181	0.19	0.257
ccp110	0.182	0.27	0.377	0.164	0.1	0.211	0.223	0.285

arhgef16	0.182	0.235	0.304	0.192	0.106	0.199	0.207	0.252
rab33b	0.182	0.246	0.435	0.237	0.107	0.202	0.212	0.296
tes	0.182	0.265	0.373	0.22	0.119	0.206	0.218	0.28
spata19	0.182	0.297	0.337	0.215	0.139	0.185	0.202	0.25
pkd1p5	0.183	0.273	0.385	0.207	0.127	0.211	0.224	0.288
pkd1p4	0.183	0.273	0.385	0.207	0.127	0.211	0.224	0.288
mir3660	0.185	0.264	0.357	0.178	0.147	0.19	0.202	0.261
mir3661	0.185	0.264	0.357	0.178	0.147	0.19	0.202	0.261
ikzf3	0.185	0.273	0.308	0.258	0.128	0.158	0.171	0.217
mir3663	0.185	0.264	0.357	0.178	0.147	0.19	0.202	0.261
mir3171	0.185	0.264	0.357	0.178	0.147	0.19	0.202	0.261
mir548t	0.185	0.264	0.357	0.178	0.147	0.19	0.202	0.261
mir3662	0.185	0.264	0.357	0.178	0.147	0.19	0.202	0.261
s100a11	0.185	0.256	0.328	0.22	0.107	0.198	0.209	0.259
nr2f2	0.185	0.231	0.234	0.124	0.249	0.201	0.208	0.232
krt75	0.185	0.264	0.27	0.121	0.108	0.187	0.199	0.231
pnpla8	0.185	0.235	0.349	0.209	0.159	0.181	0.189	0.251
dnah12	0.185	0.264	0.392	0.229	0.183	0.214	0.226	0.293
ctdsp2	0.185	0.229	0.282	0.199	0.378	0.163	0.171	0.214
har1a	0.185	0.27	0.372	0.198	0.127	0.175	0.188	0.252
prelid2	0.185	0.245	0.209	0.153	0.133	0.214	0.223	0.236
smox	0.185	0.261	0.433	0.2	0.103	0.203	0.214	0.295
mir3659	0.185	0.264	0.357	0.178	0.147	0.19	0.202	0.261
mir3664	0.185	0.264	0.357	0.178	0.147	0.19	0.202	0.261
txnl4a	0.184	0.261	0.463	0.249	0.182	0.21	0.221	0.311
chpt1	0.184	0.305	0.495	0.263	0.193	0.181	0.199	0.296
map4k2	0.184	0.212	0.245	0.214	0.145	0.188	0.193	0.224
cenpm	0.184	0.238	0.214	0.169	0.104	0.187	0.196	0.214
als2cr12	0.184	0.27	0.337	0.238	0.209	0.176	0.189	0.242
pkd1p1	0.183	0.273	0.385	0.207	0.127	0.211	0.224	0.288
pkd1p6	0.183	0.273	0.385	0.207	0.127	0.211	0.224	0.288
eda	0.184	0.224	0.199	0.132	0.141	0.227	0.233	0.244
gdf10	0.184	0.221	0.224	0.124	0.152	0.154	0.161	0.188
mir3200	0.185	0.264	0.357	0.178	0.147	0.19	0.202	0.261
mir2355	0.185	0.264	0.357	0.178	0.147	0.19	0.202	0.261
oaz2	0.185	0.274	0.416	0.219	0.14	0.194	0.208	0.283
sgpp1	0.184	0.232	0.309	0.246	0.115	0.181	0.188	0.237
sept14	0.184	0.247	0.359	0.244	0.101	0.197	0.207	0.268
fgd1	0.184	0.228	0.304	0.231	0.111	0.202	0.209	0.255
pkdrej	0.174	0.243	0.32	0.216	0.112	0.193	0.203	0.253
nol11	0.174	0.252	0.447	0.178	0.181	0.183	0.195	0.284

zfp2	0.165	0.23	0.363	0.164	0.159	0.173	0.183	0.25
daw1	0.165	0.213	0.265	0.165	0.119	0.174	0.182	0.22
pcp2	0.165	0.187	0.197	0.185	0.104	0.174	0.178	0.199
bnc1	0.165	0.26	0.359	0.226	0.134	0.175	0.188	0.25
exosc9	0.165	0.237	0.365	0.147	0.118	0.14	0.151	0.221
aacs	0.165	0.217	0.356	0.218	0.111	0.156	0.165	0.233
slc16a10	0.165	0.237	0.322	0.178	0.171	0.163	0.174	0.228
limd1	0.165	0.22	0.316	0.178	0.156	0.174	0.183	0.236
prkx	0.165	0.236	0.296	0.198	0.102	0.17	0.181	0.226
cbll1	0.165	0.214	0.243	0.118	0.112	0.154	0.162	0.195
hgsnat	0.165	0.222	0.383	0.197	0.121	0.171	0.179	0.254
atp11a	0.165	0.212	0.273	0.174	0.115	0.164	0.172	0.214
pex6	0.165	0.207	0.355	0.168	0.171	0.196	0.202	0.268
ubr7	0.165	0.221	0.337	0.209	0.172	0.168	0.176	0.237
ppfia1	0.165	0.243	0.292	0.189	0.111	0.147	0.159	0.204
bloc1s1	0.165	0.206	0.289	0.15	0.233	0.141	0.148	0.198
copg1	0.165	0.234	0.403	0.252	0.116	0.182	0.193	0.27
fndc3b	0.165	0.268	0.354	0.19	0.113	0.173	0.188	0.247
mob3a	0.164	0.202	0.252	0.151	0.117	0.158	0.164	0.201
tuba4a	0.164	0.251	0.449	0.285	0.118	0.17	0.183	0.274
frg2b	0.164	0.247	0.372	0.188	0.216	0.185	0.197	0.263
mob3c	0.164	0.202	0.252	0.151	0.117	0.158	0.164	0.201
kiaa0247	0.164	0.205	0.26	0.141	0.116	0.188	0.195	0.231
mybl2	0.164	0.287	0.419	0.214	0.213	0.161	0.179	0.256
rasd1	0.164	0.2	0.266	0.237	0.127	0.139	0.146	0.19
psat1	0.164	0.234	0.352	0.179	0.228	0.196	0.206	0.267
hnrnpa2b1	0.164	0.259	0.458	0.247	0.107	0.151	0.165	0.259
hmga1	0.165	0.273	0.451	0.207	0.204	0.158	0.174	0.263
akap1	0.165	0.201	0.288	0.237	0.115	0.13	0.137	0.188
cdk19	0.165	0.271	0.41	0.235	0.179	0.164	0.179	0.256
mir1-2	0.165	0.235	0.337	0.142	0.119	0.157	0.168	0.227
nt5dc1	0.164	0.236	0.375	0.237	0.124	0.17	0.18	0.251
trim14	0.164	0.235	0.384	0.195	0.205	0.166	0.177	0.25
cend1	0.164	0.253	0.237	0.135	0.116	0.144	0.157	0.185
znf43	0.165	0.23	0.361	0.185	0.148	0.178	0.187	0.253
dpagt1	0.165	0.248	0.35	0.209	0.175	0.165	0.177	0.238
best3	0.166	0.204	0.296	0.211	0.102	0.151	0.157	0.209
rgs12	0.166	0.195	0.244	0.2	0.112	0.141	0.146	0.184
trim25	0.166	0.182	0.298	0.138	0.109	0.158	0.161	0.216
b4galt1	0.166	0.268	0.387	0.227	0.102	0.167	0.182	0.252
itm2a	0.166	0.225	0.237	0.147	0.145	0.146	0.156	0.187

ppp1r14c	0.166	0.227	0.371	0.229	0.121	0.152	0.161	0.233
tmem190	0.166	0.263	0.348	0.225	0.186	0.17	0.184	0.242
gtf2ird2	0.166	0.242	0.383	0.195	0.179	0.186	0.196	0.267
mir19b1	0.166	0.284	0.446	0.196	0.137	0.155	0.172	0.259
eif3h	0.167	0.236	0.318	0.154	0.133	0.148	0.159	0.214
cecr6	0.167	0.257	0.401	0.224	0.156	0.178	0.192	0.266
prpf4	0.167	0.258	0.511	0.261	0.169	0.184	0.197	0.305
cpsf2	0.166	0.205	0.297	0.194	0.16	0.174	0.18	0.229
cd99	0.166	0.292	0.358	0.216	0.116	0.145	0.164	0.223
tsix	0.166	0.272	0.397	0.214	0.217	0.162	0.178	0.251
erp29	0.166	0.24	0.384	0.222	0.125	0.166	0.177	0.25
serpinf1	0.166	0.235	0.274	0.193	0.108	0.157	0.168	0.208
cdcp1	0.166	0.234	0.294	0.203	0.147	0.15	0.161	0.208
st5	0.165	0.192	0.222	0.24	0.128	0.14	0.145	0.177
ptgr2	0.165	0.252	0.429	0.177	0.135	0.175	0.188	0.272
rhobtb3	0.165	0.223	0.401	0.208	0.122	0.189	0.198	0.276
gprc5d	0.165	0.221	0.222	0.125	0.106	0.134	0.143	0.171
asun	0.165	0.276	0.388	0.191	0.109	0.185	0.201	0.268
specc1	0.165	0.201	0.28	0.191	0.104	0.162	0.168	0.213
gabpb2	0.165	0.255	0.328	0.191	0.179	0.159	0.172	0.226
hnrnpu	0.165	0.249	0.436	0.301	0.171	0.17	0.183	0.27
zbtb17	0.165	0.242	0.387	0.178	0.177	0.158	0.17	0.244
sbds	0.166	0.252	0.343	0.201	0.112	0.167	0.179	0.238
dleu1	0.166	0.241	0.317	0.17	0.163	0.169	0.18	0.232
clk4	0.166	0.237	0.411	0.239	0.133	0.181	0.192	0.272
palm2-akap2	0.165	0.245	0.379	0.259	0.144	0.175	0.186	0.256
pro2268	0.165	0.28	0.305	0.172	0.102	0.162	0.179	0.222
rbfox2	0.165	0.231	0.313	0.178	0.193	0.179	0.188	0.239
ybx1	0.164	0.254	0.436	0.202	0.17	0.147	0.16	0.249
tfap2c	0.164	0.252	0.315	0.155	0.257	0.157	0.17	0.22
eef1a1	0.162	0.222	0.381	0.244	0.129	0.19	0.199	0.27
g6pc3	0.162	0.216	0.304	0.21	0.144	0.171	0.179	0.23
tspan11	0.162	0.241	0.402	0.247	0.137	0.162	0.173	0.252
loc440895	0.162	0.217	0.305	0.171	0.102	0.17	0.178	0.229
ern2	0.162	0.211	0.335	0.181	0.105	0.165	0.173	0.234
foxr1	0.162	0.213	0.246	0.107	0.17	0.167	0.175	0.208
rabl2a	0.162	0.26	0.403	0.203	0.177	0.174	0.188	0.263
fbxo38	0.162	0.236	0.312	0.159	0.172	0.176	0.187	0.237
ccny	0.162	0.243	0.281	0.182	0.125	0.2	0.211	0.248
sox11	0.163	0.278	0.304	0.174	0.154	0.161	0.177	0.22
sprr2g	0.163	0.251	0.431	0.18	0.135	0.164	0.177	0.263

c16orf3	0.163	0.252	0.447	0.249	0.164	0.177	0.19	0.279
ruvbl2	0.163	0.223	0.404	0.136	0.102	0.163	0.173	0.254
mir127	0.163	0.259	0.32	0.154	0.158	0.144	0.158	0.211
iqsec3	0.162	0.219	0.331	0.224	0.117	0.155	0.164	0.224
slc25a5	0.162	0.201	0.269	0.168	0.129	0.179	0.185	0.226
tpte	0.162	0.238	0.357	0.181	0.175	0.178	0.19	0.253
nudt16	0.162	0.25	0.479	0.2	0.118	0.204	0.216	0.313
glipr1l1	0.162	0.278	0.507	0.23	0.152	0.164	0.181	0.287
parn	0.162	0.25	0.486	0.255	0.107	0.15	0.163	0.268
leng9	0.162	0.216	0.285	0.196	0.135	0.163	0.172	0.217
plin3	0.162	0.235	0.442	0.23	0.118	0.164	0.175	0.267
ttll7	0.162	0.204	0.311	0.173	0.101	0.153	0.16	0.216
degs1	0.161	0.228	0.376	0.209	0.154	0.182	0.192	0.262
cidea	0.162	0.207	0.287	0.155	0.163	0.171	0.178	0.225
zc3h7a	0.162	0.216	0.285	0.196	0.135	0.163	0.172	0.217
prepl	0.162	0.231	0.384	0.196	0.127	0.141	0.152	0.228
linc00299	0.162	0.232	0.329	0.186	0.164	0.144	0.155	0.214
znf16	0.162	0.234	0.455	0.206	0.191	0.17	0.18	0.275
oaz1	0.162	0.255	0.44	0.197	0.14	0.179	0.192	0.279
klhdc2	0.162	0.216	0.311	0.148	0.15	0.172	0.18	0.233
c9orf24	0.162	0.227	0.256	0.168	0.134	0.173	0.182	0.216
pbx1	0.162	0.216	0.23	0.156	0.107	0.194	0.202	0.227
usp22	0.162	0.233	0.376	0.185	0.162	0.16	0.17	0.242
hars	0.163	0.24	0.368	0.223	0.151	0.171	0.182	0.249
mybl1	0.163	0.256	0.378	0.208	0.233	0.165	0.178	0.247
magef1	0.163	0.258	0.31	0.187	0.13	0.176	0.19	0.236
rfxank	0.163	0.233	0.372	0.249	0.163	0.139	0.15	0.223
hcar3	0.163	0.198	0.214	0.194	0.115	0.14	0.146	0.174
nacc1	0.163	0.242	0.337	0.167	0.344	0.163	0.175	0.233
atg12	0.163	0.216	0.427	0.172	0.115	0.165	0.173	0.263
vps72	0.163	0.229	0.294	0.216	0.101	0.155	0.165	0.212
cstf2	0.163	0.229	0.39	0.233	0.119	0.142	0.152	0.23
rreb1	0.164	0.238	0.338	0.199	0.283	0.143	0.155	0.216
dbn1	0.164	0.246	0.373	0.217	0.12	0.162	0.174	0.243
vgll1	0.164	0.207	0.197	0.12	0.134	0.177	0.184	0.202
actn4	0.164	0.234	0.331	0.226	0.115	0.175	0.186	0.242
ceacam22p	0.164	0.205	0.199	0.252	0.141	0.15	0.157	0.178
tmed3	0.164	0.261	0.427	0.243	0.11	0.173	0.187	0.27
wdr60	0.164	0.21	0.285	0.165	0.148	0.174	0.181	0.227
siah1	0.164	0.211	0.425	0.165	0.135	0.204	0.211	0.297
fam49b	0.164	0.21	0.285	0.165	0.148	0.174	0.181	0.227

klk10	0.163	0.266	0.347	0.203	0.146	0.164	0.179	0.237
znf624	0.163	0.171	0.189	0.117	0.143	0.19	0.192	0.211
gid8	0.163	0.204	0.366	0.192	0.111	0.162	0.169	0.241
eml5	0.163	0.22	0.279	0.146	0.149	0.21	0.218	0.257
ervh-3	0.163	0.216	0.248	0.17	0.118	0.103	0.112	0.152
ppp1r3c	0.163	0.209	0.4	0.191	0.146	0.186	0.193	0.273
gadd45g	0.163	0.268	0.363	0.212	0.147	0.168	0.183	0.246
ssx2ip	0.163	0.217	0.292	0.211	0.111	0.16	0.168	0.216
dusp23	0.163	0.19	0.264	0.199	0.104	0.169	0.174	0.215
fnbp1	0.163	0.217	0.322	0.245	0.105	0.17	0.178	0.234
slc38a1	0.163	0.233	0.343	0.18	0.139	0.165	0.176	0.236
prcc	0.163	0.274	0.379	0.208	0.139	0.151	0.168	0.236
znf585a	0.163	0.171	0.189	0.117	0.143	0.19	0.192	0.211
fez1	0.163	0.216	0.31	0.198	0.101	0.179	0.187	0.239
acss2	0.163	0.215	0.369	0.168	0.122	0.153	0.161	0.234
rnd2	0.163	0.205	0.263	0.176	0.102	0.148	0.155	0.196
emp1	0.163	0.256	0.3	0.169	0.131	0.19	0.204	0.246
c8orf17	0.167	0.257	0.362	0.261	0.117	0.16	0.173	0.237
psmd12	0.167	0.243	0.467	0.211	0.202	0.181	0.192	0.289
erg	0.172	0.266	0.307	0.163	0.179	0.159	0.173	0.219
ptbp3	0.172	0.284	0.489	0.244	0.17	0.163	0.179	0.279
tpcn2	0.172	0.203	0.278	0.187	0.112	0.168	0.173	0.218
tc2n	0.172	0.233	0.318	0.208	0.117	0.167	0.177	0.23
ufm1	0.172	0.252	0.494	0.207	0.112	0.195	0.206	0.309
znf664	0.172	0.263	0.356	0.231	0.107	0.187	0.2	0.259
rnvu1-18	0.172	0.243	0.387	0.285	0.131	0.173	0.184	0.257
lxn	0.172	0.267	0.318	0.204	0.165	0.164	0.178	0.227
iscu	0.172	0.246	0.448	0.185	0.124	0.168	0.179	0.271
tusc2	0.172	0.274	0.335	0.186	0.119	0.151	0.166	0.221
akap13	0.172	0.208	0.258	0.205	0.118	0.139	0.145	0.186
son	0.172	0.246	0.372	0.236	0.158	0.164	0.175	0.244
gpr128	0.172	0.219	0.278	0.145	0.11	0.152	0.16	0.204
med4	0.172	0.261	0.37	0.179	0.158	0.177	0.19	0.254
irs3p	0.172	0.17	0.157	0.182	0.104	0.182	0.183	0.193
fam5c	0.172	0.242	0.243	0.171	0.107	0.154	0.165	0.195
uqcrq	0.172	0.209	0.228	0.118	0.11	0.163	0.169	0.198
igfl3	0.172	0.231	0.307	0.171	0.136	0.179	0.188	0.237
rnf112	0.171	0.26	0.389	0.204	0.147	0.184	0.197	0.267
nmi	0.171	0.217	0.34	0.225	0.19	0.173	0.18	0.242
zfp36l1	0.171	0.242	0.317	0.202	0.115	0.174	0.184	0.235
lrrc2	0.171	0.234	0.351	0.171	0.111	0.131	0.141	0.208

fastkd2	0.171	0.208	0.274	0.269	0.101	0.166	0.172	0.214
tff1	0.171	0.279	0.365	0.198	0.194	0.169	0.185	0.246
acad11	0.171	0.222	0.35	0.184	0.169	0.18	0.188	0.251
znf445	0.171	0.243	0.393	0.233	0.162	0.187	0.198	0.271
cst6	0.171	0.254	0.309	0.159	0.108	0.17	0.183	0.23
ccnl2	0.172	0.274	0.43	0.271	0.162	0.173	0.188	0.269
rbmxl2	0.172	0.271	0.374	0.2	0.218	0.17	0.185	0.25
c1qtnf9b-as1	0.172	0.265	0.39	0.224	0.141	0.159	0.173	0.245
blcap	0.171	0.281	0.409	0.206	0.142	0.186	0.201	0.274
rab1c	0.171	0.255	0.304	0.201	0.158	0.18	0.193	0.237
mir196a2	0.171	0.24	0.299	0.158	0.132	0.173	0.183	0.229
znf134	0.171	0.228	0.308	0.208	0.11	0.203	0.211	0.259
slc44a1	0.172	0.238	0.344	0.228	0.13	0.157	0.168	0.229
ptpn18	0.172	0.214	0.255	0.289	0.117	0.149	0.156	0.194
psme3	0.173	0.29	0.516	0.251	0.182	0.198	0.214	0.318
mir3666	0.173	0.283	0.398	0.173	0.136	0.177	0.193	0.264
mir3665	0.173	0.283	0.398	0.173	0.136	0.177	0.193	0.264
ring1	0.173	0.255	0.364	0.138	0.208	0.174	0.186	0.25
dcp2	0.173	0.254	0.521	0.24	0.152	0.176	0.188	0.301
sltm	0.173	0.241	0.419	0.169	0.184	0.181	0.191	0.273
lcp1	0.173	0.27	0.392	0.276	0.127	0.159	0.174	0.245
psma7	0.173	0.254	0.532	0.224	0.163	0.201	0.213	0.326
exoc5	0.173	0.245	0.367	0.252	0.126	0.204	0.214	0.278
tial1	0.174	0.256	0.448	0.242	0.13	0.158	0.17	0.262
ssx5	0.174	0.279	0.326	0.218	0.134	0.167	0.183	0.232
chmp4b	0.174	0.234	0.45	0.246	0.109	0.182	0.191	0.284
chmp4a	0.174	0.24	0.428	0.23	0.107	0.188	0.197	0.282
hoxd4	0.174	0.215	0.214	0.119	0.13	0.235	0.24	0.257
acsl5	0.173	0.24	0.353	0.194	0.162	0.169	0.18	0.242
rhob	0.173	0.214	0.248	0.167	0.106	0.167	0.174	0.207
epn2	0.173	0.213	0.34	0.229	0.103	0.192	0.198	0.258
dgcr8	0.173	0.253	0.419	0.172	0.127	0.165	0.177	0.259
tmc3	0.173	0.24	0.363	0.191	0.116	0.192	0.202	0.266
tmc7	0.173	0.24	0.363	0.191	0.116	0.192	0.202	0.266
gprc5a	0.173	0.25	0.323	0.21	0.135	0.167	0.178	0.231
tmc4	0.173	0.24	0.363	0.191	0.116	0.192	0.202	0.266
tmc5	0.173	0.24	0.363	0.191	0.116	0.192	0.202	0.266
obsl1	0.173	0.212	0.288	0.173	0.111	0.171	0.177	0.223
plin2	0.173	0.221	0.353	0.195	0.114	0.158	0.166	0.232
ccdc53	0.173	0.233	0.359	0.226	0.111	0.209	0.217	0.279
c1qtnf3	0.173	0.208	0.216	0.164	0.125	0.149	0.155	0.181

slc5a6	0.173	0.249	0.39	0.254	0.126	0.174	0.185	0.258
zfp36l2	0.173	0.24	0.398	0.234	0.134	0.168	0.179	0.256
cdhr5	0.173	0.215	0.26	0.159	0.102	0.195	0.202	0.236
fam173b	0.173	0.261	0.291	0.175	0.111	0.195	0.207	0.245
fer1l5	0.173	0.23	0.306	0.204	0.106	0.188	0.197	0.244
pnck	0.173	0.201	0.189	0.167	0.143	0.186	0.191	0.206
szt2	0.171	0.236	0.354	0.236	0.146	0.165	0.175	0.239
znf132	0.171	0.256	0.347	0.191	0.125	0.194	0.206	0.263
psma2	0.168	0.246	0.462	0.234	0.131	0.184	0.195	0.289
pcbp2	0.169	0.251	0.472	0.237	0.181	0.146	0.158	0.259
nsun7	0.169	0.231	0.313	0.175	0.161	0.192	0.201	0.25
abi3bp	0.168	0.259	0.405	0.199	0.128	0.168	0.182	0.258
amer2	0.168	0.199	0.215	0.117	0.151	0.243	0.247	0.265
ifi6	0.168	0.263	0.404	0.2	0.116	0.172	0.186	0.261
gng13	0.168	0.199	0.254	0.197	0.102	0.189	0.193	0.229
paip2	0.169	0.241	0.495	0.238	0.145	0.187	0.198	0.302
fam65b	0.169	0.252	0.259	0.135	0.122	0.169	0.181	0.213
prdm14	0.169	0.259	0.261	0.138	0.109	0.168	0.182	0.213
lor	0.169	0.25	0.297	0.153	0.116	0.19	0.201	0.243
tmem217	0.169	0.21	0.417	0.284	0.206	0.175	0.181	0.267
fastkd5	0.169	0.227	0.329	0.216	0.149	0.164	0.173	0.231
fastkd1	0.169	0.227	0.329	0.216	0.149	0.164	0.173	0.231
folr1	0.169	0.261	0.365	0.236	0.105	0.167	0.18	0.244
fastkd3	0.169	0.227	0.329	0.216	0.149	0.164	0.173	0.231
pde7b	0.168	0.217	0.244	0.2	0.142	0.133	0.141	0.177
trappc8	0.168	0.245	0.475	0.218	0.149	0.179	0.19	0.289
med13	0.167	0.216	0.28	0.158	0.295	0.17	0.178	0.221
myo1c	0.168	0.226	0.367	0.238	0.116	0.182	0.191	0.259
foxp1	0.168	0.267	0.315	0.172	0.139	0.149	0.164	0.213
rrn3	0.167	0.24	0.396	0.174	0.137	0.163	0.174	0.251
krt27	0.167	0.198	0.222	0.205	0.101	0.189	0.194	0.219
spdef	0.167	0.28	0.311	0.165	0.12	0.169	0.185	0.23
upf3a	0.167	0.232	0.426	0.23	0.154	0.174	0.184	0.27
rab11fip2	0.168	0.219	0.346	0.211	0.11	0.181	0.189	0.251
hand2-as1	0.168	0.259	0.365	0.193	0.192	0.191	0.204	0.266
mir661	0.168	0.219	0.25	0.124	0.101	0.16	0.168	0.203
jpx	0.168	0.236	0.325	0.202	0.127	0.155	0.165	0.221
yars	0.168	0.234	0.437	0.236	0.166	0.176	0.186	0.275
naaladl2	0.168	0.219	0.25	0.164	0.131	0.179	0.187	0.219
gpbar1	0.168	0.211	0.237	0.18	0.111	0.146	0.153	0.186
mir3120	0.168	0.23	0.334	0.193	0.167	0.163	0.173	0.232

h3f3ap4	0.168	0.245	0.445	0.236	0.259	0.19	0.201	0.29
mir191	0.169	0.254	0.307	0.158	0.119	0.167	0.179	0.226
olfm4	0.169	0.239	0.254	0.19	0.149	0.162	0.172	0.205
erc1	0.17	0.223	0.337	0.236	0.109	0.201	0.209	0.266
rpl10	0.17	0.242	0.358	0.305	0.102	0.14	0.151	0.218
c1ql1	0.17	0.254	0.366	0.214	0.159	0.177	0.19	0.254
bin3	0.17	0.245	0.386	0.222	0.126	0.185	0.196	0.267
lhx3	0.17	0.22	0.194	0.13	0.132	0.19	0.198	0.212
scg2	0.17	0.236	0.307	0.226	0.113	0.168	0.178	0.227
ak2	0.17	0.24	0.381	0.285	0.154	0.196	0.206	0.275
bai2	0.17	0.243	0.393	0.243	0.117	0.168	0.179	0.254
eif5a2	0.17	0.287	0.428	0.175	0.128	0.176	0.193	0.272
ivl	0.17	0.251	0.326	0.213	0.118	0.139	0.152	0.208
gpr26	0.17	0.205	0.295	0.199	0.12	0.181	0.187	0.235
zc3h18	0.17	0.255	0.417	0.229	0.217	0.161	0.173	0.255
trpm6	0.17	0.214	0.295	0.226	0.118	0.18	0.187	0.234
pdcd7	0.17	0.229	0.312	0.23	0.157	0.146	0.155	0.209
dcps	0.17	0.236	0.429	0.22	0.13	0.164	0.174	0.261
safb	0.17	0.232	0.354	0.18	0.38	0.14	0.15	0.217
ifi35	0.17	0.199	0.318	0.231	0.186	0.133	0.138	0.199
fabp5	0.17	0.256	0.376	0.199	0.145	0.163	0.176	0.244
tmem138	0.169	0.221	0.254	0.125	0.107	0.226	0.233	0.262
eif3m	0.169	0.243	0.392	0.206	0.171	0.18	0.191	0.265
rpl29	0.17	0.244	0.347	0.21	0.119	0.17	0.181	0.241
bach2	0.169	0.251	0.382	0.213	0.205	0.136	0.149	0.222
crnn	0.169	0.274	0.42	0.192	0.153	0.156	0.171	0.251
elavl4	0.169	0.265	0.385	0.201	0.117	0.179	0.193	0.261
hint1p1	0.169	0.226	0.296	0.142	0.11	0.192	0.201	0.245
c2orf40	0.17	0.302	0.386	0.17	0.107	0.184	0.203	0.266
mrps27	0.17	0.251	0.466	0.237	0.164	0.177	0.189	0.285
fam134c	0.17	0.211	0.251	0.138	0.186	0.168	0.175	0.21
snord12c	0.17	0.244	0.412	0.212	0.179	0.197	0.208	0.286
phf5a	0.17	0.225	0.386	0.209	0.132	0.186	0.194	0.268
hhex	0.17	0.202	0.18	0.106	0.127	0.194	0.199	0.211
mtx3	0.17	0.235	0.302	0.167	0.125	0.212	0.221	0.264
mob4	0.17	0.236	0.359	0.256	0.106	0.182	0.192	0.256
oxr1	0.14	0.182	0.278	0.136	0.101	0.14	0.147	0.196
s100a4	0.14	0.218	0.287	0.172	0.111	0.158	0.169	0.215
rps15	0.115	0.204	0.385	0.162	0.144	0.123	0.135	0.217
fitm1	0.115	0.163	0.304	0.171	0.126	0.115	0.122	0.185
tceb3c	0.115	0.193	0.329	0.172	0.185	0.123	0.135	0.2

trappc6b	0.115	0.192	0.379	0.189	0.148	0.137	0.148	0.228
bola3	0.115	0.186	0.379	0.234	0.114	0.127	0.137	0.219
c12orf75	0.115	0.187	0.295	0.146	0.129	0.11	0.12	0.177
cox6c	0.115	0.19	0.343	0.215	0.179	0.121	0.132	0.203
acsbg2	0.115	0.132	0.155	0.131	0.126	0.111	0.114	0.135
ano7	0.115	0.185	0.327	0.199	0.164	0.123	0.133	0.199
rgpd1	0.116	0.2	0.332	0.124	0.168	0.122	0.134	0.2
rgpd4	0.116	0.2	0.332	0.124	0.168	0.122	0.134	0.2
rgpd3	0.116	0.2	0.332	0.124	0.168	0.122	0.134	0.2
map7	0.115	0.193	0.288	0.168	0.166	0.112	0.124	0.178
clns1ap1	0.115	0.144	0.18	0.121	0.107	0.13	0.134	0.159
gca	0.115	0.164	0.29	0.151	0.105	0.1	0.108	0.167
nudt21	0.115	0.182	0.427	0.205	0.173	0.127	0.136	0.234
prdm8	0.115	0.19	0.363	0.152	0.121	0.113	0.124	0.202
nynrin	0.115	0.189	0.332	0.166	0.135	0.135	0.146	0.212
dmxl2	0.114	0.159	0.252	0.138	0.108	0.116	0.123	0.17
rlf	0.114	0.151	0.247	0.222	0.104	0.104	0.109	0.157
pan3	0.114	0.163	0.39	0.175	0.109	0.12	0.127	0.216
rab39b	0.114	0.146	0.204	0.125	0.105	0.115	0.12	0.154
srsf12	0.114	0.185	0.459	0.179	0.214	0.131	0.141	0.247
pex11a	0.114	0.173	0.31	0.207	0.12	0.114	0.123	0.186
timm10	0.114	0.209	0.413	0.19	0.121	0.127	0.141	0.23
znf689	0.114	0.164	0.23	0.18	0.137	0.103	0.11	0.151
nsd1	0.115	0.159	0.266	0.124	0.319	0.152	0.158	0.205
ssx4b	0.115	0.217	0.297	0.171	0.187	0.115	0.13	0.183
aspscr1	0.115	0.183	0.286	0.164	0.11	0.128	0.138	0.191
lpin3	0.115	0.151	0.246	0.122	0.143	0.122	0.127	0.173
mapre1	0.115	0.166	0.248	0.111	0.148	0.182	0.189	0.227
ptmap4	0.115	0.215	0.404	0.205	0.163	0.116	0.13	0.217
potea	0.115	0.174	0.265	0.144	0.189	0.113	0.121	0.171
rfk	0.115	0.159	0.254	0.118	0.11	0.112	0.118	0.166
rgpd2	0.116	0.2	0.332	0.124	0.168	0.122	0.134	0.2
papola	0.116	0.188	0.432	0.216	0.182	0.115	0.125	0.224
b3gnt2	0.116	0.2	0.23	0.181	0.133	0.126	0.138	0.172
u2af1l4	0.116	0.198	0.352	0.205	0.206	0.118	0.13	0.202
arf4p1	0.116	0.195	0.317	0.245	0.111	0.134	0.145	0.206
rpl28	0.116	0.188	0.34	0.158	0.111	0.124	0.134	0.203
nanos3	0.116	0.201	0.163	0.115	0.133	0.102	0.115	0.129
sec14l2	0.116	0.159	0.23	0.126	0.125	0.135	0.141	0.179
kif5b	0.116	0.17	0.325	0.182	0.137	0.139	0.147	0.213
znf123p	0.116	0.196	0.308	0.179	0.183	0.13	0.141	0.199

top1mt	0.116	0.193	0.386	0.176	0.115	0.118	0.129	0.213
ubr4	0.117	0.214	0.337	0.169	0.106	0.124	0.138	0.203
spef2	0.117	0.182	0.228	0.147	0.206	0.133	0.143	0.177
prmt1	0.117	0.185	0.376	0.194	0.239	0.115	0.125	0.207
h3f3ap5	0.117	0.195	0.405	0.19	0.239	0.139	0.15	0.237
h3f3ap6	0.117	0.195	0.405	0.19	0.239	0.139	0.15	0.237
cwc22	0.116	0.2	0.387	0.155	0.122	0.12	0.132	0.215
h3f3ap3	0.117	0.195	0.405	0.19	0.239	0.139	0.15	0.237
me2	0.116	0.141	0.211	0.156	0.136	0.122	0.126	0.162
polr1c	0.116	0.197	0.383	0.185	0.12	0.131	0.143	0.224
cabin1	0.116	0.166	0.303	0.175	0.119	0.11	0.118	0.18
rps24	0.116	0.206	0.353	0.207	0.111	0.14	0.153	0.222
pnpla5	0.116	0.155	0.298	0.125	0.122	0.129	0.135	0.195
kif1b	0.116	0.18	0.271	0.156	0.105	0.119	0.128	0.178
c12orf39	0.116	0.165	0.25	0.155	0.103	0.133	0.14	0.184
agpat2	0.116	0.146	0.215	0.152	0.111	0.118	0.123	0.16
srrm1	0.116	0.199	0.449	0.224	0.175	0.123	0.135	0.237
adam5	0.116	0.17	0.316	0.137	0.103	0.125	0.133	0.197
lpgat1	0.116	0.149	0.208	0.134	0.132	0.13	0.135	0.169
sart1	0.116	0.2	0.378	0.149	0.105	0.119	0.131	0.211
nat8	0.116	0.177	0.305	0.14	0.13	0.141	0.15	0.208
zscan25	0.116	0.168	0.336	0.169	0.11	0.119	0.127	0.199
tmed11p	0.116	0.203	0.346	0.211	0.103	0.134	0.147	0.215
parp6	0.116	0.203	0.383	0.168	0.164	0.125	0.137	0.218
folh1	0.116	0.189	0.279	0.15	0.152	0.105	0.116	0.168
pura	0.116	0.193	0.393	0.162	0.177	0.105	0.116	0.204
sec31a	0.114	0.176	0.343	0.218	0.11	0.142	0.15	0.221
zcchc7	0.114	0.18	0.274	0.129	0.105	0.131	0.14	0.189
bet1	0.113	0.171	0.31	0.188	0.108	0.116	0.125	0.188
padi2	0.113	0.199	0.344	0.188	0.137	0.119	0.131	0.201
bckdha	0.113	0.171	0.342	0.202	0.115	0.133	0.141	0.213
ubqln1	0.113	0.135	0.288	0.102	0.122	0.126	0.129	0.19
cenpn	0.112	0.199	0.359	0.162	0.205	0.112	0.125	0.2
lcn1	0.112	0.184	0.355	0.19	0.107	0.14	0.15	0.223
echs1	0.112	0.164	0.303	0.204	0.149	0.113	0.121	0.183
ssu72	0.113	0.179	0.348	0.166	0.171	0.126	0.136	0.209
c9orf96	0.113	0.149	0.242	0.103	0.122	0.136	0.141	0.184
mtf2	0.113	0.181	0.283	0.107	0.126	0.12	0.13	0.183
l3mbtl1	0.113	0.199	0.385	0.121	0.153	0.132	0.144	0.226
slc35b1	0.113	0.179	0.347	0.162	0.112	0.119	0.129	0.202
parp10	0.113	0.15	0.301	0.128	0.146	0.136	0.142	0.203

prmt5	0.113	0.197	0.35	0.183	0.165	0.118	0.13	0.203
samd9l	0.113	0.182	0.277	0.135	0.113	0.109	0.119	0.171
brd9	0.113	0.173	0.276	0.123	0.105	0.122	0.131	0.183
srsf10	0.112	0.162	0.313	0.168	0.104	0.115	0.122	0.188
rn7sk	0.112	0.189	0.433	0.186	0.207	0.119	0.13	0.229
ezh2	0.112	0.194	0.291	0.12	0.135	0.113	0.125	0.18
luc7l	0.112	0.151	0.229	0.126	0.181	0.132	0.137	0.177
polr2j	0.112	0.201	0.408	0.169	0.119	0.135	0.148	0.236
nfia	0.112	0.17	0.214	0.123	0.108	0.117	0.126	0.159
hdhd1	0.112	0.185	0.326	0.203	0.125	0.13	0.14	0.205
tnfaip1	0.112	0.156	0.246	0.126	0.127	0.122	0.128	0.173
ccnc	0.112	0.232	0.358	0.165	0.138	0.112	0.129	0.199
thap10	0.112	0.185	0.314	0.128	0.156	0.112	0.122	0.185
lrrc49	0.112	0.185	0.314	0.128	0.156	0.112	0.122	0.185
rp24	0.112	0.173	0.264	0.167	0.128	0.133	0.142	0.189
glcci1	0.112	0.162	0.199	0.143	0.372	0.101	0.109	0.14
psmb5	0.112	0.183	0.3	0.141	0.125	0.107	0.118	0.177
lyzl4	0.112	0.185	0.283	0.189	0.114	0.101	0.112	0.166
wdr74	0.112	0.211	0.389	0.151	0.137	0.137	0.151	0.231
glud1	0.112	0.175	0.344	0.162	0.155	0.125	0.134	0.207
rprm	0.112	0.197	0.323	0.112	0.162	0.103	0.115	0.18
ppp1r2p1	0.113	0.219	0.385	0.201	0.161	0.14	0.154	0.232
asz1	0.113	0.202	0.354	0.195	0.147	0.128	0.14	0.212
trip11	0.114	0.17	0.265	0.181	0.127	0.133	0.141	0.189
lonp1	0.114	0.184	0.352	0.19	0.139	0.125	0.135	0.209
poteg	0.114	0.19	0.356	0.157	0.154	0.115	0.126	0.202
trim24	0.114	0.15	0.299	0.13	0.526	0.112	0.117	0.181
dmrt3	0.114	0.178	0.233	0.117	0.157	0.151	0.16	0.195
wdr75	0.113	0.198	0.351	0.149	0.143	0.122	0.134	0.206
nip7	0.114	0.182	0.406	0.197	0.107	0.115	0.125	0.216
mfsd11	0.114	0.184	0.378	0.216	0.145	0.131	0.141	0.222
polr2g	0.114	0.183	0.29	0.14	0.107	0.141	0.151	0.204
tcirg1	0.114	0.178	0.26	0.141	0.113	0.104	0.114	0.162
clip1	0.114	0.19	0.305	0.167	0.108	0.139	0.149	0.206
gtf2ird2b	0.114	0.172	0.288	0.172	0.164	0.133	0.141	0.196
ube2l6	0.114	0.162	0.329	0.132	0.171	0.148	0.155	0.222
or8j2	0.114	0.171	0.267	0.121	0.128	0.134	0.142	0.19
h2afz	0.114	0.209	0.417	0.132	0.177	0.113	0.127	0.219
tmem30c	0.114	0.178	0.325	0.144	0.104	0.146	0.155	0.219
adprh	0.113	0.202	0.395	0.2	0.158	0.119	0.132	0.217
prmt3	0.113	0.177	0.404	0.194	0.176	0.111	0.12	0.213

exosc8	0.113	0.181	0.323	0.144	0.113	0.128	0.138	0.203
mis18bp1	0.113	0.192	0.299	0.145	0.113	0.123	0.134	0.19
phf1	0.113	0.188	0.286	0.103	0.116	0.121	0.132	0.185
donson	0.113	0.174	0.256	0.179	0.122	0.118	0.127	0.173
fmn2	0.113	0.162	0.241	0.118	0.125	0.113	0.121	0.164
uty	0.113	0.195	0.267	0.134	0.213	0.108	0.12	0.167
hprt1	0.113	0.175	0.235	0.117	0.108	0.117	0.126	0.165
clps	0.113	0.181	0.304	0.185	0.103	0.117	0.126	0.186
ctns	0.113	0.18	0.329	0.172	0.123	0.124	0.133	0.201
scarna2	0.113	0.218	0.409	0.175	0.169	0.129	0.144	0.23
igfn1	0.113	0.158	0.332	0.173	0.137	0.125	0.132	0.203
angptl4	0.113	0.148	0.179	0.103	0.124	0.113	0.119	0.145
hmgn1	0.113	0.192	0.397	0.202	0.191	0.1	0.112	0.201
snapc5	0.113	0.176	0.358	0.181	0.157	0.116	0.125	0.203
atg10	0.113	0.169	0.327	0.112	0.104	0.107	0.115	0.185
anp32b	0.117	0.208	0.296	0.178	0.132	0.102	0.116	0.171
pip	0.117	0.188	0.235	0.167	0.123	0.12	0.13	0.168
pygm	0.12	0.176	0.309	0.17	0.151	0.139	0.147	0.207
rybp	0.12	0.181	0.301	0.127	0.158	0.112	0.121	0.18
loc652993	0.12	0.181	0.315	0.147	0.125	0.132	0.141	0.203
arid3a	0.119	0.218	0.349	0.157	0.172	0.106	0.12	0.19
rusc2	0.119	0.169	0.303	0.201	0.11	0.112	0.119	0.181
klk11	0.119	0.21	0.302	0.146	0.162	0.111	0.124	0.181
alppl2	0.119	0.213	0.306	0.196	0.113	0.119	0.132	0.189
ass1	0.12	0.185	0.33	0.144	0.157	0.116	0.126	0.194
gtpbp6	0.12	0.18	0.269	0.142	0.121	0.124	0.132	0.181
cox7a1	0.12	0.191	0.354	0.181	0.172	0.118	0.128	0.203
paaf1	0.12	0.18	0.371	0.145	0.139	0.118	0.127	0.208
bves	0.12	0.188	0.241	0.147	0.113	0.134	0.143	0.181
farp1	0.12	0.172	0.224	0.16	0.145	0.129	0.137	0.172
ube2c	0.12	0.226	0.364	0.134	0.118	0.138	0.153	0.223
mir18b	0.12	0.199	0.271	0.102	0.143	0.11	0.121	0.169
paqr5	0.12	0.16	0.187	0.144	0.126	0.103	0.11	0.137
st7-ot3	0.119	0.194	0.318	0.176	0.141	0.134	0.145	0.205
prelp	0.119	0.175	0.278	0.146	0.118	0.135	0.143	0.194
csn3	0.119	0.187	0.322	0.22	0.101	0.113	0.123	0.188
gemin5	0.119	0.188	0.385	0.222	0.103	0.121	0.131	0.215
mir605	0.119	0.15	0.198	0.108	0.113	0.104	0.109	0.142
hdgfl1	0.119	0.175	0.29	0.126	0.14	0.127	0.136	0.191
orc6	0.119	0.22	0.381	0.161	0.111	0.126	0.14	0.218
phgdh	0.119	0.197	0.3	0.141	0.127	0.117	0.128	0.185

khnyn	0.119	0.194	0.358	0.178	0.124	0.135	0.146	0.219
stox1	0.119	0.171	0.215	0.152	0.114	0.112	0.12	0.154
heih	0.119	0.199	0.289	0.127	0.122	0.112	0.123	0.177
wash6p	0.119	0.187	0.297	0.181	0.167	0.115	0.125	0.183
med7	0.119	0.166	0.25	0.143	0.244	0.14	0.146	0.189
sox6	0.119	0.161	0.159	0.11	0.186	0.108	0.115	0.133
spry4-it1	0.119	0.196	0.313	0.125	0.101	0.138	0.149	0.208
gaec1	0.119	0.188	0.219	0.13	0.108	0.155	0.164	0.193
mir5047	0.119	0.202	0.392	0.153	0.149	0.126	0.138	0.222
trim3	0.119	0.152	0.28	0.129	0.132	0.161	0.165	0.217
or1g1	0.12	0.166	0.236	0.153	0.161	0.121	0.128	0.168
trnt1	0.12	0.188	0.378	0.181	0.129	0.123	0.133	0.214
ntm	0.121	0.205	0.351	0.167	0.138	0.142	0.154	0.223
dhx15	0.121	0.207	0.493	0.229	0.15	0.128	0.141	0.255
zfp82	0.121	0.208	0.314	0.14	0.185	0.123	0.135	0.194
atad2b	0.121	0.225	0.42	0.177	0.242	0.108	0.123	0.214
gage1	0.121	0.233	0.315	0.124	0.14	0.102	0.119	0.177
nova1	0.121	0.185	0.295	0.167	0.133	0.139	0.148	0.202
slc7a6	0.121	0.182	0.312	0.175	0.124	0.13	0.139	0.2
kif5a	0.121	0.19	0.348	0.198	0.16	0.143	0.152	0.222
ube3a	0.121	0.181	0.377	0.135	0.125	0.137	0.146	0.227
ldb1	0.121	0.179	0.195	0.119	0.114	0.133	0.142	0.166
mis18a	0.121	0.195	0.292	0.152	0.133	0.123	0.134	0.187
aass	0.122	0.178	0.349	0.145	0.145	0.137	0.145	0.218
bspry	0.121	0.174	0.268	0.211	0.118	0.118	0.126	0.176
dnah6	0.121	0.189	0.351	0.144	0.127	0.146	0.155	0.226
snrpb2	0.121	0.217	0.416	0.252	0.162	0.123	0.136	0.226
dnah10	0.121	0.189	0.351	0.144	0.127	0.146	0.155	0.226
dus4l	0.121	0.162	0.241	0.122	0.13	0.119	0.125	0.168
hibch	0.121	0.191	0.392	0.193	0.141	0.121	0.131	0.217
mir135a1	0.12	0.141	0.136	0.127	0.111	0.166	0.168	0.177
tmem165	0.121	0.176	0.29	0.165	0.117	0.123	0.132	0.187
micu1	0.121	0.144	0.174	0.12	0.101	0.124	0.128	0.151
zbtb4	0.12	0.216	0.334	0.156	0.184	0.125	0.139	0.203
fam172a	0.12	0.217	0.314	0.192	0.233	0.11	0.124	0.183
apcdd1	0.12	0.177	0.358	0.122	0.12	0.131	0.14	0.216
bok-as1	0.12	0.193	0.284	0.148	0.108	0.117	0.128	0.18
mir652	0.121	0.172	0.222	0.101	0.102	0.123	0.131	0.166
mir571	0.121	0.172	0.222	0.101	0.102	0.123	0.131	0.166
setdb2	0.121	0.197	0.312	0.16	0.219	0.131	0.142	0.201
cmd1h	0.121	0.163	0.22	0.124	0.133	0.131	0.137	0.172

golt1b	0.121	0.198	0.267	0.169	0.103	0.118	0.13	0.176
ckmt1b	0.121	0.188	0.377	0.206	0.12	0.136	0.146	0.226
wdr43	0.121	0.205	0.419	0.138	0.154	0.136	0.148	0.238
scmh1	0.121	0.178	0.307	0.146	0.202	0.132	0.141	0.201
unc79	0.119	0.156	0.239	0.119	0.144	0.12	0.125	0.168
usp17l9p	0.119	0.181	0.323	0.161	0.114	0.137	0.146	0.21
elf2p2	0.117	0.177	0.188	0.173	0.119	0.131	0.139	0.162
zp1	0.117	0.216	0.323	0.206	0.116	0.131	0.145	0.204
phtf1	0.117	0.18	0.263	0.183	0.144	0.133	0.142	0.188
elf2p4	0.117	0.177	0.188	0.173	0.119	0.131	0.139	0.162
elf2p1	0.117	0.177	0.188	0.173	0.119	0.131	0.139	0.162
rbm14-rbm4	0.117	0.174	0.255	0.16	0.109	0.105	0.113	0.16
elf2p3	0.117	0.177	0.188	0.173	0.119	0.131	0.139	0.162
tsku	0.117	0.181	0.177	0.114	0.158	0.144	0.153	0.171
rpl17	0.117	0.181	0.315	0.141	0.155	0.13	0.14	0.202
dhx38	0.118	0.19	0.452	0.162	0.151	0.116	0.127	0.232
arid1a	0.118	0.201	0.317	0.125	0.202	0.16	0.171	0.228
ankrd32	0.118	0.181	0.298	0.144	0.114	0.141	0.15	0.206
ftsj3	0.118	0.181	0.422	0.177	0.137	0.119	0.128	0.225
coq7	0.117	0.172	0.352	0.144	0.112	0.124	0.132	0.207
kcnk13	0.117	0.164	0.274	0.195	0.105	0.115	0.122	0.176
sptlc3	0.117	0.156	0.29	0.153	0.111	0.127	0.133	0.191
wdfy3	0.117	0.15	0.269	0.126	0.112	0.117	0.122	0.175
manea	0.117	0.159	0.255	0.224	0.121	0.147	0.153	0.197
dtwd2	0.117	0.218	0.404	0.206	0.167	0.117	0.132	0.218
ptmap3	0.117	0.218	0.404	0.206	0.167	0.117	0.132	0.218
ptmap2	0.117	0.218	0.404	0.206	0.167	0.117	0.132	0.218
sfswap	0.117	0.17	0.316	0.176	0.149	0.126	0.134	0.199
tfap4	0.117	0.197	0.3	0.185	0.122	0.103	0.115	0.173
pex12	0.117	0.185	0.38	0.176	0.143	0.126	0.136	0.218
zbed4	0.117	0.19	0.354	0.156	0.15	0.119	0.13	0.204
vps53	0.117	0.192	0.318	0.179	0.114	0.119	0.13	0.192
znf462	0.117	0.177	0.215	0.108	0.113	0.13	0.138	0.17
krt81	0.117	0.172	0.222	0.157	0.118	0.124	0.132	0.167
cst9lp1	0.117	0.183	0.301	0.121	0.135	0.143	0.153	0.209
wars2	0.117	0.165	0.238	0.146	0.18	0.126	0.133	0.174
hagh	0.117	0.184	0.344	0.192	0.126	0.133	0.143	0.213
rnf125	0.117	0.154	0.248	0.114	0.126	0.129	0.135	0.18
tssk3	0.117	0.155	0.193	0.12	0.148	0.123	0.128	0.157
polr3h	0.117	0.178	0.304	0.147	0.102	0.101	0.111	0.173
h2afy	0.118	0.208	0.377	0.147	0.213	0.123	0.136	0.215

ociad1	0.118	0.218	0.287	0.169	0.113	0.138	0.152	0.2
linc00470	0.118	0.18	0.241	0.137	0.107	0.137	0.146	0.184
fbxo16	0.118	0.204	0.304	0.198	0.122	0.125	0.137	0.193
gnl3	0.118	0.209	0.354	0.13	0.117	0.107	0.12	0.193
ttll1	0.118	0.159	0.259	0.113	0.104	0.124	0.13	0.178
znf407	0.118	0.158	0.221	0.135	0.123	0.122	0.128	0.165
pld6	0.118	0.176	0.326	0.148	0.172	0.147	0.155	0.22
gng5	0.118	0.142	0.186	0.195	0.1	0.111	0.115	0.145
taf4b	0.118	0.18	0.296	0.14	0.166	0.112	0.121	0.179
apod	0.118	0.183	0.262	0.17	0.12	0.117	0.127	0.173
spg7	0.119	0.183	0.323	0.159	0.151	0.137	0.146	0.21
dhx35	0.119	0.194	0.396	0.165	0.102	0.122	0.133	0.22
smad5-as1	0.119	0.187	0.268	0.171	0.137	0.14	0.15	0.196
slc19a2	0.119	0.187	0.317	0.163	0.108	0.109	0.119	0.183
serbp1	0.119	0.174	0.289	0.153	0.144	0.121	0.129	0.185
abcb6	0.118	0.19	0.364	0.182	0.155	0.105	0.116	0.194
linc00575	0.118	0.196	0.297	0.183	0.138	0.129	0.14	0.195
parp15	0.118	0.191	0.38	0.173	0.202	0.134	0.145	0.225
gys2	0.118	0.156	0.232	0.164	0.165	0.118	0.124	0.165
sec22a	0.118	0.169	0.318	0.194	0.136	0.147	0.154	0.218
vcx	0.118	0.209	0.358	0.183	0.144	0.136	0.149	0.22
jund	0.118	0.161	0.275	0.182	0.132	0.101	0.108	0.163
cyp51a1	0.118	0.169	0.279	0.166	0.107	0.123	0.131	0.184
mterf	0.118	0.187	0.337	0.157	0.186	0.114	0.124	0.194
ggnbp1	0.118	0.187	0.367	0.167	0.238	0.138	0.147	0.224
nrf1	0.118	0.173	0.347	0.133	0.251	0.122	0.131	0.205
ofd1p18y	0.118	0.186	0.314	0.213	0.102	0.144	0.153	0.213
ofd1p17	0.118	0.186	0.314	0.213	0.102	0.144	0.153	0.213
mybbp1a	0.118	0.194	0.352	0.166	0.292	0.124	0.135	0.208
pum1	0.118	0.224	0.373	0.159	0.1	0.124	0.139	0.214
acadl	0.118	0.17	0.277	0.157	0.171	0.127	0.134	0.187
fxn	0.118	0.193	0.42	0.167	0.141	0.121	0.132	0.226
cttnbp2	0.118	0.181	0.282	0.162	0.112	0.127	0.136	0.188
dctn1	0.118	0.19	0.32	0.172	0.11	0.134	0.145	0.207
kctd3	0.112	0.149	0.256	0.158	0.114	0.104	0.11	0.161
chst6	0.112	0.169	0.252	0.131	0.101	0.115	0.124	0.169
mapre2	0.104	0.165	0.256	0.128	0.158	0.169	0.177	0.219
kdm6a	0.104	0.194	0.304	0.121	0.188	0.121	0.134	0.191
whsc1	0.104	0.156	0.214	0.103	0.113	0.106	0.113	0.15
ift122	0.104	0.14	0.173	0.104	0.111	0.124	0.129	0.153
armcx3	0.104	0.153	0.199	0.129	0.193	0.118	0.126	0.156

cst8	0.104	0.171	0.273	0.141	0.156	0.126	0.135	0.186
znf592	0.104	0.147	0.222	0.115	0.106	0.118	0.125	0.163
slc6a8	0.104	0.169	0.357	0.158	0.16	0.118	0.127	0.205
daz3	0.104	0.183	0.277	0.145	0.251	0.105	0.116	0.169
u2af2	0.104	0.183	0.415	0.188	0.167	0.112	0.123	0.218
asl	0.105	0.179	0.335	0.157	0.156	0.122	0.133	0.202
rfx7	0.105	0.188	0.29	0.131	0.104	0.122	0.134	0.188
cpo	0.104	0.179	0.336	0.165	0.101	0.129	0.139	0.208
tubb1	0.104	0.17	0.26	0.137	0.104	0.118	0.127	0.175
rnu1-4	0.104	0.182	0.348	0.168	0.116	0.108	0.119	0.193
seh1l	0.104	0.179	0.385	0.19	0.137	0.12	0.131	0.216
phc3	0.104	0.168	0.243	0.128	0.189	0.103	0.113	0.157
snora67	0.104	0.169	0.369	0.149	0.143	0.112	0.121	0.204
polr2j2	0.103	0.195	0.427	0.16	0.178	0.131	0.144	0.239
polr2j3	0.103	0.195	0.427	0.16	0.178	0.131	0.144	0.239
tex10	0.103	0.152	0.348	0.128	0.144	0.124	0.131	0.208
orc5	0.103	0.198	0.387	0.191	0.128	0.129	0.142	0.224
smc6	0.103	0.168	0.423	0.129	0.114	0.142	0.151	0.247
bp4	0.103	0.137	0.221	0.113	0.136	0.121	0.126	0.166
megf10	0.103	0.156	0.281	0.104	0.111	0.104	0.111	0.169
foxd4l1	0.103	0.158	0.256	0.137	0.228	0.121	0.129	0.177
bhmt2	0.103	0.143	0.233	0.116	0.102	0.1	0.107	0.151
tex15	0.104	0.182	0.205	0.109	0.188	0.112	0.123	0.152
mtfr2	0.104	0.152	0.258	0.157	0.121	0.102	0.109	0.16
snora68	0.104	0.169	0.369	0.149	0.143	0.112	0.121	0.204
morf4	0.104	0.219	0.397	0.136	0.106	0.109	0.126	0.21
slc27a6	0.104	0.13	0.204	0.105	0.103	0.117	0.121	0.157
tceal7	0.103	0.175	0.287	0.133	0.117	0.107	0.118	0.174
olfml1	0.104	0.168	0.259	0.131	0.125	0.106	0.115	0.164
patl2	0.105	0.167	0.314	0.155	0.106	0.118	0.127	0.192
spata25	0.105	0.184	0.242	0.165	0.186	0.113	0.125	0.165
bcorl1	0.106	0.184	0.326	0.136	0.234	0.102	0.114	0.181
ndufs3	0.106	0.194	0.401	0.179	0.118	0.121	0.133	0.221
cox4i2	0.106	0.175	0.316	0.177	0.114	0.107	0.117	0.182
dld	0.105	0.158	0.303	0.172	0.144	0.119	0.127	0.189
pc	0.105	0.172	0.345	0.149	0.177	0.108	0.118	0.193
aaas	0.105	0.162	0.254	0.131	0.133	0.108	0.116	0.164
thcytx	0.105	0.161	0.215	0.155	0.15	0.125	0.133	0.168
lnx2	0.106	0.149	0.206	0.116	0.112	0.107	0.114	0.148
txnl4b	0.106	0.17	0.35	0.171	0.109	0.112	0.122	0.198
zhx1	0.106	0.18	0.33	0.184	0.163	0.114	0.124	0.193

kif12	0.106	0.177	0.262	0.114	0.111	0.12	0.13	0.178
commd3	0.106	0.172	0.296	0.111	0.111	0.137	0.146	0.203
eya2	0.106	0.153	0.193	0.115	0.15	0.138	0.144	0.171
alpp	0.106	0.192	0.299	0.174	0.101	0.102	0.115	0.173
rab43p1	0.106	0.177	0.342	0.191	0.137	0.123	0.133	0.205
slc25a46	0.106	0.15	0.208	0.109	0.168	0.17	0.176	0.205
pate2	0.105	0.181	0.238	0.134	0.127	0.114	0.125	0.165
cpt1a	0.105	0.141	0.274	0.136	0.196	0.112	0.117	0.174
asxl3	0.105	0.163	0.295	0.121	0.275	0.12	0.129	0.188
suz12	0.105	0.18	0.301	0.11	0.162	0.11	0.121	0.18
taf1c	0.105	0.187	0.434	0.142	0.231	0.1	0.112	0.213
ndufv1	0.105	0.185	0.395	0.149	0.126	0.114	0.125	0.213
muc3b	0.105	0.163	0.23	0.178	0.1	0.104	0.112	0.153
ebna1bp2	0.105	0.204	0.417	0.183	0.136	0.129	0.143	0.233
syt3	0.105	0.146	0.211	0.152	0.108	0.104	0.11	0.147
polg2	0.105	0.177	0.35	0.186	0.139	0.107	0.117	0.193
krt25	0.105	0.151	0.261	0.107	0.183	0.149	0.155	0.202
eefsec	0.105	0.167	0.333	0.149	0.126	0.109	0.118	0.189
shox	0.105	0.141	0.171	0.111	0.165	0.116	0.121	0.145
sf3a2	0.105	0.174	0.323	0.225	0.218	0.118	0.128	0.195
polr2e	0.105	0.181	0.399	0.193	0.198	0.128	0.138	0.227
pank3	0.105	0.159	0.328	0.159	0.155	0.111	0.118	0.189
ttbk2	0.105	0.161	0.257	0.158	0.127	0.114	0.122	0.17
bp3	0.103	0.137	0.221	0.113	0.136	0.121	0.126	0.166
bp2	0.103	0.137	0.221	0.113	0.136	0.121	0.126	0.166
spanxd	0.101	0.153	0.233	0.126	0.252	0.108	0.116	0.158
ezh1	0.101	0.171	0.277	0.111	0.134	0.114	0.124	0.177
kcnd3	0.101	0.134	0.205	0.136	0.102	0.101	0.106	0.143
spanxe	0.101	0.153	0.233	0.126	0.252	0.108	0.116	0.158
nme9	0.101	0.188	0.406	0.188	0.131	0.112	0.124	0.215
cep120	0.101	0.175	0.257	0.107	0.117	0.12	0.13	0.176
gfpt1	0.101	0.134	0.23	0.13	0.105	0.105	0.11	0.155
slc25a22	0.101	0.171	0.337	0.164	0.142	0.109	0.119	0.191
xrra1	0.101	0.184	0.335	0.161	0.101	0.113	0.125	0.194
anapc7	0.101	0.204	0.358	0.119	0.108	0.108	0.122	0.196
prpsap2	0.101	0.168	0.284	0.155	0.135	0.102	0.112	0.169
hira	0.101	0.186	0.348	0.13	0.188	0.128	0.14	0.212
mr1p1	0.101	0.19	0.297	0.173	0.144	0.105	0.117	0.175
dpf3	0.101	0.165	0.371	0.139	0.166	0.108	0.117	0.201
raver1	0.101	0.171	0.345	0.187	0.14	0.111	0.121	0.196
nup37	0.101	0.181	0.4	0.177	0.117	0.116	0.127	0.217

rps12	0.101	0.136	0.168	0.117	0.122	0.126	0.131	0.154
ddx6	0.101	0.163	0.363	0.131	0.135	0.11	0.119	0.2
rbmy1j	0.1	0.176	0.318	0.159	0.195	0.112	0.123	0.188
rbmy1d	0.1	0.176	0.318	0.159	0.195	0.112	0.123	0.188
rbmy1b	0.1	0.176	0.318	0.159	0.195	0.112	0.123	0.188
rbmy1f	0.1	0.176	0.318	0.159	0.195	0.112	0.123	0.188
rbmy1e	0.1	0.176	0.318	0.159	0.195	0.112	0.123	0.188
nlk	0.453	0.467	0.415	0.374	0.152	0.438	0.443	0.472
lars2	0.1	0.162	0.351	0.162	0.136	0.105	0.114	0.192
kdm2a	0.1	0.159	0.293	0.107	0.165	0.111	0.119	0.179
pqbp1	0.101	0.138	0.259	0.113	0.169	0.102	0.108	0.161
cpsf6	0.101	0.163	0.392	0.187	0.116	0.111	0.12	0.21
qsox1	0.101	0.191	0.396	0.17	0.104	0.111	0.123	0.211
ppp1r2p3	0.101	0.139	0.227	0.128	0.121	0.123	0.128	0.169
prd	0.101	0.124	0.165	0.128	0.104	0.109	0.113	0.138
med20	0.101	0.129	0.244	0.117	0.329	0.104	0.108	0.158
aoc2	0.101	0.148	0.21	0.136	0.141	0.108	0.115	0.151
ppp1r11	0.101	0.175	0.275	0.17	0.119	0.11	0.121	0.173
rab9bp1	0.101	0.186	0.415	0.242	0.131	0.116	0.128	0.221
zp3	0.101	0.193	0.312	0.187	0.108	0.108	0.121	0.182
pex3	0.102	0.15	0.313	0.155	0.103	0.112	0.119	0.186
cib4	0.102	0.149	0.29	0.164	0.114	0.104	0.111	0.173
znf638	0.102	0.165	0.334	0.173	0.127	0.109	0.118	0.19
cmc4	0.102	0.174	0.298	0.162	0.108	0.11	0.12	0.18
cntnap2	0.102	0.15	0.221	0.127	0.101	0.131	0.138	0.174
znf468	0.102	0.17	0.366	0.186	0.187	0.124	0.133	0.213
blzf1	0.102	0.159	0.337	0.143	0.102	0.121	0.129	0.202
slc25a13	0.103	0.163	0.274	0.14	0.112	0.107	0.116	0.17
myo15b	0.103	0.154	0.321	0.142	0.104	0.118	0.126	0.195
atod3	0.103	0.18	0.272	0.173	0.121	0.121	0.132	0.181
spint3	0.103	0.16	0.276	0.138	0.136	0.118	0.126	0.18
slc38a4	0.103	0.152	0.232	0.125	0.127	0.123	0.13	0.171
rnf20	0.103	0.195	0.419	0.139	0.149	0.115	0.128	0.222
csn1s2bp	0.103	0.176	0.31	0.198	0.132	0.105	0.115	0.179
wwc1	0.103	0.133	0.159	0.122	0.102	0.106	0.111	0.133
spesp1	0.103	0.168	0.221	0.163	0.121	0.117	0.126	0.162
prmt8	0.102	0.165	0.316	0.118	0.174	0.129	0.138	0.202
lgsn	0.102	0.156	0.231	0.124	0.108	0.114	0.122	0.162
rbck1	0.102	0.105	0.195	0.114	0.124	0.13	0.131	0.166
fopnl	0.102	0.146	0.251	0.147	0.109	0.122	0.129	0.176
eml1	0.102	0.161	0.253	0.14	0.106	0.131	0.139	0.184

rpl9	0.102	0.166	0.365	0.166	0.116	0.107	0.116	0.198
tex22	0.102	0.181	0.357	0.195	0.193	0.104	0.116	0.193
pabpn1	0.101	0.173	0.394	0.148	0.129	0.12	0.13	0.219
dph5	0.102	0.154	0.203	0.114	0.166	0.107	0.115	0.148
dhrs4l2	0.102	0.189	0.409	0.18	0.132	0.118	0.13	0.221
rps28	0.102	0.157	0.201	0.13	0.264	0.117	0.125	0.156
mocs2	0.102	0.184	0.425	0.188	0.14	0.122	0.133	0.23
wfdc2	0.102	0.192	0.231	0.101	0.101	0.116	0.128	0.164
slc43a1	0.102	0.186	0.298	0.155	0.131	0.114	0.126	0.184
tcte3	0.102	0.162	0.267	0.142	0.145	0.13	0.138	0.188
murc	0.102	0.135	0.304	0.179	0.103	0.121	0.126	0.191
scamp4	0.102	0.149	0.254	0.169	0.139	0.129	0.135	0.183
parp11	0.106	0.19	0.373	0.147	0.149	0.125	0.137	0.217
c9orf3	0.106	0.161	0.298	0.104	0.114	0.111	0.119	0.18
kctd7	0.11	0.151	0.314	0.117	0.106	0.134	0.14	0.206
akr1b10	0.11	0.173	0.339	0.148	0.114	0.104	0.113	0.187
eml3	0.11	0.196	0.314	0.156	0.146	0.141	0.152	0.211
mroh2b	0.11	0.18	0.296	0.139	0.139	0.135	0.145	0.201
yipf1	0.11	0.143	0.21	0.119	0.133	0.127	0.132	0.167
slc25a2	0.109	0.144	0.171	0.124	0.111	0.125	0.13	0.153
rbm6	0.109	0.184	0.264	0.177	0.119	0.111	0.122	0.17
lars	0.11	0.181	0.386	0.161	0.115	0.108	0.118	0.205
dbi	0.11	0.168	0.288	0.151	0.152	0.116	0.125	0.181
wdr61	0.11	0.169	0.269	0.151	0.163	0.105	0.113	0.165
nap1l2	0.11	0.192	0.318	0.134	0.157	0.128	0.14	0.201
rnf113a	0.11	0.189	0.343	0.175	0.168	0.119	0.131	0.201
h3f3a	0.11	0.195	0.402	0.171	0.246	0.112	0.124	0.213
acy1	0.11	0.196	0.392	0.199	0.121	0.108	0.12	0.207
elovl1	0.11	0.182	0.343	0.152	0.118	0.112	0.122	0.195
nova2	0.11	0.181	0.319	0.162	0.119	0.134	0.144	0.207
tubb	0.109	0.19	0.386	0.191	0.1	0.128	0.14	0.223
cep350	0.109	0.162	0.28	0.13	0.237	0.117	0.125	0.179
tmem181	0.109	0.167	0.283	0.133	0.124	0.11	0.118	0.174
c1orf52	0.109	0.171	0.256	0.134	0.136	0.105	0.114	0.162
znf847p	0.109	0.171	0.256	0.134	0.136	0.105	0.114	0.162
ubp1	0.109	0.162	0.218	0.141	0.115	0.102	0.11	0.148
spag11b	0.109	0.18	0.29	0.145	0.224	0.129	0.139	0.193
atp1b4	0.109	0.158	0.226	0.143	0.138	0.125	0.132	0.17
atxn2l	0.109	0.145	0.215	0.167	0.106	0.103	0.108	0.147
gm140	0.109	0.171	0.256	0.134	0.136	0.105	0.114	0.162
pdzrn4	0.109	0.135	0.205	0.119	0.1	0.113	0.117	0.153

dmkn	0.109	0.148	0.187	0.141	0.126	0.114	0.12	0.148
krt74	0.109	0.168	0.216	0.104	0.129	0.134	0.142	0.175
rnf103	0.109	0.151	0.315	0.106	0.131	0.146	0.152	0.217
luzp4	0.109	0.212	0.323	0.145	0.142	0.112	0.127	0.189
meaf6	0.109	0.185	0.416	0.133	0.21	0.101	0.112	0.208
fam120b	0.109	0.158	0.307	0.127	0.274	0.131	0.138	0.201
scand2p	0.109	0.17	0.335	0.185	0.102	0.134	0.142	0.212
celf4	0.11	0.16	0.237	0.12	0.147	0.114	0.122	0.164
zpbp	0.11	0.201	0.359	0.204	0.125	0.133	0.145	0.218
hoxd-as1	0.111	0.178	0.235	0.105	0.109	0.105	0.115	0.155
mcrs1	0.111	0.175	0.374	0.131	0.118	0.107	0.116	0.2
odc1	0.111	0.198	0.337	0.158	0.155	0.122	0.135	0.202
impdh1p11	0.111	0.175	0.274	0.155	0.159	0.134	0.142	0.192
ndufa12	0.111	0.168	0.297	0.184	0.148	0.125	0.133	0.192
pafah1b1p2	0.111	0.18	0.274	0.144	0.142	0.137	0.146	0.195
kcnip1	0.111	0.158	0.263	0.191	0.1	0.106	0.113	0.164
hpgds	0.111	0.158	0.194	0.153	0.119	0.101	0.108	0.139
dguok	0.111	0.181	0.336	0.161	0.122	0.125	0.135	0.204
ervk-6	0.112	0.196	0.403	0.197	0.196	0.107	0.119	0.209
mcmbp	0.112	0.22	0.415	0.192	0.112	0.128	0.143	0.232
tekt2	0.112	0.204	0.34	0.186	0.169	0.127	0.14	0.207
glud2	0.112	0.176	0.368	0.208	0.157	0.132	0.142	0.221
phf7	0.112	0.205	0.314	0.158	0.24	0.115	0.128	0.188
fabp4	0.111	0.147	0.197	0.122	0.135	0.111	0.117	0.149
wbscr22	0.111	0.207	0.348	0.123	0.19	0.109	0.123	0.194
pafah1b1p1	0.111	0.18	0.274	0.144	0.142	0.137	0.146	0.195
krt32	0.111	0.168	0.206	0.111	0.118	0.131	0.14	0.169
gatc	0.11	0.162	0.379	0.166	0.1	0.141	0.148	0.231
qrsl1	0.11	0.162	0.379	0.166	0.1	0.141	0.148	0.231
dpy19l2	0.11	0.171	0.297	0.152	0.159	0.13	0.138	0.196
rpl4	0.11	0.183	0.362	0.201	0.117	0.119	0.129	0.206
rnf6	0.11	0.151	0.212	0.112	0.181	0.113	0.119	0.155
synm	0.11	0.17	0.249	0.167	0.103	0.113	0.122	0.166
txnrd3	0.11	0.145	0.245	0.109	0.111	0.103	0.108	0.156
acss1	0.11	0.149	0.284	0.118	0.136	0.106	0.112	0.171
ube2q1	0.11	0.174	0.351	0.17	0.131	0.131	0.14	0.215
mir137	0.111	0.181	0.258	0.115	0.102	0.104	0.115	0.162
dfna5	0.111	0.176	0.263	0.143	0.12	0.136	0.145	0.191
mir1827	0.111	0.199	0.3	0.156	0.175	0.114	0.126	0.183
snrpc	0.111	0.207	0.457	0.227	0.17	0.111	0.124	0.229
fendrr	0.111	0.2	0.342	0.118	0.114	0.116	0.129	0.198

mir1287	0.111	0.166	0.273	0.129	0.124	0.129	0.137	0.188
rbbp6	0.109	0.203	0.406	0.144	0.262	0.102	0.116	0.206
tead4	0.109	0.176	0.218	0.11	0.173	0.115	0.125	0.159
med17	0.107	0.152	0.292	0.144	0.249	0.115	0.122	0.182
rn7sl3	0.107	0.165	0.328	0.158	0.149	0.102	0.11	0.181
celf1	0.107	0.181	0.342	0.164	0.158	0.108	0.119	0.191
slc27a5	0.107	0.143	0.27	0.147	0.139	0.137	0.142	0.194
atp5b	0.107	0.166	0.312	0.154	0.102	0.11	0.119	0.184
aasth13	0.107	0.15	0.215	0.124	0.123	0.106	0.112	0.15
aasth8	0.107	0.15	0.215	0.124	0.123	0.106	0.112	0.15
sf3b3	0.107	0.176	0.368	0.186	0.195	0.112	0.122	0.203
crem	0.107	0.128	0.173	0.14	0.126	0.106	0.11	0.137
fndc1	0.107	0.148	0.185	0.14	0.114	0.109	0.115	0.143
ctbp2	0.107	0.168	0.287	0.106	0.14	0.103	0.112	0.17
kdm2b	0.107	0.177	0.321	0.134	0.119	0.112	0.122	0.188
ube4b	0.107	0.172	0.357	0.129	0.104	0.121	0.13	0.208
ndufa6	0.107	0.165	0.316	0.185	0.143	0.115	0.123	0.189
ap3s2	0.107	0.157	0.261	0.206	0.102	0.113	0.12	0.17
rtn1	0.107	0.182	0.325	0.18	0.136	0.13	0.14	0.206
aasth10	0.107	0.15	0.215	0.124	0.123	0.106	0.112	0.15
aasth12	0.107	0.15	0.215	0.124	0.123	0.106	0.112	0.15
defb116	0.106	0.166	0.247	0.108	0.114	0.101	0.11	0.155
degs2	0.106	0.168	0.29	0.194	0.17	0.122	0.131	0.187
dux4l15	0.106	0.205	0.345	0.178	0.122	0.124	0.138	0.206
ythdc1	0.106	0.152	0.289	0.252	0.127	0.103	0.11	0.17
cyp4f3	0.106	0.166	0.254	0.162	0.113	0.104	0.113	0.161
itfg3	0.106	0.185	0.341	0.175	0.152	0.136	0.147	0.216
rpl23ap5	0.106	0.185	0.341	0.175	0.152	0.136	0.147	0.216
psmd3	0.106	0.185	0.336	0.18	0.105	0.112	0.124	0.194
tfb2m	0.106	0.159	0.322	0.128	0.193	0.123	0.131	0.198
spop	0.107	0.147	0.266	0.109	0.134	0.123	0.129	0.181
aasth11	0.107	0.15	0.215	0.124	0.123	0.106	0.112	0.15
aasth9	0.107	0.15	0.215	0.124	0.123	0.106	0.112	0.15
taar3	0.106	0.137	0.198	0.116	0.137	0.108	0.113	0.146
kiaa0391	0.106	0.17	0.323	0.159	0.1	0.126	0.135	0.201
naspp1	0.106	0.196	0.366	0.2	0.16	0.122	0.135	0.212
msmb	0.106	0.196	0.302	0.138	0.206	0.114	0.126	0.184
cps1	0.107	0.18	0.298	0.148	0.168	0.11	0.12	0.179
actn2	0.107	0.151	0.251	0.172	0.109	0.109	0.115	0.164
cyyr1	0.108	0.157	0.186	0.115	0.284	0.13	0.137	0.162
mpzl3	0.108	0.167	0.204	0.13	0.204	0.118	0.126	0.157

slc3a1	0.108	0.176	0.302	0.171	0.128	0.127	0.136	0.195
bcs1l	0.108	0.199	0.397	0.153	0.111	0.124	0.136	0.222
zp2	0.108	0.187	0.303	0.191	0.104	0.138	0.149	0.206
znf382	0.108	0.182	0.387	0.136	0.245	0.137	0.147	0.231
dcp1b	0.108	0.184	0.416	0.175	0.141	0.12	0.131	0.225
gabpb1	0.108	0.175	0.254	0.153	0.153	0.118	0.127	0.172
dnah3	0.108	0.172	0.301	0.119	0.175	0.135	0.144	0.202
mtl5	0.109	0.191	0.339	0.154	0.113	0.125	0.136	0.205
lsm10	0.109	0.196	0.421	0.176	0.137	0.118	0.13	0.224
znf593	0.109	0.168	0.351	0.149	0.126	0.117	0.125	0.202
abracl	0.108	0.162	0.289	0.156	0.119	0.103	0.111	0.17
usp51	0.108	0.18	0.3	0.176	0.111	0.108	0.118	0.178
sez6	0.108	0.161	0.213	0.115	0.117	0.112	0.119	0.154
msto1	0.108	0.179	0.372	0.14	0.104	0.139	0.149	0.228
acot7	0.108	0.171	0.336	0.194	0.159	0.116	0.125	0.196
ssx3	0.108	0.194	0.268	0.149	0.154	0.103	0.116	0.164
sh3tc2	0.108	0.169	0.274	0.162	0.12	0.121	0.13	0.182
psmd7	0.108	0.182	0.43	0.187	0.19	0.146	0.156	0.252
cbln4	0.108	0.155	0.186	0.133	0.127	0.105	0.112	0.14
znf155	0.108	0.182	0.349	0.155	0.167	0.121	0.131	0.205
chchd3	0.108	0.168	0.278	0.18	0.107	0.11	0.118	0.173
tyw1	0.107	0.161	0.31	0.168	0.122	0.118	0.125	0.19
tyw1b	0.107	0.161	0.31	0.168	0.122	0.118	0.125	0.19
oip5	0.108	0.189	0.313	0.131	0.115	0.109	0.12	0.183
edc4	0.108	0.182	0.343	0.187	0.126	0.119	0.13	0.202
fgfr1op	0.108	0.142	0.198	0.131	0.148	0.112	0.117	0.15
cops4	0.108	0.156	0.314	0.175	0.103	0.132	0.139	0.204
chodl	0.108	0.204	0.315	0.173	0.126	0.106	0.12	0.181
appbp2	0.108	0.155	0.318	0.129	0.226	0.117	0.124	0.192
dux1	0.108	0.184	0.341	0.209	0.169	0.126	0.136	0.206
dux5	0.108	0.184	0.341	0.209	0.169	0.126	0.136	0.206
c7orf13	0.122	0.189	0.392	0.171	0.162	0.155	0.164	0.247
srsf1	0.122	0.211	0.448	0.22	0.143	0.126	0.139	0.239
abi3	0.133	0.176	0.271	0.175	0.103	0.121	0.127	0.178
wbp4	0.133	0.169	0.281	0.246	0.142	0.139	0.145	0.197
wnk2	0.133	0.189	0.283	0.169	0.105	0.102	0.111	0.165
znf764	0.133	0.199	0.268	0.143	0.39	0.117	0.127	0.173
agpat6	0.133	0.198	0.318	0.172	0.248	0.141	0.151	0.211
atp8b2	0.133	0.182	0.273	0.158	0.144	0.145	0.152	0.2
sez6l	0.133	0.176	0.225	0.129	0.101	0.158	0.164	0.197
fscn3	0.133	0.213	0.376	0.203	0.101	0.164	0.175	0.248

aire	0.133	0.216	0.238	0.153	0.165	0.106	0.118	0.155
chchd7	0.134	0.196	0.317	0.15	0.117	0.122	0.131	0.193
ribc2	0.134	0.2	0.301	0.222	0.121	0.13	0.139	0.195
cxorf27	0.134	0.194	0.287	0.135	0.149	0.164	0.173	0.221
amd1	0.134	0.225	0.389	0.202	0.114	0.143	0.156	0.234
lrpprc	0.134	0.189	0.307	0.195	0.2	0.138	0.146	0.204
tceal8	0.133	0.207	0.333	0.162	0.14	0.134	0.145	0.209
sergef	0.134	0.206	0.431	0.26	0.117	0.156	0.166	0.259
clint1	0.133	0.183	0.348	0.23	0.113	0.135	0.143	0.215
crct1	0.133	0.162	0.26	0.159	0.113	0.116	0.121	0.17
esx1	0.133	0.217	0.247	0.135	0.172	0.142	0.155	0.19
stard10	0.133	0.189	0.277	0.148	0.18	0.124	0.133	0.183
cnot1	0.133	0.21	0.403	0.178	0.229	0.166	0.177	0.259
ampd3	0.133	0.206	0.383	0.224	0.139	0.135	0.146	0.226
ano6	0.133	0.18	0.231	0.159	0.116	0.149	0.156	0.191
rhoxf1	0.133	0.183	0.219	0.132	0.161	0.13	0.138	0.17
htra4	0.133	0.214	0.29	0.163	0.125	0.123	0.135	0.186
ak1	0.133	0.201	0.382	0.198	0.15	0.151	0.161	0.239
pex5l	0.133	0.177	0.267	0.185	0.108	0.118	0.125	0.175
ervk-4	0.133	0.186	0.356	0.139	0.127	0.125	0.134	0.208
slc10a7	0.133	0.176	0.306	0.158	0.115	0.148	0.154	0.213
hsd17b7	0.133	0.185	0.238	0.144	0.185	0.121	0.129	0.168
calcoco2	0.133	0.165	0.28	0.13	0.181	0.121	0.126	0.181
ppp1r3b	0.133	0.174	0.313	0.163	0.121	0.155	0.161	0.221
irf2bp2	0.133	0.167	0.224	0.162	0.142	0.119	0.125	0.162
pex10	0.133	0.196	0.365	0.183	0.169	0.151	0.16	0.234
ncapg2	0.134	0.229	0.329	0.149	0.129	0.153	0.166	0.224
mir18a	0.134	0.182	0.275	0.151	0.116	0.131	0.138	0.188
arid2	0.135	0.21	0.36	0.126	0.218	0.141	0.152	0.223
znf214	0.135	0.195	0.276	0.168	0.167	0.172	0.18	0.224
acsl3	0.135	0.188	0.29	0.158	0.203	0.122	0.13	0.185
mef2a	0.135	0.174	0.291	0.167	0.211	0.113	0.119	0.177
tmem141	0.135	0.189	0.202	0.131	0.106	0.158	0.166	0.189
trappc3	0.135	0.207	0.398	0.21	0.142	0.155	0.165	0.247
afap1	0.135	0.167	0.228	0.202	0.116	0.131	0.136	0.173
pou3f3	0.135	0.202	0.191	0.122	0.182	0.108	0.119	0.142
arsep1	0.135	0.203	0.313	0.164	0.135	0.159	0.169	0.225
orc3	0.135	0.211	0.316	0.172	0.101	0.142	0.153	0.211
rnf122	0.135	0.199	0.342	0.183	0.157	0.151	0.161	0.227
dnajc4	0.135	0.222	0.398	0.221	0.138	0.139	0.152	0.234
ak4	0.135	0.202	0.347	0.189	0.117	0.113	0.124	0.195

rasl10a	0.135	0.203	0.289	0.202	0.11	0.147	0.157	0.207
cnbp	0.135	0.202	0.371	0.169	0.127	0.144	0.154	0.229
alyref	0.135	0.205	0.438	0.218	0.126	0.142	0.152	0.248
spidr	0.135	0.188	0.264	0.138	0.101	0.147	0.155	0.199
sept8	0.135	0.2	0.366	0.166	0.13	0.156	0.165	0.238
c2cd4c	0.134	0.159	0.173	0.14	0.125	0.145	0.149	0.169
azin1	0.134	0.233	0.409	0.192	0.14	0.141	0.156	0.239
asns	0.134	0.196	0.336	0.149	0.116	0.125	0.135	0.202
mbnl3	0.134	0.194	0.305	0.151	0.121	0.129	0.138	0.196
znf117	0.134	0.229	0.411	0.173	0.282	0.145	0.158	0.243
gpr108	0.134	0.202	0.434	0.254	0.103	0.158	0.168	0.262
srsf7	0.134	0.205	0.408	0.217	0.148	0.136	0.146	0.234
sun3	0.134	0.2	0.315	0.171	0.117	0.138	0.147	0.206
znf366	0.134	0.185	0.205	0.11	0.391	0.105	0.114	0.144
napsb	0.134	0.214	0.341	0.205	0.131	0.148	0.159	0.223
cited2	0.134	0.176	0.267	0.11	0.206	0.125	0.132	0.18
lemd1	0.134	0.225	0.284	0.143	0.112	0.128	0.142	0.189
six5	0.134	0.19	0.243	0.126	0.147	0.168	0.176	0.211
rpl3	0.134	0.204	0.379	0.162	0.142	0.14	0.15	0.228
psmc4	0.134	0.204	0.448	0.169	0.248	0.159	0.169	0.266
ing5	0.134	0.221	0.376	0.12	0.11	0.118	0.131	0.208
bcas4	0.133	0.213	0.338	0.187	0.133	0.132	0.144	0.209
zkscan7	0.133	0.207	0.312	0.166	0.126	0.161	0.172	0.226
gpr82	0.131	0.166	0.234	0.135	0.15	0.138	0.144	0.182
xrn1	0.131	0.187	0.327	0.163	0.135	0.126	0.134	0.2
dcaf13	0.131	0.166	0.241	0.139	0.121	0.138	0.144	0.184
pcat4	0.131	0.199	0.28	0.174	0.141	0.14	0.15	0.198
zmiz2	0.131	0.153	0.149	0.118	0.363	0.116	0.12	0.136
or10j5	0.131	0.188	0.199	0.157	0.12	0.143	0.151	0.175
gpr149	0.131	0.223	0.288	0.176	0.166	0.128	0.142	0.19
defb123	0.131	0.181	0.193	0.185	0.14	0.112	0.12	0.146
defb135	0.131	0.181	0.193	0.185	0.14	0.112	0.12	0.146
macrod1	0.131	0.17	0.179	0.122	0.337	0.103	0.109	0.133
arid3c	0.131	0.205	0.379	0.146	0.284	0.136	0.146	0.225
cdh12	0.131	0.211	0.277	0.143	0.12	0.132	0.143	0.189
tmprss5	0.131	0.211	0.317	0.198	0.117	0.129	0.14	0.199
snord32a	0.131	0.21	0.324	0.191	0.122	0.118	0.13	0.192
synpo2	0.131	0.183	0.361	0.207	0.106	0.137	0.145	0.22
abhd5	0.131	0.172	0.304	0.155	0.128	0.127	0.133	0.194
coro2a	0.131	0.164	0.234	0.125	0.3	0.118	0.124	0.164
slc23a1	0.131	0.196	0.347	0.198	0.101	0.132	0.141	0.211

eif1	0.13	0.22	0.476	0.206	0.133	0.14	0.153	0.259
slc30a3	0.13	0.173	0.257	0.168	0.126	0.121	0.128	0.174
raver2	0.13	0.19	0.34	0.171	0.138	0.121	0.13	0.2
tgfbr3l	0.13	0.226	0.357	0.203	0.185	0.151	0.165	0.232
pabpc5-as1	0.13	0.226	0.357	0.203	0.185	0.151	0.165	0.232
srrm2	0.13	0.213	0.384	0.217	0.188	0.115	0.127	0.208
smim18	0.13	0.226	0.357	0.203	0.185	0.151	0.165	0.232
tfam	0.13	0.198	0.356	0.15	0.231	0.132	0.142	0.214
poteb2	0.131	0.183	0.289	0.157	0.192	0.132	0.14	0.194
nek4	0.131	0.2	0.297	0.177	0.144	0.142	0.152	0.205
tut1	0.131	0.197	0.368	0.214	0.151	0.138	0.148	0.223
prkxp1	0.131	0.221	0.298	0.22	0.14	0.128	0.141	0.193
gfer	0.131	0.22	0.339	0.219	0.119	0.143	0.156	0.219
ghitm	0.131	0.188	0.247	0.156	0.22	0.133	0.142	0.181
potec	0.131	0.183	0.289	0.157	0.192	0.132	0.14	0.194
znf17	0.131	0.204	0.326	0.166	0.208	0.131	0.141	0.204
creb3l3	0.132	0.156	0.265	0.122	0.159	0.143	0.147	0.196
myl12b	0.132	0.167	0.236	0.178	0.106	0.117	0.123	0.164
rtp3	0.132	0.173	0.221	0.129	0.123	0.131	0.137	0.171
ppfia4	0.132	0.176	0.236	0.167	0.114	0.124	0.131	0.17
gpr50	0.132	0.158	0.221	0.161	0.218	0.129	0.133	0.17
slc38a3	0.132	0.187	0.343	0.159	0.106	0.157	0.165	0.232
snord87	0.132	0.229	0.423	0.181	0.132	0.124	0.138	0.228
mtmr8	0.132	0.143	0.173	0.21	0.106	0.144	0.146	0.168
lrrc8a	0.132	0.2	0.287	0.203	0.112	0.122	0.132	0.184
st3gal3	0.132	0.235	0.313	0.18	0.107	0.127	0.141	0.196
zdhhc7	0.132	0.144	0.201	0.139	0.104	0.161	0.163	0.191
adam3a	0.133	0.21	0.266	0.169	0.112	0.128	0.139	0.182
abhd2	0.133	0.182	0.283	0.153	0.183	0.129	0.136	0.189
pspc1	0.133	0.214	0.431	0.19	0.35	0.135	0.147	0.241
mbnl1	0.133	0.202	0.366	0.158	0.115	0.138	0.148	0.222
slc25a18	0.133	0.185	0.327	0.153	0.194	0.164	0.171	0.233
irf2bp1	0.132	0.145	0.225	0.147	0.138	0.13	0.132	0.172
rna28s5	0.132	0.23	0.421	0.17	0.101	0.142	0.156	0.244
myo1a	0.132	0.198	0.321	0.185	0.105	0.157	0.166	0.225
ptprq	0.132	0.188	0.224	0.149	0.1	0.147	0.155	0.187
hnrnpa1	0.132	0.215	0.487	0.242	0.155	0.123	0.135	0.247
ctbp1	0.132	0.181	0.305	0.112	0.213	0.121	0.129	0.189
rbm44	0.132	0.237	0.383	0.235	0.149	0.137	0.152	0.227
trappc2p1	0.132	0.175	0.258	0.145	0.151	0.119	0.126	0.173
ikzf5	0.132	0.182	0.229	0.148	0.117	0.134	0.142	0.177

wibg	0.132	0.191	0.378	0.195	0.132	0.135	0.143	0.224
trim32	0.132	0.167	0.365	0.135	0.104	0.17	0.175	0.251
trim36	0.132	0.2	0.398	0.188	0.135	0.17	0.179	0.261
tmem135	0.132	0.206	0.384	0.162	0.11	0.124	0.135	0.216
mpeg1	0.132	0.224	0.275	0.252	0.13	0.118	0.131	0.177
tmem192	0.132	0.2	0.338	0.184	0.148	0.135	0.145	0.211
spanxa1	0.132	0.216	0.289	0.164	0.193	0.137	0.149	0.198
ptx4	0.132	0.163	0.183	0.101	0.114	0.147	0.152	0.174
bcas2	0.132	0.185	0.308	0.166	0.185	0.135	0.143	0.203
tfe3	0.132	0.204	0.235	0.132	0.11	0.119	0.13	0.165
kif17	0.135	0.191	0.31	0.187	0.11	0.147	0.155	0.213
gtpbp10	0.135	0.22	0.449	0.174	0.139	0.144	0.156	0.254
mdp1	0.138	0.188	0.328	0.234	0.106	0.139	0.147	0.212
galr3	0.138	0.18	0.243	0.198	0.105	0.144	0.15	0.189
sh3gl1	0.139	0.186	0.295	0.206	0.109	0.149	0.156	0.21
pcdha3	0.138	0.213	0.355	0.186	0.127	0.159	0.17	0.238
ubr2	0.138	0.214	0.381	0.144	0.111	0.148	0.159	0.235
habp4	0.138	0.193	0.326	0.238	0.122	0.13	0.139	0.203
rbm15	0.138	0.198	0.248	0.11	0.317	0.105	0.115	0.156
znf334	0.139	0.226	0.388	0.201	0.164	0.141	0.154	0.232
lasp1	0.139	0.201	0.28	0.163	0.105	0.18	0.189	0.233
ckmt1a	0.139	0.211	0.395	0.192	0.11	0.157	0.167	0.248
alg3	0.139	0.193	0.233	0.146	0.115	0.141	0.149	0.183
isca2	0.139	0.214	0.391	0.179	0.138	0.154	0.165	0.244
cuedc2	0.139	0.162	0.154	0.142	0.142	0.122	0.127	0.142
rplp2	0.139	0.197	0.355	0.228	0.139	0.13	0.139	0.212
chkb	0.139	0.203	0.3	0.167	0.129	0.159	0.168	0.22
isca1	0.139	0.214	0.385	0.189	0.133	0.147	0.157	0.236
tvp23b	0.138	0.216	0.352	0.183	0.137	0.157	0.168	0.234
tvp23a	0.138	0.216	0.352	0.183	0.137	0.157	0.168	0.234
snord33	0.138	0.211	0.281	0.182	0.101	0.12	0.131	0.18
rpp30	0.138	0.215	0.378	0.199	0.111	0.142	0.153	0.23
rasl11a	0.138	0.203	0.344	0.199	0.133	0.155	0.164	0.23
larp7	0.138	0.209	0.382	0.182	0.196	0.13	0.14	0.22
smyd3	0.138	0.211	0.31	0.169	0.112	0.135	0.146	0.202
gadd45gip1	0.138	0.208	0.265	0.181	0.216	0.12	0.131	0.175
med24	0.138	0.167	0.205	0.122	0.492	0.147	0.151	0.18
cnot6	0.138	0.202	0.368	0.183	0.195	0.164	0.173	0.246
pdha2	0.138	0.218	0.374	0.232	0.23	0.141	0.153	0.228
vapa	0.138	0.185	0.316	0.223	0.128	0.137	0.145	0.206
gtf3a	0.138	0.177	0.248	0.181	0.147	0.145	0.151	0.191

zbtb16	0.138	0.215	0.299	0.157	0.282	0.151	0.162	0.213
tuba8	0.138	0.22	0.411	0.201	0.103	0.167	0.179	0.262
cdc40	0.138	0.204	0.378	0.2	0.118	0.176	0.185	0.26
zdhhc5	0.138	0.168	0.278	0.203	0.114	0.133	0.138	0.191
pno1	0.138	0.231	0.307	0.226	0.127	0.123	0.137	0.191
fgf21	0.139	0.161	0.207	0.122	0.123	0.175	0.179	0.206
ccdc37	0.139	0.222	0.275	0.143	0.131	0.161	0.173	0.214
zdhhc19	0.14	0.181	0.308	0.228	0.111	0.139	0.145	0.204
svop	0.14	0.213	0.392	0.183	0.135	0.163	0.173	0.252
pcgf1	0.14	0.218	0.291	0.139	0.117	0.155	0.167	0.214
filip1l	0.14	0.207	0.361	0.157	0.176	0.146	0.156	0.228
ovca2	0.14	0.236	0.428	0.197	0.127	0.156	0.17	0.257
mysm1	0.14	0.192	0.282	0.135	0.203	0.144	0.152	0.202
atf5	0.14	0.193	0.312	0.15	0.134	0.145	0.153	0.211
dync1i2	0.14	0.202	0.313	0.186	0.158	0.136	0.145	0.203
hmbox1	0.14	0.215	0.294	0.138	0.197	0.13	0.141	0.192
mirlet7c	0.14	0.234	0.297	0.134	0.234	0.115	0.129	0.18
stau2	0.14	0.199	0.4	0.195	0.105	0.146	0.155	0.24
ankrd7	0.14	0.206	0.294	0.143	0.148	0.13	0.14	0.193
srsf4	0.14	0.219	0.438	0.247	0.144	0.154	0.166	0.259
ampd2	0.14	0.195	0.295	0.233	0.126	0.124	0.133	0.188
lrrn3	0.14	0.173	0.208	0.152	0.131	0.17	0.175	0.201
nr6a1	0.14	0.214	0.255	0.142	0.356	0.142	0.153	0.191
etf1	0.14	0.213	0.421	0.219	0.12	0.148	0.158	0.248
zbtb32	0.14	0.225	0.309	0.177	0.27	0.111	0.124	0.18
kif24	0.139	0.201	0.326	0.158	0.111	0.152	0.161	0.222
prdm9	0.139	0.21	0.352	0.158	0.204	0.184	0.194	0.259
tsen2	0.139	0.22	0.345	0.144	0.104	0.149	0.161	0.226
supt4h1	0.139	0.211	0.416	0.153	0.186	0.144	0.155	0.243
dph3	0.139	0.195	0.279	0.159	0.12	0.132	0.141	0.19
pex19	0.139	0.223	0.463	0.224	0.135	0.145	0.157	0.258
ugdh	0.139	0.193	0.34	0.144	0.117	0.141	0.15	0.217
znf331	0.139	0.236	0.359	0.196	0.154	0.161	0.175	0.24
arl3	0.14	0.193	0.368	0.232	0.102	0.174	0.182	0.255
atpif1	0.14	0.227	0.456	0.212	0.12	0.127	0.139	0.24
asmd	0.14	0.181	0.248	0.133	0.154	0.124	0.13	0.173
pck1	0.14	0.176	0.295	0.166	0.279	0.144	0.15	0.205
acsbg1	0.14	0.211	0.349	0.187	0.141	0.154	0.165	0.231
zkscan3	0.14	0.203	0.25	0.157	0.14	0.124	0.134	0.173
nfib	0.14	0.227	0.305	0.152	0.154	0.144	0.157	0.209
lyrm7	0.137	0.209	0.41	0.21	0.125	0.14	0.151	0.238

heatr1	0.137	0.231	0.422	0.195	0.156	0.145	0.158	0.246
fam71d	0.136	0.176	0.266	0.203	0.113	0.145	0.151	0.198
fam71f2	0.136	0.176	0.266	0.203	0.113	0.145	0.151	0.198
mir505	0.136	0.207	0.275	0.128	0.101	0.124	0.134	0.181
fam71a	0.136	0.176	0.266	0.203	0.113	0.145	0.151	0.198
fam71b	0.136	0.176	0.266	0.203	0.113	0.145	0.151	0.198
ndufa7	0.136	0.207	0.394	0.201	0.164	0.145	0.155	0.237
fam71e1	0.136	0.176	0.266	0.203	0.113	0.145	0.151	0.198
crnde	0.136	0.218	0.337	0.164	0.164	0.145	0.156	0.219
spata33	0.136	0.229	0.328	0.165	0.21	0.14	0.153	0.212
crabp2	0.136	0.171	0.229	0.128	0.137	0.169	0.174	0.207
tsnax	0.136	0.18	0.327	0.193	0.106	0.137	0.144	0.209
glul	0.136	0.211	0.29	0.165	0.151	0.142	0.153	0.202
bckdk	0.136	0.208	0.382	0.218	0.134	0.142	0.153	0.231
src	0.136	0.153	0.171	0.197	0.122	0.128	0.131	0.153
atp1a4	0.136	0.217	0.327	0.196	0.131	0.143	0.155	0.215
spata8	0.136	0.17	0.184	0.13	0.125	0.174	0.179	0.197
itgbl1	0.136	0.203	0.257	0.15	0.108	0.137	0.147	0.188
dffb	0.136	0.236	0.405	0.203	0.125	0.13	0.144	0.227
gusbp5	0.135	0.192	0.229	0.113	0.117	0.145	0.153	0.186
abhd1	0.135	0.19	0.308	0.179	0.26	0.125	0.133	0.193
abcb8	0.135	0.224	0.42	0.15	0.135	0.128	0.141	0.231
pias3	0.135	0.16	0.235	0.232	0.169	0.119	0.124	0.165
igkdel	0.135	0.176	0.18	0.108	0.111	0.134	0.14	0.161
rps10	0.135	0.223	0.396	0.23	0.121	0.13	0.143	0.225
clk3	0.135	0.21	0.324	0.183	0.105	0.127	0.138	0.2
dyx1c1	0.135	0.179	0.238	0.131	0.159	0.136	0.143	0.181
krt86	0.135	0.2	0.239	0.151	0.134	0.138	0.147	0.182
pate1	0.135	0.208	0.264	0.17	0.157	0.15	0.16	0.201
ndufaf4	0.136	0.202	0.239	0.213	0.242	0.115	0.125	0.162
wfdc5	0.136	0.212	0.4	0.21	0.118	0.15	0.161	0.243
col15a1	0.135	0.219	0.318	0.184	0.111	0.136	0.149	0.206
prmt2	0.135	0.214	0.388	0.186	0.333	0.128	0.139	0.22
bcas1	0.135	0.205	0.345	0.194	0.111	0.14	0.151	0.218
schip1	0.135	0.192	0.264	0.218	0.158	0.139	0.148	0.192
folr2	0.136	0.221	0.329	0.234	0.115	0.122	0.135	0.197
tbc1d15	0.136	0.185	0.277	0.209	0.175	0.15	0.157	0.205
ywhaz	0.137	0.165	0.245	0.228	0.109	0.113	0.118	0.162
astl	0.137	0.214	0.238	0.175	0.152	0.148	0.159	0.191
ntan1	0.137	0.194	0.308	0.158	0.129	0.142	0.15	0.207
thrap3	0.137	0.221	0.372	0.167	0.249	0.134	0.146	0.221

chchd6	0.137	0.222	0.417	0.17	0.143	0.159	0.171	0.257
snord63	0.137	0.212	0.367	0.179	0.139	0.142	0.153	0.226
tekt1	0.137	0.209	0.309	0.187	0.144	0.168	0.178	0.231
ergic3	0.137	0.208	0.311	0.177	0.108	0.153	0.163	0.218
rgs17	0.137	0.149	0.194	0.146	0.141	0.137	0.14	0.168
atl1	0.137	0.198	0.337	0.174	0.112	0.164	0.172	0.236
prdm16	0.137	0.173	0.217	0.125	0.18	0.134	0.14	0.173
calcoco1	0.137	0.152	0.209	0.118	0.189	0.11	0.113	0.149
tpcn1	0.137	0.177	0.258	0.147	0.106	0.119	0.126	0.172
sept12	0.137	0.216	0.314	0.155	0.14	0.166	0.178	0.231
atp6v0d2	0.137	0.208	0.3	0.181	0.126	0.108	0.119	0.175
midn	0.137	0.194	0.263	0.106	0.108	0.175	0.183	0.223
snord62a	0.137	0.212	0.367	0.179	0.139	0.142	0.153	0.226
snord62b	0.137	0.212	0.367	0.179	0.139	0.142	0.153	0.226
hus1b	0.136	0.211	0.34	0.153	0.101	0.164	0.175	0.237
psme1	0.136	0.215	0.363	0.192	0.112	0.126	0.138	0.211
mospd3	0.136	0.166	0.179	0.163	0.103	0.128	0.133	0.155
linc00984	0.136	0.177	0.216	0.121	0.122	0.114	0.12	0.154
mir4435-1hg	0.136	0.177	0.216	0.121	0.122	0.114	0.12	0.154
patl1	0.136	0.198	0.381	0.172	0.142	0.145	0.154	0.233
znf383	0.136	0.219	0.399	0.22	0.16	0.121	0.133	0.218
smyd5	0.137	0.191	0.277	0.172	0.319	0.152	0.16	0.207
gfm2	0.137	0.223	0.441	0.191	0.11	0.138	0.151	0.246
vnn3	0.137	0.22	0.272	0.189	0.125	0.113	0.125	0.171
znf185	0.137	0.199	0.266	0.168	0.176	0.133	0.143	0.187
fosb	0.137	0.177	0.248	0.173	0.11	0.137	0.143	0.185
ubtf	0.137	0.227	0.44	0.176	0.16	0.137	0.15	0.244
chmp7	0.137	0.188	0.365	0.209	0.132	0.153	0.161	0.236
mgat1	0.137	0.216	0.271	0.157	0.1	0.137	0.148	0.192
nol12	0.13	0.193	0.358	0.203	0.134	0.141	0.15	0.223
acbd5	0.13	0.133	0.134	0.154	0.11	0.112	0.113	0.127
rab11fip4	0.124	0.176	0.273	0.187	0.119	0.154	0.162	0.209
fhdc1	0.124	0.181	0.281	0.146	0.103	0.139	0.148	0.198
cox6b2	0.124	0.213	0.404	0.221	0.151	0.147	0.159	0.243
atad3b	0.124	0.227	0.398	0.146	0.165	0.13	0.145	0.227
scoc	0.124	0.165	0.333	0.148	0.102	0.133	0.139	0.209
klf8	0.124	0.193	0.339	0.164	0.171	0.104	0.114	0.185
ptchd3	0.124	0.17	0.259	0.137	0.106	0.129	0.136	0.182
farp2	0.125	0.167	0.237	0.176	0.108	0.134	0.14	0.18
ccnl1	0.125	0.197	0.282	0.164	0.14	0.136	0.146	0.196
ctdspl	0.125	0.193	0.284	0.15	0.101	0.13	0.14	0.191

acad9	0.125	0.178	0.308	0.156	0.136	0.132	0.14	0.2
nde1	0.125	0.193	0.262	0.142	0.105	0.104	0.114	0.161
rbm39	0.125	0.192	0.3	0.196	0.163	0.111	0.122	0.179
eya3	0.125	0.165	0.221	0.167	0.161	0.117	0.123	0.16
znf148	0.125	0.196	0.313	0.159	0.132	0.135	0.145	0.204
dennd1b	0.125	0.189	0.386	0.189	0.128	0.147	0.156	0.237
fhl5	0.124	0.16	0.212	0.169	0.118	0.113	0.119	0.153
lsm14a	0.124	0.168	0.343	0.155	0.109	0.105	0.112	0.188
bckdhb	0.124	0.19	0.364	0.216	0.116	0.135	0.145	0.221
c17orf89	0.124	0.165	0.245	0.161	0.213	0.158	0.164	0.204
tekt3	0.124	0.209	0.329	0.172	0.193	0.134	0.146	0.208
pdp1	0.124	0.148	0.258	0.135	0.144	0.122	0.126	0.176
ino80	0.124	0.208	0.434	0.181	0.147	0.163	0.174	0.267
cops7b	0.124	0.194	0.445	0.182	0.128	0.145	0.155	0.254
cpne9	0.124	0.205	0.389	0.225	0.149	0.124	0.136	0.219
acly	0.124	0.165	0.317	0.165	0.133	0.127	0.133	0.198
sohlh2	0.124	0.214	0.208	0.116	0.12	0.107	0.12	0.147
rint1	0.124	0.192	0.304	0.147	0.123	0.177	0.187	0.239
ypel4	0.124	0.194	0.34	0.185	0.155	0.135	0.145	0.213
sox12	0.124	0.185	0.259	0.132	0.231	0.123	0.132	0.177
znf606	0.124	0.21	0.423	0.268	0.255	0.119	0.132	0.225
nnat	0.124	0.193	0.288	0.178	0.14	0.148	0.157	0.208
paqr8	0.124	0.167	0.199	0.147	0.185	0.111	0.117	0.147
arid5b	0.124	0.17	0.255	0.17	0.148	0.107	0.115	0.162
lipa	0.125	0.188	0.312	0.18	0.146	0.112	0.122	0.184
mmadhc	0.125	0.178	0.314	0.19	0.102	0.123	0.13	0.194
snord80	0.125	0.178	0.341	0.133	0.138	0.102	0.11	0.183
snord77	0.125	0.178	0.341	0.133	0.138	0.102	0.11	0.183
letmd1	0.125	0.163	0.174	0.128	0.104	0.127	0.133	0.154
snord75	0.125	0.178	0.341	0.133	0.138	0.102	0.11	0.183
snord74	0.125	0.178	0.341	0.133	0.138	0.102	0.11	0.183
snord76	0.125	0.178	0.341	0.133	0.138	0.102	0.11	0.183
snord78	0.125	0.178	0.341	0.133	0.138	0.102	0.11	0.183
prss35	0.125	0.178	0.171	0.115	0.157	0.104	0.113	0.133
srp54	0.126	0.2	0.41	0.215	0.111	0.117	0.128	0.219
tdo2	0.126	0.177	0.259	0.142	0.145	0.102	0.11	0.158
xbp1p1	0.126	0.191	0.37	0.179	0.243	0.144	0.153	0.23
rpl36ap37	0.126	0.196	0.283	0.177	0.237	0.128	0.138	0.189
polr3gl	0.126	0.214	0.454	0.163	0.157	0.131	0.143	0.244
fbp1	0.126	0.182	0.325	0.177	0.209	0.146	0.154	0.218
ptma	0.126	0.221	0.448	0.181	0.158	0.107	0.121	0.222

fra9e	0.126	0.213	0.313	0.18	0.135	0.144	0.157	0.213
snord81	0.125	0.178	0.341	0.133	0.138	0.102	0.11	0.183
snord79	0.125	0.178	0.341	0.133	0.138	0.102	0.11	0.183
bp7	0.125	0.162	0.248	0.132	0.101	0.136	0.141	0.185
lgi2	0.125	0.199	0.289	0.133	0.103	0.122	0.133	0.186
znf473	0.125	0.201	0.4	0.178	0.131	0.141	0.152	0.237
hyt4	0.125	0.162	0.248	0.132	0.101	0.136	0.141	0.185
creb3	0.125	0.178	0.297	0.162	0.192	0.117	0.125	0.184
pabpc1l	0.125	0.216	0.339	0.16	0.115	0.128	0.141	0.206
gimap2	0.125	0.196	0.341	0.206	0.106	0.144	0.154	0.221
elavl2	0.125	0.225	0.399	0.184	0.12	0.133	0.147	0.229
psmd13	0.125	0.219	0.406	0.197	0.104	0.154	0.167	0.25
gls	0.125	0.194	0.335	0.175	0.182	0.127	0.137	0.204
wapal	0.125	0.207	0.35	0.149	0.127	0.157	0.168	0.235
phyhip	0.125	0.148	0.184	0.16	0.135	0.12	0.124	0.151
foxa3	0.125	0.176	0.204	0.123	0.177	0.131	0.139	0.167
cpa4	0.125	0.216	0.362	0.163	0.152	0.128	0.141	0.213
cdh12p2	0.125	0.215	0.326	0.196	0.135	0.139	0.151	0.212
map1lc3c	0.124	0.166	0.355	0.156	0.147	0.123	0.13	0.207
cygb	0.124	0.189	0.332	0.148	0.103	0.125	0.135	0.202
gpat2	0.122	0.166	0.303	0.162	0.161	0.142	0.149	0.208
ofc2	0.122	0.147	0.161	0.107	0.137	0.15	0.153	0.17
dsp	0.122	0.202	0.289	0.139	0.117	0.119	0.13	0.183
esyt3	0.122	0.195	0.328	0.15	0.13	0.133	0.144	0.208
srp9	0.122	0.204	0.448	0.197	0.108	0.136	0.148	0.248
rab6b	0.122	0.175	0.356	0.19	0.12	0.139	0.147	0.222
pcgf6	0.122	0.187	0.307	0.117	0.149	0.115	0.124	0.185
mettl7b	0.122	0.175	0.34	0.188	0.122	0.139	0.147	0.217
eddm3a	0.122	0.205	0.343	0.19	0.194	0.129	0.141	0.209
klhl32	0.123	0.197	0.39	0.224	0.154	0.121	0.132	0.216
tusc5	0.123	0.162	0.186	0.146	0.133	0.119	0.125	0.151
cystm1	0.123	0.168	0.323	0.172	0.107	0.129	0.136	0.202
tgif2ly	0.123	0.193	0.284	0.169	0.25	0.155	0.165	0.213
h2afy2	0.122	0.228	0.432	0.155	0.159	0.126	0.141	0.234
eddm3b	0.122	0.205	0.343	0.19	0.194	0.129	0.141	0.209
samd12	0.122	0.194	0.309	0.149	0.141	0.112	0.122	0.183
ap3b2	0.122	0.168	0.289	0.187	0.107	0.141	0.147	0.202
krt23	0.122	0.144	0.158	0.135	0.119	0.152	0.156	0.172
rnaseh1p2	0.122	0.145	0.175	0.119	0.117	0.131	0.134	0.158
nipbl	0.122	0.185	0.313	0.119	0.163	0.167	0.176	0.233
myl5	0.122	0.19	0.328	0.194	0.172	0.136	0.146	0.21

rnaseh1p3	0.122	0.145	0.175	0.119	0.117	0.131	0.134	0.158
abca17p	0.122	0.214	0.386	0.172	0.182	0.114	0.128	0.209
rprd1a	0.122	0.168	0.258	0.13	0.15	0.11	0.117	0.165
plk5	0.122	0.204	0.318	0.157	0.119	0.131	0.143	0.203
lpal1	0.122	0.172	0.264	0.145	0.208	0.13	0.137	0.185
map1a	0.122	0.195	0.341	0.204	0.117	0.145	0.155	0.222
smarce1	0.122	0.191	0.317	0.123	0.314	0.12	0.13	0.193
kifap3	0.122	0.155	0.228	0.139	0.143	0.163	0.168	0.203
kif4a	0.122	0.192	0.334	0.201	0.109	0.132	0.142	0.209
endog	0.122	0.213	0.337	0.167	0.127	0.124	0.138	0.203
nmnat3	0.122	0.175	0.367	0.129	0.13	0.123	0.131	0.211
retsat	0.122	0.171	0.233	0.178	0.1	0.116	0.123	0.163
pdilt	0.122	0.176	0.29	0.182	0.138	0.108	0.116	0.173
rac3	0.123	0.161	0.211	0.124	0.107	0.124	0.13	0.163
nrxn1	0.123	0.168	0.24	0.159	0.11	0.135	0.141	0.181
uhrf1bp1l	0.123	0.183	0.245	0.153	0.11	0.13	0.139	0.179
thop1	0.123	0.187	0.309	0.21	0.136	0.11	0.12	0.181
hadh	0.123	0.164	0.263	0.146	0.166	0.133	0.139	0.187
ebvs1	0.123	0.208	0.308	0.172	0.147	0.106	0.118	0.177
pomp	0.123	0.196	0.342	0.177	0.111	0.131	0.141	0.21
uggt1	0.123	0.202	0.406	0.181	0.13	0.128	0.139	0.227
ervk-19	0.123	0.199	0.406	0.17	0.111	0.118	0.129	0.218
atg4a	0.123	0.208	0.361	0.16	0.107	0.132	0.144	0.217
ca10	0.123	0.186	0.28	0.147	0.128	0.112	0.121	0.174
rnu1-14p	0.124	0.192	0.339	0.167	0.132	0.139	0.149	0.216
rnps1	0.124	0.209	0.458	0.241	0.122	0.131	0.143	0.246
plk4	0.124	0.205	0.321	0.147	0.12	0.15	0.161	0.22
ssx4	0.124	0.231	0.332	0.186	0.164	0.12	0.136	0.197
bage3	0.124	0.227	0.3	0.127	0.1	0.153	0.167	0.216
uqcrc1	0.124	0.196	0.388	0.204	0.115	0.132	0.143	0.225
mir301b	0.124	0.159	0.174	0.135	0.39	0.133	0.139	0.16
timm9	0.123	0.214	0.43	0.205	0.111	0.128	0.141	0.235
kctd16	0.123	0.171	0.272	0.157	0.111	0.144	0.151	0.2
zfas1	0.123	0.176	0.226	0.123	0.294	0.109	0.117	0.154
cytb	0.123	0.187	0.324	0.185	0.193	0.123	0.132	0.197
rps19	0.123	0.178	0.256	0.167	0.13	0.129	0.137	0.181
fam120c	0.123	0.14	0.167	0.111	0.115	0.117	0.12	0.143
mlycd	0.123	0.166	0.348	0.149	0.177	0.139	0.145	0.219
stard9	0.123	0.211	0.332	0.134	0.127	0.115	0.128	0.193
sgcz	0.123	0.144	0.141	0.139	0.121	0.139	0.142	0.154
snord50a	0.123	0.195	0.368	0.147	0.111	0.12	0.131	0.208

lco	0.123	0.17	0.198	0.189	0.119	0.103	0.11	0.14
atf6b	0.123	0.172	0.293	0.172	0.111	0.109	0.117	0.176
c3orf35	0.123	0.209	0.315	0.176	0.13	0.161	0.173	0.228
ybx2	0.123	0.219	0.341	0.171	0.157	0.12	0.134	0.2
zfp90	0.123	0.165	0.28	0.153	0.142	0.143	0.149	0.201
pex13	0.123	0.184	0.36	0.186	0.133	0.125	0.134	0.21
snord16	0.123	0.188	0.329	0.135	0.109	0.15	0.159	0.223
fam221b	0.126	0.181	0.293	0.171	0.113	0.138	0.146	0.201
srp19	0.126	0.199	0.44	0.22	0.148	0.131	0.141	0.24
hoxc4	0.129	0.183	0.195	0.154	0.103	0.134	0.142	0.167
tead2	0.129	0.191	0.221	0.136	0.167	0.147	0.156	0.186
mob3b	0.129	0.173	0.204	0.134	0.127	0.145	0.152	0.179
supv3l1	0.129	0.216	0.417	0.206	0.123	0.146	0.159	0.247
igkv1d-17	0.129	0.202	0.289	0.166	0.131	0.122	0.133	0.185
tmem37	0.129	0.191	0.276	0.176	0.107	0.149	0.158	0.205
hdac5	0.129	0.167	0.278	0.155	0.205	0.113	0.119	0.174
pkp3	0.129	0.192	0.233	0.11	0.113	0.116	0.125	0.162
gapvd1	0.129	0.146	0.163	0.12	0.115	0.137	0.14	0.159
mrpl39	0.129	0.188	0.4	0.18	0.141	0.126	0.135	0.223
mkln1	0.129	0.185	0.333	0.166	0.11	0.122	0.131	0.199
ggnbp2	0.129	0.205	0.236	0.164	0.117	0.116	0.127	0.163
plscr2	0.129	0.187	0.41	0.268	0.118	0.143	0.151	0.241
dmtn	0.129	0.197	0.33	0.18	0.125	0.136	0.146	0.21
slitrk4	0.129	0.168	0.218	0.112	0.109	0.166	0.171	0.201
dnah9	0.129	0.195	0.293	0.169	0.136	0.164	0.174	0.224
mat2b	0.129	0.201	0.316	0.195	0.1	0.11	0.121	0.183
elavl1	0.129	0.21	0.409	0.18	0.114	0.131	0.143	0.23
spata6	0.128	0.213	0.308	0.197	0.135	0.141	0.153	0.208
tpte2p3	0.128	0.162	0.218	0.106	0.105	0.137	0.142	0.176
mir181a2hg	0.128	0.168	0.286	0.195	0.151	0.113	0.12	0.176
ccdc80	0.128	0.158	0.154	0.106	0.173	0.111	0.116	0.134
acsl1	0.128	0.157	0.284	0.158	0.185	0.121	0.126	0.182
htatsf1	0.128	0.192	0.379	0.214	0.166	0.1	0.11	0.194
rbfox1	0.128	0.187	0.268	0.146	0.143	0.11	0.119	0.168
znf83	0.128	0.216	0.395	0.187	0.278	0.143	0.155	0.237
linc00340	0.128	0.186	0.275	0.124	0.174	0.146	0.155	0.202
gmnn	0.129	0.254	0.382	0.148	0.117	0.154	0.171	0.242
slc25a6	0.129	0.193	0.316	0.258	0.134	0.121	0.13	0.192
rpl19	0.129	0.229	0.269	0.162	0.114	0.126	0.14	0.182
kcnip3	0.129	0.183	0.308	0.204	0.127	0.119	0.127	0.188
art3	0.129	0.188	0.301	0.158	0.152	0.14	0.148	0.204

copz1	0.128	0.207	0.381	0.211	0.107	0.139	0.15	0.229
chaf1b	0.128	0.241	0.453	0.164	0.184	0.131	0.147	0.244
trib1	0.129	0.16	0.221	0.141	0.184	0.131	0.136	0.172
defb105b	0.129	0.211	0.309	0.113	0.114	0.165	0.176	0.229
znf317	0.13	0.212	0.351	0.227	0.24	0.132	0.144	0.213
prac	0.13	0.206	0.267	0.169	0.121	0.174	0.184	0.224
pvrl2	0.13	0.213	0.264	0.162	0.101	0.129	0.141	0.184
lpin1	0.13	0.165	0.266	0.132	0.18	0.134	0.14	0.188
dars2	0.13	0.197	0.357	0.179	0.128	0.144	0.153	0.225
zxdb	0.13	0.176	0.264	0.144	0.112	0.128	0.135	0.182
ervk-9	0.13	0.195	0.403	0.159	0.148	0.126	0.136	0.224
crisp3	0.13	0.232	0.32	0.198	0.181	0.135	0.149	0.206
pet112	0.13	0.207	0.457	0.208	0.166	0.157	0.167	0.268
atg4b	0.13	0.193	0.406	0.154	0.129	0.137	0.146	0.235
hoxb-as3	0.13	0.178	0.211	0.132	0.13	0.154	0.16	0.188
ftx	0.13	0.213	0.319	0.149	0.159	0.139	0.151	0.21
znf807	0.13	0.196	0.313	0.148	0.22	0.137	0.147	0.206
nono	0.13	0.188	0.343	0.202	0.265	0.121	0.13	0.201
lcor	0.13	0.184	0.296	0.157	0.471	0.113	0.122	0.179
klrg2	0.13	0.187	0.238	0.138	0.146	0.132	0.14	0.178
rbm4	0.13	0.202	0.38	0.177	0.115	0.129	0.139	0.219
chl1-as2	0.13	0.169	0.192	0.115	0.102	0.146	0.152	0.176
aldh16a1	0.13	0.163	0.234	0.216	0.105	0.138	0.143	0.182
zfhx4	0.13	0.2	0.262	0.186	0.138	0.14	0.15	0.192
h3f3b	0.13	0.22	0.384	0.17	0.251	0.144	0.157	0.234
tead3	0.13	0.186	0.215	0.134	0.179	0.135	0.144	0.174
slc13a5	0.13	0.173	0.307	0.163	0.131	0.124	0.131	0.192
defb107b	0.129	0.211	0.309	0.113	0.114	0.165	0.176	0.229
defb107a	0.129	0.211	0.309	0.113	0.114	0.165	0.176	0.229
plagl1	0.13	0.203	0.312	0.143	0.203	0.137	0.147	0.205
nhlh2	0.13	0.189	0.185	0.143	0.14	0.114	0.123	0.146
dnph1	0.13	0.205	0.359	0.167	0.113	0.12	0.131	0.204
rfxap	0.13	0.204	0.375	0.176	0.187	0.122	0.133	0.212
lsamp	0.13	0.173	0.224	0.14	0.126	0.159	0.166	0.198
loc100506431	0.13	0.236	0.371	0.161	0.111	0.146	0.161	0.232
usp34	0.13	0.173	0.324	0.156	0.144	0.158	0.164	0.228
elac1	0.13	0.133	0.12	0.118	0.104	0.175	0.175	0.179
paqr7	0.128	0.165	0.188	0.142	0.15	0.112	0.118	0.144
uqcc	0.128	0.223	0.383	0.179	0.107	0.115	0.128	0.208
neurod1	0.127	0.19	0.171	0.106	0.11	0.14	0.149	0.164
hipk2	0.127	0.197	0.385	0.142	0.11	0.126	0.136	0.219

rgs6	0.127	0.161	0.211	0.176	0.111	0.115	0.121	0.154
pck2	0.127	0.157	0.295	0.14	0.217	0.15	0.154	0.212
papss1	0.127	0.18	0.281	0.166	0.183	0.133	0.141	0.193
cidec	0.127	0.169	0.239	0.118	0.18	0.137	0.144	0.183
rfx4	0.127	0.175	0.248	0.156	0.285	0.108	0.115	0.16
tcea2	0.127	0.191	0.389	0.212	0.222	0.148	0.157	0.239
slc31a1	0.127	0.211	0.458	0.203	0.103	0.136	0.148	0.25
ssx6	0.127	0.199	0.312	0.142	0.207	0.133	0.143	0.202
kiaa0907	0.127	0.198	0.399	0.225	0.191	0.145	0.155	0.24
snora70	0.127	0.206	0.422	0.186	0.179	0.134	0.145	0.237
hnrnph3	0.127	0.21	0.414	0.211	0.112	0.142	0.154	0.242
slc37a4	0.127	0.178	0.287	0.151	0.166	0.125	0.133	0.187
pnrc1	0.127	0.155	0.209	0.199	0.447	0.118	0.123	0.157
tmie	0.127	0.162	0.177	0.11	0.101	0.119	0.125	0.147
ofcc1	0.126	0.181	0.221	0.131	0.135	0.143	0.151	0.182
abca12	0.126	0.213	0.32	0.134	0.153	0.125	0.138	0.198
sik1	0.126	0.131	0.179	0.134	0.126	0.132	0.133	0.16
xbp1	0.126	0.176	0.255	0.137	0.102	0.116	0.124	0.17
boll	0.126	0.216	0.346	0.15	0.192	0.142	0.154	0.22
plin4	0.126	0.181	0.286	0.146	0.127	0.135	0.143	0.196
st11	0.126	0.23	0.399	0.186	0.153	0.151	0.166	0.245
pole4	0.126	0.224	0.431	0.229	0.101	0.15	0.164	0.254
slc25a31	0.126	0.196	0.288	0.159	0.133	0.122	0.133	0.185
peg3	0.126	0.18	0.258	0.154	0.162	0.14	0.148	0.191
camta1	0.126	0.209	0.318	0.132	0.169	0.132	0.144	0.203
rorb	0.126	0.157	0.169	0.106	0.148	0.116	0.121	0.142
tef	0.126	0.179	0.273	0.159	0.115	0.117	0.125	0.175
proa	0.126	0.2	0.339	0.214	0.115	0.131	0.141	0.208
mars2	0.126	0.169	0.32	0.135	0.105	0.139	0.145	0.209
atp5a1	0.126	0.177	0.293	0.155	0.156	0.182	0.189	0.24
ndst3	0.126	0.192	0.223	0.134	0.1	0.13	0.14	0.172
exosc1	0.126	0.172	0.337	0.141	0.104	0.101	0.109	0.182
snora65	0.127	0.206	0.422	0.186	0.179	0.134	0.145	0.237
snora71b	0.127	0.206	0.422	0.186	0.179	0.134	0.145	0.237
pir	0.128	0.189	0.305	0.175	0.131	0.109	0.119	0.179
c17orf62	0.128	0.205	0.324	0.168	0.134	0.109	0.12	0.184
c22orf23	0.128	0.19	0.225	0.12	0.102	0.109	0.119	0.154
sult1c4	0.127	0.185	0.267	0.251	0.107	0.129	0.137	0.184
bcat1	0.127	0.195	0.343	0.166	0.155	0.13	0.14	0.209
krt33a	0.127	0.194	0.291	0.137	0.206	0.139	0.148	0.2
srsf2	0.127	0.217	0.445	0.229	0.168	0.128	0.141	0.24

hsbp1	0.128	0.178	0.332	0.147	0.114	0.123	0.13	0.199
med8	0.128	0.155	0.265	0.139	0.124	0.124	0.129	0.18
pou4f1	0.128	0.188	0.173	0.11	0.107	0.149	0.158	0.173
skcg-1	0.128	0.219	0.328	0.18	0.122	0.155	0.168	0.227
dgat2	0.128	0.161	0.27	0.146	0.137	0.128	0.134	0.185
mpp2	0.128	0.168	0.241	0.175	0.102	0.143	0.149	0.189
xdh	0.128	0.181	0.314	0.184	0.101	0.126	0.134	0.196
bbs10	0.128	0.167	0.2	0.125	0.112	0.163	0.168	0.193
slc26a8	0.128	0.201	0.314	0.182	0.121	0.15	0.16	0.218
hibadh	0.127	0.164	0.347	0.157	0.108	0.168	0.173	0.244
adam1a	0.127	0.204	0.254	0.176	0.137	0.131	0.142	0.182
btd	0.127	0.19	0.286	0.126	0.118	0.127	0.136	0.188
defb118	0.127	0.206	0.339	0.177	0.112	0.148	0.159	0.224
eaf1	0.127	0.162	0.267	0.162	0.152	0.144	0.149	0.198
enox2	0.127	0.204	0.382	0.183	0.128	0.126	0.137	0.217
gmpr2	0.127	0.193	0.286	0.166	0.114	0.117	0.127	0.18
snora64	0.127	0.206	0.422	0.186	0.179	0.134	0.145	0.237
snora71a	0.127	0.206	0.422	0.186	0.179	0.134	0.145	0.237
phc2	0.127	0.214	0.37	0.175	0.215	0.129	0.141	0.216
med27	0.127	0.183	0.335	0.161	0.217	0.128	0.136	0.205
rbak	0.127	0.176	0.239	0.156	0.199	0.107	0.115	0.156
eif3g	0.127	0.202	0.399	0.223	0.15	0.133	0.143	0.229
mlx	0.127	0.188	0.382	0.175	0.241	0.117	0.126	0.21
spink8	0.127	0.156	0.18	0.123	0.162	0.103	0.108	0.134
ogdh	0.127	0.205	0.416	0.207	0.172	0.149	0.16	0.249
krt10	0.127	0.204	0.25	0.109	0.122	0.137	0.148	0.186
snai3	0.189	0.276	0.291	0.169	0.117	0.192	0.205	0.242
spink9	0.138	0.192	0.244	0.129	0.109	0.133	0.141	0.18
invs	0.232	0.268	0.264	0.158	0.12	0.289	0.294	0.314
meis2	0.232	0.268	0.222	0.153	0.108	0.299	0.304	0.31
ffar1	0.232	0.268	0.302	0.275	0.18	0.226	0.232	0.27
tbc1d22b	0.199	0.219	0.216	0.156	0.104	0.271	0.274	0.287
pkp4	0.232	0.3	0.358	0.206	0.11	0.233	0.244	0.295
linc00222	0.199	0.219	0.216	0.156	0.104	0.271	0.274	0.287
frmpd1	0.231	0.276	0.385	0.312	0.149	0.239	0.247	0.308
sipa1	0.232	0.338	0.381	0.291	0.114	0.208	0.224	0.279
gpc3	0.232	0.315	0.291	0.179	0.104	0.259	0.271	0.297
mir101-1	0.232	0.324	0.416	0.19	0.248	0.2	0.215	0.283
trnag1	0.199	0.304	0.426	0.229	0.185	0.201	0.217	0.291
hoxd@	0.233	0.271	0.26	0.156	0.113	0.3	0.306	0.323
esrp2	0.233	0.292	0.307	0.169	0.156	0.239	0.248	0.284

mob1a	0.233	0.312	0.411	0.249	0.117	0.231	0.243	0.309
disc2	0.199	0.251	0.334	0.193	0.187	0.196	0.205	0.258
ywhab	0.232	0.289	0.394	0.308	0.139	0.194	0.204	0.271
asap1	0.232	0.282	0.326	0.292	0.103	0.203	0.212	0.258
gatsl3	0.199	0.219	0.216	0.156	0.104	0.271	0.274	0.287
pebp1	0.231	0.31	0.419	0.284	0.144	0.216	0.229	0.299
slc50a1	0.23	0.286	0.289	0.178	0.104	0.233	0.242	0.273
cnot10	0.23	0.34	0.504	0.296	0.233	0.242	0.258	0.348
g3bp1	0.23	0.315	0.524	0.362	0.119	0.231	0.244	0.345
mafip	0.23	0.284	0.377	0.27	0.217	0.236	0.245	0.303
dnm3	0.199	0.28	0.392	0.266	0.15	0.212	0.224	0.29
rabgap1	0.23	0.308	0.453	0.303	0.172	0.226	0.238	0.318
id2	0.23	0.339	0.364	0.226	0.133	0.207	0.224	0.274
dgkk	0.23	0.272	0.329	0.295	0.184	0.226	0.234	0.28
wdr83	0.23	0.294	0.397	0.268	0.142	0.228	0.238	0.302
egfl7	0.231	0.319	0.34	0.18	0.106	0.223	0.237	0.28
slain2	0.231	0.326	0.381	0.215	0.118	0.257	0.271	0.323
tbl3	0.231	0.296	0.374	0.242	0.207	0.228	0.238	0.294
ss18	0.231	0.331	0.435	0.233	0.256	0.228	0.244	0.314
gprc6a	0.23	0.281	0.296	0.221	0.124	0.2	0.209	0.246
hoxb6	0.23	0.291	0.295	0.186	0.188	0.269	0.278	0.307
lats2	0.199	0.278	0.351	0.194	0.116	0.215	0.226	0.279
hopx	0.199	0.297	0.369	0.196	0.113	0.2	0.214	0.272
gpr65	0.199	0.266	0.353	0.278	0.158	0.196	0.207	0.264
mir145	0.234	0.337	0.355	0.187	0.124	0.211	0.227	0.274
rps6ka3	0.234	0.273	0.341	0.305	0.162	0.225	0.232	0.282
cadm2	0.234	0.329	0.364	0.229	0.14	0.232	0.246	0.295
neto2	0.199	0.263	0.338	0.216	0.12	0.203	0.213	0.265
elp6	0.199	0.274	0.343	0.184	0.146	0.191	0.203	0.256
hoxb8	0.233	0.288	0.266	0.174	0.128	0.298	0.306	0.323
pes1	0.233	0.312	0.458	0.213	0.175	0.229	0.241	0.321
irf5	0.234	0.258	0.26	0.197	0.124	0.168	0.174	0.206
mid1	0.199	0.264	0.449	0.214	0.138	0.228	0.238	0.322
hoxd8	0.234	0.28	0.261	0.156	0.113	0.309	0.316	0.331
part1	0.234	0.29	0.309	0.228	0.303	0.217	0.226	0.265
jakmip1	0.235	0.296	0.37	0.268	0.109	0.225	0.235	0.291
wdr65	0.234	0.307	0.461	0.248	0.13	0.227	0.238	0.321
ppp2r2d	0.234	0.266	0.363	0.259	0.102	0.249	0.254	0.309
tom1l2	0.234	0.27	0.333	0.262	0.16	0.238	0.244	0.291
insc	0.234	0.318	0.414	0.237	0.118	0.252	0.265	0.328
bcar4	0.233	0.301	0.313	0.218	0.27	0.202	0.213	0.253

wdr27	0.233	0.283	0.416	0.279	0.121	0.2	0.208	0.283
sox14	0.233	0.287	0.253	0.171	0.139	0.284	0.292	0.306
eri2	0.199	0.274	0.485	0.198	0.141	0.216	0.227	0.322
hoxc9	0.199	0.255	0.263	0.147	0.139	0.247	0.255	0.28
hoxb7	0.233	0.316	0.34	0.236	0.135	0.26	0.273	0.313
traf1	0.233	0.23	0.182	0.255	0.126	0.22	0.221	0.227
mab21l2	0.233	0.27	0.237	0.186	0.149	0.26	0.266	0.28
rgl1	0.199	0.233	0.288	0.25	0.119	0.226	0.232	0.27
hrh4	0.233	0.295	0.31	0.28	0.105	0.211	0.221	0.259
agap1	0.233	0.303	0.436	0.317	0.112	0.245	0.256	0.329
thoc1	0.233	0.348	0.565	0.268	0.165	0.25	0.266	0.373
ier3	0.233	0.337	0.456	0.269	0.149	0.218	0.233	0.311
cpsf3	0.233	0.3	0.444	0.266	0.212	0.263	0.273	0.348
rgma	0.199	0.234	0.196	0.173	0.104	0.188	0.195	0.208
foxj2	0.233	0.333	0.41	0.256	0.179	0.217	0.232	0.296
robo4	0.199	0.275	0.283	0.173	0.162	0.215	0.226	0.258
lpxn	0.233	0.272	0.241	0.277	0.121	0.167	0.175	0.2
snip1	0.23	0.229	0.228	0.144	0.118	0.215	0.217	0.238
bop1	0.23	0.328	0.518	0.233	0.163	0.215	0.23	0.329
crkl	0.225	0.238	0.222	0.307	0.107	0.188	0.192	0.212
znf703	0.225	0.323	0.385	0.213	0.165	0.216	0.231	0.288
traf5	0.225	0.216	0.156	0.249	0.1	0.209	0.209	0.21
ctdnep1	0.225	0.274	0.345	0.229	0.208	0.239	0.247	0.296
tm7sf3	0.225	0.31	0.37	0.293	0.159	0.227	0.24	0.294
izumo1	0.201	0.286	0.358	0.262	0.209	0.193	0.206	0.262
stab1	0.225	0.324	0.414	0.274	0.115	0.208	0.223	0.29
pitrm1	0.225	0.255	0.245	0.168	0.171	0.27	0.275	0.293
slc25a39	0.201	0.288	0.385	0.215	0.113	0.223	0.236	0.297
mcu	0.225	0.272	0.385	0.271	0.103	0.221	0.229	0.293
cers5	0.2	0.27	0.404	0.244	0.11	0.177	0.188	0.262
hbp1	0.2	0.254	0.338	0.165	0.158	0.194	0.202	0.257
mir29a	0.226	0.327	0.403	0.177	0.163	0.227	0.242	0.303
ptpn20b	0.226	0.289	0.337	0.352	0.103	0.238	0.248	0.293
gpr34	0.2	0.265	0.353	0.262	0.118	0.19	0.2	0.258
mir708	0.225	0.298	0.309	0.158	0.119	0.21	0.222	0.26
pde8a	0.226	0.273	0.352	0.29	0.172	0.185	0.193	0.25
lhx8	0.225	0.267	0.215	0.13	0.112	0.268	0.275	0.282
tsc22d3	0.225	0.282	0.347	0.267	0.228	0.216	0.226	0.277
lrrfip1	0.223	0.259	0.338	0.168	0.119	0.201	0.208	0.261
slc7a5	0.223	0.339	0.479	0.252	0.105	0.208	0.225	0.311
prr5l	0.223	0.249	0.35	0.241	0.124	0.175	0.181	0.241

stambpl1	0.201	0.216	0.274	0.227	0.125	0.224	0.227	0.263
emx2os	0.223	0.297	0.384	0.209	0.173	0.246	0.257	0.314
mir146a	0.223	0.262	0.288	0.207	0.118	0.202	0.209	0.246
kif3b	0.223	0.293	0.389	0.246	0.161	0.258	0.268	0.327
lnx1	0.223	0.29	0.408	0.241	0.18	0.234	0.245	0.312
hoxd9	0.224	0.261	0.229	0.137	0.109	0.3	0.305	0.314
myeov	0.224	0.349	0.432	0.241	0.148	0.223	0.241	0.309
rnf41	0.224	0.248	0.292	0.228	0.132	0.238	0.243	0.279
smad4	0.224	0.216	0.137	0.157	0.116	0.257	0.257	0.248
ebag9	0.224	0.364	0.403	0.254	0.108	0.207	0.227	0.286
col4a3bp	0.201	0.289	0.447	0.253	0.126	0.179	0.193	0.278
mir376c	0.224	0.283	0.285	0.151	0.101	0.234	0.243	0.273
ajuba	0.224	0.266	0.322	0.203	0.134	0.232	0.239	0.283
ifrd1	0.226	0.302	0.397	0.242	0.13	0.205	0.218	0.283
supt20h	0.226	0.293	0.485	0.238	0.158	0.236	0.247	0.337
fbxw4p1	0.228	0.298	0.409	0.283	0.158	0.248	0.258	0.324
lace1	0.228	0.3	0.336	0.18	0.14	0.216	0.227	0.273
nuak2	0.229	0.266	0.38	0.203	0.108	0.23	0.236	0.299
ankrd18a	0.2	0.295	0.352	0.174	0.135	0.195	0.209	0.262
wdr34	0.228	0.219	0.17	0.237	0.162	0.218	0.219	0.223
ppp2r1b	0.228	0.301	0.441	0.307	0.119	0.228	0.24	0.316
rcbtb1	0.228	0.322	0.45	0.276	0.345	0.225	0.239	0.317
wsb1	0.229	0.233	0.263	0.253	0.117	0.247	0.249	0.278
gtf2ird1	0.2	0.289	0.411	0.203	0.194	0.203	0.216	0.287
otud1	0.229	0.256	0.227	0.131	0.118	0.263	0.268	0.281
spag16	0.2	0.271	0.38	0.226	0.154	0.235	0.246	0.307
tekt4p2	0.23	0.294	0.425	0.297	0.237	0.212	0.223	0.297
vezf1	0.229	0.287	0.346	0.238	0.157	0.224	0.233	0.282
znf479	0.229	0.322	0.415	0.295	0.215	0.216	0.231	0.298
twist1	0.229	0.286	0.265	0.158	0.142	0.25	0.259	0.281
hoxa5	0.229	0.304	0.326	0.189	0.164	0.268	0.279	0.316
znf641	0.228	0.285	0.414	0.293	0.123	0.228	0.238	0.308
aig1	0.228	0.281	0.344	0.167	0.228	0.268	0.276	0.321
nsun4	0.2	0.291	0.424	0.256	0.163	0.19	0.204	0.28
arfgap3	0.2	0.27	0.419	0.273	0.113	0.205	0.216	0.292
trim23	0.2	0.207	0.301	0.213	0.103	0.24	0.242	0.286
med30	0.226	0.31	0.49	0.253	0.25	0.236	0.249	0.339
stk3	0.226	0.294	0.398	0.316	0.125	0.22	0.231	0.296
dennd1c	0.226	0.284	0.378	0.269	0.109	0.241	0.25	0.308
arhgef26	0.226	0.313	0.435	0.267	0.107	0.243	0.256	0.328
zbtb34	0.226	0.303	0.457	0.235	0.295	0.236	0.248	0.328

arhgap42	0.226	0.272	0.37	0.286	0.101	0.229	0.237	0.295
hemgn	0.2	0.287	0.332	0.266	0.114	0.188	0.201	0.249
slc3a2	0.227	0.332	0.434	0.308	0.142	0.212	0.227	0.299
rpgrip1l	0.228	0.254	0.256	0.181	0.126	0.246	0.251	0.274
gper	0.227	0.278	0.262	0.197	0.319	0.192	0.201	0.228
ywhag	0.2	0.265	0.393	0.281	0.154	0.19	0.2	0.271
mir16-1	0.227	0.34	0.383	0.219	0.105	0.214	0.231	0.286
zic4	0.227	0.268	0.237	0.162	0.107	0.278	0.284	0.297
arhgap8	0.235	0.293	0.358	0.283	0.105	0.227	0.237	0.289
hoxa10	0.235	0.302	0.268	0.212	0.122	0.247	0.257	0.278
strip2	0.243	0.266	0.292	0.209	0.107	0.2	0.206	0.244
catsper2	0.243	0.306	0.354	0.286	0.13	0.232	0.242	0.291
dcaf7	0.243	0.258	0.241	0.209	0.107	0.25	0.254	0.272
foxm1	0.243	0.368	0.431	0.228	0.111	0.242	0.26	0.324
foxf2	0.197	0.262	0.303	0.185	0.136	0.217	0.226	0.266
mir93	0.243	0.362	0.424	0.198	0.114	0.227	0.245	0.309
crispld1	0.243	0.292	0.312	0.191	0.134	0.242	0.25	0.287
arl14ep	0.243	0.328	0.42	0.259	0.137	0.231	0.244	0.311
txnip	0.243	0.312	0.435	0.25	0.145	0.229	0.24	0.313
cep112	0.243	0.324	0.466	0.233	0.178	0.253	0.266	0.345
foxl1	0.243	0.296	0.243	0.132	0.108	0.246	0.255	0.269
cyth1	0.197	0.251	0.306	0.295	0.11	0.185	0.194	0.239
akap6	0.243	0.293	0.41	0.347	0.132	0.22	0.228	0.298
grhl3	0.197	0.249	0.232	0.145	0.142	0.23	0.237	0.255
golm1	0.197	0.288	0.395	0.231	0.122	0.176	0.19	0.259
dap3	0.243	0.333	0.414	0.263	0.11	0.216	0.23	0.296
lmo4	0.243	0.332	0.347	0.226	0.149	0.25	0.263	0.305
rarres2	0.242	0.304	0.289	0.229	0.131	0.224	0.234	0.263
ier2	0.242	0.327	0.469	0.266	0.17	0.225	0.239	0.321
adcy8	0.197	0.229	0.27	0.244	0.106	0.191	0.196	0.233
anxa8l2	0.241	0.329	0.359	0.24	0.127	0.245	0.258	0.304
ksr2	0.241	0.252	0.253	0.258	0.103	0.23	0.233	0.258
ptp4a3	0.241	0.355	0.393	0.289	0.104	0.226	0.244	0.298
hoxa@	0.24	0.298	0.307	0.184	0.114	0.283	0.292	0.322
lppr4	0.24	0.282	0.342	0.208	0.162	0.252	0.259	0.306
ss18l2	0.24	0.311	0.367	0.228	0.123	0.23	0.241	0.294
map3k9	0.241	0.28	0.36	0.338	0.128	0.196	0.203	0.261
cited1	0.241	0.308	0.324	0.202	0.167	0.255	0.265	0.302
phpt1	0.197	0.247	0.371	0.251	0.1	0.2	0.208	0.273
or52z1	0.241	0.323	0.361	0.269	0.16	0.251	0.264	0.311
slk	0.242	0.316	0.418	0.343	0.124	0.223	0.235	0.303

or4e1	0.241	0.323	0.361	0.269	0.16	0.251	0.264	0.311
or10j4	0.241	0.323	0.361	0.269	0.16	0.251	0.264	0.311
pkm	0.241	0.312	0.43	0.24	0.199	0.245	0.256	0.326
or5ac1	0.241	0.323	0.361	0.269	0.16	0.251	0.264	0.311
ccdc41	0.244	0.293	0.384	0.246	0.139	0.269	0.277	0.333
ccdc8	0.244	0.277	0.313	0.163	0.145	0.237	0.243	0.282
h2bfm	0.197	0.246	0.308	0.187	0.109	0.22	0.228	0.271
inhbe	0.247	0.314	0.307	0.253	0.188	0.269	0.279	0.309
plscr1	0.247	0.324	0.441	0.367	0.125	0.204	0.216	0.293
yy1ap1	0.197	0.281	0.367	0.218	0.141	0.18	0.193	0.254
tnfrsf14	0.247	0.331	0.327	0.254	0.131	0.188	0.202	0.244
slc29a3	0.197	0.258	0.326	0.133	0.12	0.189	0.199	0.249
ppp2r3a	0.246	0.325	0.427	0.286	0.171	0.276	0.288	0.353
or1j1	0.247	0.318	0.344	0.193	0.115	0.252	0.263	0.305
diexf	0.247	0.298	0.29	0.211	0.109	0.234	0.243	0.273
psmc3	0.197	0.275	0.493	0.254	0.249	0.192	0.204	0.304
nog	0.248	0.255	0.156	0.107	0.11	0.249	0.252	0.244
zbtb42	0.204	0.272	0.358	0.213	0.152	0.196	0.206	0.264
lrrn1	0.248	0.319	0.348	0.193	0.119	0.28	0.291	0.331
elk1	0.248	0.293	0.374	0.353	0.125	0.197	0.205	0.266
fbxw12	0.197	0.291	0.472	0.28	0.191	0.213	0.227	0.316
fat1	0.247	0.342	0.44	0.219	0.137	0.298	0.311	0.376
il20ra	0.246	0.334	0.372	0.297	0.124	0.21	0.224	0.277
kiaa0226	0.246	0.303	0.474	0.225	0.131	0.243	0.252	0.338
nudt16l1	0.244	0.303	0.453	0.199	0.111	0.266	0.275	0.352
khsrp	0.244	0.303	0.442	0.233	0.134	0.224	0.234	0.312
arap3	0.244	0.289	0.332	0.318	0.148	0.212	0.22	0.266
snhg16	0.244	0.356	0.441	0.249	0.102	0.254	0.27	0.337
tsks	0.244	0.336	0.456	0.31	0.22	0.225	0.24	0.317
nphp1	0.197	0.252	0.313	0.263	0.132	0.215	0.223	0.268
myl10	0.244	0.361	0.388	0.338	0.139	0.219	0.237	0.29
hoxd10	0.244	0.281	0.247	0.145	0.106	0.313	0.319	0.329
abi2	0.197	0.238	0.301	0.204	0.13	0.197	0.203	0.248
gpr27	0.245	0.321	0.451	0.321	0.186	0.252	0.264	0.339
fhl3	0.245	0.304	0.383	0.254	0.221	0.253	0.262	0.318
dync1li2	0.246	0.319	0.437	0.28	0.134	0.241	0.253	0.325
eif2ak4	0.245	0.301	0.403	0.211	0.124	0.191	0.201	0.27
sox4	0.245	0.347	0.381	0.223	0.216	0.24	0.256	0.306
trim33	0.245	0.254	0.223	0.181	0.21	0.268	0.27	0.282
mig7	0.245	0.312	0.307	0.296	0.103	0.209	0.22	0.256
pnma5	0.197	0.295	0.41	0.24	0.102	0.206	0.221	0.29

pnma6d	0.197	0.295	0.41	0.24	0.102	0.206	0.221	0.29
rfx2	0.198	0.292	0.447	0.259	0.205	0.209	0.223	0.304
sgk3	0.236	0.278	0.324	0.242	0.107	0.24	0.247	0.29
gbf1	0.236	0.323	0.49	0.332	0.115	0.237	0.25	0.338
atg14	0.198	0.252	0.405	0.17	0.118	0.181	0.19	0.266
ing2	0.198	0.278	0.5	0.211	0.171	0.182	0.194	0.297
med6	0.198	0.233	0.341	0.198	0.26	0.227	0.232	0.287
tulp2	0.236	0.295	0.356	0.191	0.125	0.239	0.248	0.298
atg7	0.236	0.306	0.492	0.193	0.122	0.239	0.25	0.341
ttc8	0.236	0.31	0.384	0.229	0.126	0.274	0.285	0.338
dsc1	0.198	0.309	0.395	0.208	0.13	0.198	0.215	0.279
wsb2	0.237	0.258	0.2	0.295	0.116	0.239	0.243	0.25
rnf43	0.198	0.275	0.423	0.191	0.116	0.235	0.246	0.32
rplp1	0.198	0.292	0.452	0.29	0.161	0.194	0.208	0.292
ipmk	0.236	0.267	0.295	0.25	0.123	0.259	0.264	0.297
zls	0.198	0.192	0.135	0.103	0.102	0.224	0.224	0.221
gcom2	0.236	0.317	0.361	0.292	0.208	0.22	0.233	0.283
c20orf57	0.236	0.313	0.344	0.379	0.116	0.202	0.214	0.262
rab25	0.236	0.324	0.392	0.237	0.141	0.242	0.255	0.312
siglec14	0.235	0.287	0.264	0.233	0.115	0.179	0.189	0.217
hoxc10	0.235	0.278	0.246	0.162	0.114	0.294	0.3	0.313
uri1	0.199	0.256	0.409	0.255	0.128	0.192	0.201	0.277
cep70	0.235	0.339	0.443	0.249	0.162	0.239	0.255	0.326
jmjd6	0.235	0.337	0.475	0.262	0.138	0.221	0.236	0.32
exoc4	0.235	0.306	0.406	0.29	0.114	0.262	0.273	0.335
rxfp2	0.235	0.28	0.24	0.205	0.185	0.205	0.214	0.233
zbtb48	0.235	0.255	0.21	0.187	0.101	0.279	0.282	0.288
mkks	0.235	0.285	0.36	0.209	0.102	0.271	0.278	0.328
snx18	0.235	0.285	0.368	0.309	0.123	0.273	0.28	0.332
tslp	0.235	0.296	0.215	0.235	0.107	0.164	0.175	0.188
evc2	0.236	0.259	0.262	0.163	0.119	0.267	0.271	0.294
tsc22d4	0.235	0.28	0.276	0.23	0.106	0.24	0.248	0.275
nkx3-1	0.235	0.308	0.341	0.151	0.228	0.23	0.241	0.286
gdf7	0.235	0.263	0.185	0.131	0.142	0.202	0.208	0.213
lrrc48	0.199	0.268	0.324	0.19	0.147	0.223	0.234	0.278
cyth4	0.237	0.3	0.392	0.355	0.131	0.27	0.279	0.337
cmklr1	0.237	0.314	0.326	0.233	0.129	0.215	0.227	0.268
mterfd3	0.239	0.327	0.52	0.27	0.195	0.239	0.253	0.35
lsinct5	0.197	0.29	0.416	0.21	0.151	0.199	0.213	0.286
caprin2	0.239	0.293	0.339	0.21	0.107	0.264	0.272	0.315
agr2	0.239	0.36	0.427	0.233	0.131	0.24	0.258	0.321

slc25a10	0.239	0.315	0.472	0.286	0.14	0.237	0.249	0.333
or2b2	0.239	0.307	0.322	0.354	0.14	0.25	0.261	0.297
pim3	0.239	0.334	0.404	0.26	0.13	0.206	0.221	0.284
anp32d	0.239	0.342	0.444	0.267	0.144	0.238	0.253	0.325
id3	0.239	0.339	0.346	0.227	0.11	0.213	0.228	0.272
dapk3	0.24	0.298	0.415	0.265	0.137	0.226	0.236	0.305
neu4	0.24	0.333	0.407	0.229	0.109	0.22	0.234	0.297
cdh22	0.24	0.3	0.231	0.194	0.114	0.256	0.265	0.274
pom121c	0.24	0.342	0.523	0.282	0.187	0.257	0.272	0.366
loc729316	0.24	0.342	0.523	0.282	0.187	0.257	0.272	0.366
wdr19	0.24	0.314	0.382	0.23	0.125	0.263	0.274	0.328
dnah1	0.24	0.32	0.457	0.277	0.132	0.25	0.262	0.339
hcar2	0.239	0.264	0.289	0.25	0.103	0.223	0.229	0.263
foxd4	0.239	0.283	0.263	0.158	0.157	0.287	0.294	0.312
mir579	0.237	0.325	0.376	0.207	0.203	0.184	0.198	0.256
arl8a	0.237	0.315	0.445	0.228	0.115	0.255	0.267	0.34
znf101	0.198	0.276	0.4	0.253	0.375	0.205	0.217	0.286
nfatc2	0.237	0.32	0.369	0.326	0.119	0.19	0.204	0.259
ackr2	0.198	0.29	0.301	0.226	0.109	0.175	0.189	0.228
cdkl2	0.198	0.231	0.261	0.25	0.105	0.147	0.154	0.192
sigirr	0.237	0.243	0.173	0.177	0.117	0.201	0.204	0.208
rab18	0.238	0.318	0.446	0.275	0.133	0.249	0.261	0.335
btnl3	0.238	0.32	0.329	0.202	0.122	0.216	0.229	0.269
htatip2	0.238	0.351	0.515	0.252	0.15	0.23	0.247	0.34
rab24	0.239	0.324	0.484	0.327	0.109	0.23	0.243	0.33
ceacam6	0.239	0.349	0.364	0.268	0.127	0.233	0.25	0.296
mirlet7a1	0.238	0.358	0.443	0.244	0.15	0.19	0.209	0.282
mir27a	0.238	0.346	0.418	0.222	0.22	0.206	0.222	0.288
ppan	0.198	0.279	0.411	0.226	0.124	0.223	0.235	0.306
nmral1	0.198	0.25	0.395	0.255	0.128	0.196	0.205	0.277
hoxa11	0.223	0.262	0.229	0.143	0.137	0.27	0.276	0.288
styk1	0.223	0.277	0.234	0.24	0.1	0.258	0.266	0.279
ndc1	0.21	0.28	0.45	0.215	0.119	0.217	0.228	0.311
rnu6v	0.21	0.261	0.414	0.279	0.17	0.206	0.214	0.29
btg1	0.21	0.296	0.378	0.275	0.144	0.187	0.2	0.262
stoml2	0.21	0.31	0.411	0.258	0.105	0.205	0.22	0.289
tufm	0.203	0.277	0.48	0.246	0.129	0.187	0.199	0.295
gng5p2	0.21	0.254	0.28	0.267	0.217	0.207	0.215	0.25
znf304	0.21	0.307	0.459	0.324	0.197	0.204	0.219	0.302
eif2b1	0.21	0.268	0.414	0.263	0.125	0.217	0.226	0.3
raph1	0.21	0.279	0.363	0.244	0.117	0.198	0.209	0.267

ik	0.21	0.298	0.407	0.281	0.128	0.188	0.202	0.272
mterfd1	0.21	0.305	0.456	0.29	0.216	0.225	0.239	0.32
znf141	0.203	0.264	0.342	0.191	0.125	0.23	0.239	0.289
rbpjp4	0.21	0.248	0.27	0.115	0.145	0.199	0.205	0.239
btc	0.203	0.264	0.24	0.205	0.112	0.209	0.218	0.239
srsf3	0.21	0.302	0.504	0.286	0.332	0.204	0.218	0.317
dnm2	0.21	0.27	0.374	0.31	0.109	0.225	0.234	0.294
evx2	0.21	0.237	0.221	0.144	0.12	0.273	0.277	0.289
sec24a	0.21	0.277	0.43	0.212	0.113	0.22	0.231	0.308
steap2	0.21	0.294	0.378	0.188	0.317	0.195	0.208	0.269
snora62	0.203	0.281	0.425	0.217	0.161	0.232	0.243	0.317
dcp1a	0.209	0.232	0.274	0.204	0.142	0.243	0.247	0.28
gpr85	0.203	0.254	0.322	0.218	0.122	0.212	0.22	0.267
ofd1	0.203	0.254	0.303	0.162	0.143	0.231	0.239	0.278
hoxb5	0.203	0.249	0.215	0.137	0.121	0.256	0.263	0.273
znf736	0.209	0.29	0.397	0.273	0.177	0.222	0.234	0.299
rpl32	0.203	0.297	0.454	0.242	0.136	0.185	0.199	0.285
tmbim6	0.209	0.28	0.4	0.236	0.1	0.189	0.201	0.271
pi15	0.209	0.284	0.351	0.238	0.171	0.213	0.224	0.277
tpd52l3	0.209	0.287	0.428	0.319	0.292	0.209	0.221	0.297
zfand5	0.209	0.197	0.214	0.21	0.101	0.243	0.242	0.261
stk31	0.209	0.306	0.388	0.228	0.136	0.22	0.234	0.294
btg4	0.209	0.309	0.442	0.222	0.127	0.21	0.224	0.302
arl2	0.209	0.297	0.509	0.292	0.135	0.235	0.248	0.346
arfgef1	0.209	0.285	0.511	0.31	0.125	0.219	0.231	0.332
slc6a16	0.203	0.295	0.49	0.298	0.163	0.212	0.226	0.32
mir198	0.211	0.314	0.356	0.205	0.113	0.203	0.218	0.269
exosc4	0.211	0.278	0.462	0.221	0.165	0.184	0.195	0.285
ttc4	0.202	0.287	0.515	0.306	0.212	0.206	0.218	0.322
c1orf53	0.212	0.318	0.413	0.242	0.135	0.222	0.238	0.304
pax5	0.212	0.297	0.263	0.208	0.112	0.19	0.203	0.229
hint2	0.212	0.29	0.394	0.235	0.152	0.215	0.227	0.291
ceacam1	0.212	0.296	0.303	0.232	0.136	0.204	0.217	0.253
tcof1	0.212	0.278	0.329	0.194	0.156	0.237	0.247	0.291
loc246784	0.202	0.295	0.441	0.245	0.241	0.214	0.228	0.306
gpr139	0.212	0.259	0.305	0.216	0.105	0.222	0.229	0.27
tsg1	0.202	0.207	0.215	0.231	0.134	0.213	0.215	0.235
mrs2	0.212	0.322	0.49	0.252	0.179	0.225	0.241	0.33
gpsm3	0.212	0.229	0.281	0.285	0.102	0.185	0.189	0.229
fis1	0.212	0.301	0.484	0.212	0.109	0.236	0.249	0.338
psmg4	0.212	0.302	0.418	0.265	0.156	0.211	0.224	0.295

ppm1b	0.212	0.268	0.354	0.268	0.14	0.225	0.234	0.288
pitpnc1	0.212	0.273	0.436	0.276	0.104	0.228	0.238	0.317
ophn1	0.202	0.254	0.34	0.2	0.138	0.201	0.21	0.264
ywhah	0.202	0.249	0.362	0.297	0.189	0.186	0.194	0.257
lmo3	0.212	0.282	0.316	0.186	0.133	0.243	0.253	0.292
ifi27l2	0.203	0.271	0.41	0.257	0.13	0.184	0.194	0.27
mir130a	0.203	0.26	0.271	0.192	0.122	0.209	0.218	0.249
ints4	0.211	0.269	0.436	0.267	0.289	0.207	0.216	0.298
znf358	0.211	0.291	0.366	0.189	0.167	0.242	0.254	0.307
ascl1	0.211	0.273	0.222	0.123	0.123	0.22	0.229	0.242
khdrbs3	0.211	0.275	0.398	0.347	0.129	0.238	0.248	0.313
dnm3os	0.211	0.268	0.281	0.146	0.124	0.231	0.24	0.271
foxp4	0.211	0.281	0.292	0.182	0.193	0.197	0.208	0.244
myo10	0.211	0.293	0.379	0.28	0.102	0.234	0.246	0.304
sbf2	0.211	0.273	0.359	0.242	0.108	0.224	0.233	0.289
wash2p	0.212	0.283	0.396	0.27	0.133	0.221	0.232	0.297
gucy2gp	0.212	0.273	0.322	0.273	0.131	0.206	0.216	0.261
pcgem1	0.211	0.297	0.376	0.178	0.129	0.214	0.227	0.285
sult6b1	0.203	0.257	0.319	0.232	0.132	0.2	0.208	0.256
areg	0.211	0.273	0.259	0.2	0.136	0.207	0.217	0.243
tpd52	0.211	0.299	0.375	0.229	0.19	0.186	0.2	0.26
ypel3	0.203	0.294	0.416	0.237	0.142	0.196	0.209	0.282
ccdc88b	0.209	0.285	0.404	0.228	0.15	0.196	0.208	0.278
dusp11	0.206	0.254	0.356	0.301	0.15	0.205	0.212	0.271
smoc1	0.206	0.257	0.248	0.154	0.16	0.236	0.244	0.265
hoxa4	0.206	0.267	0.255	0.156	0.139	0.254	0.263	0.283
rai1	0.206	0.267	0.354	0.198	0.171	0.209	0.219	0.275
ssx2	0.206	0.339	0.446	0.25	0.201	0.203	0.223	0.298
hesx1	0.206	0.236	0.161	0.103	0.181	0.237	0.242	0.239
sptlc2	0.206	0.288	0.448	0.251	0.134	0.192	0.205	0.289
har1b	0.206	0.291	0.375	0.209	0.127	0.193	0.206	0.267
sh3gl2	0.204	0.249	0.369	0.293	0.121	0.23	0.237	0.298
tubg2	0.206	0.281	0.338	0.201	0.114	0.213	0.225	0.273
cd2bp2	0.204	0.283	0.464	0.373	0.171	0.192	0.204	0.293
atf3	0.206	0.269	0.392	0.251	0.127	0.186	0.196	0.266
dlk1	0.206	0.27	0.263	0.152	0.13	0.202	0.212	0.24
znf628	0.206	0.29	0.469	0.305	0.196	0.201	0.214	0.303
dmxl1	0.206	0.276	0.395	0.215	0.16	0.237	0.248	0.312
tm4sf1	0.206	0.283	0.321	0.262	0.141	0.216	0.227	0.27
pik3r4	0.205	0.249	0.326	0.207	0.108	0.217	0.224	0.273
rasgef1b	0.205	0.244	0.284	0.241	0.139	0.212	0.218	0.255

phtf2	0.204	0.287	0.434	0.252	0.181	0.234	0.246	0.322
eda2r	0.204	0.207	0.142	0.158	0.252	0.194	0.196	0.195
tbx2	0.204	0.248	0.224	0.122	0.135	0.201	0.208	0.227
gpr113	0.204	0.284	0.325	0.258	0.153	0.182	0.194	0.241
wdr62	0.204	0.283	0.348	0.205	0.132	0.206	0.218	0.27
mir320c1	0.204	0.284	0.34	0.198	0.156	0.202	0.214	0.264
ccdc91	0.204	0.259	0.41	0.299	0.11	0.218	0.226	0.3
phf15	0.204	0.281	0.367	0.157	0.163	0.244	0.255	0.309
mir20a	0.204	0.328	0.412	0.231	0.123	0.195	0.213	0.28
slc35b3	0.205	0.316	0.37	0.206	0.179	0.225	0.241	0.294
gdf15	0.205	0.298	0.361	0.2	0.102	0.198	0.212	0.267
crim1	0.205	0.284	0.29	0.165	0.103	0.233	0.244	0.276
brox	0.205	0.262	0.428	0.244	0.117	0.209	0.218	0.297
ppp1r3e	0.205	0.259	0.365	0.261	0.188	0.216	0.225	0.284
anxa8	0.204	0.277	0.351	0.213	0.119	0.201	0.212	0.266
psmg1	0.204	0.272	0.35	0.216	0.146	0.196	0.206	0.261
hn1	0.207	0.272	0.274	0.19	0.205	0.21	0.22	0.25
epgn	0.207	0.248	0.236	0.225	0.165	0.225	0.232	0.252
mdfic	0.208	0.283	0.47	0.243	0.154	0.216	0.228	0.317
dip2a	0.208	0.284	0.414	0.207	0.212	0.204	0.215	0.288
kiaa0101	0.208	0.338	0.437	0.241	0.102	0.221	0.24	0.311
catsper1	0.208	0.27	0.324	0.27	0.133	0.19	0.2	0.248
sgsm1	0.203	0.245	0.298	0.239	0.173	0.222	0.229	0.269
mbtd1	0.208	0.281	0.346	0.208	0.138	0.227	0.238	0.287
rab21	0.208	0.268	0.375	0.257	0.125	0.24	0.249	0.308
pabpc4	0.208	0.287	0.477	0.26	0.17	0.216	0.228	0.319
dancr	0.208	0.252	0.267	0.196	0.112	0.223	0.23	0.259
mir520e	0.209	0.228	0.201	0.217	0.109	0.219	0.222	0.235
rep15	0.209	0.285	0.438	0.26	0.101	0.214	0.226	0.305
gng10	0.209	0.27	0.375	0.254	0.114	0.193	0.203	0.266
rln3	0.209	0.287	0.354	0.214	0.156	0.194	0.206	0.261
gulop	0.208	0.265	0.331	0.167	0.126	0.174	0.184	0.236
nphp4	0.208	0.272	0.359	0.222	0.139	0.231	0.24	0.295
arhgap19	0.203	0.287	0.483	0.286	0.138	0.209	0.221	0.315
kiaa0513	0.203	0.268	0.365	0.255	0.169	0.207	0.217	0.277
cnot3	0.208	0.277	0.409	0.214	0.283	0.236	0.247	0.315
ropn1	0.207	0.263	0.355	0.327	0.136	0.19	0.199	0.258
wrap73	0.207	0.25	0.267	0.184	0.131	0.165	0.173	0.208
elmod1	0.208	0.236	0.242	0.2	0.121	0.193	0.198	0.225
ppp1ca	0.207	0.279	0.447	0.301	0.125	0.203	0.214	0.298
rcan2	0.207	0.234	0.287	0.259	0.187	0.211	0.216	0.255

tp53inp2	0.207	0.296	0.399	0.211	0.196	0.214	0.227	0.293
mir133b	0.207	0.28	0.319	0.184	0.12	0.256	0.266	0.305
hsd3b7	0.204	0.259	0.311	0.171	0.135	0.204	0.213	0.257
cnot11	0.208	0.302	0.526	0.286	0.226	0.222	0.235	0.339
tor1aip2	0.204	0.27	0.409	0.192	0.105	0.198	0.209	0.282
mob2	0.208	0.295	0.445	0.249	0.122	0.22	0.233	0.313
usp6	0.208	0.274	0.394	0.241	0.119	0.239	0.249	0.313
ints9	0.208	0.291	0.437	0.242	0.206	0.229	0.241	0.318
slc39a6	0.204	0.276	0.37	0.174	0.159	0.207	0.218	0.277
capg	0.208	0.307	0.483	0.3	0.104	0.215	0.23	0.32
cpsf3l	0.208	0.291	0.437	0.242	0.206	0.229	0.241	0.318
mettl17	0.212	0.293	0.526	0.244	0.103	0.226	0.238	0.343
trnp1	0.213	0.275	0.315	0.176	0.116	0.19	0.2	0.245
diras3	0.219	0.288	0.354	0.327	0.11	0.193	0.204	0.259
cytip	0.219	0.28	0.309	0.273	0.128	0.18	0.19	0.233
tff3	0.219	0.323	0.321	0.241	0.125	0.228	0.244	0.28
tfap2a	0.219	0.278	0.294	0.151	0.143	0.246	0.255	0.287
jakmip3	0.218	0.259	0.265	0.298	0.104	0.216	0.223	0.251
hvbs7	0.218	0.308	0.442	0.219	0.17	0.196	0.21	0.289
vps26a	0.218	0.282	0.43	0.235	0.115	0.228	0.238	0.314
dnah2	0.218	0.26	0.279	0.183	0.132	0.271	0.277	0.305
znf271	0.219	0.317	0.381	0.26	0.178	0.193	0.208	0.267
hoxd1	0.219	0.275	0.248	0.158	0.108	0.286	0.293	0.308
mdfi	0.22	0.259	0.296	0.143	0.118	0.234	0.241	0.277
pid1	0.22	0.258	0.306	0.215	0.105	0.215	0.222	0.264
ephb6	0.22	0.284	0.263	0.233	0.101	0.227	0.237	0.261
mctp1	0.201	0.265	0.427	0.261	0.19	0.208	0.217	0.297
lrrc6	0.22	0.271	0.328	0.183	0.11	0.234	0.242	0.287
mir132	0.219	0.282	0.365	0.245	0.106	0.218	0.228	0.284
nme6	0.22	0.333	0.452	0.245	0.172	0.231	0.248	0.323
ppp4c	0.201	0.264	0.455	0.229	0.103	0.235	0.244	0.329
ddx5	0.218	0.293	0.429	0.198	0.295	0.188	0.2	0.278
tfpt	0.202	0.311	0.454	0.3	0.149	0.206	0.222	0.304
nr2e1	0.218	0.298	0.33	0.131	0.181	0.212	0.224	0.268
tex35	0.202	0.29	0.344	0.268	0.22	0.191	0.204	0.256
elk3	0.218	0.287	0.379	0.279	0.178	0.207	0.218	0.279
mir32	0.218	0.238	0.257	0.19	0.113	0.183	0.188	0.22
zc3h3	0.202	0.193	0.142	0.146	0.143	0.239	0.238	0.235
kiaa0368	0.218	0.297	0.487	0.282	0.157	0.224	0.236	0.328
fam72a	0.201	0.232	0.252	0.217	0.173	0.209	0.214	0.243
tnfaip8	0.201	0.231	0.236	0.248	0.102	0.172	0.177	0.205

chd5	0.218	0.317	0.411	0.169	0.147	0.224	0.239	0.304
snx9	0.218	0.261	0.314	0.28	0.125	0.224	0.231	0.275
rap2a	0.218	0.253	0.315	0.317	0.105	0.233	0.239	0.283
sf3b4	0.218	0.286	0.407	0.223	0.194	0.22	0.231	0.3
fasn	0.218	0.282	0.351	0.2	0.104	0.255	0.265	0.313
arl6	0.218	0.298	0.419	0.269	0.153	0.231	0.243	0.313
tbc1d3f	0.218	0.309	0.463	0.278	0.103	0.25	0.263	0.343
tollip	0.22	0.218	0.201	0.234	0.1	0.213	0.214	0.229
bmp15	0.22	0.274	0.252	0.185	0.139	0.206	0.215	0.238
pag1	0.222	0.258	0.274	0.407	0.124	0.191	0.198	0.232
prrx1	0.222	0.251	0.204	0.122	0.119	0.269	0.274	0.279
inpp5b	0.201	0.252	0.304	0.308	0.119	0.21	0.218	0.26
ankrd13c	0.221	0.288	0.406	0.274	0.11	0.189	0.2	0.271
c19orf59	0.221	0.332	0.445	0.308	0.14	0.212	0.228	0.304
dach1	0.201	0.246	0.262	0.151	0.253	0.211	0.218	0.248
sfn	0.221	0.329	0.442	0.232	0.178	0.231	0.247	0.32
mir1-1	0.222	0.325	0.414	0.19	0.121	0.213	0.228	0.295
c9orf114	0.222	0.245	0.252	0.223	0.136	0.234	0.238	0.263
snx16	0.222	0.263	0.267	0.229	0.123	0.235	0.241	0.268
ccar1	0.223	0.292	0.346	0.242	0.171	0.241	0.252	0.299
actr1b	0.201	0.236	0.277	0.202	0.142	0.228	0.233	0.268
carhsp1	0.201	0.243	0.367	0.253	0.106	0.189	0.196	0.261
id4	0.222	0.336	0.347	0.193	0.15	0.196	0.214	0.26
cdkl3	0.222	0.311	0.387	0.31	0.116	0.2	0.214	0.275
ddit4	0.222	0.258	0.309	0.103	0.122	0.175	0.182	0.228
slc38a2	0.221	0.271	0.356	0.221	0.187	0.205	0.214	0.27
arfgef2	0.221	0.292	0.428	0.281	0.106	0.21	0.221	0.297
slain1	0.22	0.312	0.338	0.183	0.185	0.259	0.272	0.312
znf24	0.221	0.267	0.272	0.141	0.126	0.219	0.226	0.256
c8orf44-sgk3	0.221	0.285	0.377	0.285	0.174	0.22	0.23	0.29
g0s2	0.201	0.28	0.353	0.212	0.185	0.199	0.211	0.266
ppap2c	0.201	0.273	0.421	0.283	0.116	0.213	0.224	0.299
cecr2	0.22	0.252	0.294	0.15	0.156	0.231	0.237	0.274
rnf10	0.201	0.258	0.403	0.251	0.17	0.199	0.208	0.281
stmn3	0.221	0.294	0.374	0.382	0.123	0.219	0.231	0.288
mir122	0.221	0.299	0.444	0.196	0.115	0.189	0.202	0.283
lrrc19	0.221	0.294	0.396	0.238	0.158	0.198	0.209	0.276
sept7	0.201	0.293	0.462	0.236	0.136	0.222	0.235	0.32
amhr2	0.221	0.26	0.19	0.117	0.164	0.199	0.206	0.213
mir610	0.221	0.311	0.342	0.203	0.116	0.196	0.21	0.258
gnb1l	0.221	0.231	0.229	0.111	0.12	0.191	0.194	0.218

foxa1	0.201	0.265	0.267	0.159	0.286	0.203	0.213	0.243
dyrk1a	0.221	0.286	0.389	0.245	0.135	0.224	0.234	0.297
mff	0.217	0.303	0.455	0.212	0.146	0.22	0.233	0.314
mmd2	0.217	0.275	0.318	0.228	0.133	0.189	0.198	0.244
ndnf	0.214	0.274	0.337	0.186	0.109	0.236	0.245	0.292
pkhd1l1	0.214	0.236	0.266	0.164	0.108	0.226	0.23	0.261
mapre3	0.214	0.29	0.388	0.203	0.129	0.253	0.264	0.323
rin1	0.214	0.261	0.296	0.269	0.155	0.218	0.225	0.263
gng8	0.214	0.282	0.292	0.265	0.201	0.208	0.218	0.253
parm1	0.202	0.273	0.321	0.149	0.202	0.209	0.22	0.265
dnajc22	0.214	0.288	0.455	0.234	0.105	0.226	0.237	0.32
nf2	0.215	0.306	0.394	0.246	0.12	0.227	0.241	0.302
serpinb5	0.215	0.351	0.436	0.206	0.13	0.202	0.222	0.293
rc3h2	0.215	0.312	0.349	0.267	0.141	0.189	0.204	0.254
cdv3	0.215	0.319	0.49	0.323	0.152	0.206	0.222	0.313
arf1	0.215	0.288	0.485	0.33	0.102	0.23	0.241	0.333
dclk1	0.215	0.271	0.296	0.173	0.14	0.211	0.22	0.257
phlda1	0.215	0.32	0.438	0.229	0.111	0.216	0.231	0.305
arpc5	0.215	0.296	0.431	0.303	0.206	0.209	0.221	0.297
wash1	0.215	0.277	0.433	0.262	0.112	0.228	0.237	0.315
myo16	0.214	0.293	0.409	0.283	0.112	0.206	0.218	0.288
fam21c	0.214	0.281	0.462	0.284	0.106	0.22	0.23	0.317
akap11	0.213	0.266	0.363	0.302	0.128	0.209	0.217	0.276
rqcd1	0.202	0.245	0.319	0.2	0.225	0.224	0.231	0.277
rnf151	0.213	0.243	0.308	0.283	0.12	0.234	0.239	0.281
akap4	0.213	0.3	0.414	0.322	0.146	0.2	0.213	0.284
sacm1l	0.213	0.265	0.379	0.295	0.103	0.254	0.262	0.322
mlxip	0.202	0.265	0.474	0.236	0.198	0.191	0.201	0.296
kdm6b	0.213	0.297	0.375	0.194	0.205	0.21	0.222	0.281
hoxc13	0.202	0.255	0.246	0.135	0.112	0.226	0.234	0.256
gfi1	0.213	0.309	0.332	0.244	0.146	0.189	0.204	0.249
acadvl	0.202	0.221	0.274	0.215	0.147	0.202	0.206	0.244
npdc1	0.214	0.324	0.406	0.221	0.14	0.193	0.21	0.276
kprp	0.202	0.294	0.31	0.198	0.151	0.209	0.223	0.261
phb	0.214	0.327	0.531	0.257	0.183	0.182	0.199	0.305
rab5b	0.214	0.294	0.416	0.284	0.126	0.223	0.235	0.305
ywhae	0.213	0.271	0.384	0.3	0.173	0.2	0.209	0.275
smarcad1	0.202	0.299	0.427	0.206	0.196	0.203	0.218	0.292
dpcd	0.215	0.257	0.255	0.164	0.127	0.253	0.26	0.282
arhgap11a	0.215	0.232	0.19	0.136	0.265	0.267	0.27	0.274
uvrag	0.217	0.292	0.412	0.185	0.104	0.203	0.215	0.286

ddit4l	0.217	0.252	0.276	0.113	0.128	0.164	0.171	0.209
cobl	0.217	0.267	0.305	0.19	0.117	0.27	0.277	0.312
nfrkb	0.202	0.278	0.41	0.271	0.187	0.215	0.226	0.297
slc25a32	0.202	0.284	0.399	0.227	0.108	0.176	0.188	0.259
arfgap1	0.216	0.288	0.492	0.331	0.117	0.224	0.235	0.329
hoxd3	0.216	0.273	0.26	0.158	0.128	0.281	0.289	0.308
mir616	0.217	0.298	0.329	0.227	0.206	0.179	0.192	0.239
zbtb33	0.217	0.281	0.304	0.161	0.161	0.219	0.229	0.267
defb125	0.217	0.304	0.395	0.245	0.23	0.227	0.24	0.302
hoxb9	0.217	0.277	0.271	0.165	0.139	0.277	0.285	0.307
kiaa1715	0.202	0.249	0.272	0.165	0.157	0.261	0.267	0.295
sprr1a	0.217	0.322	0.391	0.205	0.104	0.229	0.245	0.303
ctnnd2	0.217	0.279	0.332	0.201	0.132	0.209	0.219	0.266
il20rb	0.217	0.276	0.272	0.206	0.113	0.185	0.195	0.227
mir31	0.217	0.326	0.381	0.202	0.119	0.216	0.232	0.288
lrrc14	0.216	0.289	0.395	0.175	0.165	0.24	0.251	0.313
lrrc24	0.216	0.289	0.395	0.175	0.165	0.24	0.251	0.313
snai1p1	0.216	0.292	0.295	0.208	0.152	0.237	0.249	0.28
zic1	0.216	0.253	0.219	0.128	0.121	0.249	0.255	0.267
cdk8	0.216	0.309	0.455	0.259	0.278	0.204	0.218	0.3
zfhx2	0.215	0.301	0.337	0.207	0.215	0.211	0.224	0.27
pdcd6	0.215	0.29	0.484	0.321	0.152	0.213	0.225	0.318
fermt2	0.215	0.287	0.298	0.217	0.107	0.221	0.232	0.267
pcdh11y	0.215	0.261	0.271	0.122	0.144	0.198	0.206	0.238
ubr5	0.216	0.282	0.533	0.234	0.103	0.261	0.27	0.375
ebf1	0.216	0.302	0.252	0.224	0.144	0.185	0.199	0.221
cep44	0.216	0.313	0.48	0.229	0.139	0.237	0.251	0.337
cep128	0.216	0.313	0.48	0.229	0.139	0.237	0.251	0.337
dpf1	0.216	0.3	0.37	0.178	0.13	0.246	0.258	0.311
cep95	0.216	0.313	0.48	0.229	0.139	0.237	0.251	0.337
cep19	0.216	0.313	0.48	0.229	0.139	0.237	0.251	0.337
clic1	0.216	0.321	0.477	0.292	0.122	0.221	0.236	0.322
nr2c2ap	0.216	0.275	0.324	0.237	0.23	0.216	0.225	0.27
inhbc	0.248	0.287	0.264	0.244	0.151	0.249	0.256	0.277
cactin	0.248	0.27	0.321	0.22	0.152	0.222	0.227	0.271
ski	0.306	0.329	0.307	0.219	0.228	0.314	0.319	0.343
relt	0.306	0.391	0.491	0.363	0.112	0.284	0.298	0.374
klf5	0.306	0.392	0.466	0.249	0.205	0.324	0.337	0.401
ppfia3	0.192	0.254	0.305	0.202	0.123	0.19	0.199	0.243
mir155	0.304	0.42	0.462	0.297	0.101	0.271	0.29	0.353
fam53a	0.303	0.343	0.275	0.199	0.133	0.357	0.364	0.371

dnajc27	0.304	0.325	0.355	0.274	0.137	0.276	0.281	0.324
zeb1	0.306	0.411	0.443	0.223	0.175	0.309	0.325	0.38
rfx3	0.306	0.38	0.393	0.251	0.176	0.344	0.356	0.396
gcc2	0.192	0.266	0.43	0.292	0.124	0.191	0.202	0.283
mir200c	0.308	0.418	0.453	0.29	0.141	0.305	0.322	0.38
acvr2a	0.308	0.323	0.237	0.22	0.109	0.306	0.31	0.313
ptp4a1	0.307	0.405	0.483	0.335	0.135	0.293	0.308	0.378
tnip2	0.307	0.317	0.346	0.302	0.156	0.309	0.312	0.35
lmx1b	0.307	0.325	0.233	0.161	0.101	0.382	0.385	0.379
gas6	0.302	0.395	0.419	0.328	0.122	0.269	0.284	0.338
irak2	0.302	0.283	0.198	0.256	0.144	0.271	0.271	0.271
lrrc8c	0.299	0.379	0.447	0.343	0.194	0.283	0.296	0.36
mir1228	0.3	0.374	0.406	0.208	0.123	0.308	0.319	0.368
scyl2	0.3	0.348	0.406	0.311	0.154	0.323	0.331	0.382
arhgap5	0.299	0.35	0.355	0.345	0.137	0.298	0.307	0.344
kank2	0.192	0.199	0.232	0.197	0.247	0.164	0.167	0.198
vangl2	0.299	0.341	0.282	0.16	0.105	0.346	0.353	0.364
usp6nl	0.192	0.237	0.324	0.307	0.115	0.208	0.215	0.266
vps39	0.3	0.326	0.368	0.249	0.164	0.295	0.301	0.345
etv5	0.3	0.362	0.313	0.232	0.131	0.343	0.353	0.371
med19	0.301	0.438	0.544	0.296	0.166	0.273	0.294	0.381
foxf1	0.302	0.36	0.321	0.212	0.12	0.357	0.366	0.385
gbp1	0.301	0.386	0.492	0.353	0.118	0.279	0.293	0.369
dimt1	0.301	0.383	0.5	0.365	0.179	0.301	0.314	0.392
tnip3	0.301	0.294	0.237	0.281	0.101	0.274	0.275	0.286
rln1	0.192	0.266	0.33	0.222	0.133	0.184	0.195	0.246
mir200a	0.308	0.396	0.407	0.257	0.162	0.305	0.319	0.366
sorbs1	0.192	0.224	0.264	0.22	0.144	0.175	0.18	0.217
ptplb	0.192	0.26	0.353	0.227	0.112	0.201	0.211	0.268
crtac1	0.192	0.27	0.276	0.188	0.105	0.203	0.215	0.247
vstm2l	0.312	0.374	0.47	0.328	0.139	0.324	0.334	0.402
nbl1	0.312	0.36	0.357	0.261	0.129	0.35	0.358	0.39
blacat1	0.311	0.437	0.525	0.315	0.189	0.292	0.311	0.39
map4k5	0.311	0.309	0.276	0.373	0.123	0.3	0.302	0.32
foxh1	0.311	0.291	0.193	0.172	0.122	0.324	0.323	0.315
ipcef1	0.312	0.396	0.487	0.383	0.114	0.339	0.352	0.42
dusp7	0.313	0.369	0.403	0.398	0.132	0.265	0.276	0.329
zfyve9	0.313	0.32	0.308	0.248	0.118	0.321	0.324	0.349
ssbp3	0.192	0.25	0.338	0.194	0.146	0.203	0.212	0.265
mir182	0.313	0.378	0.374	0.23	0.142	0.295	0.306	0.346
lamtor2	0.313	0.359	0.446	0.252	0.155	0.285	0.294	0.36

aes	0.313	0.345	0.315	0.173	0.161	0.302	0.309	0.334
efemp1	0.192	0.275	0.34	0.18	0.124	0.196	0.208	0.26
exoc8	0.192	0.247	0.339	0.263	0.111	0.209	0.218	0.271
pdk1	0.311	0.351	0.421	0.282	0.114	0.293	0.301	0.359
pus10	0.192	0.278	0.386	0.205	0.158	0.221	0.233	0.296
skap2	0.309	0.362	0.348	0.49	0.11	0.255	0.265	0.302
pum2	0.192	0.288	0.43	0.221	0.142	0.197	0.211	0.289
hnrnpk	0.192	0.284	0.512	0.318	0.211	0.164	0.178	0.285
nr4a1	0.192	0.255	0.295	0.208	0.326	0.161	0.171	0.215
smurf1	0.308	0.313	0.298	0.201	0.117	0.314	0.317	0.34
map3k7	0.309	0.295	0.243	0.306	0.102	0.313	0.313	0.322
defb106b	0.192	0.287	0.389	0.192	0.172	0.214	0.228	0.291
znf423	0.309	0.34	0.287	0.209	0.249	0.313	0.32	0.336
eif3a	0.31	0.365	0.478	0.273	0.113	0.356	0.365	0.433
mir1226	0.31	0.405	0.464	0.297	0.188	0.285	0.3	0.366
pex11b	0.192	0.286	0.451	0.249	0.151	0.203	0.217	0.3
il1rl1	0.192	0.221	0.166	0.184	0.106	0.159	0.165	0.173
mir17hg	0.192	0.297	0.389	0.16	0.111	0.187	0.203	0.267
fhl1	0.31	0.351	0.408	0.242	0.184	0.279	0.287	0.343
tob2	0.298	0.369	0.457	0.303	0.132	0.299	0.311	0.377
khdrbs1	0.298	0.34	0.405	0.475	0.139	0.314	0.321	0.374
mdk	0.29	0.406	0.433	0.309	0.123	0.289	0.307	0.362
srpk2	0.193	0.271	0.445	0.256	0.142	0.176	0.188	0.275
ventx	0.29	0.376	0.414	0.241	0.136	0.306	0.319	0.37
elf5	0.248	0.327	0.331	0.268	0.176	0.213	0.226	0.267
dynlrb1	0.289	0.317	0.353	0.224	0.145	0.285	0.291	0.333
map3k1	0.288	0.308	0.314	0.339	0.1	0.262	0.267	0.301
adirf	0.288	0.395	0.455	0.268	0.142	0.301	0.317	0.379
ldb2	0.193	0.25	0.288	0.192	0.151	0.224	0.232	0.268
foxg1	0.29	0.33	0.253	0.167	0.117	0.338	0.345	0.349
mipol1	0.193	0.239	0.265	0.158	0.165	0.262	0.269	0.295
mir125b1	0.291	0.415	0.465	0.292	0.13	0.28	0.299	0.364
toc	0.291	0.384	0.388	0.463	0.137	0.263	0.278	0.325
pitx1	0.193	0.22	0.185	0.1	0.122	0.233	0.237	0.244
tle2	0.29	0.339	0.319	0.162	0.156	0.271	0.28	0.31
mknk2	0.29	0.335	0.415	0.317	0.1	0.233	0.241	0.306
eif3b	0.287	0.372	0.546	0.385	0.134	0.274	0.288	0.384
rnf111	0.287	0.303	0.375	0.199	0.108	0.336	0.339	0.385
prdm1	0.285	0.404	0.354	0.286	0.131	0.237	0.256	0.291
mir375	0.285	0.396	0.399	0.236	0.127	0.274	0.291	0.338
ppp2r5b	0.285	0.336	0.404	0.323	0.126	0.32	0.329	0.381

ndrg2	0.285	0.389	0.463	0.275	0.12	0.266	0.282	0.351
hes6	0.285	0.354	0.334	0.164	0.222	0.26	0.272	0.306
tcf4	0.284	0.32	0.272	0.148	0.15	0.269	0.276	0.294
map2k5	0.284	0.328	0.328	0.377	0.111	0.259	0.267	0.303
thumpd3	0.285	0.361	0.385	0.211	0.129	0.289	0.301	0.347
ppp2r5c	0.286	0.366	0.527	0.311	0.105	0.293	0.306	0.395
sh2d3c	0.286	0.351	0.363	0.361	0.137	0.227	0.239	0.285
dgkb	0.287	0.338	0.394	0.308	0.101	0.287	0.295	0.347
asb6	0.286	0.304	0.352	0.339	0.126	0.282	0.286	0.33
prdx4	0.193	0.282	0.459	0.238	0.114	0.186	0.2	0.288
mxd3	0.193	0.23	0.222	0.136	0.111	0.187	0.193	0.215
arhgap22	0.286	0.335	0.403	0.289	0.127	0.282	0.29	0.346
prkd1	0.291	0.327	0.364	0.353	0.124	0.248	0.255	0.303
bbs7	0.291	0.336	0.367	0.235	0.113	0.355	0.361	0.399
znf319	0.193	0.282	0.422	0.264	0.172	0.216	0.229	0.303
bmi1	0.193	0.3	0.356	0.18	0.121	0.192	0.208	0.261
gpr21	0.295	0.375	0.409	0.281	0.169	0.277	0.29	0.343
twist2	0.294	0.344	0.271	0.178	0.109	0.3	0.309	0.32
ptbp2	0.193	0.31	0.54	0.246	0.181	0.187	0.204	0.314
mir143hg	0.294	0.393	0.502	0.28	0.299	0.277	0.293	0.372
ndst4	0.193	0.266	0.362	0.201	0.135	0.197	0.208	0.268
hoxb13	0.295	0.392	0.392	0.226	0.256	0.332	0.347	0.387
spag6	0.193	0.28	0.415	0.245	0.132	0.209	0.222	0.295
tle1	0.297	0.354	0.371	0.16	0.184	0.288	0.298	0.341
mcam	0.297	0.436	0.449	0.31	0.113	0.278	0.299	0.356
ptpn23	0.296	0.361	0.462	0.35	0.135	0.314	0.324	0.392
ecsit	0.296	0.289	0.254	0.279	0.129	0.27	0.271	0.288
flii	0.295	0.353	0.397	0.222	0.21	0.284	0.294	0.345
klf17	0.295	0.429	0.542	0.324	0.202	0.3	0.32	0.404
znf608	0.193	0.267	0.267	0.176	0.13	0.162	0.174	0.207
znf609	0.193	0.267	0.267	0.176	0.13	0.162	0.174	0.207
lrrc16b	0.193	0.295	0.411	0.239	0.15	0.201	0.216	0.286
tax1bp3	0.292	0.341	0.409	0.211	0.153	0.288	0.296	0.352
gpr12	0.292	0.383	0.475	0.343	0.157	0.278	0.292	0.364
fmn1	0.292	0.319	0.304	0.202	0.108	0.361	0.365	0.385
fgf9	0.292	0.312	0.211	0.163	0.116	0.328	0.332	0.327
cxadr	0.193	0.278	0.345	0.228	0.143	0.179	0.192	0.247
zc3h12a	0.292	0.329	0.385	0.281	0.116	0.254	0.261	0.315
fst	0.293	0.334	0.288	0.209	0.145	0.288	0.296	0.315
ift80	0.294	0.323	0.301	0.217	0.105	0.353	0.358	0.377
tle3	0.294	0.352	0.343	0.139	0.158	0.289	0.299	0.333

mir124-1	0.294	0.393	0.4	0.25	0.184	0.296	0.312	0.357
rbm5	0.293	0.4	0.413	0.299	0.11	0.306	0.322	0.37
emilin3	0.193	0.273	0.325	0.172	0.156	0.199	0.211	0.257
tle4	0.293	0.337	0.27	0.146	0.168	0.296	0.304	0.316
ifrd2	0.192	0.29	0.381	0.219	0.174	0.192	0.207	0.27
rraga	0.191	0.207	0.259	0.114	0.108	0.151	0.155	0.195
dok1	0.351	0.408	0.404	0.557	0.13	0.337	0.347	0.388
tsc22d1	0.352	0.4	0.393	0.27	0.208	0.376	0.385	0.42
zeb2	0.353	0.432	0.371	0.232	0.119	0.365	0.378	0.403
wif1	0.351	0.413	0.361	0.193	0.115	0.373	0.383	0.407
trim8	0.35	0.399	0.46	0.41	0.114	0.328	0.337	0.398
mapk7	0.35	0.413	0.453	0.416	0.154	0.315	0.326	0.385
dgkz	0.35	0.409	0.468	0.405	0.136	0.329	0.34	0.402
c3orf17	0.355	0.4	0.307	0.167	0.107	0.324	0.333	0.347
tgfb1i1	0.356	0.413	0.427	0.339	0.29	0.327	0.338	0.387
mir520c	0.364	0.454	0.512	0.294	0.209	0.374	0.389	0.454
cyld	0.364	0.385	0.361	0.304	0.121	0.372	0.377	0.405
tcf7l1	0.364	0.413	0.335	0.181	0.14	0.363	0.372	0.389
acvr1b	0.361	0.366	0.269	0.254	0.103	0.37	0.372	0.374
ovol2	0.36	0.428	0.39	0.197	0.136	0.363	0.374	0.406
miip	0.19	0.286	0.381	0.201	0.101	0.208	0.222	0.284
c1orf85	0.19	0.283	0.472	0.258	0.181	0.179	0.193	0.286
dkk3	0.349	0.41	0.352	0.219	0.1	0.367	0.377	0.399
hdac7	0.19	0.244	0.368	0.201	0.296	0.144	0.153	0.222
vav3	0.343	0.404	0.37	0.392	0.151	0.33	0.341	0.373
hes3	0.344	0.394	0.285	0.137	0.101	0.302	0.312	0.322
mylk2	0.19	0.238	0.285	0.216	0.105	0.145	0.153	0.198
impa2	0.19	0.237	0.314	0.186	0.134	0.185	0.192	0.242
appl2	0.342	0.39	0.449	0.366	0.183	0.322	0.331	0.39
ccdc23	0.342	0.438	0.589	0.356	0.171	0.325	0.34	0.436
stk38	0.19	0.262	0.365	0.232	0.114	0.192	0.203	0.264
spry1	0.344	0.383	0.298	0.271	0.107	0.395	0.401	0.407
vangl1	0.349	0.405	0.398	0.243	0.122	0.385	0.394	0.429
dusp1	0.349	0.408	0.447	0.435	0.173	0.304	0.315	0.373
acvr1	0.349	0.361	0.258	0.249	0.153	0.365	0.369	0.369
pik3ap1	0.348	0.383	0.317	0.358	0.2	0.285	0.293	0.316
map3k3	0.344	0.363	0.35	0.399	0.148	0.34	0.345	0.375
lrig1	0.345	0.439	0.406	0.274	0.165	0.376	0.39	0.424
or2m7	0.19	0.267	0.313	0.195	0.131	0.197	0.209	0.253
atp13a1	0.19	0.274	0.38	0.245	0.152	0.201	0.214	0.277
rnf148	0.19	0.228	0.255	0.121	0.111	0.224	0.23	0.259

pdk2	0.19	0.25	0.436	0.251	0.181	0.178	0.187	0.274
smad6	0.408	0.395	0.283	0.283	0.157	0.42	0.421	0.419
rnf150	0.19	0.228	0.255	0.121	0.111	0.224	0.23	0.259
wnt10b	0.408	0.431	0.303	0.188	0.123	0.434	0.439	0.437
dkk4	0.386	0.387	0.239	0.162	0.112	0.422	0.424	0.409
wnt10a	0.394	0.421	0.301	0.218	0.101	0.43	0.436	0.434
rab9b	0.19	0.263	0.476	0.299	0.107	0.183	0.194	0.291
gid4	0.19	0.26	0.299	0.186	0.137	0.217	0.227	0.266
numbl	0.449	0.5	0.413	0.262	0.126	0.41	0.42	0.446
lef1	0.451	0.463	0.32	0.197	0.11	0.451	0.455	0.453
axin2	0.444	0.457	0.313	0.185	0.107	0.475	0.479	0.473
kank4	0.415	0.434	0.408	0.293	0.141	0.382	0.388	0.423
sorbs3	0.415	0.466	0.481	0.433	0.222	0.371	0.382	0.437
kank3	0.415	0.434	0.408	0.293	0.141	0.382	0.388	0.423
bcl9	0.383	0.415	0.341	0.19	0.101	0.374	0.381	0.398
sfrp4	0.382	0.399	0.285	0.19	0.122	0.408	0.412	0.411
hcar1	0.19	0.232	0.324	0.21	0.159	0.188	0.195	0.248
tnfaip3	0.374	0.401	0.384	0.344	0.154	0.351	0.358	0.393
smad7	0.369	0.366	0.273	0.245	0.115	0.39	0.392	0.393
map3k2	0.368	0.397	0.399	0.478	0.152	0.345	0.352	0.392
map3k8	0.365	0.396	0.354	0.393	0.148	0.333	0.34	0.368
skil	0.366	0.395	0.349	0.273	0.178	0.372	0.379	0.401
ptk6	0.375	0.424	0.385	0.465	0.136	0.336	0.345	0.379
edaradd	0.376	0.383	0.272	0.285	0.106	0.435	0.437	0.431
gas1	0.382	0.438	0.41	0.287	0.114	0.442	0.451	0.481
lphn3	0.19	0.239	0.309	0.216	0.101	0.188	0.196	0.243
sec16a	0.19	0.273	0.396	0.213	0.14	0.215	0.227	0.294
snx21	0.382	0.441	0.482	0.443	0.174	0.402	0.412	0.467
acvr2b	0.379	0.397	0.315	0.267	0.16	0.404	0.408	0.417
kif3a	0.382	0.418	0.36	0.266	0.114	0.427	0.434	0.452
c11orf83	0.191	0.261	0.479	0.234	0.157	0.183	0.194	0.292
tdgf1	0.341	0.405	0.35	0.272	0.136	0.38	0.39	0.411
glyr1	0.323	0.366	0.441	0.361	0.184	0.307	0.315	0.377
htra3	0.191	0.283	0.357	0.188	0.11	0.175	0.189	0.247
ppp1cb	0.191	0.258	0.401	0.237	0.129	0.177	0.187	0.262
pea15	0.322	0.397	0.474	0.378	0.131	0.319	0.331	0.398
tex37	0.191	0.288	0.374	0.263	0.245	0.19	0.204	0.265
mrpl46	0.191	0.273	0.453	0.256	0.145	0.21	0.222	0.308
nedd9	0.321	0.381	0.387	0.376	0.126	0.279	0.29	0.335
polr1d	0.191	0.262	0.377	0.216	0.132	0.2	0.211	0.275
rcan1	0.323	0.391	0.493	0.349	0.176	0.308	0.32	0.394

irak4	0.324	0.307	0.225	0.246	0.132	0.285	0.285	0.289
dusp21	0.325	0.375	0.425	0.465	0.137	0.282	0.292	0.349
ankrd6	0.324	0.36	0.347	0.215	0.104	0.363	0.37	0.397
dnaaf1	0.191	0.24	0.317	0.168	0.116	0.23	0.237	0.282
irak3	0.323	0.323	0.232	0.293	0.128	0.277	0.28	0.285
creb3l4	0.191	0.278	0.443	0.239	0.204	0.189	0.202	0.286
spry2	0.319	0.347	0.301	0.274	0.102	0.374	0.379	0.393
lpar2	0.318	0.374	0.393	0.33	0.113	0.301	0.311	0.357
lgr6	0.315	0.389	0.352	0.201	0.16	0.319	0.331	0.36
ptpro	0.316	0.382	0.353	0.346	0.111	0.28	0.292	0.326
rgs3	0.316	0.353	0.374	0.352	0.131	0.292	0.299	0.343
fam105b	0.315	0.333	0.333	0.177	0.104	0.336	0.34	0.37
bmx	0.314	0.363	0.305	0.364	0.155	0.27	0.28	0.303
sphk2	0.313	0.401	0.431	0.367	0.115	0.297	0.311	0.365
s1pr5	0.314	0.405	0.384	0.363	0.108	0.274	0.289	0.33
tp53i13	0.316	0.426	0.671	0.358	0.185	0.301	0.318	0.443
oser1	0.316	0.4	0.524	0.343	0.113	0.278	0.292	0.378
eif1ax	0.191	0.268	0.44	0.217	0.112	0.179	0.191	0.276
mir508	0.318	0.446	0.507	0.301	0.149	0.293	0.312	0.385
atoh8	0.191	0.233	0.199	0.171	0.144	0.227	0.234	0.244
pagr1	0.191	0.235	0.282	0.132	0.536	0.2	0.207	0.246
gnb2	0.191	0.22	0.275	0.293	0.174	0.176	0.181	0.222
mrps25	0.317	0.399	0.424	0.282	0.115	0.307	0.321	0.372
smad1	0.325	0.317	0.205	0.213	0.141	0.328	0.329	0.321
usp30	0.326	0.425	0.559	0.282	0.123	0.334	0.349	0.437
grem1	0.336	0.345	0.24	0.171	0.107	0.386	0.389	0.382
magt1	0.191	0.276	0.44	0.259	0.123	0.202	0.214	0.297
exoc6b	0.191	0.253	0.37	0.236	0.154	0.221	0.23	0.291
lgr5	0.334	0.416	0.339	0.195	0.171	0.366	0.379	0.396
hey1	0.334	0.355	0.244	0.106	0.12	0.284	0.29	0.294
wnt9b	0.334	0.368	0.276	0.182	0.1	0.374	0.38	0.383
lmo1	0.191	0.26	0.268	0.169	0.109	0.186	0.197	0.229
klf10	0.337	0.397	0.418	0.282	0.177	0.333	0.343	0.391
tax1bp1	0.337	0.348	0.351	0.32	0.147	0.337	0.341	0.374
crtc3	0.191	0.223	0.272	0.205	0.168	0.177	0.183	0.223
pim2	0.341	0.411	0.4	0.305	0.128	0.289	0.301	0.345
zmiz1	0.339	0.352	0.27	0.245	0.232	0.321	0.325	0.334
satb1	0.191	0.294	0.454	0.192	0.173	0.185	0.201	0.286
pygo2	0.338	0.362	0.335	0.168	0.119	0.344	0.349	0.375
tob1	0.338	0.398	0.446	0.335	0.167	0.342	0.353	0.408
etv4	0.334	0.396	0.365	0.24	0.129	0.361	0.371	0.4

pdlim7	0.333	0.366	0.36	0.371	0.142	0.306	0.313	0.349
tgif1	0.331	0.338	0.278	0.221	0.177	0.397	0.399	0.405
cope	0.191	0.275	0.474	0.291	0.142	0.206	0.218	0.311
smad9	0.33	0.333	0.21	0.203	0.157	0.339	0.341	0.332
yap1	0.33	0.42	0.477	0.291	0.148	0.338	0.352	0.414
smad5	0.328	0.332	0.22	0.208	0.15	0.334	0.336	0.331
map4k1	0.329	0.372	0.372	0.439	0.104	0.289	0.298	0.338
clic4	0.331	0.424	0.534	0.364	0.125	0.327	0.341	0.422
trip6	0.331	0.392	0.481	0.437	0.276	0.315	0.326	0.395
ankrd60	0.191	0.277	0.419	0.187	0.151	0.209	0.222	0.297
snai1	0.333	0.42	0.408	0.217	0.115	0.338	0.352	0.392
bnip3	0.191	0.298	0.466	0.233	0.101	0.191	0.206	0.294
ulk2	0.333	0.385	0.508	0.291	0.125	0.319	0.329	0.407
loc100289473	0.191	0.212	0.216	0.195	0.189	0.186	0.19	0.213
tnfrsf12a	0.332	0.432	0.421	0.318	0.149	0.3	0.316	0.363
sla	0.284	0.339	0.331	0.385	0.13	0.243	0.253	0.289
irak1	0.289	0.265	0.193	0.264	0.13	0.277	0.276	0.276
hoxa13	0.26	0.281	0.214	0.161	0.128	0.318	0.322	0.322
rragc	0.189	0.211	0.267	0.11	0.123	0.154	0.159	0.2
snrk	0.195	0.259	0.437	0.253	0.139	0.195	0.205	0.289
tnfsf12	0.259	0.348	0.346	0.256	0.103	0.235	0.25	0.29
sned1	0.259	0.338	0.408	0.241	0.123	0.261	0.273	0.332
bmpr1b	0.259	0.272	0.196	0.173	0.127	0.242	0.246	0.249
golph3	0.259	0.381	0.483	0.293	0.196	0.266	0.284	0.36
nags	0.195	0.238	0.331	0.148	0.15	0.183	0.191	0.246
elk4	0.195	0.273	0.421	0.276	0.192	0.18	0.192	0.271
znf521	0.261	0.3	0.247	0.193	0.159	0.248	0.255	0.27
gdf9	0.262	0.327	0.282	0.229	0.112	0.236	0.247	0.27
becn1p1	0.262	0.334	0.431	0.213	0.123	0.223	0.235	0.306
gadd45b	0.261	0.342	0.428	0.257	0.217	0.25	0.263	0.328
sapcd2	0.261	0.324	0.324	0.156	0.103	0.31	0.319	0.349
spib	0.26	0.343	0.337	0.285	0.141	0.218	0.232	0.272
prdm13	0.261	0.339	0.321	0.203	0.15	0.236	0.249	0.283
rab43	0.195	0.258	0.379	0.261	0.104	0.232	0.241	0.304
mirlet7bhg	0.259	0.371	0.602	0.287	0.232	0.248	0.265	0.38
mark3	0.196	0.223	0.277	0.19	0.113	0.206	0.211	0.249
mir23a	0.196	0.279	0.372	0.207	0.104	0.203	0.216	0.276
palm3	0.196	0.211	0.301	0.248	0.142	0.209	0.212	0.259
ccdc103	0.257	0.323	0.39	0.233	0.216	0.298	0.308	0.36
mir483	0.196	0.205	0.157	0.156	0.111	0.248	0.25	0.249
rb1cc1	0.256	0.324	0.447	0.195	0.128	0.225	0.236	0.312

snx33	0.256	0.339	0.524	0.325	0.129	0.262	0.275	0.37
stk17b	0.257	0.36	0.408	0.289	0.12	0.23	0.246	0.304
eif3i	0.257	0.294	0.312	0.219	0.176	0.256	0.263	0.298
osbpl6	0.196	0.289	0.49	0.261	0.164	0.197	0.21	0.307
thoc3	0.259	0.332	0.514	0.296	0.129	0.28	0.291	0.381
rnf11	0.258	0.275	0.322	0.243	0.13	0.305	0.308	0.344
crtc1	0.257	0.29	0.328	0.191	0.122	0.242	0.248	0.29
map4k4	0.257	0.328	0.34	0.278	0.11	0.261	0.272	0.311
prickle2	0.257	0.308	0.332	0.182	0.11	0.302	0.31	0.345
pyurf	0.262	0.316	0.445	0.34	0.108	0.252	0.261	0.335
dlc1	0.262	0.342	0.4	0.267	0.14	0.239	0.252	0.31
rarres1	0.265	0.323	0.32	0.251	0.113	0.239	0.249	0.284
crlf1	0.284	0.323	0.29	0.397	0.125	0.281	0.288	0.31
rxfp1	0.265	0.33	0.366	0.285	0.173	0.246	0.257	0.306
tssk1a	0.195	0.253	0.415	0.239	0.143	0.18	0.189	0.269
gpr183	0.265	0.341	0.337	0.26	0.108	0.239	0.251	0.29
gbp2	0.195	0.272	0.414	0.287	0.1	0.176	0.188	0.265
med29	0.265	0.363	0.438	0.273	0.144	0.27	0.285	0.349
med15	0.265	0.291	0.386	0.195	0.186	0.278	0.283	0.339
znf354b	0.265	0.358	0.473	0.276	0.216	0.276	0.29	0.365
mtmr1	0.266	0.357	0.472	0.336	0.195	0.275	0.289	0.363
errfi1	0.266	0.321	0.304	0.273	0.156	0.293	0.302	0.327
slamf7	0.266	0.356	0.328	0.316	0.116	0.206	0.22	0.257
bmpr2	0.266	0.281	0.222	0.189	0.12	0.242	0.246	0.257
srms	0.266	0.298	0.275	0.339	0.133	0.25	0.257	0.28
hepn1	0.265	0.369	0.406	0.312	0.134	0.256	0.272	0.326
fam83a	0.266	0.394	0.414	0.27	0.124	0.278	0.297	0.348
gng3	0.264	0.31	0.392	0.353	0.122	0.242	0.251	0.31
ap2a1	0.195	0.236	0.328	0.294	0.122	0.218	0.224	0.275
klf2	0.262	0.347	0.429	0.278	0.126	0.24	0.254	0.32
lphn2	0.262	0.332	0.42	0.338	0.152	0.258	0.269	0.332
stil	0.263	0.37	0.41	0.228	0.132	0.261	0.277	0.332
dusp15	0.262	0.343	0.4	0.339	0.16	0.25	0.263	0.319
trim67	0.195	0.272	0.462	0.243	0.161	0.235	0.246	0.332
mir143	0.195	0.297	0.373	0.15	0.136	0.176	0.192	0.253
mrpl43	0.262	0.379	0.522	0.282	0.175	0.282	0.299	0.386
adcy7	0.195	0.228	0.281	0.296	0.106	0.155	0.161	0.205
csrp1	0.263	0.335	0.366	0.277	0.126	0.277	0.288	0.332
lgr4	0.264	0.314	0.256	0.168	0.103	0.313	0.321	0.33
c2cd3	0.264	0.334	0.338	0.205	0.121	0.295	0.306	0.34
inhba	0.263	0.322	0.274	0.224	0.139	0.263	0.273	0.292

glis2	0.263	0.308	0.331	0.196	0.185	0.281	0.288	0.325
prss50	0.263	0.321	0.37	0.231	0.112	0.244	0.254	0.305
wdr13	0.263	0.374	0.471	0.311	0.178	0.275	0.292	0.364
zfp36	0.256	0.343	0.537	0.304	0.158	0.231	0.245	0.346
ms4a7	0.256	0.364	0.397	0.389	0.117	0.199	0.216	0.274
bmp6	0.25	0.27	0.196	0.155	0.125	0.216	0.221	0.227
ywhaq	0.251	0.3	0.444	0.349	0.184	0.24	0.248	0.325
tbx10	0.196	0.238	0.255	0.154	0.111	0.255	0.261	0.285
klf6	0.25	0.353	0.479	0.227	0.123	0.261	0.276	0.355
mir650	0.25	0.341	0.407	0.177	0.148	0.22	0.235	0.296
kiaa0586	0.25	0.262	0.276	0.15	0.102	0.32	0.322	0.344
mir206	0.25	0.326	0.319	0.185	0.235	0.242	0.254	0.288
hoxc8	0.196	0.242	0.254	0.147	0.149	0.241	0.248	0.273
eps8	0.251	0.304	0.384	0.322	0.125	0.279	0.287	0.341
pde7a	0.251	0.305	0.316	0.232	0.118	0.199	0.208	0.249
foxd2	0.251	0.287	0.254	0.175	0.103	0.314	0.319	0.331
tnip1	0.251	0.274	0.333	0.243	0.164	0.248	0.253	0.298
tifab	0.251	0.236	0.177	0.266	0.11	0.245	0.245	0.247
mir155hg	0.251	0.326	0.402	0.296	0.105	0.226	0.238	0.3
atad3c	0.196	0.29	0.412	0.197	0.15	0.187	0.201	0.274
rnd1	0.251	0.31	0.394	0.302	0.147	0.256	0.265	0.324
stc1	0.196	0.263	0.301	0.215	0.11	0.185	0.196	0.238
zyx	0.25	0.319	0.41	0.274	0.13	0.217	0.228	0.294
traip	0.249	0.246	0.226	0.255	0.119	0.233	0.235	0.252
wdr35	0.249	0.296	0.379	0.244	0.127	0.25	0.258	0.314
neurog2	0.197	0.251	0.203	0.109	0.112	0.212	0.22	0.231
bbs5	0.249	0.313	0.41	0.235	0.148	0.281	0.291	0.351
btg2	0.249	0.365	0.469	0.261	0.185	0.235	0.253	0.329
igbp1	0.248	0.319	0.475	0.36	0.11	0.241	0.252	0.336
pdcd10	0.248	0.325	0.441	0.26	0.121	0.257	0.269	0.34
plxnc1	0.196	0.279	0.25	0.209	0.103	0.197	0.21	0.233
tceal3	0.196	0.256	0.365	0.244	0.138	0.215	0.224	0.285
msi1	0.25	0.367	0.374	0.175	0.128	0.227	0.245	0.292
ehf	0.25	0.358	0.445	0.286	0.185	0.229	0.245	0.316
gmfg	0.249	0.335	0.432	0.348	0.124	0.219	0.233	0.303
fig4	0.249	0.322	0.45	0.293	0.112	0.267	0.278	0.351
stc2	0.249	0.337	0.428	0.249	0.171	0.231	0.245	0.312
bbip1	0.249	0.316	0.472	0.259	0.11	0.277	0.288	0.368
foxc2	0.251	0.302	0.28	0.143	0.146	0.274	0.283	0.305
enah	0.252	0.332	0.428	0.294	0.147	0.256	0.268	0.334
tyro3	0.254	0.319	0.315	0.316	0.103	0.232	0.243	0.278

spin2b	0.196	0.241	0.274	0.183	0.109	0.208	0.215	0.249
rab11fip1	0.254	0.32	0.387	0.323	0.121	0.243	0.254	0.31
cdk2ap2	0.254	0.368	0.443	0.263	0.181	0.302	0.318	0.379
ap1s1	0.196	0.265	0.414	0.266	0.102	0.183	0.194	0.272
map2k4p1	0.196	0.302	0.403	0.22	0.129	0.215	0.23	0.296
dlx4	0.196	0.294	0.308	0.161	0.113	0.207	0.221	0.259
lemd3	0.254	0.272	0.255	0.188	0.11	0.254	0.259	0.279
prrx2	0.254	0.289	0.212	0.139	0.12	0.307	0.312	0.312
c16orf70	0.256	0.296	0.372	0.347	0.153	0.268	0.275	0.327
cir1	0.256	0.287	0.278	0.148	0.262	0.21	0.217	0.247
fhl2	0.256	0.307	0.369	0.224	0.2	0.238	0.247	0.3
snai2	0.255	0.37	0.37	0.206	0.103	0.236	0.253	0.298
crtc2	0.255	0.277	0.338	0.191	0.164	0.227	0.232	0.28
dexi	0.254	0.293	0.305	0.272	0.112	0.223	0.23	0.266
rasgrf1	0.255	0.306	0.39	0.335	0.11	0.324	0.331	0.382
cpz	0.196	0.248	0.259	0.204	0.112	0.201	0.209	0.239
nfatc4	0.253	0.322	0.4	0.3	0.115	0.222	0.234	0.296
wdpcp	0.253	0.293	0.27	0.141	0.137	0.274	0.281	0.301
usp18	0.253	0.302	0.377	0.245	0.1	0.201	0.21	0.27
rictor	0.253	0.269	0.258	0.121	0.1	0.202	0.207	0.234
tpd52l1	0.252	0.329	0.418	0.336	0.213	0.216	0.229	0.296
stk36	0.252	0.262	0.254	0.201	0.168	0.294	0.297	0.314
oxer1	0.196	0.247	0.312	0.239	0.126	0.173	0.181	0.23
tpd52l2	0.252	0.352	0.472	0.348	0.261	0.226	0.242	0.322
metap2	0.196	0.267	0.445	0.245	0.138	0.188	0.199	0.285
bcar3	0.253	0.323	0.36	0.318	0.179	0.209	0.221	0.272
tbx6	0.253	0.279	0.201	0.112	0.102	0.29	0.294	0.293
bmpr1a	0.253	0.262	0.179	0.157	0.13	0.235	0.238	0.238
nr4a3	0.253	0.324	0.347	0.257	0.271	0.242	0.253	0.297
plek	0.253	0.321	0.433	0.38	0.12	0.26	0.271	0.34
bmpr1aps1	0.253	0.277	0.245	0.231	0.144	0.239	0.244	0.263
arfrp1	0.196	0.252	0.355	0.229	0.144	0.206	0.215	0.273
morn4	0.266	0.341	0.401	0.239	0.172	0.29	0.301	0.355
cyth2	0.265	0.319	0.442	0.347	0.103	0.277	0.286	0.356
osbp	0.194	0.25	0.414	0.281	0.104	0.189	0.198	0.277
wbp2	0.194	0.242	0.369	0.23	0.302	0.163	0.171	0.24
stk4	0.273	0.34	0.431	0.291	0.113	0.269	0.279	0.344
vac14	0.273	0.346	0.47	0.298	0.149	0.284	0.295	0.37
sel1l2	0.272	0.341	0.398	0.264	0.118	0.312	0.322	0.372
rcan3as	0.273	0.337	0.43	0.244	0.224	0.246	0.257	0.324
coa5	0.194	0.277	0.531	0.244	0.156	0.191	0.204	0.315

peli1	0.273	0.254	0.224	0.261	0.105	0.282	0.281	0.292
rab23	0.274	0.302	0.251	0.184	0.143	0.352	0.357	0.362
myocd	0.274	0.324	0.355	0.201	0.14	0.24	0.249	0.295
mir200b	0.279	0.391	0.442	0.271	0.188	0.282	0.299	0.359
mtdh	0.273	0.398	0.498	0.252	0.109	0.271	0.29	0.368
hdgf	0.279	0.395	0.495	0.296	0.164	0.29	0.307	0.382
malt1	0.266	0.302	0.312	0.28	0.101	0.269	0.276	0.309
trib3	0.272	0.328	0.405	0.231	0.138	0.257	0.266	0.326
mtfp1	0.28	0.33	0.375	0.256	0.134	0.344	0.351	0.393
rad9b	0.194	0.296	0.449	0.203	0.128	0.226	0.241	0.32
psmb7	0.28	0.338	0.336	0.236	0.125	0.298	0.307	0.34
ptpn3	0.271	0.334	0.395	0.39	0.122	0.232	0.243	0.301
tnik	0.279	0.29	0.288	0.289	0.115	0.277	0.28	0.306
ereg	0.27	0.34	0.322	0.275	0.149	0.261	0.273	0.304
pdzrn3	0.194	0.212	0.203	0.137	0.11	0.171	0.175	0.195
ulk1	0.271	0.293	0.379	0.163	0.118	0.237	0.242	0.3
rapgef1	0.272	0.296	0.293	0.348	0.105	0.243	0.248	0.279
gmnc	0.194	0.304	0.45	0.181	0.129	0.23	0.246	0.325
mtss1	0.272	0.349	0.377	0.269	0.117	0.304	0.315	0.359
heatr6	0.194	0.271	0.369	0.181	0.1	0.182	0.194	0.256
emc4	0.28	0.306	0.355	0.245	0.104	0.288	0.293	0.337
celsr1	0.272	0.319	0.294	0.173	0.115	0.329	0.336	0.355
traf4	0.274	0.282	0.245	0.293	0.109	0.259	0.262	0.278
ahi1	0.274	0.329	0.383	0.253	0.138	0.282	0.291	0.341
med12	0.277	0.299	0.258	0.172	0.159	0.347	0.35	0.359
neurog1	0.194	0.253	0.203	0.122	0.116	0.206	0.215	0.226
eif3c	0.194	0.259	0.28	0.203	0.109	0.167	0.177	0.215
rps6ka6	0.277	0.382	0.51	0.357	0.2	0.273	0.289	0.373
zc3h12c	0.194	0.272	0.401	0.247	0.116	0.202	0.214	0.284
grb10	0.279	0.293	0.273	0.339	0.127	0.259	0.263	0.286
crispld2	0.277	0.326	0.325	0.214	0.176	0.304	0.312	0.343
lamtor3	0.277	0.331	0.38	0.246	0.114	0.25	0.26	0.312
pcdh17	0.278	0.376	0.415	0.242	0.142	0.296	0.311	0.363
smg8	0.278	0.32	0.391	0.226	0.236	0.257	0.265	0.321
rhbdd2	0.278	0.345	0.396	0.353	0.116	0.237	0.248	0.305
ncs1	0.194	0.247	0.342	0.265	0.107	0.195	0.204	0.26
mtmr6	0.277	0.339	0.475	0.37	0.114	0.275	0.285	0.363
znf8	0.277	0.274	0.202	0.176	0.145	0.294	0.295	0.295
exosc5	0.277	0.301	0.315	0.315	0.113	0.246	0.251	0.288
ifnl4	0.275	0.343	0.475	0.31	0.2	0.247	0.258	0.339
nr2c2	0.275	0.3	0.32	0.249	0.26	0.281	0.286	0.32

fblim1	0.275	0.35	0.359	0.247	0.103	0.253	0.265	0.308
peli3	0.275	0.253	0.211	0.282	0.121	0.266	0.265	0.274
rttn	0.274	0.313	0.289	0.186	0.13	0.325	0.331	0.35
mob1b	0.274	0.352	0.43	0.302	0.12	0.254	0.267	0.331
strip1	0.275	0.311	0.357	0.251	0.128	0.235	0.242	0.291
sav1	0.276	0.388	0.557	0.29	0.241	0.28	0.297	0.394
peli2	0.276	0.25	0.189	0.269	0.106	0.277	0.275	0.276
atp5g1	0.194	0.279	0.422	0.274	0.173	0.2	0.213	0.289
dhx33	0.194	0.24	0.299	0.151	0.115	0.178	0.186	0.231
tspan13	0.279	0.359	0.349	0.241	0.105	0.264	0.277	0.314
bcl3	0.279	0.336	0.368	0.301	0.151	0.25	0.26	0.308
nrbp1	0.27	0.332	0.441	0.308	0.17	0.279	0.289	0.357
trappc11	0.27	0.336	0.502	0.209	0.15	0.291	0.301	0.387
sash1	0.282	0.365	0.399	0.311	0.138	0.256	0.269	0.322
pim1	0.268	0.357	0.389	0.295	0.164	0.246	0.26	0.312
meox2	0.268	0.347	0.348	0.216	0.144	0.305	0.317	0.351
rabl3	0.268	0.32	0.323	0.232	0.118	0.278	0.286	0.319
adamts12	0.195	0.273	0.312	0.207	0.149	0.172	0.184	0.229
ppm1l	0.268	0.287	0.305	0.281	0.131	0.244	0.249	0.284
id1	0.282	0.39	0.405	0.252	0.15	0.262	0.279	0.33
loc401131	0.282	0.411	0.584	0.27	0.184	0.281	0.301	0.402
kiaa1033	0.195	0.271	0.47	0.265	0.146	0.231	0.242	0.331
mir1912	0.268	0.346	0.299	0.195	0.1	0.267	0.279	0.302
mir1911	0.268	0.346	0.299	0.195	0.1	0.267	0.279	0.302
mir1913	0.268	0.346	0.299	0.195	0.1	0.267	0.279	0.302
mir1910	0.268	0.346	0.299	0.195	0.1	0.267	0.279	0.302
lrch3	0.267	0.343	0.505	0.244	0.162	0.282	0.294	0.38
arl2bp	0.267	0.369	0.507	0.373	0.143	0.28	0.295	0.378
rab1b	0.195	0.254	0.405	0.244	0.128	0.202	0.211	0.285
zmynd11	0.266	0.291	0.325	0.307	0.169	0.242	0.247	0.288
tcl1a	0.283	0.394	0.417	0.28	0.125	0.244	0.261	0.317
map7d2	0.193	0.282	0.404	0.277	0.131	0.182	0.195	0.267
tab2	0.283	0.268	0.237	0.279	0.121	0.29	0.29	0.302
appl1	0.267	0.304	0.34	0.292	0.126	0.27	0.277	0.318
rdh10	0.267	0.317	0.358	0.205	0.124	0.297	0.305	0.348
dab2	0.282	0.355	0.392	0.309	0.127	0.27	0.282	0.333
ccng2	0.282	0.357	0.403	0.296	0.193	0.265	0.277	0.331
alx3	0.283	0.32	0.269	0.167	0.152	0.339	0.345	0.355
traf6	0.267	0.249	0.192	0.268	0.114	0.257	0.257	0.26
rap1gap	0.283	0.352	0.397	0.348	0.118	0.292	0.303	0.353
rasa4b	0.269	0.298	0.323	0.36	0.102	0.28	0.285	0.321

mir1914	0.268	0.346	0.299	0.195	0.1	0.267	0.279	0.302
rps6ka5	0.28	0.309	0.367	0.337	0.109	0.26	0.266	0.316
mpp1	0.195	0.26	0.363	0.291	0.106	0.204	0.214	0.274
faim	0.269	0.35	0.341	0.266	0.149	0.227	0.24	0.279
tank	0.27	0.265	0.252	0.265	0.111	0.255	0.256	0.277
ppp2r3b	0.193	0.29	0.465	0.303	0.134	0.212	0.226	0.313
zfr	0.195	0.269	0.38	0.215	0.162	0.161	0.172	0.241
psmd11	0.195	0.274	0.483	0.262	0.112	0.222	0.234	0.327
znf384	0.193	0.263	0.307	0.206	0.125	0.146	0.158	0.206
swap70	0.269	0.375	0.373	0.32	0.106	0.226	0.243	0.289
ptpn14	0.269	0.338	0.39	0.291	0.132	0.285	0.296	0.346
isca2	0.269	0.318	0.325	0.347	0.171	0.263	0.271	0.307
tub	0.194	0.24	0.295	0.254	0.119	0.218	0.225	0.265
cyth3	0.281	0.351	0.445	0.366	0.127	0.303	0.314	0.378
hottip	0.281	0.344	0.388	0.218	0.139	0.31	0.32	0.367
fes	0.269	0.323	0.333	0.399	0.115	0.249	0.258	0.297
neurod2	0.194	0.285	0.318	0.186	0.146	0.187	0.2	0.244
tbx23p	0.27	0.329	0.346	0.175	0.106	0.28	0.29	0.328
aebp1	0.269	0.341	0.4	0.278	0.134	0.259	0.271	0.327
pikfyve	0.269	0.337	0.455	0.328	0.122	0.279	0.29	0.362
marcks	0.281	0.285	0.28	0.196	0.11	0.219	0.223	0.253
fstl3	0.281	0.34	0.352	0.27	0.168	0.288	0.298	0.337
ikzf1	0.269	0.36	0.351	0.257	0.126	0.216	0.231	0.273
tec	0.22	0.266	0.161	0.327	0.141	0.167	0.176	0.187
prg4	0.119	0.136	0.168	0.115	0.133	0.105	0.113	0.115
