# Supplementary material for: The RXFP3 receptor is functionally associated with cellular responses to oxidative stress and DNA damage
Source: Aging (Albany NY). 2019 Dec 3;11(23):11268–313. doi: 10.18632/aging.102528 (PMC6932917; doi:10.18632/aging.102528)
Supplement: Supplementary Table 8 [file aging-11-102528-s024..pdf]

Table S8. GIT2 theoretical data set. Biomedical text word items, *i.e.* Gene Symbols, associated with generic 'GIT2' linked extraction terms are represented. All proteins identified possess a Cosine Similarity score of association with the interrogator term of >0.1.

Gene Symbol	GIT2	GIT-2	CAT-2	CAT2	Cat2	Cat-2	Git2	Git-2	ARF GAP GIT2	CAT 2	Cool-interacting tyrosine phosphorylated protein 2	Cool-interacting protein 2	Cool interacting tyrosine phosphorylated protein 2
rab34	0.284	0	0.113	0.139	0.139	0.113	0.284	0	0.29	0.139	0.143	0.142	0.155
dpep3	0.113	0	0.121	0.18	0.18	0.121	0.113	0	0.11	0.18	0.186	0.185	0
cylc1	0.113	0	0	0.118	0.118	0	0.113	0	0.114	0.118	0.146	0.145	0.111
oxsr1	0.16	0	0	0.189	0.189	0	0.16	0	0.158	0.189	0.263	0.263	0.153
ppp1r14b	0.23	0	0	0.146	0.146	0	0.23	0	0.246	0.146	0.18	0.178	0.12
serpinh1p1	0.17	0	0	0.135	0.135	0	0.17	0	0.177	0.135	0.227	0.226	0.142
clvs1	0.229	0	0	0.103	0.103	0	0.229	0	0.227	0.103	0.225	0.224	0.134
pklr	0.233	0	0	0.115	0.115	0	0.233	0	0.235	0.115	0.34	0.339	0.213
rimkla	0.119	0	0	0.147	0.147	0	0.119	0	0.114	0.147	0.155	0.155	0.156
agbl4	0.124	0	0	0.101	0.101	0	0.124	0	0.121	0.101	0.177	0.177	0.118
slc25a24	0.174	0	0	0.132	0.132	0	0.174	0	0.181	0.132	0.177	0.176	0.134
pgm1	0.154	0	0	0.102	0.102	0	0.154	0	0.153	0.102	0.216	0.216	0.136
mrx23	0.153	0	0	0.199	0.199	0	0.153	0	0.147	0.199	0.248	0.248	0.145
piezo1	0.226	0	0	0.146	0.146	0	0.226	0	0.233	0.146	0.337	0.336	0.275
arf3	0.193	0	0	0.107	0.107	0	0.193	0	0.203	0.107	0.121	0.12	0.113
mrx24	0.233	0	0	0.111	0.111	0	0.233	0	0.233	0.111	0.345	0.345	0.206
cpsf6	0.168	0	0	0.111	0.111	0	0.168	0	0.169	0.111	0.199	0.198	0.131
slc17a4	0.119	0	0	0.138	0.138	0	0.119	0	0.114	0.138	0.177	0.177	0.103
svil	0.182	0	0	0.136	0.136	0	0.182	0	0.181	0.136	0.164	0.162	0.124
nudt16l1	0.242	0	0	0.139	0.139	0	0.242	0	0.236	0.139	0.253	0.252	0.201
stk24	0.504	0	0.139	0	0	0.139	0.504	0	0.499	0	0.72	0.719	0.407
nipsnap1	0.154	0.154	0	0.118	0.118	0	0.154	0.154	0	0.118	0	0	0.118
adap2	0.118	0	0.118	0.121	0.121	0.118	0.118	0	0.124	0.121	0	0	0
sntn	0.128	0	0.131	0.12	0.12	0.131	0.128	0	0.129	0.12	0	0	0
trim50	0.105	0	0	0.113	0.113	0	0.105	0	0.115	0.113	0.151	0.15	0
cib4	0.1	0	0	0.126	0.126	0	0.1	0	0.105	0.126	0.123	0.123	0
ap1m2	0.104	0	0	0.121	0.121	0	0.104	0	0.105	0.121	0.112	0.111	0
exoc6b	0.215	0	0	0.117	0.117	0	0.215	0	0.221	0.117	0.116	0.115	0
shank2	0.142	0	0	0.106	0.106	0	0.142	0	0.146	0.106	0.141	0.14	0
alg2	0.118	0	0	0.111	0.111	0	0.118	0	0.123	0.111	0	0	0.118
eea1	0.127	0	0	0.147	0.147	0	0.127	0	0.132	0.147	0	0	0.13
pikfyve	0.176	0	0	0.121	0.121	0	0.176	0	0.182	0.121	0	0	0.108
ocrl	0.121	0	0	0.114	0.114	0	0.121	0	0.129	0.114	0	0	0.136
rab4a	0.171	0	0	0.102	0.102	0	0.171	0	0.174	0.102	0	0	0.152

opa2	0	0	0	0.125	0.125	0	0	0	0.106	0.125	0.177	0.177	0.108
plek	0.201	0	0	0.108	0.108	0	0.201	0	0.201	0.108	0.102	0	0
pld1	0.106	0	0	0.203	0.203	0	0.106	0	0.106	0.203	0.1	0	0
slc2a4rg	0	0	0.145	0.141	0.141	0.145	0	0	0	0.141	0.116	0.115	0
tbc1d25	0.126	0	0	0.112	0.112	0	0.126	0	0.137	0.112	0	0	0.1
sgsm1	0.145	0	0.164	0	0	0.164	0.145	0	0.155	0	0	0	0.145
pki55	0	0	0.137	0.154	0.154	0.137	0	0	0	0.154	0	0	0.147
cygb	0	0	0.193	0.145	0.145	0.193	0	0	0	0.145	0	0	0.103
cdkl2	0	0	0.105	0.107	0.107	0.105	0	0	0	0.107	0	0	0.115
myo10	0.206	0	0	0	0	0	0.206	0	0.213	0	0.118	0.117	0.106
pdpk1	0.206	0	0	0	0	0	0.206	0	0.206	0	0.261	0.259	0.128
arfgap2	0.208	0	0	0	0	0	0.208	0	0.206	0	0.113	0.112	0.115
rrdx	0.208	0	0	0	0	0	0.208	0	0.21	0	0.336	0.336	0.189
raver1	0.209	0	0	0	0	0	0.209	0	0.221	0	0.153	0.152	0.114
wdr44	0.21	0	0	0	0	0	0.21	0	0.211	0	0.157	0.157	0.107
mst4	0.208	0	0	0	0	0	0.208	0	0.204	0	0.283	0.282	0.162
enpp7	0.207	0	0	0	0	0	0.207	0	0.203	0	0.24	0.239	0.15
cap1	0.196	0	0	0	0	0	0.196	0	0.193	0	0.217	0.216	0.159
map4k5	0.191	0	0	0	0	0	0.191	0	0.188	0	0.139	0.138	0.216
spen	0.191	0	0	0	0	0	0.191	0	0.187	0	0.294	0.294	0.167
rpsap12	0.193	0	0	0	0	0	0.193	0	0.194	0	0.282	0.281	0.151
fmnl3	0.19	0	0	0	0	0	0.19	0	0.188	0	0.134	0.133	0.106
dok2	0.189	0	0	0	0	0	0.189	0	0.191	0	0.164	0.162	0.136
ptma	0.187	0	0	0	0	0	0.187	0	0.192	0	0.11	0.11	0.102
sh3yl1	0.188	0	0	0	0	0	0.188	0	0.183	0	0.169	0.168	0.142
tnk2	0.188	0	0	0	0	0	0.188	0	0.189	0	0.383	0.382	0.257
rpsap13	0.193	0	0	0	0	0	0.193	0	0.194	0	0.282	0.281	0.151
arhgef7	0.813	0	0	0	0	0	0.813	0	0.801	0	0.9	0.9	0.55
arpc2	0.201	0	0	0	0	0	0.201	0	0.204	0	0.236	0.235	0.154
swap70	0.204	0	0	0	0	0	0.204	0	0.207	0	0.142	0.14	0.109
ap1s3	0.205	0	0	0	0	0	0.205	0	0.203	0	0.255	0.254	0.171
htra3	0.117	0	0	0	0	0	0.117	0	0.119	0	0.187	0.186	0.116
arhgap31	0.199	0	0	0	0	0	0.199	0	0.199	0	0.159	0.158	0.145
tbc1d3f	0.194	0	0	0	0	0	0.194	0	0.197	0	0.116	0.115	0.127
clns1ap1	0.198	0	0	0	0	0	0.198	0	0.197	0	0.327	0.326	0.178
cib1	0.205	0	0	0	0	0	0.205	0	0.204	0	0.266	0.266	0.153
tjap1	0.217	0	0	0	0	0	0.217	0	0.225	0	0.236	0.236	0.124
hip1	0.237	0	0	0	0	0	0.237	0	0.24	0	0.247	0.246	0.157
fnbp1l	0.113	0	0	0	0	0	0.113	0	0.117	0	0.17	0.17	0.155
zyx	0.238	0	0	0	0	0	0.238	0	0.24	0	0.119	0.118	0.109
abi2	0.238	0	0	0	0	0	0.238	0	0.237	0	0.177	0.176	0.149

srgap2	0.235	0	0	0	0	0	0.235	0	0.234	0	0.129	0.128	0.106
ebp	0.113	0	0	0	0	0	0.113	0	0.11	0	0.23	0.229	0.141
fkbp15	0.227	0	0	0	0	0	0.227	0	0.226	0	0.248	0.247	0.271
agap1	0.228	0	0	0	0	0	0.228	0	0.237	0	0.138	0.137	0.133
cdc42se1	0.23	0	0	0	0	0	0.23	0	0.23	0	0.165	0.164	0.13
sh2d3c	0.231	0	0	0	0	0	0.231	0	0.228	0	0.13	0.128	0.156
scax1	0.239	0	0	0	0	0	0.239	0	0.247	0	0.387	0.387	0.256
macf1	0.239	0	0	0	0	0	0.239	0	0.235	0	0.235	0.234	0.134
rap1gap	0.248	0	0	0	0	0	0.248	0	0.251	0	0.113	0.112	0.138
rsf1	0.248	0	0	0	0	0	0.248	0	0.25	0	0.371	0.37	0.222
copg1	0.249	0	0	0	0	0	0.249	0	0.252	0	0.236	0.235	0.154
enah	0.249	0	0	0	0	0	0.249	0	0.26	0	0.135	0.133	0.108
abi1	0.245	0	0	0	0	0	0.245	0	0.244	0	0.163	0.161	0.115
crkl	0.244	0	0	0	0	0	0.244	0	0.24	0	0.171	0.169	0.471
pkn1	0.24	0	0	0	0	0	0.24	0	0.24	0	0.223	0.221	0.116
arpc1b	0.241	0	0	0	0	0	0.241	0	0.248	0	0.4	0.4	0.257
rapgef1	0.243	0	0	0	0	0	0.243	0	0.241	0	0.15	0.148	0.477
abi3bp	0.227	0	0	0	0	0	0.227	0	0.235	0	0.236	0.235	0.17
exoc3	0.225	0	0	0	0	0	0.225	0	0.226	0	0.249	0.248	0.155
ppm1f	0.214	0	0	0	0	0	0.214	0	0.21	0	0.344	0.343	0.245
dok1	0.216	0	0	0	0	0	0.216	0	0.219	0	0.168	0.164	0.135
bmx	0.218	0	0	0	0	0	0.218	0	0.216	0	0.292	0.29	0.175
rhoj	0.219	0	0	0	0	0	0.219	0	0.217	0	0.344	0.344	0.253
cfl1	0.214	0	0	0	0	0	0.214	0	0.213	0	0.192	0.191	0.109
ankrd52	0.114	0	0	0	0	0	0.114	0	0.114	0	0.111	0.109	0.163
rasl11a	0.212	0	0	0	0	0	0.212	0	0.212	0	0.19	0.189	0.13
prdm10	0.115	0	0	0	0	0	0.115	0	0.115	0	0.172	0.171	0.114
arap3	0.213	0	0	0	0	0	0.213	0	0.217	0	0.124	0.122	0.237
arl10	0.22	0	0	0	0	0	0.22	0	0.219	0	0.221	0.221	0.141
arl9	0.22	0	0	0	0	0	0.22	0	0.219	0	0.221	0.221	0.141
arpc4	0.223	0	0	0	0	0	0.223	0	0.226	0	0.237	0.235	0.157
zcchc13	0.224	0	0	0	0	0	0.224	0	0.217	0	0.289	0.289	0.143
snx27	0.224	0	0	0	0	0	0.224	0	0.227	0	0.239	0.237	0.219
top1	0.223	0	0	0	0	0	0.223	0	0.22	0	0.325	0.325	0.168
wbscr17	0.222	0	0	0	0	0	0.222	0	0.218	0	0.148	0.147	0.132
flna	0.22	0	0	0	0	0	0.22	0	0.218	0	0.227	0.226	0.131
larp4	0.221	0	0	0	0	0	0.221	0	0.219	0	0.328	0.328	0.194
actn1	0.222	0	0	0	0	0	0.222	0	0.223	0	0.129	0.128	0.13
map3k12	0.211	0	0	0	0	0	0.211	0	0.212	0	0.277	0.276	0.178
arfgap3	0.181	0	0	0	0	0	0.181	0	0.186	0	0.116	0.115	0.134
utp23	0.149	0	0	0	0	0	0.149	0	0.147	0	0.143	0.142	0.109

potef	0.149	0	0	0	0	0	0.149	0	0.15	0	0.217	0.216	0.149
nup155	0.151	0	0	0	0	0	0.151	0	0.151	0	0.169	0.169	0.11
mrpl28	0.153	0	0	0	0	0	0.153	0	0.15	0	0.192	0.192	0.107
phpt1	0.125	0	0	0	0	0	0.125	0	0.127	0	0.12	0.118	0.136
grasp	0.147	0	0	0	0	0	0.147	0	0.147	0	0.123	0.122	0.113
rps20	0.145	0	0	0	0	0	0.145	0	0.145	0	0.186	0.186	0.107
igk	0.126	0	0	0	0	0	0.126	0	0.129	0	0.143	0.143	0.144
mrgprx4	0.146	0	0	0	0	0	0.146	0	0.142	0	0.227	0.227	0.306
bcar4	0.146	0	0	0	0	0	0.146	0	0.141	0	0.116	0.115	0.131
rpsap11	0.154	0	0	0	0	0	0.154	0	0.153	0	0.226	0.226	0.121
trmt12	0.154	0	0	0	0	0	0.154	0	0.158	0	0.234	0.234	0.143
arhgap6	0.157	0	0	0	0	0	0.157	0	0.161	0	0.17	0.17	0.122
gtse1	0.158	0	0	0	0	0	0.158	0	0.155	0	0.209	0.209	0.125
ccdc148	0.158	0	0	0	0	0	0.158	0	0.188	0	0.183	0.183	0.15
shd	0.159	0	0	0	0	0	0.159	0	0.164	0	0.131	0.129	0.123
ppfia3	0.157	0	0	0	0	0	0.157	0	0.158	0	0.153	0.152	0.11
dimt1	0.157	0	0	0	0	0	0.157	0	0.165	0	0.131	0.128	0.16
hmgb1	0.154	0	0	0	0	0	0.154	0	0.161	0	0.158	0.157	0.124
ctbp1	0.154	0	0	0	0	0	0.154	0	0.156	0	0.223	0.222	0.157
c16orf70	0.155	0	0	0	0	0	0.155	0	0.158	0	0.121	0.119	0.282
dock6	0.145	0	0	0	0	0	0.145	0	0.145	0	0.131	0.13	0.103
itsn2	0.144	0	0	0	0	0	0.144	0	0.144	0	0.322	0.321	0.246
ppp1r3f	0.135	0	0	0	0	0	0.135	0	0.133	0	0.367	0.367	0.222
bbip1	0.135	0	0	0	0	0	0.135	0	0.151	0	0.106	0.105	0.132
depdc5	0.13	0	0	0	0	0	0.13	0	0.14	0	0.217	0.217	0.113
tmprss13	0.136	0	0	0	0	0	0.136	0	0.133	0	0.187	0.187	0.101
stat5b	0.134	0	0	0	0	0	0.134	0	0.133	0	0.207	0.205	0.174
ankrd44	0.13	0	0	0	0	0	0.13	0	0.13	0	0.115	0.113	0.15
ccdc88b	0.132	0	0	0	0	0	0.132	0	0.14	0	0.167	0.166	0.141
elf3	0.132	0	0	0	0	0	0.132	0	0.131	0	0.207	0.206	0.129
znf81	0.133	0	0	0	0	0	0.133	0	0.134	0	0.323	0.323	0.196
gpr137	0.133	0	0	0	0	0	0.133	0	0.14	0	0.219	0.219	0.137
cdc42ep2	0.136	0	0	0	0	0	0.136	0	0.137	0	0.174	0.174	0.134
ddhd2	0.137	0	0	0	0	0	0.137	0	0.142	0	0.146	0.144	0.12
snx9	0.128	0	0	0	0	0	0.128	0	0.131	0	0.149	0.147	0.111
syn1	0.127	0	0	0	0	0	0.127	0	0.131	0	0.198	0.197	0.132
heatr6	0.142	0	0	0	0	0	0.142	0	0.144	0	0.192	0.191	0.114
dact2	0.126	0	0	0	0	0	0.126	0	0.124	0	0.133	0.133	0.136
gcc2	0.141	0	0	0	0	0	0.141	0	0.149	0	0.104	0.103	0.146
rab9b	0.14	0	0	0	0	0	0.14	0	0.145	0	0.113	0.111	0.114
arfrp1	0.137	0	0	0	0	0	0.137	0	0.142	0	0.116	0.116	0.107

arhgap1	0.139	0	0	0	0	0	0.139	0	0.141	0	0.279	0.279	0.2
fam50a	0.128	0	0	0	0	0	0.128	0	0.135	0	0.195	0.194	0.134
she	0.159	0	0	0	0	0	0.159	0	0.164	0	0.131	0.129	0.123
arf4	0.16	0	0	0	0	0	0.16	0	0.165	0	0.122	0.122	0.117
exoc6	0.177	0	0	0	0	0	0.177	0	0.175	0	0.197	0.197	0.132
rab26	0.178	0	0	0	0	0	0.178	0	0.179	0	0.11	0.109	0.114
iqsec1	0.179	0	0	0	0	0	0.179	0	0.18	0	0.132	0.131	0.109
ap1s2	0.18	0	0	0	0	0	0.18	0	0.186	0	0.195	0.194	0.141
nol10	0.12	0	0	0	0	0	0.12	0	0.123	0	0.205	0.205	0.112
fam101b	0.176	0	0	0	0	0	0.176	0	0.172	0	0.185	0.185	0.102
dynll1	0.175	0	0	0	0	0	0.175	0	0.172	0	0.235	0.234	0.143
sh3pxd2b	0.175	0	0	0	0	0	0.175	0	0.175	0	0.162	0.16	0.164
igfn1	0.176	0	0	0	0	0	0.176	0	0.173	0	0.109	0.108	0.19
gmip	0.176	0	0	0	0	0	0.176	0	0.18	0	0.201	0.201	0.122
pstpip1	0.181	0	0	0	0	0	0.181	0	0.183	0	0.193	0.191	0.22
was	0.25	0	0	0	0	0	0.25	0	0.25	0	0.443	0.442	0.293
ephb1	0.185	0	0	0	0	0	0.185	0	0.185	0	0.139	0.138	0.146
cnksr3	0.185	0	0	0	0	0	0.185	0	0.185	0	0.224	0.224	0.139
eif1ax	0.185	0	0	0	0	0	0.185	0	0.183	0	0.192	0.191	0.111
rap2a	0.186	0	0	0	0	0	0.186	0	0.19	0	0.136	0.135	0.157
pwp1	0.183	0	0	0	0	0	0.183	0	0.18	0	0.17	0.168	0.264
c11orf30	0.183	0	0	0	0	0	0.183	0	0.181	0	0.282	0.282	0.156
gpkow	0.119	0	0	0	0	0	0.119	0	0.116	0	0.296	0.295	0.17
pdcd10	0.182	0	0	0	0	0	0.182	0	0.19	0	0.185	0.184	0.118
capza1	0.119	0	0	0	0	0	0.119	0	0.117	0	0.135	0.134	0.134
tanc2	0.175	0	0	0	0	0	0.175	0	0.175	0	0.208	0.208	0.102
anks1a	0.174	0	0	0	0	0	0.174	0	0.176	0	0.102	0.101	0.468
tmod1	0.161	0	0	0	0	0	0.161	0	0.164	0	0.144	0.143	0.107
ttll4	0.162	0	0	0	0	0	0.162	0	0.158	0	0.221	0.22	0.147
tceal3	0.164	0	0	0	0	0	0.164	0	0.168	0	0.144	0.143	0.201
hmgb1	0.164	0	0	0	0	0	0.164	0	0.162	0	0.222	0.222	0.14
reps2	0.161	0	0	0	0	0	0.161	0	0.165	0	0.111	0.11	0.114
ptpn20a	0.161	0	0	0	0	0	0.161	0	0.165	0	0.139	0.136	0.113
bad	0.16	0	0	0	0	0	0.16	0	0.158	0	0.226	0.225	0.159
cd2ap	0.16	0	0	0	0	0	0.16	0	0.164	0	0.109	0.107	0.479
tnik	0.16	0	0	0	0	0	0.16	0	0.156	0	0.192	0.191	0.175
vps13a	0.165	0	0	0	0	0	0.165	0	0.168	0	0.192	0.192	0.12
ap1s1	0.166	0	0	0	0	0	0.166	0	0.168	0	0.165	0.164	0.118
clasp2	0.172	0	0	0	0	0	0.172	0	0.172	0	0.187	0.186	0.112
limk2	0.172	0	0	0	0	0	0.172	0	0.17	0	0.181	0.18	0.116
ephb6	0.173	0	0	0	0	0	0.173	0	0.175	0	0.101	0.1	0.433

ttll6	0.174	0	0	0	0	0	0.174	0	0.169	0	0.229	0.228	0.158
dennd1b	0.171	0	0	0	0	0	0.171	0	0.175	0	0.116	0.115	0.104
eps8	0.171	0	0	0	0	0	0.171	0	0.176	0	0.126	0.124	0.126
stk4	0.166	0	0	0	0	0	0.166	0	0.165	0	0.167	0.165	0.107
flnc	0.169	0	0	0	0	0	0.169	0	0.17	0	0.158	0.157	0.121
snx33	0.17	0	0	0	0	0	0.17	0	0.171	0	0.191	0.189	0.158
pgam4	0.119	0	0	0	0	0	0.119	0	0.121	0	0.176	0.176	0.127
dolk	0.196	0	0	0	0	0	0.196	0	0.197	0	0.291	0.291	0.176
ctnnbl1	0.548	0	0	0	0	0	0.548	0	0.54	0	0.763	0.763	0.413
stk25	0.322	0	0	0	0	0	0.322	0	0.32	0	0.465	0.464	0.267
wipf2	0.326	0	0	0	0	0	0.326	0	0.328	0	0.394	0.394	0.241
cyth4	0.327	0	0	0	0	0	0.327	0	0.328	0	0.302	0.3	0.176
sh3gl1p2	0.322	0	0	0	0	0	0.322	0	0.321	0	0.349	0.347	0.296
sh3gl1p3	0.322	0	0	0	0	0	0.322	0	0.321	0	0.349	0.347	0.296
parvb	0.561	0	0	0	0	0	0.561	0	0.556	0	0.457	0.456	0.291
arfip1	0.317	0	0	0	0	0	0.317	0	0.322	0	0.283	0.281	0.164
cdhr4	0.32	0	0	0	0	0	0.32	0	0.316	0	0.228	0.227	0.201
sh3gl1p1	0.322	0	0	0	0	0	0.322	0	0.321	0	0.349	0.347	0.296
dnajc27	0.328	0	0	0	0	0	0.328	0	0.325	0	0.377	0.375	0.265
sh3rf3	0.332	0	0	0	0	0	0.332	0	0.331	0	0.206	0.206	0.13
limk1	0.336	0	0	0	0	0	0.336	0	0.33	0	0.382	0.381	0.206
asap2	0.536	0	0	0	0	0	0.536	0	0.534	0	0.514	0.512	0.474
sorbs2	0.534	0	0	0	0	0	0.534	0	0.526	0	0.639	0.637	0.695
rinl	0.343	0	0	0	0	0	0.343	0	0.342	0	0.139	0.138	0.255
wasf1	0.54	0	0	0	0	0	0.54	0	0.536	0	0.49	0.489	0.279
asap1	0.335	0	0	0	0	0	0.335	0	0.337	0	0.271	0.269	0.44
arf1	0.334	0	0	0	0	0	0.334	0	0.351	0	0.178	0.177	0.12
cttn	0.334	0	0	0	0	0	0.334	0	0.342	0	0.254	0.253	0.152
mrx80	0.545	0	0	0	0	0	0.545	0	0.539	0	0.687	0.687	0.369
arf5	0.574	0	0	0	0	0	0.574	0	0.573	0	0.407	0.406	0.303
ankrd28	0.314	0	0	0	0	0	0.314	0	0.31	0	0.263	0.26	0.246
actr2	0.298	0	0	0	0	0	0.298	0	0.3	0	0.306	0.305	0.21
ssh1	0.299	0	0	0	0	0	0.299	0	0.305	0	0.207	0.206	0.109
nck2	0.606	0	0	0	0	0	0.606	0	0.601	0	0.589	0.587	0.325
ptk2b	0.302	0	0	0	0	0	0.302	0	0.3	0	0.183	0.18	0.165
capn5	0.297	0	0	0	0	0	0.297	0	0.293	0	0.397	0.397	0.231
kalrn	0.296	0	0	0	0	0	0.296	0	0.294	0	0.217	0.216	0.121
ptpn12	0.293	0	0	0	0	0	0.293	0	0.292	0	0.197	0.194	0.148
parva	0.609	0	0	0	0	0	0.609	0	0.601	0	0.656	0.654	0.4
lrrc15	0.293	0	0	0	0	0	0.293	0	0.29	0	0.425	0.425	0.288
baiap2	0.296	0	0	0	0	0	0.296	0	0.296	0	0.191	0.19	0.129

rltpr	0.303	0	0	0	0	0	0.303	0	0.308	0	0.122	0.121	0.121
net1	0.304	0	0	0	0	0	0.304	0	0.307	0	0.332	0.331	0.212
sorbs1	0.309	0	0	0	0	0	0.309	0	0.309	0	0.265	0.263	0.521
nck1	0.577	0	0	0	0	0	0.577	0	0.571	0	0.639	0.636	0.447
pxn	0.576	0	0	0	0	0	0.576	0	0.57	0	0.529	0.527	0.372
actr3	0.308	0	0	0	0	0	0.308	0	0.31	0	0.287	0.286	0.192
wasf2	0.588	0	0	0	0	0	0.588	0	0.583	0	0.454	0.452	0.257
arpc3	0.304	0	0	0	0	0	0.304	0	0.312	0	0.278	0.276	0.164
map3k19	0.603	0	0	0	0	0	0.603	0	0.591	0	0.893	0.892	0.482
ipcef1	0.306	0	0	0	0	0	0.306	0	0.308	0	0.252	0.25	0.171
pgam1	0.343	0	0	0	0	0	0.343	0	0.336	0	0.497	0.497	0.278
racgap1	0.344	0	0	0	0	0	0.344	0	0.346	0	0.224	0.223	0.129
tesk2	0.497	0	0	0	0	0	0.497	0	0.49	0	0.545	0.544	0.307
pak4	0.491	0	0	0	0	0	0.491	0	0.486	0	0.666	0.665	0.384
brsk2	0.49	0	0	0	0	0	0.49	0	0.483	0	0.714	0.713	0.379
ajuba	0.489	0	0	0	0	0	0.489	0	0.483	0	0.626	0.625	0.34
rhou	0.497	0	0	0	0	0	0.497	0	0.493	0	0.696	0.695	0.373
tesk1	0.407	0	0	0	0	0	0.407	0	0.404	0	0.468	0.466	0.271
snord15b	0.399	0	0	0	0	0	0.399	0	0.393	0	0.573	0.573	0.319
lims2	0.404	0	0	0	0	0	0.404	0	0.402	0	0.447	0.447	0.244
lmod2	0.406	0	0	0	0	0	0.406	0	0.401	0	0.528	0.528	0.301
elmod3	0.504	0	0	0	0	0	0.504	0	0.506	0	0.194	0.194	0.116
ppm1e	0.411	0	0	0	0	0	0.411	0	0.405	0	0.584	0.582	0.327
arhgap8	0.412	0	0	0	0	0	0.412	0	0.411	0	0.452	0.451	0.302
cdk11a	0.421	0	0	0	0	0	0.421	0	0.415	0	0.599	0.598	0.364
cdc42bpa	0.422	0	0	0	0	0	0.422	0	0.417	0	0.558	0.558	0.344
cdk11b	0.427	0	0	0	0	0	0.427	0	0.421	0	0.602	0.601	0.418
arf6	0.442	0	0	0	0	0	0.442	0	0.443	0	0.26	0.259	0.164
ilk	0.468	0	0	0	0	0	0.468	0	0.463	0	0.49	0.488	0.275
tbcb	0.471	0	0	0	0	0	0.471	0	0.464	0	0.662	0.661	0.373
abi3	0.487	0	0	0	0	0	0.487	0	0.482	0	0.464	0.462	0.286
tgfb1i1	0.412	0	0	0	0	0	0.412	0	0.409	0	0.377	0.376	0.259
lims1	0.475	0	0	0	0	0	0.475	0	0.471	0	0.482	0.48	0.291
ssh2	0.396	0	0	0	0	0	0.396	0	0.398	0	0.206	0.205	0.109
synj2	0.394	0	0	0	0	0	0.394	0	0.388	0	0.318	0.317	0.266
crk	0.359	0	0	0	0	0	0.359	0	0.356	0	0.217	0.214	0.422
lurap1	0.359	0	0	0	0	0	0.359	0	0.358	0	0.45	0.45	0.278
mrx31	0.528	0	0	0	0	0	0.528	0	0.523	0	0.801	0.801	0.448
snord15a	0.523	0	0	0	0	0	0.523	0	0.513	0	0.76	0.759	0.413
arhgap30	0.529	0	0	0	0	0	0.529	0	0.523	0	0.456	0.455	0.241
vcl	0.358	0	0	0	0	0	0.358	0	0.366	0	0.261	0.259	0.223

ppfia1	0.346	0	0	0	0	0	0.346	0	0.342	0	0.419	0.418	0.237
ssh3	0.348	0	0	0	0	0	0.348	0	0.355	0	0.305	0.304	0.156
bcar1	0.35	0	0	0	0	0	0.35	0	0.346	0	0.194	0.192	0.229
arhgap27	0.356	0	0	0	0	0	0.356	0	0.352	0	0.335	0.335	0.232
cdc42bpb	0.361	0	0	0	0	0	0.361	0	0.358	0	0.591	0.591	0.354
rsl24d1	0.365	0	0	0	0	0	0.365	0	0.367	0	0.49	0.49	0.281
mink1	0.377	0	0	0	0	0	0.377	0	0.372	0	0.513	0.512	0.303
hepacam2	0.519	0	0	0	0	0	0.519	0	0.512	0	0.745	0.744	0.444
pak7	0.385	0	0	0	0	0	0.385	0	0.383	0	0.594	0.593	0.383
pak6	0.385	0	0	0	0	0	0.385	0	0.38	0	0.549	0.549	0.309
ccdc53	0.106	0	0	0	0	0	0.106	0	0.107	0	0.104	0.102	0.12
parvg	0.375	0	0	0	0	0	0.375	0	0.37	0	0.37	0.369	0.221
myo18a	0.37	0	0	0	0	0	0.37	0	0.372	0	0.17	0.169	0.117
map4k4	0.37	0	0	0	0	0	0.37	0	0.363	0	0.498	0.497	0.311
rtkn	0.37	0	0	0	0	0	0.37	0	0.367	0	0.497	0.496	0.277
cyth2	0.291	0	0	0	0	0	0.291	0	0.294	0	0.178	0.176	0.116
bcar3	0.317	0	0	0	0	0	0.317	0	0.311	0	0.187	0.185	0.231
cripak	0.623	0	0	0	0	0	0.623	0	0.611	0	0.909	0.909	0.487
pitpnm3	0.279	0	0	0	0	0	0.279	0	0.278	0	0.214	0.213	0.188
lpxn	0.278	0	0	0	0	0	0.278	0	0.274	0	0.197	0.195	0.158
mtss1	0.265	0	0	0	0	0	0.265	0	0.268	0	0.16	0.159	0.12
git1	0.644	0	0	0	0	0	0.644	0	0.639	0	0.703	0.702	0.411
cald1	0.266	0	0	0	0	0	0.266	0	0.27	0	0.386	0.384	0.217
exoc1	0.257	0	0	0	0	0	0.257	0	0.257	0	0.35	0.35	0.218
arhgap15	0.666	0	0	0	0	0	0.666	0	0.66	0	0.702	0.702	0.392
sorbs3	0.263	0	0	0	0	0	0.263	0	0.271	0	0.198	0.195	0.263
dbnl	0.282	0	0	0	0	0	0.282	0	0.281	0	0.234	0.232	0.258
arhgap32	0.257	0	0	0	0	0	0.257	0	0.26	0	0.192	0.191	0.136
elmo1	0.623	0	0	0	0	0	0.623	0	0.616	0	0.229	0.228	0.133
rhov	0.667	0	0	0	0	0	0.667	0	0.654	0	0.902	0.901	0.506
cdc42se2	0.277	0	0	0	0	0	0.277	0	0.275	0	0.165	0.165	0.123
pak3	0.665	0	0	0	0	0	0.665	0	0.655	0	0.925	0.924	0.524
arhgef6	0.724	0	0	0	0	0	0.724	0	0.712	0	0.884	0.884	0.531
git2	0.798	0	0	0	0	0	0.798	0	0.786	0	0.896	0.896	0.531
nisch	0.271	0	0	0	0	0	0.271	0	0.267	0	0.232	0.232	0.135
fam21c	0.255	0	0	0	0	0	0.255	0	0.253	0	0.187	0.186	0.184
wasf3	0.276	0	0	0	0	0	0.276	0	0.275	0	0.213	0.212	0.105
cdc42bpg	0.27	0	0	0	0	0	0.27	0	0.27	0	0.228	0.228	0.14
scrib	0.254	0	0	0	0	0	0.254	0	0.263	0	0.277	0.276	0.178
arpc5	0.266	0	0	0	0	0	0.266	0	0.264	0	0.226	0.224	0.162
exoc2	0.276	0	0	0	0	0	0.276	0	0.276	0	0.28	0.28	0.193

elmo2	0.654	0	0	0	0	0	0.654	0	0.647	0	0.543	0.542	0.329
wipf1	0.253	0	0	0	0	0	0.253	0	0.255	0	0.382	0.38	0.26
arhgap42	0.257	0	0	0	0	0	0.257	0	0.264	0	0.283	0.282	0.163
cyth3	0.262	0	0	0	0	0	0.262	0	0.266	0	0.198	0.197	0.204
vav1	0.265	0	0	0	0	0	0.265	0	0.263	0	0.133	0.131	0.18
hip1r	0.252	0	0	0	0	0	0.252	0	0.255	0	0.256	0.256	0.26
dock1	0.686	0	0	0	0	0	0.686	0	0.678	0	0.298	0.297	0.189
slk	0.289	0	0	0	0	0	0.289	0	0.288	0	0.375	0.372	0.217
arhgef26	0.283	0	0	0	0	0	0.283	0	0.29	0	0.133	0.132	0.16
c17orf62	0.252	0	0	0	0	0	0.252	0	0.252	0	0.346	0.345	0.213
nedd9	0.26	0	0	0	0	0	0.26	0	0.258	0	0.144	0.141	0.254
snx21	0.261	0	0	0	0	0	0.261	0	0.262	0	0.127	0.125	0.118
wash1	0.285	0	0	0	0	0	0.285	0	0.284	0	0.204	0.203	0.145
pak2	0.669	0	0	0	0	0	0.669	0	0.659	0	0.911	0.91	0.516
dennd1c	0.285	0	0	0	0	0	0.285	0	0.283	0	0.143	0.142	0.115
map4k1	0.26	0	0	0	0	0	0.26	0	0.262	0	0.236	0.234	0.243
nckipsd	0.285	0	0	0	0	0	0.285	0	0.282	0	0.253	0.251	0.159
mrx27	0.258	0	0	0	0	0	0.258	0	0.255	0	0.319	0.318	0.203
rab11fip3	0.258	0	0	0	0	0	0.258	0	0.255	0	0.209	0.208	0.152
pak1ip1	0.623	0	0	0	0	0	0.623	0	0.613	0	0.941	0.94	0.517
rhoq	0.261	0	0	0	0	0	0.261	0	0.262	0	0.321	0.32	0.256
pak1	0.696	0	0	0	0	0	0.696	0	0.685	0	0.961	0.961	0.523
rimklb	0	0	0	0.159	0.159	0	0	0	0	0.159	0.111	0.111	0.132
marcks	0.21	0.21	0	0.133	0.133	0	0.21	0.21	0	0.133	0	0	0
gng5p1	0.109	0	0	0.126	0.126	0	0.109	0	0.108	0.126	0	0	0
kncn	0.11	0	0	0.116	0.116	0	0.11	0	0.125	0.116	0	0	0
rgs19	0.108	0	0	0.21	0.21	0	0.108	0	0.108	0.21	0	0	0
dgkg	0.373	0	0	0.131	0.131	0	0.373	0	0.371	0.131	0	0	0
gngt2	0.12	0	0	0.116	0.116	0	0.12	0	0.121	0.116	0	0	0
yme1l1	0.12	0	0	0.11	0.11	0	0.12	0	0.121	0.11	0	0	0
dgkb	0.126	0	0	0.118	0.118	0	0.126	0	0.141	0.118	0	0	0
akap12	0.113	0	0	0.165	0.165	0	0.113	0	0.12	0.165	0	0	0
rab11b	0.13	0	0	0.114	0.114	0	0.13	0	0.135	0.114	0	0	0
vps26b	0.101	0	0	0.119	0.119	0	0.101	0	0.103	0.119	0	0	0
myo1a	0.105	0	0	0.133	0.133	0	0.105	0	0.113	0.133	0	0	0
tuba4a	0.17	0	0	0.11	0.11	0	0.17	0	0.173	0.11	0	0	0
gnb5	0.152	0	0	0.106	0.106	0	0.152	0	0.153	0.106	0	0	0
ocm	0.176	0	0	0.111	0.111	0	0.176	0	0.178	0.111	0	0	0
tbc1d14	0.126	0	0	0.139	0.139	0	0.126	0	0.136	0.139	0	0	0
rap1b	0.135	0	0	0.181	0.181	0	0.135	0	0.139	0.181	0	0	0
sigmar1	0.112	0	0	0.123	0.123	0	0.112	0	0.11	0.123	0	0	0

arpc5l	0.136	0	0	0.187	0.187	0	0.136	0	0.134	0.187	0	0	0
magt1	0.102	0	0	0.142	0.142	0	0.102	0	0.103	0.142	0	0	0
snx6	0.115	0	0	0.104	0.104	0	0.115	0	0.118	0.104	0	0	0
pi4kb	0.128	0	0	0.111	0.111	0	0.128	0	0.13	0.111	0	0	0
ralb	0.222	0	0	0.108	0.108	0	0.222	0	0.226	0.108	0	0	0
otogl	0.117	0	0	0.106	0.106	0	0.117	0	0.128	0.106	0	0	0
nfatc3	0.11	0	0	0.129	0.129	0	0.11	0	0.11	0.129	0	0	0
rngtt	0.122	0	0	0.139	0.139	0	0.122	0	0.119	0.139	0	0	0
myo6	0.106	0	0	0.113	0.113	0	0.106	0	0.112	0.113	0	0	0
cnn3	0.14	0	0	0.234	0.234	0	0.14	0	0.153	0.234	0	0	0
sergef	0.159	0	0	0.104	0.104	0	0.159	0	0.162	0.104	0	0	0
itpka	0.106	0	0	0.212	0.212	0	0.106	0	0.108	0.212	0	0	0
lrrc16b	0.139	0	0	0.124	0.124	0	0.139	0	0.143	0.124	0	0	0
rgs7	0.137	0	0	0.168	0.168	0	0.137	0	0.138	0.168	0	0	0
dgke	0.105	0	0	0.129	0.129	0	0.105	0	0.11	0.129	0	0	0
pgam2	0	0	0	0.112	0.112	0	0	0	0.132	0.112	0.132	0.131	0
bin3	0.305	0	0	0	0	0	0.305	0	0.307	0	0.114	0.113	0.103
clip1	0.144	0	0	0	0	0	0.144	0	0.147	0	0.123	0.121	0.1
mir132	0.148	0	0	0	0	0	0.148	0	0.145	0	0.144	0.143	0.103
vav3	0.299	0	0	0	0	0	0.299	0	0.3	0	0.115	0.114	0.101
ankmy2	0.125	0	0	0	0	0	0.125	0	0.122	0	0.142	0.142	0.101
dbn1	0.173	0	0	0	0	0	0.173	0	0.2	0	0.123	0.122	0.103
apbb1ip	0.163	0	0	0	0	0	0.163	0	0.162	0	0.138	0.137	0.101
brsk1	0.198	0	0	0	0	0	0.198	0	0.197	0	0.201	0.199	0.104
tbc1d20	0.106	0	0.148	0	0	0.148	0.106	0	0.111	0	0	0	0
nip7	0.122	0	0.117	0	0	0.117	0.122	0	0.133	0	0	0	0
zfhx4	0	0	0.126	0.209	0.209	0.126	0	0	0	0.209	0	0	0
nkrf	0	0	0.161	0.168	0.168	0.161	0	0	0	0.168	0	0	0
gls	0	0	0.189	0.16	0.16	0.189	0	0	0	0.16	0	0	0
serpinb7	0	0	0.142	0.124	0.124	0.142	0	0	0	0.124	0	0	0
minos1	0	0	0.202	0.237	0.237	0.202	0	0	0	0.237	0	0	0
lrrc37b	0	0	0.115	0.121	0.121	0.115	0	0	0	0.121	0	0	0
pnma3	0	0	0.209	0.105	0.105	0.209	0	0	0	0.105	0	0	0
dhrs4l2	0	0	0.135	0.167	0.167	0.135	0	0	0	0.167	0	0	0
pde2a	0	0	0.168	0.206	0.206	0.168	0	0	0	0.206	0	0	0
bivm	0	0	0.196	0.235	0.235	0.196	0	0	0	0.235	0	0	0
hcvs	0	0	0.142	0.106	0.106	0.142	0	0	0	0.106	0	0	0
xage2b	0	0	0.169	0.24	0.24	0.169	0	0	0	0.24	0	0	0
camta1	0	0	0.203	0.16	0.16	0.203	0	0	0	0.16	0	0	0
oas2	0	0	0.123	0.161	0.161	0.123	0	0	0	0.161	0	0	0
suox	0	0	0.213	0.263	0.263	0.213	0	0	0	0.263	0	0	0

rassf10	0	0	0.211	0.105	0.105	0.211	0	0	0	0.105	0	0	0
tefm	0	0	0.121	0.124	0.124	0.121	0	0	0	0.124	0	0	0
ddah2	0	0	0.239	0.2	0.2	0.239	0	0	0	0.2	0	0	0
amt	0	0	0.158	0.215	0.215	0.158	0	0	0	0.215	0	0	0
aldh18a1	0	0	0.13	0.165	0.165	0.13	0	0	0	0.165	0	0	0
page3	0	0	0.217	0.258	0.258	0.217	0	0	0	0.258	0	0	0
page1	0	0	0.217	0.258	0.258	0.217	0	0	0	0.258	0	0	0
gatm	0	0	0.127	0.164	0.164	0.127	0	0	0	0.164	0	0	0
mir602	0	0	0.195	0.201	0.201	0.195	0	0	0	0.201	0	0	0
hspa13	0	0	0.11	0.203	0.203	0.11	0	0	0	0.203	0	0	0
cyp4b1	0	0	0.103	0.124	0.124	0.103	0	0	0	0.124	0	0	0
hnrnpl	0	0	0.131	0.202	0.202	0.131	0	0	0	0.202	0	0	0
cdo1	0	0	0.205	0.211	0.211	0.205	0	0	0	0.211	0	0	0
coq10b	0	0	0.145	0.199	0.199	0.145	0	0	0	0.199	0	0	0
bcat2	0	0	0.138	0.202	0.202	0.138	0	0	0	0.202	0	0	0
gucy1a2	0	0	0.168	0.164	0.164	0.168	0	0	0	0.164	0	0	0
got1	0	0	0.132	0.163	0.163	0.132	0	0	0	0.163	0	0	0
mthfd2	0	0	0.102	0.1	0.1	0.102	0	0	0	0.1	0	0	0
idh3g	0	0	0.286	0.24	0.24	0.286	0	0	0	0.24	0	0	0
trnq	0	0	0.26	0.242	0.242	0.26	0	0	0	0.242	0	0	0
slc30a6	0	0	0.13	0.203	0.203	0.13	0	0	0	0.203	0	0	0
xage3	0	0	0.169	0.24	0.24	0.169	0	0	0	0.24	0	0	0
fam57a	0	0	0.109	0.166	0.166	0.109	0	0	0	0.166	0	0	0
xage2	0	0	0.169	0.24	0.24	0.169	0	0	0	0.24	0	0	0
b4galnt4	0	0	0.129	0.105	0.105	0.129	0	0	0	0.105	0	0	0
idh3a	0	0	0.251	0.245	0.245	0.251	0	0	0	0.245	0	0	0
coq9	0	0	0.1	0.162	0.162	0.1	0	0	0	0.162	0	0	0
page2	0	0	0.217	0.258	0.258	0.217	0	0	0	0.258	0	0	0
crat	0	0	0.112	0.161	0.161	0.112	0	0	0	0.161	0	0	0
pyroxd2	0	0	0.132	0.161	0.161	0.132	0	0	0	0.161	0	0	0
vps51	0	0	0.167	0.249	0.249	0.167	0	0	0	0.249	0	0	0
tm7sf3	0	0	0.153	0.209	0.209	0.153	0	0	0	0.209	0	0	0
nadk2	0	0	0.211	0.123	0.123	0.211	0	0	0	0.123	0	0	0
slc7a3	0	0	0.105	0.176	0.176	0.105	0	0	0	0.176	0	0	0
usf2	0	0	0.151	0.186	0.186	0.151	0	0	0	0.186	0	0	0
rps25	0	0	0.159	0.176	0.176	0.159	0	0	0	0.176	0	0	0
hyalp1	0	0	0.14	0.177	0.177	0.14	0	0	0	0.177	0	0	0
cpb1	0	0	0.173	0.219	0.219	0.173	0	0	0	0.219	0	0	0
l2hgdh	0	0	0.316	0.177	0.177	0.316	0	0	0	0.177	0	0	0
mir661	0	0	0.156	0.177	0.177	0.156	0	0	0	0.177	0	0	0
iscu	0	0	0.14	0.219	0.219	0.14	0	0	0	0.219	0	0	0

dpep2	0	0	0.16	0.224	0.224	0.16	0	0	0	0.224	0	0	0
mocs3	0	0	0.141	0.189	0.189	0.141	0	0	0	0.189	0	0	0
klf6	0	0	0.163	0.174	0.174	0.163	0	0	0	0.174	0	0	0
clic3	0	0	0.17	0.225	0.225	0.17	0	0	0	0.225	0	0	0
cyp20a1	0	0	0.267	0.263	0.263	0.267	0	0	0	0.263	0	0	0
mdh1	0	0	0.189	0.188	0.188	0.189	0	0	0	0.188	0	0	0
cyb561d2	0	0	0.119	0.174	0.174	0.119	0	0	0	0.174	0	0	0
ehf	0	0	0.149	0.174	0.174	0.149	0	0	0	0.174	0	0	0
krt17p2	0	0	0.139	0.223	0.223	0.139	0	0	0	0.223	0	0	0
krt17p1	0	0	0.139	0.223	0.223	0.139	0	0	0	0.223	0	0	0
kynu	0	0	0.11	0.181	0.181	0.11	0	0	0	0.181	0	0	0
marc2	0	0	0.182	0.222	0.222	0.182	0	0	0	0.222	0	0	0
aox1	0	0	0.277	0.18	0.18	0.277	0	0	0	0.18	0	0	0
ddah1	0	0	0.227	0.222	0.222	0.227	0	0	0	0.222	0	0	0
gmps	0	0	0.128	0.181	0.181	0.128	0	0	0	0.181	0	0	0
slc43a1	0	0	0.123	0.221	0.221	0.123	0	0	0	0.221	0	0	0
sub1	0	0	0.157	0.181	0.181	0.157	0	0	0	0.181	0	0	0
ndufb2	0	0	0.193	0.22	0.22	0.193	0	0	0	0.22	0	0	0
vnn1	0	0	0.183	0.222	0.222	0.183	0	0	0	0.222	0	0	0
idh3b	0	0	0.286	0.222	0.222	0.286	0	0	0	0.222	0	0	0
esx1	0	0	0.124	0.178	0.178	0.124	0	0	0	0.178	0	0	0
rars2	0	0	0.306	0.223	0.223	0.306	0	0	0	0.223	0	0	0
slc3a2	0	0	0.104	0.184	0.184	0.104	0	0	0	0.184	0	0	0
ctns	0	0	0.109	0.179	0.179	0.109	0	0	0	0.179	0	0	0
ms	0	0	0.199	0.219	0.219	0.199	0	0	0	0.219	0	0	0
galm	0	0	0.195	0.103	0.103	0.195	0	0	0	0.103	0	0	0
ogdhl	0	0	0.103	0.189	0.189	0.103	0	0	0	0.189	0	0	0
slc7a15p	0	0	0.232	0.104	0.104	0.232	0	0	0	0.104	0	0	0
arg2	0	0	0.21	0.232	0.232	0.21	0	0	0	0.232	0	0	0
nd4l	0	0	0.176	0.216	0.216	0.176	0	0	0	0.216	0	0	0
rasal1	0	0	0.193	0.216	0.216	0.193	0	0	0	0.216	0	0	0
znf121	0	0	0.144	0.195	0.195	0.144	0	0	0	0.195	0	0	0
ssav1	0	0	0.19	0.231	0.231	0.19	0	0	0	0.231	0	0	0
taar2	0	0	0.169	0.181	0.181	0.169	0	0	0	0.181	0	0	0
coq10a	0	0	0.159	0.169	0.169	0.159	0	0	0	0.169	0	0	0
dhrs4l1	0	0	0.176	0.169	0.169	0.176	0	0	0	0.169	0	0	0
mir1287	0	0	0.104	0.104	0.104	0.104	0	0	0	0.104	0	0	0
gucy1a3	0	0	0.186	0.168	0.168	0.186	0	0	0	0.168	0	0	0
spink2	0	0	0.172	0.198	0.198	0.172	0	0	0	0.198	0	0	0
cd200	0	0	0.224	0.168	0.168	0.224	0	0	0	0.168	0	0	0
marc1	0	0	0.147	0.169	0.169	0.147	0	0	0	0.169	0	0	0

ass1	0	0	0.207	0.215	0.215	0.207	0	0	0	0.215	0	0	0
odc1	0	0	0.153	0.233	0.233	0.153	0	0	0	0.233	0	0	0
slc30a7	0	0	0.133	0.196	0.196	0.133	0	0	0	0.196	0	0	0
epx	0	0	0.104	0.104	0.104	0.104	0	0	0	0.104	0	0	0
coq6	0	0	0.117	0.218	0.218	0.117	0	0	0	0.218	0	0	0
dhx30	0	0	0.148	0.229	0.229	0.148	0	0	0	0.229	0	0	0
asl	0	0	0.129	0.23	0.23	0.129	0	0	0	0.23	0	0	0
cps1	0	0	0.154	0.191	0.191	0.154	0	0	0	0.191	0	0	0
gfm2	0	0	0.125	0.173	0.173	0.125	0	0	0	0.173	0	0	0
dhodh	0	0	0.115	0.173	0.173	0.115	0	0	0	0.173	0	0	0
gfm1	0	0	0.137	0.225	0.225	0.137	0	0	0	0.225	0	0	0
dyt7	0	0	0.103	0.19	0.19	0.103	0	0	0	0.19	0	0	0
c3orf52	0	0	0.314	0.172	0.172	0.314	0	0	0	0.172	0	0	0
vnn3	0	0	0.14	0.231	0.231	0.14	0	0	0	0.231	0	0	0
xdh	0	0	0.174	0.17	0.17	0.174	0	0	0	0.17	0	0	0
d2hgdh	0	0	0.321	0.17	0.17	0.321	0	0	0	0.17	0	0	0
tkt	0	0	0.171	0.193	0.193	0.171	0	0	0	0.193	0	0	0
tsnax	0	0	0.127	0.172	0.172	0.127	0	0	0	0.172	0	0	0
slc5a3	0	0	0.126	0.193	0.193	0.126	0	0	0	0.193	0	0	0
hs3st3b1	0	0	0.128	0.172	0.172	0.128	0	0	0	0.172	0	0	0
ahcy	0	0	0.127	0.192	0.192	0.127	0	0	0	0.192	0	0	0
pc	0	0	0.133	0.168	0.168	0.133	0	0	0	0.168	0	0	0
adh5	0	0	0.263	0.281	0.281	0.263	0	0	0	0.281	0	0	0
nos2p2	0	0	0.383	0.399	0.399	0.383	0	0	0	0.399	0	0	0
fam109b	0	0	0.107	0.127	0.127	0.107	0	0	0	0.127	0	0	0
fam109a	0	0	0.107	0.127	0.127	0.107	0	0	0	0.127	0	0	0
spsb1	0	0	0.372	0.402	0.402	0.372	0	0	0	0.402	0	0	0
hao2	0	0	0.128	0.14	0.14	0.128	0	0	0	0.14	0	0	0
tbxas1	0	0	0.107	0.111	0.111	0.107	0	0	0	0.111	0	0	0
luzp4	0	0	0.175	0.14	0.14	0.175	0	0	0	0.14	0	0	0
ppp1r14d	0	0	0.101	0.39	0.39	0.101	0	0	0	0.39	0	0	0
plac8	0	0	0.395	0.394	0.394	0.395	0	0	0	0.394	0	0	0
nfs1	0	0	0.329	0.397	0.397	0.329	0	0	0	0.397	0	0	0
arg1	0	0	0.365	0.436	0.436	0.365	0	0	0	0.436	0	0	0
tbc1d23	0	0	0.438	0.442	0.442	0.438	0	0	0	0.442	0	0	0
pnpla7	0	0	0.114	0.139	0.139	0.114	0	0	0	0.139	0	0	0
ndufb4	0	0	0.134	0.138	0.138	0.134	0	0	0	0.138	0	0	0
ngb	0	0	0.196	0.118	0.118	0.196	0	0	0	0.118	0	0	0
mpst	0	0	0.117	0.118	0.118	0.117	0	0	0	0.118	0	0	0
entpd5	0	0	0.112	0.483	0.483	0.112	0	0	0	0.483	0	0	0
acaa2	0	0	0.121	0.474	0.474	0.121	0	0	0	0.474	0	0	0

nos2p1	0	0	0.437	0.447	0.447	0.437	0	0	0	0.447	0	0	0
rundc3b	0	0	0.109	0.47	0.47	0.109	0	0	0	0.47	0	0	0
msra	0	0	0.117	0.139	0.139	0.117	0	0	0	0.139	0	0	0
acy3	0	0	0.128	0.141	0.141	0.128	0	0	0	0.141	0	0	0
pgm3	0	0	0.172	0.141	0.141	0.172	0	0	0	0.141	0	0	0
ldhb	0	0	0.131	0.144	0.144	0.131	0	0	0	0.144	0	0	0
idh1	0	0	0.31	0.159	0.159	0.31	0	0	0	0.159	0	0	0
mreg	0	0	0.195	0.144	0.144	0.195	0	0	0	0.144	0	0	0
stk17a	0	0	0.121	0.11	0.11	0.121	0	0	0	0.11	0	0	0
pgd	0	0	0.208	0.119	0.119	0.208	0	0	0	0.119	0	0	0
ndufa12	0	0	0.116	0.145	0.145	0.116	0	0	0	0.145	0	0	0
nnt	0	0	0.102	0.109	0.109	0.102	0	0	0	0.109	0	0	0
aco2	0	0	0.145	0.145	0.145	0.145	0	0	0	0.145	0	0	0
agxt2	0	0	0.108	0.145	0.145	0.108	0	0	0	0.145	0	0	0
cd163	0	0	0.207	0.143	0.143	0.207	0	0	0	0.143	0	0	0
snord13	0	0	0.107	0.11	0.11	0.107	0	0	0	0.11	0	0	0
spsb2	0	0	0.351	0.384	0.384	0.351	0	0	0	0.384	0	0	0
kprp	0	0	0.103	0.111	0.111	0.103	0	0	0	0.111	0	0	0
hpx	0	0	0.292	0.141	0.141	0.292	0	0	0	0.141	0	0	0
elk3	0	0	0.161	0.141	0.141	0.161	0	0	0	0.141	0	0	0
tmem86b	0	0	0.226	0.356	0.356	0.226	0	0	0	0.356	0	0	0
nanos2	0	0	0.126	0.119	0.119	0.126	0	0	0	0.119	0	0	0
slc11a1	0	0	0.176	0.142	0.142	0.176	0	0	0	0.142	0	0	0
hgsnat	0	0	0.148	0.111	0.111	0.148	0	0	0	0.111	0	0	0
irgm	0	0	0.329	0.343	0.343	0.329	0	0	0	0.343	0	0	0
glud1p5	0	0	0.16	0.137	0.137	0.16	0	0	0	0.137	0	0	0
prkca	0	0	0.114	0.483	0.483	0.114	0	0	0	0.483	0	0	0
alkbh6	0	0	0.105	0.116	0.116	0.105	0	0	0	0.116	0	0	0
mir5087	0	0	0.118	0.128	0.128	0.118	0	0	0	0.128	0	0	0
bp9	0	0	0.116	0.116	0.116	0.116	0	0	0	0.116	0	0	0
mir5094	0	0	0.118	0.128	0.128	0.118	0	0	0	0.128	0	0	0
cpox	0	0	0.121	0.131	0.131	0.121	0	0	0	0.131	0	0	0
soat1	0	0	0.114	0.115	0.115	0.114	0	0	0	0.115	0	0	0
mir939	0	0	0.69	0.688	0.688	0.69	0	0	0	0.688	0	0	0
hebp1	0	0	0.166	0.115	0.115	0.166	0	0	0	0.115	0	0	0
gulop	0	0	0.126	0.115	0.115	0.126	0	0	0	0.115	0	0	0
gls2	0	0	0.139	0.116	0.116	0.139	0	0	0	0.116	0	0	0
cyp27a1	0	0	0.103	0.13	0.13	0.103	0	0	0	0.13	0	0	0
mir5089	0	0	0.118	0.128	0.128	0.118	0	0	0	0.128	0	0	0
mir5091	0	0	0.118	0.128	0.128	0.118	0	0	0	0.128	0	0	0
mir4511	0	0	0.118	0.128	0.128	0.118	0	0	0	0.128	0	0	0

mir5092	0	0	0.118	0.128	0.128	0.118	0	0	0	0.128	0	0	0
mir5093	0	0	0.118	0.128	0.128	0.118	0	0	0	0.128	0	0	0
iba57	0	0	0.159	0.117	0.117	0.159	0	0	0	0.117	0	0	0
dhrs1	0	0	0.223	0.129	0.129	0.223	0	0	0	0.129	0	0	0
alkbh2	0	0	0.251	0.129	0.129	0.251	0	0	0	0.129	0	0	0
man2c1	0	0	0.132	0.116	0.116	0.132	0	0	0	0.116	0	0	0
fubp1	0	0	0.133	0.132	0.132	0.133	0	0	0	0.132	0	0	0
nos2	0	0	0.719	0.684	0.684	0.719	0	0	0	0.684	0	0	0
ina	0	0	0.171	0.118	0.118	0.171	0	0	0	0.118	0	0	0
acly	0	0	0.1	0.117	0.117	0.1	0	0	0	0.117	0	0	0
kif1c	0	0	0.486	0.519	0.519	0.486	0	0	0	0.519	0	0	0
tarm1	0	0	0.162	0.113	0.113	0.162	0	0	0	0.113	0	0	0
bbox1	0	0	0.118	0.135	0.135	0.118	0	0	0	0.135	0	0	0
nrn1l	0	0	0.126	0.51	0.51	0.126	0	0	0	0.51	0	0	0
is2	0	0	0.446	0.505	0.505	0.446	0	0	0	0.505	0	0	0
slc7a2	0	0	0.456	0.507	0.507	0.456	0	0	0	0.507	0	0	0
oxct1	0	0	0.123	0.136	0.136	0.123	0	0	0	0.136	0	0	0
sardh	0	0	0.106	0.135	0.135	0.106	0	0	0	0.135	0	0	0
ptbp1p	0	0	0.581	0.616	0.616	0.581	0	0	0	0.616	0	0	0
mir5088	0	0	0.118	0.128	0.128	0.118	0	0	0	0.128	0	0	0
taldo1	0	0	0.101	0.133	0.133	0.101	0	0	0	0.133	0	0	0
mlph	0	0	0.157	0.133	0.133	0.157	0	0	0	0.133	0	0	0
bp15	0	0	0.642	0.656	0.656	0.642	0	0	0	0.656	0	0	0
bp14	0	0	0.642	0.656	0.656	0.642	0	0	0	0.656	0	0	0
glul	0	0	0.101	0.134	0.134	0.101	0	0	0	0.134	0	0	0
acadl	0	0	0.106	0.117	0.117	0.106	0	0	0	0.117	0	0	0
mir5090	0	0	0.118	0.128	0.128	0.118	0	0	0	0.128	0	0	0
bp16	0	0	0.642	0.656	0.656	0.642	0	0	0	0.656	0	0	0
spsb3	0	0	0.285	0.311	0.311	0.285	0	0	0	0.311	0	0	0
pepc	0	0	0.25	0.144	0.144	0.25	0	0	0	0.144	0	0	0
serinc1	0	0	0.221	0.271	0.271	0.221	0	0	0	0.271	0	0	0
gsta4	0	0	0.111	0.108	0.108	0.111	0	0	0	0.108	0	0	0
crygep	0	0	0.117	0.277	0.277	0.117	0	0	0	0.277	0	0	0
acyp1	0	0	0.114	0.108	0.108	0.114	0	0	0	0.108	0	0	0
h6pd	0	0	0.119	0.153	0.153	0.119	0	0	0	0.153	0	0	0
psmc4	0	0	0.216	0.268	0.268	0.216	0	0	0	0.268	0	0	0
psat1	0	0	0.11	0.153	0.153	0.11	0	0	0	0.153	0	0	0
ct45a1	0	0	0.105	0.125	0.125	0.105	0	0	0	0.125	0	0	0
adhfe1	0	0	0.119	0.108	0.108	0.119	0	0	0	0.108	0	0	0
cyb5r4	0	0	0.104	0.12	0.12	0.104	0	0	0	0.12	0	0	0
uqcrq	0	0	0.128	0.151	0.151	0.128	0	0	0	0.151	0	0	0

atp8	0	0	0.148	0.151	0.151	0.148	0	0	0	0.151	0	0	0
dusp11	0	0	0.127	0.15	0.15	0.127	0	0	0	0.15	0	0	0
rars	0	0	0.189	0.282	0.282	0.189	0	0	0	0.282	0	0	0
gcdh	0	0	0.207	0.151	0.151	0.207	0	0	0	0.151	0	0	0
smtn	0	0	0.103	0.119	0.119	0.103	0	0	0	0.119	0	0	0
apopt1	0	0	0.115	0.119	0.119	0.115	0	0	0	0.119	0	0	0
mbs3	0	0	0.1	0.146	0.146	0.1	0	0	0	0.146	0	0	0
ndufa6	0	0	0.108	0.151	0.151	0.108	0	0	0	0.151	0	0	0
gpx8	0	0	0.131	0.154	0.154	0.131	0	0	0	0.154	0	0	0
rpl18	0	0	0.202	0.154	0.154	0.202	0	0	0	0.154	0	0	0
spg19	0	0	0.113	0.12	0.12	0.113	0	0	0	0.12	0	0	0
ct64	0	0	0.222	0.264	0.264	0.222	0	0	0	0.264	0	0	0
ct62	0	0	0.222	0.264	0.264	0.222	0	0	0	0.264	0	0	0
ct45a3	0	0	0.222	0.264	0.264	0.222	0	0	0	0.264	0	0	0
ct60	0	0	0.222	0.264	0.264	0.222	0	0	0	0.264	0	0	0
psph	0	0	0.11	0.121	0.121	0.11	0	0	0	0.121	0	0	0
lyar	0	0	0.279	0.159	0.159	0.279	0	0	0	0.159	0	0	0
cic	0	0	0.273	0.159	0.159	0.273	0	0	0	0.159	0	0	0
ct45a6	0	0	0.222	0.264	0.264	0.222	0	0	0	0.264	0	0	0
slx1a-sult1a3	0	0	0.131	0.159	0.159	0.131	0	0	0	0.159	0	0	0
ct45a4	0	0	0.222	0.264	0.264	0.222	0	0	0	0.264	0	0	0
ct45a5	0	0	0.222	0.264	0.264	0.222	0	0	0	0.264	0	0	0
slc27a2	0	0	0.123	0.12	0.12	0.123	0	0	0	0.12	0	0	0
ct45b1p	0	0	0.222	0.264	0.264	0.222	0	0	0	0.264	0	0	0
csad	0	0	0.104	0.157	0.157	0.104	0	0	0	0.157	0	0	0
tst	0	0	0.177	0.158	0.158	0.177	0	0	0	0.158	0	0	0
tial1	0	0	0.103	0.158	0.158	0.103	0	0	0	0.158	0	0	0
idh2	0	0	0.31	0.158	0.158	0.31	0	0	0	0.158	0	0	0
sult1c2	0	0	0.112	0.108	0.108	0.112	0	0	0	0.108	0	0	0
txnl4a	0	0	0.201	0.279	0.279	0.201	0	0	0	0.279	0	0	0
tsnax-disc1	0	0	0.18	0.147	0.147	0.18	0	0	0	0.147	0	0	0
me2	0	0	0.178	0.15	0.15	0.178	0	0	0	0.15	0	0	0
crygd	0	0	0.11	0.296	0.296	0.11	0	0	0	0.296	0	0	0
mir1275	0	0	0.17	0.148	0.148	0.17	0	0	0	0.148	0	0	0
gucy1b3	0	0	0.177	0.148	0.148	0.177	0	0	0	0.148	0	0	0
pde1b	0	0	0.113	0.148	0.148	0.113	0	0	0	0.148	0	0	0
mtbs	0	0	0.16	0.109	0.109	0.16	0	0	0	0.109	0	0	0
znf80	0	0	0.264	0.297	0.297	0.264	0	0	0	0.297	0	0	0
lcn12	0	0	0.119	0.126	0.126	0.119	0	0	0	0.126	0	0	0
ccbl1	0	0	0.102	0.146	0.146	0.102	0	0	0	0.146	0	0	0
dlst	0	0	0.103	0.126	0.126	0.103	0	0	0	0.126	0	0	0

pdss1	0	0	0.2	0.305	0.305	0.2	0	0	0	0.305	0	0	0
renbp	0	0	0.117	0.109	0.109	0.117	0	0	0	0.109	0	0	0
fech	0	0	0.241	0.147	0.147	0.241	0	0	0	0.147	0	0	0
ptdss2	0	0	0.237	0.303	0.303	0.237	0	0	0	0.303	0	0	0
csrnp3	0	0	0.272	0.293	0.293	0.272	0	0	0	0.293	0	0	0
cryga	0	0	0.239	0.295	0.295	0.239	0	0	0	0.295	0	0	0
sdha	0	0	0.15	0.108	0.108	0.15	0	0	0	0.108	0	0	0
me1	0	0	0.161	0.149	0.149	0.161	0	0	0	0.149	0	0	0
slc7a1	0	0	0.157	0.289	0.289	0.157	0	0	0	0.289	0	0	0
decr1	0	0	0.106	0.108	0.108	0.106	0	0	0	0.108	0	0	0
usp34	0	0	0.123	0.149	0.149	0.123	0	0	0	0.149	0	0	0
mb	0	0	0.123	0.149	0.149	0.123	0	0	0	0.149	0	0	0
spsb4	0	0	0.25	0.283	0.283	0.25	0	0	0	0.283	0	0	0
clint1	0.128	0	0	0	0	0	0.128	0	0.135	0	0.117	0.115	0
igkv1d-17	0.107	0	0	0	0	0	0.107	0	0.11	0	0.113	0.112	0
camk1	0.107	0	0	0	0	0	0.107	0	0.108	0	0.161	0.159	0
nek6	0.12	0	0	0	0	0	0.12	0	0.12	0	0.119	0.117	0
myl2	0.109	0	0	0	0	0	0.109	0	0.115	0	0.13	0.129	0
map3k9	0.103	0	0	0	0	0	0.103	0	0.103	0	0.114	0.112	0
mrx35	0.101	0	0	0	0	0	0.101	0	0.106	0	0.148	0.148	0
ap1m1	0.101	0	0	0	0	0	0.101	0	0.105	0	0.108	0.107	0
agbl5	0.106	0	0	0	0	0	0.106	0	0.104	0	0.133	0.133	0
scarna6	0.109	0	0	0	0	0	0.109	0	0.115	0	0.102	0.101	0
dstn	0.111	0	0	0	0	0	0.111	0	0.113	0	0.107	0.106	0
mapre1	0.106	0	0	0	0	0	0.106	0	0.108	0	0.103	0.102	0
caskin1	0.123	0	0	0	0	0	0.123	0	0.126	0	0.104	0.103	0
sap30bp	0.112	0	0	0	0	0	0.112	0	0.11	0	0.145	0.145	0
whamm	0.126	0	0	0	0	0	0.126	0	0.125	0	0.127	0.127	0
cab39	0.103	0	0	0	0	0	0.103	0	0.102	0	0.161	0.16	0
mylk	0.123	0	0	0	0	0	0.123	0	0.126	0	0.111	0.11	0
dmkn	0.112	0	0	0	0	0	0.112	0	0.114	0	0.126	0.125	0
mrx81	0.111	0	0	0	0	0	0.111	0	0.13	0	0.173	0.173	0
shank1	0.114	0	0	0	0	0	0.114	0	0.116	0	0.117	0.116	0
lcp1	0.122	0	0	0	0	0	0.122	0	0.123	0	0.107	0.105	0
gng4	0.129	0	0	0	0	0	0.129	0	0.132	0	0.102	0.101	0
pacs2	0.125	0	0	0	0	0	0.125	0	0.124	0	0.155	0.154	0
scarna9	0.109	0	0	0	0	0	0.109	0	0.115	0	0.102	0.101	0
vps35	0.106	0	0	0	0	0	0.106	0	0.109	0	0.102	0.101	0
march5	0.102	0	0	0	0	0	0.102	0	0.112	0	0.147	0.147	0
daam1	0.124	0	0	0	0	0	0.124	0	0.129	0	0.109	0.109	0
stk38	0.117	0	0	0	0	0	0.117	0	0.119	0	0.102	0.1	0

pacsin1	0.125	0	0	0	0	0	0.125	0	0.127	0	0.115	0.113	0
katnb1	0.131	0	0	0	0	0	0.131	0	0.133	0	0.13	0.128	0
ruvbl2	0.108	0	0	0	0	0	0.108	0	0.109	0	0.126	0.126	0
scarna12	0.109	0	0	0	0	0	0.109	0	0.115	0	0.102	0.101	0
clasp1	0.109	0	0	0	0	0	0.109	0	0.11	0	0.106	0.106	0
scarna17	0.109	0	0	0	0	0	0.109	0	0.115	0	0.102	0.101	0
ptprt	0.107	0	0	0	0	0	0.107	0	0.106	0	0.109	0.107	0
wash7p	0.105	0	0	0	0	0	0.105	0	0.101	0	0.108	0.107	0
arhgap24	0.125	0	0	0	0	0	0.125	0	0.123	0	0.102	0.102	0
lrfn3	0.113	0	0	0	0	0	0.113	0	0.117	0	0.111	0.111	0
dynll2	0.124	0	0	0	0	0	0.124	0	0.124	0	0.113	0.113	0
scarna7	0.109	0	0	0	0	0	0.109	0	0.115	0	0.102	0.101	0
plekhh2	0.161	0	0	0	0	0	0.161	0	0.161	0	0.122	0.122	0
cttnbp2	0.186	0	0	0	0	0	0.186	0	0.187	0	0.162	0.162	0
arfgap1	0.186	0	0	0	0	0	0.186	0	0.202	0	0.119	0.118	0
arl1	0.186	0	0	0	0	0	0.186	0	0.211	0	0.11	0.109	0
adap1	0.184	0	0	0	0	0	0.184	0	0.188	0	0.185	0.184	0
fcho2	0.183	0	0	0	0	0	0.183	0	0.192	0	0.153	0.152	0
nexn	0.182	0	0	0	0	0	0.182	0	0.18	0	0.133	0.132	0
iqgap3	0.182	0	0	0	0	0	0.182	0	0.183	0	0.127	0.127	0
rasa1	0.189	0	0	0	0	0	0.189	0	0.197	0	0.133	0.13	0
rab35	0.196	0	0	0	0	0	0.196	0	0.199	0	0.107	0.107	0
or7d2	0.209	0	0	0	0	0	0.209	0	0.205	0	0.133	0.132	0
ncf2	0.21	0	0	0	0	0	0.21	0	0.208	0	0.107	0.107	0
phldb2	0.202	0	0	0	0	0	0.202	0	0.208	0	0.102	0.101	0
usp6nl	0.201	0	0	0	0	0	0.201	0	0.201	0	0.117	0.115	0
exoc5	0.197	0	0	0	0	0	0.197	0	0.2	0	0.113	0.112	0
kptn	0.199	0	0	0	0	0	0.199	0	0.204	0	0.113	0.112	0
exoc7	0.181	0	0	0	0	0	0.181	0	0.184	0	0.157	0.156	0
znf420	0.179	0	0	0	0	0	0.179	0	0.209	0	0.116	0.115	0
c1d	0.162	0	0	0	0	0	0.162	0	0.162	0	0.154	0.153	0
acap2	0.162	0	0	0	0	0	0.162	0	0.165	0	0.11	0.109	0
arfgef2	0.164	0	0	0	0	0	0.164	0	0.185	0	0.103	0.102	0
ap1b1	0.16	0	0	0	0	0	0.16	0	0.159	0	0.111	0.11	0
arhgef2	0.158	0	0	0	0	0	0.158	0	0.161	0	0.106	0.105	0
mob1b	0.152	0	0	0	0	0	0.152	0	0.155	0	0.11	0.108	0
iqsec2	0.155	0	0	0	0	0	0.155	0	0.162	0	0.12	0.119	0
tln2	0.166	0	0	0	0	0	0.166	0	0.18	0	0.108	0.107	0
tspan6	0.168	0	0	0	0	0	0.168	0	0.166	0	0.157	0.156	0
ncf1	0.176	0	0	0	0	0	0.176	0	0.173	0	0.116	0.115	0
arl4a	0.177	0	0	0	0	0	0.177	0	0.18	0	0.121	0.12	0

ppfia4	0.174	0	0	0	0	0	0.174	0	0.176	0	0.163	0.162	0
tln1	0.174	0	0	0	0	0	0.174	0	0.175	0	0.11	0.109	0
wash2p	0.17	0	0	0	0	0	0.17	0	0.173	0	0.127	0.126	0
exoc8	0.171	0	0	0	0	0	0.171	0	0.173	0	0.109	0.108	0
dock5	0.211	0	0	0	0	0	0.211	0	0.212	0	0.104	0.103	0
tbc1d24	0.212	0	0	0	0	0	0.212	0	0.232	0	0.139	0.139	0
arfip2	0.375	0	0	0	0	0	0.375	0	0.379	0	0.166	0.166	0
rap1gds1	0.395	0	0	0	0	0	0.395	0	0.393	0	0.139	0.138	0
snx5	0.415	0	0	0	0	0	0.415	0	0.413	0	0.159	0.159	0
dock4	0.374	0	0	0	0	0	0.374	0	0.38	0	0.122	0.122	0
pip5k1a	0.345	0	0	0	0	0	0.345	0	0.344	0	0.12	0.119	0
trio	0.338	0	0	0	0	0	0.338	0	0.338	0	0.109	0.109	0
lrfn4	0.339	0	0	0	0	0	0.339	0	0.339	0	0.147	0.147	0
prex1	0.415	0	0	0	0	0	0.415	0	0.411	0	0.106	0.104	0
sh3rf1	0.425	0	0	0	0	0	0.425	0	0.424	0	0.122	0.121	0
nckap1	0.529	0	0	0	0	0	0.529	0	0.524	0	0.187	0.186	0
rac1	0.563	0	0	0	0	0	0.563	0	0.557	0	0.108	0.107	0
tiam1	0.522	0	0	0	0	0	0.522	0	0.521	0	0.179	0.178	0
rhog	0.512	0	0	0	0	0	0.512	0	0.509	0	0.154	0.153	0
chn2	0.437	0	0	0	0	0	0.437	0	0.433	0	0.109	0.108	0
psd	0.479	0	0	0	0	0	0.479	0	0.477	0	0.11	0.109	0
pip5k1c	0.317	0	0	0	0	0	0.317	0	0.32	0	0.125	0.124	0
brk1	0.279	0	0	0	0	0	0.279	0	0.277	0	0.116	0.115	0
rhobtb3	0.216	0	0	0	0	0	0.216	0	0.22	0	0.117	0.116	0
tns1	0.217	0	0	0	0	0	0.217	0	0.218	0	0.112	0.111	0
rhobtb1	0.215	0	0	0	0	0	0.215	0	0.22	0	0.141	0.141	0
itgb7	0.213	0	0	0	0	0	0.213	0	0.209	0	0.167	0.167	0
zfyve27	0.212	0	0	0	0	0	0.212	0	0.214	0	0.133	0.132	0
arl2bp	0.212	0	0	0	0	0	0.212	0	0.232	0	0.105	0.103	0
cyth1	0.219	0	0	0	0	0	0.219	0	0.22	0	0.116	0.115	0
map3k6	0.225	0	0	0	0	0	0.225	0	0.223	0	0.103	0.102	0
layn	0.252	0	0	0	0	0	0.252	0	0.249	0	0.112	0.111	0
abr	0.269	0	0	0	0	0	0.269	0	0.269	0	0.182	0.181	0
arhgap9	0.251	0	0	0	0	0	0.251	0	0.247	0	0.152	0.151	0
gbf1	0.239	0	0	0	0	0	0.239	0	0.247	0	0.108	0.107	0
nf2	0.23	0	0	0	0	0	0.23	0	0.24	0	0.18	0.179	0
ngef	0.237	0	0	0	0	0	0.237	0	0.237	0	0.129	0.129	0
arhgap12	0.152	0	0	0	0	0	0.152	0	0.151	0	0.162	0.162	0
dgcr6l	0.201	0	0	0	0	0	0.201	0	0.2	0	0.146	0.145	0
cfl2	0.145	0	0	0	0	0	0.145	0	0.143	0	0.105	0.104	0
iqsec3	0.15	0	0	0	0	0	0.15	0	0.168	0	0.103	0.102	0

copz2	0.146	0	0	0	0	0	0.146	0	0.148	0	0.105	0.104	0
rab11fip4	0.137	0	0	0	0	0	0.137	0	0.138	0	0.106	0.105	0
capg	0.151	0	0	0	0	0	0.151	0	0.158	0	0.119	0.118	0
taok3	0.134	0	0	0	0	0	0.134	0	0.131	0	0.104	0.104	0
ccdc141	0.134	0	0	0	0	0	0.134	0	0.127	0	0.112	0.111	0
gas2l3	0.141	0	0	0	0	0	0.141	0	0.139	0	0.139	0.138	0
epn3	0.148	0	0	0	0	0	0.148	0	0.156	0	0.137	0.137	0
ap1g1	0.143	0	0	0	0	0	0.143	0	0.153	0	0.114	0.113	0
ap1g2	0.143	0	0	0	0	0	0.143	0	0.147	0	0.141	0.14	0
taok2	0.137	0	0	0	0	0	0.137	0	0.135	0	0.168	0.166	0
sytl1	0.142	0	0	0	0	0	0.142	0	0.152	0	0.143	0.143	0
coro2b	0.147	0	0	0	0	0	0.147	0	0.143	0	0.109	0.109	0
fblim1	0.147	0	0	0	0	0	0.147	0	0.146	0	0.127	0.125	0
ppfia2	0.138	0	0	0	0	0	0.138	0	0.14	0	0.112	0.111	0
ppp1r9a	0.146	0	0	0	0	0	0.146	0	0.146	0	0.105	0.104	0
frmpd4	0.151	0	0	0	0	0	0.151	0	0.151	0	0.154	0.153	0
mapk11	0.151	0	0	0	0	0	0.151	0	0.15	0	0.175	0.173	0
ppp1r42	0.145	0	0	0	0	0	0.145	0	0.151	0	0.114	0.112	0
loc647323	0.139	0	0	0	0	0	0.139	0	0.148	0	0.129	0.128	0
khdrbs1	0.134	0	0	0	0	0	0.134	0	0.14	0	0.126	0.124	0
ap2s1	0.138	0	0	0	0	0	0.138	0	0.138	0	0.136	0.135	0
map3k11	0.138	0	0	0	0	0	0.138	0	0.14	0	0.122	0.12	0
zc4h2	0.132	0	0	0	0	0	0.132	0	0.131	0	0.12	0.119	0
katna1	0.132	0	0	0	0	0	0.132	0	0.135	0	0.12	0.118	0
jmjd6	0.138	0	0	0	0	0	0.138	0	0.135	0	0.104	0.103	0
rfx8	0.139	0	0	0	0	0	0.139	0	0.148	0	0.129	0.128	0
mertk	0.138	0	0	0	0	0	0.138	0	0.137	0	0.102	0.101	0
robo4	0.144	0	0	0	0	0	0.144	0	0.143	0	0.135	0.134	0
ppp1r16b	0.152	0	0	0	0	0	0.152	0	0.151	0	0.112	0.111	0
uqcr10	0	0	0	0.122	0.122	0	0	0	0	0.122	0.116	0.115	0
wnk1	0	0	0	0.204	0.204	0	0	0	0	0.204	0.113	0.112	0
cnnm2	0	0	0	0.196	0.196	0	0	0	0	0.196	0.135	0.134	0
ctpp	0	0	0	0.178	0.178	0	0	0	0	0.178	0.122	0.121	0
macom	0	0	0	0.124	0.124	0	0	0	0	0.124	0.128	0.128	0
stk39	0	0	0	0.184	0.184	0	0	0	0	0.184	0.116	0.115	0
flot1	0.143	0	0	0	0	0	0.143	0	0.145	0	0	0	0.249
rapgef5	0.196	0	0	0	0	0	0.196	0	0.197	0	0	0	0.181
stap2	0.103	0	0	0	0	0	0.103	0	0.102	0	0	0	0.349
cblc	0.144	0	0	0	0	0	0.144	0	0.142	0	0	0	0.595
dnm2	0.195	0	0	0	0	0	0.195	0	0.201	0	0	0	0.125
gif	0.118	0	0	0	0	0	0.118	0	0.117	0	0	0	0.567

chmp4a	0.118	0	0	0	0	0	0.118	0	0.129	0	0	0	0.11
sgip1	0.129	0	0	0	0	0	0.129	0	0.134	0	0	0	0.145
rab36	0.111	0	0	0	0	0	0.111	0	0.122	0	0	0	0.113
rgs3	0.245	0	0	0	0	0	0.245	0	0.254	0	0	0	0.108
pik3c2b	0.259	0	0	0	0	0	0.259	0	0.266	0	0	0	0.161
vav2	0.262	0	0	0	0	0	0.262	0	0.261	0	0	0	0.183
chmp4b	0.113	0	0	0	0	0	0.113	0	0.122	0	0	0	0.117
gcc1	0.112	0	0	0	0	0	0.112	0	0.116	0	0	0	0.174
ppp2r1a	0.113	0	0	0	0	0	0.113	0	0.113	0	0	0	0.146
lphn2	0.128	0	0	0	0	0	0.128	0	0.13	0	0	0	0.109
spry2	0.128	0	0	0	0	0	0.128	0	0.127	0	0	0	0.439
cbl	0.141	0	0	0	0	0	0.141	0	0.139	0	0	0	0.605
tmem217	0.102	0	0	0	0	0	0.102	0	0.119	0	0	0	0.136
shkbp1	0.116	0	0	0	0	0	0.116	0	0.113	0	0	0	0.527
sh3gl3	0.11	0	0	0	0	0	0.11	0	0.111	0	0	0	0.287
mapk7	0.111	0	0	0	0	0	0.111	0	0.128	0	0	0	0.138
dnm3	0.141	0	0	0	0	0	0.141	0	0.161	0	0	0	0.111
ptpn20b	0.116	0	0	0	0	0	0.116	0	0.119	0	0	0	0.123
cblb	0.129	0	0	0	0	0	0.129	0	0.128	0	0	0	0.535
arhgap36	0.145	0	0	0	0	0	0.145	0	0.149	0	0	0	0.162
sla	0.168	0	0	0	0	0	0.168	0	0.168	0	0	0	0.46
mtmr6	0.122	0	0	0	0	0	0.122	0	0.13	0	0	0	0.133
aldh16a1	0.104	0	0	0	0	0	0.104	0	0.112	0	0	0	0.312
trip6	0.162	0	0	0	0	0	0.162	0	0.164	0	0	0	0.142
epha1	0.171	0	0	0	0	0	0.171	0	0.17	0	0	0	0.128
rab5a	0.172	0	0	0	0	0	0.172	0	0.177	0	0	0	0.129
sh3kbp1	0.147	0	0	0	0	0	0.147	0	0.146	0	0	0	0.593
stap1	0.104	0	0	0	0	0	0.104	0	0.105	0	0	0	0.144
asb6	0.118	0	0	0	0	0	0.118	0	0.122	0	0	0	0.286
rab21	0.174	0	0	0	0	0	0.174	0	0.179	0	0	0	0.108
pdcd6ip	0.105	0	0	0	0	0	0.105	0	0.109	0	0	0	0.131
epha2	0.161	0	0	0	0	0	0.161	0	0.163	0	0	0	0.298
necap2	0.124	0	0	0	0	0	0.124	0	0.127	0	0	0	0.153
inppl1	0.106	0	0	0	0	0	0.106	0	0.108	0	0	0	0.152
rap2b	0.152	0	0	0	0	0	0.152	0	0.158	0	0	0	0.106
sla2	0.152	0	0	0	0	0	0.152	0	0.152	0	0	0	0.575
ankrd55	0.155	0	0	0	0	0	0.155	0	0.154	0	0	0	0.113
map7d3	0.123	0	0	0	0	0	0.123	0	0.123	0	0	0	0.133
epha10	0.152	0	0	0	0	0	0.152	0	0.151	0	0	0	0.141
gng13	0.106	0	0	0	0	0	0.106	0	0.112	0	0	0	0.106
epha8	0.158	0	0	0	0	0	0.158	0	0.158	0	0	0	0.138

foxd4l5	0.104	0	0	0	0	0	0.104	0	0.101	0	0	0	0.136
sh2b2	0.136	0	0	0	0	0	0.136	0	0.135	0	0	0	0.483
foxd4l2	0.104	0	0	0	0	0	0.104	0	0.101	0	0	0	0.136
foxd4l4	0.104	0	0	0	0	0	0.104	0	0.101	0	0	0	0.136
sh3gl2	0.119	0	0	0	0	0	0.119	0	0.131	0	0	0	0.222
rap1a	0.183	0	0	0	0	0	0.183	0	0.188	0	0	0	0.157
rap1bl	0.126	0	0	0	0	0	0.126	0	0.129	0	0	0	0.142
gdi2	0.145	0	0	0	0	0	0.145	0	0.148	0	0	0	0.106
foxd4l6	0.104	0	0	0	0	0	0.104	0	0.101	0	0	0	0.136
mknk1	0.104	0	0	0	0	0	0.104	0	0.102	0	0	0	0.144
dnm1	0.12	0	0	0	0	0	0.12	0	0.123	0	0	0	0.116
kifc2	0.125	0	0	0	0	0	0.125	0	0.125	0	0	0	0.129
eif3b	0.108	0	0	0	0	0	0.108	0	0.113	0	0	0	0.155
rrad	0	0	0	0.116	0.116	0	0	0	0	0.116	0	0	0.109
znf664	0	0	0	0.129	0.129	0	0	0	0	0.129	0	0	0.136
impdh1p11	0	0	0	0.123	0.123	0	0	0	0	0.123	0	0	0.118
akap5	0	0	0	0.116	0.116	0	0	0	0	0.116	0	0	0.138
etf1p2	0	0	0	0.126	0.126	0	0	0	0	0.126	0	0	0.152
fars2	0	0	0	0.115	0.115	0	0	0	0	0.115	0	0	0.117
sctr	0	0	0	0.182	0.182	0	0	0	0	0.182	0	0	0.186
clcn2	0	0	0	0.126	0.126	0	0	0	0	0.126	0	0	0.102
hrh3	0	0	0	0.115	0.115	0	0	0	0	0.115	0	0	0.103
vn1r1	0	0	0	0.102	0.102	0	0	0	0	0.102	0	0	0.102
asic4	0	0	0	0.12	0.12	0	0	0	0	0.12	0	0	0.14
ppip5k1	0	0	0	0.191	0.191	0	0	0	0	0.191	0	0	0.198
etf1p3	0	0	0	0.126	0.126	0	0	0	0	0.126	0	0	0.152
trpm3	0	0	0	0.127	0.127	0	0	0	0	0.127	0	0	0.168
c15orf27	0	0	0	0.119	0.119	0	0	0	0	0.119	0	0	0.109
slc24a1	0	0	0	0.182	0.182	0	0	0	0	0.182	0	0	0.112
pdcd6	0	0	0	0.126	0.126	0	0	0	0	0.126	0	0	0.133
pkd1l3	0	0	0	0.126	0.126	0	0	0	0	0.126	0	0	0.132
kcnk6	0	0	0	0.132	0.132	0	0	0	0	0.132	0	0	0.119
slc5a6	0	0	0	0.178	0.178	0	0	0	0	0.178	0	0	0.128
kcnj14	0	0	0	0.138	0.138	0	0	0	0	0.138	0	0	0.108
atf4p3	0	0	0	0.107	0.107	0	0	0	0	0.107	0	0	0.105
gpr27	0	0	0	0.114	0.114	0	0	0	0	0.114	0	0	0.14
kcnk2	0	0	0	0.106	0.106	0	0	0	0	0.106	0	0	0.161
tmem100	0	0	0	0.271	0.271	0	0	0	0	0.271	0	0	0.115
slc6a13	0	0	0	0.112	0.112	0	0	0	0	0.112	0	0	0.102
atf4p4	0	0	0	0.107	0.107	0	0	0	0	0.107	0	0	0.105
slc44a1	0	0	0	0.169	0.169	0	0	0	0	0.169	0	0	0.108

kcns3	0	0	0	0.153	0.153	0	0	0	0	0.153	0	0	0.116
ca5b	0	0	0	0.224	0.224	0	0	0	0	0.224	0	0	0.108
kcnk1	0	0	0	0.156	0.156	0	0	0	0	0.156	0	0	0.15
cacfd1	0	0	0	0.112	0.112	0	0	0	0	0.112	0	0	0.11
kcnk18	0	0	0	0.107	0.107	0	0	0	0	0.107	0	0	0.198
kcnk10	0	0	0	0.14	0.14	0	0	0	0	0.14	0	0	0.11
piezo2	0	0	0	0.114	0.114	0	0	0	0	0.114	0	0	0.215
kcnk4	0	0	0	0.132	0.132	0	0	0	0	0.132	0	0	0.163
asic1	0	0	0	0.147	0.147	0	0	0	0	0.147	0	0	0.197
fyco1	0	0	0	0.174	0.174	0	0	0	0	0.174	0	0	0.121
kcnk9	0	0	0	0.131	0.131	0	0	0	0	0.131	0	0	0.117
asic2	0	0	0	0.144	0.144	0	0	0	0	0.144	0	0	0.187
trpv4	0	0	0	0.146	0.146	0	0	0	0	0.146	0	0	0.191
kcna4	0	0	0	0.115	0.115	0	0	0	0	0.115	0	0	0.118
mrgprx3	0	0	0	0.105	0.105	0	0	0	0	0.105	0	0	0.178
tes	0	0	0	0.103	0.103	0	0	0	0	0.103	0	0	0.121
nkain3	0	0	0	0.162	0.162	0	0	0	0	0.162	0	0	0.165
hrh4	0	0	0	0.114	0.114	0	0	0	0	0.114	0	0	0.115
ppip5k2	0	0	0	0.24	0.24	0	0	0	0	0.24	0	0	0.144
s100z	0	0	0	0.111	0.111	0	0	0	0	0.111	0	0	0.104
spata6	0	0	0	0.167	0.167	0	0	0	0.101	0.167	0	0	0
rab20	0	0	0	0.105	0.105	0	0	0	0.137	0.105	0	0	0
cnn1	0	0	0	0.196	0.196	0	0	0	0.105	0.196	0	0	0
nme4	0	0	0	0.104	0.104	0	0	0	0.101	0.104	0	0	0
ovca2	0	0	0	0.109	0.109	0	0	0	0.104	0.109	0	0	0
mpp1	0	0	0	0.113	0.113	0	0	0	0.104	0.113	0	0	0
gja3	0	0	0	0.105	0.105	0	0	0	0.109	0.105	0	0	0
fmnl1	0.279	0	0	0	0	0	0.279	0	0.277	0	0.1	0	0
stk38l	0.141	0	0	0	0	0	0.141	0	0.16	0	0.101	0	0
hcls1	0.101	0	0	0	0	0	0.101	0	0.105	0	0.101	0	0
wash4p	0.102	0	0	0	0	0	0.102	0	0	0	0.103	0.103	0
ppwd1	0.1	0	0	0	0	0	0.1	0	0	0	0.134	0.134	0
arhgef16	0.124	0	0	0	0	0	0.124	0	0.141	0	0	0	0.102
arap1	0.23	0	0	0	0	0	0.23	0	0.239	0	0	0	0.102
mtif3	0.117	0	0	0	0	0	0.117	0	0.134	0	0	0	0.101
inpp4a	0.123	0	0	0	0	0	0.123	0	0.124	0	0	0	0.104
epb41l4b	0.104	0	0	0	0	0	0.104	0	0.106	0	0	0	0.1
cpne6	0	0	0	0.121	0.121	0	0	0	0	0.121	0	0	0.103
trpm2	0	0	0	0.165	0.165	0	0	0	0	0.165	0	0	0.1
adal	0	0	0.104	0	0	0.104	0	0	0	0	0	0	0.125
tas2r31	0	0	0.115	0	0	0.115	0	0	0	0	0	0	0.152

chga	0	0	0.104	0	0	0.104	0	0	0	0	0	0	0.127
mmachc	0	0	0.129	0	0	0.129	0	0	0	0	0	0	0.183
resp18	0	0	0.107	0	0	0.107	0	0	0	0	0	0	0.117
ttc39a	0.103	0	0	0	0	0	0.103	0	0	0	0	0	0.33
spred3	0.101	0	0	0	0	0	0.101	0	0	0	0	0	0.469
arhgap33	0	0	0	0	0	0	0	0	0	0	0.104	0.102	0.158
cdc42	0	0	0	0	0	0	0	0	0	0	0.144	0.144	0.123
cdc42ep1	0	0	0	0	0	0	0	0	0	0	0.213	0.213	0.162
arhgap10	0	0	0	0	0	0	0	0	0	0	0.193	0.192	0.179
mesdc1	0	0	0	0	0	0	0	0	0	0	0.125	0.124	0.117
itsn1	0	0	0	0	0	0	0	0	0	0	0.103	0.102	0.305
lcp2	0	0	0	0	0	0	0	0	0	0	0.112	0.11	0.213
hsp90b3p	0	0	0	0	0	0	0	0	0	0	0.176	0.176	0.13
fam50b	0	0	0	0	0	0	0	0	0	0	0.13	0.13	0.12
trip10	0	0	0	0	0	0	0	0	0	0	0.124	0.123	0.131
bnip2	0	0	0	0	0	0	0	0	0	0	0.138	0.138	0.126
fnbp1	0	0	0	0	0	0	0	0	0	0	0.11	0.109	0.115
cdc42p6	0	0	0	0	0	0	0	0	0	0	0.119	0.119	0.113
tbc1d10a	0.101	0	0	0	0	0	0.101	0	0.106	0	0	0	0
ighv1-3	0.101	0	0	0	0	0	0.101	0	0.118	0	0	0	0
myrip	0.1	0	0	0	0	0	0.1	0	0.1	0	0	0	0
bin2	0.101	0	0	0	0	0	0.101	0	0.101	0	0	0	0
lrrc14	0.1	0	0	0	0	0	0.1	0	0.11	0	0	0	0
stmn3	0.102	0	0	0	0	0	0.102	0	0.105	0	0	0	0
obsl1	0.104	0	0	0	0	0	0.104	0	0.104	0	0	0	0
arhgap25	0.104	0	0	0	0	0	0.104	0	0.106	0	0	0	0
mpp5	0.102	0	0	0	0	0	0.102	0	0.111	0	0	0	0
ddn	0.101	0	0	0	0	0	0.101	0	0.104	0	0	0	0
rab11fip1	0.105	0	0	0	0	0	0.105	0	0.106	0	0	0	0
eif4e3	0.105	0	0	0	0	0	0.105	0	0.102	0	0	0	0
mypn	0.102	0	0	0	0	0	0.102	0	0.104	0	0	0	0
copb2	0.1	0	0	0	0	0	0.1	0	0.101	0	0	0	0
spire2	0.105	0	0	0	0	0	0.105	0	0.105	0	0	0	0
rasa4b	0.101	0	0	0	0	0	0.101	0	0.105	0	0	0	0
tpm2	0.105	0	0	0	0	0	0.105	0	0.102	0	0	0	0
mff	0.1	0	0	0	0	0	0.1	0	0.102	0	0	0	0
rgl1	0.105	0	0	0	0	0	0.105	0	0.108	0	0	0	0
gpr158	0.1	0	0	0	0	0	0.1	0	0.101	0	0	0	0
mterfd3	0.102	0	0	0	0	0	0.102	0	0.101	0	0	0	0
ptpn21	0.103	0	0	0	0	0	0.103	0	0.103	0	0	0	0
ehd1	0.103	0	0	0	0	0	0.103	0	0.113	0	0	0	0

myom3	0.103	0	0	0	0	0	0.103	0	0.102	0	0	0	0
magi1	0.103	0	0	0	0	0	0.103	0	0.112	0	0	0	0
klhl24	0.103	0	0	0	0	0	0.103	0	0.109	0	0	0	0
rab25	0.103	0	0	0	0	0	0.103	0	0.107	0	0	0	0
lrrc24	0.1	0	0	0	0	0	0.1	0	0.11	0	0	0	0
sept7	0.103	0	0	0	0	0	0.103	0	0.11	0	0	0	0
rbm26	0.103	0	0	0	0	0	0.103	0	0.104	0	0	0	0
exph5	0.103	0	0	0	0	0	0.103	0	0.102	0	0	0	0
rgs22	0.102	0	0	0	0	0	0.102	0	0.103	0	0	0	0
tulp3	0.102	0	0	0	0	0	0.102	0	0.103	0	0	0	0
pdcl	0.104	0	0	0	0	0	0.104	0	0.104	0	0	0	0
ngdn	0.104	0	0	0	0	0	0.104	0	0.108	0	0	0	0
lin7a	0.102	0	0	0	0	0	0.102	0	0.106	0	0	0	0
clca2	0.104	0	0	0	0	0	0.104	0	0.103	0	0	0	0
cnot11	0.103	0	0	0	0	0	0.103	0	0.102	0	0	0	0
ppp1cc	0.104	0	0	0	0	0	0.104	0	0.108	0	0	0	0
ccnl2	0.102	0	0	0	0	0	0.102	0	0.113	0	0	0	0
psd4	0.104	0	0	0	0	0	0.104	0	0.112	0	0	0	0
gga2	0.104	0	0	0	0	0	0.104	0	0.142	0	0	0	0
tpm3	0.104	0	0	0	0	0	0.104	0	0.103	0	0	0	0
snap91	0.104	0	0	0	0	0	0.104	0	0.108	0	0	0	0
palld	0.102	0	0	0	0	0	0.102	0	0.109	0	0	0	0
gna11	0.142	0	0	0	0	0	0.142	0	0.142	0	0	0	0
adrbk1	0.251	0	0	0	0	0	0.251	0	0.249	0	0	0	0
pdgfrb	0.26	0	0	0	0	0	0.26	0	0.262	0	0	0	0
gna15	0.142	0	0	0	0	0	0.142	0	0.145	0	0	0	0
trdv1	0.261	0	0	0	0	0	0.261	0	0.265	0	0	0	0
ephb2	0.141	0	0	0	0	0	0.141	0	0.142	0	0	0	0
def6	0.25	0	0	0	0	0	0.25	0	0.255	0	0	0	0
noxa1	0.249	0	0	0	0	0	0.249	0	0.249	0	0	0	0
ephb4	0.142	0	0	0	0	0	0.142	0	0.144	0	0	0	0
ppp2r2b	0.143	0	0	0	0	0	0.143	0	0.149	0	0	0	0
gna14	0.142	0	0	0	0	0	0.142	0	0.143	0	0	0	0
zbtb12	0.246	0	0	0	0	0	0.246	0	0.246	0	0	0	0
sec14l3	0.249	0	0	0	0	0	0.249	0	0.245	0	0	0	0
gnai1	0.142	0	0	0	0	0	0.142	0	0.142	0	0	0	0
hace1	0.266	0	0	0	0	0	0.266	0	0.269	0	0	0	0
tpm4	0.141	0	0	0	0	0	0.141	0	0.155	0	0	0	0
pik3c2a	0.278	0	0	0	0	0	0.278	0	0.275	0	0	0	0
cep70	0.139	0	0	0	0	0	0.139	0	0.146	0	0	0	0
ralgps2	0.139	0	0	0	0	0	0.139	0	0.148	0	0	0	0

adrbk2	0.139	0	0	0	0	0	0.139	0	0.14	0	0	0	0
pcdhgc5	0.138	0	0	0	0	0	0.138	0	0.14	0	0	0	0
grk5	0.199	0	0	0	0	0	0.199	0	0.197	0	0	0	0
spef1	0.139	0	0	0	0	0	0.139	0	0.14	0	0	0	0
gdi1	0.14	0	0	0	0	0	0.14	0	0.144	0	0	0	0
myo1b	0.14	0	0	0	0	0	0.14	0	0.141	0	0	0	0
nckap1l	0.273	0	0	0	0	0	0.273	0	0.275	0	0	0	0
ffar2	0.14	0	0	0	0	0	0.14	0	0.138	0	0	0	0
snx2	0.14	0	0	0	0	0	0.14	0	0.143	0	0	0	0
tm2d1	0.275	0	0	0	0	0	0.275	0	0.274	0	0	0	0
krtap1-1	0.245	0	0	0	0	0	0.245	0	0.24	0	0	0	0
acta1	0.143	0	0	0	0	0	0.143	0	0.139	0	0	0	0
ptpn13	0.151	0	0	0	0	0	0.151	0	0.153	0	0	0	0
rgs16	0.151	0	0	0	0	0	0.151	0	0.151	0	0	0	0
actn2	0.15	0	0	0	0	0	0.15	0	0.154	0	0	0	0
kidins220	0.15	0	0	0	0	0	0.15	0	0.152	0	0	0	0
arhgdib	0.243	0	0	0	0	0	0.243	0	0.245	0	0	0	0
pyurf	0.149	0	0	0	0	0	0.149	0	0.148	0	0	0	0
sdcbp	0.151	0	0	0	0	0	0.151	0	0.155	0	0	0	0
pkn2	0.151	0	0	0	0	0	0.151	0	0.156	0	0	0	0
rab41	0.152	0	0	0	0	0	0.152	0	0.153	0	0	0	0
actn4	0.153	0	0	0	0	0	0.153	0	0.155	0	0	0	0
rgs7bp	0.152	0	0	0	0	0	0.152	0	0.151	0	0	0	0
ric8b	0.152	0	0	0	0	0	0.152	0	0.152	0	0	0	0
rnd1	0.241	0	0	0	0	0	0.241	0	0.244	0	0	0	0
rcc2	0.149	0	0	0	0	0	0.149	0	0.149	0	0	0	0
sept14	0.148	0	0	0	0	0	0.148	0	0.15	0	0	0	0
kank3	0.145	0	0	0	0	0	0.145	0	0.147	0	0	0	0
arhgef25	0.243	0	0	0	0	0	0.243	0	0.246	0	0	0	0
spata13	0.243	0	0	0	0	0	0.243	0	0.24	0	0	0	0
ccm2	0.244	0	0	0	0	0	0.244	0	0.25	0	0	0	0
ppp5c	0.144	0	0	0	0	0	0.144	0	0.143	0	0	0	0
gid8	0.144	0	0	0	0	0	0.144	0	0.143	0	0	0	0
kank4	0.145	0	0	0	0	0	0.145	0	0.147	0	0	0	0
rgs13	0.145	0	0	0	0	0	0.145	0	0.144	0	0	0	0
ibgc1	0.147	0	0	0	0	0	0.147	0	0.15	0	0	0	0
rgs4	0.148	0	0	0	0	0	0.148	0	0.15	0	0	0	0
efnb1	0.147	0	0	0	0	0	0.147	0	0.151	0	0	0	0
rgs5	0.147	0	0	0	0	0	0.147	0	0.156	0	0	0	0
eps8l3	0.146	0	0	0	0	0	0.146	0	0.146	0	0	0	0
plekhg2	0.28	0	0	0	0	0	0.28	0	0.28	0	0	0	0

s1pr4	0.288	0	0	0	0	0	0.288	0	0.285	0	0	0	0
tp73	0.134	0	0	0	0	0	0.134	0	0.134	0	0	0	0
cyfip2	0.399	0	0	0	0	0	0.399	0	0.393	0	0	0	0
kank2	0.134	0	0	0	0	0	0.134	0	0.136	0	0	0	0
map3k3	0.133	0	0	0	0	0	0.133	0	0.134	0	0	0	0
pdxp	0.405	0	0	0	0	0	0.405	0	0.398	0	0	0	0
tiam2	0.402	0	0	0	0	0	0.402	0	0.399	0	0	0	0
pskh1	0.134	0	0	0	0	0	0.134	0	0.142	0	0	0	0
gnao1	0.134	0	0	0	0	0	0.134	0	0.134	0	0	0	0
ampd2	0.135	0	0	0	0	0	0.135	0	0.137	0	0	0	0
mical2	0.135	0	0	0	0	0	0.135	0	0.136	0	0	0	0
rasa3	0.134	0	0	0	0	0	0.134	0	0.136	0	0	0	0
plxnb3	0.368	0	0	0	0	0	0.368	0	0.363	0	0	0	0
krtap11-1	0.381	0	0	0	0	0	0.381	0	0.376	0	0	0	0
rala	0.374	0	0	0	0	0	0.374	0	0.376	0	0	0	0
skap2	0.133	0	0	0	0	0	0.133	0	0.138	0	0	0	0
arhgdia	0.408	0	0	0	0	0	0.408	0	0.407	0	0	0	0
sphk1	0.132	0	0	0	0	0	0.132	0	0.132	0	0	0	0
gnaz	0.132	0	0	0	0	0	0.132	0	0.132	0	0	0	0
prps1p2	0.132	0	0	0	0	0	0.132	0	0.137	0	0	0	0
noxo1	0.132	0	0	0	0	0	0.132	0	0.131	0	0	0	0
gage2a	0.132	0	0	0	0	0	0.132	0	0.133	0	0	0	0
rab6a	0.132	0	0	0	0	0	0.132	0	0.136	0	0	0	0
rab11a	0.132	0	0	0	0	0	0.132	0	0.136	0	0	0	0
gnai3	0.132	0	0	0	0	0	0.132	0	0.132	0	0	0	0
rap2c	0.132	0	0	0	0	0	0.132	0	0.133	0	0	0	0
mapre3	0.132	0	0	0	0	0	0.132	0	0.137	0	0	0	0
smarcd2	0.41	0	0	0	0	0	0.41	0	0.403	0	0	0	0
loc100128651	0.412	0	0	0	0	0	0.412	0	0.406	0	0	0	0
clta	0.132	0	0	0	0	0	0.132	0	0.136	0	0	0	0
arfgef1	0.135	0	0	0	0	0	0.135	0	0.15	0	0	0	0
kank1	0.136	0	0	0	0	0	0.136	0	0.14	0	0	0	0
rab7a	0.137	0	0	0	0	0	0.137	0	0.139	0	0	0	0
cope	0.137	0	0	0	0	0	0.137	0	0.144	0	0	0	0
ap2b1	0.137	0	0	0	0	0	0.137	0	0.137	0	0	0	0
arhgef1	0.137	0	0	0	0	0	0.137	0	0.14	0	0	0	0
icmt	0.299	0	0	0	0	0	0.299	0	0.298	0	0	0	0
tspan13	0.292	0	0	0	0	0	0.292	0	0.292	0	0	0	0
gng3	0.137	0	0	0	0	0	0.137	0	0.138	0	0	0	0
asap3	0.138	0	0	0	0	0	0.138	0	0.137	0	0	0	0
gnaq	0.138	0	0	0	0	0	0.138	0	0.138	0	0	0	0

ptplad1	0.29	0	0	0	0	0	0.29	0	0.285	0	0	0	0
acap1	0.138	0	0	0	0	0	0.138	0	0.145	0	0	0	0
sh3gl1	0.138	0	0	0	0	0	0.138	0	0.14	0	0	0	0
arhgef4	0.291	0	0	0	0	0	0.291	0	0.287	0	0	0	0
pacsin2	0.3	0	0	0	0	0	0.3	0	0.298	0	0	0	0
nek3	0.304	0	0	0	0	0	0.304	0	0.301	0	0	0	0
ncf4	0.136	0	0	0	0	0	0.136	0	0.134	0	0	0	0
rhod	0.136	0	0	0	0	0	0.136	0	0.142	0	0	0	0
sgsm2	0.136	0	0	0	0	0	0.136	0	0.147	0	0	0	0
als2	0.34	0	0	0	0	0	0.34	0	0.342	0	0	0	0
tmod3	0.136	0	0	0	0	0	0.136	0	0.138	0	0	0	0
s1pr3	0.353	0	0	0	0	0	0.353	0	0.353	0	0	0	0
chn1	0.337	0	0	0	0	0	0.337	0	0.335	0	0	0	0
rhoh	0.328	0	0	0	0	0	0.328	0	0.326	0	0	0	0
lima1	0.137	0	0	0	0	0	0.137	0	0.153	0	0	0	0
mcf2l	0.314	0	0	0	0	0	0.314	0	0.314	0	0	0	0
ppp1r18	0.137	0	0	0	0	0	0.137	0	0.141	0	0	0	0
akip1	0.319	0	0	0	0	0	0.319	0	0.316	0	0	0	0
ccdc91	0.136	0	0	0	0	0	0.136	0	0.143	0	0	0	0
dennd5a	0.154	0	0	0	0	0	0.154	0	0.16	0	0	0	0
avl9	0.155	0	0	0	0	0	0.155	0	0.159	0	0	0	0
rab28	0.179	0	0	0	0	0	0.179	0	0.179	0	0	0	0
csag1	0.179	0	0	0	0	0	0.179	0	0.178	0	0	0	0
lpar2	0.179	0	0	0	0	0	0.179	0	0.183	0	0	0	0
shb	0.178	0	0	0	0	0	0.178	0	0.18	0	0	0	0
pik3ip1	0.178	0	0	0	0	0	0.178	0	0.174	0	0	0	0
arhgap5	0.178	0	0	0	0	0	0.178	0	0.183	0	0	0	0
inpp5j	0.181	0	0	0	0	0	0.181	0	0.184	0	0	0	0
mical1	0.182	0	0	0	0	0	0.182	0	0.18	0	0	0	0
rasgrp2	0.187	0	0	0	0	0	0.187	0	0.185	0	0	0	0
rgl2	0.187	0	0	0	0	0	0.187	0	0.189	0	0	0	0
arhgap22	0.186	0	0	0	0	0	0.186	0	0.186	0	0	0	0
bzw1	0.215	0	0	0	0	0	0.215	0	0.213	0	0	0	0
gpsm1	0.183	0	0	0	0	0	0.183	0	0.189	0	0	0	0
fcho1	0.185	0	0	0	0	0	0.185	0	0.197	0	0	0	0
efna1	0.177	0	0	0	0	0	0.177	0	0.18	0	0	0	0
ehd2	0.176	0	0	0	0	0	0.176	0	0.181	0	0	0	0
dgkz	0.223	0	0	0	0	0	0.223	0	0.236	0	0	0	0
rapgef3	0.172	0	0	0	0	0	0.172	0	0.175	0	0	0	0
eps8l2	0.172	0	0	0	0	0	0.172	0	0.171	0	0	0	0
plxnb2	0.171	0	0	0	0	0	0.171	0	0.17	0	0	0	0

grk6	0.171	0	0	0	0	0	0.171	0	0.169	0	0	0	0
zfyve20	0.171	0	0	0	0	0	0.171	0	0.171	0	0	0	0
gnb1	0.223	0	0	0	0	0	0.223	0	0.227	0	0	0	0
plcb2	0.221	0	0	0	0	0	0.221	0	0.221	0	0	0	0
ccdc125	0.174	0	0	0	0	0	0.174	0	0.175	0	0	0	0
ric8a	0.175	0	0	0	0	0	0.175	0	0.176	0	0	0	0
ptk2	0.172	0	0	0	0	0	0.172	0	0.17	0	0	0	0
dst	0.172	0	0	0	0	0	0.172	0	0.173	0	0	0	0
tubb2b	0.217	0	0	0	0	0	0.217	0	0.217	0	0	0	0
sema5a	0.214	0	0	0	0	0	0.214	0	0.216	0	0	0	0
pea15	0.187	0	0	0	0	0	0.187	0	0.189	0	0	0	0
ssx2ip	0.194	0	0	0	0	0	0.194	0	0.198	0	0	0	0
flad1	0.203	0	0	0	0	0	0.203	0	0.199	0	0	0	0
pik3r5	0.206	0	0	0	0	0	0.206	0	0.202	0	0	0	0
rab12	0.207	0	0	0	0	0	0.207	0	0.207	0	0	0	0
gpsm3	0.192	0	0	0	0	0	0.192	0	0.193	0	0	0	0
cthrc1	0.207	0	0	0	0	0	0.207	0	0.215	0	0	0	0
pik3cg	0.194	0	0	0	0	0	0.194	0	0.19	0	0	0	0
dennd6a	0.199	0	0	0	0	0	0.199	0	0.202	0	0	0	0
nfyc	0.198	0	0	0	0	0	0.198	0	0.198	0	0	0	0
s1pr5	0.198	0	0	0	0	0	0.198	0	0.197	0	0	0	0
pfn1	0.196	0	0	0	0	0	0.196	0	0.197	0	0	0	0
gpr124	0.195	0	0	0	0	0	0.195	0	0.192	0	0	0	0
rgs1	0.199	0	0	0	0	0	0.199	0	0.199	0	0	0	0
gng2	0.208	0	0	0	0	0	0.208	0	0.208	0	0	0	0
rgp1	0.192	0	0	0	0	0	0.192	0	0.197	0	0	0	0
als2cl	0.214	0	0	0	0	0	0.214	0	0.222	0	0	0	0
znf709	0.19	0	0	0	0	0	0.19	0	0.188	0	0	0	0
arhgef28	0.19	0	0	0	0	0	0.19	0	0.19	0	0	0	0
mir142	0.189	0	0	0	0	0	0.189	0	0.185	0	0	0	0
lpar1	0.188	0	0	0	0	0	0.188	0	0.189	0	0	0	0
gnb4	0.188	0	0	0	0	0	0.188	0	0.188	0	0	0	0
prps1l1	0.19	0	0	0	0	0	0.19	0	0.189	0	0	0	0
sipa1	0.191	0	0	0	0	0	0.191	0	0.194	0	0	0	0
plxnb1	0.191	0	0	0	0	0	0.191	0	0.193	0	0	0	0
tbc1d10b	0.208	0	0	0	0	0	0.208	0	0.215	0	0	0	0
vasp	0.209	0	0	0	0	0	0.209	0	0.211	0	0	0	0
dscr8	0.212	0	0	0	0	0	0.212	0	0.214	0	0	0	0
pik3r6	0.213	0	0	0	0	0	0.213	0	0.21	0	0	0	0
nfyb	0.171	0	0	0	0	0	0.171	0	0.17	0	0	0	0
ccser2	0.17	0	0	0	0	0	0.17	0	0.169	0	0	0	0

smpx	0.235	0	0	0	0	0	0.235	0	0.281	0	0	0	0
coro1c	0.16	0	0	0	0	0	0.16	0	0.167	0	0	0	0
iqgap1	0.159	0	0	0	0	0	0.159	0	0.162	0	0	0	0
znf644	0.158	0	0	0	0	0	0.158	0	0.159	0	0	0	0
fhod1	0.236	0	0	0	0	0	0.236	0	0.232	0	0	0	0
nox5	0.158	0	0	0	0	0	0.158	0	0.159	0	0	0	0
iqgap2	0.16	0	0	0	0	0	0.16	0	0.163	0	0	0	0
rab1b	0.234	0	0	0	0	0	0.234	0	0.238	0	0	0	0
copa	0.162	0	0	0	0	0	0.162	0	0.17	0	0	0	0
nxph1	0.163	0	0	0	0	0	0.163	0	0.165	0	0	0	0
trip11	0.162	0	0	0	0	0	0.162	0	0.165	0	0	0	0
schip1	0.161	0	0	0	0	0	0.161	0	0.167	0	0	0	0
slc6a7	0.16	0	0	0	0	0	0.16	0	0.167	0	0	0	0
myadm	0.233	0	0	0	0	0	0.233	0	0.232	0	0	0	0
smap1	0.236	0	0	0	0	0	0.236	0	0.239	0	0	0	0
snx1	0.158	0	0	0	0	0	0.158	0	0.166	0	0	0	0
prex2	0.238	0	0	0	0	0	0.238	0	0.236	0	0	0	0
chchd2	0.237	0	0	0	0	0	0.237	0	0.237	0	0	0	0
gna12	0.155	0	0	0	0	0	0.155	0	0.156	0	0	0	0
s1pr1	0.24	0	0	0	0	0	0.24	0	0.24	0	0	0	0
rgs8	0.155	0	0	0	0	0	0.155	0	0.155	0	0	0	0
ppp1r9b	0.155	0	0	0	0	0	0.155	0	0.157	0	0	0	0
gdpd2	0.156	0	0	0	0	0	0.156	0	0.158	0	0	0	0
lphn1	0.156	0	0	0	0	0	0.156	0	0.158	0	0	0	0
rab11fip2	0.156	0	0	0	0	0	0.156	0	0.161	0	0	0	0
fscn1	0.157	0	0	0	0	0	0.157	0	0.166	0	0	0	0
plek2	0.236	0	0	0	0	0	0.236	0	0.233	0	0	0	0
plekhg4	0.237	0	0	0	0	0	0.237	0	0.24	0	0	0	0
tbc1d21	0.156	0	0	0	0	0	0.156	0	0.164	0	0	0	0
prr5	0.233	0	0	0	0	0	0.233	0	0.234	0	0	0	0
arcn1	0.163	0	0	0	0	0	0.163	0	0.169	0	0	0	0
ptprh	0.166	0	0	0	0	0	0.166	0	0.167	0	0	0	0
lpar3	0.166	0	0	0	0	0	0.166	0	0.171	0	0	0	0
s1pr2	0.227	0	0	0	0	0	0.227	0	0.228	0	0	0	0
raph1	0.228	0	0	0	0	0	0.228	0	0.234	0	0	0	0
arap2	0.23	0	0	0	0	0	0.23	0	0.237	0	0	0	0
plcb1	0.166	0	0	0	0	0	0.166	0	0.168	0	0	0	0
eps8l1	0.166	0	0	0	0	0	0.166	0	0.167	0	0	0	0
diaph3	0.167	0	0	0	0	0	0.167	0	0.167	0	0	0	0
ppp1cb	0.168	0	0	0	0	0	0.168	0	0.169	0	0	0	0
or4f6	0.225	0	0	0	0	0	0.225	0	0.22	0	0	0	0

gna13	0.168	0	0	0	0	0	0.168	0	0.168	0	0	0	0
pip5k1b	0.225	0	0	0	0	0	0.225	0	0.224	0	0	0	0
nol12	0.168	0	0	0	0	0	0.168	0	0.182	0	0	0	0
rabif	0.165	0	0	0	0	0	0.165	0	0.166	0	0	0	0
rac1p2	0.231	0	0	0	0	0	0.231	0	0.235	0	0	0	0
sec14l4	0.164	0	0	0	0	0	0.164	0	0.161	0	0	0	0
plekha2	0.231	0	0	0	0	0	0.231	0	0.229	0	0	0	0
capn6	0.164	0	0	0	0	0	0.164	0	0.162	0	0	0	0
usp6	0.164	0	0	0	0	0	0.164	0	0.179	0	0	0	0
dock3	0.233	0	0	0	0	0	0.233	0	0.229	0	0	0	0
pik3cd	0.163	0	0	0	0	0	0.163	0	0.162	0	0	0	0
dapp1	0.164	0	0	0	0	0	0.164	0	0.164	0	0	0	0
tspan5	0.231	0	0	0	0	0	0.231	0	0.227	0	0	0	0
capns1	0.105	0	0	0	0	0	0.105	0	0.112	0	0	0	0
plcb3	0.165	0	0	0	0	0	0.165	0	0.166	0	0	0	0
rac1p3	0.231	0	0	0	0	0	0.231	0	0.235	0	0	0	0
rac1p5	0.231	0	0	0	0	0	0.231	0	0.235	0	0	0	0
rac1p4	0.231	0	0	0	0	0	0.231	0	0.235	0	0	0	0
nox1	0.131	0	0	0	0	0	0.131	0	0.129	0	0	0	0
cep85l	0.138	0	0	0	0	0	0.138	0	0.136	0	0	0	0
appl2	0.117	0	0	0	0	0	0.117	0	0.127	0	0	0	0
gsn	0.117	0	0	0	0	0	0.117	0	0.117	0	0	0	0
mtmr1	0.118	0	0	0	0	0	0.118	0	0.12	0	0	0	0
ccdc41	0.117	0	0	0	0	0	0.117	0	0.127	0	0	0	0
arhgef3	0.117	0	0	0	0	0	0.117	0	0.116	0	0	0	0
eps15	0.116	0	0	0	0	0	0.116	0	0.139	0	0	0	0
adam20p1	0.116	0	0	0	0	0	0.116	0	0.115	0	0	0	0
rab33b	0.117	0	0	0	0	0	0.117	0	0.128	0	0	0	0
rab6b	0.118	0	0	0	0	0	0.118	0	0.126	0	0	0	0
sphk2	0.118	0	0	0	0	0	0.118	0	0.119	0	0	0	0
ophn1	0.118	0	0	0	0	0	0.118	0	0.126	0	0	0	0
gnai2	0.118	0	0	0	0	0	0.118	0	0.119	0	0	0	0
rapgefl1	0.118	0	0	0	0	0	0.118	0	0.12	0	0	0	0
g3bp1	0.118	0	0	0	0	0	0.118	0	0.128	0	0	0	0
scyl2	0.118	0	0	0	0	0	0.118	0	0.12	0	0	0	0
rxfp4	0.118	0	0	0	0	0	0.118	0	0.116	0	0	0	0
bri3	0.118	0	0	0	0	0	0.118	0	0.123	0	0	0	0
elmod1	0.116	0	0	0	0	0	0.116	0	0.123	0	0	0	0
rps6kc1	0.116	0	0	0	0	0	0.116	0	0.116	0	0	0	0
coro1b	0.113	0	0	0	0	0	0.113	0	0.113	0	0	0	0
gmfg	0.114	0	0	0	0	0	0.114	0	0.116	0	0	0	0

trav6	0.114	0	0	0	0	0	0.114	0	0.117	0	0	0	0
tuba8	0.113	0	0	0	0	0	0.113	0	0.119	0	0	0	0
nol6	0.113	0	0	0	0	0	0.113	0	0.123	0	0	0	0
eif3j	0.113	0	0	0	0	0	0.113	0	0.115	0	0	0	0
rab5b	0.113	0	0	0	0	0	0.113	0	0.133	0	0	0	0
ptpn3	0.114	0	0	0	0	0	0.114	0	0.12	0	0	0	0
pik3c2g	0.114	0	0	0	0	0	0.114	0	0.114	0	0	0	0
ndufaf5	0.116	0	0	0	0	0	0.116	0	0.119	0	0	0	0
cltcl1	0.116	0	0	0	0	0	0.116	0	0.121	0	0	0	0
prr5l	0.116	0	0	0	0	0	0.116	0	0.116	0	0	0	0
dcbld2	0.115	0	0	0	0	0	0.115	0	0.119	0	0	0	0
mir23b	0.115	0	0	0	0	0	0.115	0	0.119	0	0	0	0
emr3	0.114	0	0	0	0	0	0.114	0	0.114	0	0	0	0
gage2c	0.114	0	0	0	0	0	0.114	0	0.125	0	0	0	0
ptafr	0.119	0	0	0	0	0	0.119	0	0.117	0	0	0	0
tbc1d13	0.119	0	0	0	0	0	0.119	0	0.123	0	0	0	0
nrg3-as1	0.122	0	0	0	0	0	0.122	0	0.124	0	0	0	0
zswim8	0.122	0	0	0	0	0	0.122	0	0.124	0	0	0	0
fam65c	0.122	0	0	0	0	0	0.122	0	0.124	0	0	0	0
cdrt15p2	0.122	0	0	0	0	0	0.122	0	0.124	0	0	0	0
eif4g1	0.122	0	0	0	0	0	0.122	0	0.123	0	0	0	0
ptpn1	0.122	0	0	0	0	0	0.122	0	0.124	0	0	0	0
frmpd1	0.122	0	0	0	0	0	0.122	0	0.127	0	0	0	0
obscn	0.122	0	0	0	0	0	0.122	0	0.125	0	0	0	0
cass4	0.123	0	0	0	0	0	0.123	0	0.12	0	0	0	0
ppp1r8	0.123	0	0	0	0	0	0.123	0	0.124	0	0	0	0
rac2	0.539	0	0	0	0	0	0.539	0	0.532	0	0	0	0
arhgap19	0.124	0	0	0	0	0	0.124	0	0.127	0	0	0	0
spire1	0.124	0	0	0	0	0	0.124	0	0.125	0	0	0	0
gramd4	0.124	0	0	0	0	0	0.124	0	0.124	0	0	0	0
rgs14	0.123	0	0	0	0	0	0.123	0	0.128	0	0	0	0
clasrp	0.123	0	0	0	0	0	0.123	0	0.131	0	0	0	0
itgb4	0.123	0	0	0	0	0	0.123	0	0.124	0	0	0	0
sipa1l2	0.122	0	0	0	0	0	0.122	0	0.128	0	0	0	0
afap1l2	0.122	0	0	0	0	0	0.122	0	0.121	0	0	0	0
tpm3p4	0.12	0	0	0	0	0	0.12	0	0.118	0	0	0	0
tpm3p7	0.12	0	0	0	0	0	0.12	0	0.118	0	0	0	0
loc751603	0.12	0	0	0	0	0	0.12	0	0.118	0	0	0	0
tpm3p8	0.12	0	0	0	0	0	0.12	0	0.118	0	0	0	0
tpm3p6	0.12	0	0	0	0	0	0.12	0	0.118	0	0	0	0
macc1	0.12	0	0	0	0	0	0.12	0	0.123	0	0	0	0

ppp2r5c	0.12	0	0	0	0	0	0.12	0	0.126	0	0	0	0
loc751602	0.12	0	0	0	0	0	0.12	0	0.118	0	0	0	0
tpm3p5	0.12	0	0	0	0	0	0.12	0	0.118	0	0	0	0
efnb2	0.121	0	0	0	0	0	0.121	0	0.123	0	0	0	0
grb7	0.121	0	0	0	0	0	0.121	0	0.122	0	0	0	0
copz1	0.121	0	0	0	0	0	0.121	0	0.123	0	0	0	0
evl	0.121	0	0	0	0	0	0.121	0	0.125	0	0	0	0
myoz3	0.12	0	0	0	0	0	0.12	0	0.116	0	0	0	0
asb9	0.12	0	0	0	0	0	0.12	0	0.118	0	0	0	0
coro1a	0.12	0	0	0	0	0	0.12	0	0.121	0	0	0	0
specc1	0.113	0	0	0	0	0	0.113	0	0.12	0	0	0	0
arl8a	0.113	0	0	0	0	0	0.113	0	0.124	0	0	0	0
rgs11	0.108	0	0	0	0	0	0.108	0	0.109	0	0	0	0
myl5	0.108	0	0	0	0	0	0.108	0	0.106	0	0	0	0
lrrc4b	0.108	0	0	0	0	0	0.108	0	0.114	0	0	0	0
mir138-2	0.108	0	0	0	0	0	0.108	0	0.119	0	0	0	0
pabpc1	0.108	0	0	0	0	0	0.108	0	0.11	0	0	0	0
mkln1	0.108	0	0	0	0	0	0.108	0	0.113	0	0	0	0
gnb2l1	0.108	0	0	0	0	0	0.108	0	0.108	0	0	0	0
lox	0.108	0	0	0	0	0	0.108	0	0.113	0	0	0	0
nol8	0.108	0	0	0	0	0	0.108	0	0.118	0	0	0	0
epha3	0.108	0	0	0	0	0	0.108	0	0.112	0	0	0	0
farp2	0.109	0	0	0	0	0	0.109	0	0.114	0	0	0	0
cltb	0.109	0	0	0	0	0	0.109	0	0.116	0	0	0	0
paip1	0.109	0	0	0	0	0	0.109	0	0.111	0	0	0	0
myot	0.109	0	0	0	0	0	0.109	0	0.108	0	0	0	0
gbas	0.109	0	0	0	0	0	0.109	0	0.107	0	0	0	0
tns4	0.108	0	0	0	0	0	0.108	0	0.109	0	0	0	0
ap2a1	0.108	0	0	0	0	0	0.108	0	0.112	0	0	0	0
tuba1a	0.107	0	0	0	0	0	0.107	0	0.112	0	0	0	0
fabp3p2	0.107	0	0	0	0	0	0.107	0	0.107	0	0	0	0
agfg2	0.106	0	0	0	0	0	0.106	0	0.128	0	0	0	0
lrfn2	0.106	0	0	0	0	0	0.106	0	0.109	0	0	0	0
syne3	0.106	0	0	0	0	0	0.106	0	0.106	0	0	0	0
abhd4	0.106	0	0	0	0	0	0.106	0	0.113	0	0	0	0
pop7	0.106	0	0	0	0	0	0.106	0	0.11	0	0	0	0
stk35	0.106	0	0	0	0	0	0.106	0	0.102	0	0	0	0
rab15	0.106	0	0	0	0	0	0.106	0	0.111	0	0	0	0
rasgrf1	0.106	0	0	0	0	0	0.106	0	0.112	0	0	0	0
uri1	0.106	0	0	0	0	0	0.106	0	0.116	0	0	0	0
ap2m1	0.107	0	0	0	0	0	0.107	0	0.11	0	0	0	0

mecr	0.107	0	0	0	0	0	0.107	0	0.105	0	0	0	0
csnk1g2	0.107	0	0	0	0	0	0.107	0	0.106	0	0	0	0
pdgfd	0.107	0	0	0	0	0	0.107	0	0.107	0	0	0	0
akap6	0.107	0	0	0	0	0	0.107	0	0.112	0	0	0	0
grk6p1	0.106	0	0	0	0	0	0.106	0	0.107	0	0	0	0
ankrd30bl	0.107	0	0	0	0	0	0.107	0	0.109	0	0	0	0
ptprj	0.109	0	0	0	0	0	0.109	0	0.111	0	0	0	0
lrrc16a	0.109	0	0	0	0	0	0.109	0	0.105	0	0	0	0
dnah1	0.112	0	0	0	0	0	0.112	0	0.119	0	0	0	0
rell1	0.112	0	0	0	0	0	0.112	0	0.11	0	0	0	0
rell2	0.112	0	0	0	0	0	0.112	0	0.11	0	0	0	0
myh2	0.112	0	0	0	0	0	0.112	0	0.108	0	0	0	0
trdj3	0.111	0	0	0	0	0	0.111	0	0.111	0	0	0	0
myom1	0.111	0	0	0	0	0	0.111	0	0.114	0	0	0	0
cstf2	0.111	0	0	0	0	0	0.111	0	0.115	0	0	0	0
bmp6p1	0.111	0	0	0	0	0	0.111	0	0.105	0	0	0	0
tbc1d5	0.112	0	0	0	0	0	0.112	0	0.112	0	0	0	0
fscn3	0.112	0	0	0	0	0	0.112	0	0.119	0	0	0	0
cstf2t	0.113	0	0	0	0	0	0.113	0	0.114	0	0	0	0
pi4k2a	0.113	0	0	0	0	0	0.113	0	0.114	0	0	0	0
rgs2	0.113	0	0	0	0	0	0.113	0	0.114	0	0	0	0
efnb3	0.113	0	0	0	0	0	0.113	0	0.113	0	0	0	0
rab9a	0.112	0	0	0	0	0	0.112	0	0.12	0	0	0	0
xirp2	0.112	0	0	0	0	0	0.112	0	0.142	0	0	0	0
ighv2-70	0.112	0	0	0	0	0	0.112	0	0.113	0	0	0	0
snx4	0.111	0	0	0	0	0	0.111	0	0.113	0	0	0	0
ywhab	0.11	0	0	0	0	0	0.11	0	0.112	0	0	0	0
plce1	0.109	0	0	0	0	0	0.109	0	0.112	0	0	0	0
bai1	0.109	0	0	0	0	0	0.109	0	0.112	0	0	0	0
rabep1	0.11	0	0	0	0	0	0.11	0	0.117	0	0	0	0
rab22a	0.109	0	0	0	0	0	0.109	0	0.142	0	0	0	0
flnb	0.109	0	0	0	0	0	0.109	0	0.107	0	0	0	0
diaph2	0.109	0	0	0	0	0	0.109	0	0.112	0	0	0	0
myoz1	0.109	0	0	0	0	0	0.109	0	0.105	0	0	0	0
kiaa1377	0.11	0	0	0	0	0	0.11	0	0.109	0	0	0	0
dram1	0.11	0	0	0	0	0	0.11	0	0.111	0	0	0	0
relt	0.11	0	0	0	0	0	0.11	0	0.111	0	0	0	0
nkpd1	0.11	0	0	0	0	0	0.11	0	0.109	0	0	0	0
ppp1ca	0.11	0	0	0	0	0	0.11	0	0.112	0	0	0	0
golph3	0.11	0	0	0	0	0	0.11	0	0.122	0	0	0	0
rpsap14	0.11	0	0	0	0	0	0.11	0	0.115	0	0	0	0

stk3	0.11	0	0	0	0	0	0.11	0	0.114	0	0	0	0
rgs20	0.11	0	0	0	0	0	0.11	0	0.111	0	0	0	0
pfn2	0.124	0	0	0	0	0	0.124	0	0.127	0	0	0	0
grk4	0.119	0	0	0	0	0	0.119	0	0.117	0	0	0	0
sh3rf2	0.501	0	0	0	0	0	0.501	0	0.496	0	0	0	0
inf2	0.127	0	0	0	0	0	0.127	0	0.128	0	0	0	0
rasgrp1	0.127	0	0	0	0	0	0.127	0	0.129	0	0	0	0
nrbp1	0.128	0	0	0	0	0	0.128	0	0.129	0	0	0	0
ankfy1	0.128	0	0	0	0	0	0.128	0	0.129	0	0	0	0
rac3	0.499	0	0	0	0	0	0.499	0	0.494	0	0	0	0
dlc1	0.128	0	0	0	0	0	0.128	0	0.134	0	0	0	0
snx8	0.128	0	0	0	0	0	0.128	0	0.133	0	0	0	0
dync1li1	0.127	0	0	0	0	0	0.127	0	0.126	0	0	0	0
mllt4	0.127	0	0	0	0	0	0.127	0	0.135	0	0	0	0
sema4g	0.125	0	0	0	0	0	0.125	0	0.129	0	0	0	0
unkl	0.517	0	0	0	0	0	0.517	0	0.51	0	0	0	0
rras	0.125	0	0	0	0	0	0.125	0	0.128	0	0	0	0
rufy1	0.125	0	0	0	0	0	0.125	0	0.131	0	0	0	0
fpr1	0.126	0	0	0	0	0	0.126	0	0.124	0	0	0	0
rab43	0.126	0	0	0	0	0	0.126	0	0.131	0	0	0	0
sept1	0.126	0	0	0	0	0	0.126	0	0.127	0	0	0	0
stx17	0.126	0	0	0	0	0	0.126	0	0.135	0	0	0	0
efs	0.128	0	0	0	0	0	0.128	0	0.131	0	0	0	0
baiap2l2	0.459	0	0	0	0	0	0.459	0	0.454	0	0	0	0
wdr1	0.13	0	0	0	0	0	0.13	0	0.135	0	0	0	0
gnb2	0.13	0	0	0	0	0	0.13	0	0.131	0	0	0	0
rab3d	0.13	0	0	0	0	0	0.13	0	0.135	0	0	0	0
mcidas	0.13	0	0	0	0	0	0.13	0	0.128	0	0	0	0
rab27b	0.131	0	0	0	0	0	0.131	0	0.13	0	0	0	0
srgap3	0.418	0	0	0	0	0	0.418	0	0.417	0	0	0	0
diaph1	0.131	0	0	0	0	0	0.131	0	0.133	0	0	0	0
dock2	0.424	0	0	0	0	0	0.424	0	0.418	0	0	0	0
tbc1d2	0.442	0	0	0	0	0	0.442	0	0.44	0	0	0	0
ect2	0.13	0	0	0	0	0	0.13	0	0.131	0	0	0	0
prkd1	0.129	0	0	0	0	0	0.129	0	0.129	0	0	0	0
lclat1	0.129	0	0	0	0	0	0.129	0	0.128	0	0	0	0
sh3bp1	0.456	0	0	0	0	0	0.456	0	0.454	0	0	0	0
sec14l2	0.129	0	0	0	0	0	0.129	0	0.129	0	0	0	0
unc5d	0.129	0	0	0	0	0	0.129	0	0.135	0	0	0	0
cyfip1	0.452	0	0	0	0	0	0.452	0	0.446	0	0	0	0
mtmr14	0.13	0	0	0	0	0	0.13	0	0.133	0	0	0	0

arl6	0.125	0	0	0	0	0	0.125	0	0.133	0	0	0	0
dync1li2	0.128	0	0	0	0	0	0.128	0	0.128	0	0	0	0
ralgps1	0.125	0	0	0	0	0	0.125	0	0.126	0	0	0	0
arhgdig	0.125	0	0	0	0	0	0.125	0	0.128	0	0	0	0
mark2	0.125	0	0	0	0	0	0.125	0	0.13	0	0	0	0
rilpl2	0.53	0	0	0	0	0	0.53	0	0.523	0	0	0	0
mob2	0.124	0	0	0	0	0	0.124	0	0.128	0	0	0	0
rapgef2	0.124	0	0	0	0	0	0.124	0	0.131	0	0	0	0
mtss1l	0.536	0	0	0	0	0	0.536	0	0.529	0	0	0	0
myl12b	0.125	0	0	0	0	0	0.125	0	0.124	0	0	0	0
ephb3	0.125	0	0	0	0	0	0.125	0	0.128	0	0	0	0
mut	0	0	0	0.113	0.113	0	0	0	0	0.113	0	0	0
nphp3	0	0	0	0.113	0.113	0	0	0	0	0.113	0	0	0
wnt9b	0	0	0	0.113	0.113	0	0	0	0	0.113	0	0	0
chid1	0	0	0	0.121	0.121	0	0	0	0	0.121	0	0	0
mgr4	0	0	0	0.119	0.119	0	0	0	0	0.119	0	0	0
ca11	0	0	0	0.113	0.113	0	0	0	0	0.113	0	0	0
ptos1	0	0	0	0.122	0.122	0	0	0	0	0.122	0	0	0
hpt	0	0	0	0.113	0.113	0	0	0	0	0.113	0	0	0
ext2	0	0	0	0.113	0.113	0	0	0	0	0.113	0	0	0
efcab4a	0	0	0	0.113	0.113	0	0	0	0	0.113	0	0	0
pgls	0	0	0	0.113	0.113	0	0	0	0	0.113	0	0	0
dlat	0	0	0	0.119	0.119	0	0	0	0	0.119	0	0	0
itpkb	0	0	0	0.113	0.113	0	0	0	0	0.113	0	0	0
avpi1	0	0	0	0.121	0.121	0	0	0	0	0.121	0	0	0
snord12c	0	0	0	0.113	0.113	0	0	0	0	0.113	0	0	0
myo15a	0	0	0	0.113	0.113	0	0	0	0	0.113	0	0	0
cys1	0	0	0	0.113	0.113	0	0	0	0	0.113	0	0	0
ankrd49	0	0	0	0.113	0.113	0	0	0	0	0.113	0	0	0
snora69	0	0	0	0.112	0.112	0	0	0	0	0.112	0	0	0
cox7a2p2	0	0	0	0.121	0.121	0	0	0	0	0.121	0	0	0
kcna1	0	0	0	0.112	0.112	0	0	0	0	0.112	0	0	0
kcnip2	0	0	0	0.112	0.112	0	0	0	0	0.112	0	0	0
pate4	0	0	0	0.12	0.12	0	0	0	0	0.12	0	0	0
pth2r	0	0	0	0.121	0.121	0	0	0	0	0.121	0	0	0
igkv1or2-3	0	0	0	0.112	0.112	0	0	0	0	0.112	0	0	0
tff3	0	0	0	0.112	0.112	0	0	0	0	0.112	0	0	0
cox7a2	0	0	0	0.112	0.112	0	0	0	0	0.112	0	0	0
ighj6	0	0	0	0.121	0.121	0	0	0	0	0.121	0	0	0
s100a3	0	0	0	0.116	0.116	0	0	0	0	0.116	0	0	0
aif1l	0	0	0	0.113	0.113	0	0	0	0	0.113	0	0	0

akr1b1p2	0	0	0	0.112	0.112	0	0	0	0	0.112	0	0	0
folr2	0	0	0	0.12	0.12	0	0	0	0	0.12	0	0	0
tfb1m	0	0	0	0.12	0.12	0	0	0	0	0.12	0	0	0
pygl	0	0	0	0.112	0.112	0	0	0	0	0.112	0	0	0
gusbp1	0	0	0	0.113	0.113	0	0	0	0	0.113	0	0	0
kcnj5	0	0	0	0.119	0.119	0	0	0	0	0.119	0	0	0
pkd1	0	0	0	0.113	0.113	0	0	0	0	0.113	0	0	0
usp17l27	0	0	0	0.113	0.113	0	0	0	0	0.113	0	0	0
prm1	0	0	0	0.119	0.119	0	0	0	0	0.119	0	0	0
rbmy2tp	0	0	0	0.119	0.119	0	0	0	0	0.119	0	0	0
elk1	0	0	0	0.122	0.122	0	0	0	0	0.122	0	0	0
linc00035	0	0	0	0.113	0.113	0	0	0	0	0.113	0	0	0
pdhb	0	0	0	0.113	0.113	0	0	0	0	0.113	0	0	0
slc5a2	0	0	0	0.119	0.119	0	0	0	0	0.119	0	0	0
defb106b	0	0	0	0.113	0.113	0	0	0	0	0.113	0	0	0
pds5a	0	0	0	0.114	0.114	0	0	0	0	0.114	0	0	0
amy1c	0	0	0	0.114	0.114	0	0	0	0	0.114	0	0	0
ggt1	0	0	0	0.114	0.114	0	0	0	0	0.114	0	0	0
fam120b	0	0	0	0.114	0.114	0	0	0	0	0.114	0	0	0
kcng4	0	0	0	0.119	0.119	0	0	0	0	0.119	0	0	0
pgk1	0	0	0	0.117	0.117	0	0	0	0	0.117	0	0	0
azin1	0	0	0	0.113	0.113	0	0	0	0	0.113	0	0	0
trim74	0	0	0	0.113	0.113	0	0	0	0	0.113	0	0	0
trim73	0	0	0	0.113	0.113	0	0	0	0	0.113	0	0	0
glat	0	0	0	0.113	0.113	0	0	0	0	0.113	0	0	0
amyp1	0	0	0	0.113	0.113	0	0	0	0	0.113	0	0	0
orai2	0	0	0	0.122	0.122	0	0	0	0	0.122	0	0	0
chst6	0	0	0	0.113	0.113	0	0	0	0	0.113	0	0	0
myp11	0	0	0	0.119	0.119	0	0	0	0	0.119	0	0	0
emx2os	0	0	0	0.117	0.117	0	0	0	0	0.117	0	0	0
best4	0	0	0	0.117	0.117	0	0	0	0	0.117	0	0	0
slc32a1	0	0	0	0.113	0.113	0	0	0	0	0.113	0	0	0
pkhd1	0	0	0	0.113	0.113	0	0	0	0	0.113	0	0	0
amy1a	0	0	0	0.119	0.119	0	0	0	0	0.119	0	0	0
pdxk	0	0	0	0.122	0.122	0	0	0	0	0.122	0	0	0
gtsf1l	0	0	0	0.119	0.119	0	0	0	0	0.119	0	0	0
rn7sk	0	0	0	0.121	0.121	0	0	0	0	0.121	0	0	0
bcyrn1	0	0	0	0.113	0.113	0	0	0	0	0.113	0	0	0
golim4	0	0	0	0.121	0.121	0	0	0	0	0.121	0	0	0
sfpq	0	0	0	0.117	0.117	0	0	0	0	0.117	0	0	0
igkv2or2-1	0	0	0	0.112	0.112	0	0	0	0	0.112	0	0	0

igkv1or2-6	0	0	0	0.112	0.112	0	0	0	0	0.112	0	0	0
acot11	0	0	0	0.12	0.12	0	0	0	0	0.12	0	0	0
kcna3	0	0	0	0.12	0.12	0	0	0	0	0.12	0	0	0
slitrk6	0	0	0	0.111	0.111	0	0	0	0	0.111	0	0	0
rfk	0	0	0	0.111	0.111	0	0	0	0	0.111	0	0	0
akr1b1	0	0	0	0.111	0.111	0	0	0	0	0.111	0	0	0
fsd1l	0	0	0	0.111	0.111	0	0	0	0	0.111	0	0	0
zp4	0	0	0	0.111	0.111	0	0	0	0	0.111	0	0	0
gprc6a	0	0	0	0.12	0.12	0	0	0	0	0.12	0	0	0
pou2f2	0	0	0	0.112	0.112	0	0	0	0	0.112	0	0	0
glrx	0	0	0	0.112	0.112	0	0	0	0	0.112	0	0	0
efcab4b	0	0	0	0.112	0.112	0	0	0	0	0.112	0	0	0
slc4a11	0	0	0	0.12	0.12	0	0	0	0	0.12	0	0	0
trnt1	0	0	0	0.112	0.112	0	0	0	0	0.112	0	0	0
nrbp2	0	0	0	0.12	0.12	0	0	0	0	0.12	0	0	0
bp6	0	0	0	0.12	0.12	0	0	0	0	0.12	0	0	0
tuba3d	0	0	0	0.112	0.112	0	0	0	0	0.112	0	0	0
alg14	0	0	0	0.111	0.111	0	0	0	0	0.111	0	0	0
pdk3	0	0	0	0.111	0.111	0	0	0	0	0.111	0	0	0
caps2	0	0	0	0.111	0.111	0	0	0	0	0.111	0	0	0
defb104b	0	0	0	0.111	0.111	0	0	0	0	0.111	0	0	0
sec24d	0	0	0	0.116	0.116	0	0	0	0	0.116	0	0	0
znf181	0	0	0	0.12	0.12	0	0	0	0	0.12	0	0	0
gpr20	0	0	0	0.12	0.12	0	0	0	0	0.12	0	0	0
h1fx	0	0	0	0.111	0.111	0	0	0	0	0.111	0	0	0
slc25a1	0	0	0	0.111	0.111	0	0	0	0	0.111	0	0	0
trim41	0	0	0	0.12	0.12	0	0	0	0	0.12	0	0	0
cna1	0	0	0	0.12	0.12	0	0	0	0	0.12	0	0	0
lyz	0	0	0	0.116	0.116	0	0	0	0	0.116	0	0	0
pthlh	0	0	0	0.111	0.111	0	0	0	0	0.111	0	0	0
yars2	0	0	0	0.111	0.111	0	0	0	0	0.111	0	0	0
ush1c	0	0	0	0.12	0.12	0	0	0	0	0.12	0	0	0
ptcsc1	0	0	0	0.111	0.111	0	0	0	0	0.111	0	0	0
amy2b	0	0	0	0.122	0.122	0	0	0	0	0.122	0	0	0
pde4c	0	0	0	0.12	0.12	0	0	0	0	0.12	0	0	0
lenep	0	0	0	0.112	0.112	0	0	0	0	0.112	0	0	0
glrx2	0	0	0	0.112	0.112	0	0	0	0	0.112	0	0	0
mab21l1	0	0	0	0.112	0.112	0	0	0	0	0.112	0	0	0
gss	0	0	0	0.112	0.112	0	0	0	0	0.112	0	0	0
pdzk1ip1	0	0	0	0.12	0.12	0	0	0	0	0.12	0	0	0
scamp2	0	0	0	0.12	0.12	0	0	0	0	0.12	0	0	0

srp14	0	0	0	0.12	0.12	0	0	0	0	0.12	0	0	0
slc16a4	0	0	0	0.117	0.117	0	0	0	0	0.117	0	0	0
pdhx	0	0	0	0.112	0.112	0	0	0	0	0.112	0	0	0
cherp	0	0	0	0.12	0.12	0	0	0	0	0.12	0	0	0
csn1s2bp	0	0	0	0.112	0.112	0	0	0	0	0.112	0	0	0
ptmap1	0	0	0	0.112	0.112	0	0	0	0	0.112	0	0	0
ptmap5	0	0	0	0.112	0.112	0	0	0	0	0.112	0	0	0
calb2	0	0	0	0.112	0.112	0	0	0	0	0.112	0	0	0
nt5e	0	0	0	0.112	0.112	0	0	0	0	0.112	0	0	0
dmrt3	0	0	0	0.12	0.12	0	0	0	0	0.12	0	0	0
atp7b	0	0	0	0.112	0.112	0	0	0	0	0.112	0	0	0
ndst1	0	0	0	0.112	0.112	0	0	0	0	0.112	0	0	0
zhx3	0	0	0	0.112	0.112	0	0	0	0	0.112	0	0	0
bicc1	0	0	0	0.117	0.117	0	0	0	0	0.117	0	0	0
lsm4	0	0	0	0.12	0.12	0	0	0	0	0.12	0	0	0
anxa11	0	0	0	0.112	0.112	0	0	0	0	0.112	0	0	0
adh5p2	0	0	0	0.112	0.112	0	0	0	0	0.112	0	0	0
epg5	0	0	0	0.12	0.12	0	0	0	0	0.12	0	0	0
slc46a1	0	0	0	0.12	0.12	0	0	0	0	0.12	0	0	0
psg5	0	0	0	0.112	0.112	0	0	0	0	0.112	0	0	0
gucy2ep	0	0	0	0.121	0.121	0	0	0	0	0.121	0	0	0
adh5p4	0	0	0	0.112	0.112	0	0	0	0	0.112	0	0	0
tor3a	0	0	0	0.112	0.112	0	0	0	0	0.112	0	0	0
aco1	0	0	0	0.112	0.112	0	0	0	0	0.112	0	0	0
or7e66p	0	0	0	0.122	0.122	0	0	0	0	0.122	0	0	0
or7e22p	0	0	0	0.122	0.122	0	0	0	0	0.122	0	0	0
myo3b	0	0	0	0.112	0.112	0	0	0	0	0.112	0	0	0
thoc5	0	0	0	0.112	0.112	0	0	0	0	0.112	0	0	0
slc2a13	0	0	0	0.112	0.112	0	0	0	0	0.112	0	0	0
chiap2	0	0	0	0.12	0.12	0	0	0	0	0.12	0	0	0
etf1	0	0	0	0.121	0.121	0	0	0	0	0.121	0	0	0
b3gnt5	0	0	0	0.114	0.114	0	0	0	0	0.114	0	0	0
ppp1r2p1	0	0	0	0.117	0.117	0	0	0	0	0.117	0	0	0
acot12	0	0	0	0.118	0.118	0	0	0	0	0.118	0	0	0
heatr1	0	0	0	0.116	0.116	0	0	0	0	0.116	0	0	0
oaz1	0	0	0	0.118	0.118	0	0	0	0	0.118	0	0	0
loc642355	0	0	0	0.118	0.118	0	0	0	0	0.118	0	0	0
cadps	0	0	0	0.118	0.118	0	0	0	0	0.118	0	0	0
myp18	0	0	0	0.116	0.116	0	0	0	0	0.116	0	0	0
hiat1	0	0	0	0.12	0.12	0	0	0	0	0.12	0	0	0
kcna2	0	0	0	0.118	0.118	0	0	0	0	0.118	0	0	0

trnan3	0	0	0	0.116	0.116	0	0	0	0	0.116	0	0	0
trnan2	0	0	0	0.116	0.116	0	0	0	0	0.116	0	0	0
stim2	0	0	0	0.118	0.118	0	0	0	0	0.118	0	0	0
pabpc1p4	0	0	0	0.116	0.116	0	0	0	0	0.116	0	0	0
rnu2-3p	0	0	0	0.116	0.116	0	0	0	0	0.116	0	0	0
sczd1	0	0	0	0.116	0.116	0	0	0	0	0.116	0	0	0
aimp1	0	0	0	0.117	0.117	0	0	0	0	0.117	0	0	0
pafah1b1p2	0	0	0	0.115	0.115	0	0	0	0	0.115	0	0	0
epb41	0	0	0	0.121	0.121	0	0	0	0	0.121	0	0	0
tpmtp1	0	0	0	0.122	0.122	0	0	0	0	0.122	0	0	0
kcnb1	0	0	0	0.118	0.118	0	0	0	0	0.118	0	0	0
slc2a5	0	0	0	0.115	0.115	0	0	0	0	0.115	0	0	0
tdo2	0	0	0	0.115	0.115	0	0	0	0	0.115	0	0	0
tmc2	0	0	0	0.121	0.121	0	0	0	0	0.121	0	0	0
snrpc	0	0	0	0.115	0.115	0	0	0	0	0.115	0	0	0
sln	0	0	0	0.116	0.116	0	0	0	0	0.116	0	0	0
ogdh	0	0	0	0.121	0.121	0	0	0	0	0.121	0	0	0
tec	0	0	0	0.115	0.115	0	0	0	0	0.115	0	0	0
cerk	0	0	0	0.118	0.118	0	0	0	0	0.118	0	0	0
phtf2	0	0	0	0.121	0.121	0	0	0	0	0.121	0	0	0
pafah1b1p1	0	0	0	0.115	0.115	0	0	0	0	0.115	0	0	0
si	0	0	0	0.115	0.115	0	0	0	0	0.115	0	0	0
nipal1	0	0	0	0.116	0.116	0	0	0	0	0.116	0	0	0
kcnj9	0	0	0	0.115	0.115	0	0	0	0	0.115	0	0	0
gpr98	0	0	0	0.121	0.121	0	0	0	0	0.121	0	0	0
pga5	0	0	0	0.116	0.116	0	0	0	0	0.116	0	0	0
ampd3	0	0	0	0.117	0.117	0	0	0	0	0.117	0	0	0
c2orf61	0	0	0	0.117	0.117	0	0	0	0	0.117	0	0	0
pi4k2b	0	0	0	0.122	0.122	0	0	0	0	0.122	0	0	0
rps26	0	0	0	0.116	0.116	0	0	0	0	0.116	0	0	0
kcnd1	0	0	0	0.116	0.116	0	0	0	0	0.116	0	0	0
me3	0	0	0	0.116	0.116	0	0	0	0	0.116	0	0	0
awat1	0	0	0	0.116	0.116	0	0	0	0	0.116	0	0	0
prpsap2	0	0	0	0.117	0.117	0	0	0	0	0.117	0	0	0
pde5a	0	0	0	0.117	0.117	0	0	0	0	0.117	0	0	0
gng11	0	0	0	0.116	0.116	0	0	0	0	0.116	0	0	0
sca30	0	0	0	0.116	0.116	0	0	0	0	0.116	0	0	0
b4galt5	0	0	0	0.122	0.122	0	0	0	0	0.122	0	0	0
dyt17	0	0	0	0.122	0.122	0	0	0	0	0.122	0	0	0
uox	0	0	0	0.117	0.117	0	0	0	0	0.117	0	0	0
apol5	0	0	0	0.116	0.116	0	0	0	0	0.116	0	0	0

aadac	0	0	0	0.116	0.116	0	0	0	0	0.116	0	0	0
vta1	0	0	0	0.116	0.116	0	0	0	0	0.116	0	0	0
bckdk	0	0	0	0.122	0.122	0	0	0	0	0.122	0	0	0
dgat2l6	0	0	0	0.116	0.116	0	0	0	0	0.116	0	0	0
alpl	0	0	0	0.116	0.116	0	0	0	0	0.116	0	0	0
pdia2	0	0	0	0.118	0.118	0	0	0	0	0.118	0	0	0
anxa1	0	0	0	0.118	0.118	0	0	0	0	0.118	0	0	0
pou6f2	0	0	0	0.116	0.116	0	0	0	0	0.116	0	0	0
timm13	0	0	0	0.116	0.116	0	0	0	0	0.116	0	0	0
dnajc11	0	0	0	0.121	0.121	0	0	0	0	0.121	0	0	0
eif5al1	0	0	0	0.116	0.116	0	0	0	0	0.116	0	0	0
kcnk16	0	0	0	0.118	0.118	0	0	0	0	0.118	0	0	0
hrtrt1	0	0	0	0.116	0.116	0	0	0	0	0.116	0	0	0
ndufc1	0	0	0	0.116	0.116	0	0	0	0	0.116	0	0	0
znf18	0	0	0	0.118	0.118	0	0	0	0	0.118	0	0	0
guca2b	0	0	0	0.121	0.121	0	0	0	0	0.121	0	0	0
ndufb5	0	0	0	0.116	0.116	0	0	0	0	0.116	0	0	0
gje1	0	0	0	0.121	0.121	0	0	0	0	0.121	0	0	0
ndufb1	0	0	0	0.116	0.116	0	0	0	0	0.116	0	0	0
eif2b4	0	0	0	0.121	0.121	0	0	0	0	0.121	0	0	0
ptrh1	0	0	0	0.115	0.115	0	0	0	0	0.115	0	0	0
loc440895	0	0	0	0.118	0.118	0	0	0	0	0.118	0	0	0
kcnk3	0	0	0	0.117	0.117	0	0	0	0	0.117	0	0	0
samd9l	0	0	0	0.122	0.122	0	0	0	0	0.122	0	0	0
trnal2	0	0	0	0.117	0.117	0	0	0	0	0.117	0	0	0
acot8	0	0	0	0.119	0.119	0	0	0	0	0.119	0	0	0
chchd4	0	0	0	0.114	0.114	0	0	0	0	0.114	0	0	0
gabrr2	0	0	0	0.114	0.114	0	0	0	0	0.114	0	0	0
hmgcs1	0	0	0	0.119	0.119	0	0	0	0	0.119	0	0	0
ppp2ca	0	0	0	0.119	0.119	0	0	0	0	0.119	0	0	0
trappc1	0	0	0	0.122	0.122	0	0	0	0	0.122	0	0	0
pitpna	0	0	0	0.114	0.114	0	0	0	0	0.114	0	0	0
slc18b1	0	0	0	0.114	0.114	0	0	0	0	0.114	0	0	0
cca1	0	0	0	0.114	0.114	0	0	0	0	0.114	0	0	0
acy1	0	0	0	0.114	0.114	0	0	0	0	0.114	0	0	0
krt3	0	0	0	0.119	0.119	0	0	0	0	0.119	0	0	0
slco4a1	0	0	0	0.114	0.114	0	0	0	0	0.114	0	0	0
dapl1	0	0	0	0.119	0.119	0	0	0	0	0.119	0	0	0
phospho1	0	0	0	0.122	0.122	0	0	0	0	0.122	0	0	0
slc11a2	0	0	0	0.114	0.114	0	0	0	0	0.114	0	0	0
seh1l	0	0	0	0.117	0.117	0	0	0	0	0.117	0	0	0

slc19a2	0	0	0	0.119	0.119	0	0	0	0	0.119	0	0	0
anxa2	0	0	0	0.117	0.117	0	0	0	0	0.117	0	0	0
nags	0	0	0	0.117	0.117	0	0	0	0	0.117	0	0	0
ogt	0	0	0	0.119	0.119	0	0	0	0	0.119	0	0	0
kcnk15	0	0	0	0.119	0.119	0	0	0	0	0.119	0	0	0
mbs2	0	0	0	0.114	0.114	0	0	0	0	0.114	0	0	0
gmpr2	0	0	0	0.114	0.114	0	0	0	0	0.114	0	0	0
otud1	0	0	0	0.114	0.114	0	0	0	0	0.114	0	0	0
anapc13	0	0	0	0.114	0.114	0	0	0	0	0.114	0	0	0
hsd3b7	0	0	0	0.119	0.119	0	0	0	0	0.119	0	0	0
ppp1r14a	0	0	0	0.114	0.114	0	0	0	0	0.114	0	0	0
kcns2	0	0	0	0.117	0.117	0	0	0	0	0.117	0	0	0
btf3p11	0	0	0	0.117	0.117	0	0	0	0	0.117	0	0	0
slc4a3	0	0	0	0.114	0.114	0	0	0	0	0.114	0	0	0
aoc4p	0	0	0	0.114	0.114	0	0	0	0	0.114	0	0	0
uqcrb	0	0	0	0.121	0.121	0	0	0	0	0.121	0	0	0
ccdc37	0	0	0	0.114	0.114	0	0	0	0	0.114	0	0	0
cdh23	0	0	0	0.118	0.118	0	0	0	0	0.118	0	0	0
foxi1	0	0	0	0.118	0.118	0	0	0	0	0.118	0	0	0
nt5c	0	0	0	0.118	0.118	0	0	0	0	0.118	0	0	0
slc36a3	0	0	0	0.115	0.115	0	0	0	0	0.115	0	0	0
c1orf85	0	0	0	0.115	0.115	0	0	0	0	0.115	0	0	0
retsat	0	0	0	0.115	0.115	0	0	0	0	0.115	0	0	0
nme8	0	0	0	0.115	0.115	0	0	0	0	0.115	0	0	0
dbt	0	0	0	0.115	0.115	0	0	0	0	0.115	0	0	0
btg2	0	0	0	0.121	0.121	0	0	0	0	0.121	0	0	0
rab8b	0	0	0	0.122	0.122	0	0	0	0	0.122	0	0	0
igjp1	0	0	0	0.115	0.115	0	0	0	0	0.115	0	0	0
coq2	0	0	0	0.121	0.121	0	0	0	0	0.121	0	0	0
hivep1	0	0	0	0.116	0.116	0	0	0	0	0.116	0	0	0
slc22a14	0	0	0	0.115	0.115	0	0	0	0	0.115	0	0	0
wdr13	0	0	0	0.115	0.115	0	0	0	0	0.115	0	0	0
best3	0	0	0	0.115	0.115	0	0	0	0	0.115	0	0	0
cst4	0	0	0	0.115	0.115	0	0	0	0	0.115	0	0	0
myo15b	0	0	0	0.118	0.118	0	0	0	0	0.118	0	0	0
cbln4	0	0	0	0.116	0.116	0	0	0	0	0.116	0	0	0
mogat1	0	0	0	0.119	0.119	0	0	0	0	0.119	0	0	0
ppp3ca	0	0	0	0.115	0.115	0	0	0	0	0.115	0	0	0
cabp4	0	0	0	0.119	0.119	0	0	0	0	0.119	0	0	0
cnga2	0	0	0	0.114	0.114	0	0	0	0	0.114	0	0	0
scgn	0	0	0	0.114	0.114	0	0	0	0	0.114	0	0	0

atp7a	0	0	0	0.114	0.114	0	0	0	0	0.114	0	0	0
pim1	0	0	0	0.121	0.121	0	0	0	0	0.121	0	0	0
fam69c	0	0	0	0.115	0.115	0	0	0	0	0.115	0	0	0
colgalt2	0	0	0	0.115	0.115	0	0	0	0	0.115	0	0	0
mcoln2	0	0	0	0.122	0.122	0	0	0	0	0.122	0	0	0
etfa	0	0	0	0.121	0.121	0	0	0	0	0.121	0	0	0
pdlim2	0	0	0	0.115	0.115	0	0	0	0	0.115	0	0	0
slc6a16	0	0	0	0.115	0.115	0	0	0	0	0.115	0	0	0
gnpat	0	0	0	0.115	0.115	0	0	0	0	0.115	0	0	0
pou4f2	0	0	0	0.116	0.116	0	0	0	0	0.116	0	0	0
tcp10l	0	0	0	0.122	0.122	0	0	0	0	0.122	0	0	0
kcnt1	0	0	0	0.107	0.107	0	0	0	0	0.107	0	0	0
ttc4p1	0	0	0	0.103	0.103	0	0	0	0	0.103	0	0	0
aox2p	0	0	0	0.103	0.103	0	0	0	0	0.103	0	0	0
dyx3	0	0	0	0.103	0.103	0	0	0	0	0.103	0	0	0
cant1	0	0	0	0.103	0.103	0	0	0	0	0.103	0	0	0
sbdsp1	0	0	0	0.103	0.103	0	0	0	0	0.103	0	0	0
mlycd	0	0	0	0.103	0.103	0	0	0	0	0.103	0	0	0
s100a6	0	0	0	0.103	0.103	0	0	0	0	0.103	0	0	0
tpi1p2	0	0	0	0.103	0.103	0	0	0	0	0.103	0	0	0
tpi1p1	0	0	0	0.103	0.103	0	0	0	0	0.103	0	0	0
nphs2	0	0	0	0.103	0.103	0	0	0	0	0.103	0	0	0
galk2	0	0	0	0.103	0.103	0	0	0	0	0.103	0	0	0
rnu6-50p	0	0	0	0.103	0.103	0	0	0	0	0.103	0	0	0
pdk2	0	0	0	0.104	0.104	0	0	0	0	0.104	0	0	0
znf72p	0	0	0	0.104	0.104	0	0	0	0	0.104	0	0	0
znf70	0	0	0	0.104	0.104	0	0	0	0	0.104	0	0	0
znf69	0	0	0	0.104	0.104	0	0	0	0	0.104	0	0	0
gtpbp10	0	0	0	0.104	0.104	0	0	0	0	0.104	0	0	0
bloc1s3	0	0	0	0.103	0.103	0	0	0	0	0.103	0	0	0
slc25a13	0	0	0	0.103	0.103	0	0	0	0	0.103	0	0	0
lco	0	0	0	0.103	0.103	0	0	0	0	0.103	0	0	0
alppl2	0	0	0	0.103	0.103	0	0	0	0	0.103	0	0	0
sh2d4a	0	0	0	0.103	0.103	0	0	0	0	0.103	0	0	0
rnr5	0	0	0	0.103	0.103	0	0	0	0	0.103	0	0	0
rnr3	0	0	0	0.103	0.103	0	0	0	0	0.103	0	0	0
cbs	0	0	0	0.103	0.103	0	0	0	0	0.103	0	0	0
hrasls	0	0	0	0.103	0.103	0	0	0	0	0.103	0	0	0
abcd1	0	0	0	0.103	0.103	0	0	0	0	0.103	0	0	0
atp5c1	0	0	0	0.103	0.103	0	0	0	0	0.103	0	0	0
prdx2	0	0	0	0.102	0.102	0	0	0	0	0.102	0	0	0

gpr75	0	0	0	0.102	0.102	0	0	0	0	0.102	0	0	0
hmbs	0	0	0	0.102	0.102	0	0	0	0	0.102	0	0	0
eif1	0	0	0	0.102	0.102	0	0	0	0	0.102	0	0	0
papss1	0	0	0	0.102	0.102	0	0	0	0	0.102	0	0	0
rp34	0	0	0	0.102	0.102	0	0	0	0	0.102	0	0	0
tpst1	0	0	0	0.103	0.103	0	0	0	0	0.103	0	0	0
tff1	0	0	0	0.103	0.103	0	0	0	0	0.103	0	0	0
slc7a11	0	0	0	0.103	0.103	0	0	0	0	0.103	0	0	0
fhasd	0	0	0	0.103	0.103	0	0	0	0	0.103	0	0	0
tuft1	0	0	0	0.103	0.103	0	0	0	0	0.103	0	0	0
rnr4	0	0	0	0.103	0.103	0	0	0	0	0.103	0	0	0
prss57	0	0	0	0.103	0.103	0	0	0	0	0.103	0	0	0
znf135	0	0	0	0.103	0.103	0	0	0	0	0.103	0	0	0
slc17a5	0	0	0	0.103	0.103	0	0	0	0	0.103	0	0	0
hs3st1	0	0	0	0.103	0.103	0	0	0	0	0.103	0	0	0
ctsa	0	0	0	0.103	0.103	0	0	0	0	0.103	0	0	0
znf77	0	0	0	0.103	0.103	0	0	0	0	0.103	0	0	0
eef2	0	0	0	0.104	0.104	0	0	0	0	0.104	0	0	0
myp13	0	0	0	0.104	0.104	0	0	0	0	0.104	0	0	0
kiaa1199	0	0	0	0.104	0.104	0	0	0	0	0.104	0	0	0
mtg2	0	0	0	0.104	0.104	0	0	0	0	0.104	0	0	0
wbscr27	0	0	0	0.104	0.104	0	0	0	0	0.104	0	0	0
mir455	0	0	0	0.104	0.104	0	0	0	0	0.104	0	0	0
rcan2	0	0	0	0.104	0.104	0	0	0	0	0.104	0	0	0
ptdss1	0	0	0	0.104	0.104	0	0	0	0	0.104	0	0	0
zp1	0	0	0	0.104	0.104	0	0	0	0	0.104	0	0	0
ppr1	0	0	0	0.104	0.104	0	0	0	0	0.104	0	0	0
psd2	0	0	0	0.104	0.104	0	0	0	0	0.104	0	0	0
dmrtb1	0	0	0	0.104	0.104	0	0	0	0	0.104	0	0	0
pfkfb1	0	0	0	0.104	0.104	0	0	0	0	0.104	0	0	0
atp6v0c	0	0	0	0.104	0.104	0	0	0	0	0.104	0	0	0
cuzd1	0	0	0	0.105	0.105	0	0	0	0	0.105	0	0	0
fau	0	0	0	0.105	0.105	0	0	0	0	0.105	0	0	0
pdha1	0	0	0	0.105	0.105	0	0	0	0	0.105	0	0	0
lrrc6	0	0	0	0.105	0.105	0	0	0	0	0.105	0	0	0
slc27a5	0	0	0	0.105	0.105	0	0	0	0	0.105	0	0	0
extl1	0	0	0	0.105	0.105	0	0	0	0	0.105	0	0	0
snora62	0	0	0	0.104	0.104	0	0	0	0	0.104	0	0	0
feom3	0	0	0	0.105	0.105	0	0	0	0	0.105	0	0	0
fra13a	0	0	0	0.105	0.105	0	0	0	0	0.105	0	0	0
npl	0	0	0	0.105	0.105	0	0	0	0	0.105	0	0	0

rpl9	0	0	0	0.104	0.104	0	0	0	0	0.104	0	0	0
itpripl1	0	0	0	0.104	0.104	0	0	0	0	0.104	0	0	0
cth	0	0	0	0.104	0.104	0	0	0	0	0.104	0	0	0
dfna49	0	0	0	0.104	0.104	0	0	0	0	0.104	0	0	0
glrx5	0	0	0	0.104	0.104	0	0	0	0	0.104	0	0	0
kera	0	0	0	0.104	0.104	0	0	0	0	0.104	0	0	0
cbln1	0	0	0	0.104	0.104	0	0	0	0	0.104	0	0	0
bean1	0	0	0	0.104	0.104	0	0	0	0	0.104	0	0	0
sema6b	0	0	0	0.104	0.104	0	0	0	0	0.104	0	0	0
prpf8	0	0	0	0.104	0.104	0	0	0	0	0.104	0	0	0
sca19	0	0	0	0.104	0.104	0	0	0	0	0.104	0	0	0
stath	0	0	0	0.104	0.104	0	0	0	0	0.104	0	0	0
eif1b	0	0	0	0.104	0.104	0	0	0	0	0.104	0	0	0
dhrs2	0	0	0	0.104	0.104	0	0	0	0	0.104	0	0	0
kif19	0	0	0	0.104	0.104	0	0	0	0	0.104	0	0	0
pxmp2	0	0	0	0.104	0.104	0	0	0	0	0.104	0	0	0
gcm2	0	0	0	0.104	0.104	0	0	0	0	0.104	0	0	0
kctd3	0	0	0	0.104	0.104	0	0	0	0	0.104	0	0	0
fam208b	0	0	0	0.104	0.104	0	0	0	0	0.104	0	0	0
dmbt1	0	0	0	0.104	0.104	0	0	0	0	0.104	0	0	0
ppil1	0	0	0	0.104	0.104	0	0	0	0	0.104	0	0	0
or6p1	0	0	0	0.104	0.104	0	0	0	0	0.104	0	0	0
or52a4	0	0	0	0.104	0.104	0	0	0	0	0.104	0	0	0
or1c1	0	0	0	0.104	0.104	0	0	0	0	0.104	0	0	0
slc7a10	0	0	0	0.102	0.102	0	0	0	0	0.102	0	0	0
fbp1	0	0	0	0.102	0.102	0	0	0	0	0.102	0	0	0
esd	0	0	0	0.101	0.101	0	0	0	0	0.101	0	0	0
cyb5r3	0	0	0	0.101	0.101	0	0	0	0	0.101	0	0	0
ndufa8	0	0	0	0.101	0.101	0	0	0	0	0.101	0	0	0
sar1a	0	0	0	0.101	0.101	0	0	0	0	0.101	0	0	0
hist1h2ba	0	0	0	0.101	0.101	0	0	0	0	0.101	0	0	0
rnf112	0	0	0	0.101	0.101	0	0	0	0	0.101	0	0	0
fxyd6	0	0	0	0.101	0.101	0	0	0	0	0.101	0	0	0
capns2	0	0	0	0.101	0.101	0	0	0	0	0.101	0	0	0
tut1	0	0	0	0.101	0.101	0	0	0	0	0.101	0	0	0
tmem114	0	0	0	0.101	0.101	0	0	0	0	0.101	0	0	0
clic4	0	0	0	0.101	0.101	0	0	0	0	0.101	0	0	0
dsg2	0	0	0	0.101	0.101	0	0	0	0	0.101	0	0	0
anxa5	0	0	0	0.101	0.101	0	0	0	0	0.101	0	0	0
dpy19l2	0	0	0	0.101	0.101	0	0	0	0	0.101	0	0	0
lrp2	0	0	0	0.101	0.101	0	0	0	0	0.101	0	0	0

pla2g4c	0	0	0	0.101	0.101	0	0	0	0	0.101	0	0	0
bpifa2	0	0	0	0.101	0.101	0	0	0	0	0.101	0	0	0
tys	0	0	0	0.101	0.101	0	0	0	0	0.101	0	0	0
dfna27	0	0	0	0.101	0.101	0	0	0	0	0.101	0	0	0
psma1	0	0	0	0.101	0.101	0	0	0	0	0.101	0	0	0
kcnk17	0	0	0	0.101	0.101	0	0	0	0	0.101	0	0	0
pde3a	0	0	0	0.101	0.101	0	0	0	0	0.101	0	0	0
emilin2	0	0	0	0.101	0.101	0	0	0	0	0.101	0	0	0
lamp1	0	0	0	0.101	0.101	0	0	0	0	0.101	0	0	0
gpr139	0	0	0	0.1	0.1	0	0	0	0	0.1	0	0	0
mrpl43	0	0	0	0.1	0.1	0	0	0	0	0.1	0	0	0
szt2	0	0	0	0.1	0.1	0	0	0	0	0.1	0	0	0
kcnrg	0	0	0	0.1	0.1	0	0	0	0	0.1	0	0	0
b4galt1	0	0	0	0.1	0.1	0	0	0	0	0.1	0	0	0
trnw	0	0	0	0.1	0.1	0	0	0	0	0.1	0	0	0
ppp2cbp1	0	0	0	0.122	0.122	0	0	0	0	0.122	0	0	0
peg3-as1	0	0	0	0.1	0.1	0	0	0	0	0.1	0	0	0
hist1h3h	0	0	0	0.1	0.1	0	0	0	0	0.1	0	0	0
slc26a5	0	0	0	0.1	0.1	0	0	0	0	0.1	0	0	0
hrc	0	0	0	0.1	0.1	0	0	0	0	0.1	0	0	0
nit1	0	0	0	0.1	0.1	0	0	0	0	0.1	0	0	0
ppef1	0	0	0	0.101	0.101	0	0	0	0	0.101	0	0	0
acadm	0	0	0	0.101	0.101	0	0	0	0	0.101	0	0	0
zfyve1	0	0	0	0.101	0.101	0	0	0	0	0.101	0	0	0
mccc2	0	0	0	0.101	0.101	0	0	0	0	0.101	0	0	0
nudt4	0	0	0	0.1	0.1	0	0	0	0	0.1	0	0	0
atat1	0	0	0	0.1	0.1	0	0	0	0	0.1	0	0	0
scp2	0	0	0	0.1	0.1	0	0	0	0	0.1	0	0	0
candn1	0	0	0	0.1	0.1	0	0	0	0	0.1	0	0	0
six6	0	0	0	0.1	0.1	0	0	0	0	0.1	0	0	0
kcnn1	0	0	0	0.1	0.1	0	0	0	0	0.1	0	0	0
cetn4p	0	0	0	0.101	0.101	0	0	0	0	0.101	0	0	0
sparc	0	0	0	0.101	0.101	0	0	0	0	0.101	0	0	0
znf358	0	0	0	0.102	0.102	0	0	0	0	0.102	0	0	0
stsp1	0	0	0	0.102	0.102	0	0	0	0	0.102	0	0	0
usmg5	0	0	0	0.102	0.102	0	0	0	0	0.102	0	0	0
fxn	0	0	0	0.102	0.102	0	0	0	0	0.102	0	0	0
glt8d1	0	0	0	0.102	0.102	0	0	0	0	0.102	0	0	0
dars2	0	0	0	0.102	0.102	0	0	0	0	0.102	0	0	0
hist2h2bb	0	0	0	0.102	0.102	0	0	0	0	0.102	0	0	0
myo16	0	0	0	0.102	0.102	0	0	0	0	0.102	0	0	0

smoc1	0	0	0	0.102	0.102	0	0	0	0	0.102	0	0	0
chka	0	0	0	0.102	0.102	0	0	0	0	0.102	0	0	0
tm6sf1	0	0	0	0.102	0.102	0	0	0	0	0.102	0	0	0
tm6sf2	0	0	0	0.102	0.102	0	0	0	0	0.102	0	0	0
rln3	0	0	0	0.102	0.102	0	0	0	0	0.102	0	0	0
timm17b	0	0	0	0.102	0.102	0	0	0	0	0.102	0	0	0
tenc1	0	0	0	0.102	0.102	0	0	0	0	0.102	0	0	0
acot2	0	0	0	0.102	0.102	0	0	0	0	0.102	0	0	0
acat1	0	0	0	0.102	0.102	0	0	0	0	0.102	0	0	0
prkxp1	0	0	0	0.102	0.102	0	0	0	0	0.102	0	0	0
scar3	0	0	0	0.102	0.102	0	0	0	0	0.102	0	0	0
psg7	0	0	0	0.102	0.102	0	0	0	0	0.102	0	0	0
timm10	0	0	0	0.102	0.102	0	0	0	0	0.102	0	0	0
bspry	0	0	0	0.102	0.102	0	0	0	0	0.102	0	0	0
herc2p6	0	0	0	0.102	0.102	0	0	0	0	0.102	0	0	0
akr1c2	0	0	0	0.101	0.101	0	0	0	0	0.101	0	0	0
nudt14	0	0	0	0.101	0.101	0	0	0	0	0.101	0	0	0
psg4	0	0	0	0.101	0.101	0	0	0	0	0.101	0	0	0
klhl32	0	0	0	0.101	0.101	0	0	0	0	0.101	0	0	0
kiaa1549l	0	0	0	0.101	0.101	0	0	0	0	0.101	0	0	0
bbs2	0	0	0	0.101	0.101	0	0	0	0	0.101	0	0	0
ykt6	0	0	0	0.101	0.101	0	0	0	0	0.101	0	0	0
trappc10	0	0	0	0.101	0.101	0	0	0	0	0.101	0	0	0
hexa	0	0	0	0.101	0.101	0	0	0	0	0.101	0	0	0
rai2	0	0	0	0.101	0.101	0	0	0	0	0.101	0	0	0
muc3	0	0	0	0.101	0.101	0	0	0	0	0.101	0	0	0
scar7	0	0	0	0.101	0.101	0	0	0	0	0.101	0	0	0
znf73	0	0	0	0.101	0.101	0	0	0	0	0.101	0	0	0
rpl36al	0	0	0	0.101	0.101	0	0	0	0	0.101	0	0	0
abhd14b	0	0	0	0.101	0.101	0	0	0	0	0.101	0	0	0
hcn1	0	0	0	0.101	0.101	0	0	0	0	0.101	0	0	0
fmo4	0	0	0	0.101	0.101	0	0	0	0	0.101	0	0	0
surf2	0	0	0	0.101	0.101	0	0	0	0	0.101	0	0	0
prkx	0	0	0	0.101	0.101	0	0	0	0	0.101	0	0	0
ankh	0	0	0	0.101	0.101	0	0	0	0	0.101	0	0	0
eig2	0	0	0	0.101	0.101	0	0	0	0	0.101	0	0	0
sc5d	0	0	0	0.101	0.101	0	0	0	0	0.101	0	0	0
znf355p	0	0	0	0.105	0.105	0	0	0	0	0.105	0	0	0
znf652p1	0	0	0	0.105	0.105	0	0	0	0	0.105	0	0	0
hnrnpd	0	0	0	0.109	0.109	0	0	0	0	0.109	0	0	0
zc3h4	0	0	0	0.109	0.109	0	0	0	0	0.109	0	0	0

slc37a1	0	0	0	0.109	0.109	0	0	0	0	0.109	0	0	0
loc100133315	0	0	0	0.109	0.109	0	0	0	0	0.109	0	0	0
defb105a	0	0	0	0.109	0.109	0	0	0	0	0.109	0	0	0
ifrd2	0	0	0	0.109	0.109	0	0	0	0	0.109	0	0	0
vdac1p3	0	0	0	0.109	0.109	0	0	0	0	0.109	0	0	0
mpc2	0	0	0	0.109	0.109	0	0	0	0	0.109	0	0	0
dnase2b	0	0	0	0.109	0.109	0	0	0	0	0.109	0	0	0
itpk1	0	0	0	0.109	0.109	0	0	0	0	0.109	0	0	0
psap	0	0	0	0.109	0.109	0	0	0	0	0.109	0	0	0
cdh12	0	0	0	0.109	0.109	0	0	0	0	0.109	0	0	0
kcnn4	0	0	0	0.109	0.109	0	0	0	0	0.109	0	0	0
slc24a4	0	0	0	0.109	0.109	0	0	0	0	0.109	0	0	0
psg1	0	0	0	0.11	0.11	0	0	0	0	0.11	0	0	0
pla2g2a	0	0	0	0.11	0.11	0	0	0	0	0.11	0	0	0
acot13	0	0	0	0.109	0.109	0	0	0	0	0.109	0	0	0
clcn4	0	0	0	0.109	0.109	0	0	0	0	0.109	0	0	0
clic2	0	0	0	0.109	0.109	0	0	0	0	0.109	0	0	0
slc6a8	0	0	0	0.109	0.109	0	0	0	0	0.109	0	0	0
pkdrej	0	0	0	0.109	0.109	0	0	0	0	0.109	0	0	0
atp11a	0	0	0	0.109	0.109	0	0	0	0	0.109	0	0	0
six3	0	0	0	0.109	0.109	0	0	0	0	0.109	0	0	0
sma4	0	0	0	0.109	0.109	0	0	0	0	0.109	0	0	0
hibch	0	0	0	0.108	0.108	0	0	0	0	0.108	0	0	0
ubr7	0	0	0	0.108	0.108	0	0	0	0	0.108	0	0	0
dsp	0	0	0	0.108	0.108	0	0	0	0	0.108	0	0	0
atp6v1c1	0	0	0	0.108	0.108	0	0	0	0	0.108	0	0	0
dhdds	0	0	0	0.108	0.108	0	0	0	0	0.108	0	0	0
rps5	0	0	0	0.108	0.108	0	0	0	0	0.108	0	0	0
aoc1	0	0	0	0.108	0.108	0	0	0	0	0.108	0	0	0
snora72	0	0	0	0.108	0.108	0	0	0	0	0.108	0	0	0
vmo1	0	0	0	0.108	0.108	0	0	0	0	0.108	0	0	0
ma	0	0	0	0.108	0.108	0	0	0	0	0.108	0	0	0
slc28a1	0	0	0	0.108	0.108	0	0	0	0	0.108	0	0	0
pafah1b1	0	0	0	0.108	0.108	0	0	0	0	0.108	0	0	0
muc12	0	0	0	0.109	0.109	0	0	0	0	0.109	0	0	0
rab9bp1	0	0	0	0.109	0.109	0	0	0	0	0.109	0	0	0
il1rapl1	0	0	0	0.109	0.109	0	0	0	0	0.109	0	0	0
prrt2	0	0	0	0.109	0.109	0	0	0	0	0.109	0	0	0
gapdhs	0	0	0	0.109	0.109	0	0	0	0	0.109	0	0	0
msto2p	0	0	0	0.109	0.109	0	0	0	0	0.109	0	0	0
fstl4	0	0	0	0.108	0.108	0	0	0	0	0.108	0	0	0

kcnab1	0	0	0	0.108	0.108	0	0	0	0	0.108	0	0	0
cox8bp	0	0	0	0.109	0.109	0	0	0	0	0.109	0	0	0
dap3p1	0	0	0	0.109	0.109	0	0	0	0	0.109	0	0	0
hist1h1c	0	0	0	0.11	0.11	0	0	0	0	0.11	0	0	0
bola3	0	0	0	0.11	0.11	0	0	0	0	0.11	0	0	0
slc22a10	0	0	0	0.11	0.11	0	0	0	0	0.11	0	0	0
b4galt6	0	0	0	0.11	0.11	0	0	0	0	0.11	0	0	0
ctbs	0	0	0	0.11	0.11	0	0	0	0	0.11	0	0	0
guca1c	0	0	0	0.11	0.11	0	0	0	0	0.11	0	0	0
ppap2c	0	0	0	0.11	0.11	0	0	0	0	0.11	0	0	0
dmxl1	0	0	0	0.11	0.11	0	0	0	0	0.11	0	0	0
nudt19	0	0	0	0.11	0.11	0	0	0	0	0.11	0	0	0
rnu1-4	0	0	0	0.11	0.11	0	0	0	0	0.11	0	0	0
rpph1	0	0	0	0.11	0.11	0	0	0	0	0.11	0	0	0
lipf	0	0	0	0.11	0.11	0	0	0	0	0.11	0	0	0
glt6d1	0	0	0	0.11	0.11	0	0	0	0	0.11	0	0	0
sult1a3	0	0	0	0.11	0.11	0	0	0	0	0.11	0	0	0
hspe1	0	0	0	0.111	0.111	0	0	0	0	0.111	0	0	0
dnajc4	0	0	0	0.111	0.111	0	0	0	0	0.111	0	0	0
cdh19	0	0	0	0.111	0.111	0	0	0	0	0.111	0	0	0
dld	0	0	0	0.111	0.111	0	0	0	0	0.111	0	0	0
ahsg	0	0	0	0.111	0.111	0	0	0	0	0.111	0	0	0
prps1	0	0	0	0.111	0.111	0	0	0	0	0.111	0	0	0
hcn2	0	0	0	0.111	0.111	0	0	0	0	0.111	0	0	0
gmpr	0	0	0	0.111	0.111	0	0	0	0	0.111	0	0	0
c21orf49	0	0	0	0.111	0.111	0	0	0	0	0.111	0	0	0
ankrd7	0	0	0	0.111	0.111	0	0	0	0	0.111	0	0	0
atp5f1	0	0	0	0.11	0.11	0	0	0	0	0.11	0	0	0
hist2h2aa4	0	0	0	0.11	0.11	0	0	0	0	0.11	0	0	0
adsl	0	0	0	0.11	0.11	0	0	0	0	0.11	0	0	0
slc26a4	0	0	0	0.11	0.11	0	0	0	0	0.11	0	0	0
afg3l2	0	0	0	0.11	0.11	0	0	0	0	0.11	0	0	0
antxr1	0	0	0	0.11	0.11	0	0	0	0	0.11	0	0	0
msrb2	0	0	0	0.11	0.11	0	0	0	0	0.11	0	0	0
atox1	0	0	0	0.11	0.11	0	0	0	0	0.11	0	0	0
gk6p	0	0	0	0.11	0.11	0	0	0	0	0.11	0	0	0
feb7	0	0	0	0.11	0.11	0	0	0	0	0.11	0	0	0
elk2ap	0	0	0	0.11	0.11	0	0	0	0	0.11	0	0	0
htn1	0	0	0	0.11	0.11	0	0	0	0	0.11	0	0	0
kcnj10	0	0	0	0.11	0.11	0	0	0	0	0.11	0	0	0
ppp3cb	0	0	0	0.11	0.11	0	0	0	0	0.11	0	0	0

faup1	0	0	0	0.11	0.11	0	0	0	0	0.11	0	0	0
rpl3l	0	0	0	0.11	0.11	0	0	0	0	0.11	0	0	0
pcdh9	0	0	0	0.11	0.11	0	0	0	0	0.11	0	0	0
emc4	0	0	0	0.11	0.11	0	0	0	0	0.11	0	0	0
c19orf59	0	0	0	0.11	0.11	0	0	0	0	0.11	0	0	0
atp5g1	0	0	0	0.11	0.11	0	0	0	0	0.11	0	0	0
kcnq3	0	0	0	0.11	0.11	0	0	0	0	0.11	0	0	0
prpf18	0	0	0	0.11	0.11	0	0	0	0	0.11	0	0	0
snrnp200	0	0	0	0.11	0.11	0	0	0	0	0.11	0	0	0
upk1b	0	0	0	0.108	0.108	0	0	0	0	0.108	0	0	0
slc9a3r2	0	0	0	0.108	0.108	0	0	0	0	0.108	0	0	0
tmem27	0	0	0	0.106	0.106	0	0	0	0	0.106	0	0	0
kcnn3	0	0	0	0.106	0.106	0	0	0	0	0.106	0	0	0
vwfp1	0	0	0	0.106	0.106	0	0	0	0	0.106	0	0	0
u2af2	0	0	0	0.106	0.106	0	0	0	0	0.106	0	0	0
slc30a10	0	0	0	0.106	0.106	0	0	0	0	0.106	0	0	0
emid1	0	0	0	0.106	0.106	0	0	0	0	0.106	0	0	0
hyal4	0	0	0	0.106	0.106	0	0	0	0	0.106	0	0	0
nfat5	0	0	0	0.106	0.106	0	0	0	0	0.106	0	0	0
sytl2	0	0	0	0.106	0.106	0	0	0	0	0.106	0	0	0
six2	0	0	0	0.106	0.106	0	0	0	0	0.106	0	0	0
uba52p1	0	0	0	0.106	0.106	0	0	0	0	0.106	0	0	0
uba52p2	0	0	0	0.106	0.106	0	0	0	0	0.106	0	0	0
psg10p	0	0	0	0.106	0.106	0	0	0	0	0.106	0	0	0
slc13a1	0	0	0	0.106	0.106	0	0	0	0	0.106	0	0	0
rpn2	0	0	0	0.106	0.106	0	0	0	0	0.106	0	0	0
rph3a	0	0	0	0.106	0.106	0	0	0	0	0.106	0	0	0
pctp	0	0	0	0.106	0.106	0	0	0	0	0.106	0	0	0
pate1	0	0	0	0.106	0.106	0	0	0	0	0.106	0	0	0
pigap1	0	0	0	0.106	0.106	0	0	0	0	0.106	0	0	0
cpo	0	0	0	0.106	0.106	0	0	0	0	0.106	0	0	0
tdrd6	0	0	0	0.106	0.106	0	0	0	0	0.106	0	0	0
prdx1	0	0	0	0.106	0.106	0	0	0	0	0.106	0	0	0
pfn3	0	0	0	0.106	0.106	0	0	0	0	0.106	0	0	0
alrh	0	0	0	0.106	0.106	0	0	0	0	0.106	0	0	0
cbx4	0	0	0	0.105	0.105	0	0	0	0	0.105	0	0	0
rimbp2	0	0	0	0.105	0.105	0	0	0	0	0.105	0	0	0
ndufaf1	0	0	0	0.105	0.105	0	0	0	0	0.105	0	0	0
pign	0	0	0	0.105	0.105	0	0	0	0	0.105	0	0	0
best2	0	0	0	0.105	0.105	0	0	0	0	0.105	0	0	0
ervk-20	0	0	0	0.105	0.105	0	0	0	0	0.105	0	0	0

dhx15	0	0	0	0.105	0.105	0	0	0	0	0.105	0	0	0
pqbp4	0	0	0	0.105	0.105	0	0	0	0	0.105	0	0	0
hmx2	0	0	0	0.105	0.105	0	0	0	0	0.105	0	0	0
crygfp	0	0	0	0.105	0.105	0	0	0	0	0.105	0	0	0
slc15a4	0	0	0	0.105	0.105	0	0	0	0	0.105	0	0	0
linc00114	0	0	0	0.105	0.105	0	0	0	0	0.105	0	0	0
hdgfl1	0	0	0	0.106	0.106	0	0	0	0	0.106	0	0	0
crtac1	0	0	0	0.106	0.106	0	0	0	0	0.106	0	0	0
pla2g2d	0	0	0	0.106	0.106	0	0	0	0	0.106	0	0	0
mon1a	0	0	0	0.106	0.106	0	0	0	0	0.106	0	0	0
mat2b	0	0	0	0.106	0.106	0	0	0	0	0.106	0	0	0
zdhhc1	0	0	0	0.105	0.105	0	0	0	0	0.105	0	0	0
cpvl	0	0	0	0.105	0.105	0	0	0	0	0.105	0	0	0
khnyn	0	0	0	0.105	0.105	0	0	0	0	0.105	0	0	0
ext1	0	0	0	0.105	0.105	0	0	0	0	0.105	0	0	0
adrm1	0	0	0	0.105	0.105	0	0	0	0	0.105	0	0	0
nicn1	0	0	0	0.106	0.106	0	0	0	0	0.106	0	0	0
pds5b	0	0	0	0.106	0.106	0	0	0	0	0.106	0	0	0
tpi1	0	0	0	0.108	0.108	0	0	0	0	0.108	0	0	0
arsep1	0	0	0	0.108	0.108	0	0	0	0	0.108	0	0	0
vn2r1p	0	0	0	0.108	0.108	0	0	0	0	0.108	0	0	0
rpl35a	0	0	0	0.108	0.108	0	0	0	0	0.108	0	0	0
galt	0	0	0	0.108	0.108	0	0	0	0	0.108	0	0	0
actg1p10	0	0	0	0.108	0.108	0	0	0	0	0.108	0	0	0
ndufs3	0	0	0	0.107	0.107	0	0	0	0	0.107	0	0	0
gucy2f	0	0	0	0.107	0.107	0	0	0	0	0.107	0	0	0
spag7	0	0	0	0.107	0.107	0	0	0	0	0.107	0	0	0
oatp1	0	0	0	0.107	0.107	0	0	0	0	0.107	0	0	0
pth1r	0	0	0	0.108	0.108	0	0	0	0	0.108	0	0	0
slc43a3	0	0	0	0.108	0.108	0	0	0	0	0.108	0	0	0
tulp4	0	0	0	0.108	0.108	0	0	0	0	0.108	0	0	0
tuba1c	0	0	0	0.108	0.108	0	0	0	0	0.108	0	0	0
vsnl1	0	0	0	0.108	0.108	0	0	0	0	0.108	0	0	0
ncapd2	0	0	0	0.108	0.108	0	0	0	0	0.108	0	0	0
ces5a	0	0	0	0.108	0.108	0	0	0	0	0.108	0	0	0
krt2	0	0	0	0.108	0.108	0	0	0	0	0.108	0	0	0
hist1h2aps5	0	0	0	0.108	0.108	0	0	0	0	0.108	0	0	0
fzd10	0	0	0	0.108	0.108	0	0	0	0	0.108	0	0	0
poc1b	0	0	0	0.108	0.108	0	0	0	0	0.108	0	0	0
mepe	0	0	0	0.108	0.108	0	0	0	0	0.108	0	0	0
mmgt1	0	0	0	0.107	0.107	0	0	0	0	0.107	0	0	0

znf71	0	0	0	0.107	0.107	0	0	0	0	0.107	0	0	0
mir338	0	0	0	0.107	0.107	0	0	0	0	0.107	0	0	0
catsper1	0	0	0	0.107	0.107	0	0	0	0	0.107	0	0	0
impact	0	0	0	0.107	0.107	0	0	0	0	0.107	0	0	0
slc39a13	0	0	0	0.107	0.107	0	0	0	0	0.107	0	0	0
kcnj3	0	0	0	0.107	0.107	0	0	0	0	0.107	0	0	0
dnah6	0	0	0	0.107	0.107	0	0	0	0	0.107	0	0	0
appl1	0	0	0	0.106	0.106	0	0	0	0	0.106	0	0	0
dach1	0	0	0	0.107	0.107	0	0	0	0	0.107	0	0	0
loc1720	0	0	0	0.107	0.107	0	0	0	0	0.107	0	0	0
dnah10	0	0	0	0.107	0.107	0	0	0	0	0.107	0	0	0
jph4	0	0	0	0.107	0.107	0	0	0	0	0.107	0	0	0
cdh16	0	0	0	0.107	0.107	0	0	0	0	0.107	0	0	0
paox	0	0	0	0.107	0.107	0	0	0	0	0.107	0	0	0
slc5a8	0	0	0	0.107	0.107	0	0	0	0	0.107	0	0	0
prm2	0	0	0	0.107	0.107	0	0	0	0	0.107	0	0	0
ush1g	0	0	0	0.107	0.107	0	0	0	0	0.107	0	0	0
ppap2a	0	0	0	0.107	0.107	0	0	0	0	0.107	0	0	0
neto2	0	0	0	0.107	0.107	0	0	0	0	0.107	0	0	0
syt5	0	0	0	0.107	0.107	0	0	0	0	0.107	0	0	0
prps2	0	0	0	0.107	0.107	0	0	0	0	0.107	0	0	0
fbxw12	0	0	0	0.107	0.107	0	0	0	0	0.107	0	0	0
slc9b1	0	0	0	0.111	0.111	0	0	0	0	0.111	0	0	0
nfu1	0	0	0	0.142	0.142	0	0	0	0	0.142	0	0	0
fgf23	0	0	0	0.159	0.159	0	0	0	0	0.159	0	0	0
atp13a5	0	0	0	0.159	0.159	0	0	0	0	0.159	0	0	0
emb	0	0	0	0.16	0.16	0	0	0	0	0.16	0	0	0
frmd7	0	0	0	0.16	0.16	0	0	0	0	0.16	0	0	0
candf1	0	0	0	0.159	0.159	0	0	0	0	0.159	0	0	0
rgn	0	0	0	0.159	0.159	0	0	0	0	0.159	0	0	0
atp2a3	0	0	0	0.159	0.159	0	0	0	0	0.159	0	0	0
lalba	0	0	0	0.159	0.159	0	0	0	0	0.159	0	0	0
hist2h2ba	0	0	0	0.159	0.159	0	0	0	0	0.159	0	0	0
mir328	0	0	0	0.159	0.159	0	0	0	0	0.159	0	0	0
slc22a1	0	0	0	0.16	0.16	0	0	0	0	0.16	0	0	0
edc3	0	0	0	0.16	0.16	0	0	0	0	0.16	0	0	0
slc1a5	0	0	0	0.161	0.161	0	0	0	0	0.161	0	0	0
trpc4	0	0	0	0.161	0.161	0	0	0	0	0.161	0	0	0
ivd	0	0	0	0.162	0.162	0	0	0	0	0.162	0	0	0
sgk2	0	0	0	0.162	0.162	0	0	0	0	0.162	0	0	0
slc22a24	0	0	0	0.161	0.161	0	0	0	0	0.161	0	0	0

prpsap1	0	0	0	0.161	0.161	0	0	0	0	0.161	0	0	0
hist2h2bc	0	0	0	0.16	0.16	0	0	0	0	0.16	0	0	0
cpne9	0	0	0	0.16	0.16	0	0	0	0	0.16	0	0	0
slc28a3	0	0	0	0.16	0.16	0	0	0	0	0.16	0	0	0
slc12a6	0	0	0	0.16	0.16	0	0	0	0	0.16	0	0	0
cse	0	0	0	0.159	0.159	0	0	0	0	0.159	0	0	0
lim2	0	0	0	0.158	0.158	0	0	0	0	0.158	0	0	0
slc22a6	0	0	0	0.156	0.156	0	0	0	0	0.156	0	0	0
slc26a11	0	0	0	0.156	0.156	0	0	0	0	0.156	0	0	0
agmat	0	0	0	0.157	0.157	0	0	0	0	0.157	0	0	0
mocs2	0	0	0	0.157	0.157	0	0	0	0	0.157	0	0	0
trpc3	0	0	0	0.156	0.156	0	0	0	0	0.156	0	0	0
c8orf44-sgk3	0	0	0	0.156	0.156	0	0	0	0	0.156	0	0	0
nicn2p	0	0	0	0.156	0.156	0	0	0	0	0.156	0	0	0
cpa2	0	0	0	0.156	0.156	0	0	0	0	0.156	0	0	0
alg13	0	0	0	0.156	0.156	0	0	0	0	0.156	0	0	0
sdc4	0	0	0	0.156	0.156	0	0	0	0	0.156	0	0	0
chp1	0	0	0	0.157	0.157	0	0	0	0	0.157	0	0	0
aldob	0	0	0	0.157	0.157	0	0	0	0	0.157	0	0	0
slc16a5	0	0	0	0.158	0.158	0	0	0	0	0.158	0	0	0
atp5d	0	0	0	0.158	0.158	0	0	0	0	0.158	0	0	0
ceacam8	0	0	0	0.158	0.158	0	0	0	0	0.158	0	0	0
ankrd36c	0	0	0	0.158	0.158	0	0	0	0	0.158	0	0	0
anxa8l2	0	0	0	0.158	0.158	0	0	0	0	0.158	0	0	0
slc23a2	0	0	0	0.157	0.157	0	0	0	0	0.157	0	0	0
slc6a15	0	0	0	0.157	0.157	0	0	0	0	0.157	0	0	0
pnpla8	0	0	0	0.157	0.157	0	0	0	0	0.157	0	0	0
foxe3	0	0	0	0.157	0.157	0	0	0	0	0.157	0	0	0
psg6	0	0	0	0.157	0.157	0	0	0	0	0.157	0	0	0
sycn	0	0	0	0.162	0.162	0	0	0	0	0.162	0	0	0
pigg	0	0	0	0.162	0.162	0	0	0	0	0.162	0	0	0
rnu2-1	0	0	0	0.168	0.168	0	0	0	0	0.168	0	0	0
s100g	0	0	0	0.168	0.168	0	0	0	0	0.168	0	0	0
slc8a3	0	0	0	0.169	0.169	0	0	0	0	0.169	0	0	0
hspb7	0	0	0	0.169	0.169	0	0	0	0	0.169	0	0	0
c12orf4	0	0	0	0.168	0.168	0	0	0	0	0.168	0	0	0
cd53	0	0	0	0.168	0.168	0	0	0	0	0.168	0	0	0
trpm7	0	0	0	0.167	0.167	0	0	0	0	0.167	0	0	0
catcn1	0	0	0	0.168	0.168	0	0	0	0	0.168	0	0	0
apeh	0	0	0	0.168	0.168	0	0	0	0	0.168	0	0	0
cldn14	0	0	0	0.168	0.168	0	0	0	0	0.168	0	0	0

btg1	0	0	0	0.169	0.169	0	0	0	0	0.169	0	0	0
tars	0	0	0	0.17	0.17	0	0	0	0	0.17	0	0	0
amy1b	0	0	0	0.171	0.171	0	0	0	0	0.171	0	0	0
slc12a7	0	0	0	0.171	0.171	0	0	0	0	0.171	0	0	0
gcsh	0	0	0	0.171	0.171	0	0	0	0	0.171	0	0	0
dym	0	0	0	0.172	0.172	0	0	0	0	0.172	0	0	0
slc16a3	0	0	0	0.171	0.171	0	0	0	0	0.171	0	0	0
clcnkb	0	0	0	0.171	0.171	0	0	0	0	0.171	0	0	0
sgk1	0	0	0	0.17	0.17	0	0	0	0	0.17	0	0	0
kcnj16	0	0	0	0.17	0.17	0	0	0	0	0.17	0	0	0
slc3a1	0	0	0	0.17	0.17	0	0	0	0	0.17	0	0	0
ca13	0	0	0	0.171	0.171	0	0	0	0	0.171	0	0	0
surf4	0	0	0	0.167	0.167	0	0	0	0	0.167	0	0	0
hhc3	0	0	0	0.167	0.167	0	0	0	0	0.167	0	0	0
hspb6	0	0	0	0.163	0.163	0	0	0	0	0.163	0	0	0
s100a16	0	0	0	0.164	0.164	0	0	0	0	0.164	0	0	0
nhs	0	0	0	0.164	0.164	0	0	0	0	0.164	0	0	0
slc26a6	0	0	0	0.164	0.164	0	0	0	0	0.164	0	0	0
atp8a1	0	0	0	0.163	0.163	0	0	0	0	0.163	0	0	0
slc9a3	0	0	0	0.163	0.163	0	0	0	0	0.163	0	0	0
acrv1	0	0	0	0.162	0.162	0	0	0	0	0.162	0	0	0
nys2	0	0	0	0.162	0.162	0	0	0	0	0.162	0	0	0
bckdhb	0	0	0	0.162	0.162	0	0	0	0	0.162	0	0	0
slc8b1	0	0	0	0.163	0.163	0	0	0	0	0.163	0	0	0
spesp1	0	0	0	0.164	0.164	0	0	0	0	0.164	0	0	0
rhcg	0	0	0	0.165	0.165	0	0	0	0	0.165	0	0	0
clcn3	0	0	0	0.166	0.166	0	0	0	0	0.166	0	0	0
ocm2	0	0	0	0.166	0.166	0	0	0	0	0.166	0	0	0
hspbap1	0	0	0	0.167	0.167	0	0	0	0	0.167	0	0	0
rnf24	0	0	0	0.167	0.167	0	0	0	0	0.167	0	0	0
atp1b3	0	0	0	0.166	0.166	0	0	0	0	0.166	0	0	0
slc16a1	0	0	0	0.166	0.166	0	0	0	0	0.166	0	0	0
gingf2	0	0	0	0.165	0.165	0	0	0	0	0.165	0	0	0
slc13a2	0	0	0	0.165	0.165	0	0	0	0	0.165	0	0	0
slc47a1	0	0	0	0.165	0.165	0	0	0	0	0.165	0	0	0
crym	0	0	0	0.166	0.166	0	0	0	0	0.166	0	0	0
hint2	0	0	0	0.156	0.156	0	0	0	0	0.156	0	0	0
txndc2	0	0	0	0.156	0.156	0	0	0	0	0.156	0	0	0
ttpa	0	0	0	0.147	0.147	0	0	0	0	0.147	0	0	0
grifin	0	0	0	0.148	0.148	0	0	0	0	0.148	0	0	0
caln1	0	0	0	0.148	0.148	0	0	0	0	0.148	0	0	0

nudt10	0	0	0	0.148	0.148	0	0	0	0	0.148	0	0	0
snora67	0	0	0	0.147	0.147	0	0	0	0	0.147	0	0	0
snora68	0	0	0	0.147	0.147	0	0	0	0	0.147	0	0	0
slc6a19	0	0	0	0.147	0.147	0	0	0	0	0.147	0	0	0
slc4a8	0	0	0	0.147	0.147	0	0	0	0	0.147	0	0	0
khsrp	0	0	0	0.147	0.147	0	0	0	0	0.147	0	0	0
anxa7	0	0	0	0.147	0.147	0	0	0	0	0.147	0	0	0
atp13a1	0	0	0	0.148	0.148	0	0	0	0	0.148	0	0	0
ca12	0	0	0	0.148	0.148	0	0	0	0	0.148	0	0	0
lyrm4	0	0	0	0.149	0.149	0	0	0	0	0.149	0	0	0
lpar5	0	0	0	0.149	0.149	0	0	0	0	0.149	0	0	0
txndc15	0	0	0	0.149	0.149	0	0	0	0	0.149	0	0	0
fra1e	0	0	0	0.149	0.149	0	0	0	0	0.149	0	0	0
lcn1	0	0	0	0.149	0.149	0	0	0	0	0.149	0	0	0
tufm	0	0	0	0.148	0.148	0	0	0	0	0.148	0	0	0
slc9a3p1	0	0	0	0.148	0.148	0	0	0	0	0.148	0	0	0
svop	0	0	0	0.148	0.148	0	0	0	0	0.148	0	0	0
oit3	0	0	0	0.148	0.148	0	0	0	0	0.148	0	0	0
cryz	0	0	0	0.148	0.148	0	0	0	0	0.148	0	0	0
nrgn	0	0	0	0.147	0.147	0	0	0	0	0.147	0	0	0
mir15a	0	0	0	0.146	0.146	0	0	0	0	0.146	0	0	0
ldhap3	0	0	0	0.145	0.145	0	0	0	0	0.145	0	0	0
ldhap5	0	0	0	0.145	0.145	0	0	0	0	0.145	0	0	0
ccdc47	0	0	0	0.145	0.145	0	0	0	0	0.145	0	0	0
akr1a1	0	0	0	0.145	0.145	0	0	0	0	0.145	0	0	0
lrrn3	0	0	0	0.145	0.145	0	0	0	0	0.145	0	0	0
lrp1b	0	0	0	0.145	0.145	0	0	0	0	0.145	0	0	0
atp1a4	0	0	0	0.145	0.145	0	0	0	0	0.145	0	0	0
hdhd1	0	0	0	0.145	0.145	0	0	0	0	0.145	0	0	0
ndufs2	0	0	0	0.145	0.145	0	0	0	0	0.145	0	0	0
pi4ka	0	0	0	0.145	0.145	0	0	0	0	0.145	0	0	0
ndufv2p1	0	0	0	0.146	0.146	0	0	0	0	0.146	0	0	0
clpp	0	0	0	0.146	0.146	0	0	0	0	0.146	0	0	0
slc9a2	0	0	0	0.146	0.146	0	0	0	0	0.146	0	0	0
atp6v1b1	0	0	0	0.146	0.146	0	0	0	0	0.146	0	0	0
ncs1	0	0	0	0.146	0.146	0	0	0	0	0.146	0	0	0
hmgn1	0	0	0	0.146	0.146	0	0	0	0	0.146	0	0	0
jmjd7	0	0	0	0.146	0.146	0	0	0	0	0.146	0	0	0
anxa2p2	0	0	0	0.146	0.146	0	0	0	0	0.146	0	0	0
qars	0	0	0	0.146	0.146	0	0	0	0	0.146	0	0	0
s100a7p1	0	0	0	0.146	0.146	0	0	0	0	0.146	0	0	0

s100a7p2	0	0	0	0.146	0.146	0	0	0	0	0.146	0	0	0
glud2	0	0	0	0.146	0.146	0	0	0	0	0.146	0	0	0
adam12-ot1	0	0	0	0.149	0.149	0	0	0	0	0.149	0	0	0
slc26a3	0	0	0	0.149	0.149	0	0	0	0	0.149	0	0	0
atp6v0a4	0	0	0	0.154	0.154	0	0	0	0	0.154	0	0	0
eno3	0	0	0	0.154	0.154	0	0	0	0	0.154	0	0	0
slc7a6os	0	0	0	0.154	0.154	0	0	0	0	0.154	0	0	0
pkd2	0	0	0	0.154	0.154	0	0	0	0	0.154	0	0	0
amd1	0	0	0	0.154	0.154	0	0	0	0	0.154	0	0	0
samsn1	0	0	0	0.154	0.154	0	0	0	0	0.154	0	0	0
pcca	0	0	0	0.153	0.153	0	0	0	0	0.153	0	0	0
rnu4-1	0	0	0	0.153	0.153	0	0	0	0	0.153	0	0	0
slc4a10	0	0	0	0.153	0.153	0	0	0	0	0.153	0	0	0
slc6a14	0	0	0	0.153	0.153	0	0	0	0	0.153	0	0	0
crip1p4	0	0	0	0.155	0.155	0	0	0	0	0.155	0	0	0
crip1p2	0	0	0	0.155	0.155	0	0	0	0	0.155	0	0	0
pef1	0	0	0	0.155	0.155	0	0	0	0	0.155	0	0	0
pafah1b3	0	0	0	0.155	0.155	0	0	0	0	0.155	0	0	0
bsn	0	0	0	0.155	0.155	0	0	0	0	0.155	0	0	0
rsc1a1	0	0	0	0.155	0.155	0	0	0	0	0.155	0	0	0
atp6v1e2	0	0	0	0.155	0.155	0	0	0	0	0.155	0	0	0
cryl1	0	0	0	0.155	0.155	0	0	0	0	0.155	0	0	0
kl	0	0	0	0.155	0.155	0	0	0	0	0.155	0	0	0
slc28a2	0	0	0	0.155	0.155	0	0	0	0	0.155	0	0	0
cplx4	0	0	0	0.155	0.155	0	0	0	0	0.155	0	0	0
ctaa1	0	0	0	0.155	0.155	0	0	0	0	0.155	0	0	0
ca14	0	0	0	0.153	0.153	0	0	0	0	0.153	0	0	0
mcu	0	0	0	0.152	0.152	0	0	0	0	0.152	0	0	0
aldoc	0	0	0	0.15	0.15	0	0	0	0	0.15	0	0	0
grhpr	0	0	0	0.15	0.15	0	0	0	0	0.15	0	0	0
rasgef1b	0	0	0	0.15	0.15	0	0	0	0	0.15	0	0	0
nudt16	0	0	0	0.151	0.151	0	0	0	0	0.151	0	0	0
cpd	0	0	0	0.15	0.15	0	0	0	0	0.15	0	0	0
ndufa1	0	0	0	0.15	0.15	0	0	0	0	0.15	0	0	0
mcee	0	0	0	0.149	0.149	0	0	0	0	0.149	0	0	0
snora75	0	0	0	0.15	0.15	0	0	0	0	0.15	0	0	0
nme9	0	0	0	0.15	0.15	0	0	0	0	0.15	0	0	0
nxpe2	0	0	0	0.15	0.15	0	0	0	0	0.15	0	0	0
slc38a5	0	0	0	0.151	0.151	0	0	0	0	0.151	0	0	0
tmem33	0	0	0	0.151	0.151	0	0	0	0	0.151	0	0	0
pum2	0	0	0	0.152	0.152	0	0	0	0	0.152	0	0	0

slc9a4	0	0	0	0.152	0.152	0	0	0	0	0.152	0	0	0
ldhap4	0	0	0	0.152	0.152	0	0	0	0	0.152	0	0	0
slc5a12	0	0	0	0.152	0.152	0	0	0	0	0.152	0	0	0
or1d5	0	0	0	0.152	0.152	0	0	0	0	0.152	0	0	0
lgalsl	0	0	0	0.151	0.151	0	0	0	0	0.151	0	0	0
gucy2gp	0	0	0	0.151	0.151	0	0	0	0	0.151	0	0	0
tsfm	0	0	0	0.151	0.151	0	0	0	0	0.151	0	0	0
ca7	0	0	0	0.151	0.151	0	0	0	0	0.151	0	0	0
dsc2	0	0	0	0.172	0.172	0	0	0	0	0.172	0	0	0
ctxn3	0	0	0	0.172	0.172	0	0	0	0	0.172	0	0	0
lacrt	0	0	0	0.218	0.218	0	0	0	0	0.218	0	0	0
akap7	0	0	0	0.218	0.218	0	0	0	0	0.218	0	0	0
slc34a1	0	0	0	0.218	0.218	0	0	0	0	0.218	0	0	0
clcn5	0	0	0	0.219	0.219	0	0	0	0	0.219	0	0	0
pam	0	0	0	0.218	0.218	0	0	0	0	0.218	0	0	0
cryab	0	0	0	0.218	0.218	0	0	0	0	0.218	0	0	0
atp6v1e1	0	0	0	0.214	0.214	0	0	0	0	0.214	0	0	0
slc34a3	0	0	0	0.215	0.215	0	0	0	0	0.215	0	0	0
slc12a1	0	0	0	0.215	0.215	0	0	0	0	0.215	0	0	0
s100a11	0	0	0	0.217	0.217	0	0	0	0	0.217	0	0	0
slc47a2	0	0	0	0.219	0.219	0	0	0	0	0.219	0	0	0
wnk4	0	0	0	0.22	0.22	0	0	0	0	0.22	0	0	0
clip2	0	0	0	0.224	0.224	0	0	0	0	0.224	0	0	0
slc38a3	0	0	0	0.225	0.225	0	0	0	0	0.225	0	0	0
mip	0	0	0	0.225	0.225	0	0	0	0	0.225	0	0	0
cryaa	0	0	0	0.228	0.228	0	0	0	0	0.228	0	0	0
rpl10	0	0	0	0.223	0.223	0	0	0	0	0.223	0	0	0
pla2g6	0	0	0	0.223	0.223	0	0	0	0	0.223	0	0	0
entpd6	0	0	0	0.221	0.221	0	0	0	0	0.221	0	0	0
slc20a2	0	0	0	0.222	0.222	0	0	0	0	0.222	0	0	0
ces4a	0	0	0	0.222	0.222	0	0	0	0	0.222	0	0	0
crybb2p1	0	0	0	0.222	0.222	0	0	0	0	0.222	0	0	0
oa17	0	0	0	0.213	0.213	0	0	0	0	0.213	0	0	0
oap	0	0	0	0.213	0.213	0	0	0	0	0.213	0	0	0
kcnk13	0	0	0	0.2	0.2	0	0	0	0	0.2	0	0	0
hspb2	0	0	0	0.201	0.201	0	0	0	0	0.201	0	0	0
casr	0	0	0	0.201	0.201	0	0	0	0	0.201	0	0	0
sgk3	0	0	0	0.201	0.201	0	0	0	0	0.201	0	0	0
hspb9	0	0	0	0.2	0.2	0	0	0	0	0.2	0	0	0
slc16a12	0	0	0	0.199	0.199	0	0	0	0	0.199	0	0	0
p4hb	0	0	0	0.199	0.199	0	0	0	0	0.199	0	0	0

slc23a1	0	0	0	0.199	0.199	0	0	0	0	0.199	0	0	0
slc12a4	0	0	0	0.199	0.199	0	0	0	0	0.199	0	0	0
cyctp	0	0	0	0.199	0.199	0	0	0	0	0.199	0	0	0
loc100508689	0	0	0	0.201	0.201	0	0	0	0	0.201	0	0	0
slc12a3	0	0	0	0.202	0.202	0	0	0	0	0.202	0	0	0
cabp5	0	0	0	0.208	0.208	0	0	0	0	0.208	0	0	0
supv3l1	0	0	0	0.209	0.209	0	0	0	0	0.209	0	0	0
pigw	0	0	0	0.21	0.21	0	0	0	0	0.21	0	0	0
znf706	0	0	0	0.211	0.211	0	0	0	0	0.211	0	0	0
hist2h2bd	0	0	0	0.206	0.206	0	0	0	0	0.206	0	0	0
folr1p1	0	0	0	0.206	0.206	0	0	0	0	0.206	0	0	0
cabp7	0	0	0	0.202	0.202	0	0	0	0	0.202	0	0	0
slc4a4	0	0	0	0.203	0.203	0	0	0	0	0.203	0	0	0
wbp1l	0	0	0	0.204	0.204	0	0	0	0	0.204	0	0	0
phlpp2	0	0	0	0.206	0.206	0	0	0	0	0.206	0	0	0
nrn1	0	0	0	0.229	0.229	0	0	0	0	0.229	0	0	0
trpm6	0	0	0	0.23	0.23	0	0	0	0	0.23	0	0	0
mzf1	0	0	0	0.291	0.291	0	0	0	0	0.291	0	0	0
pde1a	0	0	0	0.292	0.292	0	0	0	0	0.292	0	0	0
fkbp10	0	0	0	0.293	0.293	0	0	0	0	0.293	0	0	0
prap1	0	0	0	0.3	0.3	0	0	0	0	0.3	0	0	0
gpm6a	0	0	0	0.288	0.288	0	0	0	0	0.288	0	0	0
crygb	0	0	0	0.276	0.276	0	0	0	0	0.276	0	0	0
cryba2	0	0	0	0.259	0.259	0	0	0	0	0.259	0	0	0
cryba4	0	0	0	0.262	0.262	0	0	0	0	0.262	0	0	0
crygs	0	0	0	0.263	0.263	0	0	0	0	0.263	0	0	0
semg2	0	0	0	0.272	0.272	0	0	0	0	0.272	0	0	0
xkry	0	0	0	0.301	0.301	0	0	0	0	0.301	0	0	0
rps26p25	0	0	0	0.305	0.305	0	0	0	0	0.305	0	0	0
ralbp1	0	0	0	0.373	0.373	0	0	0	0	0.373	0	0	0
sdpr	0	0	0	0.394	0.394	0	0	0	0	0.394	0	0	0
dgkd	0	0	0	0.419	0.419	0	0	0	0	0.419	0	0	0
prr14l	0	0	0	0.436	0.436	0	0	0	0	0.436	0	0	0
lcorl	0	0	0	0.353	0.353	0	0	0	0	0.353	0	0	0
vn1r17p	0	0	0	0.337	0.337	0	0	0	0	0.337	0	0	0
camsap2	0	0	0	0.315	0.315	0	0	0	0	0.315	0	0	0
trpv5	0	0	0	0.318	0.318	0	0	0	0	0.318	0	0	0
linc00598	0	0	0	0.322	0.322	0	0	0	0	0.322	0	0	0
trpv6	0	0	0	0.329	0.329	0	0	0	0	0.329	0	0	0
crygc	0	0	0	0.259	0.259	0	0	0	0	0.259	0	0	0
g3bp2	0	0	0	0.257	0.257	0	0	0	0	0.257	0	0	0

gja8	0	0	0	0.235	0.235	0	0	0	0	0.235	0	0	0
hrnr	0	0	0	0.235	0.235	0	0	0	0	0.235	0	0	0
cryba1	0	0	0	0.236	0.236	0	0	0	0	0.236	0	0	0
adcy7	0	0	0	0.237	0.237	0	0	0	0	0.237	0	0	0
fxyd2	0	0	0	0.235	0.235	0	0	0	0	0.235	0	0	0
card10	0	0	0	0.233	0.233	0	0	0	0	0.233	0	0	0
hist1h2aj	0	0	0	0.231	0.231	0	0	0	0	0.231	0	0	0
tmem141	0	0	0	0.231	0.231	0	0	0	0	0.231	0	0	0
semg1	0	0	0	0.231	0.231	0	0	0	0	0.231	0	0	0
atp2b2	0	0	0	0.233	0.233	0	0	0	0	0.233	0	0	0
atp2b1	0	0	0	0.237	0.237	0	0	0	0	0.237	0	0	0
slc12a8	0	0	0	0.239	0.239	0	0	0	0	0.239	0	0	0
pick1	0	0	0	0.255	0.255	0	0	0	0	0.255	0	0	0
atp6v1g3	0	0	0	0.255	0.255	0	0	0	0	0.255	0	0	0
crybb2	0	0	0	0.255	0.255	0	0	0	0	0.255	0	0	0
arse	0	0	0	0.257	0.257	0	0	0	0	0.257	0	0	0
prkd2	0	0	0	0.252	0.252	0	0	0	0	0.252	0	0	0
crybb1	0	0	0	0.251	0.251	0	0	0	0	0.251	0	0	0
crybb3	0	0	0	0.242	0.242	0	0	0	0	0.242	0	0	0
dyt10	0	0	0	0.244	0.244	0	0	0	0	0.244	0	0	0
camsap3	0	0	0	0.246	0.246	0	0	0	0	0.246	0	0	0
dnah8	0	0	0	0.198	0.198	0	0	0	0	0.198	0	0	0
cca5	0	0	0	0.198	0.198	0	0	0	0	0.198	0	0	0
atp1a2	0	0	0	0.178	0.178	0	0	0	0	0.178	0	0	0
atp1a1	0	0	0	0.178	0.178	0	0	0	0	0.178	0	0	0
slc8a1	0	0	0	0.179	0.179	0	0	0	0	0.179	0	0	0
pkd2l2	0	0	0	0.179	0.179	0	0	0	0	0.179	0	0	0
dhrs7	0	0	0	0.178	0.178	0	0	0	0	0.178	0	0	0
slc13a5	0	0	0	0.177	0.177	0	0	0	0	0.177	0	0	0
awat2	0	0	0	0.177	0.177	0	0	0	0	0.177	0	0	0
dolpp1	0	0	0	0.177	0.177	0	0	0	0	0.177	0	0	0
fgd2	0	0	0	0.177	0.177	0	0	0	0	0.177	0	0	0
slc8a2	0	0	0	0.177	0.177	0	0	0	0	0.177	0	0	0
slc7a14	0	0	0	0.179	0.179	0	0	0	0	0.179	0	0	0
slc41a2	0	0	0	0.179	0.179	0	0	0	0	0.179	0	0	0
atp12a	0	0	0	0.18	0.18	0	0	0	0	0.18	0	0	0
slc26a7	0	0	0	0.18	0.18	0	0	0	0	0.18	0	0	0
crybg3	0	0	0	0.18	0.18	0	0	0	0	0.18	0	0	0
got2	0	0	0	0.18	0.18	0	0	0	0	0.18	0	0	0
slc12a9	0	0	0	0.18	0.18	0	0	0	0	0.18	0	0	0
ca5a	0	0	0	0.18	0.18	0	0	0	0	0.18	0	0	0

mcoln3	0	0	0	0.179	0.179	0	0	0	0	0.179	0	0	0
ca1	0	0	0	0.179	0.179	0	0	0	0	0.179	0	0	0
cldn16	0	0	0	0.179	0.179	0	0	0	0	0.179	0	0	0
rhbg	0	0	0	0.18	0.18	0	0	0	0	0.18	0	0	0
acss1	0	0	0	0.176	0.176	0	0	0	0	0.176	0	0	0
slc6a18	0	0	0	0.176	0.176	0	0	0	0	0.176	0	0	0
ndufs8	0	0	0	0.173	0.173	0	0	0	0	0.173	0	0	0
slc36a1	0	0	0	0.173	0.173	0	0	0	0	0.173	0	0	0
trpc7	0	0	0	0.173	0.173	0	0	0	0	0.173	0	0	0
slc13a3	0	0	0	0.174	0.174	0	0	0	0	0.174	0	0	0
melk	0	0	0	0.173	0.173	0	0	0	0	0.173	0	0	0
akap16bp	0	0	0	0.173	0.173	0	0	0	0	0.173	0	0	0
cnga1	0	0	0	0.172	0.172	0	0	0	0	0.172	0	0	0
fam65a	0	0	0	0.173	0.173	0	0	0	0	0.173	0	0	0
atp1b1	0	0	0	0.173	0.173	0	0	0	0	0.173	0	0	0
btd	0	0	0	0.173	0.173	0	0	0	0	0.173	0	0	0
ca6	0	0	0	0.174	0.174	0	0	0	0	0.174	0	0	0
sestd1	0	0	0	0.174	0.174	0	0	0	0	0.174	0	0	0
hspb8	0	0	0	0.176	0.176	0	0	0	0	0.176	0	0	0
h2bfm	0	0	0	0.176	0.176	0	0	0	0	0.176	0	0	0
fxyd4	0	0	0	0.176	0.176	0	0	0	0	0.176	0	0	0
trpc5	0	0	0	0.176	0.176	0	0	0	0	0.176	0	0	0
bbx	0	0	0	0.176	0.176	0	0	0	0	0.176	0	0	0
slc4a5	0	0	0	0.176	0.176	0	0	0	0	0.176	0	0	0
atp13a4	0	0	0	0.174	0.174	0	0	0	0	0.174	0	0	0
atp1a3	0	0	0	0.174	0.174	0	0	0	0	0.174	0	0	0
ca4	0	0	0	0.174	0.174	0	0	0	0	0.174	0	0	0
impa1	0	0	0	0.175	0.175	0	0	0	0	0.175	0	0	0
atp2c1	0	0	0	0.181	0.181	0	0	0	0	0.181	0	0	0
slc20a1	0	0	0	0.181	0.181	0	0	0	0	0.181	0	0	0
slc22a2	0	0	0	0.193	0.193	0	0	0	0	0.193	0	0	0
nefll1	0	0	0	0.193	0.193	0	0	0	0	0.193	0	0	0
trpc6	0	0	0	0.194	0.194	0	0	0	0	0.194	0	0	0
dhrs4	0	0	0	0.194	0.194	0	0	0	0	0.194	0	0	0
bpnt1	0	0	0	0.193	0.193	0	0	0	0	0.193	0	0	0
glud1	0	0	0	0.193	0.193	0	0	0	0	0.193	0	0	0
dopey2	0	0	0	0.19	0.19	0	0	0	0	0.19	0	0	0
add3	0	0	0	0.192	0.192	0	0	0	0	0.192	0	0	0
slc6a9	0	0	0	0.192	0.192	0	0	0	0	0.192	0	0	0
atp2c2	0	0	0	0.192	0.192	0	0	0	0	0.192	0	0	0
wisp2	0	0	0	0.194	0.194	0	0	0	0	0.194	0	0	0

cryzp1	0	0	0	0.194	0.194	0	0	0	0	0.194	0	0	0
ca3	0	0	0	0.197	0.197	0	0	0	0	0.197	0	0	0
bsnd	0	0	0	0.197	0.197	0	0	0	0	0.197	0	0	0
tmem109	0	0	0	0.197	0.197	0	0	0	0	0.197	0	0	0
slc34a2	0	0	0	0.197	0.197	0	0	0	0	0.197	0	0	0
slc22a7	0	0	0	0.122	0.122	0	0	0	0	0.122	0	0	0
anxa6	0	0	0	0.196	0.196	0	0	0	0	0.196	0	0	0
kcnj1	0	0	0	0.195	0.195	0	0	0	0	0.195	0	0	0
hspb3	0	0	0	0.195	0.195	0	0	0	0	0.195	0	0	0
slc9a8	0	0	0	0.195	0.195	0	0	0	0	0.195	0	0	0
fxyd1	0	0	0	0.196	0.196	0	0	0	0	0.196	0	0	0
kcnj15	0	0	0	0.19	0.19	0	0	0	0	0.19	0	0	0
wnk3	0	0	0	0.19	0.19	0	0	0	0	0.19	0	0	0
zc3h12d	0	0	0	0.184	0.184	0	0	0	0	0.184	0	0	0
mccc1	0	0	0	0.184	0.184	0	0	0	0	0.184	0	0	0
clcnka	0	0	0	0.184	0.184	0	0	0	0	0.184	0	0	0
slc12a5	0	0	0	0.184	0.184	0	0	0	0	0.184	0	0	0
prh2	0	0	0	0.183	0.183	0	0	0	0	0.183	0	0	0
reps1	0	0	0	0.183	0.183	0	0	0	0	0.183	0	0	0
slc12a2	0	0	0	0.182	0.182	0	0	0	0	0.182	0	0	0
cabp2	0	0	0	0.182	0.182	0	0	0	0	0.182	0	0	0
spag1	0	0	0	0.182	0.182	0	0	0	0	0.182	0	0	0
acss2	0	0	0	0.182	0.182	0	0	0	0	0.182	0	0	0
cyb561a3	0	0	0	0.185	0.185	0	0	0	0	0.185	0	0	0
umod	0	0	0	0.185	0.185	0	0	0	0	0.185	0	0	0
phex	0	0	0	0.187	0.187	0	0	0	0	0.187	0	0	0
xk	0	0	0	0.189	0.189	0	0	0	0	0.189	0	0	0
atp1b2	0	0	0	0.189	0.189	0	0	0	0	0.189	0	0	0
atp2b3	0	0	0	0.19	0.19	0	0	0	0	0.19	0	0	0
slc26a1	0	0	0	0.187	0.187	0	0	0	0	0.187	0	0	0
slc4a7	0	0	0	0.186	0.186	0	0	0	0	0.186	0	0	0
trpm4	0	0	0	0.186	0.186	0	0	0	0	0.186	0	0	0
edf1	0	0	0	0.186	0.186	0	0	0	0	0.186	0	0	0
rnase4	0	0	0	0.186	0.186	0	0	0	0	0.186	0	0	0
hist1h1t	0	0	0	0.145	0.145	0	0	0	0	0.145	0	0	0
bw18	0	0	0	0.196	0.196	0	0	0	0	0.196	0	0	0
aacs	0	0	0	0.127	0.127	0	0	0	0	0.127	0	0	0
slc7a5	0	0	0	0.127	0.127	0	0	0	0	0.127	0	0	0
slc7a9	0	0	0	0.127	0.127	0	0	0	0	0.127	0	0	0
hpca	0	0	0	0.127	0.127	0	0	0	0	0.127	0	0	0
cox4i2	0	0	0	0.127	0.127	0	0	0	0	0.127	0	0	0

slc19a3	0	0	0	0.127	0.127	0	0	0	0	0.127	0	0	0
pdcd7	0	0	0	0.127	0.127	0	0	0	0	0.127	0	0	0
mcoln1	0	0	0	0.127	0.127	0	0	0	0	0.127	0	0	0
znf354b	0	0	0	0.127	0.127	0	0	0	0	0.127	0	0	0
htn3	0	0	0	0.127	0.127	0	0	0	0	0.127	0	0	0
aldoa	0	0	0	0.127	0.127	0	0	0	0	0.127	0	0	0
cln5	0	0	0	0.127	0.127	0	0	0	0	0.127	0	0	0
mon1b	0	0	0	0.128	0.128	0	0	0	0	0.128	0	0	0
bckdha	0	0	0	0.128	0.128	0	0	0	0	0.128	0	0	0
slc6a1	0	0	0	0.128	0.128	0	0	0	0	0.128	0	0	0
pnp	0	0	0	0.128	0.128	0	0	0	0	0.128	0	0	0
mgr3	0	0	0	0.128	0.128	0	0	0	0	0.128	0	0	0
oxct2	0	0	0	0.128	0.128	0	0	0	0	0.128	0	0	0
rnu1-1	0	0	0	0.128	0.128	0	0	0	0	0.128	0	0	0
slc4a9	0	0	0	0.128	0.128	0	0	0	0	0.128	0	0	0
wnk2	0	0	0	0.128	0.128	0	0	0	0	0.128	0	0	0
ptp4a2p2	0	0	0	0.128	0.128	0	0	0	0	0.128	0	0	0
rtdr1	0	0	0	0.127	0.127	0	0	0	0	0.127	0	0	0
zrsr2	0	0	0	0.127	0.127	0	0	0	0	0.127	0	0	0
qsox1	0	0	0	0.126	0.126	0	0	0	0	0.126	0	0	0
serinc3	0	0	0	0.127	0.127	0	0	0	0	0.127	0	0	0
ckmt1b	0	0	0	0.127	0.127	0	0	0	0	0.127	0	0	0
psma5	0	0	0	0.127	0.127	0	0	0	0	0.127	0	0	0
spata21	0	0	0	0.126	0.126	0	0	0	0	0.126	0	0	0
kcnn2	0	0	0	0.126	0.126	0	0	0	0	0.126	0	0	0
spam1	0	0	0	0.126	0.126	0	0	0	0	0.126	0	0	0
gng5	0	0	0	0.126	0.126	0	0	0	0	0.126	0	0	0
kcnj13	0	0	0	0.126	0.126	0	0	0	0	0.126	0	0	0
kcnj12	0	0	0	0.126	0.126	0	0	0	0	0.126	0	0	0
rnu6atac	0	0	0	0.127	0.127	0	0	0	0	0.127	0	0	0
ppatp1	0	0	0	0.127	0.127	0	0	0	0	0.127	0	0	0
defb107a	0	0	0	0.127	0.127	0	0	0	0	0.127	0	0	0
tmem176b	0	0	0	0.127	0.127	0	0	0	0	0.127	0	0	0
mrs2	0	0	0	0.127	0.127	0	0	0	0	0.127	0	0	0
slc22a8	0	0	0	0.127	0.127	0	0	0	0	0.127	0	0	0
defb107b	0	0	0	0.127	0.127	0	0	0	0	0.127	0	0	0
defb105b	0	0	0	0.127	0.127	0	0	0	0	0.127	0	0	0
paicsp2	0	0	0	0.127	0.127	0	0	0	0	0.127	0	0	0
paicsp1	0	0	0	0.127	0.127	0	0	0	0	0.127	0	0	0
loc285232	0	0	0	0.127	0.127	0	0	0	0	0.127	0	0	0
sypl2	0	0	0	0.127	0.127	0	0	0	0	0.127	0	0	0

tcp10	0	0	0	0.128	0.128	0	0	0	0	0.128	0	0	0
galnt3	0	0	0	0.128	0.128	0	0	0	0	0.128	0	0	0
bcat1	0	0	0	0.13	0.13	0	0	0	0	0.13	0	0	0
slc15a1	0	0	0	0.13	0.13	0	0	0	0	0.13	0	0	0
slc10a7	0	0	0	0.13	0.13	0	0	0	0	0.13	0	0	0
mfsd11	0	0	0	0.13	0.13	0	0	0	0	0.13	0	0	0
prdx5	0	0	0	0.13	0.13	0	0	0	0	0.13	0	0	0
pvalb	0	0	0	0.13	0.13	0	0	0	0	0.13	0	0	0
rp9	0	0	0	0.13	0.13	0	0	0	0	0.13	0	0	0
pygm	0	0	0	0.13	0.13	0	0	0	0	0.13	0	0	0
defb125	0	0	0	0.13	0.13	0	0	0	0	0.13	0	0	0
atp2a1	0	0	0	0.13	0.13	0	0	0	0	0.13	0	0	0
slc15a3	0	0	0	0.13	0.13	0	0	0	0	0.13	0	0	0
aebp1	0	0	0	0.13	0.13	0	0	0	0	0.13	0	0	0
dhfrp1	0	0	0	0.131	0.131	0	0	0	0	0.131	0	0	0
mir4435-1hg	0	0	0	0.131	0.131	0	0	0	0	0.131	0	0	0
linc00984	0	0	0	0.131	0.131	0	0	0	0	0.131	0	0	0
rcvrn	0	0	0	0.131	0.131	0	0	0	0	0.131	0	0	0
pnlip	0	0	0	0.131	0.131	0	0	0	0	0.131	0	0	0
tfb2m	0	0	0	0.131	0.131	0	0	0	0	0.131	0	0	0
c12orf75	0	0	0	0.13	0.13	0	0	0	0	0.13	0	0	0
atp6v1g1	0	0	0	0.13	0.13	0	0	0	0	0.13	0	0	0
aldh5a1	0	0	0	0.13	0.13	0	0	0	0	0.13	0	0	0
pfas	0	0	0	0.13	0.13	0	0	0	0	0.13	0	0	0
dupd1	0	0	0	0.13	0.13	0	0	0	0	0.13	0	0	0
slc13a4	0	0	0	0.13	0.13	0	0	0	0	0.13	0	0	0
agps	0	0	0	0.128	0.128	0	0	0	0	0.128	0	0	0
slc26a8	0	0	0	0.129	0.129	0	0	0	0	0.129	0	0	0
polr2g	0	0	0	0.129	0.129	0	0	0	0	0.129	0	0	0
pkd2l1	0	0	0	0.129	0.129	0	0	0	0	0.129	0	0	0
hmgn2	0	0	0	0.128	0.128	0	0	0	0	0.128	0	0	0
atp6v1b2	0	0	0	0.128	0.128	0	0	0	0	0.128	0	0	0
tbcel	0	0	0	0.128	0.128	0	0	0	0	0.128	0	0	0
guca2a	0	0	0	0.128	0.128	0	0	0	0	0.128	0	0	0
ndufa7	0	0	0	0.128	0.128	0	0	0	0	0.128	0	0	0
rnu6-2	0	0	0	0.128	0.128	0	0	0	0	0.128	0	0	0
agxt	0	0	0	0.129	0.129	0	0	0	0	0.129	0	0	0
srsf2	0	0	0	0.129	0.129	0	0	0	0	0.129	0	0	0
cnnm3	0	0	0	0.13	0.13	0	0	0	0	0.13	0	0	0
camsap1	0	0	0	0.13	0.13	0	0	0	0	0.13	0	0	0
med22	0	0	0	0.13	0.13	0	0	0	0	0.13	0	0	0

otos	0	0	0	0.13	0.13	0	0	0	0	0.13	0	0	0
pld6	0	0	0	0.129	0.129	0	0	0	0	0.129	0	0	0
kcnip1	0	0	0	0.129	0.129	0	0	0	0	0.129	0	0	0
dcxr	0	0	0	0.129	0.129	0	0	0	0	0.129	0	0	0
slc5a4	0	0	0	0.129	0.129	0	0	0	0	0.129	0	0	0
kcnj2	0	0	0	0.129	0.129	0	0	0	0	0.129	0	0	0
kcnq2	0	0	0	0.129	0.129	0	0	0	0	0.129	0	0	0
slc7a6	0	0	0	0.126	0.126	0	0	0	0	0.126	0	0	0
fbxo11	0	0	0	0.126	0.126	0	0	0	0	0.126	0	0	0
akr1b1p3	0	0	0	0.124	0.124	0	0	0	0	0.124	0	0	0
akr1b1p4	0	0	0	0.124	0.124	0	0	0	0	0.124	0	0	0
mtmr2	0	0	0	0.124	0.124	0	0	0	0	0.124	0	0	0
catsperg	0	0	0	0.124	0.124	0	0	0	0	0.124	0	0	0
akr1b1p1	0	0	0	0.124	0.124	0	0	0	0	0.124	0	0	0
orai1	0	0	0	0.124	0.124	0	0	0	0	0.124	0	0	0
gnpda1	0	0	0	0.123	0.123	0	0	0	0	0.123	0	0	0
tvp23a	0	0	0	0.123	0.123	0	0	0	0	0.123	0	0	0
tvp23b	0	0	0	0.123	0.123	0	0	0	0	0.123	0	0	0
stim1	0	0	0	0.123	0.123	0	0	0	0	0.123	0	0	0
znf79	0	0	0	0.124	0.124	0	0	0	0	0.124	0	0	0
gspt1	0	0	0	0.124	0.124	0	0	0	0	0.124	0	0	0
vn1r2	0	0	0	0.124	0.124	0	0	0	0	0.124	0	0	0
vn1r5	0	0	0	0.124	0.124	0	0	0	0	0.124	0	0	0
vn1r4	0	0	0	0.124	0.124	0	0	0	0	0.124	0	0	0
spin1	0	0	0	0.124	0.124	0	0	0	0	0.124	0	0	0
vn1r3	0	0	0	0.124	0.124	0	0	0	0	0.124	0	0	0
kcnc2	0	0	0	0.124	0.124	0	0	0	0	0.124	0	0	0
alpp	0	0	0	0.124	0.124	0	0	0	0	0.124	0	0	0
pgk1p2	0	0	0	0.124	0.124	0	0	0	0	0.124	0	0	0
rp24	0	0	0	0.124	0.124	0	0	0	0	0.124	0	0	0
pdha2	0	0	0	0.124	0.124	0	0	0	0	0.124	0	0	0
hlcs	0	0	0	0.123	0.123	0	0	0	0	0.123	0	0	0
c10orf67	0	0	0	0.123	0.123	0	0	0	0	0.123	0	0	0
naalad2	0	0	0	0.122	0.122	0	0	0	0	0.122	0	0	0
b3gntl1	0	0	0	0.123	0.123	0	0	0	0	0.123	0	0	0
atad2b	0	0	0	0.123	0.123	0	0	0	0	0.123	0	0	0
isoc1	0	0	0	0.123	0.123	0	0	0	0	0.123	0	0	0
naspp1	0	0	0	0.122	0.122	0	0	0	0	0.122	0	0	0
slc9a6	0	0	0	0.122	0.122	0	0	0	0	0.122	0	0	0
msto1	0	0	0	0.122	0.122	0	0	0	0	0.122	0	0	0
oc90	0	0	0	0.122	0.122	0	0	0	0	0.122	0	0	0

rnu4atac	0	0	0	0.122	0.122	0	0	0	0	0.122	0	0	0
fam221b	0	0	0	0.122	0.122	0	0	0	0	0.122	0	0	0
obp2b	0	0	0	0.123	0.123	0	0	0	0	0.123	0	0	0
grk1	0	0	0	0.123	0.123	0	0	0	0	0.123	0	0	0
gpaa1p1	0	0	0	0.123	0.123	0	0	0	0	0.123	0	0	0
nek8	0	0	0	0.123	0.123	0	0	0	0	0.123	0	0	0
lrrc26	0	0	0	0.123	0.123	0	0	0	0	0.123	0	0	0
dsel	0	0	0	0.123	0.123	0	0	0	0	0.123	0	0	0
duh	0	0	0	0.123	0.123	0	0	0	0	0.123	0	0	0
dnase1l1	0	0	0	0.123	0.123	0	0	0	0	0.123	0	0	0
kcnk12	0	0	0	0.145	0.145	0	0	0	0	0.145	0	0	0
cdc73	0	0	0	0.123	0.123	0	0	0	0	0.123	0	0	0
slc7a13	0	0	0	0.123	0.123	0	0	0	0	0.123	0	0	0
dnah2	0	0	0	0.123	0.123	0	0	0	0	0.123	0	0	0
lypla1	0	0	0	0.124	0.124	0	0	0	0	0.124	0	0	0
slc22a25	0	0	0	0.124	0.124	0	0	0	0	0.124	0	0	0
orai3	0	0	0	0.125	0.125	0	0	0	0	0.125	0	0	0
btn1a1	0	0	0	0.125	0.125	0	0	0	0	0.125	0	0	0
plscr2	0	0	0	0.125	0.125	0	0	0	0	0.125	0	0	0
tor1b	0	0	0	0.126	0.126	0	0	0	0	0.126	0	0	0
cyp27b1	0	0	0	0.125	0.125	0	0	0	0	0.125	0	0	0
pdss2	0	0	0	0.125	0.125	0	0	0	0	0.125	0	0	0
barhl1	0	0	0	0.125	0.125	0	0	0	0	0.125	0	0	0
prr4	0	0	0	0.125	0.125	0	0	0	0	0.125	0	0	0
mocs1	0	0	0	0.125	0.125	0	0	0	0	0.125	0	0	0
anxa2p3	0	0	0	0.125	0.125	0	0	0	0	0.125	0	0	0
dmrtc1	0	0	0	0.126	0.126	0	0	0	0	0.126	0	0	0
mrpl46	0	0	0	0.126	0.126	0	0	0	0	0.126	0	0	0
slc22a9	0	0	0	0.126	0.126	0	0	0	0	0.126	0	0	0
cela3a	0	0	0	0.126	0.126	0	0	0	0	0.126	0	0	0
cacna2d4	0	0	0	0.126	0.126	0	0	0	0	0.126	0	0	0
smox	0	0	0	0.126	0.126	0	0	0	0	0.126	0	0	0
scel	0	0	0	0.126	0.126	0	0	0	0	0.126	0	0	0
dpy19l2p2	0	0	0	0.126	0.126	0	0	0	0	0.126	0	0	0
lpin2	0	0	0	0.126	0.126	0	0	0	0	0.126	0	0	0
hdgfrp3	0	0	0	0.126	0.126	0	0	0	0	0.126	0	0	0
ambp	0	0	0	0.126	0.126	0	0	0	0	0.126	0	0	0
dpy19l2p4	0	0	0	0.126	0.126	0	0	0	0	0.126	0	0	0
bpifc	0	0	0	0.125	0.125	0	0	0	0	0.125	0	0	0
bpifb6	0	0	0	0.125	0.125	0	0	0	0	0.125	0	0	0
ccbl2	0	0	0	0.124	0.124	0	0	0	0	0.124	0	0	0

rpe	0	0	0	0.124	0.124	0	0	0	0	0.124	0	0	0
slco4c1	0	0	0	0.124	0.124	0	0	0	0	0.124	0	0	0
hsf4	0	0	0	0.125	0.125	0	0	0	0	0.125	0	0	0
gba3	0	0	0	0.124	0.124	0	0	0	0	0.124	0	0	0
ppp1r1a	0	0	0	0.124	0.124	0	0	0	0	0.124	0	0	0
mir26a1	0	0	0	0.124	0.124	0	0	0	0	0.124	0	0	0
ldha	0	0	0	0.124	0.124	0	0	0	0	0.124	0	0	0
slc31a1	0	0	0	0.124	0.124	0	0	0	0	0.124	0	0	0
hvcn1	0	0	0	0.124	0.124	0	0	0	0	0.124	0	0	0
ppp2r1b	0	0	0	0.125	0.125	0	0	0	0	0.125	0	0	0
ift88	0	0	0	0.125	0.125	0	0	0	0	0.125	0	0	0
glt1d1	0	0	0	0.125	0.125	0	0	0	0	0.125	0	0	0
dhx9p1	0	0	0	0.125	0.125	0	0	0	0	0.125	0	0	0
chkb	0	0	0	0.125	0.125	0	0	0	0	0.125	0	0	0
tceal8	0	0	0	0.125	0.125	0	0	0	0	0.125	0	0	0
dfn6	0	0	0	0.125	0.125	0	0	0	0	0.125	0	0	0
pla2g2f	0	0	0	0.125	0.125	0	0	0	0	0.125	0	0	0
ldoc1	0	0	0	0.125	0.125	0	0	0	0	0.125	0	0	0
entpd8	0	0	0	0.125	0.125	0	0	0	0	0.125	0	0	0
bdh1	0	0	0	0.125	0.125	0	0	0	0	0.125	0	0	0
slc36a2	0	0	0	0.131	0.131	0	0	0	0	0.131	0	0	0
kiaa1244	0	0	0	0.125	0.125	0	0	0	0	0.125	0	0	0
mng2	0	0	0	0.14	0.14	0	0	0	0	0.14	0	0	0
slc22a20	0	0	0	0.14	0.14	0	0	0	0	0.14	0	0	0
ggt5	0	0	0	0.14	0.14	0	0	0	0	0.14	0	0	0
snhg16	0	0	0	0.14	0.14	0	0	0	0	0.14	0	0	0
eppk1	0	0	0	0.14	0.14	0	0	0	0	0.14	0	0	0
aga	0	0	0	0.14	0.14	0	0	0	0	0.14	0	0	0
ippk	0	0	0	0.139	0.139	0	0	0	0	0.139	0	0	0
fxyd7	0	0	0	0.14	0.14	0	0	0	0	0.14	0	0	0
dfna59	0	0	0	0.14	0.14	0	0	0	0	0.14	0	0	0
fpgt	0	0	0	0.14	0.14	0	0	0	0	0.14	0	0	0
gamt	0	0	0	0.14	0.14	0	0	0	0	0.14	0	0	0
slc10a2	0	0	0	0.14	0.14	0	0	0	0	0.14	0	0	0
entpd3	0	0	0	0.141	0.141	0	0	0	0	0.141	0	0	0
nd6	0	0	0	0.141	0.141	0	0	0	0	0.141	0	0	0
srm	0	0	0	0.141	0.141	0	0	0	0	0.141	0	0	0
pdzd11	0	0	0	0.141	0.141	0	0	0	0	0.141	0	0	0
amdp1	0	0	0	0.141	0.141	0	0	0	0	0.141	0	0	0
ipp	0	0	0	0.141	0.141	0	0	0	0	0.141	0	0	0
tuba4b	0	0	0	0.14	0.14	0	0	0	0	0.14	0	0	0

bpifa4p	0	0	0	0.14	0.14	0	0	0	0	0.14	0	0	0
pth	0	0	0	0.141	0.141	0	0	0	0	0.141	0	0	0
mdp1	0	0	0	0.141	0.141	0	0	0	0	0.141	0	0	0
dpep1	0	0	0	0.139	0.139	0	0	0	0	0.139	0	0	0
caprin2	0	0	0	0.139	0.139	0	0	0	0	0.139	0	0	0
cox6b1p1	0	0	0	0.138	0.138	0	0	0	0	0.138	0	0	0
mettl7b	0	0	0	0.139	0.139	0	0	0	0	0.139	0	0	0
pnpla4	0	0	0	0.139	0.139	0	0	0	0	0.139	0	0	0
c2cd2l	0	0	0	0.139	0.139	0	0	0	0	0.139	0	0	0
cox6b1p2	0	0	0	0.138	0.138	0	0	0	0	0.138	0	0	0
cox6b1p4	0	0	0	0.138	0.138	0	0	0	0	0.138	0	0	0
kcna5	0	0	0	0.138	0.138	0	0	0	0	0.138	0	0	0
atp6v1c2	0	0	0	0.138	0.138	0	0	0	0	0.138	0	0	0
adssl1	0	0	0	0.138	0.138	0	0	0	0	0.138	0	0	0
c21orf33	0	0	0	0.138	0.138	0	0	0	0	0.138	0	0	0
nagk	0	0	0	0.139	0.139	0	0	0	0	0.139	0	0	0
ext3	0	0	0	0.139	0.139	0	0	0	0	0.139	0	0	0
hmga1	0	0	0	0.139	0.139	0	0	0	0	0.139	0	0	0
atoh7	0	0	0	0.139	0.139	0	0	0	0	0.139	0	0	0
ca5ap1	0	0	0	0.139	0.139	0	0	0	0	0.139	0	0	0
gca	0	0	0	0.139	0.139	0	0	0	0	0.139	0	0	0
slc1a6	0	0	0	0.139	0.139	0	0	0	0	0.139	0	0	0
prdm8	0	0	0	0.139	0.139	0	0	0	0	0.139	0	0	0
pfdn6	0	0	0	0.139	0.139	0	0	0	0	0.139	0	0	0
slc7a8	0	0	0	0.139	0.139	0	0	0	0	0.139	0	0	0
cnga4	0	0	0	0.139	0.139	0	0	0	0	0.139	0	0	0
kcnk5	0	0	0	0.139	0.139	0	0	0	0	0.139	0	0	0
nphs1	0	0	0	0.141	0.141	0	0	0	0	0.141	0	0	0
atp4a	0	0	0	0.141	0.141	0	0	0	0	0.141	0	0	0
sms	0	0	0	0.144	0.144	0	0	0	0	0.144	0	0	0
cbln3	0	0	0	0.144	0.144	0	0	0	0	0.144	0	0	0
nav1	0	0	0	0.144	0.144	0	0	0	0	0.144	0	0	0
eef1b2p3	0	0	0	0.144	0.144	0	0	0	0	0.144	0	0	0
slc1a1	0	0	0	0.144	0.144	0	0	0	0	0.144	0	0	0
rnase2	0	0	0	0.144	0.144	0	0	0	0	0.144	0	0	0
gng10	0	0	0	0.143	0.143	0	0	0	0	0.143	0	0	0
atp2a2	0	0	0	0.143	0.143	0	0	0	0	0.143	0	0	0
slc15a2	0	0	0	0.143	0.143	0	0	0	0	0.143	0	0	0
slc41a1	0	0	0	0.143	0.143	0	0	0	0	0.143	0	0	0
tesc	0	0	0	0.144	0.144	0	0	0	0	0.144	0	0	0
rmdn3	0	0	0	0.144	0.144	0	0	0	0	0.144	0	0	0

clca1	0	0	0	0.143	0.143	0	0	0	0	0.143	0	0	0
anxa4	0	0	0	0.143	0.143	0	0	0	0	0.143	0	0	0
atp6v0e1	0	0	0	0.143	0.143	0	0	0	0	0.143	0	0	0
tnp1	0	0	0	0.143	0.143	0	0	0	0	0.143	0	0	0
nudt11	0	0	0	0.143	0.143	0	0	0	0	0.143	0	0	0
mir96	0	0	0	0.144	0.144	0	0	0	0	0.144	0	0	0
emp3	0	0	0	0.144	0.144	0	0	0	0	0.144	0	0	0
lrrc23	0	0	0	0.143	0.143	0	0	0	0	0.143	0	0	0
calb1	0	0	0	0.144	0.144	0	0	0	0	0.144	0	0	0
slc6a17	0	0	0	0.131	0.131	0	0	0	0	0.131	0	0	0
atp1b4	0	0	0	0.143	0.143	0	0	0	0	0.143	0	0	0
tmed2	0	0	0	0.143	0.143	0	0	0	0	0.143	0	0	0
slc16a6	0	0	0	0.142	0.142	0	0	0	0	0.142	0	0	0
odcp	0	0	0	0.142	0.142	0	0	0	0	0.142	0	0	0
slc9a1	0	0	0	0.142	0.142	0	0	0	0	0.142	0	0	0
aldh4a1	0	0	0	0.142	0.142	0	0	0	0	0.142	0	0	0
gsm1	0	0	0	0.142	0.142	0	0	0	0	0.142	0	0	0
msrb1	0	0	0	0.142	0.142	0	0	0	0	0.142	0	0	0
rwdd2b	0	0	0	0.141	0.141	0	0	0	0	0.141	0	0	0
ca2	0	0	0	0.141	0.141	0	0	0	0	0.141	0	0	0
mmvp2	0	0	0	0.141	0.141	0	0	0	0	0.141	0	0	0
krt12	0	0	0	0.141	0.141	0	0	0	0	0.141	0	0	0
slc6a6	0	0	0	0.142	0.142	0	0	0	0	0.142	0	0	0
tectb	0	0	0	0.142	0.142	0	0	0	0	0.142	0	0	0
slc9b2	0	0	0	0.143	0.143	0	0	0	0	0.143	0	0	0
trpc1	0	0	0	0.143	0.143	0	0	0	0	0.143	0	0	0
vdac1p5	0	0	0	0.143	0.143	0	0	0	0	0.143	0	0	0
vdac1p4	0	0	0	0.143	0.143	0	0	0	0	0.143	0	0	0
atp4b	0	0	0	0.142	0.142	0	0	0	0	0.142	0	0	0
tmem59l	0	0	0	0.142	0.142	0	0	0	0	0.142	0	0	0
atp6v1a	0	0	0	0.142	0.142	0	0	0	0	0.142	0	0	0
gja10	0	0	0	0.142	0.142	0	0	0	0	0.142	0	0	0
slc14a2	0	0	0	0.142	0.142	0	0	0	0	0.142	0	0	0
gldc	0	0	0	0.138	0.138	0	0	0	0	0.138	0	0	0
loc390998	0	0	0	0.14	0.14	0	0	0	0	0.14	0	0	0
espn	0	0	0	0.133	0.133	0	0	0	0	0.133	0	0	0
tprkb	0	0	0	0.133	0.133	0	0	0	0	0.133	0	0	0
bzrap1	0	0	0	0.133	0.133	0	0	0	0	0.133	0	0	0
prmt7	0	0	0	0.134	0.134	0	0	0	0	0.134	0	0	0
galk1	0	0	0	0.133	0.133	0	0	0	0	0.133	0	0	0
slc6a5	0	0	0	0.133	0.133	0	0	0	0	0.133	0	0	0

nudt9	0	0	0	0.133	0.133	0	0	0	0	0.133	0	0	0
usf1	0	0	0	0.133	0.133	0	0	0	0	0.133	0	0	0
extl2	0	0	0	0.133	0.133	0	0	0	0	0.133	0	0	0
glud1p3	0	0	0	0.133	0.133	0	0	0	0	0.133	0	0	0
sord	0	0	0	0.134	0.134	0	0	0	0	0.134	0	0	0
slc24a3	0	0	0	0.134	0.134	0	0	0	0	0.134	0	0	0
spns3	0	0	0	0.134	0.134	0	0	0	0	0.134	0	0	0
svopl	0	0	0	0.134	0.134	0	0	0	0	0.134	0	0	0
linc00470	0	0	0	0.135	0.135	0	0	0	0	0.135	0	0	0
pgm2l1	0	0	0	0.135	0.135	0	0	0	0	0.135	0	0	0
fabp6	0	0	0	0.134	0.134	0	0	0	0	0.134	0	0	0
glt8d2	0	0	0	0.134	0.134	0	0	0	0	0.134	0	0	0
ak1	0	0	0	0.134	0.134	0	0	0	0	0.134	0	0	0
hs3st3a1	0	0	0	0.134	0.134	0	0	0	0	0.134	0	0	0
sczd11	0	0	0	0.134	0.134	0	0	0	0	0.134	0	0	0
nudt21	0	0	0	0.134	0.134	0	0	0	0	0.134	0	0	0
barhl2	0	0	0	0.132	0.132	0	0	0	0	0.132	0	0	0
hspb1p2	0	0	0	0.132	0.132	0	0	0	0	0.132	0	0	0
trpc2	0	0	0	0.131	0.131	0	0	0	0	0.131	0	0	0
glud1p4	0	0	0	0.131	0.131	0	0	0	0	0.131	0	0	0
jph1	0	0	0	0.131	0.131	0	0	0	0	0.131	0	0	0
brms1	0	0	0	0.131	0.131	0	0	0	0	0.131	0	0	0
hagh	0	0	0	0.131	0.131	0	0	0	0	0.131	0	0	0
cnnm1	0	0	0	0.131	0.131	0	0	0	0	0.131	0	0	0
pde7a	0	0	0	0.131	0.131	0	0	0	0	0.131	0	0	0
cbx2	0	0	0	0.131	0.131	0	0	0	0	0.131	0	0	0
gapdh	0	0	0	0.131	0.131	0	0	0	0	0.131	0	0	0
cln3	0	0	0	0.131	0.131	0	0	0	0	0.131	0	0	0
sat1	0	0	0	0.131	0.131	0	0	0	0	0.131	0	0	0
proa	0	0	0	0.131	0.131	0	0	0	0	0.131	0	0	0
hpv6ai1	0	0	0	0.132	0.132	0	0	0	0	0.132	0	0	0
otc	0	0	0	0.132	0.132	0	0	0	0	0.132	0	0	0
kcna10	0	0	0	0.132	0.132	0	0	0	0	0.132	0	0	0
kcnj4	0	0	0	0.132	0.132	0	0	0	0	0.132	0	0	0
pax2	0	0	0	0.138	0.138	0	0	0	0	0.138	0	0	0
ofd1p17	0	0	0	0.132	0.132	0	0	0	0	0.132	0	0	0
mopcb1	0	0	0	0.132	0.132	0	0	0	0	0.132	0	0	0
calml5	0	0	0	0.132	0.132	0	0	0	0	0.132	0	0	0
kcnc1	0	0	0	0.132	0.132	0	0	0	0	0.132	0	0	0
ofd1p18y	0	0	0	0.132	0.132	0	0	0	0	0.132	0	0	0
prkcg	0	0	0	0.135	0.135	0	0	0	0	0.135	0	0	0

rnase1	0	0	0	0.132	0.132	0	0	0	0	0.132	0	0	0
oaz2	0	0	0	0.138	0.138	0	0	0	0	0.138	0	0	0
mctp1	0	0	0	0.137	0.137	0	0	0	0	0.137	0	0	0
skcg-1	0	0	0	0.137	0.137	0	0	0	0	0.137	0	0	0
oma1	0	0	0	0.138	0.138	0	0	0	0	0.138	0	0	0
dyt15	0	0	0	0.137	0.137	0	0	0	0	0.137	0	0	0
aadat	0	0	0	0.137	0.137	0	0	0	0	0.137	0	0	0
ndufa4	0	0	0	0.138	0.138	0	0	0	0	0.138	0	0	0
gfpt1	0	0	0	0.136	0.136	0	0	0	0	0.136	0	0	0
bw37	0	0	0	0.136	0.136	0	0	0	0	0.136	0	0	0
gabrr1	0	0	0	0.136	0.136	0	0	0	0	0.136	0	0	0
aim1	0	0	0	0.137	0.137	0	0	0	0	0.137	0	0	0
ccdc114	0	0	0	0.137	0.137	0	0	0	0	0.137	0	0	0
anxa3	0	0	0	0.137	0.137	0	0	0	0	0.137	0	0	0
sds	0	0	0	0.137	0.137	0	0	0	0	0.137	0	0	0
prdx6	0	0	0	0.137	0.137	0	0	0	0	0.137	0	0	0
hnrnpdl	0	0	0	0.137	0.137	0	0	0	0	0.137	0	0	0
thoc7	0	0	0	0.137	0.137	0	0	0	0	0.137	0	0	0
cpxm2	0	0	0	0.137	0.137	0	0	0	0	0.137	0	0	0
usp51	0	0	0	0.138	0.138	0	0	0	0	0.138	0	0	0
glulp1	0	0	0	0.135	0.135	0	0	0	0	0.135	0	0	0
slc5a1	0	0	0	0.137	0.137	0	0	0	0	0.137	0	0	0
loc100093631	0	0	0	0.136	0.136	0	0	0	0	0.136	0	0	0
gtf2ip1	0	0	0	0.136	0.136	0	0	0	0	0.136	0	0	0
slc16a8	0	0	0	0.135	0.135	0	0	0	0	0.135	0	0	0
atp6v0b	0	0	0	0.135	0.135	0	0	0	0	0.135	0	0	0
acads	0	0	0	0.135	0.135	0	0	0	0	0.135	0	0	0
hmgcl	0	0	0	0.135	0.135	0	0	0	0	0.135	0	0	0
kcnb2	0	0	0	0.135	0.135	0	0	0	0	0.135	0	0	0
sca25	0	0	0	0.135	0.135	0	0	0	0	0.135	0	0	0
glulp2	0	0	0	0.135	0.135	0	0	0	0	0.135	0	0	0
glulp3	0	0	0	0.135	0.135	0	0	0	0	0.135	0	0	0
glulp4	0	0	0	0.135	0.135	0	0	0	0	0.135	0	0	0
surf6	0	0	0	0.135	0.135	0	0	0	0	0.135	0	0	0
pawr	0	0	0	0.135	0.135	0	0	0	0	0.135	0	0	0
amelx	0	0	0	0.135	0.135	0	0	0	0	0.135	0	0	0
slc25a18	0	0	0	0.136	0.136	0	0	0	0	0.136	0	0	0
ptbp3	0	0	0	0.136	0.136	0	0	0	0	0.136	0	0	0
gdpd5	0	0	0	0.136	0.136	0	0	0	0	0.136	0	0	0
slc16a10	0	0	0	0.136	0.136	0	0	0	0	0.136	0	0	0
entpd2	0	0	0	0.136	0.136	0	0	0	0	0.136	0	0	0

loc2748	0	0	0	0.136	0.136	0	0	0	0	0.136	0	0	0
chp2	0	0	0	0.137	0.137	0	0	0	0	0.137	0	0	0
glud1p2	0	0	0	0.136	0.136	0	0	0	0	0.136	0	0	0
hist1h1e	0	0	0	0.136	0.136	0	0	0	0	0.136	0	0	0
hist1h1d	0	0	0	0.136	0.136	0	0	0	0	0.136	0	0	0
begain	0	0	0	0	0	0	0	0	0.102	0	0.106	0.105	0
cntnap4	0	0	0	0	0	0	0	0	0.104	0	0.102	0.102	0
stx8	0	0	0	0	0	0	0	0	0.103	0	0.119	0.118	0
izumo1	0	0	0	0	0	0	0	0	0.101	0	0.132	0.132	0
atp6v1h	0	0	0	0	0	0	0	0	0.1	0	0.12	0.119	0
mapk4	0	0	0	0	0	0	0	0	0	0	0.12	0.119	0.101
mrx17	0	0	0	0	0	0	0	0	0	0	0.146	0.146	0.1
mmp23a	0	0	0	0	0	0	0	0	0	0	0.101	0.101	0.101
mrx18	0	0	0	0	0	0	0	0	0	0	0.146	0.146	0.1
caskin2	0	0	0	0	0	0	0	0	0.104	0	0	0	0.104
rap1ap	0	0	0	0	0	0	0	0	0.105	0	0	0	0.113
rep15	0	0	0	0	0	0	0	0	0.1	0	0	0	0.119
nphp4	0	0	0	0	0	0	0	0	0.103	0	0	0	0.104
mir196a1	0	0	0.203	0	0	0.203	0	0	0	0	0	0	0
znf510	0	0	0.204	0	0	0.204	0	0	0	0	0	0	0
iddm3	0	0	0.204	0	0	0.204	0	0	0	0	0	0	0
htr1e	0	0	0.215	0	0	0.215	0	0	0	0	0	0	0
mir122	0	0	0.22	0	0	0.22	0	0	0	0	0	0	0
moxd1	0	0	0.221	0	0	0.221	0	0	0	0	0	0	0
tor2a	0	0	0.217	0	0	0.217	0	0	0	0	0	0	0
tmbim6	0	0	0.179	0	0	0.179	0	0	0	0	0	0	0
crip2	0	0	0.201	0	0	0.201	0	0	0	0	0	0	0
alas1	0	0	0.216	0	0	0.216	0	0	0	0	0	0	0
pradc1	0	0	0.21	0	0	0.21	0	0	0	0	0	0	0
brd9	0	0	0.195	0	0	0.195	0	0	0	0	0	0	0
alkbh3	0	0	0.186	0	0	0.186	0	0	0	0	0	0	0
slc18a2	0	0	0.188	0	0	0.188	0	0	0	0	0	0	0
megf11	0	0	0.184	0	0	0.184	0	0	0	0	0	0	0
agbl3	0	0	0.181	0	0	0.181	0	0	0	0	0	0	0
gstk1	0	0	0.18	0	0	0.18	0	0	0	0	0	0	0
qdpr	0	0	0.189	0	0	0.189	0	0	0	0	0	0	0
gucy1b2	0	0	0.189	0	0	0.189	0	0	0	0	0	0	0
osgin1	0	0	0.195	0	0	0.195	0	0	0	0	0	0	0
slc18a1	0	0	0.197	0	0	0.197	0	0	0	0	0	0	0
rassf7	0	0	0.227	0	0	0.227	0	0	0	0	0	0	0
phox2a	0	0	0.195	0	0	0.195	0	0	0	0	0	0	0

znf24	0	0	0.194	0	0	0.194	0	0	0	0	0	0	0
dpf3	0	0	0.2	0	0	0.2	0	0	0	0	0	0	0
pnmt	0	0	0.365	0	0	0.365	0	0	0	0	0	0	0
fuom	0	0	0.345	0	0	0.345	0	0	0	0	0	0	0
lix1	0	0	0.35	0	0	0.35	0	0	0	0	0	0	0
hmox2	0	0	0.343	0	0	0.343	0	0	0	0	0	0	0
dmrta2	0	0	0.343	0	0	0.343	0	0	0	0	0	0	0
bcas3	0	0	0.338	0	0	0.338	0	0	0	0	0	0	0
bach1	0	0	0.34	0	0	0.34	0	0	0	0	0	0	0
znf782	0	0	0.351	0	0	0.351	0	0	0	0	0	0	0
blvrb	0	0	0.352	0	0	0.352	0	0	0	0	0	0	0
gchfr	0	0	0.412	0	0	0.412	0	0	0	0	0	0	0
fzd9	0	0	0.179	0	0	0.179	0	0	0	0	0	0	0
hmox1	0	0	0.405	0	0	0.405	0	0	0	0	0	0	0
th	0	0	0.403	0	0	0.403	0	0	0	0	0	0	0
spr	0	0	0.367	0	0	0.367	0	0	0	0	0	0	0
ddc	0	0	0.395	0	0	0.395	0	0	0	0	0	0	0
gch1	0	0	0.338	0	0	0.338	0	0	0	0	0	0	0
dbh	0	0	0.321	0	0	0.321	0	0	0	0	0	0	0
znf124	0	0	0.259	0	0	0.259	0	0	0	0	0	0	0
fgf20	0	0	0.265	0	0	0.265	0	0	0	0	0	0	0
kiaa2022	0	0	0.247	0	0	0.247	0	0	0	0	0	0	0
nr4a2	0	0	0.243	0	0	0.243	0	0	0	0	0	0	0
pitx3	0	0	0.241	0	0	0.241	0	0	0	0	0	0	0
sall3	0	0	0.243	0	0	0.243	0	0	0	0	0	0	0
ankrd37	0	0	0.265	0	0	0.265	0	0	0	0	0	0	0
slitrk1	0	0	0.266	0	0	0.266	0	0	0	0	0	0	0
trnf	0	0	0.306	0	0	0.306	0	0	0	0	0	0	0
slc25a28	0	0	0.312	0	0	0.312	0	0	0	0	0	0	0
pts	0	0	0.301	0	0	0.301	0	0	0	0	0	0	0
blvra	0	0	0.3	0	0	0.3	0	0	0	0	0	0	0
enox1	0	0	0.272	0	0	0.272	0	0	0	0	0	0	0
mrpl3	0	0	0.24	0	0	0.24	0	0	0	0	0	0	0
auts5	0	0	0.12	0	0	0.12	0	0	0	0	0	0	0
miox	0	0	0.11	0	0	0.11	0	0	0	0	0	0	0
naa20	0	0	0.113	0	0	0.113	0	0	0	0	0	0	0
loc642502	0	0	0.115	0	0	0.115	0	0	0	0	0	0	0
alad	0	0	0.115	0	0	0.115	0	0	0	0	0	0	0
pnma6a	0	0	0.11	0	0	0.11	0	0	0	0	0	0	0
ly6e	0	0	0.11	0	0	0.11	0	0	0	0	0	0	0
nipsnap3a	0	0	0.101	0	0	0.101	0	0	0	0	0	0	0

fh	0	0	0.109	0	0	0.109	0	0	0	0	0	0	0
hsr	0	0	0.11	0	0	0.11	0	0	0	0	0	0	0
hbm	0	0	0.117	0	0	0.117	0	0	0	0	0	0	0
bola1	0	0	0.118	0	0	0.118	0	0	0	0	0	0	0
kdm2a	0	0	0.12	0	0	0.12	0	0	0	0	0	0	0
slc35g1	0	0	0.12	0	0	0.12	0	0	0	0	0	0	0
cox2	0	0	0.121	0	0	0.121	0	0	0	0	0	0	0
col21a1	0	0	0.119	0	0	0.119	0	0	0	0	0	0	0
pter	0	0	0.119	0	0	0.119	0	0	0	0	0	0	0
tmem241	0	0	0.118	0	0	0.118	0	0	0	0	0	0	0
rpl35	0	0	0.118	0	0	0.118	0	0	0	0	0	0	0
pphln1	0	0	0.118	0	0	0.118	0	0	0	0	0	0	0
hres1	0	0	0.109	0	0	0.109	0	0	0	0	0	0	0
mir125a	0	0	0.101	0	0	0.101	0	0	0	0	0	0	0
tigd2	0	0	0.107	0	0	0.107	0	0	0	0	0	0	0
smr3a	0	0	0.105	0	0	0.105	0	0	0	0	0	0	0
tigd1	0	0	0.107	0	0	0.107	0	0	0	0	0	0	0
vangl2	0	0	0.105	0	0	0.105	0	0	0	0	0	0	0
sdhc	0	0	0.107	0	0	0.107	0	0	0	0	0	0	0
dyt21	0	0	0.106	0	0	0.106	0	0	0	0	0	0	0
ftmt	0	0	0.106	0	0	0.106	0	0	0	0	0	0	0
ech1	0	0	0.107	0	0	0.107	0	0	0	0	0	0	0
phyhd1	0	0	0.107	0	0	0.107	0	0	0	0	0	0	0
tp73-as1	0	0	0.108	0	0	0.108	0	0	0	0	0	0	0
sdhaf2	0	0	0.174	0	0	0.174	0	0	0	0	0	0	0
rab40c	0	0	0.102	0	0	0.102	0	0	0	0	0	0	0
ido1	0	0	0.102	0	0	0.102	0	0	0	0	0	0	0
znf229	0	0	0.109	0	0	0.109	0	0	0	0	0	0	0
akr1c1	0	0	0.102	0	0	0.102	0	0	0	0	0	0	0
irg1	0	0	0.102	0	0	0.102	0	0	0	0	0	0	0
abcb7	0	0	0.109	0	0	0.109	0	0	0	0	0	0	0
megf10	0	0	0.105	0	0	0.105	0	0	0	0	0	0	0
atad3b	0	0	0.104	0	0	0.104	0	0	0	0	0	0	0
lmx1a	0	0	0.122	0	0	0.122	0	0	0	0	0	0	0
frrs1l	0	0	0.117	0	0	0.117	0	0	0	0	0	0	0
tcfl5	0	0	0.122	0	0	0.122	0	0	0	0	0	0	0
hmgxb4	0	0	0.148	0	0	0.148	0	0	0	0	0	0	0
tmem25	0	0	0.148	0	0	0.148	0	0	0	0	0	0	0
il10rb	0	0	0.149	0	0	0.149	0	0	0	0	0	0	0
prune	0	0	0.146	0	0	0.146	0	0	0	0	0	0	0
c12orf45	0	0	0.146	0	0	0.146	0	0	0	0	0	0	0

iyd	0	0	0.14	0	0	0.14	0	0	0	0	0	0	0
tmem163	0	0	0.145	0	0	0.145	0	0	0	0	0	0	0
tyw5	0	0	0.145	0	0	0.145	0	0	0	0	0	0	0
slc25a30	0	0	0.153	0	0	0.153	0	0	0	0	0	0	0
lias	0	0	0.153	0	0	0.153	0	0	0	0	0	0	0
lap3	0	0	0.16	0	0	0.16	0	0	0	0	0	0	0
iddm4	0	0	0.162	0	0	0.162	0	0	0	0	0	0	0
dbh-as1	0	0	0.17	0	0	0.17	0	0	0	0	0	0	0
midn	0	0	0.171	0	0	0.171	0	0	0	0	0	0	0
clf	0	0	0.159	0	0	0.159	0	0	0	0	0	0	0
tet2	0	0	0.157	0	0	0.157	0	0	0	0	0	0	0
il19	0	0	0.154	0	0	0.154	0	0	0	0	0	0	0
srxn1	0	0	0.155	0	0	0.155	0	0	0	0	0	0	0
pcdha12	0	0	0.155	0	0	0.155	0	0	0	0	0	0	0
wbs2	0	0	0.14	0	0	0.14	0	0	0	0	0	0	0
dhcr24	0	0	0.147	0	0	0.147	0	0	0	0	0	0	0
cirh1a	0	0	0.127	0	0	0.127	0	0	0	0	0	0	0
kaza	0	0	0.127	0	0	0.127	0	0	0	0	0	0	0
il17rel	0	0	0.125	0	0	0.125	0	0	0	0	0	0	0
lhfp	0	0	0.134	0	0	0.134	0	0	0	0	0	0	0
txnrd1	0	0	0.133	0	0	0.133	0	0	0	0	0	0	0
tysnd1	0	0	0.128	0	0	0.128	0	0	0	0	0	0	0
tmx2	0	0	0.129	0	0	0.129	0	0	0	0	0	0	0
ascl4	0	0	0.131	0	0	0.131	0	0	0	0	0	0	0
cmtdi2	0	0	0.133	0	0	0.133	0	0	0	0	0	0	0
por	0	0	0.135	0	0	0.135	0	0	0	0	0	0	0
mocos	0	0	0.124	0	0	0.124	0	0	0	0	0	0	0
zcchc6	0	0	0.139	0	0	0.139	0	0	0	0	0	0	0
murc	0	0	0.124	0	0	0.124	0	0	0	0	0	0	0
tma7	0	0	0.139	0	0	0.139	0	0	0	0	0	0	0
il10	0	0	0.136	0	0	0.136	0	0	0	0	0	0	0
cxxc4	0	0	0.124	0	0	0.124	0	0	0	0	0	0	0
phox2b	0	0	0.135	0	0	0.135	0	0	0	0	0	0	0
pah	0	0	0.136	0	0	0.136	0	0	0	0	0	0	0
mknk2	0.1	0	0	0	0	0	0.1	0	0	0	0	0	0
gpr79	0.1	0	0	0	0	0	0.1	0	0	0	0	0	0
mrx20	0	0	0	0	0	0	0	0	0	0	0.125	0.125	0
nim1	0	0	0	0	0	0	0	0	0	0	0.126	0.126	0
taok1	0	0	0	0	0	0	0	0	0	0	0.127	0.125	0
mrx65	0	0	0	0	0	0	0	0	0	0	0.119	0.119	0
crnkl1	0	0	0	0	0	0	0	0	0	0	0.124	0.124	0

mcm4	0	0	0	0	0	0	0	0	0	0	0.12	0.118	0
lrguk	0	0	0	0	0	0	0	0	0	0	0.133	0.133	0
pacs1	0	0	0	0	0	0	0	0	0	0	0.14	0.139	0
strada	0	0	0	0	0	0	0	0	0	0	0.14	0.14	0
plcg1	0	0	0	0	0	0	0	0	0	0	0.144	0.143	0
morc2	0	0	0	0	0	0	0	0	0	0	0.146	0.146	0
tnk1	0	0	0	0	0	0	0	0	0	0	0.193	0.192	0
arhgef5	0	0	0	0	0	0	0	0	0	0	0.191	0.19	0
sowaha	0	0	0	0	0	0	0	0	0	0	0.135	0.134	0
sowahc	0	0	0	0	0	0	0	0	0	0	0.135	0.134	0
pgk1p1	0	0	0	0	0	0	0	0	0	0	0.13	0.13	0
mrx8	0	0	0	0	0	0	0	0	0	0	0.128	0.128	0
zmat3	0	0	0	0	0	0	0	0	0	0	0.132	0.131	0
ctdnep1	0	0	0	0	0	0	0	0	0	0	0.119	0.117	0
sowahd	0	0	0	0	0	0	0	0	0	0	0.135	0.134	0
nelfcd	0	0	0	0	0	0	0	0	0	0	0.128	0.126	0
rasgrf2	0	0	0	0	0	0	0	0	0	0	0.106	0.105	0
tanc1	0	0	0	0	0	0	0	0	0	0	0.103	0.103	0
ribc2	0	0	0	0	0	0	0	0	0	0	0.103	0.102	0
spock3	0	0	0	0	0	0	0	0	0	0	0.103	0.103	0
dclk3	0	0	0	0	0	0	0	0	0	0	0.104	0.103	0
chl1	0	0	0	0	0	0	0	0	0	0	0.104	0.103	0
mmp23b	0	0	0	0	0	0	0	0	0	0	0.104	0.104	0
mrx1	0	0	0	0	0	0	0	0	0	0	0.104	0.104	0
hist4h4	0	0	0	0	0	0	0	0	0	0	0.102	0.102	0
exoc3l1	0	0	0	0	0	0	0	0	0	0	0.102	0.102	0
limd1	0	0	0	0	0	0	0	0	0	0	0.101	0.1	0
ushbp1	0	0	0	0	0	0	0	0	0	0	0.101	0.101	0
gas7	0	0	0	0	0	0	0	0	0	0	0.101	0.101	0
napb	0	0	0	0	0	0	0	0	0	0	0.101	0.101	0
wipf3	0	0	0	0	0	0	0	0	0	0	0.102	0.101	0
igkv3or2-268	0	0	0	0	0	0	0	0	0	0	0.116	0.115	0
klhl10	0	0	0	0	0	0	0	0	0	0	0.105	0.104	0
tmem158	0	0	0	0	0	0	0	0	0	0	0.103	0.102	0
wash6p	0	0	0	0	0	0	0	0	0	0	0.11	0.11	0
mrx26	0	0	0	0	0	0	0	0	0	0	0.11	0.11	0
ppa2	0	0	0	0	0	0	0	0	0	0	0.109	0.108	0
pvrl1	0	0	0	0	0	0	0	0	0	0	0.105	0.105	0
map2k1	0	0	0	0	0	0	0	0	0	0	0.111	0.11	0
matk	0	0	0	0	0	0	0	0	0	0	0.115	0.113	0
map4k2	0	0	0	0	0	0	0	0	0	0	0.113	0.112	0

efhc2	0	0	0	0	0	0	0	0	0	0	0.108	0.108	0
uso1	0	0	0	0	0	0	0	0	0	0	0.111	0.109	0
eec2	0	0	0	0	0	0	0	0	0	0	0.106	0.106	0
map3k10	0	0	0	0	0	0	0	0	0	0	0.108	0.107	0
ninj2	0	0	0	0	0	0	0	0	0	0	0.105	0.105	0
itgbl1	0	0	0	0	0	0	0	0	0	0	0.108	0.107	0
chst9	0	0	0	0	0	0	0	0	0	0	0.107	0.106	0
stradb	0	0	0	0	0	0	0	0	0	0	0.108	0.107	0
iqub	0	0	0	0	0	0	0	0	0	0	0.108	0.108	0
edc4	0	0	0	0	0	0	0	0	0	0	0	0	0.109
ntsr1	0	0	0	0	0	0	0	0	0	0	0	0	0.107
tmeff1	0	0	0	0	0	0	0	0	0	0	0	0	0.105
ddx19a	0	0	0	0	0	0	0	0	0	0	0	0	0.109
fgd1	0	0	0	0	0	0	0	0	0	0	0	0	0.108
tm9sf3	0	0	0	0	0	0	0	0	0	0	0	0	0.106
pth2	0	0	0	0	0	0	0	0	0	0	0	0	0.107
rnu12-2p	0	0	0	0	0	0	0	0	0	0	0	0	0.108
tsks	0	0	0	0	0	0	0	0	0	0	0	0	0.11
rxfp3	0	0	0	0	0	0	0	0	0	0	0	0	0.109
piwil3	0	0	0	0	0	0	0	0	0	0	0	0	0.107
rest	0	0	0	0	0	0	0	0	0	0	0	0	0.108
mycbp2	0	0	0	0	0	0	0	0	0	0	0	0	0.108
upk2	0	0	0	0	0	0	0	0	0	0	0	0	0.107
apod	0	0	0	0	0	0	0	0	0	0	0	0	0.102
ier3	0	0	0	0	0	0	0	0	0	0	0	0	0.104
cd2	0	0	0	0	0	0	0	0	0	0	0	0	0.106
plagl1	0	0	0	0	0	0	0	0	0	0	0	0	0.108
abhd12	0	0	0	0	0	0	0	0	0	0	0	0	0.102
spats2	0	0	0	0	0	0	0	0	0	0	0	0	0.101
ube2l4	0	0	0	0	0	0	0	0	0	0	0	0	0.103
ulk2	0	0	0	0	0	0	0	0	0	0	0	0	0.109
slc5a7	0	0	0	0	0	0	0	0	0	0	0	0	0.105
ube3ap1	0	0	0	0	0	0	0	0	0	0	0	0	0.103
stmn1	0	0	0	0	0	0	0	0	0	0	0	0	0.112
hs3st4	0	0	0	0	0	0	0	0	0	0	0	0	0.104
ms4a6a	0	0	0	0	0	0	0	0	0	0	0	0	0.103
ube3ap2	0	0	0	0	0	0	0	0	0	0	0	0	0.103
vn1r7p	0	0	0	0	0	0	0	0	0	0	0	0	0.166
stac2	0	0	0	0	0	0	0	0	0	0	0	0	0.195
zap70	0	0	0	0	0	0	0	0	0	0	0	0	0.198
mvb12a	0	0	0	0	0	0	0	0	0	0	0	0	0.196

ubash3b	0	0	0	0	0	0	0	0	0	0	0	0	0.204
nts	0	0	0	0	0	0	0	0	0	0	0	0	0.202
rnf115	0	0	0	0	0	0	0	0	0	0	0	0	0.202
fam92a1	0	0	0	0	0	0	0	0	0	0	0	0	0.193
slc17a8	0	0	0	0	0	0	0	0	0	0	0	0	0.19
fpr2	0	0	0	0	0	0	0	0	0	0	0	0	0.187
spry3	0	0	0	0	0	0	0	0	0	0	0	0	0.188
nipsnap3b	0	0	0	0	0	0	0	0	0	0	0	0	0.187
znf287	0	0	0	0	0	0	0	0	0	0	0	0	0.191
hmga1p1	0	0	0	0	0	0	0	0	0	0	0	0	0.189
gal	0	0	0	0	0	0	0	0	0	0	0	0	0.203
trim3	0	0	0	0	0	0	0	0	0	0	0	0	0.11
fam19a5	0	0	0	0	0	0	0	0	0	0	0	0	0.212
mrgpre	0	0	0	0	0	0	0	0	0	0	0	0	0.214
cd2bp2	0	0	0	0	0	0	0	0	0	0	0	0	0.222
selt	0	0	0	0	0	0	0	0	0	0	0	0	0.215
ostf1	0	0	0	0	0	0	0	0	0	0	0	0	0.221
laptm4a	0	0	0	0	0	0	0	0	0	0	0	0	0.218
rit2	0	0	0	0	0	0	0	0	0	0	0	0	0.213
spred1	0	0	0	0	0	0	0	0	0	0	0	0	0.214
spred2	0	0	0	0	0	0	0	0	0	0	0	0	0.211
lgsn	0	0	0	0	0	0	0	0	0	0	0	0	0.204
opcml	0	0	0	0	0	0	0	0	0	0	0	0	0.208
spry4	0	0	0	0	0	0	0	0	0	0	0	0	0.211
lrrc20	0	0	0	0	0	0	0	0	0	0	0	0	0.208
arih2	0	0	0	0	0	0	0	0	0	0	0	0	0.185
sphkap	0	0	0	0	0	0	0	0	0	0	0	0	0.184
crcp	0	0	0	0	0	0	0	0	0	0	0	0	0.16
gpr151	0	0	0	0	0	0	0	0	0	0	0	0	0.159
rtp3	0	0	0	0	0	0	0	0	0	0	0	0	0.162
macrod2	0	0	0	0	0	0	0	0	0	0	0	0	0.161
trpm5	0	0	0	0	0	0	0	0	0	0	0	0	0.164
hgs	0	0	0	0	0	0	0	0	0	0	0	0	0.167
ppp2r2a	0	0	0	0	0	0	0	0	0	0	0	0	0.169
elfn2	0	0	0	0	0	0	0	0	0	0	0	0	0.162
mpp6	0	0	0	0	0	0	0	0	0	0	0	0	0.158
gas2l2	0	0	0	0	0	0	0	0	0	0	0	0	0.156
abracl	0	0	0	0	0	0	0	0	0	0	0	0	0.159
slmap	0	0	0	0	0	0	0	0	0	0	0	0	0.164
anks1b	0	0	0	0	0	0	0	0	0	0	0	0	0.161
ndufa5	0	0	0	0	0	0	0	0	0	0	0	0	0.167

p2rx3	0	0	0	0	0	0	0	0	0	0	0	0	0.167
nalcn	0	0	0	0	0	0	0	0	0	0	0	0	0.18
mcur1	0	0	0	0	0	0	0	0	0	0	0	0	0.178
phka2	0	0	0	0	0	0	0	0	0	0	0	0	0.182
avil	0	0	0	0	0	0	0	0	0	0	0	0	0.183
dnah7	0	0	0	0	0	0	0	0	0	0	0	0	0.184
il17d	0	0	0	0	0	0	0	0	0	0	0	0	0.179
spry1	0	0	0	0	0	0	0	0	0	0	0	0	0.18
lrrc2-as1	0	0	0	0	0	0	0	0	0	0	0	0	0.17
sh3glb2	0	0	0	0	0	0	0	0	0	0	0	0	0.17
flt1p1	0	0	0	0	0	0	0	0	0	0	0	0	0.17
fli1	0	0	0	0	0	0	0	0	0	0	0	0	0.172
scn11a	0	0	0	0	0	0	0	0	0	0	0	0	0.175
ccdc22	0	0	0	0	0	0	0	0	0	0	0	0	0.219
prickle1	0	0	0	0	0	0	0	0	0	0	0	0	0.221
mzb1	0	0	0	0	0	0	0	0	0	0	0	0	0.304
tacr3	0	0	0	0	0	0	0	0	0	0	0	0	0.301
gldcp1	0	0	0	0	0	0	0	0	0	0	0	0	0.307
tcn1	0	0	0	0	0	0	0	0	0	0	0	0	0.32
tac4	0	0	0	0	0	0	0	0	0	0	0	0	0.328
tac3	0	0	0	0	0	0	0	0	0	0	0	0	0.322
gapvd1	0	0	0	0	0	0	0	0	0	0	0	0	0.303
fra10ac1	0	0	0	0	0	0	0	0	0	0	0	0	0.301
tacr2	0	0	0	0	0	0	0	0	0	0	0	0	0.29
psmd6	0	0	0	0	0	0	0	0	0	0	0	0	0.293
tacr1	0	0	0	0	0	0	0	0	0	0	0	0	0.292
cubn	0	0	0	0	0	0	0	0	0	0	0	0	0.301
fra10a	0	0	0	0	0	0	0	0	0	0	0	0	0.301
adcyap1r1	0	0	0	0	0	0	0	0	0	0	0	0	0.334
dfnb14	0	0	0	0	0	0	0	0	0	0	0	0	0.339
tac1	0	0	0	0	0	0	0	0	0	0	0	0	0.385
rnf126	0	0	0	0	0	0	0	0	0	0	0	0	0.388
ubash3a	0	0	0	0	0	0	0	0	0	0	0	0	0.413
jbs	0	0	0	0	0	0	0	0	0	0	0	0	0.432
dcaf4	0	0	0	0	0	0	0	0	0	0	0	0	0.504
mpi	0	0	0	0	0	0	0	0	0	0	0	0	0.437
adnp	0	0	0	0	0	0	0	0	0	0	0	0	0.385
vip	0	0	0	0	0	0	0	0	0	0	0	0	0.384
stt3a	0	0	0	0	0	0	0	0	0	0	0	0	0.346
adcyap1	0	0	0	0	0	0	0	0	0	0	0	0	0.339
vipr2	0	0	0	0	0	0	0	0	0	0	0	0	0.342

vipr1	0	0	0	0	0	0	0	0	0	0	0	0	0.347
bcas2	0	0	0	0	0	0	0	0	0	0	0	0	0.363
ccdc93	0	0	0	0	0	0	0	0	0	0	0	0	0.288
eri2	0	0	0	0	0	0	0	0	0	0	0	0	0.286
pirt	0	0	0	0	0	0	0	0	0	0	0	0	0.239
sidt2	0	0	0	0	0	0	0	0	0	0	0	0	0.241
mir519b	0	0	0	0	0	0	0	0	0	0	0	0	0.24
c12orf39	0	0	0	0	0	0	0	0	0	0	0	0	0.241
mep1ap2	0	0	0	0	0	0	0	0	0	0	0	0	0.244
c9orf89	0	0	0	0	0	0	0	0	0	0	0	0	0.242
pelo	0	0	0	0	0	0	0	0	0	0	0	0	0.237
c8orf17	0	0	0	0	0	0	0	0	0	0	0	0	0.239
mir500a	0	0	0	0	0	0	0	0	0	0	0	0	0.22
mrgprd	0	0	0	0	0	0	0	0	0	0	0	0	0.219
scamp3	0	0	0	0	0	0	0	0	0	0	0	0	0.229
anp32f	0	0	0	0	0	0	0	0	0	0	0	0	0.228
trpv3	0	0	0	0	0	0	0	0	0	0	0	0	0.228
mep1ap1	0	0	0	0	0	0	0	0	0	0	0	0	0.244
nkx1-1	0	0	0	0	0	0	0	0	0	0	0	0	0.244
trpv1	0	0	0	0	0	0	0	0	0	0	0	0	0.263
tff2	0	0	0	0	0	0	0	0	0	0	0	0	0.258
cpamd8	0	0	0	0	0	0	0	0	0	0	0	0	0.271
trpa1	0	0	0	0	0	0	0	0	0	0	0	0	0.272
trpm8	0	0	0	0	0	0	0	0	0	0	0	0	0.275
clybl	0	0	0	0	0	0	0	0	0	0	0	0	0.261
rps13	0	0	0	0	0	0	0	0	0	0	0	0	0.254
gmds	0	0	0	0	0	0	0	0	0	0	0	0	0.244
adnp2	0	0	0	0	0	0	0	0	0	0	0	0	0.245
mrgprx1	0	0	0	0	0	0	0	0	0	0	0	0	0.245
trpv2	0	0	0	0	0	0	0	0	0	0	0	0	0.246
asic3	0	0	0	0	0	0	0	0	0	0	0	0	0.251
pigm	0	0	0	0	0	0	0	0	0	0	0	0	0.156
scn10a	0	0	0	0	0	0	0	0	0	0	0	0	0.203
slc17a7	0	0	0	0	0	0	0	0	0	0	0	0	0.118
prepl	0	0	0	0	0	0	0	0	0	0	0	0	0.12
hhipl2	0	0	0	0	0	0	0	0	0	0	0	0	0.118
cd38	0	0	0	0	0	0	0	0	0	0	0	0	0.122
pamr1	0	0	0	0	0	0	0	0	0	0	0	0	0.119
mnf1	0	0	0	0	0	0	0	0	0	0	0	0	0.118
akap17a	0	0	0	0	0	0	0	0	0	0	0	0	0.119
pde9a	0	0	0	0	0	0	0	0	0	0	0	0	0.118

arih1	0	0	0	0	0	0	0	0	0	0	0	0	0.114
gpr65	0	0	0	0	0	0	0	0	0	0	0	0	0.116
sh3bp2	0	0	0	0	0	0	0	0	0	0	0	0	0.123
rin2	0	0	0	0	0	0	0	0	0	0	0	0	0.118
dus2	0	0	0	0	0	0	0	0	0	0	0	0	0.117
prep	0	0	0	0	0	0	0	0	0	0	0	0	0.121
mvb12b	0	0	0	0	0	0	0	0	0	0	0	0	0.122
wdr27	0	0	0	0	0	0	0	0	0	0	0	0	0.126
uchl1	0	0	0	0	0	0	0	0	0	0	0	0	0.123
htr3a	0	0	0	0	0	0	0	0	0	0	0	0	0.123
artn	0	0	0	0	0	0	0	0	0	0	0	0	0.125
cnot4	0	0	0	0	0	0	0	0	0	0	0	0	0.125
ncapd3	0	0	0	0	0	0	0	0	0	0	0	0	0.129
morn2	0	0	0	0	0	0	0	0	0	0	0	0	0.127
reep6	0	0	0	0	0	0	0	0	0	0	0	0	0.121
vstm2l	0	0	0	0	0	0	0	0	0	0	0	0	0.125
mrgprf	0	0	0	0	0	0	0	0	0	0	0	0	0.121
nell2	0	0	0	0	0	0	0	0	0	0	0	0	0.123
mycbpap	0	0	0	0	0	0	0	0	0	0	0	0	0.124
pnoc	0	0	0	0	0	0	0	0	0	0	0	0	0.121
mme	0	0	0	0	0	0	0	0	0	0	0	0	0.114
nmur1	0	0	0	0	0	0	0	0	0	0	0	0	0.115
slc50a1	0	0	0	0	0	0	0	0	0	0	0	0	0.11
synm	0	0	0	0	0	0	0	0	0	0	0	0	0.112
fignl2	0	0	0	0	0	0	0	0	0	0	0	0	0.11
ms4a7	0	0	0	0	0	0	0	0	0	0	0	0	0.116
sdf2l1	0	0	0	0	0	0	0	0	0	0	0	0	0.112
opn4	0	0	0	0	0	0	0	0	0	0	0	0	0.11
p2ry11	0	0	0	0	0	0	0	0	0	0	0	0	0.11
hcn3	0	0	0	0	0	0	0	0	0	0	0	0	0.109
anp32c	0	0	0	0	0	0	0	0	0	0	0	0	0.11
cmt2g	0	0	0	0	0	0	0	0	0	0	0	0	0.107
ywhaq	0	0	0	0	0	0	0	0	0	0	0	0	0.116
ms4a4e	0	0	0	0	0	0	0	0	0	0	0	0	0.109
kctd12	0	0	0	0	0	0	0	0	0	0	0	0	0.112
mea1	0	0	0	0	0	0	0	0	0	0	0	0	0.156
lrch3	0	0	0	0	0	0	0	0	0	0	0	0	0.114
htr4	0	0	0	0	0	0	0	0	0	0	0	0	0.113
or1e3	0	0	0	0	0	0	0	0	0	0	0	0	0.113
brd7p3	0	0	0	0	0	0	0	0	0	0	0	0	0.115
atg4c	0	0	0	0	0	0	0	0	0	0	0	0	0.113

hck	0	0	0	0	0	0	0	0	0	0	0	0	0.121
nmbr	0	0	0	0	0	0	0	0	0	0	0	0	0.113
ube2l3	0	0	0	0	0	0	0	0	0	0	0	0	0.113
hdhd1p1	0	0	0	0	0	0	0	0	0	0	0	0	0.112
hdhd1p2	0	0	0	0	0	0	0	0	0	0	0	0	0.112
cap2	0	0	0	0	0	0	0	0	0	0	0	0	0.113
htr3c	0	0	0	0	0	0	0	0	0	0	0	0	0.111
calca	0	0	0	0	0	0	0	0	0	0	0	0	0.111
ntsr2	0	0	0	0	0	0	0	0	0	0	0	0	0.127
mehmo	0	0	0	0	0	0	0	0	0	0	0	0	0.111
slc17a6	0	0	0	0	0	0	0	0	0	0	0	0	0.146
cpne2	0	0	0	0	0	0	0	0	0	0	0	0	0.146
ncapg2	0	0	0	0	0	0	0	0	0	0	0	0	0.15
psmd1	0	0	0	0	0	0	0	0	0	0	0	0	0.151
rbmxp1	0	0	0	0	0	0	0	0	0	0	0	0	0.149
grp	0	0	0	0	0	0	0	0	0	0	0	0	0.149
mageb4	0	0	0	0	0	0	0	0	0	0	0	0	0.146
ube2g1	0	0	0	0	0	0	0	0	0	0	0	0	0.147
setd8p1	0	0	0	0	0	0	0	0	0	0	0	0	0.142
pclo	0	0	0	0	0	0	0	0	0	0	0	0	0.142
npff	0	0	0	0	0	0	0	0	0	0	0	0	0.141
asxl1	0	0	0	0	0	0	0	0	0	0	0	0	0.144
setbp1	0	0	0	0	0	0	0	0	0	0	0	0	0.149
dgcr14	0	0	0	0	0	0	0	0	0	0	0	0	0.148
psd3	0	0	0	0	0	0	0	0	0	0	0	0	0.152
galr2	0	0	0	0	0	0	0	0	0	0	0	0	0.154
anp32d	0	0	0	0	0	0	0	0	0	0	0	0	0.157
tcn2	0	0	0	0	0	0	0	0	0	0	0	0	0.154
smu1	0	0	0	0	0	0	0	0	0	0	0	0	0.158
wdr19	0	0	0	0	0	0	0	0	0	0	0	0	0.128
upk1a	0	0	0	0	0	0	0	0	0	0	0	0	0.154
c14orf23	0	0	0	0	0	0	0	0	0	0	0	0	0.154
galr1	0	0	0	0	0	0	0	0	0	0	0	0	0.152
or51e2	0	0	0	0	0	0	0	0	0	0	0	0	0.152
scn9a	0	0	0	0	0	0	0	0	0	0	0	0	0.152
c4orf19	0	0	0	0	0	0	0	0	0	0	0	0	0.154
cuedc1	0	0	0	0	0	0	0	0	0	0	0	0	0.154
dgcr7	0	0	0	0	0	0	0	0	0	0	0	0	0.139
ints4	0	0	0	0	0	0	0	0	0	0	0	0	0.152
ecel1	0	0	0	0	0	0	0	0	0	0	0	0	0.13
or7e21p	0	0	0	0	0	0	0	0	0	0	0	0	0.129

gsc2	0	0	0	0	0	0	0	0	0	0	0	0	0.13
capzb	0	0	0	0	0	0	0	0	0	0	0	0	0.135
kif13b	0	0	0	0	0	0	0	0	0	0	0	0	0.135
chmp7	0	0	0	0	0	0	0	0	0	0	0	0	0.134
il31	0	0	0	0	0	0	0	0	0	0	0	0	0.128
sdcbp2	0	0	0	0	0	0	0	0	0	0	0	0	0.128
sct	0	0	0	0	0	0	0	0	0	0	0	0	0.139
myof	0	0	0	0	0	0	0	0	0	0	0	0	0.127
nkx6-3	0	0	0	0	0	0	0	0	0	0	0	0	0.125
grpr	0	0	0	0	0	0	0	0	0	0	0	0	0.128
mageb3	0	0	0	0	0	0	0	0	0	0	0	0	0.127
htr3d	0	0	0	0	0	0	0	0	0	0	0	0	0.132
cacna1h	0	0	0	0	0	0	0	0	0	0	0	0	0.128
slc18a3	0	0	0	0	0	0	0	0	0	0	0	0	0.137
echs1	0	0	0	0	0	0	0	0	0	0	0	0	0.139
pxt1	0	0	0	0	0	0	0	0	0	0	0	0	0.137
bnc1	0	0	0	0	0	0	0	0	0	0	0	0	0.14
tspan11	0	0	0	0	0	0	0	0	0	0	0	0	0.141
foxs1	0	0	0	0	0	0	0	0	0	0	0	0	0.134
slc35b1	0	0	0	0	0	0	0	0	0	0	0	0	0.137
hla-x	0	0	0	0	0	0	0	0	0	0	0	0	0.139
tas2r43	0	0	0	0	0	0	0	0	0	0	0	0	0.135
jakmip3	0	0	0	0	0	0	0	0	0	0	0	0	0.137
tlx2	0	0	0	0	0	0	0	0	0	0	0	0	0.134
galr3	0	0	0	0	0	0	0	0	0	0	0	0	0.138
