# Supplementary material for: The RXFP3 receptor is functionally associated with cellular responses to oxidative stress and DNA damage
Source: Aging (Albany NY). 2019 Dec 3;11(23):11268–313. doi: 10.18632/aging.102528 (PMC6932917; doi:10.18632/aging.102528)
Supplement: Supplementary Table 9 [file aging-11-102528-s004..pdf]

Table S9. Concatenated GIT2 signaling theoretical data set. Biomedical text word items, *i.e.* Gene Symbols, associated with both GIT2 and Cellular Signaling extraction terms are represented. Hence the concatenation of GIT2 with Cell Signaling protein sets results in the theoretical 'GIT2-Signaling' dataset. All proteins identified possess a Cosine Similarity score of association with the interrogator term of >0.1.

Gene Symbol	GIT2	GIT-2	CAT-2	CAT2	Cat2	Cat-2	Git2	Git-2	ARF GAP GIT2	CAT2	Cool-interacting tyrosine phosphorylated protein 2	Cool-interacting protein 2
ajuba	0.489	0	0	0	0	0	0.489	0	0.483	0	0.626	0.625
abi3	0.487	0	0	0	0	0	0.487	0	0.482	0	0.464	0.462
pak6	0.385	0	0	0	0	0	0.385	0	0.38	0	0.549	0.549
arhgap8	0.412	0	0	0	0	0	0.412	0	0.411	0	0.452	0.451
map4k4	0.37	0	0	0	0	0	0.37	0	0.363	0	0.498	0.497
tgfb1i1	0.412	0	0	0	0	0	0.412	0	0.409	0	0.377	0.376
sorbs1	0.309	0	0	0	0	0	0.309	0	0.309	0	0.265	0.263
asap1	0.335	0	0	0	0	0	0.335	0	0.337	0	0.271	0.269
ppfia1	0.346	0	0	0	0	0	0.346	0	0.342	0	0.419	0.418
dnajc27	0.328	0	0	0	0	0	0.328	0	0.325	0	0.377	0.375
capn5	0.297	0	0	0	0	0	0.297	0	0.293	0	0.397	0.397
nudt16l1	0.242	0	0	0.139	0.139	0	0.242	0	0.236	0.139	0.253	0.252
slk	0.289	0	0	0	0	0	0.289	0	0.288	0	0.375	0.372
crkl	0.244	0	0	0	0	0	0.244	0	0.24	0	0.171	0.169
rapgef1	0.243	0	0	0	0	0	0.243	0	0.241	0	0.15	0.148
cyth4	0.327	0	0	0	0	0	0.327	0	0.328	0	0.302	0.3
rsf1	0.248	0	0	0	0	0	0.248	0	0.25	0	0.371	0.37
c17orf62	0.252	0	0	0	0	0	0.252	0	0.252	0	0.346	0.345
bcar3	0.317	0	0	0	0	0	0.317	0	0.311	0	0.187	0.185
ipcef1	0.306	0	0	0	0	0	0.306	0	0.308	0	0.252	0.25
map4k1	0.26	0	0	0	0	0	0.26	0	0.262	0	0.236	0.234
sorbs3	0.263	0	0	0	0	0	0.263	0	0.271	0	0.198	0.195
larp4	0.221	0	0	0	0	0	0.221	0	0.219	0	0.328	0.328
arhgap42	0.257	0	0	0	0	0	0.257	0	0.264	0	0.283	0.282
arf1	0.334	0	0	0	0	0	0.334	0	0.351	0	0.178	0.177
clns1ap1	0.198	0	0	0	0	0	0.198	0	0.197	0	0.327	0.326
cyth3	0.262	0	0	0	0	0	0.262	0	0.266	0	0.198	0.197
ephb6	0.173	0	0	0	0	0	0.173	0	0.175	0	0.101	0.1
arpc5	0.266	0	0	0	0	0	0.266	0	0.264	0	0.226	0.224
nedd9	0.26	0	0	0	0	0	0.26	0	0.258	0	0.144	0.141
wash1	0.285	0	0	0	0	0	0.285	0	0.284	0	0.204	0.203
lpxn	0.278	0	0	0	0	0	0.278	0	0.274	0	0.197	0.195
copg1	0.249	0	0	0	0	0	0.249	0	0.252	0	0.236	0.235
svil	0.182	0	0	0.136	0.136	0	0.182	0	0.181	0.136	0.164	0.162

fam21c	0.255	0	0	0	0	0	0.255	0	0.253	0	0.187	0.186
abi3bp	0.227	0	0	0	0	0	0.227	0	0.235	0	0.236	0.235
cpsf6	0.168	0	0	0.111	0.111	0	0.168	0	0.169	0.111	0.199	0.198
rac3	0.499	0	0	0	0	0	0.499	0	0.494	0	0	0
rab11fip3	0.258	0	0	0	0	0	0.258	0	0.255	0	0.209	0.208
cyth2	0.291	0	0	0	0	0	0.291	0	0.294	0	0.178	0.176
arhgef26	0.283	0	0	0	0	0	0.283	0	0.29	0	0.133	0.132
sla	0.168	0	0	0	0	0	0.168	0	0.168	0	0	0
pwp1	0.183	0	0	0	0	0	0.183	0	0.18	0	0.17	0.168
rltpr	0.303	0	0	0	0	0	0.303	0	0.308	0	0.122	0.121
arf3	0.193	0	0	0.107	0.107	0	0.193	0	0.203	0.107	0.121	0.12
dennd1c	0.285	0	0	0	0	0	0.285	0	0.283	0	0.143	0.142
abi2	0.238	0	0	0	0	0	0.238	0	0.237	0	0.177	0.176
arap3	0.213	0	0	0	0	0	0.213	0	0.217	0	0.124	0.122
mtss1	0.265	0	0	0	0	0	0.265	0	0.268	0	0.16	0.159
cyp20a1	0	0	0.267	0.263	0.263	0.267	0	0	0	0.263	0	0
map4k5	0.191	0	0	0	0	0	0.191	0	0.188	0	0.139	0.138
rasl11a	0.212	0	0	0	0	0	0.212	0	0.212	0	0.19	0.189
c16orf70	0.155	0	0	0	0	0	0.155	0	0.158	0	0.121	0.119
snx21	0.261	0	0	0	0	0	0.261	0	0.262	0	0.127	0.125
spry2	0.128	0	0	0	0	0	0.128	0	0.127	0	0	0
sh2d3c	0.231	0	0	0	0	0	0.231	0	0.228	0	0.13	0.128
bin3	0.305	0	0	0	0	0	0.305	0	0.307	0	0.114	0.113
rap1gap	0.248	0	0	0	0	0	0.248	0	0.251	0	0.113	0.112
dok1	0.216	0	0	0	0	0	0.216	0	0.219	0	0.168	0.164
txnl4a	0	0	0.201	0.279	0.279	0.201	0	0	0	0.279	0	0
marcks	0.21	0.21	0	0.133	0.133	0	0.21	0.21	0	0.133	0	0
enah	0.249	0	0	0	0	0	0.249	0	0.26	0	0.135	0.133
ct45a3	0	0	0.222	0.264	0.264	0.222	0	0	0	0.264	0	0
ct45a4	0	0	0.222	0.264	0.264	0.222	0	0	0	0.264	0	0
ct45a5	0	0	0.222	0.264	0.264	0.222	0	0	0	0.264	0	0
ct45a6	0	0	0.222	0.264	0.264	0.222	0	0	0	0.264	0	0
ct45b1p	0	0	0.222	0.264	0.264	0.222	0	0	0	0.264	0	0
ct60	0	0	0.222	0.264	0.264	0.222	0	0	0	0.264	0	0
ct62	0	0	0.222	0.264	0.264	0.222	0	0	0	0.264	0	0
ct64	0	0	0.222	0.264	0.264	0.222	0	0	0	0.264	0	0
psmc4	0	0	0.216	0.268	0.268	0.216	0	0	0	0.268	0	0
exoc6b	0.215	0	0	0.117	0.117	0	0.215	0	0.221	0.117	0.116	0.115
agap1	0.228	0	0	0	0	0	0.228	0	0.237	0	0.138	0.137
vav3	0.299	0	0	0	0	0	0.299	0	0.3	0	0.115	0.114
ctbp1	0.154	0	0	0	0	0	0.154	0	0.156	0	0.223	0.222

hmgb1	0.164	0	0	0	0	0	0.164	0	0.162	0	0.222	0.222
tnik	0.16	0	0	0	0	0	0.16	0	0.156	0	0.192	0.191
snx33	0.17	0	0	0	0	0	0.17	0	0.171	0	0.191	0.189
tceal3	0.164	0	0	0	0	0	0.164	0	0.168	0	0.144	0.143
sh3pxd2b	0.175	0	0	0	0	0	0.175	0	0.175	0	0.162	0.16
raver1	0.209	0	0	0	0	0	0.209	0	0.221	0	0.153	0.152
zyx	0.238	0	0	0	0	0	0.238	0	0.24	0	0.119	0.118
stat5b	0.134	0	0	0	0	0	0.134	0	0.133	0	0.207	0.205
pdcd10	0.182	0	0	0	0	0	0.182	0	0.19	0	0.185	0.184
eif1ax	0.185	0	0	0	0	0	0.185	0	0.183	0	0.192	0.191
rap2a	0.186	0	0	0	0	0	0.186	0	0.19	0	0.136	0.135
igfn1	0.176	0	0	0	0	0	0.176	0	0.173	0	0.109	0.108
swap70	0.204	0	0	0	0	0	0.204	0	0.207	0	0.142	0.14
pikfyve	0.176	0	0	0.121	0.121	0	0.176	0	0.182	0.121	0	0
eea1	0.127	0	0	0.147	0.147	0	0.127	0	0.132	0.147	0	0
myo10	0.206	0	0	0	0	0	0.206	0	0.213	0	0.118	0.117
tbc1d3f	0.194	0	0	0	0	0	0.194	0	0.197	0	0.116	0.115
flnc	0.169	0	0	0	0	0	0.169	0	0.17	0	0.158	0.157
ap1s1	0.166	0	0	0	0	0	0.166	0	0.168	0	0.165	0.164
ass1	0	0	0.207	0.215	0.215	0.207	0	0	0	0.215	0	0
nf2	0.23	0	0	0	0	0	0.23	0	0.24	0	0.18	0.179
sgsm1	0.145	0	0.164	0	0	0.164	0.145	0	0.155	0	0	0
dimt1	0.157	0	0	0	0	0	0.157	0	0.165	0	0.131	0.128
arfgap3	0.181	0	0	0	0	0	0.181	0	0.186	0	0.116	0.115
stk4	0.166	0	0	0	0	0	0.166	0	0.165	0	0.167	0.165
heatr6	0.142	0	0	0	0	0	0.142	0	0.144	0	0.192	0.191
marc2	0	0	0.182	0.222	0.222	0.182	0	0	0	0.222	0	0
plek	0.201	0	0	0.108	0.108	0	0.201	0	0.201	0.108	0.102	0
cygb	0	0	0.193	0.145	0.145	0.193	0	0	0	0.145	0	0
ccdc88b	0.132	0	0	0	0	0	0.132	0	0.14	0	0.167	0.166
eps8	0.171	0	0	0	0	0	0.171	0	0.176	0	0.126	0.124
odc1	0	0	0.153	0.233	0.233	0.153	0	0	0	0.233	0	0
ppfia3	0.157	0	0	0	0	0	0.157	0	0.158	0	0.153	0.152
ptma	0.187	0	0	0	0	0	0.187	0	0.192	0	0.11	0.11
ocrl	0.121	0	0	0.114	0.114	0	0.121	0	0.129	0.114	0	0
shd	0.159	0	0	0	0	0	0.159	0	0.164	0	0.131	0.129
she	0.159	0	0	0	0	0	0.159	0	0.164	0	0.131	0.129
vnn3	0	0	0.14	0.231	0.231	0.14	0	0	0	0.231	0	0
arf4	0.16	0	0	0	0	0	0.16	0	0.165	0	0.122	0.122
rgs3	0.245	0	0	0	0	0	0.245	0	0.254	0	0	0
htra3	0.117	0	0	0	0	0	0.117	0	0.119	0	0.187	0.186

dennd1b	0.171	0	0	0	0	0	0.171	0	0.175	0	0.116	0.115
gfm1	0	0	0.137	0.225	0.225	0.137	0	0	0	0.225	0	0
asl	0	0	0.129	0.23	0.23	0.129	0	0	0	0.23	0	0
gbf1	0.239	0	0	0	0	0	0.239	0	0.247	0	0.108	0.107
aldh16a1	0.104	0	0	0	0	0	0.104	0	0.112	0	0	0
iscu	0	0	0.14	0.219	0.219	0.14	0	0	0	0.219	0	0
tm7sf3	0	0	0.153	0.209	0.209	0.153	0	0	0	0.209	0	0
asb6	0.118	0	0	0	0	0	0.118	0	0.122	0	0	0
gcc2	0.141	0	0	0	0	0	0.141	0	0.149	0	0.104	0.103
bcar4	0.146	0	0	0	0	0	0.146	0	0.141	0	0.116	0.115
sctr	0	0	0	0.182	0.182	0	0	0	0	0.182	0	0
slc43a1	0	0	0.123	0.221	0.221	0.123	0	0	0	0.221	0	0
snx9	0.128	0	0	0	0	0	0.128	0	0.131	0	0.149	0.147
prap1	0	0	0	0.3	0.3	0	0	0	0	0.3	0	0
dbn1	0.173	0	0	0	0	0	0.173	0	0.2	0	0.123	0.122
bbip1	0.135	0	0	0	0	0	0.135	0	0.151	0	0.106	0.105
cyth1	0.219	0	0	0	0	0	0.219	0	0.22	0	0.116	0.115
camta1	0	0	0.203	0.16	0.16	0.203	0	0	0	0.16	0	0
rhobtb3	0.216	0	0	0	0	0	0.216	0	0.22	0	0.117	0.116
cttnbp2	0.186	0	0	0	0	0	0.186	0	0.187	0	0.162	0.162
phpt1	0.125	0	0	0	0	0	0.125	0	0.127	0	0.12	0.118
cps1	0	0	0.154	0.191	0.191	0.154	0	0	0	0.191	0	0
zfhx4	0	0	0.126	0.209	0.209	0.126	0	0	0	0.209	0	0
tspan13	0.292	0	0	0	0	0	0.292	0	0.292	0	0	0
rab9b	0.14	0	0	0	0	0	0.14	0	0.145	0	0.113	0.111
arl2bp	0.212	0	0	0	0	0	0.212	0	0.232	0	0.105	0.103
fcho2	0.183	0	0	0	0	0	0.183	0	0.192	0	0.153	0.152
arfrp1	0.137	0	0	0	0	0	0.137	0	0.142	0	0.116	0.116
gls	0	0	0.189	0.16	0.16	0.189	0	0	0	0.16	0	0
xdh	0	0	0.174	0.17	0.17	0.174	0	0	0	0.17	0	0
slc30a7	0	0	0.133	0.196	0.196	0.133	0	0	0	0.196	0	0
ppfia4	0.174	0	0	0	0	0	0.174	0	0.176	0	0.163	0.162
klf6	0	0	0.163	0.174	0.174	0.163	0	0	0	0.174	0	0
tuba4a	0.17	0	0	0.11	0.11	0	0.17	0	0.173	0.11	0	0
mir661	0	0	0.156	0.177	0.177	0.156	0	0	0	0.177	0	0
dnm2	0.195	0	0	0	0	0	0.195	0	0.201	0	0	0
usp6nl	0.201	0	0	0	0	0	0.201	0	0.201	0	0.117	0.115
mir132	0.148	0	0	0	0	0	0.148	0	0.145	0	0.144	0.143
tbc1d25	0.126	0	0	0.112	0.112	0	0.126	0	0.137	0.112	0	0
ehf	0	0	0.149	0.174	0.174	0.149	0	0	0	0.174	0	0
exoc5	0.197	0	0	0	0	0	0.197	0	0.2	0	0.113	0.112

arfgap1	0.186	0	0	0	0	0	0.186	0	0.202	0	0.119	0.118
sh3gl2	0.119	0	0	0	0	0	0.119	0	0.131	0	0	0
me2	0	0	0.178	0.15	0.15	0.178	0	0	0	0.15	0	0
tbc1d14	0.126	0	0	0.139	0.139	0	0.126	0	0.136	0.139	0	0
marc1	0	0	0.147	0.169	0.169	0.147	0	0	0	0.169	0	0
lrrc16b	0.139	0	0	0.124	0.124	0	0.139	0	0.143	0.124	0	0
sergef	0.159	0	0	0.104	0.104	0	0.159	0	0.162	0.104	0	0
slc5a6	0	0	0	0.178	0.178	0	0	0	0	0.178	0	0
esx1	0	0	0.124	0.178	0.178	0.124	0	0	0	0.178	0	0
clip1	0.144	0	0	0	0	0	0.144	0	0.147	0	0.123	0.121
dhrs4l2	0	0	0.135	0.167	0.167	0.135	0	0	0	0.167	0	0
pc	0	0	0.133	0.168	0.168	0.133	0	0	0	0.168	0	0
luzp4	0	0	0.175	0.14	0.14	0.175	0	0	0	0.14	0	0
tsnax	0	0	0.127	0.172	0.172	0.127	0	0	0	0.172	0	0
gfm2	0	0	0.125	0.173	0.173	0.125	0	0	0	0.173	0	0
wash2p	0.17	0	0	0	0	0	0.17	0	0.173	0	0.127	0.126
trip6	0.162	0	0	0	0	0	0.162	0	0.164	0	0	0
ccdc53	0.106	0	0	0	0	0	0.106	0	0.107	0	0.104	0.102
fyco1	0	0	0	0.174	0.174	0	0	0	0	0.174	0	0
slc3a2	0	0	0.104	0.184	0.184	0.104	0	0	0	0.184	0	0
ctns	0	0	0.109	0.179	0.179	0.109	0	0	0	0.179	0	0
cdkl2	0	0	0.105	0.107	0.107	0.105	0	0	0	0.107	0	0
dgkb	0.126	0	0	0.118	0.118	0	0.126	0	0.141	0.118	0	0
elk3	0	0	0.161	0.141	0.141	0.161	0	0	0	0.141	0	0
rab21	0.174	0	0	0	0	0	0.174	0	0.179	0	0	0
magt1	0.102	0	0	0.142	0.142	0	0.102	0	0.103	0.142	0	0
exoc8	0.171	0	0	0	0	0	0.171	0	0.173	0	0.109	0.108
arhgdib	0.243	0	0	0	0	0	0.243	0	0.245	0	0	0
rnd1	0.241	0	0	0	0	0	0.241	0	0.244	0	0	0
bcas2	0	0	0	0	0	0	0	0	0	0	0	0
myo1a	0.105	0	0	0.133	0.133	0	0.105	0	0.113	0.133	0	0
slc44a1	0	0	0	0.169	0.169	0	0	0	0	0.169	0	0
arfgef2	0.164	0	0	0	0	0	0.164	0	0.185	0	0.103	0.102
adcy7	0	0	0	0.237	0.237	0	0	0	0	0.237	0	0
uqcrq	0	0	0.128	0.151	0.151	0.128	0	0	0	0.151	0	0
rab1b	0.234	0	0	0	0	0	0.234	0	0.238	0	0	0
dusp11	0	0	0.127	0.15	0.15	0.127	0	0	0	0.15	0	0
robo4	0.144	0	0	0	0	0	0.144	0	0.143	0	0.135	0.134
capg	0.151	0	0	0	0	0	0.151	0	0.158	0	0.119	0.118
tmem141	0	0	0	0.231	0.231	0	0	0	0	0.231	0	0
usp34	0	0	0.123	0.149	0.149	0.123	0	0	0	0.149	0	0

fblim1	0.147	0	0	0	0	0	0.147	0	0.146	0	0.127	0.125
raph1	0.228	0	0	0	0	0	0.228	0	0.234	0	0	0
trpm6	0	0	0	0.23	0.23	0	0	0	0	0.23	0	0
gcc1	0.112	0	0	0	0	0	0.112	0	0.116	0	0	0
dgkz	0.223	0	0	0	0	0	0.223	0	0.236	0	0	0
tial1	0	0	0.103	0.158	0.158	0.103	0	0	0	0.158	0	0
etf1p2	0	0	0	0.126	0.126	0	0	0	0	0.126	0	0
etf1p3	0	0	0	0.126	0.126	0	0	0	0	0.126	0	0
psat1	0	0	0.11	0.153	0.153	0.11	0	0	0	0.153	0	0
sgip1	0.129	0	0	0	0	0	0.129	0	0.134	0	0	0
mob1b	0.152	0	0	0	0	0	0.152	0	0.155	0	0.11	0.108
slc38a3	0	0	0	0.225	0.225	0	0	0	0	0.225	0	0
iqsec3	0.15	0	0	0	0	0	0.15	0	0.168	0	0.103	0.102
ndufa6	0	0	0.108	0.151	0.151	0.108	0	0	0	0.151	0	0
rpl10	0	0	0	0.223	0.223	0	0	0	0	0.223	0	0
ndufa12	0	0	0.116	0.145	0.145	0.116	0	0	0	0.145	0	0
ppp1r42	0.145	0	0	0	0	0	0.145	0	0.151	0	0.114	0.112
vps26b	0.101	0	0	0.119	0.119	0	0.101	0	0.103	0.119	0	0
dnm3	0.141	0	0	0	0	0	0.141	0	0.161	0	0	0
khdrbs1	0.134	0	0	0	0	0	0.134	0	0.14	0	0.126	0.124
katnb1	0.131	0	0	0	0	0	0.131	0	0.133	0	0.13	0.128
s100a11	0	0	0	0.217	0.217	0	0	0	0	0.217	0	0
copz2	0.146	0	0	0	0	0	0.146	0	0.148	0	0.105	0.104
pdcd6	0	0	0	0.126	0.126	0	0	0	0	0.126	0	0
ppfia2	0.138	0	0	0	0	0	0.138	0	0.14	0	0.112	0.111
katna1	0.132	0	0	0	0	0	0.132	0	0.135	0	0.12	0.118
zc4h2	0.132	0	0	0	0	0	0.132	0	0.131	0	0.12	0.119
mtmr6	0.122	0	0	0	0	0	0.122	0	0.13	0	0	0
eif3b	0.108	0	0	0	0	0	0.108	0	0.113	0	0	0
hgsnat	0	0	0.148	0.111	0.111	0.148	0	0	0	0.111	0	0
supv3l1	0	0	0	0.209	0.209	0	0	0	0	0.209	0	0
clint1	0.128	0	0	0	0	0	0.128	0	0.135	0	0.117	0.115
rab11fip4	0.137	0	0	0	0	0	0.137	0	0.138	0	0.106	0.105
jmjd6	0.138	0	0	0	0	0	0.138	0	0.135	0	0.104	0.103
gpr27	0	0	0	0.114	0.114	0	0	0	0	0.114	0	0
mapk7	0.111	0	0	0	0	0	0.111	0	0.128	0	0	0
nip7	0.122	0	0.117	0	0	0.117	0.122	0	0.133	0	0	0
march5	0.102	0	0	0	0	0	0.102	0	0.112	0	0.147	0.147
cabp7	0	0	0	0.202	0.202	0	0	0	0	0.202	0	0
gapvd1	0	0	0	0	0	0	0	0	0	0	0	0
glul	0	0	0.101	0.134	0.134	0.101	0	0	0	0.134	0	0

sgk3	0	0	0	0.201	0.201	0	0	0	0	0.201	0	0
spata6	0	0	0	0.167	0.167	0	0	0	0.101	0.167	0	0
impdh1p11	0	0	0	0.123	0.123	0	0	0	0	0.123	0	0
kcnk13	0	0	0	0.2	0.2	0	0	0	0	0.2	0	0
lphn2	0.128	0	0	0	0	0	0.128	0	0.13	0	0	0
gulop	0	0	0.126	0.115	0.115	0.126	0	0	0	0.115	0	0
slc23a1	0	0	0	0.199	0.199	0	0	0	0	0.199	0	0
s1pr5	0.198	0	0	0	0	0	0.198	0	0.197	0	0	0
dmkn	0.112	0	0	0	0	0	0.112	0	0.114	0	0.126	0.125
tmem217	0.102	0	0	0	0	0	0.102	0	0.119	0	0	0
anxa6	0	0	0	0.196	0.196	0	0	0	0	0.196	0	0
ptpn20b	0.116	0	0	0	0	0	0.116	0	0.119	0	0	0
ssx2ip	0.194	0	0	0	0	0	0.194	0	0.198	0	0	0
caskin1	0.123	0	0	0	0	0	0.123	0	0.126	0	0.104	0.103
glud1	0	0	0	0.193	0.193	0	0	0	0	0.193	0	0
lcp1	0.122	0	0	0	0	0	0.122	0	0.123	0	0.107	0.105
gpsm3	0.192	0	0	0	0	0	0.192	0	0.193	0	0	0
ruvbl2	0.108	0	0	0	0	0	0.108	0	0.109	0	0.126	0.126
sipa1	0.191	0	0	0	0	0	0.191	0	0.194	0	0	0
hrh4	0	0	0	0.114	0.114	0	0	0	0	0.114	0	0
eri2	0	0	0	0	0	0	0	0	0	0	0	0
fcho1	0.185	0	0	0	0	0	0.185	0	0.197	0	0	0
acadl	0	0	0.106	0.117	0.117	0.106	0	0	0	0.117	0	0
pea15	0.187	0	0	0	0	0	0.187	0	0.189	0	0	0
arhgap22	0.186	0	0	0	0	0	0.186	0	0.186	0	0	0
stk38	0.117	0	0	0	0	0	0.117	0	0.119	0	0.102	0.1
acly	0	0	0.1	0.117	0.117	0.1	0	0	0	0.117	0	0
igkv1d-17	0.107	0	0	0	0	0	0.107	0	0.11	0	0.113	0.112
tes	0	0	0	0.103	0.103	0	0	0	0	0.103	0	0
acss2	0	0	0	0.182	0.182	0	0	0	0	0.182	0	0
gsta4	0	0	0.111	0.108	0.108	0.111	0	0	0	0.108	0	0
lpar2	0.179	0	0	0	0	0	0.179	0	0.183	0	0	0
kprp	0	0	0.103	0.111	0.111	0.103	0	0	0	0.111	0	0
arhgap5	0.178	0	0	0	0	0	0.178	0	0.183	0	0	0
map3k9	0.103	0	0	0	0	0	0.103	0	0.103	0	0.114	0.112
gng13	0.106	0	0	0	0	0	0.106	0	0.112	0	0	0
ehd2	0.176	0	0	0	0	0	0.176	0	0.181	0	0	0
slc13a5	0	0	0	0.177	0.177	0	0	0	0	0.177	0	0
acss1	0	0	0	0.176	0.176	0	0	0	0	0.176	0	0
h2bfm	0	0	0	0.176	0.176	0	0	0	0	0.176	0	0
mapre1	0.106	0	0	0	0	0	0.106	0	0.108	0	0.103	0.102

vps35	0.106	0	0	0	0	0	0.106	0	0.109	0	0.102	0.101
mir1287	0	0	0.104	0.104	0.104	0.104	0	0	0	0.104	0	0
btd	0	0	0	0.173	0.173	0	0	0	0	0.173	0	0
fam65a	0	0	0	0.173	0.173	0	0	0	0	0.173	0	0
nol12	0.168	0	0	0	0	0	0.168	0	0.182	0	0	0
slc3a1	0	0	0	0.17	0.17	0	0	0	0	0.17	0	0
btg1	0	0	0	0.169	0.169	0	0	0	0	0.169	0	0
usp6	0.164	0	0	0	0	0	0.164	0	0.179	0	0	0
ppp1cb	0.168	0	0	0	0	0	0.168	0	0.169	0	0	0
arcn1	0.163	0	0	0	0	0	0.163	0	0.169	0	0	0
copa	0.162	0	0	0	0	0	0.162	0	0.17	0	0	0
spesp1	0	0	0	0.164	0.164	0	0	0	0	0.164	0	0
arhgef16	0.124	0	0	0	0	0	0.124	0	0.141	0	0	0
capn6	0.164	0	0	0	0	0	0.164	0	0.162	0	0	0
schip1	0.161	0	0	0	0	0	0.161	0	0.167	0	0	0
trip11	0.162	0	0	0	0	0	0.162	0	0.165	0	0	0
bckdhb	0	0	0	0.162	0.162	0	0	0	0	0.162	0	0
nr4a2	0	0	0.243	0	0	0.243	0	0	0	0	0	0
slc1a5	0	0	0	0.161	0.161	0	0	0	0	0.161	0	0
c12orf39	0	0	0	0	0	0	0	0	0	0	0	0
cpne9	0	0	0	0.16	0.16	0	0	0	0	0.16	0	0
edc3	0	0	0	0.16	0.16	0	0	0	0	0.16	0	0
znf644	0.158	0	0	0	0	0	0.158	0	0.159	0	0	0
anxa8l2	0	0	0	0.158	0.158	0	0	0	0	0.158	0	0
ceacam8	0	0	0	0.158	0.158	0	0	0	0	0.158	0	0
c8orf17	0	0	0	0	0	0	0	0	0	0	0	0
rab11fip2	0.156	0	0	0	0	0	0.156	0	0.161	0	0	0
pnpla8	0	0	0	0.157	0.157	0	0	0	0	0.157	0	0
slc23a2	0	0	0	0.157	0.157	0	0	0	0	0.157	0	0
mtif3	0.117	0	0	0	0	0	0.117	0	0.134	0	0	0
c8orf44-sgk3	0	0	0	0.156	0.156	0	0	0	0	0.156	0	0
dennd5a	0.154	0	0	0	0	0	0.154	0	0.16	0	0	0
hint2	0	0	0	0.156	0.156	0	0	0	0	0.156	0	0
txndc2	0	0	0	0.156	0.156	0	0	0	0	0.156	0	0
cpne6	0	0	0	0.121	0.121	0	0	0	0	0.121	0	0
amd1	0	0	0	0.154	0.154	0	0	0	0	0.154	0	0
actn4	0.153	0	0	0	0	0	0.153	0	0.155	0	0	0
mcu	0	0	0	0.152	0.152	0	0	0	0	0.152	0	0
or1d5	0	0	0	0.152	0.152	0	0	0	0	0.152	0	0
pum2	0	0	0	0.152	0.152	0	0	0	0	0.152	0	0
actn2	0.15	0	0	0	0	0	0.15	0	0.154	0	0	0

scamp3	0	0	0	0	0	0	0	0	0	0	0	0
gucy2gp	0	0	0	0.151	0.151	0	0	0	0	0.151	0	0
nudt16	0	0	0	0.151	0.151	0	0	0	0	0.151	0	0
rab20	0	0	0	0.105	0.105	0	0	0	0.137	0.105	0	0
cpd	0	0	0	0.15	0.15	0	0	0	0	0.15	0	0
nme9	0	0	0	0.15	0.15	0	0	0	0	0.15	0	0
rasgef1b	0	0	0	0.15	0.15	0	0	0	0	0.15	0	0
adam12-ot1	0	0	0	0.149	0.149	0	0	0	0	0.149	0	0
lcn1	0	0	0	0.149	0.149	0	0	0	0	0.149	0	0
sept14	0.148	0	0	0	0	0	0.148	0	0.15	0	0	0
pyurf	0.149	0	0	0	0	0	0.149	0	0.148	0	0	0
fnbp1	0	0	0	0	0	0	0	0	0	0	0.11	0.109
atp13a1	0	0	0	0.148	0.148	0	0	0	0	0.148	0	0
svop	0	0	0	0.148	0.148	0	0	0	0	0.148	0	0
tufm	0	0	0	0.148	0.148	0	0	0	0	0.148	0	0
mpp1	0	0	0	0.113	0.113	0	0	0	0.104	0.113	0	0
khsrp	0	0	0	0.147	0.147	0	0	0	0	0.147	0	0
snora67	0	0	0	0.147	0.147	0	0	0	0	0.147	0	0
snora68	0	0	0	0.147	0.147	0	0	0	0	0.147	0	0
mir122	0	0	0.22	0	0	0.22	0	0	0	0	0	0
glud2	0	0	0	0.146	0.146	0	0	0	0	0.146	0	0
hmgn1	0	0	0	0.146	0.146	0	0	0	0	0.146	0	0
mir15a	0	0	0	0.146	0.146	0	0	0	0	0.146	0	0
ncs1	0	0	0	0.146	0.146	0	0	0	0	0.146	0	0
cd2bp2	0	0	0	0	0	0	0	0	0	0	0	0
kank3	0.145	0	0	0	0	0	0.145	0	0.147	0	0	0
kank4	0.145	0	0	0	0	0	0.145	0	0.147	0	0	0
tpm4	0.141	0	0	0	0	0	0.141	0	0.155	0	0	0
atp1a4	0	0	0	0.145	0.145	0	0	0	0	0.145	0	0
hdhd1	0	0	0	0.145	0.145	0	0	0	0	0.145	0	0
lrrn3	0	0	0	0.145	0.145	0	0	0	0	0.145	0	0
sms	0	0	0	0.144	0.144	0	0	0	0	0.144	0	0
ovca2	0	0	0	0.109	0.109	0	0	0	0.104	0.109	0	0
gid8	0.144	0	0	0	0	0	0.144	0	0.143	0	0	0
ppp5c	0.144	0	0	0	0	0	0.144	0	0.143	0	0	0
atp1b4	0	0	0	0.143	0.143	0	0	0	0	0.143	0	0
gng10	0	0	0	0.143	0.143	0	0	0	0	0.143	0	0
slc6a6	0	0	0	0.142	0.142	0	0	0	0	0.142	0	0
tmem59l	0	0	0	0.142	0.142	0	0	0	0	0.142	0	0
cep70	0.139	0	0	0	0	0	0.139	0	0.146	0	0	0
entpd3	0	0	0	0.141	0.141	0	0	0	0	0.141	0	0

krt12	0	0	0	0.141	0.141	0	0	0	0	0.141	0	0
mdp1	0	0	0	0.141	0.141	0	0	0	0	0.141	0	0
myo1b	0.14	0	0	0	0	0	0.14	0	0.141	0	0	0
arfgef1	0.135	0	0	0	0	0	0.135	0	0.15	0	0	0
snhg16	0	0	0	0.14	0.14	0	0	0	0	0.14	0	0
tuba4b	0	0	0	0.14	0.14	0	0	0	0	0.14	0	0
cope	0.137	0	0	0	0	0	0.137	0	0.144	0	0	0
c2cd2l	0	0	0	0.139	0.139	0	0	0	0	0.139	0	0
caprin2	0	0	0	0.139	0.139	0	0	0	0	0.139	0	0
gca	0	0	0	0.139	0.139	0	0	0	0	0.139	0	0
hmga1	0	0	0	0.139	0.139	0	0	0	0	0.139	0	0
mettl7b	0	0	0	0.139	0.139	0	0	0	0	0.139	0	0
prdm8	0	0	0	0.139	0.139	0	0	0	0	0.139	0	0
sh3gl1	0.138	0	0	0	0	0	0.138	0	0.14	0	0	0
ccdc91	0.136	0	0	0	0	0	0.136	0	0.143	0	0	0
adssl1	0	0	0	0.138	0.138	0	0	0	0	0.138	0	0
atp6v1c2	0	0	0	0.138	0.138	0	0	0	0	0.138	0	0
epb41l4b	0.104	0	0	0	0	0	0.104	0	0.106	0	0	0
oaz2	0	0	0	0.138	0.138	0	0	0	0	0.138	0	0
usp51	0	0	0	0.138	0.138	0	0	0	0	0.138	0	0
opcml	0	0	0	0	0	0	0	0	0	0	0	0
gng3	0.137	0	0	0	0	0	0.137	0	0.138	0	0	0
aim1	0	0	0	0.137	0.137	0	0	0	0	0.137	0	0
mctp1	0	0	0	0.137	0.137	0	0	0	0	0.137	0	0
skcg-1	0	0	0	0.137	0.137	0	0	0	0	0.137	0	0
pskh1	0.134	0	0	0	0	0	0.134	0	0.142	0	0	0
gfpt1	0	0	0	0.136	0.136	0	0	0	0	0.136	0	0
ptbp3	0	0	0	0.136	0.136	0	0	0	0	0.136	0	0
slc16a10	0	0	0	0.136	0.136	0	0	0	0	0.136	0	0
slc25a18	0	0	0	0.136	0.136	0	0	0	0	0.136	0	0
ampd2	0.135	0	0	0	0	0	0.135	0	0.137	0	0	0
lgsn	0	0	0	0	0	0	0	0	0	0	0	0
linc00470	0	0	0	0.135	0.135	0	0	0	0	0.135	0	0
kank2	0.134	0	0	0	0	0	0.134	0	0.136	0	0	0
skap2	0.133	0	0	0	0	0	0.133	0	0.138	0	0	0
ak1	0	0	0	0.134	0.134	0	0	0	0	0.134	0	0
nudt21	0	0	0	0.134	0.134	0	0	0	0	0.134	0	0
mapre3	0.132	0	0	0	0	0	0.132	0	0.137	0	0	0
rnf115	0	0	0	0	0	0	0	0	0	0	0	0
dpf3	0	0	0.2	0	0	0.2	0	0	0	0	0	0
map3k3	0.133	0	0	0	0	0	0.133	0	0.134	0	0	0

bzrap1	0	0	0	0.133	0.133	0	0	0	0	0.133	0	0
calml5	0	0	0	0.132	0.132	0	0	0	0	0.132	0	0
ofd1p17	0	0	0	0.132	0.132	0	0	0	0	0.132	0	0
ofd1p18y	0	0	0	0.132	0.132	0	0	0	0	0.132	0	0
brms1	0	0	0	0.131	0.131	0	0	0	0	0.131	0	0
cln3	0	0	0	0.131	0.131	0	0	0	0	0.131	0	0
cnnm1	0	0	0	0.131	0.131	0	0	0	0	0.131	0	0
hagh	0	0	0	0.131	0.131	0	0	0	0	0.131	0	0
linc00984	0	0	0	0.131	0.131	0	0	0	0	0.131	0	0
mir4435-1hg	0	0	0	0.131	0.131	0	0	0	0	0.131	0	0
pde7a	0	0	0	0.131	0.131	0	0	0	0	0.131	0	0
proa	0	0	0	0.131	0.131	0	0	0	0	0.131	0	0
sat1	0	0	0	0.131	0.131	0	0	0	0	0.131	0	0
tfb2m	0	0	0	0.131	0.131	0	0	0	0	0.131	0	0
gnb2	0.13	0	0	0	0	0	0.13	0	0.131	0	0	0
aebp1	0	0	0	0.13	0.13	0	0	0	0	0.13	0	0
bcat1	0	0	0	0.13	0.13	0	0	0	0	0.13	0	0
brd9	0	0	0.195	0	0	0.195	0	0	0	0	0	0
c12orf75	0	0	0	0.13	0.13	0	0	0	0	0.13	0	0
cnnm3	0	0	0	0.13	0.13	0	0	0	0	0.13	0	0
defb125	0	0	0	0.13	0.13	0	0	0	0	0.13	0	0
dlc1	0.128	0	0	0	0	0	0.128	0	0.134	0	0	0
mfsd11	0	0	0	0.13	0.13	0	0	0	0	0.13	0	0
pygm	0	0	0	0.13	0.13	0	0	0	0	0.13	0	0
slc10a7	0	0	0	0.13	0.13	0	0	0	0	0.13	0	0
znf24	0	0	0.194	0	0	0.194	0	0	0	0	0	0
kcnip1	0	0	0	0.129	0.129	0	0	0	0	0.129	0	0
pld6	0	0	0	0.129	0.129	0	0	0	0	0.129	0	0
polr2g	0	0	0	0.129	0.129	0	0	0	0	0.129	0	0
prkd1	0.129	0	0	0	0	0	0.129	0	0.129	0	0	0
sec14l2	0.129	0	0	0	0	0	0.129	0	0.129	0	0	0
slc26a8	0	0	0	0.129	0.129	0	0	0	0	0.129	0	0
srsf2	0	0	0	0.129	0.129	0	0	0	0	0.129	0	0
nrbp1	0.128	0	0	0	0	0	0.128	0	0.129	0	0	0
bckdha	0	0	0	0.128	0.128	0	0	0	0	0.128	0	0
dync1li2	0.128	0	0	0	0	0	0.128	0	0.128	0	0	0
ndufa7	0	0	0	0.128	0.128	0	0	0	0	0.128	0	0
wnk2	0	0	0	0.128	0.128	0	0	0	0	0.128	0	0
arl6	0.125	0	0	0	0	0	0.125	0	0.133	0	0	0
rab43	0.126	0	0	0	0	0	0.126	0	0.131	0	0	0
inf2	0.127	0	0	0	0	0	0.127	0	0.128	0	0	0

aacs	0	0	0	0.127	0.127	0	0	0	0	0.127	0	0
cox4i2	0	0	0	0.127	0.127	0	0	0	0	0.127	0	0
defb105b	0	0	0	0.127	0.127	0	0	0	0	0.127	0	0
defb107a	0	0	0	0.127	0.127	0	0	0	0	0.127	0	0
defb107b	0	0	0	0.127	0.127	0	0	0	0	0.127	0	0
mrs2	0	0	0	0.127	0.127	0	0	0	0	0.127	0	0
pdcd7	0	0	0	0.127	0.127	0	0	0	0	0.127	0	0
slc7a5	0	0	0	0.127	0.127	0	0	0	0	0.127	0	0
znf354b	0	0	0	0.127	0.127	0	0	0	0	0.127	0	0
dync1li1	0.127	0	0	0	0	0	0.127	0	0.126	0	0	0
sept1	0.126	0	0	0	0	0	0.126	0	0.127	0	0	0
dpy19l2p2	0	0	0	0.126	0.126	0	0	0	0	0.126	0	0
dpy19l2p4	0	0	0	0.126	0.126	0	0	0	0	0.126	0	0
gng5	0	0	0	0.126	0.126	0	0	0	0	0.126	0	0
lpin2	0	0	0	0.126	0.126	0	0	0	0	0.126	0	0
mrpl46	0	0	0	0.126	0.126	0	0	0	0	0.126	0	0
qsox1	0	0	0	0.126	0.126	0	0	0	0	0.126	0	0
slc7a6	0	0	0	0.126	0.126	0	0	0	0	0.126	0	0
smox	0	0	0	0.126	0.126	0	0	0	0	0.126	0	0
spam1	0	0	0	0.126	0.126	0	0	0	0	0.126	0	0
spata21	0	0	0	0.126	0.126	0	0	0	0	0.126	0	0
mob2	0.124	0	0	0	0	0	0.124	0	0.128	0	0	0
ralgps1	0.125	0	0	0	0	0	0.125	0	0.126	0	0	0
arhgap19	0.124	0	0	0	0	0	0.124	0	0.127	0	0	0
barhl1	0	0	0	0.125	0.125	0	0	0	0	0.125	0	0
btn1a1	0	0	0	0.125	0.125	0	0	0	0	0.125	0	0
plscr2	0	0	0	0.125	0.125	0	0	0	0	0.125	0	0
ppp2r1b	0	0	0	0.125	0.125	0	0	0	0	0.125	0	0
tceal8	0	0	0	0.125	0.125	0	0	0	0	0.125	0	0
myl12b	0.125	0	0	0	0	0	0.125	0	0.124	0	0	0
alpp	0	0	0	0.124	0.124	0	0	0	0	0.124	0	0
gspt1	0	0	0	0.124	0.124	0	0	0	0	0.124	0	0
mir26a1	0	0	0	0.124	0.124	0	0	0	0	0.124	0	0
pdha2	0	0	0	0.124	0.124	0	0	0	0	0.124	0	0
rp24	0	0	0	0.124	0.124	0	0	0	0	0.124	0	0
slc31a1	0	0	0	0.124	0.124	0	0	0	0	0.124	0	0
eps15	0.116	0	0	0	0	0	0.116	0	0.139	0	0	0
frmpd1	0.122	0	0	0	0	0	0.122	0	0.127	0	0	0
atad2b	0	0	0	0.123	0.123	0	0	0	0	0.123	0	0
b3gntl1	0	0	0	0.123	0.123	0	0	0	0	0.123	0	0
cdc73	0	0	0	0.123	0.123	0	0	0	0	0.123	0	0

dnah2	0	0	0	0.123	0.123	0	0	0	0	0.123	0	0
gnpda1	0	0	0	0.123	0.123	0	0	0	0	0.123	0	0
nek8	0	0	0	0.123	0.123	0	0	0	0	0.123	0	0
obp2b	0	0	0	0.123	0.123	0	0	0	0	0.123	0	0
tvp23a	0	0	0	0.123	0.123	0	0	0	0	0.123	0	0
tvp23b	0	0	0	0.123	0.123	0	0	0	0	0.123	0	0
arih2	0	0	0	0	0	0	0	0	0	0	0	0
bckdk	0	0	0	0.122	0.122	0	0	0	0	0.122	0	0
elk1	0	0	0	0.122	0.122	0	0	0	0	0.122	0	0
fam221b	0	0	0	0.122	0.122	0	0	0	0	0.122	0	0
mcoln2	0	0	0	0.122	0.122	0	0	0	0	0.122	0	0
msto1	0	0	0	0.122	0.122	0	0	0	0	0.122	0	0
naspp1	0	0	0	0.122	0.122	0	0	0	0	0.122	0	0
ppp2r5c	0.12	0	0	0	0	0	0.12	0	0.126	0	0	0
rab8b	0	0	0	0.122	0.122	0	0	0	0	0.122	0	0
samd9l	0	0	0	0.122	0.122	0	0	0	0	0.122	0	0
trappc1	0	0	0	0.122	0.122	0	0	0	0	0.122	0	0
copz1	0.121	0	0	0	0	0	0.121	0	0.123	0	0	0
izumo1	0	0	0	0	0	0	0	0	0.101	0	0.132	0.132
g3bp1	0.118	0	0	0	0	0	0.118	0	0.128	0	0	0
eif2b4	0	0	0	0.121	0.121	0	0	0	0	0.121	0	0
etf1	0	0	0	0.121	0.121	0	0	0	0	0.121	0	0
golim4	0	0	0	0.121	0.121	0	0	0	0	0.121	0	0
ogdh	0	0	0	0.121	0.121	0	0	0	0	0.121	0	0
phtf2	0	0	0	0.121	0.121	0	0	0	0	0.121	0	0
pim1	0	0	0	0.121	0.121	0	0	0	0	0.121	0	0
rn7sk	0	0	0	0.121	0.121	0	0	0	0	0.121	0	0
ophn1	0.118	0	0	0	0	0	0.118	0	0.126	0	0	0
rab33b	0.117	0	0	0	0	0	0.117	0	0.128	0	0	0
rab6b	0.118	0	0	0	0	0	0.118	0	0.126	0	0	0
appl2	0.117	0	0	0	0	0	0.117	0	0.127	0	0	0
ccdc41	0.117	0	0	0	0	0	0.117	0	0.127	0	0	0
chiap2	0	0	0	0.12	0.12	0	0	0	0	0.12	0	0
dmrt3	0	0	0	0.12	0.12	0	0	0	0	0.12	0	0
epg5	0	0	0	0.12	0.12	0	0	0	0	0.12	0	0
folr2	0	0	0	0.12	0.12	0	0	0	0	0.12	0	0
gprc6a	0	0	0	0.12	0.12	0	0	0	0	0.12	0	0
lsm4	0	0	0	0.12	0.12	0	0	0	0	0.12	0	0
znf181	0	0	0	0.12	0.12	0	0	0	0	0.12	0	0
rab5b	0.113	0	0	0	0	0	0.113	0	0.133	0	0	0
tmbim6	0	0	0.179	0	0	0.179	0	0	0	0	0	0

dlat	0	0	0	0.119	0.119	0	0	0	0	0.119	0	0
hsd3b7	0	0	0	0.119	0.119	0	0	0	0	0.119	0	0
slc19a2	0	0	0	0.119	0.119	0	0	0	0	0.119	0	0
mtmr1	0.118	0	0	0	0	0	0.118	0	0.12	0	0	0
scyl2	0.118	0	0	0	0	0	0.118	0	0.12	0	0	0
spry1	0	0	0	0	0	0	0	0	0	0	0	0
elmod1	0.116	0	0	0	0	0	0.116	0	0.123	0	0	0
sphk2	0.118	0	0	0	0	0	0.118	0	0.119	0	0	0
loc440895	0	0	0	0.118	0.118	0	0	0	0	0.118	0	0
oaz1	0	0	0	0.118	0.118	0	0	0	0	0.118	0	0
znf18	0	0	0	0.118	0.118	0	0	0	0	0.118	0	0
cltcl1	0.116	0	0	0	0	0	0.116	0	0.121	0	0	0
ampd3	0	0	0	0.117	0.117	0	0	0	0	0.117	0	0
emx2os	0	0	0	0.117	0.117	0	0	0	0	0.117	0	0
nags	0	0	0	0.117	0.117	0	0	0	0	0.117	0	0
ppp1r2p1	0	0	0	0.117	0.117	0	0	0	0	0.117	0	0
prpsap2	0	0	0	0.117	0.117	0	0	0	0	0.117	0	0
seh1l	0	0	0	0.117	0.117	0	0	0	0	0.117	0	0
arl8a	0.113	0	0	0	0	0	0.113	0	0.124	0	0	0
cbln4	0	0	0	0.116	0.116	0	0	0	0	0.116	0	0
heatr1	0	0	0	0.116	0.116	0	0	0	0	0.116	0	0
prr5l	0.116	0	0	0	0	0	0.116	0	0.116	0	0	0
ptpn3	0.114	0	0	0	0	0	0.114	0	0.12	0	0	0
specc1	0.113	0	0	0	0	0	0.113	0	0.12	0	0	0
best3	0	0	0	0.115	0.115	0	0	0	0	0.115	0	0
pafah1b1p1	0	0	0	0.115	0.115	0	0	0	0	0.115	0	0
pafah1b1p2	0	0	0	0.115	0.115	0	0	0	0	0.115	0	0
retsat	0	0	0	0.115	0.115	0	0	0	0	0.115	0	0
slc6a16	0	0	0	0.115	0.115	0	0	0	0	0.115	0	0
snrpc	0	0	0	0.115	0.115	0	0	0	0	0.115	0	0
tdo2	0	0	0	0.115	0.115	0	0	0	0	0.115	0	0
tec	0	0	0	0.115	0.115	0	0	0	0	0.115	0	0
wdr13	0	0	0	0.115	0.115	0	0	0	0	0.115	0	0
tuba8	0.113	0	0	0	0	0	0.113	0	0.119	0	0	0
gmfg	0.114	0	0	0	0	0	0.114	0	0.116	0	0	0
dnah1	0.112	0	0	0	0	0	0.112	0	0.119	0	0	0
fscn3	0.112	0	0	0	0	0	0.112	0	0.119	0	0	0
acy1	0	0	0	0.114	0.114	0	0	0	0	0.114	0	0
ccdc37	0	0	0	0.114	0.114	0	0	0	0	0.114	0	0
fam120b	0	0	0	0.114	0.114	0	0	0	0	0.114	0	0
gmpr2	0	0	0	0.114	0.114	0	0	0	0	0.114	0	0

midn	0	0	0.171	0	0	0.171	0	0	0	0	0	0
otud1	0	0	0	0.114	0.114	0	0	0	0	0.114	0	0
fli1	0	0	0	0	0	0	0	0	0	0	0	0
golph3	0.11	0	0	0	0	0	0.11	0	0.122	0	0	0
azin1	0	0	0	0.113	0.113	0	0	0	0	0.113	0	0
defb106b	0	0	0	0.113	0.113	0	0	0	0	0.113	0	0
myo15a	0	0	0	0.113	0.113	0	0	0	0	0.113	0	0
snord12c	0	0	0	0.113	0.113	0	0	0	0	0.113	0	0
wnt9b	0	0	0	0.113	0.113	0	0	0	0	0.113	0	0
cstf2	0.111	0	0	0	0	0	0.111	0	0.115	0	0	0
anxa11	0	0	0	0.112	0.112	0	0	0	0	0.112	0	0
csn1s2bp	0	0	0	0.112	0.112	0	0	0	0	0.112	0	0
pygl	0	0	0	0.112	0.112	0	0	0	0	0.112	0	0
snora69	0	0	0	0.112	0.112	0	0	0	0	0.112	0	0
tbc1d5	0.112	0	0	0	0	0	0.112	0	0.112	0	0	0
tff3	0	0	0	0.112	0.112	0	0	0	0	0.112	0	0
trnt1	0	0	0	0.112	0.112	0	0	0	0	0.112	0	0
rep15	0	0	0	0	0	0	0	0	0.1	0	0	0
stk3	0.11	0	0	0	0	0	0.11	0	0.114	0	0	0
ankrd7	0	0	0	0.111	0.111	0	0	0	0	0.111	0	0
dld	0	0	0	0.111	0.111	0	0	0	0	0.111	0	0
dnajc4	0	0	0	0.111	0.111	0	0	0	0	0.111	0	0
pdk3	0	0	0	0.111	0.111	0	0	0	0	0.111	0	0
ptcsc1	0	0	0	0.111	0.111	0	0	0	0	0.111	0	0
rfk	0	0	0	0.111	0.111	0	0	0	0	0.111	0	0
farp2	0.109	0	0	0	0	0	0.109	0	0.114	0	0	0
ppp1ca	0.11	0	0	0	0	0	0.11	0	0.112	0	0	0
ywhab	0.11	0	0	0	0	0	0.11	0	0.112	0	0	0
relt	0.11	0	0	0	0	0	0.11	0	0.111	0	0	0
feb7	0	0	0	0.11	0.11	0	0	0	0	0.11	0	0
atp5g1	0	0	0	0.11	0.11	0	0	0	0	0.11	0	0
bola3	0	0	0	0.11	0.11	0	0	0	0	0.11	0	0
c19orf59	0	0	0	0.11	0.11	0	0	0	0	0.11	0	0
dmxl1	0	0	0	0.11	0.11	0	0	0	0	0.11	0	0
emc4	0	0	0	0.11	0.11	0	0	0	0	0.11	0	0
ppap2c	0	0	0	0.11	0.11	0	0	0	0	0.11	0	0
rnu1-4	0	0	0	0.11	0.11	0	0	0	0	0.11	0	0
rpl3l	0	0	0	0.11	0.11	0	0	0	0	0.11	0	0
rpph1	0	0	0	0.11	0.11	0	0	0	0	0.11	0	0
mkln1	0.108	0	0	0	0	0	0.108	0	0.113	0	0	0
ap2a1	0.108	0	0	0	0	0	0.108	0	0.112	0	0	0

uri1	0.106	0	0	0	0	0	0.106	0	0.116	0	0	0
atp11a	0	0	0	0.109	0.109	0	0	0	0	0.109	0	0
cdh12	0	0	0	0.109	0.109	0	0	0	0	0.109	0	0
defb105a	0	0	0	0.109	0.109	0	0	0	0	0.109	0	0
dnase2b	0	0	0	0.109	0.109	0	0	0	0	0.109	0	0
ifrd2	0	0	0	0.109	0.109	0	0	0	0	0.109	0	0
il1rapl1	0	0	0	0.109	0.109	0	0	0	0	0.109	0	0
pkdrej	0	0	0	0.109	0.109	0	0	0	0	0.109	0	0
rab9bp1	0	0	0	0.109	0.109	0	0	0	0	0.109	0	0
slc6a8	0	0	0	0.109	0.109	0	0	0	0	0.109	0	0
zc3h4	0	0	0	0.109	0.109	0	0	0	0	0.109	0	0
akap6	0.107	0	0	0	0	0	0.107	0	0.112	0	0	0
pabpc1	0.108	0	0	0	0	0	0.108	0	0.11	0	0	0
arsep1	0	0	0	0.108	0.108	0	0	0	0	0.108	0	0
dsp	0	0	0	0.108	0.108	0	0	0	0	0.108	0	0
fstl4	0	0	0	0.108	0.108	0	0	0	0	0.108	0	0
hibch	0	0	0	0.108	0.108	0	0	0	0	0.108	0	0
krt2	0	0	0	0.108	0.108	0	0	0	0	0.108	0	0
pafah1b1	0	0	0	0.108	0.108	0	0	0	0	0.108	0	0
rasgrf1	0.106	0	0	0	0	0	0.106	0	0.112	0	0	0
tulp4	0	0	0	0.108	0.108	0	0	0	0	0.108	0	0
ubr7	0	0	0	0.108	0.108	0	0	0	0	0.108	0	0
myl5	0.108	0	0	0	0	0	0.108	0	0.106	0	0	0
rtp3	0	0	0	0	0	0	0	0	0	0	0	0
catsper1	0	0	0	0.107	0.107	0	0	0	0	0.107	0	0
dach1	0	0	0	0.107	0.107	0	0	0	0	0.107	0	0
dnah10	0	0	0	0.107	0.107	0	0	0	0	0.107	0	0
dnah6	0	0	0	0.107	0.107	0	0	0	0	0.107	0	0
fbxw12	0	0	0	0.107	0.107	0	0	0	0	0.107	0	0
impact	0	0	0	0.107	0.107	0	0	0	0	0.107	0	0
ndufs3	0	0	0	0.107	0.107	0	0	0	0	0.107	0	0
neto2	0	0	0	0.107	0.107	0	0	0	0	0.107	0	0
appl1	0	0	0	0.106	0.106	0	0	0	0	0.106	0	0
cpo	0	0	0	0.106	0.106	0	0	0	0	0.106	0	0
crtac1	0	0	0	0.106	0.106	0	0	0	0	0.106	0	0
hdgfl1	0	0	0	0.106	0.106	0	0	0	0	0.106	0	0
mat2b	0	0	0	0.106	0.106	0	0	0	0	0.106	0	0
pate1	0	0	0	0.106	0.106	0	0	0	0	0.106	0	0
rgl1	0.105	0	0	0	0	0	0.105	0	0.108	0	0	0
slc30a10	0	0	0	0.106	0.106	0	0	0	0	0.106	0	0
u2af2	0	0	0	0.106	0.106	0	0	0	0	0.106	0	0

ccnl2	0.102	0	0	0	0	0	0.102	0	0.113	0	0	0
sept7	0.103	0	0	0	0	0	0.103	0	0.11	0	0	0
abracl	0	0	0	0	0	0	0	0	0	0	0	0
rab11fip1	0.105	0	0	0	0	0	0.105	0	0.106	0	0	0
dhx15	0	0	0	0.105	0.105	0	0	0	0	0.105	0	0
khnyn	0	0	0	0.105	0.105	0	0	0	0	0.105	0	0
lrrc6	0	0	0	0.105	0.105	0	0	0	0	0.105	0	0
ndufaf1	0	0	0	0.105	0.105	0	0	0	0	0.105	0	0
slc27a5	0	0	0	0.105	0.105	0	0	0	0	0.105	0	0
arhgap25	0.104	0	0	0	0	0	0.104	0	0.106	0	0	0
rab25	0.103	0	0	0	0	0	0.103	0	0.107	0	0	0
dmrtb1	0	0	0	0.104	0.104	0	0	0	0	0.104	0	0
gtpbp10	0	0	0	0.104	0.104	0	0	0	0	0.104	0	0
kctd3	0	0	0	0.104	0.104	0	0	0	0	0.104	0	0
obsl1	0.104	0	0	0	0	0	0.104	0	0.104	0	0	0
pdk2	0	0	0	0.104	0.104	0	0	0	0	0.104	0	0
rcan2	0	0	0	0.104	0.104	0	0	0	0	0.104	0	0
rpl9	0	0	0	0.104	0.104	0	0	0	0	0.104	0	0
smu1	0	0	0	0	0	0	0	0	0	0	0	0
snora62	0	0	0	0.104	0.104	0	0	0	0	0.104	0	0
zp1	0	0	0	0.104	0.104	0	0	0	0	0.104	0	0
lrrc14	0.1	0	0	0	0	0	0.1	0	0.11	0	0	0
lrrc24	0.1	0	0	0	0	0	0.1	0	0.11	0	0	0
alppl2	0	0	0	0.103	0.103	0	0	0	0	0.103	0	0
anp32d	0	0	0	0	0	0	0	0	0	0	0	0
lco	0	0	0	0.103	0.103	0	0	0	0	0.103	0	0
mlycd	0	0	0	0.103	0.103	0	0	0	0	0.103	0	0
slc25a13	0	0	0	0.103	0.103	0	0	0	0	0.103	0	0
stmn3	0.102	0	0	0	0	0	0.102	0	0.105	0	0	0
tff1	0	0	0	0.103	0.103	0	0	0	0	0.103	0	0
caskin2	0	0	0	0	0	0	0	0	0.104	0	0	0
cnot11	0.103	0	0	0	0	0	0.103	0	0.102	0	0	0
nphp4	0	0	0	0	0	0	0	0	0.103	0	0	0
rasa4b	0.101	0	0	0	0	0	0.101	0	0.105	0	0	0
bspry	0	0	0	0.102	0.102	0	0	0	0	0.102	0	0
dars2	0	0	0	0.102	0.102	0	0	0	0	0.102	0	0
eif1	0	0	0	0.102	0.102	0	0	0	0	0.102	0	0
fbp1	0	0	0	0.102	0.102	0	0	0	0	0.102	0	0
fxn	0	0	0	0.102	0.102	0	0	0	0	0.102	0	0
myo16	0	0	0	0.102	0.102	0	0	0	0	0.102	0	0
papss1	0	0	0	0.102	0.102	0	0	0	0	0.102	0	0

prkxp1	0	0	0	0.102	0.102	0	0	0	0	0.102	0	0
rln3	0	0	0	0.102	0.102	0	0	0	0	0.102	0	0
slc7a10	0	0	0	0.102	0.102	0	0	0	0	0.102	0	0
smoc1	0	0	0	0.102	0.102	0	0	0	0	0.102	0	0
timm10	0	0	0	0.102	0.102	0	0	0	0	0.102	0	0
znf358	0	0	0	0.102	0.102	0	0	0	0	0.102	0	0
mterfd3	0.102	0	0	0	0	0	0.102	0	0.101	0	0	0
dpy19l2	0	0	0	0.101	0.101	0	0	0	0	0.101	0	0
emilin2	0	0	0	0.101	0.101	0	0	0	0	0.101	0	0
klhl32	0	0	0	0.101	0.101	0	0	0	0	0.101	0	0
prkx	0	0	0	0.101	0.101	0	0	0	0	0.101	0	0
rnf112	0	0	0	0.101	0.101	0	0	0	0	0.101	0	0
tut1	0	0	0	0.101	0.101	0	0	0	0	0.101	0	0
zfyve1	0	0	0	0.101	0.101	0	0	0	0	0.101	0	0
mff	0.1	0	0	0	0	0	0.1	0	0.102	0	0	0
b4galt1	0	0	0	0.1	0.1	0	0	0	0	0.1	0	0
gpr139	0	0	0	0.1	0.1	0	0	0	0	0.1	0	0
hist1h3h	0	0	0	0.1	0.1	0	0	0	0	0.1	0	0
kcnrg	0	0	0	0.1	0.1	0	0	0	0	0.1	0	0
mrpl43	0	0	0	0.1	0.1	0	0	0	0	0.1	0	0
szt2	0	0	0	0.1	0.1	0	0	0	0	0.1	0	0
ints4	0	0	0	0	0	0	0	0	0	0	0	0
ncapg2	0	0	0	0	0	0	0	0	0	0	0	0
tyw5	0	0	0.145	0	0	0.145	0	0	0	0	0	0
asxl1	0	0	0	0	0	0	0	0	0	0	0	0
pclo	0	0	0	0	0	0	0	0	0	0	0	0
bnc1	0	0	0	0	0	0	0	0	0	0	0	0
tspan11	0	0	0	0	0	0	0	0	0	0	0	0
echs1	0	0	0	0	0	0	0	0	0	0	0	0
galr3	0	0	0	0	0	0	0	0	0	0	0	0
slc35b1	0	0	0	0	0	0	0	0	0	0	0	0
jakmip3	0	0	0	0	0	0	0	0	0	0	0	0
nelfcd	0	0	0	0	0	0	0	0	0	0	0.128	0.126
wdr19	0	0	0	0	0	0	0	0	0	0	0	0
mocos	0	0	0.124	0	0	0.124	0	0	0	0	0	0
murc	0	0	0.124	0	0	0.124	0	0	0	0	0	0
wdr27	0	0	0	0	0	0	0	0	0	0	0	0
vstm2l	0	0	0	0	0	0	0	0	0	0	0	0
mycbpap	0	0	0	0	0	0	0	0	0	0	0	0
nell2	0	0	0	0	0	0	0	0	0	0	0	0
kdm2a	0	0	0.12	0	0	0.12	0	0	0	0	0	0

prepl	0	0	0	0	0	0	0	0	0	0	0	0
ctdnep1	0	0	0	0	0	0	0	0	0	0	0.119	0.117
gpr65	0	0	0	0	0	0	0	0	0	0	0	0
map4k2	0	0	0	0	0	0	0	0	0	0	0.113	0.112
ms4a7	0	0	0	0	0	0	0	0	0	0	0	0
lrch3	0	0	0	0	0	0	0	0	0	0	0	0
ywhaq	0	0	0	0	0	0	0	0	0	0	0	0
synm	0	0	0	0	0	0	0	0	0	0	0	0
wash6p	0	0	0	0	0	0	0	0	0	0	0.11	0.11
slc50a1	0	0	0	0	0	0	0	0	0	0	0	0
trim3	0	0	0	0	0	0	0	0	0	0	0	0
rxfp3	0	0	0	0	0	0	0	0	0	0	0	0
itgbl1	0	0	0	0	0	0	0	0	0	0	0.108	0.107
plagl1	0	0	0	0	0	0	0	0	0	0	0	0
edc4	0	0	0	0	0	0	0	0	0	0	0	0
rest	0	0	0	0	0	0	0	0	0	0	0	0
tsks	0	0	0	0	0	0	0	0	0	0	0	0
fgd1	0	0	0	0	0	0	0	0	0	0	0	0
ulk2	0	0	0	0	0	0	0	0	0	0	0	0
megf10	0	0	0.105	0	0	0.105	0	0	0	0	0	0
vangl2	0	0	0.105	0	0	0.105	0	0	0	0	0	0
atad3b	0	0	0.104	0	0	0.104	0	0	0	0	0	0
tanc1	0	0	0	0	0	0	0	0	0	0	0.103	0.103
ribc2	0	0	0	0	0	0	0	0	0	0	0.103	0.102
ier3	0	0	0	0	0	0	0	0	0	0	0	0
apod	0	0	0	0	0	0	0	0	0	0	0	0
limd1	0	0	0	0	0	0	0	0	0	0	0.101	0.1
mknk2	0.1	0	0	0	0	0	0.1	0	0	0	0	0
