# Supplementary material for: The RXFP3 receptor is functionally associated with cellular responses to oxidative stress and DNA damage
Source: Aging (Albany NY). 2019 Dec 3;11(23):11268–313. doi: 10.18632/aging.102528 (PMC6932917; doi:10.18632/aging.102528)
Supplement: Supplementary Table 10 [file aging-11-102528-s013..pdf]

Table S10. Canonical Signaling Pathway Analysis of theoretical GIT2-Signaling dataset. Ingenuity Pathway Analysis was employed to generate a Canonical Signaling Pathway appreciation of the molecular nature of the theoretical GIT2-Signaling dataset created using latent semantic association analysis.

Canonical Signaling Pathways	-log(p-value)	Ratio	Proteins
Signal Transduction			
14-3-3-mediated Signaling	1.42	0.0634	YWHAQ,RAP2A,TUBA8,YWHAB,EDC3,TUBA4A,ELK1,H2BFM,PRKD1
Ephrin Receptor Signaling	4.18	0.0944	RAP2A,RAPGEF1,RGS3,PAK6,CRKL,ARPC5,SH2D3C,GNG13,GNG3,RAC3,GNG10,EPHB6,SORBS1,GNB2,DOK1,GNG5,MAP4K4
HGF Signaling	1.35	0.0645	RAPGEF1,MAP3K9,RAP2A,CRKL,ELK1,MAP3K3,ELK3,PRKD1
Androgen Signaling	1.52	0.0662	POLR2G,TGFB1I1,CALML5,GNB2,GNG13,GNG3,GNG5,PRKD1,GNG10
Tec Kinase Signaling	2.72	0.0778	RND1,PAK6,GNG13,BMX,GNG3,RAC3,GNG10,TEC,VAV3,GNB2,GNG5,STAT5B,PRKD1,FNBP1
Ephrin B Signaling	3.35	0.125	EPHB6,RGS3,VAV3,GNB2,GNG13,GNG3,GNG5,RAC3,GNG10
HIPPO signaling	3.44	0.118	YWHAQ,STK4,AJUBA,YWHAB,NF2,PPP1CB,PPP2R5C,STK3,PPP1CA,PPP2R1B
Phospholipase C Signaling	2.81	0.07	RAP2A,CALML5,RND1,MYL5,GNG13,PPP1CB,PLD6,GNG3,RAC3,GNG10,MARCKS,ARHGEF16,GNB2,GNG5,ADCY7,MYL12B,PRKD1,FNBP1
Protein Kinase A Signaling	2.63	0.06	CALML5,PDE7A,YWHAB,MYL5,GNG13,PPP1CB,AKAP6,PYGL,PLD6,GNG3,PTPN3,H2BFM,GNG10,YWHAQ,PYGM,FLNC,GNB2,DUSP11,PPP1CA,ELK1,GNG5,ADCY7,MYL12B,PRKD1
ERK/MAPK Signaling	2.16	0.0673	RAP2A,RAPGEF1,PAK6,YWHAB,CRKL,PPP1CB,MKNK2,RAC3,YWHAQ,PPP2R5C,PPP1CA,ELK1,PPP2R1B,ELK3
Cytoskeletal Activity			
Integrin Signaling	5.9	0.1	CAPN5,RAP2A,RAPGEF1,RND1,PAK6,ARF1,ASAP1,ACTN2,CRKL,MYL5,ARPC5,PIKFYVE,PPP1CB,BCAR3,RAC3,ARHGAP5,ARF3,ARF4,ZYX,ACTN4,NEDD9,MYL12B,FNBP1
RhoGDI Signaling	5.89	0.111	RND1,PAK6,MYL5,ARPC5,PIKFYVE,GNG13,GNG3,RAC3,ARHGDIB,GNG10,ARHGAP5,DGKZ,CDH12,ARHGEF16,GNB2,GNG5,DLC1,MYL12B,FNBP1,ARHGAP8/PRR5-ARHGAP8
Signaling by Rho Family GTPases	2.77	0.0695	MAP3K9,RND1,PAK6,MYL5,ARPC5,PIKFYVE,GNG13,GNG3,RAC3,CLIP1,GNG10,CDH12,ARHGEF16,GNB2,ELK1,GNG5,MYL12B,FNBP1
Regulation of Actin-based Motility by Rho	2.52	0.0957	RND1,PAK6,ARPC5,MYL5,PIKFYVE,PPP1CB,RAC3,MYL12B,FNBP1
Actin Cytoskeleton Signaling	1.43	0.0556	RAP2A,ABI2,PAK6,VAV3,ACTN2,CRKL,MYL5,ARPC5,PPP1CB,FGD1,ACTN4,RAC3,MYL12B
RhoA Signaling	1.77	0.0732	ARHGAP5,LPAR2,ARPC5,MYL5,PIKFYVE,PPP1CB,DLC1,MYL12B,ARHGAP8/PRR5-ARHGAP8
ILK Signaling	1.84	0.0634	RND1,FBLIM1,ACTN2,MYL5,RAC3,H2BFM,TGFB1I1,FLNC,PPP2R5C,ACTN4,PPP2R1B,DSP,FNBP1
Cell-Cell Communication			
Remodeling of Epithelial Adherens Junctions	5.79	0.174	TUBA8,MAPRE1,ACTN2,ARPC5,TUBA4A,ZYX,DNM3,RAB5B,ACTN4,CLIP1,DNM2,MAPRE3
Epithelial Adherens Junction Signaling	3.41	0.0915	RAP2A,RAPGEF1,ACTN2,ARPC5,MYL5,TUBA4A,CLIP1,TUBA8,SORBS1,SSX2IP,ZYX,ACTN4,CLINT1,FARP2
Germ Cell-Sertoli Cell Junction Signaling	2.98	0.0798	RAP2A,MAP3K9,RND1,PAK6,ACTN2,TUBA4A,RAC3,TUBA8,SORBS1,ZYX,ACTN4,CLINT1,MAP3K3,RAB8B,FNBP1
Sertoli Cell-Sertoli Cell Junction Signaling	1.79	0.0645	MAP3K9,RAP2A,TUBA8,SORBS1,ACTN2,TUBA4A,ACTN4,CLINT1,ELK1,MAP3K3,H2BFM,RAB8B
GPCR Signaling			
α-Adrenergic Signaling	3.67	0.116	RAP2A,CALML5,PYGM,GNB2,GNG13,PYGL,GNG3,GNG5,ADCY7,PRKD1,GNG10
Cardiac β-adrenergic Signaling	3.27	0.0929	PDE7A,PPP1CB,GNG13,AKAP6,GNG3,PLD6,GNG10,GNB2,PPP2R5C,PPP1CA,GNG5,PPP2R1B,ADCY7
CXCR4 Signaling	3.1	0.082	RAP2A,RND1,PAK6,MYL5,GNG13,GNG3,RAC3,GNG10,GNB2,ELK1,GNG5,ADCY7,PRKD1,MYL12B,FNBP1

Dopamine Receptor Signaling	1.95	0.0909	SMOX,NCS1,PPP1CB,PPP2R5C,PPP1CA,ADCY7,PPP2R1B
Relaxin Signaling	1.44	0.0613	PDE7A,RLN2,GNB2,GNG13,GNG3,PLD6,GNG5,ELK1,ADCY7,GNG10
Immune Function			
Leukocyte Extravasation Signaling	1.39	0.0563	ARHGAP5,TEC,RAP1GAP,CRKL,ACTN2,VAV3,SIPA1,BMX,ACTN4,DLC1,PRKD1,ARHGAP8/PRR5-ARHGAP8
CCR5 Signaling in Macrophages	1.52	0.0745	CALML5,GNB2,GNG13,GNG3,GNG5,PRKD1,GNG10
CCR3 Signaling in Eosinophils	1.85	0.0714	RAP2A,CALML5,PAK6,GNB2,PPP1CB,GNG13,GNG3,GNG5,PRKD1,GNG10
fMLP Signaling in Neutrophils	2.02	0.0758	RAP2A,CALML5,ARPC5,GNB2,GNG13,GNG3,GNG5,ELK1,PRKD1,GNG10
Thrombin Signaling	2.66	0.0717	RAP2A,RND1,MYL5,GNG13,PPP1CB,GNG3,RAC3,GNG10,ARHGEF16,GNB2,ELK1,GNG5,ADCY7,PRKD1,MYL12B,FNBP1
IL-8 Signaling	2.04	0.0651	RAP2A,RND1,GNG13,GNG3,PLD6,RAC3,GNG10,RAB11FIP2,GNB2,GNG5,MAP4K4,PRKD1,MYL12B,FNBP1
Stress Response			
SAPK/JNK Signaling	2.95	0.0957	MAP4K2,MAP3K9,RAP2A,CRKL,MAP4K1,MAP4K5,GNG5,ELK1,MAP4K4,RAC3,MAP3K3
Sirtuin Signaling Pathway	2.57	0.0651	NDUFAF1,ATP5MC1,NDUFA7,TIMM10,GLS,TUBA4A,GLUD1,ACLY,ACADL,TUBA4B,TUBA8,NDUFA6,ACSS2,NDUFA12,MLYCD,MAPK7,NDUFS3,CPS1,GLUD2
Cleavage and Polyadenylation of Pre-mRNA	2.24	0.25	CPSF6,NUDT21,CSTF2
Vitamin-C Transport	1.88	0.188	TXNDC2,SLC23A2,SLC23A1
Phagosome Maturation	1.33	0.0608	DYNC1LI1,ATP6V1C2,TUBA8,TUBA4B,DYNC1LI2,TUBA4A,PIKFYVE,RAB5B,EEA1
Disease			
Huntington's Disease Signaling	1.5	0.0556	CAPN5,ATP5MC1,REST,GLS,DNM3,GNG13,GNG3,GNG10,POLR2G,CLTCL1,GNB2,GNG5,PRKD1,DNM2
Cardiac Hypertrophy Signaling	2.48	0.0669	RAP2A,MAP3K9,CALML5,EIF2B4,RND1,MYL5,GNG13,GNG3,RAC3,GNG10,GNB2,ELK1,GNG5,ADCY7,MAP3K3,MYL12B,FNBP1
Polyamine Regulation in Colon Cancer	3.27	0.227	AZIN1,SAT1,OAZ1,OAZ2,ODC1
Breast Cancer Regulation by Stathmin1	2.83	0.0744	RAP2A,CALML5,TUBA4A,GNG13,PPP1CB,GNG3,GNG10,TUBA8,ARHGEF16,GNB2,PPP2R5C,GNG5,PPP1CA,ADCY7,PPP2R1B,PRKD1
Endocytosis Mechanisms			
Clathrin-mediated Endocytosis Signaling	1.46	0.0577	SH3GL1,AP1S1,AP2A1,SNX9,EPS15,ARPC5,CLTCL1,DNM3,SH3GL2,RAB5B,DNM2,APOD
Caveolar-mediated Endocytosis Signaling	2.74	0.113	COPZ1,ARCN1,FLNC,COPA,COPE,RAB5B,DNM2,COPG1
Virus Entry via Endocytic Pathways	1.4	0.0661	AP1S1,RAP2A,AP2A1,FLNC,CLTCL1,RAC3,DNM2,PRKD1
Cell Cycle Control			
Cell Cycle Regulation by BTG Family Proteins	1.53	0.108	BTG2,BTG1,PPP2R5C,PPP2R1B
Energy Metabolism			
Mitochondrial Dysfunction	1.33	0.0585	NDUFAF1,ATP5MC1,COX4I2,NDUFA7,NDUFA6,XDH,NDUFA12,NDUFS3,OGDH,UQCRQ
