# Supplementary material for: The RXFP3 receptor is functionally associated with cellular responses to oxidative stress and DNA damage
Source: Aging (Albany NY). 2019 Dec 3;11(23):11268–313. doi: 10.18632/aging.102528 (PMC6932917; doi:10.18632/aging.102528)
Supplement: Supplementary Table 11 [file aging-11-102528-s011..pdf]

Table S11. RXFP3 interacting proteins. Interacting proteins of RXFP3 in control conditions, after oxidative stress (H_2O_2) and DNA damage (CPT). The list termed 'stress' are all the proteins of oxidative stress and DNA damage combined, without duplicates.

Control	Oxidative Stress	DNA damage	Stress
RXFP3	MYL12A	RPS2	MYL12A
RPS27A	TPM4	RPS9	TPM4
RPS14	PTPRF	PHB	PTPRF
PSMB6	TPM1	RPS27A	TPM1
RPS11	DCAKD	CCT4	DCAKD
RPL10	NUDT5	HSPA8	NUDT5
RPS3	MYH9	CANX	MYH9
DNAJA1	STOM	RPL22	STOM
RPS27	CAPZA1	PSMD14	CAPZA1
RPS15A	OTUB1	RPL7A	OTUB1
RPL23	FLNC	RAN	FLNC
RPS9	AMFR	PSMB6	AMFR
RPL21	NEFM	RPL13A	NEFM
RPL11	ACTN4	RPL9	ACTN4
RPS26	NUP210	SSR4	NUP210
RPS4X	SNRPE	HSPA5	SNRPE
RPL18A	CAPZB	PSMB4	CAPZB
EIF4A1	ATP1A1	RPL21	ATP1A1
RPL27A	PRDX6	PSMA6	PRDX6
CCT3	IRS4	RPL24	IRS4
HNRNPA2B1	DYNLL1	PABPC1	DYNLL1
GNB2L1	ACTB	NDUFS3	ACTB
RPL13	ARPC5L	HSPA1A	ARPC5L
PSMA5	SNRPD3	PSMC4	SNRPD3
HSPA8	BAG6	RPL8	BAG6
TCP1	PCNA	RPS4X	PCNA
EIF3F	HSPA5	RPL11	HSPA5

RPS13	SNRPD2	RPS16	SNRPD2
YTHDF2	HSPB1	NCLN	HSPB1
PHB	FUS	PSMB1	FUS
RPL7A	RPLP2	GNB2L1	RPLP2
RPL4	RPL31	PSMA5	RPL31
MAP4	EEF1A1	PHB2	EEF1A1
RPL14	LMAN1	ERLIN2	LMAN1
RPL15	MYL6	ATP5A1	MYL6
RPL34	ERLIN1	RPS3	ERLIN1
RPL6	MYH10	HNRNPH3	MYH10
RPL27	HNRNPH1	RPS8	HNRNPH1
RPL7	H1FX	SSR3	H1FX
RPL18	SNRPD1	CDK1	SNRPD1
RPLP0	FAU	RPS6	FAU
RPS27L	GAPDH	PPA1	GAPDH
RPL13A	CDKN2A	RPS14	CDKN2A
RPL8	RPL17-C18orf32	EIF4A1	RPL17-C18orf32
RPL29	SNRPB	PSMA7	SNRPB
MYL6	EWSR1	NDUFA2	EWSR1
RPL10A	HSPA9	NDUFV2	HSPA9
	HIST1H1D	H1FX	HIST1H1D
	EEF1D	PABPC4	EEF1D
	HSPA8	RPS13	HSPA8
	EEF2	DARS	EEF2
	RPL27A	TRAP1	RPL27A
	CFL1	VIM	CFL1
	RPS20	RPN1	RPS20
	RPL28	SERPINH1	RPL28
	TUBA1C	UBXN1	TUBA1C
	RPL18A	RPL23	RPL18A
	VIM	AIFM1	VIM
	TUBB	RPS27L	TUBB

	RPS12	RPL15	RPS12
	HNRNPA2B1	TUBB	HNRNPA2B1
	HSP90AA1	RPS24	HSP90AA1
	CANX	NDUFA5	CANX
	RPL27	DNAJA2	RPL27
	RPL22	SCD	RPL22
	TUBB4B	NDUFA12	TUBB4B
	GNB2L1	HSPA9	GNB2L1
	YWHAZ	TUBB4B	YWHAZ
	EIF4A1	HNRNPR	EIF4A1
	TFG	PSMC5	TFG
	RPS27A	PSMC2	RPS27A
	NACA	C1QBP	NACA
	PHGDH	PPT1	PHGDH
	RPS15A	DNAJA1	RPS15A
	HSP90AB1	RPS11	HSP90AB1
	CCT3	PKM	CCT3
	NDUFS6	RARS	NDUFS6
	RXFP3	RPL18A	RXFP3
	HNRNPAB	MCM4	HNRNPAB
	ATP5B	CFL1	ATP5B
	KHSRP	RBM4	KHSRP
	TMEM263	PSMB2	TMEM263
	RPL34	ARF4	RPL34
	HSPA1A	RPL4	HSPA1A
	RPL24	PSMC3	RPL24
	RPL7A	ARF3	RPL7A
	ILF2	HNRNPH1	ILF2
	NPM1	POLR1C	NPM1
	ATP5A1	FAM98B	ATP5A1
	NHP2L1	TCP1	NHP2L1
	RPS3	DDX47	RPS3

	RPL11	IRS4	RPL11
	RPL13	CCT3	RPL13
	TCP1	NDUFS1	TCP1
	PSMD2	PYGL	PSMD2
	ILF3	RBFOX1	ILF3
	RBMX	DDX19B	RBMX
	RPS24	PSMD3	RPS24
	NDUFV2	RPL10	NDUFV2
	RPS2	IDH3B	RPS2
	ENO1	POLR2E	ENO1
	PABPC1	BAG6	PABPC1
	NCL	PHGDH	NCL
	RPL8	EIF3F	RPL8
	YWHAQ	HAX1	YWHAQ
	RPL4	AIMP1	RPL4
	HSPE1-MOB4	NCL	HSPE1-MOB4
	YBX3	RPL18	YBX3
	RPS8	PSMA1	RPS8
	YBX1	VDAC2	YBX1
	RPL13A	RTCB	RPL13A
	PRDX1	MRPS22	PRDX1
	FLOT1	WARS	FLOT1
	LDHB	HACD3	LDHB
	SOD1	RPS20	SOD1
	KRT8	KPNA2	KRT8
	SSBP1	HSPB1	SSBP1
	RPL15	GANAB	RPL15
	RPS27L	RPS7	RPS27L
	RPL23A	DNAJB6	RPL23A
	HNRNPC	STOM	HNRNPC
	HNRNPR	SLC25A11	HNRNPR
	RPS6	GTPBP4	RPS6

	RPL6	PCBP1	RPL6
	MMTAG2	TRMT112	MMTAG2
	BAG2	DDX5	BAG2
	RPL21	TOMM22	RPL21
	PSMC2	CDKN2A	PSMC2
	PRMT5	NEFM	PRMT5
	RPL18	RPL19	RPL18
	PARP1	AP3S1	PARP1
	HIST1H2BN	TUFM	HIST1H2BN
	HIST1H2AG	ATP2A2	HIST1H2AG
	PSMA7	UQCRC2	PSMA7
	H3F3B	HSP90AB1	H3F3B
	PSMA1	EMD	PSMA1
	HIST1H2AB	SSBP1	HIST1H2AB
	PSMA6	MAT2A	PSMA6
	PSMA5	SLC25A6	PSMA5
	PSMB4	DDX6	PSMB4
	HIST1H4A	SMC3	HIST1H4A
	PSMB6	HNRNPUL1	PSMB6
	PSMA3	NPM1	PSMA3
	PSMB5	RBM14	PSMB5
	RPL7	CCT6A	RPL7
		LMNA	RPS9
		CAPRIN1	PHB
		EEF1A1	CCT4
		RPL26	PSMD14
		RPL5	RAN
		DYNLRB1	RPL9
		RPS26	SSR4
		IGF2BP1	NDUFS3
		RPL31	PSMC4
		ENO1	RPS4X

		BAG2	RPS16
		RXFP3	NCLN
		DSG2	PSMB1
		ACP1	PHB2
		ARL1	ERLIN2
		ARL2	HNRNPH3
		ATP6AP1	SSR3
		DYNC1LI1	CDK1
		EMC3	PPA1
		GET4	RPS14
		HLA-C	NDUFA2
		MAGED1	PABPC4
		NEFL	RPS13
		NSF	DARS
		NTPCR	TRAP1
		STT3B	RPN1
		G3BP1	SERPINH1
		NONO	UBXN1
		OAT	RPL23
		ATP5D	AIFM1
		RPL7	NDUFA5
		NHP2L1	DNAJA2
		HNRNPA2B1	SCD
		EEF1G	NDUFA12
		ELAVL1	PSMC5
		ATP5B	C1QBP
		FAM98A	PPT1
		HNRNPAB	DNAJA1
		SNRPA1	RPS11
		HNRNPF	PKM
		DDX17	RARS
		RPL12	MCM4

		HNRNPK	RBM4
		NACA	PSMB2
		HSP90AA1	ARF4
		HIST1H1D	PSMC3
		RPLP2	ARF3
		RPS28	POLR1C
			FAM98B
			DDX47
			NDUFS1
			PYGL
			RBFOX1
			DDX19B
			PSMD3
			RPL10
			IDH3B
			POLR2E
			EIF3F
			HAX1
			AIMP1
			VDAC2
			RTCB
			MRPS22
			WARS
			HACD3
			KPNA2
			GANAB
			RPS7
			DNAJB6
			SLC25A11
			GTPBP4
			PCBP1
			TRMT112

			DDX5
			TOMM22
			RPL19
			AP3S1
			TUFM
			ATP2A2
			UQCRC2
			EMD
			MAT2A
			SLC25A6
			DDX6
			SMC3
			HNRNPUL1
			RBM14
			CCT6A
			LMNA
			CAPRIN1
			RPL26
			RPL5
			DYNLRB1
			RPS26
			IGF2BP1
			DSG2
			ACP1
			ARL1
			ARL2
			ATP6AP1
			DYNC1LI1
			EMC3
			GET4
			HLA-C
			MAGED1

			NEFL
			NSF
			NTPCR
			STT3B
			G3BP1
			NONO
			OAT
			ATP5D
			EEF1G
			ELAVL1
			FAM98A
			SNRPA1
			HNRNPF
			DDX17
			RPL12
			HNRNPK
			RPS28
