# Supplementary material for: The RXFP3 receptor is functionally associated with cellular responses to oxidative stress and DNA damage
Source: Aging (Albany NY). 2019 Dec 3;11(23):11268–313. doi: 10.18632/aging.102528 (PMC6932917; doi:10.18632/aging.102528)
Supplement: Supplementary Table 12 [file aging-11-102528-s003..pdf]

Table S12. NetworkAnalyst-based Gene Ontology analysis of the RXFP3 interactome stabilized in the presence of peroxide cellular perturbation (hypothalamic database). The proteins consistently associated with the RXFP3 receptor following exposure to hydrogen peroxide were analyzed using a human hypothalamic tissue database derived from DIFFERENTIALNET (http://netbio.bgu.ac.il/diffnet/). For the most stringent analysis process we employed a Zero Order Network approach. Gene Ontology (Biological Process) annotation was performed on all identified nodes using the built-in Gene Ontology analysis module of NetworkAnalyst (https://www.networkanalyst.ca). For each significantly-populated Gene Ontology term group (p<0.05) the total number of proteins associated with that group (Total), the expected (Expected) number of identified proteins from a random data sample (Hypergeometric test based), the actual number of GO term-populating proteins from the experimental dataset (Hits), the enrichment P value (P.Value) as well as the enrichment FDR are given (FDR).

GO Biological Process Term	Total	Expected	Hits	P.Value	FDR
MRNA metabolic process	817	5.09	37	4.11E-23	3.37E-20
Translational initiation	205	1.28	18	3.88E-16	1.59E-13
Protein targeting to membrane	158	0.984	16	2.04E-15	5.58E-13
Cellular protein complex disassembly	160	0.997	15	5.25E-14	1.08E-11
Protein complex disassembly	167	1.04	15	9.93E-14	1.37E-11
Cellular macromolecule catabolic process	849	5.29	28	9.99E-14	1.37E-11
Viral reproductive process	597	3.72	24	1.28E-13	1.50E-11
Viral reproduction	803	5	27	1.97E-13	2.02E-11
RNA catabolic process	256	1.59	17	2.85E-13	2.60E-11
Cellular component disassembly	310	1.93	18	5.24E-13	4.29E-11
Macromolecular complex disassembly	189	1.18	15	6.16E-13	4.59E-11
Protein targeting	545	3.39	22	1.51E-12	1.03E-10
Translation	698	4.35	24	3.69E-12	2.33E-10
Viral infectious cycle	241	1.5	15	2.07E-11	1.21E-09
Macromolecule catabolic process	1070	6.67	28	2.76E-11	1.42E-09
Macromolecule catabolic process	1070	6.67	28	2.76E-11	1.42E-09
Intracellular protein transport	793	4.94	24	5.36E-11	2.58E-09
Cellular nitrogen compound catabolic process	986	6.14	26	1.45E-10	6.59E-09
Intracellular transport	1510	9.4	31	7.67E-10	3.31E-08
Establishment of protein localization	1460	9.06	29	6.71E-09	2.75E-07
Protein transport	1400	8.73	28	1.27E-08	4.96E-07
Cellular catabolic process	2140	13.4	35	1.90E-08	7.06E-07
Protein folding	241	1.5	12	3.04E-08	1.08E-06
Establishment of localization in cell	2320	14.4	36	3.93E-08	1.34E-06
Catabolic process	2560	16	38	4.70E-08	1.54E-06
Multi_organism process	1710	10.6	30	6.08E-08	1.92E-06
RNA splicing, via transesterification reactions	321	2	13	8.84E-08	2.69E-06
Cellular localization	2650	16.5	37	3.94E-07	1.15E-05

RNA processing	890	5.54	20	4.09E-07	1.16E-05
Ribonucleoprotein complex biogenesis	269	1.68	11	8.56E-07	2.34E-05
Protein localization	1850	11.5	29	1.22E-06	3.23E-05
RNA splicing	478	2.98	14	1.39E-06	3.48E-05
MRNA processing	551	3.43	15	1.40E-06	3.48E-05
Macromolecule localization	2240	14	31	6.90E-06	0.000166
Actin filament_based movement	78	0.486	6	8.83E-06	0.000207
Protein polyubiquitination	177	1.1	8	1.42E-05	0.000324
Cellular protein metabolic process	4540	28.3	47	2.97E-05	0.000659
Reproduction	1860	11.6	26	4.08E-05	0.000873
Reproductive process	1740	10.9	25	4.15E-05	0.000873
Ribosome biogenesis	166	1.03	7	7.80E-05	0.0016
DNA damage response, signal transduction by p53 class mediator	117	0.729	6	8.86E-05	0.00177
Negative regulation of cellular protein metabolic process	463	2.88	11	0.000137	0.00268
Signal transduction in response to DNA damage	129	0.804	6	0.000152	0.00289
Negative regulation of cellular metabolic process	1660	10.4	23	0.000161	0.00301
Negative regulation of metabolic process	1820	11.3	24	0.000235	0.00427
S phase of mitotic cell cycle	144	0.897	6	0.000276	0.00492
G1/S transition of mitotic cell cycle	209	1.3	7	0.000324	0.00565
Interphase of mitotic cell cycle	435	2.71	10	0.000366	0.00625
S phase	153	0.953	6	0.000382	0.0064
Interphase	443	2.76	10	0.000423	0.00693
Negative regulation of protein metabolic process	540	3.36	11	0.000514	0.00826
RRNA processing	109	0.679	5	0.000588	0.00927
RRNA metabolic process	118	0.735	5	0.000843	0.013
Ribonucleoprotein complex assembly	124	0.772	5	0.00105	0.016
Interaction with host	426	2.65	9	0.00132	0.0197
Protein metabolic process	5570	34.7	49	0.00148	0.0216
DNA damage checkpoint	143	0.891	5	0.00198	0.0285
Mitotic cell cycle checkpoint	149	0.928	5	0.00237	0.0335
DNA integrity checkpoint	152	0.947	5	0.00258	0.0359
Cellular component assembly	1790	11.1	21	0.00277	0.0378
Mitochondrial transport	159	0.99	5	0.00314	0.0422
Macromolecular complex assembly	1120	6.99	15	0.00374	0.0494
Cellular protein catabolic process	518	3.23	9	0.0049	0.0637
RNA metabolic process	6010	37.5	50	0.005	0.064
Nucleobase containing compound metabolic process	8180	50.9	63	0.00574	0.0713
Nucleobase containing compound metabolic process	8180	50.9	63	0.00574	0.0713
Positive regulation of catalytic activity	1070	6.66	14	0.00613	0.075
Regulation of mitotic cell cycle	351	2.19	7	0.00626	0.0755
Amine metabolic process	268	1.67	6	0.00653	0.0776

Response to DNA damage stimulus	862	5.37	12	0.00702	0.0823
Response to oxidative stress	279	1.74	6	0.0079	0.0912
Cell cycle checkpoint	281	1.75	6	0.00816	0.093
Chromatin assembly	128	0.797	4	0.00835	0.0938
Protein targeting to mitochondrion	71	0.442	3	0.00984	0.108
Negative regulation of nucleobase_containing compound metabolic process	1130	7.05	14	0.00989	0.108
Cytoskeleton dependent intracellular transport	76	0.473	3	0.0118	0.128
Apoptotic DNA fragmentation	27	0.168	2	0.0122	0.13
Cellular component disassembly involved in execution phase of apoptosis	78	0.486	3	0.0127	0.134
Regulation of apoptotic process	1540	9.56	17	0.013	0.135
Cell cycle process	1420	8.81	16	0.0133	0.136
Heterocycle metabolic process	8430	52.5	63	0.0142	0.143
G2/M transition of mitotic cell cycle	150	0.934	4	0.0143	0.143
Regulation of programmed cell death	1550	9.67	17	0.0144	0.143
Chromatin assembly or disassembly	152	0.947	4	0.015	0.146
Cell cycle phase	1070	6.68	13	0.0152	0.147
Cellular aromatic compound metabolic process	8460	52.7	63	0.0154	0.147
Protein catabolic process	644	4.01	9	0.0187	0.174
Transport	4830	30.1	40	0.0189	0.174
Cell cycle	1860	11.6	19	0.0191	0.174
Activation of cysteine_type endopeptidase activity involved in apoptotic process	91	0.567	3	0.0191	0.174
Apoptotic nuclear changes	35	0.218	2	0.02	0.18
Protein ubiquitination	658	4.1	9	0.0211	0.188
Regulation of cell cycle	886	5.52	11	0.0214	0.189
Cellular response to stress	1620	10.1	17	0.0217	0.189
DNA packaging	172	1.07	4	0.0224	0.194
Negative regulation of RNA metabolic process	1020	6.38	12	0.0246	0.209
Establishment of localization	4910	30.6	40	0.0247	0.209
Negative regulation of catalytic activity	568	3.54	8	0.0251	0.21
Microtubule based movement	179	1.11	4	0.0255	0.211
Muscle cell differentiation	363	2.26	6	0.0257	0.211
Regulation of cell shape	106	0.66	3	0.0285	0.231
Wound healing	700	4.36	9	0.03	0.241
Base excision repair	45	0.28	2	0.0319	0.254
Nucleosome assembly	112	0.698	3	0.0327	0.258
Protein modification by small protein conjugation	713	4.44	9	0.0332	0.259
Negative regulation of cellular process	4110	25.6	34	0.0338	0.261
Aging	197	1.23	4	0.0346	0.265
Positive regulation of hydrolase activity	497	3.1	7	0.0353	0.266

Positive regulation of protein metabolic process	1080	6.75	12	0.0359	0.266
Protein export from nucleus	48	0.299	2	0.036	0.266
Axon guidance	394	2.45	6	0.0363	0.266
Glucose catabolic process	117	0.729	3	0.0366	0.266
Regulation of catalytic activity	1730	10.8	17	0.0373	0.266
Positive regulation of cysteine type endopeptidase activity involved in apoptotic process	118	0.735	3	0.0374	0.266
Mitotic cell cycle	968	6.03	11	0.0376	0.266
Positive regulation of cellular protein metabolic process	968	6.03	11	0.0376	0.266
Proteolysis	1100	6.82	12	0.0386	0.268
Cell division	507	3.16	7	0.0386	0.268
Superoxide metabolic process	52	0.324	2	0.0416	0.284
Microtubule based process	516	3.21	7	0.0418	0.284
