# Supplementary material for: The RXFP3 receptor is functionally associated with cellular responses to oxidative stress and DNA damage
Source: Aging (Albany NY). 2019 Dec 3;11(23):11268–313. doi: 10.18632/aging.102528 (PMC6932917; doi:10.18632/aging.102528)
Supplement: Supplementary Table 13 [file aging-11-102528-s008..pdf]

Table S13. NetworkAnalyst-based Gene Ontology analysis of the RXFP3 interactome stabilized in the presence of CPT cellular perturbation (hypothalamic database). The proteins consistently associated with the RXFP3 receptor following exposure to CPT were analyzed using a human hypothalamic tissue database derived from DIFFERENTIALNET (http://netbio.bgu.ac.il/diffnet/). For the most stringent analysis process we employed a Zero Order Network approach. Gene Ontology (Biological Process) annotation was performed on all identified nodes using the built-in Gene Ontology analysis module of NetworkAnalyst (https://www.networkanalyst.ca). For each significantly-populated Gene Ontology term group ($p<0.05$) the total number of proteins associated with that group (Total), the expected (Expected) number of identified proteins from a random data sample (Hypergeometric test based), the actual number of GO term-populating proteins from the experimental dataset (Hits), the enrichment P value (P.Value) as well as the enrichment FDR are given (FDR).

GO Biological Process Term	Total	Expected	Hits	P.Value	FDR
MRNA metabolic process	817	6.18	49	2.02E-32	1.66E-29
Viral reproduction	803	6.07	45	3.20E-28	1.31E-25
Cellular macromolecule catabolic process	849	6.42	44	4.00E-26	1.09E-23
Viral reproductive process	597	4.51	38	1.72E-25	3.53E-23
Macromolecule catabolic process	1070	8.1	44	4.92E-22	6.73E-20
Macromolecule catabolic process	1070	8.1	44	4.92E-22	6.73E-20
Translational initiation	205	1.55	23	7.27E-21	8.52E-19
Viral infectious cycle	241	1.82	23	2.97E-19	3.05E-17
Cellular component disassembly	310	2.34	24	6.08E-18	5.23E-16
Protein targeting to membrane	158	1.19	19	6.38E-18	5.23E-16
Cellular protein complex disassembly	160	1.21	19	8.13E-18	6.06E-16
Protein complex disassembly	167	1.26	19	1.85E-17	1.18E-15
RNA catabolic process	256	1.94	22	1.87E-17	1.18E-15
Multi organism process	1710	12.9	48	2.53E-17	1.48E-15
Macromolecular complex disassembly	189	1.43	19	1.93E-16	1.06E-14
Translation	698	5.28	31	3.71E-16	1.90E-14
Protein targeting	545	4.12	27	3.10E-15	1.50E-13
DNA damage response, signal transduction by p53 class mediator	117	0.884	15	9.22E-15	4.20E-13
Signal transduction in response to DNA damage	129	0.975	15	4.06E-14	1.75E-12
Intracellular protein transport	793	5.99	30	8.78E-14	3.60E-12
Cellular nitrogen compound catabolic process	986	7.45	33	1.17E-13	4.57E-12
Cellular catabolic process	2140	16.2	48	2.11E-13	7.87E-12
Reproductive process	1740	13.2	42	1.35E-12	4.81E-11
Reproduction	1860	14	43	2.34E-12	7.99E-11
DNA damage checkpoint	143	1.08	14	3.32E-12	1.09E-10
S phase of mitotic cell cycle	144	1.09	14	3.66E-12	1.15E-10
G1/S transition of mitotic cell cycle	209	1.58	16	3.85E-12	1.17E-10
Mitotic cell cycle checkpoint	149	1.13	14	5.85E-12	1.71E-10

DNA integrity checkpoint	152	1.15	14	7.69E-12	2.17E-10
S phase	153	1.16	14	8.41E-12	2.30E-10
RNA processing	890	6.73	29	1.03E-11	2.72E-10
Cellular protein metabolic process	4540	34.3	68	2.32E-11	5.95E-10
Catabolic process	2560	19.4	49	4.07E-11	1.01E-09
Protein polyubiquitination	177	1.34	14	6.07E-11	1.46E-09
Interphase of mitotic cell cycle	435	3.29	20	7.55E-11	1.77E-09
Interphase	443	3.35	20	1.04E-10	2.38E-09
Establishment of protein localization	1460	11	35	2.22E-10	4.92E-09
Cell cycle checkpoint	281	2.12	16	3.29E-10	7.09E-09
Ribosome biogenesis	166	1.25	13	3.47E-10	7.19E-09
Protein transport	1400	10.6	34	3.51E-10	7.19E-09
RRNA processing	109	0.824	11	5.67E-10	1.13E-08
Intracellular transport	1510	11.4	35	6.00E-10	1.17E-08
RRNA metabolic process	118	0.892	11	1.34E-09	2.55E-08
Negative regulation of cellular protein metabolic process	463	3.5	19	1.60E-09	2.94E-08
Cellular protein catabolic process	518	3.92	20	1.62E-09	2.94E-08
Interaction with host	426	3.22	18	2.87E-09	5.11E-08
Protein localization	1850	14	37	9.52E-09	1.66E-07
Regulation of apoptotic process	1540	11.6	33	1.50E-08	2.55E-07
Negative regulation of protein metabolic process	540	4.08	19	1.97E-08	3.27E-07
Regulation of programmed cell death	1550	11.7	33	1.99E-08	3.27E-07
Protein folding	241	1.82	13	3.23E-08	5.19E-07
Positive regulation of cellular protein metabolic process	968	7.32	25	4.16E-08	6.55E-07
Macromolecule localization	2240	16.9	40	4.99E-08	7.73E-07
Regulation of mitotic cell cycle	351	2.65	15	5.87E-08	8.92E-07
Protein catabolic process	644	4.87	20	6.30E-08	9.40E-07
Positive regulation of protein metabolic process	1080	8.19	26	9.09E-08	1.33E-06
Amine metabolic process	268	2.03	13	1.12E-07	1.61E-06
Ribonucleoprotein complex biogenesis	269	2.03	13	1.17E-07	1.65E-06
Cell cycle arrest	428	3.24	16	1.29E-07	1.78E-06
Protein metabolic process	5570	42.1	69	1.30E-07	1.78E-06
Negative regulation of cellular metabolic process	1660	12.6	32	3.60E-07	4.84E-06
Regulation of cellular protein metabolic process	1560	11.8	30	9.26E-07	1.22E-05
Establishment of localization in cell	2320	17.5	38	1.16E-06	1.49E-05
Negative regulation of catalytic activity	568	4.29	17	1.16E-06	1.49E-05
Response to DNA damage stimulus	862	6.52	21	1.59E-06	2.00E-05
Cellular localization	2650	20.1	41	1.68E-06	2.09E-05
Negative regulation of cell cycle	520	3.93	16	1.74E-06	2.10E-05
Positive regulation of protein modification process	867	6.55	21	1.74E-06	2.10E-05

Mitotic cell cycle	968	7.32	22	2.70E-06	3.16E-05
Negative regulation of metabolic process	1820	13.8	32	2.70E-06	3.16E-05
RNA splicing	478	3.61	15	2.97E-06	3.43E-05
Apoptotic process	2130	16.1	35	3.53E-06	3.96E-05
Apoptotic process	2130	16.1	35	3.53E-06	3.96E-05
Positive regulation of catalytic activity	1070	8.08	23	3.86E-06	4.28E-05
Cell cycle phase	1070	8.11	23	4.11E-06	4.50E-05
Programmed cell death	2160	16.3	35	4.71E-06	5.08E-05
RNA splicing, via transesterification reactions	321	2.43	12	5.45E-06	5.81E-05
Regulation of protein metabolic process	1820	13.8	31	8.21E-06	8.63E-05
Regulation of cell cycle	886	6.7	20	9.19E-06	9.54E-05
MRNA processing	551	4.16	15	1.64E-05	0.000168
Protein ubiquitination	658	4.97	16	3.34E-05	0.000338
Regulation of translation	228	1.72	9	5.70E-05	0.00057
Proteolysis	1100	8.28	21	6.09E-05	0.000602
Regulation of catalytic activity	1730	13.1	28	6.37E-05	0.000621
Protein modification by small protein conjugation	713	5.39	16	8.67E-05	0.000836
Cell cycle process	1420	10.7	24	0.000117	0.00111
Regulation of protein modification process	1250	9.43	22	0.000137	0.00129
Cellular amino acid metabolic process	670	5.06	15	0.000151	0.00139
Cellular amino acid metabolic process	670	5.06	15	0.000151	0.00139
Nucleocytoplasmic transport	388	2.93	11	0.000168	0.00153
Nuclear transport	392	2.96	11	0.000183	0.00165
Intracellular steroid hormone receptor signaling pathway	113	0.854	6	0.000213	0.0019
RNA metabolic process	6010	45.5	64	0.000229	0.00202
Cellular component disassembly involved in execution phase of apoptosis	78	0.59	5	0.000306	0.00267
Positive regulation of cellular metabolic process	2530	19.1	34	0.000336	0.0029
Cellular response to stress	1620	12.3	25	0.000377	0.00322
Nucleobase containing compound metabolic process	8180	61.8	79	0.000428	0.00358
Nucleobase containing compound metabolic process	8180	61.8	79	0.000428	0.00358
Protein export from nucleus	48	0.363	4	0.000465	0.00385
Positive regulation of translation	56	0.423	4	0.000838	0.00687
Intracellular receptor mediated signaling pathway	270	2.04	8	0.00101	0.00821
Apoptotic DNA fragmentation	27	0.204	3	0.00108	0.00865
Positive regulation of metabolic process	2690	20.3	34	0.00109	0.00868
Cell cycle	1860	14.1	26	0.00121	0.00954
Heterocycle metabolic process	8430	63.7	79	0.00151	0.0118
Androgen receptor signaling pathway	67	0.506	4	0.00164	0.0127
Cellular aromatic compound metabolic process	8460	63.9	79	0.00169	0.013
Regulation of molecular function	2250	17	29	0.00209	0.0159

Apoptotic nuclear changes	35	0.265	3	0.00231	0.0173
Negative regulation of cellular process	4110	31	45	0.00264	0.0197
Intrinsic apoptotic signaling pathway	135	1.02	5	0.00358	0.0265
Negative regulation of growth	197	1.49	6	0.00381	0.0279
Nuclear export	139	1.05	5	0.00406	0.0294
Protein import	272	2.06	7	0.00457	0.0328
Activation of cysteine type endopeptidase activity involved in apoptotic process	91	0.688	4	0.00499	0.0355
G2/M transition of mitotic cell cycle	150	1.13	5	0.00559	0.0395
Mitochondrial transport	159	1.2	5	0.00713	0.0499
Protein import into nucleus	228	1.72	6	0.00767	0.0533
Nuclear import	232	1.75	6	0.00832	0.0573
Mitochondrion organization	306	2.31	7	0.00854	0.0583
