# Supplementary material for: The RXFP3 receptor is functionally associated with cellular responses to oxidative stress and DNA damage
Source: Aging (Albany NY). 2019 Dec 3;11(23):11268–313. doi: 10.18632/aging.102528 (PMC6932917; doi:10.18632/aging.102528)
Supplement: Supplementary Table 14 [file aging-11-102528-s022..pdf]

Table S14. NetworkAnalyst-based Gene Ontology analysis of the RXFP3 interactome stabilized in the presence of peroxide cellular perturbation (generic database). The proteins consistently associated with the RXFP3 receptor following exposure to hydrogen peroxide were analyzed using a generic human tissue database derived from IMEx (https://www.imexconsortium.org/). For the most stringent analysis process we employed a Zero Order Network approach. Gene Ontology (Biological Process) annotation was performed on all identified nodes using the built-in Gene Ontology analysis module of NetworkAnalyst (https://www.networkanalyst.ca). For each significantly-populated Gene Ontology term group ($p<0.05$) the total number of proteins associated with that group (Total), the expected (Expected) number of identified proteins from a random data sample (Hypergeometric test based), the actual number of GO term-populating proteins from the experimental dataset (Hits), the enrichment P value (P.Value) as well as the enrichment FDR are given (FDR).

GO Biological Process Term	Total	Expected	Hits	P.Value	FDR
MRNA metabolic process	817	7.09	61	8.99E-43	7.37E-40
Cellular protein complex disassembly	160	1.39	34	1.75E-38	7.19E-36
Protein complex disassembly	167	1.45	34	8.54E-38	2.34E-35
Macromolecular complex disassembly	189	1.64	34	7.84E-36	1.61E-33
Protein targeting to membrane	158	1.37	32	1.85E-35	3.03E-33
Translational initiation	205	1.78	34	1.46E-34	2.00E-32
Cellular component disassembly	310	2.69	37	3.14E-32	3.68E-30
Cellular macromolecule catabolic process	849	7.37	51	2.43E-30	2.49E-28
RNA catabolic process	256	2.22	33	8.70E-30	7.93E-28
Viral reproductive process	597	5.18	44	1.58E-29	1.30E-27
Viral reproduction	803	6.97	48	2.78E-28	2.08E-26
Viral infectious cycle	241	2.09	31	6.23E-28	4.26E-26
Macromolecule catabolic process	1070	9.29	51	1.42E-25	8.33E-24
Macromolecule catabolic process	1070	9.29	51	1.42E-25	8.33E-24
Protein targeting	545	4.73	38	1.98E-24	1.08E-22
Translation	698	6.06	40	1.40E-22	7.17E-21
Intracellular protein transport	793	6.88	40	1.49E-20	7.19E-19
Cellular nitrogen compound catabolic process	986	8.56	43	9.43E-20	4.30E-18
Multi organism process	1710	14.8	52	2.58E-17	1.11E-15
Cellular catabolic process	2140	18.6	58	3.57E-17	1.47E-15
Intracellular transport	1510	13.1	47	7.80E-16	3.04E-14
Catabolic process	2560	22.3	61	1.61E-15	6.01E-14
Establishment of protein localization	1460	12.6	44	2.81E-14	1.00E-12
Protein transport	1400	12.2	43	3.71E-14	1.27E-12
Reproductive process	1740	15.1	46	8.60E-13	2.82E-11
Reproduction	1860	16.1	47	1.87E-12	5.89E-11
Establishment of localization in cell	2320	20.1	53	2.00E-12	6.09E-11
Protein localization	1850	16	46	6.66E-12	1.95E-10

Cellular localization	2650	23	56	8.78E-12	2.48E-10
DNA damage response, signal transduction by p53 class mediator	117	1.02	12	3.78E-10	1.03E-08
Protein polyubiquitination	177	1.54	14	3.98E-10	1.05E-08
Macromolecule localization	2240	19.5	48	4.16E-10	1.07E-08
Signal transduction in response to DNA damage	129	1.12	12	1.18E-09	2.94E-08
Cellular protein metabolic process	4540	39.4	72	1.24E-09	2.98E-08
Ribonucleoprotein complex biogenesis	269	2.33	16	1.41E-09	3.30E-08
G1/S transition of mitotic cell cycle	209	1.81	14	3.51E-09	7.99E-08
DNA damage checkpoint	143	1.24	12	3.88E-09	8.59E-08
S phase of mitotic cell cycle	144	1.25	12	4.20E-09	9.06E-08
Mitotic cell cycle checkpoint	149	1.29	12	6.20E-09	1.30E-07
DNA integrity checkpoint	152	1.32	12	7.77E-09	1.59E-07
S phase	153	1.33	12	8.37E-09	1.67E-07
Negative regulation of cellular protein metabolic process	463	4.02	19	1.72E-08	3.36E-07
RNA splicing, via transesterification reactions	321	2.79	16	1.76E-08	3.36E-07
Interphase of mitotic cell cycle	435	3.78	18	3.75E-08	6.99E-07
Interphase	443	3.84	18	4.95E-08	9.02E-07
Protein folding	241	2.09	13	1.69E-07	3.02E-06
Negative regulation of protein metabolic process	540	4.69	19	1.95E-07	3.39E-06
RNA processing	890	7.72	24	5.65E-07	9.66E-06
RNA splicing	478	4.15	17	7.88E-07	1.32E-05
Cell cycle checkpoint	281	2.44	13	9.77E-07	1.60E-05
MRNA processing	551	4.78	18	1.24E-06	1.99E-05
Ribonucleoprotein complex assembly	124	1.08	9	1.28E-06	2.02E-05
Regulation of mitotic cell cycle	351	3.05	14	2.13E-06	3.29E-05
Protein metabolic process	5570	48.3	74	2.28E-06	3.46E-05
Cellular protein catabolic process	518	4.5	17	2.37E-06	3.53E-05
Ribosome biogenesis	166	1.44	9	1.41E-05	0.000206
Interaction with host	426	3.7	14	1.94E-05	0.000279
Amine metabolic process	268	2.33	11	2.14E-05	0.000303
Negative regulation of cellular metabolic process	1660	14.4	31	2.59E-05	0.00036
Protein catabolic process	644	5.59	17	4.04E-05	0.000553
Response to DNA damage stimulus	862	7.48	20	5.01E-05	0.000674
RNA metabolic process	6010	52.2	74	5.58E-05	0.000737
Actin filament based movement	78	0.677	6	5.83E-05	0.000759
Negative regulation of metabolic process	1820	15.8	32	5.96E-05	0.000764
Macromolecular complex assembly	1120	9.74	23	8.71E-05	0.00109
Cell cycle arrest	428	3.71	13	8.75E-05	0.00109
Cell cycle phase	1070	9.31	22	0.000127	0.00155
Cellular component assembly	1790	15.5	30	0.000248	0.00299
Positive regulation of catalytic activity	1070	9.28	21	0.00033	0.00393

RRNA processing	109	0.946	6	0.00037	0.00433
Negative regulation of catalytic activity	568	4.93	14	0.000408	0.00471
Cell cycle process	1420	12.3	25	0.000424	0.00483
Nucleobase containing compound metabolic process	8180	71	89	0.000554	0.00614
Nucleobase containing compound metabolic process	8180	71	89	0.000554	0.00614
RRNA metabolic process	118	1.02	6	0.000565	0.00617
Protein ubiquitination	658	5.71	15	0.000572	0.00617
Negative regulation of cell cycle	520	4.51	13	0.00058	0.00618
Regulation of apoptotic process	1540	13.3	26	0.000608	0.0064
Regulation of cell cycle	886	7.69	18	0.000624	0.00647
Mitotic cell cycle	968	8.4	19	0.000663	0.00679
Regulation of programmed cell death	1550	13.5	26	0.000725	0.00734
Proteolysis	1100	9.5	20	0.00116	0.0116
Protein modification by small protein conjugation	713	6.19	15	0.00129	0.0128
Positive regulation of cellular protein metabolic process	968	8.4	18	0.00171	0.0167
Transport	4830	42	58	0.00184	0.0178
Heterocycle metabolic process	8430	73.2	89	0.00207	0.0198
Cell cycle	1860	16.1	28	0.00225	0.0212
Cellular aromatic compound metabolic process	8460	73.4	89	0.00234	0.0218
Positive regulation of protein metabolic process	1080	9.4	19	0.00246	0.0227
Establishment of localization	4910	42.6	58	0.0028	0.0255
Cellular response to stress	1620	14.1	25	0.00303	0.0273
Positive regulation of protein modification process	867	7.52	16	0.00337	0.03
Spliceosomal complex assembly	39	0.338	3	0.00464	0.0409
Chromatin assembly	128	1.11	5	0.00514	0.0448
Regulation of cellular protein metabolic process	1560	13.5	23	0.00745	0.0643
Apoptotic process	2130	18.5	29	0.00792	0.0669
Apoptotic process	2130	18.5	29	0.00792	0.0669
Activation of cysteine type endopeptidase activity involved in apoptotic process	91	0.79	4	0.00808	0.0676
Programmed cell death	2160	18.7	29	0.0094	0.0779
G2/M transition of mitotic cell cycle	150	1.3	5	0.00989	0.0811
Chromatin assembly or disassembly	152	1.32	5	0.0104	0.0847
Actin filament based process	577	5.01	11	0.0117	0.0938
Mitochondrial transport	159	1.38	5	0.0125	0.0995
Regulation of catalytic activity	1730	15	24	0.0132	0.104
Cellular amino acid metabolic process	670	5.81	12	0.0135	0.105
Cellular amino acid metabolic process	670	5.81	12	0.0135	0.105
Regulation of translation	228	1.98	6	0.0145	0.111
Protein polymerization	167	1.45	5	0.0152	0.115
Nucleosome assembly	112	0.972	4	0.0164	0.123
DNA packaging	172	1.49	5	0.017	0.127

Positive regulation of cysteine type endopeptidase activity involved in apoptotic process	118	1.02	4	0.0194	0.144
Cellular membrane organization	471	4.09	9	0.0215	0.157
Regulation of protein modification process	1250	10.8	18	0.0221	0.16
Apoptotic DNA fragmentation	27	0.234	2	0.0228	0.163
Negative regulation of translation	70	0.608	3	0.0229	0.163
Protein targeting to mitochondrion	71	0.616	3	0.0238	0.168
Protein stabilization	72	0.625	3	0.0247	0.173
Negative regulation of cellular process	4110	35.6	46	0.0266	0.185
Cytoskeleton dependent intracellular transport	76	0.66	3	0.0284	0.195
Cellular protein complex assembly	343	2.98	7	0.0299	0.205
