# Supplementary material for: The RXFP3 receptor is functionally associated with cellular responses to oxidative stress and DNA damage
Source: Aging (Albany NY). 2019 Dec 3;11(23):11268–313. doi: 10.18632/aging.102528 (PMC6932917; doi:10.18632/aging.102528)
Supplement: Supplementary Table 15 [file aging-11-102528-s010..pdf]

Table S15. NetworkAnalyst-based Gene Ontology analysis of the RXFP3 interactome stabilized in the presence of CPT cellular perturbation (generic database). The proteins consistently associated with the RXFP3 receptor following exposure to CPT were analyzed using a generic human tissue database derived from IMEx (https://www.imexconsortium.org/). For the most stringent analysis process we employed a Zero Order Network approach. Gene Ontology (Biological Process) annotation was performed on all identified nodes using the built-in Gene Ontology analysis module of NetworkAnalyst (www.networkanalyst.ca). For each significantly-populated Gene Ontology term group (p<0.05) the total number of proteins associated with that group (Total), the expected (Expected) number of identified proteins from a random data sample (Hypergeometric test based), the actual number of GO term-populating proteins from the experimental dataset (Hits), the enrichment P value (P.Value) as well as the enrichment FDR are given (FDR).

GO Biological Process Term	Total	Expected	Hits	P.Value	FDR
MRNA metabolic process	817	9.26	72	1.78E-46	1.46E-43
Protein targeting to membrane	158	1.79	39	5.72E-42	2.35E-39
Cellular protein complex disassembly	160	1.81	38	3.56E-40	9.15E-38
Translational initiation	205	2.32	41	4.46E-40	9.15E-38
Protein complex disassembly	167	1.89	38	2.12E-39	3.48E-37
Cellular macromolecule catabolic process	849	9.63	65	1.29E-37	1.76E-35
Macromolecular complex disassembly	189	2.14	38	3.44E-37	4.03E-35
Viral reproduction	803	9.1	63	5.82E-37	5.96E-35
Viral reproductive process	597	6.77	56	1.22E-36	1.11E-34
RNA catabolic process	256	2.9	41	6.49E-36	5.33E-34
Viral infectious cycle	241	2.73	40	1.16E-35	8.61E-34
Translation	698	7.91	57	4.69E-34	3.21E-32
Cellular component disassembly	310	3.51	42	1.09E-33	6.88E-32
Macromolecule catabolic process	1070	12.1	65	1.61E-31	8.80E-30
Macromolecule catabolic process	1070	12.1	65	1.61E-31	8.80E-30
Protein targeting	545	6.18	47	8.68E-29	4.45E-27
Intracellular protein transport	793	8.99	51	2.52E-25	1.22E-23
Cellular nitrogen compound catabolic process	986	11.2	54	1.52E-23	6.94E-22
Multi organism process	1710	19.4	68	2.90E-22	1.25E-20
Cellular catabolic process	2140	24.3	72	2.05E-19	8.40E-18
Intracellular transport	1510	17.1	58	7.43E-18	2.90E-16
Protein transport	1400	15.9	55	2.94E-17	1.10E-15
Establishment of protein localization	1460	16.5	56	3.30E-17	1.18E-15
Cellular protein metabolic process	4540	51.5	103	6.40E-17	2.19E-15
Reproductive process	1740	19.8	60	3.15E-16	1.03E-14
Catabolic process	2560	29.1	73	1.11E-15	3.51E-14
Reproduction	1860	21	61	1.35E-15	4.10E-14
DNA damage response, signal transduction by p53 class mediator	117	1.33	17	1.63E-14	4.77E-13

Protein localization	1850	20.9	58	7.33E-14	2.07E-12
Signal transduction in response to DNA damage	129	1.46	17	8.59E-14	2.35E-12
DNA damage checkpoint	143	1.62	17	4.84E-13	1.28E-11
Mitotic cell cycle checkpoint	149	1.69	17	9.57E-13	2.45E-11
DNA integrity checkpoint	152	1.72	17	1.33E-12	3.31E-11
G1/S transition of mitotic cell cycle	209	2.37	19	2.53E-12	6.11E-11
S phase of mitotic cell cycle	144	1.63	16	6.96E-12	1.63E-10
Macromolecule localization	2240	25.4	61	7.37E-12	1.68E-10
S phase	153	1.73	16	1.77E-11	3.93E-10
Protein metabolic process	5570	63.1	105	2.08E-11	4.48E-10
Establishment of localization in cell	2320	26.3	61	3.10E-11	6.51E-10
Ribosome biogenesis	166	1.88	16	6.15E-11	1.26E-09
Protein polyubiquitination	177	2.01	16	1.62E-10	3.24E-09
RNA processing	890	10.1	34	2.76E-10	5.38E-09
Cellular localization	2650	30.1	64	3.43E-10	6.53E-09
Cell cycle checkpoint	281	3.19	19	4.37E-10	8.14E-09
Interphase of mitotic cell cycle	435	4.93	23	7.41E-10	1.35E-08
Interphase	443	5.02	23	1.06E-09	1.88E-08
Ribonucleoprotein complex biogenesis	269	3.05	18	1.53E-09	2.67E-08
Protein folding	241	2.73	17	2.02E-09	3.44E-08
Negative regulation of cellular protein metabolic process	463	5.25	23	2.47E-09	4.13E-08
RRNA processing	109	1.24	12	3.64E-09	5.96E-08
RRNA metabolic process	118	1.34	12	9.07E-09	1.46E-07
Cellular protein catabolic process	518	5.87	23	2.04E-08	3.22E-07
Negative regulation of protein metabolic process	540	6.12	23	4.40E-08	6.80E-07
Regulation of mitotic cell cycle	351	3.98	18	9.54E-08	1.45E-06
Interaction with host	426	4.83	19	3.61E-07	5.37E-06
Cell cycle arrest	428	4.85	19	3.87E-07	5.65E-06
Amine metabolic process	268	3.04	15	3.93E-07	5.65E-06
Protein catabolic process	644	7.3	23	1.00E-06	1.42E-05
RNA splicing	478	5.42	19	2.03E-06	2.82E-05
Positive regulation of cellular protein metabolic process	968	11	28	3.92E-06	5.36E-05
Response to DNA damage stimulus	862	9.77	26	4.32E-06	5.81E-05
Regulation of apoptotic process	1540	17.4	37	6.47E-06	8.56E-05
Negative regulation of catalytic activity	568	6.44	20	6.72E-06	8.74E-05
Negative regulation of cell cycle	520	5.9	19	6.88E-06	8.81E-05
Regulation of programmed cell death	1550	17.6	37	8.45E-06	0.000107
Positive regulation of protein metabolic process	1080	12.3	29	1.15E-05	0.000142
RNA splicing, via transesterification reactions	321	3.64	14	1.74E-05	0.000213
Cellular amino acid metabolic process	670	7.6	21	2.27E-05	0.00027
Cellular amino acid metabolic process	670	7.6	21	2.27E-05	0.00027

Negative regulation of cellular metabolic process	1660	18.9	37	3.92E-05	0.00046
MRNA processing	551	6.25	18	5.36E-05	0.000619
Regulation of cellular protein metabolic process	1560	17.7	35	5.43E-05	0.000619
Regulation of translation	228	2.59	11	5.80E-05	0.000652
Positive regulation of catalytic activity	1070	12.1	27	6.63E-05	0.000735
Cellular respiration	236	2.68	11	7.91E-05	0.000865
Mitotic cell cycle	968	11	25	9.00E-05	0.000971
RNA metabolic process	6010	68.2	92	0.000106	0.00113
Positive regulation of protein modification process	867	9.83	23	0.000119	0.00126
Regulation of cell cycle	886	10	23	0.000164	0.00171
Protein ubiquitination	658	7.46	19	0.000168	0.00172
Cell cycle phase	1070	12.2	26	0.00018	0.00182
Proteolysis	1100	12.4	26	0.000248	0.00248
Negative regulation of metabolic process	1820	20.7	37	0.00026	0.00256
Intracellular steroid hormone receptor signaling pathway	113	1.28	7	0.000299	0.00292
Positive regulation of translation	56	0.635	5	0.000422	0.00407
Protein modification by small protein conjugation	713	8.08	19	0.000461	0.0044
Regulation of protein metabolic process	1820	20.7	36	0.000559	0.00527
Nucleobase containing compound metabolic process	8180	92.7	113	0.000657	0.00605
Nucleobase containing compound metabolic process	8180	92.7	113	0.000657	0.00605
Apoptotic process	2130	24.1	40	0.000689	0.00621
Apoptotic process	2130	24.1	40	0.000689	0.00621
Programmed cell death	2160	24.4	40	0.000885	0.00789
Intracellular receptor mediated signaling pathway	270	3.06	10	0.00102	0.00903
Protein export from nucleus	48	0.544	4	0.0021	0.0183
Cell cycle process	1420	16	28	0.00244	0.021
Ribonucleoprotein complex assembly	124	1.41	6	0.00288	0.0245
Heterocycle metabolic process	8430	95.6	113	0.0029	0.0245
Transport	4830	54.8	72	0.00307	0.0257
Cellular aromatic compound metabolic process	8460	95.9	113	0.00332	0.0275
Regulation of protein modification process	1250	14.2	25	0.00362	0.0297
Activation of cysteine type endopeptidase activity involved in apoptotic process	91	1.03	5	0.00374	0.0304
Intrinsic apoptotic signaling pathway	135	1.53	6	0.00438	0.0352
Nucleocytoplasmic transport	388	4.4	11	0.00474	0.0374
Cellular response to stress	1620	18.4	30	0.00474	0.0374
Establishment of localization	4910	55.7	72	0.00483	0.0378
Nuclear transport	392	4.44	11	0.00511	0.0395
Regulation of catalytic activity	1730	19.6	31	0.00641	0.0491
Androgen receptor signaling pathway	67	0.76	4	0.00701	0.0533
Mitochondrial transport	159	1.8	6	0.00953	0.0717

Positive regulation of cysteine type endopeptidase activity involved in apoptotic process	118	1.34	5	0.011	0.0821
Energy derivation by oxidation of organic compounds	437	4.95	11	0.0111	0.0821
Cellular component disassembly involved in execution phase of apoptosis	78	0.884	4	0.0119	0.0869
Protein import	272	3.08	8	0.0124	0.0901
Nuclear export	139	1.58	5	0.021	0.151
G2/M transition of mitotic cell cycle	150	1.7	5	0.0281	0.2
Cell cycle	1860	21.1	30	0.0283	0.2
Carboxylic acid metabolic process	1270	14.4	22	0.0306	0.214
Positive regulation of cellular metabolic process	2530	28.7	38	0.0368	0.256
Apoptotic DNA fragmentation	27	0.306	2	0.0373	0.257
Generation of precursor metabolites and energy	603	6.84	12	0.0418	0.285
