# Supplementary material for: The RXFP3 receptor is functionally associated with cellular responses to oxidative stress and DNA damage
Source: Aging (Albany NY). 2019 Dec 3;11(23):11268–313. doi: 10.18632/aging.102528 (PMC6932917; doi:10.18632/aging.102528)
Supplement: Supplementary Table 16 [file aging-11-102528-s006..pdf]

Table S16. NetworkAnalyst-based KEGG pathway analysis of the RXFP3 interactome stabilized in the presence of peroxide cellular perturbation (generic database). The proteins consistently associated with the RXFP3 receptor following exposure to hydrogen peroxide were analyzed using a generic human tissue database derived from IMEx (https://www.imexconsortium.org/). For the most stringent analysis process we employed a Zero Order Network approach. KEGG signaling pathway annotation was performed on all identified nodes using the built-in KEGG Pathway analysis module of NetworkAnalyst (www.networkanalyst.ca). For each significantly-populated KEGG Pathway (p<0.05) the total number of proteins associated with that group (Total), the expected (Expected) number of identified proteins from a random data sample (Hypergeometric test based), the actual number of GO term-populating proteins from the experimental dataset (Hits), the enrichment P value (P.Value) as well as the enrichment FDR are given (FDR).

**KEGG Pathway**	**Total**	**Expected**	**Hits**	**P.Value**	**FDR**
Ribosome	153	2.18	32	1.56E-29	4.95E-27
Proteasome	45	0.64	10	4.69E-10	7.46E-08
Pathogenic Escherichia coli infection	55	0.782	7	1.12E-05	0.00119
Spliceosome	134	1.91	10	1.87E-05	0.00149
Systemic lupus erythematosus	133	1.89	9	0.000107	0.00681
Protein processing in endoplasmic reticulum	165	2.35	9	0.00054	0.0286
Antigen processing and presentation	77	1.09	6	0.000753	0.0342
Tight junction	170	2.42	8	0.00282	0.112
Longevity regulating pathway - multiple species	62	0.881	4	0.0115	0.405
Prion diseases	35	0.498	3	0.0132	0.418
Salmonella infection	86	1.22	4	0.0338	0.976
Mismatch repair	23	0.327	2	0.0417	1
Legionellosis	55	0.782	3	0.043	1
