# Supplementary material for: The RXFP3 receptor is functionally associated with cellular responses to oxidative stress and DNA damage
Source: Aging (Albany NY). 2019 Dec 3;11(23):11268–313. doi: 10.18632/aging.102528 (PMC6932917; doi:10.18632/aging.102528)
Supplement: Supplementary Table 17 [file aging-11-102528-s018..pdf]

Table S17. NetworkAnalyst-based KEGG pathway analysis of the RXFP3 interactome stabilized in the presence of peroxide cellular perturbation (hypothalamic database). The proteins consistently associated with the RXFP3 receptor following exposure to hydrogen peroxide were analyzed using a human hypothalamic tissue database derived from DIFFERENTIALNET (http://netbio.bgu.ac.il/diffnet/). For the most stringent analysis process we employed a Zero Order Network approach. KEGG signaling pathway annotation was performed on all identified nodes using the built-in KEGG Pathway analysis module of NetworkAnalyst (www.networkanalyst.ca). For each significantly-populated KEGG Pathway ($p<0.05$) the total number of proteins associated with that group (Total), the expected (Expected) number of identified proteins from a random data sample (Hypergeometric test based), the actual number of GO term-populating proteins from the experimental dataset (Hits), the enrichment P value (P.Value) as well as the enrichment FDR are given (FDR).

KEGG Pathway	Total	Expected	Hits	P.Value	FDR
Ribosome	153	1.48	15	1.14E-11	3.62E-09
Pathogenic Escherichia coli infection	55	0.533	7	8.47E-07	0.000135
Protein processing in endoplasmic reticulum	165	1.6	9	2.76E-05	0.00292
Proteasome	45	0.436	5	6.75E-05	0.00537
Antigen processing and presentation	77	0.746	6	9.32E-05	0.00593
Tight junction	170	1.65	8	0.000222	0.0118
Spliceosome	134	1.3	7	0.000298	0.0135
Prion diseases	35	0.339	3	0.00458	0.182
Salmonella infection	86	0.834	4	0.0095	0.336
Phagosome	152	1.47	5	0.0157	0.463
Legionellosis	55	0.533	3	0.016	0.463
Mismatch repair	23	0.223	2	0.0206	0.538
Longevity regulating pathway - multiple species	62	0.601	3	0.022	0.538
Glycolysis / Gluconeogenesis	68	0.659	3	0.028	0.635
Cell cycle	124	1.2	4	0.0319	0.677
Biosynthesis of amino acids	75	0.727	3	0.0359	0.679
Systemic lupus erythematosus	133	1.29	4	0.0398	0.679
Base excision repair	33	0.32	2	0.0403	0.679
RNA degradation	79	0.766	3	0.0409	0.679
Estrogen signaling pathway	138	1.34	4	0.0445	0.679
Viral carcinogenesis	201	1.95	5	0.045	0.679
DNA replication	36	0.349	2	0.0473	0.679
Hypertrophic cardiomyopathy (HCM)	85	0.824	3	0.0491	0.679
