# Supplementary material for: The RXFP3 receptor is functionally associated with cellular responses to oxidative stress and DNA damage
Source: Aging (Albany NY). 2019 Dec 3;11(23):11268–313. doi: 10.18632/aging.102528 (PMC6932917; doi:10.18632/aging.102528)
Supplement: Supplementary Table 18 [file aging-11-102528-s007..pdf]

Table S18. NetworkAnalyst-based KEGG pathway analysis of the RXFP3 interactome stabilized in the presence of CPT cellular perturbation (generic database). The proteins consistently associated with the RXFP3 receptor following exposure to CPT were analyzed using a generic human tissue database derived from IMEx (www.imexconsortium.org/). For the most stringent analysis process we employed a Zero Order Network approach. KEGG signaling pathway annotation was performed on all identified nodes using the built-in KEGG Pathway analysis module of NetworkAnalyst (www.networkanalyst.ca). For each significantly-populated KEGG Pathway (p<0.05) the total number of proteins associated with that group (Total), the expected (Expected) number of identified proteins from a random data sample (Hypergeometric test based), the actual number of GO term-populating proteins from the experimental dataset (Hits), the enrichment P value (P.Value) as well as the enrichment FDR are given (FDR).

KEGG Pathway	Total	Expected	Hits	P.Value	FDR
Ribosome	153	2.49	38	8.61E-36	2.74E-33
Proteasome	45	0.733	14	4.83E-15	7.67E-13
Oxidative phosphorylation	133	2.17	12	1.49E-06	0.000158
Protein processing in endoplasmic reticulum	165	2.69	13	2.49E-06	0.000198
Parkinson's disease	142	2.31	11	1.81E-05	0.00115
Huntington's disease	193	3.14	12	6.68E-05	0.00354
Alzheimer's disease	171	2.78	11	1.00E-04	0.00456
Antigen processing and presentation	77	1.25	7	0.00024	0.00955
Spliceosome	134	2.18	7	0.00611	0.216
RNA degradation	79	1.29	5	0.00913	0.29
Non-alcoholic fatty liver disease (NAFLD)	149	2.43	7	0.0107	0.309
Legionellosis	55	0.896	4	0.0121	0.32
Epstein-Barr virus infection	201	3.27	8	0.0166	0.407
Retrograde endocannabinoid signaling	148	2.41	6	0.0335	0.761
N-Glycan biosynthesis	50	0.814	3	0.0475	1
