# Supplementary material for: The RXFP3 receptor is functionally associated with cellular responses to oxidative stress and DNA damage
Source: Aging (Albany NY). 2019 Dec 3;11(23):11268–313. doi: 10.18632/aging.102528 (PMC6932917; doi:10.18632/aging.102528)
Supplement: Supplementary Table 19 [file aging-11-102528-s001..pdf]

Table S19. NetworkAnalyst-based KEGG pathway analysis of the RXFP3 interactome stabilized in the presence of CPT cellular perturbation (hypothalamic databae). The proteins consistently associated with the RXFP3 receptor following exposure to CPT were analyzed using a human hypothalamic tissue database derived from DIFFERENTIALNET (http://netbio.bgu.ac.il/diffnet/). For the most stringent analysis process we employed a Zero Order Network approach. KEGG signaling pathway annotation was performed on all identified nodes using the built-in KEGG Pathway analysis module of NetworkAnalyst (www.networkanalyst.ca). For each significantly-populated KEGG Pathway ($p<0.05$) the total number of proteins associated with that group (Total), the expected (Expected) number of identified proteins from a random data sample (Hypergeometric test based), the actual number of GO term-populating proteins from the experimental dataset (Hits), the enrichment P value (P.Value) as well as the enrichment FDR are given (FDR).

Pathway	Total	Expected	Hits	P.Value	FDR
Proteasome	45	0.489	13	6.13E-16	1.95E-13
Ribosome	153	1.66	19	1.51E-15	2.39E-13
Antigen processing and presentation	77	0.836	7	1.80E-05	0.00191
Protein processing in endoplasmic reticulum	165	1.79	9	6.86E-05	0.00546
Parkinson's disease	142	1.54	6	0.00432	0.256
Huntington's disease	193	2.1	7	0.00483	0.256
Epstein-Barr virus infection	201	2.18	7	0.00602	0.273
RNA transport	165	1.79	6	0.00887	0.353
RNA degradation	79	0.858	4	0.0105	0.37
Spliceosome	134	1.45	5	0.015	0.477
Pathogenic Escherichia coli infection	55	0.597	3	0.0216	0.572
Legionellosis	55	0.597	3	0.0216	0.572
Cellular senescence	160	1.74	5	0.0297	0.664
Necroptosis	162	1.76	5	0.0311	0.664
HTLV-I infection	219	2.38	6	0.0313	0.664
RNA polymerase	31	0.337	2	0.0442	0.851
Cell cycle	124	1.35	4	0.0455	0.851
