# Supplementary material for: The RXFP3 receptor is functionally associated with cellular responses to oxidative stress and DNA damage
Source: Aging (Albany NY). 2019 Dec 3;11(23):11268–313. doi: 10.18632/aging.102528 (PMC6932917; doi:10.18632/aging.102528)
Supplement: Supplementary Table 20 [file aging-11-102528-s021..pdf]

Table S20. BioGRID interactomes for DNA damage response, and oxidative stress. To investigate the role for RXFP3 in oxidative stress and DNA damage response, we compared its interacting proteins in different conditions to the interacting proteins of several well-known DNA damage response and oxidative stress-related proteins, which we extracted from the freely available BioGRID database (https://thebiogrid.org/). As a control we used proteins which are so far known not to play a role in these processes. DNA damage response/repair: BRCA1, MDM2, PRKDC, TP53, MDC1, H2AFX; Oxidative stress: G3BP1, SOD1, SIRT1; Control/Non-stress: CNTRL, CRP, LONP2.

**DNA damage response/repair**						**Oxidative Stress**			**Non-Stress**		
**BRCA1**	**MDM2**	**PRKDC**	**TP53**	**MDC1**	**H2AFX**	**G3BP1**	**SOD1**	**SIRT1**	**CNTRL**	**CRP**	**LONP2**
ABL1	AATF	ABL1	ACTB	ABCD3	A2M	ADD3	AARS	ACACA	ABRACL	ACAA2	AMOT
ABLIM3	ABL1	ADRB2	AIMP2	AMFR	ABHD10	ADSL	AASDHPPT	AHCY	ANKRD26	ACTN1	CDC16
ACACA	ABL2	AHSA1	ARID1A	ANAPC1	ACTB	ASCC3	ACAT1	AKIP1	ANKRD28	APP	DCN
ACLY	ACACA	AICDA	ARIH2	ANAPC10	AIFM1	ATXN2L	ACO2	AKIRIN2	ARHGAP21	ASCC1	DKK3
AHR	ACTB	AIRE	ASH2L	ANAPC16	ALDOA	BCL7C	ACOT7	AKR1D1	ARHGEF18	ASCC2	EFTUD2
AIMP2	ADRBK1	AKT1	ATF3	ANAPC2	ALYREF	BLMH	ACTN4	AKT1	ARHGEF2	BAG3	ELAVL1
AKT1	AKT1	AKT2	ATM	ANAPC4	ANKHD1	BMI1	ACY1	ARID4B	AURKB	BMI1	FBXW11
ALDH9A1	ALPI	AKTIP	ATR	ANAPC5	ANXA2	CAPRIN1	ADK	ARNTL	BAG6	CFL1	IGFBP4
ALDOA	APEX1	ALOX15	ATRX	ANAPC7	ANXA2P2	CCDC8	ADRM1	ARRB2	C2CD3	CNTRL	IL17A
ANKRD28	AR	ANAPC2	ATXN3	ATM	ANXA4	CCDC84	ADSL	ATG5	CALR	CSRP1	IL1R2
AR	ARIH2	AP1B1	AURKA	ATP2A2	ANXA5	CENPF	AHCYL1	ATG7	CAMSAP3	EGFR	LY86
ARFGEF2	ARRB1	AP2M1	AXIN1	ATXN2	ANXA6	COX5A	AHSG	ATXN7L3	CC2D1A	ESR1	LYPD2
ARNT	ARRB2	AR	BAG2	ATXN3	APOA1BP	CROCC	AK2	BCL11A	CCDC138	FUBP3	MAP7D2
ASH2L	ATF3	ARHGEF6	BAG5	AUP1	ARRB1	CSK	AKR1A1	CAP1	CCDC14	GRB2	MAPK6
ATF1	ATF4	ASB5	BAIAP2L1	BABAM1	ARRB2	CSNK1G2	AKR1B1	CCAR2	CCDC85C	HSPB1	MTMR10
ATM	ATM	ATG101	BAK1	BANF1	ATF2	CTNNBL1	AKR1B15	CCNB1	CCNB1	MACF1	NEDD
ATR	ATP2A2	ATG4C	BANP	BARD1	ATM	CTSC	AKR1C2	CEP44	CEP128	NUP214	OLFML2B
ATRIP	ATRX	ATM	BARD1	BIRC5	ATR	DHX40	ALDH7A1	CERKL	CEP131	PRKAR2A	OS9
AURKA	AURKA	ATOH1	BAX	BRCA1	ATRX	DTX1	ALDOA	CHD3	CEP152	RNF2	PCDHA11
BABAM1	BAIAP2L1	ATRIP	BCL2	BRCC3	BANP	EDC4	AMFR	CHEK2	CEP162	SUZ12	PCDHA12
BACH1	BANP	ATRX	BCL2L1	BRE	BARD1	EIF2B3	ANXA1	CHFR	CEP170	TALDO1	PCDHA3
BAP1	BRINP1	AUP1	BCL2L2	C2ORF49	BAZ1A	EIF3A	AP3D1	CLK3	CEP170B	TES	PCDHA4
BARD1	BTRC	BAG2	BLM	CAPZA1	BIRC5	EIF3C	API5	CLOCK	CEP192	TRIP4	PCDHA8
BCL2	C19ORF10	BAG3	BMI1	CAPZB	BLM	EIF3M	APOL2	CMYA5	CEP350	UFM1	PCDHA9
BLM	CANX	BECN1	BRCA1	CASP3	BMI1	EIF4G1	APRT	CPA6	CEP85L	YBX3	PEX5
BORA	CASP3	BIRC5	BRCA2	CBX1	BRCA1	ELAVL1	ASCC1	CREB1	CEP89		RETNLB
BRAP	CCNG1	BMI1	BRCC3	CBX3	BRCA2	ENV	ASNS	CREBBP	CKAP2		RNF181
BRAT1	CDH1	BRCA1	BRD7	CDC16	BRD1	EPHA8	ATOX1	CSNK2A1	CKAP5		RSPH9
BRCA1	CDKN1A	BSG	BRE	CDC20	BTF3	FAF1	ATP1A1	CSNK2A2	CKB		SUCLA2

BRCA2	CDKN2A	C14ORF166	BTRC	CDC23	C16ORF87	FAU	ATP6V1A	CSNK2B	CLASP1		TMOD3
BRCC3	CDKN2AIP	C1D	C10ORF90	CDC27	C1ORF174	FBXO25	ATP6V1B1	CTNNB1	CLTC		TRIM25
BRD7	CHEK2	CAMKK2	CCDC8	CDC5L	C5ORF64	FLNB	BANF1	CTTN	COPB2		UGP2
BRE	CLPB	CASP3	CCNG1	CDKN2AIP	CALM1	FNDC3B	BASP1	CWC15	COX3		WWOX
BRIP1	CMSS1	CCDC8	CCT2	CENPC	CALM2	FSCN1	BCL2	DIAPH3	CSPP1		
CASP1	COPS5	CCNB1	CCT3	CHAMP1	CALR	FTSJ3	BCL2L13	DNMT1	CSRP1		
CBX3	CREBBP	CDC5L	CCT4	CHCHD3	CBX2	FUBP3	C11ORF54	DNMT3B	CYB5R3		
CCAR2	CRTC2	CDC73	CCT5	CHEK2	CBX5	FXR1	C14ORF166	DOT1L	DAPK3		
CCNA2	CRTC3	CDK2	CCT6A	CLU	CCDC86	FXR2	C1ORF86	DTL	DBT		
CCNB1	CSNK1A1	CDK4	CCT7	COA7	CENPA	G3BP2	CAT	DVL1	DLG5		
CCND1	CSNK1D	CDK9	CCT8	COPE	CETN1	GFPT1	CCAR2	DVL2	DNM2		
CCT2	CSNK2A1	CEBPB	CDC14A	COPS5	CETN2	GNB2	CCS	DYNC1H1	DSP		
CCT3	CSNK2A2	CENPA	CDK2	CPSF2	CFAP45	GNL2	CCS1	E2F1	DYNC1H1		
CCT4	CSNK2B	CFTR	CDK7	CPSF3	CFL1	H1F0	CCT2	ECT2	ERC1		
CCT7	CTBP1	CHAF1A	CDK9	CPSF4	CHD1L	H1FX	CCT7	EED	ERLIN2		
CDK16	CTBP2	CHD1L	CDKN1A	CPSF6	CLU	H2AFV	CCT8	EEF1G	EXOC4		
CDK2	CUL1	CHEK1	CDKN2A	CREBBP	CMBL	HAT1	CDC42	EGLN3	FAM167A		
CDK4	CUL4A	CHEK2	CELA2B	CSNK2A1	COPG1	HDAC6	CENPV	ELAVL1	FAM21A		
CDKN2A	DAPK3	CHRM3	CHEK1	CSRP2	CPS1	HELZ	CEP55	ELL3	GANAB		
CDKN2D	DAXX	CHRM4	CHEK2	CUL4A	CRAMP1L	HELZ2	CFL1	EP300	GPATCH1		
CEP350	DCAF8	CHUK	COPS5	CUL7	CSNK2A1	HIST2H2AA3	CFL2	EPAS1	GTSE1		
CHD9	DDB1	CIB1	CREB1	CXORF57	CTCF	HIST2H3A	CHCHD2	ESR1	HAUS2		
CHEK1	DDX24	CLK1	CREBBP	DEPDC1B	CXXC1	HMGA1	CKB	ESRRA	HAUS3		
CHEK2	DDX42	CLN3	CRYAB	DLST	CYP19A1	HMGB3	CLDN17	ESRRB	HAUS4		
CLK2	DET1	COPS5	CSNK1D	DPPA4	DDX21	HMGN1	COLEC10	EWSR1	HAUS5		
CLSPN	DHFR	CRY1	CSNK2A1	DTX2	DHX30	HNRNPA1	COMMD1	EZH2	HAUS6		
CLTC	DHRS2	CRY2	CUL7	EED	DHX9	HNRNPA3	COPA	FASN	HAUS7		
CNRIP1	DHX9	CSNK2A1	CUL9	ELAVL1	DIS3L	HNRNPAB	COPG1	FBXO7	HAUS8		
COMMD1	DIRAS3	CTDP1	CXXC1	EMD	DKC1	HNRNPH2	COX17	FEZF1	HK1		
CRBN	DLG4	CTPS2	CYLD	ENO1	DMAP1	HNRNPU	CPSF7	FGF10	HSDL2		
CREBBP	DNAJB1	CUL3	DAXX	EP300	EEF1A1	HNRNPUL1	CRIP1	FGF11	HSPA1B		
CRYZL1	DNAL4	CUL5	DDX5	EPS8	EGFR	HSP90AA1	CRYAB	FGF12	HYOU1		
CSNK1D	DYRK2	CUL7	DNAJA1	ESAM	EIF3L	HSPA4	CRYZ	FOS	IDH3B		
CSNK2B	E2F1	CWC27	DNAJC7	EXOSC2	EIF4A1	HSPA9	CUTA	FOXL2	IFT81		
CSTF2	EED	CYLD	DNMT1	EXOSC8	ELAVL1	HSPD1	DARS	FOXO1	IGF2BP2		
CTNNB1	EEF1A1	DCLRE1B	DTL	FAM175A	ELAVL2	IFI30	DBI	FOXO3	KDELR2		
CTPS1	EEF2	DCLRE1C	E4F1	FANCD2	ENO1	IFIH1	DDX39B	FOXO4	KIAA0753		
DDB1	EFTUD2	DDA1	EGR1	FLNA	ENY2	IGF2BP1	DES	FOXP3	KIAA0753		
DSP	EID1	DDX5	EHMT1	FLNB	ERP29	IGF2BP2	DNAJA1	GABARAPL1	KIAA1671		
DHX9	EIF3B	DHX38	EIF2AK2	FZR1	ESR1	IGF2BP3	DNM1L	GAPDH	KIAA1671		
DNAJA1	ELF4	DHX9	ELL	GATA4	EYA1	IK	DSTN	GTF2A1	KIF14		

DSP	EP300	DNAJC7	ELL3	GNB2L1	EYA3	IPO7	DYNLT1	H2AFZ	KIF7		
EBNA1BP2	ESR1	E4F1	EP300	GTF2E2	FANCD2	ITGB1	EEF1B2	HDAC2	LMO7		
EEF2	EXOSC6	EED	ERCC2	H2AFX	FGF3	KANK2	EEF1D	HDAC4	LONP1		
EIF3B	EZH2	EFTUD2	ERCC3	HDAC10	FGF8	KHDRBS3	EEF1G	HERC2	LUC7L2		
EIF3I	FBXO31	EGFR	ERCC6	HDAC8	FGFBP1	KIAA1524	EEF2	HES1	LUZP1		
EIF4G1	FGF11	EGLN3	ESR1	HERC2	FKBP10	KIF23	EIF1AX	HEY2	MAP3K4		
EIF5B	FHL2	EHD1	ETS1	HINFP	FOXA1	KPNA1	EIF2S1	HIC1	MAP4K4		
ELK4	FKBP1A	EIF2S2	ETS2	HMBOX1	FOXO3	KPNA2	EIF3A	HIF1A	MAP7D3		
ENO1	FKBP3	EIF4EBP1	FAM175B	HNRNPC	GAN	KPNA6	EIF3D	HIST1H2BC	MAPK6		
EP300	FOXO1	ELAVL1	FBXO11	HNRNPU	GANAB	KPNB1	EIF3E	HIST1H3A	MED4		
ERCC5	FOXO3	EMD	FBXO42	HNRNPUL2	GINS2	KRI1	EIF3H	HIST2H2AB	MGST3		
ERCC6	FOXO4	EP300	G3BP1	IMMT	GINS3	LARP1	EIF3I	HNF1A	MIB1		
ESR1	FUBP1	EPHA1	G3BP2	KPNB1	GINS4	LARP4	EIF4A3	HNF4A	MPHOSPH9		
EZH2	G3BP2	ERG	GNL3	LAS1L	GLB1	LBR	ELAVL1	HNRNPA1L2	MTA2		
EZR	GADD45A	ESR1	GTF2H1	LMNA	GON4L	LDHB	ENO1	HNRNPK	MTPN		
FAM175A	GNL3	FAF2	HCVGP1	LOC729324	H2AFY	LEMD3	ENO2	HOXA5	NAA15		
FANCA	GNL3L	FANCD2	HDAC1	LPCAT1	HAT1	LMNA	ENO3	HOXB5	NCKAP5L		
FANCD2	GORAB	FBXO6	HDAC2	LSM12	HEXA	LPP	EPDR1	HOXB9	NEDD1		
FBXO44	GSK3B	FLII	HECW1	LYAR	HIBCH	LSM14A	EPRS	HSF1	NIN		
FHL2	GTF2E2	FN1	HERC2	MCPH1	HIF1A	LSM14B	ETFB	HSP90AA1	NME7		
FLII	GTF2I	FOXRED2	HIF1A	MDM2	HIST1H1C	LSM3	EXOSC6	HSPA1L	NNT		
FLNA	HCK	FZR1	HIF1A	METTL17	HIST1H1T	LUC7L	FABP3	HSPA4	NUP205		
GNB2L1	HDAC1	GAG	HIPK2	MICAL3	HIST1H2BC	LUZP1	FABP5	HSPA5	ODF2		
GTF2I	HIF1A	GBAS	HMGB1	MKI67	HIST1H2BD	MACF1	FABP7	HSPA9	OFD1		
H2AFX	HIPK2	GRK5	HNRNPA1	MLC1	HIST1H2BM	MAGEA4	FEN1	HSPD1	PCM1		
HDAC2	HIST2H2BE	GSK3A	HNRNPK	MPG	HIST1H2BO	MAGED1	FKBP1A	ING1	PCNT		
HERC2	HIST3H3	GSK3B	HNRNPM	MRE11A	HIST1H3A	MAGED2	FKBP1B	ING2	PDHA1		
HIBADH	HMGN1	GTF2I	HSC82	MYH14	HIST1H3B	MAGOHB	FKBP7	IQCB1	PDIA3		
HIST1H2AB	HNRNPD	GZMB	HSP82	MYL12A	HIST1H4A	MAP4	FUBP1	IRS1	PDIA4		
HIST2H2AC	HNRNPK	H2AFX	HSP90AA1	MYO1D	HIST2H2AB	MAP4K4	GANAB	IRS2	PIBF1		
HMMR	HNRNPU	H3F3A	HSPA1A	NBN	HIST2H2AC	MAPRE1	GAPDH	JAK1	PLK1		
HNRNPA0	HSP90AA1	HDAC11	HSPA1B	NCL	HIST2H2BE	MARS	GDI1	JUN	POLR2C		
HNRNPA2B1	HSP90B1	HDAC3	HSPA1L	NDUFA10	HIST2H3C	MCM2	GLO1	JUND	PRKACA		
HNRNPD	HSPA8	HDAC5	HSPA4	NUMA1	HIST2H3PS2	MCM4	GLOD4	KAT2A	PRPF38B		
HNRNPF	HUWE1	HDGF	HSPA8	OBSL1	HIST2H4A	MCM5	GLRX	KAT2B	PSMD14		
HNRNPU	IER2	HDLBP	HSPA9	OSBPL8	HIST4H4	MCM7	GNB1	KAT5	PSMD7		
HSP90AA1	IER3	HHV8GK18_GP81	HSPB1	PAF1	HLTF	METAP1	GNPDA2	KAT8	PWP2		
HSP90AB2P	IGF1R	HIF1A	HUWE1	PAXIP1	HMGA1	MEX3A	GOT1	KCNA4	RAB11B		
HSPA4	IPO7	HIST1H1A	ING1	PCGF1	HNRNPA1	MFAP1	GPX4	KCNA5	RAB2A		
HSPA5	IRF1	HIST1H1A	ING4	PDHB	HNRNPA2B1	MKI67	GSTK1	KCNAB2	RAB35		
HSPA8	IRF2	HIST1H1C	ING5	PDZD11	HNRNPAB	MOV10	GSTP1	KIAA1598	RAB5B		

HSPD1	ITCH	HIST1H3A	KAT2B	PELO	HNRNPC	MPP1	GSTZ1	KPNA2	RAB7A		
IFI16	IYD	HIST1H3E	KAT5	PGAM5	HNRNPD	MRPL45	H1F0	KPNA3	RAB8A		
IFI204	JAK1	HNRNPA1	KAT6A	PHB2	HNRNPDL	MSH6	H1FX	KRT79	RPAP2		
IFI30	KAT2B	HNRNPC	KAT8	PHGDH	HNRNPH3	MSI2	HAGH	KSR1	RPGRIP1L		
INPP1	KAT5	HOXB7	KDM1A	PIAS1	HNRNPR	MTHFD1	HDAC6	KYNU	S100A4		
IQGAP1	KIAA1551	HSF1	KDM4D	PIAS4	HP1BP3	MYO6	HECW1	LAMA3	SDCCAG3		
ITPR1	KPNA1	HSP90AA1	KPNA4	PLRG1	HSD17B10	NEXN	HEXB	LANCL1	SEC24C		
JUN	KPNA6	HSP90AB1	L3MBTL1	POGZ	HSP90AA1	NOL6	HIBCH	LIMA1	SGPL1		
JUNB	KRT2	HSPA5	MAGEA2	POLR2A	HSP90B1	NOP58	HINT1	LRPPRC	SHKBP1		
JUND	LATS2	HSPA8	MAGED2	POLR2B	HSPA1A	NOSIP	HIST1H2AE	LTA4H	SLAIN2		
KARS	MAGEA2	IGF1R	MAP3K1	POLR2C	HSPA1L	NPM1	HMGB1	MAPK8	SLC39A7		
KDM1A	MAK16	IKBKAP	MAP9	POLR2E	HSPA5	NSUN2	HMGB3	MAPKAPK2	SOAT1		
KIF20A	MAP1LC3A	IKBKB	MAPK1	PPP1CA	HSPA9	NTMT1	HNRNPAB	MAPT	SORBS1		
KIF23	MAP2	ILF2	MAPK14	PPP2CA	HUWE1	NTRK1	HRNR	MAX	SPATA2		
KPNA2	MAPKAPK2	ILF3	MAPK3	PRIM1	HYOU1	NUDC	HSP90AB1	MCF2L2	SQSTM1		
LDHA	MDC1	ILK	MAPK8	PRKDC	ILF2	NUFIP2	HSP90B1	MCM10	SRP9		
LDHB	MDM2	IQCB1	MAPK9	PRKRA	IPO9	NUP188	HSPA1B	MDM2	SRSF2		
LMO4	MDM4	ITGA4	MDH1	PSMD4	KAT2A	NUP205	HSPA2	MECOM	SSR3		
MAN2C1	MRE11A	JUN	MDM2	PYCR1	KAT2B	NUP214	HSPA4	MEF2C	SSX2IP		
MAP3K3	MS4A1	KAT2A	MDM2	PYCR2	KAT5	NUP62	HSPA4L	MLLT1	SYCE1		
MCM4	MSI2	KAT5	MDM4	RAD50	KAT8	NUP98	HSPA5	MLLT3	SYCE3		
MCRS1	MTBP	KAT8	MED1	RAD51	KRR1	ORF1	HSPA8	MPHOSPH8	TANC1		
MDC1	NAT10	LGR4	MSL2	RAG1	LARP1B	PA2G4	HSPA9	MTA1	TBC1D31		
MED17	NBN	LIG4	MTA1	RAI14	LARP7	PABPC1	HSPE1	MTFR2	TCHP		
MED21	NCL	LMNA	MTA2	RBM17	LDHB	PABPC4	HSPH1	MTOR	TIMM23B		
MKI67	NEDD4	LNX1	MUC1	RBM39	LGALS3BP	PAK4	HUWE1	MYC	TJP1		
MLH1	NGFR	LPAR4	MUL1	RECQL5	LIG3	PALLD	IDH3A	MYCN	TJP2		
MNAT1	NME2	LRRFIP1	MYC	RFC2	LMNA	PAWR	ILF2	MYOD1	TMEM256		
MRE11A	NOC2L	LRRK2	NABP2	RFC4	MACROD1	PCNA	IMPDH2	NAT10	TNRC6A		
MSH2	NOLC1	LYN	NAT10	RFC5	MAGEB10	PDCD6IP	IQGAP1	NBN	TNRC6B		
MSH3	NOTCH1	MAPK8	NCL	RMDN3	MAPK8	PDLIM1	KIF5B	NCOR1	TP53BP2		
MSH6	NPIPB3	MAPK9	NCOA1	RNF4	MASP1	PDLIM4	KLC2	NDN	TRA2A		
MTA2	NPM1	MAS1	NDN	RNF8	MATR3	PDLIM5	KPNB1	NMNAT1	TRIM26		
MYC	NR0B2	MBP	NFYA	RPA1	MCM2	PDPK1	LACTB2	NOC4L	TTF2		
MYH14	NUCKS1	MCM2	NLK	RPA2	MCM4	PDS5B	LARS	NOP14	TTK		
NAT10	NUMB	MDC1	NOC2L	RPA3	MCM5	PELO	LDHA	NOS3	TTLL12		
NBN	ORF50	METTL1	NPM1	RPL29	MCM6	PES1	LDHAL6A	NPM1	TUBGCP2		
NCL	PA2G4	MEX3C	NQO1	SEC61A1	MCPH1	PFDN4	LDHAL6B	NR0B2	TUBGCP3		
NCOA1	PAK6	MGMT	NR0B2	SERBP1	MDC1	PFN2	LDHB	NR1H2	TXLNA		
NCOA2	PAP1	MKNK1	NR3C1	SF3A1	MIER1	PGAM5	LDHC	NR1H3	TXLNG		
NCOA3	PBX1	MLH1	NUMB	SH3KBP1	MIER3	PHLDB2	LGALS1	NR1H4	UPF1		

NELFB	PBXIP1	MRE11A	OBSL1	SIRT7	MLLT1	PIN1	LGALS3	NUDCD2	VIM		
NMI	PCNA	MSH6	OTUB1	SLC25A11	MMS22L	PKP2	LMNA	NUDT21	VPS33A		
NOL11	PDE4D	MSL3	OTUD5	SLC25A22	MRE11A	POLR2B	LMNB1	PAPOLA	WDR83		
NOP2	PDIA3	MTF1	PABPC1	SMARCA4	MRPS35	POP1	LMNB2	PARP1	WRAP73		
NPM1	PDLIM7	MTNR1B	PADI4	SMARCC1	MRPS7	PPIA	LOC101930400	PHLDB3	YARS2		
NUDC	PDS5A	MTOR	PARK7	SMARCC2	MSI2	PPM1D	MAP4K1	PICALM	YWHAH		
NUFIP1	PER2	MYC	PARP1	SMARCD1	MSN	PPME1	MARCKSL1	PIK3R1	ZNF326		
NUMA1	PGAM2	NBN	PATZ1	SMARCD2	NAP1L1	PPP1R10	MAT2A	PIP5K1A	ZNF622		
NUP153	PHLDB3	NCF1	PCNA	SMC1A	NBN	PPP1R18	MAVS	PML			
OLA1	PIAS4	NCF2	PDCD5	SMN1	NCL	PPP2R1A	MCM2	POP1			
ORC2	PIM1	NCF4	PDLIM7	SNRNP40	NGFR	PRDX1	MCM3	PPARA			
P4HB	PKM	NCOA6	PER2	SNRPB	NONO	PRDX6	MCM4	PPARGC1A			
PABPC1	PLK1	NFATC2	PHB	SRPK2	NPAT	PRKAA2	MCM5	PRMT1			
PAICS	PML	NOS2	PHF1	SRSF1	NPM1	PRKRA	MCM6	PSME3			
PALB2	PPAN	NOTCH1	PIAS1	SRSF9	OTUB1	PRMT1	MDH2	PTPN4			
PALB2	PPARD	NR1H4	PIAS2	SSBP3	P3H1	PRMT5	MIF	PUS7			
PARP1	PPIB	NR3C1	PIAS4	SSR1	P4HA2	PRRC2A	MOB1B	RAP1A			
PCNA	PPP2R5C	NTRK1	PIN1	STAU1	PABPC1	PRRC2C	MPI	RARA			
PGR	PRDM2	NUCB1	PLK3	SUMO2	PABPC4L	PSMD2	MRPL12	RB1			
PIAS1	PSMA7	NUP107	PML	SUPT6H	PAIP1	PTBP1	MTHFD1	RBFA			
PIAS4	PSMC3	NUP35	PPARGC1A	TAGLN2	PAK1	PTBP3	MTPN	RBM25			
PLK1	PSMC5	NUP85	PPP1R13L	TFAP4	PAPPA	PTGES3	MYBBP1A	RBP1			
POLB	PSMD10	PAN2	PPP2R1A	THRAP3	PARP1	PTPN2	MYH11	RECQL4			
POLN	PSMD2	PARP1	PPP2R5C	TOPBP1	PARP2	PUF60	NACA	RELA			
POLR2A	PSMD4	PCNA	PRKDC	TP53	PAXIP1	PUM1	NAP1L4	RICTOR			
POLR2A	PSME3	PDX1	PSMC5	TP53BP1	PBK	PUM2	NFS1	RPL6			
POLR2H	PTBP1	PGR	PSMD4	TPM1	PCBP2	PURA	NME1	RPS19BP1			
POU2F1	PYHIN1	PHGDH	PSME3	TPM3	PDCD6	PURB	NME1-NME2	RPS2			
PPFIA1	RABL6	PHKG2	PTEN	TRIP13	PDIA4	PYCR1	NME2	RPTOR			
PPP1CA	RAD23A	PIDD1	PTGS2	U2SURP	PEF1	RAB1A	NOP10	RRP8			
PPP1CB	RAD50	PINK1	PTK2	UIMC1	PGK1	RACGAP1	NSUN2	SART1			
PPP2R5C	RAD54B	PMS2	PTTG1	USP13	PHF14	RAD21	NUP93	SATB1			
PPP6C	RARA	PNKP	PTTG1IP	USP28	PKM	RANBP1	NUTF2	SCNM1			
PPP6R3	RASSF1	POT1	RAD51	USP47	PLOD1	RASSF10	OAT	SERPINB4			
PRKAA2	RASSF5	POU2F1	RBBP5	VAMP5	PML	RASSF9	OPTN	SETD7			
PRKCSH	RB1	POU5F1	RBBP6	VDAC1	POFUT1	RBBP4	P4HB	SFRP4			
PRMT1	RBBP6	PPEF1	RCHY1	VDAC2	POLR2A	RBFOX1	PABPC3	SIRT1			
PSMA5	RBM38	PPP2CA	RELA	WDR18	PPIA	RBM12B	PABPC4	SIRT2			
PSMA7	RCHY1	PPP6C	RFFL	WDR33	PPM1D	RBM22	PARK7	SIRT7			
PSMC4	RELA	PPP6C	RFWD2	WHSC1	PPM1G	RBM26	PCBP1	SKI			
PSMD1	RFPL2	PPP6R1	RFWD3	WRAP53	PPP2R4	RBMS1	PCMT1	SMAD7			

PYCARD	RFWD3	PPP6R1	RNF125	WRN	PRDX3	RBMS2	PDCD6IP	SMARCD2			
RAD18	RLIM	PPP6R2	RNF128	XRCC5	PRDX6	RCC1	PDIA3	SMEK1			
RAD50	RPL10A	PPP6R3	RNF2	YWHAZ	PRKCSH	RCC2	PDIA4	SNW1			
RAD51	RPL11	PRDX1	RNF38	ZNF251	PRKDC	RFC3	PDIA6	SOST			
RAN	RPL15	PRKAB2	RPA1	ZNF768	PSMB3	RFC4	PDXK	SPDL1			
RB1	RPL22L1	PRKDC	RPL11		PSMB4	RGPD3	PEBP1	SREBF1			
RBBP4	RPL23	PRPF8	RPL26		PTCD3	RIOK2	PFDN2	STAT3			
RBBP7	RPL26	PTER	RPL27		PTGES2	RNF2	PFN1	STK11			
RBBP8	RPL36A	RAD17	RPL4		QARS	RNF214	PGAM1	STK4			
RBL1	RPL37A	RAD21	RPL5		RAB11B	RNH1	PGK1	SUPT7L			
RBL2	RPL4	RANBP2	RPL7A		RAD17	RPAP2	PGM1	SUV39H1			
RCHY1	RPL5	RASSF1	RPL8		RAD50	RPL10	PGM5	SUZ12			
RELA	RPS14	RBBP8	RPLP0		RAD51	RPL14	PHF21A	TADA2B			
RFC1	RPS20	RBM25	RPS17		RBM19	RPL35	PHKA1	TAF2			
RFC2	RPS27	RECQL5	RPS2		RMDN1	RPS23	PITPNB	TAT			
RICTOR	RPS27A	RELA	RPS26		RNF115	RPS27L	PLS3	TBCCD1			
RPA1	RPS27L	REV	RPS3A		RNF168	RPS6	PMP2	TCEB3B			
RPGRIP1	RPS3	RFC2	RPS7		RNF2	RPS6KB2	POLR2E	THOC5			
RPL15	RPS6	RIPK4	SENP3		RNF8	RRBP1	PPA1	TLE1			
RPL21	RPS7	RMND5A	SET		RPL18	RSL1D1	PPIA	TMED10			
RPL23A	RRM1	RNF144A	SETD1A		RPL19	S100A7A	PPIAL4B	TNNI2			
RPL28	RRM2B	RPA1	SETD7		RPL23A	S100A9	PPM1A	TP53			
RPL3	RRP1	RPA2	SFN		RPL26L1	SAFB2	PPM1B	TP73			
RPL31	RSL1D1	RPA3	SIN3A		RPL37	SEC24C	PPP2CA	TPM4			
RPL34	RYBP	RPS11	SIN3B		RPL8	SFN	PPP2R1A	TRIM28			
RPL5	SENP3	RRM2	SIRT1		RPLP2	SH3RF2	PRDX1	TRMT6			
RPL7A	SETD7	RTCB	SIRT1		RPN2	SIPA1L1	PRDX2	TSC2			
RPLP2	SF3B3	RUVBL1	SIVA1		RPS10	SIRT6	PRDX4	TSPYL1			
RPS12	SFN	RUVBL1	SMAD2		RPS15	SMARCA1	PRDX5	TULP3			
RPS13	SHARPIN	RUVBL2	SMAD3		RPS16	SMC4	PRDX6	UBE2I			
RPS23	SIRT1	RYK	SMARCA4		RPS17	SMU1	PRKCSH	UFL1			
RPS24	SIRT2	SAP25	SMARCB1		RPS18	SND1	PRPH	UGDH			
RPS27	SIRT3	SCARNA22	SMG7		RPS2	SNRPF	PRR12	USP22			
RPS3A	SIRT6	SF3B6	SMN1		RPS27A	SNTB2	PSAP	USP37			
RPS6	SIRT7	SHC1	SMYD2		RPS28	SORBS1	PSMA2	USP9Y			
RPS7	SIVA1	SIRT6	SNAI1		RPS3	sorcin	PSMA6	UVSSA			
RPSA	SMARCA2	SIRT7	SOCS1		RPS3A	SPATS2L	PSMB6	VDR			
RUVBL1	SMG7	SKI	SOX4		RPS5	SPECC1L	PSMC1	VHL			
RUVBL2	SMURF1	SMARCA5	SP1		RPS6	SRI	PSMC2	VWA3B			
SETX	SNAI1	SMARCAD1	SQSTM1		RPS6KA3	SRRT	PSMC5	WDR70			
SF3A1	SNAI2	SNRPA	SRPK1		RPS9	SRSF3	PSMC6	WRN			

SKP2	SND1	SNW1	SRSF1		RUVBL1	SRSF4	PSMD11	YBX1			
SMAD3	SP1	SP1	STUB1		S100A4	STAT1	PSMD14	ZBTB38			
SMARCA2	SRC	SRF	SYVN1		SIRT7	STAU1	PSMD2	ZBTB7A			
SMARCA4	SURF2	SRP14	TADA3		SLC25A6	STAU2	PSMD4	ZEB1			
SMARCA5	SUV39H1	SRP54	TAF1		SMARCA2	STIP1	PSME2	ZMIZ1			
SMARCB1	SUZ12	SRSF10	TAF9		SMARCA4	STRAP	PTMS	ZNF263			
SMARCC1	TAF1	STAU1	TBP		SOD2	STX3	RAB21	ZNF346			
SMARCC2	TAT	SUMO2	TCF4		SRP72	SUGP2	RAB9A	ZNF354C			
SMC1A	TBP	TAT	TCP1		SS18L2	SUN1	RAP1A	ZNF514			
SMC3	TBRG1	TELO2	TFAP2A		SSB	SYCP3	RBM8A	ZNF550			
SNRNP200	TCAP	TERF1	TNFAIP3		SSBP1	SYNCRIP	RHOA	ZNF669			
SNX3	TERT	TERF1	TOP1		SSRP1	TARDBP	RNF19A	ZNF707			
SP1	TFIP11	TES	TOP2A		STK11	TCEA1	RPA3	ZNF777			
SPT4	TFRC	THEM6	TOPORS		STK4	TCP1	RPL30	ZNF785			
SRSF1	TOP1	THOC6	TP53		SUMF2	TIA1	RUVBL1	ZNF8			
SSB	TP53	THRA	TP53BP1		SUPT16H	TIAL1	RUVBL2	ZNHIT6			
SSFA2	TP53I3	THRB	TP53BP2		SUPT6H	TMA16	S100A11	ZYX			
SSRP1	TP53RK	THYN1	TP53INP1		SURF2	TMOD3	SAE1				
STAT1	TP63	TINF2	TP63		SYNCRIP	TNKS1BP1	SARS				
SUMO1	TP73	TMPO	TP73		TAF1C	TNPO1	SERPINH1				
SUPT16H	TPT1	TOP1	TRIM24		TAF5L	TNPO2	SMAD2				
TARS	TRIM13	TP53	TRIM27		TAT	TNRC6B	SND1				
TCEA2	TRIM25	TPR	TRIM28		TERF2	TOMM34	SOD1				
TCEANC	TRIM27	TPTE	TRIM39		TIAM2	TP53	SOD2				
TCP1	TRIM28	TRA2B	TRRAP		TIMELESS	TPM1	SPIN1				
TERF2	TRMT6	TRIM25	TSC22D1		TKT	TPM2	SPTBN1				
TLE4	TRP53	TTI1	TSC22D3		TMX1	TRIM21	SRP19				
TONSL	TSC22D3	TTI2	TTK		TONSL	TRIM25	SSB				
TOP1	TSG101	U2AF2	TUBA1C		TOPORS	TRIM56	SSR4				
TOP2A	TTF1	UBAC2	TUBB		TOR1A	TRIP6	STMN1				
TOPBP1	U19	UBE2I	TUBB2A		TOR1B	TUBA1C	STUB1				
TP53	U2AF2	UBXN6	TWIST1		TP53	TUBA3C	STXBP1				
TP53BP1	UBC	UL29	UBC		TP53BP1	TUBA4A	STXBP2				
TP63	UBE2A	USF1	UBD		TP53I3	TUBB3	SUMO2				
TPP2	UBE2D1	VCAM1	UBE2A		TRAF6	TUBB8	SYNE2				
TPX2	UBE2D2	VHL	UBE2I		TRAP1	TUFM	TAB1				
TUBB	UBE2D3	VPRBP	UBE2K		TRIM28	TXN	TAGLN2				
TUBG1	UBE2E2	VPU	UBE2N		TSSK6	U2AF1	TBCA				
TULP2	UBE2E3	WRN	UBE2Q1		TXNDC12	UBA1	TIAL1				
UBC	UBE2I	XPA	UBE3A		UBC	UBAP2	TIMM13				
UBE2D1	UBE2K	XRCC1	UBE4B		UPF1	UBAP2L	TKT				

UBE2D2	UBE2L3	XRCC4	UBR5		USP11	UIMC1	TPD52				
UBE2D3	UBE2M	XRCC5	UCHL1		USP17L2	UPF1	TPI1				
UBE2E1	UBE2N	XRCC6	UHRF1		VAT1	USP10	TPR				
UBE2E2	UBE4B	YBX1	UHRF2		VPR	USP5	TRAP1				
UBE2E3	UBTF	YWHAQ	UIMC1		VPS35	UTP18	TRIM28				
UBE2I	USO1	YY1	USP10		WRAP53	VASP	UBA1				
UBE2K	USP15	ZNF598	USP11		WRN	VCP	UBC				
UBE2L3	USP2	ZNF746	USP42		XPC	WDR36	UBE2M				
UBE2N	USP26		USP7		XRCC1	WWP2	UBE2N				
UBE2W	USP48		VCP		XRCC5	YARS	UBE3A				
UBXN1	USP7		VHL		XRCC6	YBX1	UCHL1				
UIMC1	VCP		VIF		YBX1	YBX3	VAT1				
USP2	VEGFA		VRK1		YBX2	YES1	VCP				
USP7	VIF		WRN		YBX3	YTHDF1	XRCC5				
VCP	WWOX		WWOX		YWHAB	YTHDF2	XRCC6				
WBSCR16	XIAP		XAF1		YWHAE	YTHDF3	YWHAB				
WDR5	XPC		YBX1		YWHAQ	YWHAB	YWHAG				
WWOX	XPO1		YWHAZ		YWHAZ	YWHAH	YWHAQ				
XIAP	XRCC6		YY1		ZBTB24	YWHAQ	YWHAZ				
XIST	YWHAQ		ZBTB17		ZC3HAV1	ZC3H14					
XRCC5	YY1		ZBTB33		ZCCHC9	ZC3H7A					
XRCC6	ZNF274		ZBTB7A		ZNF22	ZC3H7B					
YBX1	ZNF420		ZBTB8A		ZNF420	ZC3HAV1					
YWHAQ	ZNF777		ZMIZ1		ZNF574	ZNF638					
YWHAZ			ZNF420		ZSCAN25						
ZNF350											
