# Supplementary material for: The RXFP3 receptor is functionally associated with cellular responses to oxidative stress and DNA damage
Source: Aging (Albany NY). 2019 Dec 3;11(23):11268–313. doi: 10.18632/aging.102528 (PMC6932917; doi:10.18632/aging.102528)
Supplement: Supplementary Table 21 [file aging-11-102528-s017..pdf]

Table S21. GEN3VA disease signature lists. To investigate the potential role for RXFP3 in aging, we extracted three disease signatures from GEN3VA (GENE Expression and Enrichment Vector Analyzer; http://amp.pharm.mssm.edu/gen3va/): 'Aging'(found in GEN3VA under the name "Model of cerebral aging and Alzheimer's disease: temporal cortex"), 'Schizophrenia' (in GEN3VA "Schizophrenia: postmortem superior temporal cortex") and 'Aortic aneurysm' (in GEN3VA "Abdominal aortic aneurysm") the latter of which is used as a control, to assure the protein overlap between the GEN3VA signatures and our own RXFP3 interactome dataset is specific. GEN3VA is a web-based system enabling the integrative analysis of a collective of gene expression signatures identified and extracted from GEO. Allowing us to extract specific disease signatures, which we can compare to our own data.

Aging	Schizophrenia	Aortic aneurysm
ACTB	HBB	SOST
AFG3L2	PTGDS	KCNA5
AFTPH	SUZ12P1	FDCSP
AKAP11	PTPRO	MMP9
ALDOA	COX1	ADAMDEC1
ANK3	TUBB2A	MMP12
ANKRD17	FTL	TCEAL2
AP3S1	KIF5C	IGKV4-1
APP	COX2	MS4A1
ARF4	TUBB2B	C16orf54
ARHGAP21	CCNI	CXCL13
ARL3	PKD1P1	MRAP2
ARNTL	PEBP1	ITGA10
ARPC1A	NUMBL	MYOC
ARPC3	B2M	FOSB
ARPC5L	LOC493754	PLD5
ARPP19	MTURN	C10orf10
ARPP21	CNDP1	CD5L
ARX	TSPAN7	SCRG1
ASH1L	MAP2	IGLV1-36
ASPHD1	IGFBP7	IL1B

ATP1A3	RPSA	CCR7
ATP1B2	TAMM41	IGKV1OR2-108
ATP2B1	ALDOC	CYP4B1
ATP2B2	NDRG1	IGLV3-19
ATP5C1	GNB1	CD83
ATP5G1	CDK2AP1	CCL21
ATP5G2	DBI	TIMP4
ATP5G3	FAM161B	CCL4
ATP5J	CD63	IRF8
ATP5L	CD9	IL13RA2
ATP6AP2	SARAF	CLEC5A
ATXN1	CARNS1	PLN
ATXN1L	TUBB4A	FAIM3
ATXN7L3B	RPL41	IGLV2-23
BAI2	ATP6V0C	SUSD5
BASP1	CKB	CLEC4M
BCL11A	PTPRZ1	FAM46B
BEX1	ACTG1	SELL
BEX2	PPDPF	C15orf48
BHLHE22	TXNIP	CD52
BLCAP	FTH1P5	IGLJ3
BMI1	ITM2A	TRBC1
BRINP1	PHYHIP	CD2
BTF3	TF	OLR1
BZW2	SLC1A3	CASQ2
C15orf61	CAPNS1	CD27
C16orf52	RPL39	P2RX5
CA11	CCDC92	KBTBD8
CABP1	PMP2	ACADL
CACNB4	DDN	IGHM
CADM1	SEPW1	MYOCD
CADM2	ATP6V0E2	MZB1

CALM2	CNP	FBP1
CALM3	AHCYL1	LMOD1
CAMK2A	HMGN2	NPTX2
CAMK2G	ETNPPL	CXCL3
CAMK2N1	NCDN	TIMD4
CAMTA1	GDI1	IL2RG
CAMTA2	AQP4	EGR2
CAPZA2	CPLX1	TCL1A
CCNDBP1	ND2	CNTN3
CCT7	ATP6	HLA-DQA1
CCT8	GLDN	CD79A
CD81	COX6A1	BANK1
CDC42	RNASE1	LAMP3
CDKN1B	AATK	SASH3
CHCHD2	COX3	RASGRP1
CHMP2A	RPLP1	DPEP2
CHN1	APOE	PLEK
CITED2	NPIPA1	LINC01088
CLTA	LGALS1	CCL8
CLTC	LINC00844	P2RY13
CNKSR2	FAM107A	PVRIG
COPS5	SLC35E2B	VAT1L
COX7A2	IPCEF1	MXRA5
CPEB2	ERH	LDOC1
CRYAB	PRKCA	TMOD1
CSDE1	SERPINE2	PLA2G7
CSNK2B	TTC38	SLC7A5
CSPG5	NOM1	RRM2
CUL3	APLP1	PRUNE2
CXXC5	CRYM	LCK
DAZAP2	WASF3	RASL12
DCTN3	S100B	MMP1

DDRGK1	GSTM3	APOC1
DDX1	GSN	NAPSB
DDX3X	PAQR6	VPREB3
DDX5	GABRB2	RAC2
DLG2	GABBR1	CXCL8
DLGAP1	GATC	ACP5
DNAJC6	GFAP	IL7R
DPY30	PTN	HCAR3
DPYSL2	HSPB8	SLC14A1
DRG1	NDRG4	HSPB6
DSTN	UBC	STAP1
DYNC1I1	C1orf61	P2RY8
DYNC1LI2	PAIP2B	CCR5
DYNLL1	PLD3	CD180
EEF1A2	ZCCHC24	LYZ
EIF1B	GLTSCR2	NRIP3
EIF3D	IFITM2	REEP1
EIF3E	SLC1A2	BLNK
EIF3K	TNRC6C-AS1	GAPT
EIF4A2	MAL	IL2RB
EIF4G2	PRKCZ	GPR18
ELAVL2	SNHG6	RHOH
ELAVL4	B3GAT1	FHL5
ELMOD1	FAM127A	SLAMF8
ENPP2	PTMA	IGKV1-5
EPC2	TPI1	SLC12A8
EPHA4	GSTM1	NCKAP1L
ESRRG	SATB1	GREM1
EXOC3L2	MEST	MAOA
FAM131B	PRKACB	CSDC2
FAM155A	TMEM258	ITGA7
FAU	HPRT1	NCEH1

FBXL16	KLHL2	PNMAL1
FBXO11	RPL36	CD19
FBXW7	RPL35	CLEC4G
FEZ1	ITM2B	ICAM3
FIS1	PTP4A2	SH3BGR
FKBP1A	COX5A	IGLV1-40
FMR1	BRSK1	THEMIS2
FOLR1	SKP1	FMO2
FOXG1	FABP7	GPR183
FXYD7	PLLP	CD300LF
G3BP2	EPB41L4A-AS1	NLRC3
GABARAPL2	MYEOV2	RPS6KA1
GAD1	GSTM2	GALNT15
GADD45A	EIF1	BTK
GAP43	CASC3	MPEG1
GGNBP2	NISCH	CCR1
GJA1	DAAM2	TMEM35
GLUL	KIAA1598	AMICA1
GNAO1	ENC1	CD37
GNAS	PFDN5	GPR65
GPM6A	SPARCL1	FABP4
GPR158	SOX2-OT	CD69
GPRASP2	NQO1	FPR3
GRIA2	PPM1K	PLA2G2D
GTF2B	ABHD17A	TRAC
H2AFZ	KIF5A	IL10RA
HIF1A	KIAA0430	ISG20
HINT1	LOC100130429	STAC
HINT2	DYNC1I2	CD3D
HMGB1	COLCA1	HMHA1
HMGCS1	CLSTN1	ITGA8
HMGN3	MOBP	DENND2D

HMP19	ANP32B	FRZB
HNRNPK	QDPR	NPR1
HOMER1	MBP	PDZRN4
HSD17B10	GABARAP	CFP
HSP90AA1	SOWAHA	ZNF385D
ID4	LRRC8A	TNFRSF17
IGDCC4	ALDH9A1	TREM2
IGFBP2	SDC4	BCL2A1
IGIP	CEBPD	DUSP4
IRF2BP1	PAQR8	STAT4
IRS2	NARS	CNN1
KCMF1	HYOU1	NTF3
KCNA2	PLP1	CKMT2
KCNK12	DGKZ	HEY2
KHDRBS3	FMNL2	CETP
KIAA0894	CLDN11	CCL2
KIF5B	SLC39A10	FIBIN
KLF9	RAP1GAP	MBNL1-AS1
KMT2E	SYNPR	C5orf46
KRAS	TMA7	SNTA1
LGR6	DGCR2	RAMP1
LHX2	ZNF665	LILRB2
LINC00461	ANKRD37	MNDA
LINC00520	ATP9A	COL11A1
LMO4	APOBEC3C	CD86
LOC101929897	NPTXR	SYK
LPGAT1	SDCBP	FIGF
LPPR4	SPCS1	IGSF6
LRRC4B	FTH1	TMEM163
LRRN3	CCDC13	NCF2
LZTS3	MBOAT7	CSF2RB
MAGED1	SCG5	BIN2

MAGI2	CCDC152	IGFBP6
MAP1B	TNS3	TREM1
MAPK6	CHCHD10	ITK
MARCKS	RB1CC1	AIM1
MARCKSL1	SRGN	CAPG
MATR3	COX8A	PTGS2
MDH1	PODNL1	EVI2B
MEF2A	PSD3	VIT
MEF2C	OPHN1	IGJ
MEIS2	STX1B	CHMP4C
MESDC1	CBX7	EFHD1
MICAL2	BEX4	TMEM246
MINK1	OPALIN	ANGPTL1
MMADHC	OAZ1	PLAUR
MPPED2	Septin 7	SYTL1
MPV17L	CCK	CRISPLD1
MRFAP1	FGF13	NTS
MRPL33	UGP2	AOAH
MT1F	ACTR10	FERMT3
MT1G	STX1A	SNX10
MT1H	ABAT	DENND1C
MT1HL1	GABRB1	OSR1
MT1X	PKP4	LTF
MT2A	TUBA3C	ADIRF
MXI1	PPP2CB	SLC29A3
MYCBP2	PDE2A	PIK3AP1
MYT1L	KLK6	FBLN5
NAA20	NPC2	SLN
NAE1	ARL6IP1	NXPH3
NAP1L1	PHACTR3	MMP7
NAV3	USP11	TCEAL7
NBEA	BEX5	PPP1R3C

NCK2	CYB5R3	GZMB
NCKAP1	APC	FCRLA
NDFIP1	CST3	KMO
NDRG2	WIF1	IGLL3P
NDUFA12	ITM2C	LGALS9
NDUFA6	EPHX1	TBC1D10C
NDUFB2	COX7C	FCN1
NDUFB8	MALAT1	PMAIP1
NDUFB9	MOG	PTPRCAP
NDUFS2	HLA-A	LY86
NDUFS3	HLA-DPA1	KIAA0226L
NDUFV1	DDR1	AGTR1
NDUFV2	POLR2L	CORO1A
NEDD8	SQSTM1	GPR160
NEFH	SNCB	LYN
NEFL	MAG	MX2
NEFM	COPG2IT1	RERG
NELL2	TSPAN3	UCP2
NFKBIA	UBQLN4	DHRS9
NGFRAP1	MZT2A	GPSM3
NKAIN2	LOC100190986	IFI30
NKRF	UGT8	CNTN4
NLK	C20orf202	HMOX1
NME7	LOC100289333	FXYD1
NNAT	PUM2	RASSF2
NPM1	PTTG1IP	LCP1
NPTN	TMEM14C	ANGPTL7
NR2E1	GPRASP1	PYGM
NR2F2	ATP5E	CFL2
NRGN	GPR37	FCGR2B
NRXN1	NDUFAB1	GZMK
NUAK1	CTSH	HAS1

NUCKS1	FAM213A	CPNE5
OCIAD2	DDX59	CDO1
OLFM1	FUT9	AQP9
OLFM3	LGI1	POU2AF1
OPCML	TM2D3	FNDC1
OXR1	E2F4	ZBED8
P4HTM	AGAP1	LINC00936
PABPC1	CMTM5	NCF1C
PABPN1	SPTAN1	MYO1F
PACSIN1	HLA-DRB1	TLR1
PAIP2	EDIL3	CSK
PAM	DDAH1	LINC01094
PCDH10	KIF3C	NPNT
PCDH7	TEF	HLA-DQB1
PCDH8	SRI	SGCA
PCDH9	TPD52L1	SLC16A6
PCLO	MGST1	CHST2
PCMT1	HMGN1	GADD45G
PCSK1	RHOU	LRCH2
PCSK1N	TSPYL1	HS3ST2
PDCD10	C18orf32	PRKX
PEA15	LAMP1	CR2
PFDN2	Septin 2	PPL
PFN2	INPP1	BIRC3
PGAM1	CIRBP	RASSF5
PGP	NAPB	ITGA4
PHACTR1	LMO3	SOD3
PHYHIPL	BANF1	ACTC1
PIAS1	ADIPOR2	IGLC1
PJA2	PRKACA	AKR1C3
PLCB1	DNAJB1	SLC37A2
PLCB4	HLA-DRB6	HOXA4

PLK2	HLA-F	SIGLEC10
PLTP	F3	C5AR1
PPA1	SEPP1	NEXN
PPAP2B	NEK7	IGLV4-60
PPIA	KIF1A	IBSP
PPM1B	WBP2	CD24
PPP2CA	MSMO1	CTSS
PPP2R2B	BAG3	NFKBIZ
PPP3CB	COCH	LILRB1
PPP6C	ATP6V1E1	SPAG4
PRKCB	RIMS3	HSPB2
PRKCDBP	MLF2	CECR1
PRKCI	TSPYL4	PIM2
PRNP	ENOPH1	RUNX3
PRPF8	FUT8-AS1	MYL9
PRR12	PRRT2	LINC01215
PSMA1	CBX6	VAV1
PSMA2	DPP6	PRR16
PSMA6	PGRMC1	AGT
PSMB3	COX7A2L	CCL5
PSMB4	DMXL2	MIR143HG
PSMB7	RANBP1	PDE8B
PSMC4	MAPRE3	TNFRSF21
PSMD1	DCTN1	KLRB1
PSMD11	DKK3	SALL2
PSMD12	CCDC115	KCNN4
PSMD14	CYCS	PCDHB10
PSMD2	SNPH	SCO2
PSME2	ATL1	SUCNR1
PTGES3	RASEF	KIAA0895
PTPRD	SAP18	SYNC
PUM1	SLC25A22	CSF1R

PURA	SLC6A17	FRY
PURB	HIPK2	CD72
RAB10	RABAC1	ITGB2-AS1
RAB14	ENHO	ZC3H12A
RAB24	CX3CR1	IL4I1
RAB2A	MAN1B1-AS1	NLGN1
RAB6A	MTCH1	LOC100505774
RAD21	PIP4K2A	AGPAT9
RALYL	C3	OMD
RAN	TRIM44	DUSP2
RANBP9	EIF2A	NCF4
RAP1B	ARHGEF3	RGS18
RAPGEF2	NDUFS7	PRIMA1
RBBP7	RGCC	SNORA28
RBFOX1	ADCY1	WFDC1
RBFOX3	GRINA	CD3E
RBX1	CALM1	HCK
RFX3	IGSF8	OSCAR
RGS4	JUND	ARHGAP9
RHOB	ZNF721	S100A10
RIMS1	BNIP3	NOX4
RIMS2	SLC20A1	FHL1
RNASEK	HIST1H4C	CCRL2
RNF103	NET1	CYTIP
RNF111	CDIPT	RASAL3
RNF113A	CAMKK2	TYROBP
RNF146	CAP2	SLC31A2
RNF220	KCNJ4	OSBPL3
RORA	NDUFC2	PAG1
RPL0	FGF1	CXCR4
RPL17	ATP1A1	PNOC
RPL19	EEF2	KDM6B

RPL26L1	HSPA1A	DUSP10
RPL28	HSPB1	TBXAS1
RPL3	PRDX1	RAB9B
RPL30	RPL22	PXMP2
RPL32	RPL31	LEFTY2
RPL36A	RPLP2	ITGAL
RPL36AL	VIM	LMNB1
RPL37A	ARF3	FAM65B
RPS10	DDX17	NFE2L3
RPS18	PKM	CXCL16
RPS19	RPL26	TMEM237
RPS21	SCD	CEBPA
RPS23	ACTB	SBSPON
RPS24	ATP5B	CYP4X1
RPS25	CFL1	ANXA3
RPS29	EEF1A1	ADRA2C
RPS3A	GAPDH	CD1C
RPS4Y1	H3F3B	SCARA3
RPS7	LDHB	RGS19
RRAGA	NACA	GP1BA
RTN1	RPL23A	SDC1
RTN4	RPL24	SLC7A7
RUNDC3A	RPS12	HSPB7
RYR2	RPS6	MYH11
SATB2	RPS8	PDLIM3
SCAF8	TUBA1C	ZNRF3
SCG2	TUBB	SLC16A10
SCN2A	TUBB4B	AIM2
SEC14L1	YWHAQ	ANKRD35
SEC61B	YWHAZ	MAPK13
SELM	EEF1G	LINC00597
SEMA5B	RPL5	NR4A2

Septin 3	RPL9	CCL20
SERPINI1	RPS16	Mar-01
SGK1	SLC25A6	CSRP2
SGMS1	RPL10A	MFAP4
SHANK3	RPL14	LY9
SLAIN1	DNAJA1	FGR
SLC12A5	HSPA8	LTB
SLC17A7	RPL11	KLHL13
SLC24A3	RPL13	WTIP
SLC25A12	RPL34	NAP1L3
SLC25A3	RPL4	FYB
SLC25A5	RPL6	SHISA4
SLC35G2	RPL7A	VAMP8
SLC38A2	RPL8	PTPRD-AS1
SLC4A4	RPS14	CES1
SLC6A1	RPS3	DUSP27
SLTM	RPS4X	RERGL
SNAP25	RPS9	TMEM255A
SNAP91	MYL6	CDCA7L
SNRNP27	RPL13A	NTRK3
SNRPA	RPL21	NPY1R
SOD1	RPL23	TMEM47
SON	RPL7	PPIF
SOX4	RPS11	SLAMF7
SPATA24		CP
SPIN1		CD1D
SPTBN1		ARRB2
SRSF5		WISP2
SST		HOGA1
STAC2		HLA-DMB
STK39		EPSTI1
STMN2		SLC15A3

STXBP1		CDH3
SUMO1		COTL1
SUMO2		CHIT1
SV2A		TEAD3
SYBU		GPX3
SYNDIG1		FBLIM1
SYT1		MLIP
TAGLN3		FCRL1
TBCB		FCGR1B
TBPL1		GPR171
TCF4		IL6
TENM1		CD8A
THRB		APOBEC3A
TJP1		MYOZ2
TMEM147		CPED1
TMEM170B		IGKC
TMSB10		TNFSF11
TPT1		C3AR1
TSG101		TMEM71
TTC3		SLPI
TTC7B		PCOLCE2
TTYH1		RAB7B
TUBA1A		LGMN
TUBA1B		RGS5
TUBA4A		CRIP2
TUBB3		MRVI1
TXNL1		LAMA2
UBA1		TLR7
UBA2		TMEM61
UBB		MRGPRF
UBE2E2		GPR84
UBE4A		BAMBI

UBL5		GK
UBQLN2		RRAS
UBR3		LSP1
UQCRC1		HS3ST3A1
USMG5		SEMA4D
UXT		MAP4K1
VAMP2		RGN
VAPA		CRLF1
VDAC3		TMEM100
VSNL1		C12orf75
WAC		RBP7
WASF1		CYTL1
WASL		MYOM1
YPEL3		MICU3
YTHDF3		AOX1
YWHAE		CHODL
YWHAG		PDGFD
YWHAH		ITPR1-AS1
ZBTB16		NFKBIE
ZBTB18		CDH13
ZCRB1		GMFG
ZDBF2		TMEM52B
ZEB2		C8orf88
ZFAND5		NLRC5
ZFPM2		ADAP2
ZNF281		SH2B3
ZNF385A		ADRBK2
ZNF423		HK3
ZRANB2		C9orf91
ZSWIM6		ARHGEF17
		PARM1
		SIGLEC15

		HVCN1
		CLEC4A
		GALNT6
		TAGLN
		CTSG
		PARP12
		STX11
		LTBP1
		FAM83D
		MYEF2
		MAN2B1
		SGK1
		PFN2
		LGR6
		NELL2
		SELM
		IGFBP2
		BCL11A
		MYL6
		RPL13A
		RPL21
		RPL23
		RPL7
		RPS11
		DNAJA1
		HSPA8
		RPL11
		RPL13
		RPL34
		RPL4
		RPL6
		RPL7A

		RPL8
		RPS14
		RPS3
		RPS4X
		RPS9
		RPL10A
		RPL14
		PHGDH
		CHI3L1
		SPP1
		HILPDA
		RCAN2
		CA2
		LAPTM5
		ATP1A2
		PPP1R14A
		PLAC8
		MIR100HG
		CRYAB
		ZBTB16
		ACTB
		ATP5B
		CFL1
		EEF1A1
		GAPDH
		H3F3B
		LDHB
		NACA
		RPL23A
		RPL24
		RPS12
		RPS6

		RPS8
		TUBA1C
		TUBB
		TUBB4B
		YWHAQ
		YWHAZ
		EEF1G
		RPL5
		RPL9
		RPS16
		SLC25A6
		ATP1A1
		EEF2
		HSPA1A
		HSPB1
		PRDX1
		RPL22
		RPL31
		RPLP2
		VIM
		ARF3
		DDX17
		PKM
		RPL26
		SCD
