# Supplementary material for: The RXFP3 receptor is functionally associated with cellular responses to oxidative stress and DNA damage
Source: Aging (Albany NY). 2019 Dec 3;11(23):11268–313. doi: 10.18632/aging.102528 (PMC6932917; doi:10.18632/aging.102528)
Supplement: Supplementary Table 22 [file aging-11-102528-s019..pdf]

Table S22. GeneIndexer interrogation of RXFP3 'control' interactome. Latent Semantic Indexing (LSI)-based informatic platform GeneIndexer (https://geneindexer.com/) is able to measure the degree of association of biomedical gene symbol identifiers of proteins with input interrogator concept terms through unbiased Cosine Similarity Score analysis. We cross-interrogated the obtained RXFP3 'control' interactome dataset, with the following age-related syntactic concepts (Aging): *Neurodegeneration (ND), Cognitive impairment (CI), Senescence (S), Parkinson's Disease (PD), Amyotrophic lateral sclerosis (ALS), Alzheimer's Disease (AD)*; and non-age-related terms (non-Aging): *Tuberculosis (TB), Spina Bifida (SB), Asthma (A), Tourette syndrome (TS), ADHD, Achondroplasia (AP)*. GeneIndexer extracts all gene-to-word relationships from the literature using LSI, which we then averaged per interrogator term and per interrogator group, *i.e.* '*Aging*' vs. '*non-Aging*'. Here a cosine similarity score larger than 0.2 typically specifies an explicit association, while a score lower than 0.2 indicates an implied relationship, a cutoff score was set at 0.1.

	**Aging**						**Non-Aging**					
Gene	**ND**	**CI**	**AD**	**S**	**PD**	**ALS**	**TS**	**AP**	**A**	**ADHD**	**TB**	**SB**
PHB	0.150	0.118	0.146	0.203	0.000	0.000	0.000	0.000	0.000	0.000	0.000	0.000
PSMB6	0.138	0.000	0.107	0.000	0.156	0.000	0.000	0.000	0.000	0.000	0.169	0.000
HNRNPA2B1	0.123	0.111	0.000	0.100	0.000	0.000	0.000	0.000	0.000	0.000	0.000	0.000
RXFP3	0.111	0.105	0.105	0.000	0.000	0.000	0.000	0.000	0.000	0.000	0.000	0.000
PSMA5	0.200	0.150	0.272	0.000	0.000	0.000	0.000	0.000	0.000	0.000	0.000	0.000
RPL13A	0.106	0.124	0.000	0.000	0.000	0.000	0.000	0.000	0.000	0.000	0.144	0.000
RPL14	0.101	0.000	0.000	0.000	0.000	0.000	0.000	0.000	0.000	0.000	0.000	0.000
DNAJA1	0.221	0.000	0.000	0.000	0.000	0.000	0.000	0.000	0.000	0.000	0.000	0.000
RPL4	0.124	0.127	0.000	0.000	0.000	0.000	0.000	0.000	0.000	0.000	0.000	0.000
RPL21	0.000	0.159	0.106	0.000	0.000	0.000	0.000	0.000	0.000	0.000	0.000	0.000
RPL11	0.000	0.000	0.000	0.153	0.000	0.000	0.000	0.000	0.000	0.000	0.000	0.000
RPL23	0.000	0.000	0.000	0.103	0.000	0.000	0.000	0.000	0.000	0.000	0.000	0.000
MAP4	0.141	0.000	0.000	0.000	0.000	0.000	0.000	0.000	0.000	0.000	0.000	0.000
RPS26	0.131	0.000	0.000	0.000	0.000	0.000	0.000	0.000	0.000	0.000	0.000	0.000
RPL34	0.108	0.000	0.000	0.000	0.000	0.000	0.000	0.000	0.000	0.000	0.000	0.000
HSPA8	0.136	0.000	0.000	0.000	0.000	0.000	0.000	0.000	0.000	0.000	0.000	0.000
TCP1	0.000	0.000	0.000	0.000	0.000	0.000	0.000	0.000	0.000	0.000	0.000	0.102
RPLP0	0.000	0.000	0.000	0.000	0.000	0.000	0.000	0.000	0.000	0.000	0.000	0.173

**Average CSS**	0.099	0.050	0.041	0.031	0.009	0.000	0.000	0.000	0.000	0.000	0.017	0.015
**Average CSS**	0.038						0.005					
