# Supplementary material for: The RXFP3 receptor is functionally associated with cellular responses to oxidative stress and DNA damage
Source: Aging (Albany NY). 2019 Dec 3;11(23):11268–313. doi: 10.18632/aging.102528 (PMC6932917; doi:10.18632/aging.102528)
Supplement: Supplementary Table 23 [file aging-11-102528-s015..pdf]

Table S23. GeneIndexer interrogation of RXFP3 'control' interactome. Latent Semantic Indexing (LSI)-based informatic platform GeneIndexer (https://geneindexer.com/) is able to measure the degree of association of biomedical gene symbol identifiers of proteins with input interrogator concept terms through unbiased Cosine Similarity Score analysis. We cross-interrogated the obtained RXFP3 'stress' interactome dataset, with the following age-related syntactic concepts (Aging): *Neurodegeneration (ND), Cognitive impairment (CI), Senescence (S), Parkinson's Disease (PD), Amyotrophic lateral sclerosis (ALS), Alzheimer's Disease (AD)*; and non-age-related terms (non-Aging): *Tuberculosis (TB), Spina Bifida (SB), Asthma (A), Tourette syndrome (TS), ADHD, Achondroplasia (AP)*. GeneIndexer extracts all gene-to-word relationships from the literature using LSI, which we then averaged per interrogator term and per interrogator group, *i.e.* '*Aging*' vs. '*non-Aging*'. Here a cosine similarity score larger than 0.2 typically specifies an explicit association, while a score lower than 0.2 indicates an implied relationship, a cutoff score was set at 0.1.

	**Aging**						**Non-Aging**					
Gene	**ND**	**CI**	**S**	**PD**	**ALS**	**AD**	**SB**	**A**	**TS**	**TB**	**AP**	**ADHD**
**PPT1**	0.288	0.248	0.000	0.155	0.122	0.210	0.000	0.000	0.000	0.000	0.000	0.000
**FUS**	0.294	0.188	0.000	0.152	0.418	0.141	0.000	0.000	0.000	0.000	0.000	0.000
**NDUFV2**	0.274	0.281	0.000	0.127	0.000	0.215	0.000	0.000	0.000	0.000	0.000	0.129
**PSMC3**	0.249	0.113	0.113	0.245	0.000	0.102	0.000	0.000	0.000	0.000	0.000	0.000
**TUBB**	0.264	0.107	0.000	0.116	0.000	0.112	0.000	0.000	0.000	0.000	0.000	0.000
**PSMC4**	0.293	0.121	0.000	0.233	0.000	0.123	0.000	0.000	0.000	0.000	0.000	0.000
**RPL24**	0.103	0.124	0.105	0.000	0.111	0.000	0.125	0.000	0.000	0.000	0.000	0.000
**WARS**	0.184	0.183	0.000	0.111	0.000	0.162	0.000	0.119	0.000	0.000	0.000	0.000
**AIMP1**	0.113	0.134	0.000	0.136	0.000	0.000	0.000	0.120	0.000	0.164	0.000	0.000
**PSMA6**	0.157	0.160	0.138	0.000	0.000	0.159	0.000	0.000	0.000	0.000	0.000	0.000
**PHB**	0.150	0.118	0.203	0.000	0.000	0.146	0.000	0.000	0.000	0.000	0.000	0.000
**PSMA7**	0.196	0.117	0.000	0.233	0.000	0.136	0.000	0.000	0.000	0.000	0.000	0.000
**NDUFS3**	0.147	0.236	0.000	0.110	0.000	0.121	0.000	0.000	0.000	0.000	0.000	0.000
**DARS**	0.165	0.101	0.000	0.153	0.000	0.000	0.000	0.000	0.103	0.000	0.000	0.000
**NEFL**	0.186	0.130	0.000	0.000	0.194	0.000	0.147	0.000	0.000	0.000	0.000	0.000
**PSMB6**	0.138	0.000	0.000	0.156	0.000	0.107	0.000	0.000	0.000	0.169	0.000	0.000
**C1QBP**	0.000	0.186	0.129	0.000	0.000	0.162	0.000	0.000	0.000	0.189	0.000	0.000
**ARF4**	0.103	0.140	0.158	0.000	0.000	0.000	0.000	0.000	0.000	0.000	0.000	0.000

**HNRNPA2B1**	0.123	0.111	0.100	0.000	0.000	0.000	0.000	0.000	0.000	0.000	0.000	0.000
**EEF2**	0.136	0.128	0.101	0.000	0.000	0.000	0.000	0.000	0.000	0.000	0.000	0.000
**RARS**	0.113	0.117	0.000	0.107	0.000	0.000	0.000	0.000	0.000	0.000	0.000	0.000
**TUBA1C**	0.154	0.102	0.000	0.106	0.000	0.000	0.000	0.000	0.000	0.000	0.000	0.000
**TUBB4B**	0.189	0.101	0.000	0.170	0.000	0.000	0.000	0.000	0.000	0.000	0.000	0.000
**GAPDH**	0.136	0.104	0.000	0.000	0.000	0.000	0.000	0.000	0.000	0.000	0.000	0.000
**ARPC5L**	0.107	0.120	0.000	0.000	0.000	0.000	0.000	0.000	0.000	0.000	0.000	0.000
**TOMM22**	0.168	0.000	0.000	0.103	0.134	0.000	0.000	0.000	0.000	0.000	0.000	0.000
**SNRPD3**	0.101	0.113	0.000	0.000	0.109	0.000	0.000	0.000	0.000	0.000	0.000	0.000
**NDUFS1**	0.265	0.000	0.000	0.313	0.000	0.103	0.000	0.000	0.000	0.000	0.000	0.000
**PSMB1**	0.104	0.000	0.000	0.000	0.000	0.101	0.000	0.000	0.000	0.000	0.000	0.000
**RXFP3**	0.111	0.105	0.000	0.000	0.000	0.105	0.000	0.000	0.000	0.000	0.000	0.000
**HNRNPH3**	0.111	0.110	0.000	0.000	0.000	0.105	0.000	0.000	0.000	0.000	0.000	0.000
**PSMA5**	0.200	0.150	0.000	0.000	0.000	0.272	0.000	0.000	0.000	0.000	0.000	0.000
**TUFM**	0.127	0.147	0.000	0.000	0.000	0.124	0.000	0.000	0.000	0.000	0.000	0.000
**MAT2A**	0.000	0.137	0.000	0.000	0.000	0.218	0.218	0.000	0.000	0.000	0.000	0.000
**RPL13A**	0.106	0.124	0.000	0.000	0.000	0.000	0.000	0.000	0.000	0.144	0.000	0.000
**DDX5**	0.134	0.000	0.144	0.000	0.000	0.000	0.000	0.121	0.000	0.000	0.000	0.000
**RPL22**	0.000	0.000	0.336	0.000	0.000	0.000	0.000	0.000	0.265	0.000	0.000	0.279
**EMC3**	0.000	0.136	0.000	0.000	0.000	0.000	0.000	0.000	0.142	0.000	0.000	0.102
**ACP1**	0.000	0.133	0.000	0.000	0.000	0.000	0.000	0.000	0.107	0.000	0.000	0.135
**SCD**	0.000	0.114	0.000	0.000	0.000	0.119	0.000	0.000	0.000	0.000	0.234	0.000
**LMAN1**	0.000	0.000	0.000	0.000	0.000	0.105	0.000	0.000	0.000	0.240	0.212	0.000
**PSMC2**	0.131	0.000	0.106	0.000	0.000	0.000	0.000	0.000	0.000	0.000	0.000	0.000
**NACA**	0.104	0.000	0.103	0.000	0.000	0.000	0.000	0.000	0.000	0.000	0.000	0.000
**ARL2**	0.144	0.000	0.190	0.000	0.000	0.000	0.000	0.000	0.000	0.000	0.000	0.000
**HIST1H1D**	0.116	0.000	0.116	0.000	0.000	0.000	0.000	0.000	0.000	0.000	0.000	0.000
**PSMA3**	0.135	0.000	0.124	0.000	0.000	0.000	0.000	0.000	0.000	0.000	0.000	0.000
**HSPA9**	0.120	0.000	0.160	0.000	0.000	0.000	0.000	0.000	0.000	0.000	0.000	0.000
**VDAC2**	0.186	0.000	0.000	0.213	0.000	0.000	0.000	0.000	0.000	0.000	0.000	0.000
**TFG**	0.158	0.000	0.000	0.198	0.000	0.000	0.000	0.000	0.000	0.000	0.000	0.000
**TRAP1**	0.184	0.000	0.000	0.242	0.000	0.000	0.000	0.000	0.000	0.000	0.000	0.000
**SLC25A11**	0.130	0.000	0.000	0.239	0.000	0.000	0.000	0.000	0.000	0.000	0.000	0.000
**ERLIN2**	0.139	0.000	0.000	0.107	0.000	0.000	0.000	0.000	0.000	0.000	0.000	0.000

**ERLIN1**	0.147	0.000	0.000	0.141	0.000	0.000	0.000	0.000	0.000	0.000	0.000	0.000
**SNRPA1**	0.145	0.000	0.000	0.113	0.000	0.000	0.000	0.000	0.000	0.000	0.000	0.000
**PSMD2**	0.133	0.000	0.000	0.101	0.000	0.000	0.000	0.000	0.000	0.000	0.000	0.000
**HIST1H2AG**	0.120	0.000	0.000	0.000	0.000	0.000	0.000	0.000	0.000	0.000	0.000	0.000
**DNAJA1**	0.221	0.000	0.000	0.000	0.000	0.000	0.000	0.000	0.000	0.000	0.000	0.000
**DYNLRB1**	0.102	0.000	0.000	0.000	0.000	0.000	0.000	0.000	0.000	0.000	0.000	0.000
**RBFOX1**	0.000	0.144	0.103	0.000	0.000	0.000	0.000	0.000	0.000	0.000	0.000	0.000
**ARF3**	0.130	0.123	0.000	0.000	0.000	0.000	0.000	0.000	0.000	0.000	0.000	0.000
**ENO1**	0.112	0.119	0.000	0.000	0.000	0.000	0.000	0.000	0.000	0.000	0.000	0.000
**RPL4**	0.124	0.127	0.000	0.000	0.000	0.000	0.000	0.000	0.000	0.000	0.000	0.000
**NDUFA12**	0.140	0.105	0.000	0.000	0.000	0.000	0.000	0.000	0.000	0.000	0.000	0.000
**PABPC4**	0.000	0.000	0.105	0.000	0.166	0.000	0.000	0.000	0.000	0.000	0.000	0.000
**ATP5A1**	0.139	0.000	0.000	0.000	0.196	0.000	0.000	0.000	0.000	0.000	0.000	0.000
**SSR4**	0.146	0.000	0.000	0.000	0.436	0.000	0.000	0.000	0.000	0.000	0.000	0.000
**PRDX1**	0.118	0.000	0.000	0.000	0.137	0.000	0.000	0.000	0.000	0.000	0.000	0.000
**SOD1**	0.132	0.000	0.000	0.000	0.940	0.000	0.000	0.000	0.000	0.000	0.000	0.000
**UQCRC2**	0.130	0.000	0.000	0.000	0.400	0.000	0.000	0.000	0.000	0.000	0.000	0.000
**RPN1**	0.149	0.000	0.000	0.000	0.000	0.102	0.000	0.000	0.000	0.000	0.000	0.000
**RPL21**	0.000	0.159	0.000	0.000	0.000	0.106	0.000	0.000	0.000	0.000	0.000	0.000
**RPL19**	0.000	0.104	0.000	0.000	0.000	0.000	0.000	0.000	0.101	0.000	0.000	0.000
**H3F3B**	0.000	0.000	0.115	0.000	0.000	0.000	0.193	0.000	0.000	0.000	0.000	0.000
**NEFM**	0.000	0.000	0.000	0.000	0.112	0.000	0.213	0.000	0.000	0.000	0.000	0.000
**CDKN2A**	0.000	0.000	0.685	0.000	0.000	0.000	0.000	0.000	0.000	0.000	0.000	0.000
**POLR1C**	0.000	0.000	0.105	0.000	0.000	0.000	0.000	0.000	0.000	0.000	0.000	0.000
**TPM4**	0.000	0.000	0.125	0.000	0.000	0.000	0.000	0.000	0.000	0.000	0.000	0.000
**RPL26**	0.000	0.000	0.170	0.000	0.000	0.000	0.000	0.000	0.000	0.000	0.000	0.000
**NONO**	0.000	0.000	0.116	0.000	0.000	0.000	0.000	0.000	0.000	0.000	0.000	0.000
**ARL1**	0.000	0.000	0.160	0.000	0.000	0.000	0.000	0.000	0.000	0.000	0.000	0.000
**HNRNPK**	0.000	0.000	0.158	0.000	0.000	0.000	0.000	0.000	0.000	0.000	0.000	0.000
**HNRNPUL1**	0.000	0.000	0.452	0.000	0.000	0.000	0.000	0.000	0.000	0.000	0.000	0.000
**RPL23**	0.000	0.000	0.103	0.000	0.000	0.000	0.000	0.000	0.000	0.000	0.000	0.000
**MCM4**	0.000	0.000	0.172	0.000	0.000	0.000	0.000	0.000	0.000	0.000	0.000	0.000
**ELAVL1**	0.000	0.000	0.147	0.000	0.000	0.000	0.000	0.000	0.000	0.000	0.000	0.000
**RPL5**	0.000	0.000	0.129	0.000	0.000	0.000	0.000	0.000	0.000	0.000	0.000	0.000

**H1FX**	0.000	0.000	0.176	0.000	0.000	0.000	0.000	0.000	0.000	0.000	0.000	0.000
**HNRNPC**	0.000	0.000	0.105	0.000	0.000	0.000	0.000	0.000	0.000	0.000	0.000	0.000
**YBX1**	0.000	0.000	0.102	0.000	0.000	0.000	0.000	0.000	0.000	0.000	0.000	0.000
**RPS8**	0.000	0.000	0.103	0.000	0.000	0.000	0.000	0.000	0.000	0.000	0.000	0.000
**EWSR1**	0.000	0.000	0.109	0.000	0.000	0.000	0.000	0.000	0.000	0.000	0.000	0.000
**RPL11**	0.000	0.000	0.153	0.000	0.000	0.000	0.000	0.000	0.000	0.000	0.000	0.000
**PCBP1**	0.000	0.000	0.196	0.000	0.000	0.000	0.000	0.000	0.000	0.000	0.000	0.000
**LMNA**	0.000	0.000	0.106	0.000	0.000	0.000	0.000	0.000	0.000	0.000	0.000	0.000
**PSMC5**	0.112	0.000	0.000	0.000	0.000	0.000	0.000	0.000	0.000	0.000	0.000	0.000
**NTPCR**	0.106	0.000	0.000	0.000	0.000	0.000	0.000	0.000	0.000	0.000	0.000	0.000
**RPL34**	0.108	0.000	0.000	0.000	0.000	0.000	0.000	0.000	0.000	0.000	0.000	0.000
**YWHAZ**	0.100	0.000	0.000	0.000	0.000	0.000	0.000	0.000	0.000	0.000	0.000	0.000
**SNRPB**	0.103	0.000	0.000	0.000	0.000	0.000	0.000	0.000	0.000	0.000	0.000	0.000
**DNAJB6**	0.102	0.000	0.000	0.000	0.000	0.000	0.000	0.000	0.000	0.000	0.000	0.000
**NUP210**	0.104	0.000	0.000	0.000	0.000	0.000	0.000	0.000	0.000	0.000	0.000	0.000
**SMC3**	0.110	0.000	0.000	0.000	0.000	0.000	0.000	0.000	0.000	0.000	0.000	0.000
**SSBP1**	0.104	0.000	0.000	0.000	0.000	0.000	0.000	0.000	0.000	0.000	0.000	0.000
**PRDX6**	0.111	0.000	0.000	0.000	0.000	0.000	0.000	0.000	0.000	0.000	0.000	0.000
**PSMA1**	0.119	0.000	0.000	0.000	0.000	0.000	0.000	0.000	0.000	0.000	0.000	0.000
**NSF**	0.119	0.000	0.000	0.000	0.000	0.000	0.000	0.000	0.000	0.000	0.000	0.000
**PSMB2**	0.116	0.000	0.000	0.000	0.000	0.000	0.000	0.000	0.000	0.000	0.000	0.000
**HAX1**	0.148	0.000	0.000	0.000	0.000	0.000	0.000	0.000	0.000	0.000	0.000	0.000
**GET4**	0.138	0.000	0.000	0.000	0.000	0.000	0.000	0.000	0.000	0.000	0.000	0.000
**DYNLL1**	0.136	0.000	0.000	0.000	0.000	0.000	0.000	0.000	0.000	0.000	0.000	0.000
**PSMB5**	0.144	0.000	0.000	0.000	0.000	0.000	0.000	0.000	0.000	0.000	0.000	0.000
**RPS26**	0.131	0.000	0.000	0.000	0.000	0.000	0.000	0.000	0.000	0.000	0.000	0.000
**PSMD14**	0.132	0.000	0.000	0.000	0.000	0.000	0.000	0.000	0.000	0.000	0.000	0.000
**AIFM1**	0.151	0.000	0.000	0.000	0.000	0.000	0.000	0.000	0.000	0.000	0.000	0.000
**SLC25A6**	0.124	0.000	0.000	0.000	0.000	0.000	0.000	0.000	0.000	0.000	0.000	0.000
**BAG2**	0.132	0.000	0.000	0.000	0.000	0.000	0.000	0.000	0.000	0.000	0.000	0.000
**HSPA8**	0.136	0.000	0.000	0.000	0.000	0.000	0.000	0.000	0.000	0.000	0.000	0.000
**CCT6A**	0.000	0.000	0.000	0.000	0.000	0.000	0.000	0.000	0.000	0.000	0.000	0.000
**RPL28**	0.000	0.172	0.000	0.000	0.000	0.000	0.000	0.000	0.000	0.000	0.000	0.000
**PHGDH**	0.000	0.128	0.000	0.000	0.000	0.000	0.000	0.000	0.000	0.000	0.000	0.000

**CAPRIN1**	0.000	0.193	0.000	0.000	0.000	0.000	0.000	0.000	0.000	0.000	0.000	0.000
**G3BP1**	0.000	0.120	0.000	0.000	0.000	0.000	0.000	0.000	0.000	0.000	0.000	0.000
**PYGL**	0.000	0.101	0.000	0.000	0.000	0.000	0.000	0.000	0.000	0.000	0.000	0.000
**IGF2BP1**	0.000	0.128	0.000	0.000	0.000	0.000	0.000	0.000	0.000	0.000	0.000	0.000
**RPS24**	0.000	0.122	0.000	0.000	0.000	0.000	0.000	0.000	0.000	0.000	0.000	0.000
**YWHAQ**	0.000	0.000	0.000	0.000	0.115	0.000	0.000	0.000	0.000	0.000	0.000	0.000
**RPS6**	0.000	0.000	0.000	0.000	0.156	0.000	0.000	0.000	0.000	0.000	0.000	0.000
**NCLN**	0.000	0.000	0.000	0.000	0.000	0.283	0.000	0.000	0.000	0.000	0.000	0.000
**TCP1**	0.000	0.000	0.000	0.000	0.000	0.000	0.102	0.000	0.000	0.000	0.000	0.000
**FAU**	0.000	0.000	0.000	0.000	0.000	0.000	0.000	0.000	0.000	0.277	0.000	0.000
**NDUFA2**	0.000	0.000	0.000	0.000	0.000	0.000	0.000	0.000	0.000	0.319	0.000	0.000
**RPS2**	0.000	0.000	0.000	0.000	0.000	0.000	0.000	0.120	0.000	0.000	0.000	0.000
**ILF3**	0.000	0.000	0.000	0.000	0.000	0.000	0.000	0.108	0.000	0.000	0.000	0.000
**ILF2**	0.000	0.000	0.000	0.000	0.000	0.000	0.000	0.253	0.000	0.000	0.000	0.000
**Average CSS**	0.091	0.049	0.046	0.030	0.028	0.027	0.007	0.006	0.005	0.011	0.003	0.005
**Average CSS**	0.045						0.006					
