# Supplementary material for: The RXFP3 receptor is functionally associated with cellular responses to oxidative stress and DNA damage
Source: Aging (Albany NY). 2019 Dec 3;11(23):11268–313. doi: 10.18632/aging.102528 (PMC6932917; doi:10.18632/aging.102528)
Supplement: Supplementary Table 24 [file aging-11-102528-s020..pdf]

Table S24. RXFP3 constellation TOP 15 and contra-regulated proteins. TOP15 up (red) and down (green) regulated proteins, after 0.5µg and 5µg of RXFP3 overexpression. In addition, we have noted the contra-regulated proteins, which were upregulated in on cellular fraction, while being downregulated in another. Molecular function and Biological processes were identified using Uniprot (www.uniprot.org).

0.5µg RXFP3 TOP15 proteins (up vs down)

Gene Name	Protein name	Fraction	Biological process	Molecular function	Ratio
PFKP	ATP-dependent 6-phosphofructokinase, platelet type	Nucleus	Glycolysis	ATP binding	-0.933801233
UBE2A	Ubiquitin-conjugating enzyme E2 A	Cytoplasm	DNA repair	ubiquitin binding	-0.839495701
PAGR1	PAXIP1-associated glutamate-rich protein 1	Nucleus	DNA repair	estrogen receptor binding	-0.699972463
ABHD14B	Protein ABHD14B	Plasma membrane	positive regulation of transcription by RNA polymerase II	Hydrolase activity	-0.668326834
MTMR1	Myotubularin-related protein 1	Nucleus	phosphatidylinositol biosynthetic process	phosphatidylinositol-3-phosphatase activity	-0.621246409
MMS22L	Protein MMS22-like	Plasma membrane	DNA repair	double-strand break repair via homologous recombination	-0.613099215
FTSJ3	pre-rRNA processing protein FTSJ3	Cytoplasm	rRNA methylation	RNA binding	-0.597216129
TCOF1	Treacle protein	Nucleus	neural crest cell development	scaffold protein binding	-0.582391813
CDCA2	Cell division cycle-associated protein 2	Nucleus	Cell Cycle	recruitment of PPP1CC	-0.572116462
PEX16	Peroxisomal membrane protein PEX16	Nucleus	ER-dependent peroxisome organization	protein C-terminus binding	-0.542415816
C17orf85	Nuclear cap-binding protein subunit 3	Plasma membrane	defense response to virus	mRNA binding	-0.509221907
RAB35	Ras-related protein Rab-35	Cytoplasm	intracellular protein transport	GTPase activity	-0.483292704
POP1	Ribonucleases P/MRP protein subunit POP1	Nucleus	tRNA processing	RNA binding	-0.480293841

INIP	SOSS complex subunit C	Plasma membrane	DNA repair	cell-cycle checkpoint activation	-0.464897285
G3BP2	Ras GTPase-activating protein-binding protein 2	Nucleus	stress granule assembly	RNA binding	-0.443212682
HIST1H1D	Histone H1.3	Cytoplasm	regulation of transcription, DNA-templated	double-stranded DNA binding	1.69537665
HIST1H3A	Histone H3.1	Cytoplasm	telomere organization	nucleosomal DNA binding	1.29687801
HIST1H2BA	Histone H2B type 1-A	Nucleus	spermatogenesis, exchange of chromosomal proteins	DNA binding	0.98502167
NPC1	NPC intracellular cholesterol transporter 1	Nucleus	endocytosis	cholesterol binding	0.82207564
PDE7B	cAMP-specific 3',5'-cyclic phosphodiesterase 7B	Nucleus	G protein-coupled receptor signaling pathway	3',5'-cyclic-AMP phosphodiesterase activity	0.73215318
POTEKP	Putative beta-actin-like protein 3	Nucleus	platelet degranulation	ATP binding	0.72216339
NRDE2	Protein Nuclear RNAi defective-2 protein homolog	Cytoplasm	chromatin silencing by small RNA	meiotic cell cycle	0.71048568
SDHC	Succinate dehydrogenase cytochrome b560 subunit, mitochondrial	Nucleus	response to oxidative stress	succinate dehydrogenase activity	0.69611017
ALG9	Alpha-1,2-mannosyltransferase ALG9	Nucleus	dolichol-linked oligosaccharide biosynthetic process	mannosyltransferase activity	0.67601612
MEPCE	7SK snRNA methylphosphate capping enzyme	Nucleus	positive regulation of G1/S transition of mitotic cell cycle	RNA methyltransferase activity	0.66262566
HIST1H1B	Histone H1.5	Nucleus	negative regulation of DNA recombination	double-stranded DNA binding	0.65287147
RAB18	Ras-related protein Rab-18	Nucleus	brain development	GTPase activity	0.59045816
HIST1H1E	Histone H1.4	Plasma membrane	negative regulation of DNA recombination	double-stranded DNA binding	0.52578466

ZNF770	Zinc finger protein 770	Nucleus	Transcription regulation	DNA binding	0.49731068
HGS	Hepatocyte growth factor-regulated tyrosine kinase substrate	Cytoplasm	regulation of MAP kinase activity	protein domain specific binding	0.49643679

5μg RXFP3 TOP15 (Up vs Down)

Gene Name	Protein name	Fracton	Biological process	Molecular function	Ratio
PFKP	ATP-dependent 6-phosphofructokinase, platelet type	Nucleus	Glycolysis	ATP binding	-1.2301783
MMS22L	Protein MMS22-like	Plasma membrane	DNA repair	double-strand break repair via homologous recombination	-0.9509465
UBE2A	Ubiquitin-conjugating enzyme E2 A	Cytoplasm	DNA repair	ubiquitin binding	-0.8921860
MTMR1	Myotubularin-related protein 1	Nucleus	phosphatidylinositol biosynthetic process	phosphatidylinositol-3-phosphatase activity	-0.7301783
PRR14L	proline rich 14 like	Cytoplasm	age-related hearing impairment		-0.6456494
PAGR1	PAXIP1-associated glutamate-rich protein 1	Nucleus	DNA repair	estrogen receptor binding	-0.6339558
CDC73	Cell division control protein 73 - Parafibromin	Cytoplasm	Cell Cycle	RNA polymerase II complex binding	-0.6293653
PEX16	Peroxisomal membrane protein PEX16	Nucleus	ER-dependent peroxisome organization	protein C-terminus binding	-0.6256179
FTSJ3	pre-rRNA processing protein FTSJ3	Cytoplasm	rRNA methylation	RNA binding	-0.6115637
C17orf85	Nuclear cap-binding protein subunit 3	Plasma membrane	defense response to virus	mRNA binding	-0.5433843
ATXN2	Ataxin-2	Nucleus	stress granule assembly	RNA binding	-0.5270031
ENAH	Protein enabled homolog	Nucleus	axon guidance	actin binding	-0.5236093
EPN2	Epsin-2	Nucleus	endocytosis	lipid binding	-0.5171690
NEDD4	E3 ubiquitin-protein ligase NEDD4	Cytoplasm	negative regulation of transcription	proline-rich region binding	-0.5099110

			from RNA polymerase II promoter in response to UV-induced DNA damage		
UBQLN2	Ubiquilin-2	Cytoplasm	negative regulation of G protein-coupled receptor internalization	specifically reducing the rate at which receptor-arrestin complexes concentrate in clathrin-coated pits	-0.4868222
RXFP3	Relaxin-3 receptor 1	Cytoplasm	neuropeptide signaling pathway	G protein-coupled receptor activity	1.20897964
VTN	Vitronectin	Nucleus	cell migration	extracellular matrix binding	0.80193072
CA3	Carbonic anhydrase 3	Nucleus	response to oxidative stress	zinc ion binding	0.75535047
HIST1H1E	Histone H1.4	Plasma membrane	negative regulation of DNA recombination	double-stranded DNA binding	0.73601747
HIST1H1D	Histone H1.3	Cytoplasm	regulation of transcription, DNA-templated	double-stranded DNA binding	0.66586118
FKBP7	Peptidyl-prolyl cis-trans isomerase FKBP7	Nucleus	PPIase activity	calcium ion binding	0.61510583
KRT9	Keratin, type I cytoskeletal 9	Plasma membrane	spermatogenesis	structural constituent of cytoskeleton	0.59520963
HBB	Hemoglobin subunit beta	Nucleus	cellular oxidant detoxification	oxygen binding	0.58925226
LSM1	U6 snRNA-associated Sm-like protein LSm1	Plasma membrane	RNA metabolic process	RNA binding	0.55966673
AHCYL1	S-adenosylhomocysteine hydrolase-like protein 1	Nucleus	protein export from nucleus	RNA binding	0.55891345
DDX19A	ATP-dependent RNA helicase DDX19A	Nucleus	protein transport	ATP binding	0.54846789
HIST1H1B	Histone H1.5	Plasma membrane	negative regulation of DNA recombination	double-stranded DNA binding	0.52915313
UQCR10	Cytochrome b-c1 complex subunit 9	Nucleus	aerobic respiration	ubiquinol-cytochrome-c reductase activity	0.52880323

TPM3	Tropomyosin alpha-3 chain	Plasma membrane	actin filament organization	actin filament binding	0.49354763
BANF1	Barrier-to-autointegration factor	Cytoplasm	mitotic nuclear envelope reassembly	DNA binding	0.48193131

0.5μg RXFP3 contra-regulated proteins

Gene Name	Protein name	Fraction	Biological process	Molecular function	Ratio
GIG1	Tripeptidyl-peptidase 1	Cytoplasm	lysosome organization, nervous system development	peptidase activity	0.43317840
		Plasma membrane			-0.44428175
NOLC	Nucleolar and coiled-body phosphoprotein 1	Cytoplasm	mitotic cell cycle	DNA binding	0.29176850
		Nucleus			-0.25257723
RRBP1	Ribosome-binding protein 1	Cytoplasm	protein transport, osteoblast differentiation	RNA binding	0.40523133
		Nucleus			-0.28990827

5μg RXFP3 contra-regulated proteins

Gene Name	Protein name	Fraction	Biological process	Molecular function	Ratio
GPALPP1	GPALPP motifs-containing protein 1	Cytoplasm	Lipopolysaccharide-specific response protein	/	0.18859051
		Nucleus			-0.43296688
TXN2	Thioredoxin, mitochondrial	Plasma membrane	response to oxidative stress	protein disulfide oxidoreductase activity	0.74652276
		Nucleus			-0.51500787
UNC45A	Protein unc-45 homolog A	Nucleus	cell differentiation	cadherin binding	0.41764516
		Cytoplasm			-0.25480718
