# Supplementary material for: The RXFP3 receptor is functionally associated with cellular responses to oxidative stress and DNA damage
Source: Aging (Albany NY). 2019 Dec 3;11(23):11268–313. doi: 10.18632/aging.102528 (PMC6932917; doi:10.18632/aging.102528)
Supplement: Supplementary Table 25 [file aging-11-102528-s026..pdf]

Table S25. Disease continuum. To assess the potential role of RXFP3 in controlling aging-associated diseases, we employed an entirely unbiased analysis. We created a database of proteins, extracted from the entire human genome, explicitly and implicitly associated (via PubMed Central text mining using GeneIndexer (https://geneindexer.com/)) with input interrogator terms covering the majority of age-associated disease conditions. We termed this dataset of disease-associated proteins the 'Disease Continuum'. Abbreviations are as follows: Alzheimer's disease (AD); Amyotrophic Lateral Sclerosis (ALS); Liver disease (LD); Diabetes mellitus (DM); Dementia (D); Chronic obstructive pulmonary disease (COPD); Stroke (S); Chronic Kidney Disease (CKD); Frontotemporal dementia (FTD); Frontotemporal lobar degeneration (FTLD); Huntington's disease (HD); Chronic Heart Failure (CHF); Mild cognitive impairment (MCI); Neurodegeneration (N); Parkinson's disease (PD); Cancer (C).

Gene Symbol	**Alz**	**ALS**	**LD**	**DM**	**D**	**COPD**	**S**	**CKD**	**FTD**	**FTLD**	**HD**	**CHF**	**MCI**	**N**	**PD**	**C**
BDNF	0.214	0.105	0.403	0.125	0.389	0.0174	0.24	0.44	0.319	0.293	0.362	0.246	0.472	0.311	0.361	0.293
SNCAIP	0.113	0.144	0.293	0.425	0.164	0.119	0.176	0.226	0.15	0.247	0.354	0.597	0.358	0.267	0.304	0.113
TTBK2	0.386	0.115	0.188	0.214	0.201	0.105	0.138	0.261	0.236	0.17	0.401	0.219	0.232	0.303	0.315	0.214
GFAP	0.135	0.135	0.289	0.199	0.11	0.208	0.248	0.225	0.214	0.1677	0.221	0.433	0.369	0.33	0.193	0.214
SNAX1	0	0.17	0.236	0	0.21	0.153	0.19	0.276	0.19	0.134	0.496	0.199	0.259	0.477	0.394	0.2214
SAX2	0	0.249	0.28	0.221	0.23	0.219	0.115	0.292	0.226	0.418	0.165	0.165	0.105	0.295	0	0.35
CLN8	0	0.146	0.221	0.184	0.106	0.182	0.205	0.222	0.114	0.1778	0.39	0.351	0.29	0.267	0.358	0
ALS2	0.232	0.154	0.272	0.146	0.115	0.183	0.217	0.232	0.115	0.167	0.294	0.33	0.36	0.222	0	0
TOR1A	0	0.115	0.114	0.12	0.539	0.145	0	0.134	0.307	0.224	0.194	0.19	0.313	0.123	0.141	0.251
PSNP2	0.208	0.236	0.174	0.175	0.123	0	0.214	0.272	0.245	0.3144	0.162	0.17	0.154	0.262	0	0.135
MTOR	0.144	0	0.22	0.333	0.182	0.139	0.185	0.212	0.122	0.138	0.137	0.121	0.175	0.163	0.144	0.34
DARS2	0	0.152	0.267	0.224	0.139	0.154	0.144	0.25	0.111	0.1445	0.19	0.183	0.287	0.243	0	0.241
VPS13A	0	0	0.119	0.313	0.173	0.122	0.129	0.159	0.328	0.227	0.168	0.184	0.12	0.148	0.143	0.196
SPG11	0	0.125	0.324	0.245	0.168	0.161	0.128	0.149	0.222	0.17	0	0.142	0.11	0.175	0	0.373
FAAH	0.231	0.123	0.102	0	0.355	0.188	0.204	0.172	0.155	0.181	0.177	0	0.141	0.114	0	0.327
SCA9	0	0	0.107	0.111	0.102	0.186	0.129	0.103	0.277	0.323	0.123	0.259	0.154	0.11	0.143	0.339
CLN4	0	0.121	0.218	0	0.205	0.286	0.121	0.132	0.268	0.146	0	0.137	0.31	0.124	0.134	0.261
SAX1	0	0.11	0.106	0.248	0	0.332	0.16	0.125	0.121	0.236	0.146	0.145	0.191	0.127	0	0.337
ARF6	0	0	0.193	0.324	0.187	0.163	0.184	0.163	0.12	0.119	0.147	0.149	0.131	0.103	0.142	0.232
ATXN8OS	0	0	0.132	0.296	0.164	0.139	0.131	0.129	0.144	0.204	0.123	0.109	0.201	0.154	0.119	0.304
NHLRC1	0.222	0.164	0.1084	0.18	0.106	0.194	0.11	0.165	0.116	0.206	0.247	0	0.141	0.208	0	0.18
FPEVF	0	0.13	0.123	0.269	0.266	0.204	0.13	0.166	0.144	0.213	0	0.147	0.392	0	0	0.156
POLG	0	0	0.145	0.308	0.212	0.139	0.314	0.183	0.129	0.14	0.111	0.197	0.11	0.111	0	0.238
MAP1B	0.126	0.401	0.109	0.12	0.388	0	0	0.113	0.158	0.134	0	0.145	0.46	0.179	0	0
EPM2A	0.114	0.159	0.138	0	0.559	0.186	0.122	0.124	0.129	0.136	0.132	0	0.122	0.188	0	0.222

SCA21	0	0	0.138	0.111	0.196	0.173	0.182	0.147	0.18	0.187	0.15	0.137	0.183	0.117	0.142	0.282
ATXN2	0	0.121	0.118	0.243	0.211	0	0	0.113	0.208	0.176	0.103	0.305	0.112	0.248	0.119	0.248
SCA22	0	0	0.133	0.115	0.21	0.11	0.13	0.106	0.123	0.117	0.163	0.317	0.14	0.103	0.142	0.407
SERPINI1	0.106	0.249	0.134	0	0.541	0.286	0.121	0.106	0.172	0.168	0.105	0	0.187	0.104	0	0
SPAST	0	0.108	0.11	0.253	0.189	0.169	0.182	0.115	0.144	0.44	0	0.142	0.322	0.102	0	0
ATXN10	0	0	0.168	0.312	0.164	0.149	0.184	0.159	0.192	0	0.178	0.122	0.101	0.24	0.119	0.171
SCAX2	0	0	0.142	0.275	0	0.156	0.141	0.188	0	0.166	0.13	0.228	0.322	0.104	0.141	0.245
C10ORF2	0	0	0.121	0.307	0.185	0.105	0.183	0.105	0.161	0.143	0.109	0.109	0.13	0.312	0	0.263
PARK4	0	0.137	0.128	0	0.198	0	0	0.106	0.325	0.247	0.185	0.123	0.231	0.177	0.119	0.23
SCA26	0	0	0.103	0.115	0.128	0.187	0.129	0.108	0.158	0.161	0.101	0.128	0.151	0.294	0.141	0.298
SCA19	0	0	0.15	0.115	0.253	0.187	0.181	0.131	0.202	0.183	0.13	0.121	0.104	0.135	0.118	0.191
PLEKHG4	0	0	0.164	0.278	0.293	0	0.106	0.115	0.225	0.153	0.122	0.108	0.14	0.119	0.142	0.196
TRNK	0	0	0.125	0.111	0.268	0.22	0.129	0.141	0.121	0	0.173	0.157	0.113	0.112	0.134	0.343
ATXN7	0	0	0.138	0.274	0.217	0	0	0.104	0.239	0.198	0.114	0.122	0.291	0.15	0.119	0.181
ANE1	0	0.117	0.116	0.144	0.143	0.218	0.128	0.114	0.209	0.227	0	0.147	0.111	0.123	0	0.339
ATXN3	0	0.147	0.126	0.111	0.19	0	0	0.104	0.179	0.173	0.162	0.197	0.215	0.129	0.124	0.255
SPG7	0	0.13	0.159	0.266	0.307	0	0.156	0.11	0.11	0.182	0.183	0.197	0.13	0.177	0	0
SCA20	0	0	0.108	0.111	0.187	0.306	0.126	0.159	0	0.112	0.11	0.127	0.145	0.18	0.14	0.281
NDNF	0	0.108	0.35	0	0.182	0.286	0.121	0.208	0.124	0.115	0.165	0.197	0.112	0.122	0	0
SCA29	0	0	0.1	0.32	0.188	0.136	0.129	0.1	0.208	0.11	0.143	0.108	0.103	0.106	0.142	0.196
SCASI	0	0	0.1	0.288	0	0.154	0.141	0.135	0	0.143	0.188	0.157	0.253	0.155	0	0.372
SPG32	0	0.145	0.188	0.12	0.242	0.157	0.127	0.112	0.143	0.13	0.133	0.116	0.141	0.151	0	0.181
SLC1A2	0.134	0.175	0.124	0	0.228	0.286	0.1	0.107	0.219	0.111	0.157	0	0.275	0.15	0	0
CPAT1	0	0.121	0.174	0.24	0.208	0.218	0.129	0.166	0.102	0.324	0.106	0.131	0.134	0	0	0
CCAL1	0	0.417	0.121	0.23	0.123	0	0	0.11	0.184	0.202	0	0.11	0.162	0.101	0	0.288
SCA25	0	0	0.121	0.115	0.15	0.141	0.13	0.135	0.199	0.135	0.164	0.107	0.118	0.12	0.141	0.27
FRDA2	0	0	0.132	0.28	0.293	0.163	0.193	0.108	0	0	0.227	0.12	0.101	0.186	0.111	0.129
SPG30	0	0.121	0.103	0.248	0.157	0.128	0.127	0.199	0.198	0.13	0	0.155	0.106	0.16	0	0.201
SCAR7	0	0	0.13	0.282	0	0.18	0.167	0	0	0.159	0.169	0.113	0.305	0.136	0.124	0.264
ZNF828	0.1	0.16	0.401	0.108	0.22	0	0.117	0.105	0.106	0.129	0	0.337	0.123	0.119	0	0
MFSD8	0	0.125	0.125	0	0.349	0.171	0.121	0.168	0.182	0.127	0	0.138	0.117	0.401	0	0
SCAX1	0	0	0.16	0.296	0	0.205	0.145	0.187	0	0.139	0.215	0.113	0.137	0.144	0	0.278
CLAM	0	0.131	0.109	0.172	0.143	0.171	0.109	0.122	0.116	0.303	0	0.107	0.14	0.249	0	0.145
CLA3	0	0	0.124	0.278	0	0.323	0.145	0.117	0	0.141	0.107	0.181	0.126	0.106	0.108	0.245
LGI3	0.134	0.138	0.207	0	0.504	0.19	0.121	0.151	0.229	0.108	0	0	0.102	0.11	0	0
FCMTE2	0.128	0.167	0.138	0.119	0.345	0.109	0.127	0.107	0.131	0.14	0	0.101	0.163	0	0	0.218
TBCE	0.322	0.207	0.179	0.12	0.276	0	0	0	0.101	0.243	0.138	0.138	0.155	0.11	0	0
OPA3	0	0	0.116	0.281	0.168	0	0.133	0.182	0.149	0.16	0	0.221	0.288	0.109	0	0.175
TOR1B	0	0	0.22	0	0.177	0.169	0.125	0.112	0.201	0.189	0	0.144	0.17	0.207	0	0.232
PANK2	0	0	0.111	0.279	0.166	0	0.103	0.107	0.162	0.132	0.128	0.13	0.111	0.106	0	0.41
SEPT5	0	0.185	0.121	0	0.205	0	0	0.106	0.241	0.262	0.119	0	0.279	0.125	0	0.301

ERDA1	0	0	0.113	0.288	0.177	0	0	0.108	0.232	0	0.111	0.127	0.246	0.109	0.109	0.307
SMAN1	0	0.203	0.111	0.115	0.151	0	0	0.104	0.199	0.196	0.123	0.197	0.298	0.228	0	0
SPG19	0	0.191	0.109	0.266	0.201	0.146	0.13	0.22	0.103	0.136	0	0.17	0.115	0.134	0	0
KLHL8	0	0	0	0.158	0.26	0.162	0.198	0.177	0	0.218	0.127	0.148	0.114	0.114	0	0.233
SERF2	0	0.123	0.123	0.234	0.182	0	0	0.104	0.221	0.143	0.148	0	0.138	0.35	0.134	0
GABRD	0	0.355	0.189	0	0	0.508	0.107	0.112	0	0.116	0	0	0.181	0	0	0.325
ATN1	0	0.119	0.119	0.115	0.165	0	0	0.107	0.204	0.132	0.155	0.101	0.238	0.173	0.141	0.124
REEP1	0	0.129	0.161	0.259	0.273	0.14	0.13	0.198	0.144	0.145	0	0.168	0.138	0	0	0
SETX	0	0.115	0.15	0.108	0.143	0.149	0.126	0.102	0.124	0.163	0.115	0.115	0.123	0.347	0	0
NIPA1	0	0.12	0.108	0.251	0.183	0.141	0.13	0.196	0.144	0.145	0	0.132	0.175	0.138	0	0
AADAT	0	0.111	0.144	0	0.148	0.136	0.206	0.143	0.314	0.176	0.124	0	0.111	0.114	0.135	0
KLC1	0.172	0.108	0.127	0.135	0.52	0	0	0.154	0.219	0.108	0.114	0	0	0.193	0	0
CDK5R1	0	0.158	0.101	0	0.155	0.138	0.125	0.127	0.242	0.142	0.151	0	0	0.154	0	0.356
CHMP2B	0.253	0.119	0.101	0	0.245	0	0	0.109	0.185	0.222	0.183	0.191	0.114	0.116	0	0
DYT4	0	0	0	0.16	0.182	0.244	0.198	0.114	0.174	0	0.154	0.169	0	0.126	0	0.31
YWHAQP2	0.171	0.179	0	0.104	0.178	0	0	0.108	0.241	0.305	0.126	0.152	0	0.266	0	0
CLN5	0	0.152	0.203	0	0.222	0.286	0.125	0.182	0.268	0.119	0	0	0.114	0.147	0	0
CMT2H	0	0.122	0.132	0.242	0.183	0.152	0.129	0.157	0.129	0.182	0	0.124	0.117	0.138	0	0
FIMG1	0	0	0.406	0.203	0	0.181	0.148	0.111	0	0.172	0	0.219	0.101	0	0	0.265
KLHL1	0.137	0	0	0.107	0	0.196	0.166	0.132	0	0.204	0.155	0.165	0	0.119	0.108	0.315
ETL2	0	0.12	0.113	0	0.222	0.286	0.125	0.142	0.101	0.168	0	0	0.125	0.121	0	0.281
ALDH16A1	0	0.115	0.169	0.261	0.319	0	0.101	0.11	0.133	0.177	0	0.143	0.158	0.108	0	0
DYT13	0	0.125	0.114	0.119	0.143	0.134	0.127	0.114	0.131	0.154	0.161	0.152	0.125	0	0	0.19
SEPT4	0	0.335	0	0	0.231	0	0	0.115	0.296	0.247	0.183	0	0	0.132	0.248	0
ATXN8	0	0	0.194	0.115	0.168	0	0	0.126	0.245	0	0.136	0.11	0.111	0.105	0.121	0.351
HFM	0	0.127	0.11	0.263	0.199	0.207	0.13	0.21	0.144	0.11	0	0.14	0.141	0	0	0
CUEDC1	0	0.133	0.114	0	0.204	0.159	0.121	0.169	0.144	0.211	0.162	0	0.133	0.109	0.121	0
C14ORF23	0	0.122	0.185	0	0.192	0.208	0.121	0.12	0.105	0.11	0.103	0	0.242	0.132	0.135	0
ALS5	0.305	0.142	0	0.104	0.203	0	0	0.125	0.115	0.119	0.141	0.19	0	0.319	0	0
ATXN1	0	0	0.114	0.105	0.34	0	0	0.104	0.249	0.114	0.18	0	0.105	0.144	0.122	0.184
SCAR3	0	0	0.21	0.115	0	0.223	0.145	0	0	0.119	0.163	0.114	0.106	0.16	0.14	0.264
PTPN5	0	0	0.128	0	0.133	0.172	0.194	0.112	0.124	0.265	0.103	0	0.187	0.218	0.121	0
RLS2	0	0.208	0.132	0.11	0.143	0.105	0.127	0.106	0.116	0.153	0.186	0.112	0.1	0	0	0.156
SPG21	0	0.138	0.162	0.24	0.201	0	0.102	0.158	0.128	0.247	0	0.127	0.104	0.142	0	0
AFG3L2	0	0.102	0	0.102	0.189	0	0.123	0.117	0.112	0.182	0.131	0.133	0.129	0.115	0	0.297
SPG27	0	0.243	0.112	0.231	0	0.113	0.16	0.117	0.165	0.101	0	0.144	0.109	0	0	0.234
SIL1	0	0.106	0.116	0.234	0	0	0.121	0.139	0.155	0.152	0.148	0.197	0.124	0.235	0	0
SLC1A3	0	0.215	0.109	0	0.143	0.145	0.121	0.155	0.141	0.107	0.136	0	0.125	0.324	0	0
COL25A1	0.221	0	0.319	0	0.218	0	0	0.101	0.165	0.166	0.155	0	0.128	0.121	0.121	0
GIGYF2	0	0.102	0.11	0	0.167	0	0	0.109	0.14	0.15	0	0.136	0.331	0.13	0	0.337
SPG38	0	0.111	0.133	0.26	0.319	0.138	0.131	0.11	0.114	0.145	0	0.142	0.104	0	0	0

ATP13A2	0	0.109	0.225	0	0.156	0	0	0.11	0.13	0.177	0.203	0.144	0.106	0.139	0	0.208
HLN2	0.103	0	0.117	0	0.221	0	0	0.13	0.157	0.205	0.155	0	0.124	0.113	0.133	0.246
FGF14	0	0	0.109	0	0	0.203	0.157	0.116	0	0.15	0.171	0.132	0.172	0.137	0.14	0.208
DCTN1	0	0.358	0	0.113	0.269	0	0	0	0.144	0.197	0.17	0	0	0.19	0.253	0
SACS	0	0	0.132	0.275	0	0.156	0.145	0.125	0.104	0.229	0.141	0.105	0.113	0.162	0	0
MRST	0	0.108	0.104	0.11	0.143	0.182	0.127	0.11	0.142	0.161	0.161	0.103	0.104	0.127	0	0
LRRK2	0	0.163	0.119	0	0.206	0	0	0.111	0.259	0.124	0	0	0.1	0.203	0.137	0.259
C4ORF19	0	0.117	0.115	0	0.257	0.141	0.121	0.106	0.138	0.17	0.102	0	0.154	0.116	0.135	0
TUBA3D	0	0.105	0.113	0	0.216	0	0	0.14	0.435	0.229	0.155	0	0.144	0.112	0	0
ALSFTD	0.155	0.138	0.119	0.12	0.189	0	0	0.113	0.151	0.239	0.114	0	0.142	0.159	0	0
ATXN1L	0	0	0	0.186	0.267	0	0	0.112	0.364	0	0.127	0	0	0.172	0.23	0.177
JPH3	0	0	0.144	0	0.179	0	0	0.119	0.274	0.174	0.123	0	0.172	0.128	0.134	0.183
PPT1	0	0.227	0.131	0	0.182	0.172	0.125	0.12	0.167	0.128	0	0	0.122	0.119	0.136	0
PARK2	0	0.108	0.125	0	0.205	0	0	0.103	0.157	0.202	0.167	0	0.163	0.169	0	0.223
MT1XP1	0	0.155	0	0.104	0.202	0	0.115	0.134	0.245	0.108	0.158	0.176	0.101	0.122	0	0
PARK12	0	0.116	0.114	0	0.206	0	0	0.103	0.105	0.222	0.104	0.116	0.134	0.119	0	0.279
PNMA1	0	0.138	0	0	0.266	0.177	0.125	0.226	0	0.178	0.134	0	0	0.105	0.269	0
PARK7	0	0.113	0	0	0.24	0	0	0.108	0.287	0.215	0.128	0	0	0.142	0.245	0.139
ACAP3	0	0	0	0	0.37	0.133	0	0.116	0.201	0	0.14	0	0	0.156	0.231	0.269
HAAO	0.117	0	0	0	0.302	0.231	0.147	0.13	0.243	0	0.101	0	0	0.106	0.234	0
HYLS1	0	0	0.154	0	0.147	0.321	0.124	0.146	0.206	0.192	0	0.135	0.184	0	0	0
WDR41	0	0	0.105	0	0.181	0	0	0.136	0.105	0.213	0.159	0	0.104	0.203	0.121	0.281
GRIK3	0	0.37	0.174	0	0	0.532	0.141	0.104	0	0.172	0.114	0	0	0	0	0
RCHTS	0	0	0.105	0.281	0.153	0.142	0.122	0.108	0	0.179	0	0.125	0.138	0.141	0	0.107
XK	0	0	0.109	0.105	0.406	0	0.105	0.107	0.107	0	0.121	0.11	0.213	0	0	0.218
DYT7	0	0	0.16	0.275	0.165	0.146	0.122	0.113	0.124	0.138	0	0.102	0.106	0	0	0.15
MLC1	0	0	0.125	0.226	0.163	0.13	0.201	0.118	0.271	0.12	0	0.111	0.121	0	0	0
LOC643534	0	0.13	0.121	0	0.167	0	0	0.109	0.147	0.245	0.16	0	0	0.121	0.119	0.265
EVR3	0	0.104	0	0.102	0.173	0.162	0.122	0.256	0.101	0.173	0	0.126	0	0	0	0.265
AD6	0.46	0	0.16	0	0.22	0	0	0.109	0.221	0.147	0	0	0.136	0.123	0	0
METRN	0	0.33	0	0	0.339	0	0	0.167	0.107	0.113	0.128	0	0	0.138	0.248	0
LOC646506	0.145	0.134	0.103	0	0.201	0	0.118	0.107	0.239	0.155	0.141	0	0.108	0.118	0	0
PRNP	0	0.159	0	0	0.163	0	0.134	0.102	0.375	0.156	0.118	0	0	0.15	0	0.209
TARDBP	0.246	0.116	0.11	0	0.211	0	0	0.156	0.243	0.139	0.126	0	0.109	0.107	0	0
SLC1A1	0	0.109	0.125	0	0.164	0.216	0.105	0.144	0.206	0.125	0.134	0	0	0.115	0.12	0
CDH12P3	0	0.181	0	0.112	0.204	0	0	0	0.272	0.288	0.134	0	0	0.124	0.242	0
CYP46A1	0.322	0.116	0.125	0	0.314	0	0	0.112	0.126	0.169	0	0	0.143	0.129	0	0
EIG3	0	0	0.101	0.204	0	0.152	0.153	0.163	0	0.202	0	0.202	0.133	0	0	0.242
ASAH2B	0	0.124	0.139	0	0.205	0	0.201	0.102	0.177	0.11	0.172	0	0.145	0.174	0	0
TTBK1	0.114	0.124	0.142	0	0.156	0	0.198	0.114	0.242	0.105	0.103	0	0.124	0.126	0	0
PINK1	0	0.126	0.142	0	0.16	0	0	0.11	0.238	0.12	0	0	0.197	0.106	0	0.348

MBS3	0	0.113	0.107	0.105	0.219	0.104	0.13	0.107	0.117	0.11	0	0.102	0.107	0	0	0.224
S100B	0.109	0.101	0.13	0	0.29	0	0.139	0.142	0.225	0.133	0	0	0.143	0.13	0	0
DYNC1H1	0	0.144	0	0.113	0.174	0	0	0	0.121	0.448	0.118	0.143	0	0.144	0.137	0
BACE1	0.11	0.365	0.206	0	0.255	0	0	0.112	0.172	0.104	0	0	0.103	0.113	0	0
MAPT	0.392	0.125	0.167	0	0.14	0	0	0.109	0.168	0.11	0	0	0.113	0.215	0	0
SNCB	0	0.133	0.115	0	0.211	0	0	0.108	0.157	0.275	0.168	0	0.128	0.109	0.134	0
NDUFB3	0	0.13	0	0.113	0.174	0	0	0.128	0.203	0.269	0.131	0.217	0	0.172	0	0
AMLCR2	0	0	0.115	0.201	0.252	0	0	0.105	0.167	0	0.108	0.105	0.107	0	0.105	0.271
ALS7	0.163	0.14	0	0.101	0.167	0	0	0.104	0.289	0.232	0.12	0	0	0.22	0	0
RTN4	0	0.123	0.115	0.23	0.241	0	0	0.106	0.184	0.183	0	0.116	0.108	0.127	0	0
DOCK3	0.113	0.13	0.219	0	0.227	0	0	0.167	0.13	0.149	0.151	0	0.131	0.115	0	0
VPS54	0.16	0.109	0	0.114	0.359	0	0	0.106	0.194	0.132	0.13	0.117	0	0.109	0	0
CPLX2	0	0	0.19	0	0.176	0.154	0.125	0.12	0	0.292	0.11	0	0.104	0.122	0.134	0
APOD	0.14	0.116	0.365	0	0.148	0	0	0.149	0.23	0.131	0	0	0.116	0.13	0	0
GLUL	0	0.123	0	0	0.24	0.414	0.181	0.117	0.166	0.179	0	0	0	0.103	0	0
SLC30A3	0	0.118	0.107	0	0.222	0.188	0.125	0.226	0.155	0.151	0	0	0.106	0.121	0	0
NPTX2	0	0.143	0.417	0	0.214	0.173	0	0.123	0	0.215	0	0	0.129	0.101	0	0
STH	0.111	0.219	0.13	0	0.163	0	0	0.108	0.222	0.176	0.158	0	0.104	0.123	0	0
CALHM3	0.175	0.13	0.116	0	0.314	0	0	0.112	0.233	0.197	0	0	0.108	0.127	0	0
RAI1	0	0	0.11	0.19	0	0.159	0.15	0.114	0	0	0.142	0	0.105	0	0.214	0.328
PDYN	0	0.105	0.131	0	0.152	0.151	0	0.134	0.185	0.126	0	0	0.123	0.111	0	0.292
ZFAND2B	0.145	0.116	0.172	0	0.184	0	0	0.113	0.186	0	0.166	0	0.1	0.108	0	0.215
UBQLN1	0.249	0.11	0.116	0	0.14	0	0	0.167	0.179	0.123	0.146	0	0.165	0.11	0	0
AD7	0	0.12	0.191	0	0.227	0	0	0.104	0.206	0.24	0.15	0	0.104	0.162	0	0
ZFYVE26	0	0	0.124	0.224	0.139	0.184	0.122	0.116	0.122	0.164	0	0.12	0.189	0	0	0
FEB5	0	0	0.131	0	0.18	0.139	0.124	0.111	0.234	0.266	0	0	0.113	0	0	0.205
EMWX	0	0	0.147	0.202	0	0.152	0.14	0.101	0.133	0.229	0	0.115	0.102	0	0	0.178
CLN3	0	0.109	0.106	0	0.196	0.172	0.105	0.174	0.101	0.152	0	0	0.141	0.121	0.119	0
FUS	0.178	0.15	0	0	0.29	0	0	0.109	0.165	0.235	0.258	0	0	0.111	0	0
TRNW	0	0	0	0.18	0	0.16	0.144	0.12	0	0	0.111	0.126	0.221	0.167	0	0.266
SPG3B	0	0.144	0	0.112	0.225	0	0.115	0.108	0.144	0.137	0.127	0.197	0.184	0	0	0
COCH	0	0.104	0.155	0.235	0.189	0	0	0	0.145	0.311	0	0.227	0.127	0	0	0
ABCD1	0	0	0.118	0.202	0.23	0	0.1	0.127	0.201	0	0	0.233	0.152	0.13	0	0
CDK5	0.184	0.124	0	0	0.303	0	0	0.108	0.183	0.119	0.111	0	0	0.164	0	0.196
PVALB	0	0.117	0.12	0	0	0.507	0.151	0	0	0.195	0.127	0.113	0	0.159	0	0
ITM2B	0.116	0.102	0.108	0	0.25	0	0.135	0.118	0.188	0.119	0.116	0	0.117	0.119	0	0
ATL1	0	0.142	0.125	0.105	0.201	0	0.114	0.129	0.181	0.107	0	0.103	0.178	0.103	0	0
KBTBD11	0	0.11	0.208	0.234	0	0.182	0.147	0.117	0	0.129	0	0.103	0.131	0	0	0.126
TLCD1	0	0.189	0.122	0	0.205	0.154	0.125	0.163	0.144	0.154	0	0	0.108	0.122	0	0
ADNP	0.101	0.127	0.111	0	0.177	0	0	0.107	0.1	0.166	0.249	0	0.162	0.185	0	0
MGR4	0	0.115	0	0.114	0.232	0.174	0.123	0.161	0.216	0.1	0	0.108	0	0	0	0.141

SPG14	0	0.129	0.137	0.115	0	0	0.144	0	0.171	0.126	0	0.112	0.328	0.219	0	0
ZC3H14	0.101	0.141	0.14	0	0.149	0	0	0.11	0.191	0.132	0.233	0	0.123	0.161	0	0
TAAR8	0	0	0.101	0	0.199	0	0	0.101	0.311	0	0.166	0	0	0.135	0.23	0.238
GRIN1	0	0	0.124	0	0.181	0.142	0.124	0.241	0.173	0.264	0.116	0	0	0	0	0.113
CDKL1	0	0	0.192	0	0.158	0.175	0.124	0.16	0.238	0.177	0	0	0.129	0.12	0	0
SRR	0	0.101	0.147	0	0.155	0	0.192	0.131	0.168	0.214	0.125	0	0.124	0.108	0	0
MAP6	0	0.174	0.126	0	0.222	0.113	0	0.137	0.216	0.132	0.106	0	0.13	0.109	0	0
ALS3	0.106	0.103	0	0.104	0.23	0	0	0.13	0.251	0.124	0.143	0.151	0	0.117	0	0
JOSD2	0.139	0	0	0.168	0	0	0	0	0.149	0	0.278	0	0	0.107	0.312	0.305
L2HGDH	0.31	0	0	0	0.174	0.207	0.153	0.111	0.151	0	0	0.103	0.122	0.125	0	0
DYRK1A	0.189	0	0.115	0	0.219	0	0	0.127	0.156	0.285	0.142	0	0.106	0.114	0	0
GRIN3B	0	0.121	0.147	0	0.225	0	0.182	0.135	0.194	0.186	0.149	0	0	0.112	0	0
CSTB	0	0	0.105	0	0.134	0.136	0.126	0.102	0.184	0.139	0.125	0	0.175	0.103	0.121	0
DISC1	0	0.143	0.203	0	0.21	0.143	0	0.122	0.321	0.183	0	0	0.119	0	0	0
TSEN34	0	0.101	0	0	0	0.204	0.129	0.146	0	0.174	0.161	0	0.113	0.105	0	0.309
PHYHIP	0	0	0.151	0	0	0.176	0.15	0.102	0	0	0.113	0	0.183	0.117	0.108	0.34
MYP5	0	0	0.147	0	0.175	0	0	0.117	0.446	0.161	0.153	0.106	0.134	0	0	0
TMEM106B	0.107	0.13	0.141	0	0.146	0	0	0.105	0.198	0.203	0.157	0	0.121	0.128	0	0
NPTX1	0.129	0.116	0.102	0	0.514	0	0	0.127	0	0.219	0	0	0.103	0.123	0	0
ARL5B	0.162	0	0.129	0	0.167	0	0	0.115	0.295	0.219	0	0	0.102	0.243	0	0
FEB7	0	0	0.108	0	0.178	0.173	0.124	0.177	0.139	0.176	0	0.104	0.1	0	0	0.15
MYMY1	0	0.116	0.121	0.24	0.222	0	0.114	0.132	0.146	0	0	0.119	0.217	0	0	0
PARK3	0	0.134	0.128	0	0.152	0	0	0.105	0.115	0.113	0.113	0.112	0.1	0.157	0	0.197
SCA18	0	0.136	0.129	0	0.166	0	0	0.129	0.226	0.209	0.173	0	0	0.145	0.11	0
ASPA	0	0.1	0.155	0.226	0	0.163	0.153	0.117	0	0.101	0	0.125	0.142	0.14	0	0
GRIK2	0	0.108	0	0	0	0.205	0.126	0.112	0	0.448	0.126	0	0	0.192	0.105	0
SPOAN	0	0.123	0.145	0.105	0.167	0	0.116	0	0.242	0.107	0	0.176	0.13	0.111	0	0
SDIM1	0	0.266	0.133	0	0.209	0	0	0.113	0.158	0.144	0.165	0	0.102	0.131	0	0
TPPP3	0	0.227	0	0	0.232	0	0	0.107	0.217	0.178	0.111	0	0	0.105	0.243	0
HECTD2	0	0.101	0.105	0	0.226	0	0.141	0.131	0.144	0.107	0.17	0	0.16	0.132	0	0
BFIC2	0	0	0.108	0.205	0	0.18	0.167	0.147	0	0.199	0	0.11	0.139	0	0	0.161
GRIN2D	0	0	0	0	0.18	0.176	0.146	0.144	0.105	0.152	0.128	0	0	0.383	0	0
FAM178A	0	0	0	0.117	0	0.155	0.155	0.129	0	0	0.212	0.136	0	0.145	0.119	0.246
MBNL1	0	0	0	0	0.289	0	0	0.164	0.344	0	0.102	0	0	0.164	0.115	0.233
SPG9	0	0	0.131	0.199	0	0.179	0.152	0.106	0.146	0.173	0	0.199	0.125	0	0	0
RPSAP52	0.198	0	0.138	0	0.157	0	0	0.123	0.137	0.13	0	0	0.11	0.417	0	0
PQBP1	0	0	0.105	0	0.177	0.189	0.126	0.103	0.144	0.1	0.118	0	0.102	0.106	0.135	0
ATXN3L	0	0	0	0.156	0.141	0	0	0.127	0.188	0	0.108	0	0	0.109	0.226	0.35
NPTXR	0	0.105	0.122	0	0.155	0.26	0	0.123	0.259	0.131	0	0	0.124	0.122	0	0
SGSH	0	0	0.172	0	0.128	0	0	0.114	0.468	0.128	0.155	0	0.128	0.107	0	0
ICCA	0	0	0.201	0.204	0	0.131	0.158	0.104	0	0.192	0	0.122	0.106	0	0	0.181

SPG28	0	0.119	0	0.112	0	0.169	0.145	0	0.223	0.152	0	0.114	0.198	0	0	0.165
CDCP2	0	0.11	0	0	0.153	0	0	0.112	0.235	0	0.234	0	0	0.118	0.234	0.197
NPEPPS	0.288	0.132	0	0	0.293	0	0	0.113	0.106	0.171	0.166	0	0	0.124	0	0
QSER1	0	0.193	0	0	0.266	0	0	0.133	0.264	0.318	0.118	0	0	0.1	0	0
SLC30A6	0	0.185	0.144	0	0.155	0	0	0.17	0.256	0.119	0	0	0.136	0.227	0	0
GRINA	0	0	0	0	0.195	0.178	0.154	0.101	0.19	0.22	0.118	0	0	0.125	0.11	0
ATP8B4	0	0.101	0.109	0	0.21	0	0	0.184	0.268	0.263	0	0	0.101	0.155	0	0
KCTD7	0	0	0.129	0.106	0	0.224	0.148	0.195	0	0.134	0	0.122	0.214	0	0	0.118
MIR103A1	0	0.319	0.122	0	0.21	0	0	0.105	0.189	0.105	0.109	0	0.106	0.125	0	0
SPG41	0	0.23	0	0.112	0.162	0	0.108	0.174	0.186	0.136	0	0.151	0.129	0	0	0
SLC17A5	0	0	0.138	0.106	0.154	0.115	0.122	0.15	0	0.19	0	0.113	0.182	0.116	0	0
FAM72B	0	0.117	0.111	0	0.222	0	0	0.111	0.156	0.286	0.161	0	0.104	0.116	0	0
HTX2	0	0	0.123	0.205	0	0.142	0.145	0.149	0.175	0	0	0.12	0.155	0	0	0.169
FOXP2	0	0	0.102	0	0	0.165	0.157	0.129	0.125	0.229	0.141	0	0.121	0	0.214	0
FEB1	0	0.319	0	0.102	0	0.141	0.155	0.106	0	0.155	0	0.137	0	0	0	0.265
PNMA2	0	0.188	0	0	0.231	0.23	0.128	0.202	0.128	0	0	0	0.142	0.13	0	0
BMS1	0.136	0.324	0	0	0.274	0	0	0.116	0.147	0.221	0	0	0	0.159	0	0
ZNF746	0	0.123	0	0	0.347	0	0	0.115	0.168	0.21	0.111	0	0	0.105	0	0.196
HSD17B10	0.103	0	0.105	0	0.166	0	0.392	0.103	0.23	0	0	0	0.14	0.133	0	0
KCNC3	0	0	0.125	0.106	0	0.158	0.146	0.13	0	0.184	0.134	0.18	0.103	0	0	0.104
CLSTN2	0.108	0	0.172	0	0.222	0	0	0.129	0.154	0.32	0	0	0.102	0.158	0	0
ARC	0	0	0.121	0	0	0.209	0.157	0.128	0	0.472	0.175	0	0.103	0	0	0
MDD2	0.328	0	0.15	0	0.184	0	0	0.105	0.152	0.165	0	0	0.117	0	0	0.162
NPC1	0.141	0.12	0.182	0	0.172	0	0	0.105	0.148	0.197	0	0	0.153	0.142	0	0
RLS4	0	0	0	0	0.159	0	0	0.121	0.359	0.131	0	0	0.105	0.189	0	0.292
ATCAY	0	0	0.103	0.202	0	0.155	0.14	0.122	0	0.201	0	0.158	0.101	0.172	0	0
MROS	0	0	0.122	0.198	0.153	0.185	0.107	0.142	0.225	0	0	0.108	0.113	0	0	0
BACE1-AS	0.121	0	0.266	0	0.213	0	0	0.101	0.111	0.1	0.106	0	0.106	0.225	0	0
LGI1	0	0.141	0.117	0.105	0	0.139	0.167	0.143	0	0.121	0	0.169	0.101	0	0	0.146
EPRPDC	0	0	0.107	0.202	0	0.232	0.148	0.125	0	0.173	0	0.107	0.119	0	0	0.135
SREK1IP1	0	0.115	0.13	0	0.216	0	0	0.179	0.228	0.116	0.137	0	0.103	0.123	0	0
LOC644462	0	0.143	0	0.113	0.166	0	0	0.127	0.161	0.193	0.111	0.173	0	0.16	0	0
TPPP	0.1	0.111	0	0	0.234	0	0	0.122	0.126	0.193	0.18	0	0	0.108	0.172	0
KLHL14	0	0	0	0.186	0.169	0.184	0.11	0.121	0	0	0	0.117	0	0.101	0.12	0.236
CALHM1	0.106	0.206	0.125	0	0.154	0	0	0.11	0.226	0.196	0	0	0.118	0.102	0	0
EIM	0	0	0.109	0.117	0	0.164	0.139	0.103	0	0.209	0	0.132	0.125	0	0	0.245
UCHL1	0	0.198	0	0	0.145	0	0	0.169	0.187	0.113	0.174	0.207	0	0.15	0	0
WTRS	0	0	0.115	0.19	0	0.155	0.139	0.101	0	0.195	0	0	0.12	0	0	0.325
HAP1	0	0.13	0.108	0	0.167	0	0	0.11	0.154	0.163	0.147	0	0.116	0.133	0.109	0
ERVK-16	0	0.383	0	0	0.198	0	0.11	0.1	0.258	0	0.168	0	0	0.116	0	0
CLN6	0	0	0.118	0	0.181	0.114	0.105	0.206	0.202	0.194	0	0	0.104	0.108	0	0

FCMTE1	0	0.13	0.125	0.234	0	0.145	0.14	0.143	0	0.101	0	0.103	0.109	0	0	0.1
TBC1D24	0	0	0.106	0.19	0	0.182	0.148	0.111	0	0.131	0	0.127	0.172	0	0	0.163
HMP19	0	0.139	0	0.103	0.235	0	0	0.106	0.15	0.182	0.167	0.13	0	0.115	0	0
CDH12P1	0	0.124	0	0.102	0.162	0	0	0	0.191	0.192	0.187	0	0	0.125	0.24	0
TRNG	0	0	0.109	0	0.188	0	0	0.103	0.296	0	0.113	0.106	0.249	0.158	0	0
TPPP2	0	0.127	0	0	0.153	0	0	0.107	0.168	0.417	0.129	0	0	0.105	0.115	0
DNAJB2	0	0	0	0.165	0.187	0	0	0	0.191	0	0.112	0	0	0.113	0.298	0.253
PSEN1	0.331	0	0.117	0	0.196	0	0	0.101	0.15	0.197	0	0	0.102	0.12	0	0
LGMD1G	0	0.172	0	0.102	0.158	0	0	0	0.121	0.236	0.168	0.112	0.116	0.128	0	0
CDH12P4	0	0.126	0	0.102	0.291	0	0	0	0.154	0.122	0.129	0	0	0.138	0.251	0
ATG5	0	0.129	0	0	0.269	0	0	0	0.171	0	0.17	0	0	0.267	0.305	0
UTP11L	0	0.16	0.102	0	0.217	0	0	0.115	0.217	0.118	0.162	0	0.117	0.101	0	0
TPP1	0	0	0	0	0.153	0.155	0.146	0.157	0.132	0.253	0	0	0.12	0.191	0	0
DRP2	0	0	0.111	0	0.139	0	0	0.103	0.372	0.215	0	0	0.111	0	0	0.255
GRIN2B	0	0	0.132	0	0.199	0	0.125	0.103	0.167	0.247	0.191	0	0	0	0	0.142
PRND	0	0.149	0	0	0.164	0	0.124	0.102	0.205	0.263	0.166	0	0	0.133	0	0
SLC18A2	0	0	0.135	0	0.202	0	0	0	0.223	0.26	0.111	0	0	0.108	0.266	0
GPR37	0	0.12	0	0	0.145	0	0	0.108	0.236	0.224	0.187	0	0	0.128	0	0.156
MBS2	0	0.107	0.215	0.105	0	0.154	0.16	0.137	0	0	0	0.156	0.161	0	0	0.109
AD9	0	0.2	0.13	0	0.155	0	0	0.111	0.142	0.114	0.182	0	0.144	0.125	0	0
MARK1	0.134	0	0	0	0.184	0	0	0.106	0.16	0.315	0.194	0	0	0.21	0	0
GDAP1	0	0.156	0	0.113	0	0	0.12	0	0.156	0.275	0	0.119	0.244	0.116	0	0
NCSTN	0.131	0	0.198	0	0.186	0	0	0.132	0.197	0	0	0	0	0.455	0	0
MCKD1	0	0.112	0	0.113	0.163	0	0	0	0.303	0.146	0	0.116	0.125	0	0	0.22
GRM4	0	0.106	0.123	0	0	0.267	0.161	0.185	0	0.198	0.127	0	0	0.131	0	0
CPLX1	0	0	0.102	0	0	0.101	0.17	0.102	0	0.291	0.142	0	0.114	0.139	0.134	0
PSNP3	0.17	0.1	0	0	0.162	0	0	0.14	0.128	0.133	0.193	0	0	0.125	0.144	0
RTN4R	0	0.126	0.152	0.105	0.187	0	0	0	0	0.119	0	0.137	0.103	0.365	0	0
GRM5	0	0.165	0.243	0	0	0.105	0.151	0.129	0	0.114	0.233	0	0	0.154	0	0
LRRK1	0	0.11	0	0	0.145	0	0	0.174	0.154	0.251	0.102	0	0	0.16	0	0.196
RNF19A	0	0.22	0	0	0.138	0	0	0.106	0.135	0.368	0.163	0	0	0.16	0	0
GRIN2A	0	0	0.135	0	0.15	0	0.109	0.103	0.409	0.109	0.103	0	0	0	0	0.171
DPP6	0	0.167	0	0	0.316	0.148	0	0.123	0.188	0.132	0.115	0	0	0.1	0	0
PAND2	0	0	0.153	0	0.191	0	0	0	0.172	0.178	0	0.277	0.171	0	0	0.146
GNE	0	0.164	0	0.103	0.21	0	0	0	0.23	0.14	0	0.164	0.147	0.13	0	0
GAN	0	0.106	0	0.114	0.166	0	0	0.11	0.181	0.153	0.111	0.108	0.128	0.108	0	0
MRXSL	0	0	0.168	0.194	0	0.213	0.159	0.12	0	0	0	0	0.137	0	0	0.294
LOC643387	0.213	0.111	0.116	0	0.157	0	0	0.104	0.118	0.102	0.13	0	0.118	0.114	0	0
KIFAP3	0	0.154	0	0.434	0.147	0	0	0	0.208	0.179	0	0	0	0.16	0	0
SORL1	0.138	0.182	0.1	0	0.162	0	0	0.113	0.143	0.203	0	0	0.102	0.138	0	0
ENO2	0.109	0.164	0	0	0.292	0.132	0.125	0.13	0.177	0.152	0	0	0	0	0	0

SCN8A	0	0.117	0	0.113	0	0.194	0.144	0.104	0	0.265	0	0.126	0	0	0	0.213
DPYSL2	0.123	0.114	0.164	0	0.117	0	0	0	0.244	0.214	0.153	0	0	0.147	0	0
GRIK5	0	0.133	0	0	0	0.202	0.112	0.104	0	0.463	0.126	0	0	0.131	0	0
CLSTN3	0.119	0	0.151	0	0.186	0	0	0.113	0.399	0	0	0	0	0.302	0	0
GRIN3A	0	0	0.131	0	0.182	0	0.138	0.103	0.167	0.179	0.106	0	0.152	0.11	0	0
SGCE	0	0	0.139	0.117	0	0.18	0.148	0.105	0	0.127	0	0.145	0.119	0	0	0.187
LGI2	0	0.14	0.115	0	0	0.176	0.162	0.138	0	0.114	0	0.164	0.108	0	0	0.15
NEFH	0	0.123	0	0.102	0.204	0	0	0	0.17	0.265	0.127	0.135	0	0.14	0	0
OTSC5	0.191	0	0.107	0.306	0	0	0	0	0.153	0.114	0	0.169	0.224	0	0	0
PAND1	0	0	0.122	0	0.196	0	0	0.107	0.347	0.179	0	0	0.107	0	0	0.204
RBFOX3	0	0.12	0.121	0	0	0.137	0.155	0.115	0.116	0.186	0.171	0	0	0.139	0	0
GPRASP2	0	0	0.116	0	0	0.121	0.149	0.101	0	0	0.15	0	0.141	0.208	0.136	0.137
NEFL	0	0.129	0	0.102	0.174	0	0	0.104	0.188	0.161	0.151	0.132	0	0.117	0	0
CALHM2	0.117	0.125	0.13	0	0.135	0	0	0.106	0.191	0.111	0	0	0.202	0.137	0	0
SLC17A7	0	0.103	0.125	0	0.215	0.164	0.125	0.244	0	0.138	0	0	0.139	0	0	0
MARCH5	0	0.124	0	0.416	0.154	0	0	0	0.247	0	0.134	0	0	0.177	0	0
CAPN7	0	0	0	0	0.16	0	0	0	0.123	0	0.542	0	0	0.103	0.324	0
GRIA1	0	0.105	0.133	0	0	0.372	0.144	0	0	0.15	0.146	0	0	0	0	0.201
BHLHB9	0	0.106	0.108	0	0.224	0	0	0.13	0.198	0.167	0.105	0	0.102	0.111	0	0
GRM2	0	0.144	0.108	0	0	0.117	0.107	0.117	0	0.279	0.11	0	0.143	0.126	0	0
KMO	0	0	0.134	0	0.204	0	0	0.103	0.329	0	0.114	0	0	0.132	0.234	0
ZFP64	0.106	0.35	0	0	0.257	0	0	0.102	0.19	0.244	0	0	0	0	0	0
SERF1A	0	0.15	0	0.113	0.174	0	0	0.106	0.161	0.2	0.129	0	0	0.107	0.108	0
DCAF17	0	0	0.118	0.19	0	0.196	0.154	0.151	0	0	0	0.123	0.172	0	0	0.144
AIF1	0.127	0.109	0	0	0.296	0	0	0.14	0.305	0.15	0	0	0	0.119	0	0
SEZ6	0	0	0.123	0	0	0.221	0.157	0.1	0	0.129	0	0	0.101	0.174	0	0.237
PSEN2	0.142	0	0.113	0	0.174	0	0	0.153	0.342	0	0	0	0.129	0.185	0	0
DNMBP	0	0	0.101	0	0.157	0	0	0.102	0.503	0	0	0	0.143	0.23	0	0
IBM3	0	0.16	0	0.1	0.187	0	0	0	0.1	0.292	0	0.13	0	0	0	0.265
DYX5	0	0	0.107	0	0.168	0	0	0.153	0.267	0.114	0	0	0.138	0	0	0.286
GRM1	0	0.123	0.227	0	0	0.149	0.145	0.102	0	0.166	0.209	0	0	0.111	0	0
GRN	0.115	0.125	0.125	0	0.171	0	0	0.104	0.169	0.168	0	0	0.142	0.111	0	0
INA	0	0.103	0	0.113	0.181	0	0	0.112	0.158	0.114	0.221	0.122	0	0.105	0	0
GRIA3	0	0.181	0	0	0	0.204	0.132	0.114	0	0.17	0.114	0	0	0.118	0	0.196
CNTF	0	0.137	0	0.112	0.153	0	0	0	0.149	0.236	0.118	0.21	0	0.113	0	0
OPTN	0	0.132	0	0.403	0.158	0	0	0	0.1	0	0.231	0.101	0	0	0.101	0
SNCA	0	0	0	0	0.189	0	0	0.101	0.231	0.231	0.232	0	0	0.13	0.111	0
AD5	0.111	0.172	0.116	0	0.175	0	0	0.109	0.129	0.176	0	0	0.103	0.133	0	0
MIR659	0.107	0.167	0.12	0	0.137	0	0	0.104	0.168	0.111	0	0	0.195	0.115	0	0
CHRNA4	0	0	0.103	0	0.169	0.178	0	0.123	0.193	0.115	0	0	0.118	0	0	0.223
HMSNO	0	0.144	0	0.424	0	0	0	0	0.149	0.23	0	0.141	0.134	0	0	0

CNTN5	0	0.145	0.138	0.239	0.185	0.113	0	0.137	0	0.158	0	0	0.107	0	0	0
MIR29B2	0.151	0.218	0	0	0.26	0	0	0.114	0.147	0.204	0	0	0	0.126	0	0
SQSTM1	0	0.119	0	0	0.134	0	0	0.121	0.217	0.251	0.184	0	0	0.194	0	0
DBN1	0	0.366	0.101	0	0.217	0	0	0.113	0.135	0.158	0	0	0.13	0	0	0
LGI4	0	0.156	0	0.103	0	0.194	0.144	0.133	0	0.127	0	0.132	0	0	0	0.23
PCDH19	0	0	0.148	0	0	0.136	0.153	0.116	0	0.155	0	0.22	0.134	0	0	0.152
LTN1	0	0.121	0	0.102	0.138	0	0	0.107	0.196	0.144	0.126	0.15	0	0.129	0	0
MRT10	0	0	0.267	0	0	0.129	0.125	0.104	0	0.353	0	0.11	0.124	0	0	0
EFHC1	0	0.113	0.111	0	0	0.129	0.145	0.118	0.116	0.138	0	0	0.151	0	0	0.189
MAPK10	0	0	0.135	0	0.175	0	0.146	0.103	0.199	0.182	0.138	0	0	0.132	0	0
RTN3	0	0.109	0.117	0	0.215	0	0	0.113	0.146	0.136	0.12	0	0.146	0.106	0	0
MAPK8IP1	0.127	0	0	0	0.341	0	0	0.104	0.359	0	0.129	0	0	0.148	0	0
PNPLA6	0	0.147	0	0.102	0.151	0	0	0.104	0.2	0.132	0.115	0.119	0	0.138	0	0
HMNJ	0	0.105	0.109	0.238	0.215	0	0	0	0.121	0.165	0	0.116	0.136	0	0	0
GABBR1	0	0.103	0.124	0	0	0.396	0.162	0.123	0	0.129	0	0	0.167	0	0	0
TRBV5-2	0	0.145	0	0	0.145	0	0.101	0.107	0.208	0	0.153	0	0	0.11	0.234	0
MAB21L1	0	0	0.112	0	0.199	0	0	0.124	0	0	0.129	0	0.105	0.131	0.135	0.266
SLITRK1	0	0.105	0.104	0.273	0	0	0	0.241	0	0	0	0.116	0.138	0	0	0.223
GALC	0	0.164	0	0.114	0.215	0	0	0	0	0.149	0	0.183	0.169	0.206	0	0
C21ORF2	0.106	0	0.109	0	0.209	0	0.133	0.103	0.116	0.211	0	0	0.105	0.108	0	0
BAG5	0	0	0	0	0.172	0	0	0	0.162	0	0.256	0	0	0.106	0.296	0.204
SLC7A11	0	0.107	0	0	0.198	0	0	0.122	0.102	0.257	0.295	0	0	0.114	0	0
NS2	0	0	0.139	0.193	0	0.281	0.15	0	0	0	0	0.145	0.159	0	0	0.128
TFAM	0	0	0	0	0.29	0	0	0.109	0.144	0	0.111	0	0	0.109	0.105	0.326
GCDH	0	0	0.156	0	0.175	0	0.197	0.114	0.174	0	0.114	0	0.148	0.115	0	0
SPG12	0	0.2	0	0.102	0	0	0.127	0	0.258	0.209	0	0.103	0.191	0	0	0
RNF182	0	0.101	0.134	0	0.214	0	0	0.101	0.209	0.22	0	0	0.1	0.109	0	0
CPLX4	0	0	0.113	0	0	0.204	0	0	0	0.176	0.168	0.103	0.171	0.133	0.119	0
HECW1	0.158	0.104	0	0.101	0.151	0	0	0	0.236	0.202	0.126	0	0	0.109	0	0
USPL1	0	0.118	0	0.421	0.146	0	0	0	0.153	0.192	0	0	0	0.154	0	0
SYP	0	0	0.134	0	0.147	0.381	0	0.122	0	0.129	0	0	0.17	0.1	0	0
SIGMAR1	0	0.125	0.158	0	0.21	0	0	0.106	0.138	0.163	0.114	0	0	0.168	0	0
APBB3	0.164	0	0.167	0	0.187	0	0	0.102	0.284	0	0	0	0	0.276	0	0
AIC	0	0	0.168	0	0	0.177	0.234	0	0	0	0	0	0.487	0	0	0.113
HSPB8	0	0.14	0	0.113	0.168	0	0	0	0.165	0.195	0.14	0.15	0	0.107	0	0
FHDC1	0.133	0.108	0	0	0.258	0	0	0.111	0.442	0.126	0	0	0	0	0	0
BPPV	0	0	0	0.185	0	0.281	0.107	0	0	0.15	0	0.117	0.113	0	0	0.224
CTXN1	0	0.113	0	0	0.231	0.134	0	0.119	0.186	0.102	0	0	0	0.106	0	0.184
THAP1	0	0	0.103	0.204	0.122	0	0.104	0.106	0.159	0	0	0.115	0.112	0	0	0.148
UBAP2	0.125	0.188	0.124	0	0.183	0	0	0.104	0.203	0.114	0	0	0	0.131	0	0
TRIM65	0	0.125	0.118	0	0.118	0	0	0.124	0.367	0.113	0	0	0.101	0	0.103	0

BCYRN1	0	0	0.106	0	0.197	0	0	0.128	0.378	0.147	0	0	0.104	0.109	0	0
KRIT1	0	0.113	0	0	0	0.205	0.145	0.103	0.115	0	0	0.12	0.17	0	0	0.197
KCNQ2	0	0	0	0	0	0.191	0.112	0.125	0	0.199	0	0.2	0.164	0	0	0.176
MRT8	0	0	0.122	0	0	0.155	0.127	0.147	0	0.39	0	0.113	0.113	0	0	0
USP14	0	0	0.108	0	0.179	0.178	0	0.122	0.166	0.122	0.187	0	0	0.103	0	0
HMN7A	0	0.124	0	0.403	0	0	0	0	0.154	0.147	0.152	0.185	0	0	0	0
SCZD1	0.101	0	0.139	0	0.183	0	0	0	0.132	0.241	0	0	0.116	0	0	0.252
KCNIP3	0	0	0.116	0	0.175	0.114	0	0.138	0.181	0.145	0.174	0	0	0.121	0	0
UBQLN2	0.166	0.174	0	0	0.146	0	0	0.117	0.196	0	0.26	0	0	0.104	0	0
KIAA0196	0	0.151	0	0.112	0.16	0	0	0.104	0.303	0	0.128	0.103	0	0.101	0	0
SCAX3	0	0	0	0	0.143	0	0	0.106	0	0.528	0.144	0	0	0	0	0.241
CNTNAP2	0	0	0.104	0.117	0	0.194	0.139	0.126	0	0	0	0.109	0.17	0	0	0.202
CKB	0	0	0	0	0.162	0	0.16	0.12	0	0.237	0.115	0	0	0.103	0.263	0
VSNL1	0	0.105	0.12	0	0.165	0.174	0	0.134	0.101	0.132	0	0	0.112	0.117	0	0
AAAS	0	0	0.111	0.117	0.225	0	0.104	0.107	0.123	0.158	0	0.112	0.103	0	0	0
PTX4	0	0	0.185	0	0.193	0.178	0	0.123	0	0	0	0	0.48	0	0	0
GTSCR1	0	0	0.178	0.198	0	0.278	0.159	0.114	0	0	0	0	0.129	0	0	0.102
TAGLN3	0	0	0.155	0	0.197	0.134	0	0.138	0.202	0.187	0	0	0.145	0	0	0
MYP12	0	0	0.42	0	0.207	0	0	0	0.175	0.249	0	0	0.107	0	0	0
FGF20	0	0	0	0	0.285	0	0	0.127	0.148	0.233	0	0	0	0.148	0.217	0
RLS	0	0	0.164	0.117	0	0.15	0	0	0	0.127	0.151	0.113	0.16	0	0	0.175
CHAT	0	0.124	0.116	0	0.214	0	0	0.107	0.194	0.164	0	0	0.129	0.109	0	0
EMX1	0	0.117	0.127	0	0	0.117	0.166	0.118	0	0.137	0.13	0	0.127	0	0.116	0
SLC6A3	0	0	0.145	0	0.278	0	0	0	0	0.127	0	0	0	0	0	0.603
PSMA2L	0	0.119	0	0.113	0.174	0	0	0.106	0.116	0.164	0.1	0.125	0	0.136	0	0
PARK10	0	0.116	0	0	0.198	0	0	0.114	0.248	0.184	0	0	0	0.106	0	0.186
MANF	0	0.125	0	0	0.103	0	0.106	0.113	0.296	0.13	0.141	0	0	0.138	0	0
IGHMBP2	0	0.125	0	0.112	0.189	0	0	0.125	0.166	0.157	0	0.108	0	0.165	0	0
KCNN3	0	0	0.121	0	0	0.12	0	0.102	0	0	0.11	0	0.119	0	0.214	0.361
GBA	0	0	0.118	0	0.169	0	0	0.136	0.256	0.225	0	0	0.107	0.134	0	0
C10ORF112	0	0	0.455	0	0.235	0	0	0.103	0.189	0	0	0	0	0.163	0	0
TAAR7P	0	0	0.105	0	0.169	0	0	0.116	0.201	0	0.106	0	0	0.113	0.105	0.229
EPPS	0	0	0.129	0	0	0.176	0.152	0.131	0	0.216	0	0.105	0.102	0	0	0.133
SCN1A	0	0	0.106	0	0	0.17	0.169	0.117	0	0.212	0	0.112	0.147	0	0	0.11
ENUR1	0	0	0.119	0.193	0	0.279	0.152	0	0	0	0	0.11	0.155	0	0	0.134
RNF146	0	0.122	0.116	0	0.21	0	0	0.134	0.213	0	0	0	0	0.345	0	0
GAPDH	0	0.139	0	0	0.17	0	0	0	0.113	0	0.3	0	0	0.111	0.306	0
ADNP2	0	0	0.128	0	0.204	0	0	0.135	0.235	0	0.218	0	0.104	0.115	0	0
AUTS3	0.244	0	0.144	0	0.184	0.167	0.126	0.165	0	0	0	0	0.109	0	0	0
TIMM8AP1	0	0	0.184	0.117	0	0.125	0.169	0.114	0	0	0	0.122	0.104	0	0	0.203
NOVA1	0	0.151	0.103	0	0	0.364	0	0.142	0.13	0	0	0	0	0	0	0.247

LGMD1H	0	0.152	0	0.102	0.256	0	0	0	0.174	0.198	0	0.101	0.151	0	0	0
CST3	0	0.115	0	0	0.144	0.168	0.124	0.127	0.14	0.203	0	0	0	0.111	0	0
CELF3	0	0	0	0	0.11	0	0	0.12	0.21	0	0.113	0	0	0.106	0.24	0.232
MGR2	0	0	0.124	0	0	0.218	0.102	0.202	0.178	0	0	0	0.112	0	0	0.195
LIX1	0	0.129	0	0.113	0.209	0	0	0.106	0.13	0.195	0	0.124	0	0.125	0	0
HPCA	0	0.138	0	0	0	0.201	0	0.117	0	0.205	0.107	0	0	0.102	0.26	0
ND5	0	0	0	0.165	0.3	0	0.145	0	0.165	0	0	0.106	0.131	0.116	0	0
ATOD3	0	0	0.246	0	0.285	0	0	0.107	0.36	0	0	0	0	0.129	0	0
TRAK2	0	0.102	0	0.407	0	0.157	0.163	0	0	0	0.163	0	0	0.134	0	0
MIR107	0.104	0	0.123	0	0.186	0	0	0.128	0.339	0	0	0	0	0.246	0	0
TM2D3	0	0.108	0.13	0	0.208	0	0	0.14	0.108	0.32	0	0	0	0.111	0	0
SPG37	0	0.105	0	0.429	0	0	0	0	0.139	0.139	0	0.158	0.152	0	0	0
ASPG1	0.172	0	0.115	0	0	0.364	0.169	0.158	0	0	0	0	0.144	0	0	0
ALDH18A1	0	0	0.109	0	0.169	0	0.109	0.103	0.218	0	0	0	0.121	0.104	0	0.188
DNAI1	0	0.172	0.143	0	0	0	0	0	0	0	0	0.115	0.161	0	0.209	0.319
FAM155A	0.13	0	0.136	0	0.183	0	0	0.113	0.113	0	0.127	0	0.1	0	0.215	0
CTSD	0	0	0	0	0.166	0	0	0.138	0.187	0.466	0	0	0	0.16	0	0
NYS2	0	0	0.12	0.19	0	0.104	0.156	0	0	0.14	0	0.125	0.11	0	0	0.17
VAPB	0	0.138	0	0.102	0.172	0	0	0.127	0.184	0.157	0.115	0	0	0.119	0	0
GABRB3	0	0	0.129	0	0	0.136	0.127	0.202	0	0.161	0	0	0.114	0	0	0.243
NAGLU	0	0	0.176	0	0.203	0	0	0.128	0.157	0.177	0	0	0.117	0.152	0	0
DAOA	0	0	0.139	0	0.195	0	0	0	0.166	0.107	0	0	0.177	0	0	0.325
PABPN1	0	0.124	0	0.1	0.194	0	0	0	0.1	0	0.217	0	0	0.101	0.273	0
IMP5	0.102	0.102	0.111	0	0.161	0	0	0.11	0.216	0.11	0	0	0	0.194	0	0
MT3	0	0.131	0.23	0	0.222	0	0	0	0.174	0.243	0	0	0	0.106	0	0
LINGO1	0	0.168	0	0.103	0.255	0	0	0	0.165	0.13	0	0.117	0	0.167	0	0
GLRA1	0	0.147	0	0	0	0.202	0.136	0.172	0	0.218	0	0	0	0	0	0.227
MPPED2	0	0	0.136	0	0	0.118	0.145	0	0	0	0.109	0	0.251	0	0.342	0
CHRNA7	0	0	0.137	0	0.197	0.134	0	0.133	0.187	0.197	0	0	0.115	0	0	0
C7ORF25	0	0.145	0	0.113	0.188	0	0	0	0.15	0.211	0	0.153	0	0.138	0	0
GRID2	0	0	0.109	0	0	0.171	0.135	0	0	0.178	0	0	0.149	0.123	0	0.232
COX3	0	0	0	0.165	0.268	0	0.115	0	0.117	0	0	0.124	0	0.188	0.12	0
MAOB	0	0	0.133	0	0.171	0	0	0.135	0.194	0.111	0	0	0	0.134	0	0.218
TIMM8A	0	0	0.103	0.197	0	0	0.259	0.122	0	0	0	0.165	0.125	0.125	0	0
SYNDIG1L	0	0	0	0	0.172	0	0	0.109	0.119	0	0.312	0	0	0.112	0.27	0
RPS23	0.135	0	0.101	0	0.176	0	0	0.103	0.393	0	0	0	0	0.185	0	0
LOC653080	0	0.148	0	0.354	0.327	0	0	0	0.106	0.158	0	0	0	0	0	0
MRT9	0	0	0.114	0	0	0.259	0.125	0.118	0	0.159	0	0.146	0.168	0	0	0
GRIA2	0	0.22	0	0	0	0.156	0.112	0	0	0.179	0.133	0	0	0.131	0	0.156
BPTF	0	0.267	0	0	0.264	0	0	0.167	0.221	0	0	0	0	0.168	0	0
SCN2A	0	0	0	0.187	0	0.164	0.152	0.122	0	0	0	0.263	0	0	0	0.198

TMEM216	0	0	0.416	0	0	0.131	0.119	0	0	0.159	0	0.145	0.116	0	0	0
HTT	0	0	0.218	0	0.201	0	0	0	0.153	0	0.167	0	0.1	0.125	0.12	0
MAP1LC3A	0	0	0	0	0.243	0	0	0	0.118	0	0.213	0	0	0.177	0.333	0
SCZD13	0	0	0.102	0	0.23	0	0	0.114	0.173	0.218	0	0	0.109	0	0	0.137
KIAA1191	0	0	0	0	0.283	0	0	0.127	0.167	0	0.143	0	0	0.123	0.239	0
SLC25A15	0	0	0.177	0.192	0	0.147	0.154	0.106	0	0	0	0.172	0.134	0	0	0
FAM189A2	0	0	0	0.164	0	0.15	0.152	0	0	0	0.112	0.1	0	0.103	0.108	0.192
TACO1	0	0	0.112	0	0.122	0	0.115	0.103	0	0	0	0.173	0.138	0.125	0	0.193
MGR3	0	0.105	0	0	0	0.2	0.124	0.152	0.107	0.2	0	0	0	0	0	0.191
SAMHD1	0	0	0	0	0.172	0.148	0.16	0.226	0	0	0	0	0.141	0	0	0.231
MS4A6E	0	0	0.165	0	0.168	0	0	0.114	0.183	0	0	0	0.147	0.163	0.137	0
CDH19	0	0	0	0.503	0	0	0	0	0	0	0	0.574	0	0	0	0
TMEM132D	0	0.308	0.123	0	0.121	0	0	0.127	0.132	0.152	0	0	0.112	0	0	0
RAB39B	0	0	0.125	0	0	0.243	0.124	0.13	0	0	0	0	0.24	0	0	0.211
DURS1	0	0	0.284	0	0	0.17	0.142	0	0	0	0	0	0.152	0	0	0.325
DLST	0	0	0.119	0	0.179	0	0.102	0.103	0.184	0	0.125	0	0.115	0.145	0	0
PADI2	0	0.1	0	0	0.123	0	0	0.172	0.165	0.162	0.13	0	0	0.22	0	0
OTSC1	0	0	0.302	0.333	0	0	0	0	0	0.165	0	0.147	0.125	0	0	0
NTNG2	0	0	0.152	0	0	0.238	0.122	0.192	0	0.176	0	0	0.191	0	0	0
SPTBN4	0	0	0	0.156	0	0.15	0.144	0.106	0	0.103	0	0.164	0	0	0	0.246
PACRG	0	0.119	0	0	0.197	0	0	0	0.133	0.264	0	0	0	0.107	0	0.247
SRPX2	0	0	0.194	0	0	0.139	0.273	0.139	0	0	0	0	0.118	0	0	0.203
DHCR24	0.138	0	0.126	0	0.183	0	0	0.14	0.269	0	0	0	0.105	0.105	0	0
SPG45	0	0.134	0.106	0.27	0	0	0.105	0	0	0.214	0	0.118	0.119	0	0	0
GSM1	0	0	0.153	0.206	0	0	0	0	0.156	0.165	0	0.103	0.166	0	0	0.116
PRNT	0	0.132	0	0	0.198	0	0.124	0.101	0.244	0	0.149	0	0	0.116	0	0
ASD1	0	0	0.19	0	0	0.171	0	0	0.29	0	0	0.132	0.126	0	0	0.153
NGB	0	0.115	0	0	0.164	0	0.128	0.102	0.234	0.197	0	0	0	0.121	0	0
C9ORF72	0.125	0.106	0	0	0.26	0	0	0.118	0.147	0.15	0	0	0	0.155	0	0
SORT1	0.116	0.123	0	0	0.265	0	0	0	0.153	0.279	0	0	0	0.125	0	0
STUB1	0	0.118	0	0	0.203	0	0	0	0.177	0	0.435	0	0	0.125	0	0
MRT5	0	0	0.134	0	0	0.169	0.115	0.137	0	0.224	0	0.155	0.124	0	0	0
BDMR	0	0	0.189	0.194	0	0.124	0.14	0	0	0	0	0.101	0.165	0	0	0.143
EGI	0	0	0.126	0	0	0.129	0.11	0.202	0	0.196	0	0	0.129	0	0	0.164
GPM6A	0	0	0.105	0.207	0	0.216	0	0.141	0	0.278	0	0	0.108	0	0	0
PPR1	0	0	0	0	0	0.15	0.142	0.161	0	0.287	0	0	0	0	0	0.314
TRNF	0	0	0	0.155	0	0.198	0.164	0	0	0	0	0.108	0	0.185	0	0.243
ASTN1	0	0	0.107	0	0	0.211	0.145	0.101	0	0.26	0	0	0.128	0.101	0	0
FIG4	0	0.132	0	0.112	0.174	0	0	0.104	0.141	0.166	0	0.117	0	0.106	0	0
NECAB3	0.137	0	0.188	0	0.182	0	0	0.113	0.292	0	0	0	0	0.14	0	0
HSP90AB2P	0	0.142	0	0	0.198	0	0	0.109	0.102	0	0.21	0	0	0.184	0.105	0

ANKK1	0	0	0.101	0	0.15	0	0	0	0.239	0.215	0	0	0.146	0	0	0.197
SGCZ	0	0	0	0.186	0	0.193	0.143	0.119	0	0.191	0	0	0	0	0	0.216
MPDMRS	0	0	0.11	0.194	0	0.213	0.158	0	0	0.135	0	0.117	0.12	0	0	0
IDE	0.222	0	0.117	0	0.185	0	0	0.127	0.227	0	0	0	0	0.169	0	0
SLC9A9	0	0	0.146	0	0.189	0	0	0.113	0.197	0	0.242	0	0.157	0	0	0
PQBP4	0	0	0	0	0.27	0	0	0	0.161	0	0.198	0	0	0.118	0.111	0.185
TRNA	0	0.108	0	0.113	0	0	0.127	0	0.279	0	0.106	0.194	0	0.113	0	0
SCZD6	0.112	0	0.164	0	0	0	0	0	0.157	0.165	0	0	0.116	0	0	0.325
DPH5	0	0.134	0	0.392	0	0	0	0	0	0	0.138	0	0	0.173	0.202	0
CACNG2	0	0	0.131	0	0	0.156	0.119	0.152	0	0.199	0	0	0	0	0	0.281
TBP	0	0	0	0	0.164	0	0	0	0.279	0	0.151	0	0	0.115	0.115	0.214
CNSN	0	0	0.123	0	0.17	0	0.15	0.131	0.192	0	0	0	0.156	0.115	0	0
NRN1	0	0.125	0.163	0.298	0	0	0	0	0	0.196	0	0.139	0.116	0	0	0
TTPA	0	0	0.104	0	0.204	0	0	0.118	0.192	0.19	0	0	0.111	0.118	0	0
PCP4	0	0	0.117	0	0	0.129	0	0.124	0	0.197	0.108	0	0	0.102	0.259	0
GRIK1	0	0.123	0.109	0	0	0.197	0.145	0.108	0	0.131	0.101	0	0	0.122	0	0
DCLK1	0	0	0.111	0	0	0.132	0.125	0.131	0	0.182	0.123	0	0.232	0	0	0
ACO2	0	0.218	0	0	0	0	0	0	0	0	0.212	0	0	0.12	0.106	0.379
COMT	0	0	0.176	0	0.138	0	0	0	0.234	0.194	0	0	0.158	0	0	0.135
GLRA3	0	0.116	0	0	0	0.202	0.136	0.124	0	0.13	0	0	0	0	0	0.327
TM2D2	0.104	0.1	0.1	0	0.116	0	0	0.104	0.16	0.108	0	0	0.114	0.125	0	0
CMTX3	0	0.115	0	0.405	0	0	0.12	0	0	0.155	0	0.104	0.131	0	0	0
CC2D2A	0	0	0.118	0	0	0.129	0.105	0	0	0.105	0	0.147	0.113	0	0	0.312
CSE	0	0	0	0.117	0	0.171	0.142	0.101	0	0	0.199	0.123	0	0	0	0.176
TNR	0	0.109	0.13	0	0	0.358	0.141	0	0	0.184	0	0	0.107	0	0	0
HTR6	0	0	0.107	0	0.168	0	0	0.141	0.225	0.114	0	0	0.109	0	0	0.164
MRX28	0	0	0.482	0	0	0.136	0.216	0	0	0	0	0	0.194	0	0	0
MOB3B	0.119	0.109	0	0	0.252	0	0	0.103	0.328	0.117	0	0	0	0	0	0
CLU	0	0	0	0	0.159	0	0	0.109	0.272	0.238	0	0	0	0.137	0.111	0
BACE2	0.117	0	0.159	0	0.183	0	0	0.102	0.21	0	0	0	0.114	0.141	0	0
SMA4	0	0.13	0	0.399	0.183	0	0	0	0.1	0.112	0.102	0	0	0	0	0
ZNF699	0	0	0.173	0	0.19	0	0	0.11	0.166	0.262	0	0	0.123	0	0	0
SPRNP1	0	0.138	0	0	0.138	0	0.109	0.121	0.224	0	0.158	0	0	0.135	0	0
LY6E	0	0	0	0	0.156	0	0	0	0.215	0	0.246	0	0	0.106	0.115	0.183
APBA2	0.152	0	0.14	0	0.184	0	0	0.149	0.152	0	0	0	0.114	0.13	0	0
MGR	0	0.132	0	0	0	0.2	0.102	0.138	0.115	0.166	0	0	0	0	0	0.167
ATD	0	0	0.135	0.217	0	0	0.11	0	0	0.197	0	0.139	0.222	0	0	0
CCL2	0	0	0	0.277	0.467	0	0	0	0.276	0	0	0	0	0	0	0
DCX	0	0.139	0.106	0	0	0.176	0.145	0.104	0	0.124	0.12	0	0.105	0	0	0
FMR1	0.214	0	0.104	0	0.183	0	0.229	0.107	0	0	0	0	0.182	0	0	0
BSCL2	0	0.194	0	0.103	0	0	0.147	0	0.124	0.166	0	0.137	0.145	0	0	0

COHEN1	0.101	0	0.143	0.115	0	0.141	0.141	0	0	0	0	0	0.136	0	0	0.236
NRGN	0	0	0.149	0	0	0.22	0.112	0.149	0	0.266	0	0	0.116	0	0	0
DDU	0	0	0	0.507	0	0	0	0	0	0	0	0.164	0	0	0	0.34
TUBB2A	0	0.125	0	0	0	0	0	0	0	0	0.271	0	0	0.416	0.199	0
RSRC1	0	0.406	0.139	0	0	0	0	0	0.145	0.114	0	0	0.105	0	0.102	0
DFNA43	0	0	0.205	0.209	0	0	0	0	0	0.196	0	0.144	0.257	0	0	0
ECA1	0	0	0	0	0	0.339	0.222	0.133	0	0	0	0	0	0	0	0.316
COX10	0	0	0	0.156	0.187	0	0.257	0	0	0	0	0.139	0.151	0.12	0	0
HTRA2	0.104	0.114	0	0	0.154	0	0	0.113	0.126	0.132	0.135	0	0	0.132	0	0
RAB7L1	0	0.201	0	0	0.161	0	0	0.126	0.161	0	0	0	0	0.144	0.216	0
NUB1	0	0	0	0	0.175	0	0	0	0.139	0.192	0.112	0	0	0.125	0.266	0
ARX	0	0	0.101	0	0	0.171	0.132	0.107	0	0.239	0	0	0.149	0	0	0.108
HPE6	0.145	0	0.113	0	0	0.12	0.162	0.129	0.216	0	0	0	0.121	0	0	0
VCP	0	0.121	0	0	0.156	0	0	0.123	0.166	0.197	0.128	0	0	0.113	0	0
VPS41	0	0	0	0	0.242	0	0	0	0.186	0	0.156	0	0	0.112	0.308	0
GRIN2C	0	0	0.14	0	0.197	0	0.107	0.114	0.173	0.139	0.134	0	0	0	0	0
SEZ6L2	0	0	0.125	0	0	0.172	0.151	0.195	0	0	0	0	0.135	0	0	0.225
VMD1	0	0	0	0.55	0	0	0	0	0	0.17	0.181	0	0	0	0.102	0
C7ORF57	0	0.103	0	0	0.138	0	0	0.131	0.17	0.165	0.18	0	0	0.116	0	0
CACNG3	0	0	0	0	0	0.123	0.149	0.125	0	0.314	0	0	0	0	0	0.291
TRIM2	0	0.112	0	0.112	0	0.194	0.165	0.103	0	0.203	0	0	0	0.113	0	0
ATG4B	0	0	0	0	0	0	0	0	0.139	0	0.429	0	0	0.153	0.28	0
UBE4B	0	0.134	0	0.395	0	0	0	0	0.14	0	0.221	0	0	0.111	0	0
MA	0	0	0	0	0	0.191	0.131	0.125	0	0.181	0	0.134	0	0	0	0.238
NOS1	0	0.135	0	0	0.161	0	0.222	0	0.139	0	0.122	0	0	0	0	0.221
FGGY	0	0.103	0	0	0.175	0	0	0.121	0.107	0.348	0	0	0	0.144	0	0
TTR	0	0	0.113	0.192	0.176	0	0	0.112	0.127	0	0	0.134	0.144	0	0	0
NRSN1	0	0.101	0	0	0.196	0	0	0	0.171	0.216	0	0.164	0	0.149	0	0
CHRFAM7A	0	0	0.122	0	0.192	0	0	0.118	0.253	0.201	0	0	0.111	0	0	0
GRM7	0	0	0.134	0	0	0.158	0.111	0.137	0	0.247	0	0	0.21	0	0	0
AFF2	0.119	0	0.105	0	0	0.358	0.166	0.123	0	0	0	0	0.126	0	0	0
GRINL1A	0	0.112	0	0	0.198	0	0.112	0.138	0.126	0	0.163	0	0	0.147	0	0
BECN1	0	0	0	0	0.211	0	0	0	0.151	0	0.186	0	0	0.128	0.32	0
MRX31	0	0	0.45	0	0	0.169	0.127	0	0	0	0	0	0.25	0	0	0
MEHMO	0	0	0.104	0	0	0.231	0.118	0.118	0	0	0	0	0.154	0	0	0.269
NEUROD6	0	0	0.122	0	0	0.152	0.119	0.123	0	0.17	0.12	0	0.188	0	0	0
MRPL32	0	0.125	0	0.113	0.214	0	0	0	0.11	0.168	0	0.138	0	0.125	0	0
UBXN4	0	0.131	0	0	0.268	0	0	0.15	0.211	0	0.113	0	0	0.12	0	0
MAP2	0	0.125	0	0	0	0.202	0.143	0.114	0.18	0.123	0	0	0	0.106	0	0
MRX17	0	0	0.104	0.22	0	0.192	0.154	0	0	0	0	0	0.322	0	0	0
LOH19CR1	0	0	0	0.189	0	0.16	0.152	0	0	0	0	0.14	0.132	0	0	0.218

HCN1	0	0	0	0	0	0.192	0.125	0.183	0	0.166	0.137	0	0	0	0	0.184
SLC33A1	0	0	0	0.156	0.294	0	0	0.125	0.11	0	0	0.104	0	0.198	0	0
SCN9A	0	0	0	0.189	0	0.197	0.152	0.109	0	0	0	0.14	0	0	0	0.198
SLC6A9	0	0	0.101	0	0	0.167	0.102	0.127	0	0.182	0.117	0	0.189	0	0	0
CMDR	0	0	0	0.187	0.267	0	0	0	0.273	0	0	0.124	0.133	0	0	0
UBE2K	0	0	0	0	0.237	0	0	0	0.142	0	0.163	0	0	0.135	0.307	0
ERVW-4	0	0.139	0	0	0.138	0	0.113	0.101	0.166	0	0.175	0	0	0.151	0	0
MDD1	0	0.128	0.123	0	0.146	0	0	0.124	0.107	0.145	0	0	0.21	0	0	0
LOC728519	0	0.106	0	0.105	0.247	0	0	0	0.112	0.413	0	0	0	0	0	0
KIF24	0.125	0.11	0	0	0.258	0	0	0.119	0.102	0.268	0	0	0	0	0	0
SLC44A5	0	0.132	0	0.396	0.181	0	0	0.104	0.168	0	0	0	0	0	0	0
SCKL2	0	0	0.101	0	0	0.211	0.107	0	0	0	0	0.179	0.167	0	0	0.215
NPAS3	0	0	0.136	0	0	0.344	0	0.118	0	0.224	0	0	0.157	0	0	0
NEFM	0	0.103	0	0.395	0	0	0	0	0	0.197	0	0.132	0	0.151	0	0
CHRNA6	0	0	0.134	0	0.192	0.179	0	0.123	0	0.227	0	0	0.121	0	0	0
SCAX4	0	0.12	0	0	0.145	0	0.109	0.125	0.204	0.13	0	0	0	0.141	0	0
HAR1A	0	0	0.13	0	0	0.233	0.12	0.134	0.128	0.229	0	0	0	0	0	0
AIMP2	0	0.156	0	0	0.198	0	0	0	0.153	0	0.176	0	0	0.134	0	0.156
TM4SF20	0	0	0.111	0	0	0.191	0.215	0.133	0	0	0	0	0.121	0	0	0.202
SPRN	0	0.15	0	0	0.198	0	0.148	0.1	0.162	0	0.107	0	0	0.108	0	0
FRAXE	0	0	0.39	0	0	0.206	0.139	0.111	0	0	0	0	0.126	0	0	0
SCGN	0.113	0	0	0	0.306	0	0	0.104	0.141	0.308	0	0	0	0	0	0
GABRB1	0	0	0	0	0	0.25	0.132	0.1	0	0.49	0	0	0	0	0	0
PTLAH	0	0	0.119	0.218	0	0	0	0	0	0.153	0	0.1	0.128	0	0	0.253
QPCT	0	0	0.11	0	0.195	0	0	0.14	0.277	0	0	0	0.106	0.139	0	0
FEB2	0	0	0	0	0	0.127	0.16	0.126	0	0.175	0	0.113	0	0	0	0.265
GRIA4	0	0.165	0	0	0	0.21	0.128	0	0	0.161	0	0	0	0	0	0.301
APBB1	0.159	0	0.119	0	0.184	0	0	0.128	0.141	0	0	0	0.123	0.11	0	0
CCS	0.169	0.114	0	0	0.264	0	0	0	0.138	0.115	0	0	0	0.162	0	0
PSMC2	0	0	0	0	0.171	0	0	0.106	0.242	0	0.1	0	0	0.105	0.238	0
FMR3	0.105	0	0.358	0	0	0.136	0.15	0	0	0	0	0	0.213	0	0	0
TGM6	0	0	0	0	0.18	0	0.124	0.12	0	0	0.157	0	0	0.105	0.275	0
ARHGAP18	0	0.107	0.12	0	0	0	0	0	0.107	0.392	0	0	0.132	0	0.103	0
PSMC5	0	0	0	0	0.118	0	0	0	0.103	0	0.31	0	0	0.117	0.313	0
SUSD1	0.171	0.119	0	0	0.143	0	0	0.102	0.147	0.13	0	0	0	0.148	0	0
MYP13	0	0	0	0.561	0	0	0	0	0	0.112	0	0.158	0.129	0	0	0
KIAA1267	0.224	0.126	0	0	0.143	0	0	0.141	0.102	0	0.119	0	0	0.103	0	0
GLRB	0	0.108	0	0	0	0.205	0.141	0.131	0	0.169	0	0	0	0	0	0.203
DOPEY2	0	0	0.135	0	0	0.173	0.147	0.11	0	0.25	0	0	0.141	0	0	0
MRX1	0	0	0.202	0	0	0.167	0.167	0	0	0	0	0	0.419	0	0	0
ZLS	0	0	0.137	0	0	0.141	0.296	0	0	0	0	0	0.207	0	0	0.173

ECE2	0	0	0.106	0	0.169	0	0	0.102	0.111	0.15	0	0	0.199	0.116	0	0
ADCK3	0	0	0	0.184	0	0	0.201	0	0	0	0	0.128	0	0.145	0	0.292
MCS	0	0	0.13	0.223	0	0.141	0.148	0	0	0	0	0	0.307	0	0	0
FLAD1	0.13	0.118	0	0	0.261	0	0	0.114	0.102	0	0.109	0	0	0.115	0	0
UQCRQ	0	0	0	0	0.221	0.179	0.14	0.264	0	0	0	0	0	0.144	0	0
ZNF592	0	0	0	0.155	0	0.14	0.143	0	0	0	0	0	0.223	0	0.103	0.183
DFNA41	0	0	0.115	0.105	0	0	0	0	0	0.342	0	0.273	0.112	0	0	0
TSPEAR	0	0.128	0	0.1	0	0.194	0.163	0.117	0	0	0	0.112	0	0	0	0.132
PITX3	0	0	0.115	0	0	0	0	0	0	0.127	0.129	0	0.147	0.155	0.121	0.151
PCMT1	0.182	0	0.101	0	0.174	0	0	0.104	0.165	0	0	0	0.111	0.108	0	0
PIN1	0.113	0.185	0	0	0.258	0	0	0.101	0.147	0	0	0	0	0.141	0	0
ABAT	0	0	0.105	0	0	0.233	0.116	0.136	0	0.254	0	0	0.101	0	0	0
SPG26	0	0.14	0	0.347	0	0	0	0	0.123	0.18	0	0.155	0	0	0	0
BEND2	0	0	0.171	0	0	0.175	0.111	0.144	0	0	0	0	0.134	0	0	0.21
SCN2B	0	0	0	0	0	0.197	0.202	0.11	0	0	0	0.298	0	0	0	0.138
SNCG	0	0	0	0	0.19	0	0	0.107	0.203	0.214	0	0	0	0.12	0.111	0
SOD1	0.128	0.107	0	0	0.238	0	0	0	0.219	0	0.143	0	0	0.11	0	0
SCRG1	0	0.154	0	0	0.161	0	0.108	0.139	0.17	0.107	0	0	0	0.105	0	0
LI5	0	0	0	0.206	0	0	0	0	0	0	0	0.4	0.131	0	0.207	0
LINGO2	0	0.134	0	0	0.255	0	0	0.115	0.244	0	0	0	0	0.196	0	0
GOLGA8F	0.123	0	0.33	0	0	0.142	0.169	0	0	0	0	0	0.179	0	0	0
SURF1	0	0	0	0.305	0	0	0.135	0	0	0	0	0.145	0	0.358	0	0
GPR88	0	0	0.111	0	0	0	0	0	0	0.125	0.106	0	0.122	0.147	0.134	0.196
DRD5P1	0	0	0.136	0	0	0	0	0	0	0.136	0.137	0	0	0.101	0.24	0.191
APH1B	0.11	0	0.125	0	0.188	0	0	0.111	0.275	0	0	0	0	0.132	0	0
SHISA9	0	0.147	0.185	0	0	0.23	0.141	0.127	0	0	0	0	0.111	0	0	0
CHGB	0	0.131	0	0	0.197	0	0	0.126	0.244	0.122	0	0	0	0.12	0	0
UBE2D4	0	0	0	0	0.197	0	0	0.135	0.126	0	0	0	0	0.482	0	0
JRK	0	0	0.125	0	0	0.135	0.136	0.104	0	0.163	0	0	0	0	0	0.275
SPG29	0	0.177	0	0.351	0	0	0.163	0	0	0	0	0.135	0.112	0	0	0
CTPL1	0	0	0	0.106	0.16	0	0	0	0.191	0.288	0	0.192	0	0	0	0
LARS2	0	0.176	0	0	0	0	0.178	0	0.349	0	0.108	0	0	0.122	0	0
ASPG3	0.108	0	0.123	0	0	0.335	0.146	0.104	0	0	0	0	0.116	0	0	0
GJD2	0	0.21	0	0.1	0	0.153	0.165	0.139	0	0.165	0	0	0	0	0	0
MS4A4A	0	0	0.112	0	0.139	0	0	0.128	0.158	0	0	0	0.127	0.13	0.137	0
SERF1B	0	0.192	0	0.101	0.159	0	0	0	0.106	0.144	0	0	0	0.229	0	0
C9ORF4	0	0.1	0	0	0.198	0.247	0.147	0.122	0.117	0	0	0	0	0	0	0
GABRA5	0	0	0.114	0	0	0.198	0.125	0.148	0	0.235	0	0	0.111	0	0	0
CACNA1A	0	0	0	0	0	0.195	0.125	0.13	0	0.116	0	0	0	0.146	0	0.218
PSMC1	0	0	0	0	0.171	0	0	0.163	0.141	0	0.108	0	0	0.112	0.235	0
PABPC1P2	0	0	0	0.269	0	0	0	0	0.119	0	0.439	0	0	0	0.103	0

SYN2	0	0	0.129	0	0	0.152	0.141	0.171	0	0.22	0	0	0.116	0	0	0
ZBTB11	0.138	0.125	0	0	0	0	0	0	0.123	0	0.428	0	0	0	0.113	0
MRX50	0	0	0.107	0	0	0.166	0.164	0	0	0	0	0	0.49	0	0	0
AUTS5	0.113	0	0.124	0	0	0.178	0.152	0	0	0	0	0	0.36	0	0	0
KCNMB3P1	0	0	0	0	0	0.195	0.116	0.123	0	0	0.192	0	0	0	0	0.3
IMMP2L	0	0	0.196	0	0	0.174	0.133	0	0	0	0	0	0.196	0	0	0.226
IL6	0	0	0	0	0.13	0	0.125	0	0.448	0	0	0	0	0	0	0.222
METTL2A	0.103	0	0.137	0	0.185	0	0	0.127	0.202	0	0	0	0	0.17	0	0
PCBP3	0.124	0.144	0	0	0.257	0	0	0.137	0.147	0	0	0	0	0.115	0	0
WWC1	0	0	0.125	0	0.161	0	0	0.109	0.247	0.173	0	0	0.109	0	0	0
PTOS1	0	0	0.169	0.342	0	0	0	0	0	0.16	0	0.147	0.105	0	0	0
GUST	0	0	0.188	0	0	0.188	0.119	0.121	0	0.152	0	0	0.155	0	0	0
CH25H	0.113	0	0.125	0	0.188	0	0	0.112	0.269	0	0	0	0	0.115	0	0
MRT12	0	0	0.122	0	0	0.129	0.133	0.121	0	0.174	0	0.117	0.125	0	0	0
APH1A	0.428	0	0	0	0.174	0	0	0.102	0.113	0	0	0	0	0.103	0	0
FRAXA	0	0	0.177	0	0.121	0	0.102	0.131	0	0	0.141	0	0.131	0	0.116	0
SPTBN2	0	0.116	0	0	0	0.202	0	0	0	0.109	0.127	0	0	0.103	0.261	0
HPCAL1	0	0	0.115	0	0.168	0	0	0.106	0.106	0.172	0	0	0.107	0.144	0	0
SLC1A6	0	0.121	0	0	0	0.201	0.128	0.112	0	0.132	0.106	0	0	0.118	0	0
TUBA1C	0	0	0	0.129	0.345	0	0	0	0.444	0	0	0	0	0	0	0
C9ORF11	0.147	0.103	0	0	0.153	0	0	0.116	0.102	0.15	0	0	0	0.145	0	0
FSHMD1B	0	0.125	0	0.104	0.248	0	0	0	0.174	0.15	0	0.115	0	0	0	0
UBE2L1	0	0	0	0	0.14	0	0	0.197	0.128	0	0	0	0	0.45	0	0
GABRA6	0	0	0	0	0	0.198	0.14	0.108	0	0.222	0	0	0	0	0	0.244
ANIB3	0	0	0	0.107	0.535	0	0	0	0.165	0	0	0.105	0	0	0	0
SEPT1	0	0	0	0	0.532	0	0	0.127	0.251	0	0	0	0	0	0	0
SCZD7	0	0	0.135	0	0	0	0	0	0.207	0.222	0	0	0.161	0	0	0.184
ABCA2	0	0	0	0.118	0.201	0	0	0.105	0.166	0.176	0	0	0	0.142	0	0
MME	0	0	0.105	0	0.194	0	0	0.111	0.382	0.116	0	0	0	0	0	0
TOMM40	0.109	0	0.145	0	0.174	0	0	0.101	0.175	0	0	0	0.102	0.102	0	0
MRX65	0	0	0.111	0.218	0	0.215	0.161	0	0	0	0	0	0.201	0	0	0
VGF	0	0.102	0	0	0.156	0	0	0.109	0.102	0.289	0	0	0	0.148	0	0
LPPR4	0	0	0.208	0	0	0.196	0.154	0.123	0	0.109	0	0	0.116	0	0	0
EEF1A2	0	0.118	0	0	0.269	0	0	0.121	0.107	0.187	0	0	0	0.103	0	0
GABRA2	0	0.114	0.13	0	0	0.121	0.144	0.123	0	0.273	0	0	0	0	0	0
ALDOC	0	0.111	0	0	0	0.122	0.142	0.172	0	0.222	0	0	0	0.135	0	0
FRMD7	0	0	0	0.189	0	0.119	0.153	0	0	0	0	0.143	0.111	0	0	0.188
HCA1	0.129	0	0.335	0	0	0.136	0.169	0	0	0	0	0	0.133	0	0	0
CRBN	0	0	0.105	0	0	0.197	0.138	0.151	0	0	0	0	0.109	0	0	0.202
GTS	0	0	0.112	0.116	0	0.16	0	0	0	0	0	0.243	0.125	0	0	0.145
ZC3H15	0	0	0	0	0.128	0	0	0.381	0.278	0	0	0	0	0.114	0	0

APBA3	0.297	0	0	0	0.184	0	0	0.104	0.172	0	0	0	0	0.143	0	0
SCZD10	0.103	0	0.355	0	0	0	0	0	0.206	0.122	0	0	0.114	0	0	0
DGCR	0	0	0.209	0	0.191	0.132	0.125	0.129	0	0	0	0	0.113	0	0	0
USP31	0	0	0	0	0.152	0	0	0	0.149	0	0.263	0	0	0.178	0	0.156
RAB3GAP2	0	0	0.138	0.216	0	0.17	0.147	0	0	0	0	0	0.124	0	0.102	0
ALDH5A1	0	0	0.125	0	0	0.129	0.134	0.134	0	0.126	0	0	0.125	0.124	0	0
SYNPR	0	0	0.156	0	0.189	0.132	0	0.133	0	0	0	0	0.184	0.103	0	0
MRT11	0	0	0.111	0	0	0.129	0.15	0.137	0	0.142	0	0.103	0.125	0	0	0
MSD	0.134	0	0.123	0	0	0.189	0.157	0.143	0	0	0	0	0.151	0	0	0
MHAC	0.105	0	0.156	0	0	0.142	0	0	0	0	0	0.119	0.167	0	0	0.207
LCS1	0	0	0.101	0.216	0	0.198	0.152	0	0	0	0	0.102	0.127	0	0	0
ANKS1B	0.257	0.11	0	0	0.272	0	0	0.123	0.134	0	0	0	0	0	0	0
BEAN1	0	0	0	0	0	0	0	0	0	0	0.241	0	0	0.136	0.205	0.314
SCN3A	0	0	0	0	0	0.197	0.244	0.123	0	0	0	0.146	0	0	0	0.184
PPR3	0	0	0	0	0	0.198	0.112	0.16	0	0.2	0	0	0	0	0	0.223
KLK6	0	0.122	0	0	0.204	0	0	0.106	0.175	0	0	0	0	0.284	0	0
CHMP4B	0	0	0	0	0.224	0	0	0.221	0.333	0	0	0	0	0.112	0	0
GAL	0	0.112	0.1	0	0.166	0	0	0.108	0.14	0.126	0	0	0.138	0	0	0
ANKRD11	0	0	0.203	0	0	0.152	0.208	0	0	0	0	0	0.198	0	0	0.129
APLP2	0.113	0	0.132	0	0.128	0	0	0.103	0.304	0	0	0	0	0.109	0	0
ZNF804A	0	0	0.121	0	0.189	0	0	0.101	0.245	0.127	0	0	0.105	0	0	0
CACNG4	0	0	0	0	0	0.323	0.131	0.107	0	0	0	0	0	0	0	0.326
LDHD	0	0	0	0	0.188	0	0.163	0.12	0.142	0	0.155	0	0	0.119	0	0
CLSTN1	0.116	0	0.129	0	0.173	0	0	0.155	0.202	0	0	0	0	0.112	0	0
GRM8	0	0	0.151	0	0	0.188	0.142	0.117	0	0.175	0	0	0.114	0	0	0
SFXN5	0	0	0	0	0.215	0	0	0	0.139	0	0.132	0	0	0.218	0	0.181
SULT4A1	0	0	0.105	0	0.125	0	0	0.141	0.244	0	0	0	0.142	0	0	0.127
MARK4	0	0	0	0	0.229	0	0	0.263	0.163	0	0	0	0	0	0	0.229
MCOLN1	0	0	0	0	0.149	0	0	0	0.162	0.146	0	0.185	0.13	0.11	0	0
MFN2	0	0	0	0.19	0	0	0	0	0	0	0	0.271	0	0.128	0	0.292
GRID1	0	0	0.185	0	0	0.18	0.212	0	0	0.202	0	0	0	0.101	0	0
GRM3	0	0.124	0.101	0	0	0.14	0.162	0.116	0	0.135	0	0	0.101	0	0	0
DCTN5	0	0.416	0	0	0	0	0	0	0.126	0	0.223	0	0	0	0.113	0
DYX8	0.129	0	0.117	0	0	0	0	0	0.203	0.285	0	0	0.144	0	0	0
SPG24	0	0.113	0	0.362	0	0	0	0	0	0.122	0	0.157	0.123	0	0	0
PANK3	0	0	0	0	0.236	0	0	0	0.184	0	0.136	0	0	0.165	0	0.155
ANMA	0	0	0.101	0.221	0	0.215	0.152	0	0	0	0	0	0.187	0	0	0
TUKLS	0	0	0.145	0.116	0	0	0	0	0	0.199	0	0.201	0.101	0	0	0.114
TBCB	0	0.112	0	0.1	0	0	0	0	0	0	0	0.174	0	0.37	0.12	0
DFNA7	0	0	0.345	0.116	0	0	0	0	0	0.15	0	0.134	0.131	0	0	0
WBSCR2	0	0	0.229	0	0	0.149	0.175	0.103	0	0	0	0	0.22	0	0	0

DDO	0	0	0.221	0	0.247	0	0	0	0.184	0	0.114	0	0.109	0	0	0
LSAMP	0.125	0	0.125	0	0	0.155	0	0.131	0	0.144	0	0	0.194	0	0	0
APLP1	0.12	0	0.149	0	0.188	0	0	0.109	0.194	0	0	0	0	0.111	0	0
ADAM22	0	0.105	0	0.358	0	0.138	0.163	0	0	0	0	0.107	0	0	0	0
CDKL4	0	0	0.146	0	0	0.127	0.136	0.14	0	0	0	0	0.119	0	0	0.202
RCAN1	0.131	0	0.103	0	0.183	0	0	0	0	0	0.107	0	0.113	0.125	0.108	0
LRTOMT	0	0	0.112	0.214	0	0	0	0	0	0.291	0	0.121	0.132	0	0	0
OPA6	0	0.111	0	0.101	0	0	0.147	0	0	0.184	0	0.107	0.104	0.116	0	0
CHRNB2	0	0	0.114	0	0.192	0.134	0	0.135	0	0.186	0	0	0.109	0	0	0
ZDHHC17	0	0	0	0	0	0	0	0	0	0	0.248	0	0	0.146	0.202	0.273
GABRA1	0	0.184	0	0	0	0.205	0.2	0.108	0	0.172	0	0	0	0	0	0
EJM2	0	0	0.106	0	0	0.147	0.116	0.107	0	0.107	0	0	0.146	0	0	0.138
ASPM	0	0	0.143	0	0	0.114	0.114	0.203	0	0.191	0	0	0.101	0	0	0
LRRTM3	0	0	0.125	0	0.171	0	0	0.103	0.214	0	0	0	0.132	0.121	0	0
CCDC134	0.132	0	0.101	0	0.134	0	0	0.101	0.241	0	0	0	0	0	0.156	0
DFNA18	0	0	0.26	0.111	0	0	0	0	0	0.174	0	0.188	0.132	0	0	0
GABRA4	0	0	0.216	0	0	0.148	0.185	0.144	0	0.171	0	0	0	0	0	0
OTSC7	0	0	0.114	0.215	0	0	0	0	0.13	0.144	0	0.141	0.119	0	0	0
NAPSB	0	0	0	0	0.227	0	0	0.185	0.266	0	0	0	0	0.184	0	0
RELN	0	0	0.127	0	0	0.193	0.118	0.191	0	0.104	0	0	0.129	0	0	0
ERVK-23	0	0.118	0	0	0.138	0	0.122	0.107	0.377	0	0	0	0	0	0	0
JOSD1	0.151	0	0	0	0	0	0	0	0	0	0.173	0	0	0	0.405	0.132
DFNA31	0	0	0.229	0.21	0	0	0	0	0	0.181	0	0.14	0.101	0	0	0
ATG3	0	0	0	0	0.221	0	0	0	0.105	0	0.243	0	0	0.176	0.115	0
JRKL	0	0	0	0	0	0.268	0.141	0.109	0	0	0	0	0	0	0	0.34
LRRTM1	0	0	0.101	0	0	0.175	0	0.115	0	0.218	0	0	0.145	0	0.103	0
TSPAN7	0	0	0.103	0	0	0.163	0.107	0.182	0	0	0.189	0	0.113	0	0	0
DFNB15	0	0	0.276	0.116	0	0	0	0	0	0.176	0	0.146	0.14	0	0	0
WDM	0	0.138	0	0.1	0	0	0	0	0.131	0.201	0.157	0.127	0	0	0	0
SORCS1	0.103	0	0.124	0	0.134	0	0	0.126	0.242	0	0	0	0	0.125	0	0
GBA2	0	0	0	0	0.208	0	0	0.253	0.187	0	0	0	0	0.206	0	0
DRD3	0	0	0.109	0	0.28	0	0	0	0	0.133	0	0	0.108	0	0	0.223
CHODL	0	0.107	0	0.364	0	0	0	0	0.107	0.275	0	0	0	0	0	0
ANIB4	0	0	0	0	0.212	0	0	0.13	0.159	0	0	0.161	0	0	0	0.19
KCNQ3	0	0	0	0	0	0.197	0.144	0.158	0	0	0	0.163	0	0	0	0.19
SNPH	0.119	0.132	0	0.101	0	0	0	0	0.141	0.103	0.141	0	0	0.115	0	0
C14ORF28	0	0	0.219	0	0	0	0	0	0	0	0.144	0	0.107	0	0.102	0.279
SCZD2	0	0	0.101	0	0	0	0	0	0.112	0.206	0	0	0.107	0	0	0.325
CADPS2	0	0	0.163	0	0	0.161	0.234	0	0	0	0	0	0.189	0	0.103	0
THAS	0	0	0.143	0	0	0.147	0.133	0	0	0	0	0	0.282	0	0	0.145
MRSS	0	0	0.142	0	0	0.164	0.179	0.118	0	0	0	0	0.246	0	0	0

KCNAB1	0	0	0	0	0	0.269	0.147	0.166	0	0	0	0	0	0	0	0.266
BLMH	0.101	0	0	0	0.184	0	0	0.111	0.209	0	0.114	0	0	0.129	0	0
GRIK4	0	0.113	0.141	0	0	0.179	0.166	0.125	0	0.122	0	0	0	0	0	0
YWHAQP4	0.168	0.115	0	0	0.163	0	0	0.138	0.147	0	0	0	0	0.114	0	0
FAM134B	0	0	0	0.187	0.223	0	0	0.128	0.155	0	0	0.152	0	0	0	0
CDKL5	0	0	0.182	0	0	0.148	0.116	0.101	0	0	0	0	0.128	0	0	0.17
MRX18	0	0	0.185	0.116	0	0.193	0.154	0	0	0	0	0	0.196	0	0	0
GAD1	0	0.194	0.11	0	0	0.127	0.162	0.112	0	0.139	0	0	0	0	0	0
ND1	0	0	0	0	0.222	0	0.162	0	0.14	0	0	0.176	0	0.143	0	0
CLN9	0	0	0	0	0.211	0	0	0.283	0.167	0	0	0	0	0.182	0	0
DFNB55	0	0	0.109	0.214	0	0	0	0	0	0.297	0	0.101	0.122	0	0	0
DFNB47	0	0	0	0.256	0	0	0	0	0	0.236	0	0.204	0.147	0	0	0
KCNMB4	0	0	0	0	0	0.284	0.231	0.108	0	0	0	0	0	0	0	0.219
C20ORF203	0	0	0.113	0	0.172	0	0	0.16	0.158	0	0	0	0.122	0.116	0	0
METTL2B	0.125	0	0.14	0	0.186	0	0	0.101	0.167	0	0	0	0	0.121	0	0
GIGYF1	0	0.109	0	0	0.138	0	0	0	0.127	0	0	0	0	0.115	0.116	0.235
KCNS1	0	0	0	0	0	0.197	0.275	0.109	0	0	0	0.13	0	0	0	0.128
NDUFAF2	0	0	0.119	0	0	0	0.155	0.122	0	0	0	0.128	0.148	0.167	0	0
DFN6	0	0	0.146	0	0	0	0	0	0	0.136	0	0.129	0.428	0	0	0
ERVW-2	0	0.164	0	0	0.267	0	0	0.133	0.161	0	0.113	0	0	0	0	0
CACNA1H	0	0	0	0	0	0.154	0.165	0.162	0	0	0	0	0	0	0	0.356
LRRC1	0	0	0	0.184	0	0.162	0.142	0.108	0	0	0	0	0	0	0	0.24
SPPL3	0.113	0	0	0	0.297	0	0	0.145	0.165	0	0	0	0	0.115	0	0
SNAP25	0	0	0.136	0	0	0.166	0	0	0	0.163	0.102	0	0.151	0	0.116	0
GMFB	0.119	0.169	0	0	0.272	0	0	0	0.147	0	0	0	0	0.127	0	0
HCN2	0	0	0	0	0	0.268	0.139	0.121	0	0	0	0	0	0	0	0.304
MOPCB2	0	0	0	0.187	0	0	0	0	0	0	0	0.139	0.218	0	0	0.287
IP6K3	0	0	0	0	0	0	0	0	0	0	0.347	0	0	0	0.484	0
GLRA4	0	0.12	0	0	0	0.155	0.112	0.132	0	0.311	0	0	0	0	0	0
WDFY3	0	0.106	0	0	0.156	0	0	0	0.165	0	0.165	0	0	0.122	0.115	0
COQ2	0	0	0	0	0	0	0.117	0	0	0	0	0.144	0	0.227	0	0.341
DTNBP1	0	0	0.113	0	0	0	0	0	0.148	0.134	0	0	0.109	0	0	0.325
WHAMMP3	0.206	0	0.227	0	0	0.134	0.15	0	0	0	0	0	0.112	0	0	0
GSK3A	0.278	0.125	0	0	0.163	0	0	0	0.161	0	0	0	0	0.101	0	0
EFHC2	0	0	0.116	0	0	0.153	0.136	0.112	0	0	0	0	0.128	0	0	0.182
OTOP1	0	0	0.136	0	0	0	0	0	0	0.129	0.13	0.113	0.109	0	0.21	0
TMEM185A	0.277	0	0.113	0	0	0	0	0	0	0	0.197	0	0.126	0	0.114	0
UFL1	0	0	0	0	0	0	0	0	0.297	0	0.146	0	0	0.109	0.274	0
LGMD1D	0	0.112	0	0.352	0	0	0	0	0.123	0.13	0	0.109	0	0	0	0
UPP2	0.124	0	0.165	0	0.174	0	0	0.103	0.146	0	0	0	0.113	0	0	0
SH3TC1	0	0	0	0.236	0	0	0	0	0	0	0	0.221	0	0	0	0.368

NR4A2	0	0	0	0	0	0	0	0	0	0.126	0	0	0	0.42	0.105	0.173
MRX72	0	0	0.159	0	0	0.209	0.145	0.152	0	0	0	0	0.159	0	0	0
GSK3B	0.113	0	0	0	0	0	0	0	0.135	0	0.173	0	0	0.122	0	0.281
CLIP2	0	0.101	0.105	0.305	0	0	0	0	0	0	0	0.103	0.21	0	0	0
ELAVL4	0	0.122	0	0	0.247	0	0	0	0.274	0.18	0	0	0	0	0	0
CCT8L2	0	0	0	0	0	0.125	0.193	0.122	0	0	0.161	0	0	0	0	0.221
SCN4A	0	0	0	0.111	0	0.162	0.143	0.1	0	0	0	0.107	0	0	0	0.198
GDNF	0	0.173	0	0	0	0	0	0	0	0.107	0.135	0	0	0.406	0	0
UBAP1	0.101	0.159	0	0	0.163	0	0	0.102	0.103	0.193	0	0	0	0	0	0
CHRNB4	0	0	0.22	0	0	0.19	0	0.101	0	0.181	0	0	0.128	0	0	0
FAM72A	0	0	0	0	0.229	0	0	0.321	0.168	0	0	0	0	0.101	0	0
DBH	0	0	0.103	0	0.283	0	0	0	0	0.118	0	0	0.107	0	0	0.208
TEX261	0	0.117	0	0	0	0	0.111	0	0	0	0.107	0	0	0.188	0.296	0
PARK16	0	0	0	0	0.222	0	0	0.12	0.142	0	0	0	0	0.219	0.116	0
SMS	0	0	0.151	0	0	0	0.133	0	0	0	0	0	0.422	0	0.113	0
PAFAH1B1	0	0.19	0.108	0	0	0.14	0.162	0.114	0	0	0	0	0.105	0	0	0
OPA5	0	0.123	0	0.1	0	0	0.119	0	0	0.168	0	0.183	0	0.125	0	0
RMD1	0	0	0	0.106	0	0	0	0	0	0.178	0	0.17	0.206	0	0	0.157
KLHL4	0	0	0	0	0	0.123	0	0.124	0	0	0.107	0	0.129	0	0.333	0
GABRA3	0	0	0.172	0	0	0.171	0.13	0.13	0	0.213	0	0	0	0	0	0
KLC2	0.139	0.128	0	0.101	0	0	0	0	0	0	0.138	0.17	0	0.139	0	0
PPP1R1B	0	0	0.102	0	0	0	0	0	0	0.145	0.142	0	0.139	0	0	0.286
TRIM9	0	0	0.136	0	0.159	0	0	0.101	0.195	0	0	0	0	0.107	0.116	0
NXPH1	0.147	0	0.106	0	0.184	0	0	0.111	0.15	0	0	0	0.115	0	0	0
CELSR2	0.155	0.111	0	0	0.237	0	0	0	0.31	0	0	0	0	0	0	0
C16ORF57	0	0	0	0.155	0	0.245	0.143	0	0	0	0	0.106	0.163	0	0	0
PRPF40B	0	0	0.12	0	0	0.168	0.205	0.145	0	0	0	0	0.174	0	0	0
CAPS2	0.116	0	0.185	0	0	0.116	0.154	0	0	0	0	0	0.138	0	0.103	0
ZNF778	0	0	0.107	0	0	0.215	0.111	0.104	0	0	0	0	0.274	0	0	0
NLGN4X	0	0	0.106	0	0	0.125	0.228	0.101	0	0	0	0	0.251	0	0	0
MIR134	0.147	0	0.319	0	0	0.12	0	0.102	0	0	0	0	0.123	0	0	0
GABRG2	0	0	0	0	0	0.122	0.112	0.188	0	0.227	0	0	0	0	0	0.161
PRPH	0	0.108	0	0.1	0	0	0	0	0.165	0.201	0	0.103	0	0.132	0	0
LDLRAD3	0	0.109	0.121	0	0	0.161	0.145	0.116	0	0	0	0	0	0	0.155	0
CP	0	0.127	0	0	0.2	0	0	0	0	0.344	0	0	0	0.136	0	0
GUSBP1	0	0.185	0	0.496	0	0	0	0	0	0	0.126	0	0	0	0	0
CDKL3	0	0	0.119	0	0	0.152	0.142	0.208	0	0	0	0	0.186	0	0	0
PNMA6D	0	0	0	0	0	0.244	0.288	0.13	0	0	0	0	0	0.144	0	0
CGF1	0.125	0	0.126	0	0	0.142	0.157	0	0	0	0	0	0.256	0	0	0
CACNA2D1	0	0	0	0	0	0.196	0.1	0.109	0	0	0.18	0	0	0	0	0.221
MED12	0.125	0	0.138	0	0.187	0	0	0.111	0	0	0.124	0	0.12	0	0	0

PAWR	0	0.152	0	0	0.2	0	0	0	0.158	0	0	0	0	0.295	0	0
ATXN2L	0	0	0	0	0	0	0	0	0.1	0	0.134	0	0	0.189	0.111	0.27
GABBR2	0	0.16	0.115	0	0	0.121	0.144	0.1	0	0.164	0	0	0	0	0	0
ERVK-12	0	0.115	0	0	0.17	0	0.164	0.1	0.255	0	0	0	0	0	0	0
HOMER1	0	0	0.115	0	0	0	0	0	0	0.188	0.145	0	0.125	0	0	0.231
GTF2IRD2P1	0	0	0.216	0.215	0	0	0.123	0	0	0	0	0.128	0.121	0	0	0
CDK18	0	0.111	0	0	0.204	0	0	0.128	0.157	0	0	0	0	0.203	0	0
PLS3	0	0.22	0	0	0	0	0	0	0	0	0.179	0	0	0	0.403	0
TTC19	0	0	0.106	0.116	0	0	0.103	0	0	0	0	0.135	0.16	0.182	0	0
CMD1B	0	0	0.11	0.111	0	0	0.189	0	0	0.155	0	0.134	0.103	0	0	0
CNBP	0	0	0	0.184	0.366	0	0	0	0.126	0	0.126	0	0	0	0	0
ACRC	0	0	0	0.183	0.135	0	0	0	0.149	0	0.114	0	0	0	0	0.221
GLRA2	0	0	0	0	0	0.288	0.131	0.12	0	0	0	0	0	0	0	0.263
ST13	0	0	0	0	0.189	0	0	0	0.155	0	0	0	0	0.235	0.223	0
CNTN4	0	0	0.125	0	0	0.206	0.137	0.209	0	0	0	0	0.125	0	0	0
PARL	0	0	0	0.107	0.267	0	0	0	0.107	0	0	0	0	0.113	0	0.207
CHRNA2	0	0	0.12	0	0	0.233	0	0.102	0	0.245	0	0	0.101	0	0	0
OHDS	0	0	0	0.183	0	0	0	0	0.104	0	0	0.187	0.139	0	0	0.187
MRX51	0	0	0.111	0	0	0.16	0.178	0.12	0	0	0	0	0.231	0	0	0
PCSK1N	0	0.122	0	0	0.255	0	0	0.145	0.174	0	0	0	0	0.102	0	0
HDAC6	0	0.107	0	0	0.255	0	0	0	0.201	0	0.119	0	0	0.114	0	0
TMEM126A	0	0	0.109	0.105	0	0	0.199	0	0	0.155	0	0.102	0.125	0	0	0
CORT	0	0	0	0	0.152	0	0	0.101	0.126	0	0.275	0	0	0.14	0	0
SCZD3	0	0	0.134	0	0	0	0	0	0.104	0.123	0	0	0.172	0	0	0.261
LRP8	0.13	0	0.145	0	0.178	0	0	0.132	0.209	0	0	0	0	0	0	0
ILVBL	0	0	0	0	0.173	0	0	0.324	0.17	0	0	0	0	0	0	0.125
SPG16	0	0	0.12	0.111	0	0.137	0.147	0	0	0	0	0.176	0.101	0	0	0
TSC2	0	0.178	0.105	0	0	0.394	0	0.115	0	0	0	0	0	0	0	0
KCNMB2	0	0	0	0	0	0.288	0.154	0.1	0	0	0	0	0	0	0	0.25
PION	0.152	0	0.143	0	0.159	0	0	0.119	0.114	0	0	0	0	0.104	0	0
GMPPB	0	0	0	0	0.213	0	0	0.165	0	0	0.167	0	0	0.114	0	0.131
CHMP4C	0.116	0	0	0	0.181	0	0	0.102	0.244	0	0	0	0	0.147	0	0
SYN3	0	0	0.114	0	0	0.156	0.122	0.103	0	0.176	0	0	0.119	0	0	0
TSC1	0	0.121	0.129	0	0	0.16	0	0.132	0	0	0	0	0.248	0	0	0
LPPR2	0	0	0.103	0	0	0.132	0.113	0.137	0	0.197	0	0	0.108	0	0	0
PDR	0	0	0.147	0.219	0	0	0.113	0	0	0	0.182	0	0.128	0	0	0
TELAB1	0	0	0	0.362	0	0	0	0	0	0	0	0.324	0	0	0	0.102
GPR155	0	0	0	0	0	0	0	0.145	0	0	0.226	0	0	0.135	0.281	0
MAP3K15	0	0	0	0	0.235	0	0	0.116	0.142	0	0.157	0	0	0.137	0	0
VSTM2L	0	0	0.104	0	0.145	0	0	0.111	0.309	0	0	0	0	0.118	0	0
SLC6A1	0	0	0.103	0	0	0.165	0.115	0.103	0	0.195	0	0	0.106	0	0	0

NXF5	0	0	0.148	0	0	0.244	0.124	0.116	0	0	0	0	0.155	0	0	0
ATXN7L3	0	0	0	0	0	0	0	0	0	0	0.133	0	0	0.136	0.204	0.314
SYN1	0	0	0.107	0	0	0.142	0.123	0.103	0	0.208	0	0	0.104	0	0	0
C11ORF66	0	0	0	0	0.141	0.155	0	0.141	0.168	0	0	0	0	0.181	0	0
ZNF674	0	0	0.107	0	0	0.171	0.175	0.104	0	0	0	0	0.229	0	0	0
RBM20	0	0	0	0.189	0	0.129	0.155	0	0	0	0	0.173	0	0	0	0.14
UBE3A	0	0	0.116	0	0	0.107	0.153	0.112	0	0	0	0	0.151	0	0	0.146
MS4A4E	0	0.113	0.121	0	0.207	0	0	0.181	0.163	0	0	0	0	0	0	0
PICALM	0	0	0.122	0	0.193	0	0	0.103	0.122	0	0	0	0.124	0.12	0	0
IGF1	0	0.42	0	0	0	0	0	0	0	0	0.127	0	0	0	0	0.237
ATP1A3	0	0	0	0	0	0.198	0.114	0.108	0	0.146	0	0	0	0	0	0.218
DAGLA	0	0	0.122	0	0	0.143	0.157	0	0	0	0.203	0	0.157	0	0	0
TRIM47	0	0.108	0.102	0	0.166	0	0	0.112	0.177	0	0	0	0.117	0	0	0
NKAIN1	0	0	0	0	0	0.218	0.19	0.134	0	0	0	0	0	0	0	0.239
DIP2C	0.138	0	0.37	0	0	0	0	0	0	0	0.151	0	0.122	0	0	0
MRX53	0	0	0.134	0	0	0.221	0.156	0.138	0	0	0	0	0.132	0	0	0
MRX25	0	0	0.183	0	0	0.171	0.25	0	0	0	0	0	0.177	0	0	0
NTF3	0	0.155	0	0.105	0	0	0	0	0	0.206	0.141	0.174	0	0	0	0
EXOC3L2	0	0	0.14	0	0.145	0	0	0.152	0.218	0	0	0	0	0.125	0	0
DYT17	0	0	0	0.164	0	0.137	0.142	0	0	0.108	0	0.104	0	0	0	0.124
CDNF	0	0.103	0	0	0.17	0	0	0	0.105	0.16	0.132	0	0	0.109	0	0
KIAA2022	0	0	0.103	0	0	0.172	0.135	0.14	0	0	0	0	0.229	0	0	0
ALG5	0	0	0	0	0	0.214	0.15	0	0	0	0	0	0.231	0	0	0.184
SH3GL1	0	0	0	0.368	0	0	0	0	0	0	0.298	0	0	0	0.113	0
FRA10A	0.126	0	0	0	0	0	0	0	0	0	0.229	0	0	0	0.424	0
DYT2	0	0	0	0.155	0	0.175	0.165	0	0	0	0	0.122	0	0	0	0.16
KCNQ5	0	0	0	0	0	0.234	0.192	0.108	0	0	0	0	0	0	0	0.242
AQP4	0.131	0.161	0	0	0	0.177	0.108	0	0	0.198	0	0	0	0	0	0
CAPN1	0	0.11	0	0	0.153	0	0	0	0	0.129	0.17	0	0	0.108	0.105	0
PSMD8	0	0	0	0	0.191	0	0	0.133	0.142	0	0.103	0	0	0.101	0.105	0
LRRC15	0.115	0	0	0	0.181	0	0	0.123	0.254	0	0	0	0	0.101	0	0
ATP5J2	0	0	0	0	0.219	0	0.124	0.107	0.118	0	0	0	0	0.205	0	0
ATP1B2	0	0	0	0	0	0.212	0.24	0.1	0	0.221	0	0	0	0	0	0
CACNA2D4	0	0	0	0	0	0.132	0	0	0	0.292	0	0	0	0	0	0.348
CACNA2D2	0	0	0	0	0	0.154	0.125	0.164	0	0	0	0	0	0	0	0.329
ATP6V0C	0	0	0	0	0.234	0.326	0	0.102	0	0	0	0	0	0.11	0	0
APTX	0	0	0	0.184	0	0	0	0	0	0	0.176	0.107	0.145	0.159	0	0
SPPM	0	0	0	0.158	0	0	0	0	0	0.106	0	0.118	0	0	0.101	0.287
FAM193A	0	0.135	0	0	0	0	0	0.138	0	0	0.117	0	0	0.105	0.275	0
CCBL1	0	0	0	0	0	0	0	0	0	0	0.455	0	0	0.125	0.19	0
DFNA57	0	0	0.117	0.214	0	0	0	0	0	0.208	0	0.101	0.13	0	0	0

EKV3	0	0	0.148	0.113	0	0	0	0	0	0	0	0.219	0.29	0	0	0
NPAS4	0	0	0.172	0	0	0.136	0.118	0.133	0	0.105	0	0	0.106	0	0	0
GLS2	0	0	0	0	0.244	0	0.127	0.125	0	0	0.139	0	0	0.134	0	0
SSR4	0	0.123	0	0	0.163	0	0	0	0.122	0.254	0	0	0	0.107	0	0
HECW2	0	0.149	0	0	0.4	0	0	0	0.113	0	0	0	0	0.107	0	0
AUTS8	0.102	0	0.123	0	0	0.14	0.138	0.109	0	0	0	0	0.157	0	0	0
NDUFS1	0	0	0	0	0.213	0	0.146	0	0	0	0	0.11	0	0.164	0.135	0
KCNA1	0	0	0	0.106	0	0.163	0.142	0.105	0	0	0	0.119	0	0	0	0.132
PCDH11X	0	0	0.121	0	0.193	0	0	0.102	0.219	0	0	0	0.132	0	0	0
MRX42	0	0	0.101	0	0	0.137	0.211	0	0	0	0	0	0.318	0	0	0
NXF4	0	0	0.144	0	0	0.221	0.184	0.102	0	0	0	0	0.116	0	0	0
NAE1	0	0	0	0	0.246	0	0	0.208	0.14	0	0	0	0	0.172	0	0
FAM58A	0	0	0.133	0	0	0.158	0.279	0	0	0	0	0	0.196	0	0	0
MRX80	0	0	0.235	0	0	0.176	0.223	0	0	0	0	0	0.132	0	0	0
KCNV2	0	0	0	0	0	0.15	0.105	0.19	0	0.176	0	0	0	0	0	0.144
LETM1	0	0	0	0	0	0	0.114	0	0	0	0.191	0	0	0.172	0.288	0
NKAIN4	0.134	0	0	0	0	0.192	0.133	0.109	0	0	0	0	0	0	0	0.196
TMEM74	0	0	0	0	0	0	0	0	0.228	0	0.128	0	0	0.133	0.275	0
MDRV	0	0	0	0.236	0	0	0	0	0	0.202	0	0.326	0	0	0	0
EPC2	0.113	0	0.125	0	0.178	0	0	0.111	0.237	0	0	0	0	0	0	0
SLC41A1	0	0	0	0	0.152	0	0	0.105	0.142	0	0	0	0	0.145	0.218	0
DAO	0	0.111	0.147	0	0	0	0	0	0.121	0.208	0	0	0.175	0	0	0
NGF	0	0.137	0	0	0.138	0	0	0	0.22	0.161	0	0.106	0	0	0	0
ATP10A	0	0	0.138	0	0	0.119	0.152	0.126	0	0	0	0	0.226	0	0	0
DRD4	0	0	0.147	0	0.163	0	0	0	0	0.188	0	0	0.132	0	0	0.13
DLG3	0	0	0	0.429	0	0	0	0	0	0	0.1	0	0	0	0	0.231
HERC1	0	0.118	0	0.484	0	0	0	0	0	0.157	0	0	0	0	0	0
CCM2	0	0	0	0	0	0.196	0.101	0.105	0	0	0	0.158	0	0	0	0.198
LVG2	0.183	0	0.156	0	0.176	0	0	0	0.143	0	0	0	0.1	0	0	0
TK2	0	0	0	0.158	0	0	0.133	0	0	0	0	0.136	0	0.33	0	0
CCNP	0	0	0	0.349	0	0	0	0	0	0	0	0.408	0	0	0	0
NRN1L	0	0	0.131	0	0	0.19	0.152	0.107	0	0.176	0	0	0	0	0	0
HCN3	0	0	0	0	0	0.213	0	0.111	0	0	0.137	0	0	0	0	0.294
MRX26	0	0	0.137	0.116	0	0.211	0.139	0	0	0	0	0	0.152	0	0	0
GLULP1	0.126	0	0	0	0	0	0	0	0	0	0.208	0	0	0	0.421	0
HS1BP3	0	0	0	0	0.211	0	0	0.101	0.194	0	0	0	0	0.116	0	0.132
CELF4	0	0	0	0	0	0.217	0	0.109	0.215	0	0	0	0	0	0	0.213
CASP6	0	0.125	0	0	0.139	0	0	0	0.163	0	0.204	0	0	0.122	0	0
SLC17A6	0	0.109	0.105	0	0	0.132	0	0.115	0	0.153	0	0.139	0	0	0	0
SYNGAP1	0	0	0.15	0	0	0.154	0.107	0.118	0	0.105	0	0	0.119	0	0	0
WDR62	0	0	0.106	0	0	0.194	0.133	0.103	0	0	0	0	0.216	0	0	0

SUPT3H	0	0	0	0	0	0	0	0	0	0	0.241	0	0	0.171	0.117	0.222
USP48	0	0	0	0	0.21	0	0	0	0.103	0	0.134	0	0	0.168	0	0.136
LOC619539	0	0	0	0.306	0	0	0	0	0	0	0	0.193	0	0	0	0.252
APOE	0.113	0	0	0	0.184	0	0	0.102	0.196	0	0	0	0	0	0.156	0
SSD	0	0	0.146	0	0	0.145	0.15	0	0	0.174	0	0	0.136	0	0	0
DFN4	0	0	0.152	0	0	0	0	0	0	0.14	0	0.342	0.116	0	0	0
RNF170	0	0	0	0.269	0	0	0	0	0	0	0.262	0.104	0	0.115	0	0
NRXN1	0	0	0.144	0	0	0.162	0	0.101	0	0	0	0	0.343	0	0	0
KCNMB3	0	0	0	0	0	0.285	0.122	0.134	0	0	0	0	0	0	0	0.208
ATP1A2	0	0	0	0	0	0.241	0.155	0.105	0	0	0	0	0	0	0	0.247
FEB6	0	0	0	0	0	0.289	0.167	0.12	0	0	0	0	0	0	0	0.172
CIC	0	0	0	0	0	0	0	0.141	0	0	0.183	0	0	0.143	0.281	0
MFN1	0	0	0	0.218	0	0	0	0	0	0	0	0.146	0	0.112	0	0.271
CREB1	0	0	0	0	0	0.216	0.153	0	0	0	0.159	0	0	0	0	0.219
CST2	0	0	0	0	0.166	0	0.228	0.12	0.233	0	0	0	0	0	0	0
MTHFR	0	0	0	0	0.215	0	0.127	0.133	0.272	0	0	0	0	0	0	0
NTNG1	0	0	0.103	0	0	0.16	0.175	0.14	0	0	0	0	0.168	0	0	0
BSN	0	0	0.129	0	0	0.144	0.145	0	0	0.211	0	0	0	0	0.117	0
PON3	0	0.19	0	0	0.443	0	0	0	0.113	0	0	0	0	0	0	0
MAP1LC3C	0	0	0	0	0.114	0	0	0	0.138	0	0.248	0	0	0.134	0.111	0
TBC1D22A	0	0	0	0	0	0	0	0	0	0	0.125	0	0	0	0.354	0.265
CACNB4	0	0	0	0	0	0.29	0.101	0.105	0	0	0	0	0	0	0	0.248
MMVP2	0	0	0	0.3	0.192	0	0	0.13	0	0	0	0	0	0	0	0.121
OPA1	0	0	0	0.269	0	0	0	0	0	0.122	0	0.215	0	0.137	0	0
PSMC6	0	0	0	0	0.142	0	0	0.12	0.241	0	0.119	0	0	0.121	0	0
HAR1B	0	0	0	0	0	0.212	0.293	0.121	0.117	0	0	0	0	0	0	0
ERVK-2	0	0.103	0	0	0.158	0	0.164	0.1	0.218	0	0	0	0	0	0	0
SPPL2B	0.128	0	0	0	0.185	0	0	0.111	0.177	0	0	0	0	0.141	0	0
ZNF630	0	0	0.162	0	0	0.22	0.102	0.113	0	0	0	0	0.145	0	0	0
APBA1	0.184	0	0	0	0.184	0	0	0.112	0.142	0	0	0	0	0.119	0	0
COHEN2	0	0	0.162	0	0	0.174	0.2	0	0	0	0	0	0.205	0	0	0
GABRB2	0	0	0	0	0	0.15	0.127	0.108	0	0.193	0	0	0	0	0	0.161
BAHCC1	0.169	0	0	0	0	0	0	0	0	0	0	0	0	0	0.248	0.321
MRX14	0	0	0.125	0	0	0.162	0.104	0.115	0	0	0	0	0.109	0	0	0.123
MGR5	0	0	0	0	0	0.199	0.11	0.126	0	0.18	0	0	0	0	0	0.123
VARS2	0	0	0	0.167	0	0	0.132	0	0	0	0	0.104	0	0.335	0	0
IFT74	0.217	0.125	0	0	0.23	0	0	0	0.166	0	0	0	0	0	0	0
MBD5	0	0	0.105	0	0	0.247	0.138	0.117	0	0	0	0	0.13	0	0	0
KTCN4	0.152	0.106	0	0	0.268	0	0	0	0.211	0	0	0	0	0	0	0
CCDC22	0	0.482	0	0	0	0	0	0	0	0	0	0.254	0	0	0	0
CMAL	0	0.101	0	0	0	0.156	0	0	0.173	0	0	0	0	0	0	0.305

NDUFB11	0	0	0	0.185	0	0	0.121	0	0	0.196	0	0.104	0	0.129	0	0
SLC9A6	0	0	0	0	0	0.123	0.204	0.13	0.164	0	0	0	0	0.113	0	0
FGS2	0	0	0.155	0	0	0.172	0.16	0	0	0	0	0	0.247	0	0	0
DFNB44	0	0	0.111	0.215	0	0	0	0	0	0.15	0	0.113	0.144	0	0	0
KLK8	0	0	0.115	0	0	0.19	0	0.118	0	0.194	0	0	0.116	0	0	0
NAT8B	0	0	0	0	0.228	0	0	0.277	0.111	0	0	0	0	0.116	0	0
TRIM11	0	0	0	0	0.197	0	0	0.144	0.208	0	0	0	0	0.183	0	0
CALB2	0	0.112	0	0	0	0.209	0.101	0	0	0.193	0.117	0	0	0	0	0
QPRT	0	0	0	0	0	0.245	0.142	0.122	0	0	0.223	0	0	0	0	0
MRX75	0	0	0.134	0	0	0.159	0.152	0	0	0	0	0	0.286	0	0	0
RGNEF	0	0.125	0	0.437	0	0	0	0	0.169	0	0	0	0	0	0	0
PSENEN	0.105	0	0	0	0.185	0	0	0.155	0.18	0	0	0	0	0.106	0	0
GPR98	0	0	0.17	0	0	0.19	0	0	0	0.116	0	0.118	0.137	0	0	0
LMO3	0	0	0.153	0	0	0	0	0	0	0.211	0	0	0.107	0	0	0.259
ATG14	0	0	0	0	0	0	0	0	0.139	0	0.194	0	0	0.118	0.279	0
SPG20	0	0	0	0.269	0	0	0	0	0.177	0	0	0.16	0	0.124	0	0
ITM2C	0.106	0	0	0	0.181	0	0	0.115	0.215	0	0	0	0	0.113	0	0
MRX52	0	0	0.295	0	0	0.136	0.164	0	0	0	0	0	0.135	0	0	0
WHCR	0	0	0.136	0	0	0.114	0.115	0	0	0	0.127	0	0.123	0	0.113	0
PHF6	0	0	0.148	0	0	0.202	0.128	0.104	0	0	0	0	0.146	0	0	0
DLSTP1	0	0	0	0	0.216	0	0.126	0.12	0.266	0	0	0	0	0	0	0
MS4A6A	0	0.135	0.13	0	0.168	0	0	0.122	0.173	0	0	0	0	0	0	0
UBB	0	0	0	0	0.222	0	0	0.126	0.167	0	0.101	0	0	0.111	0	0
NECAB1	0.14	0	0.108	0	0	0.142	0	0.107	0	0.229	0	0	0	0	0	0
OPHN1	0	0	0.137	0	0	0.117	0.229	0.104	0	0	0	0	0.138	0	0	0
WAC	0.161	0	0	0	0.174	0	0	0.12	0.269	0	0	0	0	0	0	0
FBXO7	0	0	0	0	0.231	0	0	0.166	0.132	0	0	0	0	0.194	0	0
GSTO1	0	0.109	0	0	0.156	0	0	0.136	0.186	0	0.136	0	0	0	0	0
CACNA1I	0	0	0	0	0	0.301	0.114	0.162	0	0	0	0	0	0	0	0.145
SEPT7P2	0	0	0	0	0.166	0	0	0.182	0	0	0	0	0	0.154	0.22	0
PSMB5	0	0.106	0	0	0.203	0	0	0	0.166	0	0.124	0	0	0.123	0	0
SLC6A8	0	0	0.16	0	0	0.145	0.159	0.104	0	0	0	0	0.154	0	0	0
PPT2	0	0	0	0	0.222	0.103	0	0.115	0	0	0	0	0.138	0.143	0	0
DFN8	0	0	0.114	0.211	0	0	0	0	0	0.132	0	0.125	0.139	0	0	0
FOPNL	0	0	0.114	0	0	0.118	0.145	0.113	0	0	0	0	0	0	0	0.231
ARSA	0	0	0.107	0	0.235	0	0	0	0	0.167	0	0	0.106	0.106	0	0
KIF5A	0	0.177	0	0.115	0	0	0	0	0	0	0.22	0.105	0	0.104	0	0
DFNA47	0	0	0.181	0.116	0	0	0	0	0	0.177	0	0.134	0.113	0	0	0
BBS4	0	0	0.146	0	0	0	0	0	0	0.121	0	0.156	0.297	0	0	0
FA2H	0	0	0	0.269	0	0	0	0	0	0.106	0	0.227	0	0.117	0	0
KCNJ10	0	0.129	0	0	0	0.209	0.103	0.108	0	0.169	0	0	0	0	0	0

CRP	0	0	0	0	0.216	0	0	0.13	0.161	0	0	0	0	0	0	0.21
RAB40AL	0	0	0.109	0.215	0	0	0.123	0	0	0	0.136	0	0.134	0	0	0
MRXSA	0	0	0.145	0	0	0.141	0.102	0.119	0	0	0	0	0.21	0	0	0
GPHN	0	0	0.12	0	0	0.21	0.131	0.123	0	0.132	0	0	0	0	0	0
CLCN1	0	0	0	0.232	0	0.162	0	0	0	0	0	0.167	0	0	0	0.154
T	0	0.154	0	0	0	0	0	0	0	0	0	0	0	0	0.561	0
NDP	0	0	0	0	0	0.257	0	0	0	0.193	0	0.13	0.135	0	0	0
AUTS6	0.132	0	0.181	0	0	0.136	0.157	0	0	0	0	0	0.109	0	0	0
ATXN7L1	0	0	0	0	0	0	0	0	0	0	0.142	0	0	0.136	0.202	0.234
DYT15	0	0	0	0.157	0	0	0	0	0	0.145	0	0.131	0.129	0	0	0.152
KCNN1	0	0	0	0	0	0.235	0.177	0.134	0	0	0	0	0	0	0	0.168
AARS	0	0	0	0.229	0	0	0	0	0	0	0	0.176	0	0.308	0	0
FGS3	0	0	0.194	0	0	0.171	0.178	0	0	0	0	0	0.17	0	0	0
TAAR9	0	0	0.151	0	0.279	0	0	0	0	0	0	0	0.13	0	0	0.152
GABARAPL2	0	0	0	0	0	0	0	0	0.112	0	0.169	0	0	0.153	0.278	0
MRX15	0	0	0.105	0	0	0.166	0.207	0	0	0	0	0	0.234	0	0	0
ATG7	0	0	0	0	0.14	0	0	0	0.131	0	0.221	0	0	0.104	0.115	0
NLGN1	0	0	0.147	0	0	0.173	0.128	0	0	0	0	0	0.263	0	0	0
CHERP	0	0	0	0	0	0	0	0	0	0	0.126	0	0	0.131	0.205	0.247
RASD2	0	0	0.15	0	0	0	0	0	0	0	0.158	0	0	0	0.113	0.288
SST	0	0	0	0	0.142	0.147	0	0.115	0	0.165	0.14	0	0	0	0	0
IGHV4-28	0	0.123	0	0	0.156	0	0	0.138	0.184	0	0.108	0	0	0	0	0
DPYSL5	0.13	0.162	0	0	0	0	0	0	0	0.1	0	0.176	0	0.14	0	0
CHL1	0	0	0.135	0	0	0.152	0.109	0	0	0.2	0	0	0.112	0	0	0
GABRG1	0	0	0.118	0	0	0.147	0.152	0.12	0	0.171	0	0	0	0	0	0
TBL3	0	0	0	0.224	0	0	0	0	0	0	0.132	0	0	0	0.112	0.24
DRD5	0	0	0.141	0	0	0	0	0	0	0.119	0.132	0	0.111	0	0	0.205
GLULP2	0.126	0	0	0	0	0	0	0	0	0	0.175	0	0	0	0.407	0
RTN4IP1	0	0.101	0	0.101	0	0	0	0	0	0	0	0.14	0	0.366	0	0
NIPBL	0	0	0.155	0	0	0.186	0.133	0.101	0	0	0	0	0.133	0	0	0
NCLN	0.174	0	0	0	0.274	0	0	0	0.259	0	0	0	0	0	0	0
CHRNA5	0	0	0.137	0	0	0.159	0	0.101	0	0.182	0	0	0.128	0	0	0
AAT2	0	0.101	0	0.112	0	0.214	0	0	0	0.144	0	0.134	0	0	0	0
SNAP91	0	0	0.136	0	0.193	0.134	0	0.123	0	0	0.119	0	0	0	0	0
ABCA1	0.1	0	0	0	0.308	0	0	0	0.297	0	0	0	0	0	0	0
KIAA1147	0	0.11	0	0	0	0.209	0.139	0.123	0	0	0	0	0	0.124	0	0
SLC32A1	0	0	0.118	0	0	0.191	0.117	0.107	0	0.171	0	0	0	0	0	0
HSPA1B	0	0.136	0	0	0	0	0	0	0	0	0.216	0	0	0.156	0	0.196
DCLK2	0	0	0.101	0	0	0.212	0.158	0.105	0	0	0	0	0.128	0	0	0
LINGO3	0	0.45	0	0	0	0	0	0	0	0	0	0.254	0	0	0	0
PRS	0	0	0.154	0	0	0.168	0.122	0	0	0	0	0	0.259	0	0	0

ACAD8	0	0	0	0	0.217	0	0.1	0.119	0.267	0	0	0	0	0	0	0
WBSCR28	0	0	0.154	0	0	0.154	0.119	0.145	0	0	0	0	0.13	0	0	0
UNC79	0	0	0.154	0	0	0	0	0	0	0	0	0	0.13	0.147	0	0.269
CMD1F	0	0	0	0.339	0	0	0	0	0	0	0	0.215	0	0	0	0.145
PANK4	0	0	0	0	0.14	0	0	0.139	0.142	0	0.123	0	0	0.155	0	0
APBB2	0.116	0	0	0	0.185	0	0	0.145	0.116	0	0	0	0	0.137	0	0
FEOM3	0	0	0	0.267	0	0	0	0	0	0	0	0.166	0.103	0	0	0.161
PIEZO1	0.126	0	0	0	0.153	0	0	0.161	0.121	0	0	0	0	0.135	0	0
GABRR2	0	0	0	0	0	0.248	0.195	0.109	0	0.144	0	0	0	0	0	0
METTL19	0	0	0.144	0	0	0.149	0.103	0.114	0	0	0	0	0	0	0	0.186
SHANK3	0	0	0.119	0	0	0.173	0.115	0	0	0	0	0	0.165	0	0	0.123
CHRNA3	0	0	0.101	0	0	0.17	0	0.124	0	0.198	0	0	0.102	0	0	0
GLULP3	0.126	0	0	0	0	0	0	0	0	0	0.152	0	0	0	0.416	0
MAP1S	0	0	0	0.238	0	0	0	0	0.116	0	0.233	0	0	0.107	0	0
EMP2	0	0	0	0.291	0	0	0	0	0	0	0	0.403	0	0	0	0
TRNL1	0	0	0	0.278	0	0	0.152	0	0	0	0	0.158	0.105	0	0	0
ZNF673	0	0	0.125	0	0	0.184	0.102	0.112	0	0	0	0	0.17	0	0	0
MOPCB1	0	0	0.206	0.114	0	0.242	0	0	0	0	0	0	0.131	0	0	0
CNTFR	0	0.116	0	0.458	0	0	0	0	0.119	0	0	0	0	0	0	0
TM2D1	0.145	0	0	0	0.186	0	0	0.102	0.158	0	0	0	0	0.102	0	0
MRXS11	0	0	0.15	0	0	0.171	0.272	0	0	0	0	0	0.1	0	0	0
GPRIN1	0.112	0	0.366	0	0	0	0	0	0	0	0	0	0.11	0	0	0.104
MGR7	0.204	0	0	0	0	0.126	0.103	0.109	0	0	0	0	0	0	0	0.15
APP	0.107	0	0	0	0.187	0	0	0.104	0.185	0	0	0	0	0.109	0	0
C22ORF25	0	0	0.184	0	0	0.141	0.236	0	0	0	0	0	0.131	0	0	0
RNF103	0	0	0.182	0	0.236	0	0	0.119	0.155	0	0	0	0	0	0	0
MMVP1	0	0	0	0.107	0	0	0	0	0.17	0.117	0	0.138	0.159	0	0	0
GUSBP5	0	0	0.119	0	0	0.137	0.17	0.101	0	0	0	0	0.164	0	0	0
NLGN2	0	0	0.126	0	0	0.166	0.102	0.118	0	0	0	0	0.179	0	0	0
HRH3	0	0	0.118	0	0	0.19	0.121	0	0	0	0.144	0	0.118	0	0	0
LOC440786	0	0	0	0	0.193	0	0	0.165	0.146	0	0	0	0	0	0.186	0
AUNX1	0	0	0.165	0.116	0	0	0	0	0	0.128	0	0.153	0.127	0	0	0
GLC3B	0	0	0	0.494	0	0	0	0	0	0	0	0.195	0	0	0	0
DCR	0	0	0	0	0	0.216	0.26	0	0	0	0	0	0.107	0	0	0.106
AP3M2	0	0	0.145	0	0	0.168	0.262	0.114	0	0	0	0	0	0	0	0
ND6	0	0	0	0.321	0	0	0.135	0	0	0	0	0.106	0	0.126	0	0
FAM113B	0	0	0.121	0	0.193	0	0	0.116	0.126	0	0	0	0.132	0	0	0
SDF2L1	0.131	0	0	0	0.326	0	0	0.105	0.126	0	0	0	0	0	0	0
DFNB74	0	0	0.151	0	0	0	0	0	0	0.113	0	0.311	0.113	0	0	0
S100A16	0.133	0	0	0	0	0	0	0	0	0	0.158	0	0	0	0.396	0
ATP6	0	0	0	0.185	0	0	0.122	0	0	0	0.116	0.121	0	0.143	0	0

BICD2	0.144	0.133	0	0.114	0	0	0	0	0.119	0	0.177	0	0	0	0	0
OTX1	0	0	0	0	0	0.241	0	0	0	0.153	0.191	0	0	0	0.102	0
THOP1	0	0	0	0	0.203	0	0	0.199	0.154	0	0	0	0	0.13	0	0
IFNK	0.17	0.116	0	0	0.237	0	0	0	0.163	0	0	0	0	0	0	0
CPLX3	0	0	0.125	0	0	0.187	0	0	0	0.109	0	0	0.152	0	0.113	0
RASGRF1	0	0.122	0.119	0	0.208	0	0	0	0	0	0.111	0	0.126	0	0	0
B3GALTL	0	0	0	0	0	0	0	0	0	0	0	0	0.524	0	0	0.161
KCNA6	0	0	0	0	0	0.265	0.132	0.1	0	0	0	0	0	0	0	0.188
NR2E1	0	0	0.148	0	0	0.145	0	0	0	0.247	0	0	0.145	0	0	0
C10ORF11	0	0	0.168	0	0	0.166	0.107	0.128	0	0	0	0	0.116	0	0	0
CMD1H	0	0	0	0.223	0	0.164	0	0	0	0	0	0.154	0	0	0	0.142
PRSS12	0	0	0.148	0	0	0.166	0	0.124	0	0	0	0	0.245	0	0	0
DRD1	0	0	0.106	0	0	0	0	0	0	0.146	0.127	0	0.124	0	0	0.178
MRPL54	0	0	0	0.245	0	0	0.173	0	0	0	0	0.108	0	0	0	0.154
SCN3B	0	0	0	0	0	0.22	0.155	0.115	0	0	0	0	0	0	0	0.19
KIF21A	0	0	0	0.296	0	0	0	0	0	0	0	0.213	0.17	0	0	0
FECD3	0	0	0	0.189	0	0	0	0	0.107	0.194	0	0	0.189	0	0	0
CNA1	0	0	0	0.286	0	0	0	0	0	0	0	0.393	0	0	0	0
TRNV	0	0	0	0.189	0	0	0.15	0	0	0	0	0.1	0.129	0.111	0	0
CACNA1E	0	0	0	0	0	0.259	0.169	0.11	0	0	0	0	0	0	0	0.139
PDXK	0	0	0	0	0.156	0	0	0	0	0	0.11	0	0	0.129	0.282	0
BBS1	0	0	0.144	0	0	0	0	0	0	0.123	0	0.132	0.278	0	0	0
BCHE	0	0	0.108	0	0.138	0	0	0.143	0.156	0	0	0	0.132	0	0	0
HIP1	0	0	0.141	0.102	0	0	0	0	0	0	0.203	0	0	0.125	0.106	0
SCN10A	0	0	0	0.157	0	0.105	0.155	0	0	0	0	0.105	0	0	0	0.154
DLD	0	0	0	0	0.165	0	0.152	0	0	0	0.208	0	0	0.151	0	0
HTR1B	0	0	0.107	0	0	0	0	0	0	0.124	0.154	0	0	0	0	0.291
FRA10B	0.117	0	0	0	0	0	0	0	0	0	0.125	0	0	0	0.434	0
CCT4	0	0	0	0.503	0	0	0	0	0	0	0	0.173	0	0	0	0
BRI3	0	0	0	0	0.231	0	0	0.152	0.146	0	0	0	0	0.146	0	0
IL1RAPL1	0	0	0.111	0	0	0.134	0.138	0.14	0	0	0	0	0.152	0	0	0
DOK7	0	0	0	0.42	0	0	0	0	0	0	0	0	0.131	0	0	0.124
GOUT1	0	0	0	0.308	0	0	0	0	0.12	0	0	0.104	0	0	0	0.142
MCTP1	0.124	0	0	0	0	0	0	0	0	0	0	0	0	0	0.55	0
DERL1	0	0.172	0	0	0.203	0	0	0	0.134	0	0	0	0	0.165	0	0
DFNA49	0	0	0	0.358	0	0	0	0	0	0.154	0	0.162	0	0	0	0
MMEL1	0	0	0	0	0.314	0	0	0.242	0.118	0	0	0	0	0	0	0
DMPK	0	0	0.118	0.113	0	0	0	0	0	0	0.214	0	0.126	0	0.103	0
MRPL43	0	0	0	0.222	0	0	0.152	0	0	0	0	0.118	0	0.181	0	0
ZBTB20	0	0	0.138	0	0	0.199	0	0.101	0	0.129	0	0	0.106	0	0	0
MSI1	0	0	0	0	0.243	0	0	0.113	0.139	0.178	0	0	0	0	0	0

ATG12P1	0	0	0	0	0	0	0	0	0.122	0	0.133	0	0	0.139	0.278	0
FRG2B	0	0	0	0.236	0	0	0	0	0	0	0.334	0	0	0	0.102	0
CXORF40B	0	0	0.132	0	0	0.197	0.115	0.102	0	0	0	0	0.126	0	0	0
SHC3	0	0.139	0	0	0.177	0	0	0	0.158	0	0.198	0	0	0	0	0
TMC1	0	0	0.121	0	0	0.165	0	0	0	0.109	0	0.124	0.152	0	0	0
VWS2	0.141	0	0.383	0	0	0	0	0	0	0	0	0	0.147	0	0	0
ZDHHC13	0	0	0	0	0.178	0	0	0	0	0	0.119	0	0	0.15	0.224	0
TRIM31	0	0	0.127	0	0	0	0	0	0	0	0.14	0	0	0	0.403	0
GPR56	0	0	0.113	0	0	0.178	0.129	0.118	0	0	0	0	0.132	0	0	0
CBS	0.101	0	0.126	0	0.133	0	0	0	0	0	0.101	0	0.107	0	0.102	0
STMN2	0	0.107	0	0	0	0	0	0	0.206	0.236	0	0	0	0.121	0	0
CACNA1G	0	0	0	0	0	0.259	0.131	0.103	0	0	0	0	0	0	0	0.176
AMCN	0	0	0	0.25	0	0	0	0	0	0	0	0.419	0	0	0	0
KARS	0	0.104	0	0.437	0	0	0	0	0.128	0	0	0	0	0	0	0
GBE1	0	0	0	0.189	0	0	0.12	0	0	0	0	0.108	0.151	0.1	0	0
SPTLC1	0	0	0	0.182	0	0	0	0	0	0	0	0.292	0	0.194	0	0
MARVELD2	0	0	0	0.222	0	0	0	0	0	0	0.177	0.167	0	0	0.101	0
MECP2	0	0	0.106	0	0	0.188	0.121	0.111	0	0	0	0	0.141	0	0	0
C20ORF7	0	0	0	0.117	0	0	0.12	0	0	0	0	0.221	0.109	0.1	0	0
PTCHD1	0	0	0.16	0	0	0.145	0.102	0.143	0	0	0	0	0.117	0	0	0
OSTM1	0	0	0	0	0	0.235	0	0.131	0.183	0	0	0	0	0.117	0	0
AGTPBP1	0	0	0	0	0	0.151	0	0	0	0.142	0	0	0	0.22	0	0.152
KCNC2	0	0	0	0	0	0.276	0.146	0.133	0	0	0	0	0	0	0	0.11
TUBA1B	0	0	0	0	0.197	0	0	0.146	0.148	0	0	0	0	0.174	0	0
MRX23	0	0	0.194	0	0	0.157	0.102	0.101	0	0	0	0	0.111	0	0	0
PSMD4	0	0.106	0	0	0.133	0	0	0	0.175	0	0.116	0	0	0.134	0	0
C22DDELS	0	0	0.172	0	0	0.137	0.233	0	0	0	0	0	0.122	0	0	0
PPIF	0.163	0	0	0	0	0	0	0	0	0	0.197	0	0	0.101	0.202	0
LOC728373	0	0	0	0	0	0	0	0	0	0	0.167	0	0	0	0.496	0
ARVD6	0	0	0	0	0.306	0	0	0	0.108	0	0.249	0	0	0	0	0
PHKA1P1	0.167	0	0	0.174	0	0	0.101	0	0	0	0	0	0	0	0	0.221
MKKS	0	0	0.147	0	0	0	0	0	0	0	0	0.15	0.22	0	0	0.145
MCDR1	0	0	0	0.144	0	0	0	0	0	0.219	0.185	0	0	0	0.114	0
SLC30A4	0	0	0.151	0	0.277	0	0	0	0	0	0	0	0.105	0.129	0	0
ND4	0	0	0	0.239	0	0	0	0	0	0	0	0.271	0	0.152	0	0
LOC285299	0	0	0	0.368	0	0	0	0	0	0	0.191	0	0	0	0.103	0
GPM6B	0	0	0	0.164	0	0.219	0.151	0	0	0	0	0.127	0	0	0	0
ERVK-22	0	0.104	0	0	0.138	0	0.109	0.121	0.189	0	0	0	0	0	0	0
STBMS1	0	0	0	0.223	0	0	0	0	0	0.152	0	0.16	0.125	0	0	0
YARS	0	0	0	0.34	0	0	0	0	0	0	0.202	0.118	0	0	0	0
FRG2	0	0	0	0.368	0	0	0	0	0	0	0.179	0	0	0	0.113	0

C1QB	0	0	0	0	0.164	0	0	0.198	0.191	0	0	0	0	0.106	0	0
NOMO1	0	0	0	0	0.31	0	0	0	0.11	0	0	0	0	0	0	0.237
PPHLN1	0	0	0	0	0.153	0	0	0	0.163	0	0	0	0	0.12	0.221	0
FRA10AC1	0.126	0	0	0	0	0	0	0	0	0	0.102	0	0	0	0.429	0
HM13	0.21	0	0	0	0.334	0	0	0	0.113	0	0	0	0	0	0	0
ORMDL1	0.104	0	0	0	0.162	0	0	0.104	0.152	0	0	0	0	0.134	0	0
PNMA5	0	0	0	0	0	0.255	0.143	0.111	0	0	0	0	0	0.147	0	0
MCDR4	0	0	0	0.267	0	0	0	0	0	0.1	0	0.106	0.183	0	0	0
DLEU2	0	0.101	0	0	0.254	0	0	0.104	0.197	0	0	0	0	0	0	0
CCL3L1	0	0	0	0	0.206	0	0	0.238	0.212	0	0	0	0	0	0	0
SCO2	0	0.128	0	0.115	0	0	0.147	0	0	0	0	0.14	0	0.125	0	0
POMT2	0	0	0	0.283	0	0	0	0	0	0.22	0	0	0.152	0	0	0
SELM	0	0	0	0	0.211	0	0	0.147	0.177	0	0	0	0	0.12	0	0
SLC6A4	0.121	0	0	0	0.183	0	0	0	0.208	0	0	0	0	0	0	0.142
DFNA30	0	0	0.123	0.116	0	0	0	0	0	0.192	0	0.107	0.116	0	0	0
MRX39	0.124	0	0.127	0	0	0.136	0.138	0	0	0	0	0	0.129	0	0	0
NKAIN2	0	0	0	0	0	0.218	0.154	0.1	0	0	0	0	0	0	0	0.182
ELP3	0	0.104	0	0	0	0	0	0	0.116	0.125	0.164	0	0	0.144	0	0
DFNA42	0	0	0.113	0.207	0	0	0	0	0	0.118	0	0.11	0.105	0	0	0
MIR433	0	0	0	0	0.227	0	0	0.141	0.185	0	0	0	0	0.1	0	0
NOVA2	0	0	0.12	0	0	0.174	0.102	0.15	0	0	0	0	0.107	0	0	0
LOC619480	0	0	0	0.245	0	0	0	0	0	0.17	0.125	0	0	0	0.112	0
PHACTR1	0	0	0	0	0	0	0	0.114	0	0	0.135	0	0	0.114	0.288	0
GLS	0	0	0	0	0	0	0.116	0	0	0	0.212	0	0	0.208	0.115	0
STXBP1	0	0	0	0	0	0.237	0.134	0.13	0	0	0	0	0	0.15	0	0
FXN	0	0	0	0.117	0	0	0	0	0	0	0.173	0.135	0	0.105	0.12	0
DUX4	0	0	0	0.4	0	0	0	0	0	0	0.138	0	0	0	0.112	0
NDUFV1	0	0	0	0	0	0.256	0.123	0.123	0	0	0	0	0	0.148	0	0
ST6GALNAC5	0	0	0	0	0	0	0	0	0	0	0.139	0	0	0.125	0.204	0.181
OA24	0	0	0	0.261	0	0	0	0	0	0.17	0	0.104	0	0	0.114	0
ATAD1	0	0	0.1	0	0	0.174	0.26	0	0	0	0	0	0.115	0	0	0
SCN4B	0	0	0	0	0	0.219	0.158	0.142	0	0	0	0	0	0	0	0.129
ATG4A	0	0	0	0	0	0	0	0	0.141	0	0.386	0	0	0.121	0	0
SCYL1	0	0.114	0	0.1	0	0	0	0	0.137	0.189	0	0	0	0.108	0	0
MRX20	0	0	0.108	0.102	0	0.195	0	0	0	0	0	0	0.243	0	0	0
NF2	0	0.455	0	0	0	0.193	0	0	0	0	0	0	0	0	0	0
LOC100272228	0	0	0.141	0	0	0.122	0.165	0.116	0	0	0	0	0.103	0	0	0
MAGEA9B	0	0	0.121	0	0	0.17	0.146	0.102	0	0	0	0	0.108	0	0	0
MMDK	0	0	0	0.503	0	0	0	0	0	0.144	0	0	0	0	0	0
SLC4A11	0	0	0	0.248	0	0	0	0	0	0.172	0	0.123	0.103	0	0	0
AFG3L1P	0	0	0	0.248	0	0	0.179	0	0	0	0	0.11	0	0.108	0	0

SVIP	0	0.115	0	0	0.176	0	0	0	0.124	0	0.123	0	0	0.107	0	0
IARS2	0	0	0	0.271	0	0	0.11	0	0	0	0	0.138	0	0.124	0	0
FGS5	0	0	0.154	0	0	0.166	0.157	0	0	0	0	0	0.166	0	0	0
MRX73	0	0	0.176	0	0	0.164	0.186	0	0	0	0	0	0.117	0	0	0
NYS3	0	0	0	0.189	0	0.105	0	0	0	0	0	0.126	0	0	0	0.222
PDCD10	0	0	0	0	0	0.218	0.105	0.137	0	0	0	0	0	0	0	0.182
DFNB65	0	0	0.115	0.116	0	0	0	0	0	0.16	0	0.138	0.113	0	0	0
NLGN3	0	0	0.148	0	0	0.144	0.102	0.118	0	0	0	0	0.13	0	0	0
LEFTY1	0	0.147	0	0	0	0	0	0	0	0	0	0	0	0	0.189	0.305
TMEM70	0	0	0	0.111	0	0	0.114	0	0	0	0	0.123	0.167	0	0	0.123
OPEM	0	0	0	0.35	0	0	0	0	0	0	0	0.169	0	0	0	0.119
LOC653541	0	0	0	0.368	0	0	0	0	0	0	0.168	0	0	0	0.102	0
RARS2	0	0	0	0	0	0.242	0.137	0	0	0	0	0.133	0	0.126	0	0
LSG1	0	0	0.125	0	0	0	0	0.155	0	0.109	0.125	0	0	0.124	0	0
ALDH7A1	0	0	0	0	0	0.125	0.107	0.126	0	0	0.142	0	0.138	0	0	0
NDUFA1	0	0	0	0	0	0	0.144	0	0	0	0	0.136	0	0.355	0	0
MRXSAB	0	0	0.149	0	0	0.174	0.12	0	0	0	0	0	0.192	0	0	0
SCN1B	0	0	0	0	0	0.221	0.175	0.1	0	0	0	0	0	0	0	0.138
CXORF36	0	0	0.105	0	0	0.179	0.13	0.106	0	0	0	0	0.114	0	0	0
SUN3	0	0.189	0	0	0.148	0	0	0	0.112	0.185	0	0	0	0	0	0
BIN1	0	0	0	0	0	0	0	0	0	0	0.282	0	0	0	0.351	0
SLC8A2	0	0	0	0	0	0.242	0.105	0.131	0	0	0	0	0	0.155	0	0
TMEM59	0	0	0	0	0.197	0	0	0.125	0.205	0	0	0	0	0.105	0	0
MOCS1	0	0	0	0	0	0.254	0.132	0	0	0	0	0.102	0.144	0	0	0
AMCX5	0	0	0	0.239	0	0	0.169	0	0	0	0	0	0.224	0	0	0
KIF1A	0	0.39	0	0.117	0	0	0	0	0	0	0	0.125	0	0	0	0
KAT5	0.159	0	0	0	0.174	0	0	0	0	0	0.191	0	0	0.107	0	0
MRX5	0	0	0.165	0	0	0.167	0.188	0	0	0	0	0	0.11	0	0	0
EIF2B5	0.122	0.109	0	0.104	0	0	0.146	0	0	0	0	0	0.149	0	0	0
EGR3	0	0	0	0.392	0	0.117	0	0	0	0	0	0.121	0	0	0	0
MPPED1	0	0	0.112	0	0	0.143	0.273	0.102	0	0	0	0	0	0	0	0
LCA9	0	0	0	0.172	0	0	0	0	0	0.17	0	0.1	0	0	0	0.187
TRESMCR	0	0	0	0.181	0	0	0.138	0	0	0	0	0.202	0.108	0	0	0
CNP	0	0.13	0	0.104	0	0	0	0	0	0.15	0	0.245	0	0	0	0
DUX4L14	0	0	0	0.349	0	0	0	0	0	0	0.166	0	0	0	0.112	0
ERVW-6	0	0	0	0	0.149	0	0	0.352	0.126	0	0	0	0	0	0	0
DUX4L2	0	0	0	0.261	0	0	0	0	0	0	0.252	0	0	0	0.113	0
OPA4	0	0	0	0.321	0	0	0	0	0	0.133	0	0.172	0	0	0	0
SCN11A	0	0	0	0	0	0.151	0.141	0	0	0	0	0.132	0	0	0	0.201
SLC30A1	0	0	0.107	0	0	0	0	0.276	0	0	0	0	0.12	0.121	0	0
MRX40	0	0	0.127	0	0	0.163	0.211	0	0	0	0	0	0.123	0	0	0

CAPN5	0	0.156	0	0	0	0	0	0	0	0	0.131	0	0	0.135	0.201	0
RABGAP1L	0	0	0	0	0.152	0	0	0.147	0.17	0	0	0	0	0.154	0	0
GULP1	0.148	0	0	0	0.182	0	0	0	0.159	0	0	0	0	0.134	0	0
SLC8A3	0	0	0	0	0	0.239	0.115	0.125	0	0	0	0	0	0.144	0	0
ATF4P4	0	0	0	0.184	0	0.12	0	0.116	0	0	0	0.203	0	0	0	0
GABRG3	0	0	0.146	0	0	0.161	0.204	0.112	0	0	0	0	0	0	0	0
LOC440792	0	0	0.102	0	0	0.167	0.105	0	0	0	0	0	0.103	0	0	0.145
MRX4	0	0	0.113	0	0	0.147	0.211	0	0	0	0	0	0.151	0	0	0
EEGV1	0	0	0	0	0	0.153	0.18	0.115	0	0	0	0	0	0	0	0.173
DNAH12	0	0.101	0	0	0	0	0	0	0	0	0.121	0	0	0	0.399	0
NPC2	0	0	0	0	0.235	0	0	0	0.242	0	0	0	0	0.144	0	0
MRX37	0	0	0.114	0	0	0.168	0.211	0	0	0	0	0	0.128	0	0	0
ETM2	0	0	0	0.157	0	0	0	0	0.118	0	0.24	0.106	0	0	0	0
SBDSP1	0	0	0	0.412	0	0	0	0	0	0	0	0	0	0	0	0.208
HOXB2	0	0.11	0	0.101	0	0	0	0	0	0	0.297	0	0	0	0.112	0
SEPT3	0	0	0	0	0.16	0	0	0.147	0.111	0	0	0	0	0.202	0	0
EMP1	0	0	0	0.217	0	0	0	0	0	0	0	0.403	0	0	0	0
EXT3	0	0	0	0.23	0	0	0	0	0	0	0	0.39	0	0	0	0
GSTO2	0	0.119	0	0	0.253	0	0	0	0.103	0	0.145	0	0	0	0	0
LDHB	0	0	0	0	0.215	0	0.149	0.12	0.136	0	0	0	0	0	0	0
LOC280665	0	0	0.106	0	0	0.167	0.234	0.112	0	0	0	0	0	0	0	0
SMCR7	0.159	0	0.112	0	0	0	0.131	0	0	0	0	0	0.103	0	0.113	0
HTR1A	0	0	0.151	0	0.29	0	0	0	0	0.177	0	0	0	0	0	0
MNG3	0	0	0	0.303	0	0	0	0	0	0	0	0.204	0.11	0	0	0
MRX77	0	0	0.114	0	0	0.138	0.242	0	0	0	0	0	0.123	0	0	0
WTS	0	0	0.144	0	0	0.168	0.17	0	0	0	0	0	0.135	0	0	0
TFCP2	0.139	0	0	0	0.174	0	0	0.109	0.195	0	0	0	0	0	0	0
NCS1	0	0	0.124	0	0	0.173	0	0	0	0.2	0	0	0.119	0	0	0
MRX3	0	0	0.131	0	0	0.171	0.102	0.101	0	0	0	0	0.111	0	0	0
ARVD7	0	0	0	0.269	0	0	0.17	0	0	0	0	0	0	0	0	0.176
PSMC4	0	0	0	0	0.163	0	0	0.147	0.111	0	0	0	0	0.194	0	0
NYS4	0	0	0	0.106	0	0	0	0	0	0.115	0	0.121	0.124	0	0	0.148
HPRT1	0	0	0	0	0	0	0	0	0	0	0.156	0	0	0	0.458	0
RRS1	0	0	0	0	0.166	0	0	0	0	0	0.107	0	0	0.114	0.226	0
ITPRIP	0	0	0	0	0	0	0	0	0	0	0.225	0	0	0.19	0.198	0
ACHE	0	0	0.108	0	0.127	0	0	0.136	0.135	0	0	0	0.107	0	0	0
MTMR2	0	0.141	0	0.177	0	0	0	0	0	0	0	0.295	0	0	0	0
ZDHHC23	0	0	0	0.304	0	0	0	0	0	0	0	0.124	0	0	0	0.184
AMIGO3	0	0.188	0	0	0.121	0	0	0.154	0	0	0	0	0	0.149	0	0
VAC14	0	0.208	0	0.114	0	0	0	0	0.139	0	0	0	0	0.15	0	0
KCTD8	0	0	0	0	0	0.152	0.232	0.11	0	0	0	0.117	0	0	0	0

NRG1	0	0	0.147	0	0	0	0	0	0.137	0.163	0	0	0.163	0	0	0
SYT11	0	0	0	0	0	0	0	0	0	0	0	0	0	0.129	0.215	0.266
DFNA24	0.125	0	0.113	0	0	0	0	0	0	0.131	0	0.122	0.119	0	0	0
DFNB71	0	0	0.151	0	0	0	0	0	0	0.155	0	0.176	0.128	0	0	0
PREP	0	0	0	0	0	0	0	0.441	0	0.169	0	0	0	0	0	0
PHKG1	0.29	0	0	0	0.194	0	0	0	0.124	0	0	0	0	0	0	0
MYP4	0	0.126	0	0	0.163	0	0	0.147	0.171	0	0	0	0	0	0	0
SCZD8	0	0	0.107	0	0	0	0	0	0	0.13	0	0	0.108	0	0	0.262
SH3GL3	0	0	0	0	0	0	0	0	0	0	0.185	0	0	0.216	0.206	0
LGMBF	0	0	0	0.269	0	0	0	0	0	0.112	0.122	0	0	0	0.104	0
PTPLA	0	0	0.113	0	0.139	0	0	0.101	0.126	0	0	0	0.128	0	0	0
NETO1	0	0	0.108	0	0	0.111	0.152	0.126	0	0	0.11	0	0	0	0	0
PKHD1L1	0	0	0	0	0	0	0	0	0	0	0	0	0	0	0.392	0.214
PDB4	0	0.135	0	0	0	0	0	0	0.116	0.245	0	0.11	0	0	0	0
MRXS7	0	0	0.118	0	0	0.162	0.127	0	0	0	0	0	0.198	0	0	0
DYX3	0	0	0.194	0.103	0	0.194	0	0	0	0	0	0	0.114	0	0	0
ZDHHC15	0	0	0.15	0	0	0.144	0.124	0	0	0	0	0	0.186	0	0	0
OPALIN	0	0.194	0	0.165	0	0	0	0	0	0	0	0.245	0	0	0	0
LVNC2	0	0	0	0.244	0	0.121	0	0	0	0	0	0.113	0	0	0	0.125
HEXB	0	0	0	0	0.285	0	0	0	0.169	0	0	0	0	0.149	0	0
MRX2	0	0	0.13	0	0	0.146	0.135	0	0	0	0	0	0.192	0	0	0
OPA2	0	0	0	0.249	0	0	0	0	0	0.103	0	0.25	0	0	0	0
MSTO2P	0	0	0	0.417	0	0	0	0	0	0	0	0	0	0	0.184	0
AMBRA1	0	0	0	0	0.153	0	0	0	0.12	0	0.204	0	0	0.124	0	0
AMIGO1	0	0.151	0	0	0.151	0	0	0.145	0	0	0	0	0	0.154	0	0
TCN1	0.133	0	0	0	0.174	0	0	0.123	0.171	0	0	0	0	0	0	0
RILP	0	0	0	0.231	0	0	0	0	0	0	0.163	0	0	0.101	0.105	0
C12ORF65	0	0	0	0	0	0	0.106	0	0	0	0	0.175	0	0.319	0	0
C3ORF39	0	0	0	0	0	0	0	0	0	0	0	0	0	0	0.267	0.332
CACNG5	0	0.122	0	0	0	0.119	0.155	0	0	0	0	0	0	0	0	0.202
MTR	0.112	0	0	0	0.158	0	0	0	0	0	0.328	0	0	0	0	0
FAM184B	0.111	0	0	0	0	0	0	0	0	0	0	0	0	0	0.271	0.216
LOC100131434	0	0	0.106	0	0	0.143	0.109	0.111	0	0	0	0	0.129	0	0	0
BCS1L	0	0	0	0	0	0	0.174	0	0	0	0	0.199	0.1	0.124	0	0
ABHD14A	0	0	0	0	0	0.239	0	0.102	0	0.135	0	0	0	0.12	0	0
GARS	0	0.209	0	0.104	0	0	0	0	0	0.127	0	0.156	0	0	0	0
SPG23	0	0.153	0	0.112	0	0	0.209	0	0	0	0	0.122	0	0	0	0
VCX3B	0	0	0.107	0	0	0.146	0	0.14	0	0	0	0	0.202	0	0	0
MRX27	0	0	0.174	0	0	0.142	0.135	0	0	0	0	0	0.144	0	0	0
MRX35	0	0	0.106	0	0	0.169	0.119	0	0	0	0	0	0.201	0	0	0
TECPR1	0	0	0	0	0	0	0	0	0.11	0	0.275	0	0	0.209	0	0

C12ORF4	0	0	0	0.304	0	0	0	0	0	0	0	0.29	0	0	0	0
PSPN	0	0.121	0	0	0	0	0	0	0	0.122	0.149	0	0	0.201	0	0
MRX49	0	0	0.115	0	0	0.147	0.216	0	0	0	0	0	0.115	0	0	0
MRX78	0	0	0.106	0	0	0.147	0.236	0	0	0	0	0	0.104	0	0	0
ICAM5	0	0.168	0	0	0	0.211	0.104	0.109	0	0	0	0	0	0	0	0
ACBD5	0	0	0	0.253	0	0	0	0	0	0	0	0.13	0	0	0	0.208
HPX-2	0	0	0	0	0	0	0	0	0	0	0.154	0	0	0	0.437	0
C4ORF48	0	0	0.103	0	0	0.118	0.125	0.127	0	0	0	0	0.118	0	0	0
PHKG1P3	0.16	0	0	0.167	0	0	0.143	0	0	0	0	0	0	0	0	0.119
CDK5R2	0.127	0	0	0	0	0	0	0	0.145	0	0.215	0	0	0.102	0	0
BAG2	0.125	0	0	0	0.174	0	0	0	0.114	0	0	0	0	0.176	0	0
PPP1R3F	0	0	0.124	0	0	0.163	0.115	0	0	0	0	0	0.187	0	0	0
CNTNAP3	0	0	0	0.276	0	0	0	0	0	0	0	0.178	0	0.134	0	0
MHB	0	0	0	0.28	0	0	0	0	0	0	0	0.308	0	0	0	0
HSPA12A	0.17	0	0.12	0	0.177	0	0	0	0	0	0	0	0.12	0	0	0
CACNA1F	0	0	0	0	0	0.132	0	0	0	0.197	0	0	0	0	0	0.257
FAM127A	0	0	0	0.368	0	0	0	0	0	0	0.116	0	0	0	0.102	0
DFNB89	0	0	0.151	0	0	0	0	0	0	0.169	0	0.121	0.145	0	0	0
RDH14	0	0.102	0	0	0.114	0	0	0	0.158	0.212	0	0	0	0	0	0
TIA1	0	0.114	0	0	0	0	0	0	0.16	0	0.209	0	0	0	0.102	0
SLC17A8	0	0.143	0	0	0	0.212	0	0	0	0.118	0	0.112	0	0	0	0
SCNM1	0	0	0	0	0	0.155	0.17	0	0	0	0	0.138	0	0	0	0.121
VPS13B	0	0	0.147	0	0	0.134	0.129	0	0	0	0	0	0.174	0	0	0
VCF	0	0	0.145	0	0	0.174	0.124	0	0	0	0	0	0.141	0	0	0
IQCJ-SCHIP1	0	0	0	0.248	0	0	0	0	0	0	0	0.124	0	0	0	0.211
MRX8	0	0	0.12	0	0	0.134	0.222	0	0	0	0	0	0.107	0	0	0
FAM127B	0	0	0	0.368	0	0	0	0	0	0	0.111	0	0	0	0.103	0
APOC1	0.123	0	0	0	0.308	0	0	0	0.151	0	0	0	0	0	0	0
GLDC	0	0	0.106	0	0	0.116	0.115	0.105	0	0	0	0	0.139	0	0	0
HSPA4	0	0.136	0	0	0	0	0	0	0	0	0.135	0	0	0.113	0	0.196
PSMD7	0	0	0	0	0.168	0	0	0	0.131	0	0.155	0	0	0.126	0	0
HOMER2	0	0	0.155	0	0	0	0	0	0	0.169	0.114	0	0.141	0	0	0
CHRM2	0	0	0.131	0	0.307	0	0	0	0	0	0	0	0.141	0	0	0
EBM	0	0	0	0.287	0	0	0	0	0	0.165	0	0.127	0	0	0	0
SCLT1	0	0	0	0	0	0.151	0.111	0.134	0	0	0	0	0	0	0	0.181
PDE10A	0	0	0.115	0	0	0	0	0	0	0.134	0.122	0	0.101	0	0.105	0
MRX82	0	0	0.113	0	0	0.136	0.225	0	0	0	0	0	0.103	0	0	0
PTOS2	0	0	0	0.262	0	0.135	0	0	0	0	0	0	0.179	0	0	0
CXORF1	0.122	0	0.181	0	0	0.141	0	0.132	0	0	0	0	0	0	0	0
MTCH1	0.138	0	0	0	0.162	0	0	0	0.161	0	0	0	0	0.114	0	0
SMN2	0	0.145	0	0	0.26	0	0	0	0.17	0	0	0	0	0	0	0

SIAH1	0	0	0	0	0.206	0	0	0	0	0	0	0	0	0.151	0.218	0
CACNG8	0	0.106	0	0	0	0.209	0.101	0	0	0	0	0	0	0	0	0.158
NKAIN3	0	0	0	0	0	0.155	0.147	0.119	0	0	0	0	0	0	0	0.153
SLC6A5	0	0.124	0	0	0	0.212	0.129	0.109	0	0	0	0	0	0	0	0
PRICKLE2	0	0.138	0	0	0	0.151	0	0.117	0	0	0	0	0	0	0	0.167
MRX81	0	0	0.113	0	0	0.171	0.168	0	0	0	0	0	0.121	0	0	0
SBF2	0	0.134	0	0.165	0	0	0	0	0	0	0	0.274	0	0	0	0
CACNG7	0	0.11	0	0	0	0.209	0.11	0	0	0	0	0	0	0	0	0.144
MOXD1	0	0	0	0	0.248	0	0	0.177	0	0	0	0	0	0.148	0	0
GORAB	0	0.113	0	0	0	0	0	0	0	0	0.151	0	0	0.11	0.198	0
NPR2	0	0	0	0	0	0	0	0	0	0	0	0	0	0	0	0.572
ANO5	0	0	0	0.33	0	0	0	0	0	0	0	0.113	0	0	0	0.128
LGSN	0	0	0	0.407	0	0	0	0	0	0	0	0.164	0	0	0	0
EFHA1	0	0	0.124	0	0	0	0	0	0	0	0	0	0.145	0	0	0.301
FPH1	0	0	0	0	0	0.214	0	0.104	0	0	0	0.129	0	0	0	0.123
CALM1P1	0	0	0	0	0	0	0	0	0	0	0.175	0	0	0	0.395	0
FER1L4	0	0	0	0.19	0	0	0	0	0	0	0	0.38	0	0	0	0
EFHB	0.148	0	0	0	0	0.191	0	0	0	0	0	0	0	0	0	0.231
WBS2	0	0	0.15	0	0	0.147	0.126	0	0	0	0	0	0.147	0	0	0
TRNAL2	0	0	0	0.155	0	0	0.114	0	0	0	0.167	0.133	0	0	0	0
ARHGEF9	0	0	0.116	0	0	0.16	0.178	0.115	0	0	0	0	0	0	0	0
PHKG1P1	0.144	0	0	0.161	0	0	0.157	0	0	0	0	0	0	0	0	0.107
TCO	0	0	0	0.307	0	0	0	0	0.131	0	0	0.131	0	0	0	0
TAAR1	0	0	0	0	0	0	0	0	0	0	0.186	0	0	0.118	0	0.264
TMED10	0.165	0	0	0	0.174	0	0	0	0.123	0	0	0	0	0.106	0	0
DMRTB1	0.104	0	0	0	0.309	0	0	0	0.155	0	0	0	0	0	0	0
MRX64	0	0	0.119	0	0	0.143	0.102	0.1	0	0	0	0	0.103	0	0	0
ATF4P3	0	0	0	0.182	0	0.115	0	0.124	0	0	0	0.146	0	0	0	0
HSD11B1L	0	0	0	0	0.204	0	0	0.13	0.233	0	0	0	0	0	0	0
IFNA21	0	0.176	0	0	0.2	0	0	0	0.191	0	0	0	0	0	0	0
PICK1	0	0.119	0	0	0	0.156	0.109	0	0	0	0	0	0	0	0	0.182
ADAMTS17	0	0.137	0	0	0.261	0	0	0	0	0	0	0	0.168	0	0	0
PHKG1P2	0.151	0	0	0.173	0	0	0.118	0	0	0	0	0	0	0	0	0.124
C15ORF2	0	0	0.105	0	0	0.108	0.12	0.107	0	0	0	0	0.126	0	0	0
HSF1	0	0.136	0	0	0	0	0	0	0	0	0.119	0	0	0.114	0	0.196
IMMT	0	0	0.15	0	0	0	0.133	0	0	0	0	0	0.151	0.13	0	0
TARBP2P	0	0	0	0	0.228	0	0	0.167	0.169	0	0	0	0	0	0	0
RFH1	0	0	0	0.235	0	0	0	0	0	0	0	0.117	0	0	0	0.212
SRP68P1	0	0	0	0.117	0	0	0.195	0	0	0	0	0.104	0.147	0	0	0
MED12L	0	0	0	0	0	0	0	0	0	0.134	0	0.151	0.277	0	0	0
SLC16A2	0	0	0.108	0	0	0	0.132	0	0	0	0	0	0.117	0	0	0.205

PRX	0	0.136	0	0.115	0	0	0	0	0	0	0	0.106	0	0	0	0.205
DLG4	0	0	0	0.205	0	0	0	0	0	0	0.132	0	0	0	0	0.225
FSD1L	0	0	0	0	0.127	0	0	0.287	0.148	0	0	0	0	0	0	0
DENND4B	0	0	0	0.278	0	0	0	0	0	0	0.17	0	0	0	0.113	0
ZFP90	0	0.134	0	0.112	0	0	0	0	0	0	0.155	0.16	0	0	0	0
YARS2	0	0	0	0	0	0	0.152	0	0	0	0	0.288	0	0	0	0.121
DFNB27	0	0	0.132	0.105	0	0	0	0	0	0.105	0	0.106	0.113	0	0	0
BBS10	0	0	0.146	0	0	0	0	0	0	0.111	0	0.12	0.184	0	0	0
MIR124-1	0	0	0	0	0.244	0	0	0	0.317	0	0	0	0	0	0	0
IGAN	0	0.111	0	0	0.205	0	0	0	0.126	0	0	0.118	0	0	0	0
EDNRA	0	0	0	0	0	0	0	0	0	0	0	0	0	0	0	0.559
MFF	0	0	0	0.409	0	0	0	0	0	0	0	0	0	0.149	0	0
FAM127C	0	0	0	0.267	0	0	0	0	0	0	0.177	0	0	0	0.113	0
DNM1L	0.155	0	0	0.107	0	0	0	0	0	0	0.149	0	0	0.146	0	0
FAM124B	0	0.216	0.2	0	0	0	0	0	0	0.141	0	0	0	0	0	0
HSPA1A	0	0	0	0	0	0	0	0	0	0	0.184	0	0	0.131	0	0.242
YME1L1	0	0	0	0.206	0	0	0.111	0	0	0	0	0.113	0	0.127	0	0
LITAF	0	0	0	0.366	0	0	0	0	0	0	0	0.191	0	0	0	0
USH1A	0	0	0.148	0	0	0	0	0	0	0.138	0	0.132	0.138	0	0	0
DFNA58	0	0	0	0.219	0	0	0	0	0	0.217	0	0.12	0	0	0	0
SNX15	0	0.159	0	0.158	0	0	0	0	0	0	0	0.239	0	0	0	0
NNO1	0	0	0	0.288	0	0	0	0	0	0.109	0	0.159	0	0	0	0
ND3	0	0	0	0	0.129	0	0.149	0	0	0	0	0.121	0	0.156	0	0
PRPF40A	0	0	0	0	0	0	0	0	0	0	0.374	0	0	0	0.18	0
CX3CR1	0	0.229	0	0	0.201	0	0	0	0	0	0	0	0	0.124	0	0
UNC13C	0	0	0.145	0	0	0.17	0.112	0	0	0	0	0	0.127	0	0	0
BBS2	0	0	0.107	0	0	0	0	0	0	0	0	0.155	0.146	0	0	0.145
EJM3	0	0	0	0	0	0.109	0.101	0.117	0	0	0	0	0	0	0	0.226
MIR29B1	0	0	0	0	0.225	0	0	0.109	0.111	0	0	0	0	0.108	0	0
ATP6V0A1	0	0	0	0	0.336	0	0	0	0.104	0	0	0	0	0.113	0	0
TUBA1A	0	0.129	0	0	0	0	0	0	0.156	0.146	0	0	0	0.121	0	0
FTSJ1	0	0	0.12	0	0	0.138	0.132	0	0	0	0	0	0.162	0	0	0
CCL4	0	0	0	0	0.157	0	0	0.245	0.15	0	0	0	0	0	0	0
CYP2D6	0	0	0	0	0.198	0	0	0	0.114	0	0	0	0	0	0	0.239
MRX24	0	0	0.105	0	0	0.137	0.137	0	0	0	0	0	0.172	0	0	0
HTR2A	0	0	0	0	0.414	0	0	0	0	0	0	0	0	0	0	0.136
SYT4	0	0	0.149	0	0	0.158	0	0	0	0	0	0	0.13	0	0.113	0
OTSC4	0	0	0.154	0.102	0	0	0	0	0	0	0	0.101	0.193	0	0	0
SIPA1L1	0	0	0.13	0	0.307	0	0	0	0	0	0	0	0.113	0	0	0
CMT2G	0	0.115	0	0.112	0	0	0	0	0	0.169	0	0.154	0	0	0	0
SERPINA3	0.126	0	0	0	0.13	0	0	0.1	0.194	0	0	0	0	0	0	0

NMNAT1	0	0.103	0	0.114	0	0	0	0	0.185	0	0	0	0	0.147	0	0
NINJ2	0	0.106	0	0	0.206	0	0	0.128	0.109	0	0	0	0	0	0	0
KCNV1	0	0	0	0	0	0.151	0.113	0.108	0	0	0	0	0	0	0	0.176
INPP4A	0	0	0	0	0	0	0	0	0	0	0.111	0	0	0	0.437	0
VMA21	0	0	0	0.106	0	0	0	0	0.105	0.111	0	0.109	0.117	0	0	0
TRNI	0	0	0	0.219	0	0	0.153	0	0	0	0	0.176	0	0	0	0
ZFYVE27	0	0.155	0	0.162	0	0	0	0	0	0	0	0.231	0	0	0	0
NDE1	0	0.135	0.144	0	0	0	0	0	0	0	0.155	0	0	0	0.113	0
FRA12A	0.22	0	0.2	0	0	0	0	0	0	0	0	0	0.127	0	0	0
HTS	0	0	0	0.171	0	0	0	0	0	0	0	0.376	0	0	0	0
ATG16L1	0	0	0	0	0	0	0	0	0	0	0.2	0	0	0	0.347	0
CYFIP1	0.172	0	0.11	0	0	0.103	0	0	0	0	0	0	0.162	0	0	0
ZCCHC12	0	0	0.144	0	0	0.159	0	0.118	0	0	0	0	0.125	0	0	0
PMP22	0	0.131	0	0.159	0	0	0	0	0	0	0	0.256	0	0	0	0
TANC2	0	0	0.15	0	0	0.147	0.102	0	0	0	0	0	0.147	0	0	0
SETBP1	0	0	0.124	0	0	0.165	0.109	0	0	0	0	0	0.147	0	0	0
SLC5A7	0	0	0.111	0	0.316	0	0	0	0	0	0	0	0.118	0	0	0
TBL2	0.101	0	0	0	0	0	0.152	0.136	0	0	0	0	0	0	0.155	0
ARHGAP42	0	0	0	0.355	0	0	0	0	0	0	0	0	0	0	0.189	0
DFNA33	0	0	0.152	0	0	0	0	0	0	0.14	0	0.136	0.116	0	0	0
GLC1I	0	0	0	0.293	0	0	0.13	0	0	0	0	0.121	0	0	0	0
GJA1P1	0	0	0	0.236	0	0	0	0	0	0	0	0.308	0	0	0	0
SLC12A6	0	0	0	0.29	0	0.106	0	0	0	0	0	0.148	0	0	0	0
SEPT2	0	0.12	0	0	0.169	0	0	0	0.148	0	0	0	0	0	0.106	0
NAT8	0	0	0	0	0.116	0	0	0.144	0.121	0	0	0	0	0.162	0	0
DWS	0	0	0.116	0	0	0.168	0.132	0	0	0	0	0	0.127	0	0	0
ARFIP2	0	0	0	0	0	0	0	0	0	0	0.158	0	0	0.182	0.203	0
DFNB45	0	0	0.146	0	0	0	0	0	0	0.161	0	0.12	0.115	0	0	0
SPG34	0	0.133	0	0.104	0	0	0.121	0	0	0	0	0.184	0	0	0	0
FGD4	0	0	0	0.358	0	0	0	0	0	0	0	0.184	0	0	0	0
NOTCH3	0	0.129	0	0	0.168	0	0	0.102	0.143	0	0	0	0	0	0	0
TSHZ1	0	0.111	0	0	0.168	0	0	0.126	0.136	0	0	0	0	0	0	0
ITM2A	0	0	0	0	0.266	0	0	0.146	0.129	0	0	0	0	0	0	0
SPNS1	0	0	0	0	0	0	0	0	0	0.179	0	0	0	0.147	0.215	0
GPR85	0	0	0.132	0	0	0	0	0.254	0	0	0	0	0.153	0	0	0
PPP2R4	0.193	0	0	0	0.171	0	0	0	0.175	0	0	0	0	0	0	0
SV2A	0	0	0	0.41	0	0.129	0	0	0	0	0	0	0	0	0	0
GUCY1B3	0	0	0	0	0	0	0	0	0	0	0	0	0	0	0	0.538
CGGBP1	0.127	0	0.308	0	0	0	0	0	0	0	0	0	0.103	0	0	0
ANG	0	0.194	0	0	0.15	0	0	0	0.194	0	0	0	0	0	0	0
LEFTY2	0	0.109	0	0	0	0	0	0	0	0	0	0	0	0	0.156	0.272

C18ORF8	0.109	0	0	0	0.279	0	0	0	0	0	0	0	0	0.149	0	0
MAP3K10	0	0	0	0	0	0	0	0	0	0	0.198	0	0	0.145	0.194	0
GAK	0	0	0	0	0.287	0	0	0	0.138	0	0	0	0	0	0.111	0
MPV17	0	0	0	0	0	0	0.129	0	0	0	0	0.12	0.123	0.164	0	0
NLGN4Y	0	0	0.144	0	0	0.139	0.151	0	0	0	0	0	0.102	0	0	0
MCPH1	0	0	0.138	0	0	0	0	0	0	0	0	0	0.398	0	0	0
ZNF169	0	0	0	0.394	0	0	0	0	0	0	0	0.142	0	0	0	0
CALY	0	0	0.144	0	0	0	0	0	0	0.124	0.137	0	0.13	0	0	0
DFNB62	0	0	0.149	0	0	0	0	0	0	0.142	0	0.118	0.126	0	0	0
SLC25A12	0	0	0.151	0	0	0.143	0.122	0	0	0	0	0	0.119	0	0	0
MRS2	0	0	0	0.253	0	0	0.135	0	0	0	0	0	0	0.146	0	0
BZRAP1	0	0	0.129	0	0	0.146	0.113	0.146	0	0	0	0	0	0	0	0
PNMA6A	0	0	0	0	0	0.223	0.132	0.179	0	0	0	0	0	0	0	0
TUBB4Q	0	0	0	0.278	0	0	0	0	0	0	0.152	0	0	0	0.103	0
OTSC8	0	0	0	0.154	0	0	0	0	0	0	0	0.252	0.127	0	0	0
GUSBP3	0	0.107	0	0.111	0	0	0	0	0.167	0.148	0	0	0	0	0	0
FRG2C	0	0	0	0.349	0	0	0	0	0	0	0	0	0	0	0.183	0
TAR	0	0	0	0	0.168	0	0	0.253	0.111	0	0	0	0	0	0	0
CLCN2	0	0	0	0	0	0.221	0.207	0.104	0	0	0	0	0	0	0	0
PHGDH	0	0	0	0	0	0.222	0.185	0.125	0	0	0	0	0	0	0	0
PITRM1	0	0	0	0	0.12	0	0	0.167	0.111	0	0	0	0	0.133	0	0
DISC2	0.134	0	0.11	0	0	0	0	0	0.179	0	0	0	0.107	0	0	0
DSAP2	0	0	0	0.23	0	0	0	0	0	0	0	0.3	0	0	0	0
PRRG4	0	0	0	0	0.116	0	0	0.147	0.111	0	0	0	0	0.155	0	0
VDAC1	0.171	0.225	0	0	0	0	0	0	0	0	0	0	0	0.133	0	0
MCDR3	0	0	0	0.272	0	0	0	0	0	0.152	0	0.105	0	0	0	0
LOC202181	0	0	0	0	0	0	0	0	0	0	0.216	0	0	0.119	0.194	0
OR10A5	0	0	0	0.247	0	0	0	0	0	0	0	0.282	0	0	0	0
SYNC	0	0.12	0	0.115	0	0	0	0	0	0.294	0	0	0	0	0	0
HSPBP1	0	0	0	0	0	0	0	0	0	0	0.222	0	0	0.112	0.194	0
DUX4L5	0	0	0	0.239	0	0	0	0	0	0	0.176	0	0	0	0.113	0
DFNB68	0	0	0.123	0	0	0	0	0	0	0.11	0	0.19	0.105	0	0	0
STAC2	0	0.145	0	0.106	0	0	0	0	0	0	0	0.277	0	0	0	0
MYP18	0	0	0	0.308	0	0	0	0	0	0	0	0.22	0	0	0	0
POU3F2	0	0	0	0.287	0	0	0	0	0	0	0.128	0	0	0	0.112	0
FGD1	0	0	0	0.258	0	0	0.131	0	0	0	0	0	0.138	0	0	0
EMP3	0	0	0	0.376	0	0	0	0	0	0	0	0.151	0	0	0	0
HPT	0	0	0	0	0	0.234	0.151	0.142	0	0	0	0	0	0	0	0
EIG2	0	0	0	0	0	0.153	0.139	0.103	0	0	0	0	0	0	0	0.131
USP24	0	0	0	0	0.13	0	0	0	0.102	0	0.165	0	0	0.129	0	0
WFS1	0	0	0.107	0	0	0	0	0	0	0.152	0	0.108	0.159	0	0	0

POU3F4	0	0	0	0	0	0	0	0	0	0.185	0	0.137	0.204	0	0	0
PPT2-EGFL8	0	0	0	0	0.202	0	0	0.177	0	0	0	0	0	0.146	0	0
SYM2	0	0	0	0.237	0	0	0	0	0	0	0	0.181	0.107	0	0	0
DFNB59	0	0	0.127	0	0	0	0	0	0	0	0	0.254	0.144	0	0	0
C14ORF49	0	0	0	0.226	0	0	0	0	0	0	0	0.299	0	0	0	0
CMTDI2	0	0.103	0	0.16	0	0	0	0	0	0	0	0.261	0	0	0	0
FGD3	0	0	0	0.368	0	0	0	0	0	0	0	0.156	0	0	0	0
TREM2	0	0	0	0	0.138	0	0	0.144	0.242	0	0	0	0	0	0	0
TNFSF4	0.524	0	0	0	0	0	0	0	0	0	0	0	0	0	0	0
TPH1	0.125	0	0	0	0	0	0	0	0	0	0.181	0	0	0	0	0.217
GPD1L	0	0	0	0	0	0.181	0.188	0	0	0	0	0	0	0	0	0.154
GHRLOS	0	0	0	0	0	0	0	0	0	0	0.254	0	0	0	0	0.268
DST	0	0.103	0	0.112	0	0	0	0	0	0	0	0.204	0	0.103	0	0
FGD3	0	0	0	0.368	0	0	0	0	0	0	0	0.153	0	0	0	0
MHS6	0	0	0	0	0	0	0	0	0	0	0	0	0	0	0	0.52
PRKCG	0	0	0	0	0	0.154	0	0	0	0.117	0.126	0	0	0.123	0	0
SYNGR1	0	0	0.108	0	0	0.163	0	0.101	0	0	0	0	0.148	0	0	0
PCLO	0	0	0.11	0	0	0.169	0	0	0	0	0	0	0.241	0	0	0
CACNB2	0	0	0	0	0	0.186	0.231	0	0	0	0	0	0	0	0	0.102
GIPC2	0	0	0	0	0	0	0	0	0	0	0	0	0	0	0	0.519
DUX4L7	0	0	0	0.281	0	0	0	0	0	0	0.126	0	0	0	0.112	0
CAPN11	0	0	0	0	0	0	0	0	0	0	0.124	0	0	0	0.395	0
TDP1	0	0	0	0	0	0	0	0	0	0	0.129	0	0	0.39	0	0
SH3PXD2A	0	0	0	0	0.156	0	0	0.147	0.111	0	0	0	0	0.105	0	0
OTOF	0	0	0.154	0	0	0.145	0	0	0	0	0	0.119	0.101	0	0	0
KY	0	0.134	0	0.104	0	0	0	0	0	0.152	0	0.128	0	0	0	0
NFASC	0	0.153	0	0.101	0	0.164	0	0	0	0	0	0.1	0	0	0	0
NDIC	0	0	0	0.336	0	0	0	0	0	0	0	0.182	0	0	0	0
SLC6A11	0	0	0	0	0	0.114	0.132	0.1	0	0.172	0	0	0	0	0	0
PPARGC1A	0	0	0	0	0.145	0	0	0	0	0	0.157	0	0	0	0	0.215
HGSNAT	0	0	0	0	0	0	0	0	0	0	0	0	0.317	0.2	0	0
NAIP	0	0.103	0	0	0	0	0	0	0.131	0.175	0	0	0	0.108	0	0
TYROBP	0	0	0	0	0.321	0	0	0	0.196	0	0	0	0	0	0	0
FSHMD1A	0	0	0	0.359	0	0	0	0	0	0	0	0	0	0	0.157	0
HSPBAP1	0	0	0	0	0	0.232	0.153	0.131	0	0	0	0	0	0	0	0
QDPR	0	0	0	0	0	0	0	0	0	0	0.229	0	0	0	0	0.286
CCDC28B	0	0	0.148	0	0	0	0.131	0	0	0	0	0.123	0.113	0	0	0
HSPB1	0	0.114	0	0.104	0	0	0	0	0	0	0.15	0.147	0	0	0	0
DKBI	0	0	0	0.279	0	0	0	0	0	0	0	0.236	0	0	0	0
GCSH	0	0	0	0	0	0.231	0.171	0.113	0	0	0	0	0	0	0	0
NOL10	0	0	0	0	0	0	0	0	0	0	0.185	0	0	0.136	0.193	0

BOS2	0	0	0.149	0	0	0.142	0	0	0	0	0	0.119	0.104	0	0	0
MRX84	0	0	0.11	0	0	0.161	0.142	0	0	0	0	0	0.101	0	0	0
CIT	0	0	0.131	0	0	0.169	0	0	0	0	0	0	0.214	0	0	0
CHRNB3	0	0	0.101	0	0	0.176	0	0.119	0	0.118	0	0	0	0	0	0
GABRP	0	0	0	0	0	0.228	0.162	0.124	0	0	0	0	0	0	0	0
AKAP8L	0	0	0	0	0	0	0	0	0	0	0.155	0	0	0	0.358	0
TSPAN12	0	0	0	0	0.247	0	0	0	0	0	0	0	0	0	0	0.265
THAP11	0	0	0	0	0	0	0	0	0	0	0.34	0	0	0	0.172	0
CAPN14	0	0	0	0	0	0	0	0	0	0	0.119	0	0	0	0.392	0
ECEL1	0	0.146	0	0.101	0	0	0	0	0	0	0	0.14	0	0.124	0	0
TMEM11	0	0	0	0.403	0	0	0	0	0	0	0	0	0	0.108	0	0
NIPA2	0	0	0.124	0	0	0.141	0.11	0	0	0	0	0	0.136	0	0	0
MED25	0	0	0	0.4	0	0	0	0	0	0	0	0.111	0	0	0	0
SPPL2A	0.131	0	0	0	0.135	0	0	0	0.245	0	0	0	0	0	0	0
IQCJ	0	0	0	0.153	0	0	0	0	0	0	0	0.164	0	0	0	0.193
DNAH6	0	0	0	0	0	0	0	0	0	0	0.111	0	0	0	0	0.399
ATP8	0	0	0	0	0	0	0.173	0	0	0	0.129	0	0	0.103	0.105	0
LOC647275	0	0	0	0.156	0	0	0.109	0	0	0	0	0.137	0.108	0	0	0
FOXG1	0	0.131	0	0	0	0.208	0	0	0	0.171	0	0	0	0	0	0
STRCP1	0	0	0	0.379	0	0	0	0	0	0	0	0.13	0	0	0	0
GPX7	0	0	0	0	0.157	0	0	0.154	0.198	0	0	0	0	0	0	0
VPS26A	0	0	0	0	0.185	0	0	0.105	0.112	0	0	0	0	0.106	0	0
DFNB33	0	0	0.108	0.103	0	0	0	0	0	0	0	0.123	0.174	0	0	0
CACD	0	0	0	0.264	0	0	0	0	0	0.133	0	0.111	0	0	0	0
JAKMIP1	0	0.182	0	0	0	0.209	0	0.117	0	0	0	0	0	0	0	0
HTR7P1	0	0	0	0	0	0	0	0	0	0	0.18	0	0	0	0	0.327
NDUFB4	0	0	0	0	0	0	0.124	0	0	0	0.119	0	0	0.149	0.115	0
ATG10	0	0	0	0	0	0	0	0	0	0	0.198	0	0	0.105	0.204	0
COMMD7	0	0	0	0	0	0	0	0	0	0	0	0	0	0	0.507	0
PPIL2	0	0	0	0	0.198	0	0	0.203	0.106	0	0	0	0	0	0	0
HSPB2	0	0.15	0	0.115	0	0	0	0	0	0	0.138	0	0	0.103	0	0
SHFM1D	0	0	0.126	0	0	0	0	0	0	0	0	0.254	0.126	0	0	0
MAG	0	0.114	0	0.115	0	0	0	0	0	0.173	0	0.104	0	0	0	0
IL1A	0	0	0	0	0.157	0	0	0.19	0.159	0	0	0	0	0	0	0
DNAJA1	0	0	0	0	0	0	0	0	0	0	0.204	0	0	0.111	0.19	0
AIFM1	0	0.122	0	0	0	0	0	0	0	0.117	0.157	0	0	0.109	0	0
PRHOXNB	0	0	0	0	0	0	0	0	0	0	0	0	0	0	0.277	0.227
PCP2	0	0	0	0	0	0	0	0	0	0.107	0.153	0	0	0.129	0.115	0
CDB2	0	0	0	0.228	0	0	0	0	0	0.165	0	0.111	0	0	0	0
HDGFRP3	0	0	0	0	0.148	0	0	0.188	0.168	0	0	0	0	0	0	0
LRP1	0.125	0	0	0	0.183	0	0	0	0.196	0	0	0	0	0	0	0

UBQLN4	0	0	0	0	0	0	0	0	0	0	0.167	0	0	0.12	0.216	0
DMBX1	0	0	0	0	0	0	0	0	0	0	0	0	0	0	0.503	0
EN1	0	0	0	0	0	0	0	0	0	0	0	0	0	0	0.503	0
ERVV-1	0	0	0	0	0	0	0	0	0	0	0	0	0	0	0.503	0
DFNY1	0	0	0.149	0	0	0	0	0	0	0.119	0	0.104	0.13	0	0	0
GINGF2	0	0	0	0.229	0	0	0	0	0	0	0.163	0.11	0	0	0	0
KCNJ6	0	0	0	0	0	0	0	0	0	0.132	0	0	0	0.122	0	0.247
PTS	0	0	0	0	0	0	0	0	0	0	0	0	0.28	0	0	0.221
CCBL2	0	0	0.124	0	0	0	0	0.142	0	0	0.122	0	0	0	0.113	0
CIB4	0.232	0	0	0	0	0	0	0	0	0	0	0	0	0	0.269	0
CC2D1A	0	0	0.124	0	0	0	0	0	0	0.198	0	0	0.179	0	0	0
LRRN4	0	0	0.109	0	0	0	0	0.225	0	0	0	0	0.167	0	0	0
IS2	0	0	0	0.277	0	0.105	0	0	0	0	0	0.119	0	0	0	0
ATL3	0	0.168	0	0.216	0	0	0	0	0	0	0	0.117	0	0	0	0
CMD1C	0	0	0	0.212	0	0	0	0	0	0	0	0.289	0	0	0	0
UBIAD1	0	0	0	0.306	0	0	0	0	0	0	0	0.195	0	0	0	0
CHED1	0	0	0	0.202	0	0	0	0	0	0.185	0	0.113	0	0	0	0
ATL2	0	0.109	0	0.104	0	0	0	0	0.154	0	0	0.133	0	0	0	0
CASP12	0	0.125	0	0	0	0	0	0	0	0.152	0.115	0	0	0.107	0	0
SLC6A12	0	0	0	0	0	0.204	0.176	0.119	0	0	0	0	0	0	0	0
GAP43	0	0.118	0	0	0	0.2	0	0	0	0.18	0	0	0	0	0	0
PDE4B	0	0	0.129	0	0	0	0	0	0	0	0	0	0.111	0	0	0.257
ARID1B	0	0	0	0	0	0	0	0	0	0	0.145	0	0	0	0.352	0
TRNS1	0	0	0.126	0	0	0	0	0	0	0	0	0.254	0.117	0	0	0
ALG3	0	0	0	0	0	0.182	0.174	0	0	0	0	0	0.141	0	0	0
UGT8	0	0	0	0.175	0	0	0	0	0	0	0	0.12	0	0.2	0	0
DBNDD2	0	0	0	0	0.206	0	0	0	0.179	0	0	0	0	0.11	0	0
DYX1C1	0	0	0.111	0	0	0	0	0	0	0	0	0	0.384	0	0	0
DLGAP2	0	0	0	0.187	0	0	0.131	0	0	0	0	0	0	0	0	0.176
HIST1H2APS5	0	0	0	0	0	0	0	0	0	0	0	0	0	0	0.494	0
ADFN	0	0	0.144	0.103	0	0	0	0	0	0	0	0.122	0.125	0	0	0
BDET	0	0	0	0.173	0	0	0	0	0	0	0	0.321	0	0	0	0
OTOL1	0	0	0	0	0	0	0	0	0	0	0	0.298	0	0	0	0.195
RILPL1	0	0.101	0	0	0	0	0	0	0	0	0.166	0	0	0.109	0.117	0
FASA	0	0	0	0	0	0	0	0	0	0	0.125	0	0	0.176	0.192	0
TCERG1	0	0	0	0	0.138	0	0	0	0.126	0	0.117	0	0	0	0.112	0
TRNL2	0	0	0	0	0.166	0	0	0	0.12	0	0	0.106	0	0.101	0	0
FRA11A	0.146	0	0.194	0	0	0	0	0	0	0	0	0	0.153	0	0	0
PANX2	0	0	0	0.351	0	0.142	0	0	0	0	0	0	0	0	0	0
HTR3B	0	0	0	0	0	0	0	0	0	0	0	0	0	0	0.19	0.302
TPH2	0.109	0	0.112	0	0	0	0	0	0	0	0	0	0.136	0	0	0.135

SYVN1	0	0	0	0	0.13	0	0	0	0.139	0	0.117	0	0	0.106	0	0
TAF15	0	0.172	0	0	0.201	0	0	0	0.119	0	0	0	0	0	0	0
GABRQ	0	0	0	0	0	0.228	0.133	0.131	0	0	0	0	0	0	0	0
BDMF	0	0	0	0.106	0	0.134	0	0	0.12	0	0	0.131	0	0	0	0
CDH12P2	0	0.116	0	0	0.134	0	0	0	0.241	0	0	0	0	0	0	0
GHRL	0	0	0	0	0	0	0	0	0	0	0.218	0	0	0	0	0.272
RTN1	0	0.16	0	0.179	0	0	0	0	0	0	0	0	0	0.151	0	0
RAB39	0	0	0	0	0	0.224	0.149	0.117	0	0	0	0	0	0	0	0
MYL6B	0.49	0	0	0	0	0	0	0	0	0	0	0	0	0	0	0
FXYD1	0	0	0	0	0	0.181	0.128	0	0	0	0	0	0	0	0	0.18
NUDT4	0	0	0	0	0	0	0	0	0	0	0.383	0	0	0	0.106	0
TGM3	0	0	0	0	0	0	0	0	0	0	0.371	0	0	0	0.118	0
FOXRED2	0	0	0	0	0.381	0	0	0	0	0	0	0	0	0.108	0	0
DNAH10	0	0	0	0	0	0	0	0	0	0	0.216	0	0	0	0	0.272
GJB2	0	0	0.127	0	0	0	0	0	0	0	0	0.19	0.171	0	0	0
PON1	0	0.112	0	0	0.202	0	0	0	0.174	0	0	0	0	0	0	0
MIR217	0	0	0	0	0	0	0	0	0	0	0	0	0	0	0	0.487
BSX	0	0	0	0	0	0	0	0	0	0	0.125	0	0	0	0.362	0
AATF	0	0	0	0	0.102	0	0	0.138	0.118	0	0	0	0	0.129	0	0
DFNB5	0	0	0.152	0	0	0	0	0	0	0.119	0	0.112	0.104	0	0	0
POMT1	0	0	0	0.24	0	0	0	0	0	0.105	0	0	0.142	0	0	0
KCTD16	0	0	0	0	0	0.226	0.158	0.103	0	0	0	0	0	0	0	0
MESDC2	0.487	0	0	0	0	0	0	0	0	0	0	0	0	0	0	0
DVL1	0.172	0.114	0	0	0	0	0	0	0	0	0	0	0	0	0	0.2
TGM2	0	0	0	0	0	0	0	0	0	0	0.161	0	0	0.118	0.207	0
CRMP1	0.181	0	0	0	0	0	0	0	0	0	0.169	0	0	0.136	0	0
ADARB1	0	0.118	0	0	0	0	0	0.119	0.127	0	0.122	0	0	0	0	0
ZMYM5	0.218	0	0	0	0.153	0	0	0	0.115	0	0	0	0	0	0	0
OCLN	0	0.302	0	0	0.184	0	0	0	0	0	0	0	0	0	0	0
UACA	0	0.136	0	0	0	0	0	0	0	0	0	0	0	0.13	0.219	0
TRAK1	0	0.16	0	0.18	0	0	0	0	0	0	0	0	0	0.145	0	0
MHW2	0.132	0	0.124	0	0	0	0	0	0	0	0	0	0.124	0	0.104	0
A2M	0.144	0	0	0	0.13	0	0	0.106	0.104	0	0	0	0	0	0	0
VCX2	0	0	0.13	0	0	0.171	0	0	0	0	0	0	0.182	0	0	0
HSPA9	0	0	0	0	0.182	0	0	0	0	0	0	0	0	0.145	0	0.156
HERPUD1	0	0	0	0	0.197	0	0	0	0.168	0	0	0	0	0.118	0	0
DFNA21	0	0	0.149	0	0	0	0	0	0	0.121	0	0.106	0.107	0	0	0
CHRM4	0	0	0.13	0	0.234	0	0	0	0	0	0	0	0.119	0	0	0
DFFA	0	0	0	0	0	0	0	0	0	0	0.167	0	0	0.125	0.19	0
HIP1R	0	0	0	0	0	0	0	0	0	0	0.151	0	0	0.139	0.192	0
DFNA52	0	0	0	0.262	0	0	0	0	0	0	0	0.22	0	0	0	0

DHS	0	0	0	0.316	0	0	0	0	0	0	0	0.166	0	0	0	0
PEX5L	0	0	0	0	0	0.222	0.147	0.113	0	0	0	0	0	0	0	0
SPG25	0	0.105	0	0.114	0	0	0	0	0	0.157	0	0.105	0	0	0	0
ZNF180	0	0.126	0	0.18	0	0	0	0	0	0	0	0.174	0	0	0	0
LOC619540	0	0	0	0.347	0	0	0	0	0	0	0	0.133	0	0	0	0
CDKL2	0	0	0	0	0	0.216	0.16	0.104	0	0	0	0	0	0	0	0
KRT27	0.48	0	0	0	0	0	0	0	0	0	0	0	0	0	0	0
SCAMP5	0	0	0	0	0	0	0	0	0	0	0.114	0	0	0.172	0.193	0
DFNM2	0	0	0	0.26	0	0	0	0	0	0	0	0.107	0.112	0	0	0
DFNB13	0	0	0.125	0	0	0	0	0	0	0	0	0.239	0.115	0	0	0
TW	0	0	0	0.225	0	0	0	0	0	0	0	0.254	0	0	0	0
ACE	0	0	0	0	0.151	0	0	0	0.12	0	0	0	0	0	0	0.207
MCCC1	0	0	0	0	0	0.182	0.142	0	0	0	0	0	0.154	0	0	0
ATFB1	0	0	0	0	0	0	0	0	0	0	0	0	0	0	0.308	0.169
BAG3	0	0.114	0	0.104	0	0	0	0	0	0	0.138	0	0	0.121	0	0
C1ORF130	0	0	0	0.258	0	0	0	0	0	0	0	0.218	0	0	0	0
B4GALNT1	0	0	0	0.226	0	0	0	0	0	0.111	0	0	0	0.138	0	0
MRPS18CP2	0	0	0.128	0	0	0	0	0	0	0	0	0.166	0.181	0	0	0
ATG12	0	0	0	0	0	0	0	0	0.111	0	0.134	0	0	0.119	0.111	0
CAPN9	0	0	0	0	0	0	0	0	0	0	0.111	0	0	0	0.364	0
RPL14	0	0	0	0	0	0	0	0	0	0	0.369	0	0	0	0.106	0
PSMF1	0	0	0	0	0.163	0	0	0.142	0.17	0	0	0	0	0	0	0
BHLHE22	0.126	0	0	0	0	0	0	0.161	0	0	0	0	0	0	0.187	0
DUX4L9	0	0	0	0.247	0	0	0	0	0	0	0.125	0	0	0	0.102	0
NGFRAP1	0	0.108	0	0	0	0	0	0	0	0	0	0.254	0	0.112	0	0
ATG9B	0	0	0	0	0	0	0	0	0.228	0	0.135	0	0	0.111	0	0
SCN7A	0	0	0	0.218	0	0.137	0	0	0	0	0	0.119	0	0	0	0
ZNF175	0	0	0	0	0.174	0	0	0.163	0.137	0	0	0	0	0	0	0
KCNH5	0	0	0	0	0	0.177	0	0.111	0	0	0	0	0	0	0	0.185
KCNA4	0	0	0	0.165	0	0.179	0	0	0	0	0	0	0	0	0	0.129
D4S234E	0	0.162	0	0	0	0	0	0	0	0	0.189	0	0	0.122	0	0
GPR126	0	0	0	0.316	0	0	0	0	0	0	0	0.157	0	0	0	0
MAP1LC3B	0	0	0	0	0	0	0	0	0.203	0	0.139	0	0	0.13	0	0
GJA3	0	0	0	0.28	0	0	0	0	0	0	0	0.192	0	0	0	0
MCPH4	0	0	0	0	0	0	0	0	0	0	0	0.175	0.126	0	0	0.171
IAPP	0	0	0	0	0.174	0	0	0	0.109	0	0	0	0	0	0.189	0
C7ORF11	0	0	0	0	0	0.157	0.145	0	0	0	0	0	0.17	0	0	0
KCNQ4	0	0	0	0	0	0.18	0	0	0	0	0	0.101	0	0	0	0.19
SYT1	0	0	0.126	0	0	0.16	0	0	0	0	0	0	0	0	0.185	0
VENTXP7	0	0	0	0	0	0	0	0	0	0	0.285	0	0	0	0.186	0
CBLN3	0	0	0	0	0	0.187	0	0	0	0.169	0	0	0	0.115	0	0

BASP1P1	0	0	0.11	0.102	0	0	0	0	0	0	0	0.115	0.144	0	0	0
PLLP	0	0	0	0.29	0	0	0	0	0	0	0	0.181	0	0	0	0
PDB1	0	0.139	0	0	0.169	0	0	0	0.162	0	0	0	0	0	0	0
COPD22	0	0	0	0.257	0	0	0	0	0	0	0	0	0	0	0	0.213
RAB40C	0	0	0	0.288	0	0	0	0	0	0	0	0	0	0	0.182	0
AHI1	0	0	0.131	0	0	0	0	0	0	0.19	0	0	0.149	0	0	0
KLC3	0	0	0	0.254	0	0	0	0	0	0	0	0.216	0	0	0	0
IMPA1	0.167	0	0	0	0.181	0	0	0.122	0	0	0	0	0	0	0	0
PFM3	0	0	0	0.219	0	0	0	0	0	0.117	0	0.133	0	0	0	0
DNAJB1P1	0	0	0	0	0	0	0	0	0	0	0.236	0	0	0.116	0.117	0
ATG9A	0	0	0	0	0.123	0	0	0	0.113	0	0.132	0	0	0.101	0	0
C15ORF24	0	0	0	0.321	0	0	0	0	0.148	0	0	0	0	0	0	0
ATP1B3	0	0	0	0	0	0.129	0.158	0	0	0	0	0	0	0	0	0.181
COPD19	0	0	0	0.324	0	0	0	0	0	0	0	0	0	0	0	0.144
REST	0	0	0	0	0	0.187	0	0	0	0	0.179	0	0	0	0.102	0
HERC2P6	0	0	0	0	0	0.188	0.125	0	0	0	0	0	0	0	0.155	0
EML1	0	0	0.151	0	0	0	0	0	0	0.101	0	0.103	0.113	0	0	0
AFA	0	0	0.15	0.103	0	0	0	0	0	0	0	0.1	0.115	0	0	0
SMARCAL1	0	0	0.131	0	0	0	0.178	0	0	0	0	0	0.159	0	0	0
GNG12	0.112	0.131	0	0	0	0	0	0.122	0.103	0	0	0	0	0	0	0
IL1B	0	0	0	0	0.163	0	0	0	0.114	0	0	0	0	0	0	0.19
ACCN4	0	0	0	0	0	0.18	0	0	0	0	0	0.105	0	0	0	0.182
DGCR6	0	0	0	0	0	0.221	0.132	0.114	0	0	0	0	0	0	0	0
KCNK9	0	0	0	0	0	0.232	0.132	0.103	0	0	0	0	0	0	0	0
ATP1A4	0	0	0	0	0	0.186	0.113	0	0	0	0	0	0	0	0	0.167
CSNK1D	0.167	0	0	0	0.159	0	0	0	0.139	0	0	0	0	0	0	0
GRID2IP	0	0	0	0	0	0.18	0	0	0	0	0	0	0	0.148	0	0.137
DYX7	0	0	0.129	0	0	0	0	0	0	0	0	0	0.118	0	0	0.218
COPD23	0	0	0	0.282	0	0	0	0	0	0	0	0	0	0	0	0.183
GABRE	0	0	0	0	0	0.229	0.132	0.104	0	0	0	0	0	0	0	0
NOS3	0	0	0	0	0.157	0	0	0	0.139	0	0	0	0	0	0	0.168
SNORD115@	0	0	0	0	0	0.178	0.126	0	0	0	0	0	0	0	0	0.16
ANXA2P2	0.143	0	0	0	0	0	0	0	0	0	0	0	0	0	0.321	0
POMGNT1	0	0	0	0.24	0	0	0	0	0	0.119	0	0	0.105	0	0	0
IFT57	0	0	0	0	0	0	0	0	0	0	0.165	0	0	0.101	0.197	0
TUBA3C	0	0	0	0	0.145	0	0	0	0.129	0	0	0	0	0	0.189	0
PIGCP1	0	0	0	0	0	0.133	0.109	0	0	0	0	0	0	0	0	0.221
UBE2G1	0	0	0	0	0.186	0	0	0	0.132	0	0	0	0	0.145	0	0
GHS	0	0	0.109	0	0	0	0.175	0	0	0	0	0	0.179	0	0	0
GABRR1	0	0	0	0	0	0.227	0.132	0.104	0	0	0	0	0	0	0	0
GRIP2	0	0.131	0	0	0	0.207	0.125	0	0	0	0	0	0	0	0	0

RNR1	0	0	0.127	0	0	0	0	0	0	0	0	0.209	0.126	0	0	0
ATRX	0	0	0.123	0	0	0	0.177	0	0	0	0	0	0.162	0	0	0
HPLH1	0	0	0	0.213	0	0	0	0	0.147	0	0	0.102	0	0	0	0
KCTD12	0	0	0	0	0	0.227	0.132	0.103	0	0	0	0	0	0	0	0
KCNN2	0	0	0	0	0	0.179	0	0.153	0	0	0	0	0	0	0	0.129
UROC1	0	0	0	0	0	0.184	0.158	0	0	0	0	0	0.119	0	0	0
KIF1B	0	0.121	0	0.101	0	0	0	0	0.11	0	0	0.128	0	0	0	0
TMEM167A	0	0	0	0.282	0	0	0	0	0	0	0	0.178	0	0	0	0
GABRR3	0	0	0	0	0	0.23	0.126	0.104	0	0	0	0	0	0	0	0
KCND1	0	0	0	0	0	0.177	0	0.113	0	0	0	0	0	0	0	0.169
DUX4L4	0	0	0	0.236	0	0	0	0	0	0	0.12	0	0	0	0.103	0
CASP3	0	0	0	0	0.184	0	0	0	0.122	0	0.153	0	0	0	0	0
EPB41L5	0	0	0	0	0	0	0	0	0	0	0	0	0	0	0	0.458
CYHR1	0.189	0	0	0	0	0	0	0	0	0	0	0	0	0	0.269	0
CACNA1B	0	0	0	0	0	0.186	0.144	0	0	0	0	0	0	0	0	0.128
ENUR2	0	0	0	0.317	0	0	0	0	0	0	0	0.141	0	0	0	0
GAL3ST1	0	0	0	0.159	0	0	0	0	0	0	0	0.299	0	0	0	0
DNAJB8	0	0	0	0	0	0	0	0	0	0	0.162	0	0	0.105	0.19	0
CPNE9	0.188	0	0	0	0	0	0	0	0	0	0	0	0	0	0.269	0
FMR1-AS1	0.161	0	0.152	0	0	0	0	0	0	0	0	0	0.144	0	0	0
MOB2	0	0	0	0	0.186	0	0	0.145	0.126	0	0	0	0	0	0	0
TAMM41	0	0	0.108	0	0.236	0	0	0	0.113	0	0	0	0	0	0	0
AGER	0	0	0	0	0.218	0	0	0	0.239	0	0	0	0	0	0	0
SLC6A13	0	0	0	0	0	0.183	0.156	0.118	0	0	0	0	0	0	0	0
MTM1	0	0	0	0.342	0	0	0	0	0	0	0	0.114	0	0	0	0
MAOA	0	0	0	0	0.114	0	0	0	0.201	0	0	0	0	0	0	0.141
HOXA2	0	0	0	0.35	0	0	0	0	0	0	0	0	0	0	0.106	0
DFNB38	0	0	0.123	0	0	0	0	0	0	0.114	0	0.113	0.106	0	0	0
MNX1	0	0.103	0	0.176	0	0	0	0	0	0	0	0.177	0	0	0	0
CANDF1	0	0	0	0.304	0	0	0	0	0	0	0	0.152	0	0	0	0
NDRG4	0	0	0	0.285	0	0	0	0	0	0	0	0.17	0	0	0	0
PBCRA1	0	0	0	0.224	0	0	0	0	0	0.121	0.11	0	0	0	0	0
DLX5	0	0	0	0	0	0.297	0	0	0	0.158	0	0	0	0	0	0
SERPINA1	0	0	0	0	0.286	0	0	0	0.169	0	0	0	0	0	0	0
PNMA3	0	0	0	0	0	0.221	0.132	0.102	0	0	0	0	0	0	0	0
NPPC	0	0	0	0	0	0	0	0	0	0	0	0	0	0	0	0.454
TBR1	0	0	0	0	0	0.186	0	0	0	0	0.155	0	0	0	0.113	0
SRSF9	0	0.136	0	0	0.176	0	0	0	0.142	0	0	0	0	0	0	0
NPHP3-ACAD11	0	0	0	0	0	0	0	0	0	0.118	0	0.152	0	0	0	0.183
STAC	0	0	0	0	0	0	0	0	0	0	0.132	0.198	0	0.123	0	0
RTN4RL2	0	0.111	0	0.159	0	0	0	0	0	0	0	0.183	0	0	0	0

GPX6	0	0	0	0	0.139	0	0	0.188	0.126	0	0	0	0	0	0	0
LRPAP1	0.115	0	0	0	0.187	0	0	0	0.151	0	0	0	0	0	0	0
WDPCP	0	0.138	0	0	0	0	0	0	0.116	0	0	0	0	0	0	0.198
VSX1	0	0.148	0	0	0	0	0	0	0	0.167	0	0.137	0	0	0	0
OTSC2	0	0	0	0	0	0	0	0	0	0	0	0.21	0.126	0	0.116	0
ZNF598	0	0.148	0	0.179	0	0	0	0	0	0	0	0.125	0	0	0	0
TRPV4	0	0	0	0.311	0	0	0	0	0	0	0	0.141	0	0	0	0
PLEKHB2	0	0.345	0	0.107	0	0	0	0	0	0	0	0	0	0	0	0
KIF5C	0	0.109	0	0.101	0	0	0	0	0	0	0.138	0	0	0	0.102	0
EGR2	0	0	0	0.328	0	0	0	0	0	0	0	0.121	0	0	0	0
FIS1	0	0	0	0.297	0	0	0	0	0	0	0	0	0	0.152	0	0
RP2	0	0	0	0	0	0	0	0	0	0.161	0	0.288	0	0	0	0
SLC44A3	0	0.171	0	0	0.169	0	0	0.108	0	0	0	0	0	0	0	0
GLULP4	0.126	0	0	0	0	0	0	0	0	0	0.222	0	0	0	0.1	0
MMP3	0	0	0	0	0.286	0	0	0	0.162	0	0	0	0	0	0	0
KCNJ18	0	0	0	0.119	0	0.151	0	0	0	0	0	0	0	0	0	0.177
CTNNA3	0	0	0	0	0.112	0	0	0.198	0.137	0	0	0	0	0	0	0
LCA5L	0	0	0	0	0	0	0	0	0	0.12	0	0.105	0	0	0	0.222
CHGA	0	0.105	0	0	0.175	0	0	0	0.167	0	0	0	0	0	0	0
DUX4L3	0	0	0	0.218	0	0	0	0	0	0	0.126	0	0	0	0.102	0
PLEKHM1	0	0	0	0	0.14	0	0	0	0.151	0	0	0	0	0.154	0	0
PON2	0	0.101	0	0	0.177	0	0	0	0.167	0	0	0	0	0	0	0
DLG2	0	0	0	0.245	0	0	0	0	0	0	0	0	0	0	0	0.2
GAMT	0	0	0.123	0	0	0	0.132	0	0	0	0	0	0.19	0	0	0
KCNJ4	0	0	0	0	0	0.177	0	0	0	0	0.268	0	0	0	0	0
SLC25A19	0	0	0	0	0	0	0.11	0	0	0	0	0.221	0	0.113	0	0
SOX10	0	0	0	0.323	0	0	0	0	0	0	0	0.121	0	0	0	0
CNGA1	0	0	0	0	0	0.13	0	0	0	0.198	0.116	0	0	0	0	0
JPH4	0	0	0	0	0	0.181	0	0	0	0	0	0	0	0.107	0	0.156
FTL	0	0	0	0	0	0	0	0	0	0	0.182	0.138	0	0.123	0	0
NAV2	0	0	0	0.312	0	0	0	0	0	0	0	0.131	0	0	0	0
GALR1	0	0	0.145	0	0	0.167	0	0	0	0.131	0	0	0	0	0	0
SH3BGRL2	0	0	0	0	0	0.226	0.114	0.103	0	0	0	0	0	0	0	0
LOC246784	0	0	0	0	0	0.187	0	0.108	0	0	0	0	0	0.148	0	0
RNF166	0.126	0	0	0.161	0	0	0	0	0	0	0	0	0	0	0.155	0
KCNA2	0	0	0	0.111	0	0.131	0	0	0	0	0	0	0	0	0	0.2
FEN1	0	0	0	0	0	0	0	0	0	0	0.338	0	0	0	0.104	0
ADSL	0	0	0	0	0	0.181	0.131	0	0	0	0	0	0.13	0	0	0
DBNDD1	0	0	0.138	0	0	0	0	0	0	0	0	0	0.304	0	0	0
PCDH15	0	0	0	0	0	0	0	0	0	0.189	0	0.253	0	0	0	0
MTMR1	0	0	0	0.239	0	0	0	0	0	0	0	0.203	0	0	0	0

NOX1	0	0	0	0	0.155	0	0	0	0.139	0	0	0	0	0.147	0	0
CDH23	0	0.12	0	0	0	0	0	0	0	0.152	0	0.169	0	0	0	0
SPD3	0	0	0	0.281	0	0	0	0	0	0	0	0.16	0	0	0	0
WBSCR17	0	0	0	0	0	0.183	0.1	0.158	0	0	0	0	0	0	0	0
PPARGC1B	0	0	0	0	0	0	0	0	0	0	0.141	0	0	0	0	0.3
SYT14	0.141	0	0	0	0	0	0	0	0	0	0	0	0	0	0.3	0
MAFD1	0.104	0	0.123	0	0	0.107	0	0	0	0	0	0	0.107	0	0	0
MTX1P1	0	0	0	0	0.286	0	0	0	0.155	0	0	0	0	0	0	0
ADK	0	0	0	0	0	0.153	0.124	0	0	0	0.163	0	0	0	0	0
IGHV4-39	0	0.163	0	0	0	0	0	0.277	0	0	0	0	0	0	0	0
CAPNS1	0	0	0	0	0	0	0	0	0	0	0.125	0	0	0.125	0.19	0
NOMO2	0	0	0	0	0.167	0	0	0.129	0.143	0	0	0	0	0	0	0
LRSAM1	0.275	0	0	0.164	0	0	0	0	0	0	0	0	0	0	0	0
MSN	0	0.246	0	0	0	0.193	0	0	0	0	0	0	0	0	0	0
DFNB17	0	0	0.109	0	0	0	0	0	0	0	0	0.186	0.144	0	0	0
TMEM200B	0.144	0	0.154	0	0	0	0	0	0	0	0	0	0.141	0	0	0
MAPK8IP2	0.144	0	0	0	0.181	0	0	0	0.114	0	0	0	0	0	0	0
CACNA1C	0	0	0	0	0	0.163	0	0	0	0	0	0	0	0	0	0.275
FEZ1	0	0	0.131	0	0	0	0	0	0.149	0	0	0	0.158	0	0	0
C10ORF67	0	0.121	0	0	0	0.2	0	0	0	0	0	0	0	0	0.117	0
PIGFP1	0	0	0	0	0	0.178	0.152	0	0	0	0	0	0	0	0	0.108
KCNC4	0	0	0	0	0	0.158	0	0.13	0	0	0	0	0	0	0	0.149
HMI	0	0	0.124	0	0	0.171	0	0	0	0	0	0	0.142	0	0	0
COD2	0	0	0	0.271	0	0	0	0	0	0.166	0	0	0	0	0	0
PNKD	0	0	0	0	0	0.178	0.125	0	0	0	0	0	0	0	0	0.134
ADCY8	0	0	0.131	0	0	0	0	0	0	0.184	0	0	0.122	0	0	0
DFNB46	0	0	0	0.246	0	0	0	0	0	0	0	0.19	0	0	0	0
KERSMCR	0	0	0	0.26	0	0	0	0	0	0	0	0.176	0	0	0	0
AUTS2	0	0	0.131	0	0	0.171	0	0	0	0	0	0	0.133	0	0	0
NQO1	0	0	0	0	0	0	0	0	0	0	0	0	0	0	0	0.435
DNAJC5	0	0	0	0	0	0	0	0	0	0	0.217	0	0	0.101	0.117	0
CD200R1	0	0.111	0	0	0.148	0	0	0	0.176	0	0	0	0	0	0	0
APOC1P1	0.123	0	0	0	0.181	0	0	0	0.131	0	0	0	0	0	0	0
GGA1	0	0	0	0	0.139	0	0	0	0.296	0	0	0	0	0	0	0
COA5	0	0	0	0	0	0	0.105	0	0	0	0	0.174	0	0.156	0	0
FAM134C	0	0	0	0.219	0	0	0	0	0	0	0	0.215	0	0	0	0
SLC4A10	0	0	0	0	0	0.128	0.188	0.118	0	0	0	0	0	0	0	0
CNIH2	0	0	0	0	0	0.186	0.102	0	0	0	0	0	0	0	0	0.146
OPN5	0	0	0.122	0	0	0	0	0	0	0.164	0	0	0.147	0	0	0
RGS4	0	0	0.122	0	0	0	0	0	0	0.113	0	0	0.198	0	0	0
DUX4L6	0	0	0	0.208	0	0	0	0	0	0	0.123	0	0	0	0.102	0

CD46P1	0	0	0	0	0	0	0	0	0	0	0	0	0	0	0.218	0.214
MHW1	0.111	0	0.103	0	0	0	0	0	0	0	0	0	0.115	0	0.103	0
APOPT1	0.127	0	0	0	0	0	0	0	0	0	0	0	0	0	0.305	0
GPR137B	0	0.148	0	0.166	0	0	0	0	0	0	0	0	0	0.118	0	0
TYS	0	0	0	0	0	0.182	0.102	0	0	0	0	0.148	0	0	0	0
5-HT3C2	0	0	0	0	0	0	0	0	0	0	0	0	0	0	0.245	0.186
OFD1	0	0	0	0	0	0	0	0	0	0	0	0.107	0.126	0	0	0.198
ASMD	0	0	0	0.198	0	0	0	0	0	0	0.121	0	0	0	0.112	0
POU3F1	0	0	0	0.215	0	0	0	0	0	0	0	0.216	0	0	0	0
E2F7	0	0	0	0	0	0	0	0	0	0	0.431	0	0	0	0	0
FLJ45872	0	0	0.126	0	0	0	0	0	0.115	0	0	0	0	0	0.189	0
SCZD12	0	0	0.133	0.114	0	0	0	0	0	0	0	0	0.183	0	0	0
KIFC2	0	0.119	0	0.117	0	0	0	0	0	0	0	0.194	0	0	0	0
CKBP1	0	0	0	0	0	0	0	0	0	0	0.43	0	0	0	0	0
SH3TC2	0	0.119	0	0.106	0	0	0	0	0	0	0	0.205	0	0	0	0
TMEM67	0	0	0	0	0	0	0	0	0	0	0	0.158	0.126	0	0	0.145
KIF4B	0	0.26	0	0.169	0	0	0	0	0	0	0	0	0	0	0	0
CHCHD2P9	0	0	0	0	0	0	0	0	0	0	0	0	0	0	0.429	0
ALS2CL	0	0.138	0	0.117	0	0	0	0	0	0	0	0.174	0	0	0	0
LAMP2	0	0	0	0.275	0	0	0	0	0	0	0	0	0	0.153	0	0
CRX	0	0	0	0	0	0	0	0	0	0.165	0.149	0	0	0	0.114	0
DNM1	0	0	0.11	0.103	0	0	0	0	0	0	0.102	0	0	0	0.113	0
NBR1	0	0	0	0	0.197	0	0	0	0.107	0	0	0	0	0.124	0	0
GER	0	0	0	0.181	0	0	0	0	0	0	0	0.136	0.111	0	0	0
ZNF664	0	0	0	0.315	0	0	0	0	0	0	0	0.113	0	0	0	0
METTL21B	0	0.185	0	0	0	0	0	0	0.109	0	0.134	0	0	0	0	0
FRA16B	0.126	0	0	0	0	0	0	0	0	0	0.2	0	0	0	0.101	0
GFPT1	0	0	0	0	0	0	0	0	0	0	0	0	0	0	0	0.427
SLC26A4	0	0	0	0	0	0	0	0	0	0	0	0.252	0.175	0	0	0
LOC619409	0	0	0	0.315	0	0	0	0	0	0.112	0	0	0	0	0	0
HTR3D	0	0	0	0	0	0	0	0	0	0	0	0	0	0	0.277	0.149
ADHD4	0	0	0.149	0.113	0	0	0	0	0	0	0	0	0.164	0	0	0
NELL2	0	0	0.123	0	0	0.171	0	0.132	0	0	0	0	0	0	0	0
MYEF2	0	0	0	0	0	0	0	0	0	0	0.32	0	0	0	0.106	0
HEXIM1	0	0	0	0	0.306	0	0	0	0.12	0	0	0	0	0	0	0
KCNU1	0	0	0	0	0	0.148	0	0	0	0	0	0	0	0	0	0.277
DBX2	0	0.147	0	0	0	0	0	0	0	0	0	0	0	0	0.278	0
HEPH	0	0	0	0	0	0	0	0	0	0.165	0	0	0	0.26	0	0
RIMS1	0	0	0.123	0	0	0.159	0	0	0	0	0	0	0.143	0	0	0
MAGEL2	0.163	0	0.108	0	0	0	0	0	0	0	0	0	0.154	0	0	0
TMEM37	0	0	0	0	0	0.177	0.13	0	0	0	0	0	0	0	0	0.118

COX11	0	0.295	0	0	0	0	0	0	0	0	0	0	0	0.13	0	0
SDTY1	0	0	0	0.247	0	0	0	0	0	0	0	0.178	0	0	0	0
TRIM37	0	0	0	0.299	0	0	0	0	0	0	0	0.126	0	0	0	0
FAM114A1	0	0	0	0	0.158	0.151	0	0.116	0	0	0	0	0	0	0	0
CLPB	0	0	0	0	0	0	0	0	0	0	0.323	0	0	0	0.101	0
USP40	0	0	0	0	0.156	0	0	0	0.123	0	0	0	0	0.145	0	0
TIMM13	0	0	0	0	0	0	0.131	0	0	0	0	0.112	0	0.181	0	0
GJB6	0	0	0.128	0	0	0	0	0	0	0	0	0.187	0.109	0	0	0
GJC2	0	0.132	0	0.178	0	0	0	0	0	0	0	0.114	0	0	0	0
ANK3	0	0	0	0.235	0	0.189	0	0	0	0	0	0	0	0	0	0
SUMF1	0	0	0	0	0	0	0	0	0	0	0.195	0	0.107	0.121	0	0
KIF21B	0	0.106	0	0.178	0	0	0	0	0	0	0	0.139	0	0	0	0
CCDC64	0	0.108	0	0.104	0	0	0	0	0	0	0.211	0	0	0	0	0
SMN1	0	0.105	0	0	0	0	0	0	0.16	0.157	0	0	0	0	0	0
NINJ1	0	0.105	0	0.177	0	0	0	0	0	0	0	0.14	0	0	0	0
PCBP2P1	0.422	0	0	0	0	0	0	0	0	0	0	0	0	0	0	0
ADHD1	0	0	0.124	0	0	0	0	0	0	0	0	0	0.128	0	0	0.169
KCNK16	0	0	0	0	0	0.236	0	0	0	0	0	0	0	0	0	0.185
KCNE4	0	0	0	0	0	0	0	0	0	0	0	0	0	0	0	0.421
SNAP29	0	0	0	0.237	0	0	0	0	0	0	0	0	0	0	0.184	0
HSN1B	0	0.105	0	0.101	0	0	0	0	0	0.112	0	0.103	0	0	0	0
CCDC34	0	0	0	0	0	0.295	0	0	0	0.126	0	0	0	0	0	0
THAP3	0	0	0	0.271	0	0	0	0	0	0	0	0	0	0	0	0.149
DZANK1	0.151	0	0	0	0	0	0	0	0	0	0	0	0	0	0.269	0
KALRN	0	0	0.122	0	0	0	0	0	0	0	0.153	0	0.145	0	0	0
KTCN3	0.17	0	0	0.106	0	0	0	0	0	0	0	0.144	0	0	0	0
FHQTL	0	0	0	0	0	0	0	0	0	0	0	0	0	0	0	0.42
DFFB	0	0	0	0	0	0	0	0	0	0	0.109	0	0	0.194	0.117	0
CPNE8	0	0	0	0	0	0	0	0	0	0	0	0	0	0	0.42	0
CLIC5	0	0	0	0	0	0.257	0	0	0	0	0	0	0	0	0	0.162
MYO6	0	0	0	0	0	0	0	0	0	0.231	0	0.188	0	0	0	0
DFNB32	0	0	0	0.281	0	0	0	0	0	0	0	0.138	0	0	0	0
GLDN	0	0	0	0.229	0	0	0	0	0	0	0	0.19	0	0	0	0
INTU	0	0	0	0	0	0	0	0	0	0	0	0	0	0	0	0.419
MMADHC	0.419	0	0	0	0	0	0	0	0	0	0	0	0	0	0	0
AGBL1	0	0	0	0	0	0	0	0	0	0.117	0	0	0	0.109	0	0.192
SLC30A5	0	0	0	0	0.165	0	0	0	0.102	0	0	0	0	0.151	0	0
KLC4	0	0.111	0	0.161	0	0	0	0	0	0	0	0.146	0	0	0	0
ZNF750	0	0	0	0.235	0	0	0	0	0	0	0	0.183	0	0	0	0
SREK1	0	0	0	0	0.174	0	0	0	0.244	0	0	0	0	0	0	0
DBH-AS1	0	0.146	0	0	0	0.155	0	0.117	0	0	0	0	0	0	0	0

DSCAML1	0.108	0	0	0	0	0.192	0	0.118	0	0	0	0	0	0	0	0
C8B	0	0	0	0	0	0	0.137	0	0	0	0	0	0	0	0	0.28
OMS	0	0	0.128	0	0	0	0	0	0	0	0	0.133	0.156	0	0	0
MCEE	0.116	0	0	0	0	0	0.133	0	0	0	0	0	0.168	0	0	0
FLG2	0	0	0	0	0	0	0	0	0	0	0.305	0	0	0	0.112	0
NAP1L3	0	0	0	0	0	0	0	0	0	0	0	0	0	0	0.417	0
STX1A	0	0	0	0	0	0.187	0	0	0	0	0.127	0	0	0.103	0	0
UVRAG	0	0	0	0	0	0	0	0	0	0	0.298	0	0	0.119	0	0
SLC12A5	0	0	0	0	0	0.155	0.156	0.106	0	0	0	0	0	0	0	0
ARHGAP27	0	0	0	0	0.112	0	0	0	0.117	0	0	0	0	0	0.187	0
PSAP	0	0	0	0	0.162	0	0	0	0.13	0	0	0	0	0.124	0	0
NGFR	0	0.182	0	0	0	0	0	0	0	0.234	0	0	0	0	0	0
DUXB	0	0	0	0.189	0	0	0	0	0	0	0.114	0	0	0	0.113	0
SNX33	0	0	0	0	0.121	0	0	0	0.144	0	0	0	0	0.15	0	0
ODF3L2	0	0	0.111	0	0	0.163	0	0	0	0	0	0	0.141	0	0	0
LRRC8E	0	0	0	0	0	0	0	0	0	0	0.237	0	0	0	0.178	0
LARGE	0	0	0	0.206	0	0	0	0	0	0.104	0	0.105	0	0	0	0
NMSR	0	0.106	0	0.179	0	0	0	0	0	0	0	0.13	0	0	0	0
FRTS	0	0	0	0	0	0	0	0	0	0	0.415	0	0	0	0	0
SLC25A22	0	0	0	0	0	0.155	0.158	0.102	0	0	0	0	0	0	0	0
CAPN6	0	0.146	0	0	0	0	0	0	0	0	0.168	0	0	0	0.1	0
NSUN5	0	0	0.123	0	0	0	0.133	0	0	0	0	0	0.158	0	0	0
IGSF9B	0	0	0.137	0	0	0	0	0	0	0	0	0	0.277	0	0	0
KCNK3	0	0.105	0	0	0	0.208	0	0.101	0	0	0	0	0	0	0	0
KCNH6	0	0	0	0.279	0	0.135	0	0	0	0	0	0	0	0	0	0
SIRT1	0	0	0	0	0	0	0	0	0.146	0	0	0	0	0.144	0	0.124
SERPINA2	0	0	0	0	0.286	0	0	0	0.128	0	0	0	0	0	0	0
NNO3	0	0	0	0.164	0	0	0	0	0	0	0	0.249	0	0	0	0
DRGX	0	0.125	0	0.178	0	0	0	0	0	0	0	0.11	0	0	0	0
HCRT	0	0.111	0	0	0.177	0	0	0	0	0	0.125	0	0	0	0	0
ZNF385D	0	0	0	0	0	0.184	0.119	0.11	0	0	0	0	0	0	0	0
DBA2	0	0	0	0	0	0	0	0	0	0	0	0.159	0	0	0	0.253
DLGAP1	0	0	0	0.243	0	0	0	0	0	0	0	0	0	0	0	0.169
NUCKS1	0	0	0	0	0.118	0	0	0	0.13	0	0	0	0	0	0.164	0
ASTN2	0	0	0.123	0	0	0.136	0	0	0	0	0	0	0.153	0	0	0
OR3A1	0	0	0	0	0	0	0	0	0	0	0	0	0	0	0.412	0
RPL24	0	0	0	0.186	0	0	0	0	0	0.103	0	0.123	0	0	0	0
NAV3	0	0	0	0.286	0	0	0	0	0	0	0	0.126	0	0	0	0
TMCC1	0	0	0	0.287	0	0	0	0	0	0	0	0.125	0	0	0	0
NOS2	0	0	0	0	0.286	0	0	0	0.126	0	0	0	0	0	0	0
CASD1	0	0	0	0.266	0	0	0	0	0	0	0	0	0	0	0	0.145

CASP4	0	0.154	0	0	0	0	0	0	0	0	0.156	0	0	0.101	0	0
DRD5P2	0	0	0	0	0	0	0	0	0	0	0.108	0	0	0	0	0.303
NDRG2	0.113	0	0	0.175	0	0	0	0	0.123	0	0	0	0	0	0	0
FBXO2	0	0	0	0	0.136	0	0	0	0.124	0	0	0	0	0.15	0	0
DYNC1I1	0	0.13	0	0.101	0	0	0	0	0	0	0.179	0	0	0	0	0
HSPB6	0	0.137	0	0.101	0	0	0	0	0	0	0.172	0	0	0	0	0
CST6	0	0	0	0	0	0	0	0	0	0	0	0	0	0	0.41	0
TMEM151B	0	0	0	0	0	0	0	0	0	0	0	0	0	0	0.41	0
DUH	0.111	0	0	0.118	0	0	0	0	0	0	0	0.181	0	0	0	0
MATN4	0	0	0	0.41	0	0	0	0	0	0	0	0	0	0	0	0
SIRT2	0	0	0	0	0	0	0	0	0	0	0.308	0	0	0.101	0	0
ABHD12	0	0	0	0	0	0	0	0	0	0	0.279	0.13	0	0	0	0
SPINT3	0	0	0	0	0.26	0	0	0	0.149	0	0	0	0	0	0	0
DDN	0	0.136	0.12	0	0	0.153	0	0	0	0	0	0	0	0	0	0
MGP	0	0	0	0	0	0	0	0	0	0	0	0	0	0	0.409	0
CNTD1	0	0	0	0	0	0	0	0	0.129	0	0.164	0	0	0.115	0	0
CAMK4	0	0	0.132	0	0	0	0	0	0	0	0	0	0.276	0	0	0
HNRNPDP	0.279	0	0	0	0	0	0	0	0	0	0.129	0	0	0	0	0
PRODH	0	0	0.109	0	0	0	0	0	0	0	0	0	0.178	0	0	0.121
GNS	0	0	0	0	0	0	0	0	0	0	0	0	0.288	0.12	0	0
HTR3C	0	0	0	0	0	0	0	0	0	0	0	0	0	0	0.224	0.183
PIGAP1	0	0	0	0	0	0.158	0.104	0	0	0	0	0	0	0	0	0.145
C18ORF25	0.11	0	0.128	0	0	0	0	0	0	0	0	0	0.169	0	0	0
TMEM200C	0.144	0	0.125	0	0	0	0	0	0	0	0	0	0.138	0	0	0
ALG8	0	0	0	0	0	0	0.103	0	0	0	0	0	0.191	0	0	0.113
HOTTIP	0	0	0	0	0	0	0	0	0	0	0	0	0	0	0.407	0
PAPLN	0	0	0	0	0	0	0	0	0	0	0	0	0	0	0.407	0
RAB7A	0	0.12	0	0.179	0	0	0	0	0	0	0	0	0	0.108	0	0
NOTCH4	0	0	0	0	0.286	0	0	0	0.121	0	0	0	0	0	0	0
BEGAIN	0	0	0	0.407	0	0	0	0	0	0	0	0	0	0	0	0
EVC	0	0	0	0	0	0	0	0	0	0	0.132	0	0.274	0	0	0
ADCY1	0	0	0.131	0	0	0	0	0	0	0.149	0	0	0.126	0	0	0
DLG2-AS1	0	0	0.132	0	0	0	0	0.132	0	0	0	0	0.142	0	0	0
LNCR1	0	0	0	0.14	0	0	0	0	0.106	0	0	0.16	0	0	0	0
EIF2AK2	0.153	0.121	0	0	0	0	0	0	0	0	0.132	0	0	0	0	0
SLC2A1	0	0	0	0	0	0.178	0.107	0	0	0	0	0	0	0	0	0.121
C1ORF63	0	0	0	0	0	0	0	0	0	0	0	0	0	0	0.172	0.233
BRSK2	0	0	0	0	0	0.133	0	0.113	0	0	0	0	0	0	0	0.159
NIPSNAP1	0	0	0	0	0.119	0	0	0.142	0	0	0	0	0	0.144	0	0
BBS7	0	0	0.129	0	0	0	0	0	0	0	0	0.113	0.163	0	0	0
DYX6	0	0	0.131	0	0	0	0	0	0	0.112	0	0	0.162	0	0	0

TRPV3	0	0	0	0.193	0	0	0	0	0	0	0	0.212	0	0	0	0
LOC647859	0	0.125	0	0.101	0	0	0	0	0.179	0	0	0	0	0	0	0
PLA2G6	0	0	0	0	0.177	0	0	0	0.104	0	0	0	0	0.124	0	0
POU4F2	0	0.144	0	0	0	0	0	0	0	0.154	0	0.107	0	0	0	0
DAP3P1	0	0	0	0.247	0	0	0	0	0	0	0	0	0	0	0.157	0
TRICY1	0	0	0	0.234	0	0	0	0	0	0	0	0.17	0	0	0	0
GLIPR1L1	0	0	0	0	0	0	0	0	0	0	0	0	0	0	0	0.403
PCM1	0	0	0.127	0	0	0	0	0	0	0	0.162	0	0	0	0.114	0
MLIP	0	0	0	0	0	0	0	0	0	0	0	0	0	0	0.403	0
KIF3C	0	0.113	0	0.166	0	0	0	0	0	0	0	0.124	0	0	0	0
COL28A1	0	0	0	0.211	0	0	0	0	0	0	0	0.192	0	0	0	0
EXTL1	0	0	0	0.197	0	0	0	0	0	0	0	0.206	0	0	0	0
FAM160B2	0	0	0	0.161	0	0	0	0	0	0	0	0.242	0	0	0	0
SNX7	0	0	0	0.232	0	0	0	0	0	0	0	0.171	0	0	0	0
PAPA4	0.242	0	0	0.161	0	0	0	0	0	0	0	0	0	0	0	0
CTSZ	0	0	0	0	0	0	0	0	0	0	0.291	0	0	0	0.112	0
VLDLR	0.103	0	0	0	0.171	0	0	0	0.128	0	0	0	0	0	0	0
PHACTR2	0	0.125	0	0	0.169	0	0	0.108	0	0	0	0	0	0	0	0
RERE	0	0	0	0	0	0	0	0	0	0	0.239	0	0	0	0.162	0
CCPSO	0	0	0	0.272	0	0	0	0	0	0	0	0.129	0	0	0	0
SEPN1	0	0	0	0.26	0	0	0	0	0	0	0	0.141	0	0	0	0
PMS2CL	0.144	0	0	0	0	0	0	0	0	0	0	0.257	0	0	0	0
USP9X	0	0	0	0	0	0	0	0	0.202	0	0.199	0	0	0	0	0
TRND	0	0	0	0	0	0	0.116	0	0	0	0	0.175	0	0.11	0	0
C4ORF6	0	0	0.126	0	0	0	0	0	0	0	0	0	0.17	0	0.104	0
LHX1	0	0.144	0	0	0	0	0	0	0	0	0	0	0	0	0.256	0
BST1	0	0	0	0	0	0	0	0	0	0	0	0	0	0	0.4	0
IGHV4-4	0	0	0	0	0	0	0	0	0	0	0	0	0	0	0.4	0
ERI3	0	0	0	0	0	0	0.107	0	0	0	0.141	0	0	0.152	0	0
KIAA0415	0	0	0	0.278	0	0	0	0	0	0	0	0.122	0	0	0	0
SCG2	0	0.119	0	0	0.176	0	0	0	0.105	0	0	0	0	0	0	0
GEMIN2	0	0.284	0	0	0	0	0	0	0.116	0	0	0	0	0	0	0
LPHN3	0	0	0.11	0	0	0.163	0	0	0	0	0	0	0.126	0	0	0
CLCN7	0	0	0	0	0	0.187	0	0.104	0	0	0	0	0	0.108	0	0
ZIC4	0	0	0	0	0	0.183	0.111	0.105	0	0	0	0	0	0	0	0
GABARAPL1	0	0	0	0	0	0	0	0	0.254	0	0	0	0	0.144	0	0
SOX21	0.398	0	0	0	0	0	0	0	0	0	0	0	0	0	0	0
VDAC3	0.274	0	0	0	0	0	0	0	0	0	0	0	0	0	0	0.123
PSMD2	0	0	0	0	0	0	0	0	0	0	0.265	0	0	0.132	0	0
LRRIQ3	0	0	0	0	0	0	0	0	0	0	0	0	0	0	0.224	0.172
IL8	0	0	0	0	0	0	0	0	0	0	0	0	0	0	0	0.396

MIR328	0	0	0	0	0	0	0	0	0	0	0	0	0	0	0	0.396
SLC18A3	0	0	0.13	0	0.153	0	0	0	0	0	0	0	0.113	0	0	0
CCT5	0	0	0	0.244	0	0	0	0	0	0	0	0.152	0	0	0	0
DYNC1I2	0	0.104	0	0.101	0	0	0	0	0	0	0.191	0	0	0	0	0
DFNB66	0	0	0	0.132	0	0	0	0	0	0	0	0.11	0.153	0	0	0
DHCR7	0.107	0	0.134	0	0	0	0	0	0	0	0	0	0.154	0	0	0
VCX3A	0	0	0.141	0	0	0	0	0	0	0	0	0	0.254	0	0	0
PMP2	0	0	0	0.241	0	0	0	0	0	0	0	0.154	0	0	0	0
LTBP2	0	0	0	0	0	0	0	0	0	0	0	0	0	0	0.394	0
DPYSL4	0.156	0	0	0	0	0	0	0	0	0	0.102	0	0	0.136	0	0
CHRM1	0	0	0.13	0	0.162	0	0	0	0	0	0	0	0.102	0	0	0
SLC7A10	0	0	0	0	0	0.13	0.106	0.158	0	0	0	0	0	0	0	0
TAAR6	0	0	0.123	0	0	0	0	0	0	0	0	0	0.1	0	0	0.17
PIGA	0	0	0	0	0	0	0	0	0	0	0	0	0	0	0	0.393
HYDIN2	0	0	0	0.209	0	0	0	0	0	0	0	0	0	0	0.184	0
C7ORF16	0	0	0	0	0	0	0	0	0	0.177	0	0	0	0.216	0	0
DYM	0	0	0.123	0	0	0.164	0	0	0	0	0	0	0.106	0	0	0
C4ORF10	0	0	0	0	0	0	0	0	0	0	0.172	0	0	0.104	0.117	0
LCE2B	0.147	0	0	0	0	0	0	0	0	0	0	0	0	0	0.245	0
DAOA-AS1	0	0	0.132	0	0	0	0	0	0.128	0	0	0	0.132	0	0	0
GSC2	0	0	0	0	0	0	0	0.214	0	0	0	0	0.178	0	0	0
GLC1B	0	0	0	0.226	0	0	0	0	0	0	0	0.166	0	0	0	0
MALL	0	0	0	0.261	0	0	0	0	0	0	0	0.131	0	0	0	0
ZFHX2	0	0	0	0	0	0	0	0.392	0	0	0	0	0	0	0	0
SLEB4	0	0.124	0	0	0	0	0	0	0	0	0	0	0	0	0.267	0
FEV	0.122	0	0	0	0	0	0	0	0	0	0	0	0	0	0.269	0
TRNAL1	0	0	0	0	0	0	0.121	0	0	0	0	0.147	0	0.123	0	0
YWHAE	0	0.108	0	0	0	0	0	0	0	0	0.145	0	0	0.138	0	0
DFNB83	0	0	0.109	0	0	0	0	0	0	0	0	0.141	0.141	0	0	0
CMD1Q	0	0	0	0.211	0	0	0	0	0	0	0	0.18	0	0	0	0
PATE2	0	0	0	0	0	0.272	0	0	0	0	0	0	0	0	0	0.117
PPP4R4	0	0	0	0	0	0	0	0	0	0	0.209	0	0	0	0.18	0
PRPS1	0	0	0	0.279	0	0	0	0	0	0	0	0.11	0	0	0	0
TMEM35	0	0	0	0.204	0	0	0	0	0	0	0	0.185	0	0	0	0
SLC16A7	0	0	0	0	0	0.179	0.107	0	0	0	0	0	0	0	0	0.103
WDFY4	0	0.15	0	0	0	0	0	0	0	0	0	0	0	0	0.238	0
CCNT2	0	0	0	0	0.207	0	0	0	0.181	0	0	0	0	0	0	0
BTBD10	0.147	0.112	0	0	0	0	0	0	0.129	0	0	0	0	0	0	0
CNTN1	0	0	0	0.246	0	0.142	0	0	0	0	0	0	0	0	0	0
ZDHHC21	0	0	0	0	0	0.133	0.112	0	0	0	0	0	0	0	0	0.142
SMCR7L	0	0	0	0.242	0	0	0	0	0	0	0	0	0	0.145	0	0

FAM120C	0	0	0.13	0	0	0.147	0	0	0	0	0	0	0.11	0	0	0
GJE1	0	0	0	0.167	0	0	0	0	0	0	0	0.22	0	0	0	0
OGDH	0	0	0	0	0	0	0.116	0	0	0	0.143	0	0	0.127	0	0
AVSD1	0	0	0.131	0	0	0	0	0	0	0	0	0.14	0.115	0	0	0
DYX9	0	0	0.132	0	0	0	0	0	0.133	0	0	0	0.121	0	0	0
FARP1	0	0.101	0	0	0.15	0	0	0	0.135	0	0	0	0	0	0	0
MSX2P1	0	0	0	0	0	0	0	0	0	0	0.208	0	0	0	0.177	0
CNTNAP1	0	0.119	0	0.161	0	0	0	0	0	0	0	0.105	0	0	0	0
GJB4	0	0	0	0.184	0	0	0	0	0	0	0	0.201	0	0	0	0
RPS19P1	0	0	0	0	0	0	0	0	0	0	0	0.162	0	0	0	0.222
HLA-B	0	0	0	0	0	0	0	0	0	0	0	0	0	0	0	0.384
ZNF503	0	0	0	0	0	0	0	0	0	0	0.228	0	0	0	0.156	0
RNR2	0	0	0	0	0	0	0.112	0	0	0	0	0.16	0	0.112	0	0
PHF8	0	0	0.129	0	0	0.145	0	0	0	0	0	0	0.11	0	0	0
FAM134A	0	0	0	0.262	0	0	0	0	0	0	0	0.122	0	0	0	0
GLC1J	0	0	0	0.186	0	0	0	0	0	0	0	0.198	0	0	0	0
APEX1	0	0.184	0	0	0	0	0	0	0	0	0.2	0	0	0	0	0
SRSF11	0	0	0	0	0.164	0	0	0	0.22	0	0	0	0	0	0	0
SIK2	0.384	0	0	0	0	0	0	0	0	0	0	0	0	0	0	0
PPP2R2B	0	0	0	0	0.114	0	0	0	0	0	0.147	0	0	0.122	0	0
NAB1	0	0	0	0.272	0	0	0	0	0	0	0	0.111	0	0	0	0
ATF3	0	0.136	0	0.115	0	0	0	0	0	0	0.132	0	0	0	0	0
PHLPP1	0.126	0	0	0	0	0	0	0	0	0	0.156	0	0	0	0.1	0
MPHOSPH9	0	0.121	0.106	0	0	0	0	0	0	0	0	0	0	0	0.155	0
DOC2GP	0.113	0	0	0	0	0	0	0	0	0	0	0	0	0	0.269	0
RNR1	0	0	0.128	0	0	0	0	0	0	0	0	0.128	0.126	0	0	0
D2HGDH	0	0	0	0	0	0.13	0.103	0	0	0	0	0	0.149	0	0	0
FLRT3	0	0	0	0.124	0	0	0	0	0	0	0	0.258	0	0	0	0
HSF4	0	0	0	0.219	0	0	0	0	0	0	0	0.163	0	0	0	0
BCAN	0	0.219	0	0	0	0	0	0	0	0.163	0	0	0	0	0	0
CRB2	0.125	0	0	0	0.136	0	0	0	0.121	0	0	0	0	0	0	0
RAB5A	0	0.276	0	0.106	0	0	0	0	0	0	0	0	0	0	0	0
COPD20	0	0	0	0.271	0	0	0	0	0	0	0	0	0	0	0	0.109
PIGP	0.251	0	0	0	0	0	0	0	0	0	0	0	0	0	0	0.129
SYNJ1	0	0	0.126	0	0	0	0	0	0	0	0.153	0	0	0	0.101	0
CAPN2	0	0.135	0	0	0	0	0	0	0	0	0.145	0	0	0	0.1	0
TSPO	0	0	0	0	0	0	0.137	0	0	0.106	0	0	0	0.137	0	0
ALG9	0	0	0.109	0	0	0.158	0	0	0	0	0	0	0.113	0	0	0
NAALAD2	0	0	0	0	0	0	0	0.195	0	0	0.185	0	0	0	0	0
LOC646652	0	0.125	0	0.101	0	0	0	0	0.154	0	0	0	0	0	0	0
LOC642502	0	0	0	0	0	0	0	0	0	0	0	0	0	0	0.379	0

SKI	0	0	0	0	0	0	0	0	0	0	0	0	0	0	0.379	0
GJA9	0	0	0	0.15	0	0	0	0	0	0	0	0.229	0	0	0	0
IGHV3-23	0	0	0	0.379	0	0	0	0	0	0	0	0	0	0	0	0
HTR2C	0	0	0	0	0	0	0	0	0	0	0	0	0	0	0	0.378
RRDX	0	0	0.132	0	0	0	0.137	0	0	0	0	0	0.109	0	0	0
TBCC	0	0.206	0	0	0	0	0	0	0	0.172	0	0	0	0	0	0
EMR2	0	0	0	0	0	0	0	0	0	0	0	0	0	0	0	0.377
EMR3	0	0	0	0	0	0	0	0	0	0	0	0	0	0	0	0.377
CTNS	0	0	0.126	0	0	0	0	0	0	0	0	0	0.132	0.119	0	0
CAST	0	0.106	0	0	0	0	0	0	0	0	0.146	0	0	0.125	0	0
PRSS46	0	0	0.128	0	0	0	0	0	0	0	0	0.123	0.126	0	0	0
NOMO3	0	0	0	0	0.109	0	0	0	0.124	0	0	0	0	0	0	0.143
CACNG1	0	0	0	0	0	0.254	0	0	0	0	0	0	0	0	0	0.122
DVL3	0.109	0	0	0	0	0	0	0	0	0	0	0	0	0	0	0.267
SLC25A4	0	0	0	0	0	0	0.114	0	0	0	0	0	0	0.146	0.116	0
NKPD1	0	0	0	0	0	0	0.101	0	0	0	0.173	0	0	0	0.102	0
FBXO46	0	0	0	0	0	0	0	0	0	0	0	0	0	0	0.376	0
TTLL6	0	0	0	0	0	0	0	0	0	0	0.263	0	0	0.113	0	0
ADHD3	0	0	0.153	0.103	0	0	0	0	0	0	0	0	0.12	0	0	0
CHN1	0	0	0	0.152	0	0	0	0	0	0	0	0.224	0	0	0	0
GJA8	0	0	0	0.147	0	0	0	0	0	0	0	0.229	0	0	0	0
TAF11	0.104	0	0	0	0	0	0	0	0	0	0.272	0	0	0	0	0
FRA6E	0	0	0	0	0	0	0	0	0.177	0	0	0	0	0	0	0.198
LYRM4	0	0	0	0	0	0	0.107	0	0	0	0	0	0	0.152	0.116	0
DCTN3	0	0.12	0	0	0	0	0	0	0	0	0.155	0	0	0	0.1	0
RCM2	0	0	0	0.243	0	0	0	0	0	0	0	0.132	0	0	0	0
RAB24	0	0	0	0.21	0	0	0	0	0	0	0	0	0	0	0.164	0
TRAJ10	0	0.151	0	0	0	0	0	0	0	0	0	0	0	0	0.223	0
MYAS1	0	0.205	0	0.169	0	0	0	0	0	0	0	0	0	0	0	0
PPH2	0	0	0	0	0	0	0	0	0	0	0	0.102	0.126	0	0	0.145
TRBV5-6	0	0	0	0	0	0	0	0	0	0	0	0	0	0	0.373	0
WBSCR22	0	0	0.131	0	0	0	0.133	0	0	0	0	0	0.109	0	0	0
CDHR4	0	0	0	0	0	0.13	0	0.111	0	0	0.132	0	0	0	0	0
EWSR1	0	0.203	0	0	0	0	0	0	0.17	0	0	0	0	0	0	0
MTMR4	0	0	0	0.373	0	0	0	0	0	0	0	0	0	0	0	0
IGHV1-2	0	0	0	0	0	0	0	0	0	0	0	0	0	0	0	0.372
TBCA	0	0.124	0	0	0	0	0	0	0	0	0	0	0	0	0.248	0
PPBPL1	0.111	0	0	0	0	0	0	0	0	0	0	0	0	0	0.261	0
TRBV15	0	0	0	0	0	0	0	0	0	0	0	0	0	0	0.372	0
NTRK1	0	0	0	0	0	0	0	0	0	0	0.144	0.105	0	0.123	0	0
NDUFS7	0	0	0	0	0	0	0.15	0	0	0	0	0.109	0	0.113	0	0

CALB1	0	0	0	0	0	0	0	0	0	0.108	0.125	0	0	0.139	0	0
MYMY2	0	0.106	0	0	0	0	0	0	0.107	0	0	0.159	0	0	0	0
COTL1P1	0	0	0	0.147	0	0	0	0	0	0	0	0.225	0	0	0	0
MTMR10	0	0	0	0.372	0	0	0	0	0	0	0	0	0	0	0	0
DNAJB6	0	0	0	0	0	0	0	0	0	0	0.256	0	0	0	0.115	0
TMEM165	0.148	0	0	0	0	0	0	0	0	0	0	0	0	0	0.223	0
TTC8	0	0	0	0	0	0	0	0	0	0.118	0	0.119	0.134	0	0	0
DCDC2	0	0	0.124	0	0	0.141	0	0	0	0	0	0	0.105	0	0	0
ZNF41	0	0	0.109	0	0	0.159	0	0	0	0	0	0	0.102	0	0	0
CLRN1-AS1	0	0	0	0	0	0	0	0	0	0.191	0	0.179	0	0	0	0
RPL9P2	0	0	0	0.242	0	0	0	0	0	0	0	0.128	0	0	0	0
RIMS3	0	0	0	0	0	0.136	0	0	0	0	0	0	0	0	0	0.233
C11ORF1	0	0	0.126	0	0	0	0	0	0	0	0	0	0.129	0	0.114	0
TGM7	0	0	0	0	0	0	0	0	0	0	0.202	0	0	0	0.167	0
IDUA	0	0	0.126	0	0	0	0	0	0	0	0	0	0.138	0.105	0	0
ACAD11	0	0	0	0	0	0	0	0	0	0	0	0.261	0	0.108	0	0
NSUN5P1	0	0	0.131	0	0	0	0.133	0	0	0	0	0	0.105	0	0	0
HMU	0	0	0	0.223	0	0	0	0	0	0	0	0.146	0	0	0	0
TATDN1	0	0	0	0	0.121	0	0	0.139	0.109	0	0	0	0	0	0	0
C2ORF67	0	0	0	0	0	0	0	0	0	0	0	0	0	0	0.368	0
C5ORF54	0	0	0	0	0	0	0	0	0	0	0	0	0	0	0.368	0
CABIN1	0	0	0	0	0	0	0	0	0	0	0	0	0	0	0.368	0
CALM2P1	0	0	0	0	0	0	0	0	0	0	0	0	0	0	0.368	0
CALML4	0	0	0	0	0	0	0	0	0	0	0	0	0	0	0.368	0
CCDC140	0	0	0	0	0	0	0	0	0	0	0	0	0	0	0.368	0
FLG	0	0	0	0	0	0	0	0	0	0	0	0	0	0	0.368	0
FNDC4	0	0	0	0	0	0	0	0	0	0	0	0	0	0	0.368	0
TIMM9	0	0	0	0	0	0	0.137	0	0	0	0	0.121	0	0.11	0	0
BBS12	0	0	0	0	0	0	0	0	0	0	0	0.196	0.172	0	0	0
PMM2	0	0	0.132	0	0	0	0.132	0	0	0	0	0	0.104	0	0	0
MUT	0.128	0	0	0	0	0	0	0	0	0	0	0	0.24	0	0	0
OMG	0	0.142	0	0.107	0	0	0	0	0	0	0	0.119	0	0	0	0
GLC1K	0	0	0	0.199	0	0	0	0	0	0	0	0.169	0	0	0	0
WNK1	0	0	0	0.259	0	0	0	0	0	0	0	0.109	0	0	0	0
LOC728400	0	0	0	0	0	0	0	0	0	0	0.151	0	0	0	0.216	0
GAD2	0	0	0	0	0	0.129	0.132	0	0	0	0	0	0	0	0.106	0
DGCR14	0	0	0.122	0	0	0	0	0.111	0	0	0	0	0.134	0	0	0
AVIL	0	0.105	0	0.16	0	0	0	0	0	0	0	0.102	0	0	0	0
DFNA59	0	0	0	0.24	0	0	0	0	0	0	0	0.127	0	0	0	0
DCTN2	0	0.194	0	0	0	0	0	0	0	0	0.173	0	0	0	0	0
NEIL2	0	0	0	0	0	0	0	0	0	0	0.367	0	0	0	0	0

IQCB1	0	0	0	0	0	0	0	0	0	0.118	0	0	0	0	0	0.248
HRNR	0	0	0	0	0	0	0	0	0	0	0	0	0	0	0.366	0
MT1A	0	0.235	0	0	0	0	0	0	0	0	0	0	0	0.131	0	0
FKTN	0	0	0	0.143	0	0	0	0	0	0.118	0	0	0.105	0	0	0
GJB3	0	0	0	0.176	0	0	0	0	0	0	0	0.19	0	0	0	0
CANDN1	0.129	0.123	0	0.114	0	0	0	0	0	0	0	0	0	0	0	0
TIMM8B	0	0	0	0	0	0	0.12	0	0	0	0	0.125	0	0.12	0	0
NES	0	0.112	0	0	0	0	0	0	0	0.253	0	0	0	0	0	0
EFHD2	0.101	0	0	0	0.149	0	0	0	0.115	0	0	0	0	0	0	0
NPPB	0	0	0	0	0.23	0	0	0	0	0	0	0	0	0	0	0.134
HOXB9	0	0	0	0	0	0	0	0	0	0	0.249	0	0	0	0.115	0
NMNAT3	0	0.107	0	0	0	0	0	0	0	0	0.151	0	0	0.106	0	0
SZT2	0	0.107	0	0	0	0	0	0	0	0	0.138	0	0	0.119	0	0
IKBKAP	0	0	0	0.24	0	0	0	0	0	0	0	0.124	0	0	0	0
NTM	0	0	0.118	0	0	0.144	0	0.102	0	0	0	0	0	0	0	0
PRICKLE1	0	0.115	0	0	0	0	0	0	0	0	0.148	0	0	0	0.1	0
CNNM4	0	0.103	0	0	0	0	0	0	0	0.133	0	0.127	0	0	0	0
SLITRK6	0	0	0.109	0	0	0	0	0	0	0	0	0.254	0	0	0	0
GLM1	0	0.17	0	0	0	0.193	0	0	0	0	0	0	0	0	0	0
C15ORF63	0	0	0	0	0	0	0	0	0	0	0.14	0	0	0.116	0.106	0
SYT10	0	0	0	0	0	0.179	0	0	0	0	0	0	0	0	0.183	0
CP20	0	0	0	0	0	0	0	0	0	0	0	0	0	0	0.362	0
RNANC	0	0	0	0	0	0	0	0	0	0.136	0	0.226	0	0	0	0
RELB	0	0	0	0	0.216	0	0	0	0.146	0	0	0	0	0	0	0
HTR3E	0	0	0	0	0	0	0	0	0	0	0	0	0	0	0.129	0.232
LOC100132247	0	0	0	0	0	0	0	0	0	0	0.257	0	0	0	0.104	0
SPRR4	0.13	0	0	0	0	0	0	0	0	0	0	0	0	0	0.231	0
SPRR2A	0	0	0	0	0	0	0	0	0	0	0	0	0	0	0.361	0
CHD7	0	0	0.129	0	0	0	0	0	0	0	0	0.126	0.106	0	0	0
PHOBS	0	0	0.109	0	0	0	0	0	0	0.148	0	0	0.104	0	0	0
MPZ	0	0.106	0	0.106	0	0	0	0	0	0	0	0.149	0	0	0	0
YPEL1	0	0	0	0.23	0	0	0	0	0	0	0	0.131	0	0	0	0
CCDC64B	0	0.101	0	0.101	0	0	0	0	0	0	0.159	0	0	0	0	0
KLHL9	0	0	0	0.361	0	0	0	0	0	0	0	0	0	0	0	0
TGM1	0	0	0	0	0.131	0	0	0	0	0	0.116	0	0	0	0.113	0
C9ORF128	0	0	0	0	0	0	0.12	0	0	0	0	0.24	0	0	0	0
KIAA1279	0	0	0	0.23	0	0	0	0	0	0	0	0.13	0	0	0	0
LINGO4	0	0.106	0	0	0	0	0	0	0	0	0	0.254	0	0	0	0
HYALP	0.221	0.139	0	0	0	0	0	0	0	0	0	0	0	0	0	0
MACF1	0.36	0	0	0	0	0	0	0	0	0	0	0	0	0	0	0
INADL	0	0	0	0	0	0	0	0	0	0	0	0	0	0	0	0.359

MSTO1	0	0	0	0	0	0	0	0	0	0	0	0	0	0	0.359	0
GTF2IRD2	0	0	0.145	0.113	0	0	0	0	0	0	0	0	0.101	0	0	0
CCA1	0	0	0	0	0	0	0	0	0	0.181	0	0.178	0	0	0	0
AGMAT	0	0	0	0	0	0	0	0	0	0	0	0	0	0	0.358	0
FNTAP1	0	0	0	0	0	0	0	0	0	0	0	0	0	0	0.358	0
DFNB85	0	0	0.11	0	0	0	0	0	0	0	0	0.132	0.116	0	0	0
FBXL20	0	0	0.139	0	0	0	0	0	0	0	0	0	0.219	0	0	0
FOLH1	0.187	0	0	0	0.171	0	0	0	0	0	0	0	0	0	0	0
HNRNPA0	0	0	0	0	0	0	0	0	0	0	0.256	0	0	0	0.101	0
ADHD2	0	0	0.152	0.103	0	0	0	0	0	0	0	0	0.102	0	0	0
NDRG3	0	0.121	0	0.106	0	0	0	0	0	0	0	0.13	0	0	0	0
POM121L1P	0	0	0.11	0	0	0	0	0	0	0	0	0	0.133	0	0	0.113
CAPN13	0	0	0	0	0	0	0	0	0	0	0.241	0	0	0	0.115	0
LINC00271	0	0	0.137	0	0	0	0	0	0	0	0	0	0.219	0	0	0
CLRN2	0	0	0	0	0	0	0	0	0	0.168	0	0.188	0	0	0	0
ESPNP	0	0	0	0.198	0	0	0	0	0	0	0	0.158	0	0	0	0
RTN4RL1	0	0	0	0.242	0	0	0	0	0	0	0	0.114	0	0	0	0
RAB5B	0	0	0	0.356	0	0	0	0	0	0	0	0	0	0	0	0
ADAM10	0.153	0.203	0	0	0	0	0	0	0	0	0	0	0	0	0	0
DPY19L2P4	0	0	0	0	0	0	0	0	0	0	0.251	0	0	0	0.104	0
MMRN1	0	0	0	0	0	0	0	0	0	0	0	0	0	0	0.355	0
HSF2	0	0.202	0	0	0	0	0	0	0	0	0.153	0	0	0	0	0
SNX17	0.16	0	0	0	0.195	0	0	0	0	0	0	0	0	0	0	0
SLC25A38	0	0	0	0	0	0	0.108	0	0	0	0	0.123	0.123	0	0	0
NSUN5P2	0	0	0.132	0	0	0	0.111	0	0	0	0	0	0.111	0	0	0
GLC1L	0	0	0	0.25	0	0	0	0	0	0	0	0.104	0	0	0	0
APOF	0.175	0	0	0	0.179	0	0	0	0	0	0	0	0	0	0	0
COPD18	0	0	0	0.238	0	0	0	0	0	0	0	0	0	0	0	0.115
FXC1	0	0	0	0	0	0	0.109	0	0	0	0	0.127	0	0.117	0	0
AGBL4	0	0	0	0	0	0	0	0	0	0.132	0	0	0	0.221	0	0
VMP1	0	0	0	0	0	0	0	0	0.246	0	0	0	0	0.107	0	0
GJC3	0	0	0	0.209	0	0	0	0	0	0	0	0.144	0	0	0	0
MTRR	0.144	0	0	0	0	0	0	0	0	0	0.209	0	0	0	0	0
CEP112	0	0	0	0	0	0.188	0.165	0	0	0	0	0	0	0	0	0
CDRT1	0	0	0	0.214	0	0	0	0	0	0	0	0.138	0	0	0	0
KLK7	0	0	0	0	0.185	0	0	0	0.167	0	0	0	0	0	0	0
DYT10	0	0	0	0	0	0	0	0	0	0	0	0	0	0	0	0.351
CSN1S2BP	0	0	0	0	0	0	0	0	0	0	0	0	0	0	0.351	0
PPIC	0	0	0	0	0	0	0	0	0	0	0	0	0	0	0.351	0
ELMOD1	0	0	0	0.351	0	0	0	0	0	0	0	0	0	0	0	0
CNC	0	0	0	0	0	0	0	0	0	0	0	0.165	0	0	0	0.185

COPD17	0	0	0	0.223	0	0	0	0	0	0	0	0	0	0	0	0.127
NQO2	0	0	0	0	0	0	0	0	0	0	0	0	0	0	0	0.35
SLC45A1	0	0	0	0	0.105	0	0	0	0	0	0.143	0	0	0	0.102	0
TBX23P	0	0	0	0.243	0	0	0	0	0	0	0	0	0	0	0.107	0
COX6B1P2	0	0	0	0	0	0	0	0	0	0	0	0	0	0	0.35	0
MUC20	0	0	0	0	0	0	0	0	0	0	0	0	0	0	0.35	0
S100A11	0	0	0	0	0	0	0	0	0	0	0	0	0	0	0.35	0
RSS	0	0	0	0	0	0.13	0.113	0	0	0	0	0	0.107	0	0	0
GJD3	0	0	0	0.35	0	0	0	0	0	0	0	0	0	0	0	0
CACNB3	0	0	0	0	0	0.191	0	0	0	0	0	0	0	0	0	0.158
SOD3	0.101	0.128	0	0	0	0	0	0	0	0	0	0	0	0	0	0.12
C10ORF116	0	0	0	0	0	0	0	0	0	0	0	0	0	0	0.349	0
ELOVL2	0	0	0	0	0	0	0	0	0	0	0	0	0	0	0.349	0
MSL3P1	0	0	0	0	0	0	0	0	0	0	0	0	0	0	0.349	0
CISD2	0	0	0	0	0	0	0	0	0	0	0	0.121	0.126	0.102	0	0
NDRG1	0	0	0	0.231	0	0	0	0	0	0	0	0.118	0	0	0	0
MYMY3	0	0.181	0	0.168	0	0	0	0	0	0	0	0	0	0	0	0
DPEP3	0	0	0	0	0	0	0	0	0	0	0	0	0	0	0	0.348
JPH2	0	0	0	0	0	0	0	0	0	0	0	0	0	0	0	0.348
WDR44	0	0	0	0	0	0	0	0	0	0	0	0	0	0	0.348	0
MYO1D	0	0	0	0	0	0	0	0	0	0.12	0	0.228	0	0	0	0
EBF2	0	0.105	0	0.117	0	0	0	0	0	0	0	0.126	0	0	0	0
MIR133A2	0	0	0	0	0.184	0	0	0	0.164	0	0	0	0	0	0	0
GBD2	0.118	0	0	0	0	0	0	0	0.23	0	0	0	0	0	0	0
PRB4	0	0	0	0.348	0	0	0	0	0	0	0	0	0	0	0	0
SYT16	0.115	0	0	0	0	0	0	0	0	0	0	0	0	0	0.232	0
ANKRD33	0	0	0	0	0	0	0	0	0	0.162	0	0	0	0	0.185	0
RPTN	0.125	0	0	0	0	0	0	0	0	0	0	0	0	0	0.222	0
IBD18	0	0	0	0	0	0	0	0	0	0	0	0	0	0	0.347	0
CELF2	0	0	0	0	0.127	0	0	0	0.107	0	0	0	0	0.113	0	0
SCKL3	0	0	0	0	0	0	0	0	0	0	0	0.126	0.221	0	0	0
UBC	0	0	0	0	0	0	0	0	0.189	0	0.158	0	0	0	0	0
APAF1	0	0.172	0	0	0	0	0	0	0	0	0.175	0	0	0	0	0
OPRL1	0	0	0.129	0	0	0	0	0	0	0.218	0	0	0	0	0	0
VN1R5	0.347	0	0	0	0	0	0	0	0	0	0	0	0	0	0	0
DNAH2	0	0.137	0	0	0	0	0	0	0	0	0.105	0	0	0	0.104	0
SRGAP3	0	0	0.144	0	0	0	0	0	0	0	0	0	0.202	0	0	0
LMX1A	0	0.106	0	0	0	0	0	0	0	0.24	0	0	0	0	0	0
KBTBD13	0	0	0	0.221	0	0	0	0	0	0	0	0	0	0	0	0.124
KCNQ1	0	0	0	0	0	0	0	0	0	0	0	0	0	0	0	0.345
SLITRK5	0.139	0	0	0	0	0	0	0	0	0	0	0	0	0	0.206	0

FKRP	0	0	0	0.137	0	0	0	0	0	0.103	0	0	0.105	0	0	0
ANKFY1	0	0	0	0.208	0	0	0	0	0	0	0.137	0	0	0	0	0
CCL3	0	0	0	0	0.204	0	0	0	0.141	0	0	0	0	0	0	0
PLK2	0	0	0	0	0.218	0	0	0.127	0	0	0	0	0	0	0	0
TSNAX-DISC1	0	0	0.125	0	0	0	0	0	0	0	0	0	0.105	0	0.114	0
TRBC2	0.1	0	0	0	0	0	0	0	0	0	0	0	0	0	0.244	0
MYP6	0	0	0	0	0	0	0	0	0	0.123	0	0	0.221	0	0	0
DFNB48	0	0	0	0	0	0	0	0	0	0.224	0	0.12	0	0	0	0
LOC645355	0	0	0	0	0	0.135	0	0	0	0.209	0	0	0	0	0	0
NFKB2	0	0	0	0	0.19	0	0	0	0.154	0	0	0	0	0	0	0
PPIAL4B	0	0	0	0	0	0	0	0	0	0	0	0	0	0	0.343	0
BPNT1	0.116	0	0	0	0	0	0	0	0	0	0.227	0	0	0	0	0
EGR4	0	0	0	0.343	0	0	0	0	0	0	0	0	0	0	0	0
LIMS1	0.343	0	0	0	0	0	0	0	0	0	0	0	0	0	0	0
AKT1	0	0	0	0	0	0	0	0	0	0	0.167	0	0	0	0	0.176
NOL7	0	0	0	0	0	0	0	0	0	0	0.178	0	0	0	0.165	0
HOXA1	0	0	0	0.237	0	0	0	0	0	0	0	0	0	0	0.106	0
PDZD7	0	0	0	0	0	0	0	0	0	0.192	0	0.151	0	0	0	0
ANOP1	0	0	0	0.212	0	0	0.131	0	0	0	0	0	0	0	0	0
IGKV1OR2-0	0	0	0	0	0	0	0	0	0	0	0	0	0	0	0.342	0
CTSB	0	0	0	0	0	0.192	0	0	0	0	0	0	0	0.15	0	0
OR4E2	0	0.127	0	0	0	0	0	0	0	0	0	0.215	0	0	0	0
ZNF827	0	0	0	0	0	0	0	0	0	0	0	0	0	0	0.341	0
KLHL7	0	0	0	0	0	0	0	0	0	0.117	0	0.101	0	0.123	0	0
RAB35	0	0	0	0.341	0	0	0	0	0	0	0	0	0	0	0	0
SCN5A	0	0	0	0	0	0.153	0	0	0	0	0	0	0	0	0	0.188
MARCKSP1	0	0	0	0	0	0.212	0	0.129	0	0	0	0	0	0	0	0
HLA-A	0	0	0	0	0	0	0	0	0	0	0	0	0	0	0	0.34
ARG2	0	0	0	0	0	0	0	0	0	0	0	0	0	0	0.34	0
PCDHGA1	0	0	0	0	0	0	0	0	0	0	0	0	0	0	0.34	0
RARRES3	0	0	0	0	0	0	0	0	0	0	0	0	0	0	0.34	0
ENDOG	0	0	0	0	0	0	0	0	0	0	0.203	0	0	0.137	0	0
MOBP	0	0.229	0	0.111	0	0	0	0	0	0	0	0	0	0	0	0
CADM4	0	0	0	0.34	0	0	0	0	0	0	0	0	0	0	0	0
FDFT1	0	0	0	0.34	0	0	0	0	0	0	0	0	0	0	0	0
C4ORF34	0	0	0	0	0	0	0	0	0	0	0	0	0	0	0.187	0.153
SAMD3	0	0	0.128	0	0	0	0	0	0	0	0	0	0.108	0	0.104	0
ACTR10	0	0.113	0	0	0	0	0	0	0	0	0.127	0	0	0	0.1	0
DFNA53	0	0	0	0.163	0	0	0	0	0	0	0	0.177	0	0	0	0
GJB5	0	0	0	0.182	0	0	0	0	0	0	0	0.158	0	0	0	0
KCNJ9	0	0	0	0	0	0.214	0	0	0	0.126	0	0	0	0	0	0

HTATSF1	0	0	0	0	0.13	0	0	0	0.21	0	0	0	0	0	0	0
PAR-SN	0	0	0	0	0	0.189	0.151	0	0	0	0	0	0	0	0	0
CNPY1	0	0	0	0	0	0	0	0	0	0	0	0	0	0	0.339	0
PCP4L1	0	0	0	0	0	0	0	0	0	0	0	0	0	0	0.339	0
TRBV4-3	0	0	0	0	0	0	0	0	0	0	0	0	0	0	0.339	0
TAF4	0	0	0	0	0	0	0	0	0	0	0.339	0	0	0	0	0
CCR10	0	0	0	0.339	0	0	0	0	0	0	0	0	0	0	0	0
MTMR14	0	0	0	0.339	0	0	0	0	0	0	0	0	0	0	0	0
GJA5	0	0	0	0.21	0	0	0	0	0	0	0	0	0	0	0	0.129
PLCB4	0	0	0	0	0	0.145	0	0	0	0.194	0	0	0	0	0	0
CCNT1	0	0	0	0	0.104	0	0	0	0.235	0	0	0	0	0	0	0
STAP1	0	0	0	0	0.167	0	0	0	0.172	0	0	0	0	0	0	0
DDX20	0	0.221	0	0.117	0	0	0	0	0	0	0	0	0	0	0	0
FAM176A	0	0	0	0	0	0	0	0	0	0	0	0	0	0.123	0.215	0
HSPA7	0	0.125	0	0	0	0	0	0	0	0	0.103	0	0	0.11	0	0
RNF165	0.106	0	0.12	0	0	0	0	0	0	0	0	0	0.112	0	0	0
SHANK1	0	0	0.141	0	0	0	0	0	0	0	0	0	0.197	0	0	0
SLSN3	0	0	0	0	0	0	0	0	0	0.186	0	0.152	0	0	0	0
NPHP1	0	0	0	0	0	0	0	0	0	0	0	0.137	0	0	0	0.2
R3HCC1	0	0	0	0	0	0	0	0	0	0	0.22	0	0	0	0.117	0
TLE4	0	0	0	0	0	0	0	0	0	0	0	0	0	0	0.337	0
TRR	0	0	0	0	0	0	0	0	0	0	0	0	0	0	0.337	0
MATN3	0	0	0	0.337	0	0	0	0	0	0	0	0	0	0	0	0
MYT1	0	0	0	0.337	0	0	0	0	0	0	0	0	0	0	0	0
IGHD@	0	0	0	0.179	0	0	0	0	0	0	0	0	0	0	0.158	0
STK32B	0	0	0.143	0	0	0	0	0	0	0	0	0	0.194	0	0	0
WS2B	0	0	0	0.188	0	0	0	0	0	0	0	0.149	0	0	0	0
ZNF286A	0	0	0	0.141	0	0	0	0	0	0	0	0.196	0	0	0	0
PRKRIP1	0	0.183	0	0	0	0	0	0	0	0	0.154	0	0	0	0	0
SLC44A4	0	0.125	0	0	0	0	0	0.212	0	0	0	0	0	0	0	0
IP6K2	0	0	0	0	0	0	0	0	0	0	0	0	0	0	0.336	0
MYOT	0	0	0	0.198	0	0	0	0	0.138	0	0	0	0	0	0	0
KCNH3	0	0	0	0	0	0.183	0	0.153	0	0	0	0	0	0	0	0
HSPB3	0	0.101	0	0.117	0	0	0	0	0	0	0	0.117	0	0	0	0
ERMN	0	0.165	0	0.17	0	0	0	0	0	0	0	0	0	0	0	0
LOC440683	0	0	0	0	0	0	0	0	0	0	0.141	0	0	0	0	0.194
REM1	0	0	0	0	0	0.214	0	0	0	0	0	0	0	0	0	0.121
YWHAB	0	0.185	0	0	0	0.15	0	0	0	0	0	0	0	0	0	0
LSL	0	0	0	0	0	0	0	0	0	0	0	0	0	0	0	0.334
MARK2	0.105	0.113	0	0	0	0	0	0	0.116	0	0	0	0	0	0	0
HTR4	0	0	0.123	0	0	0	0	0	0	0	0	0	0.101	0	0	0.109

SH3GL1P2	0	0	0	0	0	0	0	0	0	0	0.165	0	0	0	0.168	0
GTF2IRD2B	0	0	0.107	0.113	0	0	0	0	0	0	0	0	0.113	0	0	0
TMEM200A	0.115	0	0.115	0	0	0	0	0	0	0	0	0	0.103	0	0	0
TMEM100	0.126	0	0	0	0	0	0	0	0	0	0	0	0	0	0	0.207
OTP	0	0	0	0	0	0	0	0	0	0	0.153	0	0	0	0.18	0
NDUFS8	0	0	0	0	0	0	0.182	0	0	0	0	0	0	0.151	0	0
KCNAB2	0	0	0	0	0	0.186	0	0.147	0	0	0	0	0	0	0	0
SYT3	0	0	0	0	0	0	0	0	0	0	0	0	0	0	0.332	0
NXNL1	0	0	0	0	0	0	0	0	0	0.103	0	0	0	0.229	0	0
ST8SIA2	0	0	0.129	0	0	0.203	0	0	0	0	0	0	0	0	0	0
SYNDIG1	0	0	0.129	0	0	0.203	0	0	0	0	0	0	0	0	0	0
CR1	0	0	0	0	0.332	0	0	0	0	0	0	0	0	0	0	0
LPIN3	0	0	0	0.332	0	0	0	0	0	0	0	0	0	0	0	0
MBNL2	0	0	0	0	0	0	0	0	0	0	0.154	0	0	0	0.178	0
DFNA27	0	0	0	0	0	0	0	0	0	0.121	0	0.211	0	0	0	0
SRSF7	0	0	0	0	0.173	0	0	0	0.159	0	0	0	0	0	0	0
UBXN1	0	0	0	0	0.175	0	0	0	0.157	0	0	0	0	0	0	0
CCT7P2	0	0	0	0	0	0	0	0	0	0	0	0	0	0	0	0.331
HK2P1	0	0	0	0	0	0	0	0	0	0	0	0	0	0	0	0.331
CTAA2	0	0	0	0.192	0	0	0	0	0	0	0	0.139	0	0	0	0
MFSD4	0	0	0	0.228	0	0	0	0	0	0	0	0.103	0	0	0	0
TJP2	0	0.169	0	0	0.162	0	0	0	0	0	0	0	0	0	0	0
RPS19P2	0	0	0	0	0	0	0	0	0	0	0	0.187	0	0	0	0.144
GUCY1A2	0.183	0	0	0	0	0	0	0	0	0	0	0	0	0	0	0.148
MYO15A	0	0	0	0	0	0	0	0	0	0.12	0	0.211	0	0	0	0
HTR3A	0	0	0	0	0	0.228	0	0	0	0	0	0	0	0	0	0.102
CNFN	0	0	0	0	0	0	0	0	0	0	0	0	0	0	0.33	0
FXR2	0.115	0	0.112	0	0	0	0	0	0	0	0	0	0.103	0	0	0
ABCB7	0	0	0	0	0	0	0.117	0	0	0	0	0.213	0	0	0	0
NTN3	0	0.113	0	0	0	0	0	0	0	0	0	0.217	0	0	0	0
ABHD6	0	0	0	0	0	0	0	0	0	0	0.33	0	0	0	0	0
CALM1	0	0	0	0	0	0	0	0	0	0	0.33	0	0	0	0	0
HADHAP1	0	0	0	0	0.22	0	0	0	0.11	0	0	0	0	0	0	0
ATP9B	0	0	0	0	0	0.154	0	0	0	0	0	0	0	0	0	0.176
SFMBT1	0	0	0	0	0.188	0	0	0	0.142	0	0	0	0	0	0	0
COPD21	0	0	0	0.213	0	0	0	0	0	0	0	0	0	0	0	0.116
HERC2P1	0	0	0	0	0	0.189	0.14	0	0	0	0	0	0	0	0	0
AAT1	0	0.145	0	0	0	0	0	0	0	0	0	0	0	0	0	0.184
PACSIN1	0	0	0	0	0	0	0	0	0	0	0.15	0	0	0	0.179	0
WHSC2	0	0	0	0	0	0	0	0	0	0	0.164	0	0	0	0.164	0
FAM86B2	0	0	0	0	0	0	0	0	0	0	0	0	0	0	0.328	0

SPRR1B	0	0	0	0	0	0	0	0	0	0	0	0	0	0	0.328	0
SLITRK2	0	0	0.125	0	0	0.203	0	0	0	0	0	0	0	0	0	0
KIF9	0	0	0	0.328	0	0	0	0	0	0	0	0	0	0	0	0
NAA10	0	0	0	0	0	0	0	0	0	0	0.151	0	0	0	0.177	0
SV2C	0	0	0	0	0	0.144	0	0	0	0	0	0	0	0	0.184	0
SMPD1	0	0	0	0	0.178	0	0	0	0	0	0	0	0	0.15	0	0
LHX3	0.181	0.147	0	0	0	0	0	0	0	0	0	0	0	0	0	0
BRSK1	0	0	0	0	0	0.163	0	0	0	0	0	0	0	0	0	0.164
TXNL4A	0	0	0	0	0	0	0	0	0	0	0	0	0	0	0.327	0
DYX4	0	0	0.109	0	0	0	0	0	0	0.102	0	0	0.116	0	0	0
TRY6	0	0	0	0	0	0	0	0	0	0	0	0.327	0	0	0	0
KCNJ14	0	0	0	0	0	0.221	0	0.106	0	0	0	0	0	0	0	0
NRG2	0	0	0	0.327	0	0	0	0	0	0	0	0	0	0	0	0
FTH1	0	0	0	0	0	0	0	0	0	0	0.142	0	0	0.185	0	0
ANKAR	0.104	0	0	0	0	0	0	0	0	0	0	0	0	0	0.222	0
SDC4	0	0	0	0	0	0	0	0	0	0	0	0	0	0	0.326	0
SLC1A4	0	0	0	0	0	0.218	0	0.108	0	0	0	0	0	0	0	0
CDH4	0	0	0	0	0.326	0	0	0	0	0	0	0	0	0	0	0
HSPB1P1	0	0	0	0.326	0	0	0	0	0	0	0	0	0	0	0	0
KWE	0	0	0	0	0	0	0	0	0.118	0	0	0.208	0	0	0	0
CACNB1	0	0	0	0	0	0.163	0	0	0	0	0	0	0	0	0	0.162
CBX8	0	0	0	0	0	0	0	0	0	0	0.221	0	0	0	0.104	0
RASGRP2	0	0	0	0	0	0	0	0	0	0	0.156	0	0	0	0.169	0
WHSC1	0	0	0	0	0	0	0	0	0	0	0.169	0	0	0	0.156	0
CNO	0	0	0.138	0	0	0	0	0	0	0	0	0	0.187	0	0	0
IDI2	0.108	0.101	0	0	0	0	0	0	0.116	0	0	0	0	0	0	0
KCNC1	0	0	0	0	0	0.17	0	0.155	0	0	0	0	0	0	0	0
SRSF2	0	0	0	0	0.143	0	0	0	0.182	0	0	0	0	0	0	0
VDAC1P3	0.126	0	0	0	0	0	0	0	0	0	0	0	0	0	0	0.198
GCH1	0	0	0	0	0	0	0	0	0	0	0	0	0	0	0	0.324
ARHGEF6	0	0	0	0	0	0	0	0	0	0	0.212	0	0	0	0.112	0
OATP1	0	0	0	0	0	0	0	0	0	0	0	0	0	0	0.324	0
PAAF1	0	0	0	0	0.136	0	0	0	0.188	0	0	0	0	0	0	0
BRD1	0	0	0.129	0	0	0	0	0	0.195	0	0	0	0	0	0	0
PAR5	0	0	0	0	0	0.19	0.134	0	0	0	0	0	0	0	0	0
PLCL1	0	0	0	0	0	0.19	0.134	0	0	0	0	0	0	0	0	0
USP53	0	0	0	0	0	0	0	0	0	0	0.144	0	0	0	0.18	0
PSD2	0	0	0	0	0	0.176	0.148	0	0	0	0	0	0	0	0	0
TRBJ2-2	0	0	0	0	0	0	0	0	0	0	0.159	0	0	0	0.164	0
LOC493754	0	0	0	0	0	0	0	0	0	0	0	0	0	0	0.323	0
OR5V1	0	0	0	0	0	0	0	0	0	0	0	0	0	0	0.323	0

RAB3GAP1	0	0	0.108	0.104	0	0	0	0	0	0	0	0	0.111	0	0	0
ELP4	0	0	0	0	0	0.189	0.134	0	0	0	0	0	0	0	0	0
FAM174B	0	0	0	0	0.323	0	0	0	0	0	0	0	0	0	0	0
ABTB1	0	0	0	0.323	0	0	0	0	0	0	0	0	0	0	0	0
GSTM1	0	0	0	0	0	0	0.147	0	0	0	0	0	0	0	0	0.176
MAP3K5	0	0	0	0	0	0	0	0	0	0	0.145	0	0	0	0.178	0
VDAC2P2	0.107	0	0	0	0	0	0	0	0	0	0	0	0	0	0	0.215
DFNA16	0	0	0	0	0	0.182	0	0	0	0	0	0.14	0	0	0	0
KIF26A	0	0	0	0.183	0	0	0	0	0	0	0	0.139	0	0	0	0
PCAP	0	0	0	0.2	0	0	0	0	0	0	0	0.122	0	0	0	0
DYSF	0	0.216	0	0.106	0	0	0	0	0	0	0	0	0	0	0	0
LRP2	0.322	0	0	0	0	0	0	0	0	0	0	0	0	0	0	0
FLVCR2	0	0	0	0	0	0	0	0	0	0	0	0	0	0	0	0.321
TPR	0	0	0	0	0	0	0	0	0	0	0.215	0	0	0	0.106	0
ANXA5	0	0	0	0	0	0	0	0	0	0	0	0	0	0	0.321	0
CTSL1P2	0	0	0	0	0	0	0	0	0	0	0	0	0	0	0.321	0
SYPL1	0	0	0	0	0	0	0	0	0	0	0	0	0	0	0.321	0
LOC100288079	0	0	0	0.321	0	0	0	0	0	0	0	0	0	0	0	0
ADAMTS20	0	0	0	0	0	0.179	0.142	0	0	0	0	0	0	0	0	0
NDUFA6	0	0	0	0	0	0	0	0	0	0	0	0	0	0.109	0.211	0
TRNAQ1	0	0	0	0	0	0	0	0	0	0	0.159	0	0	0	0.161	0
CSNK1G1	0.128	0	0	0	0	0	0	0	0	0	0.192	0	0	0	0	0
ECE1	0	0	0	0	0.177	0	0	0	0	0	0	0	0	0	0	0.143
TBC1D1	0	0.144	0	0	0	0	0	0	0	0	0	0	0	0	0	0.176
RSPH9	0	0	0	0	0	0	0	0	0	0	0	0.19	0	0	0	0.129
SPRR1A	0	0	0	0	0	0	0	0	0	0	0	0	0	0	0.319	0
MIR29A	0	0	0	0	0.188	0	0	0	0.131	0	0	0	0	0	0	0
PDE8B	0	0	0	0	0.208	0	0	0	0.111	0	0	0	0	0	0	0
TNPO2	0	0.135	0	0	0	0	0	0	0.184	0	0	0	0	0	0	0
YWHAG	0	0.125	0	0	0	0.194	0	0	0	0	0	0	0	0	0	0
KIF5B	0	0	0	0.319	0	0	0	0	0	0	0	0	0	0	0	0
ALG10	0	0	0	0	0	0.143	0	0	0	0	0	0	0	0	0	0.176
FLCN	0	0	0	0	0	0.142	0	0	0	0	0	0.177	0	0	0	0
NUS1	0.145	0	0	0	0.174	0	0	0	0	0	0	0	0	0	0	0
MHS4	0	0	0	0	0	0	0	0	0	0	0	0	0	0	0	0.318
CHRM5	0	0	0.119	0	0	0	0	0	0	0	0	0	0.199	0	0	0
MEGF9	0	0	0	0.211	0	0	0	0	0	0	0	0.107	0	0	0	0
MTX3	0.318	0	0	0	0	0	0	0	0	0	0	0	0	0	0	0
SPRR2B	0	0	0	0	0	0	0	0	0	0	0.145	0	0	0	0.173	0
UBXN7	0	0	0	0	0.142	0	0	0	0.176	0	0	0	0	0	0	0
FETUB	0	0	0	0	0	0	0	0	0	0	0	0	0	0	0.317	0

GIN1	0	0	0	0	0.186	0	0	0	0.131	0	0	0	0	0	0	0
SIRPB1	0	0	0	0	0.154	0	0	0	0.163	0	0	0	0	0	0	0
GEMIN4	0	0.196	0	0	0	0	0	0	0.121	0	0	0	0	0	0	0
TMEM88	0.317	0	0	0	0	0	0	0	0	0	0	0	0	0	0	0
HPCAL4	0	0	0	0	0	0.143	0	0	0	0	0	0	0	0	0	0.174
ZNF395	0	0	0	0	0	0	0	0	0	0	0.151	0	0	0	0.165	0
C9ORF7	0	0	0	0	0	0.127	0	0	0	0	0	0	0	0	0.189	0
FAM158A	0	0	0	0	0	0	0	0	0	0	0	0	0	0	0.316	0
HIST1H2BL	0	0	0	0	0	0	0	0	0	0	0	0	0	0	0.316	0
TAPBPL	0	0	0	0	0.189	0	0	0	0.127	0	0	0	0	0	0	0
PNOC	0	0	0	0	0	0.141	0	0	0	0.175	0	0	0	0	0	0
LRRC37A	0	0	0	0	0.174	0	0	0	0.142	0	0	0	0	0	0	0
TSHZ3	0	0	0	0	0.173	0	0	0	0.143	0	0	0	0	0	0	0
NIM1	0	0	0	0	0	0	0	0	0	0	0	0	0	0	0	0.315
NAA15	0	0	0	0	0	0	0	0	0	0	0.136	0	0	0	0.179	0
CYP27C1	0	0	0	0	0	0	0	0	0	0	0	0	0	0	0.315	0
MADD	0	0	0	0	0	0	0	0	0	0	0	0	0	0	0.315	0
RIPPLY1	0	0	0	0	0	0	0	0	0	0	0	0	0	0	0.315	0
NAB2	0	0	0	0.201	0	0	0	0	0	0	0	0.114	0	0	0	0
FRG1	0	0	0	0.315	0	0	0	0	0	0	0	0	0	0	0	0
MAP3K12	0	0	0	0	0	0	0	0	0	0	0.197	0	0	0	0.117	0
SLC17A9	0	0	0	0	0	0	0	0	0	0	0	0	0	0	0.314	0
SLC25A21	0	0	0	0	0	0	0	0	0	0	0	0	0	0	0.314	0
TTLL4	0	0	0	0	0	0	0	0	0	0	0.208	0	0	0.106	0	0
IGLV2-23	0	0	0	0.314	0	0	0	0	0	0	0	0	0	0	0	0
IGLVI-42	0	0	0	0.314	0	0	0	0	0	0	0	0	0	0	0	0
DLG1	0	0	0	0.15	0	0	0	0	0	0	0	0	0	0	0	0.163
TUBB8	0	0	0	0.207	0	0	0	0	0	0	0	0	0	0	0.106	0
ARPP19	0	0	0	0	0	0	0	0	0	0	0.177	0	0	0.136	0	0
CASP2	0	0	0	0	0	0	0	0	0	0	0.183	0	0	0.13	0	0
LPAL1	0.117	0	0	0	0.196	0	0	0	0	0	0	0	0	0	0	0
FAM109A	0.141	0	0	0.172	0	0	0	0	0	0	0	0	0	0	0	0
GPR158	0	0	0	0	0	0	0	0	0	0	0.211	0	0	0	0.101	0
PSME3	0	0	0	0	0	0	0	0	0	0	0.132	0	0	0	0.18	0
TUBBP1	0	0	0	0	0	0	0	0	0	0	0.14	0	0	0	0.172	0
IGLV@	0	0	0	0	0	0	0	0	0	0	0	0	0	0	0.312	0
PPIP5K1P1	0	0	0	0.184	0	0	0	0	0	0	0	0.128	0	0	0	0
KIAA0889	0	0	0	0	0.184	0	0	0	0.128	0	0	0	0	0	0	0
ZFP30	0.312	0	0	0	0	0	0	0	0	0	0	0	0	0	0	0
SCA31	0	0.148	0	0	0	0	0	0	0	0	0	0	0	0	0	0.163
LOC728405	0	0	0	0	0	0	0	0	0	0	0.151	0	0	0	0.16	0

SH3GL1P1	0	0	0	0	0	0	0	0	0	0	0.204	0	0	0	0.107	0
LRRC19	0	0	0	0	0	0	0	0	0	0	0	0	0	0	0.311	0
MRPL10	0	0	0	0	0	0	0.165	0	0	0	0	0	0	0.146	0	0
MNG2	0	0	0.107	0.103	0	0	0	0	0	0	0	0	0.101	0	0	0
UQCRFS1P1	0	0	0	0	0	0	0.125	0	0	0	0	0	0	0	0.185	0
ZNF219	0	0	0	0	0	0	0	0	0	0	0	0	0	0	0.31	0
RAB1A	0	0	0	0.31	0	0	0	0	0	0	0	0	0	0	0	0
CALML5	0.103	0	0	0	0	0	0	0	0	0	0	0	0	0	0.206	0
VIT	0	0	0	0	0	0	0	0	0	0	0	0	0	0	0.309	0
AMPH	0	0	0	0	0	0.137	0	0	0	0	0.172	0	0	0	0	0
KCNIP1	0	0	0	0	0	0.144	0	0.165	0	0	0	0	0	0	0	0
SMU1	0	0	0	0	0	0.147	0	0.162	0	0	0	0	0	0	0	0
HERC2P4	0	0	0	0	0	0.19	0.119	0	0	0	0	0	0	0	0	0
RPP25	0	0	0	0	0	0.161	0.148	0	0	0	0	0	0	0	0	0
PIKFYVE	0	0	0	0.309	0	0	0	0	0	0	0	0	0	0	0	0
IGKV2OR22-3	0	0	0	0	0	0	0	0	0	0	0	0	0	0	0.308	0
EIF2AK3	0	0	0	0	0	0	0	0	0	0	0.123	0	0	0.185	0	0
ACTG1P9	0	0	0	0	0	0	0	0	0	0	0	0	0	0	0.307	0
PPL	0	0	0	0	0	0	0	0	0	0	0	0	0	0	0.307	0
DHFRL1	0.163	0	0	0	0	0	0	0	0	0	0.144	0	0	0	0	0
HDAC1	0	0	0	0	0	0	0	0	0	0	0.307	0	0	0	0	0
ADORA1	0	0	0	0	0	0.179	0.128	0	0	0	0	0	0	0	0	0
ATP11C	0	0	0	0	0	0.179	0.128	0	0	0	0	0	0	0	0	0
ELMOD2	0	0	0	0.307	0	0	0	0	0	0	0	0	0	0	0	0
FAM3C	0.181	0.126	0	0	0	0	0	0	0	0	0	0	0	0	0	0
LRP12	0.307	0	0	0	0	0	0	0	0	0	0	0	0	0	0	0
COX6B1P1	0	0	0	0	0	0	0	0	0	0	0	0	0	0	0.306	0
LRRC70	0	0	0	0	0	0	0	0	0	0	0	0	0	0	0.306	0
CREB3L1	0	0.176	0	0	0	0	0	0	0	0	0	0	0	0.13	0	0
OTOGL	0	0	0	0	0	0	0	0	0	0	0	0.125	0.181	0	0	0
NRXN3	0	0	0.108	0	0	0	0	0	0	0	0	0	0.198	0	0	0
ODPF	0	0	0	0	0	0	0	0	0	0	0	0	0.306	0	0	0
MIR297	0	0	0	0	0.186	0	0	0	0.12	0	0	0	0	0	0	0
HADHB	0	0	0	0	0.149	0	0.157	0	0	0	0	0	0	0	0	0
TTC39B	0.306	0	0	0	0	0	0	0	0	0	0	0	0	0	0	0
NAA16	0	0	0	0	0	0	0	0	0	0	0.198	0	0	0	0.107	0
RPH3A	0	0	0	0	0	0	0	0	0	0	0.198	0	0	0	0.107	0
CELIAC5	0	0.124	0	0	0	0	0	0	0	0	0	0	0	0	0.181	0
SIAH2	0	0	0	0	0	0	0	0	0	0	0	0	0	0	0.305	0
STX19	0	0	0	0	0	0	0	0	0	0	0	0	0	0	0.305	0
GINGF4	0	0	0	0.185	0	0	0	0	0	0	0	0.12	0	0	0	0

INPP1	0.104	0	0	0	0	0	0	0	0	0	0.201	0	0	0	0	0
FAF1	0	0	0	0	0.187	0	0	0	0.118	0	0	0	0	0	0	0
LARP7	0	0	0	0	0.162	0	0	0	0.143	0	0	0	0	0	0	0
ANCR	0	0	0	0	0	0.179	0.126	0	0	0	0	0	0	0	0	0
ZNF304	0.133	0	0	0.172	0	0	0	0	0	0	0	0	0	0	0	0
IGHJ@	0	0	0	0.305	0	0	0	0	0	0	0	0	0	0	0	0
LMNB1	0	0	0	0.305	0	0	0	0	0	0	0	0	0	0	0	0
PHF10P1	0	0	0	0	0	0	0	0	0	0	0.138	0	0	0	0.166	0
CILD2	0	0	0	0	0	0	0	0	0	0.151	0	0	0	0	0	0.153
FIGN	0	0	0	0	0	0	0	0	0	0	0	0	0	0	0	0.304
GNPDA1	0	0	0	0	0	0	0	0	0	0	0	0	0	0	0	0.304
DPY19L2P2	0	0	0	0	0	0	0	0	0	0	0.19	0	0	0	0.114	0
CPHL1P	0	0	0	0	0	0	0	0	0	0	0	0	0	0	0.304	0
DPY19L2	0	0	0	0	0	0	0	0	0	0	0	0	0	0	0.304	0
EIF5A	0	0	0	0	0	0	0	0	0	0	0	0	0	0	0.304	0
OR9A2	0	0	0	0	0	0	0	0	0	0	0	0	0	0	0.304	0
PAFAH1B1P1	0	0	0	0	0	0	0	0	0	0	0	0	0	0	0.304	0
PMPCB	0	0	0	0	0	0	0.153	0	0	0	0	0	0	0.151	0	0
NLRP4	0	0	0	0	0	0	0	0	0	0	0.304	0	0	0	0	0
RN7SK	0	0	0	0	0.172	0	0	0	0.132	0	0	0	0	0	0	0
CSMD3	0	0	0	0	0	0.189	0.115	0	0	0	0	0	0	0	0	0
CYP2C19	0	0	0	0	0	0.191	0.113	0	0	0	0	0	0	0	0	0
SCA30	0	0	0	0.304	0	0	0	0	0	0	0	0	0	0	0	0
BANF1	0	0	0	0.304	0	0	0	0	0	0	0	0	0	0	0	0
SGSM2	0.304	0	0	0	0	0	0	0	0	0	0	0	0	0	0	0
USH1E	0	0	0	0	0	0	0	0	0	0.163	0	0.14	0	0	0	0
DLX1	0	0	0	0	0	0.114	0	0	0	0	0	0	0	0	0.189	0
ATAT1	0	0	0	0	0	0	0	0	0	0	0	0	0	0	0.303	0
HNB1	0	0	0	0.2	0	0	0	0	0	0	0	0.103	0	0	0	0
HERC2P5	0	0	0	0	0	0.19	0.113	0	0	0	0	0	0	0	0	0
IQSEC2	0	0	0	0	0	0.19	0.113	0	0	0	0	0	0	0	0	0
EIF2B2	0.162	0	0	0	0	0	0.141	0	0	0	0	0	0	0	0	0
MYOCD	0.124	0	0	0	0.179	0	0	0	0	0	0	0	0	0	0	0
RPL18AP16	0	0	0	0	0	0	0.115	0	0	0	0	0	0	0	0.187	0
SYT8	0	0	0	0	0	0	0	0	0	0	0	0	0	0	0.302	0
ULK1	0	0	0	0	0	0	0	0	0.194	0	0	0	0	0.108	0	0
VEGFA	0	0.141	0	0	0	0	0	0	0.161	0	0	0	0	0	0	0
MMP9	0	0.129	0	0	0	0	0.173	0	0	0	0	0	0	0	0	0
C9ORF30	0	0	0.129	0	0	0.173	0	0	0	0	0	0	0	0	0	0
C14ORF55	0	0.122	0	0.18	0	0	0	0	0	0	0	0	0	0	0	0
LRFN3	0	0	0	0.302	0	0	0	0	0	0	0	0	0	0	0	0

EN2	0	0	0	0	0	0	0	0	0	0.136	0	0	0	0	0.165	0
NME7	0	0	0	0	0	0	0	0	0	0	0	0	0	0	0	0.301
UNC50	0	0	0	0	0	0	0	0	0	0	0.141	0	0	0	0.16	0
VEPH1	0	0	0	0	0	0	0	0	0	0	0	0	0	0	0.301	0
GAS7	0	0	0	0	0	0	0	0	0.153	0	0	0	0	0.148	0	0
CORD4	0	0	0	0.196	0	0	0	0	0	0.105	0	0	0	0	0	0
PAOD1	0	0	0	0.301	0	0	0	0	0	0	0	0	0	0	0	0
DYX1	0	0	0.139	0	0	0	0	0	0	0	0	0	0.161	0	0	0
BAX	0	0.162	0	0	0	0	0	0	0	0	0.138	0	0	0	0	0
EIF2B4	0.131	0	0	0	0	0	0.169	0	0	0	0	0	0	0	0	0
WDYHV1	0	0	0	0	0	0	0	0	0	0	0.114	0	0	0	0.186	0
HOXB1	0	0	0	0.193	0	0	0	0	0	0	0	0	0	0	0.107	0
TSHZ2	0	0	0	0	0.154	0	0	0	0.146	0	0	0	0	0	0	0
HERC2P3	0	0	0	0	0	0.19	0.11	0	0	0	0	0	0	0	0	0
HERC2P8	0	0	0	0	0	0.19	0.11	0	0	0	0	0	0	0	0	0
RBM4	0	0	0	0	0.171	0	0	0	0.128	0	0	0	0	0	0	0
PRKAG2	0	0	0	0	0	0.142	0	0	0	0	0	0	0	0	0	0.157
UHRF2	0	0	0	0	0	0	0	0	0	0	0.141	0	0	0	0.158	0
LPHN1	0	0	0	0	0	0	0	0	0	0	0	0	0	0	0.299	0
PAK3	0	0	0.119	0	0	0	0	0	0	0	0	0	0.18	0	0	0
MYO7A	0	0	0	0	0	0	0	0	0	0.145	0	0.154	0	0	0	0
GCCD2	0	0	0	0.148	0	0	0	0	0	0	0	0.151	0	0	0	0
IBGC1	0	0.12	0	0	0	0	0	0	0	0	0.179	0	0	0	0	0
SNRPA1	0	0.147	0	0	0	0	0	0	0.152	0	0	0	0	0	0	0
GLUD1	0	0	0	0	0	0.19	0.109	0	0	0	0	0	0	0	0	0
RNY4P3	0	0	0	0	0	0	0	0	0	0	0.135	0	0	0	0.163	0
APPBP2	0	0	0	0	0.169	0	0	0	0.129	0	0	0	0	0	0	0
LEFTY3	0	0.144	0	0	0	0	0	0	0	0	0	0	0	0	0	0.154
GUK2	0	0	0	0	0	0	0	0	0	0	0	0	0	0	0	0.298
HEG1	0	0	0	0	0	0	0	0	0	0	0	0	0	0	0	0.298
LRRC63	0	0	0	0	0	0	0	0	0	0	0	0	0	0	0	0.298
EMX2	0	0	0	0	0	0.114	0	0	0	0	0	0	0	0	0.184	0
PAX2	0	0	0	0	0	0	0	0	0	0	0	0	0	0	0.298	0
PDLA	0	0	0	0.181	0	0	0	0	0	0	0	0.117	0	0	0	0
SYNM	0	0.194	0	0	0	0	0	0	0	0.104	0	0	0	0	0	0
LGV1	0	0.143	0	0	0	0	0	0	0.155	0	0	0	0	0	0	0
SLC9A1	0	0	0	0	0	0.15	0.148	0	0	0	0	0	0	0	0	0
CLDN11	0	0.118	0	0.18	0	0	0	0	0	0	0	0	0	0	0	0
C11ORF9	0	0	0	0.298	0	0	0	0	0	0	0	0	0	0	0	0
BBS5	0	0	0	0	0	0	0	0	0	0	0	0	0.193	0	0.104	0
MED20	0	0	0	0	0	0	0	0	0	0	0	0	0	0	0.297	0

TRAV12-3	0	0	0	0	0	0	0	0	0	0	0	0	0	0	0.297	0
NAT1	0	0	0	0	0.178	0	0	0	0	0	0.119	0	0	0	0	0
CXCR4	0	0	0	0	0.187	0	0	0	0.11	0	0	0	0	0	0	0
MDC1B	0	0	0	0.297	0	0	0	0	0	0	0	0	0	0	0	0
TGM5	0	0	0	0	0	0	0	0	0	0	0.135	0	0	0	0.161	0
ATP7A	0	0.165	0	0	0	0	0	0	0	0	0	0	0	0.131	0	0
ABCG1	0.125	0	0	0	0.171	0	0	0	0	0	0	0	0	0	0	0
IMPA2	0.157	0	0.139	0	0	0	0	0	0	0	0	0	0	0	0	0
F8A1	0	0	0	0	0	0	0	0	0	0	0.182	0	0	0	0.114	0
ASS1P8	0	0	0	0	0	0	0	0	0	0	0	0	0	0	0.296	0
C12ORF44	0	0	0	0	0	0	0	0	0.146	0	0	0	0	0.15	0	0
TAC1	0	0	0	0	0.178	0	0	0	0	0	0.118	0	0	0	0	0
IPW	0	0	0	0	0	0.128	0.167	0	0	0	0	0	0	0	0	0
RTN3P1	0	0.132	0	0.163	0	0	0	0	0	0	0	0	0	0	0	0
RP34	0	0	0	0	0	0	0	0	0	0.295	0	0	0	0	0	0
CHRNA10	0	0	0.123	0	0	0.172	0	0	0	0	0	0	0	0	0	0
HYOU1	0	0	0	0	0	0	0	0	0	0	0.156	0	0	0.138	0	0
USH1G	0	0	0	0	0	0	0	0	0	0.164	0	0.13	0	0	0	0
MTMR8	0	0	0	0.163	0	0	0	0	0	0	0	0.131	0	0	0	0
CHRNA9	0	0	0.124	0	0	0.17	0	0	0	0	0	0	0	0	0	0
DYRK2	0.105	0	0	0	0	0	0	0	0	0	0	0	0	0	0	0.189
VDAC2P1	0.145	0	0	0	0	0	0	0	0	0	0	0	0	0	0	0.149
POC1A	0	0	0	0	0	0	0	0	0	0	0.115	0	0	0	0.179	0
SYBU	0	0	0	0.187	0	0	0	0	0	0	0	0	0	0.107	0	0
CNTNAP5	0	0	0.108	0	0	0	0	0	0	0	0	0	0.186	0	0	0
SERINC4	0	0	0	0	0	0.181	0	0.113	0	0	0	0	0	0	0	0
ZBED3	0.294	0	0	0	0	0	0	0	0	0	0	0	0	0	0	0
SLC7A4	0.134	0	0	0	0	0	0	0	0	0	0	0	0	0	0	0.159
CAPN8	0	0	0	0	0	0	0	0	0	0	0.193	0	0	0	0.1	0
PPEF2	0	0	0	0	0	0	0	0	0	0.171	0.122	0	0	0	0	0
KCNA10	0	0	0	0	0	0.18	0	0	0	0	0	0	0	0	0	0.113
ACSL5	0	0	0	0	0	0	0	0	0	0	0	0	0	0	0.293	0
MPZL3	0	0	0	0.178	0	0	0	0	0	0	0	0.115	0	0	0	0
HCL2	0.146	0	0	0	0	0	0	0	0	0	0	0.147	0	0	0	0
FAM109B	0.187	0	0	0.106	0	0	0	0	0	0	0	0	0	0	0	0
NUDT4P1	0	0	0	0	0	0	0	0	0	0	0.13	0	0	0	0.162	0
LOC728393	0	0	0	0	0	0	0	0	0	0	0.175	0	0	0	0.117	0
WDFY3-AS2	0	0	0	0	0	0	0	0	0	0	0	0	0	0	0.292	0
WDR7	0	0	0	0	0	0	0	0	0	0	0	0	0	0	0.292	0
ZNF804B	0	0	0	0	0	0	0	0	0	0	0	0	0	0	0.292	0
TTC21B	0	0	0	0	0	0	0	0	0	0	0	0.174	0.118	0	0	0

PNPO	0	0	0	0	0	0.189	0.103	0	0	0	0	0	0	0	0	0
POU3F3	0	0	0	0.292	0	0	0	0	0	0	0	0	0	0	0	0
PSORS9	0	0	0	0.292	0	0	0	0	0	0	0	0	0	0	0	0
RAB22A	0	0	0	0.292	0	0	0	0	0	0	0	0	0	0	0	0
RAB41	0	0	0	0.292	0	0	0	0	0	0	0	0	0	0	0	0
REM2	0	0	0	0	0	0.129	0	0	0	0	0	0	0	0	0	0.162
MLKL	0	0	0	0	0	0	0	0	0	0	0.13	0	0	0	0.161	0
ZNF209P	0	0	0	0	0	0	0	0	0	0	0.126	0	0	0	0.165	0
TARBP2	0	0	0	0	0.169	0	0	0	0.122	0	0	0	0	0	0	0
CYP2C9	0	0	0	0	0	0.19	0.101	0	0	0	0	0	0	0	0	0
FBXL6	0	0	0	0	0	0	0	0	0	0	0	0	0	0	0.291	0
GNPTAB	0	0	0	0	0	0	0	0	0	0	0	0	0.147	0.144	0	0
SLI2	0	0	0.142	0	0	0	0	0	0	0	0	0	0.149	0	0	0
HNRNPA1	0	0	0	0	0.142	0	0	0	0.149	0	0	0	0	0	0	0
AMFR	0	0.141	0	0	0	0	0	0	0.15	0	0	0	0	0	0	0
ACRPV	0	0	0	0	0	0.18	0.111	0	0	0	0	0	0	0	0	0
SUSD4	0	0	0	0	0	0.181	0.11	0	0	0	0	0	0	0	0	0
HCRTR1	0	0.115	0	0	0.176	0	0	0	0	0	0	0	0	0	0	0
SLC6A2	0	0	0.14	0	0	0	0	0	0	0	0	0	0	0	0	0.15
DNAH5	0	0.15	0	0	0	0	0	0	0	0	0	0	0	0	0	0.14
RNY4P2	0	0	0	0	0	0	0	0	0	0	0.122	0	0	0	0.168	0
PPRC1	0	0	0	0	0	0	0	0	0	0	0.165	0	0	0.125	0	0
OED	0	0	0	0	0	0	0	0	0	0.168	0	0	0.122	0	0	0
ZDHHC8	0	0	0.124	0	0	0	0	0	0	0	0	0	0.166	0	0	0
GPR156	0	0	0	0	0	0.189	0.101	0	0	0	0	0	0	0	0	0
ABCA12	0	0	0	0	0	0	0	0	0	0	0	0	0	0	0.29	0
KIAA1245	0	0	0	0	0	0	0	0	0	0	0	0	0	0	0.29	0
MYP11	0	0	0	0	0	0	0	0	0	0.175	0	0.115	0	0	0	0
FH	0	0	0	0	0	0	0.117	0	0	0	0	0.173	0	0	0	0
KCNMB1	0	0	0	0	0	0.154	0	0	0	0	0	0	0	0	0	0.135
TUBG1P	0.1	0	0	0	0	0	0	0	0	0	0	0	0	0	0.189	0
ATP2B2	0	0	0	0	0	0	0	0	0	0.166	0	0.123	0	0	0	0
GLC1H	0	0	0	0.153	0	0	0	0	0	0	0	0.136	0	0	0	0
EEA1	0.122	0	0	0.167	0	0	0	0	0	0	0	0	0	0	0	0
KCNE1L	0	0	0	0	0	0.142	0	0	0	0	0	0	0	0	0	0.147
MLL2	0	0	0	0	0	0	0	0	0	0	0.113	0	0	0	0.176	0
CEP290	0	0	0	0	0	0	0	0	0	0.106	0	0.183	0	0	0	0
ZNRF1	0	0	0	0.172	0	0	0	0	0	0	0	0.117	0	0	0	0
FGF1	0	0.116	0	0	0	0	0	0	0	0.173	0	0	0	0	0	0
DNAL1	0	0	0	0	0	0	0	0	0	0	0	0.148	0	0	0	0.14
ADORA2A	0	0	0	0	0	0	0	0	0	0	0.149	0	0	0	0	0.139

C6ORF125	0	0	0	0	0	0	0.139	0	0	0	0	0	0	0.149	0	0
SLC30A7	0	0	0	0	0.134	0	0	0	0	0	0	0	0	0.154	0	0
ABCB1	0	0	0	0	0	0.158	0.13	0	0	0	0	0	0	0	0	0
CACNA1D	0	0	0	0	0	0.146	0	0	0	0	0	0	0	0	0	0.142
PIGU	0	0	0	0	0	0	0	0	0	0	0	0	0	0	0	0.288
SPACA4	0	0	0	0	0	0	0	0	0	0	0	0	0	0	0	0.288
ANXA2P3	0	0	0	0	0	0	0	0	0	0	0	0	0	0	0.288	0
NBPF13P	0	0	0	0	0	0	0	0	0	0	0	0	0	0	0.288	0
SPRR3	0	0	0	0	0	0	0	0	0	0	0	0	0	0	0.288	0
TRIM40	0	0	0	0	0	0	0	0	0	0	0	0	0	0	0.288	0
RAB3A	0	0	0.142	0	0	0	0	0	0	0	0	0	0.146	0	0	0
LGMD1F	0	0	0	0.288	0	0	0	0	0	0	0	0	0	0	0	0
MYOF	0	0	0	0.288	0	0	0	0	0	0	0	0	0	0	0	0
TMEM217	0.288	0	0	0	0	0	0	0	0	0	0	0	0	0	0	0
NKX1-1	0	0	0	0	0	0	0	0	0	0	0.128	0	0	0	0.159	0
GINGF3	0	0	0	0.153	0	0	0	0	0	0	0	0.134	0	0	0	0
APCS	0	0	0	0	0.152	0	0	0	0.135	0	0	0	0	0	0	0
PREPL	0	0	0	0	0	0.169	0.118	0	0	0	0	0	0	0	0	0
TMEM147	0.13	0	0	0	0.157	0	0	0	0	0	0	0	0	0	0	0
CRIPT	0	0	0	0.177	0	0	0	0	0	0	0	0	0	0	0	0.11
FUT11	0	0	0	0	0	0	0	0	0	0	0	0	0	0	0	0.287
ALRH	0	0	0	0	0	0	0	0	0	0	0	0	0	0	0.287	0
CAMKV	0	0	0	0	0	0	0	0	0	0	0	0	0	0	0.287	0
LOC652102	0	0	0	0	0	0	0	0	0	0	0	0	0	0	0.287	0
SPRR2F	0	0	0	0	0	0	0	0	0	0	0	0	0	0	0.287	0
TARBP1	0	0	0	0	0.172	0	0	0	0.115	0	0	0	0	0	0	0
LEMD2	0	0	0	0.287	0	0	0	0	0	0	0	0	0	0	0	0
GPX4	0	0	0	0	0.168	0	0	0	0	0	0	0	0	0	0	0.118
SLC25A36	0	0	0	0	0	0	0.137	0	0	0	0	0	0	0.149	0	0
SDHA	0	0	0	0	0	0	0.131	0	0	0	0	0.155	0	0	0	0
PCDHA12	0	0	0.141	0	0	0	0	0	0	0	0	0	0	0	0	0.145
GNB3	0.141	0	0	0	0	0	0	0	0	0	0	0	0	0	0	0.145
HTR2B	0	0	0	0	0	0	0	0	0	0	0	0	0	0	0	0.286
EXOC3	0	0	0	0	0	0	0	0	0	0	0	0	0	0	0.286	0
IP6K1	0	0	0	0	0	0	0	0	0	0	0	0	0	0	0.286	0
HEXA	0	0	0	0	0.179	0	0	0	0	0	0	0	0	0.107	0	0
NBPF3	0.286	0	0	0	0	0	0	0	0	0	0	0	0	0	0	0
ISCU	0	0	0	0	0	0	0.116	0	0	0	0	0	0	0	0	0.169
VDAC1P4	0.122	0	0	0	0	0	0	0	0	0	0	0	0	0	0	0.163
DMWD	0	0	0	0	0	0	0	0	0	0	0.184	0	0	0	0.101	0
KIAA0226	0	0	0	0	0	0	0	0	0	0	0.117	0	0	0.168	0	0

CTSE	0	0	0	0	0.134	0	0	0	0	0	0	0	0	0.151	0	0
GAA	0	0	0	0	0.132	0	0	0	0	0	0	0	0	0.153	0	0
KIF4CP	0	0.123	0	0.162	0	0	0	0	0	0	0	0	0	0	0	0
RGS9	0	0	0	0	0	0	0	0	0	0.103	0	0	0	0	0	0.182
DPM1	0	0	0	0	0	0	0.109	0	0	0	0	0	0	0	0	0.176
PRKAB2	0	0	0	0	0	0	0	0	0	0	0	0	0	0	0	0.285
ISOC2	0	0	0	0	0	0	0	0	0	0	0	0	0	0	0.285	0
CASP8	0	0	0	0	0	0	0	0	0	0	0.285	0	0	0	0	0
IGF1R	0	0	0	0	0.148	0	0	0	0	0	0	0	0	0	0	0.136
GDI1	0	0	0.119	0	0	0	0	0	0	0	0	0	0.165	0	0	0
CNR2	0	0.155	0	0	0	0	0	0	0	0	0.129	0	0	0	0	0
THBS1	0.152	0	0	0	0.132	0	0	0	0	0	0	0	0	0	0	0
MKS1	0	0	0	0	0	0	0	0	0	0	0	0.146	0.137	0	0	0
CREBBP	0	0	0	0	0.114	0	0	0	0	0	0.169	0	0	0	0	0
TBC1D25	0	0	0	0	0	0	0	0	0	0	0.108	0	0	0	0.175	0
ASS1P14	0	0	0	0	0	0	0	0	0	0	0	0	0	0	0.283	0
ERN1	0	0	0	0	0	0	0	0	0	0	0.172	0	0	0.111	0	0
UMODL1	0	0	0.141	0	0	0	0	0	0	0	0	0	0.142	0	0	0
CYP39A1	0	0	0	0	0.173	0	0	0	0.11	0	0	0	0	0	0	0
VMO1	0.283	0	0	0	0	0	0	0	0	0	0	0	0	0	0	0
ICMT	0	0	0	0	0	0	0	0	0	0	0.181	0	0	0	0.101	0
ATG4D	0	0	0	0	0	0	0	0	0	0	0.181	0	0	0.101	0	0
STRC	0	0	0	0	0	0	0	0	0	0	0	0.101	0.181	0	0	0
ESPN	0	0	0	0	0	0	0	0	0	0.162	0	0.12	0	0	0	0
NEUROD2	0	0	0	0	0	0.13	0	0	0	0.152	0	0	0	0	0	0
AVP	0	0	0	0	0.146	0	0	0	0.136	0	0	0	0	0	0	0
PSMC3	0	0	0	0	0.159	0	0	0	0.123	0	0	0	0	0	0	0
OR1F1	0	0	0	0	0	0	0	0	0	0	0	0	0	0	0	0.282
DNAH3	0	0	0	0	0	0	0	0	0	0	0.178	0	0	0	0.104	0
LOC728931	0	0	0	0	0	0	0	0	0	0	0	0	0	0	0.282	0
ODC1	0	0	0	0	0	0	0	0	0	0	0	0	0	0	0.282	0
PAOX	0	0	0	0	0	0	0	0	0	0	0	0	0	0	0.282	0
LIAS	0.172	0	0	0	0	0	0	0	0	0	0	0	0	0.11	0	0
YWHAZ	0	0.174	0	0	0	0	0	0	0	0	0.108	0	0	0	0	0
BCLAF1	0	0	0	0.282	0	0	0	0	0	0	0	0	0	0	0	0
MIR2113	0.282	0	0	0	0	0	0	0	0	0	0	0	0	0	0	0
KCNAB3	0	0	0	0	0	0.134	0	0	0	0	0	0	0	0	0	0.147
PTS-P1	0.157	0	0	0	0	0	0	0	0	0	0	0	0	0	0	0.124
EOMES	0	0	0	0	0	0	0	0	0	0	0.167	0	0	0	0.114	0
NPIPL3	0	0	0	0	0	0	0	0	0	0	0.123	0	0	0	0.158	0
C1ORF68	0.114	0	0	0	0	0	0	0	0	0	0	0	0	0	0.167	0

C20ORF4	0	0	0	0	0	0	0	0	0	0	0	0	0	0	0.281	0
FAM101A	0	0	0	0	0	0	0	0	0	0	0	0	0	0	0.281	0
LOC642355	0	0	0	0	0	0	0	0	0	0	0	0	0	0	0.281	0
PLSCR2	0	0	0	0	0	0	0	0	0	0	0	0	0	0	0.281	0
PTPMT1	0	0	0	0	0	0	0	0	0	0	0	0	0	0	0.281	0
VTNR	0	0	0	0	0	0	0	0	0	0	0	0	0	0	0.281	0
MSSD	0	0	0	0.163	0	0	0	0	0	0	0	0.118	0	0	0	0
KLK10	0	0	0	0	0.115	0	0	0	0.166	0	0	0	0	0	0	0
NNMT	0	0	0	0	0.281	0	0	0	0	0	0	0	0	0	0	0
CSGALNACT1	0	0	0	0.281	0	0	0	0	0	0	0	0	0	0	0	0
EXOC4	0	0	0	0.281	0	0	0	0	0	0	0	0	0	0	0	0
PLP1	0	0	0	0.281	0	0	0	0	0	0	0	0	0	0	0	0
SARS2	0	0	0	0.281	0	0	0	0	0	0	0	0	0	0	0	0
PPIAP18	0	0	0	0	0	0	0	0	0	0	0.102	0	0	0	0.179	0
SLC5A4	0.128	0	0	0	0	0	0	0	0	0	0	0	0	0	0	0.152
RBM17	0	0	0	0	0	0	0	0	0	0	0.118	0	0	0	0.162	0
GGTLC4P	0	0	0	0	0	0	0	0	0	0	0	0	0	0	0.28	0
IGKV1OR-3	0	0	0	0	0	0	0	0	0	0	0	0	0	0	0.28	0
EPG5	0	0	0	0	0	0	0	0	0.127	0	0.153	0	0	0	0	0
NEFLL1	0	0.117	0	0.163	0	0	0	0	0	0	0	0	0	0	0	0
TNKS1BP1	0.28	0	0	0	0	0	0	0	0	0	0	0	0	0	0	0
IDI1	0.175	0.105	0	0	0	0	0	0	0	0	0	0	0	0	0	0
PIGC	0	0	0	0	0	0	0	0	0	0	0	0	0	0	0	0.279
SLC2A12	0	0	0	0	0	0	0	0	0	0	0	0	0	0	0	0.279
WDR5B	0	0	0	0	0	0	0	0	0	0	0.162	0	0	0	0.117	0
CSMD2	0	0	0	0	0	0	0	0	0	0	0	0	0	0	0.279	0
FAM200A	0	0	0	0	0	0	0	0	0	0	0	0	0	0	0.279	0
LEPROTL1	0	0	0	0	0	0	0	0	0	0	0	0	0	0	0.279	0
SPRR2C	0	0	0	0	0	0	0	0	0	0	0	0	0	0	0.279	0
TRBV10-3	0	0	0	0	0	0	0	0	0	0	0	0	0	0	0.279	0
LIPA	0	0	0	0	0.132	0	0	0	0	0	0	0	0	0.147	0	0
DCTN4	0	0.154	0	0	0	0	0	0	0	0	0.125	0	0	0	0	0
NEDD9	0	0	0	0	0.279	0	0	0	0	0	0	0	0	0	0	0
SSNA1	0	0.111	0	0.168	0	0	0	0	0	0	0	0	0	0	0	0
LAMA2	0	0	0	0.279	0	0	0	0	0	0	0	0	0	0	0	0
MPDZ	0	0	0	0.279	0	0	0	0	0	0	0	0	0	0	0	0
METTL9	0	0	0	0	0	0	0	0	0	0	0	0	0	0	0	0.278
NUBPL	0	0	0	0	0	0	0	0	0	0	0	0	0	0	0	0.278
BCR	0	0	0	0	0	0	0	0	0	0	0	0	0	0	0.278	0
CCDC142	0	0	0	0	0	0	0	0	0	0	0	0	0	0	0.278	0
HLA-T	0	0	0	0	0	0	0	0	0	0	0	0	0	0	0.278	0

TRBV17	0	0	0	0	0	0	0	0	0	0	0	0	0	0	0.278	0
GJB1	0	0	0	0.124	0	0	0	0	0	0	0	0.154	0	0	0	0
NDOR1	0	0	0	0	0	0	0	0	0	0	0.278	0	0	0	0	0
LRP1B	0.108	0	0	0	0.17	0	0	0	0	0	0	0	0	0	0	0
NAPSA	0	0	0	0	0.278	0	0	0	0	0	0	0	0	0	0	0
ZNF536	0	0.109	0	0.169	0	0	0	0	0	0	0	0	0	0	0	0
MTMR11	0	0	0	0.278	0	0	0	0	0	0	0	0	0	0	0	0
SH3GL2	0	0	0	0	0	0	0	0	0	0	0.104	0	0	0	0.174	0
RER1	0	0	0	0	0.172	0	0	0	0.106	0	0	0	0	0	0	0
KCND3	0	0	0	0	0	0.132	0	0	0	0	0	0	0	0	0	0.145
BTBD9	0	0	0.14	0	0	0	0	0	0	0	0	0	0	0	0	0.137
PPP1R3C	0.123	0	0	0	0	0	0	0	0	0	0	0	0	0	0	0.154
VDAC1P2	0.126	0	0	0	0	0	0	0	0	0	0	0	0	0	0	0.151
C1QTNF2	0	0	0	0	0	0	0	0	0	0	0	0	0	0	0	0.277
DYNC1LI1	0	0	0	0	0	0	0	0	0	0	0.165	0	0	0	0.112	0
LOC100128651	0	0	0	0	0	0	0	0	0	0	0.162	0	0	0	0.115	0
SETD2	0	0	0	0	0	0	0	0	0	0	0.108	0	0	0	0.169	0
FAM190A	0	0	0.139	0	0	0	0	0	0	0	0	0	0.138	0	0	0
MTG1	0	0	0	0	0	0	0.115	0	0	0	0.162	0	0	0	0	0
HERC2P2	0	0	0	0	0	0.128	0.149	0	0	0	0	0	0	0	0	0
GLO1	0.121	0	0	0	0.156	0	0	0	0	0	0	0	0	0	0	0
SLC25A5P8	0.277	0	0	0	0	0	0	0	0	0	0	0	0	0	0	0
SLC5A11	0	0	0	0	0	0.174	0	0	0	0	0	0	0	0	0	0.103
FICD	0	0	0	0	0	0	0	0	0	0	0.161	0	0	0	0.115	0
SCML2	0	0	0	0	0	0	0	0	0	0	0.108	0	0	0	0.168	0
CCDC60	0	0	0.119	0	0	0	0	0	0	0	0	0	0	0	0.157	0
ATP5L	0	0	0	0	0	0	0	0	0	0	0	0	0	0	0.276	0
NDUFS4	0	0	0	0	0	0	0.125	0	0	0	0	0	0	0.151	0	0
MMACHC	0.124	0	0	0	0	0	0	0	0	0	0	0	0.152	0	0	0
PRCD	0	0	0	0	0	0	0	0	0	0.138	0	0.138	0	0	0	0
SLC27A2	0	0	0	0	0.113	0	0	0	0.163	0	0	0	0	0	0	0
ABCC2	0	0	0	0	0	0.132	0.144	0	0	0	0	0	0	0	0	0
SEN6A	0.276	0	0	0	0	0	0	0	0	0	0	0	0	0	0	0
KCNG2	0	0	0	0	0	0.132	0	0	0	0	0	0	0	0	0	0.143
RANGRF	0	0	0	0	0	0.158	0	0	0	0	0	0	0	0	0	0.117
LRRC8D	0	0	0	0	0	0	0	0	0	0	0.114	0	0	0	0.161	0
SPRR2E	0	0	0	0	0	0	0	0	0	0	0.108	0	0	0	0.167	0
MEF2D	0	0	0	0	0	0	0	0	0	0	0	0	0	0	0.275	0
ACCN2	0	0	0	0	0	0.166	0	0	0	0	0	0.109	0	0	0	0
HEXIM2	0	0	0	0	0.163	0	0	0	0.112	0	0	0	0	0	0	0
MS	0	0.113	0	0.162	0	0	0	0	0	0	0	0	0	0	0	0

NKX2-2	0.12	0.155	0	0	0	0	0	0	0	0	0	0	0	0	0	0
LOC400680	0	0	0	0	0	0	0	0	0	0	0	0	0	0	0	0.274
LRRC8B	0	0	0	0	0	0	0	0	0	0	0.114	0	0	0	0.16	0
NFE2L2	0	0	0	0	0	0	0	0	0	0	0.155	0	0	0.119	0	0
CNR1	0	0.133	0	0	0	0	0	0	0	0	0.141	0	0	0	0	0
KRT71	0	0	0	0	0	0.128	0.146	0	0	0	0	0	0	0	0	0
KLHL21	0.274	0	0	0	0	0	0	0	0	0	0	0	0	0	0	0
KCNB1	0	0	0	0	0	0.128	0	0	0	0	0	0	0	0	0	0.145
HSPA1L	0	0	0	0	0	0	0	0	0	0	0	0	0	0	0	0.273
MBNL3	0	0	0	0	0	0	0	0	0	0	0.107	0	0	0	0.166	0
NAA20	0	0	0	0	0	0	0	0	0	0	0.108	0	0	0	0.165	0
RAB11B	0	0	0	0	0	0	0	0	0	0	0.103	0	0	0	0.17	0
RCOR1	0	0	0	0	0	0	0	0	0	0	0.162	0	0	0	0.111	0
TGM4	0	0	0	0	0	0	0	0	0	0	0.167	0	0	0	0.106	0
SNORD36A	0	0	0	0	0	0	0	0	0	0	0	0	0	0	0.273	0
PRDX6	0	0.15	0	0	0	0	0	0	0	0	0	0	0	0.123	0	0
DFNM1	0	0	0	0	0	0	0	0	0	0	0	0.104	0.169	0	0	0
LOXHD1	0	0	0	0	0	0	0	0	0	0	0	0.12	0.153	0	0	0
PEX7	0	0	0	0	0	0	0	0	0	0	0.167	0	0.106	0	0	0
SLI1	0	0	0.141	0	0	0	0	0	0	0	0	0	0.132	0	0	0
LOC392787	0	0	0	0.163	0	0	0	0	0	0	0	0.11	0	0	0	0
NAV1	0	0	0	0.138	0	0	0	0	0	0	0	0.135	0	0	0	0
PURA	0	0	0	0	0.145	0	0	0	0.128	0	0	0	0	0	0	0
KDELR3	0	0	0	0.273	0	0	0	0	0	0	0	0	0	0	0	0
ATP1A1	0	0	0	0	0	0	0	0	0	0	0	0	0	0	0	0.272
AMDP1	0	0	0	0	0	0	0	0	0	0	0	0	0	0	0.272	0
EMX2OS	0	0	0	0	0	0	0	0	0	0	0	0	0	0	0.272	0
IGKV2OR22-4	0	0	0	0	0	0	0	0	0	0	0	0	0	0	0.272	0
SUCLG2	0	0	0	0	0	0	0	0	0	0	0	0	0	0	0.272	0
MACOM	0	0	0	0	0	0	0	0	0	0	0	0.146	0.126	0	0	0
ALKBH5	0	0	0	0	0	0	0	0	0	0	0	0.272	0	0	0	0
ABCG4	0	0	0	0	0.171	0	0	0	0.101	0	0	0	0	0	0	0
GAB2	0	0	0	0	0.163	0	0	0	0.109	0	0	0	0	0	0	0
LMNB2	0	0	0	0.272	0	0	0	0	0	0	0	0	0	0	0	0
CACNA2D3	0	0	0	0	0	0.153	0	0	0	0	0	0	0	0	0	0.118
KCNJ2	0	0	0	0	0	0.154	0	0	0	0	0	0	0	0	0	0.117
RPS6KA5	0	0	0	0	0	0	0	0	0	0	0.108	0	0	0	0.163	0
CTSL1P8	0	0	0	0	0	0	0	0	0	0	0	0	0	0	0.271	0
OAZ1	0	0	0	0	0	0	0	0	0	0	0	0	0	0	0.271	0
OR5I1	0	0	0	0	0	0	0	0	0	0	0	0	0	0	0.271	0
SPARCL1	0	0	0	0	0	0	0	0	0	0	0	0	0	0	0.271	0

ZSCAN21	0	0	0	0	0	0	0	0	0	0	0	0	0	0	0.271	0
DHDDS	0	0	0	0	0	0	0	0	0	0.157	0	0	0	0.114	0	0
OA3	0	0	0	0	0	0.109	0	0	0	0	0	0	0.162	0	0	0
ACSBG1	0	0	0	0	0.161	0	0	0	0.11	0	0	0	0	0	0	0
MDFIC	0	0	0	0	0.162	0	0	0	0.109	0	0	0	0	0	0	0
KIF20A	0	0	0	0.271	0	0	0	0	0	0	0	0	0	0	0	0
RAB3C	0	0	0	0.271	0	0	0	0	0	0	0	0	0	0	0	0
LATD	0	0.15	0	0	0	0	0	0	0	0	0	0	0	0	0	0.12
TSSK1A	0	0	0	0	0	0	0	0	0	0	0	0	0	0	0	0.27
RNY4P1	0	0	0	0	0	0	0	0	0	0	0.101	0	0	0	0.169	0
SLC2A4RG	0	0	0	0	0	0	0	0	0	0	0.109	0	0	0	0.161	0
PARP12	0	0	0	0	0	0	0	0	0	0	0	0	0	0	0.27	0
SIX3	0	0	0	0	0	0	0	0	0	0	0	0	0	0	0.27	0
MCOLN2	0	0	0	0	0	0	0	0	0	0	0	0.15	0	0.12	0	0
CCDC66	0	0	0	0	0	0	0	0	0	0.105	0	0	0	0.165	0	0
USH1C	0	0	0	0	0	0	0	0	0	0.15	0	0.12	0	0	0	0
PSHK2	0	0	0	0	0	0	0	0	0.137	0	0	0.133	0	0	0	0
SEMA6C	0.117	0	0	0	0	0	0	0	0	0	0	0.153	0	0	0	0
MDGA1	0	0	0.125	0	0	0.145	0	0	0	0	0	0	0	0	0	0
KLHL24	0	0.121	0	0	0	0.149	0	0	0	0	0	0	0	0	0	0
EPM2AIP1	0.142	0	0	0	0	0.128	0	0	0	0	0	0	0	0	0	0
BHLHA15	0	0	0	0.27	0	0	0	0	0	0	0	0	0	0	0	0
IGHV1OR15-1	0	0	0	0.27	0	0	0	0	0	0	0	0	0	0	0	0
TPCN1	0	0	0	0	0	0.15	0	0	0	0	0	0	0	0	0	0.119
BULN	0.157	0	0	0	0	0	0	0	0	0	0	0	0	0	0	0.112
RAB11A	0	0	0	0	0	0	0	0	0	0	0.163	0	0	0	0.106	0
CER1	0	0	0	0	0	0	0	0	0	0	0	0	0	0	0.269	0
RRM2P3	0	0	0	0	0	0	0	0	0	0	0	0	0	0	0.269	0
ZFYVE21	0	0	0	0	0	0	0	0	0	0	0	0	0	0	0.269	0
ANP32A	0	0	0	0	0	0	0	0	0	0	0.164	0	0	0.105	0	0
PLEKHB1	0	0	0	0	0	0	0	0	0	0.127	0	0.142	0	0	0	0
ADD1	0	0	0	0	0	0	0	0	0	0	0.269	0	0	0	0	0
FOXB1	0	0.146	0	0	0	0	0	0	0	0.123	0	0	0	0	0	0
KCNG3	0	0	0	0	0	0.115	0	0.154	0	0	0	0	0	0	0	0
GJB7	0	0	0	0.269	0	0	0	0	0	0	0	0	0	0	0	0
RAB15	0	0	0	0.269	0	0	0	0	0	0	0	0	0	0	0	0
DRD2	0	0	0	0	0	0	0	0	0	0.125	0	0	0	0	0	0.143
LOC401959	0.12	0	0	0	0	0	0	0	0	0	0	0	0	0	0	0.148
BDNF-AS1	0	0	0	0	0	0	0	0	0	0	0.163	0	0	0	0.105	0
HPYR1	0	0	0	0	0	0	0	0	0	0	0.167	0	0	0	0.101	0
MAPK8IPP	0	0	0	0	0	0	0	0	0	0	0.151	0	0	0	0.117	0

PAH	0	0	0.139	0	0	0	0	0	0	0	0	0	0.129	0	0	0
CREB3L2	0	0.168	0	0	0	0	0	0	0.1	0	0	0	0	0	0	0
FXYD6	0	0	0	0	0	0.159	0	0.109	0	0	0	0	0	0	0	0
CACNG6	0	0	0	0	0	0.158	0	0	0	0	0	0	0	0	0	0.109
LOC317727	0.129	0	0	0	0	0	0	0	0	0	0	0	0	0	0	0.138
AK7	0	0	0	0	0	0	0	0	0	0	0	0	0	0	0	0.267
FAS	0	0	0	0	0	0	0	0	0	0	0	0	0	0	0	0.267
MPP2	0	0	0	0	0	0	0	0	0	0	0	0	0	0	0	0.267
PIGF	0	0	0	0	0	0	0	0	0	0	0	0	0	0	0	0.267
ITPR1	0	0	0	0	0	0	0	0	0	0	0.152	0	0	0	0.115	0
C6ORF1	0	0	0	0	0	0	0	0	0	0	0	0	0	0	0.267	0
C20ORF103	0	0	0	0	0	0.116	0	0	0	0	0	0	0	0.151	0	0
ALMS1	0	0	0	0	0	0	0	0	0	0	0	0.134	0.133	0	0	0
EYA4	0	0	0	0	0	0	0	0	0	0	0	0.156	0.111	0	0	0
ALG6	0	0	0	0	0	0	0.153	0	0	0	0	0	0.114	0	0	0
TRMU	0	0	0	0	0	0	0.104	0	0	0	0	0.163	0	0	0	0
C3	0	0.145	0	0	0	0	0	0	0	0	0	0	0	0	0	0.121
GIPC1	0	0	0	0	0	0	0	0	0	0	0	0	0	0	0	0.266
SPAG16	0	0	0	0	0	0	0	0	0	0	0	0	0	0	0	0.266
MAP3K6	0	0	0	0	0	0	0	0	0	0	0.149	0	0	0	0.117	0
CELIAC2	0	0.149	0	0	0	0	0	0	0	0	0	0	0	0	0.117	0
NPM3	0	0	0	0	0	0	0	0	0	0	0	0	0	0	0.266	0
RFPL2	0	0	0	0	0	0	0	0	0	0	0	0	0	0	0.266	0
SPARC	0	0	0	0	0	0	0	0	0	0	0	0	0	0	0.266	0
TRBV4-2	0	0	0	0	0	0	0	0	0	0	0	0	0	0	0.266	0
RRNAD1	0	0	0	0	0.138	0	0	0	0.128	0	0	0	0	0	0	0
PLCL2	0	0	0	0	0	0.128	0.138	0	0	0	0	0	0	0	0	0
RDX	0	0.14	0	0	0	0.126	0	0	0	0	0	0	0	0	0	0
RCN2	0.138	0	0	0	0	0.128	0	0	0	0	0	0	0	0	0	0
FGD2	0	0	0	0.266	0	0	0	0	0	0	0	0	0	0	0	0
KIF13B	0	0	0	0.266	0	0	0	0	0	0	0	0	0	0	0	0
MTMR12	0	0	0	0.266	0	0	0	0	0	0	0	0	0	0	0	0
SOD2	0	0	0	0	0	0	0.11	0	0	0	0	0	0	0	0	0.155
KCNA7	0	0	0	0	0	0.163	0	0	0	0	0	0	0	0	0	0.102
SLC6A17	0	0	0	0	0	0.148	0	0	0	0	0	0	0	0	0.117	0
PACSIN2	0	0	0	0	0	0	0	0	0	0	0	0	0	0	0.265	0
RPH3AL	0	0	0	0	0	0	0	0	0	0	0	0	0	0	0.265	0
SRRD	0	0	0	0	0	0	0	0	0	0	0	0	0	0	0.265	0
MRPL20	0	0	0	0	0	0	0	0	0	0	0.16	0	0	0.105	0	0
MYP1	0	0	0	0	0	0	0	0	0	0.137	0	0	0.128	0	0	0
ST8SIA4	0	0	0.143	0	0	0	0	0	0	0	0	0	0.122	0	0	0

DFNB31	0	0	0	0	0	0	0	0	0	0.145	0	0.12	0	0	0	0
USH2A	0	0	0	0	0	0	0	0	0	0.143	0	0.122	0	0	0	0
TRNT	0	0	0	0	0	0	0.134	0	0	0	0	0.131	0	0	0	0
LPPR3	0	0	0	0.144	0	0	0	0	0	0	0	0.121	0	0	0	0
PRPS1L1	0	0	0	0	0	0	0.163	0	0	0	0.102	0	0	0	0	0
PSRC1	0.165	0	0	0	0	0	0	0	0.1	0	0	0	0	0	0	0
DOC2A	0	0	0.129	0	0	0.136	0	0	0	0	0	0	0	0	0	0
ARHGEF10	0	0	0	0.265	0	0	0	0	0	0	0	0	0	0	0	0
GJA10	0	0	0	0.265	0	0	0	0	0	0	0	0	0	0	0	0
LMNA	0	0	0	0.265	0	0	0	0	0	0	0	0	0	0	0	0
MB	0	0	0	0	0	0	0	0	0	0	0	0	0	0	0	0.264
PIGH	0	0	0	0	0	0	0	0	0	0	0	0	0	0	0	0.264
ACYP2	0	0	0	0	0	0	0	0	0	0	0	0	0	0	0.264	0
POTEJ	0	0	0	0	0	0	0	0	0	0	0	0	0	0	0.264	0
POTEKP	0	0	0	0	0	0	0	0	0	0	0	0	0	0	0.264	0
GABARAPL3	0	0	0	0	0	0.149	0.115	0	0	0	0	0	0	0	0	0
DNM2	0	0	0	0.264	0	0	0	0	0	0	0	0	0	0	0	0
DOCK5	0	0	0	0.264	0	0	0	0	0	0	0	0	0	0	0	0
SERINC5	0	0	0	0.264	0	0	0	0	0	0	0	0	0	0	0	0
NKX2-4	0	0	0	0	0	0	0	0	0	0	0.104	0	0	0	0.159	0
BMP6P1	0.105	0	0	0	0	0	0	0	0	0	0	0	0	0	0.158	0
THAP7	0	0	0	0	0	0	0	0	0	0	0	0	0	0	0.263	0
COX15	0	0	0	0	0	0	0.111	0	0	0	0	0	0	0.152	0	0
MTERFD1	0	0	0	0	0	0	0.111	0	0	0	0	0	0	0.152	0	0
RP32	0	0	0	0	0	0	0	0	0	0.161	0	0.102	0	0	0	0
NPY	0	0	0	0	0	0.159	0	0	0	0	0.104	0	0	0	0	0
MIR1469	0	0	0	0	0	0	0	0	0	0	0.263	0	0	0	0	0
EPHA4	0	0.145	0	0	0	0	0	0	0	0.118	0	0	0	0	0	0
NIF3L1	0	0.137	0	0	0	0	0	0	0.126	0	0	0	0	0	0	0
MAMLD1	0	0	0	0.263	0	0	0	0	0	0	0	0	0	0	0	0
NIPSNAP3B	0.263	0	0	0	0	0	0	0	0	0	0	0	0	0	0	0
C9ORF16	0	0	0	0	0	0	0	0	0	0	0.162	0	0	0	0.1	0
ARGFX	0	0	0	0	0	0	0	0	0	0	0	0	0	0	0.262	0
F8A3	0	0	0	0	0	0	0	0	0	0	0	0	0	0	0.262	0
FKBP14	0	0	0	0	0	0	0	0	0	0	0	0	0	0	0.262	0
OTC	0	0	0	0	0	0	0.147	0	0	0	0	0	0.115	0	0	0
TRNE	0	0	0	0	0	0	0.151	0	0	0	0	0.111	0	0	0	0
SETDB1	0	0	0	0	0	0	0	0	0	0	0.262	0	0	0	0	0
SNX6	0	0	0	0	0.151	0	0	0	0.111	0	0	0	0	0	0	0
C11ORF10	0	0	0	0	0	0	0	0	0	0	0	0	0	0	0.261	0
IGHV1-3	0	0	0	0	0	0	0	0	0	0	0	0	0	0	0.261	0

PROA	0	0	0	0	0	0	0	0	0	0	0	0	0	0	0.261	0
FAM126A	0	0	0	0.128	0	0	0	0	0	0	0	0.133	0	0	0	0
MICU1	0	0	0	0	0	0	0	0	0	0	0.261	0	0	0	0	0
CHCHD2P10	0	0.144	0	0	0	0	0	0	0.117	0	0	0	0	0	0	0
EMD	0	0	0	0.261	0	0	0	0	0	0	0	0	0	0	0	0
KCNK2	0	0	0	0	0	0.143	0	0	0	0	0	0	0	0	0	0.117
KCNMA1	0	0	0	0	0	0.143	0	0	0	0	0	0	0	0	0	0.117
ASS1P4	0	0	0	0	0	0	0	0	0	0	0	0	0	0	0.26	0
HLA-DRB8	0	0	0	0	0	0	0	0	0	0	0	0	0	0	0.26	0
LOC2748	0	0	0	0	0	0	0	0	0	0	0	0	0	0	0.26	0
NSD1	0	0	0	0	0	0	0	0	0	0	0	0	0.26	0	0	0
TMEM87A	0	0	0	0	0	0.158	0.102	0	0	0	0	0	0	0	0	0
CETP	0.102	0	0	0	0.158	0	0	0	0	0	0	0	0	0	0	0
ZCWPW1	0.154	0	0	0.106	0	0	0	0	0	0	0	0	0	0	0	0
TSPAN4	0.26	0	0	0	0	0	0	0	0	0	0	0	0	0	0	0
RIMS4	0	0	0	0	0	0.106	0	0	0	0	0	0	0	0	0	0.153
LRRTM2	0	0	0	0	0	0	0	0	0	0	0	0	0	0	0.259	0
RFPL3	0	0	0	0	0	0	0	0	0	0	0	0	0	0	0.259	0
TECTA	0	0	0	0	0	0	0	0	0	0	0	0.102	0.157	0	0	0
C2ORF71	0	0	0	0	0	0	0	0	0	0.137	0	0.122	0	0	0	0
GRK5	0	0	0	0	0.149	0	0	0	0.11	0	0	0	0	0	0	0
UBE3AP2	0	0	0	0	0	0.145	0.114	0	0	0	0	0	0	0	0	0
FHL1	0	0	0	0.259	0	0	0	0	0	0	0	0	0	0	0	0
CSPG4	0.137	0.122	0	0	0	0	0	0	0	0	0	0	0	0	0	0
NPC1L1	0.259	0	0	0	0	0	0	0	0	0	0	0	0	0	0	0
HTR1D	0	0	0	0	0	0	0	0	0	0	0	0	0	0	0	0.258
DUX4L15	0	0	0	0.147	0	0	0	0	0	0	0	0	0	0	0.111	0
ARL17B	0	0	0	0	0	0	0	0	0	0	0	0	0	0	0.258	0
EDDM3CP	0	0	0	0	0	0	0	0	0	0	0	0	0	0	0.258	0
RCVRN	0	0	0	0	0	0	0	0	0	0.258	0	0	0	0	0	0
SYNCRIP	0	0.113	0	0	0	0	0	0	0.145	0	0	0	0	0	0	0
KCNT1	0	0	0	0	0	0.147	0	0.111	0	0	0	0	0	0	0	0
KCNT2	0	0	0	0	0	0.155	0	0.103	0	0	0	0	0	0	0	0
KCNH7	0	0	0	0	0	0.111	0	0	0	0	0	0	0	0	0	0.146
EXTL2P1	0	0	0	0	0	0	0	0	0	0	0	0	0	0	0	0.257
TNNC1	0	0	0	0	0	0	0	0	0	0	0	0	0	0	0	0.257
HMGXB3	0	0	0	0	0	0	0	0	0	0	0.156	0	0	0	0.101	0
OAZ3	0	0	0	0	0	0	0	0	0	0	0	0	0	0	0.257	0
NAAA	0	0	0	0	0	0	0	0	0	0	0.154	0	0	0.103	0	0
NDUFA12	0	0	0	0	0	0	0.108	0	0	0	0	0	0	0.149	0	0
TIMM23B	0	0	0	0	0	0	0.106	0	0	0	0	0	0	0.151	0	0

CHDS2	0	0	0.133	0	0	0	0	0	0	0	0	0	0.124	0	0	0
MYO3A	0	0	0	0	0	0	0	0	0	0.122	0	0.135	0	0	0	0
HHC2	0	0	0	0	0	0	0	0	0.15	0	0	0.107	0	0	0	0
MPP6	0	0	0	0	0	0	0	0	0	0	0	0.257	0	0	0	0
KCTD2	0	0	0	0	0	0	0	0	0	0.102	0.155	0	0	0	0	0
PRSS57	0.102	0	0	0	0	0	0	0	0	0	0.155	0	0	0	0	0
FAF2	0	0	0	0	0.136	0	0	0	0.121	0	0	0	0	0	0	0
ZMYM6	0	0	0	0	0.139	0	0	0	0.118	0	0	0	0	0	0	0
NPTN	0	0	0.129	0	0	0.128	0	0	0	0	0	0	0	0	0	0
ARVCF	0	0	0.119	0	0	0	0	0	0	0	0	0	0	0	0	0.137
CKBE	0.1	0	0	0	0	0	0	0	0	0	0	0	0	0	0	0.156
GYS1	0.138	0	0	0	0	0	0	0	0	0	0	0	0	0	0	0.118
DNAH7	0	0	0	0	0	0	0	0	0	0	0	0	0	0	0	0.256
DNAI2	0	0	0	0	0	0	0	0	0	0	0	0	0	0	0	0.256
DOLK	0	0	0	0	0	0	0	0	0	0	0	0	0	0	0	0.256
DPCD	0	0	0	0	0	0	0	0	0	0	0	0	0	0	0	0.256
KCNE2	0	0	0	0	0	0	0	0	0	0	0	0	0	0	0	0.256
XPNPEP3	0	0	0	0	0	0	0	0	0	0	0	0	0	0	0	0.256
NTAN1	0	0	0.138	0	0	0	0	0	0	0	0	0	0.118	0	0	0
WHAMMP2	0	0	0	0	0	0.139	0.117	0	0	0	0	0	0	0	0	0
MAMSTR	0.256	0	0	0	0	0	0	0	0	0	0	0	0	0	0	0
DNAH11	0	0.147	0	0	0	0	0	0	0	0	0	0	0	0	0	0.108
RCOR2	0	0	0	0	0	0	0	0	0	0	0.137	0	0	0	0.118	0
PLCD1	0	0	0	0	0	0	0	0	0	0	0	0	0	0	0.255	0
ATG4C	0	0	0	0	0	0	0	0	0	0	0.147	0	0	0.108	0	0
GABARAP	0	0	0	0	0	0.148	0	0	0	0	0	0	0	0.107	0	0
SMPD4	0	0	0	0	0.132	0	0	0	0	0	0	0	0	0.123	0	0
SHFM2	0	0	0	0	0	0	0	0	0	0	0	0	0.255	0	0	0
CLRN1	0	0	0	0	0	0	0	0	0	0.111	0	0.144	0	0	0	0
NLRC4	0	0	0	0	0	0	0	0	0	0	0.255	0	0	0	0	0
NMTC1	0	0.103	0	0	0	0	0	0	0.152	0	0	0	0	0	0	0
NCDN	0	0	0	0	0	0.255	0	0	0	0	0	0	0	0	0	0
COPD2	0	0	0	0.255	0	0	0	0	0	0	0	0	0	0	0	0
TYRO3P	0.255	0	0	0	0	0	0	0	0	0	0	0	0	0	0	0
B9D1	0	0	0	0	0	0	0	0	0	0	0	0.134	0	0	0	0.12
ATP8B3	0	0	0	0	0	0	0	0	0	0	0	0	0	0	0	0.254
C11ORF65	0	0	0	0	0	0	0	0	0	0	0	0	0	0	0	0.254
NPR3	0	0	0	0	0	0	0	0	0	0	0	0	0	0	0	0.254
SH3GL1P3	0	0	0	0	0	0	0	0	0	0	0.147	0	0	0	0.107	0
SLC25A18	0	0	0	0	0	0	0.147	0	0	0	0	0	0	0	0.107	0
HOTAIRM1	0	0	0	0	0	0	0	0	0	0	0	0	0	0	0.254	0

TRAPPC9	0	0	0	0	0	0	0	0	0	0	0	0	0.254	0	0	0
THBS2	0.254	0	0	0	0	0	0	0	0	0	0	0	0	0	0	0
VDAC2	0.254	0	0	0	0	0	0	0	0	0	0	0	0	0	0	0
PLAU	0	0	0	0	0.134	0	0	0	0	0	0	0	0	0	0	0.119
ATOD6	0	0	0	0	0	0	0	0	0	0	0	0	0	0	0.253	0
CLC	0	0	0	0	0	0	0	0	0	0	0	0	0	0	0.253	0
SDCCAG3	0	0	0	0	0	0	0	0	0	0	0	0	0	0	0.253	0
NTRK2	0	0	0	0	0	0	0	0	0	0.145	0.108	0	0	0	0	0
KCNK15	0	0	0	0	0	0.144	0	0.109	0	0	0	0	0	0	0	0
SLC6A7	0	0	0	0	0	0.147	0	0.106	0	0	0	0	0	0	0	0
HSPB1P2	0	0	0	0.253	0	0	0	0	0	0	0	0	0	0	0	0
SLC2A3	0	0	0	0	0	0	0.113	0	0	0	0	0	0	0	0	0.139
GDF1	0	0.144	0	0	0	0	0	0	0	0	0	0	0	0	0	0.108
GUCY1B2	0	0	0	0	0	0	0	0	0	0	0	0	0	0	0	0.252
RAB8A	0	0	0	0	0	0	0	0	0	0	0.145	0	0	0	0.107	0
RAX	0	0	0	0	0	0	0	0	0	0.134	0	0	0	0	0.118	0
SPTLC2	0	0	0	0.145	0	0	0	0	0	0	0	0	0	0.107	0	0
DFNA54	0	0	0	0	0	0	0	0	0	0	0	0.104	0.148	0	0	0
PCDH11Y	0	0	0.142	0	0	0	0	0	0	0	0	0	0.11	0	0	0
BICD1	0	0.116	0	0	0	0	0	0	0	0	0.136	0	0	0	0	0
NDEL1	0	0.13	0	0	0	0	0	0	0	0	0.122	0	0	0	0	0
APOA1	0	0	0	0	0.141	0	0	0	0.111	0	0	0	0	0	0	0
DHX30	0	0	0	0	0.141	0	0	0	0.111	0	0	0	0	0	0	0
RALGAPA1	0	0	0	0	0	0.147	0	0.105	0	0	0	0	0	0	0	0
KCNE3	0	0	0	0	0	0.133	0	0	0	0	0	0	0	0	0	0.118
PITX2	0	0.108	0	0	0	0	0	0	0	0	0	0	0	0	0	0.143
ATP2A1	0	0	0	0	0	0	0	0	0	0	0	0	0	0	0	0.251
PI3	0	0	0	0	0	0	0	0	0	0	0	0	0	0	0.251	0
HSPA8	0	0.12	0	0	0	0	0	0	0	0	0	0	0	0.131	0	0
TKCR	0	0	0	0	0	0	0.125	0	0	0	0	0	0.126	0	0	0
GALT	0	0	0	0	0	0	0	0	0	0	0	0	0.251	0	0	0
CLRN3	0	0	0	0	0	0	0	0	0	0.131	0	0.12	0	0	0	0
DFNB20	0	0	0	0	0	0	0	0	0	0.131	0	0.12	0	0	0	0
CLK4	0	0	0	0	0.139	0	0	0	0.112	0	0	0	0	0	0	0
KLK3	0	0	0	0	0	0	0	0	0.251	0	0	0	0	0	0	0
USP46	0	0	0.129	0	0	0.122	0	0	0	0	0	0	0	0	0	0
CNST	0	0	0	0.251	0	0	0	0	0	0	0	0	0	0	0	0
ILKAP	0.251	0	0	0	0	0	0	0	0	0	0	0	0	0	0	0
TREML4	0.251	0	0	0	0	0	0	0	0	0	0	0	0	0	0	0
HK2	0.131	0	0	0	0	0	0	0	0	0	0	0	0	0	0	0.119
VDAC1P5	0.107	0	0	0	0	0	0	0	0	0	0	0	0	0	0	0.143

DTNA	0	0	0	0	0	0	0	0	0	0	0	0	0	0	0	0.25
RSPH4A	0	0	0	0	0	0	0	0	0	0	0	0	0	0	0	0.25
DNAJC5B	0	0	0	0	0	0	0	0	0	0	0	0	0	0	0.25	0
HIST1H2AJ	0	0	0	0	0	0	0	0	0	0	0	0	0	0	0.25	0
OTX2	0	0	0	0	0	0	0	0	0	0	0	0	0	0	0.25	0
SPRED1	0	0	0.142	0	0	0	0	0	0	0	0	0	0.108	0	0	0
CCV	0	0	0	0	0	0	0	0	0	0	0.147	0.103	0	0	0	0
HIC2	0	0	0	0	0.146	0	0	0	0.104	0	0	0	0	0	0	0
ZNF259	0	0.115	0	0	0	0	0	0	0.135	0	0	0	0	0	0	0
SNF8	0	0	0	0	0	0	0	0	0.25	0	0	0	0	0	0	0
KCNK10	0	0	0	0	0	0.144	0	0.106	0	0	0	0	0	0	0	0
SV2B	0	0	0	0	0	0.137	0	0.113	0	0	0	0	0	0	0	0
ARHGAP33	0	0	0	0.25	0	0	0	0	0	0	0	0	0	0	0	0
MRI1	0.25	0	0	0	0	0	0	0	0	0	0	0	0	0	0	0
NIPAL3	0	0	0	0	0	0	0.12	0	0	0	0	0	0	0	0	0.129
DMD	0	0	0	0	0	0	0	0	0	0	0	0	0	0	0	0.249
DNAAF1	0	0	0	0	0	0	0	0	0	0	0	0	0	0	0	0.249
DNAH9	0	0	0	0	0	0	0	0	0	0	0	0	0	0	0	0.249
DNALI1	0	0	0	0	0	0	0	0	0	0	0	0	0	0	0	0.249
ANXA4	0	0	0	0	0	0	0	0	0	0	0	0	0	0	0.249	0
CDAGS	0	0	0	0	0	0	0	0	0	0.147	0	0	0.102	0	0	0
EYS	0	0	0	0	0	0	0	0	0	0.113	0	0.136	0	0	0	0
IFT140	0	0	0	0	0	0	0	0	0	0.139	0	0.11	0	0	0	0
TUFM	0	0	0	0	0	0	0.147	0	0	0	0	0.102	0	0	0	0
SIX5	0	0	0	0	0	0	0	0	0	0	0.249	0	0	0	0	0
LINC00470	0	0	0	0	0	0	0	0	0	0.249	0	0	0	0	0	0
GPX5	0	0	0	0	0.139	0	0	0	0.11	0	0	0	0	0	0	0
PSD	0	0	0	0	0	0.119	0	0.13	0	0	0	0	0	0	0	0
SIK1	0.118	0	0	0	0	0	0	0	0	0	0	0	0	0	0	0.13
CKM	0	0	0	0	0	0	0	0	0	0	0	0	0	0	0	0.248
OR1F2P	0	0	0	0	0	0	0	0	0	0	0	0	0	0	0	0.248
PAICSP1	0	0	0	0	0	0	0	0	0	0	0	0	0	0	0	0.248
PCDP1	0	0	0	0	0	0	0	0	0	0	0	0	0	0	0	0.248
PDLIM5	0	0	0	0	0	0	0	0	0	0	0	0	0	0	0	0.248
SLC2A11	0	0	0	0	0	0	0	0	0	0	0	0	0	0	0	0.248
SLC2A6	0	0	0	0	0	0	0	0	0	0	0	0	0	0	0	0.248
COX6A1P2	0	0	0	0	0	0	0	0	0	0	0	0	0	0	0.248	0
PLA2G2F	0	0	0	0	0	0	0	0	0	0	0	0	0	0	0.248	0
XBP1	0	0.118	0	0	0	0	0	0	0	0	0	0	0	0.13	0	0
GPR55	0	0	0	0	0	0	0	0	0	0	0.248	0	0	0	0	0
CCR5	0	0	0	0	0.14	0	0	0	0.108	0	0	0	0	0	0	0

CDK9	0	0	0	0	0.138	0	0	0	0.11	0	0	0	0	0	0	0
UBXN6	0	0	0	0	0.114	0	0	0	0.134	0	0	0	0	0	0	0
COPD1	0	0	0	0.248	0	0	0	0	0	0	0	0	0	0	0	0
LCAT	0.248	0	0	0	0	0	0	0	0	0	0	0	0	0	0	0
ZNF32	0.248	0	0	0	0	0	0	0	0	0	0	0	0	0	0	0
STRADA	0	0	0	0	0	0.139	0	0	0	0	0	0	0	0	0	0.108
VDAC1P1	0.107	0	0	0	0	0	0	0	0	0	0	0	0	0	0	0.14
C22ORF23	0	0	0	0	0	0	0	0	0	0	0	0	0	0	0.247	0
IVL	0	0	0	0	0	0	0	0	0	0	0	0	0	0	0.247	0
LOC100130015	0	0	0	0	0	0	0	0	0	0	0	0	0	0	0.247	0
MN1	0	0	0	0	0	0	0	0	0	0	0	0	0	0	0.247	0
SLC38A7	0	0	0	0	0	0	0	0	0	0	0	0	0	0	0.247	0
PSMD1	0	0	0	0	0	0	0	0	0	0	0.142	0	0	0.105	0	0
IREB2	0	0	0	0	0	0	0	0	0	0.124	0	0	0	0.123	0	0
LOC728637	0	0	0.137	0	0	0	0	0	0	0	0	0	0.11	0	0	0
TEC	0	0	0	0	0	0.131	0	0	0	0	0	0.116	0	0	0	0
FABP7	0.123	0	0	0	0	0	0	0	0	0.124	0	0	0	0	0	0
IPCEF1	0	0	0	0.247	0	0	0	0	0	0	0	0	0	0	0	0
SLC35E1	0	0	0	0.247	0	0	0	0	0	0	0	0	0	0	0	0
SONP1	0	0	0	0.247	0	0	0	0	0	0	0	0	0	0	0	0
MIRLET7B	0.247	0	0	0	0	0	0	0	0	0	0	0	0	0	0	0
CCDC39	0	0	0	0	0	0	0	0	0	0	0	0	0	0	0	0.246
MIRLET7BHG	0	0	0	0	0	0	0	0	0	0	0	0	0	0.13	0.116	0
CRCS9	0	0	0	0	0	0	0	0	0	0	0	0	0	0	0.246	0
FAM101B	0	0	0	0	0	0	0	0	0	0	0	0	0	0	0.246	0
PSORS1C2	0	0	0	0	0	0	0	0	0	0	0	0	0	0	0.246	0
RNASEH1P2	0	0	0	0	0	0	0	0	0	0	0	0	0	0	0.246	0
C7ORF10	0	0	0	0	0	0	0.12	0	0	0	0	0	0.126	0	0	0
RP1	0	0	0	0	0	0	0	0	0	0.133	0	0.113	0	0	0	0
SUPT5H	0	0	0	0	0.136	0	0	0	0.11	0	0	0	0	0	0	0
EPB41L3	0	0	0	0.246	0	0	0	0	0	0	0	0	0	0	0	0
FRMPD2	0	0	0	0.246	0	0	0	0	0	0	0	0	0	0	0	0
HMGXB4	0.246	0	0	0	0	0	0	0	0	0	0	0	0	0	0	0
LRRC26	0	0	0	0	0	0.144	0	0	0	0	0	0	0	0	0	0.101
CALM1P2	0	0	0	0	0	0	0	0	0	0	0.13	0	0	0	0.115	0
LINC00302	0.101	0	0	0	0	0	0	0	0	0	0	0	0	0	0.144	0
OR10C1	0	0	0	0	0	0	0	0	0	0	0	0	0	0	0.245	0
POC1B	0	0	0	0	0	0	0	0	0	0	0	0	0	0	0.245	0
POMZP3	0	0	0	0	0	0	0	0	0	0	0	0	0	0	0.245	0
CTSS	0	0.134	0	0	0	0	0	0	0	0	0	0	0	0.111	0	0
GATM	0	0	0	0	0	0	0.103	0	0	0	0	0	0.142	0	0	0

DGCR8	0	0	0.11	0	0	0	0	0	0	0	0	0	0.135	0	0	0
TSNAX	0	0	0.142	0	0	0	0	0	0	0	0	0	0.103	0	0	0
NDUFS2	0	0	0	0	0	0	0.108	0	0	0	0	0.137	0	0	0	0
NOP56	0	0.105	0	0	0	0	0	0	0	0	0.14	0	0	0	0	0
PEBP1	0	0	0	0	0.141	0	0	0	0.104	0	0	0	0	0	0	0
KDSR	0	0.119	0	0	0	0	0	0	0.126	0	0	0	0	0	0	0
TRAPPC10	0	0	0	0	0	0.143	0.102	0	0	0	0	0	0	0	0	0
CXCL14	0	0	0	0.245	0	0	0	0	0	0	0	0	0	0	0	0
DAG1	0	0	0	0.245	0	0	0	0	0	0	0	0	0	0	0	0
LIMS2	0.245	0	0	0	0	0	0	0	0	0	0	0	0	0	0	0
HK1	0	0	0	0	0	0	0	0	0	0	0	0	0	0	0	0.244
TUBB	0	0	0	0	0	0	0	0	0	0	0.138	0	0	0	0.106	0
ERI2	0	0	0	0	0	0	0	0	0	0	0	0	0	0	0.244	0
LRSL	0	0	0	0	0	0	0.13	0	0	0	0	0	0.114	0	0	0
ALDH7A1P4	0	0	0	0	0	0	0	0	0	0	0	0.244	0	0	0	0
PDE6B	0	0	0	0	0	0	0	0	0	0.106	0.138	0	0	0	0	0
HNRNPR	0	0.102	0	0	0	0	0	0	0.142	0	0	0	0	0	0	0
GBD3	0.104	0	0	0	0	0	0	0	0.14	0	0	0	0	0	0	0
KCNH4	0	0	0	0	0	0.133	0	0.111	0	0	0	0	0	0	0	0
ASPG2	0	0	0	0	0	0.132	0.112	0	0	0	0	0	0	0	0	0
PLD1	0	0	0	0	0	0.244	0	0	0	0	0	0	0	0	0	0
HCRTR2	0	0.112	0	0	0.132	0	0	0	0	0	0	0	0	0	0	0
GHSR	0	0	0	0	0	0	0	0	0	0	0	0	0	0	0	0.243
LGALS3BP	0	0	0	0	0	0	0	0	0	0	0	0	0	0	0.243	0
NECAB2	0	0	0	0	0	0	0	0	0	0	0	0	0	0	0.243	0
OR12D2	0	0	0	0	0	0	0	0	0	0	0	0	0	0	0.243	0
ZNF248	0	0	0	0	0	0	0	0	0	0	0	0	0	0	0.243	0
ACTR1A	0	0.1	0	0	0	0	0	0	0	0	0.143	0	0	0	0	0
MAPK8IP3	0	0	0	0	0	0	0	0	0	0	0.243	0	0	0	0	0
TIAL1	0	0.134	0	0	0	0	0	0	0.109	0	0	0	0	0	0	0
RAB20	0	0	0	0.243	0	0	0	0	0	0	0	0	0	0	0	0
PPP1R3A	0.243	0	0	0	0	0	0	0	0	0	0	0	0	0	0	0
GUCY1A3	0.113	0	0	0	0	0	0	0	0	0	0	0	0	0	0	0.129
LGALS4	0	0	0	0	0	0	0	0	0	0	0	0	0	0	0.242	0
LHX2	0	0	0	0	0	0	0	0	0	0	0	0	0	0	0.242	0
MIR522	0	0	0	0	0	0	0	0	0	0	0	0	0	0	0.242	0
KLHL17	0	0	0	0	0	0.132	0	0	0	0	0	0	0	0.11	0	0
TCTN2	0	0	0	0	0	0	0	0	0	0	0	0.122	0.12	0	0	0
KIAA0355	0	0	0.137	0	0	0	0	0	0	0	0	0	0.105	0	0	0
CRELD1	0	0	0	0	0	0.13	0.112	0	0	0	0	0	0	0	0	0
PTGDS	0	0	0	0	0.242	0	0	0	0	0	0	0	0	0	0	0

SERHL2	0	0	0	0.242	0	0	0	0	0	0	0	0	0	0	0	0
ZNF709	0	0	0	0.242	0	0	0	0	0	0	0	0	0	0	0	0
B3GNT4	0.104	0	0	0	0	0	0	0	0	0	0	0	0	0	0	0.137
ZNF429	0	0	0	0	0	0	0	0	0	0	0	0	0	0	0	0.241
KIAA1430	0	0	0	0	0	0	0	0	0	0	0	0	0	0	0.241	0
BAG1	0	0	0	0	0.134	0	0	0	0	0	0	0	0	0.107	0	0
HMGB1	0	0	0	0	0.134	0	0	0	0	0	0	0	0	0.107	0	0
CHDS3	0	0	0.138	0	0	0	0	0	0	0	0	0	0.103	0	0	0
ACAN	0	0.102	0	0	0	0	0	0	0	0.139	0	0	0	0	0	0
PSMC1P9	0.241	0	0	0	0	0	0	0	0	0	0	0	0	0	0	0
FNIP1	0	0	0	0	0	0	0	0	0	0	0	0	0	0	0	0.24
SLC16A8	0	0	0	0	0	0	0	0	0	0	0	0	0	0	0	0.24
CBX4	0	0	0	0	0	0	0	0	0	0	0.128	0	0	0	0.112	0
CELF1	0	0	0	0	0	0	0	0	0	0	0.136	0	0	0	0.104	0
ASS1P1	0	0	0	0	0	0	0	0	0	0	0	0	0	0	0.24	0
ASS1P10	0	0	0	0	0	0	0	0	0	0	0	0	0	0	0.24	0
FADS2	0	0	0	0	0	0	0	0	0	0	0	0	0	0	0.24	0
HIST2H3B	0	0	0	0	0	0	0	0	0	0	0	0	0	0	0.24	0
SPRR2G	0	0	0	0	0	0	0	0	0	0	0	0	0	0	0.24	0
TUFMP1	0	0	0	0	0	0	0	0	0	0	0	0.24	0	0	0	0
RBBP4	0	0	0	0	0	0	0	0	0	0	0.24	0	0	0	0	0
MCCC2	0	0.107	0	0	0	0	0.133	0	0	0	0	0	0	0	0	0
LEMD3	0	0	0	0.24	0	0	0	0	0	0	0	0	0	0	0	0
GNMT	0.24	0	0	0	0	0	0	0	0	0	0	0	0	0	0	0
TMED7-TICAM2	0.24	0	0	0	0	0	0	0	0	0	0	0	0	0	0	0
FRA7G	0	0	0	0	0	0	0	0	0	0	0	0	0	0	0	0.239
NUAK2	0	0	0	0	0	0	0	0	0	0	0	0	0	0	0	0.239
SGSM1	0	0	0	0	0	0	0	0	0	0	0	0	0	0.123	0.116	0
DDX49	0	0	0	0	0	0	0	0	0	0	0.135	0	0	0	0.104	0
ARID1A	0	0	0	0	0	0	0	0	0	0	0	0	0	0	0.239	0
ATP5S	0	0	0	0	0	0	0	0	0	0	0	0	0	0	0.239	0
C17ORF76-AS1	0	0	0	0	0	0	0	0	0	0	0	0	0	0	0.239	0
IGKV1OR2-118	0	0	0	0	0	0	0	0	0	0	0	0	0	0	0.239	0
NDUFV3	0	0	0	0	0	0	0.111	0	0	0	0	0.128	0	0	0	0
BCL2L1	0	0.114	0	0	0	0	0	0	0.125	0	0	0	0	0	0	0
COQ7	0	0	0	0	0	0	0	0	0	0	0	0	0	0.109	0	0.129
TRIM7	0.113	0	0	0	0	0	0	0	0	0	0	0	0	0	0	0.125
AGAP3	0	0	0	0	0	0	0	0	0	0	0.133	0	0	0	0.105	0
NRBP2	0	0	0	0	0	0	0	0	0	0	0	0	0	0	0.238	0
SAT2	0	0	0	0	0	0	0	0	0	0	0	0	0	0	0.238	0

TIGD7	0	0	0	0	0	0	0	0	0	0	0	0	0	0	0.238	0
PPID	0.128	0	0	0	0	0	0	0	0	0	0	0	0	0.11	0	0
CORD8	0	0	0	0	0	0	0	0	0	0.116	0	0.122	0	0	0	0
GRXCR1	0	0	0	0	0	0	0	0	0	0.118	0	0.12	0	0	0	0
CNTN3	0	0	0	0	0.134	0	0	0.104	0	0	0	0	0	0	0	0
GLC1M	0	0	0	0.238	0	0	0	0	0	0	0	0	0	0	0	0
MARCKSL1	0	0	0	0.238	0	0	0	0	0	0	0	0	0	0	0	0
FSCN2	0	0	0	0	0	0	0	0	0	0.101	0	0.136	0	0	0	0
PIGQ	0	0	0	0	0	0	0	0	0	0	0	0	0	0	0	0.237
RIMBP3C	0	0	0	0	0	0	0	0	0	0	0	0	0	0	0	0.237
RSG1	0	0	0	0	0	0	0	0	0	0	0	0	0	0	0	0.237
SAMD8	0	0	0	0	0	0	0	0	0	0	0	0	0	0	0	0.237
SLC2A2	0	0	0	0	0	0	0	0	0	0	0	0	0	0	0	0.237
STRM	0	0	0	0	0	0	0	0	0	0	0	0	0	0	0	0.237
ATOD5	0	0	0	0	0	0	0	0	0	0	0	0	0	0	0.237	0
MTDH	0	0	0	0	0	0	0	0	0	0	0	0	0	0	0.237	0
NBPF14	0	0	0	0	0	0	0	0	0	0	0	0	0	0	0.237	0
WDR92	0	0	0	0	0	0	0	0	0	0	0	0	0	0	0.237	0
CMC1	0	0.107	0	0	0	0	0	0	0	0	0	0	0	0.13	0	0
MT2A	0	0.113	0	0	0	0	0	0	0	0	0	0	0	0.124	0	0
NPS	0	0	0.119	0	0	0	0	0	0	0	0	0	0.118	0	0	0
PROP1	0.106	0	0	0	0	0	0	0	0	0	0	0	0.131	0	0	0
TLX3	0	0.115	0	0	0	0	0	0	0	0	0	0.122	0	0	0	0
NXNL2	0	0	0	0	0	0	0	0	0	0.133	0.104	0	0	0	0	0
PTRF	0	0	0	0.237	0	0	0	0	0	0	0	0	0	0	0	0
USH1H	0	0	0	0	0	0	0	0	0	0.101	0	0.135	0	0	0	0
ANXA1	0	0	0	0	0	0	0	0	0	0	0	0	0	0	0.236	0
C12ORF51	0	0	0	0	0	0	0	0	0	0	0	0	0	0	0.236	0
CADPS	0	0	0	0	0	0	0	0	0	0	0	0	0	0	0.236	0
COX6B1P4	0	0	0	0	0	0	0	0	0	0	0	0	0	0	0.236	0
GGTLC3	0	0	0	0	0	0	0	0	0	0	0	0	0	0	0.236	0
RNU1-11P	0	0	0	0	0	0	0	0	0	0	0	0	0	0	0.236	0
BLOC1S3	0	0	0.125	0	0	0	0	0	0	0	0	0	0.111	0	0	0
PAND3	0	0	0.118	0	0	0	0	0	0	0	0	0	0.118	0	0	0
SHOX	0	0	0	0	0	0	0	0	0	0	0	0	0.236	0	0	0
GDPD5	0	0	0	0.236	0	0	0	0	0	0	0	0	0	0	0	0
UGP1	0.236	0	0	0	0	0	0	0	0	0	0	0	0	0	0	0
HTR7	0	0	0.108	0	0	0	0	0	0	0	0	0	0	0	0	0.127
MFSD10	0	0	0	0	0	0	0	0	0	0	0.118	0	0	0	0.117	0
COX6B1P3	0	0	0	0	0	0	0	0	0	0	0	0	0	0	0.235	0
CRCS11	0	0	0	0	0	0	0	0	0	0	0	0	0	0	0.235	0

MBOAT7	0	0	0	0	0	0	0	0	0	0	0	0	0	0	0.235	0
SNORD36C	0	0	0	0	0	0	0	0	0	0	0	0	0	0	0.235	0
KIAA1432	0	0	0	0.235	0	0	0	0	0	0	0	0	0	0	0	0
ZNF28	0.235	0	0	0	0	0	0	0	0	0	0	0	0	0	0	0
GML	0	0	0	0	0	0	0	0	0	0	0	0	0	0	0	0.234
MYOZ3	0	0	0	0	0	0	0	0	0	0	0	0	0	0	0	0.234
SNTB1	0	0	0	0	0	0	0	0	0	0	0	0	0	0	0	0.234
LOXL2	0	0	0	0	0	0	0	0	0	0	0	0	0	0	0.234	0
SLAIN1	0	0	0	0	0	0	0	0	0	0	0	0	0	0	0.234	0
PRDM10	0	0	0	0	0	0	0	0.234	0	0	0	0	0	0	0	0
NETO2	0	0	0	0	0	0.234	0	0	0	0	0	0	0	0	0	0
IGLC@	0	0	0	0.234	0	0	0	0	0	0	0	0	0	0	0	0
UBBP1	0	0	0	0.234	0	0	0	0	0	0	0	0	0	0	0	0
MOK	0.234	0	0	0	0	0	0	0	0	0	0	0	0	0	0	0
HK3	0.119	0	0	0	0	0	0	0	0	0	0	0	0	0	0	0.115
PLEKHF1	0	0	0	0	0	0	0	0	0	0	0	0	0	0.123	0.111	0
PRD	0	0	0	0	0	0	0	0	0	0.109	0	0	0.125	0	0	0
NAALADL2	0	0	0.108	0	0	0	0	0	0	0	0	0	0.126	0	0	0
HERC2P9	0	0	0	0	0	0.128	0.106	0	0	0	0	0	0	0	0	0
DNAAF2	0	0	0	0	0	0	0	0	0	0	0	0	0	0	0	0.233
SCEL	0	0	0	0	0	0	0	0	0	0	0	0	0	0	0.233	0
SYT2	0	0	0	0	0	0	0	0	0	0	0	0	0	0	0.233	0
TRBV14	0	0	0	0	0	0	0	0	0	0	0	0	0	0	0.233	0
NCAN	0	0	0	0	0	0	0	0	0	0.233	0	0	0	0	0	0
HSBP1	0	0	0	0.233	0	0	0	0	0	0	0	0	0	0	0	0
LPIN1	0	0	0	0.233	0	0	0	0	0	0	0	0	0	0	0	0
MRPS26	0	0	0	0.233	0	0	0	0	0	0	0	0	0	0	0	0
SOBP	0	0	0	0	0	0	0	0	0	0.106	0	0.127	0	0	0	0
TARM1	0	0	0	0	0	0	0	0	0	0.13	0	0.103	0	0	0	0
CDH8	0	0	0	0	0	0.118	0	0	0	0.115	0	0	0	0	0	0
ZNF214	0.105	0	0	0	0	0.128	0	0	0	0	0	0	0	0	0	0
MOG	0	0.115	0	0.118	0	0	0	0	0	0	0	0	0	0	0	0
AKT1S1	0.126	0.107	0	0	0	0	0	0	0	0	0	0	0	0	0	0
DPEP2	0	0	0	0	0	0	0	0	0	0	0	0	0	0	0	0.232
LOC339535	0	0	0	0	0	0	0	0	0	0	0	0	0	0	0.232	0
PAX6	0	0	0	0	0	0	0	0	0	0	0	0	0	0	0.232	0
SCAMP2	0	0	0	0	0	0	0	0	0	0	0	0	0	0	0.232	0
TAS2R7	0	0	0	0	0	0	0	0	0	0	0	0	0	0	0.232	0
TRGJ1	0	0	0	0	0	0	0	0	0	0	0	0	0	0	0.232	0
WNT1	0	0	0	0	0	0	0	0	0	0	0	0	0	0	0.232	0
OR10A2	0	0	0	0	0	0	0	0	0	0	0	0.232	0	0	0	0

NTHL1	0	0	0	0	0	0	0	0	0	0	0.232	0	0	0	0	0
RPGRIP1	0	0	0	0	0	0	0	0	0	0.232	0	0	0	0	0	0
SLEH1	0	0	0	0	0	0.115	0.117	0	0	0	0	0	0	0	0	0
HP	0	0	0	0	0.232	0	0	0	0	0	0	0	0	0	0	0
CADM2	0	0	0	0.232	0	0	0	0	0	0	0	0	0	0	0	0
HOXB3	0	0	0	0.232	0	0	0	0	0	0	0	0	0	0	0	0
MAFB	0	0	0	0.232	0	0	0	0	0	0	0	0	0	0	0	0
MTMR7	0	0	0	0.232	0	0	0	0	0	0	0	0	0	0	0	0
PXMP2	0	0	0	0.232	0	0	0	0	0	0	0	0	0	0	0	0
KIAA0319	0	0	0.119	0	0	0	0	0	0	0	0	0	0.113	0	0	0
GSTT1	0	0	0	0	0	0	0.101	0	0	0	0	0	0	0	0	0.13
ATFB3	0	0	0	0	0	0	0	0	0	0	0	0	0	0	0	0.231
DPM2	0	0	0	0	0	0	0	0	0	0	0	0	0	0	0	0.231
EDN1	0	0	0	0	0	0	0	0	0	0	0	0	0	0	0	0.231
GPAA1P1	0	0	0	0	0	0	0	0	0	0	0	0	0	0	0	0.231
GPC6	0	0	0	0	0	0	0	0	0	0	0	0	0	0	0	0.231
ISM1	0	0	0	0	0	0	0	0	0	0	0	0	0	0	0.231	0
ABT1	0	0	0	0	0	0	0	0	0	0	0.231	0	0	0	0	0
AGT	0	0	0	0	0.231	0	0	0	0	0	0	0	0	0	0	0
HPX	0.231	0	0	0	0	0	0	0	0	0	0	0	0	0	0	0
OAF	0.231	0	0	0	0	0	0	0	0	0	0	0	0	0	0	0
PEX1	0	0	0	0	0	0	0.105	0	0	0	0	0	0.126	0	0	0
DDX6	0	0	0	0	0.11	0	0	0	0.121	0	0	0	0	0	0	0
GOLM1	0	0	0	0	0.122	0	0	0	0.109	0	0	0	0	0	0	0
SLC16A6	0	0	0	0	0	0	0	0	0	0	0	0	0	0	0	0.23
FAM65C	0	0	0	0	0	0	0	0	0	0	0	0	0	0	0.23	0
FGF8	0	0	0	0	0	0	0	0	0	0	0	0	0	0	0.23	0
HMCN2	0	0	0	0	0	0	0	0	0	0	0	0	0	0	0.23	0
LTBP1	0	0	0	0	0	0	0	0	0	0	0	0	0	0	0.23	0
PLD4	0	0	0	0	0	0	0	0	0	0	0	0	0	0	0.23	0
MCOLN3	0	0	0	0	0	0	0	0	0	0.116	0	0.114	0	0	0	0
DAB1	0	0	0	0	0	0.101	0	0	0	0.129	0	0	0	0	0	0
TAGAP	0	0.101	0	0	0	0	0	0	0.129	0	0	0	0	0	0	0
PGPEP1	0	0	0	0	0	0	0	0.23	0	0	0	0	0	0	0	0
COPD4	0	0	0	0.23	0	0	0	0	0	0	0	0	0	0	0	0
NDUFS3	0	0	0	0	0	0	0.107	0	0	0	0	0	0	0.123	0	0
HSPA12B	0.106	0	0	0	0	0	0	0	0	0	0	0	0	0.124	0	0
FSTL4	0	0	0	0	0	0	0	0	0	0.121	0	0.109	0	0	0	0
AFF4	0	0	0	0	0.128	0	0	0	0.102	0	0	0	0	0	0	0
MAPK12	0.121	0	0	0	0	0	0	0	0.109	0	0	0	0	0	0	0
RPL3L	0	0.104	0	0	0	0.126	0	0	0	0	0	0	0	0	0	0

NUFIP2	0.118	0	0.112	0	0	0	0	0	0	0	0	0	0	0	0	0
HVCN1	0	0	0	0	0	0.116	0	0	0	0	0	0	0	0	0	0.113
FCHSD2	0	0	0	0	0	0	0	0	0	0	0	0	0	0	0	0.229
PIGX	0	0	0	0	0	0	0	0	0	0	0	0	0	0	0	0.229
ARG1	0	0	0	0	0	0	0	0	0	0	0	0	0	0	0.229	0
ATP5C1P1	0	0	0	0	0	0	0	0	0	0	0	0	0	0	0.229	0
HIST1H2BG	0	0	0	0	0	0	0	0	0	0	0	0	0	0	0.229	0
SAT1	0	0	0	0	0	0	0	0	0	0	0	0	0	0	0.229	0
FAM161A	0	0	0	0	0	0	0	0	0	0.115	0	0.114	0	0	0	0
ADAT3	0	0	0	0	0	0	0	0	0	0	0.229	0	0	0	0	0
SP1	0	0	0	0	0	0	0	0	0	0	0.229	0	0	0	0	0
FGFR1	0	0.129	0	0	0	0	0	0	0	0.1	0	0	0	0	0	0
GJD4	0	0	0	0.229	0	0	0	0	0	0	0	0	0	0	0	0
HEBP1	0.229	0	0	0	0	0	0	0	0	0	0	0	0	0	0	0
KIF2B	0.229	0	0	0	0	0	0	0	0	0	0	0	0	0	0	0
AGBL3	0	0	0	0	0	0	0	0	0	0.111	0	0	0	0.118	0	0
COX5A	0	0	0	0	0	0	0.106	0	0	0	0	0	0	0.123	0	0
GLRX2	0	0.106	0	0	0	0	0	0	0	0	0	0	0	0.123	0	0
DFNB14	0	0	0	0	0	0	0	0	0	0	0	0.104	0.125	0	0	0
ITPR2	0	0.105	0	0	0	0	0	0	0	0	0.124	0	0	0	0	0
UBE3AP1	0	0	0	0	0	0.122	0.107	0	0	0	0	0	0	0	0	0
SPEG	0.124	0	0	0	0.105	0	0	0	0	0	0	0	0	0	0	0
GSTP1	0	0	0	0	0	0	0.116	0	0	0	0	0	0	0	0	0.112
ADC	0	0	0	0	0	0	0	0	0	0	0	0	0	0	0.228	0
IGKV1OR-2	0	0	0	0	0	0	0	0	0	0	0	0	0	0	0.228	0
ALDOAP1	0	0	0	0	0	0	0	0	0	0	0.228	0	0	0	0	0
BRF2	0	0	0	0	0	0	0	0	0	0	0.228	0	0	0	0	0
KCNIP4	0	0	0	0	0	0.117	0	0.111	0	0	0	0	0	0	0	0
ZNF398	0.228	0	0	0	0	0	0	0	0	0	0	0	0	0	0	0
STRADB	0	0.107	0	0	0	0	0	0	0	0	0	0	0	0	0	0.121
ACRV1	0	0.118	0	0	0	0	0	0	0.11	0	0	0	0	0	0	0
CASK	0	0	0	0	0	0	0	0	0	0	0	0	0	0	0	0.227
CASQ1	0	0	0	0	0	0	0	0	0	0	0	0	0	0	0	0.227
RBP4	0	0	0	0	0	0	0	0	0	0	0	0	0	0	0	0.227
RYR2	0	0	0	0	0	0	0	0	0	0	0	0	0	0	0	0.227
TMED8	0	0	0	0	0	0	0	0	0	0	0	0	0	0	0	0.227
TDGF1	0	0	0	0	0	0	0	0	0	0	0	0	0	0	0.227	0
CCDC50	0	0	0	0	0	0	0	0	0	0	0	0.227	0	0	0	0
KHDRBS1	0	0	0	0	0	0	0	0	0.227	0	0	0	0	0	0	0
MAGI2	0	0	0	0.227	0	0	0	0	0	0	0	0	0	0	0	0
NOS1AP	0	0	0	0	0	0	0	0	0	0	0	0	0	0	0	0.226

ATP5A1P5	0	0	0	0	0	0	0	0	0	0	0	0	0	0	0.226	0
DUX4	0	0	0	0	0	0	0	0	0	0	0	0	0	0	0.226	0
H2AFV	0	0	0	0	0	0	0	0	0	0	0	0	0	0	0.226	0
MYCBP2	0	0.114	0	0	0	0	0	0	0	0	0	0.112	0	0	0	0
GPX1P2	0.226	0	0	0	0	0	0	0	0	0	0	0	0	0	0	0
SLC25A2	0	0	0	0	0	0	0.12	0	0	0	0	0	0	0	0.106	0
DGCR2	0	0	0.119	0	0	0	0	0	0	0	0	0	0.107	0	0	0
CYP4V2	0	0	0	0	0	0	0	0	0	0.105	0	0.121	0	0	0	0
SPATA7	0	0	0	0	0	0	0	0	0	0.123	0	0.103	0	0	0	0
TRA2B	0	0.106	0	0	0	0	0	0	0.12	0	0	0	0	0	0	0
KCNB2	0	0	0	0	0	0.121	0	0.105	0	0	0	0	0	0	0	0
LRRC20	0	0	0	0	0	0	0	0	0	0	0	0	0	0	0.225	0
RP22	0	0	0	0	0	0	0	0	0	0.115	0	0.11	0	0	0	0
PKM2	0	0	0	0	0	0	0	0	0	0	0.225	0	0	0	0	0
SMG8	0	0	0	0	0	0	0	0	0	0	0.225	0	0	0	0	0
SNAPC1	0	0	0	0	0	0	0	0	0	0	0.225	0	0	0	0	0
CDC37	0	0	0	0	0	0	0	0	0.225	0	0	0	0	0	0	0
MSBP1	0	0	0	0	0	0	0	0	0	0	0.119	0	0	0	0.106	0
ATP1B4	0	0	0	0	0	0	0	0	0	0	0	0	0	0	0	0.224
KCNE1	0	0	0	0	0	0	0	0	0	0	0	0	0	0	0	0.224
SEN6B	0	0	0	0	0	0	0	0	0	0	0	0	0	0	0	0.224
POU4F1	0	0.101	0	0	0	0	0	0	0	0	0	0.123	0	0	0	0
ARTN	0	0	0	0	0	0	0	0	0	0	0	0.224	0	0	0	0
GSX2	0	0	0	0	0	0	0	0	0	0	0.224	0	0	0	0	0
LIN7B	0	0	0	0	0	0	0	0	0	0	0.224	0	0	0	0	0
IFNA16	0	0	0	0	0.224	0	0	0	0	0	0	0	0	0	0	0
TSPAN2	0	0	0	0.224	0	0	0	0	0	0	0	0	0	0	0	0
PARP15	0.224	0	0	0	0	0	0	0	0	0	0	0	0	0	0	0
POU4F3	0	0	0	0	0	0	0	0	0	0.12	0	0.104	0	0	0	0
CATSPER1	0	0	0	0	0	0	0	0	0	0	0	0	0	0	0	0.223
CATSPER2	0	0	0	0	0	0	0	0	0	0	0	0	0	0	0	0.223
JSRP1	0	0	0	0	0	0	0	0	0	0	0	0	0	0	0	0.223
RYR3	0	0	0	0	0	0	0	0	0	0	0	0	0	0	0	0.223
HIST1H2BM	0	0	0	0	0	0	0	0	0	0	0	0	0	0	0.223	0
NR2C2AP	0	0	0	0	0	0	0	0	0	0	0	0	0	0	0.223	0
VAMP1	0	0	0	0	0	0	0	0	0	0	0	0	0	0	0.223	0
GLRX	0	0.114	0	0	0	0	0	0	0	0	0	0	0	0.109	0	0
NBEAL2	0	0	0	0	0	0	0	0	0	0	0	0	0.223	0	0	0
OTOS	0	0	0	0	0	0	0	0	0	0.108	0	0.115	0	0	0	0
CHMP3	0	0	0	0	0.113	0	0	0	0.11	0	0	0	0	0	0	0
GBA3	0	0	0	0	0.223	0	0	0	0	0	0	0	0	0	0	0

PANX3	0	0	0	0.223	0	0	0	0	0	0	0	0	0	0	0	0
LRRCC1	0.223	0	0	0	0	0	0	0	0	0	0	0	0	0	0	0
TSPAN11	0.223	0	0	0	0	0	0	0	0	0	0	0	0	0	0	0
FABP3	0	0	0	0	0	0	0	0	0	0	0	0	0	0	0	0.222
PIGO	0	0	0	0	0	0	0	0	0	0	0	0	0	0	0	0.222
SKOR1	0	0	0	0	0	0	0	0	0	0	0	0	0	0	0	0.222
TNNI1	0	0	0	0	0	0	0	0	0	0	0	0	0	0	0	0.222
CAPNS2	0	0	0	0	0	0	0	0	0	0	0.111	0	0	0	0.111	0
SNAP47	0	0	0	0	0	0	0	0	0	0	0	0	0	0	0.222	0
TTC7A	0	0	0	0	0	0	0	0	0	0	0	0	0	0	0.222	0
KAZA	0	0	0	0.222	0	0	0	0	0	0	0	0	0	0	0	0
NRCAM	0	0	0	0.222	0	0	0	0	0	0	0	0	0	0	0	0
GNB1L	0	0	0.119	0	0	0	0	0	0	0	0	0	0.103	0	0	0
ARVD3	0	0	0	0	0	0	0	0	0	0	0	0	0	0	0	0.221
GCHFR	0	0	0	0	0	0	0	0	0	0	0	0	0	0	0	0.221
NOS2P2	0	0	0	0	0	0	0	0	0	0	0	0	0	0	0	0.221
PPARG	0	0	0	0	0	0	0	0	0	0	0	0	0	0	0	0.221
OR2F1	0	0	0	0	0	0	0	0	0	0	0	0	0	0	0.221	0
TAF10	0	0	0	0	0	0	0	0	0	0	0.221	0	0	0	0	0
OLIG2	0	0.101	0	0	0	0	0	0	0	0.12	0	0	0	0	0	0
GUCY2D	0	0	0	0	0	0	0	0	0	0.221	0	0	0	0	0	0
GPR15	0	0	0	0	0.105	0	0	0	0.116	0	0	0	0	0	0	0
KIF20B	0	0.114	0	0.107	0	0	0	0	0	0	0	0	0	0	0	0
DSCR3	0.221	0	0	0	0	0	0	0	0	0	0	0	0	0	0	0
XKR4	0	0	0.119	0	0	0	0	0	0	0	0	0	0.102	0	0	0
DFNB40	0	0	0	0	0	0	0	0	0	0.118	0	0.103	0	0	0	0
PIGN	0	0	0	0	0	0	0	0	0	0	0	0	0	0	0	0.22
PRKAB1	0	0	0	0	0	0	0	0	0	0	0	0	0	0	0	0.22
CDK5RAP3	0	0	0	0	0	0	0	0	0	0	0.22	0	0	0	0	0
DR1	0	0	0	0	0	0	0	0	0	0	0.22	0	0	0	0	0
FAM13A	0	0	0	0	0	0.115	0	0.105	0	0	0	0	0	0	0	0
URB2	0	0	0	0.22	0	0	0	0	0	0	0	0	0	0	0	0
ITGB1BP2	0.22	0	0	0	0	0	0	0	0	0	0	0	0	0	0	0
PLAUR	0	0	0	0	0	0	0	0	0	0	0	0	0	0	0	0.219
ACTB	0	0	0	0	0	0	0	0	0	0	0	0	0	0	0.219	0
AKAP16BP	0	0	0	0	0	0	0	0	0	0	0	0	0	0	0.219	0
API5P1	0	0	0	0	0	0	0	0	0	0	0	0	0	0	0.219	0
FKBP1A	0	0	0	0	0	0	0	0	0	0	0	0	0	0	0.219	0
HIST1H4B	0	0	0	0	0	0	0	0	0	0	0	0	0	0	0.219	0
PHYH	0	0	0	0	0	0	0.104	0	0	0	0	0.115	0	0	0	0
SRSF10	0	0	0	0	0	0	0	0	0.219	0	0	0	0	0	0	0

SIRPA	0.219	0	0	0	0	0	0	0	0	0	0	0	0	0	0	0
SNAI1	0.219	0	0	0	0	0	0	0	0	0	0	0	0	0	0	0
RNF32	0	0	0	0	0	0	0	0	0	0	0	0	0	0	0	0.218
SLC5A9	0	0	0	0	0	0	0	0	0	0	0	0	0	0	0	0.218
SNTA1	0	0	0	0	0	0	0	0	0	0	0	0	0	0	0	0.218
TNNT2	0	0	0	0	0	0	0	0	0	0	0	0	0	0	0	0.218
A2LD1	0	0	0	0	0	0	0	0	0	0	0	0	0	0	0.218	0
C11ORF75	0	0	0	0	0	0	0	0	0	0	0	0	0	0	0.218	0
ECB2	0.106	0	0	0	0	0	0	0	0	0	0	0.112	0	0	0	0
ALS2CR12	0	0.115	0	0	0	0	0	0	0.103	0	0	0	0	0	0	0
SLC25A43	0	0.109	0	0	0	0	0	0	0.109	0	0	0	0	0	0	0
SHISA4	0	0	0	0.218	0	0	0	0	0	0	0	0	0	0	0	0
SLC36A2	0	0	0	0.218	0	0	0	0	0	0	0	0	0	0	0	0
TOR1AIP1	0	0	0	0.218	0	0	0	0	0	0	0	0	0	0	0	0
TRIM32	0	0	0	0.218	0	0	0	0	0	0	0	0	0	0	0	0
CYP4F22	0	0	0	0	0	0	0	0	0	0	0	0	0.116	0	0.101	0
LOC100132626	0	0	0	0	0	0	0	0	0	0	0.116	0	0	0	0.101	0
LOC728369	0	0	0	0	0	0	0	0	0	0	0.116	0	0	0	0.101	0
LOC729317	0.105	0	0	0	0	0	0	0	0	0	0	0	0	0	0	0.112
HTR5BP	0	0	0	0	0	0	0	0	0	0	0	0	0	0	0	0.217
OC90	0	0	0	0	0	0	0	0	0	0	0	0.103	0	0	0.114	0
FAN1	0	0	0	0	0	0	0	0	0	0	0	0	0	0	0.217	0
CDK5RAP1	0	0	0	0	0	0	0	0	0	0	0.113	0	0	0.104	0	0
UCHL3	0	0	0	0	0	0	0	0	0	0.102	0	0	0	0.115	0	0
DNAJC19	0	0	0	0	0	0	0.111	0	0	0	0	0.106	0	0	0	0
NT5C3P1	0	0	0	0	0	0	0.111	0	0	0	0	0.106	0	0	0	0
ITSN1	0	0	0	0	0	0	0	0	0	0	0.217	0	0	0	0	0
CAMK2A	0	0	0.11	0	0	0	0	0	0	0.107	0	0	0	0	0	0
PAPA3	0.111	0	0	0.106	0	0	0	0	0	0	0	0	0	0	0	0
CELF5	0	0	0	0	0	0	0	0	0	0	0	0	0	0	0	0.216
IFT46	0	0	0	0	0	0	0	0	0	0	0	0	0	0	0	0.216
LOC285232	0	0	0	0	0	0	0	0	0	0	0	0	0	0	0	0.216
RPS19BP1	0	0	0	0	0	0	0	0	0	0	0	0	0	0	0	0.216
RSPH6A	0	0	0	0	0	0	0	0	0	0	0	0	0	0	0	0.216
SYT12	0	0	0	0	0	0	0	0	0	0	0	0	0	0	0.216	0
LCA5	0	0	0	0	0	0	0	0	0	0.108	0	0.108	0	0	0	0
SLC1A7	0	0	0	0	0	0.216	0	0	0	0	0	0	0	0	0	0
NOTCH2	0	0	0	0	0.216	0	0	0	0	0	0	0	0	0	0	0
LOC441666	0	0	0	0.216	0	0	0	0	0	0	0	0	0	0	0	0
JUB	0.216	0	0	0	0	0	0	0	0	0	0	0	0	0	0	0
LOC728379	0	0	0	0	0	0	0	0	0	0	0.114	0	0	0	0.101	0

HCN4	0	0	0	0	0	0.112	0	0	0	0	0	0	0	0	0	0.103
CYP3A4	0	0	0	0	0	0	0	0	0	0	0	0	0	0	0	0.215
CYYR1	0	0	0	0	0	0	0	0	0	0	0.111	0	0	0	0.104	0
LALBA	0	0	0	0	0	0	0	0	0	0	0	0	0	0	0.215	0
VSIG2	0	0	0	0	0	0	0	0	0	0	0	0	0	0	0.215	0
NRADDP	0.104	0	0	0	0	0	0	0	0	0	0	0	0	0.111	0	0
TSEN54	0	0	0	0	0	0	0.106	0	0	0	0	0	0.109	0	0	0
DHX9P1	0	0	0	0	0.105	0	0	0.11	0	0	0	0	0	0	0	0
PCNX	0	0	0	0	0.215	0	0	0	0	0	0	0	0	0	0	0
PRSS22	0	0	0	0	0.215	0	0	0	0	0	0	0	0	0	0	0
PLEKHM2	0	0	0	0.215	0	0	0	0	0	0	0	0	0	0	0	0
CA5B	0	0	0	0	0	0	0	0	0	0	0	0	0	0	0	0.214
PIGW	0	0	0	0	0	0	0	0	0	0	0	0	0	0	0	0.214
ERVV-2	0	0	0	0	0	0	0	0	0	0	0	0	0	0	0.214	0
SGTB	0	0	0	0	0	0	0	0	0	0	0	0	0	0	0.214	0
SYT6	0	0	0	0	0	0	0	0	0	0	0	0	0	0	0.214	0
TCHH	0	0	0	0	0	0	0	0	0	0	0	0	0	0	0.214	0
SRD5A3	0	0	0	0	0	0	0	0	0	0	0	0	0.214	0	0	0
KCND2	0	0	0	0	0	0.109	0	0.105	0	0	0	0	0	0	0	0
IGHD3-3	0	0	0	0.214	0	0	0	0	0	0	0	0	0	0	0	0
LRFN2	0	0	0	0.214	0	0	0	0	0	0	0	0	0	0	0	0
MAGI1	0	0	0	0.214	0	0	0	0	0	0	0	0	0	0	0	0
PRDM15	0.214	0	0	0	0	0	0	0	0	0	0	0	0	0	0	0
ULK3	0.214	0	0	0	0	0	0	0	0	0	0	0	0	0	0	0
KCNH2	0	0	0	0	0	0	0	0	0	0	0	0	0	0	0	0.213
INLNE	0	0	0	0	0	0	0	0	0	0	0	0.109	0	0	0.104	0
ACTBP9	0	0	0	0	0	0	0	0	0	0	0	0	0	0	0.213	0
OAZ2	0	0	0	0	0	0	0	0	0	0	0	0	0	0	0.213	0
MIR1471	0	0	0	0	0	0	0	0	0	0	0.213	0	0	0	0	0
MOGS	0	0	0	0	0.213	0	0	0	0	0	0	0	0	0	0	0
NAT2	0	0	0	0	0.213	0	0	0	0	0	0	0	0	0	0	0
MAP4	0.213	0	0	0	0	0	0	0	0	0	0	0	0	0	0	0
CARD16	0	0	0	0	0	0	0	0	0	0	0.111	0	0	0	0.101	0
GPR179	0	0	0	0	0	0	0	0	0	0	0.111	0	0	0	0.101	0
LFS3	0	0	0	0	0	0	0	0	0	0	0	0	0	0	0	0.212
CTSL1P1	0	0	0	0	0	0	0	0	0	0	0	0	0	0	0.212	0
EVPL	0	0	0	0	0	0	0	0	0	0	0	0	0	0	0.212	0
HDLC2	0.109	0	0	0	0	0	0	0	0.103	0	0	0	0	0	0	0
SLC5A1	0	0	0	0	0	0	0	0	0	0	0	0	0	0	0	0.211
TRIP10	0	0	0	0	0	0	0	0	0	0	0.105	0	0	0	0.106	0
EVX1	0	0	0	0	0	0	0	0	0	0	0	0	0	0	0.211	0

F13A1	0	0	0	0	0	0	0	0	0	0	0	0	0	0	0.211	0
IGKC	0	0	0	0	0	0	0	0	0	0	0	0	0	0	0.211	0
IQSEC1	0	0	0	0	0	0.211	0	0	0	0	0	0	0	0	0	0
KCNH1	0	0	0	0	0	0.211	0	0	0	0	0	0	0	0	0	0
PRIMA1	0	0	0	0	0.211	0	0	0	0	0	0	0	0	0	0	0
SNTG2	0	0	0	0	0	0	0	0	0	0	0	0	0	0	0	0.21
NOP14	0	0	0	0	0	0	0	0	0	0	0.104	0	0	0	0.106	0
NAT9	0	0	0	0	0	0	0	0	0	0	0	0	0	0	0.21	0
OR1J1	0	0	0	0	0	0	0	0	0	0	0	0	0	0	0.21	0
MAN2B1	0	0	0	0	0	0	0	0	0	0	0	0	0.104	0.106	0	0
LRP2BP	0.102	0	0	0	0	0	0	0	0	0	0	0.108	0	0	0	0
DICER1	0	0	0	0	0	0	0	0	0	0	0.21	0	0	0	0	0
TMEM237	0	0.108	0	0	0	0	0	0	0	0.102	0	0	0	0	0	0
ATP5BP1	0.21	0	0	0	0	0	0	0	0	0	0	0	0	0	0	0
CD320	0.21	0	0	0	0	0	0	0	0	0	0	0	0	0	0	0
IBA57	0.21	0	0	0	0	0	0	0	0	0	0	0	0	0	0	0
GSC	0	0	0	0	0	0	0	0	0	0	0	0	0	0	0.209	0
MYCN	0	0	0	0	0	0	0	0	0	0	0	0	0	0	0.209	0
GTF2A2	0	0	0	0	0	0	0	0	0	0	0.209	0	0	0	0	0
RAPSN	0	0	0	0.209	0	0	0	0	0	0	0	0	0	0	0	0
LOC100287366	0	0	0	0	0	0	0	0	0	0	0.107	0	0	0	0.101	0
C17ORF85	0	0	0	0	0	0	0	0	0	0	0	0	0	0	0.208	0
DMXL2	0	0	0	0	0	0	0	0	0	0	0	0	0	0	0.208	0
POTEI	0	0	0	0	0	0	0	0	0	0	0	0	0	0	0.208	0
TRAV8-6	0	0	0	0	0	0	0	0	0	0	0	0	0	0	0.208	0
IKBKG	0	0	0	0	0	0	0	0	0	0	0.208	0	0	0	0	0
DHH	0	0	0	0.208	0	0	0	0	0	0	0	0	0	0	0	0
MNG1	0	0	0	0.208	0	0	0	0	0	0	0	0	0	0	0	0
NME4	0	0	0	0	0	0	0	0	0	0	0	0	0	0	0.207	0
MYP3	0	0	0	0	0	0	0	0	0	0.105	0	0.102	0	0	0	0
TUBA4A	0	0	0	0	0	0	0	0	0	0	0.207	0	0	0	0	0
ACAT1	0	0	0	0	0.207	0	0	0	0	0	0	0	0	0	0	0
SPTLC3	0	0	0	0.207	0	0	0	0	0	0	0	0	0	0	0	0
MIR26A1	0.207	0	0	0	0	0	0	0	0	0	0	0	0	0	0	0
AGA	0	0	0	0	0	0	0	0	0	0	0	0	0.101	0.105	0	0
ALG11	0	0	0	0	0	0	0.105	0	0	0	0	0	0.101	0	0	0
C19ORF59	0	0	0	0	0	0	0	0	0	0	0	0	0	0	0	0.206
CILD5	0	0	0	0	0	0	0	0	0	0	0	0	0	0	0	0.206
CR1L	0	0	0	0	0	0	0	0	0	0	0	0	0	0	0	0.206
AMD1	0	0	0	0	0	0	0	0	0	0	0	0	0	0	0.206	0
ACTBP8	0	0	0	0	0	0	0	0	0	0	0.206	0	0	0	0	0

ZNF76	0	0	0	0	0	0	0	0	0	0	0.206	0	0	0	0	0
ABCC9	0	0	0	0	0	0.206	0	0	0	0	0	0	0	0	0	0
CAB39	0	0	0	0	0	0	0	0	0	0	0	0	0	0	0	0.205
SLC16A4	0	0	0	0	0	0	0	0	0	0	0	0	0	0	0	0.205
CHRD	0	0	0	0	0	0	0	0	0	0	0	0	0	0	0.205	0
MEGF11	0.103	0	0	0	0	0	0	0	0	0	0	0.102	0	0	0	0
BSND	0	0	0	0	0	0	0	0	0	0	0	0.205	0	0	0	0
GANC	0	0	0	0	0.205	0	0	0	0	0	0	0	0	0	0	0
MIR5096	0.205	0	0	0	0	0	0	0	0	0	0	0	0	0	0	0
HMOX2	0	0	0	0	0	0	0	0	0	0	0	0	0	0	0	0.204
PRKAA1	0	0	0	0	0	0	0	0	0	0	0	0	0	0	0	0.204
LOR	0	0	0	0	0	0	0	0	0	0	0	0	0	0	0.204	0
SEL1L3	0	0	0	0	0	0	0	0	0	0	0	0	0	0	0.204	0
BCL11B	0	0	0	0	0	0	0	0	0	0	0.204	0	0	0	0	0
PGM2	0	0	0	0	0	0	0	0	0	0	0.204	0	0	0	0	0
DGKQ	0	0	0	0	0	0	0	0	0.204	0	0	0	0	0	0	0
HSPB7	0	0	0	0.204	0	0	0	0	0	0	0	0	0	0	0	0
FYCO1	0	0	0	0	0	0	0	0	0	0	0	0.103	0	0	0	0.1
CIRH1A	0	0	0	0	0	0	0	0	0	0	0	0.203	0	0	0	0
UNGP2	0	0	0	0	0	0	0	0	0	0	0.203	0	0	0	0	0
NPIP	0	0	0	0	0	0	0	0	0	0.203	0	0	0	0	0	0
NRG3	0	0	0	0	0	0	0	0	0.203	0	0	0	0	0	0	0
MCAT	0	0	0	0	0	0	0	0	0	0	0	0	0	0	0	0.202
MPP3	0	0	0	0	0	0	0	0	0	0	0	0	0	0	0	0.202
ADRA1B	0	0	0	0	0	0	0	0	0	0	0	0	0	0	0.202	0
BTD	0	0	0	0	0	0	0	0	0	0	0	0.101	0.101	0	0	0
ITPR3	0	0	0	0	0	0	0	0	0	0	0.202	0	0	0	0	0
CSTP2	0	0	0	0	0	0	0	0.202	0	0	0	0	0	0	0	0
APOA5	0.202	0	0	0	0	0	0	0	0	0	0	0	0	0	0	0
LIPG	0.202	0	0	0	0	0	0	0	0	0	0	0	0	0	0	0
TCEB2P2	0.202	0	0	0	0	0	0	0	0	0	0	0	0	0	0	0
ADRB2	0	0	0	0	0	0	0	0	0	0	0	0	0	0	0	0.201
PIGL	0	0	0	0	0	0	0	0	0	0	0	0	0	0	0	0.201
PIGV	0	0	0	0	0	0	0	0	0	0	0	0	0	0	0	0.201
UCP2	0	0	0	0	0	0	0	0	0	0	0	0	0	0	0	0.201
IGKV3OR2-268	0	0	0	0	0	0	0	0	0	0	0	0	0	0	0.201	0
TRBV6-3	0	0	0	0	0	0	0	0	0	0	0	0	0	0	0.201	0
POLB	0	0	0	0	0	0	0	0	0	0	0.201	0	0	0	0	0
CPXCR1	0	0	0	0	0	0.201	0	0	0	0	0	0	0	0	0	0
CYTH2	0	0	0	0	0	0.201	0	0	0	0	0	0	0	0	0	0
MTMR6	0	0	0	0.201	0	0	0	0	0	0	0	0	0	0	0	0

LRP6	0.201	0	0	0	0	0	0	0	0	0	0	0	0	0	0	0
MSK10	0.201	0	0	0	0	0	0	0	0	0	0	0	0	0	0	0
FUT10	0	0	0	0	0	0	0	0	0	0	0	0	0	0	0	0.2
SMPDL3A	0	0	0	0	0	0	0	0	0	0	0	0	0	0	0	0.2
UCP3	0	0	0	0	0	0	0	0	0	0	0	0	0	0	0	0.2
HIST1H3I	0	0	0	0	0	0	0	0	0	0	0	0	0	0	0.2	0
KALP	0	0	0	0	0	0	0	0	0	0	0	0	0	0	0.2	0
BRP44L	0	0	0	0	0	0	0	0	0	0	0.2	0	0	0	0	0
MCHR1	0	0	0	0	0	0	0	0	0	0	0.2	0	0	0	0	0
HIST1H2AC	0	0	0	0	0	0	0	0	0	0	0	0	0	0	0.199	0
BAI3	0.199	0	0	0	0	0	0	0	0	0	0	0	0	0	0	0
SF	0.199	0	0	0	0	0	0	0	0	0	0	0	0	0	0	0
NAMPT	0	0	0	0	0	0	0	0	0	0	0	0	0	0	0	0.198
RETN	0	0	0	0	0	0	0	0	0	0	0	0	0	0	0	0.198
BAIAP2L2	0	0	0	0	0	0	0	0	0	0	0	0	0	0	0.198	0
CTSL1P3	0	0	0	0	0	0	0	0	0	0	0	0	0	0	0.198	0
FGF17	0	0	0	0	0	0	0	0	0	0	0	0	0	0	0.198	0
TMC5	0	0	0	0	0	0	0	0	0	0	0	0.198	0	0	0	0
NR3C1	0	0	0	0	0.198	0	0	0	0	0	0	0	0	0	0	0
DMRTC1B	0.198	0	0	0	0	0	0	0	0	0	0	0	0	0	0	0
MIC12	0.198	0	0	0	0	0	0	0	0	0	0	0	0	0	0	0
NGFG	0.198	0	0	0	0	0	0	0	0	0	0	0	0	0	0	0
SPOCK2	0.198	0	0	0	0	0	0	0	0	0	0	0	0	0	0	0
ARL13B	0	0	0	0	0	0	0	0	0	0	0	0	0	0	0	0.197
ARVD4	0	0	0	0	0	0	0	0	0	0	0	0	0	0	0	0.197
PIGY	0	0	0	0	0	0	0	0	0	0	0	0	0	0	0	0.197
FKBP10	0	0	0	0	0	0	0	0	0	0	0	0	0	0	0.197	0
SBSN	0	0	0	0	0	0	0	0	0	0	0	0	0	0	0.197	0
TRBV13	0	0	0	0	0	0	0	0	0	0	0	0	0	0	0.197	0
NOL12	0	0	0	0	0	0	0	0	0	0	0	0.197	0	0	0	0
CEP41	0	0	0	0	0	0.197	0	0	0	0	0	0	0	0	0	0
PLD2	0	0	0	0	0	0.197	0	0	0	0	0	0	0	0	0	0
GYLTL1B	0	0	0	0.197	0	0	0	0	0	0	0	0	0	0	0	0
MPP7	0	0	0	0.197	0	0	0	0	0	0	0	0	0	0	0	0
ARHGAP30	0.197	0	0	0	0	0	0	0	0	0	0	0	0	0	0	0
SUGT1	0.197	0	0	0	0	0	0	0	0	0	0	0	0	0	0	0
CFC1	0	0	0	0	0	0	0	0	0	0	0	0	0	0	0	0.196
GPX1	0	0	0	0	0	0	0	0	0	0	0	0	0	0	0	0.196
SLC2A9	0	0	0	0	0	0	0	0	0	0	0	0	0	0	0	0.196
CWC22	0	0	0	0	0	0	0	0	0	0	0	0	0	0	0.196	0
PAX5	0	0	0	0	0	0	0	0	0	0	0	0	0	0	0.196	0

PFN1P2	0	0	0	0	0	0	0	0	0	0	0	0	0	0	0.196	0
PFN1P3	0	0	0	0	0	0	0	0	0	0	0	0	0	0	0.196	0
PUS10	0	0	0	0	0	0	0	0	0	0	0	0	0	0	0.196	0
ANO10	0	0	0	0	0	0	0	0	0	0	0	0.196	0	0	0	0
NTF4	0	0	0	0	0	0	0	0	0	0	0	0.196	0	0	0	0
PMCH	0	0	0	0	0	0	0	0	0	0	0.196	0	0	0	0	0
JAG1	0	0	0	0	0.196	0	0	0	0	0	0	0	0	0	0	0
C19ORF46	0	0	0	0.196	0	0	0	0	0	0	0	0	0	0	0	0
CLDN19	0	0	0	0.196	0	0	0	0	0	0	0	0	0	0	0	0
CMD1K	0	0	0	0.196	0	0	0	0	0	0	0	0	0	0	0	0
FAM208B	0	0	0	0.196	0	0	0	0	0	0	0	0	0	0	0	0
RBM11	0	0	0	0.196	0	0	0	0	0	0	0	0	0	0	0	0
RCE1	0	0	0	0.196	0	0	0	0	0	0	0	0	0	0	0	0
REEP3	0	0	0	0.196	0	0	0	0	0	0	0	0	0	0	0	0
RGAG4	0	0	0	0.196	0	0	0	0	0	0	0	0	0	0	0	0
SCHIP1	0	0	0	0.196	0	0	0	0	0	0	0	0	0	0	0	0
SEMA4F	0	0	0	0.196	0	0	0	0	0	0	0	0	0	0	0	0
SEPT9	0	0	0	0.196	0	0	0	0	0	0	0	0	0	0	0	0
LRP3	0.196	0	0	0	0	0	0	0	0	0	0	0	0	0	0	0
MIR1273F	0.196	0	0	0	0	0	0	0	0	0	0	0	0	0	0	0
MIRLET7A2	0.196	0	0	0	0	0	0	0	0	0	0	0	0	0	0	0
GALNTL6	0	0	0	0	0	0	0	0	0	0	0	0	0	0	0	0.195
TNNC2	0	0	0	0	0	0	0	0	0	0	0	0	0	0	0	0.195
SUMO1	0	0	0	0	0	0	0	0	0	0	0.195	0	0	0	0	0
SEPT7	0	0	0	0	0.195	0	0	0	0	0	0	0	0	0	0	0
APOC4-APOC2	0.195	0	0	0	0	0	0	0	0	0	0	0	0	0	0	0
GUCY2GP	0	0	0	0	0	0	0	0	0	0	0	0	0	0	0	0.194
PIGB	0	0	0	0	0	0	0	0	0	0	0	0	0	0	0	0.194
TNRC6A	0	0	0	0	0	0	0	0	0	0	0.194	0	0	0	0	0
TRRAP	0	0	0	0	0	0	0	0	0	0	0.194	0	0	0	0	0
CSPG5	0	0	0	0	0	0	0	0	0	0.194	0	0	0	0	0	0
RPGR	0	0	0	0	0	0	0	0	0	0.194	0	0	0	0	0	0
LRRC48	0	0	0	0	0	0.194	0	0	0	0	0	0	0	0	0	0
IGKV2D-40	0	0	0	0	0.194	0	0	0	0	0	0	0	0	0	0	0
PSMA5	0	0	0	0	0.194	0	0	0	0	0	0	0	0	0	0	0
SPON1	0	0	0	0	0.194	0	0	0	0	0	0	0	0	0	0	0
SRP9	0	0	0	0	0.194	0	0	0	0	0	0	0	0	0	0	0
TIAF1	0	0	0	0	0.194	0	0	0	0	0	0	0	0	0	0	0
TMEM108	0	0	0	0	0.194	0	0	0	0	0	0	0	0	0	0	0
CNPY4	0.194	0	0	0	0	0	0	0	0	0	0	0	0	0	0	0
MPV17L	0.194	0	0	0	0	0	0	0	0	0	0	0	0	0	0	0

TFAP2B	0.194	0	0	0	0	0	0	0	0	0	0	0	0	0	0	0
PKD1L2	0	0	0	0	0	0	0	0	0	0	0	0	0	0	0	0.193
LRG1	0	0	0	0	0	0	0	0	0	0	0	0	0	0	0.193	0
MUC22	0	0	0	0	0	0	0	0	0	0	0	0	0	0	0.193	0
TTC31	0	0	0	0	0	0	0	0	0	0	0	0	0	0	0.193	0
LIG1	0	0	0	0	0	0	0	0	0	0	0.193	0	0	0	0	0
PRPH2	0	0	0	0	0	0	0	0	0	0.193	0	0	0	0	0	0
PDIA5	0	0	0	0	0.193	0	0	0	0	0	0	0	0	0	0	0
NIDDM2	0	0	0	0.193	0	0	0	0	0	0	0	0	0	0	0	0
TMOD3	0.193	0	0	0	0	0	0	0	0	0	0	0	0	0	0	0
NOS2P1	0	0	0	0	0	0	0	0	0	0	0	0	0	0	0	0.192
TMEM57	0	0	0	0	0	0	0	0	0	0	0	0	0	0	0	0.192
F8A2	0	0	0	0	0	0	0	0	0	0	0	0	0	0	0.192	0
RNASEH1P3	0	0	0	0	0	0	0	0	0	0	0	0	0	0	0.192	0
LPAR6	0	0	0	0	0	0	0	0	0	0	0	0.192	0	0	0	0
MAP2K6	0	0	0	0	0	0	0	0	0	0	0.192	0	0	0	0	0
TTLL11	0	0	0	0	0	0	0	0	0	0	0.192	0	0	0	0	0
SRCIN1	0	0	0	0	0	0.192	0	0	0	0	0	0	0	0	0	0
PDIA2	0	0	0	0	0.192	0	0	0	0	0	0	0	0	0	0	0
GBP2	0	0	0	0.192	0	0	0	0	0	0	0	0	0	0	0	0
SNX18	0	0	0	0.192	0	0	0	0	0	0	0	0	0	0	0	0
MORF4L1P1	0.192	0	0	0	0	0	0	0	0	0	0	0	0	0	0	0
NIDDM3	0.192	0	0	0	0	0	0	0	0	0	0	0	0	0	0	0
BEST3	0	0	0	0	0	0	0	0	0	0	0	0	0	0	0	0.191
C1QTNF6	0	0	0	0	0	0	0	0	0	0	0	0	0	0	0	0.191
PAFAH1B1P2	0	0	0	0	0	0	0	0	0	0	0	0	0	0	0.191	0
RRM2P2	0	0	0	0	0	0	0	0	0	0	0	0	0	0	0.191	0
TRNS2	0	0	0	0	0	0	0	0	0	0	0	0.191	0	0	0	0
CNGA3	0	0	0	0	0	0	0	0	0	0.191	0	0	0	0	0	0
BANF2	0	0	0	0.191	0	0	0	0	0	0	0	0	0	0	0	0
GJA6P	0	0	0	0.191	0	0	0	0	0	0	0	0	0	0	0	0
CEP72	0.191	0	0	0	0	0	0	0	0	0	0	0	0	0	0	0
PHKG2	0.191	0	0	0	0	0	0	0	0	0	0	0	0	0	0	0
GNB2	0	0	0	0	0	0	0	0	0	0	0	0	0	0	0	0.19
KCNA5	0	0	0	0	0	0	0	0	0	0	0	0	0	0	0	0.19
PCBD1	0	0	0	0	0	0	0	0	0	0	0	0	0	0	0	0.19
PRKAG1	0	0	0	0	0	0	0	0	0	0	0	0	0	0	0	0.19
FKBP9	0	0	0	0	0	0	0	0	0	0	0	0	0	0	0.19	0
SNAPC3	0	0	0	0	0	0	0	0	0	0	0.19	0	0	0	0	0
G3BP1	0	0	0	0	0	0	0	0	0.19	0	0	0	0	0	0	0
RNF41	0	0	0	0	0	0	0	0	0.19	0	0	0	0	0	0	0

UGT1A9	0	0	0	0	0	0	0.19	0	0	0	0	0	0	0	0	0
TBC1D5	0	0	0	0.19	0	0	0	0	0	0	0	0	0	0	0	0
PCYOX1	0.19	0	0	0	0	0	0	0	0	0	0	0	0	0	0	0
AASTH38	0	0	0	0	0	0	0	0	0	0	0	0	0	0	0	0.189
AASTH7	0	0	0	0	0	0	0	0	0	0	0	0	0	0	0	0.189
ACACB	0	0	0	0	0	0	0	0	0	0	0	0	0	0	0	0.189
ACCS	0	0	0	0	0	0	0	0	0	0	0	0	0	0	0	0.189
DENND4C	0	0	0	0	0	0	0	0	0	0	0	0	0	0	0	0.189
PIGM	0	0	0	0	0	0	0	0	0	0	0	0	0	0	0	0.189
PLN	0	0	0	0	0	0	0	0	0	0	0	0	0	0	0	0.189
RYR1	0	0	0	0	0	0	0	0	0	0	0	0	0	0	0	0.189
TNNI3	0	0	0	0	0	0	0	0	0	0	0	0	0	0	0	0.189
C2CD4A	0	0	0	0	0	0	0	0	0	0	0	0	0	0	0.189	0
UNC93A	0	0	0	0	0	0	0	0	0	0	0.189	0	0	0	0	0
HERC2P7	0	0	0	0	0	0.189	0	0	0	0	0	0	0	0	0	0
C11ORF24	0.189	0	0	0	0	0	0	0	0	0	0	0	0	0	0	0
MEGF10	0.189	0	0	0	0	0	0	0	0	0	0	0	0	0	0	0
TCTE3	0	0	0	0	0	0	0	0	0	0	0	0	0	0	0	0.188
LRRTM4	0	0	0	0	0	0	0	0	0	0	0	0	0	0	0.188	0
YKT6	0	0	0	0	0	0	0	0	0	0	0	0	0	0	0.188	0
IFT43	0	0	0	0	0	0	0	0	0	0	0	0.188	0	0	0	0
NT5C1B	0	0	0	0	0	0	0	0	0	0	0.188	0	0	0	0	0
QKI	0	0	0	0.188	0	0	0	0	0	0	0	0	0	0	0	0
CHAD	0	0	0	0	0	0	0	0	0	0	0	0	0	0	0.187	0
MIXL1	0	0	0	0	0	0	0	0	0	0	0	0	0	0	0.187	0
OR8J2	0	0	0	0	0	0	0	0	0	0	0	0	0	0	0.187	0
EFEMP1	0	0	0	0	0	0	0	0	0	0.187	0	0	0	0	0	0
RHO	0	0	0	0	0	0	0	0	0	0.187	0	0	0	0	0	0
TNC	0	0	0	0	0	0	0	0	0	0.187	0	0	0	0	0	0
BDKRB1	0	0	0	0	0	0.187	0	0	0	0	0	0	0	0	0	0
CBLN2	0	0	0	0	0	0.187	0	0	0	0	0	0	0	0	0	0
AGPAT2	0	0	0	0.187	0	0	0	0	0	0	0	0	0	0	0	0
LRP10	0.187	0	0	0	0	0	0	0	0	0	0	0	0	0	0	0
NKX6-3	0.187	0	0	0	0	0	0	0	0	0	0	0	0	0	0	0
SFI1	0.187	0	0	0	0	0	0	0	0	0	0	0	0	0	0	0
AREI	0	0	0	0	0	0	0	0	0	0	0	0	0	0	0.186	0
HESX1	0	0	0	0	0	0	0	0	0	0	0	0	0	0	0.186	0
SYT13	0	0	0	0	0	0	0	0	0	0	0	0	0	0	0.186	0
CLDN14	0	0	0	0	0	0	0	0	0	0	0	0.186	0	0	0	0
MIR219-1	0	0	0	0	0	0	0	0	0	0	0.186	0	0	0	0	0
SIN3A	0	0	0	0	0	0	0	0	0	0	0.186	0	0	0	0	0

AHDC1	0	0	0	0	0	0	0	0	0.186	0	0	0	0	0	0	0
SLC38A1	0	0	0	0	0	0.186	0	0	0	0	0	0	0	0	0	0
LOC441869	0	0	0	0.186	0	0	0	0	0	0	0	0	0	0	0	0
SLC25A5P3	0	0	0	0.186	0	0	0	0	0	0	0	0	0	0	0	0
GSTA7P	0.186	0	0	0	0	0	0	0	0	0	0	0	0	0	0	0
NUS1P3	0.186	0	0	0	0	0	0	0	0	0	0	0	0	0	0	0
SGK3	0.186	0	0	0	0	0	0	0	0	0	0	0	0	0	0	0
VN1R2	0.186	0	0	0	0	0	0	0	0	0	0	0	0	0	0	0
C6ORF170	0	0	0	0	0	0	0	0	0	0	0	0	0	0	0	0.185
GBX2	0	0	0	0	0	0	0	0	0	0	0	0	0	0	0.185	0
SFMBT2	0	0	0	0	0	0	0	0	0	0	0	0	0	0	0.185	0
NUDT1	0	0	0	0	0	0	0	0	0	0	0.185	0	0	0	0	0
NEUROD1	0	0	0	0	0	0	0	0	0	0.185	0	0	0	0	0	0
IGFN1	0	0	0	0.185	0	0	0	0	0	0	0	0	0	0	0	0
CORIN	0	0	0	0	0	0	0	0	0	0	0	0	0	0	0	0.184
PRDM16	0	0	0	0	0	0	0	0	0	0	0	0	0	0	0	0.184
TMEM38A	0	0	0	0	0	0	0	0	0	0	0	0	0	0	0	0.184
GPR62	0	0	0	0	0	0	0	0	0	0	0	0	0	0	0.184	0
LELP1	0	0	0	0	0	0	0	0	0	0	0	0	0	0	0.184	0
PROL1	0	0	0	0	0	0	0	0	0	0	0	0	0	0	0.184	0
SDHAF2	0	0	0	0	0	0	0	0	0	0	0	0.184	0	0	0	0
KAT2B	0	0	0	0	0	0	0	0	0	0	0.184	0	0	0	0	0
RP6	0	0	0	0	0	0	0	0	0	0.184	0	0	0	0	0	0
EPB41L2	0	0	0	0.184	0	0	0	0	0	0	0	0	0	0	0	0
SGSM3	0	0	0	0.184	0	0	0	0	0	0	0	0	0	0	0	0
KLHL23	0.184	0	0	0	0	0	0	0	0	0	0	0	0	0	0	0
SLC5A2	0	0	0	0	0	0	0	0	0	0	0	0	0	0	0	0.183
TM4SF18	0	0	0	0	0	0	0	0	0	0	0	0	0	0	0	0.183
HNF4GP1	0	0	0	0	0	0	0	0	0	0	0	0	0	0	0.183	0
PSORS8	0	0	0	0	0	0	0	0	0	0	0	0	0	0	0.183	0
SEPT12	0	0	0	0	0	0	0	0	0	0	0	0	0	0	0.183	0
SPRR2D	0	0	0	0	0	0	0	0	0	0	0	0	0	0	0.183	0
SYT9	0	0	0	0	0	0	0	0	0	0	0	0	0	0	0.183	0
ATOH7	0	0	0	0	0	0	0	0	0	0.183	0	0	0	0	0	0
LHX8	0	0	0	0	0	0	0	0	0	0.183	0	0	0	0	0	0
RGS11	0	0	0	0	0	0	0	0	0	0.183	0	0	0	0	0	0
ERBB2IP	0	0	0	0.183	0	0	0	0	0	0	0	0	0	0	0	0
HSPB9	0	0	0	0.183	0	0	0	0	0	0	0	0	0	0	0	0
LAMA4	0	0	0	0.183	0	0	0	0	0	0	0	0	0	0	0	0
LRFN4	0	0	0	0.183	0	0	0	0	0	0	0	0	0	0	0	0
RPL23AP3	0.183	0	0	0	0	0	0	0	0	0	0	0	0	0	0	0

ADRA1A	0	0	0	0	0	0	0	0	0	0	0	0	0	0	0	0.182
ATP8A1	0	0	0	0	0	0	0	0	0	0	0	0	0	0	0.182	0
GPR97	0	0	0	0	0	0	0	0	0	0	0	0	0	0	0.182	0
OPN4	0	0	0	0	0	0	0	0	0	0.182	0	0	0	0	0	0
PTBP1	0	0	0	0	0	0	0	0	0.182	0	0	0	0	0	0	0
DDX24	0	0	0	0	0.182	0	0	0	0	0	0	0	0	0	0	0
IGKV1-37	0	0	0	0	0.182	0	0	0	0	0	0	0	0	0	0	0
KLHL22	0.182	0	0	0	0	0	0	0	0	0	0	0	0	0	0	0
PYGB	0.182	0	0	0	0	0	0	0	0	0	0	0	0	0	0	0
GEM	0	0	0	0	0	0	0	0	0	0	0	0	0	0	0	0.181
PAICSP2	0	0	0	0	0	0	0	0	0	0	0	0	0	0	0	0.181
SLC2A7	0	0	0	0	0	0	0	0	0	0	0	0	0	0	0	0.181
ASS1P7	0	0	0	0	0	0	0	0	0	0	0	0	0	0	0.181	0
KIAA0913	0	0	0	0	0	0	0	0	0	0	0	0	0	0	0.181	0
PLCH2	0	0	0	0	0	0	0	0	0	0	0	0	0	0	0.181	0
SMOX	0	0	0	0	0	0	0	0	0	0	0	0	0	0	0.181	0
TFP1	0	0	0	0	0	0	0	0	0	0	0	0	0	0	0.181	0
MEX3A	0	0	0	0	0	0	0	0	0.181	0	0	0	0	0	0	0
HSD11B1	0	0	0	0	0.181	0	0	0	0	0	0	0	0	0	0	0
COPD3	0	0	0	0.181	0	0	0	0	0	0	0	0	0	0	0	0
ITGB4	0	0	0	0.181	0	0	0	0	0	0	0	0	0	0	0	0
MAL	0	0	0	0.181	0	0	0	0	0	0	0	0	0	0	0	0
RNF10	0	0	0	0.181	0	0	0	0	0	0	0	0	0	0	0	0
C2CD2	0.181	0	0	0	0	0	0	0	0	0	0	0	0	0	0	0
SLC2AXP1	0	0	0	0	0	0	0	0	0	0	0	0	0	0	0	0.18
COLQ	0	0	0	0	0	0	0	0	0	0	0	0	0.18	0	0	0
KAT2A	0	0	0	0	0	0	0	0	0	0	0.18	0	0	0	0	0
KCNIP4-IT1	0	0	0	0	0	0	0	0	0	0	0.18	0	0	0	0	0
KLHL20	0	0	0	0	0	0	0	0	0	0	0.18	0	0	0	0	0
LIMK1	0	0	0	0	0	0	0	0	0	0	0.18	0	0	0	0	0
GNAT2	0	0	0	0	0	0	0	0	0	0.18	0	0	0	0	0	0
RAX2	0	0	0	0	0	0	0	0	0	0.18	0	0	0	0	0	0
RORB	0	0	0	0	0	0	0	0	0	0.18	0	0	0	0	0	0
ERBB4	0	0	0	0	0	0	0	0	0.18	0	0	0	0	0	0	0
NDUFB7	0	0	0	0	0	0	0.18	0	0	0	0	0	0	0	0	0
GBAP1	0	0	0	0	0.18	0	0	0	0	0	0	0	0	0	0	0
HMBOX1	0	0	0	0	0.18	0	0	0	0	0	0	0	0	0	0	0
IFNA7	0	0	0	0	0.18	0	0	0	0	0	0	0	0	0	0	0
SRA1	0.18	0	0	0	0	0	0	0	0	0	0	0	0	0	0	0
TOMM70A	0.18	0	0	0	0	0	0	0	0	0	0	0	0	0	0	0
ZXDC	0.18	0	0	0	0	0	0	0	0	0	0	0	0	0	0	0

TTLL1	0	0	0	0	0	0	0	0	0	0	0	0	0	0	0	0.179
NAA11	0	0	0	0	0	0	0	0	0	0	0	0	0	0	0.179	0
ATOH1	0	0	0	0	0	0	0	0	0	0	0	0.179	0	0	0	0
VIP	0	0	0	0	0	0	0	0	0	0	0.179	0	0	0	0	0
EEF2K	0	0	0	0	0	0	0	0	0.179	0	0	0	0	0	0	0
UNC13A	0	0	0	0	0	0.179	0	0	0	0	0	0	0	0	0	0
CCR2	0	0	0	0	0.179	0	0	0	0	0	0	0	0	0	0	0
GPR1	0	0	0	0	0.179	0	0	0	0	0	0	0	0	0	0	0
DIAPH2	0.179	0	0	0	0	0	0	0	0	0	0	0	0	0	0	0
IGSF8	0.179	0	0	0	0	0	0	0	0	0	0	0	0	0	0	0
MAPK11	0.179	0	0	0	0	0	0	0	0	0	0	0	0	0	0	0
PDGFC	0.179	0	0	0	0	0	0	0	0	0	0	0	0	0	0	0
PPP1R3B	0.179	0	0	0	0	0	0	0	0	0	0	0	0	0	0	0
MEGF8	0	0	0	0	0	0	0	0	0	0	0	0	0	0	0	0.178
SFXN4	0	0	0	0	0	0	0	0	0	0	0	0	0	0	0	0.178
IGKV1OR-4	0	0	0	0	0	0	0	0	0	0	0	0	0	0	0.178	0
SEMA5B	0	0	0	0	0	0	0	0	0	0	0	0.178	0	0	0	0
BAI1	0	0	0	0	0	0	0	0	0	0	0.178	0	0	0	0	0
LSM2	0	0	0	0	0	0	0	0	0	0	0.178	0	0	0	0	0
ATP2C1	0	0	0	0	0.178	0	0	0	0	0	0	0	0	0	0	0
UBBP3	0	0	0	0.178	0	0	0	0	0	0	0	0	0	0	0	0
LPA	0.178	0	0	0	0	0	0	0	0	0	0	0	0	0	0	0
SCARF1	0.178	0	0	0	0	0	0	0	0	0	0	0	0	0	0	0
AMPD1	0	0	0	0	0	0	0	0	0	0	0	0	0	0	0	0.177
ODCP	0	0	0	0	0	0	0	0	0	0	0	0	0	0	0.177	0
RRM2P4	0	0	0	0	0	0	0	0	0	0	0	0	0	0	0.177	0
CDAN3	0	0	0	0	0	0	0	0	0	0	0	0.177	0	0	0	0
SEMA4A	0	0	0	0	0	0	0	0	0	0	0	0.177	0	0	0	0
ENDOV	0	0	0	0	0	0	0	0	0	0	0.177	0	0	0	0	0
RN5S2@	0	0	0	0	0	0	0	0	0	0	0.177	0	0	0	0	0
ZACN	0	0	0	0	0	0.177	0	0	0	0	0	0	0	0	0	0
GJC1	0	0	0	0.177	0	0	0	0	0	0	0	0	0	0	0	0
C10ORF10	0.177	0	0	0	0	0	0	0	0	0	0	0	0	0	0	0
MORF4L1P3	0.177	0	0	0	0	0	0	0	0	0	0	0	0	0	0	0
ZNF136	0.177	0	0	0	0	0	0	0	0	0	0	0	0	0	0	0
ZNF600	0.177	0	0	0	0	0	0	0	0	0	0	0	0	0	0	0
CAV3	0	0	0	0	0	0	0	0	0	0	0	0	0	0	0	0.176
CD40LG	0	0	0	0	0	0	0	0	0	0	0	0	0	0	0	0.176
MLYCD	0	0	0	0	0	0	0	0	0	0	0	0	0	0	0	0.176
SLC2A14	0	0	0	0	0	0	0	0	0	0	0	0	0	0	0	0.176
GPNMB	0	0	0	0	0	0	0	0	0	0	0	0	0	0	0.176	0

OCRL	0	0	0	0	0	0	0	0	0	0	0	0	0.176	0	0	0
BUD13	0	0	0	0	0.176	0	0	0	0	0	0	0	0	0	0	0
TSPAN9	0.176	0	0	0	0	0	0	0	0	0	0	0	0	0	0	0
AZIN1	0	0	0	0	0	0	0	0	0	0	0	0	0	0	0.175	0
TRGJ2	0	0	0	0	0	0	0	0	0	0	0	0	0	0	0.175	0
PDCD2	0	0	0	0	0	0	0	0	0	0	0.175	0	0	0	0	0
KHDRBS2	0	0	0	0	0	0	0	0	0.175	0	0	0	0	0	0	0
MUSK	0	0	0	0.175	0	0	0	0	0	0	0	0	0	0	0	0
TMPO	0	0	0	0.175	0	0	0	0	0	0	0	0	0	0	0	0
C13ORF41	0.175	0	0	0	0	0	0	0	0	0	0	0	0	0	0	0
PRKAA2	0	0	0	0	0	0	0	0	0	0	0	0	0	0	0	0.174
SLC2A8	0	0	0	0	0	0	0	0	0	0	0	0	0	0	0	0.174
TMEM114	0	0	0	0	0	0	0	0	0	0	0	0	0	0	0	0.174
GTPBP3	0	0	0	0	0	0	0	0	0	0	0	0.174	0	0	0	0
MPG	0	0	0	0	0	0	0	0	0	0	0.174	0	0	0	0	0
NRTN	0	0	0	0	0	0	0	0	0	0	0.174	0	0	0	0	0
SAP30	0	0	0	0	0	0	0	0	0	0	0.174	0	0	0	0	0
TUSC1	0	0	0	0	0	0	0	0	0	0	0.174	0	0	0	0	0
EEF1A1P13	0	0	0	0	0	0	0	0	0	0.174	0	0	0	0	0	0
RDH8	0	0	0	0	0	0	0	0	0	0.174	0	0	0	0	0	0
ISYNA1	0.174	0	0	0	0	0	0	0	0	0	0	0	0	0	0	0
NUDT16L1	0.174	0	0	0	0	0	0	0	0	0	0	0	0	0	0	0
DNASE1L2	0	0	0	0	0	0	0	0	0	0	0	0	0	0	0.173	0
SNORD36B	0	0	0	0	0	0	0	0	0	0	0	0	0	0	0.173	0
TMPRSS3	0	0	0	0	0	0	0	0	0	0	0	0.173	0	0	0	0
COP	0	0	0	0	0	0	0	0	0	0	0.173	0	0	0	0	0
POLR3C	0	0	0	0	0	0	0	0	0	0	0.173	0	0	0	0	0
PENK	0	0	0	0	0	0	0	0	0	0.173	0	0	0	0	0	0
NPY1R	0	0	0	0	0	0.173	0	0	0	0	0	0	0	0	0	0
KIAA0146	0	0	0	0.173	0	0	0	0	0	0	0	0	0	0	0	0
ZNF295	0.173	0	0	0	0	0	0	0	0	0	0	0	0	0	0	0
SLC16A3	0	0	0	0	0	0	0	0	0	0	0	0	0	0	0	0.172
SNTB2	0	0	0	0	0	0	0	0	0	0	0	0	0	0	0	0.172
SPR	0	0	0	0	0	0	0	0	0	0	0	0	0	0	0	0.172
SYPL2	0	0	0	0	0	0	0	0	0	0	0	0	0	0	0	0.172
MCTP2	0	0	0	0	0	0	0	0	0	0	0	0	0	0	0.172	0
TBL1X	0	0	0	0	0	0	0	0	0	0	0	0	0	0	0.172	0
CNRIP1	0	0	0	0	0	0	0	0	0	0	0.172	0	0	0	0	0
TAF6	0	0	0	0	0	0	0	0	0	0	0.172	0	0	0	0	0
CARTPT	0	0	0	0	0	0	0	0	0	0.172	0	0	0	0	0	0
ATP6V1D	0	0	0	0	0	0	0	0	0.172	0	0	0	0	0	0	0

MAGEE1	0	0	0	0.172	0	0	0	0	0	0	0	0	0	0	0	0
C1HR	0.172	0	0	0	0	0	0	0	0	0	0	0	0	0	0	0
MORF4L1P2	0.172	0	0	0	0	0	0	0	0	0	0	0	0	0	0	0
CD14	0	0	0	0	0	0	0	0	0	0	0	0	0	0	0	0.171
CYSLTR1	0	0	0	0	0	0	0	0	0	0	0	0	0	0	0	0.171
RELA	0	0	0	0	0	0	0	0	0	0	0	0	0	0	0	0.171
SLC5A12	0	0	0	0	0	0	0	0	0	0	0	0	0	0	0	0.171
HIST2H2BA	0	0	0	0	0	0	0	0	0	0	0	0	0	0	0.171	0
POTEM	0	0	0	0	0	0	0	0	0	0	0	0	0	0	0.171	0
MBS1	0	0	0	0	0	0	0	0	0	0	0	0.171	0	0	0	0
FRS2	0	0	0	0	0	0	0	0	0	0	0.171	0	0	0	0	0
GTF2A1	0	0	0	0	0	0	0	0	0	0	0.171	0	0	0	0	0
TAF9	0	0	0	0	0	0	0	0	0	0	0.171	0	0	0	0	0
ARMS2	0	0	0	0	0	0	0	0	0	0.171	0	0	0	0	0	0
BZW2	0	0	0	0	0	0	0	0.171	0	0	0	0	0	0	0	0
DYNLL2	0	0	0	0.171	0	0	0	0	0	0	0	0	0	0	0	0
A3GALT2P	0	0	0	0	0	0	0	0	0	0	0	0	0	0	0	0.17
ANON	0	0	0	0	0	0	0	0	0	0	0	0	0	0	0	0.17
INVS	0	0	0	0	0	0	0	0	0	0	0	0	0	0	0	0.17
TNNT1	0	0	0	0	0	0	0	0	0	0	0	0	0	0	0	0.17
SDHC	0	0	0	0	0	0	0	0	0	0	0	0.17	0	0	0	0
GDA	0	0	0	0	0	0	0	0	0	0	0.17	0	0	0	0	0
TADA1	0	0	0	0	0	0	0	0	0	0	0.17	0	0	0	0	0
CDHR1	0	0	0	0	0	0	0	0	0	0.17	0	0	0	0	0	0
TULP1	0	0	0	0	0	0	0	0	0	0.17	0	0	0	0	0	0
NUMBL	0	0	0	0	0.17	0	0	0	0	0	0	0	0	0	0	0
CLEC18A	0.17	0	0	0	0	0	0	0	0	0	0	0	0	0	0	0
KIAA0430	0.17	0	0	0	0	0	0	0	0	0	0	0	0	0	0	0
MIS18A	0.17	0	0	0	0	0	0	0	0	0	0	0	0	0	0	0
OR7D2	0.17	0	0	0	0	0	0	0	0	0	0	0	0	0	0	0
S100A8	0.17	0	0	0	0	0	0	0	0	0	0	0	0	0	0	0
SRI	0	0	0	0	0	0	0	0	0	0	0	0	0	0	0	0.169
SYT15	0	0	0	0	0	0	0	0	0	0	0	0	0	0	0.169	0
TRNAL47P	0	0	0	0	0	0	0	0	0	0	0.169	0	0	0	0	0
ATP1B1	0	0	0	0	0	0.169	0	0	0	0	0	0	0	0	0	0
BDKRB2	0	0	0	0	0	0.169	0	0	0	0	0	0	0	0	0	0
CLCN3	0	0	0	0	0	0.169	0	0	0	0	0	0	0	0	0	0
SERPINE2	0	0	0	0	0.169	0	0	0	0	0	0	0	0	0	0	0
LPAR1	0	0	0	0.169	0	0	0	0	0	0	0	0	0	0	0	0
RPSAP13	0.169	0	0	0	0	0	0	0	0	0	0	0	0	0	0	0
AGMO	0	0	0	0	0	0	0	0	0	0	0	0	0	0	0	0.168

MRGPRD	0	0	0	0	0	0	0	0	0	0	0	0.168	0	0	0	0
PCDH20	0	0	0	0	0	0	0	0	0	0	0	0.168	0	0	0	0
AMYP1	0	0	0	0	0	0	0	0	0	0	0.168	0	0	0	0	0
SUDS3	0	0	0	0	0	0	0	0	0	0	0.168	0	0	0	0	0
TAF12	0	0	0	0	0	0	0	0	0	0	0.168	0	0	0	0	0
OPN1LW	0	0	0	0	0	0	0	0	0	0.168	0	0	0	0	0	0
SAG	0	0	0	0	0	0	0	0	0	0.168	0	0	0	0	0	0
INPP5J	0.168	0	0	0	0	0	0	0	0	0	0	0	0	0	0	0
PHKBP2	0.168	0	0	0	0	0	0	0	0	0	0	0	0	0	0	0
RASA4	0.168	0	0	0	0	0	0	0	0	0	0	0	0	0	0	0
GYG2P1	0	0	0	0	0	0	0	0	0	0	0	0	0	0	0.167	0
GUCA1B	0	0	0	0	0	0	0	0	0	0.167	0	0	0	0	0	0
OPN1MW	0	0	0	0	0	0	0	0	0	0.167	0	0	0	0	0	0
JAKMIP2	0	0	0	0	0	0.167	0	0	0	0	0	0	0	0	0	0
KCNJ11	0	0	0	0	0	0.167	0	0	0	0	0	0	0	0	0	0
KCNK6	0	0	0	0	0	0.167	0	0	0	0	0	0	0	0	0	0
C3ORF17	0.167	0	0	0	0	0	0	0	0	0	0	0	0	0	0	0
LOC729316	0.167	0	0	0	0	0	0	0	0	0	0	0	0	0	0	0
PIGZ	0	0	0	0	0	0	0	0	0	0	0	0	0	0	0	0.166
SLC2A4	0	0	0	0	0	0	0	0	0	0	0	0	0	0	0	0.166
OR2C1	0	0	0	0	0	0	0	0	0	0	0	0.166	0	0	0	0
NDUFS5	0	0	0	0	0	0	0	0	0	0	0.166	0	0	0	0	0
PDE7B	0	0	0	0	0	0	0	0	0	0	0.166	0	0	0	0	0
PYCARD	0	0	0	0	0	0	0	0	0	0	0.166	0	0	0	0	0
CRB1	0	0	0	0	0	0	0	0	0	0.166	0	0	0	0	0	0
RDH12	0	0	0	0	0	0	0	0	0	0.166	0	0	0	0	0	0
RNASEH2B	0	0	0	0	0	0	0.166	0	0	0	0	0	0	0	0	0
NPY2R	0	0	0	0	0	0.166	0	0	0	0	0	0	0	0	0	0
MMAA	0.166	0	0	0	0	0	0	0	0	0	0	0	0	0	0	0
TLE3	0.166	0	0	0	0	0	0	0	0	0	0	0	0	0	0	0
DAND5	0	0	0	0	0	0	0	0	0	0	0	0	0	0	0	0.165
PRO2268	0	0	0	0	0	0	0	0	0	0	0	0	0	0	0	0.165
CDRT15P2	0	0	0	0	0	0	0	0	0	0	0	0	0	0	0.165	0
VN2R1P	0	0	0	0	0	0	0	0	0	0	0	0.165	0	0	0	0
PDE6C	0	0	0	0	0	0	0	0	0	0.165	0	0	0	0	0	0
NSFL1C	0	0	0	0	0	0	0	0	0.165	0	0	0	0	0	0	0
UBBP4	0	0	0	0.165	0	0	0	0	0	0	0	0	0	0	0	0
MIR204	0.165	0	0	0	0	0	0	0	0	0	0	0	0	0	0	0
SIRPG	0.165	0	0	0	0	0	0	0	0	0	0	0	0	0	0	0
ADRA2A	0	0	0	0	0	0	0	0	0	0	0	0	0	0	0	0.164
B3GALNT2	0	0	0	0	0	0	0	0	0	0	0	0	0	0	0	0.164

MIR377	0	0	0	0	0	0	0	0	0	0	0	0	0	0	0	0.164
ITGBL1	0	0	0	0	0	0	0	0	0	0	0	0	0	0	0.164	0
CLCN6	0	0	0	0	0	0	0	0.164	0	0	0	0	0	0	0	0
HNRPDL	0	0	0	0	0.164	0	0	0	0	0	0	0	0	0	0	0
HDLC3	0.164	0	0	0	0	0	0	0	0	0	0	0	0	0	0	0
RFK	0.164	0	0	0	0	0	0	0	0	0	0	0	0	0	0	0
PKD1L1	0	0	0	0	0	0	0	0	0	0	0	0	0	0	0	0.163
FADS1	0	0	0	0	0	0	0	0	0	0	0	0	0	0	0.163	0
HSPB11	0	0	0	0	0	0	0	0	0	0	0	0	0	0	0.163	0
IGKV1OR1-1	0	0	0	0	0	0	0	0	0	0	0	0	0	0	0.163	0
IGKV1OR22-1	0	0	0	0	0	0	0	0	0	0	0	0	0	0	0.163	0
CHDM	0	0	0	0	0	0	0	0	0	0	0	0.163	0	0	0	0
GMPR	0	0	0	0	0	0	0	0	0	0	0.163	0	0	0	0	0
RP24	0	0	0	0	0	0	0	0	0	0.163	0	0	0	0	0	0
UFD1L	0	0	0	0	0	0	0	0	0.163	0	0	0	0	0	0	0
CXCL12	0	0	0	0	0.163	0	0	0	0	0	0	0	0	0	0	0
C1ORF167	0.163	0	0	0	0	0	0	0	0	0	0	0	0	0	0	0
ZC3H12B	0.163	0	0	0	0	0	0	0	0	0	0	0	0	0	0	0
TNNI2	0	0	0	0	0	0	0	0	0	0	0	0	0	0	0	0.162
HMBS	0	0	0	0	0	0	0	0	0	0	0	0.162	0	0	0	0
OR10A4	0	0	0	0	0	0	0	0	0	0	0	0.162	0	0	0	0
MSH3	0	0	0	0	0	0	0	0	0	0	0.162	0	0	0	0	0
PYDC2	0	0	0	0	0	0	0	0	0	0	0.162	0	0	0	0	0
FHASD	0	0	0	0	0	0	0	0	0	0.162	0	0	0	0	0	0
PDE6H	0	0	0	0	0	0	0	0	0	0.162	0	0	0	0	0	0
DIXDC1	0.162	0	0	0	0	0	0	0	0	0	0	0	0	0	0	0
MMAB	0.162	0	0	0	0	0	0	0	0	0	0	0	0	0	0	0
PCDH18	0.162	0	0	0	0	0	0	0	0	0	0	0	0	0	0	0
PPP1R3D	0.162	0	0	0	0	0	0	0	0	0	0	0	0	0	0	0
PZP	0.162	0	0	0	0	0	0	0	0	0	0	0	0	0	0	0
ZNF134	0.162	0	0	0	0	0	0	0	0	0	0	0	0	0	0	0
AQP7P1	0	0	0	0	0	0	0	0	0	0	0	0	0	0	0	0.161
C7ORF13	0	0	0	0	0	0	0	0	0	0	0	0	0	0	0	0.161
DIDO1	0	0	0	0	0	0	0	0	0	0	0	0	0	0	0	0.161
JPH1	0	0	0	0	0	0	0	0	0	0	0	0	0	0	0	0.161
MYSA	0	0	0	0	0	0	0	0	0	0	0	0	0	0	0	0.161
PIGS	0	0	0	0	0	0	0	0	0	0	0	0	0	0	0	0.161
FKBP7	0	0	0	0	0	0	0	0	0	0	0	0	0	0	0.161	0
DRG2	0	0	0	0	0	0	0	0	0	0	0	0.161	0	0	0	0
TMC2	0	0	0	0	0	0	0	0	0	0	0	0.161	0	0	0	0
EPN1	0	0	0	0	0	0	0	0	0	0	0.161	0	0	0	0	0

TAF1B	0	0	0	0	0	0	0	0	0	0	0.161	0	0	0	0	0
PALMD	0	0	0	0	0	0	0	0	0	0.161	0	0	0	0	0	0
HADHA	0	0	0	0	0	0	0.161	0	0	0	0	0	0	0	0	0
MBD1	0	0	0	0	0	0	0.161	0	0	0	0	0	0	0	0	0
C17ORF39	0	0	0	0	0	0.161	0	0	0	0	0	0	0	0	0	0
FGF22	0	0	0	0	0	0.161	0	0	0	0	0	0	0	0	0	0
FILIP1	0	0	0	0	0	0.161	0	0	0	0	0	0	0	0	0	0
ZNF488	0	0	0	0.161	0	0	0	0	0	0	0	0	0	0	0	0
C1ORF135	0.161	0	0	0	0	0	0	0	0	0	0	0	0	0	0	0
SHMT2	0.161	0	0	0	0	0	0	0	0	0	0	0	0	0	0	0
ART5	0	0	0	0	0	0	0	0	0	0	0	0	0	0	0	0.16
KIAA1199	0	0	0	0	0	0	0	0	0	0	0	0.16	0	0	0	0
MYO1C	0	0	0	0	0	0	0	0	0	0	0	0.16	0	0	0	0
OR6A2	0	0	0	0	0	0	0	0	0	0	0	0.16	0	0	0	0
GNAL	0	0	0	0	0	0	0	0	0	0	0.16	0	0	0	0	0
OPN1SW	0	0	0	0	0	0	0	0	0	0.16	0	0	0	0	0	0
MRPL18	0	0	0	0	0	0	0.16	0	0	0	0	0	0	0	0	0
MTFMT	0	0	0	0	0	0	0.16	0	0	0	0	0	0	0	0	0
KCNJ13	0	0	0	0	0	0.16	0	0	0	0	0	0	0	0	0	0
ST7-AS2	0	0	0	0	0	0.16	0	0	0	0	0	0	0	0	0	0
OTUD4	0	0	0	0	0.16	0	0	0	0	0	0	0	0	0	0	0
DPM3	0	0	0	0	0	0	0	0	0	0	0	0	0	0	0	0.159
PRL	0	0	0	0	0	0	0	0	0	0	0	0	0	0	0	0.159
FSD1	0	0	0	0	0	0	0	0	0	0	0	0	0	0	0.159	0
NUDCD3	0	0	0	0	0	0	0	0	0	0	0.159	0	0	0	0	0
PRPF3	0	0	0	0	0	0	0	0	0	0.159	0	0	0	0	0	0
KCNF1	0	0	0	0	0	0.159	0	0	0	0	0	0	0	0	0	0
IGKV1D-17	0	0	0	0	0.159	0	0	0	0	0	0	0	0	0	0	0
HSPE1	0.159	0	0	0	0	0	0	0	0	0	0	0	0	0	0	0
PHF3	0.159	0	0	0	0	0	0	0	0	0	0	0	0	0	0	0
CATSPER3	0	0	0	0	0	0	0	0	0	0	0	0	0	0	0	0.158
KPTN	0	0	0	0	0	0	0	0	0	0	0	0.158	0	0	0	0
CSN1S1	0	0	0	0	0	0	0	0	0	0	0.158	0	0	0	0	0
C4ORF11	0	0	0	0	0	0	0	0	0	0.158	0	0	0	0	0	0
MFRP-C1QTNF5	0	0	0	0	0	0	0	0	0	0.158	0	0	0	0	0	0
GPC1	0	0	0	0	0.158	0	0	0	0	0	0	0	0	0	0	0
BW34	0.158	0	0	0	0	0	0	0	0	0	0	0	0	0	0	0
FRA13A	0.158	0	0	0	0	0	0	0	0	0	0	0	0	0	0	0
PDGFD	0.158	0	0	0	0	0	0	0	0	0	0	0	0	0	0	0
DDC	0	0	0	0	0	0	0	0	0	0	0	0	0	0	0	0.157
EVC2	0	0	0	0	0	0	0	0	0	0	0	0	0.157	0	0	0

KRT24	0	0	0	0	0	0	0	0	0	0	0	0.157	0	0	0	0
OTOG	0	0	0	0	0	0	0	0	0	0	0	0.157	0	0	0	0
MLF1	0	0	0	0	0	0	0	0	0	0	0.157	0	0	0	0	0
NEDD8	0	0	0	0	0	0	0	0	0	0	0.157	0	0	0	0	0
NLRP10	0	0	0	0	0	0	0	0	0	0	0.157	0	0	0	0	0
MYO3B	0	0	0	0	0	0	0	0	0	0.157	0	0	0	0	0	0
VPS37D	0	0	0	0	0.157	0	0	0	0	0	0	0	0	0	0	0
CSNK1A1L	0.157	0	0	0	0	0	0	0	0	0	0	0	0	0	0	0
FRA7H	0.157	0	0	0	0	0	0	0	0	0	0	0	0	0	0	0
TRAP	0.157	0	0	0	0	0	0	0	0	0	0	0	0	0	0	0
ZNHIT2	0.157	0	0	0	0	0	0	0	0	0	0	0	0	0	0	0
FKBP1B	0	0	0	0	0	0	0	0	0	0	0	0	0	0	0	0.156
SNX22	0	0	0	0	0	0	0	0	0	0	0	0	0	0	0	0.156
FLVCR1	0	0	0	0	0	0	0	0	0	0	0	0.156	0	0	0	0
MHS2	0	0	0	0	0	0	0	0	0	0	0	0.156	0	0	0	0
NPHP4	0	0	0	0	0	0	0	0	0	0	0	0.156	0	0	0	0
FAUP1	0	0	0	0	0.156	0	0	0	0	0	0	0	0	0	0	0
FKBP5	0	0	0	0	0.156	0	0	0	0	0	0	0	0	0	0	0
RBP7	0	0	0	0.156	0	0	0	0	0	0	0	0	0	0	0	0
RHOQ	0	0	0	0.156	0	0	0	0	0	0	0	0	0	0	0	0
RIMKLA	0	0	0	0.156	0	0	0	0	0	0	0	0	0	0	0	0
RUFY1	0	0	0	0.156	0	0	0	0	0	0	0	0	0	0	0	0
SUN2	0	0	0	0.156	0	0	0	0	0	0	0	0	0	0	0	0
SYNE1	0	0	0	0.156	0	0	0	0	0	0	0	0	0	0	0	0
TMEM43	0	0	0	0.156	0	0	0	0	0	0	0	0	0	0	0	0
TRIM59-IFT80	0	0	0	0.156	0	0	0	0	0	0	0	0	0	0	0	0
EEF1B2P3	0.156	0	0	0	0	0	0	0	0	0	0	0	0	0	0	0
SLC2A5	0	0	0	0	0	0	0	0	0	0	0	0	0	0	0	0.155
DPY19L4	0	0	0	0	0	0	0	0	0	0	0.155	0	0	0	0	0
JPX	0	0	0	0	0	0	0	0	0	0	0.155	0	0	0	0	0
FGFR2	0	0	0	0	0	0	0	0	0	0.155	0	0	0	0	0	0
PDE6G	0	0	0	0	0	0	0	0	0	0.155	0	0	0	0	0	0
PITPNM3	0	0	0	0	0	0	0	0	0	0.155	0	0	0	0	0	0
PLEKHA1	0	0	0	0	0	0	0	0	0	0.155	0	0	0	0	0	0
PRPF8	0	0	0	0	0	0	0	0	0	0.155	0	0	0	0	0	0
COX11P1	0	0	0	0	0	0	0.155	0	0	0	0	0	0	0	0	0
SUCLG1	0	0	0	0	0	0	0.155	0	0	0	0	0	0	0	0	0
KCNG4	0	0	0	0	0	0.155	0	0	0	0	0	0	0	0	0	0
C16ORF70	0	0	0	0	0.155	0	0	0	0	0	0	0	0	0	0	0
CCR3	0	0	0	0	0.155	0	0	0	0	0	0	0	0	0	0	0
GSN	0	0	0	0	0.155	0	0	0	0	0	0	0	0	0	0	0

IGKV2-40	0	0	0	0	0.155	0	0	0	0	0	0	0	0	0	0	0
RIN2	0	0	0	0.155	0	0	0	0	0	0	0	0	0	0	0	0
ADAM19	0	0.155	0	0	0	0	0	0	0	0	0	0	0	0	0	0
CD9	0.155	0	0	0	0	0	0	0	0	0	0	0	0	0	0	0
INSRR	0.155	0	0	0	0	0	0	0	0	0	0	0	0	0	0	0
PGP	0	0	0	0	0	0	0	0	0	0	0	0	0	0	0	0.154
TOR3A	0	0	0	0	0	0	0	0	0	0	0	0	0	0	0	0.154
TPRKB	0	0	0	0	0	0	0	0	0	0	0	0	0	0	0	0.154
ATOD	0	0	0	0	0	0	0	0	0	0	0	0	0	0	0.154	0
GLC3C	0	0	0	0	0	0	0	0	0	0	0	0.154	0	0	0	0
KNCN	0	0	0	0	0	0	0	0	0	0	0	0.154	0	0	0	0
TTC26	0	0	0	0	0	0	0	0	0	0	0	0.154	0	0	0	0
GFM2	0	0	0	0	0	0	0.154	0	0	0	0	0	0	0	0	0
SLC24A2	0	0	0	0	0	0.154	0	0	0	0	0	0	0	0	0	0
EIF5AP3	0	0	0	0	0.154	0	0	0	0	0	0	0	0	0	0	0
GANAB	0	0	0	0	0.154	0	0	0	0	0	0	0	0	0	0	0
AIMP1	0	0.154	0	0	0	0	0	0	0	0	0	0	0	0	0	0
AIS2	0	0.154	0	0	0	0	0	0	0	0	0	0	0	0	0	0
COMMD1	0	0.154	0	0	0	0	0	0	0	0	0	0	0	0	0	0
GLIS3	0.154	0	0	0	0	0	0	0	0	0	0	0	0	0	0	0
ITGA11	0.154	0	0	0	0	0	0	0	0	0	0	0	0	0	0	0
OSTN	0.154	0	0	0	0	0	0	0	0	0	0	0	0	0	0	0
S100A1	0.154	0	0	0	0	0	0	0	0	0	0	0	0	0	0	0
S100A14	0.154	0	0	0	0	0	0	0	0	0	0	0	0	0	0	0
VWSM	0.154	0	0	0	0	0	0	0	0	0	0	0	0	0	0	0
CORD1	0	0	0	0	0	0	0	0	0	0	0	0	0	0	0.153	0
GBX1	0	0	0	0	0	0	0	0	0	0	0	0	0	0	0.153	0
H3F3AP4	0	0	0	0	0	0	0	0	0	0	0	0	0	0	0.153	0
SYT5	0	0	0	0	0	0	0	0	0	0	0	0	0	0	0.153	0
FAM151B	0	0	0	0	0	0	0	0	0	0	0.153	0	0	0	0	0
TAF1	0	0	0	0	0	0	0	0	0	0	0.153	0	0	0	0	0
UGT1A12P	0	0	0	0	0	0	0	0	0	0	0.153	0	0	0	0	0
PRPF31	0	0	0	0	0	0	0	0	0	0.153	0	0	0	0	0	0
VSX2	0	0	0	0	0	0	0	0	0	0.153	0	0	0	0	0	0
YOD1	0	0	0	0	0	0	0	0	0.153	0	0	0	0	0	0	0
PEX10	0	0	0	0	0	0	0.153	0	0	0	0	0	0	0	0	0
BRCD1	0	0	0	0	0	0.153	0	0	0	0	0	0	0	0	0	0
TOR2A	0	0	0	0	0.153	0	0	0	0	0	0	0	0	0	0	0
LRFN1	0	0	0	0.153	0	0	0	0	0	0	0	0	0	0	0	0
CLNS1A	0	0.153	0	0	0	0	0	0	0	0	0	0	0	0	0	0
DDIT3	0	0.153	0	0	0	0	0	0	0	0	0	0	0	0	0	0

APOH	0.153	0	0	0	0	0	0	0	0	0	0	0	0	0	0	0
LOC100506428	0.153	0	0	0	0	0	0	0	0	0	0	0	0	0	0	0
PPP3CB	0.153	0	0	0	0	0	0	0	0	0	0	0	0	0	0	0
S100A11P1	0.153	0	0	0	0	0	0	0	0	0	0	0	0	0	0	0
PTX3	0	0	0	0	0	0	0	0	0	0	0	0	0	0	0	0.152
ESYT3	0	0	0	0	0	0	0	0	0	0	0	0	0	0	0.152	0
TMC7	0	0	0	0	0	0	0	0	0	0	0	0.152	0	0	0	0
TMEM2	0	0	0	0	0	0	0	0	0	0	0	0.152	0	0	0	0
PCCA	0	0	0	0	0	0	0.152	0	0	0	0	0	0	0	0	0
SSTR1	0	0	0	0	0	0.152	0	0	0	0	0	0	0	0	0	0
GGA3	0	0	0	0	0.152	0	0	0	0	0	0	0	0	0	0	0
HMGCR	0	0	0	0	0.152	0	0	0	0	0	0	0	0	0	0	0
SNX16	0	0	0	0.152	0	0	0	0	0	0	0	0	0	0	0	0
ANKRD2	0	0.152	0	0	0	0	0	0	0	0	0	0	0	0	0	0
ATOX1	0	0.152	0	0	0	0	0	0	0	0	0	0	0	0	0	0
BIRC3	0	0.152	0	0	0	0	0	0	0	0	0	0	0	0	0	0
DBX1	0	0.152	0	0	0	0	0	0	0	0	0	0	0	0	0	0
CTNND2	0.152	0	0	0	0	0	0	0	0	0	0	0	0	0	0	0
DDX50	0.152	0	0	0	0	0	0	0	0	0	0	0	0	0	0	0
HDLCQ1	0.152	0	0	0	0	0	0	0	0	0	0	0	0	0	0	0
LRP5	0.152	0	0	0	0	0	0	0	0	0	0	0	0	0	0	0
MIC7	0.152	0	0	0	0	0	0	0	0	0	0	0	0	0	0	0
TM9SF4	0.152	0	0	0	0	0	0	0	0	0	0	0	0	0	0	0
CABLES2	0	0	0	0	0	0	0	0	0	0	0	0	0	0	0	0.151
PRKAG3	0	0	0	0	0	0	0	0	0	0	0	0	0	0	0	0.151
RAB25	0	0	0	0	0	0	0	0	0	0	0	0	0	0	0.151	0
ALDH7A1P3	0	0	0	0	0	0	0	0	0	0	0	0.151	0	0	0	0
MRGPRX4	0	0	0	0	0	0	0	0	0	0	0	0.151	0	0	0	0
HTRA1	0	0	0	0	0	0	0	0	0	0.151	0	0	0	0	0	0
LRAT	0	0	0	0	0	0	0	0	0	0.151	0	0	0	0	0	0
MIR103A2	0	0	0	0	0	0	0	0	0.151	0	0	0	0	0	0	0
PRSS1	0	0	0	0	0	0	0	0	0.151	0	0	0	0	0	0	0
CAPZA1P	0	0	0	0	0	0.151	0	0	0	0	0	0	0	0	0	0
IQSEC3	0	0	0	0	0	0.151	0	0	0	0	0	0	0	0	0	0
KCNJ16	0	0	0	0	0	0.151	0	0	0	0	0	0	0	0	0	0
CMTM2	0	0	0	0	0.151	0	0	0	0	0	0	0	0	0	0	0
FBXO40	0	0	0	0.151	0	0	0	0	0	0	0	0	0	0	0	0
C1ORF187	0	0.151	0	0	0	0	0	0	0	0	0	0	0	0	0	0
C20ORF54	0	0.151	0	0	0	0	0	0	0	0	0	0	0	0	0	0
C3AR1	0	0.151	0	0	0	0	0	0	0	0	0	0	0	0	0	0
C6ORF10	0	0.151	0	0	0	0	0	0	0	0	0	0	0	0	0	0

CALCB	0	0.151	0	0	0	0	0	0	0	0	0	0	0	0	0	0
CELSR3	0	0.151	0	0	0	0	0	0	0	0	0	0	0	0	0	0
CLDN5	0	0.151	0	0	0	0	0	0	0	0	0	0	0	0	0	0
CUTC	0	0.151	0	0	0	0	0	0	0	0	0	0	0	0	0	0
AKR7L	0.151	0	0	0	0	0	0	0	0	0	0	0	0	0	0	0
FRAT1	0.151	0	0	0	0	0	0	0	0	0	0	0	0	0	0	0
GPRIN3	0.151	0	0	0	0	0	0	0	0	0	0	0	0	0	0	0
MTHFD1	0.151	0	0	0	0	0	0	0	0	0	0	0	0	0	0	0
PARVG	0.151	0	0	0	0	0	0	0	0	0	0	0	0	0	0	0
VN1R3	0.151	0	0	0	0	0	0	0	0	0	0	0	0	0	0	0
PKD2L2	0	0	0	0	0	0	0	0	0	0	0	0	0	0	0	0.15
SLC16A1	0	0	0	0	0	0	0	0	0	0	0	0	0	0	0	0.15
GLUD1P2	0	0	0	0	0	0	0	0	0	0	0	0	0	0	0.15	0
ODF3B	0	0	0	0	0	0	0	0	0	0	0	0	0	0	0.15	0
DYNC1LI2	0	0	0	0	0	0	0	0	0	0	0.15	0	0	0	0	0
OPN3	0	0	0	0	0	0	0	0	0	0.15	0	0	0	0	0	0
NDUFAF1	0	0	0	0	0	0	0.15	0	0	0	0	0	0	0	0	0
PCCB	0	0	0	0	0	0	0.15	0	0	0	0	0	0	0	0	0
ST7-OT4	0	0	0	0	0	0.15	0	0	0	0	0	0	0	0	0	0
MEPCE	0	0	0	0	0.15	0	0	0	0	0	0	0	0	0	0	0
TTTY5	0	0	0	0.15	0	0	0	0	0	0	0	0	0	0	0	0
TXLNB	0	0	0	0.15	0	0	0	0	0	0	0	0	0	0	0	0
ADAM8	0	0.15	0	0	0	0	0	0	0	0	0	0	0	0	0	0
CASP9	0	0.15	0	0	0	0	0	0	0	0	0	0	0	0	0	0
CRLF1	0	0.15	0	0	0	0	0	0	0	0	0	0	0	0	0	0
ZNF432	0.15	0	0	0	0	0	0	0	0	0	0	0	0	0	0	0
CD52	0	0	0	0	0	0	0	0	0	0	0	0	0	0	0	0.149
CYP2D8P1	0	0	0	0	0	0	0	0	0	0	0	0	0	0	0	0.149
TAF5L	0	0	0	0	0	0	0	0	0	0	0	0	0	0	0.149	0
LHFPL2	0	0	0	0	0	0	0	0	0	0	0	0.149	0	0	0	0
OTOP2	0	0	0	0	0	0	0	0	0	0	0	0.149	0	0	0	0
BTAF1	0	0	0	0	0	0	0	0	0	0	0.149	0	0	0	0	0
CERKL	0	0	0	0	0	0	0	0	0	0.149	0	0	0	0	0	0
ACADM	0	0	0	0	0	0	0.149	0	0	0	0	0	0	0	0	0
ST7-OT3	0	0	0	0	0	0.149	0	0	0	0	0	0	0	0	0	0
LRRN1	0	0	0	0.149	0	0	0	0	0	0	0	0	0	0	0	0
AIS1	0	0.149	0	0	0	0	0	0	0	0	0	0	0	0	0	0
AOX1	0	0.149	0	0	0	0	0	0	0	0	0	0	0	0	0	0
BTNL2	0	0.149	0	0	0	0	0	0	0	0	0	0	0	0	0	0
C5AR1	0	0.149	0	0	0	0	0	0	0	0	0	0	0	0	0	0
CDH6	0	0.149	0	0	0	0	0	0	0	0	0	0	0	0	0	0

DDIT4	0	0.149	0	0	0	0	0	0	0	0	0	0	0	0	0	0
CACYBP	0.149	0	0	0	0	0	0	0	0	0	0	0	0	0	0	0
CTF2P	0.149	0	0	0	0	0	0	0	0	0	0	0	0	0	0	0
DIAPH1	0.149	0	0	0	0	0	0	0	0	0	0	0	0	0	0	0
OR7D4	0.149	0	0	0	0	0	0	0	0	0	0	0	0	0	0	0
PAR4	0.149	0	0	0	0	0	0	0	0	0	0	0	0	0	0	0
TAPVR1	0.149	0	0	0	0	0	0	0	0	0	0	0	0	0	0	0
CD55	0	0	0	0	0	0	0	0	0	0	0	0	0	0	0	0.148
GAGE7	0	0	0	0	0	0	0	0	0	0	0	0	0	0	0.148	0
TRPV1	0	0	0	0	0	0	0	0	0	0	0	0.148	0	0	0	0
MAP3K13	0	0	0	0	0	0	0	0	0	0	0.148	0	0	0	0	0
TACR3	0	0	0	0	0	0	0	0	0	0	0.148	0	0	0	0	0
TRNAK1	0	0	0	0	0	0	0	0	0	0	0.148	0	0	0	0	0
CRSA	0	0	0	0	0	0	0	0	0	0.148	0	0	0	0	0	0
GLB1L3	0	0	0	0	0	0	0	0	0	0.148	0	0	0	0	0	0
SLC25A13	0	0	0	0	0	0	0.148	0	0	0	0	0	0	0	0	0
KCNIP2	0	0	0	0	0	0.148	0	0	0	0	0	0	0	0	0	0
CCL3L3	0	0	0	0	0.148	0	0	0	0	0	0	0	0	0	0	0
ADAD1	0	0.148	0	0	0	0	0	0	0	0	0	0	0	0	0	0
ADCYAP1R1	0	0.148	0	0	0	0	0	0	0	0	0	0	0	0	0	0
AIS3	0	0.148	0	0	0	0	0	0	0	0	0	0	0	0	0	0
C6ORF27	0	0.148	0	0	0	0	0	0	0	0	0	0	0	0	0	0
LIPC	0.148	0	0	0	0	0	0	0	0	0	0	0	0	0	0	0
ZCWPW2	0.148	0	0	0	0	0	0	0	0	0	0	0	0	0	0	0
CLIC6	0	0	0	0	0	0	0	0	0	0	0	0	0	0	0	0.147
F13B	0	0	0	0	0	0	0	0	0	0	0	0	0	0	0.147	0
GGTLC5P	0	0	0	0	0	0	0	0	0	0	0	0	0	0	0.147	0
MRPS25	0	0	0	0	0	0	0	0	0	0	0	0	0	0	0.147	0
CTAA1	0	0	0	0	0	0	0	0	0	0	0	0.147	0	0	0	0
PTPRQ	0	0	0	0	0	0	0	0	0	0	0	0.147	0	0	0	0
MED30	0	0	0	0	0	0	0	0	0	0	0.147	0	0	0	0	0
PSMB1	0	0	0	0	0	0	0	0	0	0	0.147	0	0	0	0	0
USP17	0	0	0	0	0	0	0	0	0	0	0.147	0	0	0	0	0
NYX	0	0	0	0	0	0	0	0	0	0.147	0	0	0	0	0	0
ABCD1P2	0	0	0	0	0	0	0.147	0	0	0	0	0	0	0	0	0
ABCD1P3	0	0	0	0	0	0	0.147	0	0	0	0	0	0	0	0	0
AP3D1	0	0	0	0	0	0.147	0	0	0	0	0	0	0	0	0	0
CCL5	0	0	0	0	0.147	0	0	0	0	0	0	0	0	0	0	0
MMP1	0	0	0	0	0.147	0	0	0	0	0	0	0	0	0	0	0
ADAM15	0	0.147	0	0	0	0	0	0	0	0	0	0	0	0	0	0
COIL	0	0.147	0	0	0	0	0	0	0	0	0	0	0	0	0	0

DLK2	0.147	0	0	0	0	0	0	0	0	0	0	0	0	0	0	0
ESRRAP2	0.147	0	0	0	0	0	0	0	0	0	0	0	0	0	0	0
NR1D1	0.147	0	0	0	0	0	0	0	0	0	0	0	0	0	0	0
NR1H5P	0.147	0	0	0	0	0	0	0	0	0	0	0	0	0	0	0
NUFIP1	0.147	0	0	0	0	0	0	0	0	0	0	0	0	0	0	0
S100A6	0.147	0	0	0	0	0	0	0	0	0	0	0	0	0	0	0
TMEM198	0.147	0	0	0	0	0	0	0	0	0	0	0	0	0	0	0
ALDH7A1P1	0	0	0	0	0	0	0	0	0	0	0	0.146	0	0	0	0
SEMA4G	0	0	0	0	0	0	0	0	0	0	0	0.146	0	0	0	0
BRF1	0	0	0	0	0	0	0	0	0	0	0.146	0	0	0	0	0
ARCC1	0	0	0	0	0	0	0	0	0	0.146	0	0	0	0	0	0
NCAM1	0	0	0	0	0	0	0	0	0	0.146	0	0	0	0	0	0
DGUOK	0	0	0	0	0	0	0.146	0	0	0	0	0	0	0	0	0
NDUFA11	0	0	0	0	0	0	0.146	0	0	0	0	0	0	0	0	0
TBC1D7	0	0	0	0	0	0.146	0	0	0	0	0	0	0	0	0	0
USP6	0	0	0	0.146	0	0	0	0	0	0	0	0	0	0	0	0
ZNF189	0	0	0	0.146	0	0	0	0	0	0	0	0	0	0	0	0
ALCAM	0	0.146	0	0	0	0	0	0	0	0	0	0	0	0	0	0
ANKRD1	0	0.146	0	0	0	0	0	0	0	0	0	0	0	0	0	0
ANKRD46	0	0.146	0	0	0	0	0	0	0	0	0	0	0	0	0	0
BIRC2	0	0.146	0	0	0	0	0	0	0	0	0	0	0	0	0	0
BW33	0.146	0	0	0	0	0	0	0	0	0	0	0	0	0	0	0
CAPS	0.146	0	0	0	0	0	0	0	0	0	0	0	0	0	0	0
ISX	0.146	0	0	0	0	0	0	0	0	0	0	0	0	0	0	0
SUFU	0.146	0	0	0	0	0	0	0	0	0	0	0	0	0	0	0
CACNA1S	0	0	0	0	0	0	0	0	0	0	0	0	0	0	0	0.145
CATSPER4	0	0	0	0	0	0	0	0	0	0	0	0	0	0	0	0.145
MITF	0	0	0	0	0	0	0	0	0	0	0	0	0	0	0.145	0
TRBV5-4	0	0	0	0	0	0	0	0	0	0	0	0	0	0	0.145	0
MRPL24	0	0	0	0	0	0	0.145	0	0	0	0	0	0	0	0	0
PDP1	0	0	0	0	0	0	0.145	0	0	0	0	0	0	0	0	0
CNTN6	0	0	0	0	0	0.145	0	0	0	0	0	0	0	0	0	0
FGF12	0	0	0	0	0	0.145	0	0	0	0	0	0	0	0	0	0
KCNK7	0	0	0	0	0	0.145	0	0	0	0	0	0	0	0	0	0
SLC8A1	0	0	0	0	0	0.145	0	0	0	0	0	0	0	0	0	0
CYP2G1P	0	0	0	0	0.145	0	0	0	0	0	0	0	0	0	0	0
MTMR3	0	0	0	0.145	0	0	0	0	0	0	0	0	0	0	0	0
CIITA	0	0.145	0	0	0	0	0	0	0	0	0	0	0	0	0	0
CX3CL1	0	0.145	0	0	0	0	0	0	0	0	0	0	0	0	0	0
DIRC1	0	0.145	0	0	0	0	0	0	0	0	0	0	0	0	0	0
ANXA8L2	0.145	0	0	0	0	0	0	0	0	0	0	0	0	0	0	0

COPD24	0.145	0	0	0	0	0	0	0	0	0	0	0	0	0	0	0
CUBN	0.145	0	0	0	0	0	0	0	0	0	0	0	0	0	0	0
KC6	0.145	0	0	0	0	0	0	0	0	0	0	0	0	0	0	0
SAA4	0.145	0	0	0	0	0	0	0	0	0	0	0	0	0	0	0
IGFL1	0	0	0	0	0	0	0	0	0	0	0	0	0	0	0.144	0
NSDHL	0	0	0	0	0	0	0	0	0	0	0	0	0.144	0	0	0
AP2A1	0	0	0	0	0	0	0	0	0	0	0.144	0	0	0	0	0
SIN3B	0	0	0	0	0	0	0	0	0	0	0.144	0	0	0	0	0
ZNF644	0	0	0	0	0	0	0	0	0	0	0.144	0	0	0	0	0
ABCD1P4	0	0	0	0	0	0	0.144	0	0	0	0	0	0	0	0	0
ADAM17	0	0.144	0	0	0	0	0	0	0	0	0	0	0	0	0	0
ADAM21	0	0.144	0	0	0	0	0	0	0	0	0	0	0	0	0	0
AITD2	0	0.144	0	0	0	0	0	0	0	0	0	0	0	0	0	0
SMNDC1	0	0.144	0	0	0	0	0	0	0	0	0	0	0	0	0	0
C14ORF166	0.144	0	0	0	0	0	0	0	0	0	0	0	0	0	0	0
FRA5G	0.144	0	0	0	0	0	0	0	0	0	0	0	0	0	0	0
MORF4L2P1	0.144	0	0	0	0	0	0	0	0	0	0	0	0	0	0	0
S100A10	0.144	0	0	0	0	0	0	0	0	0	0	0	0	0	0	0
TSPAN6	0.144	0	0	0	0	0	0	0	0	0	0	0	0	0	0	0
CYP2D7P1	0	0	0	0	0	0	0	0	0	0	0	0	0	0	0	0.143
ASS1	0	0	0	0	0	0	0	0	0	0	0	0	0	0	0.143	0
TRPM8	0	0	0	0	0	0	0	0	0	0	0	0.143	0	0	0	0
EPS15	0	0	0	0	0	0	0	0	0	0	0.143	0	0	0	0	0
GPR75	0	0	0	0	0	0	0	0	0	0.143	0	0	0	0	0	0
SNRNP35	0	0.143	0	0	0	0	0	0	0	0	0	0	0	0	0	0
SNRPB	0	0.143	0	0	0	0	0	0	0	0	0	0	0	0	0	0
AKT2	0.143	0	0	0	0	0	0	0	0	0	0	0	0	0	0	0
APOC2	0.143	0	0	0	0	0	0	0	0	0	0	0	0	0	0	0
FAM50B	0.143	0	0	0	0	0	0	0	0	0	0	0	0	0	0	0
PUSL1	0.143	0	0	0	0	0	0	0	0	0	0	0	0	0	0	0
CASQ2	0	0	0	0	0	0	0	0	0	0	0	0	0	0	0	0.142
CATSPERG	0	0	0	0	0	0	0	0	0	0	0	0	0	0	0	0.142
SLC2A3P1	0	0	0	0	0	0	0	0	0	0	0	0	0	0	0	0.142
TMEM146	0	0	0	0	0	0	0	0	0	0	0	0	0	0	0	0.142
ANO6	0	0	0	0	0	0	0	0	0	0	0	0.142	0	0	0	0
CEACAM16	0	0	0	0	0	0	0	0	0	0	0	0.142	0	0	0	0
TMEM127	0	0	0	0	0	0	0	0	0	0	0	0.142	0	0	0	0
CASP1	0	0	0	0	0	0	0	0	0	0	0.142	0	0	0	0	0
SH3GLB1	0	0	0	0	0	0	0	0	0	0	0.142	0	0	0	0	0
PPEF1	0	0	0	0	0	0	0	0	0	0.142	0	0	0	0	0	0
SATB2	0	0	0	0	0	0.142	0	0	0	0	0	0	0	0	0	0

HDGFRP2	0	0	0	0	0.142	0	0	0	0	0	0	0	0	0	0	0
SH3D21	0	0.142	0	0	0	0	0	0	0	0	0	0	0	0	0	0
SHROOM3	0	0.142	0	0	0	0	0	0	0	0	0	0	0	0	0	0
SLIT1	0	0.142	0	0	0	0	0	0	0	0	0	0	0	0	0	0
SLIT3	0	0.142	0	0	0	0	0	0	0	0	0	0	0	0	0	0
SNRPB2	0	0.142	0	0	0	0	0	0	0	0	0	0	0	0	0	0
APOC3	0.142	0	0	0	0	0	0	0	0	0	0	0	0	0	0	0
DCDC5	0.142	0	0	0	0	0	0	0	0	0	0	0	0	0	0	0
DYRK3	0.142	0	0	0	0	0	0	0	0	0	0	0	0	0	0	0
FAM82B	0.142	0	0	0	0	0	0	0	0	0	0	0	0	0	0	0
LOC100289473	0.142	0	0	0	0	0	0	0	0	0	0	0	0	0	0	0
PLTP	0.142	0	0	0	0	0	0	0	0	0	0	0	0	0	0	0
PRKXP1	0.142	0	0	0	0	0	0	0	0	0	0	0	0	0	0	0
THAP5	0.142	0	0	0	0	0	0	0	0	0	0	0	0	0	0	0
TBC1D4	0	0	0	0	0	0	0	0	0	0	0	0	0	0	0	0.141
LTBP4	0	0	0	0	0	0	0	0	0	0	0	0	0	0	0.141	0
PUS1	0	0	0	0	0	0	0	0	0	0	0	0.141	0	0	0	0
SEC23B	0	0	0	0	0	0	0	0	0	0	0	0.141	0	0	0	0
STOML3	0	0	0	0	0	0	0	0	0	0	0	0.141	0	0	0	0
NFYA	0	0	0	0	0	0	0	0	0	0	0.141	0	0	0	0	0
ARL6	0	0	0	0	0	0	0	0	0	0.141	0	0	0	0	0	0
ARR3	0	0	0	0	0	0	0	0	0	0.141	0	0	0	0	0	0
CABP5	0	0	0	0	0	0	0	0	0	0.141	0	0	0	0	0	0
COL9A1	0	0	0	0	0	0	0	0	0	0.141	0	0	0	0	0	0
GRAMD1A	0	0	0	0	0	0	0	0	0	0.141	0	0	0	0	0	0
CPT2	0	0	0	0	0	0	0.141	0	0	0	0	0	0	0	0	0
MRPS28	0	0	0	0	0	0	0.141	0	0	0	0	0	0	0	0	0
PDP2	0	0	0	0	0	0	0.141	0	0	0	0	0	0	0	0	0
LHX6	0	0	0	0	0	0.141	0	0	0	0	0	0	0	0	0	0
ESR1	0	0	0	0	0.141	0	0	0	0	0	0	0	0	0	0	0
PDCD7	0	0	0	0	0.141	0	0	0	0	0	0	0	0	0	0	0
SOAT1	0	0	0	0	0.141	0	0	0	0	0	0	0	0	0	0	0
SKOR2	0	0.141	0	0	0	0	0	0	0	0	0	0	0	0	0	0
SLC38A5	0	0.141	0	0	0	0	0	0	0	0	0	0	0	0	0	0
SNRPC	0	0.141	0	0	0	0	0	0	0	0	0	0	0	0	0	0
SNRPD1	0	0.141	0	0	0	0	0	0	0	0	0	0	0	0	0	0
SOX14	0	0.141	0	0	0	0	0	0	0	0	0	0	0	0	0	0
AASTH42	0.141	0	0	0	0	0	0	0	0	0	0	0	0	0	0	0
CCND3	0.141	0	0	0	0	0	0	0	0	0	0	0	0	0	0	0
FRA18C	0.141	0	0	0	0	0	0	0	0	0	0	0	0	0	0	0
HMGN2P3	0.141	0	0	0	0	0	0	0	0	0	0	0	0	0	0	0

NUBP2	0.141	0	0	0	0	0	0	0	0	0	0	0	0	0	0	0
OFC4	0.141	0	0	0	0	0	0	0	0	0	0	0	0	0	0	0
PRUNE	0.141	0	0	0	0	0	0	0	0	0	0	0	0	0	0	0
RAC1P5	0.141	0	0	0	0	0	0	0	0	0	0	0	0	0	0	0
S100A4	0.141	0	0	0	0	0	0	0	0	0	0	0	0	0	0	0
ENG	0	0	0	0	0	0	0	0	0	0	0	0	0	0	0	0.14
ACTG1P1	0	0	0	0	0	0	0	0	0	0	0	0	0	0	0.14	0
DOCK8	0	0	0	0	0	0	0	0	0	0	0	0	0.14	0	0	0
TRPA1	0	0	0	0	0	0	0	0	0	0	0	0.14	0	0	0	0
FBXL5	0	0	0	0	0	0	0	0	0	0	0.14	0	0	0	0	0
TMEM107	0	0	0	0	0	0	0	0	0	0.14	0	0	0	0	0	0
RBM14-RBM4	0	0	0	0	0	0	0	0	0.14	0	0	0	0	0	0	0
ETFB	0	0	0	0	0	0	0.14	0	0	0	0	0	0	0	0	0
NDUFB2	0	0	0	0	0	0	0.14	0	0	0	0	0	0	0	0	0
UGT1A1	0	0	0	0	0	0	0.14	0	0	0	0	0	0	0	0	0
HAX1	0	0	0	0	0	0.14	0	0	0	0	0	0	0	0	0	0
KCNJ5	0	0	0	0	0	0.14	0	0	0	0	0	0	0	0	0	0
KCNK1	0	0	0	0	0	0.14	0	0	0	0	0	0	0	0	0	0
ABR	0	0	0.14	0	0	0	0	0	0	0	0	0	0	0	0	0
SNRPG	0	0.14	0	0	0	0	0	0	0	0	0	0	0	0	0	0
SOX3	0	0.14	0	0	0	0	0	0	0	0	0	0	0	0	0	0
STMN1	0	0.14	0	0	0	0	0	0	0	0	0	0	0	0	0	0
ECEL1P2	0.14	0	0	0	0	0	0	0	0	0	0	0	0	0	0	0
TRAFD1	0.14	0	0	0	0	0	0	0	0	0	0	0	0	0	0	0
RSPH10B	0	0	0	0	0	0	0	0	0	0	0	0	0	0	0	0.139
RPL27A	0	0	0	0	0	0	0	0	0	0	0	0	0	0	0.139	0
SMPD2	0	0	0	0	0	0	0	0	0	0	0	0	0	0.139	0	0
SPHK1	0	0	0	0	0	0	0	0	0	0	0	0	0	0.139	0	0
SPNS2	0	0	0	0	0	0	0	0	0	0	0	0	0	0.139	0	0
GLA	0	0	0	0	0	0	0	0	0	0	0	0	0.139	0	0	0
CFL1	0	0	0	0	0	0	0	0	0	0	0.139	0	0	0	0	0
NPM1P2	0	0	0	0	0	0	0	0	0	0	0.139	0	0	0	0	0
TACR2	0	0	0	0	0	0	0	0	0	0	0.139	0	0	0	0	0
NPLOC4	0	0	0	0	0	0	0	0	0.139	0	0	0	0	0	0	0
CSTP1	0	0	0	0	0	0	0	0.139	0	0	0	0	0	0	0	0
ATAD3A	0	0	0	0	0	0	0.139	0	0	0	0	0	0	0	0	0
DCDC1	0	0	0	0	0	0.139	0	0	0	0	0	0	0	0	0	0
EIF5AP2	0	0	0	0	0.139	0	0	0	0	0	0	0	0	0	0	0
GJA4	0	0	0	0.139	0	0	0	0	0	0	0	0	0	0	0	0
PTCPRN	0	0.139	0	0	0	0	0	0	0	0	0	0	0	0	0	0
RALGAPB	0	0.139	0	0	0	0	0	0	0	0	0	0	0	0	0	0

RAP1A	0	0.139	0	0	0	0	0	0	0	0	0	0	0	0	0	0
SCG3	0	0.139	0	0	0	0	0	0	0	0	0	0	0	0	0	0
C1R	0.139	0	0	0	0	0	0	0	0	0	0	0	0	0	0	0
GYG2	0.139	0	0	0	0	0	0	0	0	0	0	0	0	0	0	0
TRIB1	0.139	0	0	0	0	0	0	0	0	0	0	0	0	0	0	0
ZNF668	0.139	0	0	0	0	0	0	0	0	0	0	0	0	0	0	0
IGKV3OR22-2	0	0	0	0	0	0	0	0	0	0	0	0	0	0	0.138	0
QRSL1	0	0	0	0	0	0	0	0	0	0	0	0	0	0.138	0	0
SERP1	0	0	0	0	0	0	0	0	0	0	0	0	0	0.138	0	0
SLC11A2	0	0	0	0	0	0	0	0	0	0	0	0	0	0.138	0	0
VPS11	0	0	0	0	0	0	0	0	0	0	0	0	0	0.138	0	0
WIPI2	0	0	0	0	0	0	0	0	0	0	0	0	0	0.138	0	0
ANO3	0	0	0	0	0	0	0	0	0	0	0	0.138	0	0	0	0
CHM	0	0	0	0	0	0	0	0	0	0.138	0	0	0	0	0	0
RRH	0	0	0	0	0	0	0	0	0	0.138	0	0	0	0	0	0
EZR	0	0	0	0	0	0.138	0	0	0	0	0	0	0	0	0	0
KCNJ12	0	0	0	0	0	0.138	0	0	0	0	0	0	0	0	0	0
SLC6A15	0	0	0	0	0	0.138	0	0	0	0	0	0	0	0	0	0
PLEKHG5	0	0.138	0	0	0	0	0	0	0	0	0	0	0	0	0	0
PPP1R15A	0	0.138	0	0	0	0	0	0	0	0	0	0	0	0	0	0
PRG4	0	0.138	0	0	0	0	0	0	0	0	0	0	0	0	0	0
RPP38	0	0.138	0	0	0	0	0	0	0	0	0	0	0	0	0	0
SCAF4	0	0.138	0	0	0	0	0	0	0	0	0	0	0	0	0	0
ATP5B	0.138	0	0	0	0	0	0	0	0	0	0	0	0	0	0	0
BPIFA3	0.138	0	0	0	0	0	0	0	0	0	0	0	0	0	0	0
IRS3P	0.138	0	0	0	0	0	0	0	0	0	0	0	0	0	0	0
KRTAP12-2	0.138	0	0	0	0	0	0	0	0	0	0	0	0	0	0	0
LRRFIP2	0.138	0	0	0	0	0	0	0	0	0	0	0	0	0	0	0
PARVA	0.138	0	0	0	0	0	0	0	0	0	0	0	0	0	0	0
S100A5	0.138	0	0	0	0	0	0	0	0	0	0	0	0	0	0	0
S100A7P1	0.138	0	0	0	0	0	0	0	0	0	0	0	0	0	0	0
SIK3	0.138	0	0	0	0	0	0	0	0	0	0	0	0	0	0	0
CYP2D7BP	0	0	0	0	0	0	0	0	0	0	0	0	0	0	0	0.137
ASB11	0	0	0	0	0	0	0	0	0	0	0	0	0	0	0.137	0
RAB3IL1	0	0	0	0	0	0	0	0	0	0	0	0	0	0	0.137	0
TLCD2	0	0	0	0	0	0	0	0	0	0	0	0	0	0.137	0	0
TRAP1	0	0	0	0	0	0	0	0	0	0	0	0	0	0.137	0	0
UGCG	0	0	0	0	0	0	0	0	0	0	0	0	0	0.137	0	0
USP30	0	0	0	0	0	0	0	0	0	0	0	0	0	0.137	0	0
TRNM	0	0	0	0	0	0	0	0	0	0	0	0.137	0	0	0	0
RNF4	0	0	0	0	0	0	0	0	0	0	0.137	0	0	0	0	0

PDE6D	0	0	0	0	0	0	0	0	0	0.137	0	0	0	0	0	0
SRSF6	0	0	0	0	0	0	0	0	0.137	0	0	0	0	0	0	0
ZNF597	0	0	0	0	0	0	0	0.137	0	0	0	0	0	0	0	0
ABCD1P1	0	0	0	0	0	0	0.137	0	0	0	0	0	0	0	0	0
NUCB1	0	0	0	0	0.137	0	0	0	0	0	0	0	0	0	0	0
FBXW10	0	0	0	0.137	0	0	0	0	0	0	0	0	0	0	0	0
SUN1	0	0	0	0.137	0	0	0	0	0	0	0	0	0	0	0	0
ZNF723	0	0	0.137	0	0	0	0	0	0	0	0	0	0	0	0	0
ZNF726	0	0	0.137	0	0	0	0	0	0	0	0	0	0	0	0	0
ZNF878	0	0	0.137	0	0	0	0	0	0	0	0	0	0	0	0	0
RGMA	0	0.137	0	0	0	0	0	0	0	0	0	0	0	0	0	0
RHEBL1	0	0.137	0	0	0	0	0	0	0	0	0	0	0	0	0	0
ROBO3	0	0.137	0	0	0	0	0	0	0	0	0	0	0	0	0	0
RPL13AP17	0	0.137	0	0	0	0	0	0	0	0	0	0	0	0	0	0
FRA1B	0.137	0	0	0	0	0	0	0	0	0	0	0	0	0	0	0
CASKIN2	0	0	0	0	0	0	0	0	0	0	0	0	0	0	0	0.136
CUL9	0	0	0	0	0	0	0	0	0	0	0	0	0	0	0	0.136
SLC25A39	0	0	0	0	0	0	0	0	0	0	0	0	0	0	0	0.136
HYD2	0	0	0	0	0	0	0	0	0	0	0	0.136	0	0	0	0
PRSS56	0	0	0	0	0	0	0	0	0	0	0	0.136	0	0	0	0
SDHB	0	0	0	0	0	0	0	0	0	0	0	0.136	0	0	0	0
TRPV2	0	0	0	0	0	0	0	0	0	0	0	0.136	0	0	0	0
PDXP	0	0	0	0	0	0	0	0	0	0	0.136	0	0	0	0	0
TAC4	0	0	0	0	0	0	0	0	0	0	0.136	0	0	0	0	0
COQ9	0	0	0	0	0	0	0.136	0	0	0	0	0	0	0	0	0
NDUFA8	0	0	0	0	0	0	0.136	0	0	0	0	0	0	0	0	0
SLC25A20	0	0	0	0	0	0	0.136	0	0	0	0	0	0	0	0	0
SLC25A29	0	0	0	0	0	0	0.136	0	0	0	0	0	0	0	0	0
DLX6	0	0	0	0	0	0.136	0	0	0	0	0	0	0	0	0	0
ERC2	0	0	0	0	0	0.136	0	0	0	0	0	0	0	0	0	0
FLNA	0	0	0	0	0	0.136	0	0	0	0	0	0	0	0	0	0
KCNJ8	0	0	0	0	0	0.136	0	0	0	0	0	0	0	0	0	0
KCNS2	0	0	0	0	0	0.136	0	0	0	0	0	0	0	0	0	0
SAR1B	0	0	0	0.136	0	0	0	0	0	0	0	0	0	0	0	0
RASL10A	0	0	0.136	0	0	0	0	0	0	0	0	0	0	0	0	0
ZNF280D	0	0	0.136	0	0	0	0	0	0	0	0	0	0	0	0	0
XIAP	0	0.136	0	0	0	0	0	0	0	0	0	0	0	0	0	0
ASF1A	0.136	0	0	0	0	0	0	0	0	0	0	0	0	0	0	0
ATP5EP1	0.136	0	0	0	0	0	0	0	0	0	0	0	0	0	0	0
CTNNBIP1	0.136	0	0	0	0	0	0	0	0	0	0	0	0	0	0	0
LPAL2	0.136	0	0	0	0	0	0	0	0	0	0	0	0	0	0	0

NIN	0.136	0	0	0	0	0	0	0	0	0	0	0	0	0	0	0
RPSAP12	0.136	0	0	0	0	0	0	0	0	0	0	0	0	0	0	0
CABP1	0	0	0	0	0	0	0	0	0	0	0	0	0	0	0	0.135
CYP1A1	0	0	0	0	0	0	0	0	0	0	0	0	0	0	0	0.135
EYA1	0	0	0	0	0	0	0	0	0	0	0	0.135	0	0	0	0
OMP	0	0	0	0	0	0	0	0	0	0	0	0.135	0	0	0	0
CARD17	0	0	0	0	0	0	0	0	0	0	0.135	0	0	0	0	0
CASP10	0	0	0	0	0	0	0	0	0	0	0.135	0	0	0	0	0
CASP5	0	0	0	0	0	0	0	0	0	0	0.135	0	0	0	0	0
CASP7	0	0	0	0	0	0	0	0	0	0	0.135	0	0	0	0	0
RD3	0	0	0	0	0	0	0	0	0	0.135	0	0	0	0	0	0
TIMM22	0	0	0	0	0	0	0.135	0	0	0	0	0	0	0	0	0
UQCRFS1	0	0	0	0	0	0	0.135	0	0	0	0	0	0	0	0	0
HIGD1C	0	0	0.135	0	0	0	0	0	0	0	0	0	0	0	0	0
HRH1	0	0	0.135	0	0	0	0	0	0	0	0	0	0	0	0	0
HSR	0	0	0.135	0	0	0	0	0	0	0	0	0	0	0	0	0
KIAA0319L	0	0	0.135	0	0	0	0	0	0	0	0	0	0	0	0	0
SCZD11	0	0	0.135	0	0	0	0	0	0	0	0	0	0	0	0	0
TIMP2	0	0.135	0	0	0	0	0	0	0	0	0	0	0	0	0	0
TIMP3	0	0.135	0	0	0	0	0	0	0	0	0	0	0	0	0	0
TJP1	0	0.135	0	0	0	0	0	0	0	0	0	0	0	0	0	0
TNFRSF21	0	0.135	0	0	0	0	0	0	0	0	0	0	0	0	0	0
YWHAQ	0	0.135	0	0	0	0	0	0	0	0	0	0	0	0	0	0
GPIHBP1	0.135	0	0	0	0	0	0	0	0	0	0	0	0	0	0	0
MYLIP	0.135	0	0	0	0	0	0	0	0	0	0	0	0	0	0	0
NUFIP1P	0.135	0	0	0	0	0	0	0	0	0	0	0	0	0	0	0
SHMT1	0.135	0	0	0	0	0	0	0	0	0	0	0	0	0	0	0
WRB	0.135	0	0	0	0	0	0	0	0	0	0	0	0	0	0	0
POLD2P1	0	0	0	0	0	0	0	0	0	0	0	0	0	0	0	0.134
BARHL1	0	0	0	0	0	0	0	0	0	0	0	0.134	0	0	0	0
HMX3	0	0	0	0	0	0	0	0	0	0	0	0.134	0	0	0	0
ADD3	0	0	0	0	0	0	0	0	0	0	0.134	0	0	0	0	0
CNGB1	0	0	0	0	0	0	0	0	0	0.134	0	0	0	0	0	0
TUB	0	0	0	0	0	0	0	0	0	0.134	0	0	0	0	0	0
SLC37A1	0	0	0	0	0	0	0.134	0	0	0	0	0	0	0	0	0
DENND1A	0	0	0.134	0	0	0	0	0	0	0	0	0	0	0	0	0
HIGD1AP1	0	0	0.134	0	0	0	0	0	0	0	0	0	0	0	0	0
KIAA0564	0	0	0.134	0	0	0	0	0	0	0	0	0	0	0	0	0
MACROD2	0	0	0.134	0	0	0	0	0	0	0	0	0	0	0	0	0
NTSR1	0	0	0.134	0	0	0	0	0	0	0	0	0	0	0	0	0
NXF3	0	0	0.134	0	0	0	0	0	0	0	0	0	0	0	0	0

PCDHA1	0	0	0.134	0	0	0	0	0	0	0	0	0	0	0	0	0
PPP1R9A	0	0	0.134	0	0	0	0	0	0	0	0	0	0	0	0	0
TBX4	0	0.134	0	0	0	0	0	0	0	0	0	0	0	0	0	0
TIMP1	0	0.134	0	0	0	0	0	0	0	0	0	0	0	0	0	0
TNFSF12-TNFSF13	0	0.134	0	0	0	0	0	0	0	0	0	0	0	0	0	0
TRBV20-1	0	0.134	0	0	0	0	0	0	0	0	0	0	0	0	0	0
TRPM2	0	0.134	0	0	0	0	0	0	0	0	0	0	0	0	0	0
UNCX	0	0.134	0	0	0	0	0	0	0	0	0	0	0	0	0	0
UOX	0	0.134	0	0	0	0	0	0	0	0	0	0	0	0	0	0
UQCC	0	0.134	0	0	0	0	0	0	0	0	0	0	0	0	0	0
FRAT2	0.134	0	0	0	0	0	0	0	0	0	0	0	0	0	0	0
LOC100287306	0.134	0	0	0	0	0	0	0	0	0	0	0	0	0	0	0
PTCD1	0	0	0	0	0	0	0	0	0	0	0	0	0	0.133	0	0
GFRA2	0	0	0	0	0	0	0	0	0	0	0	0.133	0	0	0	0
EIF2S1	0	0	0	0	0	0	0	0	0	0	0.133	0	0	0	0	0
MEOX2	0	0	0	0	0	0	0	0	0	0	0.133	0	0	0	0	0
AQP1	0	0	0	0	0	0	0.133	0	0	0	0	0	0	0	0	0
GFM1	0	0	0	0	0	0	0.133	0	0	0	0	0	0	0	0	0
GLUD1P5	0	0	0	0	0	0	0.133	0	0	0	0	0	0	0	0	0
PEX13	0	0	0	0	0	0	0.133	0	0	0	0	0	0	0	0	0
KCNK17	0	0	0	0	0	0.133	0	0	0	0	0	0	0	0	0	0
NPY5R	0	0	0	0	0	0.133	0	0	0	0	0	0	0	0	0	0
C9ORF156	0	0	0	0	0.133	0	0	0	0	0	0	0	0	0	0	0
MIR586	0	0	0.133	0	0	0	0	0	0	0	0	0	0	0	0	0
EFNB2	0	0.133	0	0	0	0	0	0	0	0	0	0	0	0	0	0
PITX1	0	0.133	0	0	0	0	0	0	0	0	0	0	0	0	0	0
TRIM15	0	0.133	0	0	0	0	0	0	0	0	0	0	0	0	0	0
IFNR	0.133	0	0	0	0	0	0	0	0	0	0	0	0	0	0	0
MAPK1IP1L	0.133	0	0	0	0	0	0	0	0	0	0	0	0	0	0	0
PPP3R1	0.133	0	0	0	0	0	0	0	0	0	0	0	0	0	0	0
SCARB1	0.133	0	0	0	0	0	0	0	0	0	0	0	0	0	0	0
ATP11B	0	0	0	0	0	0	0	0	0	0	0	0	0	0	0	0.132
ASS1P3	0	0	0	0	0	0	0	0	0	0	0	0	0	0	0.132	0
SRM	0	0	0	0	0	0	0	0	0	0	0	0	0	0	0.132	0
AEN	0	0	0	0	0	0	0	0	0	0	0	0	0	0.132	0	0
AGBL5	0	0	0	0	0	0	0	0	0	0	0	0	0	0.132	0	0
AIFM3	0	0	0	0	0	0	0	0	0	0	0	0	0	0.132	0	0
ATM	0	0	0	0	0	0	0	0	0	0	0	0	0	0.132	0	0
ATRN	0	0	0	0	0	0	0	0	0	0	0	0	0	0.132	0	0
BECN1P1	0	0	0	0	0	0	0	0	0	0	0	0	0	0.132	0	0

BNIP3L	0	0	0	0	0	0	0	0	0	0	0	0	0	0.132	0	0
PRDX3	0	0	0	0	0	0	0	0	0	0	0	0	0	0.132	0	0
IDS	0	0	0	0	0	0	0	0	0	0	0	0	0.132	0	0	0
DPF1	0	0	0	0	0	0	0	0	0	0	0	0.132	0	0	0	0
MYO1H	0	0	0	0	0	0	0	0	0	0	0	0.132	0	0	0	0
MYP2	0	0	0	0	0	0	0	0	0	0	0	0.132	0	0	0	0
OTOA	0	0	0	0	0	0	0	0	0	0	0	0.132	0	0	0	0
GTF2B	0	0	0	0	0	0	0	0	0	0	0.132	0	0	0	0	0
HIST3H3	0	0	0	0	0	0	0	0	0	0	0.132	0	0	0	0	0
MAP3K9	0	0	0	0	0	0	0	0	0	0	0.132	0	0	0	0	0
OGG1	0	0	0	0	0	0	0	0	0	0	0.132	0	0	0	0	0
ZBED4	0	0	0	0	0	0	0	0	0	0.132	0	0	0	0	0	0
KHSRP	0	0	0	0	0	0	0	0	0.132	0	0	0	0	0	0	0
FASTKD2	0	0	0	0	0	0	0.132	0	0	0	0	0	0	0	0	0
SEC11A	0	0	0	0	0	0.132	0	0	0	0	0	0	0	0	0	0
LINC00272	0	0	0	0.132	0	0	0	0	0	0	0	0	0	0	0	0
SOX11	0	0	0	0.132	0	0	0	0	0	0	0	0	0	0	0	0
GTF2H2	0	0.132	0	0	0	0	0	0	0	0	0	0	0	0	0	0
HCG4	0	0.132	0	0	0	0	0	0	0	0	0	0	0	0	0	0
HDAC11	0	0.132	0	0	0	0	0	0	0	0	0	0	0	0	0	0
HNRNPH3	0	0.132	0	0	0	0	0	0	0	0	0	0	0	0	0	0
HOXC10	0	0.132	0	0	0	0	0	0	0	0	0	0	0	0	0	0
IFI30	0	0.132	0	0	0	0	0	0	0	0	0	0	0	0	0	0
IFNAR1	0	0.132	0	0	0	0	0	0	0	0	0	0	0	0	0	0
PCDH8	0	0.132	0	0	0	0	0	0	0	0	0	0	0	0	0	0
C12ORF33	0.132	0	0	0	0	0	0	0	0	0	0	0	0	0	0	0
CYP2T3P	0.132	0	0	0	0	0	0	0	0	0	0	0	0	0	0	0
FAM55B	0.132	0	0	0	0	0	0	0	0	0	0	0	0	0	0	0
FBXL3	0.132	0	0	0	0	0	0	0	0	0	0	0	0	0	0	0
FBXW7	0.132	0	0	0	0	0	0	0	0	0	0	0	0	0	0	0
FCHO1	0.132	0	0	0	0	0	0	0	0	0	0	0	0	0	0	0
FOLR4	0.132	0	0	0	0	0	0	0	0	0	0	0	0	0	0	0
GYS2	0.132	0	0	0	0	0	0	0	0	0	0	0	0	0	0	0
HSP90AB3P	0.132	0	0	0	0	0	0	0	0	0	0	0	0	0	0	0
NDUFB10	0.132	0	0	0	0	0	0	0	0	0	0	0	0	0	0	0
S100A7L2	0.132	0	0	0	0	0	0	0	0	0	0	0	0	0	0	0
TAPT1	0.132	0	0	0	0	0	0	0	0	0	0	0	0	0	0	0
TRIL	0.132	0	0	0	0	0	0	0	0	0	0	0	0	0	0	0
AASTH17	0	0	0	0	0	0	0	0	0	0	0	0	0	0	0	0.131
ZNF692	0	0	0	0	0	0	0	0	0	0	0	0	0	0	0	0.131
ACP2	0	0	0	0	0	0	0	0	0	0	0	0	0	0.131	0	0

AMIGO2	0	0	0	0	0	0	0	0	0	0	0	0	0	0.131	0	0
ASAH1	0	0	0	0	0	0	0	0	0	0	0	0	0	0.131	0	0
C11ORF31	0	0	0	0	0	0	0	0	0	0	0	0	0	0.131	0	0
C1QA	0	0	0	0	0	0	0	0	0	0	0	0	0	0.131	0	0
C9ORF114	0	0	0	0	0	0	0	0	0	0	0	0	0	0.131	0	0
CD68	0	0	0	0	0	0	0	0	0	0	0	0	0	0.131	0	0
CERS6	0	0	0	0	0	0	0	0	0	0	0	0	0	0.131	0	0
CTSA	0	0	0	0	0	0	0	0	0	0	0	0	0	0.131	0	0
CATSPER2P1	0	0	0	0	0	0	0	0	0	0	0	0.131	0	0	0	0
PIRT	0	0	0	0	0	0	0	0	0	0	0	0.131	0	0	0	0
HDAC7	0	0	0	0	0	0	0	0	0	0	0.131	0	0	0	0	0
NLRP1	0	0	0	0	0	0	0	0	0	0	0.131	0	0	0	0	0
C1QTNF5	0	0	0	0	0	0	0	0	0	0.131	0	0	0	0	0	0
HMX1	0	0	0	0	0	0	0	0	0	0.131	0	0	0	0	0	0
ICAM1	0	0	0	0	0.131	0	0	0	0	0	0	0	0	0	0	0
SBF1	0	0	0	0.131	0	0	0	0	0	0	0	0	0	0	0	0
ELAVL1	0	0.131	0	0	0	0	0	0	0	0	0	0	0	0	0	0
FBXO32	0	0.131	0	0	0	0	0	0	0	0	0	0	0	0	0	0
FLI1	0	0.131	0	0	0	0	0	0	0	0	0	0	0	0	0	0
GSTA4	0	0.131	0	0	0	0	0	0	0	0	0	0	0	0	0	0
HEATR7B2	0	0.131	0	0	0	0	0	0	0	0	0	0	0	0	0	0
HFE	0	0.131	0	0	0	0	0	0	0	0	0	0	0	0	0	0
IFNAR2	0	0.131	0	0	0	0	0	0	0	0	0	0	0	0	0	0
IFNB1	0	0.131	0	0	0	0	0	0	0	0	0	0	0	0	0	0
IGFALS	0	0.131	0	0	0	0	0	0	0	0	0	0	0	0	0	0
IGFBP2	0	0.131	0	0	0	0	0	0	0	0	0	0	0	0	0	0
IL21	0	0.131	0	0	0	0	0	0	0	0	0	0	0	0	0	0
ISL2	0	0.131	0	0	0	0	0	0	0	0	0	0	0	0	0	0
MIR5095	0.131	0	0	0	0	0	0	0	0	0	0	0	0	0	0	0
PPP1R2P3	0.131	0	0	0	0	0	0	0	0	0	0	0	0	0	0	0
RPL3P1	0.131	0	0	0	0	0	0	0	0	0	0	0	0	0	0	0
TMEM132A	0.131	0	0	0	0	0	0	0	0	0	0	0	0	0	0	0
NEDE	0	0	0	0	0	0	0	0	0	0	0	0.13	0	0	0	0
UROD	0	0	0	0	0	0	0	0	0	0	0	0.13	0	0	0	0
CLTC	0	0	0	0	0	0	0	0	0	0	0.13	0	0	0	0	0
EPN2	0	0	0	0	0	0	0	0	0	0	0.13	0	0	0	0	0
PHGR1	0	0	0	0	0	0	0	0	0	0	0.13	0	0	0	0	0
TLL1	0	0	0	0	0	0	0	0	0	0	0.13	0	0	0	0	0
GRK1	0	0	0	0	0	0	0	0	0	0.13	0	0	0	0	0	0
MFRP	0	0	0	0	0	0	0	0	0	0.13	0	0	0	0	0	0
RP1L1	0	0	0	0	0	0	0	0	0	0.13	0	0	0	0	0	0

KCNA3	0	0	0	0	0	0.13	0	0	0	0	0	0	0	0	0	0
LRRC4B	0	0	0	0	0	0.13	0	0	0	0	0	0	0	0	0	0
EFNB3	0	0.13	0	0	0	0	0	0	0	0	0	0	0	0	0	0
EPO	0	0.13	0	0	0	0	0	0	0	0	0	0	0	0	0	0
ETS2	0	0.13	0	0	0	0	0	0	0	0	0	0	0	0	0	0
FAM167A	0	0.13	0	0	0	0	0	0	0	0	0	0	0	0	0	0
FUT4	0	0.13	0	0	0	0	0	0	0	0	0	0	0	0	0	0
GEMIN6	0	0.13	0	0	0	0	0	0	0	0	0	0	0	0	0	0
ATIC	0.13	0	0	0	0	0	0	0	0	0	0	0	0	0	0	0
HMGCLL1	0.13	0	0	0	0	0	0	0	0	0	0	0	0	0	0	0
ILK	0.13	0	0	0	0	0	0	0	0	0	0	0	0	0	0	0
ITGB1BP3	0.13	0	0	0	0	0	0	0	0	0	0	0	0	0	0	0
OFC3	0.13	0	0	0	0	0	0	0	0	0	0	0	0	0	0	0
PPP1R3G	0.13	0	0	0	0	0	0	0	0	0	0	0	0	0	0	0
RAC1P3	0.13	0	0	0	0	0	0	0	0	0	0	0	0	0	0	0
S100A7A	0.13	0	0	0	0	0	0	0	0	0	0	0	0	0	0	0
PGAP1	0	0	0	0	0	0	0	0	0	0	0	0	0	0	0	0.129
SLC16A5	0	0	0	0	0	0	0	0	0	0	0	0	0	0	0	0.129
PPWD1	0	0	0	0	0	0	0	0	0	0	0	0	0	0	0.129	0
CTSF	0	0	0	0	0	0	0	0	0	0	0	0	0	0.129	0	0
CYBB	0	0	0	0	0	0	0	0	0	0	0	0	0	0.129	0	0
LAPTM5	0	0	0	0	0	0	0	0	0	0	0	0	0	0.129	0	0
LRPPRC	0	0	0	0	0	0	0	0	0	0	0	0	0	0.129	0	0
MGRN1	0	0	0	0	0	0	0	0	0	0	0	0	0	0.129	0	0
MTRF1L	0	0	0	0	0	0	0	0	0	0	0	0	0	0.129	0	0
NDUFB8	0	0	0	0	0	0	0	0	0	0	0	0	0	0.129	0	0
NEU4	0	0	0	0	0	0	0	0	0	0	0	0	0	0.129	0	0
OMA1	0	0	0	0	0	0	0	0	0	0	0	0	0	0.129	0	0
PIK3C3	0	0	0	0	0	0	0	0	0	0	0	0	0	0.129	0	0
PLA2G3	0	0	0	0	0	0	0	0	0	0	0	0	0	0.129	0	0
PLG	0	0	0	0	0	0	0	0	0	0	0	0	0	0.129	0	0
DGKB	0	0	0	0	0	0	0	0	0	0	0.129	0	0	0	0	0
NEIL1	0	0	0	0	0	0	0	0	0	0	0.129	0	0	0	0	0
IMPG2	0	0	0	0	0	0	0	0	0	0.129	0	0	0	0	0	0
RGS7	0	0	0	0	0	0	0	0	0	0.129	0	0	0	0	0	0
TUG1	0	0	0	0	0	0	0	0	0	0.129	0	0	0	0	0	0
TRANK1	0	0	0	0	0	0	0.129	0	0	0	0	0	0	0	0	0
TREX1	0	0	0	0	0	0	0.129	0	0	0	0	0	0	0	0	0
ABCC8	0	0	0	0	0	0.129	0	0	0	0	0	0	0	0	0	0
ARL6IP6	0	0	0	0	0	0.129	0	0	0	0	0	0	0	0	0	0
RBFOX1	0	0	0	0	0	0.129	0	0	0	0	0	0	0	0	0	0

ELMOD3	0	0	0	0.129	0	0	0	0	0	0	0	0	0	0	0	0
SYNE2	0	0	0	0.129	0	0	0	0	0	0	0	0	0	0	0	0
GDI2P1	0	0.129	0	0	0	0	0	0	0	0	0	0	0	0	0	0
ISLR2	0	0.129	0	0	0	0	0	0	0	0	0	0	0	0	0	0
KIAA1598	0	0.129	0	0	0	0	0	0	0	0	0	0	0	0	0	0
MYO9B	0	0.129	0	0	0	0	0	0	0	0	0	0	0	0	0	0
NCALD	0	0.129	0	0	0	0	0	0	0	0	0	0	0	0	0	0
NKX6-2	0	0.129	0	0	0	0	0	0	0	0	0	0	0	0	0	0
NPHS1	0	0.129	0	0	0	0	0	0	0	0	0	0	0	0	0	0
NRCLP	0	0.129	0	0	0	0	0	0	0	0	0	0	0	0	0	0
ODZ1	0	0.129	0	0	0	0	0	0	0	0	0	0	0	0	0	0
OLIG1	0	0.129	0	0	0	0	0	0	0	0	0	0	0	0	0	0
OLIG3	0	0.129	0	0	0	0	0	0	0	0	0	0	0	0	0	0
OR12D3	0	0.129	0	0	0	0	0	0	0	0	0	0	0	0	0	0
OSTF1	0	0.129	0	0	0	0	0	0	0	0	0	0	0	0	0	0
AFM	0.129	0	0	0	0	0	0	0	0	0	0	0	0	0	0	0
ARHGAP35	0.129	0	0	0	0	0	0	0	0	0	0	0	0	0	0	0
CALR	0.129	0	0	0	0	0	0	0	0	0	0	0	0	0	0	0
CHAF1B	0.129	0	0	0	0	0	0	0	0	0	0	0	0	0	0	0
CLASP2	0.129	0	0	0	0	0	0	0	0	0	0	0	0	0	0	0
ENO1P1	0.129	0	0	0	0	0	0	0	0	0	0	0	0	0	0	0
FRA19A	0.129	0	0	0	0	0	0	0	0	0	0	0	0	0	0	0
HEBP2	0.129	0	0	0	0	0	0	0	0	0	0	0	0	0	0	0
MARCH1	0.129	0	0	0	0	0	0	0	0	0	0	0	0	0	0	0
S100A7P2	0.129	0	0	0	0	0	0	0	0	0	0	0	0	0	0	0
SQRDL	0.129	0	0	0	0	0	0	0	0	0	0	0	0	0	0	0
ZNF366	0.129	0	0	0	0	0	0	0	0	0	0	0	0	0	0	0
AQP7P3	0	0	0	0	0	0	0	0	0	0	0	0	0	0	0	0.128
CHST9	0	0	0	0	0	0	0	0	0	0	0	0	0	0	0	0.128
CILD4	0	0	0	0	0	0	0	0	0	0	0	0	0	0	0	0.128
FKBP11	0	0	0	0	0	0	0	0	0	0	0	0	0	0	0.128	0
DNAJB9	0	0	0	0	0	0	0	0	0	0	0	0	0	0.128	0	0
DRAM2	0	0	0	0	0	0	0	0	0	0	0	0	0	0.128	0	0
EARS2	0	0	0	0	0	0	0	0	0	0	0	0	0	0.128	0	0
EDEM1	0	0	0	0	0	0	0	0	0	0	0	0	0	0.128	0	0
EFHD1	0	0	0	0	0	0	0	0	0	0	0	0	0	0.128	0	0
IGF2R	0	0	0	0	0	0	0	0	0	0	0	0	0	0.128	0	0
COQ6	0	0	0	0	0	0	0	0	0	0	0	0.128	0	0	0	0
GIPC3	0	0	0	0	0	0	0	0	0	0	0	0.128	0	0	0	0
CEBPA	0	0	0	0	0	0	0	0	0	0	0.128	0	0	0	0	0
DCTN6	0	0	0	0	0	0	0	0	0	0	0.128	0	0	0	0	0

MIR1470	0	0	0	0	0	0	0	0	0	0	0.128	0	0	0	0	0
ANKH	0	0	0	0	0	0	0	0	0	0.128	0	0	0	0	0	0
BFAR	0	0	0	0	0	0	0	0	0	0.128	0	0	0	0	0	0
RDH5	0	0	0	0	0	0	0	0	0	0.128	0	0	0	0	0	0
RGR	0	0	0	0	0	0	0	0	0	0.128	0	0	0	0	0	0
RPE65	0	0	0	0	0	0	0	0	0	0.128	0	0	0	0	0	0
LIFR	0	0.128	0	0	0	0	0	0	0	0	0	0	0	0	0	0
LINC00162	0	0.128	0	0	0	0	0	0	0	0	0	0	0	0	0	0
LINC00163	0	0.128	0	0	0	0	0	0	0	0	0	0	0	0	0	0
LINC00165	0	0.128	0	0	0	0	0	0	0	0	0	0	0	0	0	0
LMO4	0	0.128	0	0	0	0	0	0	0	0	0	0	0	0	0	0
MT1P3	0	0.128	0	0	0	0	0	0	0	0	0	0	0	0	0	0
CABYR	0.128	0	0	0	0	0	0	0	0	0	0	0	0	0	0	0
CRB3	0.128	0	0	0	0	0	0	0	0	0	0	0	0	0	0	0
CSDAP1	0.128	0	0	0	0	0	0	0	0	0	0	0	0	0	0	0
DPYSL3	0.128	0	0	0	0	0	0	0	0	0	0	0	0	0	0	0
FER1L5	0.128	0	0	0	0	0	0	0	0	0	0	0	0	0	0	0
FSCB	0.128	0	0	0	0	0	0	0	0	0	0	0	0	0	0	0
GCLC	0.128	0	0	0	0	0	0	0	0	0	0	0	0	0	0	0
GUCA1C	0.128	0	0	0	0	0	0	0	0	0	0	0	0	0	0	0
MSK9	0.128	0	0	0	0	0	0	0	0	0	0	0	0	0	0	0
S100Z	0.128	0	0	0	0	0	0	0	0	0	0	0	0	0	0	0
ZXDA	0.128	0	0	0	0	0	0	0	0	0	0	0	0	0	0	0
AHNAK2	0	0	0	0	0	0	0	0	0	0	0	0	0	0	0	0.127
PPATP1	0	0	0	0	0	0	0	0	0	0	0	0	0	0	0	0.127
DNAJA4	0	0	0	0	0	0	0	0	0	0	0	0	0	0.127	0	0
DNAJB12	0	0	0	0	0	0	0	0	0	0	0	0	0	0.127	0	0
FASTKD1	0	0	0	0	0	0	0	0	0	0	0	0	0	0.127	0	0
GNPTG	0	0	0	0	0	0	0	0	0	0	0	0	0	0.127	0	0
GUSB	0	0	0	0	0	0	0	0	0	0	0	0	0	0.127	0	0
KIRREL3	0	0	0	0	0	0	0	0	0	0	0	0.127	0	0	0	0
EDF1	0	0	0	0	0	0	0	0	0	0	0.127	0	0	0	0	0
CABP2	0	0	0	0	0	0	0	0	0	0.127	0	0	0	0	0	0
TMEM66	0	0	0	0	0	0	0	0	0	0.127	0	0	0	0	0	0
TSFM	0	0	0	0	0	0	0.127	0	0	0	0	0	0	0	0	0
KIRREL	0	0.127	0	0	0	0	0	0	0	0	0	0	0	0	0	0
LHX5	0	0.127	0	0	0	0	0	0	0	0	0	0	0	0	0	0
LOC100288318	0	0.127	0	0	0	0	0	0	0	0	0	0	0	0	0	0
MDGA2	0	0.127	0	0	0	0	0	0	0	0	0	0	0	0	0	0
MET	0	0.127	0	0	0	0	0	0	0	0	0	0	0	0	0	0
ESRRAP1	0.127	0	0	0	0	0	0	0	0	0	0	0	0	0	0	0

MIR510	0.127	0	0	0	0	0	0	0	0	0	0	0	0	0	0	0
NTT	0.127	0	0	0	0	0	0	0	0	0	0	0	0	0	0	0
S100A12	0.127	0	0	0	0	0	0	0	0	0	0	0	0	0	0	0
VN1R4	0.127	0	0	0	0	0	0	0	0	0	0	0	0	0	0	0
ZNF132	0.127	0	0	0	0	0	0	0	0	0	0	0	0	0	0	0
C1QTNF7	0	0	0	0	0	0	0	0	0	0	0	0	0	0	0	0.126
C6ORF221	0	0	0	0	0	0	0	0	0	0	0	0	0	0	0	0.126
CAT	0	0	0	0	0	0	0	0	0	0	0	0	0	0	0	0.126
NPPA	0	0	0	0	0	0	0	0	0	0	0	0	0	0	0	0.126
FASTKD3	0	0	0	0	0	0	0	0	0	0	0	0	0	0.126	0	0
FASTKD5	0	0	0	0	0	0	0	0	0	0	0	0	0	0.126	0	0
FTMT	0	0	0	0	0	0	0	0	0	0	0	0	0	0.126	0	0
GATC	0	0	0	0	0	0	0	0	0	0	0	0	0	0.126	0	0
GLB1	0	0	0	0	0	0	0	0	0	0	0	0	0	0.126	0	0
GM2A	0	0	0	0	0	0	0	0	0	0	0	0	0	0.126	0	0
GPR63	0	0	0	0	0	0	0	0	0	0	0	0	0	0.126	0	0
HSCB	0	0	0	0	0	0	0	0	0	0	0	0	0	0.126	0	0
HSPA6	0	0	0	0	0	0	0	0	0	0	0	0	0	0.126	0	0
HOGA1	0	0	0	0	0	0	0	0	0	0	0	0.126	0	0	0	0
NIPAL2	0	0	0	0	0	0	0	0	0	0	0	0.126	0	0	0	0
TPRN	0	0	0	0	0	0	0	0	0	0	0	0.126	0	0	0	0
TACR1	0	0	0	0	0	0	0	0	0	0	0.126	0	0	0	0	0
GRK7	0	0	0	0	0	0	0	0	0	0.126	0	0	0	0	0	0
HMCN1	0	0	0	0	0	0	0	0	0	0.126	0	0	0	0	0	0
MIAT	0	0	0	0	0	0	0	0	0	0.126	0	0	0	0	0	0
RS1	0	0	0	0	0	0	0	0	0	0.126	0	0	0	0	0	0
ZMPSTE24	0	0	0	0.126	0	0	0	0	0	0	0	0	0	0	0	0
LOH1CR1	0	0.126	0	0	0	0	0	0	0	0	0	0	0	0	0	0
LOX	0	0.126	0	0	0	0	0	0	0	0	0	0	0	0	0	0
LSM10	0	0.126	0	0	0	0	0	0	0	0	0	0	0	0	0	0
LSM11	0	0.126	0	0	0	0	0	0	0	0	0	0	0	0	0	0
MAP1A	0	0.126	0	0	0	0	0	0	0	0	0	0	0	0	0	0
MBP	0	0.126	0	0	0	0	0	0	0	0	0	0	0	0	0	0
MEOX1	0	0.126	0	0	0	0	0	0	0	0	0	0	0	0	0	0
MSTN	0	0.126	0	0	0	0	0	0	0	0	0	0	0	0	0	0
MT1E	0	0.126	0	0	0	0	0	0	0	0	0	0	0	0	0	0
DEPTOR	0.126	0	0	0	0	0	0	0	0	0	0	0	0	0	0	0
FRA2G	0.126	0	0	0	0	0	0	0	0	0	0	0	0	0	0	0
GSR	0.126	0	0	0	0	0	0	0	0	0	0	0	0	0	0	0
GTPBP1	0.126	0	0	0	0	0	0	0	0	0	0	0	0	0	0	0
GYG1	0.126	0	0	0	0	0	0	0	0	0	0	0	0	0	0	0

PLSCR4	0.126	0	0	0	0	0	0	0	0	0	0	0	0	0	0	0
POM121C	0.126	0	0	0	0	0	0	0	0	0	0	0	0	0	0	0
PPFIBP1	0.126	0	0	0	0	0	0	0	0	0	0	0	0	0	0	0
PRR5L	0.126	0	0	0	0	0	0	0	0	0	0	0	0	0	0	0
SPAG5	0.126	0	0	0	0	0	0	0	0	0	0	0	0	0	0	0
CYCS	0	0	0	0	0	0	0	0	0	0	0	0	0	0.125	0	0
JKAMP	0	0	0	0	0	0	0	0	0	0	0	0	0	0.125	0	0
NAT8L	0	0	0	0	0	0	0	0	0	0	0	0	0	0.125	0	0
NEU1	0	0	0	0	0	0	0	0	0	0	0	0	0	0.125	0	0
POGK	0	0	0	0	0	0	0	0	0	0	0	0	0	0.125	0	0
MSRB3	0	0	0	0	0	0	0	0	0	0	0	0.125	0	0	0	0
OR51B4	0	0	0	0	0	0	0	0	0	0	0	0.125	0	0	0	0
P2RX3	0	0	0	0	0	0	0	0	0	0	0	0.125	0	0	0	0
TECTB	0	0	0	0	0	0	0	0	0	0	0	0.125	0	0	0	0
TRNR	0	0	0	0	0	0	0	0	0	0	0	0.125	0	0	0	0
CEND1	0	0	0	0	0	0	0	0	0	0.125	0	0	0	0	0	0
LDHC	0	0	0	0	0.125	0	0	0	0	0	0	0	0	0	0	0
KIF3A	0	0.125	0	0	0	0	0	0	0	0	0	0	0	0	0	0
MT4	0	0.125	0	0	0	0	0	0	0	0	0	0	0	0	0	0
NR1H2	0	0.125	0	0	0	0	0	0	0	0	0	0	0	0	0	0
NSF	0	0.125	0	0	0	0	0	0	0	0	0	0	0	0	0	0
PAX1	0	0.125	0	0	0	0	0	0	0	0	0	0	0	0	0	0
ABCG8	0.125	0	0	0	0	0	0	0	0	0	0	0	0	0	0	0
CA11	0.125	0	0	0	0	0	0	0	0	0	0	0	0	0	0	0
DYRK1B	0.125	0	0	0	0	0	0	0	0	0	0	0	0	0	0	0
GSTM5P1	0.125	0	0	0	0	0	0	0	0	0	0	0	0	0	0	0
LIPI	0.125	0	0	0	0	0	0	0	0	0	0	0	0	0	0	0
SLC2A3P2	0	0	0	0	0	0	0	0	0	0	0	0	0	0	0	0.124
FNTAP2	0	0	0	0	0	0	0	0	0	0	0	0	0	0	0.124	0
GLUD1P3	0	0	0	0	0	0	0	0	0	0	0	0	0	0	0.124	0
PKDCC	0	0	0	0	0	0	0	0	0	0	0	0	0	0	0.124	0
CERS1	0	0	0	0	0	0	0	0	0	0	0	0	0	0.124	0	0
LAMP1	0	0	0	0	0	0	0	0	0	0	0	0	0	0.124	0	0
LONP1	0	0	0	0	0	0	0	0	0	0	0	0	0	0.124	0	0
MTRF1	0	0	0	0	0	0	0	0	0	0	0	0	0	0.124	0	0
FAM188B2	0	0	0	0	0	0	0	0	0	0	0	0.124	0	0	0	0
TMC4	0	0	0	0	0	0	0	0	0	0	0	0.124	0	0	0	0
CTBP1	0	0	0	0	0	0	0	0	0	0	0.124	0	0	0	0	0
DAGLB	0	0	0	0	0	0	0	0	0	0	0.124	0	0	0	0	0
ACAD9	0	0	0	0	0	0	0.124	0	0	0	0	0	0	0	0	0
MRPL22	0	0	0	0	0	0	0.124	0	0	0	0	0	0	0	0	0

MRPL38	0	0	0	0	0	0	0.124	0	0	0	0	0	0	0	0	0
NDUFS6	0	0	0	0	0	0	0.124	0	0	0	0	0	0	0	0	0
CHRNB1	0	0	0	0	0	0.124	0	0	0	0	0	0	0	0	0	0
CUX2	0	0	0	0	0	0.124	0	0	0	0	0	0	0	0	0	0
L1RE4	0	0	0	0	0	0.124	0	0	0	0	0	0	0	0	0	0
SSTR4	0	0	0	0	0	0.124	0	0	0	0	0	0	0	0	0	0
ND2	0	0	0	0	0.124	0	0	0	0	0	0	0	0	0	0	0
CNTNAP4	0	0	0	0.124	0	0	0	0	0	0	0	0	0	0	0	0
SRBD1	0	0	0	0.124	0	0	0	0	0	0	0	0	0	0	0	0
C11ORF96	0	0	0.124	0	0	0	0	0	0	0	0	0	0	0	0	0
ABLIM1	0	0.124	0	0	0	0	0	0	0	0	0	0	0	0	0	0
CD226	0	0.124	0	0	0	0	0	0	0	0	0	0	0	0	0	0
FMO1	0	0.124	0	0	0	0	0	0	0	0	0	0	0	0	0	0
GALR2	0	0.124	0	0	0	0	0	0	0	0	0	0	0	0	0	0
IGFBP3	0	0.124	0	0	0	0	0	0	0	0	0	0	0	0	0	0
MTF1	0	0.124	0	0	0	0	0	0	0	0	0	0	0	0	0	0
MYT1L	0	0.124	0	0	0	0	0	0	0	0	0	0	0	0	0	0
NOTO	0	0.124	0	0	0	0	0	0	0	0	0	0	0	0	0	0
SNRPD3	0	0.124	0	0	0	0	0	0	0	0	0	0	0	0	0	0
TBCD	0	0.124	0	0	0	0	0	0	0	0	0	0	0	0	0	0
ALDH1L1	0.124	0	0	0	0	0	0	0	0	0	0	0	0	0	0	0
FAM60A	0.124	0	0	0	0	0	0	0	0	0	0	0	0	0	0	0
LPL	0.124	0	0	0	0	0	0	0	0	0	0	0	0	0	0	0
RPS6KA3	0.124	0	0	0	0	0	0	0	0	0	0	0	0	0	0	0
SENP2	0.124	0	0	0	0	0	0	0	0	0	0	0	0	0	0	0
CYP1A2	0	0	0	0	0	0	0	0	0	0	0	0	0	0	0	0.123
ACO1	0	0	0	0	0	0	0	0	0	0	0	0	0	0.123	0	0
LPPR1	0	0	0	0	0	0	0	0	0	0	0	0	0	0.123	0	0
MAP6D1	0	0	0	0	0	0	0	0	0	0	0	0	0	0.123	0	0
MTIF3	0	0	0	0	0	0	0	0	0	0	0	0	0	0.123	0	0
DFNB51	0	0	0	0	0	0	0	0	0	0	0	0.123	0	0	0	0
MYH14	0	0	0	0	0	0	0	0	0	0	0	0.123	0	0	0	0
TMC6	0	0	0	0	0	0	0	0	0	0	0	0.123	0	0	0	0
TRAPPC2	0	0	0	0	0	0	0	0	0	0	0	0.123	0	0	0	0
CLTB	0	0	0	0	0	0	0	0	0	0	0.123	0	0	0	0	0
TAF2	0	0	0	0	0	0	0	0	0	0	0.123	0	0	0	0	0
TAF7	0	0	0	0	0	0	0	0	0	0	0.123	0	0	0	0	0
FOSB	0	0	0	0	0	0	0	0	0	0.123	0	0	0	0	0	0
PROX2	0	0	0	0	0	0	0	0	0	0.123	0	0	0	0	0	0
AUH	0	0	0	0	0	0	0.123	0	0	0	0	0	0	0	0	0
COX4I2	0	0	0	0	0	0	0.123	0	0	0	0	0	0	0	0	0

PEX6	0	0	0	0	0	0	0.123	0	0	0	0	0	0	0	0	0
PHKA2	0	0	0	0	0	0	0.123	0	0	0	0	0	0	0	0	0
POLG2	0	0	0	0	0	0	0.123	0	0	0	0	0	0	0	0	0
KCNK18	0	0	0	0	0	0.123	0	0	0	0	0	0	0	0	0	0
BANK1	0	0.123	0	0	0	0	0	0	0	0	0	0	0	0	0	0
C10ORF27	0	0.123	0	0	0	0	0	0	0	0	0	0	0	0	0	0
CRYAB	0	0.123	0	0	0	0	0	0	0	0	0	0	0	0	0	0
FBL	0	0.123	0	0	0	0	0	0	0	0	0	0	0	0	0	0
FOLH1B	0	0.123	0	0	0	0	0	0	0	0	0	0	0	0	0	0
GEMIN5	0	0.123	0	0	0	0	0	0	0	0	0	0	0	0	0	0
GEMIN8	0	0.123	0	0	0	0	0	0	0	0	0	0	0	0	0	0
GPR161	0	0.123	0	0	0	0	0	0	0	0	0	0	0	0	0	0
GRIPAP1	0	0.123	0	0	0	0	0	0	0	0	0	0	0	0	0	0
MZB1	0	0.123	0	0	0	0	0	0	0	0	0	0	0	0	0	0
NKX6-1	0	0.123	0	0	0	0	0	0	0	0	0	0	0	0	0	0
NODAL	0	0.123	0	0	0	0	0	0	0	0	0	0	0	0	0	0
ALAS1	0.123	0	0	0	0	0	0	0	0	0	0	0	0	0	0	0
BTRC	0.123	0	0	0	0	0	0	0	0	0	0	0	0	0	0	0
EIF2B3	0.123	0	0	0	0	0	0	0	0	0	0	0	0	0	0	0
LDLRAP1	0.123	0	0	0	0	0	0	0	0	0	0	0	0	0	0	0
MTHFD2	0.123	0	0	0	0	0	0	0	0	0	0	0	0	0	0	0
NCOA2	0.123	0	0	0	0	0	0	0	0	0	0	0	0	0	0	0
ZNF280B	0.123	0	0	0	0	0	0	0	0	0	0	0	0	0	0	0
ADRB1	0	0	0	0	0	0	0	0	0	0	0	0	0	0	0	0.122
BRD7P3	0	0	0	0	0	0	0	0	0	0	0	0	0	0	0	0.122
PIGG	0	0	0	0	0	0	0	0	0	0	0	0	0	0	0	0.122
ASIP	0	0	0	0	0	0	0	0	0	0	0	0	0	0.122	0	0
CLPP	0	0	0	0	0	0	0	0	0	0	0	0	0	0.122	0	0
COX2	0	0	0	0	0	0	0	0	0	0	0	0	0	0.122	0	0
COX6C	0	0	0	0	0	0	0	0	0	0	0	0	0	0.122	0	0
C6ORF120	0	0	0	0	0	0	0	0	0	0	0.122	0	0	0	0	0
PIK3R4	0	0	0	0	0	0	0	0	0	0	0.122	0	0	0	0	0
BEST1	0	0	0	0	0	0	0	0	0	0.122	0	0	0	0	0	0
CDK1	0	0	0	0	0	0	0	0	0.122	0	0	0	0	0	0	0
CMM4	0	0	0	0	0	0	0	0	0.122	0	0	0	0	0	0	0
DEFT1P2	0	0	0	0	0	0	0	0	0.122	0	0	0	0	0	0	0
FMNL1	0	0	0	0	0	0	0	0	0.122	0	0	0	0	0	0	0
HNRNPA2B1	0	0	0	0	0	0	0	0	0.122	0	0	0	0	0	0	0
KHDRBS3	0	0	0	0	0	0	0	0	0.122	0	0	0	0	0	0	0
MAP2K1	0	0	0	0	0	0	0	0	0.122	0	0	0	0	0	0	0
PRMT8	0	0	0	0	0	0	0	0	0.122	0	0	0	0	0	0	0

DPYS	0	0	0	0	0	0	0.122	0	0	0	0	0	0	0	0	0
ETHE1	0	0	0	0	0	0	0.122	0	0	0	0	0	0	0	0	0
FARS2	0	0	0	0	0	0	0.122	0	0	0	0	0	0	0	0	0
FRA1E	0	0	0	0	0	0	0.122	0	0	0	0	0	0	0	0	0
GK	0	0	0	0	0	0	0.122	0	0	0	0	0	0	0	0	0
GLYCTK	0	0	0	0	0	0	0.122	0	0	0	0	0	0	0	0	0
MBD6	0	0	0	0	0	0	0.122	0	0	0	0	0	0	0	0	0
MOCS2	0	0	0	0	0	0	0.122	0	0	0	0	0	0	0	0	0
CHP2	0	0	0	0	0	0.122	0	0	0	0	0	0	0	0	0	0
FXYD7	0	0	0	0	0	0.122	0	0	0	0	0	0	0	0	0	0
TMEM163	0	0	0	0	0	0.122	0	0	0	0	0	0	0	0	0	0
KIF17	0	0	0.122	0	0	0	0	0	0	0	0	0	0	0	0	0
HGF	0	0.122	0	0	0	0	0	0	0	0	0	0	0	0	0	0
IL21R	0	0.122	0	0	0	0	0	0	0	0	0	0	0	0	0	0
IL6R	0	0.122	0	0	0	0	0	0	0	0	0	0	0	0	0	0
ISL1	0	0.122	0	0	0	0	0	0	0	0	0	0	0	0	0	0
TNFSF13B	0	0.122	0	0	0	0	0	0	0	0	0	0	0	0	0	0
ABCG5	0.122	0	0	0	0	0	0	0	0	0	0	0	0	0	0	0
DHFR	0.122	0	0	0	0	0	0	0	0	0	0	0	0	0	0	0
DVL2	0.122	0	0	0	0	0	0	0	0	0	0	0	0	0	0	0
FAM153B	0.122	0	0	0	0	0	0	0	0	0	0	0	0	0	0	0
MIR320A	0.122	0	0	0	0	0	0	0	0	0	0	0	0	0	0	0
TCN2	0.122	0	0	0	0	0	0	0	0	0	0	0	0	0	0	0
ZNF131	0.122	0	0	0	0	0	0	0	0	0	0	0	0	0	0	0
ZNF26	0.122	0	0	0	0	0	0	0	0	0	0	0	0	0	0	0
CXADR	0	0	0	0	0	0	0	0	0	0	0	0	0	0	0	0.121
IFT80	0	0	0	0	0	0	0	0	0	0	0	0.121	0	0	0	0
OR10J5	0	0	0	0	0	0	0	0	0	0	0	0.121	0	0	0	0
NPM1	0	0	0	0	0	0	0	0	0	0	0.121	0	0	0	0	0
TAF5	0	0	0	0	0	0	0	0	0	0	0.121	0	0	0	0	0
NGLY1	0	0	0	0	0	0	0	0	0.121	0	0	0	0	0	0	0
ATPAF2	0	0	0	0	0	0	0.121	0	0	0	0	0	0	0	0	0
BCKDHA	0	0	0	0	0	0	0.121	0	0	0	0	0	0	0	0	0
MRPL52	0	0	0	0	0	0	0.121	0	0	0	0	0	0	0	0	0
RNASEH2A	0	0	0	0	0	0	0.121	0	0	0	0	0	0	0	0	0
RRIS	0	0	0	0	0	0	0.121	0	0	0	0	0	0	0	0	0
ZDHHC3	0	0	0	0	0	0.121	0	0	0	0	0	0	0	0	0	0
DLGAP3	0	0	0.121	0	0	0	0	0	0	0	0	0	0	0	0	0
IFITM8P	0	0	0.121	0	0	0	0	0	0	0	0	0	0	0	0	0
INTS4	0	0	0.121	0	0	0	0	0	0	0	0	0	0	0	0	0
KIAA0513	0	0	0.121	0	0	0	0	0	0	0	0	0	0	0	0	0

NF1	0	0	0.121	0	0	0	0	0	0	0	0	0	0	0	0	0
NXF2	0	0	0.121	0	0	0	0	0	0	0	0	0	0	0	0	0
PPP1R9B	0	0	0.121	0	0	0	0	0	0	0	0	0	0	0	0	0
TBX6	0	0.121	0	0	0	0	0	0	0	0	0	0	0	0	0	0
TMEM39A	0	0.121	0	0	0	0	0	0	0	0	0	0	0	0	0	0
TMOD4	0	0.121	0	0	0	0	0	0	0	0	0	0	0	0	0	0
TNFSF12	0	0.121	0	0	0	0	0	0	0	0	0	0	0	0	0	0
TRMT1L	0	0.121	0	0	0	0	0	0	0	0	0	0	0	0	0	0
UBE2H	0	0.121	0	0	0	0	0	0	0	0	0	0	0	0	0	0
VANGL2	0	0.121	0	0	0	0	0	0	0	0	0	0	0	0	0	0
CLEC4D	0.121	0	0	0	0	0	0	0	0	0	0	0	0	0	0	0
DYRK4	0.121	0	0	0	0	0	0	0	0	0	0	0	0	0	0	0
ECEL1P3	0.121	0	0	0	0	0	0	0	0	0	0	0	0	0	0	0
FRA11B	0.121	0	0	0	0	0	0	0	0	0	0	0	0	0	0	0
GPRIN2	0.121	0	0	0	0	0	0	0	0	0	0	0	0	0	0	0
NFATC1	0.121	0	0	0	0	0	0	0	0	0	0	0	0	0	0	0
PALM2-AKAP2	0	0	0	0	0	0	0	0	0	0	0	0	0	0	0	0.12
ANO8	0	0	0	0	0	0	0	0	0	0	0	0.12	0	0	0	0
KRT9	0	0	0	0	0	0	0	0	0	0	0	0.12	0	0	0	0
MTO1	0	0	0	0	0	0	0	0	0	0	0	0.12	0	0	0	0
GRM6	0	0	0	0	0	0	0	0	0	0.12	0	0	0	0	0	0
PDE6A	0	0	0	0	0	0	0	0	0	0.12	0	0	0	0	0	0
ABCG2	0	0	0	0	0	0	0.12	0	0	0	0	0	0	0	0	0
ATP1B3P1	0	0	0	0	0	0.12	0	0	0	0	0	0	0	0	0	0
PPP3CC	0	0	0.12	0	0	0	0	0	0	0	0	0	0	0	0	0
VAPA	0	0.12	0	0	0	0	0	0	0	0	0	0	0	0	0	0
AKAP11	0.12	0	0	0	0	0	0	0	0	0	0	0	0	0	0	0
C21ORF33	0.12	0	0	0	0	0	0	0	0	0	0	0	0	0	0	0
LOC100271918	0.12	0	0	0	0	0	0	0	0	0	0	0	0	0	0	0
QPCTL	0.12	0	0	0	0	0	0	0	0	0	0	0	0	0	0	0
RFC1	0.12	0	0	0	0	0	0	0	0	0	0	0	0	0	0	0
YBX1P10	0.12	0	0	0	0	0	0	0	0	0	0	0	0	0	0	0
APLN	0	0	0	0	0	0	0	0	0	0	0	0	0	0	0	0.119
PGS1	0	0	0	0	0	0	0	0	0	0	0	0	0	0	0	0.119
TNNT3	0	0	0	0	0	0	0	0	0	0	0	0	0	0	0	0.119
CDSN	0	0	0	0	0	0	0	0	0	0	0	0	0	0	0.119	0
RILPL2	0	0	0	0	0	0	0	0	0	0	0	0	0	0.119	0	0
SLC25A14	0	0	0	0	0	0	0	0	0	0	0	0	0	0.119	0	0
ST8SIA1	0	0	0	0	0	0	0	0	0	0	0	0	0	0.119	0	0
STX8	0	0	0	0	0	0	0	0	0	0	0	0	0	0.119	0	0

TMED4	0	0	0	0	0	0	0	0	0	0	0	0	0	0.119	0	0
VPS35	0	0	0	0	0	0	0	0	0	0	0	0	0	0.119	0	0
LMBRD1	0	0	0	0	0	0	0	0	0	0	0	0	0.119	0	0	0
FOXI3	0	0	0	0	0	0	0	0	0	0	0	0.119	0	0	0	0
IFT27	0	0	0	0	0	0	0	0	0	0	0	0.119	0	0	0	0
NDUFB9	0	0	0	0	0	0	0	0	0	0	0	0.119	0	0	0	0
SDHD	0	0	0	0	0	0	0	0	0	0	0	0.119	0	0	0	0
TMC3	0	0	0	0	0	0	0	0	0	0	0	0.119	0	0	0	0
BAIAP2	0	0	0	0	0	0	0	0	0	0	0.119	0	0	0	0	0
MIR30A	0	0	0	0	0	0	0	0	0	0	0.119	0	0	0	0	0
OPN1MW2	0	0	0	0	0	0	0	0	0	0.119	0	0	0	0	0	0
VCPIP1	0	0	0	0	0	0	0	0	0.119	0	0	0	0	0	0	0
TRNAP1	0	0	0	0	0	0	0.119	0	0	0	0	0	0	0	0	0
KCNH8	0	0	0	0	0	0.119	0	0	0	0	0	0	0	0	0	0
KCNJ3	0	0	0	0	0	0.119	0	0	0	0	0	0	0	0	0	0
CAMKK2	0	0	0.119	0	0	0	0	0	0	0	0	0	0	0	0	0
CDC42SE2	0	0	0.119	0	0	0	0	0	0	0	0	0	0	0	0	0
TDO2	0	0	0.119	0	0	0	0	0	0	0	0	0	0	0	0	0
POP7	0	0.119	0	0	0	0	0	0	0	0	0	0	0	0	0	0
PSMB9	0	0.119	0	0	0	0	0	0	0	0	0	0	0	0	0	0
RAPGEF6	0	0.119	0	0	0	0	0	0	0	0	0	0	0	0	0	0
REXO4	0	0.119	0	0	0	0	0	0	0	0	0	0	0	0	0	0
RHEB	0	0.119	0	0	0	0	0	0	0	0	0	0	0	0	0	0
RRAD	0	0.119	0	0	0	0	0	0	0	0	0	0	0	0	0	0
SEMA4D	0	0.119	0	0	0	0	0	0	0	0	0	0	0	0	0	0
SNRPD2	0	0.119	0	0	0	0	0	0	0	0	0	0	0	0	0	0
SNRPE	0	0.119	0	0	0	0	0	0	0	0	0	0	0	0	0	0
SNRPF	0	0.119	0	0	0	0	0	0	0	0	0	0	0	0	0	0
TBX18	0	0.119	0	0	0	0	0	0	0	0	0	0	0	0	0	0
TBX20	0	0.119	0	0	0	0	0	0	0	0	0	0	0	0	0	0
ZNF433	0	0.119	0	0	0	0	0	0	0	0	0	0	0	0	0	0
ANGPTL3	0.119	0	0	0	0	0	0	0	0	0	0	0	0	0	0	0
CIB3	0.119	0	0	0	0	0	0	0	0	0	0	0	0	0	0	0
CKAP5	0.119	0	0	0	0	0	0	0	0	0	0	0	0	0	0	0
CTPS	0.119	0	0	0	0	0	0	0	0	0	0	0	0	0	0	0
FRAXB	0.119	0	0	0	0	0	0	0	0	0	0	0	0	0	0	0
NR1D2	0.119	0	0	0	0	0	0	0	0	0	0	0	0	0	0	0
PAQR9	0.119	0	0	0	0	0	0	0	0	0	0	0	0	0	0	0
RAD54B	0.119	0	0	0	0	0	0	0	0	0	0	0	0	0	0	0
TRIM29	0.119	0	0	0	0	0	0	0	0	0	0	0	0	0	0	0
ZNF140	0.119	0	0	0	0	0	0	0	0	0	0	0	0	0	0	0

ZNF808	0.119	0	0	0	0	0	0	0	0	0	0	0	0	0	0	0
CST9	0	0	0	0	0	0	0	0	0	0	0	0	0	0	0	0.118
SRL	0	0	0	0	0	0	0	0	0	0	0	0	0	0	0	0.118
VN1R7P	0	0	0	0	0	0	0	0	0	0	0	0.118	0	0	0	0
EIF2C2	0	0	0	0	0	0	0	0	0	0	0.118	0	0	0	0	0
FAAH2	0	0	0	0	0	0	0	0	0	0	0.118	0	0	0	0	0
GNG7	0	0	0	0	0	0	0	0	0	0	0.118	0	0	0	0	0
AIPL1	0	0	0	0	0	0	0	0	0	0.118	0	0	0	0	0	0
CNGB3	0	0	0	0	0	0	0	0	0	0.118	0	0	0	0	0	0
RP23	0	0	0	0	0	0	0	0	0	0.118	0	0	0	0	0	0
SERPINF1	0	0	0	0	0	0	0	0	0	0.118	0	0	0	0	0	0
SOX9	0	0	0	0	0	0	0	0	0	0.118	0	0	0	0	0	0
CSF2	0	0.118	0	0	0	0	0	0	0	0	0	0	0	0	0	0
SEMA6A	0	0.118	0	0	0	0	0	0	0	0	0	0	0	0	0	0
ANKRA2	0.118	0	0	0	0	0	0	0	0	0	0	0	0	0	0	0
APOA4	0.118	0	0	0	0	0	0	0	0	0	0	0	0	0	0	0
GPR27	0.118	0	0	0	0	0	0	0	0	0	0	0	0	0	0	0
GSS	0.118	0	0	0	0	0	0	0	0	0	0	0	0	0	0	0
RAP1GAP	0.118	0	0	0	0	0	0	0	0	0	0	0	0	0	0	0
S7	0.118	0	0	0	0	0	0	0	0	0	0	0	0	0	0	0
SPICE1	0.118	0	0	0	0	0	0	0	0	0	0	0	0	0	0	0
SPON2	0.118	0	0	0	0	0	0	0	0	0	0	0	0	0	0	0
SSPO	0.118	0	0	0	0	0	0	0	0	0	0	0	0	0	0	0
C19ORF43	0	0	0	0	0	0	0	0	0	0	0	0.117	0	0	0	0
SEMA4B	0	0	0	0	0	0	0	0	0	0	0	0.117	0	0	0	0
C7ORF30	0	0	0	0	0	0	0.117	0	0	0	0	0	0	0	0	0
COX18	0	0	0	0	0	0	0.117	0	0	0	0	0	0	0	0	0
COX6B1	0	0	0	0	0	0	0.117	0	0	0	0	0	0	0	0	0
HADH	0	0	0	0	0	0	0.117	0	0	0	0	0	0	0	0	0
MRPS16	0	0	0	0	0	0	0.117	0	0	0	0	0	0	0	0	0
NAGS	0	0	0	0	0	0	0.117	0	0	0	0	0	0	0	0	0
PDHB	0	0	0	0	0	0	0.117	0	0	0	0	0	0	0	0	0
PSPH	0	0	0	0	0	0	0.117	0	0	0	0	0	0	0	0	0
AXIN1	0.117	0	0	0	0	0	0	0	0	0	0	0	0	0	0	0
BOLA2B	0.117	0	0	0	0	0	0	0	0	0	0	0	0	0	0	0
EIF2AK4	0.117	0	0	0	0	0	0	0	0	0	0	0	0	0	0	0
ITGA10	0.117	0	0	0	0	0	0	0	0	0	0	0	0	0	0	0
NACA	0.117	0	0	0	0	0	0	0	0	0	0	0	0	0	0	0
PCBP2P2	0.117	0	0	0	0	0	0	0	0	0	0	0	0	0	0	0
XCE	0.117	0	0	0	0	0	0	0	0	0	0	0	0	0	0	0
EFR3A	0	0	0	0	0	0	0	0	0	0	0	0.116	0	0	0	0

FAM151A	0	0	0	0	0	0	0	0	0	0	0	0.116	0	0	0	0
LHFPL1	0	0	0	0	0	0	0	0	0	0	0	0.116	0	0	0	0
ABCA4	0	0	0	0	0	0	0	0	0	0.116	0	0	0	0	0	0
CABP4	0	0	0	0	0	0	0	0	0	0.116	0	0	0	0	0	0
ARSE	0	0	0	0	0	0	0.116	0	0	0	0	0	0	0	0	0
COX7B	0	0	0	0	0	0	0.116	0	0	0	0	0	0	0	0	0
MRPL21	0	0	0	0	0	0	0.116	0	0	0	0	0	0	0	0	0
MRPL55	0	0	0	0	0	0	0.116	0	0	0	0	0	0	0	0	0
PTCD2	0	0	0	0	0	0	0.116	0	0	0	0	0	0	0	0	0
XS	0	0	0	0.116	0	0	0	0	0	0	0	0	0	0	0	0
APC2	0.116	0	0	0	0	0	0	0	0	0	0	0	0	0	0	0
BW32	0.116	0	0	0	0	0	0	0	0	0	0	0	0	0	0	0
C14ORF129	0.116	0	0	0	0	0	0	0	0	0	0	0	0	0	0	0
CDR1	0.116	0	0	0	0	0	0	0	0	0	0	0	0	0	0	0
CEP192	0.116	0	0	0	0	0	0	0	0	0	0	0	0	0	0	0
CHE2	0.116	0	0	0	0	0	0	0	0	0	0	0	0	0	0	0
CSNK1A1	0.116	0	0	0	0	0	0	0	0	0	0	0	0	0	0	0
DIAPH3	0.116	0	0	0	0	0	0	0	0	0	0	0	0	0	0	0
FRA6F	0.116	0	0	0	0	0	0	0	0	0	0	0	0	0	0	0
GRHL3	0.116	0	0	0	0	0	0	0	0	0	0	0	0	0	0	0
INSM2	0.116	0	0	0	0	0	0	0	0	0	0	0	0	0	0	0
TNFSF18	0.116	0	0	0	0	0	0	0	0	0	0	0	0	0	0	0
ZNF460	0.116	0	0	0	0	0	0	0	0	0	0	0	0	0	0	0
ADAMTSL4	0	0	0	0	0	0	0	0	0	0	0	0.115	0	0	0	0
CDAN1	0	0	0	0	0	0	0	0	0	0	0	0.115	0	0	0	0
IFT122	0	0	0	0	0	0	0	0	0	0	0	0.115	0	0	0	0
LHFP	0	0	0	0	0	0	0	0	0	0	0	0.115	0	0	0	0
OTOP3	0	0	0	0	0	0	0	0	0	0	0	0.115	0	0	0	0
NEIL3	0	0	0	0	0	0	0	0	0	0	0.115	0	0	0	0	0
PGK1P1	0	0	0	0	0	0	0	0	0	0	0.115	0	0	0	0	0
ELOVL4	0	0	0	0	0	0	0	0	0	0.115	0	0	0	0	0	0
IMPDH1	0	0	0	0	0	0	0	0	0	0.115	0	0	0	0	0	0
KIAA0556	0	0	0	0	0	0	0	0	0	0.115	0	0	0	0	0	0
ACADS	0	0	0	0	0	0	0.115	0	0	0	0	0	0	0	0	0
SUCLA2	0	0	0	0	0	0	0.115	0	0	0	0	0	0	0	0	0
SUOX	0	0	0	0	0	0	0.115	0	0	0	0	0	0	0	0	0
UQCRC2	0	0	0	0	0	0	0.115	0	0	0	0	0	0	0	0	0
ANXA6	0.115	0	0	0	0	0	0	0	0	0	0	0	0	0	0	0
EPF5	0.115	0	0	0	0	0	0	0	0	0	0	0	0	0	0	0
MIR302C	0.115	0	0	0	0	0	0	0	0	0	0	0	0	0	0	0
MX2	0.115	0	0	0	0	0	0	0	0	0	0	0	0	0	0	0

PMVK	0.115	0	0	0	0	0	0	0	0	0	0	0	0	0	0	0
RPS29	0.115	0	0	0	0	0	0	0	0	0	0	0	0	0	0	0
ZNF205	0.115	0	0	0	0	0	0	0	0	0	0	0	0	0	0	0
RMRP	0	0	0	0	0	0	0	0	0	0	0	0	0.114	0	0	0
ALDH7A1P2	0	0	0	0	0	0	0	0	0	0	0	0.114	0	0	0	0
HSCRM2	0	0	0	0	0	0	0	0	0	0	0	0.114	0	0	0	0
LRRC4C	0	0	0	0	0	0	0	0	0	0	0	0.114	0	0	0	0
STOM	0	0	0	0	0	0	0	0	0	0	0	0.114	0	0	0	0
FAM53B	0	0	0	0	0	0	0	0	0	0.114	0	0	0	0	0	0
FGF9	0	0	0	0	0	0	0	0	0	0.114	0	0	0	0	0	0
GNAT1	0	0	0	0	0	0	0	0	0	0.114	0	0	0	0	0	0
PITPNM2	0	0	0	0	0	0	0	0	0	0.114	0	0	0	0	0	0
BCKDHB	0	0	0	0	0	0	0.114	0	0	0	0	0	0	0	0	0
PEX2	0	0	0	0	0	0	0.114	0	0	0	0	0	0	0	0	0
UPB1	0	0	0	0	0	0	0.114	0	0	0	0	0	0	0	0	0
NALCN	0	0	0	0	0	0.114	0	0	0	0	0	0	0	0	0	0
TTC37	0	0	0	0	0	0.114	0	0	0	0	0	0	0	0	0	0
ACAT2	0.114	0	0	0	0	0	0	0	0	0	0	0	0	0	0	0
APPL2	0.114	0	0	0	0	0	0	0	0	0	0	0	0	0	0	0
C16ORF7	0.114	0	0	0	0	0	0	0	0	0	0	0	0	0	0	0
CAPZA1	0.114	0	0	0	0	0	0	0	0	0	0	0	0	0	0	0
CD47	0.114	0	0	0	0	0	0	0	0	0	0	0	0	0	0	0
EIF2B1	0.114	0	0	0	0	0	0	0	0	0	0	0	0	0	0	0
EIF2S2	0.114	0	0	0	0	0	0	0	0	0	0	0	0	0	0	0
GSTP1P1	0.114	0	0	0	0	0	0	0	0	0	0	0	0	0	0	0
PLA1A	0.114	0	0	0	0	0	0	0	0	0	0	0	0	0	0	0
PMS2LP2	0.114	0	0	0	0	0	0	0	0	0	0	0	0	0	0	0
PPP1R2	0.114	0	0	0	0	0	0	0	0	0	0	0	0	0	0	0
TTIM1	0.114	0	0	0	0	0	0	0	0	0	0	0	0	0	0	0
ADRB3	0	0	0	0	0	0	0	0	0	0	0	0	0	0	0	0.113
TAF9P3	0	0	0	0	0	0	0	0	0	0	0	0	0	0	0	0.113
CATCN1	0	0	0	0	0	0	0	0	0	0	0	0.113	0	0	0	0
OR8A1	0	0	0	0	0	0	0	0	0	0	0	0.113	0	0	0	0
VUR	0	0	0	0	0	0	0	0	0	0	0	0.113	0	0	0	0
MIR1251	0	0	0	0	0	0	0	0	0	0	0.113	0	0	0	0	0
NRL	0	0	0	0	0	0	0	0	0	0.113	0	0	0	0	0	0
GOT1	0	0	0	0	0	0	0.113	0	0	0	0	0	0	0	0	0
METTL17	0	0	0	0	0	0	0.113	0	0	0	0	0	0	0	0	0
MGC1	0	0	0	0	0	0	0.113	0	0	0	0	0	0	0	0	0
UQCR11	0	0	0	0	0	0	0.113	0	0	0	0	0	0	0	0	0
UNC80	0	0	0	0	0	0.113	0	0	0	0	0	0	0	0	0	0

CST8	0	0	0	0	0.113	0	0	0	0	0	0	0	0	0	0	0
AIF1L	0.113	0	0	0	0	0	0	0	0	0	0	0	0	0	0	0
APOC4	0.113	0	0	0	0	0	0	0	0	0	0	0	0	0	0	0
EPF8	0.113	0	0	0	0	0	0	0	0	0	0	0	0	0	0	0
FRA16D	0.113	0	0	0	0	0	0	0	0	0	0	0	0	0	0	0
PPP2R5D	0.113	0	0	0	0	0	0	0	0	0	0	0	0	0	0	0
RADIL	0.113	0	0	0	0	0	0	0	0	0	0	0	0	0	0	0
RANBP9	0.113	0	0	0	0	0	0	0	0	0	0	0	0	0	0	0
RICTOR	0.113	0	0	0	0	0	0	0	0	0	0	0	0	0	0	0
SLC31A1P1	0.113	0	0	0	0	0	0	0	0	0	0	0	0	0	0	0
CD46	0	0	0	0	0	0	0	0	0	0	0	0	0	0	0	0.112
SHROOM4	0	0	0	0	0	0	0	0	0	0	0	0	0	0	0	0.112
ACCN3	0	0	0	0	0	0	0	0	0	0	0	0.112	0	0	0	0
GFRA3	0	0	0	0	0	0	0	0	0	0	0	0.112	0	0	0	0
HAUS3	0	0	0	0	0	0	0	0	0	0	0.112	0	0	0	0	0
MAP3K11	0	0	0	0	0	0	0	0	0	0	0.112	0	0	0	0	0
UNGP1	0	0	0	0	0	0	0	0	0	0	0.112	0	0	0	0	0
ZNF513	0	0	0	0	0	0	0	0	0	0.112	0	0	0	0	0	0
ANAPC16	0.112	0	0	0	0	0	0	0	0	0	0	0	0	0	0	0
ATP5E	0.112	0	0	0	0	0	0	0	0	0	0	0	0	0	0	0
CTH	0.112	0	0	0	0	0	0	0	0	0	0	0	0	0	0	0
ECEL1P1	0.112	0	0	0	0	0	0	0	0	0	0	0	0	0	0	0
MCU	0.112	0	0	0	0	0	0	0	0	0	0	0	0	0	0	0
S100A2	0.112	0	0	0	0	0	0	0	0	0	0	0	0	0	0	0
C8A	0	0	0	0	0	0	0	0	0	0	0	0	0	0	0	0.111
CCDC149	0	0	0	0	0	0	0	0	0	0	0	0	0	0	0.111	0
SPATA18	0	0	0	0	0	0	0	0	0	0	0	0	0	0.111	0	0
PCNXL2	0	0	0	0	0	0	0	0	0	0	0	0.111	0	0	0	0
SLC26A5	0	0	0	0	0	0	0	0	0	0	0	0.111	0	0	0	0
GUCA1A	0	0	0	0	0	0	0	0	0	0.111	0	0	0	0	0	0
GUCY2F	0	0	0	0	0	0	0	0	0	0.111	0	0	0	0	0	0
RBP3	0	0	0	0	0	0	0	0	0	0.111	0	0	0	0	0	0
RGS9BP	0	0	0	0	0	0	0	0	0	0.111	0	0	0	0	0	0
ROM1	0	0	0	0	0	0	0	0	0	0.111	0	0	0	0	0	0
WDR17	0	0	0	0	0	0	0	0	0	0.111	0	0	0	0	0	0
MIR1307	0	0	0	0	0	0	0	0	0.111	0	0	0	0	0	0	0
MIR2110	0	0	0	0	0	0	0	0	0.111	0	0	0	0	0	0	0
EXOSC1	0	0	0	0	0	0	0.111	0	0	0	0	0	0	0	0	0
FXR1	0	0	0.111	0	0	0	0	0	0	0	0	0	0	0	0	0
FAM47A	0	0.111	0	0	0	0	0	0	0	0	0	0	0	0	0	0
GAR1	0	0.111	0	0	0	0	0	0	0	0	0	0	0	0	0	0

RALGAPA2	0	0.111	0	0	0	0	0	0	0	0	0	0	0	0	0	0
TIAM1	0	0.111	0	0	0	0	0	0	0	0	0	0	0	0	0	0
BP33	0.111	0	0	0	0	0	0	0	0	0	0	0	0	0	0	0
BP9	0.111	0	0	0	0	0	0	0	0	0	0	0	0	0	0	0
BPIFB6	0.111	0	0	0	0	0	0	0	0	0	0	0	0	0	0	0
CD151	0.111	0	0	0	0	0	0	0	0	0	0	0	0	0	0	0
EML2	0.111	0	0	0	0	0	0	0	0	0	0	0	0	0	0	0
FRA1M	0.111	0	0	0	0	0	0	0	0	0	0	0	0	0	0	0
HN1	0.111	0	0	0	0	0	0	0	0	0	0	0	0	0	0	0
HPE1	0.111	0	0	0	0	0	0	0	0	0	0	0	0	0	0	0
PFKL	0.111	0	0	0	0	0	0	0	0	0	0	0	0	0	0	0
RPAIN	0.111	0	0	0	0	0	0	0	0	0	0	0	0	0	0	0
SCAR2	0.111	0	0	0	0	0	0	0	0	0	0	0	0	0	0	0
SCGB1D4	0.111	0	0	0	0	0	0	0	0	0	0	0	0	0	0	0
ZNF101	0.111	0	0	0	0	0	0	0	0	0	0	0	0	0	0	0
DNAJB11	0	0	0	0	0	0	0	0	0	0	0	0	0	0.11	0	0
M6PR	0	0	0	0	0	0	0	0	0	0	0	0	0	0.11	0	0
PLAT	0	0	0	0	0	0	0	0	0	0	0	0	0	0.11	0	0
SLC25A27	0	0	0	0	0	0	0	0	0	0	0	0	0	0.11	0	0
MMEDF	0	0	0	0	0	0	0	0	0	0	0	0	0.11	0	0	0
LHFPL3	0	0	0	0	0	0	0	0	0	0	0	0.11	0	0	0	0
LHFPL5	0	0	0	0	0	0	0	0	0	0	0	0.11	0	0	0	0
ND4L	0	0	0	0	0	0	0	0	0	0	0	0.11	0	0	0	0
PIEZO2	0	0	0	0	0	0	0	0	0	0	0	0.11	0	0	0	0
TMIE	0	0	0	0	0	0	0	0	0	0	0	0.11	0	0	0	0
TAF13	0	0	0	0	0	0	0	0	0	0	0.11	0	0	0	0	0
UBE2E1	0	0	0	0	0	0	0	0	0	0	0.11	0	0	0	0	0
NUP62	0	0	0	0	0	0	0	0	0.11	0	0	0	0	0	0	0
POLR2A	0	0	0	0	0	0	0	0	0.11	0	0	0	0	0	0	0
AMMECR1	0	0	0	0	0	0	0.11	0	0	0	0	0	0	0	0	0
UGT2B7	0	0	0	0	0	0	0.11	0	0	0	0	0	0	0	0	0
UQCRB	0	0	0	0	0	0	0.11	0	0	0	0	0	0	0	0	0
ADAMTS14	0	0.11	0	0	0	0	0	0	0	0	0	0	0	0	0	0
CD200	0	0.11	0	0	0	0	0	0	0	0	0	0	0	0	0	0
GALR3	0	0.11	0	0	0	0	0	0	0	0	0	0	0	0	0	0
GCLM	0	0.11	0	0	0	0	0	0	0	0	0	0	0	0	0	0
LGALS3	0	0.11	0	0	0	0	0	0	0	0	0	0	0	0	0	0
NGRN	0	0.11	0	0	0	0	0	0	0	0	0	0	0	0	0	0
OR2H1	0	0.11	0	0	0	0	0	0	0	0	0	0	0	0	0	0
PSORS1C1	0	0.11	0	0	0	0	0	0	0	0	0	0	0	0	0	0
AURKAPS1	0.11	0	0	0	0	0	0	0	0	0	0	0	0	0	0	0

MXRA8	0.11	0	0	0	0	0	0	0	0	0	0	0	0	0	0	0
PRKD3	0.11	0	0	0	0	0	0	0	0	0	0	0	0	0	0	0
PUS3	0.11	0	0	0	0	0	0	0	0	0	0	0	0	0	0	0
RPS9P1	0.11	0	0	0	0	0	0	0	0	0	0	0	0	0	0	0
SORCS2	0.11	0	0	0	0	0	0	0	0	0	0	0	0	0	0	0
STBD1	0.11	0	0	0	0	0	0	0	0	0	0	0	0	0	0	0
TRIM59	0.11	0	0	0	0	0	0	0	0	0	0	0	0	0	0	0
ZCCHC8	0.11	0	0	0	0	0	0	0	0	0	0	0	0	0	0	0
ZNHIT3	0.11	0	0	0	0	0	0	0	0	0	0	0	0	0	0	0
ATRNL1	0	0	0	0	0	0	0	0	0	0	0	0	0	0.109	0	0
CERS2	0	0	0	0	0	0	0	0	0	0	0	0	0	0.109	0	0
COX17P1	0	0	0	0	0	0	0	0	0	0	0	0	0	0.109	0	0
DNAJA2	0	0	0	0	0	0	0	0	0	0	0	0	0	0.109	0	0
DNAJB1	0	0	0	0	0	0	0	0	0	0	0	0	0	0.109	0	0
MANBA	0	0	0	0	0	0	0	0	0	0	0	0	0	0.109	0	0
PNPLA7	0	0	0	0	0	0	0	0	0	0	0	0	0	0.109	0	0
ANKMY2	0	0	0	0	0	0	0	0	0	0	0	0.109	0	0	0	0
PPOX	0	0	0	0	0	0	0	0	0	0	0	0.109	0	0	0	0
TRIOBP	0	0	0	0	0	0	0	0	0	0	0	0.109	0	0	0	0
PDC	0	0	0	0	0	0	0	0	0	0.109	0	0	0	0	0	0
C17ORF42	0	0	0	0	0	0	0.109	0	0	0	0	0	0	0	0	0
DLAT	0	0	0	0	0	0	0.109	0	0	0	0	0	0	0	0	0
UGT1A4	0	0	0	0	0	0	0.109	0	0	0	0	0	0	0	0	0
ABCC1	0	0	0	0	0	0.109	0	0	0	0	0	0	0	0	0	0
KCNK4	0	0	0	0	0	0.109	0	0	0	0	0	0	0	0	0	0
KCNK5	0	0	0	0	0	0.109	0	0	0	0	0	0	0	0	0	0
KCNS3	0	0	0	0	0	0.109	0	0	0	0	0	0	0	0	0	0
DSCAM	0	0	0.109	0	0	0	0	0	0	0	0	0	0	0	0	0
LRRC37B	0	0	0.109	0	0	0	0	0	0	0	0	0	0	0	0	0
FAM69A	0	0.109	0	0	0	0	0	0	0	0	0	0	0	0	0	0
GEMIN7	0	0.109	0	0	0	0	0	0	0	0	0	0	0	0	0	0
GRIP1	0	0.109	0	0	0	0	0	0	0	0	0	0	0	0	0	0
HT	0	0.109	0	0	0	0	0	0	0	0	0	0	0	0	0	0
IGFBP4	0	0.109	0	0	0	0	0	0	0	0	0	0	0	0	0	0
IL26	0	0.109	0	0	0	0	0	0	0	0	0	0	0	0	0	0
KIF3B	0	0.109	0	0	0	0	0	0	0	0	0	0	0	0	0	0
LBX1	0	0.109	0	0	0	0	0	0	0	0	0	0	0	0	0	0
NKX1-2	0	0.109	0	0	0	0	0	0	0	0	0	0	0	0	0	0
P2RX4	0	0.109	0	0	0	0	0	0	0	0	0	0	0	0	0	0
THM	0	0.109	0	0	0	0	0	0	0	0	0	0	0	0	0	0
TNFSF14	0	0.109	0	0	0	0	0	0	0	0	0	0	0	0	0	0

BPIFB2	0.109	0	0	0	0	0	0	0	0	0	0	0	0	0	0	0
BPIFB5P	0.109	0	0	0	0	0	0	0	0	0	0	0	0	0	0	0
BPIFC	0.109	0	0	0	0	0	0	0	0	0	0	0	0	0	0	0
C17ORF89	0.109	0	0	0	0	0	0	0	0	0	0	0	0	0	0	0
CLF	0.109	0	0	0	0	0	0	0	0	0	0	0	0	0	0	0
FRA16A	0.109	0	0	0	0	0	0	0	0	0	0	0	0	0	0	0
FTH1P15	0.109	0	0	0	0	0	0	0	0	0	0	0	0	0	0	0
GNAI2P1	0.109	0	0	0	0	0	0	0	0	0	0	0	0	0	0	0
MAGED4	0.109	0	0	0	0	0	0	0	0	0	0	0	0	0	0	0
PGM5P1	0.109	0	0	0	0	0	0	0	0	0	0	0	0	0	0	0
PHKBP1	0.109	0	0	0	0	0	0	0	0	0	0	0	0	0	0	0
PPIEL	0.109	0	0	0	0	0	0	0	0	0	0	0	0	0	0	0
B3GALT6	0	0	0	0	0	0	0	0	0	0	0	0	0	0	0	0.108
SGPL1	0	0	0	0	0	0	0	0	0	0	0	0	0	0.108	0	0
VPS16	0	0	0	0	0	0	0	0	0	0	0	0	0	0.108	0	0
WDR45L	0	0	0	0	0	0	0	0	0	0	0	0	0	0.108	0	0
GPR171	0	0	0	0	0	0	0	0	0	0	0	0.108	0	0	0	0
HMX2	0	0	0	0	0	0	0	0	0	0	0	0.108	0	0	0	0
LHFPL4	0	0	0	0	0	0	0	0	0	0	0	0.108	0	0	0	0
SLC12A2	0	0	0	0	0	0	0	0	0	0	0	0.108	0	0	0	0
COX16	0	0	0	0	0	0	0.108	0	0	0	0	0	0	0	0	0
COX19	0	0	0	0	0	0	0.108	0	0	0	0	0	0	0	0	0
PDHA1	0	0	0	0	0	0	0.108	0	0	0	0	0	0	0	0	0
RNASEH2C	0	0	0	0	0	0	0.108	0	0	0	0	0	0	0	0	0
RPP30	0	0	0	0	0	0	0.108	0	0	0	0	0	0	0	0	0
RRM2B	0	0	0	0	0	0	0.108	0	0	0	0	0	0	0	0	0
SCO1	0	0	0	0	0	0	0.108	0	0	0	0	0	0	0	0	0
TAZ	0	0	0	0	0	0	0.108	0	0	0	0	0	0	0	0	0
CLCN4	0	0	0	0	0	0.108	0	0	0	0	0	0	0	0	0	0
DLX2	0	0	0	0	0	0.108	0	0	0	0	0	0	0	0	0	0
RPS27P3	0	0	0.108	0	0	0	0	0	0	0	0	0	0	0	0	0
TCERG1L	0	0	0.108	0	0	0	0	0	0	0	0	0	0	0	0	0
ADCYAP1	0	0.108	0	0	0	0	0	0	0	0	0	0	0	0	0	0
CLEC16A	0	0.108	0	0	0	0	0	0	0	0	0	0	0	0	0	0
CSF3	0	0.108	0	0	0	0	0	0	0	0	0	0	0	0	0	0
DYNC2LI1	0	0.108	0	0	0	0	0	0	0	0	0	0	0	0	0	0
PPP4R2	0	0.108	0	0	0	0	0	0	0	0	0	0	0	0	0	0
RPS6	0	0.108	0	0	0	0	0	0	0	0	0	0	0	0	0	0
RUFY3	0	0.108	0	0	0	0	0	0	0	0	0	0	0	0	0	0
SNUPN	0	0.108	0	0	0	0	0	0	0	0	0	0	0	0	0	0
YWHAH	0	0.108	0	0	0	0	0	0	0	0	0	0	0	0	0	0

ZBTB43	0	0.108	0	0	0	0	0	0	0	0	0	0	0	0	0	0
ADCK1	0.108	0	0	0	0	0	0	0	0	0	0	0	0	0	0	0
APOBR	0.108	0	0	0	0	0	0	0	0	0	0	0	0	0	0	0
CD93	0.108	0	0	0	0	0	0	0	0	0	0	0	0	0	0	0
CLNS1AP1	0.108	0	0	0	0	0	0	0	0	0	0	0	0	0	0	0
CYP2T2P	0.108	0	0	0	0	0	0	0	0	0	0	0	0	0	0	0
DEF6	0.108	0	0	0	0	0	0	0	0	0	0	0	0	0	0	0
HMGCL	0.108	0	0	0	0	0	0	0	0	0	0	0	0	0	0	0
S100A9	0.108	0	0	0	0	0	0	0	0	0	0	0	0	0	0	0
TC2N	0.108	0	0	0	0	0	0	0	0	0	0	0	0	0	0	0
ZNF451	0.108	0	0	0	0	0	0	0	0	0	0	0	0	0	0	0
TTC1	0	0	0	0	0	0	0	0	0	0	0	0	0	0	0.107	0
PSORS3	0	0	0	0	0	0	0	0	0.107	0	0	0	0	0	0	0
SRSF4	0	0	0	0	0	0	0	0	0.107	0	0	0	0	0	0	0
FOXRED1	0	0	0	0	0	0	0.107	0	0	0	0	0	0	0	0	0
NDUFA9	0	0	0	0	0	0	0.107	0	0	0	0	0	0	0	0	0
PC	0	0	0	0	0	0	0.107	0	0	0	0	0	0	0	0	0
PET112	0	0	0	0	0	0	0.107	0	0	0	0	0	0	0	0	0
SLC25A6	0	0	0	0	0	0	0.107	0	0	0	0	0	0	0	0	0
FBXO33	0	0	0	0	0	0.107	0	0	0	0	0	0	0	0	0	0
KCNG1	0	0	0	0	0	0.107	0	0	0	0	0	0	0	0	0	0
SSTR2	0	0	0	0	0	0.107	0	0	0	0	0	0	0	0	0	0
SSTR3	0	0	0	0	0	0.107	0	0	0	0	0	0	0	0	0	0
ST7-AS1	0	0	0	0	0	0.107	0	0	0	0	0	0	0	0	0	0
NARF	0	0	0	0.107	0	0	0	0	0	0	0	0	0	0	0	0
C9ORF174	0	0.107	0	0	0	0	0	0	0	0	0	0	0	0	0	0
CD163	0	0.107	0	0	0	0	0	0	0	0	0	0	0	0	0	0
CLCF1	0	0.107	0	0	0	0	0	0	0	0	0	0	0	0	0	0
CMM	0	0.107	0	0	0	0	0	0	0	0	0	0	0	0	0	0
CNN3	0	0.107	0	0	0	0	0	0	0	0	0	0	0	0	0	0
DLL1	0	0.107	0	0	0	0	0	0	0	0	0	0	0	0	0	0
DNAH1	0	0.107	0	0	0	0	0	0	0	0	0	0	0	0	0	0
BATF3	0.107	0	0	0	0	0	0	0	0	0	0	0	0	0	0	0
BHMT	0.107	0	0	0	0	0	0	0	0	0	0	0	0	0	0	0
BPIFB4	0.107	0	0	0	0	0	0	0	0	0	0	0	0	0	0	0
C10ORF88	0.107	0	0	0	0	0	0	0	0	0	0	0	0	0	0	0
C18ORF1	0.107	0	0	0	0	0	0	0	0	0	0	0	0	0	0	0
CLASP1	0.107	0	0	0	0	0	0	0	0	0	0	0	0	0	0	0
CSRNP1	0.107	0	0	0	0	0	0	0	0	0	0	0	0	0	0	0
CYB5R1	0.107	0	0	0	0	0	0	0	0	0	0	0	0	0	0	0
DEAF1	0.107	0	0	0	0	0	0	0	0	0	0	0	0	0	0	0

FBXL14	0.107	0	0	0	0	0	0	0	0	0	0	0	0	0	0	0
FMNL2	0.107	0	0	0	0	0	0	0	0	0	0	0	0	0	0	0
INMT	0.107	0	0	0	0	0	0	0	0	0	0	0	0	0	0	0
NCOA5	0.107	0	0	0	0	0	0	0	0	0	0	0	0	0	0	0
OB10Q	0.107	0	0	0	0	0	0	0	0	0	0	0	0	0	0	0
PAQR6	0.107	0	0	0	0	0	0	0	0	0	0	0	0	0	0	0
PPP1R3E	0.107	0	0	0	0	0	0	0	0	0	0	0	0	0	0	0
SH3BGR	0.107	0	0	0	0	0	0	0	0	0	0	0	0	0	0	0
ALG10B	0	0	0	0	0	0	0	0	0	0	0	0	0	0	0	0.106
ILDR1	0	0	0	0	0	0	0	0	0	0	0	0.106	0	0	0	0
MRGPRE	0	0	0	0	0	0	0	0	0	0	0	0.106	0	0	0	0
UROS	0	0	0	0	0	0	0	0	0	0	0	0.106	0	0	0	0
PDAP1	0	0	0	0	0	0	0	0	0	0	0.106	0	0	0	0	0
CFH	0	0	0	0	0	0	0	0	0	0.106	0	0	0	0	0	0
CYMD	0	0	0	0	0	0	0	0	0	0.106	0	0	0	0	0	0
POMC	0	0	0	0	0	0	0	0	0	0.106	0	0	0	0	0	0
AMT	0	0	0	0	0	0	0.106	0	0	0	0	0	0	0	0	0
BCKDK	0	0	0	0	0	0	0.106	0	0	0	0	0	0	0	0	0
C7	0	0	0	0	0	0	0.106	0	0	0	0	0	0	0	0	0
GLUD2	0	0	0	0	0	0	0.106	0	0	0	0	0	0	0	0	0
OXCT1	0	0	0	0	0	0	0.106	0	0	0	0	0	0	0	0	0
KIAA1468	0	0	0	0	0	0.106	0	0	0	0	0	0	0	0	0	0
A2ML1	0.106	0	0	0	0	0	0	0	0	0	0	0	0	0	0	0
ACRPS	0.106	0	0	0	0	0	0	0	0	0	0	0	0	0	0	0
CENPO	0.106	0	0	0	0	0	0	0	0	0	0	0	0	0	0	0
CHDS1	0.106	0	0	0	0	0	0	0	0	0	0	0	0	0	0	0
CSNK1E	0.106	0	0	0	0	0	0	0	0	0	0	0	0	0	0	0
CYB561D2	0.106	0	0	0	0	0	0	0	0	0	0	0	0	0	0	0
FAM82A1	0.106	0	0	0	0	0	0	0	0	0	0	0	0	0	0	0
PRDM6	0.106	0	0	0	0	0	0	0	0	0	0	0	0	0	0	0
ZBTB12	0.106	0	0	0	0	0	0	0	0	0	0	0	0	0	0	0
ZNF181	0.106	0	0	0	0	0	0	0	0	0	0	0	0	0	0	0
ANO9	0	0	0	0	0	0	0	0	0	0	0	0	0	0	0	0.105
NTRK3	0	0	0	0	0	0	0	0	0	0	0	0.105	0	0	0	0
RIPK2	0	0	0	0	0	0	0	0	0	0	0.105	0	0	0	0	0
TRMEP1	0	0	0	0	0	0	0	0	0	0	0.105	0	0	0	0	0
PALM	0	0	0	0	0	0	0	0	0	0.105	0	0	0	0	0	0
HCCS	0	0	0	0	0	0	0.105	0	0	0	0	0	0	0	0	0
OGDHL	0	0	0	0	0	0	0.105	0	0	0	0	0	0	0	0	0
UQCR10	0	0	0	0	0	0	0.105	0	0	0	0	0	0	0	0	0
ALAD	0.105	0	0	0	0	0	0	0	0	0	0	0	0	0	0	0

BW16	0.105	0	0	0	0	0	0	0	0	0	0	0	0	0	0	0
BW17	0.105	0	0	0	0	0	0	0	0	0	0	0	0	0	0	0
C12ORF52	0.105	0	0	0	0	0	0	0	0	0	0	0	0	0	0	0
CYP7A1	0.105	0	0	0	0	0	0	0	0	0	0	0	0	0	0	0
DAB2	0.105	0	0	0	0	0	0	0	0	0	0	0	0	0	0	0
DCAF7	0.105	0	0	0	0	0	0	0	0	0	0	0	0	0	0	0
EIF4A1P1	0.105	0	0	0	0	0	0	0	0	0	0	0	0	0	0	0
FRA1H	0.105	0	0	0	0	0	0	0	0	0	0	0	0	0	0	0
PDPK1	0.105	0	0	0	0	0	0	0	0	0	0	0	0	0	0	0
PRDM4	0.105	0	0	0	0	0	0	0	0	0	0	0	0	0	0	0
RAC1P2	0.105	0	0	0	0	0	0	0	0	0	0	0	0	0	0	0
S100P	0.105	0	0	0	0	0	0	0	0	0	0	0	0	0	0	0
SLC5A3	0.105	0	0	0	0	0	0	0	0	0	0	0	0	0	0	0
CD59	0	0	0	0	0	0	0	0	0	0	0	0	0	0	0	0.104
SLC2A10	0	0	0	0	0	0	0	0	0	0	0	0	0	0	0	0.104
C17ORF28	0	0	0	0	0	0	0	0	0	0	0	0.104	0	0	0	0
HFE2	0	0	0	0	0	0	0	0	0	0	0	0.104	0	0	0	0
PHOX2B	0	0	0	0	0	0	0	0	0	0	0	0.104	0	0	0	0
WDR35	0	0	0	0	0	0	0	0	0	0	0	0.104	0	0	0	0
ZNF791	0	0	0	0	0	0	0	0	0	0	0	0.104	0	0	0	0
NR2E3	0	0	0	0	0	0	0	0	0	0.104	0	0	0	0	0	0
TRIM4	0	0	0	0	0	0	0	0	0.104	0	0	0	0	0	0	0
MRPL34	0	0	0	0	0	0	0.104	0	0	0	0	0	0	0	0	0
MRPS22	0	0	0	0	0	0	0.104	0	0	0	0	0	0	0	0	0
PDHX	0	0	0	0	0	0	0.104	0	0	0	0	0	0	0	0	0
UGT1A5	0	0	0	0	0	0	0.104	0	0	0	0	0	0	0	0	0
UQCRC1	0	0	0	0	0	0	0.104	0	0	0	0	0	0	0	0	0
PORCN	0	0	0	0	0	0.104	0	0	0	0	0	0	0	0	0	0
LYZ	0	0	0	0	0.104	0	0	0	0	0	0	0	0	0	0	0
TMEM47	0	0	0	0.104	0	0	0	0	0	0	0	0	0	0	0	0
AIDA	0.104	0	0	0	0	0	0	0	0	0	0	0	0	0	0	0
AXIN2	0.104	0	0	0	0	0	0	0	0	0	0	0	0	0	0	0
CHDS4	0.104	0	0	0	0	0	0	0	0	0	0	0	0	0	0	0
DMRTC1	0.104	0	0	0	0	0	0	0	0	0	0	0	0	0	0	0
NCOA3	0.104	0	0	0	0	0	0	0	0	0	0	0	0	0	0	0
PGM2L1	0.104	0	0	0	0	0	0	0	0	0	0	0	0	0	0	0
TMOD2	0.104	0	0	0	0	0	0	0	0	0	0	0	0	0	0	0
TRDN	0	0	0	0	0	0	0	0	0	0	0	0	0	0	0	0.103
CPOX	0	0	0	0	0	0	0	0	0	0	0	0.103	0	0	0	0
KTCN2	0	0	0	0	0	0	0	0	0	0	0	0.103	0	0	0	0
PFN2	0	0	0	0	0	0	0	0	0	0	0.103	0	0	0	0	0

PPP3CA	0	0	0	0	0	0	0	0	0	0	0.103	0	0	0	0	0
TRNAP2	0	0	0	0	0	0	0	0	0	0	0.103	0	0	0	0	0
CAPN3	0	0	0	0	0	0	0	0	0	0.103	0	0	0	0	0	0
CLUL1	0	0	0	0	0	0	0	0	0	0.103	0	0	0	0	0	0
FGFR3	0	0	0	0	0	0	0	0	0	0.103	0	0	0	0	0	0
NDUFV2	0	0	0	0	0	0	0.103	0	0	0	0	0	0	0	0	0
CD4	0	0	0	0	0.103	0	0	0	0	0	0	0	0	0	0	0
ANXA9	0.103	0	0	0	0	0	0	0	0	0	0	0	0	0	0	0
APOA2	0.103	0	0	0	0	0	0	0	0	0	0	0	0	0	0	0
CSNK1G3	0.103	0	0	0	0	0	0	0	0	0	0	0	0	0	0	0
HLA-DPA2	0.103	0	0	0	0	0	0	0	0	0	0	0	0	0	0	0
HSPA14	0.103	0	0	0	0	0	0	0	0	0	0	0	0	0	0	0
MIR1273G	0.103	0	0	0	0	0	0	0	0	0	0	0	0	0	0	0
NFATC3	0.103	0	0	0	0	0	0	0	0	0	0	0	0	0	0	0
PARVB	0.103	0	0	0	0	0	0	0	0	0	0	0	0	0	0	0
RWDD3	0.103	0	0	0	0	0	0	0	0	0	0	0	0	0	0	0
S100A13	0.103	0	0	0	0	0	0	0	0	0	0	0	0	0	0	0
S100A3	0.103	0	0	0	0	0	0	0	0	0	0	0	0	0	0	0
SETD4	0.103	0	0	0	0	0	0	0	0	0	0	0	0	0	0	0
SLC19A1	0.103	0	0	0	0	0	0	0	0	0	0	0	0	0	0	0
TFAP2E	0.103	0	0	0	0	0	0	0	0	0	0	0	0	0	0	0
YBX1P1	0.103	0	0	0	0	0	0	0	0	0	0	0	0	0	0	0
ZC3H12A	0.103	0	0	0	0	0	0	0	0	0	0	0	0	0	0	0
AQP7P2	0	0	0	0	0	0	0	0	0	0	0	0	0	0	0	0.102
STK11	0	0	0	0	0	0	0	0	0	0	0	0	0	0	0	0.102
SBDS	0	0	0	0	0	0	0	0	0	0	0	0	0.102	0	0	0
DFNA5	0	0	0	0	0	0	0	0	0	0	0	0.102	0	0	0	0
FADS6	0	0	0	0	0	0	0	0	0	0	0	0.102	0	0	0	0
FECH	0	0	0	0	0	0	0	0	0	0	0	0.102	0	0	0	0
KIF12	0	0	0	0	0	0	0	0	0	0	0	0.102	0	0	0	0
KRT74	0	0	0	0	0	0	0	0	0	0	0	0.102	0	0	0	0
LIPH	0	0	0	0	0	0	0	0	0	0	0	0.102	0	0	0	0
MYO1F	0	0	0	0	0	0	0	0	0	0	0	0.102	0	0	0	0
EPRS	0	0	0	0	0	0	0	0	0	0	0.102	0	0	0	0	0
RLBP1	0	0	0	0	0	0	0	0	0	0.102	0	0	0	0	0	0
UNC119	0	0	0	0	0	0	0	0	0	0.102	0	0	0	0	0	0
ZCRB1	0	0	0	0	0	0	0	0	0.102	0	0	0	0	0	0	0
ACADVL	0	0	0	0	0	0	0.102	0	0	0	0	0	0	0	0	0
ATPIF1	0	0	0	0	0	0	0.102	0	0	0	0	0	0	0	0	0
CRLS1	0	0	0	0	0	0	0.102	0	0	0	0	0	0	0	0	0
CYTB	0	0	0	0	0	0	0.102	0	0	0	0	0	0	0	0	0

DBT	0	0	0	0	0	0	0.102	0	0	0	0	0	0	0	0	0
A2MP1	0.102	0	0	0	0	0	0	0	0	0	0	0	0	0	0	0
APOO	0.102	0	0	0	0	0	0	0	0	0	0	0	0	0	0	0
ATP5L2	0.102	0	0	0	0	0	0	0	0	0	0	0	0	0	0	0
ATPAF1	0.102	0	0	0	0	0	0	0	0	0	0	0	0	0	0	0
AURKAIP1	0.102	0	0	0	0	0	0	0	0	0	0	0	0	0	0	0
COPD26	0.102	0	0	0	0	0	0	0	0	0	0	0	0	0	0	0
DOCK6	0.102	0	0	0	0	0	0	0	0	0	0	0	0	0	0	0
EOS	0.102	0	0	0	0	0	0	0	0	0	0	0	0	0	0	0
NKD1	0.102	0	0	0	0	0	0	0	0	0	0	0	0	0	0	0
PYGL	0.102	0	0	0	0	0	0	0	0	0	0	0	0	0	0	0
RAC1P4	0.102	0	0	0	0	0	0	0	0	0	0	0	0	0	0	0
RASD1	0.102	0	0	0	0	0	0	0	0	0	0	0	0	0	0	0
THBS4	0.102	0	0	0	0	0	0	0	0	0	0	0	0	0	0	0
TUBGCP5	0.102	0	0	0	0	0	0	0	0	0	0	0	0	0	0	0
ANKS4B	0	0	0	0	0	0	0	0	0	0	0	0.101	0	0	0	0
CTPP	0	0	0	0	0	0	0	0	0	0	0	0.101	0	0	0	0
DFNB26	0	0	0	0	0	0	0	0	0	0	0	0.101	0	0	0	0
HPC5	0	0	0	0	0	0	0	0	0	0	0	0.101	0	0	0	0
KRTAP5-1	0	0	0	0	0	0	0	0	0	0	0	0.101	0	0	0	0
PHOX2A	0	0	0	0	0	0	0	0	0	0	0	0.101	0	0	0	0
HHC3	0	0	0	0	0	0.101	0	0	0	0	0	0	0	0	0	0
ARHGAP31	0.101	0	0	0	0	0	0	0	0	0	0	0	0	0	0	0
ATHS	0.101	0	0	0	0	0	0	0	0	0	0	0	0	0	0	0
C8ORF44-SGK3	0.101	0	0	0	0	0	0	0	0	0	0	0	0	0	0	0
C8ORF73	0.101	0	0	0	0	0	0	0	0	0	0	0	0	0	0	0
CAPZA2	0.101	0	0	0	0	0	0	0	0	0	0	0	0	0	0	0
CARNS1	0.101	0	0	0	0	0	0	0	0	0	0	0	0	0	0	0
COPD25	0.101	0	0	0	0	0	0	0	0	0	0	0	0	0	0	0
NFATC4	0.101	0	0	0	0	0	0	0	0	0	0	0	0	0	0	0
PIAS1	0.101	0	0	0	0	0	0	0	0	0	0	0	0	0	0	0
RIBC1	0.101	0	0	0	0	0	0	0	0	0	0	0	0	0	0	0
RORA	0.101	0	0	0	0	0	0	0	0	0	0	0	0	0	0	0
SBNO2	0.101	0	0	0	0	0	0	0	0	0	0	0	0	0	0	0
TMEM185B	0.101	0	0	0	0	0	0	0	0	0	0	0	0	0	0	0
ACCN1	0	0	0	0	0	0	0	0	0	0	0	0.1	0	0	0	0
IMPG1	0	0	0	0	0	0	0	0	0	0.1	0	0	0	0	0	0
IDSP1	0	0	0	0	0	0	0.1	0	0	0	0	0	0	0	0	0
ZFP57	0	0	0	0.1	0	0	0	0	0	0	0	0	0	0	0	0
ARAF3P	0.1	0	0	0	0	0	0	0	0	0	0	0	0	0	0	0
ARHGDIG	0.1	0	0	0	0	0	0	0	0	0	0	0	0	0	0	0

FGF11	0.1	0	0	0	0	0	0	0	0	0	0	0	0	0	0
NFU1	0.1	0	0	0	0	0	0	0	0	0	0	0	0	0	0
SCARF2	0.1	0	0	0	0	0	0	0	0	0	0	0	0	0	0
