# Supplementary material for: The RXFP3 receptor is functionally associated with cellular responses to oxidative stress and DNA damage
Source: Aging (Albany NY). 2019 Dec 3;11(23):11268–313. doi: 10.18632/aging.102528 (PMC6932917; doi:10.18632/aging.102528)
Supplement: Supplementary Table 26 [file aging-11-102528-s027..pdf]

Table S26. Therapeutic continuum. To identify potential therapeutic targets that could interact and potentially control the 'Disease Continuum' listed in Table S25 in a multidimensional manner we investigated the proteins within this continuum with interrogators most strongly associated with battery of terms linked to central nervous system function, energy metabolism and GPCR-focused signaling (via PubMed Central text mining using GeneIndexer (https://geneindexer.com/): 1) GPCR , 2)gpcr, 3) G protein-coupled receptor, 4) G protein coupled receptor, 5) heptahelical, 6) G protein, 7) receptor, 8) target, 9) therapeutic, 10) serpentine, 11) seven transmembrane, 12) brain, 13) cortex, 14) hippocampus, 15) central nervous system, 16) CNS, 17) diabetes, 18) energy, 19) metabolism, 20) glucose, and 21) mitochondria). Ranking the Cosine Similarity scores of the resultant factors that were prominent in the disease continuum and the 'Therapeutic Interrogators' we found that, based on a correlation ranking probability score ($p<0.001$, ***) there were 37 specific protein targets demonstrating a number of correlations within the therapeutic interrogators that was greater than the 99% percentile (representing only 0.67% of the input 'Disease Continuum' dataset). The top three proteins that could represent effective targets against age-related diseases were, AVPR1B (arginine vasopressin receptor 1B), MAS1 (MAS1 proto-oncogene, G protein-coupled receptor) and RLN3, the cognate ligand for the RXFP3.

Gene Symbol	**1**	**2**	**3**	**4**	**5**	**6**	**7**	**8**	**9**	**10**	**11**	**12**	**13**	**14**	**15**	**16**	**17**	**18**	**19**	**20**	**21**
AVPR1B	0.12	0.12	0.26	0.29	0.12	0.12	0.24	0.13	0.15	0.11	0.11	0.20	0.20	0.18	0.26	0.12	0.14	0.14	0.13	0.16	0.00
MAS1	0.15	0.15	0.24	0.27	0.12	0.11	0.21	0.12	0.21	0.11	0.11	0.18	0.14	0.14	0.28	0.12	0.00	0.11	0.10	0.11	0.00
RLN3	0.14	0.14	0.28	0.32	0.13	0.18	0.26	0.20	0.20	0.00	0.12	0.22	0.20	0.15	0.28	0.10	0.14	0.11	0.10	0.13	0.00
VIPR2	0.22	0.22	0.44	0.47	0.15	0.21	0.40	0.17	0.17	0.13	0.14	0.28	0.23	0.20	0.36	0.14	0.00	0.23	0.11	0.00	0.00
MCHR1	0.21	0.21	0.36	0.37	0.11	0.16	0.31	0.17	0.18	0.00	0.13	0.23	0.20	0.17	0.30	0.11	0.00	0.30	0.14	0.15	0.00
GRK4	0.32	0.32	0.36	0.36	0.24	0.20	0.28	0.17	0.11	0.10	0.12	0.16	0.15	0.00	0.18	0.00	0.12	0.10	0.11	0.11	0.00
NPY5R	0.12	0.12	0.26	0.29	0.00	0.11	0.24	0.14	0.15	0.00	0.10	0.22	0.15	0.21	0.27	0.11	0.11	0.29	0.12	0.14	0.00
GRK5	0.39	0.39	0.36	0.33	0.29	0.17	0.26	0.20	0.21	0.11	0.10	0.16	0.13	0.13	0.20	0.10	0.00	0.11	0.00	0.00	0.00
ADCYAP1R1	0.19	0.19	0.26	0.27	0.15	0.12	0.21	0.12	0.17	0.12	0.12	0.18	0.16	0.18	0.28	0.12	0.00	0.00	0.00	0.11	0.00
ADRBK1	0.41	0.41	0.31	0.24	0.33	0.11	0.17	0.14	0.18	0.00	0.00	0.12	0.13	0.12	0.16	0.11	0.10	0.14	0.00	0.11	0.00
ADCY3	0.13	0.13	0.27	0.34	0.00	0.24	0.24	0.17	0.12	0.00	0.12	0.27	0.23	0.19	0.29	0.13	0.11	0.00	0.10	0.11	0.00
MTNR1A	0.20	0.20	0.33	0.37	0.12	0.15	0.30	0.18	0.20	0.12	0.00	0.23	0.19	0.19	0.27	0.00	0.00	0.11	0.12	0.10	0.00
HRH3	0.20	0.20	0.35	0.35	0.00	0.12	0.30	0.18	0.25	0.00	0.11	0.25	0.26	0.26	0.34	0.18	0.00	0.21	0.11	0.13	0.00
AVPR1A	0.11	0.11	0.24	0.26	0.13	0.00	0.22	0.11	0.15	0.13	0.00	0.15	0.17	0.13	0.22	0.00	0.15	0.11	0.12	0.13	0.00
GPR50	0.15	0.15	0.34	0.42	0.00	0.28	0.34	0.16	0.12	0.00	0.10	0.25	0.16	0.12	0.30	0.00	0.13	0.17	0.19	0.11	0.00
HYOU1	0.00	0.00	0.12	0.25	0.00	0.25	0.16	0.25	0.30	0.00	0.14	0.23	0.17	0.15	0.21	0.10	0.17	0.13	0.15	0.20	0.17
GPR3	0.33	0.33	0.48	0.52	0.20	0.31	0.40	0.19	0.15	0.17	0.21	0.31	0.23	0.17	0.36	0.14	0.00	0.00	0.00	0.00	0.00

RGS10	0.23	0.23	0.34	0.42	0.13	0.32	0.29	0.27	0.17	0.11	0.12	0.29	0.21	0.18	0.26	0.10	0.00	0.00	0.00	0.00	0.00
NTSR1	0.19	0.19	0.36	0.39	0.13	0.17	0.33	0.27	0.29	0.00	0.15	0.23	0.24	0.23	0.31	0.14	0.00	0.15	0.00	0.00	0.00
PTH2	0.20	0.20	0.40	0.46	0.17	0.26	0.37	0.20	0.15	0.00	0.17	0.27	0.21	0.20	0.37	0.17	0.00	0.15	0.00	0.00	0.00
GPR156	0.24	0.24	0.58	0.67	0.18	0.26	0.62	0.25	0.13	0.00	0.27	0.30	0.23	0.22	0.36	0.17	0.00	0.11	0.00	0.00	0.00
TAAR1	0.19	0.19	0.41	0.46	0.11	0.18	0.40	0.24	0.27	0.00	0.18	0.29	0.25	0.21	0.35	0.14	0.00	0.13	0.00	0.00	0.00
HTR4	0.22	0.22	0.32	0.35	0.14	0.14	0.29	0.17	0.22	0.00	0.14	0.25	0.26	0.26	0.30	0.14	0.00	0.12	0.00	0.00	0.00
GALR2	0.19	0.19	0.42	0.46	0.13	0.20	0.39	0.21	0.24	0.00	0.17	0.32	0.26	0.30	0.43	0.22	0.00	0.13	0.00	0.00	0.00
GALR3	0.18	0.18	0.41	0.49	0.11	0.32	0.38	0.18	0.17	0.00	0.19	0.37	0.24	0.26	0.46	0.20	0.00	0.11	0.00	0.00	0.00
GPR85	0.18	0.18	0.38	0.53	0.11	0.43	0.40	0.23	0.13	0.00	0.18	0.55	0.43	0.39	0.56	0.31	0.00	0.00	0.15	0.00	0.00
GPR37	0.16	0.16	0.35	0.46	0.00	0.35	0.35	0.20	0.21	0.00	0.24	0.35	0.25	0.21	0.35	0.16	0.00	0.10	0.11	0.00	0.00
NPS	0.12	0.12	0.34	0.41	0.00	0.23	0.34	0.23	0.22	0.00	0.11	0.30	0.26	0.27	0.39	0.18	0.00	0.19	0.00	0.10	0.00
GRPR	0.15	0.15	0.32	0.35	0.00	0.15	0.30	0.20	0.28	0.00	0.13	0.24	0.17	0.17	0.30	0.13	0.00	0.11	0.00	0.13	0.00
NPFF	0.13	0.13	0.34	0.38	0.00	0.18	0.31	0.18	0.17	0.00	0.12	0.28	0.20	0.20	0.40	0.19	0.00	0.21	0.00	0.11	0.00
GALR1	0.13	0.13	0.32	0.36	0.00	0.14	0.30	0.19	0.20	0.00	0.11	0.26	0.25	0.30	0.35	0.18	0.00	0.15	0.00	0.11	0.00
NTS	0.15	0.15	0.28	0.30	0.00	0.15	0.24	0.24	0.24	0.00	0.00	0.21	0.19	0.17	0.26	0.10	0.00	0.21	0.12	0.12	0.00
SLC16A2	0.00	0.00	0.12	0.21	0.00	0.20	0.14	0.16	0.13	0.16	0.18	0.29	0.22	0.13	0.29	0.12	0.00	0.13	0.24	0.11	0.00
MTNR1B	0.15	0.15	0.28	0.31	0.00	0.13	0.26	0.13	0.14	0.00	0.00	0.19	0.14	0.15	0.22	0.00	0.29	0.14	0.16	0.25	0.00
KCNA3	0.00	0.00	0.11	0.19	0.00	0.15	0.13	0.19	0.20	0.00	0.14	0.19	0.11	0.16	0.23	0.16	0.19	0.15	0.10	0.18	0.00
ABAT	0.00	0.00	0.11	0.22	0.00	0.17	0.16	0.14	0.19	0.00	0.00	0.31	0.28	0.24	0.29	0.17	0.12	0.16	0.29	0.12	0.15
PPIF	0.00	0.00	0.00	0.20	0.00	0.20	0.12	0.19	0.28	0.00	0.13	0.23	0.13	0.16	0.20	0.10	0.12	0.27	0.19	0.17	0.45
MRGPRX3	0.25	0.25	0.50	0.57	0.14	0.34	0.47	0.23	0.15	0.00	0.20	0.27	0.17	0.12	0.40	0.16	0.00	0.00	0.00	0.00	0.00
HTR5A	0.17	0.17	0.30	0.36	0.13	0.18	0.30	0.14	0.14	0.00	0.10	0.26	0.25	0.23	0.32	0.14	0.00	0.00	0.00	0.00	0.00
GPR6	0.27	0.27	0.52	0.61	0.11	0.42	0.48	0.19	0.14	0.00	0.26	0.46	0.32	0.25	0.47	0.24	0.00	0.00	0.00	0.00	0.00
LPAR1	0.24	0.24	0.36	0.35	0.12	0.13	0.29	0.18	0.25	0.00	0.10	0.21	0.19	0.19	0.30	0.23	0.00	0.00	0.00	0.00	0.00
HTR1F	0.23	0.23	0.38	0.42	0.14	0.22	0.35	0.14	0.16	0.00	0.16	0.26	0.20	0.17	0.33	0.14	0.00	0.00	0.00	0.00	0.00
MRGPRE	0.23	0.23	0.50	0.59	0.13	0.35	0.50	0.22	0.16	0.00	0.27	0.32	0.15	0.19	0.45	0.18	0.00	0.00	0.00	0.00	0.00
GABBR2	0.21	0.21	0.36	0.42	0.18	0.20	0.35	0.19	0.13	0.00	0.16	0.31	0.33	0.34	0.36	0.23	0.00	0.00	0.00	0.00	0.00
HTR7	0.20	0.20	0.30	0.33	0.14	0.12	0.27	0.16	0.23	0.00	0.11	0.21	0.23	0.23	0.29	0.14	0.00	0.00	0.00	0.00	0.00
GRM8	0.12	0.12	0.29	0.35	0.13	0.17	0.29	0.17	0.16	0.00	0.11	0.27	0.28	0.29	0.32	0.14	0.00	0.00	0.00	0.00	0.00
GPR56	0.18	0.18	0.31	0.42	0.11	0.35	0.31	0.31	0.22	0.00	0.24	0.31	0.25	0.19	0.36	0.19	0.00	0.00	0.00	0.00	0.00
HTR1D	0.18	0.18	0.29	0.34	0.11	0.16	0.28	0.14	0.15	0.00	0.12	0.22	0.21	0.17	0.32	0.14	0.00	0.00	0.00	0.00	0.00
MRGPRD	0.13	0.13	0.36	0.43	0.12	0.23	0.36	0.24	0.15	0.00	0.20	0.22	0.17	0.16	0.39	0.18	0.00	0.00	0.00	0.00	0.00
GRM4	0.16	0.16	0.31	0.37	0.20	0.18	0.30	0.23	0.23	0.00	0.10	0.24	0.28	0.30	0.30	0.18	0.00	0.00	0.00	0.00	0.00
GRM7	0.12	0.12	0.27	0.33	0.14	0.18	0.26	0.15	0.14	0.00	0.10	0.25	0.28	0.31	0.29	0.15	0.00	0.00	0.00	0.00	0.00
MSANTD3	0.22	0.22	0.47	0.55	0.16	0.23	0.49	0.19	0.15	0.00	0.21	0.33	0.29	0.35	0.38	0.20	0.00	0.00	0.00	0.00	0.00
RXFP2	0.23	0.23	0.40	0.42	0.17	0.19	0.36	0.21	0.18	0.19	0.16	0.17	0.14	0.00	0.26	0.00	0.00	0.13	0.00	0.00	0.00
RASD2	0.17	0.17	0.37	0.45	0.00	0.30	0.35	0.25	0.19	0.16	0.13	0.32	0.23	0.19	0.31	0.00	0.00	0.13	0.00	0.00	0.00
MRGPRX1	0.47	0.47	0.69	0.68	0.28	0.27	0.60	0.19	0.00	0.14	0.25	0.21	0.11	0.00	0.32	0.11	0.00	0.17	0.00	0.00	0.00
PPP1R9B	0.16	0.16	0.27	0.35	0.11	0.26	0.25	0.23	0.10	0.00	0.00	0.32	0.26	0.27	0.30	0.11	0.00	0.12	0.00	0.00	0.00
HRH2	0.20	0.20	0.35	0.35	0.12	0.13	0.31	0.18	0.25	0.00	0.00	0.17	0.14	0.13	0.23	0.13	0.00	0.13	0.00	0.00	0.00

OR5D18	0.19	0.19	0.46	0.58	0.11	0.36	0.48	0.24	0.00	0.00	0.31	0.32	0.18	0.15	0.48	0.20	0.00	0.11	0.00	0.00	0.00
TACR3	0.13	0.13	0.31	0.34	0.00	0.14	0.29	0.12	0.13	0.00	0.11	0.22	0.19	0.18	0.30	0.15	0.00	0.11	0.00	0.00	0.00
OPRL1	0.19	0.19	0.32	0.32	0.00	0.12	0.27	0.12	0.16	0.00	0.13	0.21	0.21	0.21	0.29	0.15	0.00	0.13	0.00	0.00	0.00
OR1B1	0.27	0.27	0.40	0.43	0.16	0.22	0.37	0.11	0.12	0.11	0.15	0.13	0.00	0.00	0.19	0.00	0.10	0.00	0.18	0.00	0.00
KCNJ5	0.11	0.11	0.24	0.27	0.00	0.17	0.20	0.15	0.12	0.00	0.10	0.19	0.14	0.13	0.19	0.00	0.00	0.12	0.11	0.00	0.00
GPR62	0.38	0.38	0.54	0.60	0.20	0.41	0.47	0.00	0.00	0.00	0.26	0.48	0.39	0.33	0.40	0.20	0.00	0.12	0.11	0.00	0.00
VIPR1	0.19	0.19	0.30	0.32	0.15	0.12	0.26	0.16	0.22	0.15	0.15	0.14	0.11	0.00	0.24	0.00	0.00	0.00	0.00	0.11	0.00
CORT	0.10	0.10	0.26	0.33	0.00	0.21	0.25	0.17	0.19	0.00	0.00	0.26	0.24	0.25	0.24	0.14	0.12	0.00	0.00	0.12	0.00
NPY2R	0.13	0.13	0.24	0.25	0.00	0.00	0.22	0.13	0.15	0.00	0.11	0.17	0.14	0.19	0.25	0.11	0.00	0.27	0.00	0.11	0.00
TULP3	0.13	0.13	0.28	0.48	0.00	0.57	0.29	0.22	0.00	0.00	0.17	0.31	0.12	0.00	0.36	0.11	0.11	0.11	0.00	0.10	0.00
PLCB1	0.15	0.15	0.22	0.25	0.14	0.15	0.16	0.19	0.14	0.00	0.00	0.18	0.22	0.21	0.16	0.00	0.00	0.00	0.11	0.14	0.00
SORT1	0.00	0.00	0.23	0.34	0.00	0.22	0.28	0.24	0.21	0.00	0.17	0.19	0.12	0.16	0.24	0.14	0.10	0.00	0.17	0.16	0.00
SLC18A1	0.00	0.00	0.11	0.23	0.00	0.23	0.15	0.13	0.11	0.00	0.15	0.26	0.24	0.16	0.31	0.11	0.10	0.00	0.13	0.11	0.00
HRH1	0.19	0.19	0.31	0.30	0.00	0.00	0.25	0.15	0.23	0.00	0.00	0.18	0.19	0.18	0.27	0.14	0.00	0.21	0.12	0.10	0.00
SLC5A3	0.00	0.00	0.11	0.27	0.00	0.31	0.15	0.21	0.17	0.00	0.16	0.35	0.27	0.21	0.31	0.16	0.00	0.13	0.27	0.22	0.00
KCNK10	0.00	0.00	0.20	0.30	0.00	0.23	0.21	0.19	0.15	0.00	0.16	0.26	0.19	0.19	0.27	0.14	0.00	0.14	0.11	0.14	0.00
BDKRB1	0.18	0.18	0.21	0.21	0.13	0.00	0.17	0.13	0.20	0.00	0.00	0.11	0.00	0.13	0.15	0.00	0.15	0.13	0.10	0.13	0.00
PDE8B	0.00	0.00	0.14	0.24	0.00	0.26	0.15	0.15	0.11	0.12	0.00	0.28	0.20	0.13	0.22	0.00	0.16	0.11	0.21	0.19	0.00
ATRN	0.00	0.00	0.20	0.29	0.00	0.24	0.22	0.14	0.14	0.00	0.16	0.22	0.12	0.00	0.29	0.19	0.13	0.18	0.15	0.12	0.00
APOD	0.00	0.00	0.13	0.23	0.00	0.19	0.16	0.17	0.21	0.00	0.00	0.25	0.22	0.22	0.29	0.20	0.19	0.14	0.25	0.18	0.00
MARK2	0.00	0.00	0.12	0.23	0.00	0.28	0.14	0.27	0.17	0.00	0.00	0.23	0.17	0.13	0.24	0.11	0.14	0.15	0.11	0.19	0.00
VGF	0.00	0.00	0.16	0.27	0.00	0.25	0.17	0.22	0.20	0.00	0.00	0.32	0.25	0.28	0.39	0.21	0.16	0.26	0.16	0.23	0.00
SLC38A2	0.00	0.00	0.00	0.23	0.00	0.27	0.13	0.23	0.12	0.00	0.14	0.25	0.21	0.17	0.27	0.13	0.10	0.20	0.24	0.24	0.00
TSPO	0.00	0.00	0.15	0.26	0.00	0.18	0.20	0.20	0.23	0.00	0.12	0.22	0.23	0.20	0.26	0.16	0.00	0.15	0.18	0.00	0.27
NAT8L	0.00	0.00	0.14	0.35	0.00	0.46	0.18	0.21	0.15	0.00	0.19	0.40	0.24	0.17	0.37	0.19	0.00	0.17	0.33	0.00	0.22
LDHD	0.00	0.00	0.16	0.30	0.00	0.28	0.20	0.14	0.16	0.00	0.17	0.32	0.13	0.00	0.22	0.14	0.00	0.21	0.31	0.16	0.34
ADK	0.00	0.00	0.13	0.25	0.00	0.23	0.15	0.22	0.24	0.00	0.00	0.28	0.23	0.22	0.23	0.00	0.14	0.19	0.26	0.20	0.12
SLC25A27	0.00	0.00	0.00	0.21	0.00	0.26	0.11	0.17	0.14	0.00	0.12	0.23	0.14	0.11	0.21	0.00	0.17	0.32	0.27	0.19	0.37
SPTLC1	0.00	0.00	0.11	0.24	0.00	0.30	0.13	0.20	0.21	0.00	0.13	0.20	0.00	0.00	0.28	0.17	0.11	0.12	0.33	0.14	0.10
TAAR7P	0.22	0.22	0.52	0.58	0.12	0.25	0.52	0.13	0.12	0.00	0.22	0.25	0.17	0.14	0.29	0.00	0.00	0.00	0.00	0.00	0.00
TAAR8	0.22	0.22	0.52	0.58	0.12	0.25	0.52	0.13	0.12	0.00	0.22	0.25	0.17	0.14	0.29	0.00	0.00	0.00	0.00	0.00	0.00
GNAL	0.23	0.23	0.37	0.43	0.17	0.28	0.31	0.21	0.14	0.00	0.13	0.26	0.21	0.15	0.29	0.00	0.00	0.00	0.00	0.00	0.00
GABBR1	0.15	0.15	0.24	0.26	0.15	0.10	0.21	0.14	0.13	0.00	0.00	0.21	0.29	0.30	0.28	0.21	0.00	0.00	0.00	0.00	0.00
PLCB4	0.14	0.14	0.23	0.29	0.13	0.20	0.20	0.18	0.10	0.00	0.00	0.26	0.30	0.29	0.29	0.16	0.00	0.00	0.00	0.00	0.00
GRM2	0.15	0.15	0.25	0.28	0.20	0.13	0.22	0.17	0.24	0.00	0.00	0.19	0.25	0.31	0.25	0.15	0.00	0.00	0.00	0.00	0.00
RGS9	0.23	0.23	0.29	0.35	0.11	0.26	0.24	0.20	0.14	0.00	0.00	0.26	0.22	0.18	0.29	0.11	0.00	0.00	0.00	0.00	0.00
VN2R1P	0.17	0.17	0.45	0.56	0.14	0.32	0.46	0.18	0.00	0.00	0.27	0.31	0.22	0.18	0.45	0.19	0.00	0.00	0.00	0.00	0.00
GPR151	0.33	0.33	0.61	0.70	0.17	0.45	0.57	0.15	0.00	0.00	0.29	0.50	0.29	0.24	0.58	0.28	0.00	0.00	0.00	0.00	0.00
KCTD16	0.17	0.17	0.44	0.53	0.12	0.27	0.47	0.14	0.00	0.00	0.23	0.48	0.36	0.38	0.47	0.28	0.00	0.00	0.00	0.00	0.00
OR9A2	0.22	0.22	0.48	0.63	0.14	0.49	0.49	0.19	0.00	0.00	0.27	0.41	0.21	0.19	0.48	0.25	0.00	0.00	0.00	0.00	0.00

KCTD12	0.13	0.13	0.34	0.48	0.00	0.40	0.37	0.17	0.12	0.00	0.16	0.37	0.22	0.22	0.34	0.15	0.00	0.00	0.00	0.00	0.00
KCNJ9	0.11	0.11	0.25	0.30	0.00	0.18	0.23	0.15	0.11	0.00	0.13	0.25	0.23	0.22	0.29	0.15	0.00	0.00	0.00	0.00	0.00
GRM6	0.11	0.11	0.25	0.34	0.00	0.21	0.26	0.15	0.13	0.00	0.11	0.18	0.14	0.13	0.27	0.11	0.00	0.00	0.00	0.00	0.00
CHRM5	0.21	0.21	0.28	0.30	0.00	0.14	0.24	0.15	0.17	0.00	0.11	0.17	0.22	0.21	0.26	0.12	0.00	0.00	0.00	0.00	0.00
LPHN3	0.14	0.14	0.37	0.48	0.00	0.33	0.38	0.19	0.14	0.00	0.22	0.40	0.28	0.29	0.42	0.17	0.00	0.00	0.00	0.00	0.00
OR51E2	0.13	0.13	0.33	0.48	0.00	0.38	0.36	0.32	0.20	0.00	0.19	0.27	0.15	0.12	0.35	0.15	0.00	0.00	0.00	0.00	0.00
AVPR2	0.18	0.18	0.26	0.28	0.15	0.12	0.23	0.13	0.16	0.12	0.15	0.00	0.13	0.00	0.15	0.00	0.14	0.00	0.00	0.00	0.00
RGS2	0.24	0.24	0.26	0.27	0.16	0.14	0.19	0.20	0.16	0.00	0.00	0.13	0.17	0.11	0.16	0.00	0.11	0.00	0.00	0.00	0.00
SSTR2	0.15	0.15	0.22	0.21	0.10	0.00	0.17	0.12	0.19	0.00	0.00	0.14	0.18	0.20	0.12	0.10	0.11	0.00	0.00	0.00	0.00
SSTR4	0.15	0.15	0.27	0.27	0.00	0.11	0.22	0.11	0.18	0.00	0.00	0.19	0.21	0.23	0.15	0.11	0.11	0.00	0.00	0.00	0.00
GRK6	0.44	0.44	0.42	0.38	0.32	0.20	0.29	0.20	0.20	0.15	0.15	0.14	0.00	0.00	0.16	0.00	0.00	0.14	0.00	0.00	0.00
ADRBK2	0.36	0.36	0.35	0.32	0.29	0.16	0.25	0.16	0.16	0.00	0.00	0.16	0.15	0.13	0.21	0.00	0.00	0.11	0.00	0.00	0.00
RGS14	0.26	0.26	0.33	0.39	0.16	0.34	0.25	0.23	0.00	0.12	0.00	0.24	0.18	0.16	0.22	0.00	0.00	0.12	0.00	0.00	0.00
ATRNL1	0.20	0.20	0.32	0.43	0.10	0.37	0.32	0.15	0.00	0.14	0.23	0.27	0.00	0.00	0.35	0.16	0.00	0.21	0.00	0.00	0.00
CHRM4	0.21	0.21	0.24	0.26	0.00	0.12	0.21	0.17	0.16	0.00	0.00	0.19	0.24	0.24	0.29	0.16	0.00	0.11	0.00	0.00	0.00
PNOC	0.10	0.10	0.23	0.26	0.00	0.11	0.21	0.14	0.17	0.00	0.00	0.20	0.21	0.22	0.29	0.15	0.00	0.15	0.00	0.00	0.00
ZMYND19	0.11	0.11	0.33	0.53	0.00	0.57	0.35	0.18	0.00	0.00	0.16	0.59	0.41	0.35	0.52	0.23	0.00	0.13	0.00	0.00	0.00
DGKB	0.11	0.11	0.27	0.42	0.00	0.40	0.29	0.24	0.10	0.00	0.00	0.41	0.31	0.31	0.34	0.16	0.00	0.00	0.16	0.00	0.00
HTR2C	0.15	0.15	0.16	0.17	0.17	0.00	0.15	0.11	0.16	0.00	0.00	0.12	0.14	0.13	0.17	0.00	0.00	0.15	0.10	0.00	0.00
SRR	0.00	0.00	0.20	0.34	0.00	0.23	0.26	0.19	0.19	0.00	0.10	0.39	0.34	0.35	0.38	0.23	0.00	0.11	0.23	0.00	0.00
SLC5A7	0.00	0.00	0.12	0.25	0.00	0.23	0.17	0.16	0.15	0.00	0.20	0.30	0.26	0.23	0.36	0.17	0.00	0.11	0.12	0.00	0.00
TRPM3	0.00	0.00	0.24	0.37	0.00	0.29	0.27	0.22	0.15	0.00	0.19	0.25	0.11	0.14	0.28	0.15	0.11	0.00	0.00	0.17	0.00
PTPRS	0.00	0.00	0.19	0.31	0.00	0.27	0.25	0.22	0.13	0.00	0.20	0.29	0.19	0.20	0.38	0.23	0.12	0.00	0.00	0.12	0.00
CAPN5	0.00	0.00	0.16	0.31	0.00	0.34	0.20	0.21	0.15	0.00	0.10	0.28	0.16	0.14	0.31	0.14	0.17	0.00	0.00	0.11	0.00
KCNK9	0.00	0.00	0.12	0.22	0.00	0.18	0.15	0.20	0.19	0.00	0.16	0.27	0.25	0.23	0.33	0.21	0.00	0.12	0.00	0.11	0.00
MZB1	0.00	0.00	0.20	0.29	0.00	0.24	0.20	0.17	0.21	0.00	0.13	0.19	0.12	0.15	0.27	0.11	0.00	0.00	0.10	0.16	0.00
SLC30A3	0.00	0.00	0.10	0.22	0.00	0.21	0.15	0.17	0.17	0.00	0.13	0.36	0.35	0.44	0.32	0.22	0.00	0.00	0.11	0.12	0.00
SGPP1	0.17	0.17	0.30	0.34	0.00	0.27	0.24	0.22	0.20	0.00	0.16	0.13	0.00	0.00	0.13	0.00	0.10	0.00	0.21	0.13	0.00
SORCS1	0.00	0.00	0.21	0.37	0.00	0.36	0.27	0.16	0.11	0.00	0.17	0.30	0.14	0.17	0.28	0.00	0.33	0.00	0.16	0.26	0.00
FRK	0.00	0.00	0.18	0.36	0.00	0.38	0.24	0.29	0.17	0.00	0.12	0.21	0.10	0.12	0.22	0.00	0.17	0.00	0.10	0.18	0.00
ZDHHC17	0.00	0.00	0.13	0.30	0.00	0.37	0.18	0.23	0.19	0.00	0.13	0.29	0.13	0.13	0.23	0.00	0.14	0.00	0.11	0.12	0.00
TOR2A	0.00	0.00	0.26	0.39	0.00	0.33	0.29	0.18	0.19	0.00	0.13	0.25	0.14	0.00	0.29	0.12	0.17	0.00	0.14	0.12	0.00
PTGDS	0.00	0.00	0.10	0.18	0.00	0.13	0.13	0.17	0.18	0.00	0.00	0.17	0.14	0.10	0.20	0.12	0.12	0.00	0.16	0.12	0.00
MGRN1	0.16	0.16	0.26	0.34	0.00	0.25	0.26	0.18	0.00	0.11	0.14	0.17	0.00	0.00	0.23	0.00	0.00	0.21	0.12	0.11	0.00
TDO2	0.00	0.00	0.13	0.26	0.00	0.24	0.17	0.22	0.20	0.00	0.00	0.28	0.21	0.18	0.29	0.12	0.00	0.10	0.29	0.10	0.00
MGLL	0.00	0.00	0.17	0.26	0.00	0.20	0.19	0.26	0.22	0.00	0.00	0.23	0.17	0.18	0.26	0.14	0.00	0.18	0.26	0.10	0.00
CAMKK2	0.00	0.00	0.17	0.28	0.00	0.25	0.20	0.31	0.14	0.00	0.00	0.27	0.21	0.24	0.27	0.14	0.00	0.21	0.14	0.15	0.00
HDC	0.00	0.00	0.16	0.22	0.00	0.12	0.17	0.14	0.20	0.00	0.00	0.18	0.17	0.14	0.24	0.14	0.00	0.15	0.18	0.10	0.00
SLC1A4	0.00	0.00	0.11	0.27	0.00	0.28	0.16	0.15	0.00	0.00	0.22	0.35	0.31	0.25	0.35	0.18	0.00	0.17	0.21	0.18	0.00
RTN2	0.00	0.00	0.16	0.39	0.00	0.52	0.22	0.19	0.00	0.00	0.27	0.36	0.14	0.19	0.36	0.16	0.00	0.11	0.11	0.16	0.00

SLC16A7	0.00	0.00	0.00	0.21	0.00	0.21	0.12	0.18	0.11	0.00	0.16	0.34	0.29	0.25	0.28	0.17	0.00	0.28	0.26	0.28	0.00
SLC7A11	0.00	0.00	0.00	0.23	0.00	0.23	0.14	0.24	0.27	0.00	0.14	0.26	0.21	0.21	0.27	0.15	0.00	0.10	0.16	0.13	0.00
SLC38A1	0.00	0.00	0.00	0.22	0.00	0.26	0.11	0.16	0.12	0.00	0.16	0.35	0.31	0.24	0.30	0.17	0.00	0.15	0.25	0.18	0.00
GLP1R	0.12	0.12	0.26	0.27	0.00	0.00	0.23	0.16	0.27	0.00	0.14	0.13	0.00	0.00	0.20	0.00	0.23	0.24	0.15	0.28	0.00
TPST2	0.00	0.00	0.21	0.32	0.00	0.28	0.24	0.18	0.13	0.14	0.16	0.20	0.00	0.00	0.22	0.00	0.14	0.11	0.15	0.18	0.00
ENSA	0.00	0.00	0.20	0.35	0.00	0.34	0.24	0.21	0.14	0.00	0.00	0.30	0.19	0.18	0.27	0.00	0.27	0.14	0.19	0.28	0.00
PNMT	0.00	0.00	0.11	0.19	0.00	0.14	0.13	0.15	0.12	0.00	0.00	0.16	0.15	0.13	0.26	0.00	0.13	0.14	0.15	0.12	0.00
NUCB2	0.00	0.00	0.18	0.25	0.00	0.21	0.18	0.18	0.15	0.00	0.00	0.21	0.12	0.10	0.25	0.00	0.19	0.30	0.16	0.25	0.00
KLF7	0.00	0.00	0.14	0.30	0.00	0.35	0.19	0.35	0.00	0.00	0.00	0.28	0.23	0.18	0.43	0.20	0.24	0.12	0.12	0.24	0.00
FAAH	0.00	0.00	0.12	0.17	0.00	0.00	0.13	0.16	0.21	0.00	0.00	0.16	0.15	0.16	0.22	0.13	0.11	0.14	0.19	0.12	0.00
SLC45A1	0.00	0.00	0.00	0.34	0.00	0.57	0.14	0.19	0.00	0.00	0.20	0.42	0.22	0.14	0.35	0.15	0.13	0.12	0.14	0.24	0.00
CERS1	0.00	0.00	0.12	0.32	0.00	0.50	0.15	0.25	0.16	0.00	0.13	0.33	0.15	0.12	0.29	0.16	0.00	0.00	0.26	0.00	0.12
RTN3	0.00	0.00	0.12	0.29	0.00	0.37	0.17	0.24	0.21	0.00	0.21	0.35	0.22	0.25	0.34	0.20	0.00	0.00	0.11	0.00	0.19
PEX5	0.00	0.00	0.15	0.29	0.00	0.32	0.19	0.15	0.10	0.00	0.14	0.23	0.13	0.00	0.28	0.13	0.00	0.20	0.29	0.00	0.28
ABCD2	0.00	0.00	0.13	0.29	0.00	0.36	0.17	0.18	0.13	0.00	0.16	0.26	0.14	0.00	0.28	0.12	0.00	0.14	0.31	0.00	0.18
TTPA	0.00	0.00	0.10	0.23	0.00	0.23	0.15	0.15	0.21	0.00	0.00	0.23	0.14	0.12	0.23	0.12	0.00	0.11	0.29	0.00	0.10
ALDH5A1	0.00	0.00	0.11	0.20	0.00	0.16	0.14	0.12	0.18	0.00	0.00	0.33	0.29	0.25	0.30	0.23	0.00	0.16	0.31	0.00	0.20
KYNU	0.00	0.00	0.12	0.25	0.00	0.27	0.15	0.10	0.14	0.00	0.00	0.30	0.20	0.13	0.25	0.11	0.00	0.12	0.34	0.00	0.12
KMO	0.00	0.00	0.11	0.26	0.00	0.30	0.15	0.16	0.20	0.00	0.00	0.37	0.27	0.20	0.28	0.13	0.00	0.11	0.35	0.00	0.16
DDO	0.00	0.00	0.15	0.31	0.00	0.33	0.19	0.16	0.17	0.00	0.00	0.41	0.32	0.31	0.34	0.16	0.00	0.15	0.29	0.00	0.16
CCBL1	0.00	0.00	0.12	0.27	0.00	0.31	0.15	0.13	0.20	0.00	0.00	0.37	0.22	0.17	0.27	0.15	0.00	0.17	0.37	0.00	0.21
AADAT	0.00	0.00	0.17	0.30	0.00	0.24	0.22	0.14	0.24	0.00	0.00	0.39	0.31	0.32	0.33	0.21	0.00	0.13	0.28	0.00	0.11
PHYH	0.00	0.00	0.14	0.31	0.00	0.38	0.18	0.16	0.13	0.00	0.00	0.30	0.15	0.12	0.30	0.13	0.00	0.14	0.33	0.00	0.19
PREP	0.00	0.00	0.12	0.24	0.00	0.27	0.13	0.22	0.17	0.00	0.00	0.34	0.29	0.25	0.31	0.14	0.00	0.15	0.20	0.00	0.12
RIMKLA	0.00	0.00	0.17	0.38	0.00	0.52	0.18	0.14	0.00	0.00	0.11	0.43	0.22	0.18	0.44	0.25	0.00	0.11	0.26	0.00	0.12
GABARAPL1	0.00	0.00	0.26	0.42	0.00	0.37	0.31	0.27	0.00	0.00	0.18	0.26	0.14	0.11	0.29	0.10	0.00	0.14	0.14	0.00	0.18
NMNAT2	0.00	0.00	0.00	0.29	0.00	0.47	0.11	0.22	0.12	0.00	0.11	0.37	0.16	0.13	0.30	0.13	0.00	0.15	0.32	0.00	0.20
ACSL4	0.00	0.00	0.13	0.26	0.00	0.29	0.16	0.19	0.14	0.00	0.00	0.27	0.17	0.10	0.17	0.00	0.00	0.21	0.38	0.13	0.21
PPP3R2	0.00	0.00	0.13	0.27	0.00	0.29	0.18	0.27	0.22	0.00	0.00	0.24	0.15	0.21	0.19	0.00	0.00	0.16	0.15	0.13	0.17
MRS2	0.00	0.00	0.00	0.29	0.00	0.52	0.10	0.26	0.17	0.00	0.18	0.28	0.10	0.00	0.26	0.17	0.00	0.18	0.26	0.13	0.43
SFXN1	0.00	0.00	0.00	0.21	0.00	0.31	0.10	0.17	0.10	0.00	0.00	0.25	0.12	0.11	0.25	0.11	0.00	0.15	0.32	0.10	0.19
CKB	0.00	0.00	0.00	0.19	0.00	0.22	0.10	0.17	0.17	0.00	0.00	0.30	0.20	0.16	0.28	0.14	0.00	0.24	0.25	0.11	0.18
GLS	0.00	0.00	0.00	0.23	0.00	0.24	0.14	0.18	0.16	0.00	0.00	0.32	0.22	0.16	0.29	0.17	0.00	0.18	0.29	0.14	0.19
GLUL	0.00	0.00	0.00	0.17	0.00	0.16	0.11	0.18	0.19	0.00	0.00	0.28	0.27	0.24	0.29	0.24	0.00	0.17	0.24	0.16	0.10
SLC6A8	0.00	0.00	0.00	0.19	0.00	0.24	0.00	0.13	0.15	0.00	0.14	0.33	0.27	0.22	0.30	0.16	0.00	0.22	0.25	0.14	0.11
PID1	0.00	0.00	0.15	0.33	0.00	0.39	0.21	0.25	0.14	0.00	0.10	0.18	0.00	0.00	0.14	0.00	0.26	0.25	0.27	0.38	0.14
PDIA2	0.00	0.00	0.12	0.25	0.00	0.26	0.16	0.25	0.18	0.00	0.16	0.12	0.00	0.00	0.15	0.00	0.16	0.14	0.11	0.17	0.12
SERP1	0.00	0.00	0.18	0.34	0.00	0.39	0.21	0.23	0.15	0.00	0.22	0.15	0.00	0.00	0.20	0.00	0.21	0.11	0.13	0.21	0.12
PITRM1	0.00	0.00	0.00	0.28	0.00	0.43	0.12	0.22	0.11	0.00	0.00	0.31	0.11	0.13	0.22	0.00	0.18	0.20	0.26	0.21	0.21
GLUD1	0.00	0.00	0.00	0.21	0.00	0.22	0.13	0.14	0.12	0.00	0.00	0.27	0.17	0.14	0.21	0.00	0.15	0.26	0.32	0.22	0.23

AARS	0.00	0.00	0.10	0.26	0.00	0.37	0.13	0.19	0.14	0.00	0.00	0.20	0.00	0.00	0.28	0.12	0.12	0.22	0.26	0.15	0.18
ACSS2	0.00	0.00	0.00	0.25	0.00	0.41	0.10	0.30	0.14	0.00	0.00	0.30	0.13	0.00	0.24	0.12	0.10	0.23	0.42	0.17	0.20
SLC25A12	0.00	0.00	0.00	0.23	0.00	0.29	0.12	0.10	0.00	0.00	0.00	0.38	0.28	0.24	0.33	0.19	0.14	0.22	0.28	0.19	0.28
AIFM1	0.00	0.00	0.00	0.15	0.00	0.14	0.00	0.20	0.23	0.00	0.00	0.15	0.11	0.13	0.17	0.10	0.10	0.16	0.15	0.12	0.35
RGS4	0.23	0.23	0.24	0.26	0.13	0.15	0.17	0.18	0.15	0.00	0.00	0.20	0.25	0.17	0.20	0.00	0.00	0.00	0.00	0.00	0.00
HTR6	0.14	0.14	0.24	0.28	0.12	0.12	0.24	0.13	0.21	0.00	0.00	0.20	0.22	0.21	0.22	0.00	0.00	0.00	0.00	0.00	0.00
HOMER2	0.10	0.10	0.27	0.37	0.12	0.25	0.29	0.20	0.15	0.00	0.00	0.24	0.24	0.27	0.27	0.00	0.00	0.00	0.00	0.00	0.00
HOMER3	0.12	0.12	0.32	0.43	0.14	0.29	0.33	0.21	0.00	0.00	0.12	0.26	0.21	0.26	0.28	0.00	0.00	0.00	0.00	0.00	0.00
ZDHHC3	0.16	0.16	0.39	0.57	0.12	0.43	0.44	0.20	0.00	0.00	0.30	0.30	0.19	0.18	0.27	0.00	0.00	0.00	0.00	0.00	0.00
NPSR1	0.11	0.11	0.28	0.32	0.00	0.17	0.28	0.14	0.18	0.00	0.13	0.16	0.13	0.12	0.24	0.00	0.00	0.00	0.00	0.00	0.00
GPRASP1	0.23	0.23	0.37	0.46	0.00	0.30	0.36	0.25	0.22	0.00	0.15	0.25	0.18	0.16	0.27	0.00	0.00	0.00	0.00	0.00	0.00
CRCP	0.20	0.20	0.34	0.36	0.00	0.18	0.30	0.16	0.16	0.00	0.17	0.19	0.13	0.00	0.31	0.13	0.00	0.00	0.00	0.00	0.00
C5AR1	0.16	0.16	0.21	0.22	0.00	0.10	0.18	0.14	0.21	0.00	0.11	0.11	0.11	0.00	0.19	0.11	0.00	0.00	0.00	0.00	0.00
GPR88	0.15	0.15	0.30	0.37	0.00	0.21	0.30	0.18	0.18	0.00	0.00	0.31	0.30	0.27	0.30	0.15	0.00	0.00	0.00	0.00	0.00
ADCY1	0.14	0.14	0.25	0.30	0.00	0.17	0.23	0.19	0.14	0.00	0.00	0.28	0.33	0.33	0.32	0.20	0.00	0.00	0.00	0.00	0.00
GPR63	0.39	0.39	0.57	0.60	0.19	0.41	0.46	0.00	0.00	0.00	0.22	0.43	0.31	0.25	0.39	0.19	0.00	0.00	0.00	0.00	0.00
OR10H3	0.14	0.14	0.49	0.63	0.00	0.32	0.56	0.29	0.00	0.00	0.21	0.35	0.25	0.22	0.50	0.25	0.00	0.00	0.00	0.00	0.00
GPR155	0.17	0.17	0.36	0.56	0.00	0.56	0.38	0.12	0.00	0.00	0.26	0.54	0.35	0.29	0.51	0.25	0.00	0.00	0.00	0.00	0.00
CC2D1B	0.10	0.10	0.26	0.47	0.00	0.48	0.32	0.37	0.00	0.00	0.13	0.33	0.19	0.18	0.34	0.14	0.00	0.00	0.00	0.00	0.00
GNG12	0.12	0.12	0.29	0.47	0.00	0.45	0.31	0.17	0.00	0.00	0.11	0.36	0.20	0.18	0.31	0.11	0.00	0.00	0.00	0.00	0.00
NALCN	0.11	0.11	0.29	0.40	0.00	0.32	0.29	0.13	0.00	0.00	0.22	0.31	0.19	0.20	0.36	0.13	0.00	0.00	0.00	0.00	0.00
OR2M3	0.11	0.11	0.37	0.57	0.00	0.50	0.42	0.18	0.00	0.00	0.22	0.31	0.16	0.13	0.36	0.13	0.00	0.00	0.00	0.00	0.00
KCNJ3	0.15	0.15	0.27	0.30	0.00	0.16	0.22	0.15	0.00	0.00	0.12	0.22	0.19	0.18	0.23	0.11	0.00	0.00	0.00	0.00	0.00
CALY	0.10	0.10	0.27	0.39	0.00	0.29	0.30	0.15	0.00	0.00	0.12	0.36	0.31	0.31	0.33	0.13	0.00	0.00	0.00	0.00	0.00
OR1J1	0.14	0.14	0.53	0.65	0.00	0.29	0.60	0.24	0.00	0.00	0.28	0.29	0.16	0.15	0.44	0.20	0.00	0.00	0.00	0.00	0.00
KCTD8	0.13	0.13	0.34	0.47	0.00	0.33	0.39	0.16	0.00	0.00	0.24	0.40	0.26	0.28	0.42	0.23	0.00	0.00	0.00	0.00	0.00
GRIK1	0.00	0.00	0.22	0.30	0.10	0.12	0.25	0.12	0.15	0.00	0.15	0.19	0.23	0.30	0.27	0.17	0.00	0.00	0.00	0.00	0.00
PROK2	0.12	0.12	0.30	0.34	0.00	0.18	0.27	0.20	0.22	0.00	0.11	0.20	0.00	0.00	0.33	0.11	0.00	0.16	0.00	0.00	0.00
NPY1R	0.11	0.11	0.22	0.25	0.00	0.00	0.20	0.13	0.14	0.00	0.00	0.18	0.16	0.22	0.26	0.12	0.00	0.24	0.00	0.00	0.00
SLC6A1	0.00	0.00	0.11	0.20	0.00	0.13	0.15	0.17	0.16	0.00	0.19	0.21	0.29	0.30	0.27	0.19	0.00	0.13	0.00	0.00	0.00
SLC18A3	0.00	0.00	0.12	0.22	0.00	0.18	0.15	0.17	0.14	0.00	0.13	0.26	0.28	0.25	0.38	0.20	0.00	0.10	0.00	0.00	0.00
GLRA1	0.00	0.00	0.17	0.27	0.00	0.11	0.24	0.12	0.12	0.00	0.20	0.13	0.11	0.12	0.25	0.15	0.00	0.11	0.00	0.00	0.00
ASIC3	0.00	0.00	0.17	0.23	0.00	0.13	0.19	0.18	0.20	0.00	0.17	0.15	0.10	0.12	0.27	0.13	0.00	0.10	0.00	0.00	0.00
GPR132	0.16	0.16	0.36	0.43	0.11	0.23	0.35	0.28	0.25	0.00	0.13	0.13	0.00	0.00	0.18	0.00	0.00	0.00	0.16	0.00	0.00
NAALAD2	0.00	0.00	0.15	0.30	0.00	0.33	0.18	0.13	0.14	0.00	0.14	0.36	0.21	0.21	0.31	0.17	0.00	0.00	0.27	0.00	0.00
CA8	0.00	0.00	0.14	0.32	0.00	0.42	0.18	0.21	0.10	0.00	0.13	0.41	0.29	0.25	0.38	0.24	0.00	0.00	0.13	0.00	0.00
SORL1	0.00	0.00	0.17	0.27	0.00	0.21	0.21	0.18	0.20	0.00	0.14	0.26	0.19	0.19	0.26	0.14	0.00	0.00	0.15	0.00	0.00
TOR1A	0.00	0.00	0.11	0.20	0.00	0.20	0.13	0.17	0.20	0.00	0.16	0.25	0.24	0.18	0.29	0.16	0.00	0.00	0.11	0.00	0.00
MMEL1	0.00	0.00	0.13	0.26	0.00	0.30	0.15	0.14	0.15	0.00	0.16	0.29	0.19	0.16	0.26	0.11	0.00	0.00	0.14	0.00	0.00
CCDC22	0.00	0.00	0.12	0.33	0.00	0.51	0.15	0.11	0.12	0.00	0.15	0.47	0.27	0.21	0.49	0.27	0.00	0.00	0.10	0.00	0.00

CLSTN2	0.00	0.00	0.12	0.23	0.00	0.25	0.16	0.13	0.11	0.00	0.12	0.37	0.25	0.30	0.29	0.15	0.00	0.00	0.14	0.00	0.00
APLP1	0.00	0.00	0.16	0.30	0.00	0.30	0.21	0.17	0.11	0.00	0.19	0.29	0.17	0.18	0.29	0.11	0.00	0.00	0.12	0.00	0.00
APBA2	0.00	0.00	0.16	0.29	0.00	0.27	0.20	0.20	0.15	0.00	0.16	0.32	0.18	0.24	0.30	0.12	0.00	0.00	0.10	0.00	0.00
LRP8	0.00	0.00	0.17	0.26	0.00	0.16	0.22	0.14	0.11	0.00	0.11	0.21	0.22	0.21	0.24	0.13	0.00	0.00	0.10	0.00	0.00
CNP	0.00	0.00	0.11	0.21	0.00	0.19	0.15	0.18	0.12	0.00	0.11	0.27	0.27	0.18	0.43	0.40	0.00	0.00	0.11	0.00	0.00
APLP2	0.00	0.00	0.12	0.24	0.00	0.23	0.17	0.19	0.14	0.00	0.17	0.25	0.16	0.16	0.26	0.11	0.00	0.00	0.13	0.00	0.00
SIGMAR1	0.00	0.00	0.26	0.35	0.00	0.20	0.28	0.20	0.25	0.00	0.15	0.29	0.28	0.32	0.34	0.21	0.00	0.00	0.12	0.00	0.00
SLC6A9	0.00	0.00	0.16	0.28	0.00	0.18	0.22	0.18	0.17	0.00	0.18	0.32	0.35	0.34	0.38	0.23	0.00	0.00	0.11	0.00	0.00
PNPLA6	0.00	0.00	0.11	0.25	0.00	0.30	0.13	0.23	0.23	0.00	0.13	0.30	0.21	0.16	0.37	0.23	0.00	0.00	0.21	0.00	0.00
APBA1	0.00	0.00	0.19	0.30	0.00	0.24	0.23	0.20	0.11	0.00	0.18	0.30	0.14	0.21	0.28	0.11	0.00	0.00	0.11	0.00	0.00
LPPR4	0.00	0.00	0.20	0.35	0.00	0.36	0.24	0.25	0.10	0.00	0.12	0.44	0.40	0.46	0.42	0.26	0.00	0.00	0.11	0.00	0.00
THOP1	0.00	0.00	0.19	0.31	0.00	0.31	0.20	0.22	0.14	0.00	0.13	0.32	0.21	0.21	0.27	0.00	0.00	0.12	0.17	0.00	0.00
ADARB1	0.00	0.00	0.13	0.25	0.00	0.21	0.17	0.24	0.21	0.00	0.00	0.19	0.12	0.14	0.26	0.12	0.00	0.11	0.15	0.00	0.00
PDE4B	0.00	0.00	0.15	0.24	0.00	0.19	0.17	0.20	0.22	0.00	0.00	0.32	0.30	0.26	0.29	0.16	0.00	0.12	0.12	0.00	0.00
PENK	0.00	0.00	0.13	0.20	0.00	0.15	0.15	0.15	0.14	0.00	0.00	0.19	0.20	0.18	0.25	0.11	0.00	0.13	0.11	0.00	0.00
MAP6	0.00	0.00	0.12	0.26	0.00	0.27	0.16	0.20	0.15	0.00	0.00	0.41	0.38	0.36	0.43	0.24	0.00	0.11	0.12	0.00	0.00
IMPA1	0.00	0.00	0.13	0.27	0.00	0.32	0.15	0.22	0.18	0.00	0.00	0.35	0.26	0.23	0.32	0.15	0.00	0.13	0.24	0.00	0.00
VLDLR	0.00	0.00	0.14	0.22	0.00	0.13	0.19	0.17	0.16	0.00	0.00	0.17	0.18	0.15	0.20	0.12	0.00	0.12	0.21	0.00	0.00
SLC36A2	0.00	0.00	0.11	0.31	0.00	0.43	0.16	0.13	0.00	0.00	0.31	0.30	0.20	0.11	0.34	0.16	0.00	0.10	0.18	0.00	0.00
SLC7A10	0.00	0.00	0.17	0.36	0.00	0.38	0.24	0.14	0.00	0.00	0.28	0.39	0.31	0.26	0.40	0.21	0.00	0.12	0.15	0.00	0.00
SLC1A1	0.00	0.00	0.00	0.18	0.00	0.13	0.12	0.14	0.13	0.00	0.14	0.26	0.30	0.30	0.28	0.19	0.00	0.13	0.14	0.00	0.00
ADCYAP1	0.12	0.12	0.15	0.16	0.11	0.00	0.11	0.00	0.14	0.00	0.00	0.12	0.13	0.15	0.23	0.12	0.00	0.00	0.00	0.14	0.00
CACNA1E	0.00	0.00	0.14	0.20	0.00	0.13	0.14	0.14	0.12	0.00	0.10	0.21	0.14	0.24	0.23	0.14	0.00	0.00	0.00	0.15	0.00
SHC3	0.00	0.00	0.24	0.35	0.00	0.25	0.29	0.26	0.18	0.00	0.14	0.25	0.14	0.22	0.32	0.14	0.00	0.00	0.00	0.12	0.00
KCNN1	0.00	0.00	0.13	0.24	0.00	0.27	0.13	0.14	0.13	0.00	0.15	0.30	0.15	0.25	0.31	0.17	0.00	0.00	0.00	0.11	0.00
SCGN	0.00	0.00	0.12	0.25	0.00	0.36	0.12	0.21	0.11	0.00	0.00	0.33	0.28	0.27	0.28	0.13	0.20	0.00	0.00	0.21	0.00
CHGB	0.00	0.00	0.11	0.22	0.00	0.23	0.13	0.15	0.12	0.00	0.00	0.25	0.20	0.21	0.28	0.11	0.14	0.00	0.00	0.14	0.00
NNAT	0.00	0.00	0.11	0.25	0.00	0.30	0.15	0.23	0.12	0.00	0.00	0.33	0.15	0.17	0.36	0.20	0.13	0.00	0.00	0.15	0.00
MOK	0.00	0.00	0.16	0.28	0.00	0.22	0.22	0.27	0.32	0.00	0.00	0.19	0.11	0.13	0.23	0.11	0.19	0.00	0.00	0.12	0.00
MAPK8IP1	0.00	0.00	0.13	0.26	0.00	0.25	0.18	0.24	0.16	0.00	0.00	0.25	0.16	0.21	0.26	0.10	0.13	0.00	0.00	0.16	0.00
EXOC4	0.00	0.00	0.17	0.34	0.00	0.36	0.22	0.21	0.00	0.00	0.12	0.25	0.12	0.12	0.29	0.12	0.17	0.00	0.00	0.22	0.00
ADORA1	0.10	0.10	0.16	0.16	0.00	0.00	0.12	0.11	0.14	0.00	0.00	0.00	0.16	0.15	0.12	0.00	0.10	0.10	0.00	0.10	0.00
UNC13A	0.00	0.00	0.13	0.24	0.00	0.20	0.17	0.25	0.00	0.00	0.00	0.21	0.14	0.20	0.26	0.11	0.12	0.11	0.00	0.18	0.00
GAL	0.00	0.00	0.16	0.19	0.00	0.00	0.15	0.13	0.16	0.00	0.00	0.18	0.20	0.24	0.29	0.16	0.12	0.16	0.00	0.14	0.00
ARPP19	0.00	0.00	0.19	0.38	0.00	0.46	0.22	0.28	0.13	0.00	0.00	0.41	0.29	0.26	0.36	0.13	0.00	0.00	0.14	0.12	0.00
FLOT1	0.00	0.00	0.20	0.30	0.00	0.26	0.22	0.20	0.00	0.00	0.16	0.24	0.17	0.15	0.28	0.12	0.00	0.00	0.13	0.14	0.00
LNPEP	0.00	0.00	0.14	0.23	0.00	0.17	0.17	0.18	0.13	0.00	0.00	0.20	0.18	0.18	0.21	0.00	0.14	0.00	0.15	0.20	0.00
NEU3	0.00	0.00	0.12	0.26	0.00	0.25	0.18	0.29	0.27	0.00	0.13	0.19	0.00	0.00	0.20	0.12	0.12	0.00	0.15	0.19	0.00
SLC5A11	0.00	0.00	0.00	0.27	0.00	0.38	0.14	0.00	0.15	0.00	0.22	0.29	0.21	0.17	0.25	0.12	0.10	0.00	0.13	0.13	0.00
SLC36A1	0.00	0.00	0.10	0.26	0.00	0.33	0.13	0.18	0.12	0.00	0.28	0.22	0.17	0.00	0.20	0.00	0.00	0.14	0.20	0.16	0.00

EEF2K	0.00	0.00	0.11	0.27	0.00	0.35	0.14	0.36	0.19	0.00	0.00	0.22	0.11	0.15	0.19	0.00	0.00	0.16	0.11	0.12	0.00
PITPNA	0.00	0.00	0.23	0.37	0.00	0.37	0.25	0.19	0.00	0.00	0.15	0.25	0.11	0.00	0.27	0.10	0.00	0.14	0.25	0.13	0.00
CAMKK1	0.00	0.00	0.14	0.26	0.00	0.27	0.18	0.28	0.00	0.00	0.00	0.26	0.19	0.23	0.25	0.12	0.00	0.14	0.13	0.13	0.00
BSX	0.00	0.00	0.12	0.28	0.00	0.37	0.14	0.29	0.00	0.00	0.00	0.31	0.15	0.11	0.35	0.14	0.00	0.32	0.20	0.12	0.00
TRH	0.00	0.00	0.17	0.22	0.00	0.14	0.16	0.17	0.00	0.00	0.00	0.21	0.19	0.18	0.27	0.12	0.00	0.20	0.14	0.16	0.00
PCSK1N	0.00	0.00	0.00	0.20	0.00	0.23	0.11	0.14	0.11	0.00	0.00	0.32	0.22	0.22	0.30	0.16	0.00	0.14	0.14	0.12	0.00
TSC1	0.00	0.00	0.00	0.14	0.00	0.10	0.11	0.25	0.22	0.00	0.00	0.15	0.13	0.11	0.19	0.13	0.00	0.18	0.13	0.11	0.00
FABP3	0.00	0.00	0.10	0.19	0.00	0.18	0.13	0.16	0.16	0.00	0.00	0.18	0.12	0.00	0.18	0.00	0.19	0.27	0.25	0.22	0.00
PHLPP1	0.00	0.00	0.11	0.26	0.00	0.30	0.15	0.41	0.27	0.00	0.00	0.18	0.00	0.11	0.15	0.00	0.13	0.12	0.15	0.20	0.00
PIK3C3	0.00	0.00	0.19	0.25	0.00	0.17	0.19	0.23	0.17	0.00	0.00	0.12	0.00	0.11	0.15	0.00	0.10	0.15	0.15	0.19	0.00
PCMT1	0.00	0.00	0.00	0.24	0.00	0.28	0.13	0.24	0.18	0.00	0.00	0.26	0.17	0.17	0.21	0.00	0.11	0.12	0.25	0.12	0.00
IDE	0.00	0.00	0.00	0.16	0.00	0.14	0.10	0.15	0.16	0.00	0.00	0.18	0.12	0.12	0.14	0.00	0.26	0.15	0.21	0.22	0.00
EIF2B1	0.00	0.00	0.00	0.20	0.00	0.28	0.10	0.18	0.10	0.00	0.00	0.19	0.10	0.10	0.22	0.00	0.15	0.15	0.13	0.17	0.00
GLO1	0.00	0.00	0.00	0.19	0.00	0.15	0.14	0.17	0.22	0.00	0.00	0.19	0.13	0.11	0.16	0.00	0.21	0.10	0.22	0.17	0.00
SPTLC2	0.00	0.00	0.10	0.25	0.00	0.36	0.12	0.19	0.17	0.00	0.00	0.19	0.00	0.00	0.21	0.11	0.18	0.12	0.36	0.20	0.00
CARTPT	0.00	0.00	0.13	0.16	0.00	0.00	0.12	0.12	0.00	0.00	0.00	0.18	0.19	0.18	0.27	0.14	0.12	0.26	0.13	0.15	0.00
SLC2A3	0.00	0.00	0.00	0.13	0.00	0.15	0.00	0.16	0.11	0.00	0.00	0.25	0.20	0.18	0.23	0.14	0.19	0.20	0.23	0.38	0.00
RTN4IP1	0.00	0.00	0.17	0.34	0.00	0.38	0.23	0.18	0.13	0.00	0.21	0.40	0.24	0.22	0.50	0.43	0.00	0.00	0.00	0.00	0.31
SDIM1	0.00	0.00	0.19	0.41	0.00	0.53	0.24	0.29	0.21	0.00	0.16	0.49	0.27	0.30	0.39	0.21	0.00	0.00	0.00	0.00	0.13
NPTX2	0.00	0.00	0.20	0.37	0.00	0.31	0.27	0.24	0.19	0.00	0.14	0.38	0.32	0.36	0.40	0.23	0.00	0.00	0.00	0.00	0.13
NPTX1	0.00	0.00	0.17	0.35	0.00	0.31	0.25	0.26	0.19	0.00	0.15	0.38	0.30	0.37	0.40	0.23	0.00	0.00	0.00	0.00	0.16
RTN1	0.00	0.00	0.15	0.35	0.00	0.46	0.19	0.27	0.16	0.00	0.23	0.39	0.23	0.21	0.41	0.21	0.00	0.00	0.00	0.00	0.11
SPNS1	0.00	0.00	0.11	0.27	0.00	0.39	0.15	0.17	0.13	0.00	0.18	0.28	0.14	0.16	0.32	0.20	0.00	0.00	0.00	0.00	0.33
KIF1B	0.00	0.00	0.10	0.27	0.00	0.31	0.16	0.20	0.11	0.00	0.13	0.32	0.17	0.13	0.38	0.22	0.00	0.00	0.00	0.00	0.12
NPTXR	0.00	0.00	0.21	0.43	0.00	0.42	0.30	0.27	0.18	0.00	0.18	0.43	0.31	0.33	0.44	0.26	0.00	0.00	0.00	0.00	0.15
TRAK1	0.00	0.00	0.17	0.41	0.00	0.51	0.25	0.27	0.11	0.00	0.14	0.28	0.12	0.00	0.29	0.12	0.00	0.11	0.00	0.00	0.28
SNPH	0.00	0.00	0.13	0.29	0.00	0.29	0.20	0.23	0.10	0.00	0.00	0.34	0.20	0.22	0.38	0.21	0.00	0.17	0.00	0.00	0.30
TUBA1A	0.00	0.00	0.11	0.21	0.00	0.20	0.13	0.18	0.15	0.00	0.00	0.28	0.23	0.16	0.30	0.15	0.00	0.11	0.00	0.00	0.16
NEUROD6	0.00	0.00	0.12	0.22	0.00	0.19	0.15	0.24	0.13	0.00	0.00	0.31	0.38	0.35	0.44	0.31	0.00	0.10	0.00	0.00	0.10
TRAK2	0.00	0.00	0.23	0.46	0.00	0.50	0.31	0.18	0.00	0.00	0.17	0.34	0.15	0.12	0.33	0.13	0.00	0.13	0.00	0.00	0.25
KIF1A	0.00	0.00	0.11	0.29	0.00	0.38	0.16	0.17	0.00	0.00	0.12	0.29	0.15	0.12	0.36	0.14	0.00	0.13	0.00	0.00	0.13
SPNS2	0.11	0.11	0.22	0.29	0.00	0.30	0.18	0.14	0.11	0.00	0.16	0.13	0.00	0.00	0.15	0.00	0.00	0.00	0.14	0.00	0.23
CHCHD10	0.00	0.00	0.28	0.45	0.00	0.36	0.35	0.28	0.17	0.00	0.11	0.20	0.16	0.12	0.19	0.00	0.00	0.00	0.26	0.00	0.22
CERS2	0.00	0.00	0.13	0.28	0.00	0.38	0.14	0.29	0.22	0.00	0.12	0.25	0.11	0.00	0.23	0.15	0.00	0.00	0.32	0.00	0.12
DAO	0.00	0.00	0.12	0.23	0.00	0.18	0.17	0.11	0.16	0.00	0.00	0.26	0.27	0.23	0.25	0.11	0.00	0.00	0.20	0.00	0.11
PLA2G3	0.00	0.00	0.11	0.26	0.00	0.30	0.16	0.25	0.21	0.00	0.00	0.25	0.15	0.13	0.24	0.13	0.00	0.00	0.19	0.00	0.13
SERINC4	0.00	0.00	0.11	0.34	0.00	0.58	0.14	0.11	0.00	0.00	0.32	0.44	0.25	0.24	0.32	0.21	0.00	0.00	0.28	0.00	0.11
ACSM4	0.00	0.00	0.24	0.46	0.00	0.57	0.26	0.16	0.00	0.00	0.14	0.39	0.20	0.16	0.38	0.16	0.00	0.00	0.19	0.00	0.13
EFHD1	0.00	0.00	0.12	0.37	0.00	0.61	0.16	0.22	0.00	0.00	0.14	0.55	0.32	0.27	0.45	0.25	0.00	0.00	0.11	0.00	0.33
NIPSNAP1	0.00	0.00	0.15	0.38	0.00	0.53	0.20	0.18	0.00	0.00	0.15	0.44	0.18	0.20	0.37	0.17	0.00	0.00	0.19	0.00	0.20

ABCA2	0.00	0.00	0.00	0.25	0.00	0.32	0.14	0.18	0.15	0.00	0.19	0.33	0.21	0.19	0.33	0.26	0.00	0.00	0.27	0.00	0.12
PPT1	0.00	0.00	0.00	0.19	0.00	0.22	0.12	0.17	0.27	0.00	0.11	0.36	0.27	0.26	0.35	0.29	0.00	0.00	0.17	0.00	0.12
CLN3	0.00	0.00	0.00	0.22	0.00	0.24	0.14	0.16	0.23	0.00	0.15	0.32	0.25	0.23	0.35	0.26	0.00	0.00	0.15	0.00	0.11
STARD3	0.00	0.00	0.13	0.31	0.00	0.39	0.18	0.23	0.11	0.00	0.18	0.19	0.11	0.00	0.18	0.00	0.00	0.15	0.24	0.00	0.21
PPID	0.00	0.00	0.12	0.26	0.00	0.24	0.17	0.22	0.25	0.00	0.13	0.21	0.00	0.12	0.22	0.00	0.00	0.19	0.15	0.00	0.42
GABARAP	0.00	0.00	0.22	0.36	0.00	0.29	0.28	0.26	0.00	0.00	0.16	0.22	0.16	0.14	0.26	0.00	0.00	0.13	0.10	0.00	0.14
LRPPRC	0.00	0.00	0.15	0.37	0.00	0.44	0.22	0.28	0.13	0.00	0.00	0.24	0.10	0.00	0.27	0.11	0.00	0.20	0.23	0.00	0.31
PEX2	0.00	0.00	0.00	0.25	0.00	0.35	0.13	0.17	0.10	0.00	0.14	0.25	0.14	0.00	0.26	0.11	0.00	0.11	0.28	0.00	0.21
CCS	0.00	0.00	0.00	0.21	0.00	0.25	0.11	0.18	0.17	0.00	0.00	0.21	0.12	0.13	0.23	0.12	0.00	0.11	0.19	0.00	0.15
QPRT	0.00	0.00	0.00	0.24	0.00	0.38	0.10	0.14	0.12	0.00	0.00	0.38	0.26	0.23	0.26	0.15	0.00	0.14	0.39	0.00	0.13
NMNAT1	0.00	0.00	0.00	0.25	0.00	0.33	0.13	0.27	0.25	0.00	0.00	0.29	0.14	0.14	0.34	0.19	0.00	0.15	0.27	0.00	0.18
ACSBG1	0.00	0.00	0.00	0.28	0.00	0.43	0.13	0.15	0.00	0.00	0.12	0.35	0.21	0.12	0.29	0.15	0.00	0.14	0.36	0.00	0.20
CASP12	0.00	0.00	0.00	0.18	0.00	0.17	0.11	0.17	0.24	0.00	0.00	0.16	0.14	0.17	0.18	0.12	0.15	0.00	0.00	0.13	0.17
MAP3K15	0.00	0.00	0.12	0.35	0.00	0.55	0.17	0.29	0.22	0.00	0.00	0.32	0.12	0.14	0.17	0.00	0.00	0.00	0.17	0.12	0.17
SGMS1	0.00	0.00	0.14	0.31	0.00	0.37	0.18	0.29	0.20	0.00	0.12	0.19	0.00	0.00	0.15	0.00	0.15	0.00	0.32	0.17	0.14
TMBIM6	0.00	0.00	0.14	0.28	0.00	0.30	0.17	0.32	0.29	0.00	0.17	0.18	0.00	0.00	0.16	0.00	0.00	0.15	0.16	0.16	0.21
CERS6	0.00	0.00	0.13	0.30	0.00	0.42	0.15	0.34	0.24	0.00	0.16	0.21	0.00	0.00	0.15	0.00	0.00	0.10	0.24	0.10	0.24
UCHL3	0.00	0.00	0.00	0.26	0.00	0.38	0.12	0.28	0.15	0.00	0.00	0.26	0.16	0.16	0.27	0.00	0.00	0.19	0.19	0.14	0.16
GLS2	0.00	0.00	0.00	0.27	0.00	0.36	0.13	0.28	0.18	0.00	0.00	0.32	0.17	0.14	0.23	0.00	0.00	0.17	0.31	0.14	0.24
SLC25A14	0.00	0.00	0.00	0.22	0.00	0.33	0.00	0.13	0.12	0.00	0.12	0.33	0.20	0.16	0.26	0.00	0.00	0.31	0.29	0.12	0.38
COX11	0.00	0.00	0.00	0.30	0.00	0.49	0.12	0.18	0.00	0.00	0.00	0.31	0.13	0.11	0.31	0.12	0.00	0.20	0.25	0.13	0.37
THEM4	0.00	0.00	0.10	0.30	0.00	0.42	0.16	0.39	0.21	0.00	0.00	0.22	0.00	0.00	0.17	0.00	0.13	0.12	0.16	0.23	0.23
ATP5J	0.00	0.00	0.12	0.25	0.00	0.30	0.14	0.15	0.14	0.00	0.00	0.20	0.00	0.00	0.18	0.00	0.15	0.17	0.21	0.11	0.27
SLC29A3	0.00	0.00	0.00	0.23	0.00	0.34	0.11	0.12	0.13	0.00	0.21	0.18	0.00	0.00	0.19	0.00	0.22	0.12	0.21	0.20	0.11
HMGCS1	0.00	0.00	0.00	0.21	0.00	0.27	0.12	0.23	0.13	0.00	0.00	0.22	0.11	0.00	0.17	0.00	0.12	0.20	0.45	0.16	0.18
PLA2G6	0.00	0.00	0.00	0.14	0.00	0.11	0.00	0.15	0.17	0.00	0.00	0.15	0.13	0.11	0.13	0.00	0.15	0.11	0.19	0.14	0.12
MSRA	0.00	0.00	0.00	0.19	0.00	0.25	0.00	0.24	0.24	0.00	0.00	0.24	0.16	0.13	0.22	0.00	0.14	0.18	0.25	0.13	0.21
TXN2	0.00	0.00	0.00	0.15	0.00	0.19	0.00	0.20	0.21	0.00	0.00	0.16	0.12	0.11	0.17	0.00	0.15	0.11	0.17	0.16	0.22
GLRX	0.00	0.00	0.00	0.15	0.00	0.20	0.00	0.18	0.19	0.00	0.00	0.17	0.13	0.11	0.16	0.00	0.11	0.11	0.18	0.15	0.16
RGSL1	0.18	0.18	0.25	0.37	0.11	0.38	0.21	0.28	0.16	0.00	0.12	0.20	0.00	0.00	0.19	0.00	0.00	0.00	0.00	0.00	0.00
GIT1	0.14	0.14	0.25	0.31	0.00	0.23	0.24	0.21	0.13	0.00	0.00	0.20	0.13	0.13	0.22	0.00	0.00	0.00	0.00	0.00	0.00
DRD5	0.14	0.14	0.22	0.23	0.00	0.10	0.19	0.11	0.16	0.00	0.00	0.17	0.23	0.20	0.21	0.00	0.00	0.00	0.00	0.00	0.00
GNG7	0.17	0.17	0.32	0.46	0.00	0.41	0.32	0.27	0.16	0.00	0.00	0.31	0.18	0.17	0.26	0.00	0.00	0.00	0.00	0.00	0.00
OXTR	0.13	0.13	0.24	0.23	0.15	0.00	0.20	0.00	0.11	0.18	0.00	0.12	0.11	0.12	0.18	0.00	0.00	0.00	0.00	0.00	0.00
OPN5	0.22	0.22	0.33	0.43	0.00	0.37	0.31	0.13	0.00	0.00	0.14	0.26	0.15	0.13	0.30	0.00	0.00	0.00	0.00	0.00	0.00
NSF	0.11	0.11	0.25	0.35	0.00	0.22	0.28	0.22	0.00	0.00	0.18	0.18	0.12	0.18	0.23	0.00	0.00	0.00	0.00	0.00	0.00
GPR15	0.12	0.12	0.30	0.35	0.00	0.21	0.30	0.21	0.15	0.00	0.17	0.11	0.00	0.00	0.18	0.11	0.00	0.00	0.00	0.00	0.00
MRGPRX4	0.13	0.13	0.40	0.48	0.00	0.29	0.40	0.24	0.18	0.00	0.18	0.19	0.00	0.00	0.35	0.14	0.00	0.00	0.00	0.00	0.00
GPR126	0.23	0.23	0.42	0.56	0.00	0.47	0.41	0.20	0.14	0.00	0.32	0.26	0.00	0.00	0.42	0.19	0.00	0.00	0.00	0.00	0.00
GRM1	0.13	0.13	0.19	0.20	0.21	0.00	0.14	0.13	0.16	0.00	0.00	0.00	0.17	0.22	0.14	0.11	0.00	0.00	0.00	0.00	0.00

HTR1B	0.13	0.13	0.16	0.18	0.00	0.00	0.15	0.10	0.16	0.00	0.00	0.11	0.18	0.18	0.19	0.10	0.00	0.00	0.00	0.00	0.00
CNR2	0.15	0.15	0.17	0.19	0.00	0.00	0.15	0.14	0.22	0.00	0.00	0.14	0.12	0.15	0.20	0.15	0.00	0.00	0.00	0.00	0.00
HOMER1	0.00	0.00	0.22	0.29	0.11	0.15	0.23	0.16	0.17	0.00	0.00	0.19	0.24	0.29	0.22	0.10	0.00	0.00	0.00	0.00	0.00
GRM3	0.00	0.00	0.20	0.26	0.15	0.12	0.19	0.13	0.20	0.00	0.00	0.21	0.28	0.31	0.25	0.15	0.00	0.00	0.00	0.00	0.00
CACNA1H	0.00	0.00	0.13	0.20	0.00	0.12	0.14	0.18	0.19	0.00	0.11	0.17	0.11	0.17	0.21	0.13	0.00	0.00	0.00	0.00	0.00
NEU1	0.00	0.00	0.13	0.22	0.00	0.18	0.18	0.13	0.20	0.00	0.13	0.19	0.14	0.12	0.25	0.15	0.00	0.00	0.00	0.00	0.00
SEMA3C	0.00	0.00	0.10	0.20	0.00	0.15	0.16	0.22	0.16	0.00	0.12	0.18	0.18	0.13	0.33	0.19	0.00	0.00	0.00	0.00	0.00
CDKL2	0.00	0.00	0.25	0.43	0.00	0.39	0.32	0.24	0.13	0.00	0.12	0.35	0.25	0.27	0.34	0.15	0.00	0.00	0.00	0.00	0.00
SLITRK1	0.00	0.00	0.15	0.31	0.00	0.36	0.19	0.17	0.14	0.00	0.15	0.31	0.20	0.14	0.41	0.20	0.00	0.00	0.00	0.00	0.00
RTN4	0.00	0.00	0.11	0.19	0.00	0.16	0.14	0.19	0.25	0.00	0.15	0.27	0.28	0.29	0.41	0.37	0.00	0.00	0.00	0.00	0.00
PSPN	0.00	0.00	0.24	0.35	0.00	0.22	0.30	0.20	0.18	0.00	0.13	0.27	0.21	0.19	0.41	0.20	0.00	0.00	0.00	0.00	0.00
EPHB1	0.00	0.00	0.17	0.24	0.00	0.12	0.22	0.22	0.17	0.00	0.16	0.18	0.20	0.21	0.28	0.19	0.00	0.00	0.00	0.00	0.00
SCYL1	0.00	0.00	0.14	0.34	0.00	0.44	0.20	0.25	0.17	0.00	0.12	0.24	0.12	0.12	0.32	0.15	0.00	0.00	0.00	0.00	0.00
MANF	0.00	0.00	0.13	0.27	0.00	0.32	0.16	0.25	0.27	0.00	0.14	0.30	0.25	0.26	0.33	0.20	0.00	0.00	0.00	0.00	0.00
GULP1	0.00	0.00	0.29	0.51	0.00	0.49	0.37	0.27	0.13	0.00	0.19	0.31	0.13	0.16	0.31	0.12	0.00	0.00	0.00	0.00	0.00
GRIA3	0.00	0.00	0.13	0.22	0.00	0.12	0.19	0.11	0.13	0.00	0.14	0.15	0.17	0.23	0.22	0.15	0.00	0.00	0.00	0.00	0.00
NUMB	0.00	0.00	0.16	0.27	0.00	0.22	0.19	0.33	0.16	0.00	0.11	0.27	0.20	0.15	0.34	0.19	0.00	0.00	0.00	0.00	0.00
GRIK5	0.00	0.00	0.24	0.34	0.00	0.18	0.29	0.11	0.10	0.00	0.17	0.27	0.25	0.31	0.32	0.19	0.00	0.00	0.00	0.00	0.00
NIPA1	0.00	0.00	0.14	0.28	0.00	0.30	0.18	0.12	0.15	0.00	0.20	0.25	0.14	0.11	0.33	0.15	0.00	0.00	0.00	0.00	0.00
PICK1	0.00	0.00	0.24	0.36	0.00	0.22	0.30	0.22	0.13	0.00	0.20	0.25	0.21	0.25	0.30	0.15	0.00	0.00	0.00	0.00	0.00
ASIC1	0.00	0.00	0.15	0.22	0.00	0.13	0.17	0.19	0.22	0.00	0.18	0.23	0.19	0.23	0.31	0.19	0.00	0.00	0.00	0.00	0.00
KCNK5	0.00	0.00	0.12	0.25	0.00	0.22	0.17	0.22	0.22	0.00	0.22	0.20	0.15	0.13	0.22	0.13	0.00	0.00	0.00	0.00	0.00
LPPR2	0.00	0.00	0.24	0.39	0.00	0.34	0.30	0.24	0.10	0.00	0.10	0.51	0.42	0.49	0.50	0.32	0.00	0.00	0.00	0.00	0.00
GFRA1	0.00	0.00	0.15	0.21	0.00	0.10	0.18	0.17	0.16	0.00	0.10	0.12	0.14	0.12	0.28	0.12	0.00	0.00	0.00	0.00	0.00
LINGO1	0.00	0.00	0.18	0.30	0.00	0.24	0.24	0.21	0.21	0.00	0.15	0.29	0.23	0.20	0.46	0.37	0.00	0.00	0.00	0.00	0.00
D4S234E	0.00	0.00	0.26	0.45	0.00	0.40	0.33	0.29	0.12	0.00	0.19	0.35	0.22	0.28	0.33	0.15	0.00	0.00	0.00	0.00	0.00
GPC1	0.00	0.00	0.10	0.22	0.00	0.22	0.15	0.20	0.19	0.00	0.18	0.24	0.16	0.11	0.28	0.15	0.00	0.00	0.00	0.00	0.00
CACNA1B	0.00	0.00	0.15	0.22	0.00	0.13	0.16	0.16	0.11	0.00	0.10	0.21	0.14	0.21	0.26	0.15	0.00	0.00	0.00	0.00	0.00
FAM168B	0.00	0.00	0.19	0.35	0.00	0.37	0.25	0.26	0.15	0.00	0.18	0.36	0.23	0.23	0.51	0.42	0.00	0.00	0.00	0.00	0.00
OMG	0.00	0.00	0.15	0.28	0.00	0.27	0.20	0.14	0.14	0.00	0.20	0.26	0.17	0.15	0.48	0.43	0.00	0.00	0.00	0.00	0.00
PIEZO1	0.00	0.00	0.16	0.30	0.00	0.33	0.19	0.26	0.25	0.00	0.27	0.26	0.10	0.16	0.27	0.13	0.00	0.00	0.00	0.00	0.00
PEX5L	0.00	0.00	0.17	0.35	0.00	0.38	0.22	0.22	0.15	0.00	0.15	0.37	0.22	0.26	0.32	0.13	0.00	0.00	0.00	0.00	0.00
ALS2	0.00	0.00	0.14	0.26	0.00	0.28	0.17	0.17	0.17	0.00	0.11	0.25	0.15	0.15	0.33	0.20	0.00	0.00	0.00	0.00	0.00
NXPH1	0.00	0.00	0.22	0.38	0.00	0.35	0.29	0.17	0.14	0.00	0.18	0.36	0.24	0.23	0.37	0.14	0.00	0.00	0.00	0.00	0.00
NRTN	0.00	0.00	0.21	0.28	0.00	0.15	0.25	0.18	0.18	0.00	0.10	0.17	0.14	0.13	0.33	0.14	0.00	0.00	0.00	0.00	0.00
BAI3	0.00	0.00	0.21	0.37	0.00	0.37	0.27	0.22	0.20	0.00	0.13	0.37	0.26	0.22	0.37	0.13	0.00	0.00	0.00	0.00	0.00
CDH12	0.00	0.00	0.11	0.27	0.00	0.37	0.15	0.18	0.15	0.00	0.18	0.27	0.17	0.11	0.32	0.14	0.00	0.00	0.00	0.00	0.00
NDNF	0.00	0.00	0.11	0.28	0.00	0.39	0.16	0.16	0.17	0.00	0.10	0.59	0.47	0.49	0.62	0.44	0.00	0.00	0.00	0.00	0.00
KCNN2	0.00	0.00	0.13	0.24	0.00	0.20	0.17	0.18	0.15	0.00	0.15	0.24	0.17	0.25	0.26	0.14	0.00	0.00	0.00	0.00	0.00
RTN4RL2	0.00	0.00	0.21	0.35	0.00	0.28	0.28	0.22	0.21	0.00	0.23	0.41	0.32	0.34	0.55	0.43	0.00	0.00	0.00	0.00	0.00

GPM6A	0.00	0.00	0.20	0.36	0.00	0.34	0.26	0.20	0.12	0.00	0.21	0.51	0.45	0.38	0.57	0.43	0.00	0.00	0.00	0.00	0.00
DSCAM	0.00	0.00	0.19	0.34	0.00	0.28	0.27	0.22	0.13	0.00	0.19	0.37	0.31	0.23	0.52	0.31	0.00	0.00	0.00	0.00	0.00
GFRA3	0.00	0.00	0.22	0.31	0.00	0.18	0.28	0.20	0.15	0.00	0.14	0.17	0.13	0.10	0.36	0.14	0.00	0.00	0.00	0.00	0.00
LRRC15	0.00	0.00	0.16	0.35	0.00	0.43	0.22	0.38	0.23	0.00	0.17	0.29	0.12	0.14	0.27	0.13	0.00	0.00	0.00	0.00	0.00
TEX261	0.00	0.00	0.40	0.54	0.00	0.31	0.48	0.21	0.13	0.00	0.16	0.30	0.19	0.29	0.27	0.15	0.00	0.00	0.00	0.00	0.00
KIDINS220	0.00	0.00	0.27	0.40	0.00	0.31	0.33	0.30	0.18	0.00	0.17	0.27	0.20	0.24	0.31	0.13	0.00	0.00	0.00	0.00	0.00
KCNK2	0.00	0.00	0.14	0.23	0.00	0.16	0.16	0.17	0.20	0.00	0.15	0.20	0.18	0.18	0.26	0.15	0.00	0.00	0.00	0.00	0.00
CREB3L1	0.00	0.00	0.12	0.30	0.00	0.39	0.17	0.31	0.18	0.00	0.17	0.22	0.15	0.15	0.27	0.13	0.00	0.00	0.00	0.00	0.00
GRIK2	0.00	0.00	0.21	0.29	0.00	0.12	0.25	0.12	0.15	0.00	0.14	0.20	0.21	0.26	0.26	0.15	0.00	0.00	0.00	0.00	0.00
PANX1	0.00	0.00	0.20	0.30	0.00	0.20	0.23	0.18	0.14	0.00	0.18	0.19	0.14	0.17	0.24	0.15	0.00	0.00	0.00	0.00	0.00
GRIK4	0.00	0.00	0.22	0.34	0.00	0.19	0.29	0.12	0.11	0.00	0.15	0.28	0.29	0.35	0.34	0.20	0.00	0.00	0.00	0.00	0.00
RTN4R	0.00	0.00	0.14	0.25	0.00	0.18	0.20	0.20	0.26	0.00	0.19	0.29	0.28	0.30	0.47	0.42	0.00	0.00	0.00	0.00	0.00
NGFRAP1	0.00	0.00	0.20	0.37	0.00	0.38	0.25	0.28	0.15	0.00	0.11	0.31	0.21	0.24	0.37	0.20	0.00	0.00	0.00	0.00	0.00
KCNK18	0.00	0.00	0.15	0.29	0.00	0.31	0.19	0.18	0.13	0.00	0.21	0.27	0.12	0.16	0.29	0.14	0.00	0.00	0.00	0.00	0.00
KCNK3	0.00	0.00	0.11	0.20	0.00	0.15	0.14	0.17	0.18	0.00	0.19	0.26	0.25	0.24	0.32	0.20	0.00	0.00	0.00	0.00	0.00
ODZ1	0.00	0.00	0.17	0.38	0.00	0.49	0.22	0.25	0.11	0.00	0.15	0.40	0.28	0.25	0.45	0.20	0.00	0.00	0.00	0.00	0.00
CHST10	0.00	0.00	0.13	0.29	0.00	0.31	0.20	0.17	0.11	0.00	0.19	0.37	0.26	0.25	0.38	0.20	0.00	0.00	0.00	0.00	0.00
CRBN	0.00	0.00	0.11	0.31	0.00	0.46	0.15	0.26	0.10	0.00	0.10	0.36	0.21	0.25	0.34	0.15	0.00	0.00	0.00	0.00	0.00
SEZ6L2	0.00	0.00	0.16	0.34	0.00	0.44	0.21	0.21	0.17	0.00	0.20	0.40	0.25	0.24	0.35	0.19	0.00	0.00	0.00	0.00	0.00
ASTN1	0.00	0.00	0.21	0.38	0.00	0.38	0.27	0.22	0.13	0.00	0.14	0.48	0.43	0.39	0.53	0.35	0.00	0.00	0.00	0.00	0.00
USP14	0.00	0.00	0.15	0.32	0.00	0.36	0.20	0.25	0.19	0.00	0.13	0.28	0.19	0.20	0.33	0.16	0.00	0.00	0.00	0.00	0.00
CNTN5	0.00	0.00	0.15	0.30	0.00	0.31	0.21	0.15	0.12	0.00	0.24	0.45	0.33	0.30	0.53	0.34	0.00	0.00	0.00	0.00	0.00
IL1RAPL1	0.00	0.00	0.24	0.40	0.00	0.37	0.30	0.15	0.10	0.00	0.17	0.40	0.27	0.30	0.36	0.17	0.00	0.00	0.00	0.00	0.00
SLC8A3	0.00	0.00	0.10	0.22	0.00	0.23	0.13	0.16	0.14	0.00	0.15	0.34	0.27	0.32	0.27	0.16	0.00	0.00	0.00	0.00	0.00
TNR	0.00	0.00	0.10	0.22	0.00	0.18	0.17	0.15	0.15	0.00	0.13	0.33	0.32	0.34	0.45	0.35	0.00	0.00	0.00	0.00	0.00
SYNC	0.00	0.00	0.13	0.25	0.00	0.26	0.16	0.12	0.16	0.00	0.12	0.23	0.12	0.13	0.31	0.11	0.00	0.00	0.00	0.00	0.00
NETO2	0.00	0.00	0.33	0.53	0.00	0.43	0.40	0.29	0.14	0.00	0.23	0.33	0.18	0.21	0.36	0.17	0.00	0.00	0.00	0.00	0.00
TM2D1	0.00	0.00	0.25	0.39	0.00	0.35	0.30	0.25	0.18	0.00	0.17	0.27	0.11	0.15	0.25	0.11	0.00	0.00	0.00	0.00	0.00
VSNL1	0.00	0.00	0.23	0.36	0.00	0.28	0.27	0.30	0.19	0.00	0.12	0.35	0.28	0.32	0.32	0.16	0.00	0.00	0.00	0.00	0.00
PTPN5	0.00	0.00	0.20	0.35	0.00	0.30	0.25	0.21	0.20	0.00	0.16	0.35	0.27	0.36	0.31	0.17	0.00	0.00	0.00	0.00	0.00
TM2D2	0.00	0.00	0.19	0.35	0.00	0.42	0.22	0.26	0.19	0.00	0.15	0.32	0.14	0.21	0.28	0.11	0.00	0.00	0.00	0.00	0.00
SLITRK6	0.00	0.00	0.25	0.43	0.00	0.43	0.31	0.23	0.10	0.00	0.24	0.50	0.33	0.30	0.60	0.36	0.00	0.00	0.00	0.00	0.00
CHRNA10	0.00	0.00	0.21	0.32	0.00	0.20	0.28	0.15	0.11	0.00	0.17	0.19	0.11	0.14	0.28	0.11	0.00	0.00	0.00	0.00	0.00
SPON1	0.00	0.00	0.18	0.35	0.00	0.34	0.26	0.27	0.21	0.00	0.21	0.27	0.16	0.14	0.33	0.17	0.00	0.00	0.00	0.00	0.00
LXN	0.00	0.00	0.12	0.32	0.00	0.42	0.18	0.32	0.19	0.00	0.12	0.32	0.17	0.16	0.36	0.17	0.00	0.00	0.00	0.00	0.00
RGMA	0.00	0.00	0.20	0.33	0.00	0.25	0.27	0.26	0.23	0.00	0.17	0.28	0.24	0.23	0.46	0.34	0.00	0.00	0.00	0.00	0.00
FBXO2	0.00	0.00	0.14	0.35	0.00	0.43	0.21	0.29	0.10	0.00	0.19	0.25	0.11	0.14	0.29	0.11	0.00	0.00	0.00	0.00	0.00
ITPRIP	0.00	0.00	0.28	0.45	0.00	0.43	0.32	0.31	0.14	0.00	0.12	0.36	0.20	0.24	0.34	0.17	0.00	0.00	0.00	0.00	0.00
PPFIA1	0.00	0.00	0.23	0.40	0.00	0.37	0.29	0.29	0.12	0.00	0.18	0.28	0.16	0.18	0.28	0.11	0.00	0.00	0.00	0.00	0.00
SNX12	0.00	0.00	0.17	0.34	0.00	0.39	0.22	0.21	0.17	0.00	0.12	0.49	0.36	0.33	0.50	0.34	0.00	0.00	0.00	0.00	0.00

KCNQ3	0.00	0.00	0.10	0.17	0.00	0.14	0.11	0.15	0.12	0.00	0.16	0.23	0.18	0.24	0.28	0.17	0.00	0.00	0.00	0.00	0.00
PRIMA1	0.00	0.00	0.13	0.30	0.00	0.35	0.19	0.26	0.14	0.00	0.18	0.26	0.18	0.17	0.31	0.11	0.00	0.00	0.00	0.00	0.00
KCNK1	0.00	0.00	0.13	0.25	0.00	0.25	0.15	0.14	0.12	0.00	0.22	0.28	0.21	0.18	0.31	0.17	0.00	0.00	0.00	0.00	0.00
ASIC2	0.00	0.00	0.15	0.24	0.00	0.17	0.18	0.19	0.20	0.00	0.17	0.23	0.18	0.21	0.33	0.17	0.00	0.00	0.00	0.00	0.00
CNTN1	0.00	0.00	0.16	0.27	0.00	0.19	0.22	0.16	0.12	0.00	0.27	0.31	0.24	0.22	0.50	0.34	0.00	0.00	0.00	0.00	0.00
NGEF	0.00	0.00	0.24	0.37	0.00	0.29	0.30	0.26	0.12	0.00	0.11	0.25	0.19	0.20	0.31	0.17	0.00	0.00	0.00	0.00	0.00
UNC79	0.00	0.00	0.18	0.32	0.00	0.30	0.21	0.15	0.12	0.00	0.17	0.28	0.17	0.15	0.34	0.11	0.00	0.00	0.00	0.00	0.00
KCNK4	0.00	0.00	0.17	0.29	0.00	0.28	0.19	0.14	0.12	0.00	0.18	0.29	0.16	0.17	0.32	0.16	0.00	0.00	0.00	0.00	0.00
NRN1L	0.00	0.00	0.21	0.41	0.00	0.44	0.28	0.27	0.12	0.00	0.18	0.54	0.44	0.43	0.60	0.36	0.00	0.00	0.00	0.00	0.00
PTX4	0.00	0.00	0.18	0.36	0.00	0.40	0.24	0.19	0.12	0.00	0.12	0.39	0.31	0.39	0.38	0.19	0.00	0.00	0.00	0.00	0.00
CDH9	0.00	0.00	0.12	0.28	0.00	0.34	0.17	0.18	0.11	0.00	0.18	0.33	0.24	0.20	0.34	0.16	0.00	0.00	0.00	0.00	0.00
SEMA4F	0.00	0.00	0.18	0.34	0.00	0.36	0.24	0.27	0.18	0.00	0.19	0.25	0.15	0.10	0.34	0.16	0.00	0.00	0.00	0.00	0.00
GRIA4	0.00	0.00	0.17	0.27	0.00	0.15	0.23	0.11	0.10	0.00	0.15	0.19	0.20	0.26	0.25	0.16	0.00	0.00	0.00	0.00	0.00
GFRA2	0.00	0.00	0.20	0.27	0.00	0.14	0.25	0.17	0.16	0.00	0.11	0.18	0.16	0.16	0.36	0.16	0.00	0.00	0.00	0.00	0.00
TMEM132D	0.00	0.00	0.12	0.27	0.00	0.37	0.16	0.12	0.12	0.00	0.11	0.32	0.27	0.22	0.31	0.18	0.00	0.00	0.00	0.00	0.00
SRPX2	0.00	0.00	0.16	0.33	0.00	0.40	0.21	0.30	0.17	0.00	0.14	0.29	0.19	0.12	0.31	0.12	0.00	0.00	0.00	0.00	0.00
MAGEE1	0.00	0.00	0.16	0.34	0.00	0.47	0.19	0.19	0.12	0.00	0.17	0.37	0.13	0.18	0.36	0.16	0.00	0.00	0.00	0.00	0.00
CTNND2	0.00	0.00	0.13	0.25	0.00	0.26	0.17	0.21	0.11	0.00	0.15	0.25	0.17	0.16	0.28	0.12	0.00	0.00	0.00	0.00	0.00
CTSE	0.00	0.00	0.14	0.26	0.00	0.25	0.19	0.19	0.19	0.00	0.11	0.19	0.14	0.12	0.21	0.12	0.00	0.00	0.00	0.00	0.00
JAKMIP2	0.00	0.00	0.34	0.55	0.00	0.50	0.41	0.27	0.12	0.00	0.20	0.37	0.23	0.20	0.38	0.17	0.00	0.00	0.00	0.00	0.00
NCS1	0.00	0.00	0.23	0.33	0.00	0.24	0.24	0.23	0.14	0.00	0.12	0.35	0.29	0.35	0.35	0.19	0.00	0.00	0.00	0.00	0.00
ASTN2	0.00	0.00	0.21	0.36	0.00	0.32	0.28	0.11	0.11	0.00	0.13	0.34	0.25	0.21	0.37	0.19	0.00	0.00	0.00	0.00	0.00
CHODL	0.00	0.00	0.17	0.39	0.00	0.48	0.23	0.16	0.12	0.00	0.23	0.32	0.12	0.13	0.36	0.16	0.00	0.00	0.00	0.00	0.00
SLITRK5	0.00	0.00	0.28	0.49	0.00	0.48	0.35	0.23	0.11	0.00	0.27	0.52	0.35	0.29	0.61	0.36	0.00	0.00	0.00	0.00	0.00
MMP24	0.00	0.00	0.20	0.36	0.00	0.36	0.24	0.26	0.19	0.00	0.20	0.31	0.16	0.16	0.34	0.18	0.00	0.00	0.00	0.00	0.00
RTN4RL1	0.00	0.00	0.25	0.42	0.00	0.37	0.32	0.18	0.11	0.00	0.28	0.38	0.24	0.22	0.56	0.44	0.00	0.00	0.00	0.00	0.00
CD200R1	0.00	0.00	0.29	0.43	0.00	0.26	0.38	0.24	0.24	0.00	0.20	0.21	0.12	0.12	0.31	0.18	0.00	0.00	0.00	0.00	0.00
CACNG2	0.00	0.00	0.17	0.26	0.00	0.16	0.21	0.16	0.10	0.00	0.15	0.22	0.21	0.28	0.25	0.17	0.00	0.00	0.00	0.00	0.00
RNF103	0.00	0.00	0.15	0.35	0.00	0.44	0.20	0.30	0.16	0.00	0.11	0.31	0.23	0.24	0.32	0.11	0.00	0.00	0.00	0.00	0.00
DTNA	0.00	0.00	0.15	0.25	0.00	0.21	0.18	0.11	0.14	0.00	0.16	0.21	0.11	0.12	0.28	0.11	0.00	0.00	0.00	0.00	0.00
CNGA2	0.00	0.00	0.22	0.30	0.00	0.19	0.23	0.18	0.11	0.00	0.17	0.25	0.19	0.18	0.32	0.16	0.00	0.00	0.00	0.00	0.00
CPLX2	0.00	0.00	0.11	0.24	0.00	0.20	0.16	0.22	0.14	0.00	0.11	0.32	0.31	0.33	0.36	0.18	0.00	0.00	0.00	0.00	0.00
SEMA3B	0.00	0.00	0.11	0.22	0.00	0.17	0.18	0.25	0.17	0.00	0.15	0.18	0.16	0.11	0.31	0.18	0.00	0.00	0.00	0.00	0.00
PLXNA1	0.00	0.00	0.16	0.28	0.00	0.22	0.23	0.21	0.12	0.00	0.18	0.18	0.17	0.11	0.33	0.18	0.00	0.00	0.00	0.00	0.00
KCNIP3	0.00	0.00	0.14	0.25	0.00	0.23	0.16	0.26	0.16	0.00	0.13	0.29	0.24	0.34	0.28	0.18	0.00	0.00	0.00	0.00	0.00
CACNA2D1	0.00	0.00	0.16	0.26	0.00	0.20	0.18	0.15	0.15	0.00	0.16	0.26	0.14	0.22	0.29	0.16	0.00	0.00	0.00	0.00	0.00
AGRN	0.00	0.00	0.13	0.23	0.00	0.13	0.20	0.17	0.18	0.00	0.18	0.20	0.17	0.16	0.30	0.18	0.00	0.00	0.00	0.00	0.00
ARTN	0.00	0.00	0.21	0.30	0.00	0.17	0.26	0.26	0.26	0.00	0.10	0.18	0.14	0.14	0.36	0.15	0.00	0.00	0.00	0.00	0.00
SEMA5A	0.00	0.00	0.16	0.28	0.00	0.27	0.21	0.25	0.16	0.00	0.15	0.25	0.20	0.14	0.36	0.18	0.00	0.00	0.00	0.00	0.00
SEMA4A	0.00	0.00	0.21	0.37	0.00	0.32	0.28	0.24	0.18	0.00	0.21	0.26	0.17	0.13	0.47	0.27	0.00	0.00	0.00	0.00	0.00

SEMA4B	0.00	0.00	0.23	0.39	0.00	0.34	0.30	0.26	0.11	0.00	0.23	0.29	0.21	0.17	0.45	0.24	0.00	0.00	0.00	0.00	0.00
LRRN1	0.00	0.00	0.16	0.36	0.00	0.49	0.21	0.30	0.14	0.00	0.11	0.39	0.23	0.18	0.46	0.27	0.00	0.00	0.00	0.00	0.00
ADAM22	0.00	0.00	0.19	0.37	0.00	0.41	0.25	0.25	0.14	0.00	0.24	0.34	0.16	0.19	0.41	0.22	0.00	0.00	0.00	0.00	0.00
RGMB	0.00	0.00	0.21	0.37	0.00	0.32	0.29	0.25	0.13	0.00	0.20	0.31	0.23	0.23	0.46	0.26	0.00	0.00	0.00	0.00	0.00
ICAM5	0.00	0.00	0.17	0.30	0.00	0.22	0.25	0.20	0.13	0.00	0.20	0.30	0.28	0.31	0.34	0.25	0.00	0.00	0.00	0.00	0.00
SLC6A12	0.00	0.00	0.14	0.28	0.00	0.23	0.20	0.19	0.13	0.00	0.21	0.32	0.35	0.35	0.34	0.21	0.00	0.00	0.00	0.00	0.00
MDGA2	0.00	0.00	0.12	0.24	0.00	0.23	0.19	0.14	0.10	0.00	0.13	0.24	0.22	0.15	0.38	0.24	0.00	0.00	0.00	0.00	0.00
PLXNB3	0.00	0.00	0.26	0.42	0.00	0.37	0.31	0.27	0.14	0.00	0.20	0.33	0.23	0.20	0.44	0.23	0.00	0.00	0.00	0.00	0.00
KLHL1	0.00	0.00	0.11	0.28	0.00	0.39	0.14	0.20	0.11	0.00	0.11	0.37	0.20	0.20	0.37	0.23	0.00	0.00	0.00	0.00	0.00
SEMA6D	0.00	0.00	0.27	0.44	0.00	0.31	0.36	0.27	0.12	0.00	0.23	0.28	0.21	0.16	0.48	0.26	0.00	0.00	0.00	0.00	0.00
NOVA2	0.00	0.00	0.18	0.38	0.00	0.39	0.26	0.29	0.15	0.00	0.11	0.37	0.24	0.27	0.41	0.24	0.00	0.00	0.00	0.00	0.00
SLC6A11	0.00	0.00	0.17	0.29	0.00	0.21	0.22	0.15	0.13	0.00	0.19	0.39	0.41	0.39	0.39	0.27	0.00	0.00	0.00	0.00	0.00
ECEL1	0.00	0.00	0.14	0.33	0.00	0.38	0.20	0.25	0.15	0.00	0.16	0.41	0.28	0.25	0.54	0.31	0.00	0.00	0.00	0.00	0.00
UNC5A	0.00	0.00	0.19	0.32	0.00	0.21	0.27	0.31	0.13	0.00	0.17	0.19	0.22	0.10	0.41	0.26	0.00	0.00	0.00	0.00	0.00
CHL1	0.00	0.00	0.16	0.28	0.00	0.24	0.22	0.23	0.17	0.00	0.17	0.35	0.36	0.33	0.45	0.30	0.00	0.00	0.00	0.00	0.00
FGF14	0.00	0.00	0.14	0.29	0.00	0.34	0.19	0.16	0.12	0.00	0.15	0.38	0.26	0.27	0.40	0.22	0.00	0.00	0.00	0.00	0.00
NCAM2	0.00	0.00	0.15	0.31	0.00	0.30	0.22	0.25	0.11	0.00	0.21	0.29	0.23	0.19	0.40	0.22	0.00	0.00	0.00	0.00	0.00
NTNG1	0.00	0.00	0.20	0.35	0.00	0.28	0.27	0.15	0.12	0.00	0.19	0.38	0.36	0.27	0.46	0.27	0.00	0.00	0.00	0.00	0.00
CNTFR	0.00	0.00	0.22	0.35	0.00	0.19	0.31	0.21	0.18	0.00	0.13	0.18	0.14	0.14	0.36	0.22	0.00	0.00	0.00	0.00	0.00
AMIGO2	0.00	0.00	0.21	0.44	0.00	0.54	0.27	0.25	0.12	0.00	0.31	0.41	0.20	0.23	0.41	0.21	0.00	0.00	0.00	0.00	0.00
NINJ1	0.00	0.00	0.15	0.33	0.00	0.41	0.21	0.28	0.21	0.00	0.14	0.31	0.16	0.14	0.45	0.30	0.00	0.00	0.00	0.00	0.00
GPRIN1	0.00	0.00	0.29	0.46	0.00	0.39	0.34	0.28	0.13	0.00	0.11	0.40	0.32	0.26	0.45	0.22	0.00	0.00	0.00	0.00	0.00
PLXNA3	0.00	0.00	0.15	0.28	0.00	0.24	0.22	0.21	0.12	0.00	0.16	0.24	0.24	0.17	0.37	0.22	0.00	0.00	0.00	0.00	0.00
GLRA3	0.00	0.00	0.26	0.38	0.00	0.20	0.33	0.17	0.14	0.00	0.18	0.26	0.20	0.23	0.37	0.22	0.00	0.00	0.00	0.00	0.00
SEMA6A	0.00	0.00	0.15	0.26	0.00	0.20	0.21	0.24	0.11	0.00	0.18	0.22	0.27	0.18	0.42	0.29	0.00	0.00	0.00	0.00	0.00
GLRB	0.00	0.00	0.20	0.34	0.00	0.19	0.29	0.15	0.12	0.00	0.17	0.24	0.20	0.19	0.36	0.21	0.00	0.00	0.00	0.00	0.00
NEO1	0.00	0.00	0.20	0.31	0.00	0.19	0.27	0.26	0.14	0.00	0.17	0.20	0.21	0.13	0.40	0.26	0.00	0.00	0.00	0.00	0.00
LGI1	0.00	0.00	0.13	0.25	0.00	0.26	0.17	0.20	0.19	0.00	0.16	0.31	0.23	0.28	0.36	0.21	0.00	0.00	0.00	0.00	0.00
SUSD4	0.00	0.00	0.14	0.37	0.00	0.54	0.19	0.19	0.12	0.00	0.16	0.37	0.19	0.13	0.41	0.24	0.00	0.00	0.00	0.00	0.00
PLXNB2	0.00	0.00	0.28	0.42	0.00	0.33	0.34	0.25	0.14	0.00	0.22	0.26	0.17	0.13	0.41	0.21	0.00	0.00	0.00	0.00	0.00
CADM4	0.00	0.00	0.18	0.39	0.00	0.45	0.26	0.28	0.13	0.00	0.32	0.36	0.17	0.13	0.45	0.29	0.00	0.00	0.00	0.00	0.00
NUMBL	0.00	0.00	0.19	0.35	0.00	0.34	0.25	0.34	0.12	0.00	0.11	0.31	0.23	0.19	0.38	0.21	0.00	0.00	0.00	0.00	0.00
PLXDC1	0.00	0.00	0.21	0.38	0.00	0.37	0.27	0.30	0.30	0.00	0.25	0.35	0.20	0.16	0.42	0.24	0.00	0.00	0.00	0.00	0.00
LSAMP	0.00	0.00	0.14	0.30	0.00	0.32	0.20	0.22	0.17	0.00	0.12	0.36	0.35	0.32	0.40	0.23	0.00	0.00	0.00	0.00	0.00
NDRG3	0.00	0.00	0.15	0.34	0.00	0.47	0.19	0.35	0.21	0.00	0.13	0.32	0.14	0.10	0.37	0.24	0.00	0.00	0.00	0.00	0.00
GRINA	0.00	0.00	0.24	0.37	0.00	0.26	0.29	0.18	0.16	0.00	0.16	0.36	0.25	0.31	0.39	0.26	0.00	0.00	0.00	0.00	0.00
RYK	0.00	0.00	0.26	0.42	0.00	0.35	0.33	0.27	0.13	0.00	0.22	0.29	0.20	0.15	0.41	0.23	0.00	0.00	0.00	0.00	0.00
AVIL	0.00	0.00	0.21	0.40	0.00	0.39	0.29	0.29	0.11	0.00	0.19	0.28	0.19	0.16	0.48	0.26	0.00	0.00	0.00	0.00	0.00
GRIN3A	0.00	0.00	0.19	0.28	0.00	0.15	0.24	0.15	0.16	0.00	0.11	0.27	0.26	0.29	0.34	0.26	0.00	0.00	0.00	0.00	0.00
NRCAM	0.00	0.00	0.14	0.24	0.00	0.19	0.20	0.20	0.12	0.00	0.22	0.27	0.26	0.17	0.45	0.29	0.00	0.00	0.00	0.00	0.00

SEMA3E	0.00	0.00	0.14	0.25	0.00	0.18	0.20	0.27	0.19	0.00	0.16	0.20	0.21	0.15	0.36	0.21	0.00	0.00	0.00	0.00	0.00
CNTNAP4	0.00	0.00	0.15	0.33	0.00	0.44	0.19	0.27	0.13	0.00	0.17	0.35	0.22	0.20	0.40	0.25	0.00	0.00	0.00	0.00	0.00
SSTR1	0.15	0.15	0.25	0.26	0.00	0.00	0.21	0.12	0.18	0.00	0.00	0.17	0.20	0.19	0.14	0.00	0.12	0.00	0.00	0.00	0.00
MOG	0.00	0.00	0.11	0.21	0.00	0.16	0.16	0.15	0.16	0.00	0.16	0.15	0.12	0.00	0.34	0.33	0.11	0.00	0.00	0.00	0.00
BCL11A	0.00	0.00	0.16	0.27	0.00	0.24	0.20	0.28	0.14	0.00	0.00	0.22	0.16	0.13	0.30	0.14	0.11	0.00	0.00	0.00	0.00
TRIM47	0.00	0.00	0.12	0.29	0.00	0.43	0.15	0.17	0.15	0.00	0.00	0.35	0.21	0.18	0.27	0.14	0.12	0.00	0.00	0.00	0.00
AIF1	0.00	0.00	0.12	0.21	0.00	0.17	0.16	0.18	0.25	0.00	0.00	0.22	0.21	0.23	0.30	0.28	0.15	0.00	0.00	0.00	0.00
PTPRD	0.00	0.00	0.17	0.31	0.00	0.29	0.23	0.20	0.00	0.00	0.19	0.29	0.19	0.17	0.33	0.17	0.11	0.00	0.00	0.00	0.00
CHRM2	0.21	0.21	0.20	0.19	0.00	0.00	0.15	0.11	0.13	0.00	0.00	0.11	0.18	0.18	0.19	0.00	0.00	0.11	0.00	0.00	0.00
CHRM1	0.21	0.21	0.19	0.18	0.00	0.00	0.14	0.13	0.15	0.00	0.00	0.11	0.19	0.21	0.18	0.00	0.00	0.11	0.00	0.00	0.00
KCNJ2	0.00	0.00	0.10	0.17	0.00	0.12	0.12	0.14	0.15	0.00	0.14	0.17	0.11	0.12	0.19	0.00	0.00	0.10	0.00	0.00	0.00
P2RX2	0.00	0.00	0.19	0.24	0.00	0.11	0.21	0.15	0.12	0.00	0.15	0.10	0.00	0.12	0.21	0.12	0.00	0.12	0.00	0.00	0.00
NDN	0.00	0.00	0.14	0.27	0.00	0.25	0.20	0.31	0.14	0.00	0.00	0.27	0.18	0.16	0.36	0.16	0.00	0.10	0.00	0.00	0.00
HAP1	0.00	0.00	0.17	0.29	0.00	0.27	0.20	0.17	0.15	0.00	0.00	0.31	0.22	0.17	0.30	0.14	0.00	0.12	0.00	0.00	0.00
ADCY8	0.00	0.00	0.22	0.30	0.00	0.19	0.22	0.21	0.15	0.00	0.00	0.30	0.29	0.33	0.29	0.17	0.00	0.10	0.00	0.00	0.00
BRSK1	0.00	0.00	0.11	0.27	0.00	0.35	0.16	0.34	0.10	0.00	0.00	0.27	0.17	0.15	0.29	0.15	0.00	0.16	0.00	0.00	0.00
NRXN3	0.00	0.00	0.23	0.36	0.00	0.25	0.29	0.16	0.00	0.00	0.21	0.35	0.22	0.24	0.40	0.20	0.00	0.10	0.00	0.00	0.00
SYT4	0.00	0.00	0.15	0.28	0.00	0.26	0.19	0.22	0.00	0.00	0.12	0.36	0.27	0.34	0.34	0.17	0.00	0.11	0.00	0.00	0.00
CHRM3	0.22	0.22	0.22	0.20	0.00	0.00	0.15	0.12	0.14	0.00	0.00	0.00	0.14	0.14	0.17	0.00	0.11	0.13	0.00	0.00	0.00
ADAP1	0.11	0.11	0.25	0.40	0.00	0.43	0.25	0.21	0.00	0.00	0.00	0.36	0.24	0.22	0.31	0.00	0.00	0.00	0.12	0.00	0.00
APBB1	0.00	0.00	0.14	0.27	0.00	0.23	0.19	0.22	0.13	0.00	0.11	0.23	0.14	0.17	0.23	0.00	0.00	0.00	0.15	0.00	0.00
LRPAP1	0.00	0.00	0.18	0.29	0.00	0.19	0.24	0.15	0.11	0.00	0.11	0.15	0.12	0.11	0.16	0.00	0.00	0.00	0.19	0.00	0.00
UBQLN1	0.00	0.00	0.14	0.28	0.00	0.29	0.19	0.22	0.15	0.00	0.16	0.19	0.12	0.11	0.21	0.00	0.00	0.00	0.10	0.00	0.00
ENPEP	0.00	0.00	0.15	0.26	0.00	0.22	0.18	0.23	0.21	0.00	0.12	0.23	0.18	0.12	0.23	0.00	0.00	0.00	0.18	0.00	0.00
LRP2	0.00	0.00	0.17	0.26	0.00	0.13	0.22	0.13	0.11	0.00	0.11	0.13	0.15	0.00	0.18	0.11	0.00	0.00	0.19	0.00	0.00
MCOLN1	0.00	0.00	0.13	0.26	0.00	0.27	0.17	0.15	0.17	0.00	0.18	0.22	0.10	0.00	0.25	0.15	0.00	0.00	0.13	0.00	0.00
NPC2	0.00	0.00	0.10	0.23	0.00	0.25	0.15	0.19	0.19	0.00	0.15	0.18	0.11	0.00	0.22	0.14	0.00	0.00	0.23	0.00	0.00
FOLH1	0.00	0.00	0.11	0.19	0.00	0.14	0.14	0.16	0.22	0.00	0.14	0.15	0.00	0.12	0.21	0.13	0.00	0.00	0.19	0.00	0.00
DAGLB	0.00	0.00	0.19	0.27	0.00	0.16	0.23	0.20	0.11	0.00	0.00	0.33	0.30	0.31	0.37	0.25	0.00	0.00	0.12	0.00	0.00
ZNF512B	0.00	0.00	0.11	0.26	0.00	0.32	0.15	0.46	0.24	0.00	0.00	0.21	0.10	0.11	0.22	0.11	0.00	0.00	0.17	0.00	0.00
NPEPPS	0.00	0.00	0.12	0.29	0.00	0.36	0.16	0.24	0.19	0.00	0.00	0.32	0.20	0.20	0.28	0.11	0.00	0.00	0.17	0.00	0.00
CBWD1	0.00	0.00	0.16	0.39	0.00	0.54	0.20	0.24	0.12	0.00	0.00	0.36	0.22	0.16	0.35	0.16	0.00	0.00	0.14	0.00	0.00
PDE1B	0.00	0.00	0.18	0.31	0.00	0.30	0.19	0.20	0.15	0.00	0.00	0.35	0.25	0.22	0.26	0.12	0.00	0.00	0.15	0.00	0.00
ADNP	0.00	0.00	0.15	0.25	0.00	0.20	0.17	0.21	0.26	0.00	0.00	0.35	0.31	0.37	0.40	0.24	0.00	0.00	0.10	0.00	0.00
PPP1R17	0.00	0.00	0.11	0.30	0.00	0.42	0.17	0.21	0.15	0.00	0.00	0.38	0.23	0.22	0.32	0.16	0.00	0.00	0.13	0.00	0.00
NAE1	0.00	0.00	0.10	0.27	0.00	0.35	0.15	0.32	0.15	0.00	0.00	0.25	0.13	0.15	0.27	0.11	0.00	0.00	0.13	0.00	0.00
HPX	0.00	0.00	0.12	0.25	0.00	0.23	0.17	0.15	0.16	0.00	0.00	0.21	0.13	0.13	0.25	0.15	0.00	0.00	0.18	0.00	0.00
NR4A2	0.00	0.00	0.12	0.20	0.00	0.11	0.17	0.22	0.20	0.00	0.00	0.16	0.22	0.18	0.25	0.15	0.00	0.00	0.10	0.00	0.00
PDE10A	0.00	0.00	0.17	0.28	0.00	0.26	0.18	0.21	0.19	0.00	0.00	0.35	0.31	0.25	0.30	0.13	0.00	0.00	0.12	0.00	0.00
CH25H	0.00	0.00	0.21	0.34	0.00	0.26	0.27	0.23	0.21	0.00	0.00	0.20	0.12	0.12	0.21	0.11	0.00	0.00	0.28	0.00	0.00

LYPD1	0.00	0.00	0.24	0.43	0.00	0.49	0.28	0.42	0.19	0.00	0.00	0.32	0.19	0.14	0.34	0.13	0.00	0.00	0.15	0.00	0.00
RORA	0.00	0.00	0.15	0.23	0.00	0.13	0.20	0.20	0.13	0.00	0.00	0.17	0.21	0.14	0.24	0.18	0.00	0.00	0.18	0.00	0.00
CUEDC1	0.00	0.00	0.12	0.27	0.00	0.32	0.16	0.17	0.16	0.00	0.00	0.34	0.24	0.26	0.31	0.17	0.00	0.00	0.13	0.00	0.00
C4ORF19	0.00	0.00	0.12	0.27	0.00	0.32	0.16	0.17	0.16	0.00	0.00	0.34	0.24	0.26	0.31	0.17	0.00	0.00	0.13	0.00	0.00
C14ORF23	0.00	0.00	0.12	0.27	0.00	0.32	0.16	0.17	0.16	0.00	0.00	0.34	0.24	0.26	0.31	0.17	0.00	0.00	0.13	0.00	0.00
CYP7B1	0.00	0.00	0.19	0.28	0.00	0.18	0.24	0.23	0.22	0.00	0.00	0.17	0.12	0.11	0.18	0.10	0.00	0.00	0.34	0.00	0.00
CAMK4	0.00	0.00	0.12	0.21	0.00	0.18	0.14	0.23	0.13	0.00	0.00	0.22	0.19	0.26	0.20	0.10	0.00	0.00	0.11	0.00	0.00
BEX1	0.00	0.00	0.17	0.36	0.00	0.41	0.23	0.32	0.16	0.00	0.00	0.39	0.23	0.22	0.42	0.20	0.00	0.00	0.11	0.00	0.00
ACP2	0.00	0.00	0.11	0.26	0.00	0.29	0.17	0.14	0.14	0.00	0.00	0.24	0.12	0.11	0.21	0.12	0.00	0.00	0.17	0.00	0.00
ECE2	0.00	0.00	0.16	0.24	0.00	0.20	0.18	0.17	0.25	0.00	0.00	0.32	0.25	0.23	0.30	0.15	0.00	0.00	0.13	0.00	0.00
HAAO	0.00	0.00	0.11	0.26	0.00	0.30	0.15	0.14	0.22	0.00	0.00	0.40	0.28	0.25	0.30	0.19	0.00	0.00	0.32	0.00	0.00
RBFOX1	0.00	0.00	0.12	0.27	0.00	0.32	0.16	0.26	0.13	0.00	0.00	0.30	0.16	0.16	0.33	0.14	0.00	0.00	0.13	0.00	0.00
SLC44A3	0.00	0.00	0.13	0.39	0.00	0.61	0.18	0.15	0.00	0.00	0.23	0.51	0.29	0.25	0.53	0.34	0.00	0.00	0.11	0.00	0.00
ATP2B2	0.00	0.00	0.11	0.20	0.00	0.17	0.14	0.13	0.00	0.00	0.17	0.24	0.16	0.16	0.24	0.14	0.00	0.00	0.12	0.00	0.00
CLSTN3	0.00	0.00	0.14	0.29	0.00	0.38	0.17	0.24	0.00	0.00	0.16	0.37	0.19	0.23	0.31	0.15	0.00	0.00	0.10	0.00	0.00
NGRN	0.00	0.00	0.15	0.40	0.00	0.65	0.19	0.17	0.00	0.00	0.16	0.64	0.34	0.30	0.59	0.33	0.00	0.00	0.11	0.00	0.00
SERINC5	0.00	0.00	0.14	0.39	0.00	0.58	0.20	0.18	0.00	0.00	0.28	0.40	0.23	0.18	0.41	0.29	0.00	0.00	0.16	0.00	0.00
ATP6V0C	0.00	0.00	0.14	0.34	0.00	0.43	0.20	0.21	0.00	0.00	0.23	0.34	0.18	0.14	0.31	0.15	0.00	0.00	0.11	0.00	0.00
LDLRAD3	0.00	0.00	0.25	0.40	0.00	0.36	0.30	0.16	0.00	0.00	0.25	0.32	0.18	0.19	0.27	0.10	0.00	0.00	0.14	0.00	0.00
LPPR1	0.00	0.00	0.26	0.41	0.00	0.41	0.29	0.22	0.00	0.00	0.17	0.62	0.49	0.43	0.65	0.44	0.00	0.00	0.11	0.00	0.00
LANCL1	0.00	0.00	0.22	0.45	0.00	0.60	0.24	0.16	0.00	0.00	0.13	0.43	0.19	0.16	0.40	0.18	0.00	0.00	0.16	0.00	0.00
ASCL4	0.00	0.00	0.10	0.34	0.00	0.60	0.13	0.16	0.00	0.00	0.14	0.41	0.23	0.18	0.33	0.14	0.00	0.00	0.14	0.00	0.00
GDPD5	0.00	0.00	0.15	0.42	0.00	0.61	0.21	0.31	0.00	0.00	0.16	0.43	0.26	0.16	0.42	0.20	0.00	0.00	0.17	0.00	0.00
FA2H	0.00	0.00	0.00	0.21	0.00	0.32	0.11	0.12	0.15	0.00	0.13	0.31	0.18	0.11	0.42	0.35	0.00	0.00	0.28	0.00	0.00
SLC39A3	0.00	0.00	0.00	0.28	0.00	0.35	0.15	0.22	0.16	0.00	0.18	0.29	0.21	0.22	0.30	0.17	0.00	0.00	0.16	0.00	0.00
ST8SIA1	0.00	0.00	0.00	0.19	0.00	0.21	0.12	0.22	0.24	0.00	0.12	0.25	0.16	0.17	0.31	0.23	0.00	0.00	0.11	0.00	0.00
B4GALNT1	0.00	0.00	0.00	0.17	0.00	0.17	0.12	0.14	0.24	0.00	0.16	0.26	0.18	0.18	0.37	0.31	0.00	0.00	0.12	0.00	0.00
SLC30A6	0.00	0.00	0.00	0.27	0.00	0.40	0.13	0.15	0.12	0.00	0.21	0.37	0.25	0.26	0.30	0.14	0.00	0.00	0.14	0.00	0.00
SLC30A1	0.00	0.00	0.00	0.20	0.00	0.22	0.12	0.17	0.17	0.00	0.12	0.28	0.23	0.27	0.25	0.15	0.00	0.00	0.17	0.00	0.00
KCNJ10	0.00	0.00	0.00	0.18	0.00	0.14	0.11	0.12	0.13	0.00	0.18	0.25	0.23	0.23	0.30	0.23	0.00	0.00	0.10	0.00	0.00
QPCTL	0.00	0.00	0.00	0.25	0.00	0.38	0.11	0.16	0.17	0.00	0.11	0.30	0.14	0.16	0.27	0.10	0.00	0.00	0.15	0.00	0.00
NPC1	0.00	0.00	0.00	0.15	0.00	0.11	0.11	0.14	0.21	0.00	0.11	0.20	0.16	0.15	0.25	0.26	0.00	0.00	0.23	0.00	0.00
SLC30A7	0.00	0.00	0.00	0.29	0.00	0.43	0.14	0.20	0.13	0.00	0.22	0.32	0.21	0.20	0.29	0.13	0.00	0.00	0.19	0.00	0.00
NEU4	0.00	0.00	0.00	0.26	0.00	0.40	0.13	0.21	0.21	0.00	0.15	0.32	0.16	0.12	0.28	0.19	0.00	0.00	0.16	0.00	0.00
ARSG	0.00	0.00	0.13	0.31	0.00	0.41	0.18	0.14	0.11	0.00	0.15	0.22	0.10	0.00	0.18	0.00	0.12	0.00	0.16	0.00	0.00
CALHM1	0.00	0.00	0.11	0.19	0.00	0.17	0.13	0.00	0.12	0.00	0.14	0.18	0.12	0.17	0.12	0.00	0.11	0.00	0.11	0.00	0.00
CD68	0.00	0.00	0.13	0.25	0.00	0.19	0.20	0.21	0.23	0.00	0.10	0.14	0.00	0.00	0.19	0.12	0.10	0.00	0.11	0.00	0.00
TSHR	0.15	0.15	0.19	0.18	0.15	0.00	0.16	0.14	0.00	0.39	0.13	0.00	0.00	0.00	0.12	0.00	0.00	0.12	0.11	0.00	0.00
DIO1	0.00	0.00	0.11	0.20	0.00	0.17	0.14	0.20	0.15	0.14	0.00	0.14	0.10	0.00	0.15	0.00	0.00	0.14	0.27	0.00	0.00
PNPLA7	0.00	0.00	0.11	0.33	0.00	0.52	0.14	0.23	0.13	0.00	0.10	0.32	0.12	0.00	0.25	0.00	0.00	0.14	0.28	0.00	0.00

PSPH	0.00	0.00	0.11	0.25	0.00	0.31	0.14	0.21	0.16	0.00	0.00	0.29	0.17	0.13	0.22	0.00	0.00	0.13	0.32	0.00	0.00
MAGEL2	0.00	0.00	0.14	0.27	0.00	0.33	0.16	0.18	0.00	0.00	0.00	0.36	0.22	0.19	0.38	0.16	0.00	0.14	0.11	0.00	0.00
QPCT	0.00	0.00	0.00	0.21	0.00	0.27	0.11	0.18	0.19	0.00	0.00	0.33	0.26	0.27	0.31	0.15	0.00	0.11	0.19	0.00	0.00
MT3	0.00	0.00	0.00	0.18	0.00	0.19	0.10	0.17	0.17	0.00	0.00	0.26	0.23	0.24	0.28	0.20	0.00	0.12	0.12	0.00	0.00
PHGDH	0.00	0.00	0.00	0.25	0.00	0.31	0.13	0.27	0.16	0.00	0.00	0.35	0.28	0.21	0.34	0.22	0.00	0.14	0.29	0.00	0.00
SLC6A15	0.00	0.00	0.11	0.29	0.00	0.38	0.15	0.00	0.00	0.00	0.29	0.42	0.34	0.28	0.37	0.18	0.00	0.11	0.17	0.00	0.00
SLC38A5	0.00	0.00	0.00	0.24	0.00	0.38	0.00	0.12	0.14	0.00	0.18	0.36	0.27	0.19	0.37	0.19	0.00	0.11	0.21	0.00	0.00
CLCN3	0.00	0.00	0.11	0.23	0.00	0.20	0.15	0.16	0.19	0.00	0.20	0.20	0.16	0.16	0.18	0.00	0.00	0.00	0.00	0.12	0.00
ITPR2	0.00	0.00	0.23	0.30	0.00	0.16	0.24	0.15	0.11	0.00	0.12	0.16	0.18	0.17	0.21	0.00	0.00	0.00	0.00	0.13	0.00
SCG2	0.00	0.00	0.18	0.28	0.00	0.24	0.19	0.23	0.15	0.00	0.00	0.27	0.23	0.22	0.33	0.14	0.00	0.00	0.00	0.10	0.00
CACNA1D	0.00	0.00	0.11	0.17	0.00	0.12	0.11	0.12	0.12	0.00	0.00	0.19	0.11	0.20	0.22	0.12	0.00	0.00	0.00	0.14	0.00
CADPS	0.00	0.00	0.16	0.30	0.00	0.30	0.20	0.25	0.00	0.00	0.10	0.30	0.20	0.22	0.30	0.12	0.00	0.00	0.00	0.14	0.00
AHI1	0.00	0.00	0.13	0.24	0.00	0.26	0.15	0.21	0.19	0.00	0.00	0.25	0.15	0.00	0.28	0.10	0.17	0.00	0.00	0.12	0.00
SHROOM3	0.00	0.00	0.13	0.28	0.00	0.33	0.17	0.21	0.11	0.00	0.00	0.20	0.11	0.00	0.28	0.12	0.15	0.00	0.00	0.12	0.00
PCLO	0.00	0.00	0.12	0.25	0.00	0.28	0.15	0.14	0.00	0.00	0.00	0.31	0.24	0.28	0.30	0.12	0.14	0.00	0.00	0.13	0.00
UNC13B	0.00	0.00	0.12	0.25	0.00	0.24	0.16	0.25	0.00	0.00	0.00	0.23	0.15	0.21	0.27	0.11	0.14	0.00	0.00	0.17	0.00
SST	0.00	0.00	0.14	0.15	0.00	0.00	0.11	0.10	0.13	0.00	0.00	0.11	0.18	0.19	0.14	0.13	0.13	0.00	0.00	0.12	0.00
KCNB1	0.00	0.00	0.00	0.14	0.00	0.14	0.00	0.18	0.16	0.00	0.15	0.20	0.14	0.18	0.22	0.13	0.15	0.00	0.00	0.18	0.00
HCRTR2	0.11	0.11	0.23	0.23	0.00	0.00	0.19	0.00	0.00	0.00	0.00	0.16	0.18	0.11	0.24	0.11	0.00	0.21	0.00	0.12	0.00
HCRTR1	0.11	0.11	0.22	0.21	0.00	0.00	0.17	0.00	0.00	0.00	0.00	0.16	0.17	0.11	0.23	0.11	0.00	0.22	0.00	0.13	0.00
NHLH2	0.00	0.00	0.17	0.29	0.00	0.28	0.20	0.30	0.00	0.00	0.00	0.25	0.16	0.12	0.44	0.22	0.00	0.21	0.00	0.11	0.00
CCK	0.00	0.00	0.17	0.17	0.00	0.00	0.15	0.10	0.10	0.00	0.00	0.13	0.13	0.13	0.21	0.14	0.00	0.17	0.00	0.14	0.00
CNTF	0.00	0.00	0.13	0.21	0.00	0.00	0.18	0.14	0.23	0.00	0.00	0.17	0.17	0.19	0.33	0.27	0.00	0.15	0.00	0.11	0.00
CCKBR	0.00	0.00	0.24	0.24	0.00	0.00	0.21	0.13	0.15	0.00	0.00	0.12	0.12	0.13	0.19	0.11	0.00	0.13	0.00	0.11	0.00
TRPM5	0.00	0.00	0.27	0.32	0.00	0.16	0.26	0.18	0.14	0.00	0.17	0.14	0.00	0.00	0.23	0.00	0.13	0.14	0.00	0.18	0.00
BBS2	0.00	0.00	0.17	0.25	0.00	0.26	0.16	0.12	0.15	0.00	0.11	0.19	0.00	0.00	0.24	0.00	0.19	0.15	0.00	0.12	0.00
BDKRB2	0.21	0.21	0.19	0.17	0.16	0.00	0.14	0.00	0.13	0.00	0.00	0.00	0.00	0.11	0.12	0.00	0.11	0.11	0.00	0.11	0.00
SYT7	0.00	0.00	0.14	0.27	0.00	0.24	0.18	0.25	0.00	0.00	0.14	0.20	0.00	0.10	0.22	0.00	0.14	0.12	0.00	0.24	0.00
ATP2C1	0.00	0.00	0.10	0.23	0.00	0.28	0.12	0.17	0.13	0.00	0.21	0.20	0.11	0.00	0.16	0.00	0.00	0.00	0.15	0.13	0.00
INPP4A	0.00	0.00	0.16	0.33	0.00	0.40	0.19	0.22	0.11	0.00	0.00	0.30	0.15	0.15	0.24	0.00	0.00	0.00	0.20	0.11	0.00
SLC30A5	0.00	0.00	0.12	0.33	0.00	0.48	0.17	0.22	0.00	0.00	0.25	0.25	0.11	0.13	0.21	0.00	0.00	0.00	0.19	0.12	0.00
SPHK1	0.15	0.15	0.21	0.19	0.00	0.00	0.15	0.13	0.21	0.00	0.00	0.12	0.00	0.00	0.15	0.10	0.00	0.00	0.15	0.10	0.00
FIG4	0.00	0.00	0.00	0.22	0.00	0.30	0.12	0.21	0.16	0.00	0.00	0.25	0.14	0.12	0.39	0.27	0.00	0.00	0.16	0.14	0.00
NEU2	0.00	0.00	0.00	0.25	0.00	0.33	0.14	0.20	0.18	0.00	0.00	0.26	0.13	0.11	0.24	0.10	0.00	0.00	0.17	0.11	0.00
PRKCSH	0.00	0.00	0.16	0.32	0.00	0.34	0.21	0.18	0.12	0.00	0.17	0.17	0.00	0.00	0.17	0.00	0.14	0.00	0.14	0.16	0.00
DGKQ	0.00	0.00	0.23	0.37	0.00	0.36	0.25	0.19	0.00	0.00	0.00	0.20	0.16	0.10	0.17	0.00	0.11	0.00	0.16	0.11	0.00
NOS1AP	0.00	0.00	0.11	0.19	0.00	0.17	0.14	0.00	0.13	0.00	0.00	0.18	0.12	0.13	0.22	0.00	0.22	0.00	0.10	0.11	0.00
GSK3A	0.00	0.00	0.00	0.15	0.00	0.12	0.10	0.24	0.22	0.00	0.00	0.14	0.12	0.14	0.16	0.00	0.12	0.00	0.11	0.18	0.00
CREM	0.00	0.00	0.00	0.19	0.00	0.17	0.12	0.24	0.12	0.00	0.00	0.15	0.15	0.17	0.19	0.00	0.12	0.00	0.12	0.13	0.00
MTMR14	0.00	0.00	0.14	0.28	0.00	0.34	0.17	0.20	0.11	0.00	0.10	0.14	0.00	0.00	0.13	0.00	0.00	0.12	0.17	0.11	0.00

CRYM	0.00	0.00	0.16	0.34	0.00	0.41	0.20	0.24	0.13	0.00	0.00	0.28	0.14	0.00	0.23	0.00	0.00	0.14	0.26	0.14	0.00
SCD5	0.00	0.00	0.13	0.31	0.00	0.46	0.15	0.15	0.00	0.00	0.14	0.34	0.12	0.00	0.22	0.00	0.00	0.23	0.34	0.14	0.00
SLC7A7	0.00	0.00	0.00	0.23	0.00	0.29	0.13	0.11	0.12	0.00	0.24	0.23	0.15	0.00	0.24	0.00	0.00	0.11	0.19	0.12	0.00
PLD1	0.00	0.00	0.13	0.16	0.00	0.11	0.10	0.20	0.13	0.00	0.00	0.14	0.00	0.12	0.11	0.00	0.00	0.11	0.12	0.12	0.00
GAPDH	0.00	0.00	0.00	0.17	0.00	0.14	0.11	0.20	0.17	0.00	0.00	0.19	0.14	0.11	0.18	0.00	0.00	0.12	0.18	0.16	0.00
INSIG1	0.00	0.00	0.11	0.20	0.00	0.20	0.14	0.27	0.14	0.00	0.13	0.00	0.00	0.00	0.14	0.00	0.16	0.13	0.25	0.14	0.00
ERN1	0.00	0.00	0.12	0.23	0.00	0.23	0.14	0.26	0.22	0.00	0.15	0.00	0.00	0.00	0.13	0.00	0.17	0.15	0.15	0.22	0.00
SGMS2	0.00	0.00	0.13	0.26	0.00	0.31	0.16	0.24	0.21	0.00	0.00	0.17	0.00	0.00	0.11	0.00	0.16	0.12	0.35	0.17	0.00
TNMD	0.00	0.00	0.11	0.25	0.00	0.30	0.15	0.19	0.18	0.00	0.00	0.18	0.00	0.00	0.22	0.00	0.23	0.11	0.18	0.19	0.00
AKT1S1	0.00	0.00	0.12	0.25	0.00	0.25	0.17	0.39	0.23	0.00	0.00	0.14	0.00	0.00	0.16	0.00	0.15	0.20	0.18	0.24	0.00
NPC1L1	0.00	0.00	0.13	0.22	0.00	0.16	0.17	0.21	0.18	0.00	0.00	0.11	0.00	0.00	0.13	0.00	0.13	0.12	0.30	0.11	0.00
LAMTOR1	0.00	0.00	0.15	0.34	0.00	0.42	0.19	0.38	0.13	0.00	0.00	0.13	0.00	0.00	0.16	0.00	0.11	0.17	0.13	0.14	0.00
MLXIPL	0.00	0.00	0.11	0.21	0.00	0.20	0.14	0.29	0.13	0.00	0.00	0.11	0.00	0.00	0.12	0.00	0.30	0.31	0.34	0.39	0.00
OSBPL8	0.00	0.00	0.15	0.31	0.00	0.36	0.20	0.37	0.16	0.00	0.00	0.11	0.00	0.00	0.13	0.00	0.27	0.13	0.29	0.26	0.00
TUB	0.00	0.00	0.16	0.26	0.00	0.23	0.18	0.17	0.12	0.00	0.00	0.17	0.00	0.00	0.23	0.00	0.28	0.29	0.24	0.30	0.00
S100A16	0.00	0.00	0.15	0.33	0.00	0.43	0.19	0.29	0.13	0.00	0.00	0.21	0.00	0.00	0.17	0.00	0.30	0.28	0.26	0.33	0.00
CYP8B1	0.00	0.00	0.14	0.27	0.00	0.26	0.19	0.24	0.11	0.00	0.00	0.15	0.00	0.00	0.13	0.00	0.12	0.10	0.33	0.12	0.00
CERS4	0.00	0.00	0.12	0.28	0.00	0.46	0.12	0.15	0.00	0.00	0.13	0.21	0.00	0.00	0.17	0.00	0.22	0.13	0.33	0.18	0.00
SGIP1	0.00	0.00	0.25	0.43	0.00	0.41	0.31	0.15	0.00	0.00	0.15	0.25	0.00	0.00	0.21	0.00	0.15	0.19	0.11	0.15	0.00
SLC37A4	0.00	0.00	0.00	0.21	0.00	0.24	0.12	0.16	0.20	0.00	0.11	0.12	0.00	0.00	0.14	0.00	0.22	0.17	0.30	0.33	0.00
SLC6A19	0.00	0.00	0.00	0.21	0.00	0.27	0.11	0.11	0.00	0.00	0.23	0.17	0.15	0.00	0.18	0.00	0.10	0.17	0.20	0.18	0.00
CLIC5	0.00	0.00	0.00	0.26	0.00	0.43	0.10	0.16	0.00	0.00	0.20	0.22	0.13	0.00	0.19	0.00	0.10	0.13	0.15	0.14	0.00
SLC5A2	0.00	0.00	0.00	0.17	0.00	0.23	0.00	0.11	0.13	0.00	0.15	0.12	0.14	0.00	0.15	0.00	0.33	0.14	0.21	0.37	0.00
BDNF-AS1	0.00	0.00	0.00	0.20	0.00	0.28	0.11	0.17	0.00	0.00	0.00	0.22	0.13	0.17	0.21	0.00	0.24	0.15	0.12	0.17	0.00
EIF2B4	0.00	0.00	0.00	0.20	0.00	0.27	0.00	0.17	0.12	0.00	0.00	0.20	0.11	0.12	0.21	0.00	0.14	0.13	0.11	0.17	0.00
BCL2L2	0.00	0.00	0.11	0.22	0.00	0.23	0.15	0.28	0.21	0.00	0.10	0.18	0.12	0.14	0.22	0.00	0.00	0.00	0.00	0.00	0.22
CNTD1	0.00	0.00	0.20	0.33	0.00	0.31	0.25	0.23	0.15	0.00	0.10	0.18	0.00	0.11	0.22	0.11	0.00	0.00	0.00	0.00	0.20
PAK7	0.00	0.00	0.11	0.26	0.00	0.35	0.14	0.29	0.16	0.00	0.00	0.24	0.15	0.15	0.26	0.13	0.00	0.00	0.00	0.00	0.13
HRK	0.00	0.00	0.10	0.25	0.00	0.28	0.16	0.28	0.18	0.00	0.00	0.20	0.10	0.14	0.27	0.11	0.00	0.00	0.00	0.00	0.23
CRMP1	0.00	0.00	0.10	0.26	0.00	0.32	0.15	0.24	0.17	0.00	0.00	0.37	0.27	0.24	0.39	0.21	0.00	0.00	0.00	0.00	0.12
LSG1	0.00	0.00	0.12	0.35	0.00	0.55	0.16	0.26	0.10	0.00	0.00	0.51	0.36	0.39	0.50	0.31	0.00	0.00	0.00	0.00	0.16
MAPK10	0.00	0.00	0.16	0.23	0.00	0.20	0.16	0.23	0.21	0.00	0.00	0.26	0.18	0.25	0.27	0.16	0.00	0.00	0.00	0.00	0.13
DYNC1I2	0.00	0.00	0.14	0.35	0.00	0.44	0.21	0.25	0.11	0.00	0.00	0.35	0.21	0.15	0.36	0.13	0.00	0.00	0.00	0.00	0.20
PAWR	0.00	0.00	0.15	0.25	0.00	0.20	0.19	0.32	0.32	0.00	0.00	0.18	0.13	0.16	0.20	0.11	0.00	0.00	0.00	0.00	0.12
CASP4	0.00	0.00	0.10	0.22	0.00	0.21	0.15	0.20	0.20	0.00	0.00	0.14	0.11	0.13	0.20	0.13	0.00	0.00	0.00	0.00	0.12
HPCA	0.00	0.00	0.21	0.35	0.00	0.36	0.22	0.20	0.00	0.00	0.10	0.40	0.30	0.34	0.34	0.19	0.00	0.00	0.00	0.00	0.11
KIAA1279	0.00	0.00	0.11	0.32	0.00	0.53	0.15	0.20	0.00	0.00	0.11	0.39	0.21	0.13	0.44	0.16	0.00	0.00	0.00	0.00	0.17
TMPRSS5	0.00	0.00	0.14	0.38	0.00	0.54	0.20	0.18	0.00	0.00	0.26	0.33	0.18	0.12	0.34	0.16	0.00	0.00	0.00	0.00	0.14
CEP112	0.00	0.00	0.21	0.42	0.00	0.46	0.29	0.21	0.00	0.00	0.13	0.41	0.33	0.27	0.40	0.20	0.00	0.00	0.00	0.00	0.10
MMD	0.00	0.00	0.32	0.57	0.00	0.59	0.40	0.24	0.00	0.00	0.27	0.36	0.16	0.17	0.36	0.20	0.00	0.00	0.00	0.00	0.16

DYNLT1	0.00	0.00	0.15	0.32	0.00	0.39	0.18	0.22	0.00	0.00	0.13	0.27	0.16	0.10	0.33	0.12	0.00	0.00	0.00	0.00	0.10
INTS4	0.00	0.00	0.18	0.45	0.00	0.67	0.23	0.29	0.00	0.00	0.11	0.44	0.32	0.25	0.36	0.14	0.00	0.00	0.00	0.00	0.11
KIAA0513	0.00	0.00	0.18	0.45	0.00	0.67	0.23	0.29	0.00	0.00	0.11	0.44	0.32	0.25	0.36	0.14	0.00	0.00	0.00	0.00	0.11
ITM2B	0.00	0.00	0.00	0.22	0.00	0.29	0.12	0.17	0.19	0.00	0.16	0.30	0.16	0.21	0.26	0.12	0.00	0.00	0.00	0.00	0.12
KIF5C	0.00	0.00	0.10	0.28	0.00	0.34	0.17	0.14	0.00	0.00	0.00	0.29	0.17	0.10	0.30	0.12	0.00	0.12	0.00	0.00	0.18
SGPL1	0.10	0.10	0.22	0.30	0.00	0.26	0.21	0.26	0.24	0.00	0.00	0.18	0.00	0.00	0.19	0.00	0.00	0.00	0.24	0.00	0.11
APBB2	0.00	0.00	0.14	0.31	0.00	0.35	0.21	0.25	0.17	0.00	0.13	0.21	0.00	0.12	0.20	0.00	0.00	0.00	0.12	0.00	0.11
GNB1L	0.00	0.00	0.11	0.28	0.00	0.35	0.16	0.23	0.11	0.00	0.00	0.23	0.12	0.11	0.23	0.00	0.00	0.00	0.12	0.00	0.11
CCBL2	0.00	0.00	0.13	0.32	0.00	0.41	0.18	0.12	0.16	0.00	0.00	0.39	0.22	0.19	0.29	0.00	0.00	0.00	0.28	0.00	0.13
DHCR24	0.00	0.00	0.12	0.23	0.00	0.24	0.16	0.28	0.25	0.00	0.00	0.24	0.16	0.16	0.20	0.00	0.00	0.00	0.27	0.00	0.11
CYB561	0.00	0.00	0.14	0.34	0.00	0.43	0.18	0.16	0.00	0.00	0.17	0.28	0.14	0.11	0.25	0.00	0.00	0.00	0.22	0.00	0.23
SLC9A6	0.00	0.00	0.10	0.26	0.00	0.36	0.13	0.14	0.00	0.00	0.30	0.27	0.18	0.13	0.19	0.00	0.00	0.00	0.12	0.00	0.11
ATCAY	0.00	0.00	0.13	0.33	0.00	0.44	0.19	0.18	0.00	0.00	0.00	0.50	0.35	0.32	0.45	0.23	0.00	0.00	0.13	0.00	0.13
GMPPB	0.00	0.00	0.12	0.34	0.00	0.50	0.18	0.17	0.00	0.00	0.00	0.43	0.21	0.24	0.34	0.17	0.00	0.00	0.13	0.00	0.11
AGBL5	0.00	0.00	0.12	0.33	0.00	0.54	0.14	0.20	0.00	0.00	0.00	0.37	0.18	0.13	0.29	0.12	0.00	0.00	0.11	0.00	0.14
AGBL4	0.00	0.00	0.12	0.33	0.00	0.54	0.14	0.16	0.00	0.00	0.00	0.38	0.19	0.14	0.29	0.12	0.00	0.00	0.12	0.00	0.10
ADNP2	0.00	0.00	0.00	0.31	0.00	0.50	0.13	0.25	0.22	0.00	0.00	0.46	0.33	0.33	0.37	0.19	0.00	0.00	0.14	0.00	0.10
FTH1	0.00	0.00	0.00	0.21	0.00	0.20	0.14	0.17	0.16	0.00	0.00	0.21	0.11	0.12	0.24	0.11	0.00	0.00	0.21	0.00	0.15
IREB2	0.00	0.00	0.00	0.20	0.00	0.20	0.12	0.20	0.16	0.00	0.00	0.21	0.13	0.15	0.26	0.12	0.00	0.00	0.26	0.00	0.14
SELM	0.00	0.00	0.00	0.29	0.00	0.47	0.11	0.23	0.14	0.00	0.00	0.30	0.15	0.16	0.24	0.10	0.00	0.00	0.14	0.00	0.12
CLN8	0.00	0.00	0.00	0.19	0.00	0.18	0.13	0.00	0.26	0.00	0.11	0.33	0.28	0.28	0.38	0.33	0.00	0.00	0.15	0.00	0.12
PAFAH2	0.00	0.00	0.00	0.22	0.00	0.29	0.12	0.15	0.15	0.00	0.00	0.25	0.15	0.11	0.16	0.00	0.11	0.00	0.31	0.00	0.14
PDSS2	0.00	0.00	0.00	0.21	0.00	0.25	0.15	0.19	0.23	0.00	0.10	0.13	0.00	0.00	0.18	0.15	0.11	0.00	0.17	0.00	0.16
KCNJ8	0.00	0.00	0.12	0.18	0.00	0.14	0.13	0.12	0.17	0.00	0.12	0.14	0.00	0.00	0.12	0.00	0.00	0.10	0.12	0.00	0.12
FIS1	0.00	0.00	0.12	0.29	0.00	0.37	0.16	0.25	0.11	0.00	0.14	0.13	0.00	0.00	0.16	0.00	0.00	0.21	0.15	0.00	0.50
ABCC9	0.00	0.00	0.11	0.18	0.00	0.13	0.14	0.12	0.17	0.00	0.12	0.12	0.00	0.00	0.10	0.00	0.00	0.12	0.12	0.00	0.10
HSPA9	0.00	0.00	0.11	0.25	0.00	0.29	0.14	0.26	0.21	0.00	0.00	0.21	0.12	0.00	0.21	0.00	0.00	0.13	0.12	0.00	0.25
ABCD3	0.00	0.00	0.10	0.27	0.00	0.38	0.14	0.10	0.00	0.00	0.17	0.25	0.13	0.00	0.25	0.00	0.00	0.13	0.26	0.00	0.23
PEX13	0.00	0.00	0.14	0.31	0.00	0.39	0.19	0.13	0.00	0.00	0.15	0.23	0.12	0.00	0.28	0.00	0.00	0.13	0.20	0.00	0.26
PRODH	0.00	0.00	0.00	0.21	0.00	0.21	0.14	0.23	0.19	0.00	0.00	0.17	0.18	0.11	0.15	0.00	0.00	0.12	0.20	0.00	0.19
PPP3R1	0.00	0.00	0.00	0.19	0.00	0.19	0.13	0.25	0.23	0.00	0.00	0.16	0.11	0.15	0.17	0.00	0.00	0.12	0.11	0.00	0.12
HSD17B10	0.00	0.00	0.00	0.24	0.00	0.28	0.14	0.18	0.18	0.00	0.00	0.29	0.19	0.18	0.21	0.00	0.00	0.12	0.34	0.00	0.23
MRPL51	0.00	0.00	0.00	0.31	0.00	0.57	0.11	0.18	0.00	0.00	0.14	0.35	0.19	0.18	0.29	0.00	0.00	0.14	0.15	0.00	0.31
HSD17B4	0.00	0.00	0.00	0.21	0.00	0.23	0.13	0.13	0.11	0.00	0.00	0.23	0.15	0.00	0.20	0.10	0.00	0.13	0.40	0.00	0.21
NGB	0.00	0.00	0.00	0.18	0.00	0.20	0.00	0.18	0.20	0.00	0.00	0.27	0.23	0.21	0.30	0.16	0.00	0.17	0.14	0.00	0.15
NMNAT3	0.00	0.00	0.00	0.23	0.00	0.37	0.00	0.25	0.19	0.00	0.00	0.30	0.13	0.11	0.29	0.14	0.00	0.19	0.36	0.00	0.29
KIF5B	0.00	0.00	0.00	0.22	0.00	0.28	0.12	0.20	0.00	0.00	0.00	0.25	0.15	0.14	0.28	0.11	0.00	0.13	0.00	0.13	0.16
DEGS1	0.00	0.00	0.14	0.29	0.00	0.38	0.15	0.29	0.20	0.00	0.00	0.22	0.00	0.00	0.19	0.00	0.00	0.14	0.35	0.16	0.15
ULK1	0.00	0.00	0.14	0.30	0.00	0.34	0.20	0.34	0.13	0.00	0.00	0.17	0.00	0.00	0.26	0.00	0.00	0.19	0.14	0.11	0.13
PPRC1	0.00	0.00	0.13	0.30	0.00	0.32	0.20	0.34	0.12	0.00	0.00	0.12	0.00	0.00	0.17	0.00	0.00	0.24	0.21	0.10	0.31

ATG7	0.00	0.00	0.10	0.20	0.00	0.17	0.14	0.26	0.19	0.00	0.00	0.11	0.00	0.00	0.18	0.00	0.00	0.25	0.23	0.19	0.22
STBD1	0.00	0.00	0.11	0.37	0.00	0.63	0.15	0.17	0.00	0.00	0.17	0.23	0.00	0.00	0.16	0.00	0.00	0.15	0.20	0.20	0.14
ARV1	0.00	0.00	0.13	0.30	0.00	0.42	0.16	0.18	0.00	0.00	0.24	0.17	0.00	0.00	0.11	0.00	0.00	0.14	0.42	0.13	0.10
VDAC1	0.00	0.00	0.00	0.21	0.00	0.22	0.12	0.18	0.15	0.00	0.17	0.17	0.00	0.00	0.17	0.00	0.00	0.24	0.17	0.11	0.47
SLC25A4	0.00	0.00	0.00	0.19	0.00	0.20	0.11	0.15	0.19	0.00	0.12	0.15	0.00	0.00	0.14	0.00	0.00	0.28	0.20	0.11	0.47
PGAM1	0.00	0.00	0.00	0.26	0.00	0.41	0.10	0.20	0.15	0.00	0.00	0.28	0.10	0.00	0.15	0.00	0.00	0.18	0.27	0.18	0.15
SLC27A2	0.00	0.00	0.00	0.25	0.00	0.36	0.12	0.12	0.00	0.00	0.14	0.26	0.12	0.00	0.17	0.00	0.00	0.22	0.42	0.11	0.21
GAMT	0.00	0.00	0.00	0.16	0.00	0.25	0.00	0.14	0.18	0.00	0.00	0.28	0.15	0.13	0.21	0.00	0.00	0.27	0.33	0.15	0.20
PPP3CA	0.00	0.00	0.00	0.14	0.00	0.13	0.00	0.22	0.22	0.00	0.00	0.16	0.13	0.19	0.15	0.00	0.00	0.13	0.10	0.10	0.12
EEF2	0.00	0.00	0.00	0.20	0.00	0.29	0.00	0.33	0.17	0.00	0.00	0.21	0.13	0.17	0.17	0.00	0.00	0.22	0.18	0.18	0.10
FLAD1	0.00	0.00	0.00	0.23	0.00	0.38	0.00	0.14	0.13	0.00	0.00	0.31	0.15	0.00	0.23	0.13	0.00	0.16	0.33	0.11	0.29
OGDH	0.00	0.00	0.00	0.23	0.00	0.33	0.00	0.17	0.00	0.00	0.00	0.36	0.22	0.17	0.27	0.12	0.00	0.26	0.34	0.14	0.41
PLIN3	0.00	0.00	0.16	0.31	0.00	0.31	0.21	0.23	0.00	0.00	0.16	0.00	0.00	0.00	0.11	0.00	0.11	0.22	0.26	0.19	0.11
RBP7	0.00	0.00	0.13	0.30	0.00	0.37	0.17	0.22	0.00	0.00	0.00	0.22	0.00	0.00	0.25	0.00	0.12	0.25	0.38	0.18	0.10
CIDEB	0.00	0.00	0.11	0.30	0.00	0.39	0.18	0.33	0.00	0.00	0.00	0.12	0.00	0.00	0.12	0.00	0.17	0.25	0.35	0.23	0.20
SIRT3	0.00	0.00	0.00	0.20	0.00	0.24	0.10	0.26	0.19	0.00	0.00	0.13	0.00	0.00	0.13	0.00	0.13	0.31	0.31	0.17	0.33
CYTB	0.00	0.00	0.00	0.20	0.00	0.24	0.11	0.18	0.14	0.00	0.00	0.15	0.00	0.00	0.12	0.00	0.14	0.29	0.35	0.19	0.45
CTH	0.00	0.00	0.00	0.18	0.00	0.18	0.11	0.16	0.21	0.00	0.00	0.14	0.00	0.00	0.16	0.00	0.14	0.13	0.31	0.17	0.11
GSTA4	0.00	0.00	0.00	0.18	0.00	0.19	0.11	0.16	0.14	0.00	0.00	0.15	0.00	0.00	0.13	0.00	0.13	0.12	0.28	0.13	0.16
SQLE	0.00	0.00	0.00	0.26	0.00	0.35	0.14	0.28	0.19	0.00	0.00	0.20	0.00	0.00	0.12	0.00	0.15	0.12	0.36	0.18	0.10
GK	0.00	0.00	0.00	0.24	0.00	0.29	0.13	0.15	0.15	0.00	0.00	0.18	0.00	0.00	0.15	0.00	0.24	0.29	0.37	0.31	0.16
PYGM	0.00	0.00	0.00	0.19	0.00	0.19	0.11	0.13	0.16	0.00	0.00	0.17	0.00	0.00	0.18	0.00	0.13	0.23	0.27	0.20	0.13
OXCT1	0.00	0.00	0.00	0.16	0.00	0.25	0.00	0.17	0.14	0.00	0.00	0.27	0.12	0.00	0.18	0.00	0.20	0.38	0.50	0.32	0.31
GCLM	0.00	0.00	0.00	0.14	0.00	0.14	0.00	0.13	0.12	0.00	0.00	0.16	0.12	0.00	0.15	0.00	0.13	0.13	0.25	0.14	0.15
AK1	0.00	0.00	0.00	0.18	0.00	0.20	0.00	0.15	0.16	0.00	0.00	0.22	0.12	0.00	0.18	0.00	0.14	0.33	0.35	0.25	0.23
GATM	0.00	0.00	0.00	0.16	0.00	0.25	0.00	0.15	0.18	0.00	0.00	0.24	0.13	0.00	0.18	0.00	0.17	0.20	0.35	0.17	0.15
UQCRFS1	0.00	0.00	0.00	0.21	0.00	0.38	0.00	0.17	0.13	0.00	0.00	0.25	0.11	0.00	0.17	0.00	0.11	0.23	0.31	0.12	0.45
SLC25A22	0.00	0.00	0.00	0.23	0.00	0.43	0.00	0.13	0.00	0.00	0.14	0.27	0.11	0.00	0.18	0.00	0.17	0.25	0.32	0.30	0.31
MFN2	0.00	0.00	0.00	0.16	0.00	0.20	0.00	0.22	0.18	0.00	0.00	0.13	0.00	0.00	0.20	0.10	0.13	0.21	0.15	0.14	0.38
TMEM185B	0.30	0.30	0.42	0.53	0.19	0.45	0.38	0.13	0.00	0.00	0.20	0.26	0.00	0.00	0.26	0.00	0.00	0.00	0.00	0.00	0.00
FLNA	0.12	0.12	0.20	0.21	0.00	0.12	0.16	0.11	0.11	0.00	0.13	0.12	0.00	0.00	0.14	0.00	0.00	0.00	0.00	0.00	0.00
SNX6	0.11	0.11	0.24	0.40	0.00	0.38	0.29	0.33	0.15	0.00	0.15	0.17	0.00	0.00	0.12	0.00	0.00	0.00	0.00	0.00	0.00
LINC00272	0.12	0.12	0.15	0.35	0.00	0.52	0.16	0.27	0.15	0.00	0.11	0.18	0.00	0.00	0.12	0.00	0.00	0.00	0.00	0.00	0.00
LOC100288079	0.12	0.12	0.15	0.35	0.00	0.52	0.16	0.27	0.15	0.00	0.11	0.18	0.00	0.00	0.12	0.00	0.00	0.00	0.00	0.00	0.00
MIR802	0.12	0.12	0.24	0.36	0.00	0.39	0.23	0.45	0.25	0.00	0.00	0.20	0.11	0.00	0.18	0.00	0.00	0.00	0.00	0.00	0.00
GRM5	0.13	0.13	0.17	0.19	0.18	0.00	0.13	0.14	0.19	0.00	0.00	0.00	0.17	0.21	0.13	0.00	0.00	0.00	0.00	0.00	0.00
ADRA2C	0.12	0.12	0.17	0.17	0.15	0.00	0.13	0.00	0.16	0.00	0.00	0.12	0.12	0.11	0.21	0.00	0.00	0.00	0.00	0.00	0.00
TMED10	0.00	0.00	0.22	0.38	0.00	0.39	0.24	0.22	0.12	0.00	0.25	0.26	0.13	0.14	0.24	0.00	0.00	0.00	0.00	0.00	0.00
SSBP4	0.00	0.00	0.10	0.27	0.00	0.40	0.14	0.19	0.11	0.00	0.10	0.26	0.15	0.16	0.24	0.00	0.00	0.00	0.00	0.00	0.00
CHRNA9	0.00	0.00	0.19	0.29	0.00	0.18	0.24	0.22	0.16	0.00	0.15	0.14	0.11	0.13	0.24	0.00	0.00	0.00	0.00	0.00	0.00

PHACTR1	0.00	0.00	0.19	0.41	0.00	0.52	0.25	0.29	0.16	0.00	0.15	0.34	0.18	0.15	0.29	0.00	0.00	0.00	0.00	0.00	0.00
ARHGEF15	0.00	0.00	0.29	0.38	0.00	0.27	0.32	0.22	0.13	0.00	0.13	0.21	0.13	0.14	0.22	0.00	0.00	0.00	0.00	0.00	0.00
KCNJ12	0.00	0.00	0.11	0.20	0.00	0.19	0.12	0.15	0.11	0.00	0.15	0.22	0.13	0.14	0.23	0.00	0.00	0.00	0.00	0.00	0.00
LYNX1	0.00	0.00	0.25	0.40	0.00	0.31	0.32	0.30	0.27	0.00	0.16	0.22	0.15	0.15	0.24	0.00	0.00	0.00	0.00	0.00	0.00
KCNK6	0.00	0.00	0.10	0.25	0.00	0.30	0.14	0.12	0.13	0.00	0.23	0.24	0.11	0.12	0.25	0.00	0.00	0.00	0.00	0.00	0.00
SYNDIG1L	0.00	0.00	0.14	0.39	0.00	0.62	0.17	0.15	0.16	0.00	0.21	0.42	0.22	0.17	0.28	0.00	0.00	0.00	0.00	0.00	0.00
DGCR2	0.00	0.00	0.13	0.29	0.00	0.37	0.17	0.20	0.11	0.00	0.11	0.27	0.13	0.11	0.26	0.00	0.00	0.00	0.00	0.00	0.00
DNAJC5	0.00	0.00	0.15	0.29	0.00	0.28	0.18	0.20	0.13	0.00	0.17	0.25	0.17	0.17	0.29	0.00	0.00	0.00	0.00	0.00	0.00
DBNDD2	0.00	0.00	0.14	0.40	0.00	0.57	0.23	0.34	0.12	0.00	0.12	0.30	0.12	0.12	0.28	0.00	0.00	0.00	0.00	0.00	0.00
LZTFL1	0.00	0.00	0.18	0.39	0.00	0.55	0.22	0.39	0.22	0.00	0.13	0.25	0.10	0.11	0.21	0.00	0.00	0.00	0.00	0.00	0.00
FAM72B	0.00	0.00	0.18	0.36	0.00	0.41	0.25	0.24	0.18	0.00	0.13	0.29	0.11	0.21	0.21	0.00	0.00	0.00	0.00	0.00	0.00
PCDH11Y	0.00	0.00	0.14	0.34	0.00	0.42	0.20	0.18	0.11	0.00	0.17	0.29	0.15	0.10	0.30	0.00	0.00	0.00	0.00	0.00	0.00
SLC22A23	0.00	0.00	0.18	0.37	0.00	0.43	0.24	0.21	0.14	0.00	0.23	0.30	0.16	0.12	0.32	0.00	0.00	0.00	0.00	0.00	0.00
GABRP	0.00	0.00	0.26	0.41	0.00	0.29	0.35	0.24	0.14	0.00	0.18	0.23	0.17	0.14	0.24	0.00	0.00	0.00	0.00	0.00	0.00
NDP	0.00	0.00	0.12	0.21	0.00	0.19	0.15	0.12	0.17	0.10	0.11	0.17	0.00	0.00	0.25	0.11	0.00	0.00	0.00	0.00	0.00
SLIT3	0.00	0.00	0.15	0.24	0.00	0.14	0.21	0.20	0.10	0.00	0.15	0.14	0.15	0.00	0.32	0.21	0.00	0.00	0.00	0.00	0.00
NRG3	0.00	0.00	0.16	0.28	0.00	0.21	0.22	0.17	0.16	0.00	0.12	0.16	0.14	0.00	0.25	0.11	0.00	0.00	0.00	0.00	0.00
SPON2	0.00	0.00	0.20	0.34	0.00	0.28	0.27	0.26	0.26	0.00	0.12	0.19	0.10	0.00	0.28	0.14	0.00	0.00	0.00	0.00	0.00
RAB23	0.00	0.00	0.12	0.27	0.00	0.32	0.16	0.26	0.13	0.00	0.11	0.29	0.14	0.00	0.35	0.20	0.00	0.00	0.00	0.00	0.00
CLDN11	0.00	0.00	0.11	0.24	0.00	0.26	0.15	0.19	0.18	0.00	0.19	0.21	0.12	0.00	0.33	0.28	0.00	0.00	0.00	0.00	0.00
MAL2	0.00	0.00	0.13	0.31	0.00	0.38	0.19	0.23	0.12	0.00	0.27	0.22	0.12	0.00	0.25	0.13	0.00	0.00	0.00	0.00	0.00
UNC5C	0.00	0.00	0.16	0.26	0.00	0.17	0.23	0.24	0.11	0.00	0.13	0.19	0.23	0.00	0.37	0.24	0.00	0.00	0.00	0.00	0.00
NTN3	0.00	0.00	0.18	0.33	0.00	0.29	0.24	0.23	0.11	0.00	0.19	0.20	0.14	0.00	0.40	0.26	0.00	0.00	0.00	0.00	0.00
CCR10	0.00	0.00	0.28	0.34	0.00	0.20	0.29	0.15	0.16	0.00	0.17	0.17	0.11	0.00	0.32	0.23	0.00	0.00	0.00	0.00	0.00
CALCB	0.00	0.00	0.25	0.34	0.00	0.28	0.27	0.17	0.14	0.00	0.12	0.23	0.12	0.00	0.29	0.13	0.00	0.00	0.00	0.00	0.00
AGBL2	0.00	0.00	0.17	0.41	0.00	0.61	0.20	0.31	0.10	0.00	0.11	0.32	0.13	0.00	0.29	0.11	0.00	0.00	0.00	0.00	0.00
SPG21	0.00	0.00	0.19	0.39	0.00	0.43	0.26	0.19	0.11	0.00	0.19	0.28	0.14	0.00	0.34	0.16	0.00	0.00	0.00	0.00	0.00
ROBO3	0.00	0.00	0.14	0.24	0.00	0.16	0.20	0.19	0.11	0.00	0.13	0.15	0.18	0.00	0.35	0.24	0.00	0.00	0.00	0.00	0.00
GAS1	0.00	0.00	0.16	0.32	0.00	0.30	0.23	0.38	0.25	0.00	0.16	0.21	0.10	0.00	0.29	0.12	0.00	0.00	0.00	0.00	0.00
EMP1	0.00	0.00	0.15	0.32	0.00	0.39	0.20	0.35	0.24	0.00	0.15	0.23	0.13	0.00	0.31	0.18	0.00	0.00	0.00	0.00	0.00
GFRA4	0.00	0.00	0.30	0.43	0.00	0.31	0.36	0.19	0.11	0.00	0.20	0.21	0.10	0.00	0.34	0.11	0.00	0.00	0.00	0.00	0.00
FZD3	0.00	0.00	0.20	0.29	0.00	0.23	0.23	0.22	0.00	0.18	0.17	0.19	0.15	0.00	0.30	0.17	0.00	0.00	0.00	0.00	0.00
SCN10A	0.00	0.00	0.13	0.20	0.00	0.14	0.15	0.18	0.20	0.00	0.15	0.15	0.00	0.13	0.32	0.19	0.00	0.00	0.00	0.00	0.00
SCN11A	0.00	0.00	0.15	0.25	0.00	0.18	0.19	0.16	0.16	0.00	0.17	0.20	0.00	0.16	0.36	0.20	0.00	0.00	0.00	0.00	0.00
TREM2	0.00	0.00	0.27	0.40	0.00	0.24	0.35	0.22	0.25	0.00	0.22	0.18	0.00	0.10	0.28	0.17	0.00	0.00	0.00	0.00	0.00
NTRK2	0.11	0.11	0.11	0.13	0.00	0.00	0.11	0.12	0.13	0.00	0.00	0.00	0.16	0.20	0.18	0.12	0.00	0.00	0.00	0.00	0.00
DPYSL2	0.00	0.00	0.10	0.22	0.00	0.22	0.14	0.22	0.19	0.00	0.00	0.36	0.27	0.29	0.41	0.25	0.00	0.00	0.00	0.00	0.00
CDKL3	0.00	0.00	0.12	0.33	0.00	0.48	0.17	0.24	0.13	0.00	0.00	0.34	0.18	0.15	0.30	0.12	0.00	0.00	0.00	0.00	0.00
MAP3K13	0.00	0.00	0.18	0.36	0.00	0.40	0.24	0.25	0.12	0.00	0.00	0.29	0.15	0.17	0.34	0.16	0.00	0.00	0.00	0.00	0.00
LIMK1	0.00	0.00	0.13	0.23	0.00	0.22	0.16	0.23	0.18	0.00	0.00	0.18	0.17	0.15	0.21	0.12	0.00	0.00	0.00	0.00	0.00

PREX2	0.00	0.00	0.21	0.31	0.00	0.30	0.21	0.30	0.13	0.00	0.00	0.21	0.13	0.15	0.23	0.11	0.00	0.00	0.00	0.00	0.00
PDE4A	0.00	0.00	0.15	0.24	0.00	0.21	0.16	0.20	0.18	0.00	0.00	0.28	0.22	0.20	0.23	0.13	0.00	0.00	0.00	0.00	0.00
FGF20	0.00	0.00	0.18	0.32	0.00	0.29	0.25	0.26	0.18	0.00	0.00	0.26	0.19	0.15	0.29	0.11	0.00	0.00	0.00	0.00	0.00
EGR3	0.00	0.00	0.16	0.29	0.00	0.22	0.24	0.33	0.19	0.00	0.00	0.19	0.20	0.22	0.33	0.16	0.00	0.00	0.00	0.00	0.00
NDRG4	0.00	0.00	0.13	0.31	0.00	0.42	0.16	0.31	0.19	0.00	0.00	0.40	0.25	0.25	0.40	0.24	0.00	0.00	0.00	0.00	0.00
POU4F1	0.00	0.00	0.10	0.19	0.00	0.16	0.14	0.30	0.11	0.00	0.00	0.16	0.17	0.12	0.39	0.24	0.00	0.00	0.00	0.00	0.00
SP4	0.00	0.00	0.13	0.27	0.00	0.25	0.19	0.30	0.14	0.00	0.00	0.21	0.17	0.16	0.31	0.12	0.00	0.00	0.00	0.00	0.00
FEZF1	0.00	0.00	0.16	0.30	0.00	0.32	0.20	0.39	0.15	0.00	0.00	0.31	0.28	0.23	0.45	0.30	0.00	0.00	0.00	0.00	0.00
GMFB	0.00	0.00	0.13	0.29	0.00	0.34	0.17	0.22	0.25	0.00	0.00	0.30	0.20	0.21	0.30	0.24	0.00	0.00	0.00	0.00	0.00
C1QB	0.00	0.00	0.13	0.24	0.00	0.19	0.18	0.15	0.15	0.00	0.00	0.19	0.16	0.17	0.30	0.16	0.00	0.00	0.00	0.00	0.00
NTF4	0.00	0.00	0.14	0.20	0.00	0.11	0.17	0.19	0.16	0.00	0.00	0.13	0.14	0.19	0.27	0.15	0.00	0.00	0.00	0.00	0.00
GRIN3B	0.00	0.00	0.22	0.31	0.00	0.17	0.26	0.15	0.14	0.00	0.00	0.28	0.24	0.28	0.35	0.24	0.00	0.00	0.00	0.00	0.00
DAGLA	0.00	0.00	0.19	0.26	0.00	0.15	0.21	0.19	0.12	0.00	0.00	0.30	0.33	0.36	0.35	0.25	0.00	0.00	0.00	0.00	0.00
KALRN	0.00	0.00	0.21	0.32	0.00	0.28	0.23	0.22	0.11	0.00	0.00	0.35	0.29	0.32	0.33	0.16	0.00	0.00	0.00	0.00	0.00
NR2E1	0.00	0.00	0.16	0.27	0.00	0.23	0.20	0.35	0.21	0.00	0.00	0.29	0.28	0.25	0.39	0.24	0.00	0.00	0.00	0.00	0.00
CAMK2N1	0.00	0.00	0.20	0.39	0.00	0.39	0.27	0.36	0.15	0.00	0.00	0.32	0.20	0.27	0.23	0.11	0.00	0.00	0.00	0.00	0.00
DYX1C1	0.00	0.00	0.11	0.24	0.00	0.29	0.15	0.19	0.11	0.00	0.00	0.29	0.26	0.21	0.28	0.10	0.00	0.00	0.00	0.00	0.00
FOXP1	0.00	0.00	0.12	0.21	0.00	0.17	0.16	0.32	0.23	0.00	0.00	0.20	0.20	0.16	0.29	0.15	0.00	0.00	0.00	0.00	0.00
RCAN1	0.00	0.00	0.13	0.24	0.00	0.21	0.16	0.31	0.31	0.00	0.00	0.23	0.14	0.16	0.24	0.10	0.00	0.00	0.00	0.00	0.00
OPHN1	0.00	0.00	0.15	0.29	0.00	0.30	0.19	0.17	0.12	0.00	0.00	0.37	0.29	0.30	0.38	0.22	0.00	0.00	0.00	0.00	0.00
BPTF	0.00	0.00	0.10	0.31	0.00	0.41	0.16	0.43	0.15	0.00	0.00	0.24	0.14	0.15	0.31	0.13	0.00	0.00	0.00	0.00	0.00
GABRA5	0.00	0.00	0.16	0.25	0.00	0.10	0.23	0.12	0.10	0.00	0.00	0.21	0.25	0.28	0.23	0.14	0.00	0.00	0.00	0.00	0.00
FEV	0.00	0.00	0.11	0.22	0.00	0.18	0.16	0.27	0.16	0.00	0.00	0.26	0.25	0.18	0.40	0.22	0.00	0.00	0.00	0.00	0.00
SYNGAP1	0.00	0.00	0.20	0.32	0.00	0.23	0.26	0.19	0.12	0.00	0.00	0.32	0.28	0.36	0.33	0.18	0.00	0.00	0.00	0.00	0.00
BCL11B	0.00	0.00	0.15	0.25	0.00	0.19	0.20	0.35	0.17	0.00	0.00	0.14	0.19	0.15	0.28	0.18	0.00	0.00	0.00	0.00	0.00
NREP	0.00	0.00	0.19	0.37	0.00	0.40	0.24	0.28	0.19	0.00	0.00	0.39	0.28	0.28	0.40	0.22	0.00	0.00	0.00	0.00	0.00
GRID2	0.00	0.00	0.14	0.21	0.00	0.12	0.18	0.13	0.11	0.00	0.00	0.14	0.23	0.20	0.23	0.17	0.00	0.00	0.00	0.00	0.00
DPYSL3	0.00	0.00	0.11	0.27	0.00	0.35	0.16	0.25	0.17	0.00	0.00	0.41	0.28	0.24	0.47	0.26	0.00	0.00	0.00	0.00	0.00
ARC	0.00	0.00	0.12	0.21	0.00	0.17	0.13	0.21	0.16	0.00	0.00	0.23	0.30	0.39	0.24	0.18	0.00	0.00	0.00	0.00	0.00
SEP-05	0.00	0.00	0.12	0.29	0.00	0.36	0.16	0.23	0.11	0.00	0.00	0.35	0.21	0.21	0.34	0.11	0.00	0.00	0.00	0.00	0.00
KLHL2	0.00	0.00	0.13	0.36	0.00	0.54	0.18	0.38	0.14	0.00	0.00	0.34	0.19	0.18	0.34	0.18	0.00	0.00	0.00	0.00	0.00
MARCKS	0.00	0.00	0.13	0.23	0.00	0.25	0.14	0.24	0.15	0.00	0.00	0.24	0.19	0.19	0.24	0.10	0.00	0.00	0.00	0.00	0.00
NPAS3	0.00	0.00	0.14	0.28	0.00	0.29	0.19	0.20	0.21	0.00	0.00	0.33	0.29	0.26	0.37	0.19	0.00	0.00	0.00	0.00	0.00
PHYHIP	0.00	0.00	0.13	0.34	0.00	0.48	0.17	0.25	0.12	0.00	0.00	0.43	0.26	0.25	0.41	0.19	0.00	0.00	0.00	0.00	0.00
GRIN2C	0.00	0.00	0.18	0.26	0.00	0.12	0.22	0.14	0.12	0.00	0.00	0.22	0.25	0.27	0.29	0.20	0.00	0.00	0.00	0.00	0.00
CDNF	0.00	0.00	0.13	0.24	0.00	0.26	0.16	0.14	0.12	0.00	0.00	0.26	0.22	0.23	0.28	0.14	0.00	0.00	0.00	0.00	0.00
MAP3K12	0.00	0.00	0.12	0.29	0.00	0.33	0.17	0.29	0.16	0.00	0.00	0.24	0.17	0.13	0.32	0.14	0.00	0.00	0.00	0.00	0.00
TRIM2	0.00	0.00	0.10	0.31	0.00	0.42	0.17	0.27	0.14	0.00	0.00	0.41	0.29	0.33	0.47	0.29	0.00	0.00	0.00	0.00	0.00
OLIG1	0.00	0.00	0.10	0.20	0.00	0.18	0.16	0.25	0.16	0.00	0.00	0.22	0.26	0.17	0.44	0.43	0.00	0.00	0.00	0.00	0.00
DCLK1	0.00	0.00	0.14	0.27	0.00	0.32	0.16	0.28	0.13	0.00	0.00	0.37	0.35	0.37	0.36	0.21	0.00	0.00	0.00	0.00	0.00

SYNPO	0.00	0.00	0.11	0.23	0.00	0.27	0.15	0.20	0.13	0.00	0.00	0.21	0.23	0.19	0.25	0.21	0.00	0.00	0.00	0.00	0.00
TBCE	0.00	0.00	0.10	0.21	0.00	0.20	0.15	0.18	0.27	0.00	0.00	0.19	0.14	0.14	0.35	0.21	0.00	0.00	0.00	0.00	0.00
CHN1	0.00	0.00	0.18	0.31	0.00	0.31	0.22	0.24	0.12	0.00	0.00	0.29	0.24	0.20	0.38	0.21	0.00	0.00	0.00	0.00	0.00
METRN	0.00	0.00	0.21	0.36	0.00	0.32	0.27	0.31	0.27	0.00	0.00	0.44	0.37	0.34	0.55	0.42	0.00	0.00	0.00	0.00	0.00
NOVA1	0.00	0.00	0.15	0.35	0.00	0.35	0.23	0.28	0.16	0.00	0.00	0.29	0.17	0.16	0.38	0.19	0.00	0.00	0.00	0.00	0.00
LMO4	0.00	0.00	0.14	0.28	0.00	0.27	0.20	0.40	0.21	0.00	0.00	0.23	0.20	0.16	0.37	0.19	0.00	0.00	0.00	0.00	0.00
GRIN2D	0.00	0.00	0.16	0.24	0.00	0.11	0.21	0.12	0.13	0.00	0.00	0.20	0.22	0.24	0.28	0.19	0.00	0.00	0.00	0.00	0.00
RASGRF1	0.00	0.00	0.17	0.25	0.00	0.20	0.18	0.21	0.15	0.00	0.00	0.26	0.18	0.24	0.27	0.13	0.00	0.00	0.00	0.00	0.00
FABP7	0.00	0.00	0.15	0.23	0.00	0.18	0.18	0.24	0.19	0.00	0.00	0.29	0.31	0.25	0.39	0.31	0.00	0.00	0.00	0.00	0.00
MIR134	0.00	0.00	0.15	0.28	0.00	0.26	0.20	0.40	0.26	0.00	0.00	0.35	0.28	0.31	0.33	0.17	0.00	0.00	0.00	0.00	0.00
KRIT1	0.00	0.00	0.12	0.21	0.00	0.24	0.13	0.18	0.13	0.00	0.00	0.22	0.13	0.11	0.27	0.13	0.00	0.00	0.00	0.00	0.00
DCDC2	0.00	0.00	0.10	0.23	0.00	0.30	0.13	0.18	0.11	0.00	0.00	0.31	0.28	0.21	0.31	0.13	0.00	0.00	0.00	0.00	0.00
NPAS4	0.00	0.00	0.14	0.35	0.00	0.43	0.22	0.38	0.14	0.00	0.00	0.40	0.33	0.41	0.44	0.26	0.00	0.00	0.00	0.00	0.00
PRUNE2	0.00	0.00	0.14	0.34	0.00	0.44	0.19	0.37	0.26	0.00	0.00	0.32	0.16	0.16	0.38	0.17	0.00	0.00	0.00	0.00	0.00
DPYSL5	0.00	0.00	0.11	0.27	0.00	0.31	0.18	0.26	0.18	0.00	0.00	0.41	0.35	0.32	0.50	0.34	0.00	0.00	0.00	0.00	0.00
PITX3	0.00	0.00	0.10	0.17	0.00	0.11	0.13	0.20	0.17	0.00	0.00	0.19	0.22	0.14	0.30	0.17	0.00	0.00	0.00	0.00	0.00
MAP1B	0.00	0.00	0.11	0.23	0.00	0.22	0.15	0.18	0.12	0.00	0.00	0.32	0.29	0.29	0.41	0.26	0.00	0.00	0.00	0.00	0.00
CIT	0.00	0.00	0.14	0.27	0.00	0.32	0.16	0.25	0.11	0.00	0.00	0.35	0.34	0.30	0.38	0.23	0.00	0.00	0.00	0.00	0.00
LMO3	0.00	0.00	0.13	0.31	0.00	0.42	0.17	0.45	0.15	0.00	0.00	0.35	0.30	0.26	0.43	0.23	0.00	0.00	0.00	0.00	0.00
GDF7	0.00	0.00	0.14	0.27	0.00	0.23	0.21	0.22	0.12	0.00	0.00	0.22	0.21	0.18	0.37	0.23	0.00	0.00	0.00	0.00	0.00
VPS54	0.00	0.00	0.12	0.21	0.00	0.16	0.16	0.16	0.23	0.00	0.00	0.19	0.18	0.19	0.30	0.23	0.00	0.00	0.00	0.00	0.00
CACNA1G	0.00	0.00	0.10	0.17	0.00	0.13	0.11	0.17	0.17	0.00	0.00	0.18	0.10	0.15	0.21	0.13	0.00	0.00	0.00	0.00	0.00
NRN1	0.00	0.00	0.14	0.32	0.00	0.34	0.21	0.33	0.25	0.00	0.00	0.41	0.36	0.37	0.51	0.30	0.00	0.00	0.00	0.00	0.00
SERPINE2	0.00	0.00	0.11	0.19	0.00	0.15	0.14	0.18	0.17	0.00	0.00	0.22	0.18	0.17	0.29	0.17	0.00	0.00	0.00	0.00	0.00
PPP1R14C	0.00	0.00	0.21	0.35	0.00	0.39	0.22	0.20	0.14	0.00	0.00	0.34	0.22	0.20	0.32	0.11	0.00	0.00	0.00	0.00	0.00
MAPK8IP3	0.00	0.00	0.14	0.27	0.00	0.28	0.18	0.25	0.14	0.00	0.00	0.20	0.13	0.14	0.28	0.13	0.00	0.00	0.00	0.00	0.00
NELL2	0.00	0.00	0.24	0.44	0.00	0.45	0.30	0.27	0.00	0.00	0.14	0.50	0.39	0.40	0.53	0.28	0.00	0.00	0.00	0.00	0.00
AGBL3	0.00	0.00	0.15	0.36	0.00	0.56	0.17	0.18	0.00	0.00	0.12	0.38	0.17	0.14	0.36	0.15	0.00	0.00	0.00	0.00	0.00
OR6A2	0.00	0.00	0.33	0.44	0.00	0.27	0.37	0.22	0.00	0.00	0.21	0.32	0.25	0.21	0.49	0.27	0.00	0.00	0.00	0.00	0.00
FKBP15	0.00	0.00	0.14	0.39	0.00	0.55	0.21	0.21	0.00	0.00	0.13	0.32	0.18	0.12	0.35	0.13	0.00	0.00	0.00	0.00	0.00
PRSS12	0.00	0.00	0.11	0.30	0.00	0.37	0.18	0.15	0.00	0.00	0.14	0.47	0.36	0.40	0.46	0.27	0.00	0.00	0.00	0.00	0.00
PPP1R9A	0.00	0.00	0.22	0.36	0.00	0.32	0.25	0.23	0.00	0.00	0.11	0.37	0.26	0.30	0.32	0.13	0.00	0.00	0.00	0.00	0.00
NAV1	0.00	0.00	0.14	0.37	0.00	0.56	0.19	0.24	0.00	0.00	0.20	0.43	0.25	0.19	0.53	0.29	0.00	0.00	0.00	0.00	0.00
MEGF11	0.00	0.00	0.36	0.58	0.00	0.55	0.43	0.21	0.00	0.00	0.35	0.37	0.18	0.16	0.50	0.28	0.00	0.00	0.00	0.00	0.00
OR4E2	0.00	0.00	0.25	0.42	0.00	0.38	0.30	0.15	0.00	0.00	0.20	0.38	0.25	0.21	0.49	0.28	0.00	0.00	0.00	0.00	0.00
SCAMP4	0.00	0.00	0.21	0.46	0.00	0.60	0.27	0.15	0.00	0.00	0.21	0.48	0.25	0.25	0.40	0.15	0.00	0.00	0.00	0.00	0.00
DOC2A	0.00	0.00	0.17	0.33	0.00	0.32	0.22	0.20	0.00	0.00	0.11	0.36	0.22	0.27	0.34	0.13	0.00	0.00	0.00	0.00	0.00
CNTN3	0.00	0.00	0.18	0.37	0.00	0.43	0.25	0.17	0.00	0.00	0.25	0.42	0.28	0.27	0.48	0.25	0.00	0.00	0.00	0.00	0.00
NCDN	0.00	0.00	0.20	0.42	0.00	0.52	0.25	0.27	0.00	0.00	0.12	0.47	0.35	0.34	0.50	0.29	0.00	0.00	0.00	0.00	0.00
OPALIN	0.00	0.00	0.13	0.32	0.00	0.46	0.17	0.16	0.00	0.00	0.22	0.33	0.18	0.14	0.49	0.43	0.00	0.00	0.00	0.00	0.00

SLC44A5	0.00	0.00	0.11	0.33	0.00	0.52	0.15	0.11	0.00	0.00	0.19	0.52	0.30	0.26	0.56	0.43	0.00	0.00	0.00	0.00	0.00
CELSR3	0.00	0.00	0.12	0.25	0.00	0.29	0.16	0.22	0.00	0.00	0.12	0.27	0.25	0.15	0.44	0.29	0.00	0.00	0.00	0.00	0.00
GABRG1	0.00	0.00	0.20	0.32	0.00	0.20	0.28	0.11	0.00	0.00	0.12	0.27	0.26	0.27	0.29	0.14	0.00	0.00	0.00	0.00	0.00
GRID1	0.00	0.00	0.29	0.45	0.00	0.33	0.36	0.15	0.00	0.00	0.19	0.31	0.24	0.26	0.34	0.15	0.00	0.00	0.00	0.00	0.00
SESTD1	0.00	0.00	0.25	0.45	0.00	0.52	0.28	0.20	0.00	0.00	0.14	0.37	0.17	0.15	0.37	0.14	0.00	0.00	0.00	0.00	0.00
TMEM141	0.00	0.00	0.12	0.30	0.00	0.49	0.13	0.17	0.00	0.00	0.12	0.46	0.33	0.22	0.57	0.39	0.00	0.00	0.00	0.00	0.00
CBLN4	0.00	0.00	0.25	0.41	0.00	0.35	0.32	0.24	0.00	0.00	0.19	0.45	0.37	0.33	0.49	0.28	0.00	0.00	0.00	0.00	0.00
NECAB3	0.00	0.00	0.13	0.34	0.00	0.49	0.16	0.23	0.00	0.00	0.17	0.44	0.24	0.26	0.35	0.13	0.00	0.00	0.00	0.00	0.00
NECAB1	0.00	0.00	0.17	0.38	0.00	0.53	0.20	0.14	0.00	0.00	0.16	0.60	0.44	0.44	0.49	0.29	0.00	0.00	0.00	0.00	0.00
GFRAL	0.00	0.00	0.26	0.46	0.00	0.49	0.32	0.24	0.00	0.00	0.13	0.37	0.19	0.24	0.36	0.15	0.00	0.00	0.00	0.00	0.00
SLITRK3	0.00	0.00	0.26	0.48	0.00	0.53	0.33	0.21	0.00	0.00	0.29	0.54	0.32	0.26	0.62	0.37	0.00	0.00	0.00	0.00	0.00
SEMA6C	0.00	0.00	0.14	0.28	0.00	0.26	0.21	0.17	0.00	0.00	0.19	0.38	0.35	0.30	0.55	0.39	0.00	0.00	0.00	0.00	0.00
MDGA1	0.00	0.00	0.15	0.31	0.00	0.35	0.21	0.18	0.00	0.00	0.22	0.38	0.39	0.28	0.45	0.27	0.00	0.00	0.00	0.00	0.00
AMIGO3	0.00	0.00	0.19	0.41	0.00	0.51	0.26	0.22	0.00	0.00	0.28	0.47	0.27	0.32	0.47	0.26	0.00	0.00	0.00	0.00	0.00
LOC100133315	0.00	0.00	0.29	0.41	0.00	0.30	0.30	0.17	0.00	0.00	0.21	0.24	0.14	0.13	0.32	0.15	0.00	0.00	0.00	0.00	0.00
BZRAP1	0.00	0.00	0.19	0.43	0.00	0.53	0.26	0.17	0.00	0.00	0.12	0.41	0.26	0.30	0.38	0.15	0.00	0.00	0.00	0.00	0.00
SYNDIG1	0.00	0.00	0.34	0.45	0.00	0.20	0.43	0.12	0.00	0.00	0.23	0.42	0.32	0.40	0.43	0.26	0.00	0.00	0.00	0.00	0.00
CBLN1	0.00	0.00	0.24	0.39	0.00	0.32	0.29	0.17	0.00	0.00	0.16	0.40	0.38	0.30	0.47	0.26	0.00	0.00	0.00	0.00	0.00
KCNJ4	0.00	0.00	0.17	0.26	0.00	0.20	0.18	0.14	0.00	0.00	0.15	0.27	0.20	0.20	0.26	0.13	0.00	0.00	0.00	0.00	0.00
MOXD1	0.00	0.00	0.11	0.31	0.00	0.45	0.16	0.30	0.00	0.00	0.12	0.29	0.22	0.16	0.28	0.12	0.00	0.00	0.00	0.00	0.00
CNTN6	0.00	0.00	0.19	0.35	0.00	0.34	0.26	0.18	0.00	0.00	0.28	0.45	0.37	0.33	0.56	0.40	0.00	0.00	0.00	0.00	0.00
CNTNAP3	0.00	0.00	0.19	0.35	0.00	0.38	0.24	0.13	0.00	0.00	0.24	0.52	0.39	0.35	0.64	0.46	0.00	0.00	0.00	0.00	0.00
PROSAPIP1	0.00	0.00	0.19	0.34	0.00	0.39	0.23	0.12	0.00	0.00	0.11	0.39	0.25	0.27	0.32	0.13	0.00	0.00	0.00	0.00	0.00
POLR2M	0.00	0.00	0.18	0.40	0.00	0.45	0.24	0.19	0.00	0.00	0.14	0.31	0.17	0.22	0.33	0.13	0.00	0.00	0.00	0.00	0.00
LOC246784	0.00	0.00	0.13	0.38	0.00	0.66	0.16	0.13	0.00	0.00	0.12	0.59	0.38	0.36	0.48	0.26	0.00	0.00	0.00	0.00	0.00
OR8A1	0.00	0.00	0.20	0.30	0.00	0.22	0.23	0.19	0.00	0.00	0.19	0.29	0.22	0.20	0.44	0.26	0.00	0.00	0.00	0.00	0.00
ARHGAP32	0.00	0.00	0.25	0.39	0.00	0.37	0.28	0.27	0.00	0.00	0.11	0.28	0.19	0.21	0.30	0.15	0.00	0.00	0.00	0.00	0.00
TRPC2	0.00	0.00	0.24	0.32	0.00	0.20	0.25	0.16	0.00	0.00	0.13	0.21	0.13	0.14	0.27	0.13	0.00	0.00	0.00	0.00	0.00
CNTN2	0.00	0.00	0.12	0.20	0.00	0.11	0.17	0.22	0.00	0.00	0.14	0.18	0.24	0.14	0.39	0.29	0.00	0.00	0.00	0.00	0.00
NYAP1	0.00	0.00	0.34	0.48	0.00	0.27	0.44	0.22	0.00	0.00	0.32	0.24	0.23	0.13	0.43	0.26	0.00	0.00	0.00	0.00	0.00
LRRTM2	0.00	0.00	0.18	0.36	0.00	0.39	0.25	0.21	0.00	0.00	0.23	0.43	0.26	0.27	0.48	0.26	0.00	0.00	0.00	0.00	0.00
TRMT1L	0.00	0.00	0.13	0.34	0.00	0.49	0.17	0.18	0.00	0.00	0.11	0.53	0.36	0.29	0.59	0.37	0.00	0.00	0.00	0.00	0.00
OR10A4	0.00	0.00	0.19	0.30	0.00	0.21	0.23	0.20	0.00	0.00	0.18	0.29	0.24	0.21	0.44	0.27	0.00	0.00	0.00	0.00	0.00
HPCAL4	0.00	0.00	0.11	0.26	0.00	0.41	0.11	0.11	0.00	0.00	0.11	0.38	0.18	0.25	0.28	0.15	0.00	0.00	0.00	0.00	0.00
NLGN2	0.00	0.00	0.19	0.32	0.00	0.21	0.27	0.18	0.00	0.00	0.17	0.36	0.31	0.31	0.44	0.26	0.00	0.00	0.00	0.00	0.00
SEZ6	0.00	0.00	0.16	0.37	0.00	0.47	0.21	0.17	0.00	0.00	0.16	0.50	0.41	0.42	0.46	0.26	0.00	0.00	0.00	0.00	0.00
LRRC7	0.00	0.00	0.16	0.32	0.00	0.36	0.22	0.16	0.00	0.00	0.17	0.29	0.20	0.23	0.27	0.13	0.00	0.00	0.00	0.00	0.00
SLITRK2	0.00	0.00	0.23	0.42	0.00	0.45	0.28	0.17	0.00	0.00	0.23	0.46	0.30	0.24	0.52	0.27	0.00	0.00	0.00	0.00	0.00
STAC2	0.00	0.00	0.15	0.24	0.00	0.24	0.17	0.17	0.00	0.00	0.13	0.33	0.21	0.21	0.49	0.27	0.00	0.00	0.00	0.00	0.00
AP3M2	0.00	0.00	0.17	0.39	0.00	0.45	0.25	0.13	0.00	0.00	0.22	0.37	0.21	0.21	0.34	0.14	0.00	0.00	0.00	0.00	0.00

DPY19L4	0.00	0.00	0.38	0.56	0.00	0.40	0.46	0.31	0.00	0.00	0.14	0.47	0.32	0.37	0.48	0.25	0.00	0.00	0.00	0.00	0.00
SGSM1	0.00	0.00	0.23	0.46	0.00	0.54	0.29	0.29	0.00	0.00	0.12	0.48	0.36	0.31	0.50	0.27	0.00	0.00	0.00	0.00	0.00
PLCL2	0.00	0.00	0.34	0.48	0.00	0.29	0.42	0.23	0.00	0.00	0.15	0.27	0.19	0.23	0.28	0.12	0.00	0.00	0.00	0.00	0.00
ATAD1	0.00	0.00	0.18	0.30	0.00	0.20	0.25	0.17	0.00	0.00	0.11	0.27	0.23	0.37	0.24	0.13	0.00	0.00	0.00	0.00	0.00
SLC24A2	0.00	0.00	0.13	0.26	0.00	0.30	0.14	0.12	0.00	0.00	0.24	0.33	0.21	0.22	0.29	0.14	0.00	0.00	0.00	0.00	0.00
GRID2IP	0.00	0.00	0.29	0.45	0.00	0.36	0.35	0.18	0.00	0.00	0.14	0.30	0.24	0.26	0.31	0.15	0.00	0.00	0.00	0.00	0.00
CACNA2D2	0.00	0.00	0.14	0.26	0.00	0.26	0.17	0.14	0.00	0.00	0.15	0.35	0.20	0.23	0.35	0.25	0.00	0.00	0.00	0.00	0.00
SHANK2	0.00	0.00	0.27	0.38	0.00	0.30	0.28	0.11	0.00	0.00	0.13	0.32	0.27	0.23	0.29	0.11	0.00	0.00	0.00	0.00	0.00
UNC80	0.00	0.00	0.30	0.40	0.00	0.26	0.31	0.16	0.00	0.00	0.22	0.30	0.15	0.20	0.34	0.18	0.00	0.00	0.00	0.00	0.00
ASIC4	0.00	0.00	0.17	0.32	0.00	0.34	0.20	0.13	0.00	0.00	0.23	0.35	0.16	0.16	0.38	0.18	0.00	0.00	0.00	0.00	0.00
LGI4	0.00	0.00	0.15	0.33	0.00	0.39	0.21	0.15	0.00	0.00	0.23	0.32	0.20	0.20	0.50	0.33	0.00	0.00	0.00	0.00	0.00
CDK18	0.00	0.00	0.11	0.35	0.00	0.57	0.15	0.18	0.00	0.00	0.11	0.38	0.20	0.18	0.31	0.11	0.00	0.00	0.00	0.00	0.00
CNGA4	0.00	0.00	0.25	0.35	0.00	0.24	0.27	0.11	0.00	0.00	0.18	0.34	0.18	0.24	0.35	0.18	0.00	0.00	0.00	0.00	0.00
TMEM57	0.00	0.00	0.19	0.39	0.00	0.51	0.22	0.14	0.00	0.00	0.15	0.32	0.21	0.15	0.32	0.11	0.00	0.00	0.00	0.00	0.00
GABRR2	0.00	0.00	0.24	0.38	0.00	0.28	0.32	0.17	0.00	0.00	0.12	0.29	0.27	0.26	0.34	0.18	0.00	0.00	0.00	0.00	0.00
EPB41L2	0.00	0.00	0.21	0.40	0.00	0.41	0.27	0.19	0.00	0.00	0.25	0.29	0.14	0.12	0.34	0.11	0.00	0.00	0.00	0.00	0.00
KIRREL3	0.00	0.00	0.18	0.36	0.00	0.43	0.24	0.22	0.00	0.00	0.17	0.41	0.33	0.29	0.50	0.32	0.00	0.00	0.00	0.00	0.00
PPFIA3	0.00	0.00	0.13	0.36	0.00	0.53	0.19	0.18	0.00	0.00	0.12	0.49	0.26	0.27	0.47	0.22	0.00	0.00	0.00	0.00	0.00
CBLN2	0.00	0.00	0.29	0.46	0.00	0.37	0.35	0.17	0.00	0.00	0.22	0.51	0.43	0.36	0.54	0.32	0.00	0.00	0.00	0.00	0.00
TRIM9	0.00	0.00	0.15	0.36	0.00	0.44	0.22	0.28	0.00	0.00	0.19	0.44	0.29	0.27	0.46	0.22	0.00	0.00	0.00	0.00	0.00
STRN	0.00	0.00	0.23	0.41	0.00	0.47	0.27	0.29	0.00	0.00	0.17	0.40	0.20	0.22	0.40	0.19	0.00	0.00	0.00	0.00	0.00
CPLX4	0.00	0.00	0.13	0.26	0.00	0.23	0.18	0.17	0.00	0.00	0.15	0.33	0.24	0.27	0.41	0.22	0.00	0.00	0.00	0.00	0.00
NAV2	0.00	0.00	0.14	0.34	0.00	0.48	0.18	0.26	0.00	0.00	0.16	0.43	0.27	0.21	0.55	0.32	0.00	0.00	0.00	0.00	0.00
NTM	0.00	0.00	0.15	0.33	0.00	0.38	0.22	0.19	0.00	0.00	0.24	0.45	0.35	0.29	0.52	0.32	0.00	0.00	0.00	0.00	0.00
NCALD	0.00	0.00	0.28	0.43	0.00	0.38	0.31	0.17	0.00	0.00	0.22	0.33	0.19	0.20	0.40	0.18	0.00	0.00	0.00	0.00	0.00
LRRC4C	0.00	0.00	0.23	0.38	0.00	0.33	0.29	0.21	0.00	0.00	0.21	0.26	0.16	0.14	0.38	0.18	0.00	0.00	0.00	0.00	0.00
AATK	0.00	0.00	0.16	0.37	0.00	0.46	0.23	0.27	0.00	0.00	0.13	0.40	0.25	0.25	0.37	0.17	0.00	0.00	0.00	0.00	0.00
CACNG4	0.00	0.00	0.24	0.39	0.00	0.32	0.30	0.11	0.00	0.00	0.22	0.35	0.20	0.26	0.33	0.17	0.00	0.00	0.00	0.00	0.00
ANO10	0.00	0.00	0.13	0.32	0.00	0.43	0.17	0.15	0.00	0.00	0.26	0.36	0.18	0.14	0.43	0.22	0.00	0.00	0.00	0.00	0.00
SYT10	0.00	0.00	0.13	0.32	0.00	0.40	0.17	0.12	0.00	0.00	0.18	0.43	0.26	0.26	0.37	0.17	0.00	0.00	0.00	0.00	0.00
AP3B2	0.00	0.00	0.13	0.37	0.00	0.51	0.21	0.18	0.00	0.00	0.12	0.37	0.17	0.18	0.33	0.11	0.00	0.00	0.00	0.00	0.00
KIAA0319L	0.00	0.00	0.24	0.43	0.00	0.44	0.31	0.14	0.00	0.00	0.17	0.33	0.27	0.19	0.37	0.17	0.00	0.00	0.00	0.00	0.00
LPPR3	0.00	0.00	0.27	0.36	0.00	0.20	0.33	0.16	0.00	0.00	0.25	0.44	0.35	0.33	0.59	0.44	0.00	0.00	0.00	0.00	0.00
SYNGR3	0.00	0.00	0.13	0.37	0.00	0.51	0.18	0.24	0.00	0.00	0.19	0.42	0.25	0.23	0.35	0.11	0.00	0.00	0.00	0.00	0.00
CLSTN1	0.00	0.00	0.13	0.29	0.00	0.34	0.18	0.14	0.00	0.00	0.18	0.38	0.20	0.24	0.34	0.17	0.00	0.00	0.00	0.00	0.00
SV2C	0.00	0.00	0.14	0.29	0.00	0.29	0.19	0.18	0.00	0.00	0.18	0.36	0.27	0.27	0.37	0.17	0.00	0.00	0.00	0.00	0.00
LRFN5	0.00	0.00	0.23	0.39	0.00	0.36	0.30	0.15	0.00	0.00	0.23	0.37	0.24	0.26	0.43	0.22	0.00	0.00	0.00	0.00	0.00
LRRTM1	0.00	0.00	0.18	0.34	0.00	0.31	0.24	0.17	0.00	0.00	0.16	0.45	0.34	0.35	0.45	0.22	0.00	0.00	0.00	0.00	0.00
TRANK1	0.00	0.00	0.12	0.29	0.00	0.39	0.16	0.12	0.00	0.00	0.16	0.39	0.25	0.27	0.36	0.17	0.00	0.00	0.00	0.00	0.00
CACNG8	0.00	0.00	0.31	0.45	0.00	0.29	0.37	0.13	0.00	0.00	0.24	0.34	0.21	0.31	0.33	0.17	0.00	0.00	0.00	0.00	0.00

KIF13B	0.00	0.00	0.19	0.37	0.00	0.45	0.23	0.26	0.00	0.00	0.12	0.31	0.16	0.12	0.33	0.11	0.00	0.00	0.00	0.00	0.00
AMIGO1	0.00	0.00	0.14	0.34	0.00	0.45	0.20	0.20	0.00	0.00	0.27	0.46	0.25	0.31	0.44	0.25	0.00	0.00	0.00	0.00	0.00
CAMK1G	0.00	0.00	0.23	0.41	0.00	0.39	0.30	0.26	0.00	0.00	0.13	0.42	0.27	0.29	0.40	0.22	0.00	0.00	0.00	0.00	0.00
OMP	0.00	0.00	0.16	0.25	0.00	0.18	0.19	0.21	0.00	0.00	0.12	0.21	0.20	0.17	0.36	0.20	0.00	0.00	0.00	0.00	0.00
PCDH20	0.00	0.00	0.20	0.36	0.00	0.36	0.26	0.20	0.00	0.00	0.24	0.41	0.29	0.25	0.52	0.33	0.00	0.00	0.00	0.00	0.00
GRIP2	0.00	0.00	0.28	0.42	0.00	0.28	0.36	0.16	0.00	0.00	0.20	0.32	0.24	0.30	0.34	0.19	0.00	0.00	0.00	0.00	0.00
GABRA6	0.00	0.00	0.17	0.25	0.00	0.10	0.24	0.12	0.00	0.00	0.11	0.21	0.25	0.24	0.27	0.19	0.00	0.00	0.00	0.00	0.00
NRSN1	0.00	0.00	0.13	0.34	0.00	0.48	0.18	0.21	0.00	0.00	0.17	0.51	0.33	0.31	0.55	0.31	0.00	0.00	0.00	0.00	0.00
HPCAL1	0.00	0.00	0.17	0.35	0.00	0.44	0.19	0.15	0.00	0.00	0.14	0.48	0.37	0.33	0.39	0.20	0.00	0.00	0.00	0.00	0.00
MAP6D1	0.00	0.00	0.12	0.34	0.00	0.53	0.17	0.20	0.00	0.00	0.13	0.51	0.35	0.35	0.42	0.21	0.00	0.00	0.00	0.00	0.00
GABRR1	0.00	0.00	0.25	0.38	0.00	0.23	0.32	0.18	0.00	0.00	0.18	0.26	0.24	0.24	0.35	0.20	0.00	0.00	0.00	0.00	0.00
JAKMIP1	0.00	0.00	0.30	0.51	0.00	0.48	0.37	0.24	0.00	0.00	0.18	0.38	0.28	0.25	0.42	0.21	0.00	0.00	0.00	0.00	0.00
SLC6A5	0.00	0.00	0.14	0.28	0.00	0.25	0.19	0.15	0.00	0.00	0.25	0.31	0.29	0.24	0.39	0.21	0.00	0.00	0.00	0.00	0.00
OR10J5	0.00	0.00	0.34	0.47	0.00	0.33	0.36	0.24	0.00	0.00	0.23	0.28	0.19	0.15	0.41	0.20	0.00	0.00	0.00	0.00	0.00
FSTL4	0.00	0.00	0.13	0.32	0.00	0.46	0.16	0.19	0.00	0.00	0.13	0.28	0.18	0.15	0.41	0.20	0.00	0.00	0.00	0.00	0.00
SEMA6B	0.00	0.00	0.20	0.36	0.00	0.30	0.28	0.19	0.00	0.00	0.22	0.24	0.20	0.14	0.39	0.20	0.00	0.00	0.00	0.00	0.00
NLGN1	0.00	0.00	0.19	0.30	0.00	0.19	0.25	0.17	0.00	0.00	0.18	0.31	0.26	0.29	0.36	0.20	0.00	0.00	0.00	0.00	0.00
PCDHAC2	0.00	0.00	0.17	0.38	0.00	0.44	0.24	0.23	0.00	0.00	0.20	0.33	0.22	0.19	0.42	0.20	0.00	0.00	0.00	0.00	0.00
SV2A	0.00	0.00	0.15	0.29	0.00	0.22	0.22	0.19	0.00	0.00	0.18	0.32	0.25	0.27	0.38	0.21	0.00	0.00	0.00	0.00	0.00
NPTN	0.00	0.00	0.24	0.45	0.00	0.41	0.34	0.19	0.00	0.00	0.25	0.43	0.33	0.37	0.42	0.21	0.00	0.00	0.00	0.00	0.00
SCN7A	0.00	0.00	0.13	0.27	0.00	0.30	0.16	0.14	0.00	0.00	0.16	0.34	0.19	0.17	0.41	0.21	0.00	0.00	0.00	0.00	0.00
OR51B4	0.00	0.00	0.21	0.28	0.00	0.16	0.24	0.15	0.00	0.00	0.11	0.20	0.17	0.15	0.36	0.21	0.00	0.00	0.00	0.00	0.00
KCNT2	0.00	0.00	0.16	0.28	0.00	0.25	0.19	0.14	0.00	0.00	0.19	0.36	0.24	0.28	0.35	0.20	0.00	0.00	0.00	0.00	0.00
NRXN2	0.00	0.00	0.23	0.36	0.00	0.26	0.30	0.14	0.00	0.00	0.22	0.35	0.23	0.24	0.41	0.20	0.00	0.00	0.00	0.00	0.00
CPLX3	0.00	0.00	0.14	0.31	0.00	0.31	0.21	0.27	0.00	0.00	0.16	0.36	0.27	0.27	0.45	0.21	0.00	0.00	0.00	0.00	0.00
FJX1	0.00	0.00	0.14	0.32	0.00	0.43	0.18	0.24	0.00	0.00	0.14	0.35	0.28	0.18	0.37	0.19	0.00	0.00	0.00	0.00	0.00
ANO8	0.00	0.00	0.12	0.33	0.00	0.49	0.15	0.12	0.00	0.00	0.26	0.39	0.18	0.13	0.42	0.21	0.00	0.00	0.00	0.00	0.00
FGF13	0.00	0.00	0.14	0.31	0.00	0.36	0.19	0.21	0.00	0.00	0.16	0.35	0.19	0.16	0.43	0.19	0.00	0.00	0.00	0.00	0.00
CBLN3	0.00	0.00	0.23	0.41	0.00	0.38	0.30	0.18	0.00	0.00	0.18	0.46	0.39	0.36	0.51	0.32	0.00	0.00	0.00	0.00	0.00
NLGN3	0.00	0.00	0.18	0.30	0.00	0.22	0.24	0.14	0.00	0.00	0.18	0.35	0.28	0.29	0.40	0.21	0.00	0.00	0.00	0.00	0.00
NEURL	0.00	0.00	0.19	0.39	0.00	0.44	0.26	0.36	0.00	0.00	0.12	0.35	0.18	0.18	0.40	0.19	0.00	0.00	0.00	0.00	0.00
IGSF9	0.00	0.00	0.25	0.45	0.00	0.43	0.34	0.23	0.00	0.00	0.25	0.44	0.32	0.32	0.56	0.32	0.00	0.00	0.00	0.00	0.00
NETO1	0.00	0.00	0.31	0.49	0.00	0.37	0.38	0.14	0.00	0.00	0.23	0.40	0.27	0.33	0.42	0.22	0.00	0.00	0.00	0.00	0.00
PLEKHB1	0.00	0.00	0.16	0.36	0.00	0.46	0.21	0.21	0.00	0.00	0.18	0.38	0.23	0.18	0.44	0.21	0.00	0.00	0.00	0.00	0.00
LPPR5	0.00	0.00	0.19	0.41	0.00	0.54	0.23	0.22	0.00	0.00	0.15	0.53	0.33	0.29	0.52	0.32	0.00	0.00	0.00	0.00	0.00
KCNH5	0.00	0.00	0.10	0.26	0.00	0.33	0.14	0.14	0.00	0.00	0.19	0.40	0.25	0.28	0.38	0.21	0.00	0.00	0.00	0.00	0.00
SV2B	0.00	0.00	0.13	0.27	0.00	0.24	0.19	0.16	0.00	0.00	0.18	0.33	0.24	0.26	0.38	0.19	0.00	0.00	0.00	0.00	0.00
GPHN	0.00	0.00	0.15	0.29	0.00	0.21	0.23	0.17	0.00	0.00	0.11	0.31	0.30	0.28	0.35	0.19	0.00	0.00	0.00	0.00	0.00
LRRC4B	0.00	0.00	0.21	0.34	0.00	0.22	0.31	0.17	0.00	0.00	0.23	0.35	0.25	0.28	0.36	0.21	0.00	0.00	0.00	0.00	0.00
NKX1-2	0.00	0.00	0.23	0.39	0.00	0.35	0.29	0.23	0.00	0.00	0.11	0.34	0.27	0.18	0.49	0.32	0.00	0.00	0.00	0.00	0.00

STOML3	0.00	0.00	0.25	0.41	0.00	0.43	0.26	0.15	0.00	0.00	0.25	0.34	0.20	0.15	0.42	0.19	0.00	0.00	0.00	0.00	0.00
CNTN4	0.00	0.00	0.12	0.27	0.00	0.35	0.16	0.15	0.00	0.00	0.16	0.34	0.23	0.19	0.39	0.19	0.00	0.00	0.00	0.00	0.00
SYT2	0.00	0.00	0.16	0.30	0.00	0.23	0.22	0.20	0.00	0.00	0.17	0.22	0.13	0.16	0.30	0.14	0.00	0.00	0.00	0.00	0.00
OR2A7	0.00	0.00	0.32	0.43	0.00	0.29	0.35	0.20	0.00	0.00	0.17	0.29	0.22	0.18	0.40	0.23	0.00	0.00	0.00	0.00	0.00
LRRTM3	0.00	0.00	0.16	0.32	0.00	0.38	0.20	0.16	0.00	0.00	0.21	0.37	0.20	0.20	0.39	0.17	0.00	0.00	0.00	0.00	0.00
KIF26A	0.00	0.00	0.24	0.40	0.00	0.38	0.30	0.25	0.00	0.00	0.11	0.29	0.12	0.10	0.40	0.16	0.00	0.00	0.00	0.00	0.00
FAT3	0.00	0.00	0.18	0.41	0.00	0.55	0.24	0.25	0.00	0.00	0.21	0.42	0.24	0.17	0.47	0.24	0.00	0.00	0.00	0.00	0.00
ZDHHC23	0.00	0.00	0.15	0.29	0.00	0.35	0.19	0.18	0.00	0.00	0.14	0.37	0.22	0.24	0.45	0.24	0.00	0.00	0.00	0.00	0.00
STOML1	0.00	0.00	0.13	0.35	0.00	0.53	0.17	0.13	0.00	0.00	0.28	0.35	0.17	0.17	0.30	0.12	0.00	0.00	0.00	0.00	0.00
NXPH3	0.00	0.00	0.27	0.49	0.00	0.49	0.35	0.18	0.00	0.00	0.26	0.44	0.25	0.23	0.48	0.20	0.00	0.00	0.00	0.00	0.00
NFASC	0.00	0.00	0.10	0.23	0.00	0.22	0.16	0.14	0.00	0.00	0.26	0.29	0.19	0.16	0.50	0.37	0.00	0.00	0.00	0.00	0.00
PANX2	0.00	0.00	0.13	0.30	0.00	0.34	0.17	0.17	0.00	0.00	0.20	0.37	0.28	0.26	0.39	0.24	0.00	0.00	0.00	0.00	0.00
PLCL1	0.00	0.00	0.27	0.41	0.00	0.30	0.34	0.23	0.00	0.00	0.11	0.27	0.19	0.22	0.27	0.12	0.00	0.00	0.00	0.00	0.00
ERMN	0.00	0.00	0.11	0.31	0.00	0.44	0.17	0.26	0.00	0.00	0.13	0.28	0.20	0.13	0.43	0.36	0.00	0.00	0.00	0.00	0.00
UNC13C	0.00	0.00	0.14	0.30	0.00	0.32	0.20	0.21	0.00	0.00	0.11	0.38	0.33	0.35	0.39	0.24	0.00	0.00	0.00	0.00	0.00
LINGO4	0.00	0.00	0.17	0.35	0.00	0.43	0.21	0.14	0.00	0.00	0.26	0.43	0.26	0.20	0.53	0.31	0.00	0.00	0.00	0.00	0.00
RCN2	0.00	0.00	0.25	0.46	0.00	0.50	0.30	0.26	0.00	0.00	0.20	0.34	0.19	0.19	0.31	0.11	0.00	0.00	0.00	0.00	0.00
LGI2	0.00	0.00	0.17	0.37	0.00	0.51	0.21	0.15	0.00	0.00	0.24	0.36	0.19	0.22	0.38	0.16	0.00	0.00	0.00	0.00	0.00
GLRA2	0.00	0.00	0.23	0.38	0.00	0.26	0.30	0.13	0.00	0.00	0.19	0.33	0.21	0.22	0.42	0.24	0.00	0.00	0.00	0.00	0.00
NRXN1	0.00	0.00	0.16	0.26	0.00	0.16	0.22	0.14	0.00	0.00	0.18	0.28	0.22	0.23	0.33	0.16	0.00	0.00	0.00	0.00	0.00
CPLX1	0.00	0.00	0.11	0.25	0.00	0.23	0.17	0.22	0.00	0.00	0.14	0.30	0.25	0.28	0.35	0.16	0.00	0.00	0.00	0.00	0.00
LINGO3	0.00	0.00	0.17	0.38	0.00	0.52	0.21	0.15	0.00	0.00	0.23	0.51	0.34	0.27	0.61	0.35	0.00	0.00	0.00	0.00	0.00
DTNB	0.00	0.00	0.15	0.30	0.00	0.36	0.19	0.15	0.00	0.00	0.14	0.30	0.15	0.16	0.32	0.11	0.00	0.00	0.00	0.00	0.00
GABRR3	0.00	0.00	0.32	0.49	0.00	0.38	0.39	0.15	0.00	0.00	0.16	0.42	0.36	0.35	0.44	0.24	0.00	0.00	0.00	0.00	0.00
SRCIN1	0.00	0.00	0.20	0.37	0.00	0.36	0.27	0.29	0.00	0.00	0.12	0.29	0.21	0.21	0.28	0.12	0.00	0.00	0.00	0.00	0.00
PCDH9	0.00	0.00	0.11	0.31	0.00	0.47	0.15	0.11	0.00	0.00	0.24	0.43	0.30	0.24	0.44	0.25	0.00	0.00	0.00	0.00	0.00
DSCAML1	0.00	0.00	0.15	0.34	0.00	0.41	0.22	0.19	0.00	0.00	0.19	0.48	0.35	0.29	0.55	0.37	0.00	0.00	0.00	0.00	0.00
CDH10	0.00	0.00	0.10	0.26	0.00	0.37	0.13	0.10	0.00	0.00	0.19	0.33	0.22	0.17	0.33	0.15	0.00	0.00	0.00	0.00	0.00
SLITRK4	0.00	0.00	0.26	0.48	0.00	0.53	0.33	0.21	0.00	0.00	0.29	0.54	0.32	0.26	0.62	0.37	0.00	0.00	0.00	0.00	0.00
KLHL17	0.00	0.00	0.26	0.47	0.00	0.44	0.34	0.26	0.00	0.00	0.12	0.37	0.23	0.27	0.38	0.15	0.00	0.00	0.00	0.00	0.00
PCDHGB7	0.00	0.00	0.33	0.53	0.00	0.45	0.42	0.29	0.00	0.00	0.34	0.27	0.12	0.11	0.39	0.15	0.00	0.00	0.00	0.00	0.00
SEMA4C	0.00	0.00	0.19	0.35	0.00	0.36	0.25	0.21	0.00	0.00	0.21	0.36	0.27	0.23	0.45	0.25	0.00	0.00	0.00	0.00	0.00
FLVCR2	0.00	0.00	0.24	0.45	0.00	0.50	0.29	0.15	0.00	0.00	0.31	0.33	0.15	0.10	0.37	0.12	0.00	0.00	0.00	0.00	0.00
OR10A5	0.00	0.00	0.29	0.43	0.00	0.38	0.32	0.11	0.00	0.00	0.26	0.33	0.21	0.17	0.44	0.25	0.00	0.00	0.00	0.00	0.00
GABRE	0.00	0.00	0.22	0.35	0.00	0.22	0.30	0.14	0.00	0.00	0.13	0.25	0.21	0.20	0.30	0.15	0.00	0.00	0.00	0.00	0.00
RMST	0.00	0.00	0.12	0.30	0.00	0.45	0.14	0.17	0.00	0.00	0.11	0.37	0.23	0.14	0.42	0.24	0.00	0.00	0.00	0.00	0.00
DRGX	0.00	0.00	0.13	0.28	0.00	0.39	0.15	0.22	0.00	0.00	0.12	0.49	0.35	0.29	0.70	0.48	0.00	0.00	0.00	0.00	0.00
SYT12	0.00	0.00	0.20	0.38	0.00	0.37	0.26	0.24	0.00	0.00	0.13	0.36	0.22	0.21	0.35	0.16	0.00	0.00	0.00	0.00	0.00
CACNB4	0.00	0.00	0.11	0.20	0.00	0.17	0.14	0.13	0.00	0.00	0.11	0.22	0.15	0.21	0.24	0.15	0.00	0.00	0.00	0.00	0.00
CAPN11	0.00	0.00	0.11	0.30	0.00	0.46	0.14	0.22	0.00	0.00	0.11	0.36	0.18	0.20	0.33	0.15	0.00	0.00	0.00	0.00	0.00

CACNA1I	0.00	0.00	0.14	0.22	0.00	0.20	0.13	0.11	0.00	0.00	0.16	0.24	0.14	0.17	0.26	0.15	0.00	0.00	0.00	0.00	0.00
SEMA4G	0.00	0.00	0.19	0.34	0.00	0.29	0.26	0.16	0.00	0.00	0.21	0.34	0.28	0.22	0.47	0.29	0.00	0.00	0.00	0.00	0.00
C1ORF187	0.00	0.00	0.22	0.34	0.00	0.25	0.28	0.21	0.00	0.00	0.18	0.33	0.32	0.25	0.49	0.35	0.00	0.00	0.00	0.00	0.00
CNIH2	0.00	0.00	0.40	0.54	0.00	0.32	0.47	0.18	0.00	0.00	0.28	0.38	0.24	0.28	0.45	0.24	0.00	0.00	0.00	0.00	0.00
LGI3	0.00	0.00	0.22	0.43	0.00	0.48	0.27	0.25	0.00	0.00	0.21	0.47	0.29	0.34	0.44	0.23	0.00	0.00	0.00	0.00	0.00
LAMP5	0.00	0.00	0.19	0.40	0.00	0.48	0.26	0.16	0.00	0.00	0.23	0.43	0.28	0.25	0.39	0.23	0.00	0.00	0.00	0.00	0.00
MEGF9	0.00	0.00	0.28	0.50	0.00	0.51	0.36	0.18	0.00	0.00	0.31	0.41	0.25	0.20	0.52	0.30	0.00	0.00	0.00	0.00	0.00
KCNJ16	0.00	0.00	0.11	0.24	0.00	0.27	0.14	0.14	0.00	0.00	0.19	0.26	0.16	0.14	0.25	0.11	0.00	0.00	0.00	0.00	0.00
CXORF36	0.00	0.00	0.11	0.28	0.00	0.45	0.12	0.12	0.00	0.00	0.11	0.32	0.17	0.14	0.32	0.11	0.00	0.00	0.00	0.00	0.00
SEMA3D	0.00	0.00	0.16	0.30	0.00	0.26	0.24	0.18	0.00	0.00	0.19	0.23	0.18	0.14	0.35	0.16	0.00	0.00	0.00	0.00	0.00
GLRA4	0.00	0.00	0.23	0.38	0.00	0.24	0.33	0.11	0.00	0.00	0.18	0.31	0.20	0.22	0.40	0.23	0.00	0.00	0.00	0.00	0.00
SEMA5B	0.00	0.00	0.14	0.29	0.00	0.27	0.21	0.22	0.00	0.00	0.21	0.33	0.27	0.22	0.54	0.34	0.00	0.00	0.00	0.00	0.00
PCDHGA3	0.00	0.00	0.11	0.27	0.00	0.26	0.20	0.16	0.00	0.00	0.26	0.35	0.25	0.26	0.39	0.23	0.00	0.00	0.00	0.00	0.00
DNM3	0.00	0.00	0.25	0.40	0.00	0.35	0.29	0.20	0.00	0.00	0.10	0.31	0.18	0.17	0.30	0.11	0.00	0.00	0.00	0.00	0.00
NLGN4X	0.00	0.00	0.15	0.27	0.00	0.26	0.20	0.10	0.00	0.00	0.14	0.29	0.20	0.19	0.29	0.11	0.00	0.00	0.00	0.00	0.00
GABRB1	0.00	0.00	0.24	0.34	0.00	0.18	0.30	0.13	0.00	0.00	0.11	0.27	0.28	0.28	0.30	0.16	0.00	0.00	0.00	0.00	0.00
IGDCC3	0.00	0.00	0.15	0.35	0.00	0.46	0.21	0.16	0.00	0.00	0.20	0.34	0.18	0.13	0.45	0.23	0.00	0.00	0.00	0.00	0.00
FLRT3	0.00	0.00	0.21	0.40	0.00	0.42	0.28	0.21	0.00	0.00	0.23	0.36	0.19	0.15	0.48	0.23	0.00	0.00	0.00	0.00	0.00
GUCY2E	0.00	0.00	0.25	0.35	0.00	0.24	0.27	0.15	0.00	0.00	0.21	0.26	0.17	0.12	0.36	0.17	0.00	0.00	0.00	0.00	0.00
NTNG2	0.00	0.00	0.22	0.40	0.00	0.36	0.30	0.19	0.00	0.00	0.22	0.44	0.40	0.32	0.54	0.30	0.00	0.00	0.00	0.00	0.00
KLHL24	0.00	0.00	0.39	0.54	0.00	0.32	0.46	0.18	0.00	0.00	0.25	0.27	0.15	0.22	0.32	0.11	0.00	0.00	0.00	0.00	0.00
JPH3	0.00	0.00	0.15	0.27	0.00	0.27	0.18	0.00	0.14	0.00	0.13	0.25	0.13	0.14	0.26	0.13	0.00	0.00	0.00	0.00	0.00
LARGE	0.00	0.00	0.10	0.18	0.00	0.18	0.12	0.00	0.21	0.00	0.19	0.19	0.11	0.11	0.29	0.16	0.00	0.00	0.00	0.00	0.00
GRIK3	0.00	0.00	0.25	0.35	0.00	0.17	0.30	0.00	0.11	0.00	0.15	0.24	0.24	0.30	0.29	0.15	0.00	0.00	0.00	0.00	0.00
GRIA2	0.00	0.00	0.10	0.17	0.00	0.00	0.15	0.11	0.13	0.00	0.11	0.10	0.14	0.22	0.17	0.14	0.00	0.00	0.00	0.00	0.00
CHRNB2	0.00	0.00	0.10	0.17	0.00	0.00	0.16	0.14	0.15	0.00	0.10	0.13	0.18	0.21	0.19	0.10	0.00	0.00	0.00	0.00	0.00
EPHA4	0.00	0.00	0.12	0.19	0.00	0.00	0.16	0.19	0.13	0.00	0.14	0.15	0.21	0.20	0.27	0.22	0.00	0.00	0.00	0.00	0.00
EPHB2	0.00	0.00	0.14	0.19	0.00	0.00	0.17	0.20	0.15	0.00	0.15	0.13	0.15	0.16	0.20	0.13	0.00	0.00	0.00	0.00	0.00
HTR3A	0.00	0.00	0.19	0.25	0.00	0.00	0.22	0.14	0.18	0.00	0.15	0.11	0.14	0.15	0.24	0.13	0.00	0.00	0.00	0.00	0.00
SLC12A5	0.00	0.00	0.00	0.18	0.00	0.13	0.13	0.18	0.10	0.00	0.11	0.22	0.27	0.27	0.29	0.21	0.00	0.00	0.00	0.00	0.00
DAG1	0.00	0.00	0.00	0.16	0.00	0.11	0.13	0.12	0.20	0.00	0.16	0.18	0.14	0.12	0.27	0.17	0.00	0.00	0.00	0.00	0.00
NAV3	0.00	0.00	0.00	0.28	0.00	0.44	0.13	0.20	0.16	0.00	0.12	0.29	0.14	0.11	0.36	0.17	0.00	0.00	0.00	0.00	0.00
MLC1	0.00	0.00	0.00	0.22	0.00	0.32	0.12	0.12	0.14	0.00	0.15	0.32	0.21	0.17	0.34	0.20	0.00	0.00	0.00	0.00	0.00
SCN3B	0.00	0.00	0.00	0.18	0.00	0.22	0.11	0.14	0.12	0.00	0.19	0.24	0.10	0.16	0.31	0.17	0.00	0.00	0.00	0.00	0.00
CLN6	0.00	0.00	0.00	0.20	0.00	0.27	0.11	0.12	0.21	0.00	0.18	0.31	0.20	0.17	0.34	0.25	0.00	0.00	0.00	0.00	0.00
SLC12A6	0.00	0.00	0.00	0.20	0.00	0.23	0.11	0.17	0.11	0.00	0.15	0.24	0.18	0.13	0.29	0.17	0.00	0.00	0.00	0.00	0.00
CDH4	0.00	0.00	0.00	0.21	0.00	0.25	0.13	0.24	0.13	0.00	0.14	0.25	0.21	0.13	0.34	0.21	0.00	0.00	0.00	0.00	0.00
BCAN	0.00	0.00	0.00	0.21	0.00	0.25	0.13	0.10	0.12	0.00	0.13	0.39	0.32	0.33	0.44	0.31	0.00	0.00	0.00	0.00	0.00
SCN2B	0.00	0.00	0.00	0.19	0.00	0.22	0.12	0.14	0.15	0.00	0.23	0.28	0.14	0.20	0.37	0.24	0.00	0.00	0.00	0.00	0.00
SLC12A4	0.00	0.00	0.00	0.24	0.00	0.24	0.15	0.19	0.11	0.00	0.13	0.20	0.13	0.11	0.23	0.11	0.00	0.00	0.00	0.00	0.00

MAG	0.00	0.00	0.00	0.19	0.00	0.15	0.15	0.13	0.13	0.00	0.17	0.19	0.15	0.13	0.41	0.40	0.00	0.00	0.00	0.00	0.00
CSPG4	0.00	0.00	0.00	0.17	0.00	0.14	0.13	0.17	0.24	0.00	0.12	0.21	0.23	0.20	0.35	0.32	0.00	0.00	0.00	0.00	0.00
SEPT7P2	0.00	0.00	0.00	0.32	0.00	0.55	0.12	0.24	0.16	0.00	0.10	0.41	0.20	0.16	0.34	0.19	0.00	0.00	0.00	0.00	0.00
KCNH1	0.00	0.00	0.00	0.21	0.00	0.22	0.12	0.24	0.21	0.00	0.13	0.22	0.11	0.14	0.21	0.10	0.00	0.00	0.00	0.00	0.00
CNTNAP2	0.00	0.00	0.00	0.19	0.00	0.20	0.13	0.12	0.11	0.00	0.16	0.33	0.25	0.23	0.43	0.29	0.00	0.00	0.00	0.00	0.00
ST8SIA2	0.00	0.00	0.00	0.19	0.00	0.22	0.11	0.17	0.11	0.00	0.11	0.28	0.24	0.28	0.30	0.18	0.00	0.00	0.00	0.00	0.00
PCDH17	0.00	0.00	0.00	0.29	0.00	0.46	0.14	0.24	0.15	0.00	0.13	0.28	0.19	0.14	0.29	0.14	0.00	0.00	0.00	0.00	0.00
SPRR1A	0.00	0.00	0.00	0.23	0.00	0.31	0.12	0.17	0.10	0.00	0.10	0.22	0.16	0.12	0.32	0.18	0.00	0.00	0.00	0.00	0.00
SLC8A2	0.00	0.00	0.00	0.22	0.00	0.26	0.12	0.13	0.10	0.00	0.17	0.38	0.28	0.33	0.30	0.19	0.00	0.00	0.00	0.00	0.00
ST8SIA4	0.00	0.00	0.00	0.17	0.00	0.19	0.11	0.17	0.15	0.00	0.10	0.25	0.23	0.27	0.31	0.22	0.00	0.00	0.00	0.00	0.00
KCNQ5	0.00	0.00	0.00	0.19	0.00	0.22	0.10	0.13	0.11	0.00	0.16	0.26	0.14	0.20	0.26	0.12	0.00	0.00	0.00	0.00	0.00
CLCN2	0.00	0.00	0.00	0.19	0.00	0.17	0.11	0.10	0.14	0.00	0.22	0.19	0.16	0.15	0.22	0.13	0.00	0.00	0.00	0.00	0.00
PNMA3	0.00	0.00	0.00	0.32	0.00	0.58	0.12	0.21	0.17	0.00	0.13	0.38	0.17	0.16	0.36	0.17	0.00	0.00	0.00	0.00	0.00
SLC22A3	0.00	0.00	0.00	0.18	0.00	0.16	0.13	0.16	0.20	0.00	0.15	0.18	0.16	0.12	0.22	0.00	0.11	0.00	0.00	0.00	0.00
KIRREL	0.00	0.00	0.12	0.26	0.00	0.29	0.18	0.15	0.00	0.00	0.18	0.19	0.15	0.00	0.28	0.21	0.10	0.00	0.00	0.00	0.00
NEUROD2	0.00	0.00	0.13	0.28	0.00	0.32	0.17	0.29	0.00	0.00	0.00	0.36	0.34	0.33	0.47	0.26	0.11	0.00	0.00	0.00	0.00
DRP2	0.00	0.00	0.00	0.23	0.00	0.27	0.14	0.10	0.15	0.00	0.00	0.28	0.19	0.19	0.31	0.13	0.12	0.00	0.00	0.00	0.00
LINGO2	0.00	0.00	0.12	0.26	0.00	0.34	0.16	0.00	0.00	0.00	0.12	0.31	0.22	0.17	0.38	0.21	0.10	0.00	0.00	0.00	0.00
KCNN3	0.00	0.00	0.00	0.16	0.00	0.16	0.00	0.12	0.17	0.00	0.10	0.22	0.16	0.19	0.24	0.11	0.12	0.00	0.00	0.00	0.00
ARRB2	0.41	0.41	0.26	0.18	0.44	0.00	0.14	0.12	0.15	0.12	0.00	0.00	0.00	0.00	0.12	0.00	0.00	0.12	0.00	0.00	0.00
LHCGR	0.21	0.21	0.27	0.25	0.23	0.00	0.21	0.16	0.00	0.27	0.16	0.00	0.00	0.00	0.15	0.00	0.00	0.12	0.00	0.00	0.00
CRHBP	0.00	0.00	0.18	0.24	0.00	0.15	0.19	0.13	0.12	0.00	0.00	0.19	0.18	0.19	0.25	0.00	0.00	0.12	0.00	0.00	0.00
STOM	0.00	0.00	0.11	0.29	0.00	0.40	0.14	0.12	0.00	0.00	0.26	0.25	0.10	0.00	0.28	0.11	0.00	0.10	0.00	0.00	0.00
BRSK2	0.00	0.00	0.00	0.26	0.00	0.35	0.15	0.33	0.15	0.00	0.00	0.30	0.19	0.18	0.32	0.17	0.00	0.12	0.00	0.00	0.00
SLC32A1	0.00	0.00	0.00	0.22	0.00	0.18	0.16	0.16	0.00	0.00	0.11	0.33	0.36	0.32	0.38	0.24	0.00	0.14	0.00	0.00	0.00
SYT1	0.00	0.00	0.00	0.19	0.00	0.14	0.13	0.23	0.00	0.00	0.12	0.17	0.14	0.17	0.22	0.10	0.00	0.11	0.00	0.00	0.00
NAT8	0.00	0.00	0.10	0.30	0.00	0.50	0.12	0.19	0.13	0.00	0.23	0.31	0.12	0.00	0.21	0.00	0.00	0.00	0.29	0.00	0.00
MYLIP	0.00	0.00	0.18	0.35	0.00	0.33	0.25	0.36	0.17	0.00	0.11	0.20	0.11	0.00	0.23	0.00	0.00	0.00	0.19	0.00	0.00
NIPA2	0.00	0.00	0.12	0.27	0.00	0.36	0.15	0.12	0.16	0.00	0.20	0.25	0.11	0.00	0.25	0.00	0.00	0.00	0.11	0.00	0.00
SULT2B1	0.00	0.00	0.12	0.29	0.00	0.32	0.18	0.19	0.13	0.00	0.11	0.21	0.10	0.00	0.17	0.00	0.00	0.00	0.30	0.00	0.00
APBA3	0.00	0.00	0.17	0.33	0.00	0.33	0.22	0.26	0.12	0.00	0.18	0.25	0.00	0.13	0.23	0.00	0.00	0.00	0.15	0.00	0.00
NDFIP1	0.00	0.00	0.16	0.35	0.00	0.39	0.23	0.30	0.16	0.00	0.10	0.22	0.00	0.11	0.26	0.00	0.00	0.00	0.11	0.00	0.00
HSPA12A	0.00	0.00	0.11	0.29	0.00	0.43	0.15	0.14	0.11	0.00	0.00	0.30	0.22	0.16	0.21	0.00	0.00	0.00	0.18	0.00	0.00
PICALM	0.00	0.00	0.13	0.25	0.00	0.23	0.18	0.17	0.11	0.00	0.00	0.24	0.13	0.14	0.21	0.00	0.00	0.00	0.10	0.00	0.00
HINT1	0.00	0.00	0.17	0.31	0.00	0.34	0.20	0.28	0.21	0.00	0.00	0.24	0.17	0.15	0.24	0.00	0.00	0.00	0.11	0.00	0.00
CELF2	0.00	0.00	0.10	0.27	0.00	0.34	0.14	0.32	0.17	0.00	0.00	0.24	0.13	0.14	0.24	0.00	0.00	0.00	0.15	0.00	0.00
CBR1	0.00	0.00	0.12	0.24	0.00	0.24	0.14	0.21	0.20	0.00	0.00	0.25	0.17	0.13	0.20	0.00	0.00	0.00	0.32	0.00	0.00
ASMT	0.00	0.00	0.19	0.31	0.00	0.31	0.21	0.14	0.12	0.00	0.00	0.25	0.14	0.11	0.23	0.00	0.00	0.00	0.18	0.00	0.00
PDE11A	0.00	0.00	0.12	0.24	0.00	0.27	0.14	0.17	0.15	0.00	0.00	0.24	0.19	0.12	0.20	0.00	0.00	0.00	0.12	0.00	0.00
NCSTN	0.00	0.00	0.00	0.18	0.00	0.18	0.12	0.16	0.16	0.00	0.20	0.16	0.11	0.12	0.16	0.00	0.00	0.00	0.11	0.00	0.00

PSEN2	0.00	0.00	0.00	0.15	0.00	0.11	0.11	0.13	0.16	0.00	0.15	0.14	0.13	0.15	0.12	0.00	0.00	0.00	0.10	0.00	0.00
SLC30A4	0.00	0.00	0.00	0.25	0.00	0.31	0.14	0.15	0.18	0.00	0.16	0.29	0.18	0.18	0.23	0.00	0.00	0.00	0.22	0.00	0.00
A3GALT2P	0.00	0.00	0.16	0.30	0.00	0.29	0.22	0.20	0.24	0.00	0.15	0.18	0.00	0.00	0.25	0.14	0.00	0.00	0.12	0.00	0.00
MTMR2	0.00	0.00	0.11	0.25	0.00	0.33	0.14	0.15	0.11	0.00	0.14	0.20	0.00	0.00	0.34	0.22	0.00	0.00	0.11	0.00	0.00
NUS1	0.00	0.00	0.28	0.45	0.00	0.44	0.32	0.27	0.18	0.00	0.28	0.22	0.00	0.00	0.26	0.10	0.00	0.00	0.13	0.00	0.00
ST3GAL5	0.00	0.00	0.14	0.27	0.00	0.29	0.18	0.25	0.24	0.00	0.13	0.22	0.00	0.00	0.24	0.13	0.00	0.00	0.17	0.00	0.00
GRHL3	0.00	0.00	0.11	0.21	0.00	0.19	0.14	0.20	0.12	0.00	0.00	0.15	0.11	0.00	0.28	0.18	0.00	0.00	0.13	0.00	0.00
SMPD3	0.00	0.00	0.14	0.29	0.00	0.28	0.19	0.27	0.21	0.00	0.00	0.21	0.10	0.00	0.20	0.11	0.00	0.00	0.17	0.00	0.00
C11ORF1	0.00	0.00	0.17	0.33	0.00	0.39	0.20	0.19	0.00	0.00	0.00	0.45	0.37	0.35	0.40	0.20	0.00	0.00	0.10	0.00	0.00
RAVER2	0.00	0.00	0.14	0.38	0.00	0.52	0.21	0.28	0.00	0.00	0.00	0.32	0.15	0.18	0.34	0.14	0.00	0.00	0.11	0.00	0.00
PAFAH1B3	0.00	0.00	0.14	0.29	0.00	0.33	0.19	0.17	0.00	0.00	0.00	0.31	0.24	0.19	0.25	0.10	0.00	0.00	0.10	0.00	0.00
PRMT8	0.00	0.00	0.11	0.34	0.00	0.51	0.16	0.26	0.00	0.00	0.00	0.37	0.22	0.17	0.35	0.17	0.00	0.00	0.14	0.00	0.00
CPEB3	0.00	0.00	0.18	0.42	0.00	0.51	0.25	0.30	0.00	0.00	0.00	0.31	0.19	0.27	0.30	0.10	0.00	0.00	0.11	0.00	0.00
DDC	0.00	0.00	0.00	0.17	0.00	0.13	0.11	0.17	0.20	0.00	0.00	0.24	0.23	0.15	0.28	0.12	0.00	0.00	0.18	0.00	0.00
NAGLU	0.00	0.00	0.00	0.19	0.00	0.21	0.12	0.11	0.28	0.00	0.00	0.29	0.19	0.16	0.30	0.22	0.00	0.00	0.18	0.00	0.00
PADI2	0.00	0.00	0.00	0.25	0.00	0.32	0.13	0.15	0.16	0.00	0.00	0.32	0.22	0.21	0.33	0.27	0.00	0.00	0.16	0.00	0.00
CYP46A1	0.00	0.00	0.00	0.14	0.00	0.14	0.10	0.13	0.16	0.00	0.00	0.22	0.19	0.20	0.22	0.15	0.00	0.00	0.27	0.00	0.00
GRAMD1A	0.00	0.00	0.00	0.27	0.00	0.45	0.12	0.18	0.14	0.00	0.00	0.47	0.37	0.31	0.38	0.21	0.00	0.00	0.16	0.00	0.00
DYRK1A	0.00	0.00	0.00	0.22	0.00	0.24	0.13	0.27	0.22	0.00	0.00	0.32	0.24	0.24	0.29	0.14	0.00	0.00	0.13	0.00	0.00
ZC3H14	0.00	0.00	0.00	0.31	0.00	0.52	0.12	0.23	0.12	0.00	0.00	0.34	0.16	0.19	0.32	0.13	0.00	0.00	0.11	0.00	0.00
HNRNPR	0.00	0.00	0.00	0.27	0.00	0.41	0.11	0.31	0.14	0.00	0.00	0.23	0.12	0.12	0.33	0.15	0.00	0.00	0.14	0.00	0.00
NTAN1	0.00	0.00	0.00	0.26	0.00	0.41	0.11	0.20	0.11	0.00	0.00	0.35	0.24	0.27	0.31	0.14	0.00	0.00	0.19	0.00	0.00
PQBP1	0.00	0.00	0.00	0.26	0.00	0.41	0.12	0.21	0.11	0.00	0.00	0.27	0.14	0.12	0.28	0.10	0.00	0.00	0.13	0.00	0.00
SLC6A7	0.00	0.00	0.11	0.29	0.00	0.42	0.14	0.00	0.00	0.00	0.22	0.48	0.38	0.33	0.44	0.24	0.00	0.00	0.14	0.00	0.00
CTXN1	0.00	0.00	0.11	0.31	0.00	0.51	0.14	0.00	0.00	0.00	0.14	0.63	0.43	0.35	0.56	0.38	0.00	0.00	0.11	0.00	0.00
SLC33A1	0.00	0.00	0.00	0.29	0.00	0.49	0.13	0.14	0.00	0.00	0.23	0.34	0.17	0.13	0.34	0.17	0.00	0.00	0.15	0.00	0.00
SLC1A6	0.00	0.00	0.00	0.20	0.00	0.17	0.13	0.12	0.00	0.00	0.16	0.29	0.31	0.31	0.32	0.23	0.00	0.00	0.11	0.00	0.00
SLC44A2	0.00	0.00	0.00	0.32	0.00	0.49	0.15	0.19	0.00	0.00	0.27	0.31	0.15	0.12	0.34	0.17	0.00	0.00	0.14	0.00	0.00
SLC17A5	0.00	0.00	0.00	0.20	0.00	0.27	0.10	0.00	0.18	0.00	0.20	0.31	0.21	0.14	0.35	0.25	0.00	0.00	0.17	0.00	0.00
PPT2	0.00	0.00	0.00	0.22	0.00	0.35	0.11	0.00	0.16	0.00	0.11	0.31	0.15	0.13	0.28	0.20	0.00	0.00	0.20	0.00	0.00
BACE1	0.00	0.00	0.00	0.12	0.00	0.10	0.00	0.17	0.23	0.00	0.16	0.17	0.13	0.19	0.17	0.13	0.00	0.00	0.15	0.00	0.00
ATP7A	0.00	0.00	0.00	0.14	0.00	0.13	0.00	0.10	0.16	0.00	0.11	0.20	0.14	0.14	0.21	0.11	0.00	0.00	0.19	0.00	0.00
ECE1	0.00	0.00	0.13	0.19	0.00	0.12	0.15	0.17	0.21	0.00	0.00	0.15	0.12	0.00	0.17	0.00	0.12	0.00	0.13	0.00	0.00
PTER	0.00	0.00	0.14	0.31	0.00	0.48	0.15	0.10	0.00	0.00	0.11	0.28	0.11	0.00	0.22	0.00	0.16	0.00	0.21	0.00	0.00
ABCG1	0.00	0.00	0.10	0.18	0.00	0.13	0.14	0.19	0.18	0.00	0.00	0.13	0.00	0.00	0.15	0.11	0.13	0.00	0.21	0.00	0.00
FDFT1	0.00	0.00	0.00	0.23	0.00	0.33	0.12	0.23	0.14	0.00	0.00	0.24	0.11	0.00	0.24	0.12	0.12	0.00	0.36	0.00	0.00
STRADA	0.00	0.00	0.14	0.28	0.00	0.31	0.18	0.37	0.12	0.00	0.00	0.15	0.11	0.00	0.21	0.00	0.00	0.27	0.15	0.00	0.00
ENO2	0.00	0.00	0.00	0.12	0.00	0.16	0.00	0.14	0.16	0.00	0.00	0.24	0.19	0.22	0.29	0.20	0.00	0.10	0.13	0.00	0.00
ASPA	0.00	0.00	0.00	0.16	0.00	0.24	0.00	0.12	0.19	0.00	0.00	0.32	0.24	0.19	0.38	0.32	0.00	0.12	0.24	0.00	0.00
SNAPIN	0.00	0.00	0.19	0.36	0.00	0.33	0.25	0.28	0.00	0.00	0.14	0.25	0.16	0.17	0.27	0.00	0.00	0.00	0.00	0.13	0.00

VAMP1	0.00	0.00	0.14	0.29	0.00	0.27	0.19	0.21	0.00	0.00	0.20	0.25	0.15	0.17	0.30	0.00	0.00	0.00	0.00	0.12	0.00
VAMP2	0.00	0.00	0.10	0.19	0.00	0.13	0.13	0.21	0.00	0.00	0.15	0.13	0.11	0.12	0.21	0.00	0.00	0.00	0.00	0.16	0.00
STXBP1	0.00	0.00	0.10	0.21	0.00	0.17	0.14	0.24	0.00	0.00	0.12	0.21	0.16	0.19	0.27	0.00	0.00	0.00	0.00	0.16	0.00
CAMK2B	0.00	0.00	0.14	0.24	0.00	0.19	0.17	0.20	0.00	0.00	0.00	0.22	0.18	0.23	0.19	0.10	0.00	0.00	0.00	0.10	0.00
CADPS2	0.00	0.00	0.15	0.30	0.00	0.30	0.20	0.21	0.00	0.00	0.00	0.40	0.33	0.36	0.38	0.21	0.00	0.00	0.00	0.11	0.00
CAPS2	0.00	0.00	0.12	0.28	0.00	0.33	0.18	0.22	0.00	0.00	0.00	0.42	0.33	0.38	0.38	0.24	0.00	0.00	0.00	0.12	0.00
SEL1L	0.00	0.00	0.15	0.30	0.00	0.35	0.18	0.30	0.20	0.00	0.21	0.14	0.00	0.00	0.20	0.00	0.16	0.00	0.00	0.12	0.00
NR3C2	0.00	0.00	0.16	0.22	0.00	0.00	0.19	0.21	0.17	0.00	0.00	0.11	0.18	0.17	0.18	0.00	0.12	0.00	0.00	0.10	0.00
ARX	0.00	0.00	0.00	0.13	0.00	0.18	0.00	0.18	0.11	0.00	0.00	0.23	0.21	0.14	0.27	0.15	0.12	0.00	0.00	0.12	0.00
GNAT3	0.00	0.00	0.30	0.33	0.00	0.17	0.27	0.17	0.14	0.00	0.11	0.15	0.00	0.00	0.27	0.00	0.00	0.12	0.00	0.16	0.00
RPS6KA1	0.00	0.00	0.10	0.19	0.00	0.17	0.12	0.27	0.15	0.00	0.00	0.12	0.00	0.13	0.12	0.00	0.00	0.13	0.00	0.10	0.00
RPS6KA3	0.00	0.00	0.13	0.22	0.00	0.18	0.15	0.27	0.19	0.00	0.00	0.16	0.00	0.15	0.16	0.00	0.00	0.12	0.00	0.12	0.00
MKKS	0.00	0.00	0.11	0.18	0.00	0.23	0.10	0.10	0.12	0.00	0.00	0.16	0.00	0.00	0.23	0.00	0.20	0.13	0.00	0.12	0.00
RPGRIP1L	0.00	0.00	0.14	0.25	0.00	0.33	0.14	0.17	0.13	0.00	0.00	0.20	0.00	0.00	0.24	0.00	0.15	0.11	0.00	0.11	0.00
BBS4	0.00	0.00	0.14	0.23	0.00	0.26	0.14	0.13	0.14	0.00	0.00	0.20	0.00	0.00	0.26	0.00	0.16	0.14	0.00	0.11	0.00
GAD2	0.00	0.00	0.00	0.11	0.00	0.00	0.00	0.17	0.16	0.00	0.00	0.17	0.23	0.20	0.25	0.17	0.26	0.13	0.00	0.15	0.00
PIKFYVE	0.00	0.00	0.14	0.28	0.00	0.28	0.18	0.23	0.11	0.00	0.12	0.15	0.00	0.00	0.18	0.00	0.00	0.00	0.18	0.22	0.00
NOX3	0.00	0.00	0.13	0.22	0.00	0.19	0.16	0.22	0.16	0.00	0.11	0.14	0.00	0.00	0.22	0.00	0.00	0.00	0.10	0.12	0.00
CAV2	0.00	0.00	0.13	0.21	0.00	0.18	0.15	0.18	0.14	0.00	0.12	0.16	0.00	0.00	0.17	0.00	0.00	0.00	0.12	0.10	0.00
ASAH2	0.00	0.00	0.13	0.24	0.00	0.29	0.13	0.17	0.16	0.00	0.10	0.16	0.00	0.00	0.14	0.00	0.00	0.00	0.28	0.13	0.00
SMPD2	0.00	0.00	0.19	0.28	0.00	0.24	0.20	0.25	0.22	0.00	0.00	0.17	0.00	0.00	0.19	0.12	0.00	0.00	0.20	0.11	0.00
UGCG	0.00	0.00	0.00	0.20	0.00	0.24	0.11	0.27	0.31	0.00	0.00	0.24	0.14	0.00	0.25	0.20	0.00	0.00	0.25	0.11	0.00
BTBD10	0.00	0.00	0.13	0.34	0.00	0.49	0.18	0.36	0.20	0.00	0.00	0.26	0.00	0.00	0.18	0.00	0.20	0.00	0.13	0.29	0.00
RAB18	0.00	0.00	0.13	0.31	0.00	0.46	0.16	0.24	0.00	0.00	0.13	0.24	0.00	0.00	0.21	0.00	0.13	0.00	0.14	0.16	0.00
ADAP2	0.00	0.00	0.14	0.35	0.00	0.55	0.16	0.14	0.00	0.00	0.11	0.29	0.00	0.00	0.25	0.00	0.10	0.00	0.14	0.12	0.00
LRP2BP	0.00	0.00	0.15	0.30	0.00	0.37	0.19	0.10	0.00	0.00	0.11	0.13	0.00	0.00	0.12	0.00	0.33	0.00	0.14	0.15	0.00
ACE2	0.00	0.00	0.11	0.15	0.00	0.00	0.12	0.17	0.23	0.00	0.00	0.11	0.11	0.00	0.17	0.00	0.16	0.00	0.11	0.10	0.00
AKR1B1	0.00	0.00	0.00	0.15	0.00	0.11	0.12	0.16	0.19	0.00	0.00	0.10	0.11	0.00	0.14	0.00	0.26	0.00	0.21	0.18	0.00
CNDP1	0.00	0.00	0.00	0.20	0.00	0.21	0.14	0.11	0.14	0.00	0.00	0.16	0.12	0.00	0.18	0.00	0.33	0.00	0.17	0.22	0.00
ADA	0.00	0.00	0.00	0.14	0.00	0.00	0.11	0.13	0.17	0.00	0.00	0.15	0.13	0.11	0.20	0.00	0.14	0.00	0.16	0.11	0.00
HSD11B2	0.00	0.00	0.00	0.15	0.00	0.00	0.12	0.17	0.15	0.00	0.00	0.10	0.17	0.11	0.15	0.00	0.15	0.00	0.20	0.13	0.00
GHRHR	0.00	0.00	0.18	0.23	0.00	0.11	0.20	0.15	0.16	0.00	0.00	0.11	0.00	0.00	0.17	0.00	0.00	0.12	0.13	0.11	0.00
RPS6KB2	0.00	0.00	0.12	0.24	0.00	0.25	0.15	0.37	0.20	0.00	0.00	0.13	0.00	0.00	0.14	0.00	0.00	0.14	0.14	0.15	0.00
MYO1C	0.00	0.00	0.11	0.26	0.00	0.30	0.15	0.22	0.00	0.00	0.10	0.14	0.00	0.00	0.21	0.00	0.00	0.14	0.11	0.16	0.00
GAA	0.00	0.00	0.00	0.14	0.00	0.11	0.00	0.12	0.28	0.00	0.11	0.11	0.00	0.00	0.18	0.11	0.00	0.11	0.15	0.11	0.00
MIR33B	0.00	0.00	0.11	0.23	0.00	0.24	0.15	0.31	0.10	0.00	0.00	0.12	0.00	0.00	0.00	0.00	0.22	0.23	0.47	0.27	0.00
APOM	0.00	0.00	0.12	0.22	0.00	0.19	0.16	0.16	0.14	0.00	0.00	0.00	0.00	0.00	0.10	0.00	0.28	0.10	0.26	0.20	0.00
SERPINA6	0.00	0.00	0.12	0.21	0.00	0.17	0.16	0.14	0.00	0.00	0.00	0.12	0.00	0.00	0.19	0.00	0.15	0.12	0.17	0.15	0.00
VAC14	0.00	0.00	0.11	0.29	0.00	0.39	0.15	0.26	0.00	0.00	0.00	0.20	0.00	0.00	0.26	0.00	0.11	0.11	0.23	0.23	0.00
AGPAT2	0.00	0.00	0.00	0.20	0.00	0.28	0.11	0.18	0.17	0.00	0.00	0.14	0.00	0.00	0.12	0.00	0.24	0.23	0.34	0.27	0.00

TAT	0.00	0.00	0.00	0.24	0.00	0.26	0.14	0.18	0.12	0.00	0.00	0.17	0.00	0.00	0.18	0.00	0.12	0.13	0.22	0.15	0.00
CRTC1	0.00	0.00	0.00	0.21	0.00	0.21	0.13	0.41	0.25	0.00	0.00	0.12	0.00	0.00	0.16	0.00	0.11	0.25	0.13	0.19	0.00
PRKAG2	0.00	0.00	0.00	0.18	0.00	0.20	0.11	0.20	0.15	0.00	0.00	0.14	0.00	0.00	0.19	0.00	0.17	0.33	0.25	0.24	0.00
ELOVL6	0.00	0.00	0.00	0.20	0.00	0.26	0.11	0.23	0.15	0.00	0.00	0.12	0.00	0.00	0.13	0.00	0.26	0.26	0.39	0.29	0.00
IGFBP2	0.00	0.00	0.00	0.14	0.00	0.11	0.10	0.15	0.17	0.00	0.00	0.10	0.00	0.00	0.15	0.00	0.14	0.10	0.11	0.16	0.00
MPI	0.00	0.00	0.00	0.22	0.00	0.23	0.14	0.00	0.14	0.00	0.11	0.18	0.00	0.00	0.16	0.00	0.10	0.10	0.24	0.16	0.00
BSCL2	0.00	0.00	0.00	0.18	0.00	0.25	0.00	0.12	0.14	0.00	0.11	0.14	0.00	0.00	0.23	0.00	0.22	0.18	0.21	0.23	0.00
EPM2A	0.00	0.00	0.00	0.19	0.00	0.32	0.00	0.24	0.19	0.00	0.00	0.24	0.10	0.00	0.23	0.00	0.11	0.15	0.20	0.19	0.00
EIF2B5	0.00	0.00	0.00	0.15	0.00	0.24	0.00	0.17	0.14	0.00	0.00	0.18	0.00	0.11	0.21	0.00	0.17	0.14	0.13	0.20	0.00
EIF2B3	0.00	0.00	0.00	0.17	0.00	0.27	0.00	0.18	0.10	0.00	0.00	0.18	0.00	0.10	0.21	0.00	0.16	0.13	0.11	0.17	0.00
HSD11B1	0.00	0.00	0.00	0.14	0.00	0.00	0.11	0.15	0.15	0.00	0.00	0.00	0.13	0.12	0.11	0.00	0.19	0.12	0.25	0.20	0.00
TMEM132A	0.00	0.00	0.14	0.31	0.00	0.37	0.18	0.26	0.17	0.00	0.15	0.20	0.11	0.00	0.16	0.00	0.00	0.00	0.00	0.00	0.16
SH3RF2	0.00	0.00	0.14	0.35	0.00	0.50	0.19	0.34	0.16	0.00	0.00	0.32	0.14	0.16	0.27	0.00	0.00	0.00	0.00	0.00	0.14
ERAL1	0.00	0.00	0.22	0.45	0.00	0.55	0.26	0.30	0.00	0.00	0.11	0.26	0.12	0.16	0.30	0.00	0.00	0.00	0.00	0.00	0.24
TRAPPC4	0.00	0.00	0.14	0.37	0.00	0.54	0.19	0.24	0.00	0.00	0.12	0.29	0.15	0.14	0.27	0.00	0.00	0.00	0.00	0.00	0.17
DOPEY1	0.00	0.00	0.11	0.31	0.00	0.52	0.14	0.23	0.00	0.00	0.11	0.26	0.12	0.11	0.27	0.00	0.00	0.00	0.00	0.00	0.10
TBCB	0.00	0.00	0.12	0.31	0.00	0.41	0.17	0.24	0.13	0.00	0.00	0.26	0.15	0.00	0.37	0.18	0.00	0.00	0.00	0.00	0.12
MAP1S	0.00	0.00	0.11	0.33	0.00	0.46	0.17	0.23	0.00	0.00	0.00	0.28	0.14	0.11	0.31	0.13	0.00	0.00	0.00	0.00	0.21
KIF17	0.00	0.00	0.10	0.28	0.00	0.35	0.16	0.22	0.00	0.00	0.00	0.33	0.25	0.25	0.34	0.15	0.00	0.00	0.00	0.00	0.12
TTLL7	0.00	0.00	0.11	0.32	0.00	0.49	0.14	0.25	0.00	0.00	0.00	0.35	0.19	0.13	0.35	0.12	0.00	0.00	0.00	0.00	0.14
UTP11L	0.00	0.00	0.00	0.27	0.00	0.39	0.14	0.26	0.19	0.00	0.00	0.49	0.34	0.38	0.44	0.28	0.00	0.00	0.00	0.00	0.19
RNF19A	0.00	0.00	0.00	0.25	0.00	0.35	0.12	0.19	0.12	0.00	0.00	0.24	0.13	0.12	0.27	0.13	0.00	0.00	0.00	0.00	0.12
PNMA1	0.00	0.00	0.00	0.27	0.00	0.43	0.12	0.24	0.24	0.00	0.00	0.38	0.22	0.21	0.39	0.27	0.00	0.00	0.00	0.00	0.16
FEZ1	0.00	0.00	0.00	0.28	0.00	0.38	0.15	0.24	0.12	0.00	0.00	0.37	0.32	0.25	0.39	0.16	0.00	0.00	0.00	0.00	0.14
TPPP	0.00	0.00	0.00	0.23	0.00	0.33	0.11	0.29	0.25	0.00	0.00	0.26	0.16	0.15	0.27	0.17	0.00	0.00	0.00	0.00	0.11
MTRNR2L1	0.00	0.00	0.00	0.25	0.00	0.41	0.11	0.14	0.12	0.00	0.00	0.27	0.12	0.18	0.20	0.10	0.00	0.00	0.00	0.00	0.37
PNMA6D	0.00	0.00	0.00	0.32	0.00	0.61	0.12	0.11	0.00	0.00	0.16	0.49	0.22	0.23	0.41	0.19	0.00	0.00	0.00	0.00	0.21
PNMA5	0.00	0.00	0.00	0.32	0.00	0.61	0.12	0.11	0.00	0.00	0.16	0.49	0.22	0.23	0.41	0.19	0.00	0.00	0.00	0.00	0.21
SYBU	0.00	0.00	0.00	0.33	0.00	0.53	0.15	0.18	0.00	0.00	0.16	0.47	0.28	0.27	0.44	0.22	0.00	0.00	0.00	0.00	0.21
KIFC2	0.00	0.00	0.00	0.34	0.00	0.56	0.14	0.18	0.00	0.00	0.11	0.38	0.20	0.13	0.39	0.17	0.00	0.00	0.00	0.00	0.10
HVCN1	0.00	0.00	0.11	0.26	0.00	0.26	0.16	0.23	0.15	0.00	0.24	0.12	0.00	0.00	0.13	0.00	0.00	0.12	0.00	0.00	0.12
DYNLL2	0.00	0.00	0.13	0.30	0.00	0.36	0.19	0.21	0.00	0.00	0.00	0.26	0.13	0.11	0.28	0.00	0.00	0.10	0.00	0.00	0.25
PARK7	0.00	0.00	0.00	0.13	0.00	0.12	0.00	0.18	0.24	0.00	0.00	0.17	0.14	0.13	0.19	0.11	0.00	0.10	0.00	0.00	0.16
ASAH2C	0.00	0.00	0.15	0.31	0.00	0.43	0.15	0.16	0.11	0.00	0.14	0.23	0.00	0.00	0.16	0.00	0.00	0.00	0.25	0.00	0.17
ATG9A	0.00	0.00	0.17	0.33	0.00	0.37	0.22	0.25	0.11	0.00	0.13	0.16	0.00	0.00	0.21	0.00	0.00	0.00	0.13	0.00	0.14
CERS5	0.00	0.00	0.11	0.28	0.00	0.45	0.11	0.27	0.11	0.00	0.11	0.22	0.00	0.00	0.15	0.00	0.00	0.00	0.30	0.00	0.20
STARD5	0.00	0.00	0.15	0.34	0.00	0.47	0.20	0.20	0.00	0.00	0.23	0.22	0.14	0.00	0.16	0.00	0.00	0.00	0.26	0.00	0.15
C12ORF10	0.00	0.00	0.10	0.29	0.00	0.45	0.14	0.21	0.00	0.00	0.00	0.24	0.14	0.11	0.23	0.00	0.00	0.00	0.15	0.00	0.15
ZDHHC8	0.00	0.00	0.11	0.23	0.00	0.26	0.14	0.11	0.00	0.00	0.00	0.24	0.18	0.17	0.22	0.00	0.00	0.00	0.14	0.00	0.11
ADC	0.00	0.00	0.00	0.26	0.00	0.35	0.13	0.33	0.23	0.00	0.00	0.29	0.19	0.12	0.21	0.00	0.00	0.00	0.19	0.00	0.14

PPP3CC	0.00	0.00	0.00	0.22	0.00	0.28	0.13	0.18	0.15	0.00	0.00	0.26	0.18	0.22	0.18	0.00	0.00	0.00	0.12	0.00	0.18
SFXN5	0.00	0.00	0.12	0.32	0.00	0.43	0.17	0.11	0.00	0.00	0.13	0.27	0.00	0.00	0.26	0.10	0.00	0.00	0.11	0.00	0.22
RPL24	0.00	0.00	0.00	0.24	0.00	0.40	0.00	0.24	0.10	0.00	0.00	0.28	0.18	0.14	0.36	0.18	0.00	0.00	0.11	0.00	0.16
TECR	0.00	0.00	0.00	0.27	0.00	0.48	0.10	0.00	0.00	0.00	0.16	0.45	0.24	0.18	0.36	0.19	0.00	0.00	0.32	0.00	0.14
ATP5A1	0.00	0.00	0.10	0.27	0.00	0.32	0.15	0.20	0.14	0.00	0.00	0.20	0.00	0.00	0.16	0.00	0.00	0.21	0.27	0.00	0.27
PHB	0.00	0.00	0.11	0.23	0.00	0.19	0.16	0.30	0.22	0.00	0.00	0.11	0.00	0.00	0.13	0.00	0.00	0.14	0.19	0.00	0.23
YARS	0.00	0.00	0.10	0.27	0.00	0.37	0.13	0.19	0.12	0.00	0.00	0.16	0.00	0.00	0.23	0.00	0.00	0.12	0.17	0.00	0.17
CAPNS1	0.00	0.00	0.10	0.21	0.00	0.22	0.13	0.24	0.24	0.00	0.00	0.17	0.00	0.00	0.20	0.00	0.00	0.12	0.11	0.00	0.16
TRAP1	0.00	0.00	0.15	0.35	0.00	0.40	0.21	0.32	0.24	0.00	0.00	0.18	0.00	0.00	0.19	0.00	0.00	0.13	0.14	0.00	0.31
OXR1	0.00	0.00	0.11	0.34	0.00	0.51	0.15	0.28	0.16	0.00	0.00	0.26	0.00	0.00	0.26	0.00	0.00	0.12	0.18	0.00	0.29
TMEM11	0.00	0.00	0.12	0.29	0.00	0.43	0.13	0.14	0.00	0.00	0.11	0.15	0.00	0.00	0.11	0.00	0.00	0.24	0.15	0.00	0.69
BNIP1	0.00	0.00	0.11	0.34	0.00	0.50	0.16	0.27	0.00	0.00	0.13	0.12	0.00	0.00	0.14	0.00	0.00	0.11	0.13	0.00	0.40
STARD4	0.00	0.00	0.13	0.32	0.00	0.43	0.18	0.27	0.00	0.00	0.18	0.17	0.00	0.00	0.14	0.00	0.00	0.13	0.31	0.00	0.13
STOML2	0.00	0.00	0.00	0.30	0.00	0.43	0.14	0.24	0.17	0.00	0.17	0.20	0.00	0.00	0.20	0.00	0.00	0.12	0.11	0.00	0.28
LETM1	0.00	0.00	0.00	0.33	0.00	0.55	0.14	0.26	0.11	0.00	0.15	0.25	0.00	0.00	0.17	0.00	0.00	0.17	0.14	0.00	0.41
PDXK	0.00	0.00	0.00	0.24	0.00	0.35	0.11	0.16	0.14	0.00	0.00	0.30	0.17	0.00	0.22	0.00	0.00	0.19	0.38	0.00	0.22
MVD	0.00	0.00	0.00	0.27	0.00	0.38	0.14	0.25	0.14	0.00	0.00	0.24	0.11	0.00	0.15	0.00	0.00	0.13	0.39	0.00	0.19
ATP5J2	0.00	0.00	0.00	0.23	0.00	0.32	0.11	0.14	0.17	0.00	0.00	0.26	0.13	0.00	0.16	0.00	0.00	0.23	0.21	0.00	0.38
FTMT	0.00	0.00	0.00	0.23	0.00	0.28	0.13	0.27	0.18	0.00	0.00	0.19	0.00	0.11	0.20	0.00	0.00	0.12	0.26	0.00	0.28
ACO1	0.00	0.00	0.00	0.19	0.00	0.20	0.11	0.18	0.13	0.00	0.00	0.18	0.00	0.11	0.20	0.00	0.00	0.13	0.29	0.00	0.19
PPP3CB	0.00	0.00	0.00	0.17	0.00	0.19	0.11	0.22	0.21	0.00	0.00	0.17	0.00	0.14	0.14	0.00	0.00	0.14	0.12	0.00	0.14
IMMP2L	0.00	0.00	0.00	0.22	0.00	0.37	0.00	0.12	0.11	0.00	0.00	0.29	0.15	0.14	0.24	0.00	0.00	0.21	0.21	0.00	0.43
ENDOG	0.00	0.00	0.00	0.19	0.00	0.25	0.00	0.27	0.24	0.00	0.00	0.18	0.13	0.14	0.18	0.00	0.00	0.10	0.13	0.00	0.34
AFG3L2	0.00	0.00	0.00	0.17	0.00	0.30	0.00	0.12	0.13	0.00	0.00	0.21	0.11	0.00	0.31	0.19	0.00	0.12	0.13	0.00	0.26
ABCD1	0.00	0.00	0.00	0.15	0.00	0.25	0.00	0.00	0.15	0.00	0.12	0.25	0.16	0.00	0.26	0.17	0.00	0.13	0.28	0.00	0.20
UQCRC1	0.00	0.00	0.00	0.23	0.00	0.41	0.00	0.16	0.00	0.00	0.00	0.29	0.14	0.10	0.26	0.10	0.00	0.23	0.27	0.00	0.44
MRPL10	0.00	0.00	0.00	0.28	0.00	0.52	0.00	0.24	0.00	0.00	0.00	0.36	0.18	0.15	0.30	0.12	0.00	0.17	0.22	0.00	0.35
GCDH	0.00	0.00	0.00	0.12	0.00	0.23	0.00	0.00	0.17	0.00	0.00	0.28	0.17	0.10	0.24	0.14	0.00	0.22	0.37	0.00	0.27
MMD2	0.00	0.00	0.31	0.52	0.00	0.53	0.36	0.14	0.00	0.00	0.27	0.24	0.00	0.00	0.24	0.00	0.15	0.00	0.00	0.14	0.21
KIF11	0.00	0.00	0.00	0.16	0.00	0.21	0.00	0.24	0.19	0.00	0.00	0.14	0.11	0.00	0.18	0.00	0.14	0.11	0.00	0.11	0.14
HSPB2	0.00	0.00	0.00	0.12	0.00	0.15	0.00	0.16	0.22	0.00	0.00	0.11	0.00	0.00	0.15	0.10	0.11	0.11	0.00	0.11	0.11
PRDX3	0.00	0.00	0.00	0.14	0.00	0.23	0.00	0.20	0.20	0.00	0.00	0.18	0.14	0.00	0.17	0.00	0.14	0.00	0.17	0.14	0.21
GCLC	0.00	0.00	0.00	0.12	0.00	0.13	0.00	0.12	0.13	0.00	0.00	0.13	0.11	0.00	0.12	0.00	0.15	0.00	0.20	0.14	0.13
AIFM3	0.00	0.00	0.00	0.27	0.00	0.37	0.14	0.28	0.18	0.00	0.10	0.17	0.00	0.00	0.00	0.00	0.00	0.16	0.20	0.12	0.50
TMEM161A	0.00	0.00	0.00	0.30	0.00	0.49	0.12	0.26	0.16	0.00	0.11	0.16	0.00	0.00	0.00	0.00	0.00	0.17	0.27	0.18	0.25
GRHPR	0.00	0.00	0.00	0.21	0.00	0.30	0.11	0.11	0.11	0.00	0.00	0.15	0.00	0.00	0.12	0.00	0.00	0.14	0.37	0.11	0.17
ACAT1	0.00	0.00	0.00	0.20	0.00	0.18	0.13	0.17	0.17	0.00	0.00	0.18	0.00	0.00	0.13	0.00	0.00	0.20	0.40	0.15	0.18
BNIP3L	0.00	0.00	0.00	0.24	0.00	0.31	0.13	0.32	0.19	0.00	0.00	0.12	0.00	0.00	0.17	0.00	0.00	0.13	0.12	0.14	0.31
MFN1	0.00	0.00	0.00	0.21	0.00	0.29	0.00	0.22	0.13	0.00	0.10	0.15	0.00	0.00	0.18	0.00	0.00	0.20	0.15	0.10	0.48
COQ7	0.00	0.00	0.00	0.24	0.00	0.39	0.00	0.19	0.17	0.00	0.00	0.26	0.10	0.00	0.22	0.00	0.00	0.22	0.31	0.13	0.37

GSS	0.00	0.00	0.00	0.16	0.00	0.21	0.00	0.16	0.16	0.00	0.00	0.20	0.11	0.00	0.18	0.00	0.00	0.17	0.33	0.15	0.15
ASS1	0.00	0.00	0.00	0.12	0.00	0.16	0.00	0.13	0.20	0.00	0.00	0.21	0.16	0.00	0.17	0.00	0.00	0.12	0.27	0.10	0.12
CYCS	0.00	0.00	0.00	0.15	0.00	0.17	0.00	0.17	0.17	0.00	0.00	0.16	0.00	0.10	0.17	0.00	0.00	0.17	0.17	0.12	0.41
CKMT1B	0.00	0.00	0.00	0.21	0.00	0.28	0.00	0.13	0.00	0.00	0.00	0.30	0.17	0.14	0.25	0.00	0.00	0.34	0.31	0.14	0.34
NDUFS1	0.00	0.00	0.00	0.19	0.00	0.33	0.00	0.11	0.00	0.00	0.00	0.26	0.14	0.11	0.19	0.00	0.00	0.27	0.34	0.12	0.55
SIRT2	0.00	0.00	0.00	0.18	0.00	0.24	0.00	0.29	0.22	0.00	0.00	0.17	0.00	0.00	0.21	0.11	0.00	0.18	0.19	0.11	0.15
PCTP	0.00	0.00	0.00	0.26	0.00	0.33	0.16	0.21	0.00	0.00	0.00	0.15	0.00	0.00	0.10	0.00	0.18	0.24	0.44	0.21	0.15
LIAS	0.00	0.00	0.00	0.16	0.00	0.24	0.00	0.16	0.17	0.00	0.00	0.18	0.00	0.00	0.10	0.00	0.31	0.23	0.41	0.33	0.29
COX10	0.00	0.00	0.00	0.19	0.00	0.32	0.00	0.14	0.16	0.00	0.00	0.21	0.00	0.00	0.18	0.00	0.11	0.22	0.31	0.16	0.43
MPV17	0.00	0.00	0.00	0.19	0.00	0.32	0.00	0.14	0.16	0.00	0.00	0.17	0.00	0.00	0.19	0.00	0.13	0.12	0.23	0.10	0.27
PANK4	0.00	0.00	0.00	0.17	0.00	0.30	0.00	0.12	0.11	0.00	0.00	0.22	0.00	0.00	0.15	0.00	0.34	0.20	0.37	0.32	0.21
TFAM	0.00	0.00	0.00	0.15	0.00	0.18	0.00	0.19	0.15	0.00	0.00	0.16	0.00	0.00	0.18	0.00	0.20	0.29	0.21	0.18	0.31
HADH	0.00	0.00	0.00	0.16	0.00	0.23	0.00	0.11	0.13	0.00	0.00	0.20	0.00	0.00	0.12	0.00	0.26	0.39	0.45	0.36	0.28
COX1	0.00	0.00	0.00	0.18	0.00	0.20	0.00	0.17	0.17	0.00	0.00	0.13	0.00	0.00	0.12	0.00	0.15	0.16	0.22	0.11	0.34
ACADVL	0.00	0.00	0.00	0.15	0.00	0.19	0.00	0.12	0.15	0.00	0.00	0.14	0.00	0.00	0.11	0.00	0.12	0.35	0.44	0.19	0.30
SIRT5	0.00	0.00	0.00	0.22	0.00	0.32	0.00	0.20	0.11	0.00	0.00	0.18	0.00	0.00	0.14	0.00	0.11	0.24	0.32	0.12	0.29
LONP1	0.00	0.00	0.00	0.22	0.00	0.38	0.00	0.23	0.15	0.00	0.00	0.18	0.00	0.00	0.14	0.00	0.11	0.20	0.29	0.12	0.42
SDHA	0.00	0.00	0.00	0.17	0.00	0.30	0.00	0.17	0.13	0.00	0.00	0.15	0.00	0.00	0.14	0.00	0.12	0.28	0.33	0.15	0.44
ACADM	0.00	0.00	0.00	0.15	0.00	0.15	0.00	0.12	0.13	0.00	0.00	0.14	0.00	0.00	0.13	0.00	0.10	0.32	0.41	0.15	0.28
GPD1L	0.00	0.00	0.00	0.16	0.00	0.25	0.00	0.18	0.15	0.00	0.00	0.12	0.00	0.00	0.13	0.00	0.11	0.14	0.21	0.11	0.13
FXN	0.00	0.00	0.00	0.14	0.00	0.18	0.00	0.18	0.21	0.00	0.00	0.15	0.00	0.00	0.22	0.00	0.13	0.19	0.26	0.12	0.28
G6PC3	0.00	0.00	0.00	0.19	0.00	0.25	0.00	0.14	0.15	0.00	0.00	0.16	0.00	0.00	0.12	0.00	0.20	0.22	0.32	0.31	0.11
SIRT4	0.00	0.00	0.00	0.20	0.00	0.30	0.00	0.18	0.12	0.00	0.00	0.16	0.00	0.00	0.13	0.00	0.22	0.31	0.38	0.25	0.28
DLD	0.00	0.00	0.00	0.15	0.00	0.27	0.00	0.12	0.00	0.00	0.00	0.24	0.13	0.00	0.17	0.00	0.11	0.28	0.36	0.17	0.33
PANK3	0.00	0.00	0.00	0.18	0.00	0.38	0.00	0.14	0.00	0.00	0.00	0.30	0.11	0.00	0.17	0.00	0.16	0.29	0.49	0.36	0.19
UQCRC2	0.00	0.00	0.00	0.23	0.00	0.43	0.00	0.10	0.00	0.00	0.00	0.26	0.12	0.00	0.21	0.00	0.12	0.22	0.34	0.11	0.52
COQ9	0.00	0.00	0.00	0.15	0.00	0.25	0.00	0.00	0.18	0.00	0.00	0.28	0.11	0.00	0.18	0.00	0.11	0.20	0.39	0.12	0.29
COQ10B	0.00	0.00	0.00	0.10	0.00	0.00	0.00	0.11	0.22	0.00	0.00	0.21	0.11	0.00	0.14	0.00	0.14	0.13	0.33	0.12	0.12
SLC25A29	0.00	0.00	0.00	0.20	0.00	0.33	0.00	0.00	0.00	0.00	0.12	0.25	0.13	0.00	0.18	0.00	0.14	0.26	0.35	0.17	0.32
DLST	0.00	0.00	0.00	0.18	0.00	0.32	0.00	0.00	0.00	0.00	0.00	0.28	0.14	0.10	0.17	0.00	0.10	0.22	0.36	0.15	0.35
TAS2R43	0.10	0.10	0.40	0.46	0.00	0.26	0.39	0.17	0.15	0.00	0.19	0.00	0.00	0.00	0.18	0.00	0.00	0.00	0.00	0.00	0.00
LPAR6	0.19	0.19	0.39	0.44	0.00	0.29	0.34	0.13	0.11	0.00	0.16	0.00	0.00	0.00	0.17	0.00	0.00	0.00	0.00	0.00	0.00
FSHR	0.16	0.16	0.23	0.22	0.21	0.00	0.19	0.14	0.00	0.28	0.15	0.00	0.00	0.00	0.12	0.00	0.00	0.00	0.00	0.00	0.00
VN1R1	0.16	0.16	0.42	0.52	0.12	0.34	0.42	0.00	0.00	0.00	0.25	0.23	0.00	0.00	0.32	0.00	0.00	0.00	0.00	0.00	0.00
VN1R7P	0.20	0.20	0.47	0.57	0.13	0.37	0.45	0.00	0.00	0.00	0.24	0.22	0.00	0.00	0.30	0.00	0.00	0.00	0.00	0.00	0.00
ERLIN2	0.12	0.12	0.30	0.47	0.00	0.48	0.31	0.26	0.00	0.00	0.29	0.14	0.00	0.00	0.20	0.00	0.00	0.00	0.00	0.00	0.00
GPR143	0.15	0.15	0.18	0.27	0.00	0.26	0.17	0.10	0.00	0.00	0.16	0.12	0.00	0.00	0.18	0.00	0.00	0.00	0.00	0.00	0.00
GPR1	0.14	0.14	0.31	0.41	0.00	0.32	0.31	0.18	0.00	0.00	0.21	0.18	0.00	0.00	0.21	0.00	0.00	0.00	0.00	0.00	0.00
RNF170	0.11	0.11	0.35	0.47	0.00	0.34	0.37	0.21	0.00	0.00	0.24	0.11	0.00	0.00	0.23	0.00	0.00	0.00	0.00	0.00	0.00
KLHL12	0.13	0.13	0.30	0.45	0.00	0.41	0.33	0.29	0.00	0.00	0.13	0.12	0.00	0.00	0.22	0.00	0.00	0.00	0.00	0.00	0.00

SNX1	0.11	0.11	0.28	0.44	0.00	0.39	0.32	0.29	0.00	0.00	0.17	0.13	0.00	0.00	0.15	0.00	0.00	0.00	0.00	0.00	0.00
GPRASP2	0.13	0.13	0.37	0.51	0.00	0.43	0.38	0.19	0.00	0.00	0.16	0.23	0.00	0.00	0.24	0.00	0.00	0.00	0.00	0.00	0.00
FZD4	0.00	0.00	0.21	0.27	0.00	0.19	0.23	0.24	0.21	0.21	0.17	0.12	0.00	0.00	0.20	0.00	0.00	0.00	0.00	0.00	0.00
TSPAN12	0.00	0.00	0.14	0.25	0.00	0.30	0.16	0.19	0.15	0.15	0.22	0.13	0.00	0.00	0.17	0.00	0.00	0.00	0.00	0.00	0.00
HTR7P1	0.19	0.19	0.42	0.52	0.00	0.32	0.42	0.00	0.00	0.00	0.18	0.24	0.11	0.00	0.22	0.00	0.00	0.00	0.00	0.00	0.00
LOC440683	0.24	0.24	0.45	0.53	0.00	0.34	0.42	0.00	0.00	0.00	0.18	0.18	0.10	0.00	0.20	0.00	0.00	0.00	0.00	0.00	0.00
DRD5P1	0.15	0.15	0.29	0.35	0.00	0.23	0.28	0.00	0.00	0.00	0.12	0.20	0.14	0.00	0.18	0.00	0.00	0.00	0.00	0.00	0.00
IGDCC4	0.00	0.00	0.16	0.38	0.00	0.52	0.21	0.20	0.13	0.00	0.20	0.32	0.12	0.00	0.28	0.00	0.00	0.00	0.00	0.00	0.00
SLC7A4	0.00	0.00	0.12	0.24	0.00	0.28	0.16	0.14	0.13	0.00	0.17	0.16	0.12	0.00	0.19	0.00	0.00	0.00	0.00	0.00	0.00
NLRP6	0.00	0.00	0.29	0.43	0.00	0.33	0.33	0.22	0.17	0.00	0.11	0.15	0.12	0.00	0.23	0.00	0.00	0.00	0.00	0.00	0.00
NPR2	0.00	0.00	0.17	0.26	0.00	0.12	0.22	0.16	0.12	0.00	0.16	0.17	0.11	0.00	0.23	0.00	0.00	0.00	0.00	0.00	0.00
ALG10	0.00	0.00	0.10	0.26	0.00	0.36	0.13	0.17	0.13	0.00	0.23	0.24	0.00	0.10	0.23	0.00	0.00	0.00	0.00	0.00	0.00
NRADDP	0.00	0.00	0.29	0.46	0.00	0.38	0.37	0.25	0.13	0.00	0.33	0.18	0.00	0.10	0.22	0.00	0.00	0.00	0.00	0.00	0.00
TM2D3	0.00	0.00	0.15	0.33	0.00	0.43	0.20	0.26	0.20	0.00	0.11	0.25	0.00	0.13	0.25	0.00	0.00	0.00	0.00	0.00	0.00
TMEM59	0.00	0.00	0.13	0.30	0.00	0.38	0.18	0.29	0.14	0.00	0.21	0.25	0.00	0.11	0.22	0.00	0.00	0.00	0.00	0.00	0.00
DRD3	0.11	0.11	0.16	0.17	0.00	0.00	0.14	0.00	0.13	0.00	0.00	0.12	0.16	0.12	0.15	0.00	0.00	0.00	0.00	0.00	0.00
PLK2	0.00	0.00	0.11	0.28	0.00	0.32	0.18	0.37	0.22	0.00	0.00	0.21	0.13	0.15	0.22	0.00	0.00	0.00	0.00	0.00	0.00
CHRFAM7A	0.00	0.00	0.13	0.25	0.00	0.21	0.20	0.13	0.13	0.00	0.00	0.14	0.10	0.12	0.16	0.00	0.00	0.00	0.00	0.00	0.00
XKR4	0.00	0.00	0.14	0.24	0.00	0.21	0.19	0.12	0.19	0.00	0.00	0.20	0.17	0.16	0.22	0.00	0.00	0.00	0.00	0.00	0.00
CHRNA6	0.00	0.00	0.14	0.23	0.00	0.12	0.21	0.10	0.11	0.00	0.00	0.20	0.19	0.18	0.24	0.00	0.00	0.00	0.00	0.00	0.00
PPP1R1B	0.00	0.00	0.17	0.23	0.00	0.13	0.18	0.23	0.24	0.00	0.00	0.20	0.25	0.23	0.20	0.00	0.00	0.00	0.00	0.00	0.00
NUDT9P1	0.00	0.00	0.16	0.28	0.00	0.22	0.23	0.15	0.22	0.00	0.00	0.15	0.12	0.14	0.21	0.00	0.00	0.00	0.00	0.00	0.00
YWHAH	0.00	0.00	0.13	0.21	0.00	0.16	0.15	0.19	0.10	0.00	0.00	0.18	0.14	0.16	0.18	0.00	0.00	0.00	0.00	0.00	0.00
AKAP5	0.00	0.00	0.21	0.27	0.00	0.19	0.21	0.19	0.10	0.00	0.00	0.23	0.20	0.26	0.21	0.00	0.00	0.00	0.00	0.00	0.00
YWHAQ	0.00	0.00	0.11	0.17	0.00	0.15	0.12	0.19	0.12	0.00	0.00	0.13	0.10	0.12	0.13	0.00	0.00	0.00	0.00	0.00	0.00
RASGRF2	0.00	0.00	0.21	0.35	0.00	0.34	0.24	0.28	0.11	0.00	0.00	0.25	0.15	0.22	0.26	0.00	0.00	0.00	0.00	0.00	0.00
WWC1	0.00	0.00	0.14	0.29	0.00	0.30	0.20	0.26	0.16	0.00	0.00	0.26	0.22	0.21	0.23	0.00	0.00	0.00	0.00	0.00	0.00
PEBP1	0.00	0.00	0.13	0.22	0.00	0.19	0.14	0.28	0.32	0.00	0.00	0.18	0.15	0.15	0.20	0.00	0.00	0.00	0.00	0.00	0.00
C14ORF28	0.00	0.00	0.21	0.31	0.00	0.23	0.24	0.14	0.15	0.00	0.00	0.28	0.29	0.21	0.25	0.00	0.00	0.00	0.00	0.00	0.00
ITPR1	0.00	0.00	0.16	0.22	0.00	0.11	0.17	0.12	0.10	0.00	0.00	0.11	0.16	0.17	0.15	0.00	0.00	0.00	0.00	0.00	0.00
PRKCG	0.00	0.00	0.13	0.17	0.00	0.12	0.13	0.14	0.13	0.00	0.00	0.13	0.17	0.19	0.18	0.00	0.00	0.00	0.00	0.00	0.00
C11ORF96	0.00	0.00	0.16	0.32	0.00	0.37	0.19	0.18	0.10	0.00	0.00	0.27	0.20	0.31	0.19	0.00	0.00	0.00	0.00	0.00	0.00
HIP1	0.00	0.00	0.19	0.36	0.00	0.31	0.27	0.23	0.16	0.00	0.00	0.24	0.11	0.11	0.26	0.00	0.00	0.00	0.00	0.00	0.00
CIB1	0.00	0.00	0.18	0.32	0.00	0.34	0.20	0.28	0.15	0.00	0.00	0.21	0.12	0.14	0.22	0.00	0.00	0.00	0.00	0.00	0.00
KIAA0355	0.00	0.00	0.19	0.28	0.00	0.27	0.21	0.16	0.14	0.00	0.00	0.17	0.14	0.15	0.16	0.00	0.00	0.00	0.00	0.00	0.00
SH3RF1	0.00	0.00	0.18	0.35	0.00	0.35	0.24	0.38	0.21	0.00	0.00	0.21	0.11	0.14	0.26	0.00	0.00	0.00	0.00	0.00	0.00
RANBP9	0.00	0.00	0.24	0.40	0.00	0.38	0.29	0.36	0.15	0.00	0.00	0.21	0.12	0.13	0.23	0.00	0.00	0.00	0.00	0.00	0.00
DLG2-AS1	0.00	0.00	0.12	0.31	0.00	0.42	0.16	0.14	0.12	0.00	0.00	0.29	0.21	0.23	0.25	0.00	0.00	0.00	0.00	0.00	0.00
UBE3A	0.00	0.00	0.11	0.21	0.00	0.18	0.15	0.27	0.17	0.00	0.00	0.24	0.17	0.19	0.24	0.00	0.00	0.00	0.00	0.00	0.00
FKBP5	0.00	0.00	0.13	0.25	0.00	0.19	0.20	0.25	0.22	0.00	0.00	0.12	0.12	0.14	0.18	0.00	0.00	0.00	0.00	0.00	0.00

CC2D1A	0.00	0.00	0.20	0.35	0.00	0.33	0.24	0.32	0.16	0.00	0.00	0.19	0.16	0.14	0.24	0.00	0.00	0.00	0.00	0.00	0.00
CAMK2N2	0.00	0.00	0.20	0.37	0.00	0.40	0.25	0.36	0.16	0.00	0.00	0.28	0.16	0.24	0.18	0.00	0.00	0.00	0.00	0.00	0.00
FOSB	0.00	0.00	0.14	0.24	0.00	0.18	0.17	0.30	0.24	0.00	0.00	0.17	0.16	0.19	0.22	0.00	0.00	0.00	0.00	0.00	0.00
YWHAE	0.00	0.00	0.11	0.16	0.00	0.12	0.11	0.19	0.14	0.00	0.00	0.17	0.15	0.15	0.16	0.00	0.00	0.00	0.00	0.00	0.00
PDE4D	0.00	0.00	0.14	0.18	0.00	0.14	0.12	0.15	0.13	0.00	0.00	0.21	0.18	0.16	0.19	0.00	0.00	0.00	0.00	0.00	0.00
YWHAB	0.00	0.00	0.10	0.15	0.00	0.12	0.11	0.19	0.13	0.00	0.00	0.15	0.13	0.16	0.14	0.00	0.00	0.00	0.00	0.00	0.00
YWHAG	0.00	0.00	0.12	0.20	0.00	0.17	0.14	0.21	0.15	0.00	0.00	0.20	0.15	0.16	0.19	0.00	0.00	0.00	0.00	0.00	0.00
SCAMP5	0.00	0.00	0.14	0.35	0.00	0.42	0.21	0.21	0.00	0.00	0.24	0.28	0.13	0.14	0.24	0.00	0.00	0.00	0.00	0.00	0.00
KIAA0319	0.00	0.00	0.12	0.26	0.00	0.30	0.16	0.14	0.00	0.00	0.12	0.25	0.20	0.14	0.25	0.00	0.00	0.00	0.00	0.00	0.00
SNX3	0.00	0.00	0.22	0.42	0.00	0.44	0.28	0.24	0.00	0.00	0.22	0.28	0.13	0.11	0.31	0.00	0.00	0.00	0.00	0.00	0.00
NAPA	0.00	0.00	0.16	0.30	0.00	0.27	0.21	0.21	0.00	0.00	0.20	0.19	0.12	0.12	0.26	0.00	0.00	0.00	0.00	0.00	0.00
ARVCF	0.00	0.00	0.11	0.25	0.00	0.32	0.14	0.14	0.00	0.00	0.18	0.21	0.14	0.11	0.23	0.00	0.00	0.00	0.00	0.00	0.00
C16ORF70	0.00	0.00	0.34	0.51	0.00	0.44	0.39	0.17	0.00	0.00	0.27	0.38	0.16	0.20	0.31	0.00	0.00	0.00	0.00	0.00	0.00
TSPAN7	0.00	0.00	0.15	0.34	0.00	0.44	0.20	0.13	0.00	0.00	0.22	0.31	0.16	0.13	0.29	0.00	0.00	0.00	0.00	0.00	0.00
DGCR6	0.00	0.00	0.21	0.40	0.00	0.48	0.25	0.14	0.00	0.00	0.15	0.32	0.14	0.11	0.34	0.00	0.00	0.00	0.00	0.00	0.00
STX12	0.00	0.00	0.19	0.36	0.00	0.35	0.25	0.23	0.00	0.00	0.20	0.23	0.10	0.14	0.27	0.00	0.00	0.00	0.00	0.00	0.00
SPTBN2	0.00	0.00	0.15	0.31	0.00	0.35	0.20	0.17	0.00	0.00	0.16	0.30	0.16	0.17	0.29	0.00	0.00	0.00	0.00	0.00	0.00
C3ORF58	0.00	0.00	0.11	0.33	0.00	0.54	0.14	0.18	0.00	0.00	0.16	0.30	0.13	0.11	0.28	0.00	0.00	0.00	0.00	0.00	0.00
AP3D1	0.00	0.00	0.12	0.27	0.00	0.27	0.19	0.17	0.00	0.00	0.16	0.22	0.11	0.15	0.26	0.00	0.00	0.00	0.00	0.00	0.00
SYT11	0.00	0.00	0.12	0.30	0.00	0.39	0.16	0.15	0.00	0.00	0.15	0.29	0.12	0.13	0.25	0.00	0.00	0.00	0.00	0.00	0.00
SNAP29	0.00	0.00	0.17	0.36	0.00	0.38	0.24	0.24	0.00	0.00	0.18	0.24	0.12	0.13	0.31	0.00	0.00	0.00	0.00	0.00	0.00
UNC50	0.00	0.00	0.20	0.47	0.00	0.61	0.26	0.22	0.00	0.00	0.24	0.32	0.17	0.12	0.30	0.00	0.00	0.00	0.00	0.00	0.00
PCDHA1	0.00	0.00	0.18	0.35	0.00	0.36	0.24	0.13	0.00	0.00	0.15	0.28	0.19	0.17	0.29	0.00	0.00	0.00	0.00	0.00	0.00
SHC2	0.00	0.00	0.29	0.44	0.00	0.36	0.35	0.24	0.00	0.00	0.16	0.28	0.16	0.21	0.33	0.00	0.00	0.00	0.00	0.00	0.00
SYT3	0.00	0.00	0.16	0.33	0.00	0.34	0.21	0.20	0.00	0.00	0.14	0.27	0.11	0.14	0.26	0.00	0.00	0.00	0.00	0.00	0.00
CSMD2	0.00	0.00	0.16	0.33	0.00	0.41	0.20	0.17	0.00	0.00	0.15	0.23	0.16	0.15	0.23	0.00	0.00	0.00	0.00	0.00	0.00
SLC9A9	0.00	0.00	0.12	0.28	0.00	0.37	0.15	0.14	0.00	0.00	0.22	0.29	0.20	0.16	0.21	0.00	0.00	0.00	0.00	0.00	0.00
SNAP91	0.00	0.00	0.16	0.32	0.00	0.30	0.23	0.16	0.00	0.00	0.11	0.33	0.17	0.22	0.28	0.00	0.00	0.00	0.00	0.00	0.00
ITM2C	0.00	0.00	0.10	0.31	0.00	0.49	0.14	0.15	0.00	0.00	0.18	0.33	0.13	0.15	0.26	0.00	0.00	0.00	0.00	0.00	0.00
CHRNA2	0.00	0.00	0.15	0.25	0.00	0.14	0.22	0.00	0.11	0.00	0.12	0.18	0.16	0.18	0.21	0.00	0.00	0.00	0.00	0.00	0.00
CHRNA4	0.00	0.00	0.11	0.18	0.00	0.00	0.16	0.12	0.15	0.00	0.11	0.15	0.18	0.19	0.18	0.00	0.00	0.00	0.00	0.00	0.00
CHP2	0.00	0.00	0.00	0.30	0.00	0.44	0.15	0.38	0.20	0.00	0.12	0.25	0.18	0.18	0.17	0.00	0.00	0.00	0.00	0.00	0.00
DNAJC9	0.00	0.00	0.00	0.31	0.00	0.54	0.12	0.21	0.11	0.00	0.11	0.30	0.17	0.12	0.25	0.00	0.00	0.00	0.00	0.00	0.00
LRRN4	0.00	0.00	0.00	0.29	0.00	0.45	0.14	0.32	0.28	0.00	0.13	0.27	0.13	0.15	0.24	0.00	0.00	0.00	0.00	0.00	0.00
PVRL1	0.00	0.00	0.16	0.26	0.00	0.17	0.22	0.18	0.14	0.00	0.30	0.17	0.00	0.00	0.22	0.13	0.00	0.00	0.00	0.00	0.00
EMP3	0.00	0.00	0.15	0.33	0.00	0.39	0.21	0.28	0.17	0.00	0.17	0.19	0.00	0.00	0.28	0.16	0.00	0.00	0.00	0.00	0.00
VAPB	0.00	0.00	0.13	0.26	0.00	0.27	0.16	0.24	0.18	0.00	0.22	0.16	0.00	0.00	0.25	0.10	0.00	0.00	0.00	0.00	0.00
SMPD4	0.00	0.00	0.15	0.33	0.00	0.43	0.20	0.35	0.16	0.00	0.18	0.23	0.00	0.00	0.20	0.11	0.00	0.00	0.00	0.00	0.00
HECW2	0.00	0.00	0.18	0.31	0.00	0.34	0.20	0.28	0.11	0.00	0.12	0.18	0.00	0.00	0.27	0.11	0.00	0.00	0.00	0.00	0.00
CLCF1	0.00	0.00	0.25	0.40	0.00	0.26	0.34	0.26	0.21	0.00	0.13	0.15	0.00	0.00	0.30	0.16	0.00	0.00	0.00	0.00	0.00

OSTM1	0.00	0.00	0.12	0.25	0.00	0.30	0.15	0.19	0.15	0.00	0.16	0.23	0.00	0.00	0.27	0.14	0.00	0.00	0.00	0.00	0.00
M6PR	0.00	0.00	0.21	0.33	0.00	0.22	0.27	0.16	0.14	0.00	0.20	0.18	0.00	0.00	0.23	0.10	0.00	0.00	0.00	0.00	0.00
TRPV3	0.00	0.00	0.20	0.27	0.00	0.15	0.23	0.18	0.20	0.00	0.13	0.12	0.00	0.00	0.24	0.10	0.00	0.00	0.00	0.00	0.00
DHH	0.00	0.00	0.14	0.24	0.00	0.19	0.18	0.24	0.15	0.00	0.11	0.14	0.00	0.00	0.27	0.15	0.00	0.00	0.00	0.00	0.00
MAL	0.00	0.00	0.12	0.27	0.00	0.29	0.18	0.24	0.17	0.00	0.20	0.13	0.00	0.00	0.26	0.20	0.00	0.00	0.00	0.00	0.00
TNFRSF21	0.00	0.00	0.25	0.41	0.00	0.31	0.34	0.32	0.31	0.00	0.19	0.18	0.00	0.00	0.28	0.19	0.00	0.00	0.00	0.00	0.00
CLDN5	0.00	0.00	0.11	0.19	0.00	0.17	0.13	0.16	0.21	0.00	0.19	0.17	0.00	0.00	0.21	0.15	0.00	0.00	0.00	0.00	0.00
GLDN	0.00	0.00	0.11	0.27	0.00	0.32	0.18	0.17	0.13	0.00	0.29	0.21	0.00	0.00	0.40	0.28	0.00	0.00	0.00	0.00	0.00
IL34	0.00	0.00	0.27	0.40	0.00	0.27	0.34	0.23	0.29	0.00	0.15	0.21	0.00	0.00	0.23	0.11	0.00	0.00	0.00	0.00	0.00
EMP2	0.00	0.00	0.19	0.37	0.00	0.40	0.26	0.31	0.19	0.00	0.24	0.17	0.00	0.00	0.27	0.12	0.00	0.00	0.00	0.00	0.00
CRLF1	0.00	0.00	0.22	0.38	0.00	0.29	0.32	0.24	0.21	0.00	0.15	0.15	0.00	0.00	0.31	0.16	0.00	0.00	0.00	0.00	0.00
EMCN	0.00	0.00	0.13	0.24	0.00	0.21	0.18	0.22	0.18	0.00	0.17	0.17	0.00	0.00	0.31	0.15	0.00	0.00	0.00	0.00	0.00
LAPTM5	0.00	0.00	0.17	0.39	0.00	0.42	0.26	0.37	0.18	0.00	0.15	0.19	0.00	0.00	0.25	0.11	0.00	0.00	0.00	0.00	0.00
TRPV2	0.00	0.00	0.19	0.26	0.00	0.15	0.21	0.19	0.20	0.00	0.14	0.13	0.00	0.00	0.23	0.12	0.00	0.00	0.00	0.00	0.00
ZNF592	0.00	0.00	0.13	0.29	0.00	0.40	0.17	0.28	0.14	0.00	0.00	0.25	0.13	0.00	0.24	0.11	0.00	0.00	0.00	0.00	0.00
NUB1	0.00	0.00	0.10	0.26	0.00	0.37	0.13	0.29	0.14	0.00	0.00	0.19	0.10	0.00	0.30	0.11	0.00	0.00	0.00	0.00	0.00
POU4F2	0.00	0.00	0.11	0.20	0.00	0.17	0.15	0.34	0.13	0.00	0.00	0.15	0.16	0.00	0.36	0.23	0.00	0.00	0.00	0.00	0.00
PDCD10	0.00	0.00	0.11	0.25	0.00	0.32	0.15	0.24	0.17	0.00	0.00	0.24	0.11	0.00	0.32	0.14	0.00	0.00	0.00	0.00	0.00
HDAC11	0.00	0.00	0.11	0.26	0.00	0.32	0.15	0.39	0.27	0.00	0.00	0.19	0.12	0.00	0.26	0.18	0.00	0.00	0.00	0.00	0.00
IKBKAP	0.00	0.00	0.14	0.26	0.00	0.28	0.17	0.21	0.20	0.00	0.00	0.17	0.12	0.00	0.29	0.15	0.00	0.00	0.00	0.00	0.00
RGNEF	0.00	0.00	0.23	0.34	0.00	0.31	0.24	0.28	0.15	0.00	0.00	0.20	0.11	0.00	0.29	0.14	0.00	0.00	0.00	0.00	0.00
KIF3A	0.00	0.00	0.11	0.20	0.00	0.20	0.13	0.20	0.11	0.00	0.00	0.18	0.13	0.00	0.26	0.15	0.00	0.00	0.00	0.00	0.00
MYO10	0.00	0.00	0.16	0.32	0.00	0.34	0.22	0.22	0.00	0.00	0.13	0.21	0.14	0.00	0.30	0.14	0.00	0.00	0.00	0.00	0.00
ZNF358	0.00	0.00	0.12	0.34	0.00	0.53	0.17	0.15	0.00	0.00	0.14	0.34	0.14	0.00	0.36	0.18	0.00	0.00	0.00	0.00	0.00
ESYT2	0.00	0.00	0.14	0.36	0.00	0.51	0.18	0.22	0.00	0.00	0.13	0.26	0.11	0.00	0.38	0.13	0.00	0.00	0.00	0.00	0.00
CELSR1	0.00	0.00	0.18	0.32	0.00	0.36	0.21	0.19	0.00	0.00	0.20	0.24	0.13	0.00	0.35	0.15	0.00	0.00	0.00	0.00	0.00
OR2C1	0.00	0.00	0.25	0.41	0.00	0.37	0.30	0.14	0.00	0.00	0.23	0.23	0.17	0.00	0.38	0.20	0.00	0.00	0.00	0.00	0.00
SLIT1	0.00	0.00	0.13	0.21	0.00	0.12	0.18	0.15	0.00	0.00	0.14	0.15	0.19	0.00	0.34	0.24	0.00	0.00	0.00	0.00	0.00
CLCN6	0.00	0.00	0.12	0.30	0.00	0.36	0.17	0.12	0.00	0.00	0.28	0.26	0.11	0.00	0.27	0.11	0.00	0.00	0.00	0.00	0.00
MAGI2	0.00	0.00	0.22	0.31	0.00	0.26	0.24	0.17	0.00	0.00	0.15	0.21	0.11	0.00	0.23	0.10	0.00	0.00	0.00	0.00	0.00
ANO6	0.00	0.00	0.13	0.35	0.00	0.51	0.17	0.15	0.00	0.00	0.29	0.32	0.12	0.00	0.37	0.17	0.00	0.00	0.00	0.00	0.00
FAM198B	0.00	0.00	0.10	0.28	0.00	0.41	0.14	0.17	0.00	0.00	0.16	0.35	0.18	0.00	0.40	0.20	0.00	0.00	0.00	0.00	0.00
PRICKLE2	0.00	0.00	0.13	0.28	0.00	0.37	0.16	0.23	0.00	0.00	0.12	0.24	0.12	0.00	0.30	0.17	0.00	0.00	0.00	0.00	0.00
FLOT2	0.00	0.00	0.22	0.33	0.00	0.28	0.24	0.21	0.00	0.00	0.21	0.20	0.12	0.00	0.25	0.10	0.00	0.00	0.00	0.00	0.00
TMEM35	0.00	0.00	0.19	0.26	0.00	0.22	0.19	0.15	0.00	0.00	0.10	0.15	0.14	0.00	0.30	0.11	0.00	0.00	0.00	0.00	0.00
DLL3	0.00	0.00	0.13	0.22	0.00	0.17	0.16	0.22	0.00	0.00	0.13	0.18	0.13	0.00	0.32	0.17	0.00	0.00	0.00	0.00	0.00
MACF1	0.00	0.00	0.12	0.32	0.00	0.43	0.17	0.20	0.00	0.00	0.14	0.26	0.13	0.00	0.31	0.11	0.00	0.00	0.00	0.00	0.00
SCHIP1	0.00	0.00	0.16	0.39	0.00	0.54	0.22	0.27	0.00	0.00	0.17	0.29	0.12	0.00	0.39	0.16	0.00	0.00	0.00	0.00	0.00
DCC	0.00	0.00	0.14	0.21	0.00	0.00	0.18	0.20	0.13	0.00	0.13	0.13	0.19	0.00	0.32	0.23	0.00	0.00	0.00	0.00	0.00
NTN1	0.00	0.00	0.14	0.20	0.00	0.00	0.18	0.21	0.16	0.00	0.12	0.14	0.20	0.00	0.34	0.25	0.00	0.00	0.00	0.00	0.00

LAMB2	0.00	0.00	0.00	0.16	0.00	0.15	0.12	0.12	0.12	0.00	0.11	0.15	0.11	0.00	0.23	0.14	0.00	0.00	0.00	0.00	0.00
ZC3H15	0.00	0.00	0.23	0.44	0.00	0.43	0.33	0.44	0.21	0.00	0.00	0.20	0.00	0.11	0.27	0.10	0.00	0.00	0.00	0.00	0.00
ZNF598	0.00	0.00	0.27	0.43	0.00	0.31	0.36	0.32	0.00	0.00	0.13	0.18	0.00	0.16	0.29	0.12	0.00	0.00	0.00	0.00	0.00
ACAP3	0.00	0.00	0.11	0.35	0.00	0.63	0.14	0.13	0.00	0.00	0.14	0.33	0.00	0.10	0.30	0.10	0.00	0.00	0.00	0.00	0.00
PPEF1	0.00	0.00	0.12	0.31	0.00	0.50	0.13	0.17	0.00	0.00	0.16	0.33	0.00	0.12	0.31	0.12	0.00	0.00	0.00	0.00	0.00
EPOR	0.00	0.00	0.12	0.19	0.00	0.00	0.15	0.15	0.17	0.00	0.11	0.12	0.00	0.10	0.18	0.12	0.00	0.00	0.00	0.00	0.00
SCN9A	0.00	0.00	0.00	0.16	0.00	0.13	0.11	0.13	0.19	0.00	0.15	0.15	0.00	0.11	0.28	0.15	0.00	0.00	0.00	0.00	0.00
TLX2	0.00	0.00	0.11	0.25	0.00	0.27	0.15	0.26	0.00	0.00	0.00	0.22	0.11	0.11	0.36	0.18	0.00	0.00	0.00	0.00	0.00
AGTPBP1	0.00	0.00	0.12	0.20	0.00	0.16	0.15	0.15	0.00	0.00	0.00	0.19	0.25	0.21	0.27	0.21	0.00	0.00	0.00	0.00	0.00
EFHC1	0.00	0.00	0.10	0.23	0.00	0.32	0.12	0.11	0.00	0.00	0.00	0.26	0.20	0.18	0.26	0.13	0.00	0.00	0.00	0.00	0.00
NPAS1	0.00	0.00	0.17	0.36	0.00	0.41	0.24	0.31	0.00	0.00	0.00	0.34	0.28	0.21	0.45	0.20	0.00	0.00	0.00	0.00	0.00
SIPA1L2	0.00	0.00	0.14	0.30	0.00	0.43	0.16	0.14	0.00	0.00	0.00	0.46	0.32	0.37	0.37	0.21	0.00	0.00	0.00	0.00	0.00
ARHGEF9	0.00	0.00	0.23	0.37	0.00	0.31	0.28	0.21	0.00	0.00	0.00	0.32	0.27	0.28	0.35	0.18	0.00	0.00	0.00	0.00	0.00
GABRA3	0.00	0.00	0.17	0.27	0.00	0.12	0.25	0.14	0.00	0.00	0.00	0.22	0.26	0.25	0.25	0.14	0.00	0.00	0.00	0.00	0.00
EGR4	0.00	0.00	0.12	0.31	0.00	0.41	0.17	0.24	0.00	0.00	0.00	0.23	0.18	0.18	0.29	0.11	0.00	0.00	0.00	0.00	0.00
RAPGEF5	0.00	0.00	0.17	0.29	0.00	0.35	0.18	0.22	0.00	0.00	0.00	0.23	0.21	0.17	0.27	0.11	0.00	0.00	0.00	0.00	0.00
RILPL2	0.00	0.00	0.14	0.30	0.00	0.37	0.19	0.29	0.00	0.00	0.00	0.27	0.15	0.14	0.29	0.17	0.00	0.00	0.00	0.00	0.00
BARHL1	0.00	0.00	0.17	0.31	0.00	0.30	0.22	0.36	0.00	0.00	0.00	0.29	0.28	0.18	0.51	0.34	0.00	0.00	0.00	0.00	0.00
TANC2	0.00	0.00	0.11	0.21	0.00	0.21	0.16	0.14	0.00	0.00	0.00	0.37	0.33	0.44	0.32	0.21	0.00	0.00	0.00	0.00	0.00
LOC280665	0.00	0.00	0.14	0.29	0.00	0.34	0.18	0.16	0.00	0.00	0.00	0.57	0.45	0.50	0.45	0.32	0.00	0.00	0.00	0.00	0.00
POU3F1	0.00	0.00	0.11	0.24	0.00	0.26	0.15	0.28	0.00	0.00	0.00	0.24	0.24	0.19	0.45	0.32	0.00	0.00	0.00	0.00	0.00
MYCBP2	0.00	0.00	0.25	0.41	0.00	0.37	0.28	0.33	0.00	0.00	0.00	0.32	0.23	0.19	0.42	0.20	0.00	0.00	0.00	0.00	0.00
NELF	0.00	0.00	0.16	0.28	0.00	0.34	0.16	0.21	0.00	0.00	0.00	0.30	0.18	0.21	0.36	0.17	0.00	0.00	0.00	0.00	0.00
AGBL1	0.00	0.00	0.14	0.34	0.00	0.51	0.17	0.19	0.00	0.00	0.00	0.32	0.16	0.12	0.32	0.13	0.00	0.00	0.00	0.00	0.00
FBXO33	0.00	0.00	0.12	0.37	0.00	0.57	0.18	0.31	0.00	0.00	0.00	0.30	0.16	0.19	0.29	0.11	0.00	0.00	0.00	0.00	0.00
PRKAR1B	0.00	0.00	0.16	0.30	0.00	0.29	0.20	0.22	0.00	0.00	0.00	0.28	0.25	0.25	0.31	0.13	0.00	0.00	0.00	0.00	0.00
CYFIP1	0.00	0.00	0.10	0.28	0.00	0.39	0.14	0.21	0.00	0.00	0.00	0.33	0.21	0.18	0.32	0.13	0.00	0.00	0.00	0.00	0.00
SIPA1L1	0.00	0.00	0.19	0.35	0.00	0.33	0.26	0.26	0.00	0.00	0.00	0.33	0.25	0.30	0.32	0.13	0.00	0.00	0.00	0.00	0.00
NRGN	0.00	0.00	0.16	0.28	0.00	0.26	0.19	0.23	0.00	0.00	0.00	0.35	0.34	0.38	0.32	0.18	0.00	0.00	0.00	0.00	0.00
DLGAP3	0.00	0.00	0.20	0.32	0.00	0.30	0.24	0.12	0.00	0.00	0.00	0.32	0.25	0.27	0.31	0.14	0.00	0.00	0.00	0.00	0.00
STMN4	0.00	0.00	0.15	0.37	0.00	0.47	0.20	0.24	0.00	0.00	0.00	0.45	0.31	0.26	0.47	0.25	0.00	0.00	0.00	0.00	0.00
PCP4	0.00	0.00	0.17	0.34	0.00	0.40	0.21	0.23	0.00	0.00	0.00	0.44	0.34	0.33	0.44	0.27	0.00	0.00	0.00	0.00	0.00
HMX3	0.00	0.00	0.10	0.24	0.00	0.29	0.15	0.23	0.00	0.00	0.00	0.23	0.17	0.10	0.37	0.18	0.00	0.00	0.00	0.00	0.00
VSTM2L	0.00	0.00	0.17	0.36	0.00	0.42	0.22	0.12	0.00	0.00	0.00	0.41	0.29	0.26	0.38	0.19	0.00	0.00	0.00	0.00	0.00
TBR1	0.00	0.00	0.11	0.19	0.00	0.17	0.14	0.23	0.00	0.00	0.00	0.21	0.34	0.24	0.31	0.25	0.00	0.00	0.00	0.00	0.00
HDGFRP3	0.00	0.00	0.16	0.39	0.00	0.53	0.22	0.31	0.00	0.00	0.00	0.45	0.31	0.28	0.50	0.25	0.00	0.00	0.00	0.00	0.00
SYN3	0.00	0.00	0.12	0.25	0.00	0.21	0.18	0.17	0.00	0.00	0.00	0.33	0.31	0.37	0.35	0.18	0.00	0.00	0.00	0.00	0.00
FAM124B	0.00	0.00	0.12	0.30	0.00	0.41	0.17	0.17	0.00	0.00	0.00	0.34	0.20	0.17	0.34	0.13	0.00	0.00	0.00	0.00	0.00
SHANK3	0.00	0.00	0.19	0.27	0.00	0.22	0.20	0.12	0.00	0.00	0.00	0.29	0.22	0.23	0.28	0.12	0.00	0.00	0.00	0.00	0.00
NHLH1	0.00	0.00	0.12	0.26	0.00	0.32	0.16	0.24	0.00	0.00	0.00	0.29	0.22	0.14	0.46	0.24	0.00	0.00	0.00	0.00	0.00

PSD	0.00	0.00	0.22	0.40	0.00	0.47	0.25	0.24	0.00	0.00	0.00	0.39	0.26	0.27	0.31	0.12	0.00	0.00	0.00	0.00	0.00
GABRA4	0.00	0.00	0.17	0.27	0.00	0.13	0.26	0.14	0.00	0.00	0.00	0.24	0.28	0.30	0.25	0.15	0.00	0.00	0.00	0.00	0.00
TTC3	0.00	0.00	0.10	0.35	0.00	0.61	0.14	0.20	0.00	0.00	0.00	0.37	0.18	0.15	0.36	0.12	0.00	0.00	0.00	0.00	0.00
PSD2	0.00	0.00	0.15	0.28	0.00	0.41	0.15	0.15	0.00	0.00	0.00	0.33	0.22	0.22	0.27	0.12	0.00	0.00	0.00	0.00	0.00
NAA16	0.00	0.00	0.16	0.39	0.00	0.55	0.21	0.30	0.00	0.00	0.00	0.30	0.13	0.10	0.29	0.10	0.00	0.00	0.00	0.00	0.00
SAMD4A	0.00	0.00	0.15	0.34	0.00	0.41	0.20	0.30	0.00	0.00	0.00	0.33	0.22	0.31	0.34	0.15	0.00	0.00	0.00	0.00	0.00
IQUB	0.00	0.00	0.14	0.36	0.00	0.47	0.19	0.17	0.00	0.00	0.00	0.37	0.22	0.18	0.33	0.12	0.00	0.00	0.00	0.00	0.00
CAMK1	0.00	0.00	0.15	0.24	0.00	0.23	0.16	0.24	0.00	0.00	0.00	0.23	0.18	0.22	0.22	0.10	0.00	0.00	0.00	0.00	0.00
FXR2	0.00	0.00	0.13	0.28	0.00	0.36	0.15	0.19	0.00	0.00	0.00	0.27	0.18	0.19	0.27	0.12	0.00	0.00	0.00	0.00	0.00
RND2	0.00	0.00	0.15	0.30	0.00	0.39	0.17	0.23	0.00	0.00	0.00	0.29	0.22	0.16	0.35	0.15	0.00	0.00	0.00	0.00	0.00
RIMS1	0.00	0.00	0.14	0.27	0.00	0.24	0.18	0.21	0.00	0.00	0.00	0.25	0.18	0.26	0.27	0.12	0.00	0.00	0.00	0.00	0.00
ERC2	0.00	0.00	0.10	0.28	0.00	0.34	0.17	0.13	0.00	0.00	0.00	0.35	0.20	0.24	0.34	0.14	0.00	0.00	0.00	0.00	0.00
CDK5R2	0.00	0.00	0.11	0.26	0.00	0.30	0.16	0.19	0.00	0.00	0.00	0.36	0.24	0.26	0.32	0.18	0.00	0.00	0.00	0.00	0.00
TPRG1L	0.00	0.00	0.11	0.32	0.00	0.43	0.17	0.24	0.00	0.00	0.00	0.31	0.18	0.21	0.30	0.11	0.00	0.00	0.00	0.00	0.00
IQSEC3	0.00	0.00	0.16	0.34	0.00	0.52	0.17	0.11	0.00	0.00	0.00	0.47	0.28	0.30	0.37	0.15	0.00	0.00	0.00	0.00	0.00
PCP2	0.00	0.00	0.19	0.33	0.00	0.32	0.22	0.19	0.00	0.00	0.00	0.36	0.29	0.23	0.46	0.26	0.00	0.00	0.00	0.00	0.00
DLGAP2	0.00	0.00	0.19	0.28	0.00	0.23	0.21	0.14	0.00	0.00	0.00	0.29	0.21	0.25	0.29	0.15	0.00	0.00	0.00	0.00	0.00
KNDC1	0.00	0.00	0.17	0.40	0.00	0.59	0.21	0.27	0.00	0.00	0.00	0.41	0.23	0.23	0.40	0.15	0.00	0.00	0.00	0.00	0.00
POU3F3	0.00	0.00	0.16	0.29	0.00	0.28	0.21	0.29	0.00	0.00	0.00	0.26	0.29	0.22	0.42	0.27	0.00	0.00	0.00	0.00	0.00
TRIM11	0.00	0.00	0.10	0.33	0.00	0.49	0.17	0.42	0.00	0.00	0.00	0.30	0.21	0.16	0.39	0.15	0.00	0.00	0.00	0.00	0.00
SHANK1	0.00	0.00	0.25	0.37	0.00	0.29	0.28	0.14	0.00	0.00	0.00	0.32	0.24	0.29	0.32	0.14	0.00	0.00	0.00	0.00	0.00
DPY19L1	0.00	0.00	0.13	0.30	0.00	0.43	0.16	0.25	0.00	0.00	0.00	0.51	0.51	0.39	0.54	0.36	0.00	0.00	0.00	0.00	0.00
BARHL2	0.00	0.00	0.15	0.29	0.00	0.28	0.21	0.35	0.00	0.00	0.00	0.29	0.29	0.17	0.53	0.37	0.00	0.00	0.00	0.00	0.00
BAIAP2	0.00	0.00	0.19	0.31	0.00	0.27	0.22	0.19	0.00	0.00	0.00	0.23	0.20	0.15	0.25	0.10	0.00	0.00	0.00	0.00	0.00
MARCKSL1	0.00	0.00	0.17	0.35	0.00	0.40	0.22	0.24	0.00	0.00	0.00	0.27	0.15	0.13	0.35	0.16	0.00	0.00	0.00	0.00	0.00
DAB1	0.00	0.00	0.13	0.21	0.00	0.15	0.18	0.16	0.00	0.00	0.00	0.17	0.24	0.21	0.22	0.15	0.00	0.00	0.00	0.00	0.00
NKX6-2	0.00	0.00	0.11	0.25	0.00	0.32	0.15	0.27	0.00	0.00	0.00	0.29	0.30	0.19	0.47	0.34	0.00	0.00	0.00	0.00	0.00
PROX2	0.00	0.00	0.12	0.31	0.00	0.44	0.17	0.26	0.00	0.00	0.00	0.35	0.26	0.22	0.42	0.23	0.00	0.00	0.00	0.00	0.00
DBN1	0.00	0.00	0.14	0.29	0.00	0.30	0.19	0.19	0.00	0.00	0.00	0.37	0.34	0.32	0.36	0.22	0.00	0.00	0.00	0.00	0.00
ABR	0.00	0.00	0.18	0.32	0.00	0.37	0.21	0.21	0.00	0.00	0.00	0.26	0.14	0.17	0.30	0.14	0.00	0.00	0.00	0.00	0.00
TANC1	0.00	0.00	0.14	0.30	0.00	0.40	0.17	0.15	0.00	0.00	0.00	0.36	0.24	0.33	0.29	0.11	0.00	0.00	0.00	0.00	0.00
LRRK1	0.00	0.00	0.12	0.30	0.00	0.43	0.15	0.14	0.00	0.00	0.00	0.38	0.25	0.19	0.38	0.17	0.00	0.00	0.00	0.00	0.00
LHX8	0.00	0.00	0.12	0.25	0.00	0.28	0.16	0.24	0.00	0.00	0.00	0.25	0.24	0.20	0.35	0.18	0.00	0.00	0.00	0.00	0.00
BSN	0.00	0.00	0.12	0.26	0.00	0.24	0.19	0.15	0.00	0.00	0.00	0.33	0.29	0.30	0.37	0.22	0.00	0.00	0.00	0.00	0.00
TAGLN3	0.00	0.00	0.15	0.39	0.00	0.53	0.21	0.22	0.00	0.00	0.00	0.45	0.34	0.31	0.38	0.16	0.00	0.00	0.00	0.00	0.00
EBF2	0.00	0.00	0.16	0.30	0.00	0.33	0.20	0.30	0.00	0.00	0.00	0.28	0.24	0.17	0.49	0.30	0.00	0.00	0.00	0.00	0.00
DDN	0.00	0.00	0.11	0.29	0.00	0.40	0.17	0.22	0.00	0.00	0.00	0.33	0.30	0.25	0.31	0.22	0.00	0.00	0.00	0.00	0.00
EFHA1	0.00	0.00	0.11	0.31	0.00	0.51	0.13	0.16	0.00	0.00	0.00	0.42	0.23	0.19	0.33	0.16	0.00	0.00	0.00	0.00	0.00
IGSF9B	0.00	0.00	0.12	0.27	0.00	0.27	0.20	0.26	0.00	0.00	0.00	0.40	0.34	0.38	0.47	0.29	0.00	0.00	0.00	0.00	0.00
SRGAP3	0.00	0.00	0.15	0.30	0.00	0.32	0.20	0.20	0.00	0.00	0.00	0.36	0.31	0.20	0.46	0.29	0.00	0.00	0.00	0.00	0.00

RIT2	0.00	0.00	0.26	0.39	0.00	0.35	0.28	0.28	0.00	0.00	0.00	0.27	0.17	0.18	0.35	0.12	0.00	0.00	0.00	0.00	0.00
NAB1	0.00	0.00	0.14	0.31	0.00	0.37	0.18	0.36	0.00	0.00	0.00	0.17	0.11	0.11	0.30	0.16	0.00	0.00	0.00	0.00	0.00
DCLK2	0.00	0.00	0.11	0.28	0.00	0.41	0.15	0.24	0.00	0.00	0.00	0.44	0.36	0.38	0.41	0.24	0.00	0.00	0.00	0.00	0.00
OCM	0.00	0.00	0.15	0.32	0.00	0.41	0.19	0.17	0.00	0.00	0.00	0.27	0.11	0.11	0.26	0.12	0.00	0.00	0.00	0.00	0.00
ZNF878	0.00	0.00	0.14	0.34	0.00	0.42	0.21	0.37	0.00	0.00	0.00	0.28	0.20	0.29	0.31	0.13	0.00	0.00	0.00	0.00	0.00
ZFHX2	0.00	0.00	0.12	0.36	0.00	0.55	0.18	0.34	0.00	0.00	0.00	0.53	0.38	0.38	0.50	0.28	0.00	0.00	0.00	0.00	0.00
FAM114A1	0.00	0.00	0.14	0.33	0.00	0.47	0.17	0.16	0.00	0.00	0.00	0.58	0.46	0.41	0.59	0.38	0.00	0.00	0.00	0.00	0.00
RAB40C	0.00	0.00	0.17	0.31	0.00	0.35	0.19	0.11	0.00	0.00	0.00	0.32	0.16	0.15	0.32	0.19	0.00	0.00	0.00	0.00	0.00
POU3F4	0.00	0.00	0.11	0.23	0.00	0.25	0.15	0.22	0.00	0.00	0.00	0.23	0.15	0.15	0.34	0.16	0.00	0.00	0.00	0.00	0.00
WASF1	0.00	0.00	0.14	0.26	0.00	0.25	0.17	0.18	0.00	0.00	0.00	0.20	0.17	0.11	0.23	0.13	0.00	0.00	0.00	0.00	0.00
MAP1A	0.00	0.00	0.15	0.30	0.00	0.31	0.20	0.16	0.00	0.00	0.00	0.32	0.22	0.20	0.37	0.16	0.00	0.00	0.00	0.00	0.00
CDKL1	0.00	0.00	0.11	0.30	0.00	0.46	0.14	0.14	0.00	0.00	0.00	0.43	0.24	0.23	0.31	0.14	0.00	0.00	0.00	0.00	0.00
ZSWIM6	0.00	0.00	0.14	0.30	0.00	0.41	0.16	0.12	0.00	0.00	0.00	0.25	0.16	0.23	0.23	0.10	0.00	0.00	0.00	0.00	0.00
PDYN	0.00	0.00	0.17	0.20	0.00	0.00	0.16	0.12	0.15	0.00	0.00	0.20	0.21	0.24	0.27	0.15	0.00	0.00	0.00	0.00	0.00
NTRK3	0.00	0.00	0.15	0.18	0.00	0.00	0.17	0.19	0.16	0.00	0.00	0.13	0.17	0.18	0.27	0.15	0.00	0.00	0.00	0.00	0.00
GABRA2	0.00	0.00	0.16	0.23	0.00	0.00	0.23	0.12	0.10	0.00	0.00	0.18	0.23	0.23	0.25	0.14	0.00	0.00	0.00	0.00	0.00
NTF3	0.00	0.00	0.11	0.15	0.00	0.00	0.13	0.17	0.16	0.00	0.00	0.12	0.17	0.20	0.27	0.18	0.00	0.00	0.00	0.00	0.00
GABRD	0.00	0.00	0.17	0.26	0.00	0.00	0.24	0.16	0.12	0.00	0.00	0.22	0.28	0.31	0.26	0.18	0.00	0.00	0.00	0.00	0.00
OLIG2	0.00	0.00	0.00	0.15	0.00	0.11	0.12	0.21	0.17	0.00	0.00	0.20	0.27	0.19	0.41	0.42	0.00	0.00	0.00	0.00	0.00
CEND1	0.00	0.00	0.00	0.22	0.00	0.30	0.12	0.27	0.13	0.00	0.00	0.40	0.39	0.31	0.52	0.39	0.00	0.00	0.00	0.00	0.00
NDRG2	0.00	0.00	0.00	0.22	0.00	0.26	0.12	0.37	0.30	0.00	0.00	0.22	0.15	0.14	0.23	0.13	0.00	0.00	0.00	0.00	0.00
NAIP	0.00	0.00	0.00	0.21	0.00	0.22	0.13	0.20	0.30	0.00	0.00	0.18	0.11	0.11	0.27	0.15	0.00	0.00	0.00	0.00	0.00
EMX1	0.00	0.00	0.00	0.15	0.00	0.12	0.11	0.21	0.13	0.00	0.00	0.24	0.39	0.32	0.34	0.30	0.00	0.00	0.00	0.00	0.00
SOX11	0.00	0.00	0.00	0.21	0.00	0.26	0.12	0.34	0.17	0.00	0.00	0.26	0.21	0.16	0.40	0.24	0.00	0.00	0.00	0.00	0.00
CPEB1	0.00	0.00	0.00	0.25	0.00	0.32	0.13	0.38	0.11	0.00	0.00	0.24	0.21	0.25	0.27	0.14	0.00	0.00	0.00	0.00	0.00
RBFOX3	0.00	0.00	0.00	0.17	0.00	0.13	0.12	0.20	0.14	0.00	0.00	0.24	0.31	0.29	0.32	0.28	0.00	0.00	0.00	0.00	0.00
KLK6	0.00	0.00	0.00	0.18	0.00	0.19	0.11	0.22	0.29	0.00	0.00	0.20	0.11	0.13	0.22	0.16	0.00	0.00	0.00	0.00	0.00
KLK8	0.00	0.00	0.00	0.23	0.00	0.24	0.15	0.23	0.25	0.00	0.00	0.29	0.24	0.28	0.33	0.23	0.00	0.00	0.00	0.00	0.00
RNF182	0.00	0.00	0.00	0.32	0.00	0.52	0.14	0.27	0.10	0.00	0.00	0.40	0.22	0.23	0.33	0.15	0.00	0.00	0.00	0.00	0.00
SERPINI1	0.00	0.00	0.00	0.23	0.00	0.25	0.14	0.23	0.21	0.00	0.00	0.35	0.28	0.31	0.40	0.27	0.00	0.00	0.00	0.00	0.00
SATB2	0.00	0.00	0.00	0.23	0.00	0.30	0.13	0.37	0.15	0.00	0.00	0.19	0.20	0.15	0.31	0.16	0.00	0.00	0.00	0.00	0.00
KIAA1191	0.00	0.00	0.00	0.25	0.00	0.40	0.11	0.15	0.15	0.00	0.00	0.39	0.21	0.25	0.27	0.12	0.00	0.00	0.00	0.00	0.00
SCRG1	0.00	0.00	0.00	0.31	0.00	0.48	0.15	0.19	0.10	0.00	0.00	0.37	0.17	0.18	0.33	0.19	0.00	0.00	0.00	0.00	0.00
FAM107A	0.00	0.00	0.00	0.26	0.00	0.47	0.10	0.28	0.13	0.00	0.00	0.29	0.14	0.11	0.27	0.12	0.00	0.00	0.00	0.00	0.00
SIM2	0.00	0.00	0.00	0.24	0.00	0.31	0.13	0.33	0.16	0.00	0.00	0.26	0.16	0.13	0.33	0.15	0.00	0.00	0.00	0.00	0.00
NOG	0.00	0.00	0.00	0.15	0.00	0.11	0.12	0.20	0.16	0.00	0.00	0.13	0.13	0.13	0.23	0.15	0.00	0.00	0.00	0.00	0.00
CELF4	0.00	0.00	0.00	0.24	0.00	0.35	0.11	0.22	0.14	0.00	0.00	0.26	0.12	0.16	0.31	0.12	0.00	0.00	0.00	0.00	0.00
FOXP2	0.00	0.00	0.00	0.17	0.00	0.20	0.10	0.22	0.14	0.00	0.00	0.31	0.33	0.26	0.36	0.20	0.00	0.00	0.00	0.00	0.00
YWHAQP2	0.00	0.00	0.00	0.21	0.00	0.30	0.10	0.11	0.13	0.00	0.00	0.27	0.17	0.19	0.32	0.23	0.00	0.00	0.00	0.00	0.00
SNCG	0.00	0.00	0.00	0.16	0.00	0.15	0.10	0.21	0.27	0.00	0.00	0.21	0.17	0.19	0.27	0.17	0.00	0.00	0.00	0.00	0.00

EOMES	0.00	0.00	0.00	0.18	0.00	0.13	0.13	0.28	0.13	0.00	0.00	0.17	0.22	0.16	0.28	0.21	0.00	0.00	0.00	0.00	0.00
ELP3	0.00	0.00	0.00	0.28	0.00	0.42	0.12	0.31	0.12	0.00	0.00	0.24	0.16	0.13	0.31	0.13	0.00	0.00	0.00	0.00	0.00
DOCK3	0.00	0.00	0.00	0.24	0.00	0.34	0.13	0.24	0.15	0.00	0.00	0.32	0.20	0.22	0.29	0.17	0.00	0.00	0.00	0.00	0.00
GAS7	0.00	0.00	0.00	0.28	0.00	0.41	0.14	0.35	0.18	0.00	0.00	0.33	0.22	0.21	0.35	0.17	0.00	0.00	0.00	0.00	0.00
ZBTB20	0.00	0.00	0.00	0.27	0.00	0.39	0.14	0.34	0.15	0.00	0.00	0.31	0.29	0.27	0.31	0.17	0.00	0.00	0.00	0.00	0.00
PRND	0.00	0.00	0.00	0.19	0.00	0.21	0.12	0.12	0.12	0.00	0.00	0.19	0.12	0.14	0.26	0.18	0.00	0.00	0.00	0.00	0.00
ZIC2	0.00	0.00	0.00	0.22	0.00	0.27	0.13	0.29	0.10	0.00	0.00	0.23	0.20	0.13	0.38	0.22	0.00	0.00	0.00	0.00	0.00
MSI1	0.00	0.00	0.00	0.19	0.00	0.23	0.11	0.33	0.21	0.00	0.00	0.24	0.22	0.19	0.31	0.21	0.00	0.00	0.00	0.00	0.00
PRDM10	0.00	0.00	0.00	0.26	0.00	0.39	0.14	0.31	0.12	0.00	0.00	0.40	0.27	0.23	0.48	0.30	0.00	0.00	0.00	0.00	0.00
DPYSL4	0.00	0.00	0.00	0.29	0.00	0.45	0.13	0.27	0.11	0.00	0.00	0.47	0.33	0.30	0.50	0.29	0.00	0.00	0.00	0.00	0.00
INA	0.00	0.00	0.00	0.23	0.00	0.25	0.15	0.20	0.11	0.00	0.00	0.31	0.24	0.22	0.44	0.31	0.00	0.00	0.00	0.00	0.00
ELAVL4	0.00	0.00	0.00	0.21	0.00	0.23	0.12	0.31	0.17	0.00	0.00	0.26	0.23	0.23	0.41	0.22	0.00	0.00	0.00	0.00	0.00
ELAVL3	0.00	0.00	0.00	0.24	0.00	0.31	0.12	0.32	0.11	0.00	0.00	0.27	0.20	0.16	0.44	0.21	0.00	0.00	0.00	0.00	0.00
VIP	0.00	0.00	0.13	0.14	0.00	0.00	0.10	0.00	0.18	0.11	0.00	0.11	0.12	0.12	0.22	0.13	0.00	0.00	0.00	0.00	0.00
ZACN	0.00	0.00	0.31	0.51	0.00	0.47	0.36	0.00	0.00	0.00	0.29	0.43	0.22	0.23	0.38	0.18	0.00	0.00	0.00	0.00	0.00
UMODL1	0.00	0.00	0.14	0.32	0.00	0.44	0.17	0.00	0.00	0.00	0.18	0.35	0.22	0.19	0.36	0.18	0.00	0.00	0.00	0.00	0.00
SYNPR	0.00	0.00	0.11	0.32	0.00	0.45	0.16	0.00	0.00	0.00	0.19	0.43	0.31	0.36	0.34	0.16	0.00	0.00	0.00	0.00	0.00
VEPH1	0.00	0.00	0.14	0.33	0.00	0.49	0.17	0.00	0.00	0.00	0.12	0.56	0.36	0.27	0.61	0.41	0.00	0.00	0.00	0.00	0.00
CNTNAP1	0.00	0.00	0.10	0.20	0.00	0.20	0.14	0.00	0.00	0.00	0.24	0.27	0.18	0.15	0.49	0.39	0.00	0.00	0.00	0.00	0.00
GABRQ	0.00	0.00	0.27	0.42	0.00	0.30	0.35	0.00	0.00	0.00	0.17	0.32	0.25	0.22	0.36	0.17	0.00	0.00	0.00	0.00	0.00
CXORF1	0.00	0.00	0.11	0.33	0.00	0.62	0.11	0.00	0.00	0.00	0.12	0.47	0.28	0.33	0.29	0.13	0.00	0.00	0.00	0.00	0.00
KCNK15	0.00	0.00	0.11	0.26	0.00	0.35	0.13	0.00	0.00	0.00	0.25	0.36	0.21	0.19	0.31	0.17	0.00	0.00	0.00	0.00	0.00
STAC	0.00	0.00	0.16	0.35	0.00	0.50	0.20	0.00	0.00	0.00	0.15	0.45	0.25	0.25	0.47	0.23	0.00	0.00	0.00	0.00	0.00
SHISA9	0.00	0.00	0.22	0.31	0.00	0.19	0.26	0.00	0.00	0.00	0.12	0.31	0.30	0.42	0.26	0.16	0.00	0.00	0.00	0.00	0.00
HMP19	0.00	0.00	0.11	0.34	0.00	0.57	0.14	0.00	0.00	0.00	0.15	0.48	0.28	0.25	0.47	0.26	0.00	0.00	0.00	0.00	0.00
CNGA1	0.00	0.00	0.14	0.26	0.00	0.26	0.16	0.00	0.00	0.00	0.14	0.27	0.16	0.17	0.29	0.13	0.00	0.00	0.00	0.00	0.00
GPM6B	0.00	0.00	0.12	0.30	0.00	0.39	0.17	0.00	0.00	0.00	0.21	0.43	0.30	0.21	0.52	0.40	0.00	0.00	0.00	0.00	0.00
JPH4	0.00	0.00	0.25	0.40	0.00	0.34	0.29	0.00	0.00	0.00	0.25	0.44	0.27	0.35	0.41	0.24	0.00	0.00	0.00	0.00	0.00
DLG2	0.00	0.00	0.16	0.23	0.00	0.18	0.18	0.00	0.00	0.00	0.13	0.24	0.16	0.18	0.26	0.14	0.00	0.00	0.00	0.00	0.00
OR10A2	0.00	0.00	0.23	0.34	0.00	0.25	0.28	0.00	0.00	0.00	0.21	0.26	0.22	0.18	0.38	0.22	0.00	0.00	0.00	0.00	0.00
CACNG3	0.00	0.00	0.22	0.37	0.00	0.31	0.28	0.00	0.00	0.00	0.20	0.34	0.21	0.26	0.33	0.17	0.00	0.00	0.00	0.00	0.00
CACNG7	0.00	0.00	0.26	0.40	0.00	0.29	0.32	0.00	0.00	0.00	0.23	0.32	0.17	0.24	0.28	0.15	0.00	0.00	0.00	0.00	0.00
WBSCR17	0.00	0.00	0.13	0.34	0.00	0.55	0.15	0.00	0.00	0.00	0.15	0.67	0.45	0.39	0.56	0.32	0.00	0.00	0.00	0.00	0.00
SLC1A7	0.00	0.00	0.10	0.27	0.00	0.28	0.17	0.00	0.00	0.00	0.23	0.22	0.18	0.20	0.23	0.11	0.00	0.00	0.00	0.00	0.00
KCNJ14	0.00	0.00	0.13	0.24	0.00	0.25	0.15	0.00	0.00	0.00	0.17	0.35	0.23	0.23	0.34	0.20	0.00	0.00	0.00	0.00	0.00
KIAA2022	0.00	0.00	0.17	0.35	0.00	0.49	0.18	0.00	0.00	0.00	0.13	0.52	0.33	0.28	0.45	0.21	0.00	0.00	0.00	0.00	0.00
GABRA1	0.00	0.00	0.13	0.19	0.00	0.00	0.19	0.13	0.00	0.00	0.10	0.13	0.21	0.23	0.16	0.13	0.00	0.00	0.00	0.00	0.00
GABRB2	0.00	0.00	0.17	0.25	0.00	0.00	0.24	0.12	0.00	0.00	0.11	0.17	0.23	0.22	0.21	0.12	0.00	0.00	0.00	0.00	0.00
GABRG2	0.00	0.00	0.16	0.23	0.00	0.00	0.23	0.12	0.00	0.00	0.12	0.16	0.22	0.22	0.21	0.13	0.00	0.00	0.00	0.00	0.00
SYNGR1	0.00	0.00	0.00	0.30	0.00	0.44	0.15	0.10	0.00	0.00	0.12	0.39	0.27	0.27	0.34	0.12	0.00	0.00	0.00	0.00	0.00

ANK3	0.00	0.00	0.00	0.20	0.00	0.22	0.12	0.12	0.00	0.00	0.21	0.24	0.18	0.18	0.31	0.15	0.00	0.00	0.00	0.00	0.00
CDH6	0.00	0.00	0.00	0.20	0.00	0.22	0.13	0.24	0.00	0.00	0.14	0.24	0.24	0.12	0.40	0.26	0.00	0.00	0.00	0.00	0.00
ATP1B2	0.00	0.00	0.00	0.24	0.00	0.26	0.16	0.12	0.00	0.00	0.18	0.24	0.15	0.11	0.23	0.12	0.00	0.00	0.00	0.00	0.00
MOBP	0.00	0.00	0.00	0.23	0.00	0.35	0.11	0.10	0.00	0.00	0.11	0.28	0.18	0.12	0.41	0.35	0.00	0.00	0.00	0.00	0.00
SPTBN4	0.00	0.00	0.00	0.20	0.00	0.28	0.11	0.11	0.00	0.00	0.17	0.30	0.18	0.20	0.42	0.27	0.00	0.00	0.00	0.00	0.00
OTOS	0.00	0.00	0.00	0.25	0.00	0.42	0.10	0.15	0.00	0.00	0.15	0.27	0.12	0.13	0.32	0.15	0.00	0.00	0.00	0.00	0.00
KCNT1	0.00	0.00	0.00	0.23	0.00	0.26	0.13	0.13	0.00	0.00	0.19	0.29	0.19	0.20	0.28	0.15	0.00	0.00	0.00	0.00	0.00
KIFAP3	0.00	0.00	0.00	0.28	0.00	0.39	0.14	0.19	0.00	0.00	0.11	0.26	0.17	0.13	0.30	0.14	0.00	0.00	0.00	0.00	0.00
KCNIP4	0.00	0.00	0.00	0.21	0.00	0.30	0.10	0.16	0.00	0.00	0.16	0.30	0.18	0.24	0.24	0.13	0.00	0.00	0.00	0.00	0.00
CDH8	0.00	0.00	0.00	0.22	0.00	0.25	0.14	0.20	0.00	0.00	0.13	0.29	0.38	0.26	0.42	0.28	0.00	0.00	0.00	0.00	0.00
SYT14	0.00	0.00	0.00	0.31	0.00	0.51	0.12	0.13	0.00	0.00	0.17	0.38	0.16	0.15	0.32	0.12	0.00	0.00	0.00	0.00	0.00
SLC12A7	0.00	0.00	0.00	0.23	0.00	0.23	0.14	0.18	0.00	0.00	0.15	0.22	0.17	0.12	0.26	0.13	0.00	0.00	0.00	0.00	0.00
TMOD2	0.00	0.00	0.00	0.30	0.00	0.51	0.12	0.19	0.00	0.00	0.14	0.43	0.24	0.25	0.33	0.13	0.00	0.00	0.00	0.00	0.00
SLC17A8	0.00	0.00	0.00	0.19	0.00	0.17	0.13	0.14	0.00	0.00	0.12	0.28	0.34	0.31	0.39	0.26	0.00	0.00	0.00	0.00	0.00
C4ORF48	0.00	0.00	0.00	0.29	0.00	0.53	0.11	0.13	0.00	0.00	0.13	0.47	0.32	0.26	0.42	0.22	0.00	0.00	0.00	0.00	0.00
MFSD4	0.00	0.00	0.00	0.27	0.00	0.36	0.15	0.25	0.00	0.00	0.10	0.36	0.20	0.20	0.46	0.23	0.00	0.00	0.00	0.00	0.00
RUFY3	0.00	0.00	0.00	0.32	0.00	0.49	0.16	0.27	0.00	0.00	0.10	0.41	0.26	0.24	0.47	0.24	0.00	0.00	0.00	0.00	0.00
ATP6V1G1	0.00	0.00	0.00	0.26	0.00	0.41	0.11	0.11	0.00	0.00	0.17	0.35	0.20	0.11	0.28	0.11	0.00	0.00	0.00	0.00	0.00
KCNAB2	0.00	0.00	0.00	0.23	0.00	0.29	0.14	0.14	0.00	0.00	0.15	0.34	0.18	0.20	0.37	0.24	0.00	0.00	0.00	0.00	0.00
MPPED1	0.00	0.00	0.00	0.30	0.00	0.55	0.11	0.11	0.00	0.00	0.12	0.51	0.35	0.28	0.44	0.25	0.00	0.00	0.00	0.00	0.00
KCNAB1	0.00	0.00	0.00	0.18	0.00	0.22	0.10	0.11	0.00	0.00	0.12	0.30	0.18	0.25	0.30	0.19	0.00	0.00	0.00	0.00	0.00
RBPMS2	0.00	0.00	0.00	0.32	0.00	0.50	0.15	0.16	0.00	0.00	0.13	0.34	0.20	0.18	0.36	0.20	0.00	0.00	0.00	0.00	0.00
TOR1B	0.00	0.00	0.00	0.29	0.00	0.48	0.13	0.12	0.00	0.00	0.20	0.44	0.29	0.22	0.45	0.25	0.00	0.00	0.00	0.00	0.00
ADAMTS20	0.00	0.00	0.00	0.21	0.00	0.27	0.13	0.11	0.00	0.00	0.13	0.23	0.14	0.16	0.26	0.11	0.00	0.00	0.00	0.00	0.00
ATP1A3	0.00	0.00	0.00	0.20	0.00	0.20	0.14	0.12	0.00	0.00	0.13	0.27	0.21	0.19	0.25	0.12	0.00	0.00	0.00	0.00	0.00
KIF3C	0.00	0.00	0.00	0.32	0.00	0.52	0.14	0.12	0.00	0.00	0.15	0.40	0.21	0.16	0.43	0.22	0.00	0.00	0.00	0.00	0.00
TMEM163	0.00	0.00	0.00	0.30	0.00	0.43	0.15	0.16	0.00	0.00	0.20	0.37	0.27	0.26	0.31	0.14	0.00	0.00	0.00	0.00	0.00
SCN3A	0.00	0.00	0.00	0.18	0.00	0.19	0.11	0.00	0.12	0.00	0.17	0.24	0.15	0.21	0.31	0.19	0.00	0.00	0.00	0.00	0.00
HCN2	0.00	0.00	0.00	0.14	0.00	0.11	0.00	0.14	0.13	0.00	0.13	0.20	0.19	0.21	0.26	0.16	0.00	0.00	0.00	0.00	0.00
HCN3	0.00	0.00	0.00	0.18	0.00	0.21	0.00	0.13	0.11	0.00	0.11	0.32	0.24	0.24	0.35	0.21	0.00	0.00	0.00	0.00	0.00
KCNQ2	0.00	0.00	0.00	0.14	0.00	0.13	0.00	0.14	0.14	0.00	0.16	0.22	0.17	0.23	0.28	0.18	0.00	0.00	0.00	0.00	0.00
SGCE	0.00	0.00	0.00	0.14	0.00	0.19	0.00	0.11	0.19	0.00	0.12	0.25	0.17	0.14	0.27	0.12	0.00	0.00	0.00	0.00	0.00
KCND3	0.00	0.00	0.00	0.15	0.00	0.16	0.00	0.15	0.12	0.00	0.12	0.24	0.18	0.23	0.24	0.15	0.00	0.00	0.00	0.00	0.00
KCND2	0.00	0.00	0.00	0.15	0.00	0.15	0.00	0.14	0.12	0.00	0.12	0.22	0.19	0.26	0.22	0.15	0.00	0.00	0.00	0.00	0.00
KCNIP2	0.00	0.00	0.00	0.18	0.00	0.22	0.00	0.15	0.10	0.00	0.14	0.23	0.14	0.18	0.22	0.12	0.00	0.00	0.00	0.00	0.00
KCNA2	0.00	0.00	0.00	0.15	0.00	0.16	0.00	0.13	0.13	0.00	0.15	0.19	0.12	0.17	0.27	0.20	0.00	0.00	0.00	0.00	0.00
KCNA6	0.00	0.00	0.00	0.13	0.00	0.23	0.00	0.11	0.10	0.00	0.13	0.27	0.13	0.19	0.28	0.20	0.00	0.00	0.00	0.00	0.00
SEMA3A	0.00	0.00	0.00	0.15	0.00	0.00	0.12	0.16	0.15	0.00	0.12	0.15	0.22	0.16	0.30	0.21	0.00	0.00	0.00	0.00	0.00
NRP2	0.00	0.00	0.00	0.15	0.00	0.00	0.14	0.16	0.16	0.00	0.12	0.13	0.20	0.15	0.27	0.18	0.00	0.00	0.00	0.00	0.00
L1CAM	0.00	0.00	0.00	0.13	0.00	0.00	0.11	0.15	0.13	0.00	0.16	0.12	0.20	0.12	0.25	0.17	0.00	0.00	0.00	0.00	0.00

BST1	0.00	0.00	0.18	0.32	0.00	0.28	0.23	0.18	0.16	0.00	0.19	0.13	0.00	0.00	0.15	0.00	0.12	0.00	0.00	0.00	0.00
HSPA13	0.00	0.00	0.12	0.31	0.00	0.44	0.17	0.20	0.13	0.00	0.11	0.20	0.00	0.00	0.21	0.00	0.10	0.00	0.00	0.00	0.00
SPINK4	0.00	0.00	0.19	0.36	0.00	0.45	0.21	0.11	0.00	0.00	0.14	0.27	0.11	0.00	0.30	0.00	0.12	0.00	0.00	0.00	0.00
KLF16	0.00	0.00	0.18	0.38	0.00	0.45	0.23	0.33	0.00	0.00	0.00	0.23	0.15	0.12	0.26	0.00	0.10	0.00	0.00	0.00	0.00
OLIG3	0.00	0.00	0.00	0.18	0.00	0.19	0.12	0.19	0.11	0.00	0.00	0.15	0.16	0.00	0.31	0.24	0.15	0.00	0.00	0.00	0.00
NKX2-2	0.00	0.00	0.00	0.16	0.00	0.14	0.12	0.27	0.13	0.00	0.00	0.15	0.18	0.00	0.35	0.31	0.12	0.00	0.00	0.00	0.00
KIAA1598	0.00	0.00	0.00	0.19	0.00	0.24	0.11	0.15	0.00	0.00	0.00	0.29	0.20	0.15	0.34	0.17	0.13	0.00	0.00	0.00	0.00
CAST	0.00	0.00	0.00	0.16	0.00	0.16	0.00	0.20	0.23	0.00	0.00	0.20	0.13	0.17	0.21	0.12	0.11	0.00	0.00	0.00	0.00
SLC44A4	0.00	0.00	0.00	0.21	0.00	0.33	0.10	0.00	0.00	0.00	0.12	0.22	0.14	0.12	0.28	0.15	0.12	0.00	0.00	0.00	0.00
DNM1	0.00	0.00	0.15	0.23	0.00	0.16	0.17	0.15	0.00	0.00	0.00	0.21	0.12	0.12	0.19	0.00	0.00	0.11	0.00	0.00	0.00
PRKAR1A	0.00	0.00	0.10	0.16	0.00	0.15	0.11	0.20	0.00	0.00	0.00	0.13	0.14	0.11	0.15	0.00	0.00	0.11	0.00	0.00	0.00
RHEBP1	0.00	0.00	0.15	0.36	0.00	0.50	0.20	0.33	0.00	0.00	0.00	0.30	0.15	0.15	0.28	0.00	0.00	0.11	0.00	0.00	0.00
CRHR1	0.00	0.00	0.16	0.15	0.00	0.00	0.13	0.11	0.14	0.00	0.00	0.11	0.12	0.14	0.18	0.00	0.00	0.12	0.00	0.00	0.00
CRHR2	0.00	0.00	0.15	0.16	0.00	0.00	0.13	0.11	0.16	0.00	0.00	0.12	0.11	0.12	0.20	0.00	0.00	0.16	0.00	0.00	0.00
CHAT	0.00	0.00	0.00	0.13	0.00	0.10	0.00	0.15	0.14	0.00	0.00	0.16	0.21	0.17	0.30	0.18	0.00	0.12	0.00	0.00	0.00
BBS1	0.00	0.00	0.11	0.20	0.00	0.27	0.11	0.13	0.16	0.00	0.00	0.17	0.00	0.00	0.22	0.00	0.12	0.11	0.00	0.00	0.00
GAD1	0.00	0.00	0.00	0.10	0.00	0.00	0.00	0.14	0.11	0.00	0.00	0.14	0.27	0.22	0.20	0.15	0.14	0.11	0.00	0.00	0.00
ADAM17	0.13	0.13	0.11	0.13	0.00	0.00	0.10	0.14	0.21	0.00	0.16	0.00	0.00	0.00	0.10	0.00	0.00	0.00	0.10	0.00	0.00
SEC63	0.00	0.00	0.13	0.31	0.00	0.47	0.15	0.13	0.12	0.00	0.22	0.16	0.00	0.00	0.17	0.00	0.00	0.00	0.11	0.00	0.00
HYAL3	0.00	0.00	0.13	0.28	0.00	0.31	0.18	0.19	0.21	0.00	0.15	0.19	0.00	0.00	0.18	0.00	0.00	0.00	0.15	0.00	0.00
COL4A3BP	0.00	0.00	0.12	0.29	0.00	0.35	0.17	0.26	0.15	0.00	0.18	0.11	0.00	0.00	0.16	0.00	0.00	0.00	0.14	0.00	0.00
FAF2	0.00	0.00	0.10	0.31	0.00	0.49	0.16	0.38	0.12	0.00	0.11	0.17	0.00	0.00	0.19	0.00	0.00	0.00	0.14	0.00	0.00
MBTPS2	0.00	0.00	0.12	0.26	0.00	0.40	0.12	0.23	0.10	0.00	0.25	0.15	0.00	0.00	0.19	0.00	0.00	0.00	0.13	0.00	0.00
AMFR	0.00	0.00	0.12	0.26	0.00	0.29	0.16	0.32	0.23	0.00	0.17	0.14	0.00	0.00	0.17	0.00	0.00	0.00	0.12	0.00	0.00
SCARB2	0.00	0.00	0.17	0.32	0.00	0.28	0.24	0.16	0.14	0.00	0.21	0.15	0.00	0.00	0.20	0.00	0.00	0.00	0.13	0.00	0.00
OSTBETA	0.00	0.00	0.17	0.31	0.00	0.26	0.23	0.19	0.10	0.00	0.21	0.15	0.00	0.00	0.16	0.00	0.00	0.00	0.21	0.00	0.00
HFE2	0.00	0.00	0.13	0.24	0.00	0.18	0.19	0.12	0.14	0.00	0.17	0.11	0.00	0.00	0.21	0.00	0.00	0.00	0.18	0.00	0.00
GMDS	0.00	0.00	0.14	0.35	0.00	0.48	0.19	0.29	0.18	0.00	0.12	0.25	0.00	0.00	0.20	0.00	0.00	0.00	0.17	0.00	0.00
OSTALPHA	0.00	0.00	0.16	0.29	0.00	0.23	0.22	0.19	0.12	0.00	0.19	0.14	0.00	0.00	0.15	0.00	0.00	0.00	0.21	0.00	0.00
SUMF2	0.00	0.00	0.11	0.31	0.00	0.47	0.15	0.21	0.14	0.00	0.14	0.17	0.00	0.00	0.17	0.00	0.00	0.00	0.17	0.00	0.00
LAMP2	0.00	0.00	0.11	0.21	0.00	0.20	0.15	0.14	0.15	0.00	0.14	0.14	0.00	0.00	0.17	0.00	0.00	0.00	0.11	0.00	0.00
KDSR	0.00	0.00	0.16	0.31	0.00	0.38	0.19	0.19	0.15	0.00	0.11	0.21	0.00	0.00	0.27	0.00	0.00	0.00	0.11	0.00	0.00
ABCA7	0.00	0.00	0.12	0.27	0.00	0.33	0.17	0.17	0.12	0.00	0.17	0.20	0.00	0.00	0.19	0.00	0.00	0.00	0.19	0.00	0.00
MGAT3	0.00	0.00	0.11	0.26	0.00	0.28	0.17	0.26	0.24	0.00	0.15	0.19	0.00	0.00	0.16	0.00	0.00	0.00	0.13	0.00	0.00
APOL2	0.00	0.00	0.21	0.40	0.00	0.43	0.26	0.22	0.11	0.00	0.12	0.19	0.00	0.00	0.19	0.00	0.00	0.00	0.13	0.00	0.00
TMPRSS6	0.00	0.00	0.13	0.25	0.00	0.22	0.18	0.20	0.19	0.00	0.13	0.10	0.00	0.00	0.16	0.00	0.00	0.00	0.20	0.00	0.00
A4GALT	0.00	0.00	0.12	0.25	0.00	0.29	0.16	0.17	0.21	0.00	0.15	0.15	0.00	0.00	0.19	0.00	0.00	0.00	0.12	0.00	0.00
PPIL2	0.00	0.00	0.13	0.34	0.00	0.45	0.20	0.24	0.11	0.00	0.18	0.21	0.00	0.00	0.18	0.00	0.00	0.00	0.12	0.00	0.00
EBP	0.00	0.00	0.11	0.26	0.00	0.34	0.14	0.12	0.11	0.00	0.13	0.21	0.00	0.00	0.16	0.00	0.00	0.00	0.25	0.00	0.00
FLVCR1	0.00	0.00	0.14	0.32	0.00	0.37	0.20	0.18	0.12	0.00	0.22	0.20	0.00	0.00	0.27	0.00	0.00	0.00	0.12	0.00	0.00

CYS1	0.00	0.00	0.13	0.21	0.00	0.19	0.15	0.15	0.19	0.00	0.11	0.00	0.10	0.00	0.13	0.00	0.00	0.00	0.11	0.00	0.00
SOAT1	0.00	0.00	0.11	0.23	0.00	0.20	0.16	0.19	0.18	0.00	0.00	0.15	0.11	0.00	0.11	0.00	0.00	0.00	0.29	0.00	0.00
XPNPEP1	0.00	0.00	0.11	0.25	0.00	0.33	0.13	0.15	0.16	0.00	0.00	0.24	0.13	0.00	0.17	0.00	0.00	0.00	0.24	0.00	0.00
UPP2	0.00	0.00	0.16	0.32	0.00	0.39	0.19	0.22	0.18	0.00	0.00	0.25	0.11	0.00	0.18	0.00	0.00	0.00	0.33	0.00	0.00
APOL3	0.00	0.00	0.11	0.29	0.00	0.41	0.15	0.17	0.11	0.00	0.00	0.22	0.10	0.00	0.16	0.00	0.00	0.00	0.13	0.00	0.00
OSBPL6	0.00	0.00	0.18	0.44	0.00	0.71	0.19	0.18	0.00	0.00	0.27	0.38	0.13	0.00	0.28	0.00	0.00	0.00	0.19	0.00	0.00
LAPTM4A	0.00	0.00	0.16	0.38	0.00	0.52	0.21	0.11	0.00	0.00	0.34	0.25	0.11	0.00	0.20	0.00	0.00	0.00	0.13	0.00	0.00
ZDHHC13	0.00	0.00	0.00	0.27	0.00	0.38	0.14	0.17	0.15	0.00	0.11	0.30	0.15	0.00	0.24	0.00	0.00	0.00	0.15	0.00	0.00
ABCA5	0.00	0.00	0.00	0.27	0.00	0.42	0.13	0.17	0.15	0.00	0.17	0.25	0.11	0.00	0.20	0.00	0.00	0.00	0.17	0.00	0.00
APBB3	0.00	0.00	0.12	0.31	0.00	0.43	0.17	0.18	0.00	0.00	0.15	0.27	0.00	0.13	0.19	0.00	0.00	0.00	0.13	0.00	0.00
APH1B	0.00	0.00	0.00	0.20	0.00	0.27	0.11	0.14	0.12	0.00	0.20	0.19	0.00	0.11	0.17	0.00	0.00	0.00	0.11	0.00	0.00
PZP	0.00	0.00	0.15	0.30	0.00	0.27	0.21	0.13	0.00	0.00	0.00	0.22	0.11	0.10	0.24	0.00	0.00	0.00	0.15	0.00	0.00
NXF2	0.00	0.00	0.14	0.36	0.00	0.51	0.19	0.25	0.00	0.00	0.00	0.25	0.15	0.13	0.28	0.00	0.00	0.00	0.11	0.00	0.00
USP48	0.00	0.00	0.12	0.35	0.00	0.54	0.17	0.17	0.00	0.00	0.00	0.35	0.16	0.13	0.27	0.00	0.00	0.00	0.13	0.00	0.00
PIP4K2A	0.00	0.00	0.13	0.27	0.00	0.29	0.16	0.19	0.00	0.00	0.00	0.22	0.18	0.14	0.19	0.00	0.00	0.00	0.14	0.00	0.00
DGKI	0.00	0.00	0.13	0.30	0.00	0.38	0.18	0.20	0.00	0.00	0.00	0.28	0.18	0.17	0.29	0.00	0.00	0.00	0.12	0.00	0.00
TSNAX	0.00	0.00	0.12	0.30	0.00	0.41	0.15	0.19	0.00	0.00	0.00	0.28	0.25	0.21	0.26	0.00	0.00	0.00	0.11	0.00	0.00
HNMT	0.00	0.00	0.12	0.19	0.00	0.16	0.14	0.00	0.16	0.00	0.00	0.19	0.14	0.10	0.21	0.00	0.00	0.00	0.22	0.00	0.00
ANKH	0.00	0.00	0.00	0.18	0.00	0.16	0.13	0.13	0.17	0.00	0.00	0.13	0.11	0.11	0.18	0.00	0.00	0.00	0.15	0.00	0.00
RAI1	0.00	0.00	0.00	0.21	0.00	0.27	0.12	0.20	0.17	0.00	0.00	0.22	0.10	0.10	0.26	0.00	0.00	0.00	0.13	0.00	0.00
IMPA2	0.00	0.00	0.00	0.23	0.00	0.31	0.11	0.16	0.15	0.00	0.00	0.19	0.13	0.12	0.20	0.00	0.00	0.00	0.13	0.00	0.00
EEF1A2	0.00	0.00	0.00	0.24	0.00	0.27	0.15	0.26	0.23	0.00	0.00	0.23	0.15	0.13	0.24	0.00	0.00	0.00	0.13	0.00	0.00
NQO2	0.00	0.00	0.00	0.16	0.00	0.15	0.11	0.18	0.18	0.00	0.00	0.16	0.12	0.14	0.14	0.00	0.00	0.00	0.20	0.00	0.00
SMS	0.00	0.00	0.00	0.21	0.00	0.30	0.11	0.16	0.12	0.00	0.00	0.29	0.16	0.11	0.23	0.00	0.00	0.00	0.25	0.00	0.00
DGCR8	0.00	0.00	0.00	0.21	0.00	0.28	0.10	0.47	0.20	0.00	0.00	0.18	0.15	0.13	0.24	0.00	0.00	0.00	0.12	0.00	0.00
SULT4A1	0.00	0.00	0.00	0.27	0.00	0.42	0.13	0.12	0.15	0.00	0.00	0.38	0.24	0.19	0.29	0.00	0.00	0.00	0.19	0.00	0.00
ATP13A4	0.00	0.00	0.12	0.32	0.00	0.54	0.13	0.00	0.00	0.00	0.22	0.34	0.15	0.11	0.27	0.00	0.00	0.00	0.10	0.00	0.00
SLC11A2	0.00	0.00	0.00	0.16	0.00	0.13	0.11	0.00	0.11	0.00	0.16	0.18	0.13	0.14	0.20	0.00	0.00	0.00	0.22	0.00	0.00
HEPH	0.00	0.00	0.00	0.20	0.00	0.20	0.12	0.00	0.12	0.00	0.12	0.20	0.12	0.14	0.23	0.00	0.00	0.00	0.25	0.00	0.00
BACE2	0.00	0.00	0.00	0.17	0.00	0.22	0.00	0.18	0.20	0.00	0.17	0.24	0.11	0.14	0.18	0.00	0.00	0.00	0.15	0.00	0.00
ASAH1	0.00	0.00	0.16	0.26	0.00	0.26	0.17	0.26	0.25	0.00	0.00	0.17	0.00	0.00	0.18	0.10	0.00	0.00	0.22	0.00	0.00
IFRD1	0.00	0.00	0.12	0.29	0.00	0.34	0.18	0.32	0.17	0.00	0.00	0.20	0.00	0.00	0.29	0.10	0.00	0.00	0.16	0.00	0.00
MTMR7	0.00	0.00	0.12	0.36	0.00	0.58	0.16	0.17	0.00	0.00	0.18	0.32	0.00	0.00	0.28	0.10	0.00	0.00	0.17	0.00	0.00
C18ORF8	0.00	0.00	0.00	0.21	0.00	0.32	0.12	0.13	0.12	0.00	0.22	0.15	0.00	0.00	0.19	0.14	0.00	0.00	0.16	0.00	0.00
GBA2	0.00	0.00	0.00	0.20	0.00	0.25	0.12	0.16	0.20	0.00	0.15	0.18	0.00	0.00	0.18	0.11	0.00	0.00	0.18	0.00	0.00
CTSA	0.00	0.00	0.00	0.19	0.00	0.24	0.11	0.10	0.19	0.00	0.11	0.17	0.00	0.00	0.22	0.12	0.00	0.00	0.11	0.00	0.00
B3GNT5	0.00	0.00	0.00	0.23	0.00	0.34	0.13	0.19	0.13	0.00	0.15	0.22	0.00	0.00	0.24	0.16	0.00	0.00	0.12	0.00	0.00
SUMF1	0.00	0.00	0.00	0.24	0.00	0.32	0.14	0.20	0.23	0.00	0.11	0.18	0.00	0.00	0.24	0.15	0.00	0.00	0.15	0.00	0.00
SMPD1	0.00	0.00	0.00	0.16	0.00	0.12	0.12	0.14	0.25	0.00	0.00	0.16	0.12	0.00	0.20	0.19	0.00	0.00	0.19	0.00	0.00
DHCR7	0.00	0.00	0.00	0.18	0.00	0.20	0.11	0.15	0.18	0.00	0.00	0.20	0.13	0.00	0.21	0.13	0.00	0.00	0.33	0.00	0.00

QKI	0.00	0.00	0.00	0.18	0.00	0.17	0.11	0.17	0.11	0.00	0.00	0.20	0.16	0.00	0.34	0.32	0.00	0.00	0.15	0.00	0.00
PSAP	0.00	0.00	0.00	0.19	0.00	0.15	0.14	0.15	0.21	0.00	0.00	0.19	0.13	0.00	0.29	0.25	0.00	0.00	0.12	0.00	0.00
CERCAM	0.00	0.00	0.00	0.25	0.00	0.38	0.14	0.12	0.00	0.00	0.26	0.28	0.11	0.00	0.31	0.15	0.00	0.00	0.14	0.00	0.00
ABCG4	0.00	0.00	0.00	0.26	0.00	0.33	0.15	0.16	0.00	0.00	0.15	0.25	0.12	0.00	0.20	0.13	0.00	0.00	0.22	0.00	0.00
GAL3ST1	0.00	0.00	0.00	0.20	0.00	0.27	0.11	0.12	0.00	0.00	0.19	0.23	0.11	0.00	0.38	0.34	0.00	0.00	0.18	0.00	0.00
UGT8	0.00	0.00	0.00	0.15	0.00	0.19	0.00	0.12	0.16	0.00	0.16	0.25	0.15	0.00	0.43	0.42	0.00	0.00	0.14	0.00	0.00
ALDOC	0.00	0.00	0.00	0.23	0.00	0.28	0.13	0.18	0.00	0.00	0.00	0.33	0.31	0.24	0.34	0.22	0.00	0.00	0.17	0.00	0.00
GUSB	0.00	0.00	0.00	0.19	0.00	0.12	0.15	0.00	0.23	0.00	0.00	0.17	0.13	0.10	0.21	0.16	0.00	0.00	0.16	0.00	0.00
MAN2B1	0.00	0.00	0.00	0.23	0.00	0.30	0.13	0.00	0.15	0.00	0.00	0.27	0.15	0.13	0.24	0.13	0.00	0.00	0.19	0.00	0.00
TPP1	0.00	0.00	0.00	0.12	0.00	0.21	0.00	0.11	0.23	0.00	0.00	0.22	0.14	0.14	0.25	0.17	0.00	0.00	0.12	0.00	0.00
CP	0.00	0.00	0.00	0.16	0.00	0.16	0.00	0.13	0.17	0.00	0.00	0.20	0.14	0.17	0.22	0.12	0.00	0.00	0.23	0.00	0.00
HMOX2	0.00	0.00	0.00	0.14	0.00	0.13	0.00	0.13	0.19	0.00	0.00	0.21	0.18	0.18	0.23	0.13	0.00	0.00	0.16	0.00	0.00
SRD5A1	0.00	0.00	0.00	0.17	0.00	0.00	0.14	0.18	0.16	0.00	0.00	0.16	0.19	0.16	0.19	0.12	0.00	0.00	0.25	0.00	0.00
SLC18A2	0.00	0.00	0.00	0.15	0.00	0.00	0.11	0.14	0.18	0.00	0.00	0.20	0.26	0.20	0.27	0.15	0.00	0.00	0.13	0.00	0.00
SLC6A13	0.00	0.00	0.00	0.24	0.00	0.29	0.13	0.00	0.00	0.00	0.25	0.40	0.37	0.30	0.41	0.26	0.00	0.00	0.12	0.00	0.00
ZNF259	0.00	0.00	0.19	0.37	0.00	0.39	0.24	0.25	0.16	0.00	0.00	0.11	0.00	0.00	0.21	0.00	0.13	0.00	0.12	0.00	0.00
RBM17	0.00	0.00	0.12	0.31	0.00	0.42	0.18	0.33	0.22	0.00	0.00	0.18	0.00	0.00	0.18	0.00	0.11	0.00	0.12	0.00	0.00
CELSR2	0.00	0.00	0.15	0.26	0.00	0.28	0.18	0.19	0.12	0.00	0.00	0.19	0.00	0.00	0.21	0.00	0.16	0.00	0.15	0.00	0.00
IGHMBP2	0.00	0.00	0.00	0.20	0.00	0.29	0.00	0.19	0.18	0.00	0.00	0.19	0.11	0.00	0.32	0.16	0.10	0.00	0.12	0.00	0.00
PRMT2	0.00	0.00	0.17	0.34	0.00	0.35	0.22	0.33	0.24	0.00	0.00	0.15	0.00	0.00	0.16	0.00	0.00	0.11	0.18	0.00	0.00
RPS6	0.00	0.00	0.10	0.24	0.00	0.30	0.13	0.30	0.17	0.00	0.00	0.12	0.00	0.00	0.15	0.00	0.00	0.19	0.12	0.00	0.00
GLTP	0.00	0.00	0.00	0.26	0.00	0.37	0.12	0.26	0.11	0.00	0.13	0.15	0.00	0.00	0.19	0.00	0.00	0.17	0.21	0.00	0.00
HSPB8	0.00	0.00	0.00	0.21	0.00	0.25	0.11	0.25	0.25	0.00	0.00	0.14	0.00	0.00	0.22	0.12	0.00	0.14	0.11	0.00	0.00
CA2	0.00	0.00	0.00	0.11	0.00	0.00	0.00	0.12	0.16	0.00	0.12	0.14	0.16	0.00	0.14	0.11	0.00	0.11	0.11	0.00	0.00
SLC1A3	0.00	0.00	0.00	0.13	0.00	0.00	0.00	0.12	0.13	0.00	0.00	0.21	0.30	0.30	0.26	0.26	0.00	0.11	0.13	0.00	0.00
SLC1A2	0.00	0.00	0.00	0.13	0.00	0.00	0.00	0.13	0.18	0.00	0.00	0.21	0.26	0.29	0.25	0.24	0.00	0.11	0.13	0.00	0.00
VMP1	0.00	0.00	0.12	0.30	0.00	0.41	0.16	0.45	0.24	0.00	0.12	0.10	0.00	0.00	0.14	0.00	0.00	0.00	0.00	0.11	0.00
TUBA1C	0.00	0.00	0.17	0.34	0.00	0.38	0.21	0.23	0.11	0.00	0.10	0.19	0.00	0.00	0.23	0.00	0.00	0.00	0.00	0.11	0.00
RAB5A	0.00	0.00	0.17	0.24	0.00	0.17	0.19	0.23	0.11	0.00	0.10	0.11	0.00	0.00	0.12	0.00	0.00	0.00	0.00	0.12	0.00
RPS6KA2	0.00	0.00	0.12	0.25	0.00	0.25	0.16	0.27	0.15	0.00	0.00	0.16	0.00	0.15	0.16	0.00	0.00	0.00	0.00	0.12	0.00
REM2	0.00	0.00	0.16	0.31	0.00	0.36	0.18	0.33	0.11	0.00	0.00	0.25	0.00	0.16	0.21	0.00	0.00	0.00	0.00	0.14	0.00
GIGYF1	0.00	0.00	0.34	0.48	0.00	0.34	0.41	0.21	0.00	0.00	0.14	0.22	0.00	0.13	0.27	0.00	0.00	0.00	0.00	0.12	0.00
CACNB3	0.00	0.00	0.15	0.25	0.00	0.21	0.17	0.15	0.00	0.00	0.13	0.22	0.00	0.16	0.23	0.00	0.00	0.00	0.00	0.13	0.00
MAPK8IP2	0.00	0.00	0.22	0.38	0.00	0.36	0.28	0.22	0.00	0.00	0.00	0.24	0.11	0.15	0.26	0.00	0.00	0.00	0.00	0.14	0.00
GJD2	0.00	0.00	0.00	0.19	0.00	0.14	0.12	0.14	0.00	0.00	0.00	0.20	0.24	0.25	0.29	0.21	0.00	0.00	0.00	0.13	0.00
SYN1	0.00	0.00	0.00	0.18	0.00	0.15	0.12	0.18	0.00	0.00	0.00	0.26	0.26	0.33	0.28	0.17	0.00	0.00	0.00	0.10	0.00
GJA10	0.00	0.00	0.00	0.22	0.00	0.27	0.11	0.00	0.00	0.00	0.13	0.22	0.16	0.12	0.26	0.15	0.00	0.00	0.00	0.11	0.00
RIMS2	0.00	0.00	0.11	0.25	0.00	0.29	0.15	0.21	0.00	0.00	0.00	0.22	0.00	0.16	0.22	0.00	0.14	0.00	0.00	0.19	0.00
RAB3B	0.00	0.00	0.00	0.23	0.00	0.29	0.12	0.17	0.00	0.00	0.00	0.24	0.12	0.16	0.19	0.00	0.11	0.00	0.00	0.19	0.00
KCNC2	0.00	0.00	0.00	0.13	0.00	0.19	0.00	0.00	0.00	0.00	0.13	0.25	0.18	0.22	0.30	0.22	0.14	0.00	0.00	0.14	0.00

CCKAR	0.00	0.00	0.23	0.23	0.00	0.00	0.21	0.11	0.10	0.11	0.00	0.11	0.00	0.00	0.20	0.00	0.00	0.19	0.00	0.12	0.00
CFL2	0.00	0.00	0.00	0.25	0.00	0.33	0.13	0.21	0.14	0.00	0.00	0.19	0.12	0.00	0.20	0.00	0.00	0.11	0.00	0.11	0.00
XBP1	0.00	0.00	0.00	0.16	0.00	0.13	0.11	0.24	0.22	0.00	0.10	0.00	0.00	0.00	0.15	0.00	0.18	0.10	0.00	0.18	0.00
BBS12	0.00	0.00	0.11	0.22	0.00	0.32	0.12	0.12	0.00	0.00	0.00	0.15	0.00	0.00	0.19	0.00	0.18	0.13	0.00	0.14	0.00
WFS1	0.00	0.00	0.00	0.15	0.00	0.16	0.00	0.12	0.13	0.00	0.00	0.12	0.00	0.10	0.16	0.00	0.32	0.11	0.00	0.25	0.00
OSBPL9	0.00	0.00	0.19	0.40	0.00	0.47	0.25	0.32	0.14	0.00	0.23	0.13	0.00	0.00	0.00	0.00	0.00	0.00	0.21	0.12	0.00
MIR29A	0.00	0.00	0.11	0.23	0.00	0.26	0.14	0.51	0.33	0.00	0.00	0.14	0.00	0.00	0.13	0.00	0.00	0.00	0.12	0.11	0.00
EIF2D	0.00	0.00	0.15	0.36	0.00	0.47	0.20	0.12	0.00	0.00	0.12	0.21	0.00	0.00	0.18	0.00	0.00	0.00	0.13	0.13	0.00
PPAPDC2	0.00	0.00	0.15	0.35	0.00	0.53	0.18	0.29	0.00	0.00	0.12	0.19	0.00	0.00	0.11	0.00	0.00	0.00	0.30	0.12	0.00
LRP10	0.00	0.00	0.28	0.48	0.00	0.47	0.35	0.25	0.00	0.00	0.22	0.26	0.00	0.00	0.20	0.00	0.00	0.00	0.17	0.11	0.00
NAGPA	0.00	0.00	0.17	0.33	0.00	0.34	0.23	0.12	0.00	0.00	0.20	0.19	0.00	0.00	0.16	0.00	0.00	0.00	0.19	0.13	0.00
EEA1	0.00	0.00	0.18	0.31	0.00	0.27	0.22	0.22	0.00	0.00	0.13	0.18	0.00	0.00	0.20	0.00	0.00	0.00	0.13	0.13	0.00
MTMR4	0.00	0.00	0.15	0.36	0.00	0.50	0.20	0.25	0.00	0.00	0.12	0.17	0.00	0.00	0.10	0.00	0.00	0.00	0.15	0.10	0.00
PMM1	0.00	0.00	0.00	0.23	0.00	0.35	0.10	0.18	0.19	0.00	0.11	0.26	0.00	0.00	0.23	0.00	0.00	0.00	0.25	0.12	0.00
ATP6V0A1	0.00	0.00	0.00	0.27	0.00	0.33	0.14	0.15	0.00	0.00	0.19	0.27	0.13	0.00	0.22	0.00	0.00	0.00	0.15	0.13	0.00
GM2A	0.00	0.00	0.00	0.21	0.00	0.24	0.13	0.13	0.15	0.00	0.00	0.18	0.00	0.00	0.21	0.12	0.00	0.00	0.17	0.12	0.00
APOO	0.00	0.00	0.12	0.25	0.00	0.28	0.16	0.16	0.14	0.00	0.00	0.13	0.00	0.00	0.00	0.00	0.22	0.00	0.29	0.16	0.00
SEC61A2	0.00	0.00	0.16	0.31	0.00	0.37	0.19	0.21	0.00	0.00	0.21	0.12	0.00	0.00	0.00	0.00	0.22	0.00	0.14	0.27	0.00
BP9	0.00	0.00	0.23	0.33	0.00	0.19	0.30	0.12	0.00	0.00	0.15	0.12	0.00	0.00	0.00	0.00	0.23	0.00	0.14	0.18	0.00
SAA2	0.00	0.00	0.16	0.26	0.00	0.22	0.18	0.13	0.15	0.00	0.00	0.00	0.00	0.00	0.12	0.00	0.12	0.00	0.14	0.11	0.00
OLR1	0.00	0.00	0.13	0.20	0.00	0.12	0.17	0.16	0.19	0.00	0.00	0.00	0.00	0.00	0.11	0.00	0.17	0.00	0.11	0.12	0.00
SLC2A4RG	0.00	0.00	0.10	0.30	0.00	0.42	0.16	0.33	0.00	0.00	0.00	0.14	0.00	0.00	0.17	0.00	0.13	0.00	0.12	0.21	0.00
HEXDC	0.00	0.00	0.00	0.24	0.00	0.33	0.13	0.24	0.23	0.00	0.00	0.13	0.00	0.00	0.11	0.00	0.26	0.00	0.17	0.16	0.00
CNDP2	0.00	0.00	0.15	0.29	0.00	0.29	0.20	0.00	0.00	0.00	0.00	0.17	0.00	0.10	0.16	0.00	0.29	0.00	0.11	0.14	0.00
DDIT4	0.00	0.00	0.00	0.19	0.00	0.21	0.12	0.43	0.28	0.00	0.00	0.13	0.00	0.00	0.17	0.00	0.00	0.22	0.17	0.18	0.00
LIPA	0.00	0.00	0.00	0.20	0.00	0.23	0.12	0.15	0.20	0.00	0.00	0.18	0.00	0.00	0.16	0.00	0.00	0.11	0.34	0.10	0.00
EIF2AK4	0.00	0.00	0.00	0.20	0.00	0.23	0.12	0.30	0.21	0.00	0.00	0.14	0.00	0.00	0.16	0.00	0.00	0.14	0.17	0.16	0.00
LGTN	0.00	0.00	0.19	0.37	0.00	0.40	0.25	0.00	0.00	0.00	0.25	0.26	0.00	0.00	0.23	0.00	0.00	0.11	0.13	0.13	0.00
PROP1	0.00	0.00	0.00	0.12	0.00	0.00	0.00	0.18	0.13	0.00	0.00	0.11	0.11	0.11	0.16	0.00	0.00	0.11	0.13	0.13	0.00
MIR33A	0.00	0.00	0.00	0.22	0.00	0.26	0.14	0.33	0.11	0.00	0.00	0.11	0.00	0.00	0.00	0.00	0.14	0.14	0.40	0.17	0.00
IAPP	0.00	0.00	0.10	0.15	0.00	0.11	0.00	0.13	0.18	0.00	0.00	0.00	0.00	0.00	0.15	0.00	0.27	0.23	0.13	0.25	0.00
GHR	0.00	0.00	0.10	0.17	0.00	0.00	0.14	0.11	0.12	0.00	0.00	0.00	0.00	0.00	0.12	0.00	0.12	0.11	0.11	0.12	0.00
ASIP	0.00	0.00	0.10	0.14	0.00	0.00	0.12	0.13	0.11	0.00	0.00	0.00	0.00	0.00	0.13	0.00	0.16	0.19	0.14	0.15	0.00
NR1H2	0.00	0.00	0.10	0.16	0.00	0.00	0.14	0.20	0.18	0.00	0.00	0.00	0.00	0.00	0.16	0.00	0.12	0.14	0.20	0.15	0.00
INSIG2	0.00	0.00	0.00	0.15	0.00	0.16	0.10	0.20	0.12	0.00	0.00	0.00	0.00	0.00	0.11	0.00	0.24	0.16	0.24	0.18	0.00
G6PC	0.00	0.00	0.00	0.16	0.00	0.15	0.10	0.15	0.15	0.00	0.00	0.00	0.00	0.00	0.12	0.00	0.26	0.23	0.32	0.36	0.00
PRKAB2	0.00	0.00	0.00	0.21	0.00	0.27	0.11	0.19	0.00	0.00	0.00	0.18	0.00	0.00	0.18	0.00	0.19	0.39	0.26	0.31	0.00
FBP1	0.00	0.00	0.00	0.17	0.00	0.25	0.00	0.17	0.15	0.00	0.00	0.18	0.00	0.00	0.12	0.00	0.26	0.24	0.33	0.35	0.00
DMPK	0.00	0.00	0.00	0.14	0.00	0.18	0.00	0.11	0.16	0.00	0.00	0.12	0.00	0.00	0.16	0.00	0.10	0.10	0.11	0.10	0.00
EIF2B2	0.00	0.00	0.00	0.18	0.00	0.29	0.00	0.15	0.11	0.00	0.00	0.18	0.00	0.00	0.21	0.00	0.16	0.10	0.11	0.16	0.00

NHLRC1	0.00	0.00	0.00	0.18	0.00	0.31	0.00	0.24	0.20	0.00	0.00	0.21	0.00	0.00	0.21	0.00	0.12	0.17	0.20	0.22	0.00
ALMS1	0.00	0.00	0.00	0.18	0.00	0.29	0.00	0.16	0.15	0.00	0.00	0.11	0.00	0.00	0.16	0.00	0.29	0.18	0.20	0.27	0.00
GYS1	0.00	0.00	0.00	0.15	0.00	0.19	0.00	0.16	0.15	0.00	0.00	0.14	0.00	0.00	0.14	0.00	0.27	0.26	0.30	0.38	0.00
SLC19A2	0.00	0.00	0.00	0.18	0.00	0.28	0.00	0.14	0.00	0.00	0.18	0.15	0.00	0.00	0.21	0.00	0.13	0.10	0.26	0.16	0.00
CLOCK	0.00	0.00	0.00	0.12	0.00	0.00	0.00	0.14	0.11	0.00	0.00	0.11	0.11	0.00	0.16	0.00	0.12	0.12	0.17	0.13	0.00
ATP10A	0.00	0.00	0.00	0.19	0.00	0.28	0.00	0.00	0.00	0.00	0.10	0.26	0.00	0.12	0.20	0.00	0.19	0.16	0.17	0.18	0.00
SLC2A1	0.00	0.00	0.00	0.00	0.00	0.00	0.00	0.11	0.00	0.00	0.11	0.13	0.13	0.00	0.13	0.12	0.13	0.17	0.15	0.28	0.00
HAX1	0.00	0.00	0.14	0.30	0.00	0.32	0.19	0.28	0.20	0.00	0.15	0.19	0.00	0.00	0.21	0.00	0.00	0.00	0.00	0.00	0.19
DPH5	0.00	0.00	0.21	0.38	0.00	0.41	0.25	0.24	0.16	0.00	0.10	0.20	0.00	0.00	0.27	0.00	0.00	0.00	0.00	0.00	0.14
DNAJA1	0.00	0.00	0.12	0.27	0.00	0.34	0.16	0.20	0.13	0.00	0.11	0.17	0.00	0.00	0.19	0.00	0.00	0.00	0.00	0.00	0.19
PLEKHF1	0.00	0.00	0.17	0.36	0.00	0.42	0.23	0.30	0.13	0.00	0.11	0.11	0.00	0.00	0.11	0.00	0.00	0.00	0.00	0.00	0.24
WIPI2	0.00	0.00	0.20	0.40	0.00	0.49	0.24	0.31	0.11	0.00	0.21	0.10	0.00	0.00	0.12	0.00	0.00	0.00	0.00	0.00	0.11
TUBA1B	0.00	0.00	0.12	0.27	0.00	0.33	0.15	0.26	0.13	0.00	0.00	0.20	0.11	0.00	0.21	0.00	0.00	0.00	0.00	0.00	0.15
BAG1	0.00	0.00	0.14	0.25	0.00	0.20	0.19	0.28	0.27	0.00	0.00	0.15	0.00	0.12	0.18	0.00	0.00	0.00	0.00	0.00	0.11
BLOC1S2	0.00	0.00	0.13	0.37	0.00	0.53	0.19	0.25	0.00	0.00	0.00	0.28	0.12	0.12	0.28	0.00	0.00	0.00	0.00	0.00	0.12
SEP-04	0.00	0.00	0.00	0.23	0.00	0.35	0.10	0.27	0.17	0.00	0.00	0.28	0.18	0.15	0.26	0.00	0.00	0.00	0.00	0.00	0.20
DYNC1I1	0.00	0.00	0.00	0.24	0.00	0.35	0.11	0.18	0.00	0.00	0.00	0.28	0.19	0.12	0.31	0.12	0.00	0.00	0.00	0.00	0.17
KLC1	0.00	0.00	0.00	0.23	0.00	0.29	0.13	0.17	0.00	0.00	0.00	0.28	0.19	0.17	0.29	0.11	0.00	0.00	0.00	0.00	0.13
KIF5A	0.00	0.00	0.00	0.22	0.00	0.27	0.13	0.11	0.00	0.00	0.00	0.26	0.17	0.11	0.32	0.15	0.00	0.00	0.00	0.00	0.13
TPGS1	0.00	0.00	0.00	0.30	0.00	0.45	0.15	0.14	0.00	0.00	0.00	0.41	0.20	0.19	0.37	0.15	0.00	0.00	0.00	0.00	0.13
BHLHB9	0.00	0.00	0.00	0.18	0.00	0.35	0.00	0.31	0.23	0.00	0.00	0.23	0.11	0.14	0.21	0.13	0.00	0.00	0.00	0.00	0.18
DYNC1H1	0.00	0.00	0.00	0.19	0.00	0.26	0.00	0.13	0.12	0.00	0.00	0.24	0.16	0.10	0.31	0.15	0.00	0.00	0.00	0.00	0.13
LTN1	0.00	0.00	0.00	0.26	0.00	0.44	0.00	0.23	0.10	0.00	0.00	0.27	0.13	0.13	0.35	0.19	0.00	0.00	0.00	0.00	0.14
SH3GLB1	0.00	0.00	0.14	0.31	0.00	0.33	0.20	0.29	0.14	0.00	0.00	0.17	0.00	0.00	0.16	0.00	0.00	0.12	0.00	0.00	0.22
TUBB	0.00	0.00	0.11	0.24	0.00	0.26	0.13	0.19	0.00	0.00	0.00	0.19	0.10	0.00	0.19	0.00	0.00	0.12	0.00	0.00	0.18
RHOT1	0.00	0.00	0.11	0.27	0.00	0.38	0.13	0.21	0.00	0.00	0.00	0.24	0.12	0.00	0.21	0.00	0.00	0.18	0.00	0.00	0.38
FDPS	0.00	0.00	0.11	0.23	0.00	0.27	0.15	0.27	0.18	0.00	0.00	0.18	0.00	0.00	0.16	0.00	0.00	0.00	0.35	0.00	0.15
COX2	0.00	0.00	0.11	0.18	0.00	0.14	0.13	0.22	0.23	0.00	0.00	0.10	0.00	0.00	0.11	0.00	0.00	0.00	0.16	0.00	0.19
CYP27A1	0.00	0.00	0.12	0.22	0.00	0.18	0.16	0.18	0.19	0.00	0.00	0.14	0.00	0.00	0.16	0.00	0.00	0.00	0.37	0.00	0.11
STARD3NL	0.00	0.00	0.10	0.29	0.00	0.43	0.15	0.18	0.00	0.00	0.21	0.18	0.00	0.00	0.12	0.00	0.00	0.00	0.26	0.00	0.15
PEX12	0.00	0.00	0.12	0.29	0.00	0.39	0.16	0.12	0.00	0.00	0.16	0.16	0.00	0.00	0.20	0.00	0.00	0.00	0.19	0.00	0.21
MSMO1	0.00	0.00	0.12	0.33	0.00	0.52	0.15	0.16	0.00	0.00	0.14	0.26	0.00	0.00	0.23	0.00	0.00	0.00	0.34	0.00	0.13
VPS35	0.00	0.00	0.19	0.40	0.00	0.45	0.26	0.22	0.00	0.00	0.21	0.23	0.00	0.00	0.24	0.00	0.00	0.00	0.11	0.00	0.14
PXMP2	0.00	0.00	0.12	0.37	0.00	0.57	0.17	0.13	0.00	0.00	0.20	0.27	0.00	0.00	0.28	0.00	0.00	0.00	0.23	0.00	0.22
SPG20	0.00	0.00	0.13	0.32	0.00	0.44	0.18	0.24	0.00	0.00	0.12	0.20	0.00	0.00	0.24	0.00	0.00	0.00	0.12	0.00	0.17
PEX16	0.00	0.00	0.13	0.33	0.00	0.46	0.17	0.14	0.00	0.00	0.19	0.15	0.00	0.00	0.20	0.00	0.00	0.00	0.13	0.00	0.23
MTCH1	0.00	0.00	0.13	0.36	0.00	0.53	0.18	0.13	0.00	0.00	0.26	0.25	0.00	0.00	0.16	0.00	0.00	0.00	0.12	0.00	0.45
C2ORF18	0.00	0.00	0.00	0.35	0.00	0.57	0.16	0.32	0.19	0.00	0.21	0.21	0.00	0.00	0.16	0.00	0.00	0.00	0.13	0.00	0.25
C11ORF31	0.00	0.00	0.00	0.30	0.00	0.48	0.13	0.30	0.13	0.00	0.00	0.22	0.10	0.00	0.19	0.00	0.00	0.00	0.16	0.00	0.31
PRDX6	0.00	0.00	0.00	0.18	0.00	0.18	0.10	0.17	0.19	0.00	0.00	0.15	0.13	0.00	0.13	0.00	0.00	0.00	0.16	0.00	0.13

PRDX5	0.00	0.00	0.00	0.22	0.00	0.31	0.11	0.24	0.18	0.00	0.00	0.17	0.11	0.00	0.18	0.00	0.00	0.00	0.19	0.00	0.26
SAT1	0.00	0.00	0.00	0.18	0.00	0.19	0.10	0.28	0.26	0.00	0.00	0.17	0.16	0.00	0.16	0.00	0.00	0.00	0.24	0.00	0.11
TTLL4	0.00	0.00	0.00	0.29	0.00	0.49	0.11	0.14	0.00	0.00	0.00	0.34	0.17	0.10	0.26	0.00	0.00	0.00	0.11	0.00	0.15
PRDX2	0.00	0.00	0.00	0.16	0.00	0.17	0.00	0.20	0.24	0.00	0.00	0.14	0.13	0.10	0.15	0.00	0.00	0.00	0.13	0.00	0.14
HTRA2	0.00	0.00	0.00	0.17	0.00	0.20	0.00	0.20	0.26	0.00	0.00	0.19	0.12	0.13	0.19	0.00	0.00	0.00	0.11	0.00	0.29
ALDH16A1	0.00	0.00	0.00	0.28	0.00	0.54	0.00	0.14	0.00	0.00	0.11	0.29	0.13	0.00	0.25	0.12	0.00	0.00	0.18	0.00	0.18
SZT2	0.00	0.00	0.00	0.26	0.00	0.48	0.00	0.13	0.00	0.00	0.00	0.33	0.21	0.21	0.28	0.14	0.00	0.00	0.15	0.00	0.24
MAOB	0.00	0.00	0.00	0.12	0.00	0.00	0.00	0.11	0.13	0.00	0.00	0.18	0.22	0.17	0.19	0.11	0.00	0.00	0.17	0.00	0.11
ALDH7A1	0.00	0.00	0.00	0.20	0.00	0.29	0.00	0.16	0.17	0.00	0.00	0.21	0.12	0.00	0.18	0.00	0.11	0.00	0.31	0.00	0.15
TMEM14A	0.00	0.00	0.00	0.26	0.00	0.39	0.12	0.27	0.15	0.00	0.17	0.13	0.00	0.00	0.00	0.00	0.00	0.14	0.11	0.00	0.58
ATG10	0.00	0.00	0.24	0.36	0.00	0.33	0.26	0.28	0.00	0.00	0.13	0.00	0.00	0.00	0.13	0.00	0.00	0.17	0.16	0.00	0.28
SLIRP	0.00	0.00	0.14	0.38	0.00	0.50	0.22	0.36	0.00	0.00	0.00	0.17	0.00	0.00	0.16	0.00	0.00	0.21	0.25	0.00	0.42
GABARAPL2	0.00	0.00	0.16	0.31	0.00	0.34	0.20	0.22	0.00	0.00	0.00	0.16	0.00	0.00	0.21	0.00	0.00	0.11	0.11	0.00	0.15
TAZ	0.00	0.00	0.00	0.23	0.00	0.33	0.12	0.25	0.17	0.00	0.00	0.17	0.00	0.00	0.17	0.00	0.00	0.16	0.21	0.00	0.22
PPA2	0.00	0.00	0.00	0.29	0.00	0.47	0.12	0.22	0.12	0.00	0.00	0.22	0.00	0.00	0.12	0.00	0.00	0.13	0.24	0.00	0.37
DNM1L	0.00	0.00	0.00	0.23	0.00	0.29	0.12	0.25	0.17	0.00	0.00	0.19	0.00	0.00	0.18	0.00	0.00	0.17	0.14	0.00	0.41
TPI1	0.00	0.00	0.00	0.19	0.00	0.23	0.10	0.17	0.16	0.00	0.00	0.18	0.00	0.00	0.18	0.00	0.00	0.15	0.19	0.00	0.13
MAP1LC3B	0.00	0.00	0.00	0.22	0.00	0.26	0.14	0.27	0.12	0.00	0.00	0.15	0.00	0.00	0.20	0.00	0.00	0.12	0.13	0.00	0.18
NDUFS5	0.00	0.00	0.00	0.25	0.00	0.39	0.12	0.17	0.14	0.00	0.00	0.13	0.00	0.00	0.15	0.00	0.00	0.13	0.14	0.00	0.33
MAR-05	0.00	0.00	0.00	0.26	0.00	0.37	0.13	0.27	0.13	0.00	0.00	0.12	0.00	0.00	0.13	0.00	0.00	0.15	0.13	0.00	0.46
MAP1LC3A	0.00	0.00	0.00	0.19	0.00	0.20	0.12	0.27	0.17	0.00	0.00	0.11	0.00	0.00	0.17	0.00	0.00	0.14	0.14	0.00	0.19
TIMM23	0.00	0.00	0.00	0.32	0.00	0.50	0.14	0.16	0.00	0.00	0.15	0.23	0.00	0.00	0.21	0.00	0.00	0.18	0.20	0.00	0.54
FXC1	0.00	0.00	0.00	0.31	0.00	0.58	0.10	0.10	0.00	0.00	0.19	0.20	0.00	0.00	0.18	0.00	0.00	0.15	0.17	0.00	0.49
PEX6	0.00	0.00	0.00	0.26	0.00	0.41	0.12	0.10	0.00	0.00	0.11	0.13	0.00	0.00	0.17	0.00	0.00	0.12	0.18	0.00	0.22
STARD6	0.00	0.00	0.00	0.30	0.00	0.51	0.13	0.14	0.00	0.00	0.16	0.21	0.00	0.00	0.14	0.00	0.00	0.15	0.23	0.00	0.27
TIMM10	0.00	0.00	0.00	0.31	0.00	0.52	0.13	0.16	0.00	0.00	0.18	0.19	0.00	0.00	0.21	0.00	0.00	0.21	0.15	0.00	0.46
ACOT13	0.00	0.00	0.00	0.31	0.00	0.49	0.15	0.23	0.00	0.00	0.00	0.31	0.18	0.00	0.23	0.00	0.00	0.17	0.33	0.00	0.18
GLRX2	0.00	0.00	0.00	0.14	0.00	0.24	0.00	0.18	0.19	0.00	0.00	0.16	0.10	0.00	0.16	0.00	0.00	0.10	0.18	0.00	0.27
PINK1	0.00	0.00	0.00	0.13	0.00	0.12	0.00	0.14	0.20	0.00	0.00	0.16	0.12	0.00	0.16	0.00	0.00	0.13	0.13	0.00	0.24
NDUFS4	0.00	0.00	0.00	0.20	0.00	0.31	0.00	0.13	0.12	0.00	0.00	0.23	0.12	0.00	0.20	0.00	0.00	0.23	0.28	0.00	0.45
MRPL20	0.00	0.00	0.00	0.30	0.00	0.53	0.00	0.20	0.00	0.00	0.00	0.25	0.15	0.16	0.21	0.00	0.00	0.15	0.16	0.00	0.36
IMMT	0.00	0.00	0.00	0.23	0.00	0.46	0.00	0.20	0.00	0.00	0.00	0.28	0.16	0.14	0.21	0.00	0.00	0.23	0.25	0.00	0.52
GLUD2	0.00	0.00	0.00	0.23	0.00	0.33	0.00	0.13	0.00	0.00	0.00	0.34	0.18	0.14	0.26	0.00	0.00	0.24	0.32	0.00	0.25
C22ORF25	0.00	0.00	0.00	0.29	0.00	0.55	0.00	0.13	0.00	0.00	0.00	0.34	0.13	0.11	0.25	0.00	0.00	0.10	0.14	0.00	0.30
COX5A	0.00	0.00	0.00	0.22	0.00	0.41	0.00	0.13	0.00	0.00	0.00	0.27	0.12	0.13	0.22	0.00	0.00	0.15	0.22	0.00	0.43
PANK2	0.00	0.00	0.00	0.12	0.00	0.22	0.00	0.00	0.17	0.00	0.00	0.23	0.12	0.00	0.22	0.11	0.00	0.13	0.26	0.00	0.21
UQCRQ	0.00	0.00	0.00	0.21	0.00	0.41	0.00	0.00	0.00	0.00	0.00	0.35	0.22	0.21	0.25	0.12	0.00	0.18	0.26	0.00	0.43
NDUFB11	0.00	0.00	0.00	0.26	0.00	0.53	0.00	0.00	0.00	0.00	0.00	0.39	0.18	0.17	0.38	0.17	0.00	0.15	0.16	0.00	0.37
GCSH	0.00	0.00	0.00	0.21	0.00	0.37	0.00	0.00	0.00	0.00	0.00	0.34	0.19	0.12	0.29	0.15	0.00	0.13	0.32	0.00	0.23
MTERFD1	0.00	0.00	0.00	0.17	0.00	0.33	0.00	0.30	0.12	0.00	0.00	0.11	0.00	0.00	0.13	0.00	0.12	0.21	0.25	0.00	0.43

ATG5	0.00	0.00	0.00	0.17	0.00	0.16	0.12	0.26	0.19	0.00	0.00	0.00	0.00	0.00	0.15	0.00	0.00	0.17	0.16	0.13	0.18
COX15	0.00	0.00	0.00	0.25	0.00	0.45	0.00	0.16	0.15	0.00	0.00	0.27	0.00	0.00	0.19	0.00	0.00	0.18	0.27	0.10	0.41
GPX7	0.00	0.00	0.00	0.17	0.00	0.24	0.00	0.21	0.14	0.00	0.00	0.18	0.00	0.00	0.13	0.00	0.00	0.12	0.21	0.10	0.15
DGUOK	0.00	0.00	0.00	0.17	0.00	0.31	0.00	0.16	0.20	0.00	0.00	0.19	0.00	0.00	0.16	0.00	0.00	0.14	0.31	0.11	0.27
PYCR1	0.00	0.00	0.00	0.14	0.00	0.28	0.00	0.10	0.12	0.00	0.00	0.19	0.00	0.00	0.13	0.00	0.00	0.12	0.38	0.11	0.20
FH	0.00	0.00	0.00	0.14	0.00	0.22	0.00	0.22	0.19	0.00	0.00	0.15	0.00	0.00	0.14	0.00	0.00	0.20	0.28	0.12	0.26
SC5DL	0.00	0.00	0.00	0.20	0.00	0.36	0.00	0.16	0.00	0.00	0.00	0.25	0.11	0.00	0.18	0.00	0.00	0.13	0.41	0.11	0.13
L2HGDH	0.00	0.00	0.00	0.12	0.00	0.24	0.00	0.00	0.13	0.00	0.00	0.26	0.12	0.00	0.18	0.00	0.00	0.14	0.32	0.11	0.25
OTC	0.00	0.00	0.00	0.12	0.00	0.12	0.00	0.00	0.13	0.00	0.00	0.18	0.13	0.00	0.15	0.00	0.00	0.16	0.24	0.10	0.14
ADSL	0.00	0.00	0.00	0.00	0.00	0.18	0.00	0.12	0.21	0.00	0.00	0.23	0.14	0.00	0.16	0.00	0.00	0.23	0.38	0.14	0.16
GBE1	0.00	0.00	0.00	0.11	0.00	0.21	0.00	0.00	0.19	0.00	0.00	0.20	0.00	0.00	0.24	0.13	0.00	0.14	0.27	0.16	0.13
ACAD11	0.00	0.00	0.00	0.21	0.00	0.34	0.00	0.00	0.00	0.00	0.00	0.36	0.18	0.00	0.30	0.18	0.00	0.26	0.38	0.12	0.23
PDHA1	0.00	0.00	0.00	0.15	0.00	0.20	0.00	0.13	0.00	0.00	0.00	0.16	0.00	0.00	0.11	0.00	0.11	0.29	0.30	0.22	0.23
CRAT	0.00	0.00	0.00	0.16	0.00	0.31	0.00	0.12	0.00	0.00	0.00	0.20	0.00	0.00	0.11	0.00	0.12	0.36	0.43	0.18	0.32
SPTLC3	0.00	0.00	0.00	0.24	0.00	0.41	0.00	0.15	0.00	0.00	0.00	0.22	0.00	0.00	0.19	0.00	0.17	0.11	0.35	0.15	0.11
BCKDHA	0.00	0.00	0.00	0.18	0.00	0.28	0.00	0.14	0.00	0.00	0.00	0.16	0.00	0.00	0.12	0.00	0.11	0.25	0.32	0.18	0.20
LARS2	0.00	0.00	0.00	0.21	0.00	0.42	0.00	0.16	0.00	0.00	0.00	0.17	0.00	0.00	0.15	0.00	0.19	0.17	0.20	0.11	0.34
COX4I1	0.00	0.00	0.00	0.20	0.00	0.33	0.00	0.12	0.00	0.00	0.00	0.23	0.00	0.00	0.20	0.00	0.14	0.30	0.35	0.18	0.45
ACAD9	0.00	0.00	0.00	0.19	0.00	0.33	0.00	0.12	0.00	0.00	0.00	0.25	0.00	0.00	0.19	0.00	0.12	0.34	0.44	0.16	0.38
AUH	0.00	0.00	0.00	0.23	0.00	0.43	0.00	0.14	0.00	0.00	0.00	0.19	0.00	0.00	0.16	0.00	0.16	0.13	0.30	0.11	0.16
ATP8	0.00	0.00	0.00	0.14	0.00	0.25	0.00	0.00	0.14	0.00	0.00	0.15	0.00	0.00	0.12	0.00	0.19	0.23	0.26	0.11	0.41
ACADS	0.00	0.00	0.00	0.15	0.00	0.18	0.00	0.00	0.10	0.00	0.00	0.15	0.00	0.00	0.11	0.00	0.16	0.30	0.44	0.20	0.28
CPT2	0.00	0.00	0.00	0.11	0.00	0.17	0.00	0.00	0.12	0.00	0.00	0.10	0.00	0.00	0.11	0.00	0.12	0.29	0.34	0.15	0.26
SLC25A13	0.00	0.00	0.00	0.14	0.00	0.22	0.00	0.00	0.13	0.00	0.00	0.19	0.00	0.00	0.15	0.00	0.10	0.19	0.31	0.15	0.21
CISD2	0.00	0.00	0.00	0.20	0.00	0.36	0.00	0.00	0.00	0.00	0.12	0.13	0.00	0.00	0.16	0.00	0.22	0.11	0.12	0.13	0.21
GUCA1B	0.10	0.10	0.17	0.24	0.00	0.22	0.15	0.12	0.00	0.00	0.00	0.11	0.00	0.00	0.17	0.00	0.00	0.00	0.00	0.00	0.00
FRAT2	0.00	0.00	0.11	0.25	0.00	0.31	0.16	0.28	0.10	0.15	0.00	0.13	0.00	0.00	0.13	0.00	0.00	0.00	0.00	0.00	0.00
DRD5P2	0.18	0.18	0.30	0.38	0.00	0.31	0.28	0.00	0.00	0.00	0.12	0.17	0.00	0.00	0.17	0.00	0.00	0.00	0.00	0.00	0.00
OR5G1P	0.11	0.11	0.28	0.47	0.00	0.50	0.31	0.00	0.00	0.00	0.21	0.19	0.00	0.00	0.22	0.00	0.00	0.00	0.00	0.00	0.00
RAB34	0.00	0.00	0.17	0.35	0.00	0.42	0.21	0.31	0.13	0.00	0.12	0.12	0.00	0.00	0.11	0.00	0.00	0.00	0.00	0.00	0.00
WIPI1	0.00	0.00	0.19	0.40	0.00	0.50	0.24	0.39	0.11	0.00	0.13	0.11	0.00	0.00	0.13	0.00	0.00	0.00	0.00	0.00	0.00
ADAM15	0.00	0.00	0.12	0.21	0.00	0.21	0.14	0.19	0.23	0.00	0.20	0.13	0.00	0.00	0.13	0.00	0.00	0.00	0.00	0.00	0.00
PITPNM3	0.00	0.00	0.26	0.44	0.00	0.48	0.28	0.20	0.13	0.00	0.19	0.20	0.00	0.00	0.21	0.00	0.00	0.00	0.00	0.00	0.00
SLURP1	0.00	0.00	0.23	0.39	0.00	0.31	0.30	0.17	0.20	0.00	0.20	0.13	0.00	0.00	0.23	0.00	0.00	0.00	0.00	0.00	0.00
OTSC2	0.00	0.00	0.12	0.27	0.00	0.35	0.17	0.17	0.14	0.00	0.11	0.11	0.00	0.00	0.16	0.00	0.00	0.00	0.00	0.00	0.00
VPS33A	0.00	0.00	0.13	0.30	0.00	0.35	0.18	0.23	0.13	0.00	0.11	0.17	0.00	0.00	0.20	0.00	0.00	0.00	0.00	0.00	0.00
FAM134B	0.00	0.00	0.12	0.30	0.00	0.43	0.16	0.25	0.20	0.00	0.11	0.21	0.00	0.00	0.27	0.00	0.00	0.00	0.00	0.00	0.00
OTOP1	0.00	0.00	0.22	0.35	0.00	0.29	0.26	0.20	0.14	0.00	0.18	0.15	0.00	0.00	0.25	0.00	0.00	0.00	0.00	0.00	0.00
ANKS1B	0.00	0.00	0.20	0.37	0.00	0.38	0.26	0.31	0.20	0.00	0.14	0.18	0.00	0.00	0.23	0.00	0.00	0.00	0.00	0.00	0.00
BRWD3	0.00	0.00	0.10	0.31	0.00	0.49	0.15	0.29	0.12	0.00	0.10	0.17	0.00	0.00	0.16	0.00	0.00	0.00	0.00	0.00	0.00

CLDN16	0.00	0.00	0.11	0.22	0.00	0.25	0.13	0.17	0.19	0.00	0.20	0.12	0.00	0.00	0.15	0.00	0.00	0.00	0.00	0.00	0.00
CHMP3	0.00	0.00	0.19	0.35	0.00	0.38	0.23	0.31	0.10	0.00	0.15	0.12	0.00	0.00	0.18	0.00	0.00	0.00	0.00	0.00	0.00
PKD2L1	0.00	0.00	0.22	0.33	0.00	0.29	0.24	0.12	0.11	0.00	0.24	0.19	0.00	0.00	0.21	0.00	0.00	0.00	0.00	0.00	0.00
MCOLN3	0.00	0.00	0.15	0.25	0.00	0.21	0.19	0.15	0.11	0.00	0.18	0.15	0.00	0.00	0.21	0.00	0.00	0.00	0.00	0.00	0.00
ITM2A	0.00	0.00	0.12	0.32	0.00	0.40	0.18	0.21	0.13	0.00	0.16	0.20	0.00	0.00	0.20	0.00	0.00	0.00	0.00	0.00	0.00
MPZL1	0.00	0.00	0.21	0.39	0.00	0.40	0.28	0.20	0.10	0.00	0.28	0.18	0.00	0.00	0.23	0.00	0.00	0.00	0.00	0.00	0.00
ADAM10	0.00	0.00	0.12	0.16	0.00	0.10	0.13	0.20	0.25	0.00	0.21	0.12	0.00	0.00	0.14	0.00	0.00	0.00	0.00	0.00	0.00
PSMD2	0.00	0.00	0.13	0.31	0.00	0.42	0.17	0.31	0.10	0.00	0.10	0.18	0.00	0.00	0.20	0.00	0.00	0.00	0.00	0.00	0.00
UFL1	0.00	0.00	0.13	0.33	0.00	0.46	0.18	0.39	0.18	0.00	0.13	0.19	0.00	0.00	0.19	0.00	0.00	0.00	0.00	0.00	0.00
CD33	0.00	0.00	0.20	0.35	0.00	0.25	0.29	0.24	0.22	0.00	0.27	0.12	0.00	0.00	0.16	0.00	0.00	0.00	0.00	0.00	0.00
ADAM9	0.00	0.00	0.12	0.20	0.00	0.18	0.14	0.19	0.25	0.00	0.19	0.11	0.00	0.00	0.11	0.00	0.00	0.00	0.00	0.00	0.00
BICC1	0.00	0.00	0.13	0.24	0.00	0.25	0.17	0.17	0.18	0.00	0.15	0.10	0.00	0.00	0.15	0.00	0.00	0.00	0.00	0.00	0.00
KIAA0196	0.00	0.00	0.12	0.32	0.00	0.48	0.16	0.19	0.11	0.00	0.13	0.18	0.00	0.00	0.20	0.00	0.00	0.00	0.00	0.00	0.00
SIRPB1	0.00	0.00	0.33	0.52	0.00	0.38	0.43	0.24	0.11	0.00	0.31	0.17	0.00	0.00	0.22	0.00	0.00	0.00	0.00	0.00	0.00
LAMP1	0.00	0.00	0.13	0.23	0.00	0.17	0.18	0.18	0.14	0.00	0.17	0.11	0.00	0.00	0.16	0.00	0.00	0.00	0.00	0.00	0.00
PKD1L3	0.00	0.00	0.30	0.41	0.00	0.31	0.32	0.18	0.13	0.00	0.24	0.16	0.00	0.00	0.25	0.00	0.00	0.00	0.00	0.00	0.00
MEP1B	0.00	0.00	0.11	0.27	0.00	0.31	0.17	0.13	0.13	0.00	0.20	0.16	0.00	0.00	0.16	0.00	0.00	0.00	0.00	0.00	0.00
SYVN1	0.00	0.00	0.11	0.25	0.00	0.29	0.15	0.29	0.22	0.00	0.17	0.15	0.00	0.00	0.17	0.00	0.00	0.00	0.00	0.00	0.00
NOMO2	0.00	0.00	0.14	0.27	0.00	0.32	0.18	0.23	0.14	0.00	0.14	0.13	0.00	0.00	0.21	0.00	0.00	0.00	0.00	0.00	0.00
NOMO1	0.00	0.00	0.14	0.26	0.00	0.31	0.17	0.17	0.11	0.00	0.11	0.12	0.00	0.00	0.20	0.00	0.00	0.00	0.00	0.00	0.00
DNAJB9	0.00	0.00	0.13	0.26	0.00	0.33	0.15	0.27	0.21	0.00	0.20	0.12	0.00	0.00	0.12	0.00	0.00	0.00	0.00	0.00	0.00
HERPUD1	0.00	0.00	0.12	0.26	0.00	0.31	0.15	0.29	0.18	0.00	0.18	0.14	0.00	0.00	0.17	0.00	0.00	0.00	0.00	0.00	0.00
PSMB5	0.00	0.00	0.11	0.27	0.00	0.33	0.16	0.27	0.24	0.00	0.11	0.15	0.00	0.00	0.18	0.00	0.00	0.00	0.00	0.00	0.00
SPPL2B	0.00	0.00	0.14	0.26	0.00	0.31	0.17	0.14	0.11	0.00	0.31	0.14	0.00	0.00	0.12	0.00	0.00	0.00	0.00	0.00	0.00
COLQ	0.00	0.00	0.11	0.23	0.00	0.23	0.15	0.13	0.10	0.00	0.13	0.11	0.00	0.00	0.20	0.00	0.00	0.00	0.00	0.00	0.00
STAB1	0.00	0.00	0.30	0.43	0.00	0.27	0.38	0.21	0.22	0.00	0.23	0.12	0.00	0.00	0.18	0.00	0.00	0.00	0.00	0.00	0.00
GOLIM4	0.00	0.00	0.16	0.35	0.00	0.43	0.20	0.26	0.10	0.00	0.24	0.11	0.00	0.00	0.14	0.00	0.00	0.00	0.00	0.00	0.00
IL1RAPL2	0.00	0.00	0.32	0.47	0.00	0.36	0.38	0.13	0.13	0.00	0.21	0.23	0.00	0.00	0.24	0.00	0.00	0.00	0.00	0.00	0.00
TMEM100	0.00	0.00	0.16	0.28	0.00	0.29	0.20	0.16	0.10	0.00	0.20	0.14	0.00	0.00	0.19	0.00	0.00	0.00	0.00	0.00	0.00
DNAJB6	0.00	0.00	0.11	0.30	0.00	0.43	0.14	0.32	0.14	0.00	0.13	0.16	0.00	0.00	0.20	0.00	0.00	0.00	0.00	0.00	0.00
LRP1B	0.00	0.00	0.21	0.38	0.00	0.35	0.28	0.29	0.19	0.00	0.20	0.19	0.00	0.00	0.19	0.00	0.00	0.00	0.00	0.00	0.00
SLC35C1	0.00	0.00	0.12	0.28	0.00	0.38	0.16	0.19	0.17	0.00	0.22	0.18	0.00	0.00	0.20	0.00	0.00	0.00	0.00	0.00	0.00
NSMAF	0.00	0.00	0.21	0.36	0.00	0.32	0.26	0.25	0.18	0.00	0.13	0.16	0.00	0.00	0.18	0.00	0.00	0.00	0.00	0.00	0.00
TMEM108	0.00	0.00	0.24	0.39	0.00	0.38	0.30	0.20	0.10	0.00	0.18	0.16	0.00	0.00	0.20	0.00	0.00	0.00	0.00	0.00	0.00
PIGA	0.00	0.00	0.10	0.25	0.00	0.26	0.16	0.20	0.15	0.00	0.20	0.11	0.00	0.00	0.20	0.00	0.00	0.00	0.00	0.00	0.00
TM9SF2	0.00	0.00	0.25	0.46	0.00	0.52	0.30	0.23	0.10	0.00	0.25	0.19	0.00	0.00	0.20	0.00	0.00	0.00	0.00	0.00	0.00
DNAJB2	0.00	0.00	0.16	0.33	0.00	0.41	0.20	0.27	0.20	0.00	0.10	0.23	0.00	0.00	0.23	0.00	0.00	0.00	0.00	0.00	0.00
APPBP2	0.00	0.00	0.15	0.36	0.00	0.47	0.20	0.37	0.13	0.00	0.14	0.23	0.00	0.00	0.21	0.00	0.00	0.00	0.00	0.00	0.00
CHMP5	0.00	0.00	0.18	0.35	0.00	0.41	0.23	0.32	0.15	0.00	0.13	0.12	0.00	0.00	0.14	0.00	0.00	0.00	0.00	0.00	0.00
CREB3L2	0.00	0.00	0.11	0.30	0.00	0.44	0.14	0.37	0.20	0.00	0.13	0.16	0.00	0.00	0.18	0.00	0.00	0.00	0.00	0.00	0.00

MUSK	0.00	0.00	0.17	0.29	0.00	0.18	0.25	0.19	0.11	0.00	0.18	0.12	0.00	0.00	0.25	0.00	0.00	0.00	0.00	0.00	0.00
MCFD2	0.00	0.00	0.17	0.35	0.00	0.41	0.22	0.20	0.12	0.00	0.20	0.17	0.00	0.00	0.27	0.00	0.00	0.00	0.00	0.00	0.00
MCOLN2	0.00	0.00	0.20	0.38	0.00	0.42	0.24	0.21	0.13	0.00	0.26	0.21	0.00	0.00	0.24	0.00	0.00	0.00	0.00	0.00	0.00
DERL1	0.00	0.00	0.13	0.27	0.00	0.31	0.16	0.24	0.16	0.00	0.25	0.11	0.00	0.00	0.15	0.00	0.00	0.00	0.00	0.00	0.00
KRTCAP2	0.00	0.00	0.12	0.30	0.00	0.44	0.15	0.21	0.12	0.00	0.24	0.17	0.00	0.00	0.12	0.00	0.00	0.00	0.00	0.00	0.00
HGS	0.00	0.00	0.22	0.34	0.00	0.24	0.27	0.26	0.12	0.00	0.13	0.10	0.00	0.00	0.14	0.00	0.00	0.00	0.00	0.00	0.00
SH3PXD2A	0.00	0.00	0.12	0.28	0.00	0.33	0.17	0.25	0.17	0.00	0.13	0.21	0.00	0.00	0.21	0.00	0.00	0.00	0.00	0.00	0.00
EFHD2	0.00	0.00	0.21	0.41	0.00	0.46	0.27	0.26	0.11	0.00	0.16	0.16	0.00	0.00	0.17	0.00	0.00	0.00	0.00	0.00	0.00
NCLN	0.00	0.00	0.14	0.30	0.00	0.34	0.20	0.30	0.10	0.00	0.15	0.13	0.00	0.00	0.18	0.00	0.00	0.00	0.00	0.00	0.00
ANO1	0.00	0.00	0.14	0.26	0.00	0.24	0.16	0.16	0.19	0.00	0.23	0.17	0.00	0.00	0.21	0.00	0.00	0.00	0.00	0.00	0.00
UBXN4	0.00	0.00	0.11	0.28	0.00	0.46	0.13	0.22	0.11	0.00	0.23	0.17	0.00	0.00	0.15	0.00	0.00	0.00	0.00	0.00	0.00
NOMO3	0.00	0.00	0.14	0.25	0.00	0.25	0.18	0.18	0.13	0.00	0.11	0.11	0.00	0.00	0.20	0.00	0.00	0.00	0.00	0.00	0.00
IL1R2	0.00	0.00	0.21	0.31	0.00	0.16	0.28	0.15	0.21	0.00	0.13	0.12	0.00	0.00	0.17	0.00	0.00	0.00	0.00	0.00	0.00
RNF41	0.00	0.00	0.21	0.38	0.00	0.32	0.29	0.38	0.27	0.00	0.11	0.13	0.00	0.00	0.18	0.00	0.00	0.00	0.00	0.00	0.00
CCDC50	0.00	0.00	0.27	0.48	0.00	0.47	0.35	0.34	0.13	0.00	0.14	0.13	0.00	0.00	0.19	0.00	0.00	0.00	0.00	0.00	0.00
LY6E	0.00	0.00	0.16	0.35	0.00	0.40	0.23	0.20	0.19	0.00	0.20	0.17	0.00	0.00	0.19	0.00	0.00	0.00	0.00	0.00	0.00
CRB2	0.00	0.00	0.15	0.33	0.00	0.42	0.20	0.22	0.11	0.00	0.21	0.16	0.00	0.00	0.18	0.00	0.00	0.00	0.00	0.00	0.00
RSPO4	0.00	0.00	0.19	0.32	0.00	0.36	0.22	0.22	0.00	0.12	0.17	0.12	0.00	0.00	0.20	0.00	0.00	0.00	0.00	0.00	0.00
NAA15	0.00	0.00	0.15	0.34	0.00	0.41	0.20	0.33	0.19	0.00	0.00	0.25	0.13	0.00	0.26	0.00	0.00	0.00	0.00	0.00	0.00
SIAH1	0.00	0.00	0.11	0.22	0.00	0.24	0.14	0.38	0.20	0.00	0.00	0.15	0.10	0.00	0.20	0.00	0.00	0.00	0.00	0.00	0.00
DNM2	0.00	0.00	0.15	0.23	0.00	0.17	0.17	0.14	0.12	0.00	0.00	0.17	0.11	0.00	0.19	0.00	0.00	0.00	0.00	0.00	0.00
MIR124-1	0.00	0.00	0.10	0.25	0.00	0.33	0.14	0.54	0.32	0.00	0.00	0.20	0.10	0.00	0.19	0.00	0.00	0.00	0.00	0.00	0.00
ARHGAP18	0.00	0.00	0.12	0.26	0.00	0.36	0.14	0.29	0.20	0.00	0.00	0.17	0.13	0.00	0.20	0.00	0.00	0.00	0.00	0.00	0.00
MYO1B	0.00	0.00	0.12	0.27	0.00	0.34	0.15	0.15	0.00	0.00	0.13	0.23	0.13	0.00	0.21	0.00	0.00	0.00	0.00	0.00	0.00
KIF3B	0.00	0.00	0.11	0.27	0.00	0.35	0.14	0.17	0.00	0.00	0.11	0.20	0.11	0.00	0.23	0.00	0.00	0.00	0.00	0.00	0.00
RDX	0.00	0.00	0.11	0.17	0.00	0.17	0.10	0.16	0.00	0.00	0.10	0.11	0.13	0.00	0.15	0.00	0.00	0.00	0.00	0.00	0.00
ATAT1	0.00	0.00	0.29	0.49	0.00	0.50	0.33	0.30	0.00	0.00	0.12	0.23	0.12	0.00	0.32	0.00	0.00	0.00	0.00	0.00	0.00
IFT52	0.00	0.00	0.18	0.36	0.00	0.51	0.18	0.14	0.00	0.00	0.16	0.30	0.12	0.00	0.29	0.00	0.00	0.00	0.00	0.00	0.00
NUCB1	0.00	0.00	0.20	0.38	0.00	0.45	0.21	0.28	0.00	0.00	0.21	0.24	0.11	0.00	0.21	0.00	0.00	0.00	0.00	0.00	0.00
TRAPPC9	0.00	0.00	0.15	0.36	0.00	0.47	0.20	0.22	0.00	0.00	0.11	0.26	0.11	0.00	0.28	0.00	0.00	0.00	0.00	0.00	0.00
SSNA1	0.00	0.00	0.12	0.38	0.00	0.61	0.17	0.21	0.00	0.00	0.13	0.24	0.13	0.00	0.30	0.00	0.00	0.00	0.00	0.00	0.00
FSD1L	0.00	0.00	0.13	0.34	0.00	0.52	0.16	0.14	0.00	0.00	0.19	0.26	0.10	0.00	0.18	0.00	0.00	0.00	0.00	0.00	0.00
MYO6	0.00	0.00	0.10	0.21	0.00	0.19	0.14	0.23	0.00	0.00	0.11	0.13	0.11	0.00	0.19	0.00	0.00	0.00	0.00	0.00	0.00
FOXRED2	0.00	0.00	0.00	0.28	0.00	0.44	0.13	0.26	0.14	0.00	0.21	0.19	0.10	0.00	0.16	0.00	0.00	0.00	0.00	0.00	0.00
MIR488	0.00	0.00	0.22	0.35	0.00	0.27	0.28	0.48	0.28	0.00	0.00	0.12	0.00	0.11	0.13	0.00	0.00	0.00	0.00	0.00	0.00
ANP32A	0.00	0.00	0.19	0.32	0.00	0.31	0.22	0.41	0.21	0.00	0.00	0.21	0.00	0.10	0.23	0.00	0.00	0.00	0.00	0.00	0.00
LOC100422561	0.00	0.00	0.21	0.32	0.00	0.30	0.24	0.14	0.22	0.00	0.00	0.25	0.00	0.10	0.23	0.00	0.00	0.00	0.00	0.00	0.00
SPRED1	0.00	0.00	0.19	0.33	0.00	0.31	0.24	0.29	0.20	0.00	0.00	0.17	0.00	0.11	0.22	0.00	0.00	0.00	0.00	0.00	0.00
CACNA2D3	0.00	0.00	0.14	0.28	0.00	0.36	0.16	0.17	0.00	0.00	0.16	0.28	0.00	0.12	0.23	0.00	0.00	0.00	0.00	0.00	0.00
PATE2	0.00	0.00	0.15	0.32	0.00	0.43	0.16	0.13	0.00	0.00	0.20	0.26	0.00	0.14	0.24	0.00	0.00	0.00	0.00	0.00	0.00

CACNB1	0.00	0.00	0.13	0.23	0.00	0.20	0.15	0.11	0.00	0.00	0.12	0.21	0.00	0.13	0.21	0.00	0.00	0.00	0.00	0.00	0.00
TSPEAR	0.00	0.00	0.20	0.40	0.00	0.52	0.24	0.10	0.00	0.00	0.26	0.22	0.00	0.11	0.27	0.00	0.00	0.00	0.00	0.00	0.00
ANO2	0.00	0.00	0.18	0.35	0.00	0.40	0.20	0.13	0.00	0.00	0.27	0.28	0.00	0.11	0.27	0.00	0.00	0.00	0.00	0.00	0.00
CACNB2	0.00	0.00	0.00	0.18	0.00	0.18	0.11	0.13	0.10	0.00	0.12	0.19	0.00	0.14	0.17	0.00	0.00	0.00	0.00	0.00	0.00
TNIK	0.00	0.00	0.19	0.38	0.00	0.44	0.24	0.36	0.00	0.00	0.00	0.22	0.12	0.13	0.23	0.00	0.00	0.00	0.00	0.00	0.00
SYNJ1	0.00	0.00	0.15	0.31	0.00	0.30	0.21	0.13	0.00	0.00	0.00	0.33	0.18	0.18	0.26	0.00	0.00	0.00	0.00	0.00	0.00
DENND1A	0.00	0.00	0.16	0.32	0.00	0.38	0.21	0.14	0.00	0.00	0.00	0.23	0.10	0.10	0.21	0.00	0.00	0.00	0.00	0.00	0.00
TSNAXIP1	0.00	0.00	0.11	0.32	0.00	0.52	0.14	0.10	0.00	0.00	0.00	0.23	0.15	0.12	0.18	0.00	0.00	0.00	0.00	0.00	0.00
IQSEC2	0.00	0.00	0.20	0.37	0.00	0.41	0.24	0.18	0.00	0.00	0.00	0.32	0.17	0.23	0.28	0.00	0.00	0.00	0.00	0.00	0.00
MOB2	0.00	0.00	0.17	0.45	0.00	0.69	0.23	0.36	0.00	0.00	0.00	0.34	0.15	0.13	0.29	0.00	0.00	0.00	0.00	0.00	0.00
RIMS3	0.00	0.00	0.12	0.30	0.00	0.39	0.17	0.20	0.00	0.00	0.00	0.29	0.11	0.14	0.26	0.00	0.00	0.00	0.00	0.00	0.00
PAK3	0.00	0.00	0.15	0.24	0.00	0.23	0.16	0.17	0.00	0.00	0.00	0.23	0.19	0.18	0.24	0.00	0.00	0.00	0.00	0.00	0.00
PPP1R10	0.00	0.00	0.13	0.33	0.00	0.46	0.18	0.34	0.00	0.00	0.00	0.23	0.12	0.13	0.22	0.00	0.00	0.00	0.00	0.00	0.00
ARL6IP6	0.00	0.00	0.15	0.35	0.00	0.51	0.18	0.20	0.00	0.00	0.00	0.36	0.19	0.21	0.30	0.00	0.00	0.00	0.00	0.00	0.00
ZCCHC12	0.00	0.00	0.11	0.33	0.00	0.50	0.18	0.30	0.00	0.00	0.00	0.25	0.19	0.19	0.26	0.00	0.00	0.00	0.00	0.00	0.00
PJA2	0.00	0.00	0.12	0.35	0.00	0.56	0.16	0.29	0.00	0.00	0.00	0.32	0.21	0.20	0.28	0.00	0.00	0.00	0.00	0.00	0.00
SH3GL3	0.00	0.00	0.22	0.38	0.00	0.37	0.27	0.18	0.00	0.00	0.00	0.29	0.14	0.10	0.25	0.00	0.00	0.00	0.00	0.00	0.00
PDLIM5	0.00	0.00	0.15	0.31	0.00	0.38	0.19	0.21	0.00	0.00	0.00	0.30	0.20	0.19	0.28	0.00	0.00	0.00	0.00	0.00	0.00
PACSIN1	0.00	0.00	0.16	0.34	0.00	0.36	0.22	0.14	0.00	0.00	0.00	0.31	0.16	0.11	0.26	0.00	0.00	0.00	0.00	0.00	0.00
AMPH	0.00	0.00	0.15	0.29	0.00	0.27	0.20	0.15	0.00	0.00	0.00	0.28	0.14	0.16	0.23	0.00	0.00	0.00	0.00	0.00	0.00
NT3	0.00	0.00	0.13	0.24	0.00	0.24	0.18	0.17	0.00	0.00	0.00	0.13	0.11	0.12	0.20	0.00	0.00	0.00	0.00	0.00	0.00
GDI1	0.00	0.00	0.10	0.24	0.00	0.32	0.12	0.18	0.00	0.00	0.00	0.24	0.12	0.15	0.19	0.00	0.00	0.00	0.00	0.00	0.00
SLC6A2	0.00	0.00	0.10	0.15	0.00	0.00	0.12	0.15	0.17	0.00	0.00	0.13	0.16	0.14	0.21	0.00	0.00	0.00	0.00	0.00	0.00
YWHAZ	0.00	0.00	0.12	0.15	0.00	0.00	0.11	0.15	0.11	0.00	0.00	0.14	0.11	0.13	0.11	0.00	0.00	0.00	0.00	0.00	0.00
USP46	0.00	0.00	0.00	0.27	0.00	0.37	0.14	0.28	0.11	0.00	0.00	0.19	0.14	0.14	0.25	0.00	0.00	0.00	0.00	0.00	0.00
MIR107	0.00	0.00	0.00	0.21	0.00	0.29	0.11	0.56	0.37	0.00	0.00	0.19	0.12	0.11	0.16	0.00	0.00	0.00	0.00	0.00	0.00
TTBK1	0.00	0.00	0.00	0.20	0.00	0.23	0.13	0.11	0.13	0.00	0.00	0.26	0.17	0.22	0.19	0.00	0.00	0.00	0.00	0.00	0.00
SNCAIP	0.00	0.00	0.00	0.19	0.00	0.23	0.11	0.17	0.17	0.00	0.00	0.22	0.13	0.13	0.23	0.00	0.00	0.00	0.00	0.00	0.00
PCM1	0.00	0.00	0.00	0.22	0.00	0.30	0.11	0.22	0.18	0.00	0.00	0.25	0.20	0.11	0.23	0.00	0.00	0.00	0.00	0.00	0.00
MEF2D	0.00	0.00	0.00	0.18	0.00	0.19	0.12	0.31	0.13	0.00	0.00	0.18	0.14	0.16	0.21	0.00	0.00	0.00	0.00	0.00	0.00
EML1	0.00	0.00	0.20	0.36	0.00	0.45	0.22	0.00	0.00	0.00	0.13	0.28	0.12	0.10	0.28	0.00	0.00	0.00	0.00	0.00	0.00
CACNG5	0.00	0.00	0.25	0.39	0.00	0.31	0.30	0.00	0.00	0.00	0.21	0.28	0.12	0.17	0.25	0.00	0.00	0.00	0.00	0.00	0.00
GABRG3	0.00	0.00	0.21	0.32	0.00	0.18	0.29	0.00	0.00	0.00	0.12	0.24	0.20	0.21	0.24	0.00	0.00	0.00	0.00	0.00	0.00
FXYD6	0.00	0.00	0.12	0.27	0.00	0.36	0.15	0.00	0.00	0.00	0.16	0.33	0.23	0.20	0.29	0.00	0.00	0.00	0.00	0.00	0.00
FCHO1	0.00	0.00	0.26	0.43	0.00	0.37	0.32	0.00	0.00	0.00	0.18	0.25	0.12	0.12	0.22	0.00	0.00	0.00	0.00	0.00	0.00
SCZD13	0.00	0.00	0.28	0.37	0.00	0.14	0.37	0.00	0.00	0.00	0.13	0.18	0.17	0.15	0.22	0.00	0.00	0.00	0.00	0.00	0.00
CALHM2	0.00	0.00	0.13	0.20	0.00	0.18	0.14	0.00	0.00	0.00	0.15	0.26	0.15	0.24	0.15	0.00	0.00	0.00	0.00	0.00	0.00
FEB-05	0.00	0.00	0.14	0.27	0.00	0.31	0.18	0.00	0.00	0.00	0.15	0.19	0.11	0.15	0.14	0.00	0.00	0.00	0.00	0.00	0.00
CALHM3	0.00	0.00	0.13	0.20	0.00	0.18	0.14	0.00	0.00	0.00	0.15	0.26	0.15	0.24	0.15	0.00	0.00	0.00	0.00	0.00	0.00
KCNJ13	0.00	0.00	0.10	0.24	0.00	0.31	0.12	0.00	0.00	0.00	0.20	0.25	0.14	0.11	0.24	0.00	0.00	0.00	0.00	0.00	0.00

NKAIN2	0.00	0.00	0.00	0.30	0.00	0.49	0.13	0.16	0.00	0.00	0.17	0.31	0.13	0.10	0.30	0.00	0.00	0.00	0.00	0.00	0.00
OSMR	0.00	0.00	0.21	0.34	0.00	0.20	0.29	0.27	0.25	0.00	0.14	0.00	0.00	0.00	0.21	0.14	0.00	0.00	0.00	0.00	0.00
TYROBP	0.00	0.00	0.21	0.30	0.00	0.13	0.28	0.20	0.20	0.00	0.21	0.00	0.00	0.00	0.19	0.10	0.00	0.00	0.00	0.00	0.00
TRPM8	0.00	0.00	0.17	0.23	0.00	0.12	0.19	0.20	0.22	0.00	0.16	0.00	0.00	0.00	0.22	0.11	0.00	0.00	0.00	0.00	0.00
TRAV24	0.00	0.00	0.11	0.26	0.00	0.29	0.17	0.25	0.20	0.00	0.00	0.15	0.00	0.00	0.16	0.11	0.00	0.00	0.00	0.00	0.00
ULK4	0.00	0.00	0.13	0.23	0.00	0.28	0.16	0.18	0.16	0.00	0.00	0.18	0.00	0.00	0.26	0.11	0.00	0.00	0.00	0.00	0.00
SGK196	0.00	0.00	0.13	0.23	0.00	0.28	0.16	0.18	0.16	0.00	0.00	0.18	0.00	0.00	0.26	0.11	0.00	0.00	0.00	0.00	0.00
HSPA12B	0.00	0.00	0.17	0.36	0.00	0.47	0.21	0.20	0.14	0.00	0.00	0.22	0.00	0.00	0.22	0.11	0.00	0.00	0.00	0.00	0.00
IFNA21	0.00	0.00	0.14	0.31	0.00	0.38	0.19	0.19	0.14	0.00	0.00	0.19	0.00	0.00	0.22	0.12	0.00	0.00	0.00	0.00	0.00
KCNK7	0.00	0.00	0.13	0.31	0.00	0.42	0.17	0.11	0.00	0.00	0.23	0.29	0.00	0.00	0.33	0.15	0.00	0.00	0.00	0.00	0.00
VANGL2	0.00	0.00	0.11	0.18	0.00	0.17	0.12	0.19	0.00	0.00	0.12	0.13	0.00	0.00	0.27	0.18	0.00	0.00	0.00	0.00	0.00
PIEZO2	0.00	0.00	0.11	0.25	0.00	0.31	0.14	0.21	0.00	0.00	0.22	0.17	0.00	0.00	0.26	0.11	0.00	0.00	0.00	0.00	0.00
SSR4	0.00	0.00	0.15	0.36	0.00	0.46	0.21	0.14	0.00	0.00	0.24	0.23	0.00	0.00	0.27	0.11	0.00	0.00	0.00	0.00	0.00
MPP6	0.00	0.00	0.22	0.37	0.00	0.42	0.24	0.15	0.00	0.00	0.28	0.25	0.00	0.00	0.28	0.10	0.00	0.00	0.00	0.00	0.00
IGF2R	0.00	0.00	0.15	0.20	0.00	0.00	0.18	0.15	0.16	0.00	0.13	0.11	0.00	0.00	0.17	0.10	0.00	0.00	0.00	0.00	0.00
UTRN	0.00	0.00	0.00	0.15	0.00	0.13	0.11	0.13	0.20	0.00	0.12	0.14	0.00	0.00	0.21	0.11	0.00	0.00	0.00	0.00	0.00
CCDC125	0.00	0.00	0.00	0.23	0.00	0.39	0.10	0.13	0.13	0.00	0.13	0.20	0.00	0.00	0.22	0.13	0.00	0.00	0.00	0.00	0.00
EPB41L3	0.00	0.00	0.00	0.21	0.00	0.25	0.12	0.29	0.20	0.00	0.16	0.21	0.00	0.00	0.28	0.16	0.00	0.00	0.00	0.00	0.00
MARVELD2	0.00	0.00	0.00	0.23	0.00	0.35	0.11	0.14	0.11	0.00	0.22	0.14	0.00	0.00	0.21	0.13	0.00	0.00	0.00	0.00	0.00
ISL2	0.00	0.00	0.12	0.20	0.00	0.11	0.17	0.23	0.00	0.00	0.00	0.12	0.17	0.00	0.38	0.26	0.00	0.00	0.00	0.00	0.00
PHOX2A	0.00	0.00	0.10	0.19	0.00	0.19	0.13	0.25	0.00	0.00	0.00	0.16	0.15	0.00	0.36	0.15	0.00	0.00	0.00	0.00	0.00
SOX14	0.00	0.00	0.12	0.28	0.00	0.38	0.16	0.29	0.00	0.00	0.00	0.28	0.17	0.00	0.42	0.21	0.00	0.00	0.00	0.00	0.00
NCKAP1	0.00	0.00	0.14	0.33	0.00	0.41	0.19	0.21	0.00	0.00	0.00	0.25	0.16	0.00	0.28	0.12	0.00	0.00	0.00	0.00	0.00
PRPH	0.00	0.00	0.11	0.19	0.00	0.13	0.14	0.17	0.00	0.00	0.00	0.14	0.12	0.00	0.38	0.24	0.00	0.00	0.00	0.00	0.00
RNF6	0.00	0.00	0.13	0.36	0.00	0.53	0.19	0.39	0.00	0.00	0.00	0.26	0.12	0.00	0.28	0.12	0.00	0.00	0.00	0.00	0.00
FGD4	0.00	0.00	0.11	0.25	0.00	0.35	0.13	0.14	0.00	0.00	0.00	0.16	0.10	0.00	0.26	0.14	0.00	0.00	0.00	0.00	0.00
RASL10A	0.00	0.00	0.00	0.26	0.00	0.41	0.12	0.32	0.13	0.00	0.00	0.28	0.11	0.00	0.28	0.10	0.00	0.00	0.00	0.00	0.00
CCM2	0.00	0.00	0.00	0.20	0.00	0.25	0.12	0.17	0.17	0.00	0.00	0.23	0.12	0.00	0.32	0.16	0.00	0.00	0.00	0.00	0.00
NF2	0.00	0.00	0.00	0.17	0.00	0.15	0.11	0.22	0.19	0.00	0.00	0.16	0.11	0.00	0.26	0.15	0.00	0.00	0.00	0.00	0.00
TLX3	0.00	0.00	0.00	0.19	0.00	0.22	0.12	0.32	0.16	0.00	0.00	0.16	0.14	0.00	0.38	0.21	0.00	0.00	0.00	0.00	0.00
CIC	0.00	0.00	0.00	0.26	0.00	0.36	0.14	0.28	0.24	0.00	0.00	0.25	0.11	0.00	0.27	0.14	0.00	0.00	0.00	0.00	0.00
LAMA4	0.00	0.00	0.00	0.18	0.00	0.17	0.13	0.15	0.15	0.00	0.00	0.17	0.11	0.00	0.25	0.11	0.00	0.00	0.00	0.00	0.00
ATN1	0.00	0.00	0.00	0.18	0.00	0.21	0.11	0.10	0.18	0.00	0.00	0.21	0.13	0.00	0.24	0.11	0.00	0.00	0.00	0.00	0.00
MNX1	0.00	0.00	0.00	0.20	0.00	0.17	0.15	0.25	0.15	0.00	0.00	0.13	0.13	0.00	0.38	0.26	0.00	0.00	0.00	0.00	0.00
GPR137B	0.00	0.00	0.12	0.35	0.00	0.56	0.16	0.00	0.00	0.00	0.20	0.34	0.11	0.00	0.35	0.16	0.00	0.00	0.00	0.00	0.00
PLLP	0.00	0.00	0.17	0.39	0.00	0.54	0.21	0.00	0.00	0.00	0.26	0.34	0.15	0.00	0.42	0.26	0.00	0.00	0.00	0.00	0.00
SLC18B1	0.00	0.00	0.12	0.35	0.00	0.58	0.14	0.00	0.00	0.00	0.30	0.33	0.12	0.00	0.34	0.11	0.00	0.00	0.00	0.00	0.00
CDH19	0.00	0.00	0.10	0.30	0.00	0.51	0.12	0.00	0.00	0.00	0.19	0.31	0.11	0.00	0.42	0.20	0.00	0.00	0.00	0.00	0.00
OR2B6	0.00	0.00	0.20	0.31	0.00	0.27	0.22	0.00	0.00	0.00	0.15	0.22	0.12	0.00	0.32	0.14	0.00	0.00	0.00	0.00	0.00
MATN4	0.00	0.00	0.00	0.27	0.00	0.43	0.12	0.10	0.00	0.00	0.14	0.25	0.11	0.00	0.31	0.12	0.00	0.00	0.00	0.00	0.00

ANO3	0.00	0.00	0.00	0.31	0.00	0.56	0.12	0.16	0.00	0.00	0.22	0.33	0.11	0.00	0.36	0.18	0.00	0.00	0.00	0.00	0.00
SHISA4	0.00	0.00	0.00	0.31	0.00	0.52	0.14	0.20	0.00	0.00	0.21	0.27	0.14	0.00	0.32	0.32	0.00	0.00	0.00	0.00	0.00
DST	0.00	0.00	0.00	0.18	0.00	0.21	0.10	0.13	0.00	0.00	0.13	0.15	0.14	0.00	0.30	0.15	0.00	0.00	0.00	0.00	0.00
P2RX3	0.00	0.00	0.19	0.24	0.00	0.00	0.21	0.16	0.14	0.00	0.11	0.00	0.00	0.10	0.20	0.11	0.00	0.00	0.00	0.00	0.00
IQCJ	0.00	0.00	0.00	0.29	0.00	0.52	0.12	0.15	0.00	0.00	0.19	0.32	0.00	0.11	0.43	0.22	0.00	0.00	0.00	0.00	0.00
IQCJ-SCHIP1	0.00	0.00	0.00	0.29	0.00	0.52	0.12	0.15	0.00	0.00	0.19	0.32	0.00	0.11	0.43	0.22	0.00	0.00	0.00	0.00	0.00
TMC1	0.00	0.00	0.00	0.18	0.00	0.21	0.11	0.11	0.00	0.00	0.16	0.15	0.00	0.12	0.23	0.10	0.00	0.00	0.00	0.00	0.00
SCN4B	0.00	0.00	0.00	0.15	0.00	0.18	0.00	0.10	0.12	0.00	0.18	0.23	0.00	0.14	0.26	0.13	0.00	0.00	0.00	0.00	0.00
KCNQ4	0.00	0.00	0.00	0.16	0.00	0.16	0.00	0.14	0.13	0.00	0.15	0.15	0.00	0.12	0.23	0.10	0.00	0.00	0.00	0.00	0.00
KCNMB4	0.00	0.00	0.00	0.17	0.00	0.19	0.00	0.15	0.11	0.00	0.14	0.23	0.00	0.19	0.19	0.11	0.00	0.00	0.00	0.00	0.00
NRP1	0.00	0.00	0.00	0.13	0.00	0.00	0.12	0.14	0.16	0.00	0.12	0.00	0.16	0.11	0.22	0.15	0.00	0.00	0.00	0.00	0.00
DLG4	0.00	0.00	0.13	0.15	0.00	0.00	0.13	0.10	0.00	0.00	0.00	0.16	0.15	0.18	0.17	0.10	0.00	0.00	0.00	0.00	0.00
GABRB3	0.00	0.00	0.15	0.22	0.00	0.00	0.21	0.12	0.00	0.00	0.00	0.19	0.22	0.24	0.22	0.13	0.00	0.00	0.00	0.00	0.00
KCNJ6	0.00	0.00	0.15	0.16	0.00	0.00	0.13	0.12	0.00	0.00	0.00	0.13	0.20	0.17	0.17	0.13	0.00	0.00	0.00	0.00	0.00
SYP	0.00	0.00	0.00	0.15	0.00	0.10	0.11	0.13	0.00	0.00	0.00	0.20	0.22	0.25	0.25	0.16	0.00	0.00	0.00	0.00	0.00
SLC17A7	0.00	0.00	0.00	0.15	0.00	0.12	0.10	0.13	0.00	0.00	0.00	0.26	0.35	0.32	0.36	0.25	0.00	0.00	0.00	0.00	0.00
ZNRF1	0.00	0.00	0.00	0.34	0.00	0.53	0.17	0.30	0.00	0.00	0.00	0.36	0.19	0.19	0.46	0.25	0.00	0.00	0.00	0.00	0.00
KLHL8	0.00	0.00	0.00	0.24	0.00	0.41	0.11	0.19	0.00	0.00	0.00	0.27	0.16	0.13	0.27	0.16	0.00	0.00	0.00	0.00	0.00
SYN2	0.00	0.00	0.00	0.22	0.00	0.16	0.16	0.18	0.00	0.00	0.00	0.27	0.26	0.31	0.30	0.16	0.00	0.00	0.00	0.00	0.00
KIAA1147	0.00	0.00	0.00	0.31	0.00	0.50	0.14	0.10	0.00	0.00	0.00	0.64	0.47	0.49	0.54	0.36	0.00	0.00	0.00	0.00	0.00
C10ORF27	0.00	0.00	0.00	0.29	0.00	0.47	0.12	0.18	0.00	0.00	0.00	0.38	0.29	0.26	0.36	0.18	0.00	0.00	0.00	0.00	0.00
ZSCAN21	0.00	0.00	0.00	0.31	0.00	0.49	0.15	0.30	0.00	0.00	0.00	0.32	0.24	0.19	0.44	0.23	0.00	0.00	0.00	0.00	0.00
POU3F2	0.00	0.00	0.00	0.20	0.00	0.23	0.12	0.30	0.00	0.00	0.00	0.25	0.23	0.16	0.38	0.23	0.00	0.00	0.00	0.00	0.00
HSPBAP1	0.00	0.00	0.00	0.34	0.00	0.64	0.11	0.19	0.00	0.00	0.00	0.38	0.20	0.16	0.30	0.11	0.00	0.00	0.00	0.00	0.00
LMX1A	0.00	0.00	0.00	0.18	0.00	0.21	0.11	0.25	0.00	0.00	0.00	0.28	0.32	0.22	0.45	0.30	0.00	0.00	0.00	0.00	0.00
ATOH1	0.00	0.00	0.00	0.14	0.00	0.11	0.10	0.26	0.00	0.00	0.00	0.14	0.18	0.10	0.35	0.24	0.00	0.00	0.00	0.00	0.00
FOXG1	0.00	0.00	0.00	0.14	0.00	0.11	0.10	0.26	0.00	0.00	0.00	0.20	0.24	0.19	0.31	0.22	0.00	0.00	0.00	0.00	0.00
OCM2	0.00	0.00	0.00	0.24	0.00	0.34	0.11	0.17	0.00	0.00	0.00	0.22	0.13	0.12	0.26	0.11	0.00	0.00	0.00	0.00	0.00
LHX6	0.00	0.00	0.00	0.21	0.00	0.24	0.13	0.28	0.00	0.00	0.00	0.24	0.35	0.25	0.33	0.24	0.00	0.00	0.00	0.00	0.00
DOPEY2	0.00	0.00	0.00	0.27	0.00	0.50	0.10	0.21	0.00	0.00	0.00	0.44	0.33	0.30	0.41	0.19	0.00	0.00	0.00	0.00	0.00
NEUROG1	0.00	0.00	0.00	0.17	0.00	0.16	0.12	0.29	0.00	0.00	0.00	0.21	0.25	0.17	0.41	0.28	0.00	0.00	0.00	0.00	0.00
FBXL20	0.00	0.00	0.00	0.29	0.00	0.39	0.16	0.21	0.00	0.00	0.00	0.27	0.17	0.25	0.29	0.11	0.00	0.00	0.00	0.00	0.00
FOXN4	0.00	0.00	0.00	0.27	0.00	0.38	0.15	0.27	0.00	0.00	0.00	0.34	0.28	0.18	0.51	0.33	0.00	0.00	0.00	0.00	0.00
LHX9	0.00	0.00	0.00	0.20	0.00	0.21	0.14	0.26	0.00	0.00	0.00	0.26	0.32	0.17	0.44	0.32	0.00	0.00	0.00	0.00	0.00
FOXB1	0.00	0.00	0.00	0.22	0.00	0.25	0.14	0.19	0.00	0.00	0.00	0.31	0.27	0.21	0.46	0.30	0.00	0.00	0.00	0.00	0.00
RNF112	0.00	0.00	0.00	0.33	0.00	0.57	0.13	0.23	0.00	0.00	0.00	0.40	0.22	0.18	0.39	0.16	0.00	0.00	0.00	0.00	0.00
MPPED2	0.00	0.00	0.00	0.31	0.00	0.60	0.10	0.14	0.00	0.00	0.00	0.40	0.19	0.15	0.30	0.12	0.00	0.00	0.00	0.00	0.00
DPF1	0.00	0.00	0.00	0.28	0.00	0.49	0.10	0.28	0.00	0.00	0.00	0.29	0.15	0.11	0.40	0.17	0.00	0.00	0.00	0.00	0.00
OTP	0.00	0.00	0.00	0.22	0.00	0.31	0.12	0.19	0.00	0.00	0.00	0.35	0.31	0.19	0.47	0.32	0.00	0.00	0.00	0.00	0.00
ZNF180	0.00	0.00	0.00	0.32	0.00	0.57	0.12	0.14	0.00	0.00	0.00	0.44	0.25	0.22	0.54	0.38	0.00	0.00	0.00	0.00	0.00

JRK	0.00	0.00	0.00	0.27	0.00	0.46	0.11	0.15	0.00	0.00	0.00	0.30	0.17	0.20	0.30	0.12	0.00	0.00	0.00	0.00	0.00
ABHD14A	0.00	0.00	0.00	0.30	0.00	0.53	0.13	0.23	0.00	0.00	0.00	0.50	0.37	0.32	0.48	0.32	0.00	0.00	0.00	0.00	0.00
RAB3GAP2	0.00	0.00	0.00	0.22	0.00	0.33	0.11	0.13	0.00	0.00	0.00	0.32	0.16	0.13	0.33	0.14	0.00	0.00	0.00	0.00	0.00
CUX2	0.00	0.00	0.00	0.23	0.00	0.28	0.14	0.25	0.00	0.00	0.00	0.31	0.37	0.26	0.40	0.26	0.00	0.00	0.00	0.00	0.00
TSHZ1	0.00	0.00	0.00	0.23	0.00	0.36	0.10	0.29	0.00	0.00	0.00	0.22	0.15	0.10	0.31	0.13	0.00	0.00	0.00	0.00	0.00
BASP1	0.00	0.00	0.00	0.28	0.00	0.41	0.13	0.27	0.00	0.00	0.00	0.37	0.25	0.20	0.38	0.21	0.00	0.00	0.00	0.00	0.00
SEP-03	0.00	0.00	0.00	0.32	0.00	0.49	0.15	0.20	0.00	0.00	0.00	0.47	0.29	0.28	0.44	0.22	0.00	0.00	0.00	0.00	0.00
STMN2	0.00	0.00	0.00	0.23	0.00	0.27	0.13	0.27	0.00	0.00	0.00	0.32	0.29	0.23	0.45	0.28	0.00	0.00	0.00	0.00	0.00
MYT1L	0.00	0.00	0.00	0.29	0.00	0.43	0.15	0.29	0.00	0.00	0.00	0.36	0.28	0.21	0.48	0.27	0.00	0.00	0.00	0.00	0.00
MAB21L1	0.00	0.00	0.00	0.25	0.00	0.37	0.12	0.17	0.00	0.00	0.00	0.28	0.15	0.10	0.31	0.12	0.00	0.00	0.00	0.00	0.00
DISC1	0.00	0.00	0.00	0.15	0.00	0.15	0.00	0.14	0.15	0.00	0.00	0.28	0.32	0.28	0.26	0.13	0.00	0.00	0.00	0.00	0.00
PNMA2	0.00	0.00	0.00	0.19	0.00	0.36	0.00	0.15	0.20	0.00	0.00	0.30	0.17	0.16	0.29	0.17	0.00	0.00	0.00	0.00	0.00
REST	0.00	0.00	0.00	0.15	0.00	0.12	0.00	0.37	0.20	0.00	0.00	0.22	0.20	0.19	0.28	0.17	0.00	0.00	0.00	0.00	0.00
AFF3	0.00	0.00	0.00	0.22	0.00	0.38	0.00	0.27	0.13	0.00	0.00	0.26	0.17	0.15	0.25	0.11	0.00	0.00	0.00	0.00	0.00
HCN4	0.00	0.00	0.00	0.16	0.00	0.13	0.00	0.16	0.12	0.00	0.00	0.18	0.13	0.13	0.27	0.13	0.00	0.00	0.00	0.00	0.00
SPAST	0.00	0.00	0.00	0.15	0.00	0.22	0.00	0.11	0.14	0.00	0.00	0.22	0.17	0.13	0.29	0.17	0.00	0.00	0.00	0.00	0.00
DCX	0.00	0.00	0.00	0.13	0.00	0.14	0.00	0.20	0.15	0.00	0.00	0.28	0.35	0.32	0.31	0.24	0.00	0.00	0.00	0.00	0.00
FUS	0.00	0.00	0.00	0.15	0.00	0.17	0.00	0.23	0.23	0.00	0.00	0.14	0.14	0.12	0.20	0.10	0.00	0.00	0.00	0.00	0.00
S100B	0.00	0.00	0.00	0.12	0.00	0.10	0.00	0.17	0.14	0.00	0.00	0.18	0.15	0.21	0.22	0.18	0.00	0.00	0.00	0.00	0.00
GRN	0.00	0.00	0.00	0.12	0.00	0.10	0.00	0.19	0.28	0.00	0.00	0.18	0.16	0.18	0.21	0.15	0.00	0.00	0.00	0.00	0.00
ASPM	0.00	0.00	0.00	0.16	0.00	0.25	0.00	0.20	0.19	0.00	0.00	0.24	0.22	0.13	0.26	0.12	0.00	0.00	0.00	0.00	0.00
SERF2	0.00	0.00	0.00	0.23	0.00	0.40	0.00	0.19	0.24	0.00	0.00	0.26	0.14	0.12	0.33	0.18	0.00	0.00	0.00	0.00	0.00
SNCB	0.00	0.00	0.00	0.16	0.00	0.18	0.00	0.15	0.19	0.00	0.00	0.25	0.22	0.22	0.30	0.19	0.00	0.00	0.00	0.00	0.00
BCYRN1	0.00	0.00	0.00	0.25	0.00	0.43	0.00	0.25	0.16	0.00	0.00	0.41	0.23	0.25	0.35	0.19	0.00	0.00	0.00	0.00	0.00
CDK5R1	0.00	0.00	0.00	0.14	0.00	0.12	0.00	0.21	0.19	0.00	0.00	0.23	0.22	0.26	0.25	0.17	0.00	0.00	0.00	0.00	0.00
UCHL1	0.00	0.00	0.00	0.12	0.00	0.10	0.00	0.13	0.15	0.00	0.00	0.12	0.12	0.13	0.22	0.10	0.00	0.00	0.00	0.00	0.00
HCN1	0.00	0.00	0.00	0.12	0.00	0.10	0.00	0.14	0.15	0.00	0.00	0.21	0.22	0.25	0.28	0.19	0.00	0.00	0.00	0.00	0.00
CSTB	0.00	0.00	0.00	0.15	0.00	0.21	0.00	0.20	0.24	0.00	0.00	0.21	0.17	0.16	0.23	0.14	0.00	0.00	0.00	0.00	0.00
PAFAH1B1	0.00	0.00	0.00	0.13	0.00	0.15	0.00	0.14	0.11	0.00	0.00	0.25	0.27	0.21	0.24	0.16	0.00	0.00	0.00	0.00	0.00
CHAMP1	0.00	0.00	0.00	0.18	0.00	0.25	0.00	0.16	0.15	0.00	0.00	0.33	0.30	0.27	0.44	0.33	0.00	0.00	0.00	0.00	0.00
HAPLN4	0.00	0.00	0.00	0.24	0.00	0.35	0.13	0.00	0.00	0.00	0.13	0.40	0.27	0.25	0.42	0.22	0.00	0.00	0.00	0.00	0.00
KCNH4	0.00	0.00	0.00	0.24	0.00	0.36	0.11	0.00	0.00	0.00	0.19	0.42	0.26	0.31	0.36	0.23	0.00	0.00	0.00	0.00	0.00
HAPLN2	0.00	0.00	0.00	0.24	0.00	0.35	0.13	0.00	0.00	0.00	0.15	0.42	0.28	0.26	0.46	0.29	0.00	0.00	0.00	0.00	0.00
DPP6	0.00	0.00	0.00	0.21	0.00	0.28	0.11	0.00	0.00	0.00	0.16	0.32	0.19	0.24	0.31	0.17	0.00	0.00	0.00	0.00	0.00
CDH7	0.00	0.00	0.00	0.24	0.00	0.43	0.00	0.21	0.00	0.00	0.15	0.33	0.18	0.11	0.40	0.21	0.00	0.00	0.00	0.00	0.00
KCNH8	0.00	0.00	0.00	0.18	0.00	0.26	0.00	0.15	0.00	0.00	0.12	0.27	0.13	0.18	0.24	0.13	0.00	0.00	0.00	0.00	0.00
KCNIP1	0.00	0.00	0.00	0.19	0.00	0.25	0.00	0.14	0.00	0.00	0.16	0.31	0.22	0.28	0.26	0.16	0.00	0.00	0.00	0.00	0.00
KCNH6	0.00	0.00	0.00	0.18	0.00	0.24	0.00	0.14	0.00	0.00	0.12	0.28	0.16	0.19	0.36	0.23	0.00	0.00	0.00	0.00	0.00
KCNC1	0.00	0.00	0.00	0.11	0.00	0.16	0.00	0.11	0.00	0.00	0.12	0.23	0.19	0.22	0.29	0.22	0.00	0.00	0.00	0.00	0.00
KCNA4	0.00	0.00	0.00	0.16	0.00	0.17	0.00	0.12	0.00	0.00	0.13	0.22	0.13	0.19	0.25	0.18	0.00	0.00	0.00	0.00	0.00

KCNH3	0.00	0.00	0.00	0.17	0.00	0.26	0.00	0.11	0.00	0.00	0.16	0.34	0.20	0.28	0.31	0.20	0.00	0.00	0.00	0.00	0.00
ANKRD65	0.00	0.00	0.00	0.20	0.00	0.32	0.00	0.18	0.00	0.00	0.18	0.28	0.15	0.14	0.37	0.23	0.00	0.00	0.00	0.00	0.00
POMGNT1	0.00	0.00	0.00	0.14	0.00	0.21	0.00	0.00	0.20	0.00	0.13	0.27	0.17	0.16	0.30	0.16	0.00	0.00	0.00	0.00	0.00
FKTN	0.00	0.00	0.00	0.16	0.00	0.23	0.00	0.00	0.20	0.00	0.14	0.24	0.16	0.14	0.30	0.15	0.00	0.00	0.00	0.00	0.00
CLN5	0.00	0.00	0.00	0.18	0.00	0.26	0.00	0.00	0.24	0.00	0.14	0.34	0.24	0.21	0.36	0.28	0.00	0.00	0.00	0.00	0.00
SCN1B	0.00	0.00	0.00	0.15	0.00	0.17	0.00	0.00	0.11	0.00	0.19	0.23	0.11	0.16	0.29	0.17	0.00	0.00	0.00	0.00	0.00
LOC646506	0.00	0.00	0.00	0.15	0.00	0.22	0.00	0.00	0.14	0.00	0.10	0.30	0.22	0.28	0.19	0.14	0.00	0.00	0.00	0.00	0.00
POMT1	0.00	0.00	0.00	0.15	0.00	0.25	0.00	0.00	0.16	0.00	0.12	0.27	0.18	0.16	0.31	0.15	0.00	0.00	0.00	0.00	0.00
SCN2A	0.00	0.00	0.00	0.11	0.00	0.16	0.00	0.00	0.12	0.00	0.15	0.22	0.12	0.18	0.28	0.19	0.00	0.00	0.00	0.00	0.00
KCTD7	0.00	0.00	0.00	0.19	0.00	0.35	0.00	0.00	0.12	0.00	0.13	0.26	0.12	0.15	0.24	0.12	0.00	0.00	0.00	0.00	0.00
MFSD8	0.00	0.00	0.00	0.12	0.00	0.24	0.00	0.00	0.17	0.00	0.13	0.28	0.17	0.16	0.28	0.20	0.00	0.00	0.00	0.00	0.00
SCN8A	0.00	0.00	0.00	0.12	0.00	0.11	0.00	0.00	0.16	0.00	0.16	0.21	0.15	0.20	0.34	0.25	0.00	0.00	0.00	0.00	0.00
POMT2	0.00	0.00	0.00	0.17	0.00	0.29	0.00	0.00	0.16	0.00	0.13	0.27	0.16	0.15	0.28	0.11	0.00	0.00	0.00	0.00	0.00
KCNA1	0.00	0.00	0.00	0.00	0.00	0.12	0.00	0.13	0.16	0.00	0.13	0.21	0.17	0.21	0.29	0.25	0.00	0.00	0.00	0.00	0.00
IFI30	0.00	0.00	0.11	0.25	0.00	0.27	0.17	0.27	0.18	0.00	0.11	0.00	0.00	0.00	0.18	0.00	0.17	0.00	0.00	0.00	0.00
CMIP	0.00	0.00	0.16	0.34	0.00	0.40	0.21	0.25	0.13	0.00	0.00	0.13	0.00	0.00	0.17	0.00	0.11	0.00	0.00	0.00	0.00
ARTC1	0.00	0.00	0.16	0.28	0.00	0.28	0.20	0.28	0.25	0.00	0.00	0.13	0.00	0.00	0.14	0.00	0.12	0.00	0.00	0.00	0.00
TNFAIP1	0.00	0.00	0.12	0.27	0.00	0.33	0.18	0.20	0.11	0.00	0.00	0.13	0.00	0.00	0.16	0.00	0.12	0.00	0.00	0.00	0.00
HM13	0.00	0.00	0.11	0.25	0.00	0.34	0.14	0.17	0.00	0.00	0.28	0.15	0.00	0.00	0.16	0.00	0.11	0.00	0.00	0.00	0.00
TGM6	0.00	0.00	0.00	0.22	0.00	0.27	0.14	0.19	0.22	0.00	0.11	0.12	0.00	0.00	0.17	0.00	0.11	0.00	0.00	0.00	0.00
AGTR2	0.00	0.00	0.13	0.15	0.15	0.00	0.12	0.00	0.14	0.10	0.00	0.00	0.12	0.00	0.15	0.00	0.10	0.00	0.00	0.00	0.00
UMOD	0.00	0.00	0.10	0.19	0.00	0.16	0.14	0.00	0.15	0.00	0.12	0.00	0.11	0.00	0.13	0.00	0.16	0.00	0.00	0.00	0.00
WDR41	0.00	0.00	0.00	0.19	0.00	0.25	0.10	0.00	0.11	0.00	0.00	0.28	0.22	0.18	0.23	0.00	0.10	0.00	0.00	0.00	0.00
SOD3	0.00	0.00	0.00	0.11	0.00	0.10	0.00	0.12	0.16	0.00	0.00	0.13	0.12	0.11	0.13	0.00	0.15	0.00	0.00	0.00	0.00
TRBV5-2	0.00	0.00	0.00	0.22	0.00	0.31	0.12	0.15	0.18	0.00	0.00	0.13	0.00	0.00	0.20	0.18	0.13	0.00	0.00	0.00	0.00
FAM69A	0.00	0.00	0.12	0.28	0.00	0.40	0.16	0.00	0.00	0.00	0.19	0.21	0.00	0.00	0.25	0.11	0.15	0.00	0.00	0.00	0.00
IL1R1	0.00	0.00	0.10	0.15	0.00	0.00	0.13	0.00	0.16	0.00	0.00	0.11	0.00	0.12	0.15	0.13	0.13	0.00	0.00	0.00	0.00
EPO	0.00	0.00	0.00	0.14	0.00	0.00	0.11	0.11	0.16	0.00	0.00	0.11	0.00	0.13	0.16	0.11	0.11	0.00	0.00	0.00	0.00
CST3	0.00	0.00	0.00	0.00	0.00	0.10	0.00	0.11	0.18	0.00	0.00	0.15	0.14	0.13	0.16	0.12	0.17	0.00	0.00	0.00	0.00
GNAS	0.14	0.14	0.13	0.13	0.23	0.00	0.00	0.12	0.00	0.17	0.00	0.00	0.00	0.00	0.11	0.00	0.00	0.11	0.00	0.00	0.00
STRADB	0.00	0.00	0.13	0.31	0.00	0.40	0.18	0.30	0.14	0.00	0.00	0.12	0.00	0.00	0.16	0.00	0.00	0.13	0.00	0.00	0.00
MARK4	0.00	0.00	0.00	0.27	0.00	0.45	0.10	0.29	0.13	0.00	0.00	0.25	0.10	0.00	0.21	0.00	0.00	0.11	0.00	0.00	0.00
ATP1B1	0.00	0.00	0.00	0.19	0.00	0.21	0.12	0.12	0.00	0.00	0.19	0.17	0.11	0.00	0.15	0.00	0.00	0.11	0.00	0.00	0.00
MYO1A	0.00	0.00	0.00	0.26	0.00	0.36	0.13	0.16	0.00	0.00	0.14	0.20	0.12	0.00	0.20	0.00	0.00	0.10	0.00	0.00	0.00
ATP1B3	0.00	0.00	0.00	0.23	0.00	0.27	0.14	0.10	0.00	0.00	0.16	0.23	0.11	0.00	0.17	0.00	0.00	0.11	0.00	0.00	0.00
SLC17A6	0.00	0.00	0.00	0.13	0.00	0.10	0.00	0.13	0.00	0.00	0.00	0.20	0.31	0.26	0.35	0.23	0.00	0.11	0.00	0.00	0.00
PLEKHA8	0.00	0.00	0.11	0.32	0.00	0.50	0.15	0.32	0.16	0.00	0.18	0.14	0.00	0.00	0.00	0.00	0.00	0.00	0.15	0.00	0.00
MBTPS1	0.00	0.00	0.14	0.25	0.00	0.27	0.16	0.25	0.13	0.00	0.21	0.00	0.00	0.00	0.14	0.00	0.00	0.00	0.13	0.00	0.00
TFR2	0.00	0.00	0.14	0.22	0.00	0.14	0.18	0.13	0.15	0.00	0.12	0.00	0.00	0.00	0.13	0.00	0.00	0.00	0.18	0.00	0.00
NPHP3	0.00	0.00	0.11	0.18	0.00	0.20	0.12	0.10	0.20	0.00	0.11	0.00	0.00	0.00	0.13	0.00	0.00	0.00	0.13	0.00	0.00

TPO	0.00	0.00	0.00	0.16	0.00	0.14	0.12	0.14	0.11	0.24	0.10	0.00	0.00	0.00	0.11	0.00	0.00	0.00	0.10	0.00	0.00
PLA2G15	0.00	0.00	0.13	0.26	0.00	0.28	0.18	0.16	0.14	0.00	0.00	0.15	0.00	0.00	0.11	0.00	0.00	0.00	0.30	0.00	0.00
AK8	0.00	0.00	0.16	0.34	0.00	0.47	0.18	0.18	0.11	0.00	0.00	0.26	0.00	0.00	0.23	0.00	0.00	0.00	0.17	0.00	0.00
CERK	0.00	0.00	0.18	0.31	0.00	0.30	0.21	0.28	0.21	0.00	0.00	0.17	0.00	0.00	0.15	0.00	0.00	0.00	0.23	0.00	0.00
ACER3	0.00	0.00	0.17	0.30	0.00	0.40	0.15	0.25	0.15	0.00	0.00	0.16	0.00	0.00	0.11	0.00	0.00	0.00	0.22	0.00	0.00
UBR1	0.00	0.00	0.13	0.27	0.00	0.30	0.16	0.32	0.19	0.00	0.00	0.14	0.00	0.00	0.20	0.00	0.00	0.00	0.10	0.00	0.00
MIR433	0.00	0.00	0.15	0.31	0.00	0.37	0.19	0.37	0.20	0.00	0.00	0.16	0.00	0.00	0.14	0.00	0.00	0.00	0.10	0.00	0.00
XPNPEP2	0.00	0.00	0.10	0.21	0.00	0.24	0.12	0.17	0.13	0.00	0.00	0.17	0.00	0.00	0.16	0.00	0.00	0.00	0.22	0.00	0.00
SRSF9	0.00	0.00	0.12	0.30	0.00	0.35	0.17	0.28	0.12	0.00	0.00	0.12	0.00	0.00	0.17	0.00	0.00	0.00	0.14	0.00	0.00
KAT5	0.00	0.00	0.13	0.23	0.00	0.18	0.17	0.37	0.18	0.00	0.00	0.10	0.00	0.00	0.15	0.00	0.00	0.00	0.10	0.00	0.00
LSS	0.00	0.00	0.12	0.26	0.00	0.29	0.16	0.21	0.18	0.00	0.00	0.19	0.00	0.00	0.16	0.00	0.00	0.00	0.37	0.00	0.00
UBE2G1	0.00	0.00	0.15	0.31	0.00	0.38	0.18	0.28	0.00	0.00	0.12	0.19	0.00	0.00	0.18	0.00	0.00	0.00	0.10	0.00	0.00
SMAP1	0.00	0.00	0.17	0.36	0.00	0.51	0.20	0.11	0.00	0.00	0.14	0.20	0.00	0.00	0.16	0.00	0.00	0.00	0.12	0.00	0.00
EBPL	0.00	0.00	0.18	0.39	0.00	0.52	0.23	0.15	0.00	0.00	0.19	0.23	0.00	0.00	0.13	0.00	0.00	0.00	0.31	0.00	0.00
C1ORF85	0.00	0.00	0.20	0.47	0.00	0.62	0.26	0.26	0.00	0.00	0.14	0.24	0.00	0.00	0.23	0.00	0.00	0.00	0.15	0.00	0.00
OSBP2	0.00	0.00	0.11	0.32	0.00	0.50	0.15	0.21	0.00	0.00	0.13	0.21	0.00	0.00	0.21	0.00	0.00	0.00	0.20	0.00	0.00
NIPSNAP3A	0.00	0.00	0.12	0.36	0.00	0.54	0.18	0.25	0.00	0.00	0.17	0.17	0.00	0.00	0.17	0.00	0.00	0.00	0.15	0.00	0.00
OSBP	0.00	0.00	0.17	0.38	0.00	0.49	0.22	0.26	0.00	0.00	0.22	0.19	0.00	0.00	0.18	0.00	0.00	0.00	0.16	0.00	0.00
LBR	0.00	0.00	0.14	0.31	0.00	0.35	0.20	0.24	0.00	0.00	0.14	0.13	0.00	0.00	0.19	0.00	0.00	0.00	0.13	0.00	0.00
XRN1	0.00	0.00	0.13	0.38	0.00	0.60	0.18	0.25	0.00	0.00	0.11	0.25	0.00	0.00	0.30	0.00	0.00	0.00	0.11	0.00	0.00
MTMR6	0.00	0.00	0.13	0.34	0.00	0.48	0.17	0.30	0.00	0.00	0.11	0.20	0.00	0.00	0.14	0.00	0.00	0.00	0.15	0.00	0.00
SAR1B	0.00	0.00	0.12	0.30	0.00	0.50	0.12	0.19	0.00	0.00	0.13	0.16	0.00	0.00	0.14	0.00	0.00	0.00	0.17	0.00	0.00
DYT10	0.00	0.00	0.13	0.32	0.00	0.43	0.16	0.21	0.00	0.00	0.15	0.21	0.00	0.00	0.15	0.00	0.00	0.00	0.14	0.00	0.00
OSBPL2	0.00	0.00	0.18	0.40	0.00	0.52	0.23	0.23	0.00	0.00	0.19	0.24	0.00	0.00	0.17	0.00	0.00	0.00	0.32	0.00	0.00
OSBPL1A	0.00	0.00	0.18	0.37	0.00	0.46	0.23	0.27	0.00	0.00	0.16	0.23	0.00	0.00	0.20	0.00	0.00	0.00	0.22	0.00	0.00
ATP8B5P	0.00	0.00	0.13	0.35	0.00	0.56	0.15	0.15	0.00	0.00	0.29	0.20	0.00	0.00	0.14	0.00	0.00	0.00	0.11	0.00	0.00
ANXA6	0.00	0.00	0.14	0.26	0.00	0.27	0.17	0.20	0.00	0.00	0.13	0.15	0.00	0.00	0.16	0.00	0.00	0.00	0.13	0.00	0.00
TM7SF2	0.00	0.00	0.16	0.37	0.00	0.49	0.22	0.24	0.00	0.00	0.21	0.22	0.00	0.00	0.21	0.00	0.00	0.00	0.26	0.00	0.00
LDLRAP1	0.00	0.00	0.18	0.30	0.00	0.24	0.24	0.14	0.00	0.00	0.13	0.15	0.00	0.00	0.16	0.00	0.00	0.00	0.20	0.00	0.00
OSBPL3	0.00	0.00	0.22	0.44	0.00	0.56	0.26	0.26	0.00	0.00	0.24	0.29	0.00	0.00	0.24	0.00	0.00	0.00	0.23	0.00	0.00
FAM189B	0.00	0.00	0.13	0.37	0.00	0.57	0.19	0.23	0.00	0.00	0.12	0.18	0.00	0.00	0.16	0.00	0.00	0.00	0.10	0.00	0.00
PLBD2	0.00	0.00	0.10	0.33	0.00	0.53	0.14	0.11	0.00	0.00	0.12	0.25	0.00	0.00	0.19	0.00	0.00	0.00	0.17	0.00	0.00
OSBPL5	0.00	0.00	0.15	0.33	0.00	0.38	0.21	0.26	0.00	0.00	0.16	0.19	0.00	0.00	0.18	0.00	0.00	0.00	0.12	0.00	0.00
MTMR3	0.00	0.00	0.11	0.32	0.00	0.50	0.14	0.24	0.00	0.00	0.15	0.16	0.00	0.00	0.13	0.00	0.00	0.00	0.17	0.00	0.00
VAPA	0.00	0.00	0.12	0.32	0.00	0.47	0.16	0.26	0.00	0.00	0.27	0.22	0.00	0.00	0.24	0.00	0.00	0.00	0.11	0.00	0.00
ABCA12	0.00	0.00	0.00	0.20	0.00	0.26	0.11	0.11	0.14	0.00	0.13	0.13	0.00	0.00	0.17	0.00	0.00	0.00	0.18	0.00	0.00
TCIRG1	0.00	0.00	0.00	0.22	0.00	0.22	0.14	0.15	0.16	0.00	0.14	0.19	0.00	0.00	0.20	0.00	0.00	0.00	0.15	0.00	0.00
COMMD1	0.00	0.00	0.00	0.23	0.00	0.32	0.11	0.29	0.16	0.00	0.10	0.15	0.00	0.00	0.19	0.00	0.00	0.00	0.13	0.00	0.00
LGSN	0.00	0.00	0.00	0.30	0.00	0.51	0.12	0.22	0.13	0.00	0.10	0.24	0.00	0.00	0.27	0.00	0.00	0.00	0.16	0.00	0.00
GOLM1	0.00	0.00	0.00	0.20	0.00	0.27	0.11	0.22	0.27	0.00	0.11	0.14	0.00	0.00	0.12	0.00	0.00	0.00	0.10	0.00	0.00

SLC46A1	0.00	0.00	0.00	0.21	0.00	0.23	0.12	0.14	0.12	0.00	0.19	0.14	0.00	0.00	0.18	0.00	0.00	0.00	0.29	0.00	0.00
CLCN5	0.00	0.00	0.00	0.19	0.00	0.19	0.12	0.11	0.11	0.00	0.18	0.00	0.12	0.00	0.12	0.00	0.00	0.00	0.13	0.00	0.00
HDGFL1	0.00	0.00	0.10	0.35	0.00	0.64	0.13	0.15	0.00	0.00	0.00	0.35	0.10	0.00	0.26	0.00	0.00	0.00	0.11	0.00	0.00
CYP2G1P	0.00	0.00	0.15	0.35	0.00	0.43	0.21	0.12	0.00	0.00	0.00	0.26	0.12	0.00	0.27	0.00	0.00	0.00	0.24	0.00	0.00
DNTTIP2	0.00	0.00	0.18	0.35	0.00	0.40	0.21	0.30	0.00	0.00	0.00	0.18	0.12	0.00	0.18	0.00	0.00	0.00	0.13	0.00	0.00
NCEH1	0.00	0.00	0.00	0.25	0.00	0.35	0.13	0.30	0.21	0.00	0.00	0.18	0.11	0.00	0.13	0.00	0.00	0.00	0.31	0.00	0.00
QDPR	0.00	0.00	0.00	0.21	0.00	0.21	0.13	0.11	0.13	0.00	0.00	0.21	0.11	0.00	0.22	0.00	0.00	0.00	0.20	0.00	0.00
ENO1	0.00	0.00	0.00	0.20	0.00	0.24	0.11	0.27	0.23	0.00	0.00	0.20	0.11	0.00	0.18	0.00	0.00	0.00	0.13	0.00	0.00
FMO1	0.00	0.00	0.00	0.20	0.00	0.25	0.11	0.12	0.19	0.00	0.00	0.22	0.13	0.00	0.20	0.00	0.00	0.00	0.33	0.00	0.00
STS	0.00	0.00	0.00	0.19	0.00	0.16	0.14	0.14	0.17	0.00	0.00	0.17	0.16	0.00	0.15	0.00	0.00	0.00	0.23	0.00	0.00
GABARAPL3	0.00	0.00	0.24	0.40	0.00	0.39	0.29	0.00	0.00	0.00	0.14	0.32	0.15	0.00	0.23	0.00	0.00	0.00	0.12	0.00	0.00
SLC52A3	0.00	0.00	0.00	0.27	0.00	0.43	0.12	0.16	0.00	0.00	0.25	0.17	0.10	0.00	0.17	0.00	0.00	0.00	0.15	0.00	0.00
ATP6V1B1	0.00	0.00	0.00	0.20	0.00	0.23	0.10	0.12	0.00	0.00	0.22	0.16	0.12	0.00	0.15	0.00	0.00	0.00	0.11	0.00	0.00
SLC41A1	0.00	0.00	0.00	0.28	0.00	0.43	0.12	0.11	0.00	0.00	0.28	0.27	0.15	0.00	0.21	0.00	0.00	0.00	0.15	0.00	0.00
CTNS	0.00	0.00	0.00	0.17	0.00	0.24	0.00	0.12	0.21	0.00	0.11	0.17	0.12	0.00	0.19	0.00	0.00	0.00	0.18	0.00	0.00
A2M	0.00	0.00	0.00	0.18	0.00	0.14	0.13	0.10	0.12	0.00	0.00	0.12	0.00	0.10	0.15	0.00	0.00	0.00	0.10	0.00	0.00
ARL5B	0.00	0.00	0.00	0.26	0.00	0.41	0.12	0.15	0.00	0.00	0.00	0.25	0.11	0.10	0.17	0.00	0.00	0.00	0.10	0.00	0.00
BACE1-AS	0.00	0.00	0.00	0.15	0.00	0.24	0.00	0.26	0.25	0.00	0.00	0.28	0.15	0.21	0.19	0.00	0.00	0.00	0.13	0.00	0.00
SREK1IP1	0.00	0.00	0.00	0.14	0.00	0.31	0.00	0.11	0.13	0.00	0.00	0.30	0.16	0.21	0.18	0.00	0.00	0.00	0.11	0.00	0.00
CRABP1	0.00	0.00	0.12	0.18	0.00	0.11	0.15	0.22	0.00	0.00	0.00	0.12	0.00	0.00	0.23	0.13	0.00	0.00	0.13	0.00	0.00
LRP1	0.00	0.00	0.10	0.16	0.00	0.00	0.14	0.10	0.12	0.00	0.00	0.12	0.00	0.00	0.14	0.11	0.00	0.00	0.15	0.00	0.00
UBE4B	0.00	0.00	0.00	0.24	0.00	0.37	0.11	0.31	0.15	0.00	0.00	0.22	0.00	0.00	0.30	0.12	0.00	0.00	0.10	0.00	0.00
ABHD12	0.00	0.00	0.00	0.16	0.00	0.16	0.11	0.11	0.15	0.00	0.00	0.21	0.00	0.00	0.23	0.14	0.00	0.00	0.22	0.00	0.00
CLCN7	0.00	0.00	0.00	0.18	0.00	0.20	0.11	0.00	0.15	0.00	0.18	0.19	0.00	0.00	0.21	0.12	0.00	0.00	0.12	0.00	0.00
GLB1	0.00	0.00	0.00	0.18	0.00	0.18	0.12	0.00	0.22	0.00	0.00	0.17	0.12	0.00	0.22	0.12	0.00	0.00	0.10	0.00	0.00
B3GALT4	0.00	0.00	0.00	0.22	0.00	0.44	0.00	0.22	0.12	0.00	0.00	0.29	0.10	0.00	0.25	0.15	0.00	0.00	0.12	0.00	0.00
ATXN2	0.00	0.00	0.00	0.15	0.00	0.19	0.00	0.11	0.16	0.00	0.00	0.19	0.13	0.00	0.21	0.11	0.00	0.00	0.10	0.00	0.00
SGSH	0.00	0.00	0.00	0.15	0.00	0.21	0.00	0.10	0.28	0.00	0.00	0.21	0.12	0.00	0.24	0.15	0.00	0.00	0.15	0.00	0.00
GALC	0.00	0.00	0.00	0.14	0.00	0.13	0.00	0.13	0.30	0.00	0.00	0.21	0.16	0.00	0.37	0.39	0.00	0.00	0.17	0.00	0.00
AGA	0.00	0.00	0.00	0.18	0.00	0.25	0.00	0.00	0.16	0.00	0.11	0.23	0.14	0.00	0.26	0.16	0.00	0.00	0.14	0.00	0.00
PPT2-EGFL8	0.00	0.00	0.00	0.17	0.00	0.34	0.00	0.00	0.11	0.00	0.10	0.26	0.12	0.00	0.17	0.13	0.00	0.00	0.26	0.00	0.00
ZNF597	0.00	0.00	0.00	0.33	0.00	0.58	0.12	0.00	0.00	0.00	0.00	0.52	0.30	0.27	0.41	0.21	0.00	0.00	0.13	0.00	0.00
NXF4	0.00	0.00	0.00	0.30	0.00	0.55	0.00	0.21	0.00	0.00	0.00	0.35	0.22	0.20	0.30	0.10	0.00	0.00	0.14	0.00	0.00
ARSA	0.00	0.00	0.00	0.11	0.00	0.12	0.00	0.00	0.22	0.00	0.00	0.20	0.17	0.12	0.28	0.27	0.00	0.00	0.19	0.00	0.00
CTSD	0.00	0.00	0.00	0.12	0.00	0.00	0.00	0.16	0.19	0.00	0.00	0.13	0.12	0.10	0.15	0.12	0.00	0.00	0.10	0.00	0.00
TLCD1	0.00	0.00	0.00	0.24	0.00	0.50	0.00	0.00	0.00	0.00	0.23	0.37	0.19	0.19	0.27	0.19	0.00	0.00	0.10	0.00	0.00
B3GALT1	0.00	0.00	0.00	0.26	0.00	0.50	0.00	0.00	0.00	0.00	0.23	0.34	0.14	0.11	0.24	0.13	0.00	0.00	0.17	0.00	0.00
PCSK9	0.00	0.00	0.10	0.18	0.00	0.12	0.14	0.19	0.19	0.00	0.00	0.00	0.00	0.00	0.12	0.00	0.14	0.00	0.18	0.00	0.00
SCAP	0.00	0.00	0.00	0.19	0.00	0.21	0.13	0.22	0.12	0.00	0.14	0.00	0.00	0.00	0.14	0.00	0.13	0.00	0.25	0.00	0.00
ZNF202	0.00	0.00	0.11	0.29	0.00	0.40	0.17	0.27	0.00	0.00	0.00	0.11	0.00	0.00	0.13	0.00	0.14	0.00	0.20	0.00	0.00

HMGCR	0.00	0.00	0.00	0.14	0.00	0.12	0.10	0.20	0.23	0.00	0.00	0.12	0.00	0.00	0.15	0.00	0.15	0.00	0.24	0.00	0.00
MAN2A1	0.00	0.00	0.00	0.25	0.00	0.35	0.13	0.10	0.00	0.00	0.19	0.17	0.00	0.00	0.17	0.00	0.13	0.00	0.15	0.00	0.00
ZFAND2B	0.00	0.00	0.00	0.15	0.00	0.30	0.00	0.19	0.27	0.00	0.00	0.17	0.00	0.10	0.17	0.00	0.12	0.00	0.14	0.00	0.00
PTBP1	0.00	0.00	0.00	0.22	0.00	0.26	0.11	0.28	0.11	0.00	0.00	0.14	0.00	0.00	0.21	0.00	0.00	0.12	0.19	0.00	0.00
ATOX1	0.00	0.00	0.00	0.22	0.00	0.31	0.00	0.21	0.14	0.00	0.11	0.20	0.00	0.00	0.21	0.00	0.00	0.10	0.16	0.00	0.00
NKPD1	0.00	0.00	0.00	0.20	0.00	0.25	0.10	0.00	0.17	0.00	0.00	0.26	0.11	0.00	0.16	0.00	0.00	0.11	0.27	0.00	0.00
DBH	0.00	0.00	0.00	0.12	0.00	0.00	0.00	0.12	0.14	0.00	0.00	0.11	0.14	0.12	0.22	0.00	0.00	0.11	0.10	0.00	0.00
GM2AP1	0.00	0.00	0.00	0.24	0.00	0.34	0.13	0.17	0.00	0.00	0.00	0.16	0.00	0.00	0.19	0.10	0.00	0.14	0.25	0.00	0.00
BCHE	0.00	0.00	0.00	0.00	0.00	0.00	0.00	0.11	0.18	0.00	0.00	0.11	0.13	0.15	0.14	0.00	0.16	0.13	0.12	0.00	0.00
MT2A	0.00	0.00	0.00	0.00	0.00	0.00	0.00	0.00	0.11	0.00	0.00	0.11	0.13	0.14	0.14	0.14	0.10	0.10	0.11	0.00	0.00
BAG2	0.00	0.00	0.13	0.28	0.00	0.35	0.17	0.28	0.10	0.00	0.00	0.11	0.00	0.00	0.14	0.00	0.00	0.00	0.00	0.10	0.00
TRIP10	0.00	0.00	0.16	0.33	0.00	0.35	0.22	0.25	0.11	0.00	0.00	0.15	0.00	0.00	0.15	0.00	0.00	0.00	0.00	0.16	0.00
STX16	0.00	0.00	0.14	0.29	0.00	0.33	0.18	0.20	0.00	0.00	0.24	0.10	0.00	0.00	0.16	0.00	0.00	0.00	0.00	0.11	0.00
SLC50A1	0.00	0.00	0.24	0.47	0.00	0.52	0.31	0.29	0.00	0.00	0.28	0.22	0.00	0.00	0.28	0.00	0.00	0.00	0.00	0.11	0.00
MYO5B	0.00	0.00	0.18	0.34	0.00	0.34	0.23	0.18	0.00	0.00	0.18	0.17	0.00	0.00	0.17	0.00	0.00	0.00	0.00	0.11	0.00
STX6	0.00	0.00	0.17	0.32	0.00	0.31	0.22	0.27	0.00	0.00	0.23	0.16	0.00	0.00	0.19	0.00	0.00	0.00	0.00	0.16	0.00
STX1A	0.00	0.00	0.00	0.15	0.00	0.11	0.00	0.21	0.00	0.00	0.14	0.12	0.10	0.12	0.19	0.00	0.00	0.00	0.00	0.13	0.00
CDK5	0.00	0.00	0.00	0.11	0.00	0.00	0.00	0.15	0.16	0.00	0.00	0.16	0.14	0.19	0.18	0.11	0.00	0.00	0.00	0.12	0.00
KCNB2	0.00	0.00	0.00	0.18	0.00	0.27	0.00	0.00	0.00	0.00	0.14	0.30	0.18	0.23	0.28	0.16	0.00	0.00	0.00	0.10	0.00
DNAJB1P1	0.00	0.00	0.11	0.27	0.00	0.41	0.14	0.25	0.16	0.00	0.00	0.13	0.00	0.00	0.00	0.00	0.12	0.00	0.00	0.15	0.00
ATF6	0.00	0.00	0.00	0.19	0.00	0.21	0.10	0.25	0.18	0.00	0.16	0.00	0.00	0.00	0.12	0.00	0.20	0.00	0.00	0.19	0.00
ARHGAP22	0.00	0.00	0.16	0.33	0.00	0.42	0.20	0.23	0.00	0.00	0.00	0.20	0.00	0.00	0.18	0.00	0.26	0.00	0.00	0.16	0.00
RPL14	0.00	0.00	0.11	0.31	0.00	0.49	0.14	0.12	0.00	0.00	0.00	0.20	0.00	0.00	0.17	0.00	0.21	0.00	0.00	0.13	0.00
CPA1	0.00	0.00	0.00	0.22	0.00	0.26	0.13	0.21	0.12	0.00	0.00	0.16	0.00	0.00	0.27	0.00	0.20	0.00	0.00	0.16	0.00
CHGA	0.00	0.00	0.00	0.14	0.00	0.12	0.00	0.17	0.18	0.00	0.00	0.12	0.11	0.00	0.19	0.00	0.13	0.00	0.00	0.12	0.00
KCNMB1	0.00	0.00	0.00	0.13	0.00	0.12	0.00	0.16	0.16	0.00	0.00	0.13	0.00	0.11	0.14	0.00	0.16	0.00	0.00	0.13	0.00
NEUROD1	0.00	0.00	0.00	0.11	0.00	0.00	0.00	0.21	0.00	0.00	0.00	0.14	0.18	0.14	0.28	0.17	0.20	0.00	0.00	0.15	0.00
SLC9A1	0.00	0.00	0.00	0.11	0.00	0.00	0.00	0.17	0.16	0.00	0.12	0.11	0.18	0.12	0.00	0.00	0.00	0.14	0.00	0.11	0.00
RAB11A	0.00	0.00	0.18	0.27	0.00	0.23	0.18	0.21	0.00	0.00	0.13	0.00	0.00	0.00	0.11	0.00	0.00	0.13	0.00	0.14	0.00
ARL4C	0.00	0.00	0.21	0.37	0.00	0.40	0.23	0.26	0.00	0.00	0.00	0.14	0.00	0.00	0.13	0.00	0.00	0.00	0.17	0.11	0.00
MTM1	0.00	0.00	0.00	0.22	0.00	0.30	0.11	0.15	0.14	0.00	0.00	0.12	0.00	0.00	0.15	0.00	0.00	0.00	0.15	0.12	0.00
BLVRA	0.00	0.00	0.00	0.22	0.00	0.26	0.12	0.18	0.15	0.00	0.00	0.16	0.00	0.00	0.14	0.00	0.00	0.00	0.22	0.10	0.00
SAA1	0.00	0.00	0.12	0.20	0.00	0.13	0.15	0.14	0.20	0.00	0.00	0.00	0.00	0.00	0.00	0.00	0.16	0.00	0.14	0.14	0.00
MIR133A1	0.00	0.00	0.00	0.22	0.00	0.32	0.11	0.45	0.26	0.00	0.00	0.12	0.00	0.00	0.00	0.00	0.16	0.00	0.14	0.16	0.00
LVG2	0.00	0.00	0.00	0.18	0.00	0.23	0.12	0.13	0.11	0.00	0.00	0.00	0.00	0.00	0.14	0.00	0.22	0.00	0.17	0.15	0.00
GC	0.00	0.00	0.00	0.17	0.00	0.14	0.12	0.12	0.00	0.00	0.00	0.11	0.00	0.00	0.17	0.00	0.16	0.00	0.19	0.10	0.00
PEPD	0.00	0.00	0.00	0.17	0.00	0.22	0.00	0.16	0.20	0.00	0.00	0.13	0.00	0.00	0.12	0.00	0.11	0.00	0.21	0.11	0.00
SRD5A3	0.00	0.00	0.00	0.21	0.00	0.32	0.00	0.17	0.16	0.00	0.00	0.19	0.00	0.00	0.19	0.00	0.16	0.00	0.33	0.20	0.00
ATP10D	0.00	0.00	0.00	0.27	0.00	0.45	0.11	0.00	0.00	0.00	0.17	0.20	0.00	0.00	0.17	0.00	0.26	0.00	0.26	0.20	0.00
MAT1A	0.00	0.00	0.00	0.19	0.00	0.26	0.00	0.22	0.18	0.00	0.00	0.16	0.00	0.00	0.13	0.00	0.00	0.13	0.35	0.11	0.00

NR1H4	0.00	0.00	0.13	0.20	0.00	0.00	0.18	0.24	0.21	0.00	0.00	0.00	0.00	0.00	0.00	0.00	0.12	0.15	0.23	0.15	0.00
MTTP	0.00	0.00	0.00	0.16	0.00	0.11	0.12	0.13	0.11	0.00	0.00	0.00	0.00	0.00	0.00	0.00	0.18	0.15	0.27	0.17	0.00
CYP7A1	0.00	0.00	0.00	0.17	0.00	0.11	0.15	0.20	0.12	0.00	0.00	0.00	0.00	0.00	0.00	0.00	0.14	0.13	0.30	0.12	0.00
CPA2	0.00	0.00	0.00	0.18	0.00	0.32	0.00	0.15	0.00	0.00	0.00	0.17	0.00	0.00	0.17	0.00	0.15	0.16	0.14	0.11	0.00
TRIM37	0.00	0.00	0.00	0.22	0.00	0.39	0.00	0.16	0.00	0.00	0.00	0.19	0.00	0.00	0.22	0.00	0.17	0.12	0.19	0.18	0.00
AGL	0.00	0.00	0.00	0.11	0.00	0.21	0.00	0.00	0.14	0.00	0.00	0.14	0.00	0.00	0.13	0.00	0.13	0.16	0.27	0.22	0.00
SLC2A10	0.00	0.00	0.00	0.18	0.00	0.29	0.00	0.00	0.00	0.00	0.11	0.13	0.00	0.00	0.14	0.00	0.37	0.15	0.23	0.39	0.00
TMED4	0.00	0.00	0.14	0.32	0.00	0.46	0.16	0.28	0.13	0.00	0.19	0.12	0.00	0.00	0.00	0.00	0.00	0.00	0.00	0.00	0.19
ST13	0.00	0.00	0.24	0.37	0.00	0.34	0.26	0.27	0.16	0.00	0.12	0.00	0.00	0.00	0.17	0.00	0.00	0.00	0.00	0.00	0.13
DNAJB1	0.00	0.00	0.11	0.22	0.00	0.26	0.14	0.20	0.14	0.00	0.00	0.11	0.00	0.00	0.17	0.00	0.00	0.00	0.00	0.00	0.11
FAF1	0.00	0.00	0.12	0.31	0.00	0.37	0.19	0.33	0.14	0.00	0.00	0.14	0.00	0.00	0.22	0.00	0.00	0.00	0.00	0.00	0.11
UVRAG	0.00	0.00	0.15	0.33	0.00	0.39	0.19	0.33	0.14	0.00	0.00	0.13	0.00	0.00	0.16	0.00	0.00	0.00	0.00	0.00	0.16
DRAM2	0.00	0.00	0.10	0.31	0.00	0.48	0.15	0.43	0.17	0.00	0.00	0.11	0.00	0.00	0.14	0.00	0.00	0.00	0.00	0.00	0.12
DNAJA3	0.00	0.00	0.15	0.31	0.00	0.33	0.21	0.35	0.24	0.00	0.00	0.14	0.00	0.00	0.19	0.00	0.00	0.00	0.00	0.00	0.16
HSPBP1	0.00	0.00	0.13	0.26	0.00	0.31	0.16	0.28	0.25	0.00	0.00	0.14	0.00	0.00	0.15	0.00	0.00	0.00	0.00	0.00	0.12
DFFB	0.00	0.00	0.12	0.27	0.00	0.28	0.18	0.29	0.18	0.00	0.00	0.14	0.00	0.00	0.22	0.00	0.00	0.00	0.00	0.00	0.17
APOL6	0.00	0.00	0.12	0.30	0.00	0.42	0.17	0.27	0.17	0.00	0.00	0.13	0.00	0.00	0.14	0.00	0.00	0.00	0.00	0.00	0.23
VPS41	0.00	0.00	0.13	0.36	0.00	0.50	0.20	0.21	0.00	0.00	0.18	0.20	0.00	0.00	0.21	0.00	0.00	0.00	0.00	0.00	0.16
VPS53	0.00	0.00	0.12	0.34	0.00	0.48	0.18	0.35	0.00	0.00	0.15	0.12	0.00	0.00	0.13	0.00	0.00	0.00	0.00	0.00	0.21
HS1BP3	0.00	0.00	0.19	0.39	0.00	0.48	0.24	0.23	0.00	0.00	0.11	0.18	0.00	0.00	0.20	0.00	0.00	0.00	0.00	0.00	0.14
WDR45L	0.00	0.00	0.22	0.45	0.00	0.55	0.28	0.33	0.00	0.00	0.17	0.13	0.00	0.00	0.16	0.00	0.00	0.00	0.00	0.00	0.10
TMEM85	0.00	0.00	0.00	0.37	0.00	0.68	0.14	0.33	0.12	0.00	0.15	0.18	0.00	0.00	0.13	0.00	0.00	0.00	0.00	0.00	0.26
WDR35	0.00	0.00	0.00	0.25	0.00	0.43	0.11	0.13	0.11	0.00	0.11	0.17	0.00	0.00	0.20	0.00	0.00	0.00	0.00	0.00	0.11
TUBB2A	0.00	0.00	0.13	0.27	0.00	0.29	0.15	0.19	0.00	0.00	0.00	0.22	0.12	0.00	0.21	0.00	0.00	0.00	0.00	0.00	0.15
TUBA4A	0.00	0.00	0.15	0.29	0.00	0.30	0.18	0.18	0.00	0.00	0.00	0.23	0.14	0.00	0.24	0.00	0.00	0.00	0.00	0.00	0.15
PSMD1	0.00	0.00	0.00	0.27	0.00	0.39	0.12	0.30	0.13	0.00	0.00	0.18	0.00	0.10	0.22	0.00	0.00	0.00	0.00	0.00	0.14
DFFA	0.00	0.00	0.00	0.25	0.00	0.29	0.15	0.34	0.20	0.00	0.00	0.16	0.00	0.12	0.21	0.00	0.00	0.00	0.00	0.00	0.20
KLC2	0.00	0.00	0.00	0.31	0.00	0.45	0.16	0.18	0.00	0.00	0.00	0.29	0.14	0.11	0.30	0.00	0.00	0.00	0.00	0.00	0.11
APAF1	0.00	0.00	0.00	0.13	0.00	0.11	0.00	0.21	0.20	0.00	0.00	0.12	0.10	0.11	0.19	0.00	0.00	0.00	0.00	0.00	0.23
CASP2	0.00	0.00	0.00	0.15	0.00	0.15	0.00	0.21	0.20	0.00	0.00	0.12	0.10	0.11	0.17	0.00	0.00	0.00	0.00	0.00	0.19
GSN	0.00	0.00	0.00	0.16	0.00	0.16	0.00	0.18	0.16	0.00	0.00	0.14	0.13	0.12	0.16	0.00	0.00	0.00	0.00	0.00	0.11
CASP6	0.00	0.00	0.00	0.15	0.00	0.13	0.00	0.21	0.16	0.00	0.00	0.13	0.12	0.14	0.17	0.00	0.00	0.00	0.00	0.00	0.14
GAN	0.00	0.00	0.00	0.19	0.00	0.31	0.00	0.16	0.14	0.00	0.00	0.23	0.13	0.00	0.38	0.22	0.00	0.00	0.00	0.00	0.10
ZNF746	0.00	0.00	0.00	0.13	0.00	0.14	0.00	0.18	0.00	0.00	0.00	0.22	0.17	0.15	0.20	0.10	0.00	0.00	0.00	0.00	0.11
NDEL1	0.00	0.00	0.00	0.15	0.00	0.22	0.00	0.18	0.00	0.00	0.00	0.28	0.27	0.18	0.28	0.14	0.00	0.00	0.00	0.00	0.12
HTT	0.00	0.00	0.00	0.14	0.00	0.00	0.11	0.13	0.22	0.00	0.00	0.14	0.12	0.00	0.12	0.00	0.00	0.15	0.00	0.00	0.11
TMEM74	0.00	0.00	0.11	0.28	0.00	0.40	0.14	0.25	0.00	0.00	0.00	0.12	0.00	0.00	0.12	0.00	0.00	0.00	0.11	0.00	0.13
ATG9B	0.00	0.00	0.00	0.24	0.00	0.38	0.12	0.30	0.13	0.00	0.00	0.12	0.00	0.00	0.16	0.00	0.00	0.00	0.14	0.00	0.11
GPX6	0.00	0.00	0.00	0.20	0.00	0.24	0.12	0.18	0.11	0.00	0.00	0.21	0.00	0.00	0.16	0.00	0.00	0.00	0.18	0.00	0.12
SBDS	0.00	0.00	0.00	0.22	0.00	0.32	0.11	0.22	0.19	0.00	0.00	0.12	0.00	0.00	0.18	0.00	0.00	0.00	0.12	0.00	0.11

IDI1	0.00	0.00	0.00	0.26	0.00	0.44	0.11	0.22	0.12	0.00	0.00	0.22	0.00	0.00	0.13	0.00	0.00	0.00	0.40	0.00	0.18
UACA	0.00	0.00	0.00	0.29	0.00	0.41	0.16	0.32	0.22	0.00	0.00	0.17	0.00	0.00	0.19	0.00	0.00	0.00	0.12	0.00	0.19
ATG4C	0.00	0.00	0.00	0.23	0.00	0.38	0.10	0.37	0.18	0.00	0.00	0.11	0.00	0.00	0.16	0.00	0.00	0.00	0.11	0.00	0.12
PEX7	0.00	0.00	0.15	0.30	0.00	0.36	0.18	0.00	0.00	0.00	0.13	0.19	0.00	0.00	0.22	0.00	0.00	0.00	0.22	0.00	0.17
PEX10	0.00	0.00	0.11	0.28	0.00	0.41	0.15	0.00	0.00	0.00	0.16	0.17	0.00	0.00	0.22	0.00	0.00	0.00	0.16	0.00	0.21
SIDT2	0.00	0.00	0.16	0.41	0.00	0.63	0.20	0.00	0.00	0.00	0.35	0.33	0.00	0.00	0.21	0.00	0.00	0.00	0.14	0.00	0.10
ABCB10	0.00	0.00	0.00	0.29	0.00	0.50	0.11	0.14	0.00	0.00	0.21	0.21	0.00	0.00	0.17	0.00	0.00	0.00	0.19	0.00	0.31
MPV17L	0.00	0.00	0.00	0.34	0.00	0.57	0.14	0.13	0.00	0.00	0.18	0.26	0.00	0.00	0.15	0.00	0.00	0.00	0.21	0.00	0.39
PEX1	0.00	0.00	0.00	0.23	0.00	0.36	0.11	0.11	0.00	0.00	0.13	0.15	0.00	0.00	0.20	0.00	0.00	0.00	0.20	0.00	0.18
XPNPEP3	0.00	0.00	0.00	0.32	0.00	0.59	0.12	0.17	0.00	0.00	0.16	0.27	0.00	0.00	0.16	0.00	0.00	0.00	0.20	0.00	0.27
PSENEN	0.00	0.00	0.00	0.18	0.00	0.23	0.00	0.16	0.10	0.00	0.19	0.15	0.00	0.00	0.12	0.00	0.00	0.00	0.12	0.00	0.10
PTDSS2	0.00	0.00	0.00	0.25	0.00	0.42	0.00	0.19	0.00	0.00	0.12	0.27	0.11	0.00	0.20	0.00	0.00	0.00	0.32	0.00	0.15
HSD11B1L	0.00	0.00	0.00	0.22	0.00	0.36	0.00	0.14	0.00	0.00	0.00	0.30	0.16	0.18	0.16	0.00	0.00	0.00	0.29	0.00	0.11
RPP25	0.00	0.00	0.00	0.30	0.00	0.57	0.00	0.24	0.00	0.00	0.00	0.28	0.20	0.15	0.25	0.00	0.00	0.00	0.17	0.00	0.17
GARS	0.00	0.00	0.00	0.23	0.00	0.36	0.10	0.14	0.00	0.00	0.00	0.17	0.00	0.00	0.33	0.16	0.00	0.00	0.14	0.00	0.10
CLN9	0.00	0.00	0.00	0.22	0.00	0.43	0.00	0.27	0.24	0.00	0.00	0.30	0.00	0.00	0.23	0.16	0.00	0.00	0.22	0.00	0.13
SPG7	0.00	0.00	0.00	0.19	0.00	0.33	0.00	0.00	0.10	0.00	0.13	0.22	0.00	0.00	0.29	0.16	0.00	0.00	0.10	0.00	0.28
DARS2	0.00	0.00	0.00	0.00	0.00	0.18	0.00	0.00	0.17	0.00	0.00	0.29	0.18	0.15	0.33	0.27	0.00	0.00	0.15	0.00	0.20
C19ORF12	0.00	0.00	0.00	0.00	0.00	0.19	0.00	0.00	0.11	0.00	0.00	0.24	0.11	0.13	0.24	0.16	0.00	0.00	0.16	0.00	0.29
TOMM40	0.00	0.00	0.00	0.21	0.00	0.28	0.12	0.11	0.00	0.00	0.00	0.18	0.00	0.00	0.14	0.00	0.11	0.00	0.17	0.00	0.22
MSRB3	0.00	0.00	0.00	0.22	0.00	0.42	0.00	0.25	0.12	0.00	0.00	0.14	0.00	0.00	0.14	0.00	0.20	0.00	0.19	0.00	0.18
AOX1	0.00	0.00	0.00	0.19	0.00	0.26	0.00	0.00	0.10	0.00	0.00	0.23	0.12	0.00	0.19	0.00	0.11	0.00	0.36	0.00	0.12
RNF185	0.00	0.00	0.10	0.26	0.00	0.34	0.15	0.19	0.00	0.00	0.11	0.00	0.00	0.00	0.00	0.00	0.00	0.16	0.13	0.00	0.47
MFF	0.00	0.00	0.00	0.30	0.00	0.45	0.14	0.25	0.00	0.00	0.13	0.11	0.00	0.00	0.00	0.00	0.00	0.20	0.17	0.00	0.67
ATAD3C	0.00	0.00	0.00	0.32	0.00	0.59	0.11	0.32	0.00	0.00	0.21	0.18	0.00	0.00	0.00	0.00	0.00	0.16	0.14	0.00	0.52
NDUFA11	0.00	0.00	0.00	0.26	0.00	0.50	0.00	0.19	0.11	0.00	0.12	0.17	0.00	0.00	0.00	0.00	0.00	0.15	0.19	0.00	0.44
SLC25A48	0.00	0.00	0.00	0.30	0.00	0.55	0.00	0.26	0.15	0.00	0.14	0.21	0.00	0.00	0.00	0.00	0.00	0.20	0.26	0.00	0.45
SLC25A47	0.00	0.00	0.00	0.30	0.00	0.55	0.00	0.26	0.15	0.00	0.14	0.21	0.00	0.00	0.00	0.00	0.00	0.20	0.26	0.00	0.45
TECPR1	0.00	0.00	0.21	0.33	0.00	0.28	0.25	0.20	0.00	0.00	0.00	0.00	0.00	0.00	0.12	0.00	0.00	0.11	0.10	0.00	0.22
SQSTM1	0.00	0.00	0.00	0.17	0.00	0.13	0.12	0.22	0.18	0.00	0.00	0.00	0.00	0.00	0.17	0.00	0.00	0.12	0.11	0.00	0.12
BECN1	0.00	0.00	0.00	0.16	0.00	0.13	0.12	0.27	0.28	0.00	0.00	0.00	0.00	0.00	0.15	0.00	0.00	0.13	0.11	0.00	0.19
MED30	0.00	0.00	0.00	0.29	0.00	0.47	0.13	0.34	0.00	0.00	0.00	0.18	0.00	0.00	0.24	0.00	0.00	0.12	0.16	0.00	0.13
PUS1	0.00	0.00	0.00	0.29	0.00	0.45	0.13	0.14	0.00	0.00	0.00	0.19	0.00	0.00	0.16	0.00	0.00	0.12	0.29	0.00	0.28
AMBRA1	0.00	0.00	0.00	0.26	0.00	0.36	0.14	0.30	0.00	0.00	0.00	0.18	0.00	0.00	0.23	0.00	0.00	0.12	0.11	0.00	0.34
SCP2	0.00	0.00	0.00	0.24	0.00	0.27	0.14	0.16	0.00	0.00	0.00	0.16	0.00	0.00	0.13	0.00	0.00	0.23	0.37	0.00	0.21
CRYZ	0.00	0.00	0.00	0.25	0.00	0.35	0.12	0.25	0.00	0.00	0.00	0.17	0.00	0.00	0.12	0.00	0.00	0.12	0.28	0.00	0.17
PGAM5	0.00	0.00	0.00	0.27	0.00	0.43	0.12	0.23	0.00	0.00	0.00	0.13	0.00	0.00	0.11	0.00	0.00	0.11	0.16	0.00	0.35
ATG4B	0.00	0.00	0.00	0.23	0.00	0.34	0.11	0.22	0.00	0.00	0.00	0.17	0.00	0.00	0.19	0.00	0.00	0.11	0.16	0.00	0.15
ATG3	0.00	0.00	0.00	0.22	0.00	0.32	0.12	0.24	0.00	0.00	0.00	0.10	0.00	0.00	0.16	0.00	0.00	0.15	0.16	0.00	0.19
COQ2	0.00	0.00	0.00	0.16	0.00	0.29	0.00	0.12	0.15	0.00	0.00	0.17	0.00	0.00	0.16	0.00	0.00	0.14	0.30	0.00	0.32

NDUFAF2	0.00	0.00	0.00	0.20	0.00	0.42	0.00	0.19	0.11	0.00	0.00	0.21	0.00	0.00	0.23	0.00	0.00	0.17	0.18	0.00	0.32
PDSS1	0.00	0.00	0.00	0.17	0.00	0.28	0.00	0.14	0.18	0.00	0.00	0.18	0.00	0.00	0.11	0.00	0.00	0.19	0.43	0.00	0.37
NDUFS3	0.00	0.00	0.00	0.20	0.00	0.39	0.00	0.20	0.13	0.00	0.00	0.19	0.00	0.00	0.16	0.00	0.00	0.17	0.22	0.00	0.48
TK2	0.00	0.00	0.00	0.15	0.00	0.28	0.00	0.16	0.22	0.00	0.00	0.18	0.00	0.00	0.16	0.00	0.00	0.17	0.31	0.00	0.30
ISCU	0.00	0.00	0.00	0.23	0.00	0.39	0.00	0.23	0.12	0.00	0.00	0.16	0.00	0.00	0.17	0.00	0.00	0.19	0.29	0.00	0.41
SCO2	0.00	0.00	0.00	0.16	0.00	0.31	0.00	0.16	0.19	0.00	0.00	0.17	0.00	0.00	0.20	0.00	0.00	0.20	0.25	0.00	0.38
ETHE1	0.00	0.00	0.00	0.20	0.00	0.37	0.00	0.20	0.14	0.00	0.00	0.17	0.00	0.00	0.11	0.00	0.00	0.15	0.28	0.00	0.35
ACO2	0.00	0.00	0.00	0.18	0.00	0.29	0.00	0.22	0.12	0.00	0.00	0.17	0.00	0.00	0.14	0.00	0.00	0.19	0.27	0.00	0.38
ADCK3	0.00	0.00	0.00	0.20	0.00	0.41	0.00	0.16	0.11	0.00	0.00	0.18	0.00	0.00	0.15	0.00	0.00	0.11	0.21	0.00	0.29
MTRF1	0.00	0.00	0.00	0.27	0.00	0.52	0.00	0.29	0.13	0.00	0.00	0.14	0.00	0.00	0.14	0.00	0.00	0.13	0.16	0.00	0.40
HLCS	0.00	0.00	0.00	0.19	0.00	0.36	0.00	0.19	0.12	0.00	0.00	0.17	0.00	0.00	0.16	0.00	0.00	0.14	0.30	0.00	0.14
AGXT	0.00	0.00	0.00	0.17	0.00	0.21	0.00	0.10	0.15	0.00	0.00	0.11	0.00	0.00	0.14	0.00	0.00	0.16	0.30	0.00	0.18
BOLA3	0.00	0.00	0.00	0.29	0.00	0.49	0.11	0.00	0.00	0.00	0.13	0.15	0.00	0.00	0.13	0.00	0.00	0.13	0.21	0.00	0.36
TIMM22	0.00	0.00	0.00	0.30	0.00	0.51	0.12	0.00	0.00	0.00	0.20	0.19	0.00	0.00	0.18	0.00	0.00	0.21	0.15	0.00	0.53
TIMM9	0.00	0.00	0.00	0.28	0.00	0.47	0.11	0.00	0.00	0.00	0.19	0.17	0.00	0.00	0.18	0.00	0.00	0.21	0.17	0.00	0.53
SAMM50	0.00	0.00	0.00	0.22	0.00	0.44	0.00	0.14	0.00	0.00	0.12	0.15	0.00	0.00	0.12	0.00	0.00	0.25	0.21	0.00	0.72
MINOS1	0.00	0.00	0.00	0.27	0.00	0.51	0.00	0.11	0.00	0.00	0.18	0.18	0.00	0.00	0.17	0.00	0.00	0.20	0.16	0.00	0.64
SLC25A30	0.00	0.00	0.00	0.20	0.00	0.40	0.00	0.11	0.00	0.00	0.12	0.23	0.00	0.00	0.13	0.00	0.00	0.11	0.32	0.00	0.31
PREPL	0.00	0.00	0.00	0.23	0.00	0.48	0.00	0.18	0.00	0.00	0.17	0.25	0.00	0.00	0.23	0.00	0.00	0.11	0.17	0.00	0.15
PMPCB	0.00	0.00	0.00	0.21	0.00	0.38	0.00	0.14	0.00	0.00	0.10	0.18	0.00	0.00	0.14	0.00	0.00	0.15	0.23	0.00	0.42
CLPP	0.00	0.00	0.00	0.26	0.00	0.53	0.00	0.12	0.00	0.00	0.12	0.23	0.00	0.00	0.18	0.00	0.00	0.18	0.24	0.00	0.39
DNAJC19	0.00	0.00	0.00	0.27	0.00	0.54	0.00	0.13	0.00	0.00	0.13	0.20	0.00	0.00	0.20	0.00	0.00	0.19	0.19	0.00	0.47
ABCB6	0.00	0.00	0.00	0.27	0.00	0.48	0.00	0.15	0.00	0.00	0.22	0.23	0.00	0.00	0.16	0.00	0.00	0.14	0.25	0.00	0.24
TIMM23B	0.00	0.00	0.00	0.29	0.00	0.58	0.00	0.11	0.00	0.00	0.17	0.22	0.00	0.00	0.17	0.00	0.00	0.18	0.16	0.00	0.62
SLC25A39	0.00	0.00	0.00	0.26	0.00	0.53	0.00	0.11	0.00	0.00	0.16	0.21	0.00	0.00	0.14	0.00	0.00	0.18	0.25	0.00	0.49
SLC25A19	0.00	0.00	0.00	0.17	0.00	0.38	0.00	0.00	0.13	0.00	0.11	0.23	0.00	0.00	0.17	0.00	0.00	0.19	0.27	0.00	0.43
PNPO	0.00	0.00	0.00	0.18	0.00	0.34	0.00	0.11	0.00	0.00	0.00	0.28	0.13	0.00	0.20	0.00	0.00	0.11	0.39	0.00	0.16
COX6C	0.00	0.00	0.00	0.23	0.00	0.42	0.00	0.17	0.00	0.00	0.00	0.27	0.11	0.00	0.18	0.00	0.00	0.14	0.25	0.00	0.38
SLC25A15	0.00	0.00	0.00	0.11	0.00	0.27	0.00	0.00	0.12	0.00	0.00	0.21	0.12	0.00	0.17	0.00	0.00	0.16	0.28	0.00	0.26
HMGCL	0.00	0.00	0.00	0.15	0.00	0.22	0.00	0.00	0.12	0.00	0.00	0.21	0.10	0.00	0.15	0.00	0.00	0.23	0.42	0.00	0.24
ASL	0.00	0.00	0.00	0.15	0.00	0.21	0.00	0.00	0.13	0.00	0.00	0.25	0.16	0.00	0.20	0.00	0.00	0.14	0.29	0.00	0.13
SUOX	0.00	0.00	0.00	0.13	0.00	0.23	0.00	0.00	0.13	0.00	0.00	0.21	0.13	0.00	0.17	0.00	0.00	0.18	0.36	0.00	0.28
NDUFV1	0.00	0.00	0.00	0.20	0.00	0.43	0.00	0.00	0.00	0.00	0.00	0.27	0.12	0.00	0.23	0.12	0.00	0.18	0.23	0.00	0.41
GNPAT	0.00	0.00	0.00	0.22	0.00	0.34	0.00	0.00	0.00	0.00	0.00	0.30	0.17	0.00	0.24	0.11	0.00	0.17	0.41	0.00	0.27
MRPL43	0.00	0.00	0.00	0.22	0.00	0.46	0.00	0.19	0.00	0.00	0.00	0.13	0.00	0.00	0.13	0.00	0.10	0.18	0.16	0.00	0.47
APOPT1	0.00	0.00	0.13	0.28	0.00	0.33	0.19	0.26	0.18	0.00	0.10	0.00	0.00	0.00	0.00	0.00	0.00	0.00	0.00	0.11	0.47
GAPDHS	0.00	0.00	0.00	0.22	0.00	0.37	0.00	0.15	0.00	0.00	0.00	0.20	0.00	0.00	0.14	0.00	0.00	0.17	0.22	0.11	0.18
PINK1-AS	0.00	0.00	0.00	0.24	0.00	0.41	0.00	0.20	0.00	0.00	0.00	0.23	0.00	0.00	0.14	0.00	0.00	0.22	0.19	0.10	0.46
OGDHL	0.00	0.00	0.00	0.16	0.00	0.28	0.00	0.00	0.00	0.00	0.00	0.27	0.11	0.00	0.12	0.00	0.00	0.34	0.45	0.18	0.54
GLYCTK	0.00	0.00	0.00	0.21	0.00	0.39	0.00	0.00	0.00	0.00	0.00	0.30	0.10	0.00	0.17	0.00	0.00	0.16	0.45	0.15	0.22

PPARGC1A	0.00	0.00	0.00	0.13	0.00	0.00	0.10	0.13	0.15	0.00	0.00	0.00	0.00	0.00	0.00	0.00	0.12	0.22	0.14	0.10	0.11
NDUFB6	0.00	0.00	0.00	0.17	0.00	0.35	0.00	0.13	0.00	0.00	0.00	0.13	0.00	0.00	0.00	0.00	0.32	0.40	0.41	0.35	0.39
PARL	0.00	0.00	0.00	0.19	0.00	0.29	0.00	0.15	0.00	0.00	0.00	0.00	0.00	0.00	0.10	0.00	0.26	0.20	0.21	0.23	0.41
ND3	0.00	0.00	0.00	0.11	0.00	0.18	0.00	0.00	0.00	0.00	0.00	0.13	0.00	0.00	0.13	0.00	0.22	0.20	0.20	0.13	0.35
GSTK1	0.00	0.00	0.00	0.22	0.00	0.35	0.00	0.00	0.00	0.00	0.00	0.19	0.00	0.00	0.13	0.00	0.19	0.16	0.32	0.20	0.18
HADHA	0.00	0.00	0.00	0.14	0.00	0.22	0.00	0.00	0.00	0.00	0.00	0.16	0.00	0.00	0.11	0.00	0.10	0.26	0.39	0.16	0.30
REP15	0.10	0.10	0.35	0.47	0.00	0.27	0.42	0.19	0.00	0.00	0.22	0.00	0.00	0.00	0.00	0.00	0.00	0.00	0.00	0.00	0.00
HPYR1	0.00	0.00	0.17	0.34	0.00	0.45	0.21	0.22	0.11	0.00	0.14	0.11	0.00	0.00	0.00	0.00	0.00	0.00	0.00	0.00	0.00
TAS2R3	0.14	0.14	0.40	0.43	0.00	0.15	0.40	0.00	0.00	0.00	0.22	0.00	0.00	0.00	0.14	0.00	0.00	0.00	0.00	0.00	0.00
EDEM1	0.00	0.00	0.13	0.27	0.00	0.31	0.16	0.23	0.15	0.00	0.28	0.00	0.00	0.00	0.13	0.00	0.00	0.00	0.00	0.00	0.00
EDA	0.00	0.00	0.13	0.22	0.00	0.13	0.19	0.13	0.12	0.00	0.10	0.00	0.00	0.00	0.17	0.00	0.00	0.00	0.00	0.00	0.00
DOK7	0.00	0.00	0.13	0.24	0.00	0.17	0.20	0.13	0.16	0.00	0.12	0.00	0.00	0.00	0.23	0.00	0.00	0.00	0.00	0.00	0.00
CHRNA1	0.00	0.00	0.13	0.24	0.00	0.12	0.21	0.11	0.11	0.00	0.17	0.00	0.00	0.00	0.20	0.00	0.00	0.00	0.00	0.00	0.00
CD1B	0.00	0.00	0.16	0.27	0.00	0.19	0.23	0.17	0.11	0.00	0.17	0.00	0.00	0.00	0.15	0.00	0.00	0.00	0.00	0.00	0.00
PKHD1	0.00	0.00	0.12	0.21	0.00	0.22	0.14	0.16	0.20	0.00	0.15	0.00	0.00	0.00	0.12	0.00	0.00	0.00	0.00	0.00	0.00
GAB2	0.00	0.00	0.15	0.23	0.00	0.16	0.19	0.23	0.21	0.00	0.11	0.00	0.00	0.00	0.12	0.00	0.00	0.00	0.00	0.00	0.00
TTC7A	0.00	0.00	0.13	0.25	0.00	0.22	0.20	0.15	0.20	0.00	0.10	0.00	0.00	0.00	0.18	0.00	0.00	0.00	0.00	0.00	0.00
CD1C	0.00	0.00	0.17	0.30	0.00	0.24	0.24	0.20	0.13	0.00	0.18	0.00	0.00	0.00	0.16	0.00	0.00	0.00	0.00	0.00	0.00
MFGE8	0.00	0.00	0.11	0.23	0.00	0.20	0.17	0.20	0.24	0.00	0.13	0.00	0.00	0.00	0.16	0.00	0.00	0.00	0.00	0.00	0.00
LMAN1	0.00	0.00	0.12	0.24	0.00	0.24	0.16	0.15	0.12	0.00	0.18	0.00	0.00	0.00	0.16	0.00	0.00	0.00	0.00	0.00	0.00
ACVRL1	0.00	0.00	0.16	0.23	0.00	0.14	0.20	0.14	0.19	0.00	0.13	0.00	0.00	0.00	0.18	0.00	0.00	0.00	0.00	0.00	0.00
RAB7B	0.00	0.00	0.20	0.37	0.00	0.36	0.27	0.27	0.15	0.00	0.11	0.00	0.00	0.00	0.10	0.00	0.00	0.00	0.00	0.00	0.00
UPK3A	0.00	0.00	0.11	0.26	0.00	0.32	0.15	0.22	0.23	0.00	0.18	0.00	0.00	0.00	0.14	0.00	0.00	0.00	0.00	0.00	0.00
EDAR	0.00	0.00	0.14	0.25	0.00	0.18	0.20	0.19	0.11	0.00	0.11	0.00	0.00	0.00	0.18	0.00	0.00	0.00	0.00	0.00	0.00
TMEM185A	0.13	0.13	0.19	0.26	0.00	0.26	0.17	0.00	0.00	0.00	0.00	0.16	0.00	0.00	0.15	0.00	0.00	0.00	0.00	0.00	0.00
RNF11	0.00	0.00	0.18	0.36	0.00	0.38	0.25	0.36	0.15	0.00	0.00	0.15	0.00	0.00	0.17	0.00	0.00	0.00	0.00	0.00	0.00
CTSZ	0.00	0.00	0.11	0.25	0.00	0.33	0.15	0.24	0.18	0.00	0.00	0.17	0.00	0.00	0.18	0.00	0.00	0.00	0.00	0.00	0.00
TP53BP2	0.00	0.00	0.12	0.27	0.00	0.33	0.17	0.39	0.17	0.00	0.00	0.12	0.00	0.00	0.16	0.00	0.00	0.00	0.00	0.00	0.00
HDAC6	0.00	0.00	0.12	0.20	0.00	0.15	0.14	0.30	0.30	0.00	0.00	0.13	0.00	0.00	0.16	0.00	0.00	0.00	0.00	0.00	0.00
NBR1	0.00	0.00	0.17	0.34	0.00	0.38	0.22	0.30	0.10	0.00	0.00	0.11	0.00	0.00	0.19	0.00	0.00	0.00	0.00	0.00	0.00
PSMD4	0.00	0.00	0.13	0.29	0.00	0.32	0.17	0.25	0.16	0.00	0.00	0.13	0.00	0.00	0.21	0.00	0.00	0.00	0.00	0.00	0.00
ARHGAP26	0.00	0.00	0.16	0.31	0.00	0.38	0.19	0.26	0.13	0.00	0.00	0.22	0.00	0.00	0.22	0.00	0.00	0.00	0.00	0.00	0.00
MAP3K10	0.00	0.00	0.19	0.35	0.00	0.36	0.23	0.27	0.12	0.00	0.00	0.21	0.00	0.00	0.21	0.00	0.00	0.00	0.00	0.00	0.00
AGAP3	0.00	0.00	0.17	0.34	0.00	0.43	0.20	0.28	0.11	0.00	0.00	0.17	0.00	0.00	0.16	0.00	0.00	0.00	0.00	0.00	0.00
RAB7A	0.00	0.00	0.12	0.24	0.00	0.24	0.16	0.22	0.15	0.00	0.00	0.13	0.00	0.00	0.16	0.00	0.00	0.00	0.00	0.00	0.00
SLTM	0.00	0.00	0.14	0.35	0.00	0.50	0.19	0.34	0.11	0.00	0.00	0.18	0.00	0.00	0.19	0.00	0.00	0.00	0.00	0.00	0.00
TIAF1	0.00	0.00	0.12	0.31	0.00	0.37	0.20	0.31	0.20	0.00	0.00	0.12	0.00	0.00	0.11	0.00	0.00	0.00	0.00	0.00	0.00
BIN1	0.00	0.00	0.12	0.25	0.00	0.23	0.17	0.24	0.18	0.00	0.00	0.21	0.00	0.00	0.18	0.00	0.00	0.00	0.00	0.00	0.00
RNASEH2B	0.00	0.00	0.12	0.29	0.00	0.40	0.15	0.20	0.11	0.00	0.00	0.10	0.00	0.00	0.20	0.00	0.00	0.00	0.00	0.00	0.00
PSMD8	0.00	0.00	0.13	0.33	0.00	0.43	0.18	0.26	0.11	0.00	0.00	0.15	0.00	0.00	0.20	0.00	0.00	0.00	0.00	0.00	0.00

GTF2I	0.00	0.00	0.13	0.27	0.00	0.27	0.17	0.33	0.10	0.00	0.00	0.13	0.00	0.00	0.20	0.00	0.00	0.00	0.00	0.00	0.00
DDX20	0.00	0.00	0.12	0.29	0.00	0.37	0.17	0.34	0.15	0.00	0.00	0.10	0.00	0.00	0.24	0.00	0.00	0.00	0.00	0.00	0.00
SIAH2	0.00	0.00	0.11	0.23	0.00	0.25	0.16	0.38	0.21	0.00	0.00	0.14	0.00	0.00	0.19	0.00	0.00	0.00	0.00	0.00	0.00
PSME3	0.00	0.00	0.10	0.28	0.00	0.35	0.15	0.36	0.19	0.00	0.00	0.14	0.00	0.00	0.19	0.00	0.00	0.00	0.00	0.00	0.00
UBE2L6	0.00	0.00	0.13	0.30	0.00	0.35	0.19	0.38	0.14	0.00	0.00	0.14	0.00	0.00	0.18	0.00	0.00	0.00	0.00	0.00	0.00
MIR29B1	0.00	0.00	0.11	0.24	0.00	0.28	0.15	0.48	0.37	0.00	0.00	0.15	0.00	0.00	0.13	0.00	0.00	0.00	0.00	0.00	0.00
UBE2L3	0.00	0.00	0.13	0.27	0.00	0.27	0.18	0.29	0.11	0.00	0.00	0.13	0.00	0.00	0.17	0.00	0.00	0.00	0.00	0.00	0.00
UBE2M	0.00	0.00	0.17	0.33	0.00	0.33	0.22	0.30	0.11	0.00	0.00	0.17	0.00	0.00	0.22	0.00	0.00	0.00	0.00	0.00	0.00
HSPA6	0.00	0.00	0.12	0.29	0.00	0.40	0.16	0.21	0.14	0.00	0.00	0.16	0.00	0.00	0.18	0.00	0.00	0.00	0.00	0.00	0.00
MYOZ2	0.00	0.00	0.10	0.24	0.00	0.32	0.14	0.18	0.14	0.00	0.00	0.14	0.00	0.00	0.17	0.00	0.00	0.00	0.00	0.00	0.00
CDK11B	0.00	0.00	0.12	0.31	0.00	0.39	0.18	0.34	0.11	0.00	0.00	0.14	0.00	0.00	0.19	0.00	0.00	0.00	0.00	0.00	0.00
TGIF1	0.00	0.00	0.13	0.25	0.00	0.22	0.18	0.29	0.13	0.00	0.00	0.13	0.00	0.00	0.20	0.00	0.00	0.00	0.00	0.00	0.00
EFEMP1	0.00	0.00	0.11	0.21	0.00	0.24	0.13	0.21	0.26	0.00	0.00	0.10	0.00	0.00	0.19	0.00	0.00	0.00	0.00	0.00	0.00
UQCC	0.00	0.00	0.12	0.26	0.00	0.29	0.16	0.33	0.32	0.00	0.00	0.15	0.00	0.00	0.19	0.00	0.00	0.00	0.00	0.00	0.00
TRIM32	0.00	0.00	0.11	0.27	0.00	0.33	0.16	0.30	0.20	0.00	0.00	0.13	0.00	0.00	0.21	0.00	0.00	0.00	0.00	0.00	0.00
SET	0.00	0.00	0.16	0.28	0.00	0.24	0.19	0.41	0.20	0.00	0.00	0.12	0.00	0.00	0.16	0.00	0.00	0.00	0.00	0.00	0.00
AATF	0.00	0.00	0.13	0.31	0.00	0.37	0.19	0.45	0.22	0.00	0.00	0.16	0.00	0.00	0.18	0.00	0.00	0.00	0.00	0.00	0.00
MIR132	0.00	0.00	0.15	0.28	0.00	0.26	0.20	0.55	0.37	0.00	0.00	0.13	0.00	0.00	0.17	0.00	0.00	0.00	0.00	0.00	0.00
NEDD9	0.00	0.00	0.16	0.26	0.00	0.24	0.19	0.26	0.16	0.00	0.00	0.12	0.00	0.00	0.19	0.00	0.00	0.00	0.00	0.00	0.00
GUCA1A	0.00	0.00	0.16	0.22	0.00	0.20	0.14	0.13	0.11	0.00	0.00	0.13	0.00	0.00	0.19	0.00	0.00	0.00	0.00	0.00	0.00
HIST1H4C	0.00	0.00	0.11	0.26	0.00	0.31	0.15	0.37	0.14	0.00	0.00	0.11	0.00	0.00	0.15	0.00	0.00	0.00	0.00	0.00	0.00
FAM72A	0.00	0.00	0.13	0.35	0.00	0.49	0.18	0.41	0.25	0.00	0.00	0.21	0.00	0.00	0.16	0.00	0.00	0.00	0.00	0.00	0.00
PSMA7	0.00	0.00	0.10	0.30	0.00	0.41	0.17	0.34	0.15	0.00	0.00	0.11	0.00	0.00	0.17	0.00	0.00	0.00	0.00	0.00	0.00
GAK	0.00	0.00	0.19	0.34	0.00	0.34	0.25	0.26	0.16	0.00	0.00	0.19	0.00	0.00	0.17	0.00	0.00	0.00	0.00	0.00	0.00
ARHGEF6	0.00	0.00	0.20	0.31	0.00	0.30	0.21	0.21	0.12	0.00	0.00	0.19	0.00	0.00	0.21	0.00	0.00	0.00	0.00	0.00	0.00
RBM14	0.00	0.00	0.15	0.36	0.00	0.42	0.23	0.48	0.13	0.00	0.00	0.17	0.00	0.00	0.21	0.00	0.00	0.00	0.00	0.00	0.00
NAB2	0.00	0.00	0.11	0.28	0.00	0.32	0.18	0.40	0.15	0.00	0.00	0.11	0.00	0.00	0.23	0.00	0.00	0.00	0.00	0.00	0.00
CSNK1A1	0.00	0.00	0.13	0.25	0.00	0.27	0.15	0.30	0.16	0.00	0.00	0.13	0.00	0.00	0.14	0.00	0.00	0.00	0.00	0.00	0.00
CARD16	0.00	0.00	0.15	0.35	0.00	0.39	0.23	0.25	0.20	0.00	0.00	0.13	0.00	0.00	0.15	0.00	0.00	0.00	0.00	0.00	0.00
NEK8	0.00	0.00	0.12	0.22	0.00	0.25	0.14	0.18	0.24	0.00	0.00	0.10	0.00	0.00	0.14	0.00	0.00	0.00	0.00	0.00	0.00
PPME1	0.00	0.00	0.12	0.31	0.00	0.45	0.15	0.39	0.15	0.00	0.00	0.23	0.00	0.00	0.16	0.00	0.00	0.00	0.00	0.00	0.00
PRKRA	0.00	0.00	0.11	0.26	0.00	0.33	0.16	0.33	0.14	0.00	0.00	0.11	0.00	0.00	0.21	0.00	0.00	0.00	0.00	0.00	0.00
SHOC2	0.00	0.00	0.21	0.35	0.00	0.35	0.24	0.26	0.13	0.00	0.00	0.13	0.00	0.00	0.17	0.00	0.00	0.00	0.00	0.00	0.00
APCS	0.00	0.00	0.11	0.22	0.00	0.19	0.16	0.16	0.21	0.00	0.00	0.10	0.00	0.00	0.17	0.00	0.00	0.00	0.00	0.00	0.00
STUB1	0.00	0.00	0.12	0.20	0.00	0.16	0.15	0.23	0.22	0.00	0.00	0.10	0.00	0.00	0.15	0.00	0.00	0.00	0.00	0.00	0.00
PKN1	0.00	0.00	0.14	0.25	0.00	0.27	0.15	0.26	0.13	0.00	0.00	0.15	0.00	0.00	0.15	0.00	0.00	0.00	0.00	0.00	0.00
SAMD9	0.00	0.00	0.13	0.32	0.00	0.42	0.19	0.33	0.26	0.00	0.00	0.11	0.00	0.00	0.18	0.00	0.00	0.00	0.00	0.00	0.00
GORAB	0.00	0.00	0.17	0.34	0.00	0.42	0.21	0.28	0.16	0.00	0.00	0.23	0.00	0.00	0.24	0.00	0.00	0.00	0.00	0.00	0.00
C4BPA	0.00	0.00	0.12	0.26	0.00	0.26	0.18	0.12	0.12	0.00	0.00	0.10	0.00	0.00	0.17	0.00	0.00	0.00	0.00	0.00	0.00
RPS6KA6	0.00	0.00	0.14	0.29	0.00	0.33	0.18	0.40	0.24	0.00	0.00	0.18	0.00	0.00	0.16	0.00	0.00	0.00	0.00	0.00	0.00

CAPN7	0.00	0.00	0.10	0.28	0.00	0.43	0.13	0.20	0.10	0.00	0.00	0.24	0.00	0.00	0.23	0.00	0.00	0.00	0.00	0.00	0.00
HSP90AB2P	0.00	0.00	0.14	0.27	0.00	0.27	0.20	0.28	0.25	0.00	0.00	0.16	0.00	0.00	0.16	0.00	0.00	0.00	0.00	0.00	0.00
MAK	0.00	0.00	0.17	0.36	0.00	0.45	0.21	0.23	0.11	0.00	0.00	0.15	0.00	0.00	0.18	0.00	0.00	0.00	0.00	0.00	0.00
MAP3K6	0.00	0.00	0.12	0.26	0.00	0.34	0.15	0.29	0.20	0.00	0.00	0.10	0.00	0.00	0.13	0.00	0.00	0.00	0.00	0.00	0.00
C14ORF129	0.00	0.00	0.00	0.22	0.00	0.34	0.12	0.34	0.12	0.12	0.00	0.14	0.00	0.00	0.15	0.00	0.00	0.00	0.00	0.00	0.00
DOK6	0.00	0.00	0.28	0.44	0.00	0.39	0.35	0.23	0.00	0.00	0.15	0.21	0.00	0.00	0.32	0.00	0.00	0.00	0.00	0.00	0.00
RANGRF	0.00	0.00	0.13	0.30	0.00	0.41	0.15	0.23	0.00	0.00	0.11	0.13	0.00	0.00	0.19	0.00	0.00	0.00	0.00	0.00	0.00
TRPC4AP	0.00	0.00	0.21	0.37	0.00	0.39	0.24	0.20	0.00	0.00	0.22	0.15	0.00	0.00	0.16	0.00	0.00	0.00	0.00	0.00	0.00
PIRT	0.00	0.00	0.17	0.28	0.00	0.23	0.22	0.22	0.00	0.00	0.11	0.12	0.00	0.00	0.22	0.00	0.00	0.00	0.00	0.00	0.00
OTOL1	0.00	0.00	0.13	0.34	0.00	0.49	0.18	0.21	0.00	0.00	0.17	0.15	0.00	0.00	0.25	0.00	0.00	0.00	0.00	0.00	0.00
CHMP4C	0.00	0.00	0.11	0.30	0.00	0.48	0.15	0.21	0.00	0.00	0.13	0.20	0.00	0.00	0.21	0.00	0.00	0.00	0.00	0.00	0.00
STX8	0.00	0.00	0.15	0.33	0.00	0.37	0.21	0.25	0.00	0.00	0.25	0.13	0.00	0.00	0.17	0.00	0.00	0.00	0.00	0.00	0.00
VTI1B	0.00	0.00	0.16	0.33	0.00	0.32	0.23	0.25	0.00	0.00	0.21	0.13	0.00	0.00	0.19	0.00	0.00	0.00	0.00	0.00	0.00
SCFD1	0.00	0.00	0.14	0.35	0.00	0.47	0.19	0.28	0.00	0.00	0.21	0.19	0.00	0.00	0.23	0.00	0.00	0.00	0.00	0.00	0.00
ANKFY1	0.00	0.00	0.13	0.36	0.00	0.56	0.18	0.13	0.00	0.00	0.19	0.26	0.00	0.00	0.23	0.00	0.00	0.00	0.00	0.00	0.00
FAM160A2	0.00	0.00	0.11	0.36	0.00	0.60	0.17	0.28	0.00	0.00	0.14	0.11	0.00	0.00	0.13	0.00	0.00	0.00	0.00	0.00	0.00
HOOK2	0.00	0.00	0.12	0.33	0.00	0.49	0.16	0.22	0.00	0.00	0.14	0.16	0.00	0.00	0.20	0.00	0.00	0.00	0.00	0.00	0.00
CCDC91	0.00	0.00	0.14	0.28	0.00	0.29	0.20	0.10	0.00	0.00	0.11	0.13	0.00	0.00	0.10	0.00	0.00	0.00	0.00	0.00	0.00
ERVFRD-1	0.00	0.00	0.12	0.31	0.00	0.42	0.16	0.26	0.00	0.00	0.18	0.21	0.00	0.00	0.30	0.00	0.00	0.00	0.00	0.00	0.00
VPS33B	0.00	0.00	0.13	0.32	0.00	0.40	0.19	0.19	0.00	0.00	0.18	0.15	0.00	0.00	0.19	0.00	0.00	0.00	0.00	0.00	0.00
GGA2	0.00	0.00	0.18	0.31	0.00	0.27	0.23	0.16	0.00	0.00	0.15	0.12	0.00	0.00	0.11	0.00	0.00	0.00	0.00	0.00	0.00
MMRN1	0.00	0.00	0.12	0.27	0.00	0.32	0.16	0.13	0.00	0.00	0.12	0.11	0.00	0.00	0.18	0.00	0.00	0.00	0.00	0.00	0.00
TMIE	0.00	0.00	0.14	0.27	0.00	0.31	0.17	0.11	0.00	0.00	0.16	0.12	0.00	0.00	0.17	0.00	0.00	0.00	0.00	0.00	0.00
TFG	0.00	0.00	0.18	0.37	0.00	0.43	0.24	0.21	0.00	0.00	0.13	0.18	0.00	0.00	0.22	0.00	0.00	0.00	0.00	0.00	0.00
NGLY1	0.00	0.00	0.11	0.30	0.00	0.42	0.15	0.19	0.00	0.00	0.21	0.13	0.00	0.00	0.16	0.00	0.00	0.00	0.00	0.00	0.00
SCYL2	0.00	0.00	0.29	0.44	0.00	0.33	0.36	0.26	0.00	0.00	0.19	0.18	0.00	0.00	0.22	0.00	0.00	0.00	0.00	0.00	0.00
PLEKHM1	0.00	0.00	0.13	0.31	0.00	0.42	0.17	0.17	0.00	0.00	0.15	0.15	0.00	0.00	0.15	0.00	0.00	0.00	0.00	0.00	0.00
STON2	0.00	0.00	0.19	0.33	0.00	0.28	0.26	0.11	0.00	0.00	0.12	0.20	0.00	0.00	0.20	0.00	0.00	0.00	0.00	0.00	0.00
OC90	0.00	0.00	0.15	0.33	0.00	0.38	0.20	0.21	0.00	0.00	0.16	0.15	0.00	0.00	0.28	0.00	0.00	0.00	0.00	0.00	0.00
VPS18	0.00	0.00	0.15	0.36	0.00	0.47	0.21	0.30	0.00	0.00	0.13	0.12	0.00	0.00	0.17	0.00	0.00	0.00	0.00	0.00	0.00
SNX7	0.00	0.00	0.18	0.34	0.00	0.46	0.18	0.18	0.00	0.00	0.11	0.15	0.00	0.00	0.13	0.00	0.00	0.00	0.00	0.00	0.00
STX10	0.00	0.00	0.18	0.36	0.00	0.42	0.23	0.20	0.00	0.00	0.23	0.12	0.00	0.00	0.13	0.00	0.00	0.00	0.00	0.00	0.00
MYO1E	0.00	0.00	0.15	0.35	0.00	0.47	0.20	0.18	0.00	0.00	0.14	0.18	0.00	0.00	0.18	0.00	0.00	0.00	0.00	0.00	0.00
YOD1	0.00	0.00	0.17	0.39	0.00	0.54	0.22	0.23	0.00	0.00	0.22	0.15	0.00	0.00	0.14	0.00	0.00	0.00	0.00	0.00	0.00
ATP8B2	0.00	0.00	0.13	0.37	0.00	0.57	0.18	0.26	0.00	0.00	0.17	0.20	0.00	0.00	0.21	0.00	0.00	0.00	0.00	0.00	0.00
UBQLN2	0.00	0.00	0.20	0.36	0.00	0.40	0.23	0.27	0.00	0.00	0.17	0.13	0.00	0.00	0.19	0.00	0.00	0.00	0.00	0.00	0.00
KAL1	0.00	0.00	0.15	0.22	0.00	0.22	0.15	0.10	0.00	0.00	0.11	0.17	0.00	0.00	0.27	0.00	0.00	0.00	0.00	0.00	0.00
STX7	0.00	0.00	0.19	0.36	0.00	0.34	0.25	0.28	0.00	0.00	0.22	0.13	0.00	0.00	0.20	0.00	0.00	0.00	0.00	0.00	0.00
UBE2J1	0.00	0.00	0.20	0.39	0.00	0.43	0.26	0.24	0.00	0.00	0.23	0.12	0.00	0.00	0.14	0.00	0.00	0.00	0.00	0.00	0.00
FYCO1	0.00	0.00	0.12	0.35	0.00	0.58	0.15	0.16	0.00	0.00	0.15	0.27	0.00	0.00	0.31	0.00	0.00	0.00	0.00	0.00	0.00

FGF12	0.00	0.00	0.19	0.39	0.00	0.43	0.25	0.26	0.00	0.00	0.17	0.28	0.00	0.00	0.34	0.00	0.00	0.00	0.00	0.00	0.00
RER1	0.00	0.00	0.25	0.45	0.00	0.48	0.31	0.16	0.00	0.00	0.38	0.15	0.00	0.00	0.15	0.00	0.00	0.00	0.00	0.00	0.00
PALM2-AKAP2	0.00	0.00	0.11	0.32	0.00	0.52	0.14	0.15	0.00	0.00	0.14	0.26	0.00	0.00	0.20	0.00	0.00	0.00	0.00	0.00	0.00
NIPAL3	0.00	0.00	0.20	0.41	0.00	0.52	0.26	0.17	0.00	0.00	0.21	0.28	0.00	0.00	0.32	0.00	0.00	0.00	0.00	0.00	0.00
SNX17	0.00	0.00	0.24	0.41	0.00	0.38	0.31	0.22	0.00	0.00	0.24	0.20	0.00	0.00	0.16	0.00	0.00	0.00	0.00	0.00	0.00
SPPL3	0.00	0.00	0.14	0.29	0.00	0.41	0.16	0.16	0.00	0.00	0.30	0.19	0.00	0.00	0.17	0.00	0.00	0.00	0.00	0.00	0.00
COPG2	0.00	0.00	0.17	0.35	0.00	0.44	0.20	0.11	0.00	0.00	0.18	0.20	0.00	0.00	0.25	0.00	0.00	0.00	0.00	0.00	0.00
UBXN6	0.00	0.00	0.14	0.34	0.00	0.49	0.18	0.25	0.00	0.00	0.21	0.13	0.00	0.00	0.18	0.00	0.00	0.00	0.00	0.00	0.00
SPPL2A	0.00	0.00	0.19	0.33	0.00	0.35	0.23	0.17	0.00	0.00	0.34	0.14	0.00	0.00	0.14	0.00	0.00	0.00	0.00	0.00	0.00
PLEKHB2	0.00	0.00	0.19	0.42	0.00	0.53	0.24	0.15	0.00	0.00	0.28	0.23	0.00	0.00	0.22	0.00	0.00	0.00	0.00	0.00	0.00
WAC	0.00	0.00	0.12	0.37	0.00	0.55	0.18	0.35	0.00	0.00	0.13	0.11	0.00	0.00	0.15	0.00	0.00	0.00	0.00	0.00	0.00
NSFL1C	0.00	0.00	0.14	0.34	0.00	0.44	0.20	0.29	0.00	0.00	0.17	0.12	0.00	0.00	0.19	0.00	0.00	0.00	0.00	0.00	0.00
FLNB	0.00	0.00	0.16	0.27	0.00	0.29	0.19	0.15	0.00	0.00	0.15	0.16	0.00	0.00	0.22	0.00	0.00	0.00	0.00	0.00	0.00
LRSAM1	0.00	0.00	0.18	0.40	0.00	0.48	0.25	0.32	0.00	0.00	0.17	0.12	0.00	0.00	0.22	0.00	0.00	0.00	0.00	0.00	0.00
ALS2CL	0.00	0.00	0.12	0.30	0.00	0.44	0.16	0.22	0.00	0.00	0.13	0.14	0.00	0.00	0.18	0.00	0.00	0.00	0.00	0.00	0.00
AP1S2	0.00	0.00	0.16	0.30	0.00	0.30	0.22	0.12	0.00	0.00	0.16	0.19	0.00	0.00	0.19	0.00	0.00	0.00	0.00	0.00	0.00
TULP2	0.00	0.00	0.11	0.35	0.00	0.57	0.15	0.14	0.00	0.00	0.12	0.27	0.00	0.00	0.25	0.00	0.00	0.00	0.00	0.00	0.00
COG5	0.00	0.00	0.13	0.32	0.00	0.48	0.16	0.17	0.00	0.00	0.19	0.17	0.00	0.00	0.19	0.00	0.00	0.00	0.00	0.00	0.00
UFD1L	0.00	0.00	0.14	0.29	0.00	0.37	0.15	0.24	0.00	0.00	0.14	0.14	0.00	0.00	0.19	0.00	0.00	0.00	0.00	0.00	0.00
TMEM192	0.00	0.00	0.14	0.35	0.00	0.52	0.19	0.12	0.00	0.00	0.29	0.24	0.00	0.00	0.19	0.00	0.00	0.00	0.00	0.00	0.00
SCAMP3	0.00	0.00	0.20	0.39	0.00	0.41	0.26	0.15	0.00	0.00	0.20	0.19	0.00	0.00	0.18	0.00	0.00	0.00	0.00	0.00	0.00
DLG1	0.00	0.00	0.13	0.18	0.00	0.14	0.13	0.14	0.00	0.00	0.14	0.16	0.00	0.00	0.18	0.00	0.00	0.00	0.00	0.00	0.00
ZFYVE27	0.00	0.00	0.17	0.40	0.00	0.56	0.22	0.28	0.00	0.00	0.18	0.22	0.00	0.00	0.28	0.00	0.00	0.00	0.00	0.00	0.00
NOL12	0.00	0.00	0.11	0.32	0.00	0.53	0.13	0.21	0.00	0.00	0.13	0.23	0.00	0.00	0.26	0.00	0.00	0.00	0.00	0.00	0.00
ATL2	0.00	0.00	0.11	0.28	0.00	0.47	0.12	0.15	0.00	0.00	0.22	0.17	0.00	0.00	0.21	0.00	0.00	0.00	0.00	0.00	0.00
WDFY3	0.00	0.00	0.14	0.36	0.00	0.51	0.18	0.23	0.00	0.00	0.11	0.22	0.00	0.00	0.23	0.00	0.00	0.00	0.00	0.00	0.00
AAK1	0.00	0.00	0.24	0.40	0.00	0.34	0.31	0.23	0.00	0.00	0.12	0.14	0.00	0.00	0.14	0.00	0.00	0.00	0.00	0.00	0.00
ZDHHC21	0.00	0.00	0.20	0.39	0.00	0.42	0.27	0.13	0.00	0.00	0.19	0.22	0.00	0.00	0.26	0.00	0.00	0.00	0.00	0.00	0.00
RAB15	0.00	0.00	0.22	0.40	0.00	0.39	0.28	0.26	0.00	0.00	0.15	0.19	0.00	0.00	0.16	0.00	0.00	0.00	0.00	0.00	0.00
MPP1	0.00	0.00	0.16	0.33	0.00	0.44	0.19	0.19	0.00	0.00	0.20	0.22	0.00	0.00	0.25	0.00	0.00	0.00	0.00	0.00	0.00
FLNC	0.00	0.00	0.12	0.24	0.00	0.30	0.14	0.14	0.00	0.00	0.12	0.16	0.00	0.00	0.20	0.00	0.00	0.00	0.00	0.00	0.00
CNST	0.00	0.00	0.22	0.38	0.00	0.36	0.26	0.12	0.00	0.00	0.27	0.11	0.00	0.00	0.12	0.00	0.00	0.00	0.00	0.00	0.00
ASIC5	0.00	0.00	0.11	0.26	0.00	0.30	0.15	0.12	0.00	0.00	0.23	0.23	0.00	0.00	0.23	0.00	0.00	0.00	0.00	0.00	0.00
GGA3	0.00	0.00	0.17	0.30	0.00	0.29	0.22	0.22	0.00	0.00	0.14	0.12	0.00	0.00	0.11	0.00	0.00	0.00	0.00	0.00	0.00
SNX15	0.00	0.00	0.22	0.42	0.00	0.50	0.26	0.14	0.00	0.00	0.23	0.17	0.00	0.00	0.18	0.00	0.00	0.00	0.00	0.00	0.00
TECTB	0.00	0.00	0.13	0.30	0.00	0.38	0.18	0.19	0.00	0.00	0.22	0.13	0.00	0.00	0.24	0.00	0.00	0.00	0.00	0.00	0.00
WRB	0.00	0.00	0.21	0.46	0.00	0.64	0.26	0.15	0.00	0.00	0.28	0.22	0.00	0.00	0.21	0.00	0.00	0.00	0.00	0.00	0.00
VTA1	0.00	0.00	0.21	0.39	0.00	0.43	0.26	0.31	0.00	0.00	0.13	0.16	0.00	0.00	0.19	0.00	0.00	0.00	0.00	0.00	0.00
WDR45	0.00	0.00	0.26	0.49	0.00	0.57	0.32	0.36	0.00	0.00	0.16	0.14	0.00	0.00	0.16	0.00	0.00	0.00	0.00	0.00	0.00
PTCHD3	0.00	0.00	0.17	0.37	0.00	0.48	0.20	0.17	0.00	0.00	0.17	0.21	0.00	0.00	0.21	0.00	0.00	0.00	0.00	0.00	0.00

MS4A14	0.00	0.00	0.14	0.38	0.00	0.61	0.17	0.19	0.00	0.00	0.17	0.16	0.00	0.00	0.17	0.00	0.00	0.00	0.00	0.00	0.00
FAM109B	0.00	0.00	0.23	0.38	0.00	0.35	0.28	0.12	0.00	0.00	0.17	0.16	0.00	0.00	0.17	0.00	0.00	0.00	0.00	0.00	0.00
JOSD1	0.00	0.00	0.16	0.38	0.00	0.56	0.21	0.24	0.00	0.00	0.12	0.19	0.00	0.00	0.20	0.00	0.00	0.00	0.00	0.00	0.00
GCC2	0.00	0.00	0.14	0.34	0.00	0.46	0.19	0.23	0.00	0.00	0.17	0.11	0.00	0.00	0.14	0.00	0.00	0.00	0.00	0.00	0.00
HMGN4	0.00	0.00	0.12	0.42	0.00	0.76	0.15	0.16	0.00	0.00	0.14	0.23	0.00	0.00	0.16	0.00	0.00	0.00	0.00	0.00	0.00
FAM109A	0.00	0.00	0.23	0.38	0.00	0.35	0.28	0.12	0.00	0.00	0.17	0.16	0.00	0.00	0.17	0.00	0.00	0.00	0.00	0.00	0.00
WG	0.00	0.00	0.17	0.33	0.00	0.44	0.20	0.11	0.00	0.00	0.14	0.24	0.00	0.00	0.25	0.00	0.00	0.00	0.00	0.00	0.00
VPS26A	0.00	0.00	0.21	0.40	0.00	0.44	0.26	0.18	0.00	0.00	0.19	0.24	0.00	0.00	0.26	0.00	0.00	0.00	0.00	0.00	0.00
PGAP1	0.00	0.00	0.17	0.33	0.00	0.41	0.20	0.22	0.00	0.00	0.29	0.18	0.00	0.00	0.23	0.00	0.00	0.00	0.00	0.00	0.00
EHD4	0.00	0.00	0.24	0.42	0.00	0.42	0.30	0.24	0.00	0.00	0.20	0.25	0.00	0.00	0.31	0.00	0.00	0.00	0.00	0.00	0.00
PSMC3P	0.00	0.00	0.14	0.38	0.00	0.67	0.15	0.10	0.00	0.00	0.12	0.19	0.00	0.00	0.18	0.00	0.00	0.00	0.00	0.00	0.00
FOXI1	0.00	0.00	0.10	0.28	0.00	0.34	0.16	0.28	0.00	0.00	0.10	0.12	0.00	0.00	0.18	0.00	0.00	0.00	0.00	0.00	0.00
UBXN1	0.00	0.00	0.14	0.31	0.00	0.40	0.18	0.21	0.00	0.00	0.13	0.12	0.00	0.00	0.16	0.00	0.00	0.00	0.00	0.00	0.00
DNAH12	0.00	0.00	0.19	0.39	0.00	0.46	0.24	0.18	0.00	0.00	0.12	0.24	0.00	0.00	0.20	0.00	0.00	0.00	0.00	0.00	0.00
PKHD1L1	0.00	0.00	0.30	0.49	0.00	0.54	0.33	0.15	0.00	0.00	0.28	0.16	0.00	0.00	0.18	0.00	0.00	0.00	0.00	0.00	0.00
SPEG	0.00	0.00	0.12	0.31	0.00	0.45	0.16	0.20	0.00	0.00	0.16	0.24	0.00	0.00	0.24	0.00	0.00	0.00	0.00	0.00	0.00
ADAM2	0.00	0.00	0.15	0.29	0.00	0.33	0.18	0.11	0.00	0.00	0.25	0.17	0.00	0.00	0.20	0.00	0.00	0.00	0.00	0.00	0.00
GIPC3	0.00	0.00	0.19	0.32	0.00	0.37	0.21	0.11	0.00	0.00	0.21	0.16	0.00	0.00	0.18	0.00	0.00	0.00	0.00	0.00	0.00
VPS29	0.00	0.00	0.19	0.41	0.00	0.47	0.26	0.19	0.00	0.00	0.19	0.16	0.00	0.00	0.19	0.00	0.00	0.00	0.00	0.00	0.00
VPS11	0.00	0.00	0.16	0.37	0.00	0.47	0.22	0.28	0.00	0.00	0.13	0.13	0.00	0.00	0.16	0.00	0.00	0.00	0.00	0.00	0.00
CHMP4B	0.00	0.00	0.13	0.30	0.00	0.40	0.16	0.21	0.00	0.00	0.16	0.14	0.00	0.00	0.18	0.00	0.00	0.00	0.00	0.00	0.00
CHERP	0.00	0.00	0.23	0.40	0.00	0.45	0.25	0.19	0.00	0.00	0.19	0.17	0.00	0.00	0.13	0.00	0.00	0.00	0.00	0.00	0.00
RABEP1	0.00	0.00	0.18	0.35	0.00	0.36	0.24	0.29	0.00	0.00	0.12	0.20	0.00	0.00	0.19	0.00	0.00	0.00	0.00	0.00	0.00
UBQLN4	0.00	0.00	0.10	0.28	0.00	0.44	0.12	0.24	0.00	0.00	0.12	0.13	0.00	0.00	0.15	0.00	0.00	0.00	0.00	0.00	0.00
VPS16	0.00	0.00	0.17	0.38	0.00	0.49	0.22	0.28	0.00	0.00	0.15	0.21	0.00	0.00	0.25	0.00	0.00	0.00	0.00	0.00	0.00
PTPRQ	0.00	0.00	0.12	0.28	0.00	0.36	0.17	0.20	0.00	0.00	0.22	0.14	0.00	0.00	0.19	0.00	0.00	0.00	0.00	0.00	0.00
HNRNPH3	0.00	0.00	0.16	0.39	0.00	0.49	0.22	0.25	0.00	0.00	0.12	0.16	0.00	0.00	0.20	0.00	0.00	0.00	0.00	0.00	0.00
C16ORF58	0.00	0.00	0.18	0.44	0.00	0.65	0.23	0.28	0.00	0.00	0.12	0.20	0.00	0.00	0.32	0.00	0.00	0.00	0.00	0.00	0.00
FREM1	0.00	0.00	0.16	0.31	0.00	0.33	0.21	0.13	0.00	0.00	0.15	0.14	0.00	0.00	0.23	0.00	0.00	0.00	0.00	0.00	0.00
FAM20A	0.00	0.00	0.10	0.34	0.00	0.53	0.16	0.22	0.00	0.00	0.12	0.21	0.00	0.00	0.27	0.00	0.00	0.00	0.00	0.00	0.00
ILDR1	0.00	0.00	0.17	0.33	0.00	0.40	0.20	0.12	0.00	0.00	0.23	0.17	0.00	0.00	0.17	0.00	0.00	0.00	0.00	0.00	0.00
LRRC1	0.00	0.00	0.10	0.29	0.00	0.51	0.12	0.12	0.00	0.00	0.20	0.19	0.00	0.00	0.16	0.00	0.00	0.00	0.00	0.00	0.00
CDR1	0.00	0.00	0.10	0.26	0.00	0.36	0.14	0.19	0.00	0.00	0.10	0.16	0.00	0.00	0.17	0.00	0.00	0.00	0.00	0.00	0.00
REM1	0.00	0.00	0.16	0.31	0.00	0.36	0.19	0.24	0.00	0.00	0.11	0.18	0.00	0.00	0.14	0.00	0.00	0.00	0.00	0.00	0.00
LRP3	0.00	0.00	0.32	0.50	0.00	0.41	0.40	0.11	0.00	0.00	0.23	0.24	0.00	0.00	0.22	0.00	0.00	0.00	0.00	0.00	0.00
SVIP	0.00	0.00	0.13	0.29	0.00	0.41	0.15	0.21	0.00	0.00	0.22	0.11	0.00	0.00	0.12	0.00	0.00	0.00	0.00	0.00	0.00
VPS45	0.00	0.00	0.19	0.39	0.00	0.47	0.24	0.23	0.00	0.00	0.21	0.16	0.00	0.00	0.16	0.00	0.00	0.00	0.00	0.00	0.00
RIN2	0.00	0.00	0.13	0.29	0.00	0.41	0.16	0.15	0.00	0.00	0.11	0.11	0.00	0.00	0.12	0.00	0.00	0.00	0.00	0.00	0.00
GGA1	0.00	0.00	0.16	0.28	0.00	0.27	0.20	0.18	0.00	0.00	0.15	0.11	0.00	0.00	0.11	0.00	0.00	0.00	0.00	0.00	0.00
SYNE1	0.00	0.00	0.11	0.25	0.00	0.31	0.14	0.13	0.00	0.00	0.18	0.17	0.00	0.00	0.22	0.00	0.00	0.00	0.00	0.00	0.00

MYPOP	0.00	0.00	0.13	0.41	0.00	0.62	0.19	0.20	0.00	0.00	0.13	0.22	0.00	0.00	0.23	0.00	0.00	0.00	0.00	0.00	0.00
GPR98	0.00	0.00	0.15	0.23	0.00	0.23	0.16	0.00	0.12	0.00	0.17	0.15	0.00	0.00	0.24	0.00	0.00	0.00	0.00	0.00	0.00
CNGA3	0.00	0.00	0.15	0.21	0.00	0.15	0.15	0.00	0.12	0.00	0.11	0.12	0.00	0.00	0.23	0.00	0.00	0.00	0.00	0.00	0.00
KCNK17	0.00	0.00	0.00	0.21	0.00	0.27	0.11	0.12	0.10	0.00	0.15	0.17	0.00	0.00	0.14	0.00	0.00	0.00	0.00	0.00	0.00
WDR19	0.00	0.00	0.00	0.26	0.00	0.45	0.11	0.20	0.16	0.00	0.14	0.16	0.00	0.00	0.17	0.00	0.00	0.00	0.00	0.00	0.00
BRI3	0.00	0.00	0.00	0.27	0.00	0.46	0.11	0.33	0.20	0.00	0.11	0.26	0.00	0.00	0.18	0.00	0.00	0.00	0.00	0.00	0.00
ARSI	0.00	0.00	0.00	0.28	0.00	0.51	0.10	0.21	0.14	0.00	0.14	0.17	0.00	0.00	0.17	0.00	0.00	0.00	0.00	0.00	0.00
ODAM	0.00	0.00	0.00	0.25	0.00	0.39	0.12	0.16	0.12	0.00	0.13	0.14	0.00	0.00	0.13	0.00	0.00	0.00	0.00	0.00	0.00
VCPIP1	0.00	0.00	0.00	0.29	0.00	0.42	0.14	0.30	0.15	0.00	0.17	0.11	0.00	0.00	0.13	0.00	0.00	0.00	0.00	0.00	0.00
ST6GAL1	0.00	0.00	0.00	0.21	0.00	0.20	0.15	0.20	0.22	0.00	0.17	0.11	0.00	0.00	0.13	0.00	0.00	0.00	0.00	0.00	0.00
CCDC104	0.00	0.00	0.00	0.30	0.00	0.53	0.13	0.22	0.18	0.00	0.13	0.23	0.00	0.00	0.23	0.00	0.00	0.00	0.00	0.00	0.00
MATN3	0.00	0.00	0.00	0.20	0.00	0.28	0.10	0.11	0.11	0.00	0.12	0.12	0.00	0.00	0.21	0.00	0.00	0.00	0.00	0.00	0.00
ADRA2A	0.14	0.14	0.15	0.14	0.17	0.00	0.00	0.00	0.13	0.00	0.00	0.00	0.12	0.00	0.17	0.00	0.00	0.00	0.00	0.00	0.00
MSN	0.00	0.00	0.11	0.15	0.00	0.14	0.11	0.16	0.13	0.00	0.00	0.00	0.10	0.00	0.14	0.00	0.00	0.00	0.00	0.00	0.00
RFX4	0.00	0.00	0.17	0.35	0.00	0.44	0.20	0.30	0.00	0.00	0.00	0.22	0.10	0.00	0.25	0.00	0.00	0.00	0.00	0.00	0.00
MED12	0.00	0.00	0.15	0.28	0.00	0.27	0.20	0.28	0.00	0.00	0.00	0.19	0.11	0.00	0.27	0.00	0.00	0.00	0.00	0.00	0.00
HERC1	0.00	0.00	0.12	0.31	0.00	0.48	0.16	0.20	0.00	0.00	0.00	0.22	0.11	0.00	0.26	0.00	0.00	0.00	0.00	0.00	0.00
CDC42SE2	0.00	0.00	0.15	0.32	0.00	0.40	0.18	0.15	0.00	0.00	0.00	0.20	0.16	0.00	0.20	0.00	0.00	0.00	0.00	0.00	0.00
EXOC6B	0.00	0.00	0.14	0.35	0.00	0.55	0.17	0.23	0.00	0.00	0.00	0.31	0.11	0.00	0.27	0.00	0.00	0.00	0.00	0.00	0.00
OPN3	0.00	0.00	0.15	0.27	0.00	0.36	0.14	0.14	0.00	0.00	0.00	0.23	0.11	0.00	0.27	0.00	0.00	0.00	0.00	0.00	0.00
HIP1R	0.00	0.00	0.18	0.36	0.00	0.37	0.24	0.18	0.00	0.00	0.00	0.24	0.11	0.00	0.24	0.00	0.00	0.00	0.00	0.00	0.00
SH3GL2	0.00	0.00	0.23	0.37	0.00	0.30	0.28	0.19	0.00	0.00	0.00	0.24	0.11	0.00	0.21	0.00	0.00	0.00	0.00	0.00	0.00
NXF2B	0.00	0.00	0.17	0.40	0.00	0.53	0.22	0.28	0.00	0.00	0.00	0.22	0.11	0.00	0.23	0.00	0.00	0.00	0.00	0.00	0.00
DIRAS2	0.00	0.00	0.10	0.24	0.00	0.38	0.12	0.18	0.00	0.00	0.00	0.18	0.10	0.00	0.15	0.00	0.00	0.00	0.00	0.00	0.00
REEP3	0.00	0.00	0.13	0.31	0.00	0.47	0.15	0.15	0.00	0.00	0.00	0.25	0.12	0.00	0.28	0.00	0.00	0.00	0.00	0.00	0.00
GPSM2	0.00	0.00	0.12	0.26	0.00	0.38	0.11	0.28	0.00	0.00	0.00	0.20	0.19	0.00	0.26	0.00	0.00	0.00	0.00	0.00	0.00
FCHSD1	0.00	0.00	0.13	0.37	0.00	0.52	0.20	0.11	0.00	0.00	0.00	0.26	0.11	0.00	0.27	0.00	0.00	0.00	0.00	0.00	0.00
TAAR6	0.00	0.00	0.11	0.18	0.00	0.15	0.14	0.00	0.12	0.00	0.00	0.12	0.12	0.00	0.14	0.00	0.00	0.00	0.00	0.00	0.00
EDN3	0.00	0.00	0.19	0.21	0.00	0.00	0.18	0.11	0.13	0.00	0.00	0.12	0.11	0.00	0.23	0.00	0.00	0.00	0.00	0.00	0.00
ATXN1	0.00	0.00	0.00	0.18	0.00	0.18	0.12	0.18	0.22	0.00	0.00	0.18	0.12	0.00	0.21	0.00	0.00	0.00	0.00	0.00	0.00
CDK5RAP2	0.00	0.00	0.00	0.23	0.00	0.34	0.11	0.20	0.14	0.00	0.00	0.23	0.13	0.00	0.23	0.00	0.00	0.00	0.00	0.00	0.00
GTF2IRD1	0.00	0.00	0.00	0.21	0.00	0.26	0.11	0.32	0.18	0.00	0.00	0.18	0.11	0.00	0.22	0.00	0.00	0.00	0.00	0.00	0.00
USP9X	0.00	0.00	0.00	0.25	0.00	0.35	0.13	0.30	0.14	0.00	0.00	0.21	0.12	0.00	0.23	0.00	0.00	0.00	0.00	0.00	0.00
ANXA4	0.00	0.00	0.00	0.26	0.00	0.34	0.14	0.21	0.00	0.00	0.12	0.21	0.12	0.00	0.23	0.00	0.00	0.00	0.00	0.00	0.00
SDF2L1	0.00	0.00	0.00	0.26	0.00	0.43	0.11	0.11	0.00	0.00	0.17	0.25	0.10	0.00	0.14	0.00	0.00	0.00	0.00	0.00	0.00
SLC12A8	0.00	0.00	0.00	0.24	0.00	0.31	0.14	0.10	0.00	0.00	0.15	0.20	0.16	0.00	0.25	0.00	0.00	0.00	0.00	0.00	0.00
IFT20	0.00	0.00	0.00	0.23	0.00	0.35	0.10	0.16	0.00	0.00	0.10	0.24	0.14	0.00	0.25	0.00	0.00	0.00	0.00	0.00	0.00
SHROOM4	0.00	0.00	0.00	0.27	0.00	0.47	0.11	0.19	0.00	0.00	0.15	0.26	0.12	0.00	0.27	0.00	0.00	0.00	0.00	0.00	0.00
FAM120C	0.00	0.00	0.00	0.28	0.00	0.46	0.12	0.11	0.00	0.00	0.14	0.27	0.10	0.00	0.22	0.00	0.00	0.00	0.00	0.00	0.00
XK	0.00	0.00	0.00	0.23	0.00	0.33	0.10	0.00	0.11	0.00	0.19	0.21	0.10	0.00	0.23	0.00	0.00	0.00	0.00	0.00	0.00

RPS6KA5	0.00	0.00	0.15	0.24	0.00	0.18	0.18	0.29	0.20	0.00	0.00	0.00	0.00	0.13	0.12	0.00	0.00	0.00	0.00	0.00	0.00
ANKRD11	0.00	0.00	0.11	0.28	0.00	0.38	0.16	0.32	0.00	0.00	0.00	0.24	0.00	0.10	0.25	0.00	0.00	0.00	0.00	0.00	0.00
RABGAP1L	0.00	0.00	0.00	0.33	0.00	0.56	0.13	0.28	0.11	0.00	0.00	0.26	0.00	0.11	0.22	0.00	0.00	0.00	0.00	0.00	0.00
TMEM37	0.00	0.00	0.17	0.31	0.00	0.31	0.20	0.00	0.00	0.00	0.15	0.28	0.00	0.11	0.19	0.00	0.00	0.00	0.00	0.00	0.00
CACFD1	0.00	0.00	0.15	0.22	0.00	0.11	0.17	0.00	0.00	0.00	0.19	0.22	0.00	0.17	0.13	0.00	0.00	0.00	0.00	0.00	0.00
MCTP2	0.00	0.00	0.17	0.34	0.00	0.41	0.21	0.00	0.00	0.00	0.21	0.20	0.00	0.11	0.19	0.00	0.00	0.00	0.00	0.00	0.00
CACNA2D4	0.00	0.00	0.13	0.24	0.00	0.27	0.14	0.00	0.00	0.00	0.10	0.23	0.00	0.11	0.21	0.00	0.00	0.00	0.00	0.00	0.00
NKAIN4	0.00	0.00	0.00	0.30	0.00	0.51	0.13	0.14	0.00	0.00	0.23	0.27	0.00	0.11	0.27	0.00	0.00	0.00	0.00	0.00	0.00
METTL2B	0.00	0.00	0.00	0.23	0.00	0.38	0.11	0.14	0.00	0.00	0.16	0.23	0.00	0.10	0.20	0.00	0.00	0.00	0.00	0.00	0.00
EFR3A	0.00	0.00	0.00	0.33	0.00	0.58	0.13	0.19	0.00	0.00	0.17	0.28	0.00	0.12	0.25	0.00	0.00	0.00	0.00	0.00	0.00
METTL2A	0.00	0.00	0.00	0.23	0.00	0.38	0.11	0.14	0.00	0.00	0.16	0.23	0.00	0.10	0.20	0.00	0.00	0.00	0.00	0.00	0.00
KCNC4	0.00	0.00	0.00	0.20	0.00	0.32	0.00	0.19	0.18	0.00	0.12	0.24	0.00	0.13	0.21	0.00	0.00	0.00	0.00	0.00	0.00
HTR1A	0.11	0.11	0.11	0.14	0.00	0.00	0.00	0.00	0.15	0.00	0.00	0.00	0.13	0.14	0.12	0.00	0.00	0.00	0.00	0.00	0.00
HTR2A	0.10	0.10	0.00	0.11	0.18	0.00	0.00	0.00	0.11	0.00	0.00	0.00	0.12	0.11	0.11	0.00	0.00	0.00	0.00	0.00	0.00
CHRNA7	0.00	0.00	0.00	0.16	0.00	0.00	0.14	0.11	0.19	0.00	0.10	0.00	0.14	0.20	0.16	0.00	0.00	0.00	0.00	0.00	0.00
FAM155A	0.00	0.00	0.17	0.27	0.00	0.26	0.19	0.00	0.00	0.00	0.00	0.26	0.17	0.14	0.23	0.00	0.00	0.00	0.00	0.00	0.00
AUTS2	0.00	0.00	0.13	0.22	0.00	0.22	0.16	0.00	0.00	0.00	0.00	0.20	0.17	0.14	0.20	0.00	0.00	0.00	0.00	0.00	0.00
SCZD8	0.00	0.00	0.12	0.18	0.00	0.13	0.14	0.00	0.00	0.00	0.00	0.16	0.19	0.13	0.17	0.00	0.00	0.00	0.00	0.00	0.00
CHRNB3	0.00	0.00	0.13	0.22	0.00	0.12	0.20	0.00	0.00	0.00	0.00	0.15	0.13	0.13	0.19	0.00	0.00	0.00	0.00	0.00	0.00
GUSBP5	0.00	0.00	0.00	0.18	0.00	0.22	0.11	0.10	0.00	0.00	0.00	0.23	0.15	0.13	0.25	0.00	0.00	0.00	0.00	0.00	0.00
RPH3A	0.00	0.00	0.00	0.22	0.00	0.29	0.12	0.19	0.00	0.00	0.00	0.23	0.14	0.17	0.22	0.00	0.00	0.00	0.00	0.00	0.00
CDC42EP3	0.00	0.00	0.00	0.25	0.00	0.38	0.12	0.19	0.00	0.00	0.00	0.21	0.18	0.13	0.23	0.00	0.00	0.00	0.00	0.00	0.00
ADD2	0.00	0.00	0.00	0.20	0.00	0.21	0.12	0.12	0.00	0.00	0.00	0.21	0.13	0.15	0.23	0.00	0.00	0.00	0.00	0.00	0.00
PHACTR2	0.00	0.00	0.00	0.28	0.00	0.48	0.11	0.15	0.00	0.00	0.00	0.37	0.22	0.21	0.27	0.00	0.00	0.00	0.00	0.00	0.00
TSN	0.00	0.00	0.00	0.28	0.00	0.39	0.13	0.26	0.00	0.00	0.00	0.23	0.18	0.17	0.26	0.00	0.00	0.00	0.00	0.00	0.00
DBNDD1	0.00	0.00	0.00	0.25	0.00	0.33	0.13	0.20	0.00	0.00	0.00	0.34	0.26	0.23	0.32	0.00	0.00	0.00	0.00	0.00	0.00
CDK5RAP1	0.00	0.00	0.00	0.32	0.00	0.52	0.14	0.15	0.00	0.00	0.00	0.36	0.11	0.11	0.27	0.00	0.00	0.00	0.00	0.00	0.00
NXF3	0.00	0.00	0.00	0.33	0.00	0.59	0.12	0.26	0.00	0.00	0.00	0.27	0.15	0.13	0.27	0.00	0.00	0.00	0.00	0.00	0.00
BRD1	0.00	0.00	0.00	0.27	0.00	0.41	0.12	0.24	0.00	0.00	0.00	0.23	0.15	0.11	0.24	0.00	0.00	0.00	0.00	0.00	0.00
CHRNA3	0.00	0.00	0.11	0.18	0.00	0.00	0.16	0.00	0.11	0.00	0.00	0.15	0.13	0.15	0.20	0.00	0.00	0.00	0.00	0.00	0.00
CHRNA5	0.00	0.00	0.11	0.17	0.00	0.00	0.17	0.00	0.11	0.00	0.00	0.14	0.12	0.13	0.19	0.00	0.00	0.00	0.00	0.00	0.00
CDKL5	0.00	0.00	0.00	0.13	0.00	0.21	0.00	0.13	0.13	0.00	0.00	0.21	0.14	0.14	0.19	0.00	0.00	0.00	0.00	0.00	0.00
CLU	0.00	0.00	0.00	0.13	0.00	0.10	0.00	0.13	0.20	0.00	0.00	0.15	0.13	0.12	0.16	0.00	0.00	0.00	0.00	0.00	0.00
MIR103A1	0.00	0.00	0.00	0.11	0.00	0.25	0.00	0.19	0.19	0.00	0.00	0.22	0.15	0.18	0.11	0.00	0.00	0.00	0.00	0.00	0.00
COL25A1	0.00	0.00	0.00	0.19	0.00	0.26	0.00	0.10	0.15	0.00	0.00	0.24	0.15	0.19	0.20	0.00	0.00	0.00	0.00	0.00	0.00
GDNF-AS1	0.00	0.00	0.00	0.21	0.00	0.39	0.00	0.10	0.15	0.00	0.00	0.33	0.14	0.17	0.17	0.00	0.00	0.00	0.00	0.00	0.00
ZNF804A	0.00	0.00	0.00	0.16	0.00	0.17	0.00	0.11	0.12	0.00	0.00	0.17	0.20	0.17	0.17	0.00	0.00	0.00	0.00	0.00	0.00
DTNBP1	0.00	0.00	0.00	0.15	0.00	0.13	0.00	0.12	0.14	0.00	0.00	0.19	0.22	0.21	0.18	0.00	0.00	0.00	0.00	0.00	0.00
PCDH11X	0.00	0.00	0.00	0.22	0.00	0.32	0.11	0.00	0.00	0.00	0.12	0.25	0.13	0.10	0.24	0.00	0.00	0.00	0.00	0.00	0.00
CTTNBP2	0.00	0.00	0.00	0.27	0.00	0.45	0.10	0.00	0.00	0.00	0.11	0.27	0.19	0.13	0.24	0.00	0.00	0.00	0.00	0.00	0.00

JRKL	0.00	0.00	0.00	0.35	0.00	0.69	0.11	0.00	0.00	0.00	0.12	0.37	0.11	0.12	0.23	0.00	0.00	0.00	0.00	0.00	0.00
LITAF	0.00	0.00	0.13	0.28	0.00	0.32	0.18	0.26	0.21	0.00	0.00	0.00	0.00	0.00	0.21	0.10	0.00	0.00	0.00	0.00	0.00
CCL4	0.00	0.00	0.13	0.16	0.00	0.00	0.13	0.17	0.19	0.12	0.00	0.00	0.00	0.00	0.13	0.12	0.00	0.00	0.00	0.00	0.00
TRPA1	0.00	0.00	0.16	0.19	0.00	0.00	0.17	0.16	0.20	0.00	0.11	0.00	0.00	0.00	0.21	0.11	0.00	0.00	0.00	0.00	0.00
ARL13B	0.00	0.00	0.12	0.25	0.00	0.32	0.14	0.27	0.00	0.00	0.00	0.20	0.00	0.00	0.27	0.11	0.00	0.00	0.00	0.00	0.00
CX3CL1	0.00	0.00	0.12	0.17	0.00	0.00	0.13	0.16	0.20	0.00	0.00	0.11	0.00	0.00	0.19	0.19	0.00	0.00	0.00	0.00	0.00
BMP5	0.00	0.00	0.00	0.20	0.00	0.18	0.15	0.18	0.13	0.00	0.00	0.14	0.00	0.00	0.22	0.11	0.00	0.00	0.00	0.00	0.00
ZNF175	0.00	0.00	0.00	0.26	0.00	0.38	0.14	0.34	0.21	0.00	0.00	0.19	0.00	0.00	0.22	0.11	0.00	0.00	0.00	0.00	0.00
SH3TC2	0.00	0.00	0.14	0.28	0.00	0.33	0.17	0.00	0.00	0.00	0.16	0.11	0.00	0.00	0.29	0.15	0.00	0.00	0.00	0.00	0.00
GPATCH8	0.00	0.00	0.13	0.32	0.00	0.45	0.17	0.00	0.00	0.00	0.15	0.24	0.00	0.00	0.28	0.10	0.00	0.00	0.00	0.00	0.00
TMEM47	0.00	0.00	0.00	0.30	0.00	0.53	0.13	0.20	0.00	0.00	0.21	0.19	0.00	0.00	0.21	0.10	0.00	0.00	0.00	0.00	0.00
MALL	0.00	0.00	0.00	0.28	0.00	0.48	0.13	0.11	0.00	0.00	0.22	0.16	0.00	0.00	0.24	0.15	0.00	0.00	0.00	0.00	0.00
FAM126A	0.00	0.00	0.00	0.18	0.00	0.34	0.00	0.20	0.15	0.00	0.10	0.22	0.00	0.00	0.25	0.15	0.00	0.00	0.00	0.00	0.00
DLL1	0.00	0.00	0.00	0.14	0.00	0.00	0.11	0.19	0.12	0.00	0.14	0.12	0.00	0.00	0.21	0.13	0.00	0.00	0.00	0.00	0.00
FGFR3	0.00	0.00	0.00	0.14	0.00	0.00	0.12	0.14	0.17	0.00	0.12	0.00	0.10	0.00	0.12	0.10	0.00	0.00	0.00	0.00	0.00
GSC2	0.00	0.00	0.00	0.26	0.00	0.42	0.11	0.22	0.00	0.00	0.00	0.27	0.15	0.00	0.35	0.13	0.00	0.00	0.00	0.00	0.00
TSHZ3	0.00	0.00	0.00	0.25	0.00	0.39	0.12	0.34	0.00	0.00	0.00	0.22	0.14	0.00	0.29	0.11	0.00	0.00	0.00	0.00	0.00
POU6F2	0.00	0.00	0.00	0.23	0.00	0.36	0.11	0.26	0.00	0.00	0.00	0.19	0.11	0.00	0.32	0.16	0.00	0.00	0.00	0.00	0.00
SIX3	0.00	0.00	0.00	0.17	0.00	0.19	0.11	0.21	0.00	0.00	0.00	0.21	0.16	0.00	0.28	0.15	0.00	0.00	0.00	0.00	0.00
VSX2	0.00	0.00	0.00	0.16	0.00	0.14	0.11	0.26	0.00	0.00	0.00	0.15	0.17	0.00	0.34	0.22	0.00	0.00	0.00	0.00	0.00
LHX3	0.00	0.00	0.00	0.19	0.00	0.17	0.14	0.26	0.00	0.00	0.00	0.14	0.15	0.00	0.35	0.20	0.00	0.00	0.00	0.00	0.00
PRICKLE1	0.00	0.00	0.00	0.23	0.00	0.35	0.11	0.36	0.00	0.00	0.00	0.22	0.12	0.00	0.29	0.14	0.00	0.00	0.00	0.00	0.00
TSHZ2	0.00	0.00	0.00	0.25	0.00	0.40	0.11	0.35	0.00	0.00	0.00	0.22	0.14	0.00	0.29	0.11	0.00	0.00	0.00	0.00	0.00
LHX4	0.00	0.00	0.00	0.21	0.00	0.32	0.11	0.26	0.00	0.00	0.00	0.21	0.14	0.00	0.33	0.16	0.00	0.00	0.00	0.00	0.00
HMX2	0.00	0.00	0.00	0.22	0.00	0.29	0.12	0.19	0.00	0.00	0.00	0.23	0.13	0.00	0.37	0.18	0.00	0.00	0.00	0.00	0.00
ZIC3	0.00	0.00	0.00	0.22	0.00	0.26	0.14	0.23	0.00	0.00	0.00	0.18	0.14	0.00	0.32	0.15	0.00	0.00	0.00	0.00	0.00
ATXN7	0.00	0.00	0.00	0.15	0.00	0.22	0.00	0.16	0.19	0.00	0.00	0.20	0.12	0.00	0.24	0.13	0.00	0.00	0.00	0.00	0.00
LMX1B	0.00	0.00	0.00	0.13	0.00	0.12	0.00	0.21	0.10	0.00	0.00	0.15	0.16	0.00	0.32	0.21	0.00	0.00	0.00	0.00	0.00
TPPP3	0.00	0.00	0.00	0.25	0.00	0.42	0.00	0.30	0.18	0.00	0.00	0.24	0.13	0.00	0.25	0.12	0.00	0.00	0.00	0.00	0.00
IFT140	0.00	0.00	0.00	0.22	0.00	0.34	0.00	0.15	0.00	0.00	0.12	0.26	0.12	0.00	0.33	0.16	0.00	0.00	0.00	0.00	0.00
GJC1	0.00	0.00	0.00	0.17	0.00	0.16	0.00	0.10	0.00	0.00	0.12	0.14	0.13	0.00	0.21	0.10	0.00	0.00	0.00	0.00	0.00
C1ORF130	0.00	0.00	0.00	0.21	0.00	0.34	0.00	0.15	0.00	0.00	0.12	0.23	0.12	0.00	0.49	0.44	0.00	0.00	0.00	0.00	0.00
CHST3	0.00	0.00	0.00	0.16	0.00	0.31	0.00	0.10	0.00	0.00	0.12	0.24	0.13	0.00	0.30	0.18	0.00	0.00	0.00	0.00	0.00
LAMA2	0.00	0.00	0.00	0.13	0.00	0.00	0.10	0.00	0.20	0.00	0.10	0.13	0.11	0.00	0.24	0.15	0.00	0.00	0.00	0.00	0.00
RIMS4	0.00	0.00	0.00	0.24	0.00	0.31	0.12	0.00	0.00	0.00	0.12	0.28	0.00	0.17	0.24	0.10	0.00	0.00	0.00	0.00	0.00
KCND1	0.00	0.00	0.00	0.15	0.00	0.26	0.00	0.15	0.00	0.00	0.16	0.24	0.00	0.17	0.20	0.12	0.00	0.00	0.00	0.00	0.00
GRIN1	0.00	0.00	0.00	0.14	0.00	0.00	0.10	0.10	0.11	0.00	0.00	0.00	0.15	0.17	0.15	0.14	0.00	0.00	0.00	0.00	0.00
EN2	0.00	0.00	0.00	0.13	0.00	0.12	0.00	0.13	0.00	0.00	0.00	0.24	0.25	0.15	0.32	0.24	0.00	0.00	0.00	0.00	0.00
DLX5	0.00	0.00	0.00	0.13	0.00	0.13	0.00	0.23	0.00	0.00	0.00	0.15	0.18	0.15	0.21	0.11	0.00	0.00	0.00	0.00	0.00
CCDC64B	0.00	0.00	0.00	0.20	0.00	0.34	0.00	0.18	0.00	0.00	0.00	0.35	0.23	0.17	0.37	0.18	0.00	0.00	0.00	0.00	0.00

CCDC64	0.00	0.00	0.00	0.20	0.00	0.34	0.00	0.18	0.00	0.00	0.00	0.35	0.23	0.17	0.37	0.18	0.00	0.00	0.00	0.00	0.00
DLX1	0.00	0.00	0.00	0.13	0.00	0.12	0.00	0.19	0.00	0.00	0.00	0.19	0.27	0.19	0.29	0.20	0.00	0.00	0.00	0.00	0.00
EMX2	0.00	0.00	0.00	0.11	0.00	0.14	0.00	0.23	0.00	0.00	0.00	0.21	0.30	0.19	0.32	0.25	0.00	0.00	0.00	0.00	0.00
MARCKSP1	0.00	0.00	0.00	0.25	0.00	0.49	0.00	0.13	0.00	0.00	0.00	0.43	0.30	0.31	0.37	0.17	0.00	0.00	0.00	0.00	0.00
HAR1A	0.00	0.00	0.00	0.20	0.00	0.34	0.00	0.12	0.00	0.00	0.00	0.49	0.48	0.38	0.50	0.33	0.00	0.00	0.00	0.00	0.00
WDR62	0.00	0.00	0.00	0.23	0.00	0.39	0.00	0.15	0.00	0.00	0.00	0.27	0.20	0.16	0.28	0.10	0.00	0.00	0.00	0.00	0.00
GAP43	0.00	0.00	0.00	0.13	0.00	0.11	0.00	0.17	0.00	0.00	0.00	0.18	0.25	0.28	0.29	0.21	0.00	0.00	0.00	0.00	0.00
HAR1B	0.00	0.00	0.00	0.21	0.00	0.34	0.00	0.13	0.00	0.00	0.00	0.48	0.47	0.36	0.50	0.33	0.00	0.00	0.00	0.00	0.00
DLX2	0.00	0.00	0.00	0.11	0.00	0.10	0.00	0.22	0.00	0.00	0.00	0.16	0.24	0.17	0.25	0.18	0.00	0.00	0.00	0.00	0.00
DLX6	0.00	0.00	0.00	0.16	0.00	0.18	0.00	0.24	0.00	0.00	0.00	0.18	0.21	0.15	0.24	0.11	0.00	0.00	0.00	0.00	0.00
ZIC4	0.00	0.00	0.00	0.21	0.00	0.40	0.00	0.24	0.00	0.00	0.00	0.31	0.30	0.21	0.38	0.24	0.00	0.00	0.00	0.00	0.00
SIX6	0.00	0.00	0.00	0.18	0.00	0.26	0.00	0.23	0.00	0.00	0.00	0.25	0.17	0.11	0.33	0.15	0.00	0.00	0.00	0.00	0.00
MAP2	0.00	0.00	0.00	0.13	0.00	0.12	0.00	0.16	0.00	0.00	0.00	0.23	0.30	0.29	0.26	0.20	0.00	0.00	0.00	0.00	0.00
NEUROG2	0.00	0.00	0.00	0.15	0.00	0.13	0.00	0.27	0.00	0.00	0.00	0.21	0.29	0.19	0.42	0.31	0.00	0.00	0.00	0.00	0.00
OTX1	0.00	0.00	0.00	0.14	0.00	0.14	0.00	0.19	0.00	0.00	0.00	0.25	0.30	0.19	0.37	0.28	0.00	0.00	0.00	0.00	0.00
CCDC141	0.00	0.00	0.00	0.19	0.00	0.33	0.00	0.17	0.00	0.00	0.00	0.30	0.29	0.21	0.30	0.15	0.00	0.00	0.00	0.00	0.00
NDE1	0.00	0.00	0.00	0.15	0.00	0.25	0.00	0.16	0.00	0.00	0.00	0.27	0.27	0.17	0.27	0.12	0.00	0.00	0.00	0.00	0.00
TBC1D24	0.00	0.00	0.00	0.21	0.00	0.47	0.00	0.13	0.00	0.00	0.00	0.31	0.16	0.18	0.26	0.11	0.00	0.00	0.00	0.00	0.00
SEP-07	0.00	0.00	0.00	0.22	0.00	0.40	0.00	0.21	0.00	0.00	0.00	0.28	0.24	0.18	0.29	0.12	0.00	0.00	0.00	0.00	0.00
RAX	0.00	0.00	0.00	0.16	0.00	0.17	0.00	0.19	0.00	0.00	0.00	0.20	0.23	0.14	0.33	0.20	0.00	0.00	0.00	0.00	0.00
SMAN1	0.00	0.00	0.00	0.10	0.00	0.12	0.00	0.00	0.18	0.00	0.00	0.24	0.17	0.13	0.41	0.25	0.00	0.00	0.00	0.00	0.00
SPG11	0.00	0.00	0.00	0.14	0.00	0.20	0.00	0.00	0.12	0.00	0.00	0.21	0.16	0.10	0.26	0.16	0.00	0.00	0.00	0.00	0.00
LIX1	0.00	0.00	0.00	0.16	0.00	0.25	0.00	0.00	0.17	0.00	0.00	0.26	0.18	0.15	0.38	0.22	0.00	0.00	0.00	0.00	0.00
MECP2	0.00	0.00	0.00	0.11	0.00	0.00	0.00	0.18	0.16	0.00	0.00	0.21	0.20	0.20	0.20	0.13	0.00	0.00	0.00	0.00	0.00
NF1	0.00	0.00	0.00	0.10	0.00	0.00	0.00	0.15	0.21	0.00	0.00	0.16	0.12	0.11	0.24	0.17	0.00	0.00	0.00	0.00	0.00
PVALB	0.00	0.00	0.00	0.12	0.00	0.00	0.00	0.15	0.13	0.00	0.00	0.13	0.24	0.24	0.21	0.16	0.00	0.00	0.00	0.00	0.00
TARDBP	0.00	0.00	0.00	0.00	0.00	0.12	0.00	0.16	0.22	0.00	0.00	0.16	0.17	0.17	0.23	0.15	0.00	0.00	0.00	0.00	0.00
RTN3P1	0.00	0.00	0.00	0.31	0.00	0.61	0.00	0.00	0.00	0.00	0.19	0.36	0.15	0.12	0.38	0.21	0.00	0.00	0.00	0.00	0.00
GJB5	0.00	0.00	0.00	0.21	0.00	0.28	0.00	0.00	0.00	0.00	0.11	0.20	0.16	0.11	0.26	0.11	0.00	0.00	0.00	0.00	0.00
KCNAB3	0.00	0.00	0.00	0.16	0.00	0.25	0.00	0.00	0.00	0.00	0.15	0.33	0.14	0.19	0.28	0.18	0.00	0.00	0.00	0.00	0.00
KCNV1	0.00	0.00	0.00	0.17	0.00	0.34	0.00	0.00	0.00	0.00	0.16	0.32	0.16	0.20	0.25	0.15	0.00	0.00	0.00	0.00	0.00
KCNF1	0.00	0.00	0.00	0.21	0.00	0.39	0.00	0.00	0.00	0.00	0.18	0.38	0.14	0.17	0.28	0.14	0.00	0.00	0.00	0.00	0.00
KCNG3	0.00	0.00	0.00	0.18	0.00	0.26	0.00	0.00	0.00	0.00	0.18	0.29	0.16	0.20	0.25	0.14	0.00	0.00	0.00	0.00	0.00
C20ORF203	0.00	0.00	0.00	0.31	0.00	0.61	0.00	0.00	0.00	0.00	0.13	0.46	0.24	0.22	0.35	0.16	0.00	0.00	0.00	0.00	0.00
KCNC3	0.00	0.00	0.00	0.13	0.00	0.20	0.00	0.00	0.00	0.00	0.12	0.29	0.22	0.24	0.33	0.23	0.00	0.00	0.00	0.00	0.00
SLC4A10	0.00	0.00	0.00	0.20	0.00	0.29	0.00	0.00	0.00	0.00	0.24	0.34	0.32	0.24	0.27	0.17	0.00	0.00	0.00	0.00	0.00
EFHB	0.00	0.00	0.00	0.20	0.00	0.27	0.00	0.00	0.00	0.00	0.17	0.33	0.12	0.18	0.21	0.16	0.00	0.00	0.00	0.00	0.00
KCNG1	0.00	0.00	0.00	0.19	0.00	0.33	0.00	0.00	0.00	0.00	0.14	0.35	0.14	0.16	0.27	0.12	0.00	0.00	0.00	0.00	0.00
KCNS1	0.00	0.00	0.00	0.18	0.00	0.27	0.00	0.00	0.00	0.00	0.11	0.33	0.16	0.20	0.35	0.20	0.00	0.00	0.00	0.00	0.00
CCL3L1	0.00	0.00	0.13	0.19	0.00	0.12	0.16	0.17	0.18	0.00	0.00	0.00	0.00	0.00	0.13	0.00	0.11	0.00	0.00	0.00	0.00

KLK1	0.00	0.00	0.13	0.19	0.00	0.14	0.14	0.16	0.20	0.00	0.00	0.00	0.00	0.00	0.13	0.00	0.14	0.00	0.00	0.00	0.00
IFNK	0.00	0.00	0.16	0.29	0.00	0.27	0.22	0.22	0.18	0.00	0.00	0.00	0.00	0.00	0.14	0.00	0.11	0.00	0.00	0.00	0.00
CD1E	0.00	0.00	0.11	0.24	0.00	0.24	0.17	0.10	0.00	0.00	0.17	0.00	0.00	0.00	0.17	0.00	0.11	0.00	0.00	0.00	0.00
ARL6	0.00	0.00	0.15	0.30	0.00	0.44	0.15	0.15	0.00	0.00	0.00	0.20	0.00	0.00	0.22	0.00	0.10	0.00	0.00	0.00	0.00
BBS9	0.00	0.00	0.11	0.26	0.00	0.45	0.10	0.11	0.00	0.00	0.00	0.18	0.00	0.00	0.16	0.00	0.10	0.00	0.00	0.00	0.00
EXOC3L2	0.00	0.00	0.00	0.22	0.00	0.27	0.13	0.11	0.14	0.00	0.00	0.20	0.00	0.00	0.13	0.00	0.10	0.00	0.00	0.00	0.00
TAGAP	0.00	0.00	0.00	0.23	0.00	0.30	0.13	0.12	0.11	0.00	0.00	0.14	0.00	0.00	0.14	0.00	0.20	0.00	0.00	0.00	0.00
METTL21B	0.00	0.00	0.00	0.24	0.00	0.37	0.12	0.19	0.19	0.00	0.00	0.11	0.00	0.00	0.13	0.00	0.16	0.00	0.00	0.00	0.00
KIF21B	0.00	0.00	0.00	0.23	0.00	0.36	0.12	0.12	0.00	0.00	0.10	0.20	0.00	0.00	0.27	0.00	0.13	0.00	0.00	0.00	0.00
ZNF699	0.00	0.00	0.00	0.19	0.00	0.27	0.10	0.00	0.00	0.00	0.00	0.19	0.17	0.13	0.18	0.00	0.11	0.00	0.00	0.00	0.00
FMN2	0.00	0.00	0.00	0.23	0.00	0.40	0.00	0.19	0.00	0.00	0.00	0.24	0.24	0.16	0.29	0.00	0.14	0.00	0.00	0.00	0.00
ZNF227	0.00	0.00	0.00	0.27	0.00	0.46	0.11	0.13	0.00	0.00	0.00	0.21	0.00	0.00	0.28	0.15	0.17	0.00	0.00	0.00	0.00
CTSS	0.00	0.00	0.00	0.14	0.00	0.00	0.11	0.17	0.24	0.00	0.00	0.11	0.00	0.00	0.17	0.12	0.15	0.00	0.00	0.00	0.00
KCNH7	0.00	0.00	0.00	0.15	0.00	0.21	0.00	0.00	0.00	0.00	0.12	0.23	0.00	0.13	0.25	0.13	0.11	0.00	0.00	0.00	0.00
C10ORF67	0.00	0.00	0.00	0.11	0.00	0.13	0.00	0.00	0.00	0.00	0.00	0.26	0.30	0.23	0.34	0.25	0.12	0.00	0.00	0.00	0.00
TRIM65	0.00	0.00	0.00	0.00	0.00	0.11	0.00	0.00	0.11	0.00	0.00	0.23	0.19	0.18	0.19	0.12	0.16	0.00	0.00	0.00	0.00
AQP4	0.00	0.00	0.00	0.00	0.00	0.00	0.00	0.11	0.16	0.00	0.00	0.15	0.15	0.16	0.19	0.15	0.10	0.00	0.00	0.00	0.00
TCERG1	0.00	0.00	0.14	0.31	0.00	0.38	0.18	0.29	0.12	0.00	0.00	0.00	0.00	0.00	0.18	0.00	0.00	0.11	0.00	0.00	0.00
OXT	0.00	0.00	0.12	0.13	0.00	0.00	0.11	0.00	0.00	0.14	0.00	0.11	0.00	0.10	0.18	0.00	0.00	0.11	0.00	0.00	0.00
CNR1	0.13	0.13	0.00	0.11	0.00	0.00	0.00	0.00	0.14	0.00	0.00	0.00	0.12	0.14	0.13	0.00	0.00	0.11	0.00	0.00	0.00
MTMR12	0.00	0.00	0.12	0.30	0.00	0.48	0.14	0.13	0.00	0.00	0.15	0.17	0.00	0.00	0.00	0.00	0.00	0.00	0.15	0.00	0.00
TMEM97	0.00	0.00	0.00	0.22	0.00	0.34	0.11	0.36	0.25	0.00	0.10	0.13	0.00	0.00	0.00	0.00	0.00	0.00	0.16	0.00	0.00
MED25	0.00	0.00	0.15	0.36	0.00	0.44	0.22	0.41	0.11	0.00	0.00	0.00	0.00	0.00	0.22	0.00	0.00	0.00	0.11	0.00	0.00
MIR613	0.00	0.00	0.21	0.34	0.00	0.32	0.26	0.47	0.14	0.00	0.00	0.00	0.00	0.00	0.11	0.00	0.00	0.00	0.18	0.00	0.00
RDH5	0.00	0.00	0.12	0.18	0.00	0.18	0.11	0.12	0.14	0.00	0.00	0.00	0.00	0.00	0.13	0.00	0.00	0.00	0.24	0.00	0.00
ATP8B1	0.00	0.00	0.00	0.18	0.00	0.21	0.11	0.17	0.17	0.00	0.13	0.00	0.00	0.00	0.11	0.00	0.00	0.00	0.17	0.00	0.00
SREBF2	0.00	0.00	0.00	0.15	0.00	0.12	0.12	0.23	0.13	0.00	0.10	0.00	0.00	0.00	0.13	0.00	0.00	0.00	0.20	0.00	0.00
APOL4	0.00	0.00	0.11	0.28	0.00	0.39	0.15	0.11	0.00	0.00	0.00	0.20	0.00	0.00	0.16	0.00	0.00	0.00	0.15	0.00	0.00
SAA4	0.00	0.00	0.11	0.26	0.00	0.31	0.15	0.12	0.00	0.00	0.00	0.12	0.00	0.00	0.11	0.00	0.00	0.00	0.16	0.00	0.00
EXOC6	0.00	0.00	0.12	0.29	0.00	0.34	0.17	0.21	0.00	0.00	0.00	0.14	0.00	0.00	0.17	0.00	0.00	0.00	0.15	0.00	0.00
CLK4	0.00	0.00	0.13	0.38	0.00	0.55	0.18	0.20	0.00	0.00	0.00	0.27	0.00	0.00	0.23	0.00	0.00	0.00	0.13	0.00	0.00
HNRPDL	0.00	0.00	0.10	0.35	0.00	0.54	0.15	0.32	0.00	0.00	0.00	0.18	0.00	0.00	0.19	0.00	0.00	0.00	0.15	0.00	0.00
OCRL	0.00	0.00	0.11	0.23	0.00	0.28	0.13	0.10	0.00	0.00	0.00	0.17	0.00	0.00	0.14	0.00	0.00	0.00	0.14	0.00	0.00
PCBP3	0.00	0.00	0.11	0.32	0.00	0.47	0.14	0.26	0.00	0.00	0.00	0.22	0.00	0.00	0.21	0.00	0.00	0.00	0.12	0.00	0.00
ACER1	0.00	0.00	0.14	0.25	0.00	0.34	0.12	0.18	0.00	0.00	0.00	0.17	0.00	0.00	0.12	0.00	0.00	0.00	0.21	0.00	0.00
RBM4	0.00	0.00	0.00	0.27	0.00	0.43	0.11	0.39	0.12	0.00	0.00	0.21	0.00	0.00	0.23	0.00	0.00	0.00	0.15	0.00	0.00
CSNK1E	0.00	0.00	0.00	0.19	0.00	0.18	0.12	0.24	0.15	0.00	0.00	0.11	0.00	0.00	0.14	0.00	0.00	0.00	0.11	0.00	0.00
MBNL1	0.00	0.00	0.00	0.22	0.00	0.28	0.12	0.21	0.16	0.00	0.00	0.14	0.00	0.00	0.19	0.00	0.00	0.00	0.12	0.00	0.00
TIAL1	0.00	0.00	0.00	0.22	0.00	0.27	0.12	0.32	0.17	0.00	0.00	0.12	0.00	0.00	0.18	0.00	0.00	0.00	0.13	0.00	0.00
MIR96	0.00	0.00	0.00	0.24	0.00	0.31	0.13	0.52	0.29	0.00	0.00	0.16	0.00	0.00	0.20	0.00	0.00	0.00	0.12	0.00	0.00

TRA2B	0.00	0.00	0.00	0.24	0.00	0.33	0.12	0.25	0.18	0.00	0.00	0.13	0.00	0.00	0.20	0.00	0.00	0.00	0.17	0.00	0.00
DMWD	0.00	0.00	0.00	0.27	0.00	0.39	0.13	0.20	0.11	0.00	0.00	0.24	0.00	0.00	0.25	0.00	0.00	0.00	0.11	0.00	0.00
MIR29B2	0.00	0.00	0.00	0.25	0.00	0.38	0.12	0.30	0.20	0.00	0.00	0.16	0.00	0.00	0.12	0.00	0.00	0.00	0.11	0.00	0.00
HPD	0.00	0.00	0.00	0.22	0.00	0.30	0.12	0.12	0.17	0.00	0.00	0.15	0.00	0.00	0.16	0.00	0.00	0.00	0.23	0.00	0.00
GCHFR	0.00	0.00	0.00	0.24	0.00	0.30	0.13	0.24	0.17	0.00	0.00	0.18	0.00	0.00	0.19	0.00	0.00	0.00	0.17	0.00	0.00
TFCP2	0.00	0.00	0.00	0.23	0.00	0.27	0.14	0.36	0.16	0.00	0.00	0.15	0.00	0.00	0.19	0.00	0.00	0.00	0.11	0.00	0.00
CHIT1	0.00	0.00	0.00	0.18	0.00	0.19	0.13	0.13	0.24	0.00	0.00	0.13	0.00	0.00	0.17	0.00	0.00	0.00	0.14	0.00	0.00
MVK	0.00	0.00	0.00	0.21	0.00	0.25	0.12	0.18	0.16	0.00	0.00	0.11	0.00	0.00	0.13	0.00	0.00	0.00	0.30	0.00	0.00
CSNK1D	0.00	0.00	0.00	0.22	0.00	0.27	0.11	0.24	0.11	0.00	0.00	0.17	0.00	0.00	0.16	0.00	0.00	0.00	0.11	0.00	0.00
SRSF2	0.00	0.00	0.00	0.21	0.00	0.24	0.10	0.24	0.13	0.00	0.00	0.10	0.00	0.00	0.18	0.00	0.00	0.00	0.17	0.00	0.00
NAA10	0.00	0.00	0.00	0.22	0.00	0.31	0.11	0.40	0.26	0.00	0.00	0.16	0.00	0.00	0.15	0.00	0.00	0.00	0.13	0.00	0.00
PTPLA	0.00	0.00	0.00	0.29	0.00	0.43	0.15	0.23	0.11	0.00	0.00	0.25	0.00	0.00	0.22	0.00	0.00	0.00	0.14	0.00	0.00
LMBRD1	0.00	0.00	0.11	0.25	0.00	0.35	0.13	0.00	0.00	0.00	0.11	0.15	0.00	0.00	0.16	0.00	0.00	0.00	0.30	0.00	0.00
NIPAL4	0.00	0.00	0.14	0.30	0.00	0.38	0.18	0.00	0.00	0.00	0.21	0.13	0.00	0.00	0.16	0.00	0.00	0.00	0.12	0.00	0.00
ATP11C	0.00	0.00	0.00	0.29	0.00	0.49	0.13	0.13	0.00	0.00	0.19	0.21	0.00	0.00	0.19	0.00	0.00	0.00	0.11	0.00	0.00
MTMR1	0.00	0.00	0.00	0.27	0.00	0.45	0.12	0.13	0.00	0.00	0.15	0.20	0.00	0.00	0.17	0.00	0.00	0.00	0.14	0.00	0.00
ATP9A	0.00	0.00	0.00	0.28	0.00	0.48	0.11	0.10	0.00	0.00	0.20	0.18	0.00	0.00	0.16	0.00	0.00	0.00	0.11	0.00	0.00
SLC40A1	0.00	0.00	0.00	0.16	0.00	0.12	0.12	0.00	0.12	0.00	0.10	0.11	0.00	0.00	0.17	0.00	0.00	0.00	0.20	0.00	0.00
GNPTG	0.00	0.00	0.00	0.24	0.00	0.31	0.14	0.00	0.15	0.00	0.11	0.16	0.00	0.00	0.16	0.00	0.00	0.00	0.12	0.00	0.00
PION	0.00	0.00	0.00	0.14	0.00	0.18	0.00	0.23	0.25	0.00	0.11	0.16	0.00	0.00	0.12	0.00	0.00	0.00	0.16	0.00	0.00
DHDDS	0.00	0.00	0.00	0.26	0.00	0.49	0.00	0.14	0.13	0.00	0.15	0.20	0.00	0.00	0.18	0.00	0.00	0.00	0.18	0.00	0.00
GANC	0.00	0.00	0.00	0.15	0.00	0.17	0.00	0.16	0.20	0.00	0.15	0.11	0.00	0.00	0.15	0.00	0.00	0.00	0.13	0.00	0.00
ABCA3	0.00	0.00	0.00	0.15	0.00	0.26	0.00	0.17	0.17	0.00	0.14	0.14	0.00	0.00	0.15	0.00	0.00	0.00	0.14	0.00	0.00
ALG12	0.00	0.00	0.00	0.22	0.00	0.36	0.00	0.10	0.10	0.00	0.25	0.14	0.00	0.00	0.17	0.00	0.00	0.00	0.11	0.00	0.00
ATP7B	0.00	0.00	0.00	0.11	0.00	0.15	0.00	0.13	0.19	0.00	0.10	0.15	0.00	0.00	0.15	0.00	0.00	0.00	0.20	0.00	0.00
GBA3	0.00	0.00	0.00	0.18	0.00	0.27	0.00	0.17	0.19	0.00	0.13	0.15	0.00	0.00	0.15	0.00	0.00	0.00	0.19	0.00	0.00
SLC26A2	0.00	0.00	0.00	0.16	0.00	0.23	0.00	0.12	0.13	0.00	0.17	0.11	0.00	0.00	0.14	0.00	0.00	0.00	0.13	0.00	0.00
APH1A	0.00	0.00	0.00	0.18	0.00	0.22	0.00	0.16	0.12	0.00	0.20	0.15	0.00	0.00	0.15	0.00	0.00	0.00	0.13	0.00	0.00
DPM1	0.00	0.00	0.00	0.23	0.00	0.40	0.00	0.17	0.10	0.00	0.18	0.20	0.00	0.00	0.17	0.00	0.00	0.00	0.21	0.00	0.00
ENPP7	0.00	0.00	0.00	0.24	0.00	0.41	0.00	0.18	0.11	0.00	0.10	0.19	0.00	0.00	0.12	0.00	0.00	0.00	0.32	0.00	0.00
SPR	0.00	0.00	0.00	0.16	0.00	0.24	0.00	0.11	0.12	0.00	0.00	0.22	0.11	0.00	0.20	0.00	0.00	0.00	0.24	0.00	0.00
NT5C3	0.00	0.00	0.00	0.20	0.00	0.34	0.00	0.13	0.12	0.00	0.00	0.22	0.10	0.00	0.19	0.00	0.00	0.00	0.26	0.00	0.00
NAAA	0.00	0.00	0.00	0.24	0.00	0.39	0.00	0.12	0.11	0.00	0.00	0.28	0.12	0.00	0.21	0.00	0.00	0.00	0.23	0.00	0.00
ALDH1A1	0.00	0.00	0.00	0.15	0.00	0.00	0.13	0.19	0.19	0.00	0.00	0.16	0.13	0.00	0.19	0.00	0.00	0.00	0.26	0.00	0.00
NSDHL	0.00	0.00	0.00	0.21	0.00	0.27	0.11	0.00	0.00	0.00	0.12	0.21	0.11	0.00	0.19	0.00	0.00	0.00	0.26	0.00	0.00
RPS23	0.00	0.00	0.00	0.32	0.00	0.56	0.12	0.13	0.00	0.00	0.00	0.28	0.00	0.11	0.17	0.00	0.00	0.00	0.15	0.00	0.00
CHMP2B	0.00	0.00	0.00	0.17	0.00	0.25	0.00	0.15	0.15	0.00	0.00	0.18	0.00	0.13	0.21	0.00	0.00	0.00	0.11	0.00	0.00
UBE2D4	0.00	0.00	0.00	0.20	0.00	0.44	0.00	0.17	0.00	0.00	0.00	0.31	0.14	0.13	0.20	0.00	0.00	0.00	0.17	0.00	0.00
NXF5	0.00	0.00	0.00	0.25	0.00	0.50	0.00	0.17	0.00	0.00	0.00	0.30	0.15	0.13	0.24	0.00	0.00	0.00	0.11	0.00	0.00
TPH1	0.00	0.00	0.00	0.12	0.00	0.00	0.00	0.11	0.14	0.00	0.00	0.14	0.12	0.11	0.17	0.00	0.00	0.00	0.11	0.00	0.00

TPH2	0.00	0.00	0.00	0.11	0.00	0.00	0.00	0.11	0.14	0.00	0.00	0.15	0.16	0.14	0.20	0.00	0.00	0.00	0.11	0.00	0.00
MME	0.00	0.00	0.00	0.10	0.00	0.00	0.00	0.15	0.22	0.00	0.00	0.14	0.16	0.15	0.16	0.00	0.00	0.00	0.10	0.00	0.00
PMP2	0.00	0.00	0.00	0.28	0.00	0.46	0.12	0.00	0.00	0.00	0.15	0.25	0.00	0.00	0.35	0.23	0.00	0.00	0.16	0.00	0.00
PAH	0.00	0.00	0.00	0.12	0.00	0.00	0.00	0.12	0.19	0.00	0.00	0.15	0.12	0.00	0.20	0.11	0.00	0.00	0.16	0.00	0.00
TMEM167A	0.00	0.00	0.00	0.11	0.00	0.12	0.00	0.20	0.26	0.00	0.00	0.00	0.00	0.12	0.18	0.12	0.00	0.00	0.15	0.00	0.00
PLEKHG3	0.00	0.00	0.00	0.16	0.00	0.26	0.00	0.00	0.00	0.00	0.00	0.33	0.21	0.21	0.24	0.10	0.00	0.00	0.14	0.00	0.00
ST8SIA5	0.00	0.00	0.00	0.21	0.00	0.43	0.00	0.00	0.00	0.00	0.00	0.41	0.19	0.19	0.30	0.16	0.00	0.00	0.19	0.00	0.00
HEXB	0.00	0.00	0.00	0.11	0.00	0.00	0.00	0.00	0.22	0.00	0.00	0.19	0.14	0.11	0.24	0.22	0.00	0.00	0.14	0.00	0.00
HNP1	0.00	0.00	0.00	0.25	0.00	0.42	0.11	0.21	0.00	0.00	0.00	0.11	0.00	0.00	0.11	0.00	0.12	0.00	0.11	0.00	0.00
NNMT	0.00	0.00	0.00	0.16	0.00	0.20	0.00	0.21	0.22	0.00	0.00	0.14	0.00	0.00	0.14	0.00	0.11	0.00	0.32	0.00	0.00
ATXN3	0.00	0.00	0.00	0.13	0.00	0.17	0.00	0.14	0.18	0.00	0.00	0.14	0.00	0.00	0.18	0.00	0.00	0.11	0.10	0.00	0.00
GALE	0.00	0.00	0.00	0.15	0.00	0.27	0.00	0.11	0.17	0.00	0.00	0.17	0.00	0.00	0.19	0.00	0.00	0.12	0.29	0.00	0.00
PNP	0.00	0.00	0.00	0.15	0.00	0.17	0.00	0.13	0.17	0.00	0.00	0.15	0.00	0.00	0.16	0.00	0.00	0.14	0.22	0.00	0.00
RAB22A	0.00	0.00	0.17	0.33	0.00	0.37	0.21	0.20	0.00	0.00	0.13	0.13	0.00	0.00	0.00	0.00	0.00	0.00	0.00	0.13	0.00
RAB8A	0.00	0.00	0.12	0.25	0.00	0.29	0.14	0.19	0.00	0.00	0.00	0.13	0.00	0.00	0.12	0.00	0.00	0.00	0.00	0.17	0.00
WNK1	0.00	0.00	0.00	0.18	0.00	0.19	0.11	0.19	0.11	0.00	0.00	0.10	0.00	0.00	0.18	0.00	0.00	0.00	0.00	0.11	0.00
EIF2S1	0.00	0.00	0.00	0.12	0.00	0.18	0.00	0.23	0.17	0.00	0.00	0.11	0.00	0.11	0.13	0.00	0.00	0.00	0.00	0.13	0.00
RAB3A	0.00	0.00	0.00	0.15	0.00	0.18	0.00	0.18	0.00	0.00	0.00	0.17	0.13	0.18	0.18	0.00	0.00	0.00	0.00	0.14	0.00
RAB3GAP1	0.00	0.00	0.00	0.16	0.00	0.29	0.00	0.17	0.00	0.00	0.00	0.25	0.12	0.12	0.21	0.00	0.00	0.00	0.00	0.13	0.00
SNAP25	0.00	0.00	0.00	0.15	0.00	0.00	0.11	0.20	0.00	0.00	0.00	0.16	0.18	0.20	0.22	0.00	0.00	0.00	0.00	0.12	0.00
RAB14	0.00	0.00	0.11	0.26	0.00	0.33	0.15	0.29	0.00	0.00	0.00	0.11	0.00	0.00	0.00	0.00	0.13	0.00	0.00	0.23	0.00
ATF3	0.00	0.00	0.00	0.17	0.00	0.14	0.11	0.27	0.25	0.00	0.00	0.00	0.00	0.00	0.19	0.00	0.13	0.00	0.00	0.14	0.00
EIF2AK3	0.00	0.00	0.00	0.14	0.00	0.17	0.00	0.22	0.22	0.00	0.11	0.00	0.00	0.00	0.12	0.00	0.14	0.00	0.00	0.19	0.00
RFX6	0.00	0.00	0.00	0.22	0.00	0.38	0.00	0.20	0.14	0.00	0.00	0.15	0.00	0.00	0.20	0.00	0.25	0.00	0.00	0.14	0.00
KCNMA1	0.00	0.00	0.00	0.00	0.00	0.00	0.00	0.16	0.16	0.00	0.11	0.11	0.00	0.12	0.13	0.00	0.10	0.00	0.00	0.11	0.00
HCRT	0.00	0.00	0.12	0.11	0.00	0.00	0.00	0.00	0.00	0.00	0.00	0.11	0.14	0.00	0.20	0.11	0.00	0.23	0.00	0.11	0.00
ZPLD1	0.00	0.00	0.00	0.17	0.00	0.26	0.00	0.12	0.00	0.00	0.00	0.16	0.00	0.00	0.17	0.00	0.20	0.14	0.00	0.14	0.00
GULOP	0.00	0.00	0.00	0.18	0.00	0.25	0.00	0.21	0.22	0.00	0.00	0.16	0.00	0.00	0.17	0.00	0.00	0.00	0.32	0.12	0.00
APOBR	0.00	0.00	0.20	0.34	0.00	0.30	0.25	0.19	0.00	0.00	0.00	0.00	0.00	0.00	0.00	0.00	0.13	0.00	0.27	0.12	0.00
KL	0.00	0.00	0.00	0.15	0.00	0.10	0.12	0.14	0.17	0.00	0.00	0.00	0.00	0.00	0.00	0.00	0.13	0.00	0.20	0.12	0.00
HDLC3	0.00	0.00	0.00	0.18	0.00	0.19	0.13	0.17	0.16	0.00	0.00	0.00	0.00	0.00	0.00	0.00	0.21	0.00	0.22	0.14	0.00
PLTP	0.00	0.00	0.00	0.16	0.00	0.15	0.12	0.15	0.17	0.00	0.00	0.00	0.00	0.00	0.00	0.00	0.14	0.00	0.23	0.10	0.00
APOL1	0.00	0.00	0.00	0.18	0.00	0.22	0.12	0.15	0.12	0.00	0.00	0.00	0.00	0.00	0.00	0.00	0.22	0.00	0.15	0.12	0.00
APOC1	0.00	0.00	0.00	0.14	0.00	0.12	0.10	0.13	0.15	0.00	0.00	0.00	0.00	0.00	0.00	0.00	0.16	0.00	0.21	0.11	0.00
HP	0.00	0.00	0.00	0.14	0.00	0.00	0.10	0.14	0.17	0.00	0.00	0.00	0.00	0.00	0.12	0.00	0.19	0.00	0.15	0.13	0.00
LUZP2	0.00	0.00	0.00	0.21	0.00	0.26	0.12	0.00	0.00	0.00	0.00	0.20	0.00	0.00	0.21	0.00	0.18	0.00	0.23	0.15	0.00
PCBD1	0.00	0.00	0.00	0.18	0.00	0.30	0.00	0.19	0.00	0.00	0.00	0.13	0.00	0.00	0.15	0.00	0.16	0.00	0.21	0.13	0.00
FUCA1	0.00	0.00	0.00	0.13	0.00	0.19	0.00	0.00	0.16	0.00	0.00	0.16	0.00	0.00	0.13	0.00	0.20	0.00	0.21	0.19	0.00
SLC2A9	0.00	0.00	0.00	0.15	0.00	0.21	0.00	0.00	0.00	0.00	0.11	0.12	0.00	0.00	0.12	0.00	0.28	0.00	0.21	0.28	0.00
CAT	0.00	0.00	0.00	0.12	0.00	0.00	0.00	0.00	0.11	0.00	0.00	0.00	0.11	0.11	0.11	0.00	0.15	0.00	0.11	0.10	0.00

NR1H3	0.00	0.00	0.00	0.14	0.00	0.00	0.11	0.19	0.16	0.00	0.00	0.00	0.00	0.00	0.12	0.00	0.00	0.13	0.17	0.12	0.00
CKBE	0.00	0.00	0.00	0.12	0.00	0.10	0.00	0.10	0.20	0.00	0.00	0.00	0.00	0.00	0.00	0.00	0.11	0.16	0.19	0.13	0.00
LCAT	0.00	0.00	0.00	0.12	0.00	0.11	0.00	0.11	0.13	0.00	0.00	0.00	0.00	0.00	0.00	0.00	0.14	0.11	0.26	0.10	0.00
AMPD1	0.00	0.00	0.00	0.12	0.00	0.13	0.00	0.00	0.16	0.00	0.00	0.00	0.00	0.00	0.12	0.00	0.16	0.14	0.22	0.14	0.00
SIRT1	0.00	0.00	0.00	0.11	0.00	0.00	0.00	0.17	0.21	0.00	0.00	0.00	0.00	0.00	0.14	0.00	0.13	0.22	0.18	0.14	0.00
BMIQ4	0.00	0.00	0.00	0.11	0.00	0.18	0.00	0.00	0.00	0.00	0.00	0.11	0.00	0.00	0.12	0.00	0.29	0.20	0.13	0.22	0.00
BMIQ3	0.00	0.00	0.00	0.11	0.00	0.18	0.00	0.00	0.00	0.00	0.00	0.11	0.00	0.00	0.12	0.00	0.29	0.20	0.13	0.22	0.00
ALDH3A2	0.00	0.00	0.00	0.18	0.00	0.25	0.00	0.00	0.00	0.00	0.00	0.18	0.00	0.00	0.17	0.00	0.12	0.11	0.35	0.13	0.00
TGM7	0.00	0.00	0.13	0.27	0.00	0.24	0.21	0.26	0.18	0.00	0.17	0.00	0.00	0.00	0.00	0.00	0.00	0.00	0.00	0.00	0.13
CHMP6	0.00	0.00	0.14	0.34	0.00	0.48	0.18	0.26	0.00	0.00	0.12	0.00	0.00	0.00	0.13	0.00	0.00	0.00	0.00	0.00	0.11
BAG5	0.00	0.00	0.15	0.28	0.00	0.32	0.18	0.19	0.00	0.00	0.00	0.14	0.00	0.00	0.16	0.00	0.00	0.00	0.00	0.00	0.15
CCT4	0.00	0.00	0.13	0.32	0.00	0.44	0.16	0.23	0.00	0.00	0.00	0.20	0.00	0.00	0.26	0.00	0.00	0.00	0.00	0.00	0.11
SERGEF	0.00	0.00	0.14	0.39	0.00	0.65	0.16	0.17	0.00	0.00	0.00	0.17	0.00	0.00	0.14	0.00	0.00	0.00	0.00	0.00	0.12
MAP1LC3C	0.00	0.00	0.12	0.27	0.00	0.33	0.16	0.22	0.00	0.00	0.00	0.12	0.00	0.00	0.16	0.00	0.00	0.00	0.00	0.00	0.15
BAG3	0.00	0.00	0.00	0.23	0.00	0.27	0.14	0.31	0.30	0.00	0.00	0.14	0.00	0.00	0.21	0.00	0.00	0.00	0.00	0.00	0.11
TBCC	0.00	0.00	0.00	0.28	0.00	0.44	0.12	0.24	0.16	0.00	0.00	0.20	0.00	0.00	0.22	0.00	0.00	0.00	0.00	0.00	0.14
IFT57	0.00	0.00	0.00	0.27	0.00	0.43	0.12	0.29	0.12	0.00	0.00	0.22	0.00	0.00	0.23	0.00	0.00	0.00	0.00	0.00	0.20
AIMP2	0.00	0.00	0.00	0.24	0.00	0.33	0.12	0.30	0.18	0.00	0.00	0.15	0.00	0.00	0.19	0.00	0.00	0.00	0.00	0.00	0.12
C19ORF46	0.00	0.00	0.00	0.33	0.00	0.61	0.11	0.18	0.00	0.00	0.13	0.15	0.00	0.00	0.14	0.00	0.00	0.00	0.00	0.00	0.14
DYNLT3	0.00	0.00	0.00	0.32	0.00	0.49	0.15	0.17	0.00	0.00	0.14	0.20	0.00	0.00	0.25	0.00	0.00	0.00	0.00	0.00	0.13
DEF8	0.00	0.00	0.00	0.36	0.00	0.67	0.13	0.18	0.00	0.00	0.15	0.23	0.00	0.00	0.20	0.00	0.00	0.00	0.00	0.00	0.21
KLC3	0.00	0.00	0.00	0.30	0.00	0.50	0.12	0.14	0.00	0.00	0.00	0.27	0.12	0.00	0.30	0.00	0.00	0.00	0.00	0.00	0.14
KIF2A	0.00	0.00	0.00	0.23	0.00	0.36	0.00	0.24	0.11	0.00	0.00	0.24	0.16	0.00	0.25	0.00	0.00	0.00	0.00	0.00	0.13
GDAP1	0.00	0.00	0.00	0.13	0.00	0.22	0.00	0.00	0.13	0.00	0.10	0.16	0.00	0.00	0.30	0.19	0.00	0.00	0.00	0.00	0.17
TPPP2	0.00	0.00	0.00	0.22	0.00	0.38	0.00	0.21	0.00	0.00	0.00	0.25	0.12	0.00	0.27	0.15	0.00	0.00	0.00	0.00	0.11
CAPN1	0.00	0.00	0.00	0.13	0.00	0.00	0.00	0.16	0.23	0.00	0.00	0.16	0.00	0.16	0.17	0.12	0.00	0.00	0.00	0.00	0.11
RHOT2	0.00	0.00	0.00	0.27	0.00	0.41	0.12	0.19	0.00	0.00	0.00	0.20	0.00	0.00	0.17	0.00	0.00	0.15	0.00	0.00	0.36
TBCA	0.00	0.00	0.00	0.26	0.00	0.41	0.11	0.19	0.00	0.00	0.00	0.17	0.00	0.00	0.24	0.00	0.00	0.17	0.00	0.00	0.16
RGAG4	0.00	0.00	0.00	0.22	0.00	0.42	0.00	0.15	0.00	0.00	0.13	0.17	0.00	0.00	0.14	0.00	0.00	0.11	0.00	0.00	0.39
KLC4	0.00	0.00	0.00	0.15	0.00	0.22	0.00	0.13	0.00	0.00	0.00	0.13	0.11	0.00	0.20	0.00	0.00	0.14	0.00	0.00	0.17
SLC25A37	0.00	0.00	0.00	0.27	0.00	0.40	0.13	0.23	0.00	0.00	0.00	0.16	0.00	0.00	0.18	0.00	0.00	0.00	0.23	0.00	0.29
MRPL40	0.00	0.00	0.00	0.33	0.00	0.56	0.14	0.27	0.00	0.00	0.00	0.30	0.00	0.00	0.24	0.00	0.00	0.00	0.12	0.00	0.18
CYP51A1	0.00	0.00	0.00	0.23	0.00	0.31	0.11	0.21	0.00	0.00	0.00	0.15	0.00	0.00	0.14	0.00	0.00	0.00	0.29	0.00	0.11
PRDX1	0.00	0.00	0.00	0.17	0.00	0.19	0.00	0.26	0.24	0.00	0.00	0.12	0.00	0.00	0.13	0.00	0.00	0.00	0.12	0.00	0.12
THAP5	0.00	0.00	0.00	0.22	0.00	0.49	0.00	0.35	0.15	0.00	0.00	0.16	0.00	0.00	0.12	0.00	0.00	0.00	0.10	0.00	0.32
SEPN1	0.00	0.00	0.00	0.21	0.00	0.31	0.00	0.14	0.17	0.00	0.00	0.13	0.00	0.00	0.20	0.00	0.00	0.00	0.11	0.00	0.12
GPX4	0.00	0.00	0.00	0.13	0.00	0.16	0.00	0.16	0.11	0.00	0.00	0.14	0.00	0.00	0.14	0.00	0.00	0.00	0.18	0.00	0.16
FTL	0.00	0.00	0.00	0.17	0.00	0.19	0.00	0.11	0.13	0.00	0.00	0.16	0.00	0.00	0.20	0.00	0.00	0.00	0.22	0.00	0.10
IDI2	0.00	0.00	0.00	0.25	0.00	0.48	0.00	0.17	0.11	0.00	0.00	0.23	0.00	0.00	0.15	0.00	0.00	0.00	0.29	0.00	0.14
TDP1	0.00	0.00	0.00	0.15	0.00	0.25	0.00	0.20	0.20	0.00	0.00	0.12	0.00	0.00	0.17	0.00	0.00	0.00	0.17	0.00	0.13

APTX	0.00	0.00	0.00	0.14	0.00	0.25	0.00	0.15	0.19	0.00	0.00	0.15	0.00	0.00	0.20	0.00	0.00	0.00	0.15	0.00	0.14
FECH	0.00	0.00	0.00	0.12	0.00	0.14	0.00	0.12	0.13	0.00	0.00	0.11	0.00	0.00	0.12	0.00	0.00	0.00	0.28	0.00	0.17
MMACHC	0.00	0.00	0.00	0.16	0.00	0.25	0.00	0.12	0.19	0.00	0.00	0.14	0.00	0.00	0.17	0.00	0.00	0.00	0.28	0.00	0.15
DPYS	0.00	0.00	0.00	0.19	0.00	0.33	0.00	0.14	0.15	0.00	0.00	0.27	0.00	0.00	0.24	0.00	0.00	0.00	0.31	0.00	0.11
ALDH18A1	0.00	0.00	0.00	0.18	0.00	0.33	0.00	0.12	0.15	0.00	0.00	0.27	0.00	0.00	0.17	0.00	0.00	0.00	0.32	0.00	0.17
ABCD4	0.00	0.00	0.00	0.24	0.00	0.42	0.10	0.00	0.00	0.00	0.19	0.24	0.00	0.00	0.20	0.00	0.00	0.00	0.21	0.00	0.14
PEX26	0.00	0.00	0.00	0.26	0.00	0.41	0.12	0.00	0.00	0.00	0.17	0.13	0.00	0.00	0.16	0.00	0.00	0.00	0.17	0.00	0.25
SLC25A28	0.00	0.00	0.00	0.25	0.00	0.48	0.00	0.15	0.00	0.00	0.14	0.21	0.00	0.00	0.19	0.00	0.00	0.00	0.26	0.00	0.33
TSPO2	0.00	0.00	0.00	0.28	0.00	0.56	0.00	0.20	0.00	0.00	0.21	0.17	0.00	0.00	0.12	0.00	0.00	0.00	0.21	0.00	0.15
ATP11B	0.00	0.00	0.00	0.31	0.00	0.60	0.00	0.12	0.00	0.00	0.27	0.22	0.00	0.00	0.17	0.00	0.00	0.00	0.11	0.00	0.11
DOLK	0.00	0.00	0.00	0.26	0.00	0.49	0.00	0.14	0.00	0.00	0.17	0.21	0.00	0.00	0.16	0.00	0.00	0.00	0.25	0.00	0.12
CYB5R3	0.00	0.00	0.00	0.15	0.00	0.26	0.00	0.11	0.00	0.00	0.00	0.17	0.11	0.00	0.16	0.00	0.00	0.00	0.23	0.00	0.16
MSS51	0.00	0.00	0.00	0.23	0.00	0.45	0.00	0.13	0.00	0.00	0.00	0.16	0.10	0.00	0.14	0.00	0.00	0.00	0.17	0.00	0.32
ALDH3B1	0.00	0.00	0.00	0.23	0.00	0.36	0.00	0.12	0.00	0.00	0.00	0.23	0.14	0.00	0.16	0.00	0.00	0.00	0.29	0.00	0.11
ATP13A2	0.00	0.00	0.00	0.12	0.00	0.21	0.00	0.00	0.14	0.00	0.00	0.18	0.13	0.00	0.18	0.00	0.00	0.00	0.10	0.00	0.11
PARK2	0.00	0.00	0.00	0.10	0.00	0.00	0.00	0.11	0.14	0.00	0.00	0.12	0.10	0.00	0.13	0.00	0.00	0.00	0.10	0.00	0.16
TSEN34	0.00	0.00	0.00	0.16	0.00	0.40	0.00	0.10	0.00	0.00	0.00	0.18	0.00	0.00	0.22	0.11	0.00	0.00	0.14	0.00	0.13
SCAX3	0.00	0.00	0.00	0.00	0.00	0.00	0.00	0.00	0.10	0.00	0.00	0.27	0.27	0.21	0.33	0.32	0.00	0.00	0.11	0.00	0.11
MTHFD1L	0.00	0.00	0.00	0.17	0.00	0.32	0.00	0.15	0.00	0.00	0.00	0.18	0.00	0.00	0.14	0.00	0.17	0.00	0.31	0.00	0.20
FOXRED1	0.00	0.00	0.00	0.26	0.00	0.50	0.00	0.21	0.15	0.00	0.00	0.16	0.00	0.00	0.00	0.00	0.00	0.12	0.17	0.00	0.35
OMA1	0.00	0.00	0.00	0.20	0.00	0.44	0.00	0.14	0.00	0.00	0.18	0.17	0.00	0.00	0.00	0.00	0.00	0.17	0.15	0.00	0.52
ATG12	0.00	0.00	0.00	0.24	0.00	0.33	0.12	0.28	0.00	0.00	0.00	0.00	0.00	0.00	0.15	0.00	0.00	0.14	0.15	0.00	0.27
MMADHC	0.00	0.00	0.00	0.30	0.00	0.51	0.12	0.00	0.00	0.00	0.00	0.25	0.00	0.00	0.25	0.00	0.00	0.12	0.34	0.00	0.20
SSBP1	0.00	0.00	0.00	0.21	0.00	0.41	0.00	0.25	0.00	0.00	0.00	0.14	0.00	0.00	0.19	0.00	0.00	0.15	0.18	0.00	0.28
KARS	0.00	0.00	0.00	0.24	0.00	0.38	0.00	0.26	0.00	0.00	0.00	0.14	0.00	0.00	0.22	0.00	0.00	0.15	0.17	0.00	0.21
TRMU	0.00	0.00	0.00	0.15	0.00	0.40	0.00	0.11	0.00	0.00	0.00	0.12	0.00	0.00	0.10	0.00	0.00	0.16	0.21	0.00	0.39
TUFM	0.00	0.00	0.00	0.21	0.00	0.47	0.00	0.12	0.00	0.00	0.00	0.16	0.00	0.00	0.14	0.00	0.00	0.17	0.21	0.00	0.40
IBA57	0.00	0.00	0.00	0.13	0.00	0.37	0.00	0.11	0.00	0.00	0.00	0.14	0.00	0.00	0.11	0.00	0.00	0.11	0.38	0.00	0.40
SCO1	0.00	0.00	0.00	0.20	0.00	0.41	0.00	0.12	0.00	0.00	0.00	0.14	0.00	0.00	0.15	0.00	0.00	0.15	0.21	0.00	0.40
GTPBP3	0.00	0.00	0.00	0.27	0.00	0.55	0.00	0.17	0.00	0.00	0.00	0.19	0.00	0.00	0.12	0.00	0.00	0.17	0.21	0.00	0.41
MTIF3	0.00	0.00	0.00	0.22	0.00	0.44	0.00	0.13	0.00	0.00	0.00	0.17	0.00	0.00	0.18	0.00	0.00	0.15	0.18	0.00	0.40
PRPS1	0.00	0.00	0.00	0.17	0.00	0.32	0.00	0.11	0.00	0.00	0.00	0.21	0.00	0.00	0.19	0.00	0.00	0.11	0.25	0.00	0.10
NDUFS6	0.00	0.00	0.00	0.25	0.00	0.51	0.00	0.18	0.00	0.00	0.00	0.14	0.00	0.00	0.14	0.00	0.00	0.14	0.14	0.00	0.37
TSFM	0.00	0.00	0.00	0.20	0.00	0.47	0.00	0.11	0.00	0.00	0.00	0.20	0.00	0.00	0.17	0.00	0.00	0.17	0.24	0.00	0.38
NFU1	0.00	0.00	0.00	0.22	0.00	0.50	0.00	0.15	0.00	0.00	0.00	0.17	0.00	0.00	0.18	0.00	0.00	0.16	0.28	0.00	0.38
ATG4D	0.00	0.00	0.00	0.23	0.00	0.43	0.00	0.23	0.00	0.00	0.00	0.14	0.00	0.00	0.10	0.00	0.00	0.11	0.16	0.00	0.41
NDUFAF1	0.00	0.00	0.00	0.22	0.00	0.43	0.00	0.13	0.00	0.00	0.00	0.12	0.00	0.00	0.17	0.00	0.00	0.15	0.18	0.00	0.43
PTCD3	0.00	0.00	0.00	0.26	0.00	0.63	0.00	0.24	0.00	0.00	0.00	0.17	0.00	0.00	0.11	0.00	0.00	0.18	0.25	0.00	0.52
FASTKD2	0.00	0.00	0.00	0.29	0.00	0.54	0.00	0.35	0.00	0.00	0.00	0.18	0.00	0.00	0.11	0.00	0.00	0.21	0.22	0.00	0.53
PTCD1	0.00	0.00	0.00	0.22	0.00	0.56	0.00	0.24	0.00	0.00	0.00	0.14	0.00	0.00	0.10	0.00	0.00	0.18	0.27	0.00	0.54

IARS2	0.00	0.00	0.00	0.18	0.00	0.41	0.00	0.10	0.00	0.00	0.00	0.15	0.00	0.00	0.14	0.00	0.00	0.20	0.23	0.00	0.52
NDUFS7	0.00	0.00	0.00	0.20	0.00	0.37	0.00	0.13	0.00	0.00	0.00	0.13	0.00	0.00	0.13	0.00	0.00	0.20	0.24	0.00	0.50
COX5B	0.00	0.00	0.00	0.24	0.00	0.42	0.00	0.18	0.00	0.00	0.00	0.17	0.00	0.00	0.13	0.00	0.00	0.14	0.23	0.00	0.44
GFM2	0.00	0.00	0.00	0.16	0.00	0.47	0.00	0.11	0.00	0.00	0.00	0.15	0.00	0.00	0.12	0.00	0.00	0.19	0.22	0.00	0.45
LYRM4	0.00	0.00	0.00	0.24	0.00	0.53	0.00	0.14	0.00	0.00	0.00	0.17	0.00	0.00	0.14	0.00	0.00	0.16	0.26	0.00	0.49
SMCR7	0.00	0.00	0.00	0.18	0.00	0.38	0.00	0.10	0.00	0.00	0.00	0.21	0.00	0.00	0.19	0.00	0.00	0.15	0.14	0.00	0.37
NDUFA9	0.00	0.00	0.00	0.26	0.00	0.45	0.00	0.13	0.00	0.00	0.00	0.19	0.00	0.00	0.16	0.00	0.00	0.18	0.23	0.00	0.49
ATPAF2	0.00	0.00	0.00	0.21	0.00	0.49	0.00	0.11	0.00	0.00	0.00	0.15	0.00	0.00	0.16	0.00	0.00	0.16	0.19	0.00	0.36
GGTLC1	0.00	0.00	0.00	0.26	0.00	0.45	0.00	0.14	0.00	0.00	0.00	0.24	0.00	0.00	0.14	0.00	0.00	0.12	0.34	0.00	0.11
HSCB	0.00	0.00	0.00	0.27	0.00	0.58	0.00	0.18	0.00	0.00	0.00	0.18	0.00	0.00	0.13	0.00	0.00	0.11	0.18	0.00	0.34
SARS2	0.00	0.00	0.00	0.23	0.00	0.46	0.00	0.20	0.00	0.00	0.00	0.15	0.00	0.00	0.17	0.00	0.00	0.10	0.15	0.00	0.34
MCCC2	0.00	0.00	0.00	0.13	0.00	0.28	0.00	0.00	0.10	0.00	0.00	0.18	0.00	0.00	0.19	0.00	0.00	0.11	0.22	0.00	0.17
NDUFB8	0.00	0.00	0.00	0.21	0.00	0.40	0.00	0.00	0.14	0.00	0.00	0.19	0.00	0.00	0.12	0.00	0.00	0.13	0.18	0.00	0.33
D2HGDH	0.00	0.00	0.00	0.11	0.00	0.19	0.00	0.00	0.13	0.00	0.00	0.20	0.00	0.00	0.14	0.00	0.00	0.13	0.32	0.00	0.25
NAGS	0.00	0.00	0.00	0.15	0.00	0.32	0.00	0.00	0.14	0.00	0.00	0.20	0.00	0.00	0.14	0.00	0.00	0.19	0.38	0.00	0.24
ETFDH	0.00	0.00	0.00	0.17	0.00	0.26	0.00	0.00	0.12	0.00	0.00	0.14	0.00	0.00	0.12	0.00	0.00	0.24	0.35	0.00	0.31
OPA1	0.00	0.00	0.00	0.11	0.00	0.16	0.00	0.00	0.11	0.00	0.00	0.11	0.00	0.00	0.17	0.00	0.00	0.15	0.14	0.00	0.36
MUT	0.00	0.00	0.00	0.15	0.00	0.24	0.00	0.00	0.11	0.00	0.00	0.16	0.00	0.00	0.19	0.00	0.00	0.13	0.32	0.00	0.17
MCEE	0.00	0.00	0.00	0.16	0.00	0.34	0.00	0.00	0.10	0.00	0.00	0.14	0.00	0.00	0.14	0.00	0.00	0.13	0.36	0.00	0.16
YME1L1	0.00	0.00	0.00	0.30	0.00	0.66	0.00	0.00	0.00	0.00	0.19	0.24	0.00	0.00	0.14	0.00	0.00	0.18	0.22	0.00	0.53
MNF1	0.00	0.00	0.00	0.26	0.00	0.56	0.00	0.00	0.00	0.00	0.10	0.24	0.00	0.00	0.12	0.00	0.00	0.22	0.23	0.00	0.71
YARS2	0.00	0.00	0.00	0.20	0.00	0.40	0.00	0.00	0.00	0.00	0.10	0.18	0.00	0.00	0.10	0.00	0.00	0.22	0.27	0.00	0.49
UGP1	0.00	0.00	0.00	0.23	0.00	0.41	0.00	0.00	0.00	0.00	0.14	0.21	0.00	0.00	0.13	0.00	0.00	0.10	0.30	0.00	0.13
TIMM8A	0.00	0.00	0.00	0.26	0.00	0.44	0.00	0.00	0.00	0.00	0.14	0.22	0.00	0.00	0.23	0.00	0.00	0.13	0.14	0.00	0.37
TIMM13	0.00	0.00	0.00	0.28	0.00	0.49	0.00	0.00	0.00	0.00	0.19	0.21	0.00	0.00	0.21	0.00	0.00	0.19	0.16	0.00	0.52
TIMM8B	0.00	0.00	0.00	0.23	0.00	0.46	0.00	0.00	0.00	0.00	0.20	0.19	0.00	0.00	0.17	0.00	0.00	0.18	0.15	0.00	0.50
MIPEP	0.00	0.00	0.00	0.22	0.00	0.47	0.00	0.00	0.00	0.00	0.11	0.25	0.00	0.00	0.18	0.00	0.00	0.11	0.23	0.00	0.35
NDUFV2	0.00	0.00	0.00	0.14	0.00	0.25	0.00	0.00	0.00	0.00	0.00	0.13	0.00	0.00	0.12	0.00	0.18	0.11	0.18	0.00	0.29
POLG	0.00	0.00	0.00	0.00	0.00	0.11	0.00	0.00	0.16	0.00	0.00	0.11	0.00	0.00	0.13	0.00	0.11	0.19	0.18	0.00	0.30
MT1XP1	0.00	0.00	0.00	0.00	0.00	0.18	0.00	0.00	0.00	0.00	0.00	0.15	0.00	0.00	0.14	0.13	0.12	0.19	0.18	0.00	0.53
APIP	0.00	0.00	0.00	0.20	0.00	0.33	0.00	0.15	0.15	0.00	0.00	0.16	0.00	0.00	0.00	0.00	0.11	0.00	0.00	0.12	0.39
MAP3K5	0.00	0.00	0.00	0.14	0.00	0.14	0.00	0.19	0.22	0.00	0.00	0.00	0.00	0.00	0.13	0.00	0.11	0.00	0.00	0.12	0.11
FASTKD1	0.00	0.00	0.00	0.22	0.00	0.49	0.00	0.12	0.00	0.00	0.00	0.17	0.00	0.00	0.00	0.00	0.00	0.34	0.28	0.10	0.73
FASTKD3	0.00	0.00	0.00	0.22	0.00	0.49	0.00	0.12	0.00	0.00	0.00	0.17	0.00	0.00	0.00	0.00	0.00	0.34	0.28	0.10	0.73
FASTKD5	0.00	0.00	0.00	0.22	0.00	0.49	0.00	0.12	0.00	0.00	0.00	0.17	0.00	0.00	0.00	0.00	0.00	0.34	0.28	0.10	0.73
TFB1M	0.00	0.00	0.00	0.22	0.00	0.37	0.00	0.25	0.00	0.00	0.00	0.00	0.00	0.00	0.00	0.00	0.22	0.24	0.22	0.18	0.36
SUCLA2	0.00	0.00	0.00	0.19	0.00	0.39	0.00	0.00	0.00	0.00	0.00	0.22	0.00	0.00	0.00	0.00	0.13	0.25	0.37	0.21	0.44
MTPAP	0.00	0.00	0.00	0.18	0.00	0.41	0.00	0.00	0.00	0.00	0.00	0.12	0.00	0.00	0.00	0.00	0.21	0.16	0.24	0.11	0.32
TUFMP1	0.00	0.00	0.00	0.20	0.00	0.56	0.00	0.00	0.00	0.00	0.00	0.13	0.00	0.00	0.00	0.00	0.18	0.11	0.11	0.11	0.44
ND5	0.00	0.00	0.00	0.00	0.00	0.21	0.00	0.00	0.00	0.00	0.00	0.13	0.00	0.00	0.12	0.00	0.18	0.21	0.21	0.13	0.41

ND1	0.00	0.00	0.00	0.00	0.00	0.17	0.00	0.00	0.00	0.00	0.00	0.12	0.00	0.00	0.13	0.00	0.27	0.23	0.21	0.18	0.38
HHC3	0.17	0.17	0.26	0.27	0.16	0.19	0.21	0.00	0.00	0.00	0.00	0.00	0.00	0.00	0.00	0.00	0.00	0.00	0.00	0.00	0.00
JKAMP	0.00	0.00	0.15	0.30	0.00	0.39	0.17	0.24	0.11	0.00	0.19	0.00	0.00	0.00	0.00	0.00	0.00	0.00	0.00	0.00	0.00
PKD2	0.00	0.00	0.12	0.16	0.00	0.13	0.11	0.15	0.18	0.00	0.13	0.00	0.00	0.00	0.00	0.00	0.00	0.00	0.00	0.00	0.00
MIR616	0.00	0.00	0.10	0.28	0.00	0.34	0.17	0.48	0.32	0.00	0.00	0.11	0.00	0.00	0.00	0.00	0.00	0.00	0.00	0.00	0.00
AP1AR	0.00	0.00	0.16	0.32	0.00	0.35	0.21	0.19	0.00	0.00	0.13	0.11	0.00	0.00	0.00	0.00	0.00	0.00	0.00	0.00	0.00
ANKS6	0.00	0.00	0.11	0.21	0.00	0.30	0.11	0.00	0.12	0.00	0.12	0.11	0.00	0.00	0.00	0.00	0.00	0.00	0.00	0.00	0.00
DRD1	0.14	0.14	0.15	0.14	0.00	0.00	0.00	0.00	0.11	0.00	0.00	0.00	0.15	0.11	0.00	0.00	0.00	0.00	0.00	0.00	0.00
NSD1	0.00	0.00	0.12	0.25	0.00	0.26	0.17	0.27	0.14	0.00	0.00	0.00	0.00	0.00	0.15	0.00	0.00	0.00	0.00	0.00	0.00
RPS19	0.00	0.00	0.17	0.29	0.00	0.28	0.19	0.28	0.21	0.00	0.00	0.00	0.00	0.00	0.17	0.00	0.00	0.00	0.00	0.00	0.00
GPS2	0.00	0.00	0.24	0.39	0.00	0.31	0.30	0.45	0.15	0.00	0.00	0.00	0.00	0.00	0.16	0.00	0.00	0.00	0.00	0.00	0.00
TRPS1	0.00	0.00	0.10	0.25	0.00	0.30	0.15	0.35	0.18	0.00	0.00	0.00	0.00	0.00	0.14	0.00	0.00	0.00	0.00	0.00	0.00
TTC21B	0.00	0.00	0.12	0.25	0.00	0.30	0.16	0.34	0.12	0.00	0.00	0.00	0.00	0.00	0.14	0.00	0.00	0.00	0.00	0.00	0.00
SOS1	0.00	0.00	0.16	0.21	0.00	0.14	0.16	0.15	0.11	0.00	0.00	0.00	0.00	0.00	0.15	0.00	0.00	0.00	0.00	0.00	0.00
PSMF1	0.00	0.00	0.10	0.27	0.00	0.34	0.15	0.37	0.16	0.00	0.00	0.00	0.00	0.00	0.19	0.00	0.00	0.00	0.00	0.00	0.00
UNC13D	0.00	0.00	0.11	0.21	0.00	0.18	0.16	0.26	0.12	0.00	0.00	0.00	0.00	0.00	0.15	0.00	0.00	0.00	0.00	0.00	0.00
IGFBP4	0.00	0.00	0.11	0.19	0.00	0.15	0.15	0.16	0.13	0.00	0.00	0.00	0.00	0.00	0.16	0.00	0.00	0.00	0.00	0.00	0.00
TBC1D5	0.00	0.00	0.17	0.34	0.00	0.41	0.22	0.16	0.00	0.00	0.16	0.00	0.00	0.00	0.11	0.00	0.00	0.00	0.00	0.00	0.00
VPS4B	0.00	0.00	0.16	0.31	0.00	0.37	0.19	0.23	0.00	0.00	0.14	0.00	0.00	0.00	0.14	0.00	0.00	0.00	0.00	0.00	0.00
CHMP2A	0.00	0.00	0.13	0.29	0.00	0.39	0.16	0.25	0.00	0.00	0.12	0.00	0.00	0.00	0.12	0.00	0.00	0.00	0.00	0.00	0.00
VPS4A	0.00	0.00	0.11	0.25	0.00	0.33	0.13	0.25	0.00	0.00	0.11	0.00	0.00	0.00	0.14	0.00	0.00	0.00	0.00	0.00	0.00
NPLOC4	0.00	0.00	0.17	0.34	0.00	0.42	0.19	0.24	0.00	0.00	0.16	0.00	0.00	0.00	0.15	0.00	0.00	0.00	0.00	0.00	0.00
SNF8	0.00	0.00	0.21	0.38	0.00	0.43	0.26	0.27	0.00	0.00	0.12	0.00	0.00	0.00	0.14	0.00	0.00	0.00	0.00	0.00	0.00
RAPSN	0.00	0.00	0.13	0.26	0.00	0.18	0.22	0.15	0.00	0.00	0.16	0.00	0.00	0.00	0.23	0.00	0.00	0.00	0.00	0.00	0.00
CEACAM16	0.00	0.00	0.15	0.32	0.00	0.40	0.21	0.17	0.00	0.00	0.26	0.00	0.00	0.00	0.17	0.00	0.00	0.00	0.00	0.00	0.00
VPS39	0.00	0.00	0.21	0.42	0.00	0.43	0.30	0.30	0.00	0.00	0.15	0.00	0.00	0.00	0.16	0.00	0.00	0.00	0.00	0.00	0.00
RAB9A	0.00	0.00	0.14	0.29	0.00	0.34	0.18	0.25	0.00	0.00	0.12	0.00	0.00	0.00	0.11	0.00	0.00	0.00	0.00	0.00	0.00
VPS28	0.00	0.00	0.19	0.35	0.00	0.36	0.25	0.29	0.00	0.00	0.12	0.00	0.00	0.00	0.13	0.00	0.00	0.00	0.00	0.00	0.00
CHRNE	0.00	0.00	0.14	0.25	0.00	0.13	0.21	0.00	0.10	0.00	0.16	0.00	0.00	0.00	0.19	0.00	0.00	0.00	0.00	0.00	0.00
NPHP4	0.00	0.00	0.10	0.21	0.00	0.30	0.11	0.00	0.12	0.00	0.11	0.00	0.00	0.00	0.15	0.00	0.00	0.00	0.00	0.00	0.00
TRPV4	0.00	0.00	0.15	0.19	0.00	0.00	0.15	0.14	0.21	0.00	0.15	0.00	0.00	0.00	0.19	0.00	0.00	0.00	0.00	0.00	0.00
BMPR2	0.00	0.00	0.12	0.18	0.00	0.00	0.16	0.16	0.20	0.00	0.10	0.00	0.00	0.00	0.15	0.00	0.00	0.00	0.00	0.00	0.00
SGCA	0.00	0.00	0.00	0.16	0.00	0.16	0.11	0.13	0.23	0.00	0.13	0.00	0.00	0.00	0.15	0.00	0.00	0.00	0.00	0.00	0.00
CR1	0.00	0.00	0.00	0.17	0.00	0.11	0.14	0.13	0.17	0.00	0.11	0.00	0.00	0.00	0.13	0.00	0.00	0.00	0.00	0.00	0.00
TMPRSS3	0.00	0.00	0.00	0.21	0.00	0.27	0.11	0.19	0.17	0.00	0.19	0.00	0.00	0.00	0.14	0.00	0.00	0.00	0.00	0.00	0.00
DYSF	0.00	0.00	0.00	0.17	0.00	0.17	0.11	0.13	0.26	0.00	0.12	0.00	0.00	0.00	0.16	0.00	0.00	0.00	0.00	0.00	0.00
ARIH1	0.00	0.00	0.13	0.33	0.00	0.44	0.18	0.28	0.00	0.00	0.00	0.16	0.00	0.00	0.18	0.00	0.00	0.00	0.00	0.00	0.00
EPS15	0.00	0.00	0.20	0.33	0.00	0.26	0.26	0.18	0.00	0.00	0.00	0.15	0.00	0.00	0.16	0.00	0.00	0.00	0.00	0.00	0.00
PRPF40A	0.00	0.00	0.12	0.31	0.00	0.41	0.17	0.19	0.00	0.00	0.00	0.14	0.00	0.00	0.21	0.00	0.00	0.00	0.00	0.00	0.00
MED13L	0.00	0.00	0.16	0.38	0.00	0.55	0.21	0.21	0.00	0.00	0.00	0.24	0.00	0.00	0.28	0.00	0.00	0.00	0.00	0.00	0.00

ARHGAP10	0.00	0.00	0.13	0.26	0.00	0.35	0.15	0.19	0.00	0.00	0.00	0.14	0.00	0.00	0.12	0.00	0.00	0.00	0.00	0.00	0.00
AKAP8L	0.00	0.00	0.12	0.35	0.00	0.52	0.16	0.26	0.00	0.00	0.00	0.16	0.00	0.00	0.18	0.00	0.00	0.00	0.00	0.00	0.00
SAP30	0.00	0.00	0.13	0.28	0.00	0.33	0.17	0.33	0.00	0.00	0.00	0.11	0.00	0.00	0.17	0.00	0.00	0.00	0.00	0.00	0.00
ITSN1	0.00	0.00	0.19	0.33	0.00	0.32	0.23	0.21	0.00	0.00	0.00	0.24	0.00	0.00	0.22	0.00	0.00	0.00	0.00	0.00	0.00
ATXN2L	0.00	0.00	0.14	0.30	0.00	0.41	0.18	0.15	0.00	0.00	0.00	0.16	0.00	0.00	0.15	0.00	0.00	0.00	0.00	0.00	0.00
ARHGAP42	0.00	0.00	0.13	0.32	0.00	0.45	0.17	0.21	0.00	0.00	0.00	0.15	0.00	0.00	0.17	0.00	0.00	0.00	0.00	0.00	0.00
RASGEF1B	0.00	0.00	0.18	0.35	0.00	0.46	0.20	0.22	0.00	0.00	0.00	0.19	0.00	0.00	0.20	0.00	0.00	0.00	0.00	0.00	0.00
MYO9A	0.00	0.00	0.12	0.30	0.00	0.46	0.14	0.19	0.00	0.00	0.00	0.20	0.00	0.00	0.21	0.00	0.00	0.00	0.00	0.00	0.00
DOCK5	0.00	0.00	0.10	0.24	0.00	0.37	0.11	0.13	0.00	0.00	0.00	0.14	0.00	0.00	0.19	0.00	0.00	0.00	0.00	0.00	0.00
NTF6A	0.00	0.00	0.17	0.31	0.00	0.38	0.20	0.14	0.00	0.00	0.00	0.11	0.00	0.00	0.19	0.00	0.00	0.00	0.00	0.00	0.00
EDARADD	0.00	0.00	0.15	0.28	0.00	0.24	0.21	0.18	0.00	0.00	0.00	0.10	0.00	0.00	0.19	0.00	0.00	0.00	0.00	0.00	0.00
PSMC5	0.00	0.00	0.14	0.30	0.00	0.33	0.19	0.30	0.00	0.00	0.00	0.10	0.00	0.00	0.18	0.00	0.00	0.00	0.00	0.00	0.00
ANKMY2	0.00	0.00	0.14	0.24	0.00	0.31	0.13	0.14	0.00	0.00	0.00	0.13	0.00	0.00	0.19	0.00	0.00	0.00	0.00	0.00	0.00
FHL5	0.00	0.00	0.14	0.32	0.00	0.42	0.18	0.31	0.00	0.00	0.00	0.14	0.00	0.00	0.17	0.00	0.00	0.00	0.00	0.00	0.00
TRIM31	0.00	0.00	0.11	0.32	0.00	0.52	0.14	0.29	0.00	0.00	0.00	0.16	0.00	0.00	0.16	0.00	0.00	0.00	0.00	0.00	0.00
FGD2	0.00	0.00	0.13	0.30	0.00	0.43	0.15	0.11	0.00	0.00	0.00	0.17	0.00	0.00	0.24	0.00	0.00	0.00	0.00	0.00	0.00
CBX4	0.00	0.00	0.10	0.23	0.00	0.27	0.14	0.37	0.00	0.00	0.00	0.14	0.00	0.00	0.21	0.00	0.00	0.00	0.00	0.00	0.00
PIK3R4	0.00	0.00	0.18	0.36	0.00	0.41	0.23	0.27	0.00	0.00	0.00	0.12	0.00	0.00	0.12	0.00	0.00	0.00	0.00	0.00	0.00
TBX10	0.00	0.00	0.13	0.27	0.00	0.32	0.18	0.16	0.00	0.00	0.00	0.17	0.00	0.00	0.22	0.00	0.00	0.00	0.00	0.00	0.00
OFC2	0.00	0.00	0.11	0.25	0.00	0.37	0.15	0.11	0.00	0.00	0.00	0.13	0.00	0.00	0.14	0.00	0.00	0.00	0.00	0.00	0.00
RAB5C	0.00	0.00	0.11	0.30	0.00	0.39	0.17	0.22	0.00	0.00	0.00	0.11	0.00	0.00	0.11	0.00	0.00	0.00	0.00	0.00	0.00
ERI3	0.00	0.00	0.12	0.36	0.00	0.50	0.20	0.27	0.00	0.00	0.00	0.21	0.00	0.00	0.21	0.00	0.00	0.00	0.00	0.00	0.00
IPCEF1	0.00	0.00	0.19	0.34	0.00	0.46	0.19	0.26	0.00	0.00	0.00	0.17	0.00	0.00	0.20	0.00	0.00	0.00	0.00	0.00	0.00
SH3GL1	0.00	0.00	0.17	0.36	0.00	0.41	0.23	0.28	0.00	0.00	0.00	0.25	0.00	0.00	0.25	0.00	0.00	0.00	0.00	0.00	0.00
ZNF214	0.00	0.00	0.10	0.26	0.00	0.37	0.13	0.12	0.00	0.00	0.00	0.22	0.00	0.00	0.21	0.00	0.00	0.00	0.00	0.00	0.00
RILP	0.00	0.00	0.14	0.32	0.00	0.38	0.20	0.28	0.00	0.00	0.00	0.13	0.00	0.00	0.17	0.00	0.00	0.00	0.00	0.00	0.00
DMRTB1	0.00	0.00	0.11	0.35	0.00	0.60	0.15	0.23	0.00	0.00	0.00	0.29	0.00	0.00	0.30	0.00	0.00	0.00	0.00	0.00	0.00
PSMB4	0.00	0.00	0.10	0.31	0.00	0.42	0.17	0.21	0.00	0.00	0.00	0.18	0.00	0.00	0.21	0.00	0.00	0.00	0.00	0.00	0.00
RAB39A	0.00	0.00	0.10	0.32	0.00	0.52	0.15	0.14	0.00	0.00	0.00	0.25	0.00	0.00	0.21	0.00	0.00	0.00	0.00	0.00	0.00
PSMC4	0.00	0.00	0.12	0.34	0.00	0.48	0.18	0.30	0.00	0.00	0.00	0.16	0.00	0.00	0.23	0.00	0.00	0.00	0.00	0.00	0.00
ARL8B	0.00	0.00	0.11	0.33	0.00	0.53	0.14	0.34	0.00	0.00	0.00	0.18	0.00	0.00	0.20	0.00	0.00	0.00	0.00	0.00	0.00
FGD3	0.00	0.00	0.13	0.30	0.00	0.46	0.13	0.16	0.00	0.00	0.00	0.16	0.00	0.00	0.21	0.00	0.00	0.00	0.00	0.00	0.00
USP31	0.00	0.00	0.10	0.31	0.00	0.47	0.15	0.12	0.00	0.00	0.00	0.23	0.00	0.00	0.16	0.00	0.00	0.00	0.00	0.00	0.00
ZFAND5	0.00	0.00	0.15	0.37	0.00	0.48	0.22	0.26	0.00	0.00	0.00	0.13	0.00	0.00	0.17	0.00	0.00	0.00	0.00	0.00	0.00
TTC8	0.00	0.00	0.11	0.24	0.00	0.36	0.12	0.12	0.00	0.00	0.00	0.20	0.00	0.00	0.25	0.00	0.00	0.00	0.00	0.00	0.00
ZC4H2	0.00	0.00	0.11	0.32	0.00	0.51	0.13	0.29	0.00	0.00	0.00	0.18	0.00	0.00	0.19	0.00	0.00	0.00	0.00	0.00	0.00
STON1	0.00	0.00	0.15	0.35	0.00	0.43	0.22	0.16	0.00	0.00	0.00	0.23	0.00	0.00	0.24	0.00	0.00	0.00	0.00	0.00	0.00
PSMC3	0.00	0.00	0.15	0.35	0.00	0.42	0.21	0.34	0.00	0.00	0.00	0.12	0.00	0.00	0.20	0.00	0.00	0.00	0.00	0.00	0.00
RILPL1	0.00	0.00	0.12	0.30	0.00	0.40	0.17	0.30	0.00	0.00	0.00	0.20	0.00	0.00	0.20	0.00	0.00	0.00	0.00	0.00	0.00
RERE	0.00	0.00	0.16	0.35	0.00	0.46	0.21	0.31	0.00	0.00	0.00	0.17	0.00	0.00	0.24	0.00	0.00	0.00	0.00	0.00	0.00

ARFIP2	0.00	0.00	0.15	0.29	0.00	0.39	0.16	0.23	0.00	0.00	0.00	0.18	0.00	0.00	0.17	0.00	0.00	0.00	0.00	0.00	0.00
TUBA3C	0.00	0.00	0.14	0.32	0.00	0.42	0.18	0.23	0.00	0.00	0.00	0.21	0.00	0.00	0.26	0.00	0.00	0.00	0.00	0.00	0.00
ZFP64	0.00	0.00	0.13	0.35	0.00	0.48	0.19	0.25	0.00	0.00	0.00	0.21	0.00	0.00	0.22	0.00	0.00	0.00	0.00	0.00	0.00
CCT5	0.00	0.00	0.11	0.28	0.00	0.40	0.13	0.24	0.00	0.00	0.00	0.19	0.00	0.00	0.21	0.00	0.00	0.00	0.00	0.00	0.00
AASTH38	0.00	0.00	0.15	0.22	0.00	0.20	0.18	0.00	0.12	0.00	0.00	0.11	0.00	0.00	0.15	0.00	0.00	0.00	0.00	0.00	0.00
TAAR9	0.00	0.00	0.20	0.25	0.00	0.15	0.22	0.00	0.15	0.00	0.00	0.12	0.00	0.00	0.17	0.00	0.00	0.00	0.00	0.00	0.00
PPP2R4	0.00	0.00	0.00	0.20	0.00	0.25	0.11	0.29	0.17	0.00	0.00	0.15	0.00	0.00	0.12	0.00	0.00	0.00	0.00	0.00	0.00
IFT74	0.00	0.00	0.00	0.29	0.00	0.49	0.12	0.30	0.11	0.00	0.00	0.15	0.00	0.00	0.18	0.00	0.00	0.00	0.00	0.00	0.00
SAMHD1	0.00	0.00	0.00	0.22	0.00	0.34	0.11	0.26	0.19	0.00	0.00	0.12	0.00	0.00	0.18	0.00	0.00	0.00	0.00	0.00	0.00
HECW1	0.00	0.00	0.00	0.25	0.00	0.35	0.14	0.34	0.14	0.00	0.00	0.18	0.00	0.00	0.24	0.00	0.00	0.00	0.00	0.00	0.00
TGM1	0.00	0.00	0.00	0.18	0.00	0.18	0.12	0.17	0.17	0.00	0.00	0.11	0.00	0.00	0.16	0.00	0.00	0.00	0.00	0.00	0.00
ANG	0.00	0.00	0.00	0.17	0.00	0.15	0.11	0.21	0.26	0.00	0.00	0.11	0.00	0.00	0.18	0.00	0.00	0.00	0.00	0.00	0.00
PDCD2	0.00	0.00	0.00	0.28	0.00	0.41	0.14	0.35	0.13	0.00	0.00	0.15	0.00	0.00	0.18	0.00	0.00	0.00	0.00	0.00	0.00
CSMD1	0.00	0.00	0.00	0.22	0.00	0.27	0.13	0.21	0.19	0.00	0.00	0.15	0.00	0.00	0.17	0.00	0.00	0.00	0.00	0.00	0.00
RRS1	0.00	0.00	0.00	0.27	0.00	0.42	0.12	0.20	0.13	0.00	0.00	0.19	0.00	0.00	0.18	0.00	0.00	0.00	0.00	0.00	0.00
RRP1B	0.00	0.00	0.00	0.31	0.00	0.49	0.14	0.41	0.21	0.00	0.00	0.15	0.00	0.00	0.15	0.00	0.00	0.00	0.00	0.00	0.00
CHD2	0.00	0.00	0.00	0.29	0.00	0.47	0.13	0.33	0.10	0.00	0.00	0.15	0.00	0.00	0.23	0.00	0.00	0.00	0.00	0.00	0.00
DAPK1	0.00	0.00	0.00	0.19	0.00	0.18	0.13	0.25	0.20	0.00	0.00	0.12	0.00	0.00	0.15	0.00	0.00	0.00	0.00	0.00	0.00
MBNL3	0.00	0.00	0.00	0.24	0.00	0.39	0.10	0.19	0.11	0.00	0.00	0.12	0.00	0.00	0.16	0.00	0.00	0.00	0.00	0.00	0.00
PNKD	0.00	0.00	0.00	0.27	0.00	0.39	0.13	0.19	0.15	0.00	0.00	0.19	0.00	0.00	0.18	0.00	0.00	0.00	0.00	0.00	0.00
STK39	0.00	0.00	0.00	0.20	0.00	0.19	0.13	0.18	0.11	0.00	0.00	0.11	0.00	0.00	0.19	0.00	0.00	0.00	0.00	0.00	0.00
UBB	0.00	0.00	0.00	0.18	0.00	0.17	0.11	0.23	0.16	0.00	0.00	0.11	0.00	0.00	0.18	0.00	0.00	0.00	0.00	0.00	0.00
CAMTA1	0.00	0.00	0.00	0.26	0.00	0.41	0.13	0.33	0.20	0.00	0.00	0.24	0.00	0.00	0.23	0.00	0.00	0.00	0.00	0.00	0.00
TCF4	0.00	0.00	0.00	0.16	0.00	0.15	0.11	0.29	0.12	0.00	0.00	0.14	0.00	0.00	0.23	0.00	0.00	0.00	0.00	0.00	0.00
NUCKS1	0.00	0.00	0.00	0.31	0.00	0.47	0.14	0.31	0.12	0.00	0.00	0.18	0.00	0.00	0.17	0.00	0.00	0.00	0.00	0.00	0.00
HSPA8	0.00	0.00	0.00	0.17	0.00	0.13	0.13	0.15	0.15	0.00	0.00	0.11	0.00	0.00	0.16	0.00	0.00	0.00	0.00	0.00	0.00
WHSC1	0.00	0.00	0.00	0.24	0.00	0.31	0.14	0.38	0.26	0.00	0.00	0.14	0.00	0.00	0.16	0.00	0.00	0.00	0.00	0.00	0.00
PRSS3	0.00	0.00	0.00	0.21	0.00	0.26	0.12	0.28	0.22	0.00	0.00	0.14	0.00	0.00	0.18	0.00	0.00	0.00	0.00	0.00	0.00
TGM3	0.00	0.00	0.00	0.19	0.00	0.25	0.10	0.23	0.19	0.00	0.00	0.12	0.00	0.00	0.15	0.00	0.00	0.00	0.00	0.00	0.00
G3BP1	0.00	0.00	0.00	0.28	0.00	0.38	0.15	0.42	0.18	0.00	0.00	0.14	0.00	0.00	0.17	0.00	0.00	0.00	0.00	0.00	0.00
ZNF124	0.00	0.00	0.00	0.30	0.00	0.49	0.14	0.26	0.13	0.00	0.00	0.21	0.00	0.00	0.19	0.00	0.00	0.00	0.00	0.00	0.00
PLDN	0.00	0.00	0.00	0.18	0.00	0.20	0.12	0.14	0.10	0.00	0.00	0.11	0.00	0.00	0.15	0.00	0.00	0.00	0.00	0.00	0.00
TAF15	0.00	0.00	0.00	0.25	0.00	0.38	0.11	0.33	0.15	0.00	0.00	0.13	0.00	0.00	0.21	0.00	0.00	0.00	0.00	0.00	0.00
UBE2K	0.00	0.00	0.00	0.26	0.00	0.35	0.14	0.31	0.13	0.00	0.00	0.18	0.00	0.00	0.20	0.00	0.00	0.00	0.00	0.00	0.00
MIR181C	0.00	0.00	0.00	0.22	0.00	0.34	0.12	0.48	0.29	0.00	0.00	0.17	0.00	0.00	0.11	0.00	0.00	0.00	0.00	0.00	0.00
CRISPLD1	0.00	0.00	0.00	0.22	0.00	0.33	0.12	0.10	0.12	0.00	0.00	0.13	0.00	0.00	0.16	0.00	0.00	0.00	0.00	0.00	0.00
PPP2R2A	0.00	0.00	0.00	0.20	0.00	0.27	0.11	0.32	0.12	0.00	0.00	0.17	0.00	0.00	0.16	0.00	0.00	0.00	0.00	0.00	0.00
MIR133B	0.00	0.00	0.00	0.24	0.00	0.30	0.14	0.49	0.33	0.00	0.00	0.13	0.00	0.00	0.13	0.00	0.00	0.00	0.00	0.00	0.00
CTSC	0.00	0.00	0.00	0.16	0.00	0.16	0.11	0.20	0.20	0.00	0.00	0.12	0.00	0.00	0.17	0.00	0.00	0.00	0.00	0.00	0.00
ATXN10	0.00	0.00	0.00	0.22	0.00	0.34	0.11	0.17	0.13	0.00	0.00	0.18	0.00	0.00	0.23	0.00	0.00	0.00	0.00	0.00	0.00

MAPK12	0.00	0.00	0.00	0.20	0.00	0.24	0.11	0.25	0.15	0.00	0.00	0.14	0.00	0.00	0.12	0.00	0.00	0.00	0.00	0.00	0.00
SETD2	0.00	0.00	0.00	0.26	0.00	0.40	0.12	0.43	0.16	0.00	0.00	0.13	0.00	0.00	0.16	0.00	0.00	0.00	0.00	0.00	0.00
FRG1	0.00	0.00	0.00	0.25	0.00	0.41	0.11	0.18	0.10	0.00	0.00	0.14	0.00	0.00	0.19	0.00	0.00	0.00	0.00	0.00	0.00
CUL9	0.00	0.00	0.00	0.25	0.00	0.39	0.12	0.37	0.13	0.00	0.00	0.12	0.00	0.00	0.18	0.00	0.00	0.00	0.00	0.00	0.00
BRK1	0.00	0.00	0.00	0.22	0.00	0.34	0.11	0.22	0.18	0.00	0.00	0.13	0.00	0.00	0.18	0.00	0.00	0.00	0.00	0.00	0.00
KLK10	0.00	0.00	0.00	0.21	0.00	0.22	0.13	0.23	0.24	0.00	0.00	0.11	0.00	0.00	0.10	0.00	0.00	0.00	0.00	0.00	0.00
EYA4	0.00	0.00	0.00	0.23	0.00	0.34	0.11	0.20	0.14	0.00	0.00	0.14	0.00	0.00	0.20	0.00	0.00	0.00	0.00	0.00	0.00
ERVW-6	0.00	0.00	0.00	0.23	0.00	0.36	0.11	0.19	0.16	0.00	0.00	0.15	0.00	0.00	0.13	0.00	0.00	0.00	0.00	0.00	0.00
HSPA7	0.00	0.00	0.00	0.27	0.00	0.41	0.13	0.24	0.17	0.00	0.00	0.18	0.00	0.00	0.21	0.00	0.00	0.00	0.00	0.00	0.00
UBE2E1	0.00	0.00	0.00	0.24	0.00	0.34	0.13	0.32	0.11	0.00	0.00	0.13	0.00	0.00	0.19	0.00	0.00	0.00	0.00	0.00	0.00
CTSF	0.00	0.00	0.00	0.26	0.00	0.39	0.13	0.19	0.13	0.00	0.00	0.20	0.00	0.00	0.18	0.00	0.00	0.00	0.00	0.00	0.00
PLS3	0.00	0.00	0.00	0.25	0.00	0.39	0.11	0.24	0.19	0.00	0.00	0.18	0.00	0.00	0.22	0.00	0.00	0.00	0.00	0.00	0.00
ATXN1L	0.00	0.00	0.00	0.24	0.00	0.36	0.13	0.25	0.17	0.00	0.00	0.20	0.00	0.00	0.23	0.00	0.00	0.00	0.00	0.00	0.00
DOCK8	0.00	0.00	0.00	0.22	0.00	0.31	0.13	0.20	0.22	0.00	0.00	0.14	0.00	0.00	0.19	0.00	0.00	0.00	0.00	0.00	0.00
FBXO7	0.00	0.00	0.00	0.26	0.00	0.39	0.13	0.34	0.16	0.00	0.00	0.12	0.00	0.00	0.16	0.00	0.00	0.00	0.00	0.00	0.00
DNMBP	0.00	0.00	0.00	0.23	0.00	0.31	0.13	0.17	0.11	0.00	0.00	0.14	0.00	0.00	0.13	0.00	0.00	0.00	0.00	0.00	0.00
CNGB1	0.00	0.00	0.11	0.25	0.00	0.34	0.12	0.00	0.00	0.00	0.12	0.18	0.00	0.00	0.20	0.00	0.00	0.00	0.00	0.00	0.00
PKD1L2	0.00	0.00	0.30	0.42	0.00	0.39	0.30	0.00	0.00	0.00	0.27	0.18	0.00	0.00	0.19	0.00	0.00	0.00	0.00	0.00	0.00
CACNA1F	0.00	0.00	0.11	0.19	0.00	0.19	0.11	0.00	0.00	0.00	0.10	0.14	0.00	0.00	0.19	0.00	0.00	0.00	0.00	0.00	0.00
GPR171	0.00	0.00	0.17	0.35	0.00	0.48	0.20	0.00	0.00	0.00	0.23	0.13	0.00	0.00	0.20	0.00	0.00	0.00	0.00	0.00	0.00
CACNG1	0.00	0.00	0.17	0.30	0.00	0.25	0.22	0.00	0.00	0.00	0.17	0.18	0.00	0.00	0.17	0.00	0.00	0.00	0.00	0.00	0.00
SGCZ	0.00	0.00	0.10	0.22	0.00	0.31	0.13	0.00	0.00	0.00	0.22	0.18	0.00	0.00	0.19	0.00	0.00	0.00	0.00	0.00	0.00
NYX	0.00	0.00	0.14	0.25	0.00	0.27	0.15	0.00	0.00	0.00	0.13	0.18	0.00	0.00	0.24	0.00	0.00	0.00	0.00	0.00	0.00
CLINT1	0.00	0.00	0.16	0.32	0.00	0.34	0.21	0.00	0.00	0.00	0.13	0.17	0.00	0.00	0.14	0.00	0.00	0.00	0.00	0.00	0.00
PRSS46	0.00	0.00	0.15	0.26	0.00	0.27	0.19	0.00	0.00	0.00	0.23	0.14	0.00	0.00	0.19	0.00	0.00	0.00	0.00	0.00	0.00
MPZL3	0.00	0.00	0.11	0.26	0.00	0.38	0.15	0.00	0.00	0.00	0.20	0.14	0.00	0.00	0.22	0.00	0.00	0.00	0.00	0.00	0.00
CLVS2	0.00	0.00	0.11	0.24	0.00	0.30	0.16	0.00	0.00	0.00	0.11	0.19	0.00	0.00	0.14	0.00	0.00	0.00	0.00	0.00	0.00
LACE1	0.00	0.00	0.12	0.27	0.00	0.37	0.15	0.00	0.00	0.00	0.14	0.19	0.00	0.00	0.24	0.00	0.00	0.00	0.00	0.00	0.00
GCCD2	0.00	0.00	0.15	0.25	0.00	0.26	0.19	0.00	0.00	0.00	0.13	0.11	0.00	0.00	0.15	0.00	0.00	0.00	0.00	0.00	0.00
CLCN4	0.00	0.00	0.12	0.28	0.00	0.31	0.16	0.00	0.00	0.00	0.29	0.19	0.00	0.00	0.15	0.00	0.00	0.00	0.00	0.00	0.00
GCCD3	0.00	0.00	0.15	0.25	0.00	0.26	0.19	0.00	0.00	0.00	0.13	0.11	0.00	0.00	0.15	0.00	0.00	0.00	0.00	0.00	0.00
MGR7	0.00	0.00	0.23	0.33	0.00	0.24	0.28	0.00	0.00	0.00	0.17	0.10	0.00	0.00	0.13	0.00	0.00	0.00	0.00	0.00	0.00
CHRND	0.00	0.00	0.17	0.29	0.00	0.15	0.25	0.00	0.00	0.00	0.20	0.10	0.00	0.00	0.20	0.00	0.00	0.00	0.00	0.00	0.00
ITGBL1	0.00	0.00	0.17	0.37	0.00	0.46	0.23	0.00	0.00	0.00	0.12	0.22	0.00	0.00	0.17	0.00	0.00	0.00	0.00	0.00	0.00
CHRNB1	0.00	0.00	0.18	0.30	0.00	0.18	0.26	0.00	0.00	0.00	0.20	0.12	0.00	0.00	0.18	0.00	0.00	0.00	0.00	0.00	0.00
DYT15	0.00	0.00	0.13	0.21	0.00	0.25	0.13	0.00	0.00	0.00	0.13	0.12	0.00	0.00	0.14	0.00	0.00	0.00	0.00	0.00	0.00
CACNG6	0.00	0.00	0.21	0.37	0.00	0.37	0.25	0.00	0.00	0.00	0.20	0.26	0.00	0.00	0.20	0.00	0.00	0.00	0.00	0.00	0.00
PRRG4	0.00	0.00	0.14	0.30	0.00	0.42	0.17	0.00	0.00	0.00	0.18	0.20	0.00	0.00	0.17	0.00	0.00	0.00	0.00	0.00	0.00
SLC26A5	0.00	0.00	0.00	0.19	0.00	0.21	0.10	0.18	0.00	0.00	0.20	0.11	0.00	0.00	0.19	0.00	0.00	0.00	0.00	0.00	0.00
KIFC3	0.00	0.00	0.00	0.30	0.00	0.48	0.13	0.14	0.00	0.00	0.14	0.23	0.00	0.00	0.22	0.00	0.00	0.00	0.00	0.00	0.00

KCNU1	0.00	0.00	0.00	0.19	0.00	0.23	0.10	0.13	0.00	0.00	0.16	0.14	0.00	0.00	0.13	0.00	0.00	0.00	0.00	0.00	0.00
CLDN14	0.00	0.00	0.00	0.22	0.00	0.26	0.12	0.11	0.00	0.00	0.22	0.13	0.00	0.00	0.16	0.00	0.00	0.00	0.00	0.00	0.00
ATP1A4	0.00	0.00	0.00	0.23	0.00	0.35	0.11	0.12	0.00	0.00	0.15	0.21	0.00	0.00	0.14	0.00	0.00	0.00	0.00	0.00	0.00
GRXCR1	0.00	0.00	0.00	0.20	0.00	0.28	0.12	0.13	0.00	0.00	0.14	0.12	0.00	0.00	0.20	0.00	0.00	0.00	0.00	0.00	0.00
CASD1	0.00	0.00	0.00	0.28	0.00	0.53	0.10	0.14	0.00	0.00	0.17	0.30	0.00	0.00	0.27	0.00	0.00	0.00	0.00	0.00	0.00
RAB36	0.00	0.00	0.00	0.28	0.00	0.46	0.12	0.23	0.00	0.00	0.13	0.16	0.00	0.00	0.14	0.00	0.00	0.00	0.00	0.00	0.00
TMEM147	0.00	0.00	0.00	0.25	0.00	0.41	0.12	0.14	0.00	0.00	0.28	0.11	0.00	0.00	0.14	0.00	0.00	0.00	0.00	0.00	0.00
ORMDL1	0.00	0.00	0.00	0.27	0.00	0.46	0.11	0.14	0.00	0.00	0.29	0.19	0.00	0.00	0.14	0.00	0.00	0.00	0.00	0.00	0.00
C14ORF133	0.00	0.00	0.00	0.25	0.00	0.39	0.11	0.18	0.00	0.00	0.15	0.10	0.00	0.00	0.15	0.00	0.00	0.00	0.00	0.00	0.00
CDHR1	0.00	0.00	0.00	0.24	0.00	0.37	0.10	0.13	0.00	0.00	0.14	0.16	0.00	0.00	0.24	0.00	0.00	0.00	0.00	0.00	0.00
SNX33	0.00	0.00	0.00	0.26	0.00	0.37	0.13	0.19	0.00	0.00	0.15	0.18	0.00	0.00	0.12	0.00	0.00	0.00	0.00	0.00	0.00
KIAA1432	0.00	0.00	0.00	0.29	0.00	0.46	0.12	0.10	0.00	0.00	0.16	0.13	0.00	0.00	0.11	0.00	0.00	0.00	0.00	0.00	0.00
ANK1	0.00	0.00	0.00	0.18	0.00	0.19	0.10	0.14	0.00	0.00	0.16	0.16	0.00	0.00	0.20	0.00	0.00	0.00	0.00	0.00	0.00
C14ORF49	0.00	0.00	0.00	0.24	0.00	0.39	0.11	0.16	0.00	0.00	0.21	0.12	0.00	0.00	0.22	0.00	0.00	0.00	0.00	0.00	0.00
VWA1	0.00	0.00	0.00	0.22	0.00	0.32	0.11	0.11	0.00	0.00	0.13	0.16	0.00	0.00	0.24	0.00	0.00	0.00	0.00	0.00	0.00
KCNE4	0.00	0.00	0.00	0.19	0.00	0.26	0.10	0.12	0.00	0.00	0.18	0.18	0.00	0.00	0.17	0.00	0.00	0.00	0.00	0.00	0.00
ESPN	0.00	0.00	0.00	0.22	0.00	0.26	0.13	0.14	0.00	0.00	0.10	0.16	0.00	0.00	0.22	0.00	0.00	0.00	0.00	0.00	0.00
FAM134C	0.00	0.00	0.00	0.33	0.00	0.57	0.13	0.19	0.00	0.00	0.11	0.28	0.00	0.00	0.33	0.00	0.00	0.00	0.00	0.00	0.00
OTOR	0.00	0.00	0.00	0.27	0.00	0.44	0.12	0.19	0.00	0.00	0.13	0.24	0.00	0.00	0.24	0.00	0.00	0.00	0.00	0.00	0.00
CTNNA3	0.00	0.00	0.00	0.20	0.00	0.29	0.10	0.16	0.00	0.00	0.14	0.17	0.00	0.00	0.18	0.00	0.00	0.00	0.00	0.00	0.00
COG7	0.00	0.00	0.00	0.30	0.00	0.47	0.14	0.16	0.00	0.00	0.25	0.13	0.00	0.00	0.17	0.00	0.00	0.00	0.00	0.00	0.00
SUN2	0.00	0.00	0.00	0.26	0.00	0.38	0.12	0.16	0.00	0.00	0.16	0.10	0.00	0.00	0.14	0.00	0.00	0.00	0.00	0.00	0.00
CRELD1	0.00	0.00	0.00	0.23	0.00	0.39	0.10	0.11	0.00	0.00	0.12	0.13	0.00	0.00	0.17	0.00	0.00	0.00	0.00	0.00	0.00
PSMC1	0.00	0.00	0.00	0.29	0.00	0.44	0.13	0.23	0.00	0.00	0.11	0.19	0.00	0.00	0.24	0.00	0.00	0.00	0.00	0.00	0.00
PCNXL3	0.00	0.00	0.00	0.29	0.00	0.49	0.13	0.12	0.00	0.00	0.16	0.19	0.00	0.00	0.18	0.00	0.00	0.00	0.00	0.00	0.00
SYT16	0.00	0.00	0.00	0.30	0.00	0.48	0.12	0.15	0.00	0.00	0.14	0.27	0.00	0.00	0.23	0.00	0.00	0.00	0.00	0.00	0.00
ANKZF1	0.00	0.00	0.00	0.25	0.00	0.43	0.10	0.22	0.00	0.00	0.13	0.10	0.00	0.00	0.16	0.00	0.00	0.00	0.00	0.00	0.00
SYNE2	0.00	0.00	0.00	0.22	0.00	0.32	0.11	0.15	0.00	0.00	0.16	0.11	0.00	0.00	0.16	0.00	0.00	0.00	0.00	0.00	0.00
FAM20C	0.00	0.00	0.00	0.24	0.00	0.41	0.10	0.13	0.00	0.00	0.11	0.17	0.00	0.00	0.18	0.00	0.00	0.00	0.00	0.00	0.00
TXLNB	0.00	0.00	0.00	0.27	0.00	0.45	0.11	0.12	0.00	0.00	0.13	0.22	0.00	0.00	0.28	0.00	0.00	0.00	0.00	0.00	0.00
EPB41	0.00	0.00	0.00	0.21	0.00	0.23	0.11	0.16	0.00	0.00	0.16	0.17	0.00	0.00	0.21	0.00	0.00	0.00	0.00	0.00	0.00
FRAS1	0.00	0.00	0.00	0.22	0.00	0.31	0.12	0.12	0.00	0.00	0.15	0.12	0.00	0.00	0.22	0.00	0.00	0.00	0.00	0.00	0.00
SIL1	0.00	0.00	0.00	0.21	0.00	0.36	0.00	0.11	0.11	0.00	0.17	0.17	0.00	0.00	0.20	0.00	0.00	0.00	0.00	0.00	0.00
NBAS	0.00	0.00	0.00	0.24	0.00	0.44	0.00	0.18	0.15	0.00	0.14	0.17	0.00	0.00	0.18	0.00	0.00	0.00	0.00	0.00	0.00
EXT1	0.00	0.00	0.00	0.15	0.00	0.18	0.00	0.14	0.14	0.00	0.11	0.10	0.00	0.00	0.19	0.00	0.00	0.00	0.00	0.00	0.00
PORCN	0.00	0.00	0.00	0.20	0.00	0.36	0.00	0.21	0.11	0.00	0.11	0.15	0.00	0.00	0.17	0.00	0.00	0.00	0.00	0.00	0.00
KIAA1199	0.00	0.00	0.00	0.23	0.00	0.42	0.00	0.31	0.18	0.00	0.10	0.14	0.00	0.00	0.14	0.00	0.00	0.00	0.00	0.00	0.00
KCNRG	0.00	0.00	0.00	0.23	0.00	0.44	0.00	0.31	0.18	0.00	0.13	0.18	0.00	0.00	0.18	0.00	0.00	0.00	0.00	0.00	0.00
NAPSB	0.00	0.00	0.00	0.21	0.00	0.33	0.00	0.21	0.18	0.00	0.16	0.19	0.00	0.00	0.12	0.00	0.00	0.00	0.00	0.00	0.00
TUSC3	0.00	0.00	0.00	0.20	0.00	0.41	0.00	0.20	0.13	0.00	0.11	0.19	0.00	0.00	0.17	0.00	0.00	0.00	0.00	0.00	0.00

ADAMTS17	0.00	0.00	0.00	0.14	0.00	0.25	0.00	0.14	0.14	0.00	0.18	0.16	0.00	0.00	0.16	0.00	0.00	0.00	0.00	0.00	0.00
LOC100130063	0.00	0.00	0.15	0.26	0.00	0.32	0.16	0.00	0.00	0.00	0.00	0.18	0.14	0.00	0.17	0.00	0.00	0.00	0.00	0.00	0.00
PAND3	0.00	0.00	0.13	0.18	0.00	0.11	0.15	0.00	0.00	0.00	0.00	0.12	0.12	0.00	0.14	0.00	0.00	0.00	0.00	0.00	0.00
NCKAP5	0.00	0.00	0.23	0.35	0.00	0.30	0.26	0.00	0.00	0.00	0.00	0.14	0.10	0.00	0.14	0.00	0.00	0.00	0.00	0.00	0.00
APC2	0.00	0.00	0.00	0.25	0.00	0.40	0.12	0.33	0.00	0.00	0.00	0.23	0.11	0.00	0.25	0.00	0.00	0.00	0.00	0.00	0.00
RALGAPA1	0.00	0.00	0.00	0.28	0.00	0.51	0.11	0.30	0.00	0.00	0.00	0.26	0.11	0.00	0.26	0.00	0.00	0.00	0.00	0.00	0.00
CENPJ	0.00	0.00	0.00	0.28	0.00	0.42	0.14	0.26	0.00	0.00	0.00	0.17	0.11	0.00	0.20	0.00	0.00	0.00	0.00	0.00	0.00
KAT6B	0.00	0.00	0.00	0.24	0.00	0.35	0.11	0.37	0.00	0.00	0.00	0.17	0.12	0.00	0.24	0.00	0.00	0.00	0.00	0.00	0.00
PCNT	0.00	0.00	0.00	0.18	0.00	0.27	0.00	0.21	0.11	0.00	0.00	0.17	0.16	0.00	0.20	0.00	0.00	0.00	0.00	0.00	0.00
CTSH	0.00	0.00	0.00	0.16	0.00	0.21	0.00	0.19	0.21	0.00	0.00	0.18	0.11	0.00	0.17	0.00	0.00	0.00	0.00	0.00	0.00
SEP-01	0.00	0.00	0.00	0.28	0.00	0.49	0.00	0.28	0.14	0.00	0.00	0.24	0.14	0.00	0.24	0.00	0.00	0.00	0.00	0.00	0.00
PAX8	0.00	0.00	0.00	0.13	0.00	0.11	0.00	0.20	0.00	0.17	0.00	0.12	0.11	0.00	0.18	0.00	0.00	0.00	0.00	0.00	0.00
SLC24A1	0.00	0.00	0.10	0.24	0.00	0.36	0.00	0.00	0.00	0.00	0.24	0.25	0.10	0.00	0.22	0.00	0.00	0.00	0.00	0.00	0.00
ATP6V0A4	0.00	0.00	0.00	0.22	0.00	0.31	0.00	0.13	0.00	0.00	0.20	0.18	0.12	0.00	0.15	0.00	0.00	0.00	0.00	0.00	0.00
KLHL14	0.00	0.00	0.00	0.22	0.00	0.38	0.00	0.17	0.00	0.00	0.20	0.24	0.10	0.00	0.22	0.00	0.00	0.00	0.00	0.00	0.00
TMEM237	0.00	0.00	0.00	0.26	0.00	0.47	0.00	0.12	0.00	0.00	0.11	0.24	0.12	0.00	0.27	0.00	0.00	0.00	0.00	0.00	0.00
NTRK1	0.00	0.00	0.12	0.14	0.00	0.00	0.13	0.14	0.15	0.00	0.00	0.00	0.00	0.12	0.18	0.00	0.00	0.00	0.00	0.00	0.00
NGFG	0.00	0.00	0.00	0.18	0.00	0.18	0.13	0.20	0.14	0.00	0.00	0.00	0.00	0.15	0.15	0.00	0.00	0.00	0.00	0.00	0.00
CNO	0.00	0.00	0.00	0.26	0.00	0.35	0.14	0.17	0.00	0.00	0.00	0.20	0.00	0.11	0.19	0.00	0.00	0.00	0.00	0.00	0.00
BLOC1S3	0.00	0.00	0.00	0.22	0.00	0.32	0.12	0.11	0.00	0.00	0.00	0.20	0.00	0.10	0.17	0.00	0.00	0.00	0.00	0.00	0.00
CAPN2	0.00	0.00	0.00	0.14	0.00	0.11	0.00	0.19	0.20	0.00	0.00	0.16	0.00	0.13	0.18	0.00	0.00	0.00	0.00	0.00	0.00
KCNMB3	0.00	0.00	0.00	0.15	0.00	0.19	0.00	0.10	0.00	0.00	0.13	0.18	0.00	0.10	0.14	0.00	0.00	0.00	0.00	0.00	0.00
SCLT1	0.00	0.00	0.00	0.13	0.00	0.13	0.00	0.11	0.00	0.00	0.21	0.17	0.00	0.11	0.21	0.00	0.00	0.00	0.00	0.00	0.00
KCNMB2	0.00	0.00	0.00	0.16	0.00	0.17	0.00	0.13	0.00	0.00	0.16	0.19	0.00	0.11	0.18	0.00	0.00	0.00	0.00	0.00	0.00
CHRNB4	0.00	0.00	0.13	0.20	0.00	0.00	0.19	0.00	0.00	0.00	0.00	0.16	0.13	0.15	0.21	0.00	0.00	0.00	0.00	0.00	0.00
RSRC1	0.00	0.00	0.00	0.17	0.00	0.20	0.10	0.00	0.00	0.00	0.00	0.23	0.26	0.22	0.22	0.00	0.00	0.00	0.00	0.00	0.00
C18ORF25	0.00	0.00	0.00	0.21	0.00	0.41	0.00	0.12	0.00	0.00	0.00	0.21	0.15	0.14	0.21	0.00	0.00	0.00	0.00	0.00	0.00
MARK1	0.00	0.00	0.00	0.19	0.00	0.31	0.00	0.16	0.00	0.00	0.00	0.24	0.13	0.16	0.17	0.00	0.00	0.00	0.00	0.00	0.00
RNF165	0.00	0.00	0.00	0.21	0.00	0.41	0.00	0.12	0.00	0.00	0.00	0.21	0.15	0.14	0.21	0.00	0.00	0.00	0.00	0.00	0.00
TMEM200A	0.00	0.00	0.00	0.21	0.00	0.41	0.00	0.12	0.00	0.00	0.00	0.21	0.15	0.14	0.21	0.00	0.00	0.00	0.00	0.00	0.00
NUFIP1	0.00	0.00	0.00	0.25	0.00	0.46	0.00	0.24	0.00	0.00	0.00	0.17	0.10	0.10	0.15	0.00	0.00	0.00	0.00	0.00	0.00
TMEM200B	0.00	0.00	0.00	0.21	0.00	0.41	0.00	0.12	0.00	0.00	0.00	0.21	0.15	0.14	0.21	0.00	0.00	0.00	0.00	0.00	0.00
CHURC1	0.00	0.00	0.00	0.24	0.00	0.42	0.00	0.26	0.00	0.00	0.00	0.22	0.13	0.12	0.27	0.00	0.00	0.00	0.00	0.00	0.00
AFF2	0.00	0.00	0.00	0.18	0.00	0.30	0.00	0.12	0.00	0.00	0.00	0.21	0.13	0.11	0.18	0.00	0.00	0.00	0.00	0.00	0.00
RAB39B	0.00	0.00	0.00	0.26	0.00	0.53	0.00	0.11	0.00	0.00	0.00	0.35	0.14	0.15	0.26	0.00	0.00	0.00	0.00	0.00	0.00
TMEM200C	0.00	0.00	0.00	0.21	0.00	0.41	0.00	0.12	0.00	0.00	0.00	0.21	0.15	0.14	0.21	0.00	0.00	0.00	0.00	0.00	0.00
DYX6	0.00	0.00	0.00	0.15	0.00	0.22	0.00	0.00	0.11	0.00	0.00	0.18	0.16	0.13	0.15	0.00	0.00	0.00	0.00	0.00	0.00
ASAH2B	0.00	0.00	0.00	0.19	0.00	0.38	0.00	0.00	0.12	0.00	0.00	0.27	0.13	0.13	0.18	0.00	0.00	0.00	0.00	0.00	0.00
STH	0.00	0.00	0.00	0.12	0.00	0.15	0.00	0.00	0.11	0.00	0.00	0.14	0.10	0.13	0.11	0.00	0.00	0.00	0.00	0.00	0.00
DAOA	0.00	0.00	0.00	0.15	0.00	0.13	0.00	0.00	0.12	0.00	0.00	0.19	0.23	0.19	0.17	0.00	0.00	0.00	0.00	0.00	0.00

ANKK1	0.00	0.00	0.00	0.14	0.00	0.00	0.12	0.00	0.11	0.00	0.00	0.13	0.14	0.13	0.15	0.00	0.00	0.00	0.00	0.00	0.00
FMR1	0.00	0.00	0.00	0.11	0.00	0.00	0.00	0.13	0.10	0.00	0.00	0.12	0.14	0.15	0.11	0.00	0.00	0.00	0.00	0.00	0.00
CNNM4	0.00	0.00	0.00	0.29	0.00	0.52	0.00	0.00	0.00	0.00	0.21	0.31	0.12	0.10	0.31	0.00	0.00	0.00	0.00	0.00	0.00
CDH2	0.00	0.00	0.00	0.00	0.00	0.00	0.00	0.13	0.14	0.00	0.13	0.12	0.10	0.12	0.17	0.00	0.00	0.00	0.00	0.00	0.00
CX3CR1	0.00	0.00	0.10	0.13	0.00	0.00	0.10	0.12	0.15	0.00	0.00	0.00	0.00	0.00	0.16	0.18	0.00	0.00	0.00	0.00	0.00
MPZ	0.00	0.00	0.00	0.14	0.00	0.13	0.00	0.12	0.14	0.00	0.14	0.00	0.00	0.00	0.29	0.26	0.00	0.00	0.00	0.00	0.00
PMP22	0.00	0.00	0.00	0.11	0.00	0.11	0.00	0.12	0.15	0.00	0.12	0.00	0.00	0.00	0.28	0.26	0.00	0.00	0.00	0.00	0.00
TBX20	0.00	0.00	0.00	0.21	0.00	0.29	0.11	0.26	0.00	0.00	0.00	0.15	0.00	0.00	0.32	0.11	0.00	0.00	0.00	0.00	0.00
RNF10	0.00	0.00	0.00	0.34	0.00	0.65	0.11	0.31	0.00	0.00	0.00	0.21	0.00	0.00	0.31	0.16	0.00	0.00	0.00	0.00	0.00
HES2	0.00	0.00	0.00	0.26	0.00	0.37	0.14	0.27	0.00	0.00	0.00	0.19	0.00	0.00	0.28	0.11	0.00	0.00	0.00	0.00	0.00
NEFH	0.00	0.00	0.00	0.16	0.00	0.11	0.12	0.13	0.00	0.00	0.00	0.13	0.00	0.00	0.26	0.21	0.00	0.00	0.00	0.00	0.00
SPRN	0.00	0.00	0.00	0.27	0.00	0.40	0.14	0.12	0.00	0.00	0.00	0.22	0.00	0.00	0.26	0.11	0.00	0.00	0.00	0.00	0.00
HOXA1	0.00	0.00	0.00	0.15	0.00	0.13	0.10	0.26	0.00	0.00	0.00	0.13	0.00	0.00	0.27	0.15	0.00	0.00	0.00	0.00	0.00
POU4F3	0.00	0.00	0.00	0.18	0.00	0.22	0.11	0.28	0.00	0.00	0.00	0.13	0.00	0.00	0.32	0.17	0.00	0.00	0.00	0.00	0.00
OSR1	0.00	0.00	0.00	0.22	0.00	0.30	0.12	0.30	0.00	0.00	0.00	0.17	0.00	0.00	0.26	0.11	0.00	0.00	0.00	0.00	0.00
MAFB	0.00	0.00	0.00	0.16	0.00	0.17	0.11	0.27	0.00	0.00	0.00	0.11	0.00	0.00	0.26	0.13	0.00	0.00	0.00	0.00	0.00
CASS4	0.00	0.00	0.00	0.20	0.00	0.35	0.00	0.23	0.13	0.00	0.00	0.19	0.00	0.00	0.27	0.17	0.00	0.00	0.00	0.00	0.00
USPL1	0.00	0.00	0.00	0.15	0.00	0.24	0.00	0.14	0.24	0.00	0.00	0.16	0.00	0.00	0.29	0.18	0.00	0.00	0.00	0.00	0.00
SYNM	0.00	0.00	0.00	0.21	0.00	0.30	0.00	0.15	0.14	0.00	0.00	0.20	0.00	0.00	0.27	0.13	0.00	0.00	0.00	0.00	0.00
SETX	0.00	0.00	0.00	0.16	0.00	0.29	0.00	0.11	0.15	0.00	0.00	0.15	0.00	0.00	0.24	0.12	0.00	0.00	0.00	0.00	0.00
PRX	0.00	0.00	0.00	0.18	0.00	0.24	0.11	0.00	0.00	0.00	0.16	0.15	0.00	0.00	0.40	0.30	0.00	0.00	0.00	0.00	0.00
BAHCC1	0.00	0.00	0.00	0.24	0.00	0.45	0.00	0.12	0.00	0.00	0.12	0.24	0.00	0.00	0.32	0.11	0.00	0.00	0.00	0.00	0.00
VEZT	0.00	0.00	0.00	0.19	0.00	0.37	0.00	0.27	0.00	0.00	0.15	0.18	0.00	0.00	0.23	0.11	0.00	0.00	0.00	0.00	0.00
FAM184B	0.00	0.00	0.00	0.24	0.00	0.45	0.00	0.12	0.00	0.00	0.12	0.24	0.00	0.00	0.32	0.11	0.00	0.00	0.00	0.00	0.00
NOTCH3	0.00	0.00	0.00	0.12	0.00	0.00	0.00	0.21	0.20	0.00	0.10	0.11	0.00	0.00	0.19	0.12	0.00	0.00	0.00	0.00	0.00
HOXA2	0.00	0.00	0.00	0.15	0.00	0.14	0.00	0.25	0.00	0.00	0.00	0.13	0.10	0.00	0.30	0.20	0.00	0.00	0.00	0.00	0.00
ATOH7	0.00	0.00	0.00	0.17	0.00	0.23	0.00	0.25	0.00	0.00	0.00	0.19	0.17	0.00	0.39	0.22	0.00	0.00	0.00	0.00	0.00
CLIP2	0.00	0.00	0.00	0.21	0.00	0.38	0.00	0.18	0.00	0.00	0.00	0.26	0.17	0.00	0.31	0.13	0.00	0.00	0.00	0.00	0.00
KIF15	0.00	0.00	0.00	0.15	0.00	0.29	0.00	0.17	0.00	0.00	0.00	0.21	0.21	0.00	0.27	0.11	0.00	0.00	0.00	0.00	0.00
HESX1	0.00	0.00	0.00	0.14	0.00	0.15	0.00	0.23	0.00	0.00	0.00	0.13	0.10	0.00	0.25	0.14	0.00	0.00	0.00	0.00	0.00
FOXI2	0.00	0.00	0.00	0.25	0.00	0.46	0.00	0.31	0.00	0.00	0.00	0.22	0.12	0.00	0.34	0.14	0.00	0.00	0.00	0.00	0.00
SOX3	0.00	0.00	0.00	0.18	0.00	0.25	0.00	0.26	0.00	0.00	0.00	0.19	0.13	0.00	0.30	0.12	0.00	0.00	0.00	0.00	0.00
MYH14	0.00	0.00	0.00	0.17	0.00	0.26	0.00	0.14	0.00	0.00	0.00	0.19	0.11	0.00	0.25	0.10	0.00	0.00	0.00	0.00	0.00
ZFP90	0.00	0.00	0.00	0.24	0.00	0.49	0.00	0.30	0.00	0.00	0.00	0.24	0.11	0.00	0.29	0.11	0.00	0.00	0.00	0.00	0.00
ZNF488	0.00	0.00	0.00	0.20	0.00	0.33	0.00	0.29	0.00	0.00	0.00	0.18	0.14	0.00	0.42	0.44	0.00	0.00	0.00	0.00	0.00
FOXI3	0.00	0.00	0.00	0.19	0.00	0.30	0.00	0.17	0.00	0.00	0.00	0.18	0.10	0.00	0.28	0.11	0.00	0.00	0.00	0.00	0.00
EN1	0.00	0.00	0.00	0.13	0.00	0.00	0.10	0.18	0.00	0.00	0.00	0.19	0.20	0.00	0.33	0.25	0.00	0.00	0.00	0.00	0.00
AAAS	0.00	0.00	0.00	0.19	0.00	0.29	0.00	0.00	0.11	0.00	0.00	0.21	0.14	0.00	0.31	0.14	0.00	0.00	0.00	0.00	0.00
GJC3	0.00	0.00	0.00	0.19	0.00	0.24	0.00	0.00	0.00	0.00	0.17	0.20	0.13	0.00	0.33	0.26	0.00	0.00	0.00	0.00	0.00
GJC2	0.00	0.00	0.00	0.16	0.00	0.21	0.00	0.00	0.00	0.00	0.14	0.21	0.13	0.00	0.35	0.32	0.00	0.00	0.00	0.00	0.00

GRIA1	0.00	0.00	0.00	0.16	0.00	0.00	0.13	0.00	0.12	0.00	0.00	0.00	0.13	0.19	0.13	0.11	0.00	0.00	0.00	0.00	0.00
GRIN2B	0.00	0.00	0.00	0.13	0.00	0.00	0.10	0.00	0.12	0.00	0.00	0.00	0.15	0.17	0.13	0.11	0.00	0.00	0.00	0.00	0.00
GRIN2A	0.00	0.00	0.00	0.14	0.00	0.00	0.11	0.00	0.12	0.00	0.00	0.00	0.15	0.18	0.15	0.13	0.00	0.00	0.00	0.00	0.00
GDNF	0.00	0.00	0.00	0.12	0.00	0.00	0.00	0.15	0.19	0.00	0.00	0.00	0.12	0.11	0.21	0.11	0.00	0.00	0.00	0.00	0.00
CCDC85C	0.00	0.00	0.00	0.18	0.00	0.27	0.00	0.00	0.00	0.00	0.00	0.48	0.48	0.40	0.43	0.34	0.00	0.00	0.00	0.00	0.00
AUTS3	0.00	0.00	0.00	0.16	0.00	0.27	0.00	0.00	0.00	0.00	0.00	0.33	0.21	0.20	0.28	0.11	0.00	0.00	0.00	0.00	0.00
CDHR4	0.00	0.00	0.00	0.16	0.00	0.30	0.00	0.00	0.00	0.00	0.00	0.56	0.31	0.29	0.36	0.24	0.00	0.00	0.00	0.00	0.00
KCNS2	0.00	0.00	0.00	0.18	0.00	0.33	0.00	0.00	0.00	0.00	0.00	0.31	0.12	0.15	0.25	0.12	0.00	0.00	0.00	0.00	0.00
DWS	0.00	0.00	0.00	0.12	0.00	0.23	0.00	0.00	0.00	0.00	0.00	0.20	0.16	0.13	0.24	0.11	0.00	0.00	0.00	0.00	0.00
DHX9P1	0.00	0.00	0.00	0.22	0.00	0.51	0.00	0.00	0.00	0.00	0.00	0.26	0.11	0.11	0.24	0.12	0.00	0.00	0.00	0.00	0.00
KBTBD11	0.00	0.00	0.00	0.17	0.00	0.34	0.00	0.00	0.00	0.00	0.00	0.26	0.14	0.10	0.37	0.23	0.00	0.00	0.00	0.00	0.00
SLC12A2	0.00	0.00	0.00	0.11	0.00	0.00	0.00	0.12	0.00	0.00	0.00	0.13	0.19	0.14	0.19	0.12	0.00	0.00	0.00	0.00	0.00
GFAP	0.00	0.00	0.00	0.11	0.00	0.00	0.00	0.00	0.11	0.00	0.00	0.11	0.13	0.17	0.16	0.20	0.00	0.00	0.00	0.00	0.00
FCMTE2	0.00	0.00	0.00	0.00	0.00	0.17	0.00	0.00	0.10	0.00	0.00	0.18	0.21	0.21	0.15	0.12	0.00	0.00	0.00	0.00	0.00
SCA18	0.00	0.00	0.00	0.00	0.00	0.11	0.00	0.00	0.11	0.00	0.00	0.21	0.15	0.11	0.28	0.20	0.00	0.00	0.00	0.00	0.00
SCN1A	0.00	0.00	0.00	0.00	0.00	0.00	0.00	0.00	0.15	0.00	0.12	0.17	0.14	0.18	0.22	0.16	0.00	0.00	0.00	0.00	0.00
PPBPL1	0.00	0.00	0.12	0.24	0.00	0.28	0.15	0.16	0.16	0.00	0.00	0.00	0.00	0.00	0.00	0.00	0.11	0.00	0.00	0.00	0.00
AIRE	0.00	0.00	0.00	0.15	0.00	0.14	0.10	0.18	0.15	0.00	0.00	0.00	0.00	0.00	0.16	0.00	0.17	0.00	0.00	0.00	0.00
F12	0.00	0.00	0.00	0.15	0.00	0.13	0.11	0.11	0.13	0.00	0.00	0.00	0.00	0.00	0.12	0.00	0.13	0.00	0.00	0.00	0.00
IGAD1	0.00	0.00	0.15	0.20	0.00	0.00	0.21	0.00	0.10	0.00	0.14	0.00	0.00	0.00	0.13	0.00	0.13	0.00	0.00	0.00	0.00
BP10	0.00	0.00	0.11	0.18	0.00	0.21	0.12	0.00	0.00	0.00	0.00	0.11	0.00	0.00	0.15	0.00	0.15	0.00	0.00	0.00	0.00
BP12	0.00	0.00	0.11	0.18	0.00	0.21	0.12	0.00	0.00	0.00	0.00	0.11	0.00	0.00	0.15	0.00	0.15	0.00	0.00	0.00	0.00
BP13	0.00	0.00	0.11	0.18	0.00	0.21	0.12	0.00	0.00	0.00	0.00	0.11	0.00	0.00	0.15	0.00	0.15	0.00	0.00	0.00	0.00
HYT1	0.00	0.00	0.10	0.20	0.00	0.29	0.11	0.00	0.00	0.00	0.00	0.15	0.00	0.00	0.15	0.00	0.16	0.00	0.00	0.00	0.00
BP11	0.00	0.00	0.11	0.18	0.00	0.21	0.12	0.00	0.00	0.00	0.00	0.11	0.00	0.00	0.15	0.00	0.15	0.00	0.00	0.00	0.00
BBS5	0.00	0.00	0.11	0.22	0.00	0.34	0.10	0.00	0.00	0.00	0.00	0.15	0.00	0.00	0.16	0.00	0.16	0.00	0.00	0.00	0.00
SKOR1	0.00	0.00	0.00	0.19	0.00	0.28	0.11	0.14	0.00	0.00	0.00	0.12	0.00	0.00	0.16	0.00	0.11	0.00	0.00	0.00	0.00
MPHOSPH9	0.00	0.00	0.00	0.19	0.00	0.29	0.00	0.14	0.17	0.00	0.00	0.17	0.00	0.00	0.15	0.00	0.12	0.00	0.00	0.00	0.00
FOXN3	0.00	0.00	0.00	0.22	0.00	0.47	0.00	0.33	0.12	0.00	0.00	0.13	0.00	0.00	0.17	0.00	0.13	0.00	0.00	0.00	0.00
COL18A1	0.00	0.00	0.00	0.12	0.00	0.12	0.00	0.16	0.25	0.00	0.00	0.11	0.00	0.00	0.14	0.00	0.11	0.00	0.00	0.00	0.00
KIF12	0.00	0.00	0.00	0.26	0.00	0.45	0.00	0.17	0.00	0.00	0.13	0.13	0.00	0.00	0.15	0.00	0.17	0.00	0.00	0.00	0.00
ADM	0.00	0.00	0.14	0.15	0.00	0.00	0.12	0.00	0.15	0.00	0.00	0.00	0.12	0.00	0.17	0.00	0.10	0.00	0.00	0.00	0.00
PPR3	0.00	0.00	0.00	0.11	0.00	0.17	0.00	0.00	0.00	0.00	0.00	0.18	0.16	0.17	0.13	0.00	0.10	0.00	0.00	0.00	0.00
NPHS2	0.00	0.00	0.00	0.13	0.00	0.16	0.00	0.13	0.13	0.00	0.00	0.00	0.00	0.00	0.12	0.13	0.14	0.00	0.00	0.00	0.00
NINJ2	0.00	0.00	0.00	0.13	0.00	0.15	0.00	0.00	0.00	0.00	0.00	0.15	0.00	0.11	0.22	0.11	0.21	0.00	0.00	0.00	0.00
FNIP1	0.00	0.00	0.00	0.27	0.00	0.40	0.14	0.31	0.00	0.00	0.00	0.15	0.00	0.00	0.18	0.00	0.00	0.19	0.00	0.00	0.00
PPP2CA	0.00	0.00	0.00	0.12	0.00	0.16	0.00	0.19	0.11	0.00	0.00	0.13	0.00	0.00	0.10	0.00	0.00	0.11	0.00	0.00	0.00
MIP	0.00	0.00	0.00	0.17	0.00	0.20	0.00	0.15	0.00	0.00	0.00	0.13	0.11	0.00	0.20	0.00	0.00	0.11	0.00	0.00	0.00
CRH	0.00	0.00	0.00	0.11	0.00	0.00	0.00	0.10	0.11	0.00	0.00	0.00	0.13	0.13	0.17	0.00	0.00	0.14	0.00	0.00	0.00
ACHE	0.00	0.00	0.00	0.00	0.00	0.00	0.00	0.13	0.16	0.00	0.00	0.11	0.16	0.18	0.16	0.00	0.00	0.12	0.00	0.00	0.00

HSPB7	0.00	0.00	0.00	0.21	0.00	0.34	0.00	0.15	0.00	0.00	0.00	0.15	0.00	0.00	0.19	0.00	0.12	0.11	0.00	0.00	0.00
APOA1BP	0.00	0.00	0.11	0.30	0.00	0.40	0.17	0.17	0.00	0.00	0.00	0.15	0.00	0.00	0.00	0.00	0.00	0.00	0.23	0.00	0.00
MIR758	0.00	0.00	0.00	0.19	0.00	0.26	0.10	0.32	0.14	0.00	0.00	0.13	0.00	0.00	0.00	0.00	0.00	0.00	0.23	0.00	0.00
PCBP2	0.00	0.00	0.00	0.25	0.00	0.32	0.12	0.34	0.12	0.00	0.00	0.00	0.00	0.00	0.14	0.00	0.00	0.00	0.13	0.00	0.00
TIA1	0.00	0.00	0.00	0.23	0.00	0.26	0.14	0.34	0.21	0.00	0.00	0.00	0.00	0.00	0.15	0.00	0.00	0.00	0.11	0.00	0.00
TREX1	0.00	0.00	0.00	0.19	0.00	0.23	0.11	0.20	0.19	0.00	0.00	0.00	0.00	0.00	0.19	0.00	0.00	0.00	0.11	0.00	0.00
RLBP1	0.00	0.00	0.00	0.19	0.00	0.23	0.11	0.14	0.14	0.00	0.00	0.00	0.00	0.00	0.17	0.00	0.00	0.00	0.14	0.00	0.00
LIPH	0.00	0.00	0.16	0.27	0.00	0.32	0.16	0.00	0.00	0.00	0.12	0.00	0.00	0.00	0.13	0.00	0.00	0.00	0.19	0.00	0.00
ABCC6	0.00	0.00	0.00	0.15	0.00	0.17	0.00	0.11	0.18	0.00	0.11	0.00	0.00	0.00	0.12	0.00	0.00	0.00	0.13	0.00	0.00
SRSF7	0.00	0.00	0.00	0.28	0.00	0.37	0.14	0.25	0.00	0.00	0.00	0.11	0.00	0.00	0.16	0.00	0.00	0.00	0.15	0.00	0.00
GPX5	0.00	0.00	0.00	0.23	0.00	0.33	0.11	0.16	0.00	0.00	0.00	0.20	0.00	0.00	0.17	0.00	0.00	0.00	0.17	0.00	0.00
FXR1	0.00	0.00	0.00	0.27	0.00	0.38	0.13	0.22	0.00	0.00	0.00	0.21	0.00	0.00	0.22	0.00	0.00	0.00	0.11	0.00	0.00
SREK1	0.00	0.00	0.00	0.34	0.00	0.51	0.15	0.22	0.00	0.00	0.00	0.17	0.00	0.00	0.19	0.00	0.00	0.00	0.12	0.00	0.00
CELF3	0.00	0.00	0.00	0.28	0.00	0.47	0.11	0.20	0.00	0.00	0.00	0.28	0.00	0.00	0.25	0.00	0.00	0.00	0.10	0.00	0.00
SRSF6	0.00	0.00	0.00	0.28	0.00	0.37	0.14	0.27	0.00	0.00	0.00	0.10	0.00	0.00	0.17	0.00	0.00	0.00	0.14	0.00	0.00
GNPTAB	0.00	0.00	0.00	0.20	0.00	0.24	0.12	0.00	0.16	0.00	0.00	0.14	0.00	0.00	0.18	0.00	0.00	0.00	0.13	0.00	0.00
ERVK-11	0.00	0.00	0.00	0.16	0.00	0.33	0.00	0.16	0.18	0.00	0.00	0.14	0.00	0.00	0.14	0.00	0.00	0.00	0.11	0.00	0.00
GNE	0.00	0.00	0.00	0.18	0.00	0.26	0.00	0.18	0.24	0.00	0.00	0.17	0.00	0.00	0.22	0.00	0.00	0.00	0.18	0.00	0.00
DDX6	0.00	0.00	0.00	0.19	0.00	0.37	0.00	0.38	0.14	0.00	0.00	0.13	0.00	0.00	0.17	0.00	0.00	0.00	0.15	0.00	0.00
PTS	0.00	0.00	0.00	0.14	0.00	0.21	0.00	0.12	0.16	0.00	0.00	0.19	0.00	0.00	0.20	0.00	0.00	0.00	0.20	0.00	0.00
NDRG1	0.00	0.00	0.00	0.18	0.00	0.21	0.00	0.34	0.31	0.00	0.00	0.11	0.00	0.00	0.18	0.00	0.00	0.00	0.11	0.00	0.00
UBIAD1	0.00	0.00	0.00	0.23	0.00	0.39	0.00	0.19	0.17	0.00	0.00	0.13	0.00	0.00	0.13	0.00	0.00	0.00	0.19	0.00	0.00
BLMH	0.00	0.00	0.00	0.19	0.00	0.28	0.00	0.17	0.17	0.00	0.00	0.20	0.00	0.00	0.16	0.00	0.00	0.00	0.19	0.00	0.00
SMARCAL1	0.00	0.00	0.00	0.15	0.00	0.32	0.00	0.18	0.14	0.00	0.00	0.10	0.00	0.00	0.19	0.00	0.00	0.00	0.11	0.00	0.00
RNASEH2A	0.00	0.00	0.00	0.23	0.00	0.46	0.00	0.29	0.15	0.00	0.00	0.13	0.00	0.00	0.16	0.00	0.00	0.00	0.14	0.00	0.00
GSTZ1	0.00	0.00	0.00	0.16	0.00	0.21	0.00	0.12	0.10	0.00	0.00	0.13	0.00	0.00	0.11	0.00	0.00	0.00	0.29	0.00	0.00
CERKL	0.00	0.00	0.00	0.23	0.00	0.37	0.00	0.11	0.13	0.00	0.00	0.18	0.00	0.00	0.21	0.00	0.00	0.00	0.14	0.00	0.00
CRTAP	0.00	0.00	0.00	0.18	0.00	0.32	0.00	0.11	0.12	0.00	0.00	0.14	0.00	0.00	0.16	0.00	0.00	0.00	0.11	0.00	0.00
GCH1	0.00	0.00	0.00	0.12	0.00	0.14	0.00	0.12	0.18	0.00	0.00	0.13	0.00	0.00	0.17	0.00	0.00	0.00	0.13	0.00	0.00
FAH	0.00	0.00	0.00	0.16	0.00	0.19	0.00	0.16	0.26	0.00	0.00	0.16	0.00	0.00	0.18	0.00	0.00	0.00	0.23	0.00	0.00
CNBP	0.00	0.00	0.00	0.20	0.00	0.35	0.00	0.19	0.12	0.00	0.00	0.12	0.00	0.00	0.18	0.00	0.00	0.00	0.12	0.00	0.00
HMBS	0.00	0.00	0.00	0.11	0.00	0.13	0.00	0.13	0.16	0.00	0.00	0.13	0.00	0.00	0.15	0.00	0.00	0.00	0.25	0.00	0.00
EHMT1	0.00	0.00	0.00	0.18	0.00	0.24	0.00	0.32	0.10	0.00	0.00	0.10	0.00	0.00	0.15	0.00	0.00	0.00	0.11	0.00	0.00
LCMT1	0.00	0.00	0.00	0.25	0.00	0.48	0.00	0.38	0.12	0.00	0.00	0.23	0.00	0.00	0.18	0.00	0.00	0.00	0.11	0.00	0.00
ATXN8	0.00	0.00	0.00	0.15	0.00	0.25	0.00	0.12	0.18	0.00	0.00	0.15	0.00	0.00	0.15	0.00	0.00	0.00	0.10	0.00	0.00
IYD	0.00	0.00	0.00	0.22	0.00	0.36	0.00	0.16	0.00	0.17	0.00	0.16	0.00	0.00	0.16	0.00	0.00	0.00	0.22	0.00	0.00
CYBRD1	0.00	0.00	0.00	0.21	0.00	0.22	0.13	0.00	0.00	0.00	0.14	0.12	0.00	0.00	0.14	0.00	0.00	0.00	0.28	0.00	0.00
ABCA10	0.00	0.00	0.00	0.28	0.00	0.50	0.11	0.00	0.00	0.00	0.28	0.24	0.00	0.00	0.19	0.00	0.00	0.00	0.21	0.00	0.00
ATP8A2	0.00	0.00	0.00	0.29	0.00	0.56	0.00	0.16	0.00	0.00	0.22	0.26	0.00	0.00	0.21	0.00	0.00	0.00	0.12	0.00	0.00
C14ORF1	0.00	0.00	0.00	0.33	0.00	0.68	0.00	0.27	0.00	0.00	0.17	0.21	0.00	0.00	0.16	0.00	0.00	0.00	0.22	0.00	0.00

ST6GALNAC6	0.00	0.00	0.00	0.19	0.00	0.46	0.00	0.18	0.00	0.00	0.11	0.21	0.00	0.00	0.12	0.00	0.00	0.00	0.16	0.00	0.00
ABCA6	0.00	0.00	0.00	0.24	0.00	0.42	0.00	0.11	0.00	0.00	0.21	0.17	0.00	0.00	0.13	0.00	0.00	0.00	0.19	0.00	0.00
TBC1D14	0.00	0.00	0.00	0.26	0.00	0.47	0.00	0.12	0.00	0.00	0.10	0.23	0.00	0.00	0.22	0.00	0.00	0.00	0.11	0.00	0.00
NIPSNAP3B	0.00	0.00	0.00	0.26	0.00	0.49	0.00	0.14	0.00	0.00	0.14	0.18	0.00	0.00	0.14	0.00	0.00	0.00	0.18	0.00	0.00
MAN2B2	0.00	0.00	0.00	0.25	0.00	0.46	0.00	0.11	0.00	0.00	0.11	0.23	0.00	0.00	0.16	0.00	0.00	0.00	0.11	0.00	0.00
PIGP	0.00	0.00	0.00	0.28	0.00	0.56	0.00	0.13	0.00	0.00	0.16	0.34	0.00	0.00	0.26	0.00	0.00	0.00	0.15	0.00	0.00
ABCA13	0.00	0.00	0.00	0.25	0.00	0.43	0.00	0.10	0.00	0.00	0.14	0.20	0.00	0.00	0.16	0.00	0.00	0.00	0.16	0.00	0.00
ALG3	0.00	0.00	0.00	0.20	0.00	0.37	0.00	0.00	0.11	0.00	0.18	0.20	0.00	0.00	0.21	0.00	0.00	0.00	0.11	0.00	0.00
ALG8	0.00	0.00	0.00	0.14	0.00	0.30	0.00	0.00	0.12	0.00	0.18	0.13	0.00	0.00	0.16	0.00	0.00	0.00	0.12	0.00	0.00
HGSNAT	0.00	0.00	0.00	0.18	0.00	0.32	0.00	0.00	0.15	0.00	0.15	0.17	0.00	0.00	0.19	0.00	0.00	0.00	0.13	0.00	0.00
SLC7A9	0.00	0.00	0.00	0.18	0.00	0.26	0.00	0.00	0.11	0.00	0.20	0.12	0.00	0.00	0.18	0.00	0.00	0.00	0.13	0.00	0.00
NPHP3-ACAD11	0.00	0.00	0.00	0.13	0.00	0.22	0.00	0.00	0.13	0.00	0.10	0.12	0.00	0.00	0.14	0.00	0.00	0.00	0.11	0.00	0.00
PMM2	0.00	0.00	0.00	0.16	0.00	0.25	0.00	0.00	0.16	0.00	0.11	0.17	0.00	0.00	0.22	0.00	0.00	0.00	0.17	0.00	0.00
MANBA	0.00	0.00	0.00	0.21	0.00	0.33	0.00	0.00	0.13	0.00	0.11	0.20	0.00	0.00	0.20	0.00	0.00	0.00	0.16	0.00	0.00
ADH4	0.00	0.00	0.00	0.15	0.00	0.10	0.12	0.00	0.00	0.00	0.00	0.13	0.12	0.00	0.16	0.00	0.00	0.00	0.29	0.00	0.00
ADH7	0.00	0.00	0.00	0.17	0.00	0.10	0.13	0.00	0.00	0.00	0.00	0.15	0.13	0.00	0.19	0.00	0.00	0.00	0.27	0.00	0.00
CTSO	0.00	0.00	0.00	0.23	0.00	0.43	0.00	0.10	0.00	0.00	0.00	0.27	0.12	0.00	0.18	0.00	0.00	0.00	0.14	0.00	0.00
ARSB	0.00	0.00	0.00	0.13	0.00	0.19	0.00	0.00	0.16	0.00	0.00	0.17	0.11	0.00	0.17	0.00	0.00	0.00	0.20	0.00	0.00
SLC3A1	0.00	0.00	0.00	0.19	0.00	0.28	0.00	0.00	0.00	0.00	0.22	0.16	0.13	0.00	0.21	0.00	0.00	0.00	0.12	0.00	0.00
ATP13A5	0.00	0.00	0.00	0.20	0.00	0.47	0.00	0.00	0.00	0.00	0.16	0.28	0.11	0.00	0.20	0.00	0.00	0.00	0.11	0.00	0.00
MFSD10	0.00	0.00	0.00	0.26	0.00	0.53	0.00	0.00	0.00	0.00	0.31	0.20	0.10	0.00	0.13	0.00	0.00	0.00	0.13	0.00	0.00
WDYHV1	0.00	0.00	0.00	0.28	0.00	0.58	0.00	0.17	0.00	0.00	0.00	0.27	0.00	0.10	0.19	0.00	0.00	0.00	0.13	0.00	0.00
FSBP	0.00	0.00	0.00	0.18	0.00	0.28	0.00	0.15	0.00	0.00	0.00	0.23	0.00	0.18	0.13	0.00	0.00	0.00	0.11	0.00	0.00
TF	0.00	0.00	0.00	0.14	0.00	0.00	0.10	0.00	0.11	0.00	0.00	0.13	0.00	0.11	0.15	0.00	0.00	0.00	0.17	0.00	0.00
TTR	0.00	0.00	0.00	0.00	0.00	0.00	0.00	0.13	0.16	0.00	0.00	0.11	0.11	0.12	0.19	0.00	0.00	0.00	0.12	0.00	0.00
SMN1	0.00	0.00	0.00	0.10	0.00	0.12	0.00	0.12	0.24	0.00	0.00	0.00	0.00	0.00	0.21	0.12	0.00	0.00	0.10	0.00	0.00
IDUA	0.00	0.00	0.00	0.13	0.00	0.15	0.00	0.00	0.26	0.00	0.00	0.16	0.00	0.00	0.20	0.14	0.00	0.00	0.13	0.00	0.00
APOC4	0.00	0.00	0.11	0.26	0.00	0.29	0.16	0.13	0.00	0.00	0.00	0.00	0.00	0.00	0.00	0.00	0.14	0.00	0.26	0.00	0.00
ABCG5	0.00	0.00	0.00	0.14	0.00	0.10	0.12	0.14	0.12	0.00	0.00	0.00	0.00	0.00	0.00	0.00	0.11	0.00	0.23	0.00	0.00
ABCG8	0.00	0.00	0.00	0.13	0.00	0.10	0.11	0.14	0.12	0.00	0.00	0.00	0.00	0.00	0.00	0.00	0.12	0.00	0.23	0.00	0.00
SHPK	0.00	0.00	0.00	0.23	0.00	0.34	0.11	0.00	0.00	0.00	0.00	0.11	0.00	0.00	0.11	0.00	0.13	0.00	0.11	0.00	0.00
RPSAP52	0.00	0.00	0.00	0.17	0.00	0.31	0.00	0.00	0.00	0.00	0.00	0.19	0.00	0.10	0.11	0.00	0.10	0.00	0.14	0.00	0.00
SOAT2	0.00	0.00	0.00	0.15	0.00	0.21	0.00	0.16	0.13	0.00	0.00	0.12	0.00	0.00	0.00	0.00	0.00	0.10	0.37	0.00	0.00
LYZ	0.00	0.00	0.00	0.15	0.00	0.22	0.00	0.18	0.17	0.00	0.00	0.00	0.00	0.00	0.12	0.00	0.00	0.12	0.11	0.00	0.00
PABPN1	0.00	0.00	0.00	0.21	0.00	0.28	0.00	0.17	0.17	0.00	0.00	0.00	0.00	0.00	0.17	0.00	0.00	0.10	0.13	0.00	0.00
PCYT1A	0.00	0.00	0.00	0.18	0.00	0.25	0.00	0.24	0.00	0.00	0.00	0.12	0.00	0.00	0.12	0.00	0.00	0.13	0.27	0.00	0.00
THRA	0.00	0.00	0.00	0.13	0.00	0.00	0.12	0.18	0.00	0.00	0.00	0.11	0.00	0.00	0.14	0.00	0.00	0.13	0.13	0.00	0.00
HPRT1	0.00	0.00	0.00	0.10	0.00	0.00	0.00	0.17	0.22	0.00	0.00	0.14	0.00	0.00	0.17	0.00	0.00	0.11	0.15	0.00	0.00
TSC2	0.00	0.00	0.00	0.13	0.00	0.00	0.00	0.24	0.20	0.00	0.00	0.13	0.00	0.00	0.16	0.00	0.00	0.17	0.11	0.00	0.00
APOA4	0.00	0.00	0.00	0.12	0.00	0.00	0.00	0.10	0.13	0.00	0.00	0.00	0.00	0.00	0.10	0.00	0.13	0.12	0.18	0.00	0.00

DDIT3	0.00	0.00	0.00	0.13	0.00	0.13	0.00	0.18	0.23	0.00	0.00	0.00	0.00	0.00	0.12	0.00	0.16	0.00	0.00	0.16	0.00
AGER	0.00	0.00	0.00	0.12	0.00	0.00	0.10	0.13	0.18	0.00	0.00	0.00	0.00	0.00	0.12	0.00	0.18	0.00	0.00	0.11	0.00
AVP	0.00	0.00	0.10	0.14	0.00	0.00	0.10	0.00	0.00	0.00	0.00	0.00	0.12	0.00	0.17	0.00	0.15	0.00	0.00	0.11	0.00
HMSNO	0.00	0.00	0.00	0.10	0.00	0.20	0.00	0.00	0.00	0.00	0.00	0.11	0.00	0.00	0.24	0.11	0.22	0.00	0.00	0.12	0.00
INSR	0.00	0.00	0.00	0.13	0.00	0.00	0.10	0.10	0.11	0.00	0.00	0.00	0.00	0.00	0.11	0.00	0.00	0.11	0.00	0.11	0.00
OB4	0.00	0.00	0.00	0.16	0.00	0.26	0.00	0.00	0.00	0.00	0.00	0.13	0.00	0.00	0.13	0.00	0.22	0.17	0.00	0.16	0.00
BW1	0.00	0.00	0.00	0.16	0.00	0.26	0.00	0.00	0.00	0.00	0.00	0.13	0.00	0.00	0.13	0.00	0.22	0.17	0.00	0.16	0.00
MEHMO	0.00	0.00	0.00	0.00	0.00	0.00	0.00	0.00	0.00	0.00	0.00	0.19	0.12	0.14	0.10	0.00	0.13	0.11	0.00	0.12	0.00
POU1F1	0.00	0.00	0.00	0.13	0.00	0.00	0.12	0.20	0.11	0.00	0.00	0.00	0.00	0.00	0.15	0.00	0.00	0.00	0.11	0.11	0.00
ABCB11	0.00	0.00	0.00	0.13	0.00	0.12	0.00	0.15	0.15	0.00	0.00	0.00	0.00	0.00	0.00	0.00	0.11	0.00	0.18	0.11	0.00
ALOX12	0.00	0.00	0.00	0.13	0.00	0.00	0.00	0.17	0.21	0.00	0.00	0.00	0.00	0.00	0.12	0.00	0.19	0.00	0.20	0.15	0.00
SBDSP1	0.00	0.00	0.00	0.14	0.00	0.40	0.00	0.00	0.00	0.00	0.00	0.10	0.00	0.00	0.12	0.00	0.13	0.00	0.14	0.11	0.00
RNPEPL1	0.00	0.00	0.00	0.19	0.00	0.38	0.00	0.00	0.00	0.00	0.00	0.21	0.00	0.00	0.13	0.00	0.30	0.00	0.13	0.15	0.00
MSTN	0.00	0.00	0.00	0.14	0.00	0.00	0.11	0.16	0.22	0.00	0.00	0.00	0.00	0.00	0.00	0.00	0.00	0.18	0.13	0.13	0.00
RPL18AP16	0.00	0.00	0.00	0.11	0.00	0.36	0.00	0.00	0.00	0.00	0.16	0.14	0.00	0.00	0.00	0.00	0.00	0.10	0.13	0.12	0.00
PHKB	0.00	0.00	0.00	0.18	0.00	0.29	0.00	0.00	0.00	0.00	0.00	0.20	0.00	0.00	0.12	0.00	0.00	0.14	0.19	0.14	0.00
ABCC8	0.00	0.00	0.00	0.13	0.00	0.00	0.11	0.00	0.16	0.00	0.00	0.00	0.00	0.00	0.00	0.00	0.17	0.12	0.14	0.18	0.00
SREBF1	0.00	0.00	0.00	0.11	0.00	0.00	0.00	0.20	0.13	0.00	0.00	0.00	0.00	0.00	0.00	0.00	0.11	0.11	0.16	0.12	0.00
GCK	0.00	0.00	0.00	0.11	0.00	0.00	0.00	0.00	0.12	0.00	0.00	0.00	0.00	0.00	0.11	0.00	0.30	0.19	0.20	0.37	0.00
BECN1P1	0.00	0.00	0.11	0.26	0.00	0.32	0.16	0.40	0.30	0.00	0.00	0.00	0.00	0.00	0.00	0.00	0.00	0.00	0.00	0.00	0.16
POGK	0.00	0.00	0.00	0.25	0.00	0.42	0.12	0.25	0.16	0.00	0.00	0.10	0.00	0.00	0.00	0.00	0.00	0.00	0.00	0.00	0.33
KIAA0226	0.00	0.00	0.13	0.31	0.00	0.41	0.18	0.31	0.00	0.00	0.00	0.00	0.00	0.00	0.14	0.00	0.00	0.00	0.00	0.00	0.11
AEN	0.00	0.00	0.00	0.24	0.00	0.37	0.11	0.37	0.13	0.00	0.00	0.00	0.00	0.00	0.11	0.00	0.00	0.00	0.00	0.00	0.17
PLEKHM2	0.00	0.00	0.00	0.22	0.00	0.33	0.11	0.14	0.00	0.00	0.10	0.00	0.00	0.00	0.14	0.00	0.00	0.00	0.00	0.00	0.12
C9ORF114	0.00	0.00	0.00	0.28	0.00	0.49	0.11	0.23	0.00	0.00	0.00	0.12	0.00	0.00	0.16	0.00	0.00	0.00	0.00	0.00	0.33
NXNL1	0.00	0.00	0.00	0.22	0.00	0.38	0.00	0.23	0.19	0.00	0.00	0.15	0.00	0.00	0.18	0.00	0.00	0.00	0.00	0.00	0.12
PPP2R2B	0.00	0.00	0.00	0.17	0.00	0.26	0.00	0.26	0.16	0.00	0.00	0.20	0.00	0.00	0.17	0.00	0.00	0.00	0.00	0.00	0.13
NDUFB7	0.00	0.00	0.00	0.28	0.00	0.59	0.00	0.11	0.00	0.00	0.16	0.13	0.00	0.00	0.11	0.00	0.00	0.00	0.00	0.00	0.39
MAPRE3	0.00	0.00	0.00	0.23	0.00	0.38	0.00	0.25	0.00	0.00	0.00	0.17	0.13	0.00	0.23	0.00	0.00	0.00	0.00	0.00	0.11
TTLL6	0.00	0.00	0.00	0.23	0.00	0.43	0.00	0.20	0.00	0.00	0.00	0.21	0.11	0.00	0.20	0.00	0.00	0.00	0.00	0.00	0.17
TTLL11	0.00	0.00	0.00	0.15	0.00	0.25	0.00	0.16	0.00	0.00	0.00	0.15	0.14	0.00	0.16	0.00	0.00	0.00	0.00	0.00	0.16
C21ORF2	0.00	0.00	0.00	0.25	0.00	0.48	0.00	0.00	0.00	0.00	0.00	0.31	0.12	0.12	0.22	0.00	0.00	0.00	0.00	0.00	0.12
MRPL32	0.00	0.00	0.00	0.14	0.00	0.27	0.00	0.00	0.11	0.00	0.00	0.20	0.00	0.00	0.31	0.20	0.00	0.00	0.00	0.00	0.16
C7ORF25	0.00	0.00	0.00	0.14	0.00	0.27	0.00	0.00	0.11	0.00	0.00	0.20	0.00	0.00	0.31	0.20	0.00	0.00	0.00	0.00	0.16
TMEM126A	0.00	0.00	0.00	0.11	0.00	0.24	0.00	0.00	0.00	0.00	0.12	0.11	0.00	0.00	0.20	0.11	0.00	0.00	0.00	0.00	0.16
REEP1	0.00	0.00	0.00	0.15	0.00	0.26	0.00	0.00	0.00	0.00	0.11	0.17	0.00	0.00	0.24	0.11	0.00	0.00	0.00	0.00	0.14
SLC35E1	0.00	0.00	0.00	0.23	0.00	0.45	0.00	0.13	0.00	0.00	0.00	0.21	0.00	0.00	0.15	0.00	0.00	0.12	0.00	0.00	0.13
C15ORF27	0.00	0.00	0.00	0.12	0.00	0.13	0.00	0.00	0.00	0.00	0.19	0.13	0.00	0.00	0.13	0.00	0.00	0.15	0.00	0.00	0.15
RRM2B	0.00	0.00	0.00	0.16	0.00	0.27	0.00	0.30	0.25	0.00	0.00	0.00	0.00	0.00	0.11	0.00	0.00	0.00	0.19	0.00	0.23
ATG4A	0.00	0.00	0.00	0.22	0.00	0.38	0.00	0.25	0.10	0.00	0.00	0.00	0.00	0.00	0.14	0.00	0.00	0.00	0.13	0.00	0.21

RMRP	0.00	0.00	0.00	0.15	0.00	0.33	0.00	0.15	0.10	0.00	0.00	0.00	0.00	0.00	0.14	0.00	0.00	0.00	0.11	0.00	0.11
HSD3B7	0.00	0.00	0.00	0.19	0.00	0.27	0.10	0.00	0.00	0.00	0.00	0.15	0.00	0.00	0.11	0.00	0.00	0.00	0.40	0.00	0.13
COX8C	0.00	0.00	0.00	0.30	0.00	0.42	0.17	0.00	0.00	0.00	0.00	0.21	0.00	0.00	0.16	0.00	0.00	0.00	0.13	0.00	0.32
UROC1	0.00	0.00	0.00	0.13	0.00	0.35	0.00	0.12	0.00	0.00	0.00	0.21	0.00	0.00	0.12	0.00	0.00	0.00	0.41	0.00	0.17
SPATA18	0.00	0.00	0.00	0.26	0.00	0.47	0.00	0.25	0.00	0.00	0.00	0.20	0.00	0.00	0.27	0.00	0.00	0.00	0.13	0.00	0.32
FBXL5	0.00	0.00	0.00	0.21	0.00	0.33	0.00	0.22	0.00	0.00	0.00	0.11	0.00	0.00	0.17	0.00	0.00	0.00	0.10	0.00	0.12
NDUFB9	0.00	0.00	0.00	0.20	0.00	0.46	0.00	0.11	0.00	0.00	0.00	0.18	0.00	0.00	0.13	0.00	0.00	0.00	0.18	0.00	0.33
GLRX5	0.00	0.00	0.00	0.15	0.00	0.27	0.00	0.17	0.00	0.00	0.00	0.11	0.00	0.00	0.12	0.00	0.00	0.00	0.28	0.00	0.34
POP7	0.00	0.00	0.00	0.22	0.00	0.52	0.00	0.17	0.00	0.00	0.00	0.11	0.00	0.00	0.16	0.00	0.00	0.00	0.16	0.00	0.19
ABCB7	0.00	0.00	0.00	0.21	0.00	0.38	0.00	0.12	0.00	0.00	0.00	0.18	0.00	0.00	0.16	0.00	0.00	0.00	0.29	0.00	0.29
ALAS2	0.00	0.00	0.00	0.15	0.00	0.20	0.00	0.12	0.00	0.00	0.00	0.13	0.00	0.00	0.15	0.00	0.00	0.00	0.26	0.00	0.15
MRPS12	0.00	0.00	0.00	0.30	0.00	0.59	0.00	0.31	0.00	0.00	0.00	0.17	0.00	0.00	0.16	0.00	0.00	0.00	0.12	0.00	0.23
MOCS2	0.00	0.00	0.00	0.19	0.00	0.36	0.00	0.11	0.00	0.00	0.00	0.19	0.00	0.00	0.21	0.00	0.00	0.00	0.30	0.00	0.22
ALDH7A1P1	0.00	0.00	0.00	0.24	0.00	0.48	0.00	0.14	0.00	0.00	0.00	0.22	0.00	0.00	0.15	0.00	0.00	0.00	0.21	0.00	0.11
SARS	0.00	0.00	0.00	0.21	0.00	0.45	0.00	0.14	0.00	0.00	0.00	0.16	0.00	0.00	0.17	0.00	0.00	0.00	0.17	0.00	0.28
MOCS1	0.00	0.00	0.00	0.17	0.00	0.30	0.00	0.00	0.11	0.00	0.00	0.21	0.00	0.00	0.22	0.00	0.00	0.00	0.30	0.00	0.19
MMAB	0.00	0.00	0.00	0.16	0.00	0.28	0.00	0.00	0.10	0.00	0.00	0.11	0.00	0.00	0.12	0.00	0.00	0.00	0.38	0.00	0.12
UROD	0.00	0.00	0.00	0.14	0.00	0.16	0.00	0.00	0.14	0.00	0.00	0.11	0.00	0.00	0.11	0.00	0.00	0.00	0.28	0.00	0.14
CPOX	0.00	0.00	0.00	0.12	0.00	0.21	0.00	0.00	0.10	0.00	0.00	0.15	0.00	0.00	0.12	0.00	0.00	0.00	0.31	0.00	0.25
GLDC	0.00	0.00	0.00	0.18	0.00	0.28	0.00	0.00	0.15	0.00	0.00	0.23	0.00	0.00	0.21	0.00	0.00	0.00	0.23	0.00	0.11
SFXN4	0.00	0.00	0.00	0.33	0.00	0.70	0.00	0.00	0.00	0.00	0.20	0.30	0.00	0.00	0.21	0.00	0.00	0.00	0.16	0.00	0.25
FAM178A	0.00	0.00	0.00	0.24	0.00	0.53	0.00	0.00	0.00	0.00	0.11	0.31	0.00	0.00	0.20	0.00	0.00	0.00	0.16	0.00	0.11
MRXSL	0.00	0.00	0.00	0.00	0.00	0.19	0.00	0.00	0.00	0.00	0.00	0.27	0.16	0.00	0.24	0.15	0.00	0.00	0.12	0.00	0.11
NUDT1	0.00	0.00	0.00	0.10	0.00	0.16	0.00	0.12	0.00	0.00	0.00	0.13	0.00	0.00	0.00	0.00	0.10	0.00	0.14	0.00	0.17
MALSU1	0.00	0.00	0.00	0.22	0.00	0.49	0.00	0.12	0.00	0.00	0.00	0.17	0.00	0.00	0.00	0.00	0.00	0.27	0.28	0.00	0.75
MRRF	0.00	0.00	0.00	0.26	0.00	0.56	0.00	0.16	0.00	0.00	0.00	0.14	0.00	0.00	0.00	0.00	0.00	0.19	0.20	0.00	0.56
CHCHD3	0.00	0.00	0.00	0.23	0.00	0.47	0.00	0.16	0.00	0.00	0.00	0.16	0.00	0.00	0.00	0.00	0.00	0.27	0.23	0.00	0.63
USP30	0.00	0.00	0.00	0.17	0.00	0.45	0.00	0.21	0.00	0.00	0.00	0.18	0.00	0.00	0.00	0.00	0.00	0.24	0.29	0.00	0.71
POLG2	0.00	0.00	0.00	0.14	0.00	0.31	0.00	0.12	0.00	0.00	0.00	0.00	0.00	0.00	0.10	0.00	0.00	0.15	0.17	0.00	0.30
POLRMT	0.00	0.00	0.00	0.21	0.00	0.45	0.00	0.26	0.00	0.00	0.00	0.00	0.00	0.00	0.13	0.00	0.00	0.16	0.18	0.00	0.31
MTRF1L	0.00	0.00	0.00	0.23	0.00	0.48	0.00	0.21	0.00	0.00	0.00	0.00	0.00	0.00	0.12	0.00	0.00	0.16	0.22	0.00	0.48
C8ORF38	0.00	0.00	0.00	0.19	0.00	0.46	0.00	0.12	0.00	0.00	0.00	0.00	0.00	0.00	0.14	0.00	0.00	0.15	0.16	0.00	0.42
TFB2M	0.00	0.00	0.00	0.22	0.00	0.38	0.00	0.27	0.00	0.00	0.00	0.00	0.00	0.00	0.12	0.00	0.00	0.19	0.17	0.00	0.35
SDHC	0.00	0.00	0.00	0.11	0.00	0.18	0.00	0.10	0.00	0.00	0.00	0.00	0.00	0.00	0.12	0.00	0.00	0.12	0.17	0.00	0.27
TRND	0.00	0.00	0.00	0.12	0.00	0.31	0.00	0.11	0.00	0.00	0.00	0.00	0.00	0.00	0.13	0.00	0.00	0.22	0.12	0.00	0.44
SDHB	0.00	0.00	0.00	0.00	0.00	0.15	0.00	0.11	0.12	0.00	0.00	0.00	0.00	0.00	0.15	0.00	0.00	0.13	0.17	0.00	0.23
NDUFV3	0.00	0.00	0.00	0.20	0.00	0.41	0.00	0.00	0.00	0.00	0.00	0.18	0.00	0.00	0.12	0.00	0.00	0.17	0.30	0.00	0.39
HCCS	0.00	0.00	0.00	0.22	0.00	0.42	0.00	0.00	0.00	0.00	0.00	0.22	0.00	0.00	0.16	0.00	0.00	0.11	0.18	0.00	0.32
DBT	0.00	0.00	0.00	0.17	0.00	0.30	0.00	0.00	0.00	0.00	0.00	0.16	0.00	0.00	0.15	0.00	0.00	0.15	0.23	0.00	0.18
PCCB	0.00	0.00	0.00	0.14	0.00	0.27	0.00	0.00	0.00	0.00	0.00	0.17	0.00	0.00	0.13	0.00	0.00	0.15	0.28	0.00	0.23

BCS1L	0.00	0.00	0.00	0.19	0.00	0.39	0.00	0.00	0.00	0.00	0.00	0.20	0.00	0.00	0.21	0.00	0.00	0.17	0.24	0.00	0.42
NDUFS8	0.00	0.00	0.00	0.17	0.00	0.43	0.00	0.00	0.00	0.00	0.00	0.17	0.00	0.00	0.14	0.00	0.00	0.17	0.22	0.00	0.45
ATP5S	0.00	0.00	0.00	0.14	0.00	0.24	0.00	0.00	0.00	0.00	0.00	0.14	0.00	0.00	0.10	0.00	0.00	0.32	0.21	0.00	0.38
NDUFS2	0.00	0.00	0.00	0.19	0.00	0.40	0.00	0.00	0.00	0.00	0.00	0.15	0.00	0.00	0.14	0.00	0.00	0.18	0.20	0.00	0.47
MYO19	0.00	0.00	0.00	0.21	0.00	0.44	0.00	0.00	0.00	0.00	0.00	0.19	0.00	0.00	0.15	0.00	0.00	0.15	0.11	0.00	0.39
HADHB	0.00	0.00	0.00	0.18	0.00	0.28	0.00	0.00	0.00	0.00	0.00	0.20	0.00	0.00	0.14	0.00	0.00	0.21	0.36	0.00	0.30
ACAD8	0.00	0.00	0.00	0.20	0.00	0.38	0.00	0.00	0.00	0.00	0.00	0.22	0.00	0.00	0.12	0.00	0.00	0.18	0.42	0.00	0.30
COA5	0.00	0.00	0.00	0.15	0.00	0.38	0.00	0.00	0.00	0.00	0.00	0.10	0.00	0.00	0.12	0.00	0.00	0.15	0.18	0.00	0.50
NDUFA12	0.00	0.00	0.00	0.13	0.00	0.39	0.00	0.00	0.00	0.00	0.00	0.19	0.00	0.00	0.11	0.00	0.00	0.20	0.24	0.00	0.58
PET112	0.00	0.00	0.00	0.24	0.00	0.59	0.00	0.00	0.00	0.00	0.00	0.18	0.00	0.00	0.13	0.00	0.00	0.19	0.19	0.00	0.59
ETFA	0.00	0.00	0.00	0.13	0.00	0.26	0.00	0.00	0.00	0.00	0.00	0.13	0.00	0.00	0.13	0.00	0.00	0.22	0.34	0.00	0.26
UQCR11	0.00	0.00	0.00	0.18	0.00	0.47	0.00	0.00	0.00	0.00	0.00	0.20	0.00	0.00	0.10	0.00	0.00	0.19	0.31	0.00	0.56
MMAA	0.00	0.00	0.00	0.16	0.00	0.34	0.00	0.00	0.00	0.00	0.00	0.11	0.00	0.00	0.13	0.00	0.00	0.11	0.32	0.00	0.21
ETFB	0.00	0.00	0.00	0.17	0.00	0.31	0.00	0.00	0.00	0.00	0.00	0.13	0.00	0.00	0.13	0.00	0.00	0.20	0.31	0.00	0.27
NDUFA1	0.00	0.00	0.00	0.19	0.00	0.40	0.00	0.00	0.00	0.00	0.00	0.20	0.00	0.00	0.15	0.00	0.00	0.23	0.27	0.00	0.49
ACADSB	0.00	0.00	0.00	0.19	0.00	0.31	0.00	0.00	0.00	0.00	0.00	0.17	0.00	0.00	0.12	0.00	0.00	0.16	0.42	0.00	0.25
MRPL24	0.00	0.00	0.00	0.23	0.00	0.57	0.00	0.00	0.00	0.00	0.00	0.18	0.00	0.00	0.12	0.00	0.00	0.16	0.17	0.00	0.43
COX18	0.00	0.00	0.00	0.19	0.00	0.47	0.00	0.00	0.00	0.00	0.00	0.15	0.00	0.00	0.13	0.00	0.00	0.16	0.20	0.00	0.52
SURF1	0.00	0.00	0.00	0.20	0.00	0.36	0.00	0.00	0.00	0.00	0.00	0.18	0.00	0.00	0.21	0.00	0.00	0.14	0.19	0.00	0.32
NDUFA8	0.00	0.00	0.00	0.22	0.00	0.51	0.00	0.00	0.00	0.00	0.00	0.19	0.00	0.00	0.12	0.00	0.00	0.15	0.20	0.00	0.40
MRPL18	0.00	0.00	0.00	0.23	0.00	0.57	0.00	0.00	0.00	0.00	0.00	0.18	0.00	0.00	0.12	0.00	0.00	0.16	0.17	0.00	0.43
MRPS28	0.00	0.00	0.00	0.23	0.00	0.57	0.00	0.00	0.00	0.00	0.00	0.18	0.00	0.00	0.12	0.00	0.00	0.16	0.17	0.00	0.43
PCCA	0.00	0.00	0.00	0.15	0.00	0.30	0.00	0.00	0.00	0.00	0.00	0.14	0.00	0.00	0.12	0.00	0.00	0.16	0.29	0.00	0.20
MCCC1	0.00	0.00	0.00	0.11	0.00	0.26	0.00	0.00	0.00	0.00	0.00	0.15	0.00	0.00	0.13	0.00	0.00	0.11	0.20	0.00	0.18
GFM1	0.00	0.00	0.00	0.25	0.00	0.49	0.00	0.00	0.00	0.00	0.00	0.22	0.00	0.00	0.16	0.00	0.00	0.17	0.27	0.00	0.35
TMEM70	0.00	0.00	0.00	0.12	0.00	0.35	0.00	0.00	0.00	0.00	0.00	0.15	0.00	0.00	0.13	0.00	0.00	0.19	0.26	0.00	0.43
ACSF3	0.00	0.00	0.00	0.17	0.00	0.41	0.00	0.00	0.00	0.00	0.00	0.17	0.00	0.00	0.12	0.00	0.00	0.21	0.39	0.00	0.35
AMT	0.00	0.00	0.00	0.16	0.00	0.33	0.00	0.00	0.00	0.00	0.00	0.19	0.00	0.00	0.19	0.00	0.00	0.11	0.30	0.00	0.19
AFG3L1P	0.00	0.00	0.00	0.20	0.00	0.48	0.00	0.00	0.00	0.00	0.00	0.18	0.00	0.00	0.17	0.00	0.00	0.12	0.14	0.00	0.44
OPA3	0.00	0.00	0.00	0.15	0.00	0.29	0.00	0.00	0.00	0.00	0.00	0.19	0.00	0.00	0.20	0.00	0.00	0.11	0.15	0.00	0.32
NDUFA6	0.00	0.00	0.00	0.18	0.00	0.45	0.00	0.00	0.00	0.00	0.00	0.22	0.00	0.00	0.14	0.00	0.00	0.19	0.24	0.00	0.54
TRNA	0.00	0.00	0.00	0.00	0.00	0.25	0.00	0.00	0.00	0.00	0.00	0.15	0.00	0.00	0.16	0.11	0.00	0.21	0.16	0.00	0.53
TRNAP1	0.00	0.00	0.00	0.22	0.00	0.36	0.00	0.00	0.00	0.00	0.00	0.13	0.00	0.00	0.00	0.00	0.13	0.13	0.16	0.00	0.29
C10ORF2	0.00	0.00	0.00	0.00	0.00	0.24	0.00	0.00	0.00	0.00	0.00	0.13	0.00	0.00	0.17	0.00	0.12	0.15	0.16	0.00	0.32
SLC25A36	0.00	0.00	0.00	0.11	0.00	0.32	0.00	0.00	0.00	0.00	0.00	0.18	0.00	0.00	0.00	0.00	0.00	0.38	0.43	0.22	0.76
PTCD2	0.00	0.00	0.00	0.18	0.00	0.46	0.00	0.00	0.00	0.00	0.00	0.22	0.00	0.00	0.00	0.00	0.00	0.24	0.40	0.12	0.67
ND2	0.00	0.00	0.00	0.12	0.00	0.14	0.00	0.00	0.00	0.00	0.00	0.00	0.00	0.00	0.00	0.00	0.27	0.13	0.20	0.16	0.24
ATP6	0.00	0.00	0.00	0.00	0.00	0.22	0.00	0.00	0.11	0.00	0.00	0.00	0.00	0.00	0.00	0.00	0.16	0.23	0.22	0.12	0.43
ND4	0.00	0.00	0.00	0.00	0.00	0.20	0.00	0.00	0.00	0.00	0.00	0.00	0.00	0.00	0.12	0.00	0.22	0.17	0.16	0.13	0.34
ASMD	0.00	0.00	0.17	0.26	0.00	0.15	0.23	0.19	0.15	0.00	0.00	0.00	0.00	0.00	0.00	0.00	0.00	0.00	0.00	0.00	0.00

KRT24	0.00	0.00	0.11	0.22	0.00	0.32	0.11	0.20	0.12	0.00	0.00	0.00	0.00	0.00	0.00	0.00	0.00	0.00	0.00	0.00	0.00
MTMR10	0.00	0.00	0.12	0.21	0.00	0.27	0.11	0.16	0.00	0.00	0.12	0.00	0.00	0.00	0.00	0.00	0.00	0.00	0.00	0.00	0.00
MTMR11	0.00	0.00	0.12	0.21	0.00	0.27	0.11	0.16	0.00	0.00	0.12	0.00	0.00	0.00	0.00	0.00	0.00	0.00	0.00	0.00	0.00
ATG14	0.00	0.00	0.13	0.27	0.00	0.36	0.16	0.27	0.00	0.00	0.12	0.00	0.00	0.00	0.00	0.00	0.00	0.00	0.00	0.00	0.00
SEC61G	0.00	0.00	0.10	0.26	0.00	0.38	0.13	0.15	0.00	0.00	0.24	0.00	0.00	0.00	0.00	0.00	0.00	0.00	0.00	0.00	0.00
LEMD2	0.00	0.00	0.13	0.26	0.00	0.31	0.17	0.16	0.00	0.00	0.18	0.00	0.00	0.00	0.00	0.00	0.00	0.00	0.00	0.00	0.00
UBXN2B	0.00	0.00	0.11	0.29	0.00	0.42	0.15	0.23	0.00	0.00	0.19	0.00	0.00	0.00	0.00	0.00	0.00	0.00	0.00	0.00	0.00
KDELR3	0.00	0.00	0.17	0.32	0.00	0.34	0.22	0.16	0.00	0.00	0.32	0.00	0.00	0.00	0.00	0.00	0.00	0.00	0.00	0.00	0.00
SNX8	0.00	0.00	0.18	0.30	0.00	0.33	0.20	0.14	0.00	0.00	0.27	0.00	0.00	0.00	0.00	0.00	0.00	0.00	0.00	0.00	0.00
TBC1D15	0.00	0.00	0.19	0.36	0.00	0.44	0.22	0.23	0.00	0.00	0.00	0.11	0.00	0.00	0.00	0.00	0.00	0.00	0.00	0.00	0.00
TBL3	0.00	0.00	0.11	0.30	0.00	0.56	0.12	0.00	0.00	0.00	0.16	0.15	0.00	0.00	0.00	0.00	0.00	0.00	0.00	0.00	0.00
AVL9	0.00	0.00	0.00	0.27	0.00	0.41	0.13	0.13	0.00	0.00	0.17	0.12	0.00	0.00	0.00	0.00	0.00	0.00	0.00	0.00	0.00
RAB3IL1	0.00	0.00	0.00	0.20	0.00	0.26	0.11	0.13	0.00	0.00	0.00	0.16	0.00	0.12	0.00	0.00	0.00	0.00	0.00	0.00	0.00
CCDC134	0.00	0.00	0.00	0.22	0.00	0.37	0.00	0.15	0.12	0.00	0.00	0.19	0.00	0.10	0.00	0.00	0.00	0.00	0.00	0.00	0.00
TNPO2	0.00	0.00	0.13	0.26	0.00	0.25	0.17	0.24	0.00	0.00	0.00	0.00	0.00	0.00	0.13	0.00	0.00	0.00	0.00	0.00	0.00
RAB33B	0.00	0.00	0.11	0.29	0.00	0.43	0.15	0.23	0.00	0.00	0.00	0.00	0.00	0.00	0.10	0.00	0.00	0.00	0.00	0.00	0.00
TAF10	0.00	0.00	0.13	0.27	0.00	0.30	0.18	0.32	0.00	0.00	0.00	0.00	0.00	0.00	0.13	0.00	0.00	0.00	0.00	0.00	0.00
GTF2B	0.00	0.00	0.11	0.21	0.00	0.19	0.16	0.26	0.00	0.00	0.00	0.00	0.00	0.00	0.16	0.00	0.00	0.00	0.00	0.00	0.00
EFCAB6	0.00	0.00	0.19	0.35	0.00	0.33	0.26	0.27	0.00	0.00	0.00	0.00	0.00	0.00	0.12	0.00	0.00	0.00	0.00	0.00	0.00
C1R	0.00	0.00	0.12	0.25	0.00	0.27	0.15	0.14	0.00	0.00	0.00	0.00	0.00	0.00	0.17	0.00	0.00	0.00	0.00	0.00	0.00
TAF4	0.00	0.00	0.12	0.25	0.00	0.30	0.16	0.30	0.00	0.00	0.00	0.00	0.00	0.00	0.17	0.00	0.00	0.00	0.00	0.00	0.00
CCZ1	0.00	0.00	0.11	0.33	0.00	0.53	0.14	0.32	0.00	0.00	0.00	0.00	0.00	0.00	0.13	0.00	0.00	0.00	0.00	0.00	0.00
C12ORF44	0.00	0.00	0.12	0.35	0.00	0.56	0.17	0.30	0.00	0.00	0.00	0.00	0.00	0.00	0.14	0.00	0.00	0.00	0.00	0.00	0.00
EIF3L	0.00	0.00	0.12	0.31	0.00	0.46	0.17	0.31	0.00	0.00	0.00	0.00	0.00	0.00	0.24	0.00	0.00	0.00	0.00	0.00	0.00
GUCY2D	0.00	0.00	0.13	0.19	0.00	0.18	0.11	0.00	0.13	0.00	0.00	0.00	0.00	0.00	0.16	0.00	0.00	0.00	0.00	0.00	0.00
OPTN	0.00	0.00	0.00	0.17	0.00	0.19	0.10	0.13	0.13	0.00	0.00	0.00	0.00	0.00	0.15	0.00	0.00	0.00	0.00	0.00	0.00
FLI1	0.00	0.00	0.00	0.17	0.00	0.16	0.11	0.34	0.21	0.00	0.00	0.00	0.00	0.00	0.12	0.00	0.00	0.00	0.00	0.00	0.00
RNASEL	0.00	0.00	0.00	0.16	0.00	0.16	0.11	0.22	0.19	0.00	0.00	0.00	0.00	0.00	0.15	0.00	0.00	0.00	0.00	0.00	0.00
DRAM1	0.00	0.00	0.00	0.25	0.00	0.36	0.13	0.45	0.22	0.00	0.00	0.00	0.00	0.00	0.10	0.00	0.00	0.00	0.00	0.00	0.00
WAS	0.00	0.00	0.00	0.16	0.00	0.13	0.10	0.12	0.11	0.00	0.00	0.00	0.00	0.00	0.12	0.00	0.00	0.00	0.00	0.00	0.00
BIRC2	0.00	0.00	0.00	0.17	0.00	0.13	0.12	0.19	0.24	0.00	0.00	0.00	0.00	0.00	0.12	0.00	0.00	0.00	0.00	0.00	0.00
NLRP7	0.00	0.00	0.00	0.24	0.00	0.31	0.14	0.17	0.13	0.00	0.00	0.00	0.00	0.00	0.15	0.00	0.00	0.00	0.00	0.00	0.00
NOL7	0.00	0.00	0.00	0.25	0.00	0.45	0.11	0.42	0.12	0.00	0.00	0.00	0.00	0.00	0.14	0.00	0.00	0.00	0.00	0.00	0.00
HTATSF1	0.00	0.00	0.00	0.22	0.00	0.27	0.13	0.33	0.10	0.00	0.00	0.00	0.00	0.00	0.14	0.00	0.00	0.00	0.00	0.00	0.00
TBX1	0.00	0.00	0.00	0.15	0.00	0.13	0.11	0.25	0.11	0.00	0.00	0.00	0.00	0.00	0.17	0.00	0.00	0.00	0.00	0.00	0.00
SERPING1	0.00	0.00	0.00	0.17	0.00	0.16	0.10	0.12	0.16	0.00	0.00	0.00	0.00	0.00	0.15	0.00	0.00	0.00	0.00	0.00	0.00
LTF	0.00	0.00	0.00	0.18	0.00	0.11	0.14	0.18	0.18	0.00	0.00	0.00	0.00	0.00	0.12	0.00	0.00	0.00	0.00	0.00	0.00
FANCB	0.00	0.00	0.00	0.20	0.00	0.25	0.11	0.15	0.12	0.00	0.00	0.00	0.00	0.00	0.11	0.00	0.00	0.00	0.00	0.00	0.00
AREI	0.00	0.00	0.11	0.18	0.00	0.18	0.14	0.00	0.00	0.00	0.10	0.00	0.00	0.00	0.11	0.00	0.00	0.00	0.00	0.00	0.00
NIPAL2	0.00	0.00	0.20	0.30	0.00	0.30	0.21	0.00	0.00	0.00	0.28	0.00	0.00	0.00	0.12	0.00	0.00	0.00	0.00	0.00	0.00

HMGXB3	0.00	0.00	0.23	0.35	0.00	0.29	0.27	0.00	0.00	0.00	0.16	0.00	0.00	0.00	0.11	0.00	0.00	0.00	0.00	0.00	0.00
CACNA1S	0.00	0.00	0.11	0.20	0.00	0.13	0.15	0.00	0.00	0.00	0.10	0.00	0.00	0.00	0.18	0.00	0.00	0.00	0.00	0.00	0.00
RNF139	0.00	0.00	0.00	0.26	0.00	0.40	0.13	0.33	0.00	0.00	0.15	0.00	0.00	0.00	0.15	0.00	0.00	0.00	0.00	0.00	0.00
MYO15A	0.00	0.00	0.00	0.17	0.00	0.19	0.10	0.11	0.00	0.00	0.13	0.00	0.00	0.00	0.18	0.00	0.00	0.00	0.00	0.00	0.00
UBXN7	0.00	0.00	0.00	0.27	0.00	0.44	0.13	0.24	0.00	0.00	0.11	0.00	0.00	0.00	0.17	0.00	0.00	0.00	0.00	0.00	0.00
WNK4	0.00	0.00	0.00	0.18	0.00	0.15	0.12	0.17	0.00	0.00	0.11	0.00	0.00	0.00	0.14	0.00	0.00	0.00	0.00	0.00	0.00
LEMD3	0.00	0.00	0.00	0.28	0.00	0.37	0.16	0.20	0.00	0.00	0.13	0.00	0.00	0.00	0.15	0.00	0.00	0.00	0.00	0.00	0.00
STX11	0.00	0.00	0.00	0.25	0.00	0.30	0.15	0.28	0.00	0.00	0.12	0.00	0.00	0.00	0.15	0.00	0.00	0.00	0.00	0.00	0.00
VPS52	0.00	0.00	0.00	0.26	0.00	0.38	0.13	0.17	0.00	0.00	0.13	0.00	0.00	0.00	0.11	0.00	0.00	0.00	0.00	0.00	0.00
SGCG	0.00	0.00	0.00	0.15	0.00	0.16	0.10	0.00	0.21	0.00	0.14	0.00	0.00	0.00	0.15	0.00	0.00	0.00	0.00	0.00	0.00
VCP	0.00	0.00	0.00	0.18	0.00	0.21	0.00	0.19	0.18	0.00	0.16	0.00	0.00	0.00	0.14	0.00	0.00	0.00	0.00	0.00	0.00
CLDN23	0.00	0.00	0.00	0.20	0.00	0.33	0.00	0.20	0.15	0.00	0.21	0.00	0.00	0.00	0.10	0.00	0.00	0.00	0.00	0.00	0.00
HSPG2	0.00	0.00	0.00	0.14	0.00	0.12	0.00	0.11	0.13	0.00	0.11	0.00	0.00	0.00	0.16	0.00	0.00	0.00	0.00	0.00	0.00
ITGB4	0.00	0.00	0.00	0.15	0.00	0.00	0.12	0.16	0.16	0.00	0.14	0.00	0.00	0.00	0.16	0.00	0.00	0.00	0.00	0.00	0.00
FARP1	0.00	0.00	0.11	0.25	0.00	0.38	0.12	0.00	0.00	0.00	0.00	0.20	0.00	0.00	0.17	0.00	0.00	0.00	0.00	0.00	0.00
ECA1	0.00	0.00	0.11	0.17	0.00	0.16	0.14	0.00	0.00	0.00	0.00	0.10	0.00	0.00	0.12	0.00	0.00	0.00	0.00	0.00	0.00
TULP1	0.00	0.00	0.13	0.23	0.00	0.29	0.14	0.00	0.00	0.00	0.00	0.12	0.00	0.00	0.18	0.00	0.00	0.00	0.00	0.00	0.00
FTH1P15	0.00	0.00	0.20	0.36	0.00	0.40	0.24	0.00	0.00	0.00	0.00	0.14	0.00	0.00	0.12	0.00	0.00	0.00	0.00	0.00	0.00
GIGYF2	0.00	0.00	0.13	0.22	0.00	0.22	0.16	0.00	0.00	0.00	0.00	0.14	0.00	0.00	0.20	0.00	0.00	0.00	0.00	0.00	0.00
ARHGAP27	0.00	0.00	0.16	0.32	0.00	0.41	0.19	0.00	0.00	0.00	0.00	0.17	0.00	0.00	0.12	0.00	0.00	0.00	0.00	0.00	0.00
HOOK1	0.00	0.00	0.00	0.25	0.00	0.37	0.12	0.15	0.00	0.00	0.00	0.15	0.00	0.00	0.18	0.00	0.00	0.00	0.00	0.00	0.00
PSMD7	0.00	0.00	0.00	0.29	0.00	0.45	0.13	0.27	0.00	0.00	0.00	0.16	0.00	0.00	0.23	0.00	0.00	0.00	0.00	0.00	0.00
SBF1	0.00	0.00	0.00	0.31	0.00	0.52	0.13	0.34	0.00	0.00	0.00	0.15	0.00	0.00	0.18	0.00	0.00	0.00	0.00	0.00	0.00
MAMLD1	0.00	0.00	0.00	0.28	0.00	0.46	0.12	0.20	0.00	0.00	0.00	0.16	0.00	0.00	0.19	0.00	0.00	0.00	0.00	0.00	0.00
DRG2	0.00	0.00	0.00	0.32	0.00	0.59	0.11	0.28	0.00	0.00	0.00	0.18	0.00	0.00	0.24	0.00	0.00	0.00	0.00	0.00	0.00
RBM14-RBM4	0.00	0.00	0.00	0.28	0.00	0.43	0.12	0.42	0.00	0.00	0.00	0.20	0.00	0.00	0.23	0.00	0.00	0.00	0.00	0.00	0.00
PPP2R5E	0.00	0.00	0.00	0.25	0.00	0.39	0.12	0.28	0.00	0.00	0.00	0.22	0.00	0.00	0.17	0.00	0.00	0.00	0.00	0.00	0.00
COTL1P1	0.00	0.00	0.00	0.28	0.00	0.47	0.12	0.10	0.00	0.00	0.00	0.10	0.00	0.00	0.18	0.00	0.00	0.00	0.00	0.00	0.00
ZNF224	0.00	0.00	0.00	0.30	0.00	0.50	0.12	0.43	0.00	0.00	0.00	0.13	0.00	0.00	0.13	0.00	0.00	0.00	0.00	0.00	0.00
NIF3L1	0.00	0.00	0.00	0.34	0.00	0.63	0.13	0.25	0.00	0.00	0.00	0.25	0.00	0.00	0.29	0.00	0.00	0.00	0.00	0.00	0.00
DYNC2H1	0.00	0.00	0.00	0.25	0.00	0.38	0.11	0.18	0.00	0.00	0.00	0.19	0.00	0.00	0.23	0.00	0.00	0.00	0.00	0.00	0.00
STOX1	0.00	0.00	0.00	0.22	0.00	0.34	0.11	0.19	0.00	0.00	0.00	0.19	0.00	0.00	0.21	0.00	0.00	0.00	0.00	0.00	0.00
ACTR1A	0.00	0.00	0.00	0.27	0.00	0.42	0.11	0.18	0.00	0.00	0.00	0.19	0.00	0.00	0.20	0.00	0.00	0.00	0.00	0.00	0.00
ZNF395	0.00	0.00	0.00	0.32	0.00	0.51	0.15	0.38	0.00	0.00	0.00	0.19	0.00	0.00	0.23	0.00	0.00	0.00	0.00	0.00	0.00
NUFIP2	0.00	0.00	0.00	0.27	0.00	0.46	0.10	0.17	0.00	0.00	0.00	0.17	0.00	0.00	0.14	0.00	0.00	0.00	0.00	0.00	0.00
ZNF219	0.00	0.00	0.00	0.30	0.00	0.55	0.11	0.34	0.00	0.00	0.00	0.22	0.00	0.00	0.26	0.00	0.00	0.00	0.00	0.00	0.00
RNF146	0.00	0.00	0.00	0.21	0.00	0.32	0.10	0.21	0.00	0.00	0.00	0.20	0.00	0.00	0.16	0.00	0.00	0.00	0.00	0.00	0.00
MUTED	0.00	0.00	0.00	0.21	0.00	0.28	0.12	0.13	0.00	0.00	0.00	0.17	0.00	0.00	0.16	0.00	0.00	0.00	0.00	0.00	0.00
PSMC2	0.00	0.00	0.00	0.28	0.00	0.40	0.14	0.26	0.00	0.00	0.00	0.16	0.00	0.00	0.25	0.00	0.00	0.00	0.00	0.00	0.00
MYO1F	0.00	0.00	0.00	0.24	0.00	0.40	0.10	0.19	0.00	0.00	0.00	0.13	0.00	0.00	0.15	0.00	0.00	0.00	0.00	0.00	0.00

KPTN	0.00	0.00	0.00	0.24	0.00	0.39	0.11	0.15	0.00	0.00	0.00	0.12	0.00	0.00	0.18	0.00	0.00	0.00	0.00	0.00	0.00
TBX19	0.00	0.00	0.00	0.25	0.00	0.31	0.15	0.22	0.00	0.00	0.00	0.12	0.00	0.00	0.24	0.00	0.00	0.00	0.00	0.00	0.00
SFSWAP	0.00	0.00	0.00	0.33	0.00	0.52	0.14	0.16	0.00	0.00	0.00	0.22	0.00	0.00	0.23	0.00	0.00	0.00	0.00	0.00	0.00
USP12	0.00	0.00	0.00	0.29	0.00	0.45	0.13	0.24	0.00	0.00	0.00	0.18	0.00	0.00	0.22	0.00	0.00	0.00	0.00	0.00	0.00
DGCR14	0.00	0.00	0.00	0.25	0.00	0.41	0.10	0.17	0.00	0.00	0.00	0.23	0.00	0.00	0.26	0.00	0.00	0.00	0.00	0.00	0.00
DACH2	0.00	0.00	0.00	0.24	0.00	0.29	0.14	0.28	0.00	0.00	0.00	0.21	0.00	0.00	0.26	0.00	0.00	0.00	0.00	0.00	0.00
PSMA1	0.00	0.00	0.00	0.29	0.00	0.46	0.12	0.22	0.00	0.00	0.00	0.15	0.00	0.00	0.16	0.00	0.00	0.00	0.00	0.00	0.00
UBE2E2	0.00	0.00	0.00	0.24	0.00	0.31	0.13	0.21	0.00	0.00	0.00	0.14	0.00	0.00	0.18	0.00	0.00	0.00	0.00	0.00	0.00
PJA1	0.00	0.00	0.00	0.27	0.00	0.41	0.13	0.35	0.00	0.00	0.00	0.21	0.00	0.00	0.19	0.00	0.00	0.00	0.00	0.00	0.00
FIGN	0.00	0.00	0.00	0.23	0.00	0.37	0.12	0.16	0.00	0.00	0.00	0.18	0.00	0.00	0.24	0.00	0.00	0.00	0.00	0.00	0.00
KIF20B	0.00	0.00	0.00	0.31	0.00	0.55	0.12	0.18	0.00	0.00	0.00	0.19	0.00	0.00	0.21	0.00	0.00	0.00	0.00	0.00	0.00
TBX22	0.00	0.00	0.00	0.23	0.00	0.32	0.13	0.21	0.00	0.00	0.00	0.13	0.00	0.00	0.20	0.00	0.00	0.00	0.00	0.00	0.00
OBSL1	0.00	0.00	0.00	0.27	0.00	0.48	0.11	0.17	0.00	0.00	0.00	0.16	0.00	0.00	0.18	0.00	0.00	0.00	0.00	0.00	0.00
HPS4	0.00	0.00	0.00	0.19	0.00	0.22	0.11	0.10	0.00	0.00	0.00	0.11	0.00	0.00	0.14	0.00	0.00	0.00	0.00	0.00	0.00
UBE2H	0.00	0.00	0.00	0.32	0.00	0.48	0.16	0.27	0.00	0.00	0.00	0.20	0.00	0.00	0.23	0.00	0.00	0.00	0.00	0.00	0.00
MID2	0.00	0.00	0.00	0.28	0.00	0.51	0.12	0.18	0.00	0.00	0.00	0.20	0.00	0.00	0.25	0.00	0.00	0.00	0.00	0.00	0.00
HPS6	0.00	0.00	0.00	0.20	0.00	0.24	0.12	0.16	0.00	0.00	0.00	0.12	0.00	0.00	0.13	0.00	0.00	0.00	0.00	0.00	0.00
MYO3B	0.00	0.00	0.00	0.28	0.00	0.46	0.13	0.14	0.00	0.00	0.00	0.24	0.00	0.00	0.24	0.00	0.00	0.00	0.00	0.00	0.00
MYO3A	0.00	0.00	0.00	0.22	0.00	0.33	0.10	0.18	0.00	0.00	0.00	0.15	0.00	0.00	0.18	0.00	0.00	0.00	0.00	0.00	0.00
FGF3	0.00	0.00	0.00	0.18	0.00	0.14	0.14	0.19	0.00	0.00	0.00	0.10	0.00	0.00	0.17	0.00	0.00	0.00	0.00	0.00	0.00
PSMA4	0.00	0.00	0.00	0.23	0.00	0.32	0.12	0.11	0.00	0.00	0.00	0.14	0.00	0.00	0.14	0.00	0.00	0.00	0.00	0.00	0.00
TWF2	0.00	0.00	0.00	0.27	0.00	0.45	0.11	0.19	0.00	0.00	0.00	0.20	0.00	0.00	0.21	0.00	0.00	0.00	0.00	0.00	0.00
PSMC6	0.00	0.00	0.00	0.30	0.00	0.46	0.14	0.26	0.00	0.00	0.00	0.21	0.00	0.00	0.25	0.00	0.00	0.00	0.00	0.00	0.00
TATDN1	0.00	0.00	0.00	0.27	0.00	0.48	0.11	0.28	0.00	0.00	0.00	0.21	0.00	0.00	0.16	0.00	0.00	0.00	0.00	0.00	0.00
TPRG1	0.00	0.00	0.00	0.21	0.00	0.32	0.10	0.17	0.00	0.00	0.00	0.12	0.00	0.00	0.14	0.00	0.00	0.00	0.00	0.00	0.00
PPP2R1B	0.00	0.00	0.00	0.23	0.00	0.34	0.11	0.26	0.00	0.00	0.00	0.17	0.00	0.00	0.12	0.00	0.00	0.00	0.00	0.00	0.00
AP3B1	0.00	0.00	0.00	0.22	0.00	0.24	0.15	0.15	0.00	0.00	0.00	0.15	0.00	0.00	0.19	0.00	0.00	0.00	0.00	0.00	0.00
FGD1	0.00	0.00	0.00	0.25	0.00	0.36	0.12	0.17	0.00	0.00	0.00	0.15	0.00	0.00	0.19	0.00	0.00	0.00	0.00	0.00	0.00
PSMA3	0.00	0.00	0.00	0.31	0.00	0.46	0.15	0.27	0.00	0.00	0.00	0.15	0.00	0.00	0.22	0.00	0.00	0.00	0.00	0.00	0.00
HAUS3	0.00	0.00	0.00	0.24	0.00	0.40	0.10	0.14	0.00	0.00	0.00	0.15	0.00	0.00	0.20	0.00	0.00	0.00	0.00	0.00	0.00
SPEF2	0.00	0.00	0.00	0.28	0.00	0.46	0.11	0.15	0.00	0.00	0.00	0.20	0.00	0.00	0.20	0.00	0.00	0.00	0.00	0.00	0.00
HSF4	0.00	0.00	0.00	0.23	0.00	0.30	0.13	0.23	0.00	0.00	0.00	0.14	0.00	0.00	0.22	0.00	0.00	0.00	0.00	0.00	0.00
CSMD3	0.00	0.00	0.00	0.23	0.00	0.35	0.11	0.14	0.00	0.00	0.00	0.13	0.00	0.00	0.11	0.00	0.00	0.00	0.00	0.00	0.00
PPP2CB	0.00	0.00	0.00	0.21	0.00	0.28	0.11	0.23	0.00	0.00	0.00	0.19	0.00	0.00	0.13	0.00	0.00	0.00	0.00	0.00	0.00
ANKRD49	0.00	0.00	0.00	0.27	0.00	0.39	0.15	0.14	0.00	0.00	0.00	0.13	0.00	0.00	0.15	0.00	0.00	0.00	0.00	0.00	0.00
SPAG16	0.00	0.00	0.00	0.27	0.00	0.45	0.11	0.15	0.00	0.00	0.00	0.18	0.00	0.00	0.20	0.00	0.00	0.00	0.00	0.00	0.00
RS1	0.00	0.00	0.00	0.19	0.00	0.23	0.11	0.00	0.15	0.00	0.00	0.11	0.00	0.00	0.19	0.00	0.00	0.00	0.00	0.00	0.00
SERPINA3	0.00	0.00	0.00	0.11	0.00	0.11	0.00	0.14	0.13	0.00	0.00	0.10	0.00	0.00	0.12	0.00	0.00	0.00	0.00	0.00	0.00
CEP290	0.00	0.00	0.00	0.18	0.00	0.25	0.00	0.12	0.16	0.00	0.00	0.14	0.00	0.00	0.20	0.00	0.00	0.00	0.00	0.00	0.00
COIL	0.00	0.00	0.00	0.20	0.00	0.39	0.00	0.23	0.11	0.00	0.00	0.10	0.00	0.00	0.21	0.00	0.00	0.00	0.00	0.00	0.00

THAP1	0.00	0.00	0.00	0.18	0.00	0.30	0.00	0.23	0.13	0.00	0.00	0.13	0.00	0.00	0.21	0.00	0.00	0.00	0.00	0.00	0.00
FRG2B	0.00	0.00	0.00	0.22	0.00	0.40	0.00	0.12	0.10	0.00	0.00	0.11	0.00	0.00	0.12	0.00	0.00	0.00	0.00	0.00	0.00
DFNA5	0.00	0.00	0.00	0.21	0.00	0.32	0.00	0.28	0.14	0.00	0.00	0.10	0.00	0.00	0.13	0.00	0.00	0.00	0.00	0.00	0.00
TAR	0.00	0.00	0.00	0.16	0.00	0.28	0.00	0.32	0.19	0.00	0.00	0.10	0.00	0.00	0.15	0.00	0.00	0.00	0.00	0.00	0.00
AFF4	0.00	0.00	0.00	0.21	0.00	0.35	0.00	0.36	0.12	0.00	0.00	0.14	0.00	0.00	0.17	0.00	0.00	0.00	0.00	0.00	0.00
GTSCR1	0.00	0.00	0.00	0.16	0.00	0.29	0.00	0.11	0.13	0.00	0.00	0.14	0.00	0.00	0.18	0.00	0.00	0.00	0.00	0.00	0.00
MCPH1	0.00	0.00	0.00	0.17	0.00	0.22	0.00	0.20	0.16	0.00	0.00	0.10	0.00	0.00	0.15	0.00	0.00	0.00	0.00	0.00	0.00
WHSC2	0.00	0.00	0.00	0.24	0.00	0.40	0.00	0.32	0.13	0.00	0.00	0.13	0.00	0.00	0.19	0.00	0.00	0.00	0.00	0.00	0.00
CAPN3	0.00	0.00	0.00	0.13	0.00	0.16	0.00	0.15	0.21	0.00	0.00	0.14	0.00	0.00	0.19	0.00	0.00	0.00	0.00	0.00	0.00
NEK1	0.00	0.00	0.00	0.22	0.00	0.35	0.00	0.20	0.15	0.00	0.00	0.12	0.00	0.00	0.16	0.00	0.00	0.00	0.00	0.00	0.00
ERVW-4	0.00	0.00	0.00	0.18	0.00	0.34	0.00	0.15	0.16	0.00	0.00	0.14	0.00	0.00	0.16	0.00	0.00	0.00	0.00	0.00	0.00
COCH	0.00	0.00	0.00	0.17	0.00	0.25	0.00	0.14	0.15	0.00	0.00	0.12	0.00	0.00	0.21	0.00	0.00	0.00	0.00	0.00	0.00
MIR562	0.00	0.00	0.00	0.15	0.00	0.28	0.00	0.28	0.18	0.00	0.00	0.12	0.00	0.00	0.11	0.00	0.00	0.00	0.00	0.00	0.00
IFT122	0.00	0.00	0.00	0.22	0.00	0.37	0.00	0.15	0.10	0.00	0.00	0.12	0.00	0.00	0.16	0.00	0.00	0.00	0.00	0.00	0.00
KY	0.00	0.00	0.00	0.19	0.00	0.30	0.00	0.12	0.12	0.00	0.00	0.14	0.00	0.00	0.23	0.00	0.00	0.00	0.00	0.00	0.00
TSEN54	0.00	0.00	0.00	0.22	0.00	0.44	0.00	0.30	0.10	0.00	0.00	0.15	0.00	0.00	0.21	0.00	0.00	0.00	0.00	0.00	0.00
SIX1	0.00	0.00	0.00	0.13	0.00	0.18	0.00	0.27	0.17	0.00	0.00	0.13	0.00	0.00	0.19	0.00	0.00	0.00	0.00	0.00	0.00
UBR2	0.00	0.00	0.00	0.23	0.00	0.36	0.00	0.30	0.11	0.00	0.00	0.11	0.00	0.00	0.16	0.00	0.00	0.00	0.00	0.00	0.00
ANKRD1	0.00	0.00	0.00	0.19	0.00	0.27	0.00	0.24	0.18	0.00	0.00	0.14	0.00	0.00	0.21	0.00	0.00	0.00	0.00	0.00	0.00
EIF2AK2	0.00	0.00	0.00	0.11	0.00	0.12	0.00	0.16	0.16	0.00	0.00	0.12	0.00	0.00	0.14	0.00	0.00	0.00	0.00	0.00	0.00
LOC647859	0.00	0.00	0.00	0.18	0.00	0.29	0.00	0.12	0.20	0.00	0.00	0.14	0.00	0.00	0.21	0.00	0.00	0.00	0.00	0.00	0.00
ZNF644	0.00	0.00	0.00	0.21	0.00	0.39	0.00	0.35	0.12	0.00	0.00	0.12	0.00	0.00	0.16	0.00	0.00	0.00	0.00	0.00	0.00
TMEM127	0.00	0.00	0.00	0.19	0.00	0.30	0.00	0.20	0.12	0.00	0.00	0.11	0.00	0.00	0.17	0.00	0.00	0.00	0.00	0.00	0.00
DLEU2	0.00	0.00	0.00	0.21	0.00	0.37	0.00	0.27	0.14	0.00	0.00	0.13	0.00	0.00	0.15	0.00	0.00	0.00	0.00	0.00	0.00
LYST	0.00	0.00	0.00	0.14	0.00	0.13	0.00	0.16	0.17	0.00	0.00	0.11	0.00	0.00	0.15	0.00	0.00	0.00	0.00	0.00	0.00
SETBP1	0.00	0.00	0.00	0.22	0.00	0.41	0.00	0.25	0.17	0.00	0.00	0.17	0.00	0.00	0.17	0.00	0.00	0.00	0.00	0.00	0.00
UBAP1	0.00	0.00	0.00	0.20	0.00	0.36	0.00	0.16	0.15	0.00	0.00	0.16	0.00	0.00	0.15	0.00	0.00	0.00	0.00	0.00	0.00
ANKRD2	0.00	0.00	0.00	0.22	0.00	0.36	0.00	0.24	0.15	0.00	0.00	0.16	0.00	0.00	0.19	0.00	0.00	0.00	0.00	0.00	0.00
HTT-AS1	0.00	0.00	0.00	0.15	0.00	0.21	0.00	0.16	0.16	0.00	0.00	0.18	0.00	0.00	0.13	0.00	0.00	0.00	0.00	0.00	0.00
MIR137	0.00	0.00	0.00	0.19	0.00	0.31	0.00	0.45	0.28	0.00	0.00	0.18	0.00	0.00	0.15	0.00	0.00	0.00	0.00	0.00	0.00
RPL35A	0.00	0.00	0.00	0.26	0.00	0.49	0.00	0.20	0.13	0.00	0.00	0.16	0.00	0.00	0.16	0.00	0.00	0.00	0.00	0.00	0.00
SEP-09	0.00	0.00	0.00	0.18	0.00	0.33	0.00	0.27	0.20	0.00	0.00	0.16	0.00	0.00	0.21	0.00	0.00	0.00	0.00	0.00	0.00
SIX5	0.00	0.00	0.00	0.17	0.00	0.26	0.00	0.22	0.12	0.00	0.00	0.20	0.00	0.00	0.23	0.00	0.00	0.00	0.00	0.00	0.00
NBPF23	0.00	0.00	0.00	0.20	0.00	0.31	0.00	0.14	0.16	0.00	0.00	0.19	0.00	0.00	0.18	0.00	0.00	0.00	0.00	0.00	0.00
FAM134A	0.00	0.00	0.00	0.22	0.00	0.40	0.00	0.36	0.27	0.00	0.00	0.19	0.00	0.00	0.21	0.00	0.00	0.00	0.00	0.00	0.00
NAPSA	0.00	0.00	0.00	0.18	0.00	0.30	0.00	0.21	0.17	0.00	0.00	0.15	0.00	0.00	0.16	0.00	0.00	0.00	0.00	0.00	0.00
EPC2	0.00	0.00	0.00	0.20	0.00	0.37	0.00	0.23	0.12	0.00	0.00	0.18	0.00	0.00	0.18	0.00	0.00	0.00	0.00	0.00	0.00
NXNL2	0.00	0.00	0.00	0.23	0.00	0.42	0.00	0.18	0.12	0.00	0.00	0.15	0.00	0.00	0.19	0.00	0.00	0.00	0.00	0.00	0.00
HSF2	0.00	0.00	0.00	0.21	0.00	0.31	0.00	0.32	0.11	0.00	0.00	0.15	0.00	0.00	0.24	0.00	0.00	0.00	0.00	0.00	0.00
KIF4B	0.00	0.00	0.00	0.33	0.00	0.57	0.13	0.00	0.00	0.00	0.12	0.30	0.00	0.00	0.23	0.00	0.00	0.00	0.00	0.00	0.00

AP4E1	0.00	0.00	0.00	0.28	0.00	0.45	0.13	0.00	0.00	0.00	0.12	0.20	0.00	0.00	0.20	0.00	0.00	0.00	0.00	0.00	0.00
NKAIN3	0.00	0.00	0.00	0.28	0.00	0.50	0.12	0.00	0.00	0.00	0.26	0.27	0.00	0.00	0.25	0.00	0.00	0.00	0.00	0.00	0.00
AP4S1	0.00	0.00	0.00	0.29	0.00	0.46	0.15	0.00	0.00	0.00	0.15	0.19	0.00	0.00	0.17	0.00	0.00	0.00	0.00	0.00	0.00
JOSD2	0.00	0.00	0.00	0.24	0.00	0.40	0.11	0.00	0.00	0.00	0.12	0.16	0.00	0.00	0.14	0.00	0.00	0.00	0.00	0.00	0.00
KRTAP24-1	0.00	0.00	0.00	0.33	0.00	0.60	0.12	0.00	0.00	0.00	0.15	0.17	0.00	0.00	0.15	0.00	0.00	0.00	0.00	0.00	0.00
NKAIN1	0.00	0.00	0.00	0.28	0.00	0.50	0.12	0.00	0.00	0.00	0.26	0.27	0.00	0.00	0.25	0.00	0.00	0.00	0.00	0.00	0.00
LHFPL1	0.00	0.00	0.00	0.29	0.00	0.49	0.12	0.00	0.00	0.00	0.27	0.20	0.00	0.00	0.17	0.00	0.00	0.00	0.00	0.00	0.00
TMC5	0.00	0.00	0.00	0.30	0.00	0.51	0.13	0.00	0.00	0.00	0.32	0.20	0.00	0.00	0.20	0.00	0.00	0.00	0.00	0.00	0.00
OTOG	0.00	0.00	0.00	0.24	0.00	0.36	0.13	0.00	0.00	0.00	0.19	0.18	0.00	0.00	0.22	0.00	0.00	0.00	0.00	0.00	0.00
TMC3	0.00	0.00	0.00	0.30	0.00	0.51	0.13	0.00	0.00	0.00	0.32	0.20	0.00	0.00	0.20	0.00	0.00	0.00	0.00	0.00	0.00
TMC7	0.00	0.00	0.00	0.30	0.00	0.51	0.13	0.00	0.00	0.00	0.32	0.20	0.00	0.00	0.20	0.00	0.00	0.00	0.00	0.00	0.00
MFRP-C1QTNF5	0.00	0.00	0.00	0.23	0.00	0.37	0.10	0.00	0.00	0.00	0.14	0.18	0.00	0.00	0.20	0.00	0.00	0.00	0.00	0.00	0.00
MFAP4	0.00	0.00	0.00	0.27	0.00	0.39	0.14	0.00	0.00	0.00	0.15	0.17	0.00	0.00	0.18	0.00	0.00	0.00	0.00	0.00	0.00
KCNK16	0.00	0.00	0.00	0.21	0.00	0.26	0.11	0.00	0.00	0.00	0.20	0.17	0.00	0.00	0.13	0.00	0.00	0.00	0.00	0.00	0.00
TMC4	0.00	0.00	0.00	0.30	0.00	0.51	0.13	0.00	0.00	0.00	0.32	0.20	0.00	0.00	0.20	0.00	0.00	0.00	0.00	0.00	0.00
SPPL2C	0.00	0.00	0.00	0.20	0.00	0.27	0.12	0.00	0.00	0.00	0.16	0.13	0.00	0.00	0.11	0.00	0.00	0.00	0.00	0.00	0.00
ALS2CR12	0.00	0.00	0.00	0.29	0.00	0.62	0.00	0.15	0.00	0.00	0.12	0.21	0.00	0.00	0.12	0.00	0.00	0.00	0.00	0.00	0.00
C9ORF11	0.00	0.00	0.00	0.21	0.00	0.37	0.00	0.12	0.00	0.00	0.12	0.11	0.00	0.00	0.13	0.00	0.00	0.00	0.00	0.00	0.00
CYYR1	0.00	0.00	0.00	0.29	0.00	0.59	0.00	0.13	0.00	0.00	0.14	0.25	0.00	0.00	0.17	0.00	0.00	0.00	0.00	0.00	0.00
SOBP	0.00	0.00	0.00	0.17	0.00	0.24	0.00	0.13	0.00	0.00	0.10	0.11	0.00	0.00	0.18	0.00	0.00	0.00	0.00	0.00	0.00
ZMYM3	0.00	0.00	0.00	0.25	0.00	0.42	0.00	0.11	0.00	0.00	0.12	0.26	0.00	0.00	0.20	0.00	0.00	0.00	0.00	0.00	0.00
ATP6V0B	0.00	0.00	0.00	0.28	0.00	0.54	0.00	0.15	0.00	0.00	0.22	0.21	0.00	0.00	0.17	0.00	0.00	0.00	0.00	0.00	0.00
GOLT1A	0.00	0.00	0.00	0.24	0.00	0.49	0.00	0.13	0.00	0.00	0.23	0.11	0.00	0.00	0.13	0.00	0.00	0.00	0.00	0.00	0.00
ERVW-2	0.00	0.00	0.00	0.25	0.00	0.45	0.00	0.12	0.00	0.00	0.12	0.19	0.00	0.00	0.18	0.00	0.00	0.00	0.00	0.00	0.00
SPTB	0.00	0.00	0.00	0.18	0.00	0.20	0.00	0.12	0.00	0.00	0.10	0.14	0.00	0.00	0.16	0.00	0.00	0.00	0.00	0.00	0.00
FER1L4	0.00	0.00	0.00	0.27	0.00	0.49	0.00	0.11	0.00	0.00	0.22	0.17	0.00	0.00	0.19	0.00	0.00	0.00	0.00	0.00	0.00
TRAPPC2	0.00	0.00	0.00	0.22	0.00	0.42	0.00	0.13	0.00	0.00	0.10	0.13	0.00	0.00	0.17	0.00	0.00	0.00	0.00	0.00	0.00
ATL3	0.00	0.00	0.00	0.24	0.00	0.44	0.00	0.15	0.00	0.00	0.25	0.17	0.00	0.00	0.20	0.00	0.00	0.00	0.00	0.00	0.00
OTOF	0.00	0.00	0.00	0.19	0.00	0.24	0.00	0.14	0.00	0.00	0.14	0.17	0.00	0.00	0.22	0.00	0.00	0.00	0.00	0.00	0.00
EXTL1	0.00	0.00	0.00	0.18	0.00	0.36	0.00	0.11	0.00	0.00	0.12	0.14	0.00	0.00	0.14	0.00	0.00	0.00	0.00	0.00	0.00
KNCN	0.00	0.00	0.00	0.22	0.00	0.43	0.00	0.13	0.00	0.00	0.11	0.17	0.00	0.00	0.21	0.00	0.00	0.00	0.00	0.00	0.00
DFNB31	0.00	0.00	0.00	0.17	0.00	0.24	0.00	0.13	0.00	0.00	0.13	0.13	0.00	0.00	0.20	0.00	0.00	0.00	0.00	0.00	0.00
POF1B	0.00	0.00	0.00	0.23	0.00	0.45	0.00	0.12	0.00	0.00	0.13	0.13	0.00	0.00	0.15	0.00	0.00	0.00	0.00	0.00	0.00
FAM188B2	0.00	0.00	0.00	0.27	0.00	0.52	0.00	0.17	0.00	0.00	0.12	0.18	0.00	0.00	0.19	0.00	0.00	0.00	0.00	0.00	0.00
NAT8B	0.00	0.00	0.00	0.24	0.00	0.46	0.00	0.18	0.00	0.00	0.26	0.18	0.00	0.00	0.15	0.00	0.00	0.00	0.00	0.00	0.00
TTL	0.00	0.00	0.00	0.23	0.00	0.46	0.00	0.17	0.00	0.00	0.11	0.19	0.00	0.00	0.12	0.00	0.00	0.00	0.00	0.00	0.00
PIGB	0.00	0.00	0.00	0.24	0.00	0.42	0.00	0.13	0.00	0.00	0.23	0.12	0.00	0.00	0.12	0.00	0.00	0.00	0.00	0.00	0.00
RFT1	0.00	0.00	0.00	0.25	0.00	0.47	0.00	0.11	0.00	0.00	0.33	0.11	0.00	0.00	0.11	0.00	0.00	0.00	0.00	0.00	0.00
KCNE3	0.00	0.00	0.00	0.16	0.00	0.20	0.00	0.11	0.00	0.00	0.17	0.15	0.00	0.00	0.17	0.00	0.00	0.00	0.00	0.00	0.00
FKRP	0.00	0.00	0.00	0.13	0.00	0.19	0.00	0.00	0.20	0.00	0.13	0.16	0.00	0.00	0.20	0.00	0.00	0.00	0.00	0.00	0.00

ANO5	0.00	0.00	0.00	0.21	0.00	0.37	0.00	0.00	0.10	0.00	0.21	0.15	0.00	0.00	0.17	0.00	0.00	0.00	0.00	0.00	0.00
MKS1	0.00	0.00	0.00	0.18	0.00	0.28	0.00	0.00	0.11	0.00	0.11	0.15	0.00	0.00	0.22	0.00	0.00	0.00	0.00	0.00	0.00
TMEM67	0.00	0.00	0.00	0.17	0.00	0.28	0.00	0.00	0.12	0.00	0.16	0.17	0.00	0.00	0.19	0.00	0.00	0.00	0.00	0.00	0.00
NPHP1	0.00	0.00	0.00	0.19	0.00	0.28	0.00	0.00	0.13	0.00	0.11	0.12	0.00	0.00	0.17	0.00	0.00	0.00	0.00	0.00	0.00
VPS13A	0.00	0.00	0.00	0.20	0.00	0.30	0.00	0.00	0.13	0.00	0.11	0.20	0.00	0.00	0.20	0.00	0.00	0.00	0.00	0.00	0.00
EDNRA	0.00	0.00	0.17	0.17	0.00	0.00	0.14	0.00	0.12	0.00	0.00	0.00	0.11	0.00	0.13	0.00	0.00	0.00	0.00	0.00	0.00
MET	0.00	0.00	0.00	0.12	0.00	0.00	0.10	0.13	0.17	0.00	0.00	0.00	0.10	0.00	0.12	0.00	0.00	0.00	0.00	0.00	0.00
PHF6	0.00	0.00	0.00	0.17	0.00	0.33	0.00	0.20	0.00	0.00	0.00	0.21	0.12	0.00	0.22	0.00	0.00	0.00	0.00	0.00	0.00
KRT83	0.00	0.00	0.00	0.21	0.00	0.40	0.00	0.11	0.00	0.00	0.00	0.13	0.10	0.00	0.20	0.00	0.00	0.00	0.00	0.00	0.00
WNK3	0.00	0.00	0.00	0.18	0.00	0.22	0.00	0.18	0.00	0.00	0.00	0.16	0.13	0.00	0.19	0.00	0.00	0.00	0.00	0.00	0.00
DSEL	0.00	0.00	0.00	0.25	0.00	0.48	0.00	0.12	0.00	0.00	0.00	0.30	0.13	0.00	0.27	0.00	0.00	0.00	0.00	0.00	0.00
ZFYVE26	0.00	0.00	0.00	0.19	0.00	0.34	0.00	0.11	0.00	0.00	0.00	0.21	0.11	0.00	0.23	0.00	0.00	0.00	0.00	0.00	0.00
KIF4A	0.00	0.00	0.00	0.25	0.00	0.42	0.00	0.30	0.00	0.00	0.00	0.21	0.13	0.00	0.27	0.00	0.00	0.00	0.00	0.00	0.00
MYO5A	0.00	0.00	0.00	0.15	0.00	0.13	0.00	0.13	0.00	0.00	0.00	0.14	0.11	0.00	0.15	0.00	0.00	0.00	0.00	0.00	0.00
VCX3A	0.00	0.00	0.00	0.26	0.00	0.49	0.00	0.15	0.00	0.00	0.00	0.24	0.10	0.00	0.23	0.00	0.00	0.00	0.00	0.00	0.00
DCTN1	0.00	0.00	0.00	0.18	0.00	0.27	0.00	0.15	0.00	0.00	0.00	0.19	0.13	0.00	0.22	0.00	0.00	0.00	0.00	0.00	0.00
OXSR1	0.00	0.00	0.00	0.18	0.00	0.22	0.00	0.14	0.00	0.00	0.00	0.13	0.11	0.00	0.18	0.00	0.00	0.00	0.00	0.00	0.00
ELP4	0.00	0.00	0.00	0.25	0.00	0.46	0.00	0.30	0.00	0.00	0.00	0.15	0.11	0.00	0.21	0.00	0.00	0.00	0.00	0.00	0.00
MBD5	0.00	0.00	0.00	0.16	0.00	0.33	0.00	0.12	0.00	0.00	0.00	0.22	0.11	0.00	0.17	0.00	0.00	0.00	0.00	0.00	0.00
ZNF365	0.00	0.00	0.00	0.18	0.00	0.32	0.00	0.10	0.00	0.00	0.00	0.16	0.12	0.00	0.15	0.00	0.00	0.00	0.00	0.00	0.00
DNAI1	0.00	0.00	0.00	0.18	0.00	0.27	0.00	0.00	0.11	0.00	0.00	0.21	0.12	0.00	0.25	0.00	0.00	0.00	0.00	0.00	0.00
KRT71	0.00	0.00	0.00	0.20	0.00	0.32	0.00	0.00	0.00	0.00	0.11	0.16	0.10	0.00	0.24	0.00	0.00	0.00	0.00	0.00	0.00
C21ORF91	0.00	0.00	0.00	0.23	0.00	0.42	0.00	0.00	0.00	0.00	0.10	0.23	0.12	0.00	0.27	0.00	0.00	0.00	0.00	0.00	0.00
TIMM8AP1	0.00	0.00	0.00	0.18	0.00	0.40	0.00	0.00	0.00	0.00	0.12	0.27	0.11	0.00	0.20	0.00	0.00	0.00	0.00	0.00	0.00
DYM	0.00	0.00	0.00	0.24	0.00	0.40	0.00	0.00	0.00	0.00	0.18	0.22	0.10	0.00	0.21	0.00	0.00	0.00	0.00	0.00	0.00
FREM2	0.00	0.00	0.00	0.18	0.00	0.28	0.00	0.00	0.00	0.00	0.12	0.16	0.11	0.00	0.24	0.00	0.00	0.00	0.00	0.00	0.00
EJM2	0.00	0.00	0.00	0.13	0.00	0.14	0.10	0.00	0.00	0.00	0.00	0.14	0.00	0.13	0.13	0.00	0.00	0.00	0.00	0.00	0.00
HECTD2	0.00	0.00	0.00	0.11	0.00	0.16	0.00	0.00	0.10	0.00	0.00	0.13	0.00	0.10	0.12	0.00	0.00	0.00	0.00	0.00	0.00
CACNA1C	0.00	0.00	0.00	0.11	0.00	0.00	0.00	0.13	0.13	0.00	0.00	0.13	0.00	0.16	0.13	0.00	0.00	0.00	0.00	0.00	0.00
FRRS1L	0.00	0.00	0.00	0.23	0.00	0.48	0.00	0.00	0.00	0.00	0.14	0.26	0.00	0.11	0.14	0.00	0.00	0.00	0.00	0.00	0.00
PCNX	0.00	0.00	0.00	0.26	0.00	0.57	0.00	0.00	0.00	0.00	0.00	0.31	0.12	0.11	0.21	0.00	0.00	0.00	0.00	0.00	0.00
ADHD1	0.00	0.00	0.00	0.11	0.00	0.10	0.00	0.00	0.00	0.00	0.00	0.13	0.16	0.13	0.15	0.00	0.00	0.00	0.00	0.00	0.00
ODF3L2	0.00	0.00	0.00	0.13	0.00	0.15	0.00	0.00	0.00	0.00	0.00	0.18	0.16	0.12	0.17	0.00	0.00	0.00	0.00	0.00	0.00
NLGN4Y	0.00	0.00	0.00	0.17	0.00	0.23	0.00	0.00	0.00	0.00	0.00	0.19	0.12	0.11	0.18	0.00	0.00	0.00	0.00	0.00	0.00
CPAT1	0.00	0.00	0.00	0.11	0.00	0.23	0.00	0.00	0.00	0.00	0.00	0.20	0.15	0.10	0.21	0.00	0.00	0.00	0.00	0.00	0.00
TUBA3D	0.00	0.00	0.00	0.19	0.00	0.39	0.00	0.00	0.00	0.00	0.00	0.29	0.11	0.11	0.18	0.00	0.00	0.00	0.00	0.00	0.00
AUTS5	0.00	0.00	0.00	0.13	0.00	0.21	0.00	0.00	0.00	0.00	0.00	0.20	0.14	0.10	0.17	0.00	0.00	0.00	0.00	0.00	0.00
EFHC2	0.00	0.00	0.00	0.17	0.00	0.27	0.00	0.00	0.00	0.00	0.00	0.21	0.15	0.16	0.17	0.00	0.00	0.00	0.00	0.00	0.00
MAFD1	0.00	0.00	0.00	0.12	0.00	0.17	0.00	0.00	0.00	0.00	0.00	0.16	0.15	0.11	0.15	0.00	0.00	0.00	0.00	0.00	0.00
PTCHD1	0.00	0.00	0.00	0.19	0.00	0.29	0.00	0.00	0.00	0.00	0.00	0.26	0.15	0.13	0.22	0.00	0.00	0.00	0.00	0.00	0.00

FAM190A	0.00	0.00	0.00	0.15	0.00	0.19	0.00	0.00	0.00	0.00	0.00	0.23	0.21	0.14	0.19	0.00	0.00	0.00	0.00	0.00	0.00
DAOA-AS1	0.00	0.00	0.00	0.17	0.00	0.20	0.00	0.00	0.00	0.00	0.00	0.17	0.19	0.14	0.16	0.00	0.00	0.00	0.00	0.00	0.00
TSNAX-DISC1	0.00	0.00	0.00	0.14	0.00	0.28	0.00	0.00	0.00	0.00	0.00	0.24	0.21	0.19	0.14	0.00	0.00	0.00	0.00	0.00	0.00
LOC728637	0.00	0.00	0.00	0.13	0.00	0.17	0.00	0.00	0.00	0.00	0.00	0.13	0.15	0.11	0.15	0.00	0.00	0.00	0.00	0.00	0.00
CNTNAP5	0.00	0.00	0.00	0.19	0.00	0.29	0.00	0.00	0.00	0.00	0.00	0.25	0.16	0.11	0.26	0.00	0.00	0.00	0.00	0.00	0.00
TTBK2	0.00	0.00	0.00	0.18	0.00	0.31	0.00	0.00	0.00	0.00	0.00	0.24	0.11	0.10	0.19	0.00	0.00	0.00	0.00	0.00	0.00
DISC2	0.00	0.00	0.00	0.17	0.00	0.30	0.00	0.00	0.00	0.00	0.00	0.22	0.19	0.15	0.20	0.00	0.00	0.00	0.00	0.00	0.00
SSD	0.00	0.00	0.00	0.15	0.00	0.16	0.00	0.00	0.00	0.00	0.00	0.23	0.21	0.17	0.18	0.00	0.00	0.00	0.00	0.00	0.00
SCZD12	0.00	0.00	0.00	0.11	0.00	0.15	0.00	0.00	0.00	0.00	0.00	0.16	0.15	0.11	0.17	0.00	0.00	0.00	0.00	0.00	0.00
DYX5	0.00	0.00	0.00	0.13	0.00	0.24	0.00	0.00	0.00	0.00	0.00	0.17	0.15	0.10	0.14	0.00	0.00	0.00	0.00	0.00	0.00
SCAX4	0.00	0.00	0.00	0.00	0.00	0.18	0.00	0.00	0.11	0.00	0.00	0.17	0.10	0.12	0.12	0.00	0.00	0.00	0.00	0.00	0.00
LRRK2	0.00	0.00	0.00	0.00	0.00	0.00	0.00	0.12	0.18	0.00	0.00	0.15	0.15	0.11	0.17	0.00	0.00	0.00	0.00	0.00	0.00
PCDH19	0.00	0.00	0.00	0.00	0.00	0.17	0.00	0.00	0.00	0.00	0.10	0.21	0.15	0.15	0.19	0.00	0.00	0.00	0.00	0.00	0.00
C1QA	0.00	0.00	0.00	0.15	0.00	0.00	0.12	0.14	0.20	0.00	0.00	0.00	0.00	0.00	0.20	0.13	0.00	0.00	0.00	0.00	0.00
CCL3	0.00	0.00	0.00	0.12	0.00	0.00	0.00	0.13	0.18	0.13	0.00	0.00	0.00	0.00	0.13	0.14	0.00	0.00	0.00	0.00	0.00
C11ORF9	0.00	0.00	0.00	0.30	0.00	0.62	0.00	0.17	0.00	0.00	0.00	0.25	0.00	0.00	0.34	0.22	0.00	0.00	0.00	0.00	0.00
HMX1	0.00	0.00	0.00	0.21	0.00	0.31	0.00	0.20	0.00	0.00	0.00	0.19	0.00	0.00	0.34	0.12	0.00	0.00	0.00	0.00	0.00
ZFP57	0.00	0.00	0.00	0.21	0.00	0.37	0.00	0.23	0.00	0.00	0.00	0.16	0.00	0.00	0.24	0.11	0.00	0.00	0.00	0.00	0.00
HOXB2	0.00	0.00	0.00	0.13	0.00	0.17	0.00	0.26	0.00	0.00	0.00	0.12	0.00	0.00	0.27	0.15	0.00	0.00	0.00	0.00	0.00
YPEL1	0.00	0.00	0.00	0.29	0.00	0.60	0.00	0.20	0.00	0.00	0.00	0.22	0.00	0.00	0.33	0.16	0.00	0.00	0.00	0.00	0.00
ARHGEF10	0.00	0.00	0.00	0.20	0.00	0.33	0.00	0.13	0.00	0.00	0.00	0.16	0.00	0.00	0.23	0.10	0.00	0.00	0.00	0.00	0.00
NEFL	0.00	0.00	0.00	0.14	0.00	0.11	0.00	0.13	0.00	0.00	0.00	0.15	0.00	0.00	0.28	0.22	0.00	0.00	0.00	0.00	0.00
SERF1B	0.00	0.00	0.00	0.12	0.00	0.22	0.00	0.00	0.21	0.00	0.00	0.12	0.00	0.00	0.24	0.13	0.00	0.00	0.00	0.00	0.00
CDH12P1	0.00	0.00	0.00	0.16	0.00	0.31	0.00	0.00	0.11	0.00	0.00	0.21	0.00	0.00	0.28	0.11	0.00	0.00	0.00	0.00	0.00
SERF1A	0.00	0.00	0.00	0.10	0.00	0.20	0.00	0.00	0.23	0.00	0.00	0.12	0.00	0.00	0.21	0.11	0.00	0.00	0.00	0.00	0.00
CDH12P3	0.00	0.00	0.00	0.16	0.00	0.31	0.00	0.00	0.11	0.00	0.00	0.21	0.00	0.00	0.28	0.11	0.00	0.00	0.00	0.00	0.00
CDH12P2	0.00	0.00	0.00	0.12	0.00	0.25	0.00	0.00	0.14	0.00	0.00	0.16	0.00	0.00	0.23	0.10	0.00	0.00	0.00	0.00	0.00
CCDC66	0.00	0.00	0.00	0.16	0.00	0.28	0.00	0.00	0.12	0.00	0.00	0.13	0.00	0.00	0.21	0.10	0.00	0.00	0.00	0.00	0.00
CDH12P4	0.00	0.00	0.00	0.16	0.00	0.31	0.00	0.00	0.11	0.00	0.00	0.21	0.00	0.00	0.28	0.11	0.00	0.00	0.00	0.00	0.00
ATL1	0.00	0.00	0.00	0.19	0.00	0.30	0.00	0.00	0.00	0.00	0.14	0.18	0.00	0.00	0.26	0.10	0.00	0.00	0.00	0.00	0.00
SACS	0.00	0.00	0.00	0.16	0.00	0.32	0.00	0.00	0.00	0.00	0.10	0.18	0.00	0.00	0.26	0.11	0.00	0.00	0.00	0.00	0.00
SBF2	0.00	0.00	0.00	0.22	0.00	0.35	0.00	0.00	0.00	0.00	0.12	0.16	0.00	0.00	0.29	0.18	0.00	0.00	0.00	0.00	0.00
CLDN19	0.00	0.00	0.00	0.20	0.00	0.35	0.00	0.00	0.00	0.00	0.20	0.16	0.00	0.00	0.23	0.12	0.00	0.00	0.00	0.00	0.00
SH3TC1	0.00	0.00	0.00	0.20	0.00	0.39	0.00	0.00	0.00	0.00	0.14	0.14	0.00	0.00	0.27	0.13	0.00	0.00	0.00	0.00	0.00
TUBB3	0.00	0.00	0.11	0.12	0.00	0.00	0.00	0.10	0.00	0.00	0.00	0.00	0.13	0.00	0.16	0.12	0.00	0.00	0.00	0.00	0.00
PHOX2B	0.00	0.00	0.00	0.12	0.00	0.11	0.00	0.18	0.00	0.00	0.00	0.00	0.11	0.00	0.31	0.15	0.00	0.00	0.00	0.00	0.00
TMCC1	0.00	0.00	0.00	0.24	0.00	0.48	0.00	0.00	0.00	0.00	0.00	0.27	0.12	0.00	0.36	0.14	0.00	0.00	0.00	0.00	0.00
HYLS1	0.00	0.00	0.00	0.12	0.00	0.25	0.00	0.00	0.00	0.00	0.00	0.23	0.16	0.00	0.27	0.14	0.00	0.00	0.00	0.00	0.00
CHST14	0.00	0.00	0.00	0.13	0.00	0.30	0.00	0.00	0.00	0.00	0.00	0.23	0.14	0.00	0.27	0.12	0.00	0.00	0.00	0.00	0.00
SOLH	0.00	0.00	0.00	0.31	0.00	0.66	0.00	0.00	0.00	0.00	0.00	0.33	0.12	0.00	0.31	0.11	0.00	0.00	0.00	0.00	0.00

PAX2	0.00	0.00	0.00	0.00	0.00	0.00	0.00	0.14	0.12	0.00	0.00	0.12	0.16	0.00	0.21	0.16	0.00	0.00	0.00	0.00	0.00
NGFR	0.00	0.00	0.00	0.11	0.00	0.00	0.00	0.11	0.15	0.00	0.00	0.00	0.00	0.11	0.15	0.14	0.00	0.00	0.00	0.00	0.00
NGF	0.00	0.00	0.00	0.11	0.00	0.00	0.00	0.13	0.16	0.00	0.00	0.00	0.00	0.14	0.16	0.11	0.00	0.00	0.00	0.00	0.00
RELN	0.00	0.00	0.00	0.11	0.00	0.00	0.00	0.12	0.00	0.00	0.00	0.00	0.26	0.21	0.13	0.13	0.00	0.00	0.00	0.00	0.00
NRG1	0.00	0.00	0.00	0.13	0.00	0.00	0.00	0.00	0.17	0.00	0.00	0.00	0.15	0.13	0.15	0.11	0.00	0.00	0.00	0.00	0.00
HPE6	0.00	0.00	0.00	0.00	0.00	0.22	0.00	0.00	0.00	0.00	0.00	0.30	0.18	0.18	0.23	0.16	0.00	0.00	0.00	0.00	0.00
CLAM	0.00	0.00	0.00	0.00	0.00	0.15	0.00	0.00	0.00	0.00	0.00	0.26	0.23	0.17	0.25	0.17	0.00	0.00	0.00	0.00	0.00
CALB2	0.00	0.00	0.00	0.00	0.00	0.00	0.00	0.15	0.00	0.00	0.00	0.10	0.25	0.21	0.18	0.16	0.00	0.00	0.00	0.00	0.00
SNAX1	0.00	0.00	0.00	0.00	0.00	0.00	0.00	0.00	0.11	0.00	0.00	0.19	0.15	0.17	0.19	0.18	0.00	0.00	0.00	0.00	0.00
LOC643387	0.00	0.00	0.00	0.00	0.00	0.00	0.00	0.00	0.19	0.00	0.00	0.19	0.17	0.20	0.22	0.17	0.00	0.00	0.00	0.00	0.00
PLAT	0.00	0.00	0.00	0.00	0.00	0.00	0.00	0.00	0.12	0.00	0.00	0.12	0.13	0.17	0.17	0.13	0.00	0.00	0.00	0.00	0.00
HYT2	0.00	0.00	0.11	0.15	0.00	0.15	0.10	0.00	0.00	0.00	0.00	0.00	0.00	0.00	0.12	0.00	0.13	0.00	0.00	0.00	0.00
BP8	0.00	0.00	0.11	0.15	0.00	0.15	0.10	0.00	0.00	0.00	0.00	0.00	0.00	0.00	0.12	0.00	0.13	0.00	0.00	0.00	0.00
BP24	0.00	0.00	0.14	0.20	0.00	0.20	0.13	0.00	0.00	0.00	0.00	0.00	0.00	0.00	0.15	0.00	0.14	0.00	0.00	0.00	0.00
SCAF4	0.00	0.00	0.00	0.25	0.00	0.39	0.11	0.25	0.00	0.00	0.00	0.00	0.00	0.00	0.12	0.00	0.23	0.00	0.00	0.00	0.00
EIF4A1P1	0.00	0.00	0.00	0.17	0.00	0.21	0.10	0.00	0.11	0.00	0.00	0.00	0.00	0.00	0.11	0.00	0.13	0.00	0.00	0.00	0.00
THM	0.00	0.00	0.00	0.17	0.00	0.26	0.00	0.17	0.22	0.00	0.00	0.00	0.00	0.00	0.13	0.00	0.19	0.00	0.00	0.00	0.00
KAZA	0.00	0.00	0.00	0.12	0.00	0.23	0.00	0.15	0.20	0.00	0.00	0.00	0.00	0.00	0.17	0.00	0.12	0.00	0.00	0.00	0.00
COL8A2	0.00	0.00	0.00	0.18	0.00	0.26	0.00	0.10	0.14	0.00	0.00	0.00	0.00	0.00	0.14	0.00	0.12	0.00	0.00	0.00	0.00
MYAS1	0.00	0.00	0.13	0.17	0.00	0.00	0.17	0.00	0.00	0.00	0.13	0.00	0.00	0.00	0.10	0.00	0.14	0.00	0.00	0.00	0.00
STQTL8	0.00	0.00	0.00	0.20	0.00	0.29	0.11	0.00	0.00	0.00	0.00	0.12	0.00	0.00	0.14	0.00	0.12	0.00	0.00	0.00	0.00
NAALADL2	0.00	0.00	0.00	0.21	0.00	0.29	0.11	0.00	0.00	0.00	0.00	0.15	0.00	0.00	0.15	0.00	0.12	0.00	0.00	0.00	0.00
SH3BGRL2	0.00	0.00	0.00	0.27	0.00	0.49	0.10	0.00	0.00	0.00	0.00	0.17	0.00	0.00	0.14	0.00	0.12	0.00	0.00	0.00	0.00
CCT7P2	0.00	0.00	0.00	0.18	0.00	0.22	0.10	0.00	0.00	0.00	0.00	0.15	0.00	0.00	0.11	0.00	0.10	0.00	0.00	0.00	0.00
USP40	0.00	0.00	0.00	0.14	0.00	0.23	0.00	0.12	0.00	0.00	0.00	0.10	0.00	0.00	0.13	0.00	0.11	0.00	0.00	0.00	0.00
DGCR	0.00	0.00	0.00	0.12	0.00	0.18	0.00	0.00	0.17	0.00	0.00	0.11	0.00	0.00	0.13	0.00	0.11	0.00	0.00	0.00	0.00
TMEM39A	0.00	0.00	0.00	0.12	0.00	0.13	0.00	0.00	0.11	0.00	0.00	0.10	0.00	0.00	0.14	0.00	0.17	0.00	0.00	0.00	0.00
CHCHD2P10	0.00	0.00	0.00	0.13	0.00	0.21	0.00	0.00	0.13	0.00	0.00	0.11	0.00	0.00	0.15	0.00	0.17	0.00	0.00	0.00	0.00
CTSB	0.00	0.00	0.00	0.10	0.00	0.00	0.00	0.13	0.17	0.00	0.00	0.10	0.00	0.00	0.11	0.00	0.11	0.00	0.00	0.00	0.00
KCNA7	0.00	0.00	0.00	0.17	0.00	0.39	0.00	0.00	0.00	0.00	0.14	0.23	0.00	0.00	0.18	0.00	0.11	0.00	0.00	0.00	0.00
TMCO1	0.00	0.00	0.00	0.25	0.00	0.50	0.00	0.00	0.00	0.00	0.16	0.21	0.00	0.00	0.17	0.00	0.10	0.00	0.00	0.00	0.00
SLC12A3	0.00	0.00	0.00	0.13	0.00	0.11	0.00	0.12	0.00	0.00	0.00	0.00	0.12	0.00	0.13	0.00	0.11	0.00	0.00	0.00	0.00
TAMM41	0.00	0.00	0.00	0.13	0.00	0.21	0.00	0.00	0.00	0.00	0.00	0.13	0.12	0.00	0.13	0.00	0.10	0.00	0.00	0.00	0.00
C7ORF58	0.00	0.00	0.00	0.20	0.00	0.34	0.00	0.00	0.00	0.00	0.00	0.19	0.10	0.00	0.15	0.00	0.11	0.00	0.00	0.00	0.00
LINC00271	0.00	0.00	0.00	0.18	0.00	0.28	0.00	0.00	0.00	0.00	0.00	0.20	0.13	0.00	0.17	0.00	0.17	0.00	0.00	0.00	0.00
DYX9	0.00	0.00	0.00	0.14	0.00	0.22	0.00	0.00	0.00	0.00	0.00	0.14	0.11	0.00	0.12	0.00	0.10	0.00	0.00	0.00	0.00
NKAPL	0.00	0.00	0.00	0.00	0.00	0.10	0.00	0.00	0.00	0.00	0.00	0.16	0.17	0.12	0.14	0.00	0.13	0.00	0.00	0.00	0.00
EGI	0.00	0.00	0.00	0.00	0.00	0.20	0.00	0.00	0.00	0.00	0.00	0.13	0.10	0.12	0.11	0.00	0.11	0.00	0.00	0.00	0.00
ISL1	0.00	0.00	0.00	0.14	0.00	0.00	0.11	0.19	0.00	0.00	0.00	0.00	0.00	0.00	0.25	0.17	0.11	0.00	0.00	0.00	0.00
FXYD1	0.00	0.00	0.00	0.18	0.00	0.19	0.00	0.12	0.00	0.00	0.14	0.14	0.00	0.00	0.00	0.00	0.00	0.16	0.00	0.00	0.00

CLCN1	0.00	0.00	0.00	0.12	0.00	0.12	0.00	0.00	0.17	0.00	0.15	0.00	0.00	0.00	0.16	0.00	0.00	0.11	0.00	0.00	0.00
HSPB3	0.00	0.00	0.00	0.24	0.00	0.44	0.00	0.11	0.00	0.00	0.00	0.19	0.00	0.00	0.22	0.00	0.00	0.11	0.00	0.00	0.00
KIF2B	0.00	0.00	0.00	0.16	0.00	0.28	0.00	0.16	0.00	0.00	0.00	0.12	0.00	0.00	0.18	0.00	0.00	0.11	0.00	0.00	0.00
ATP1A2	0.00	0.00	0.00	0.10	0.00	0.00	0.00	0.00	0.00	0.00	0.12	0.14	0.12	0.00	0.11	0.00	0.00	0.14	0.00	0.00	0.00
SCARB1	0.00	0.00	0.10	0.16	0.00	0.00	0.14	0.13	0.15	0.00	0.00	0.00	0.00	0.00	0.00	0.00	0.00	0.00	0.18	0.00	0.00
MIR422A	0.00	0.00	0.00	0.21	0.00	0.29	0.12	0.41	0.18	0.00	0.00	0.00	0.00	0.00	0.00	0.00	0.00	0.00	0.25	0.00	0.00
ACAT2	0.00	0.00	0.00	0.19	0.00	0.22	0.12	0.15	0.12	0.00	0.00	0.00	0.00	0.00	0.00	0.00	0.00	0.00	0.36	0.00	0.00
PLA2G4A	0.00	0.00	0.00	0.11	0.00	0.00	0.00	0.10	0.12	0.00	0.00	0.00	0.11	0.11	0.00	0.00	0.00	0.00	0.13	0.00	0.00
FUCA2	0.00	0.00	0.00	0.18	0.00	0.26	0.00	0.16	0.23	0.00	0.00	0.00	0.00	0.00	0.12	0.00	0.00	0.00	0.11	0.00	0.00
IMPDH1	0.00	0.00	0.00	0.16	0.00	0.23	0.00	0.15	0.21	0.00	0.00	0.00	0.00	0.00	0.14	0.00	0.00	0.00	0.18	0.00	0.00
APOBEC1	0.00	0.00	0.00	0.16	0.00	0.16	0.00	0.21	0.16	0.00	0.00	0.00	0.00	0.00	0.12	0.00	0.00	0.00	0.24	0.00	0.00
HNRNPA1	0.00	0.00	0.00	0.17	0.00	0.20	0.00	0.27	0.15	0.00	0.00	0.00	0.00	0.00	0.13	0.00	0.00	0.00	0.19	0.00	0.00
RECQL4	0.00	0.00	0.00	0.12	0.00	0.28	0.00	0.17	0.13	0.00	0.00	0.00	0.00	0.00	0.14	0.00	0.00	0.00	0.12	0.00	0.00
GEMIN4	0.00	0.00	0.00	0.19	0.00	0.38	0.00	0.27	0.14	0.00	0.00	0.00	0.00	0.00	0.16	0.00	0.00	0.00	0.11	0.00	0.00
DHFR	0.00	0.00	0.00	0.14	0.00	0.13	0.00	0.17	0.13	0.00	0.00	0.00	0.00	0.00	0.16	0.00	0.00	0.00	0.23	0.00	0.00
EIF2C2	0.00	0.00	0.00	0.15	0.00	0.22	0.00	0.45	0.24	0.00	0.00	0.00	0.00	0.00	0.14	0.00	0.00	0.00	0.11	0.00	0.00
DKC1	0.00	0.00	0.00	0.14	0.00	0.28	0.00	0.20	0.12	0.00	0.00	0.00	0.00	0.00	0.13	0.00	0.00	0.00	0.12	0.00	0.00
SMN2	0.00	0.00	0.00	0.13	0.00	0.16	0.00	0.15	0.24	0.00	0.00	0.00	0.00	0.00	0.17	0.00	0.00	0.00	0.11	0.00	0.00
ZMPSTE24	0.00	0.00	0.00	0.11	0.00	0.19	0.00	0.15	0.16	0.00	0.00	0.00	0.00	0.00	0.11	0.00	0.00	0.00	0.12	0.00	0.00
RDH12	0.00	0.00	0.00	0.13	0.00	0.19	0.00	0.11	0.15	0.00	0.00	0.00	0.00	0.00	0.11	0.00	0.00	0.00	0.20	0.00	0.00
KEAP1	0.00	0.00	0.00	0.12	0.00	0.12	0.00	0.23	0.18	0.00	0.00	0.00	0.00	0.00	0.13	0.00	0.00	0.00	0.10	0.00	0.00
ALG6	0.00	0.00	0.00	0.11	0.00	0.21	0.00	0.00	0.13	0.00	0.15	0.00	0.00	0.00	0.13	0.00	0.00	0.00	0.14	0.00	0.00
DIP2C	0.00	0.00	0.00	0.27	0.00	0.53	0.00	0.11	0.00	0.00	0.00	0.25	0.00	0.00	0.17	0.00	0.00	0.00	0.11	0.00	0.00
RDH14	0.00	0.00	0.00	0.17	0.00	0.24	0.00	0.14	0.00	0.00	0.00	0.12	0.00	0.00	0.12	0.00	0.00	0.00	0.31	0.00	0.00
IMPAD1	0.00	0.00	0.00	0.16	0.00	0.31	0.00	0.13	0.00	0.00	0.00	0.14	0.00	0.00	0.15	0.00	0.00	0.00	0.14	0.00	0.00
LDB3	0.00	0.00	0.00	0.21	0.00	0.34	0.00	0.13	0.00	0.00	0.00	0.15	0.00	0.00	0.18	0.00	0.00	0.00	0.11	0.00	0.00
ZCRB1	0.00	0.00	0.00	0.33	0.00	0.64	0.00	0.20	0.00	0.00	0.00	0.28	0.00	0.00	0.20	0.00	0.00	0.00	0.14	0.00	0.00
RBM28	0.00	0.00	0.00	0.20	0.00	0.35	0.00	0.14	0.00	0.00	0.00	0.11	0.00	0.00	0.16	0.00	0.00	0.00	0.13	0.00	0.00
SRSF11	0.00	0.00	0.00	0.24	0.00	0.43	0.00	0.20	0.00	0.00	0.00	0.11	0.00	0.00	0.13	0.00	0.00	0.00	0.15	0.00	0.00
ALKBH5	0.00	0.00	0.00	0.20	0.00	0.45	0.00	0.22	0.00	0.00	0.00	0.17	0.00	0.00	0.19	0.00	0.00	0.00	0.15	0.00	0.00
FMO3	0.00	0.00	0.00	0.12	0.00	0.17	0.00	0.00	0.14	0.00	0.00	0.14	0.00	0.00	0.16	0.00	0.00	0.00	0.30	0.00	0.00
GSTO1	0.00	0.00	0.00	0.11	0.00	0.14	0.00	0.00	0.11	0.00	0.00	0.14	0.00	0.00	0.13	0.00	0.00	0.00	0.25	0.00	0.00
UROS	0.00	0.00	0.00	0.12	0.00	0.21	0.00	0.00	0.12	0.00	0.00	0.12	0.00	0.00	0.13	0.00	0.00	0.00	0.25	0.00	0.00
NAGA	0.00	0.00	0.00	0.17	0.00	0.28	0.00	0.00	0.19	0.00	0.00	0.17	0.00	0.00	0.17	0.00	0.00	0.00	0.16	0.00	0.00
GALT	0.00	0.00	0.00	0.11	0.00	0.15	0.00	0.00	0.14	0.00	0.00	0.12	0.00	0.00	0.14	0.00	0.00	0.00	0.23	0.00	0.00
GNS	0.00	0.00	0.00	0.12	0.00	0.29	0.00	0.00	0.14	0.00	0.00	0.17	0.00	0.00	0.16	0.00	0.00	0.00	0.18	0.00	0.00
GLA	0.00	0.00	0.00	0.12	0.00	0.11	0.00	0.00	0.27	0.00	0.00	0.12	0.00	0.00	0.15	0.00	0.00	0.00	0.14	0.00	0.00
BTD	0.00	0.00	0.00	0.13	0.00	0.26	0.00	0.00	0.15	0.00	0.00	0.17	0.00	0.00	0.21	0.00	0.00	0.00	0.22	0.00	0.00
IDS	0.00	0.00	0.00	0.13	0.00	0.20	0.00	0.00	0.16	0.00	0.00	0.13	0.00	0.00	0.15	0.00	0.00	0.00	0.12	0.00	0.00
ELOVL4	0.00	0.00	0.00	0.16	0.00	0.24	0.00	0.00	0.11	0.00	0.00	0.11	0.00	0.00	0.18	0.00	0.00	0.00	0.14	0.00	0.00

CTBS	0.00	0.00	0.00	0.26	0.00	0.44	0.00	0.00	0.00	0.00	0.12	0.22	0.00	0.00	0.15	0.00	0.00	0.00	0.15	0.00	0.00
MPDU1	0.00	0.00	0.00	0.25	0.00	0.49	0.00	0.00	0.00	0.00	0.18	0.18	0.00	0.00	0.19	0.00	0.00	0.00	0.13	0.00	0.00
ABCA8	0.00	0.00	0.00	0.12	0.00	0.31	0.00	0.00	0.00	0.00	0.14	0.20	0.00	0.00	0.12	0.00	0.00	0.00	0.18	0.00	0.00
ABCA9	0.00	0.00	0.00	0.28	0.00	0.52	0.00	0.00	0.00	0.00	0.22	0.25	0.00	0.00	0.19	0.00	0.00	0.00	0.19	0.00	0.00
ST6GALNAC5	0.00	0.00	0.00	0.21	0.00	0.45	0.00	0.00	0.00	0.00	0.13	0.29	0.00	0.00	0.17	0.00	0.00	0.00	0.17	0.00	0.00
CSGALNACT1	0.00	0.00	0.00	0.16	0.00	0.34	0.00	0.00	0.00	0.00	0.10	0.19	0.00	0.00	0.20	0.00	0.00	0.00	0.13	0.00	0.00
ALG1	0.00	0.00	0.00	0.15	0.00	0.35	0.00	0.00	0.00	0.00	0.14	0.15	0.00	0.00	0.16	0.00	0.00	0.00	0.13	0.00	0.00
ALPL	0.00	0.00	0.00	0.11	0.00	0.00	0.00	0.11	0.12	0.00	0.00	0.00	0.10	0.00	0.11	0.00	0.00	0.00	0.20	0.00	0.00
SULT6B1	0.00	0.00	0.00	0.24	0.00	0.42	0.00	0.00	0.00	0.00	0.00	0.29	0.14	0.00	0.21	0.00	0.00	0.00	0.16	0.00	0.00
PCDHA12	0.00	0.00	0.00	0.00	0.00	0.00	0.00	0.11	0.16	0.00	0.00	0.15	0.14	0.00	0.13	0.00	0.00	0.00	0.16	0.00	0.00
DICER1	0.00	0.00	0.00	0.00	0.00	0.00	0.00	0.36	0.25	0.00	0.00	0.00	0.12	0.00	0.19	0.15	0.00	0.00	0.12	0.00	0.00
HEXA	0.00	0.00	0.00	0.00	0.00	0.00	0.00	0.00	0.18	0.00	0.00	0.15	0.11	0.00	0.20	0.17	0.00	0.00	0.13	0.00	0.00
GBA	0.00	0.00	0.00	0.00	0.00	0.00	0.00	0.00	0.21	0.00	0.00	0.16	0.14	0.00	0.22	0.19	0.00	0.00	0.14	0.00	0.00
ATP8B4	0.00	0.00	0.00	0.10	0.00	0.21	0.00	0.00	0.00	0.00	0.00	0.16	0.00	0.11	0.00	0.00	0.14	0.00	0.12	0.00	0.00
PON3	0.00	0.00	0.00	0.13	0.00	0.15	0.00	0.00	0.13	0.00	0.00	0.00	0.00	0.00	0.10	0.00	0.14	0.00	0.15	0.00	0.00
GOUT1	0.00	0.00	0.00	0.16	0.00	0.23	0.00	0.00	0.00	0.00	0.00	0.11	0.00	0.00	0.11	0.00	0.17	0.00	0.12	0.00	0.00
BHMT	0.00	0.00	0.00	0.13	0.00	0.15	0.00	0.12	0.00	0.00	0.00	0.00	0.00	0.00	0.11	0.00	0.00	0.14	0.34	0.00	0.00
SERPINA7	0.00	0.00	0.00	0.16	0.00	0.18	0.00	0.11	0.00	0.00	0.00	0.00	0.00	0.00	0.13	0.00	0.00	0.12	0.20	0.00	0.00
THRB	0.00	0.00	0.00	0.13	0.00	0.00	0.11	0.19	0.00	0.00	0.00	0.00	0.00	0.00	0.13	0.00	0.00	0.11	0.13	0.00	0.00
PHKG1	0.00	0.00	0.00	0.19	0.00	0.30	0.00	0.00	0.00	0.00	0.00	0.22	0.00	0.00	0.12	0.00	0.00	0.10	0.14	0.00	0.00
APOC2	0.00	0.00	0.00	0.15	0.00	0.14	0.00	0.11	0.00	0.00	0.00	0.00	0.00	0.00	0.00	0.00	0.12	0.10	0.23	0.00	0.00
EXT2	0.00	0.00	0.00	0.11	0.00	0.19	0.00	0.00	0.10	0.00	0.00	0.00	0.00	0.00	0.12	0.00	0.23	0.00	0.00	0.13	0.00
HNF1B	0.00	0.00	0.00	0.00	0.00	0.11	0.00	0.15	0.12	0.00	0.00	0.00	0.00	0.00	0.10	0.00	0.24	0.00	0.00	0.18	0.00
STQTL5	0.00	0.00	0.00	0.15	0.00	0.25	0.00	0.00	0.00	0.00	0.00	0.11	0.00	0.00	0.12	0.00	0.43	0.00	0.00	0.23	0.00
PLEKHG4	0.00	0.00	0.00	0.12	0.00	0.24	0.00	0.00	0.00	0.00	0.00	0.12	0.00	0.00	0.17	0.00	0.14	0.00	0.00	0.14	0.00
MTMR8	0.00	0.00	0.00	0.23	0.00	0.36	0.10	0.00	0.00	0.00	0.17	0.00	0.00	0.00	0.00	0.00	0.00	0.00	0.19	0.13	0.00
PHKA2	0.00	0.00	0.00	0.18	0.00	0.31	0.00	0.00	0.00	0.00	0.00	0.21	0.00	0.00	0.14	0.00	0.00	0.00	0.17	0.11	0.00
GALK1	0.00	0.00	0.00	0.16	0.00	0.27	0.00	0.00	0.00	0.00	0.00	0.14	0.00	0.00	0.15	0.00	0.00	0.00	0.21	0.10	0.00
CNSN	0.00	0.00	0.00	0.00	0.00	0.17	0.00	0.00	0.16	0.00	0.00	0.14	0.00	0.00	0.00	0.00	0.29	0.00	0.18	0.17	0.00
LACTB2	0.00	0.00	0.00	0.11	0.00	0.20	0.00	0.00	0.00	0.00	0.00	0.12	0.00	0.00	0.00	0.00	0.00	0.12	0.27	0.12	0.00
BP3	0.00	0.00	0.00	0.17	0.00	0.22	0.00	0.00	0.00	0.00	0.00	0.00	0.00	0.00	0.00	0.00	0.27	0.11	0.23	0.21	0.00
BP2	0.00	0.00	0.00	0.17	0.00	0.22	0.00	0.00	0.00	0.00	0.00	0.00	0.00	0.00	0.00	0.00	0.27	0.11	0.23	0.21	0.00
BP4	0.00	0.00	0.00	0.17	0.00	0.22	0.00	0.00	0.00	0.00	0.00	0.00	0.00	0.00	0.00	0.00	0.27	0.11	0.23	0.21	0.00
INTS10	0.00	0.00	0.00	0.12	0.00	0.17	0.00	0.00	0.00	0.00	0.00	0.00	0.00	0.00	0.00	0.00	0.23	0.20	0.30	0.21	0.00
NIDDM2	0.00	0.00	0.00	0.12	0.00	0.18	0.00	0.00	0.00	0.00	0.00	0.00	0.00	0.00	0.00	0.00	0.61	0.19	0.20	0.54	0.00
KCNJ11	0.00	0.00	0.00	0.11	0.00	0.00	0.00	0.00	0.15	0.00	0.00	0.00	0.00	0.00	0.00	0.00	0.18	0.13	0.13	0.16	0.00
SNX16	0.00	0.00	0.20	0.32	0.00	0.31	0.23	0.16	0.00	0.00	0.00	0.00	0.00	0.00	0.00	0.00	0.00	0.00	0.00	0.00	0.16
FAM176A	0.00	0.00	0.00	0.31	0.00	0.49	0.15	0.32	0.00	0.00	0.17	0.00	0.00	0.00	0.00	0.00	0.00	0.00	0.00	0.00	0.32
CASP7	0.00	0.00	0.00	0.14	0.00	0.12	0.00	0.17	0.17	0.00	0.00	0.00	0.00	0.00	0.15	0.00	0.00	0.00	0.00	0.00	0.15
TTLL1	0.00	0.00	0.00	0.22	0.00	0.35	0.10	0.00	0.00	0.00	0.00	0.20	0.00	0.00	0.18	0.00	0.00	0.00	0.00	0.00	0.12

BICD2	0.00	0.00	0.00	0.23	0.00	0.42	0.00	0.22	0.00	0.00	0.00	0.21	0.00	0.00	0.21	0.00	0.00	0.00	0.00	0.00	0.10
TEFM	0.00	0.00	0.00	0.15	0.00	0.39	0.00	0.11	0.00	0.00	0.00	0.11	0.00	0.00	0.13	0.00	0.00	0.00	0.00	0.00	0.27
VAT1	0.00	0.00	0.00	0.26	0.00	0.46	0.00	0.22	0.00	0.00	0.00	0.19	0.00	0.00	0.13	0.00	0.00	0.00	0.00	0.00	0.17
DCTN4	0.00	0.00	0.00	0.20	0.00	0.36	0.00	0.11	0.00	0.00	0.00	0.20	0.00	0.00	0.19	0.00	0.00	0.00	0.00	0.00	0.11
UTP6	0.00	0.00	0.00	0.26	0.00	0.49	0.00	0.22	0.00	0.00	0.00	0.19	0.00	0.00	0.21	0.00	0.00	0.00	0.00	0.00	0.23
SPEF1	0.00	0.00	0.00	0.30	0.00	0.60	0.00	0.12	0.00	0.00	0.00	0.26	0.00	0.00	0.22	0.00	0.00	0.00	0.00	0.00	0.12
PACRG	0.00	0.00	0.00	0.17	0.00	0.27	0.00	0.13	0.00	0.00	0.00	0.18	0.00	0.00	0.21	0.00	0.00	0.00	0.00	0.00	0.13
DNAL1	0.00	0.00	0.00	0.17	0.00	0.36	0.00	0.16	0.00	0.00	0.00	0.11	0.00	0.00	0.14	0.00	0.00	0.00	0.00	0.00	0.13
DNAH1	0.00	0.00	0.00	0.21	0.00	0.35	0.00	0.16	0.00	0.00	0.00	0.20	0.00	0.00	0.23	0.00	0.00	0.00	0.00	0.00	0.12
TBCD	0.00	0.00	0.00	0.25	0.00	0.43	0.00	0.20	0.00	0.00	0.00	0.19	0.00	0.00	0.23	0.00	0.00	0.00	0.00	0.00	0.16
CASP9	0.00	0.00	0.00	0.00	0.00	0.00	0.00	0.13	0.15	0.00	0.00	0.00	0.00	0.11	0.14	0.00	0.10	0.00	0.00	0.00	0.17
CMC2	0.00	0.00	0.00	0.15	0.00	0.29	0.00	0.13	0.00	0.00	0.00	0.11	0.00	0.00	0.00	0.00	0.00	0.00	0.20	0.00	0.41
SAMD8	0.00	0.00	0.00	0.16	0.00	0.39	0.00	0.00	0.00	0.00	0.17	0.18	0.00	0.00	0.00	0.00	0.00	0.00	0.29	0.00	0.11
TRNAG1	0.00	0.00	0.00	0.21	0.00	0.44	0.00	0.22	0.00	0.00	0.00	0.00	0.00	0.00	0.14	0.00	0.00	0.00	0.11	0.00	0.24
DLSTP1	0.00	0.00	0.00	0.24	0.00	0.52	0.00	0.00	0.00	0.00	0.00	0.27	0.00	0.00	0.14	0.00	0.00	0.00	0.17	0.00	0.17
UOX	0.00	0.00	0.00	0.23	0.00	0.35	0.00	0.00	0.00	0.00	0.00	0.16	0.00	0.00	0.17	0.00	0.00	0.00	0.23	0.00	0.13
PRPS1L1	0.00	0.00	0.00	0.16	0.00	0.36	0.00	0.00	0.00	0.00	0.00	0.25	0.00	0.00	0.19	0.00	0.00	0.00	0.29	0.00	0.11
ILVBL	0.00	0.00	0.00	0.22	0.00	0.57	0.00	0.00	0.00	0.00	0.00	0.27	0.00	0.00	0.18	0.00	0.00	0.00	0.16	0.00	0.12
PPOX	0.00	0.00	0.00	0.14	0.00	0.23	0.00	0.00	0.00	0.00	0.00	0.14	0.00	0.00	0.14	0.00	0.00	0.00	0.27	0.00	0.22
MRPL19	0.00	0.00	0.00	0.22	0.00	0.40	0.00	0.00	0.00	0.00	0.00	0.21	0.00	0.00	0.14	0.00	0.00	0.00	0.12	0.00	0.22
ATG12P1	0.00	0.00	0.00	0.13	0.00	0.20	0.00	0.23	0.00	0.00	0.00	0.00	0.00	0.00	0.00	0.00	0.00	0.17	0.20	0.00	0.35
MIRLET7BHG	0.00	0.00	0.00	0.14	0.00	0.22	0.00	0.26	0.00	0.00	0.00	0.00	0.00	0.00	0.00	0.00	0.00	0.17	0.15	0.00	0.49
SMCR7L	0.00	0.00	0.00	0.15	0.00	0.31	0.00	0.16	0.00	0.00	0.00	0.00	0.00	0.00	0.00	0.00	0.00	0.25	0.19	0.00	0.72
TRNM	0.00	0.00	0.00	0.14	0.00	0.35	0.00	0.18	0.00	0.00	0.00	0.00	0.00	0.00	0.00	0.00	0.00	0.17	0.16	0.00	0.44
VARS2	0.00	0.00	0.00	0.00	0.00	0.20	0.00	0.19	0.21	0.00	0.00	0.00	0.00	0.00	0.00	0.00	0.00	0.13	0.15	0.00	0.28
SQRDL	0.00	0.00	0.00	0.24	0.00	0.53	0.00	0.00	0.00	0.00	0.00	0.24	0.00	0.00	0.00	0.00	0.00	0.19	0.38	0.00	0.54
GATC	0.00	0.00	0.00	0.14	0.00	0.43	0.00	0.00	0.00	0.00	0.00	0.12	0.00	0.00	0.00	0.00	0.00	0.22	0.21	0.00	0.65
UQCRFS1P1	0.00	0.00	0.00	0.16	0.00	0.45	0.00	0.00	0.00	0.00	0.00	0.14	0.00	0.00	0.00	0.00	0.00	0.19	0.20	0.00	0.70
QRSL1	0.00	0.00	0.00	0.14	0.00	0.43	0.00	0.00	0.00	0.00	0.00	0.12	0.00	0.00	0.00	0.00	0.00	0.22	0.21	0.00	0.65
EARS2	0.00	0.00	0.00	0.14	0.00	0.43	0.00	0.00	0.00	0.00	0.00	0.12	0.00	0.00	0.00	0.00	0.00	0.22	0.21	0.00	0.65
MRPS22	0.00	0.00	0.00	0.12	0.00	0.38	0.00	0.00	0.00	0.00	0.00	0.12	0.00	0.00	0.00	0.00	0.00	0.15	0.14	0.00	0.34
CMC1	0.00	0.00	0.00	0.18	0.00	0.40	0.00	0.00	0.00	0.00	0.00	0.16	0.00	0.00	0.00	0.00	0.00	0.16	0.25	0.00	0.60
NDUFV2P1	0.00	0.00	0.00	0.15	0.00	0.42	0.00	0.00	0.00	0.00	0.00	0.11	0.00	0.00	0.00	0.00	0.00	0.13	0.15	0.00	0.44
COX14	0.00	0.00	0.00	0.12	0.00	0.38	0.00	0.00	0.00	0.00	0.00	0.12	0.00	0.00	0.00	0.00	0.00	0.15	0.24	0.00	0.51
COX19	0.00	0.00	0.00	0.18	0.00	0.49	0.00	0.00	0.00	0.00	0.00	0.13	0.00	0.00	0.00	0.00	0.00	0.15	0.21	0.00	0.55
COX17P1	0.00	0.00	0.00	0.15	0.00	0.42	0.00	0.00	0.00	0.00	0.00	0.14	0.00	0.00	0.00	0.00	0.00	0.16	0.22	0.00	0.47
C20ORF7	0.00	0.00	0.00	0.00	0.00	0.30	0.00	0.00	0.00	0.00	0.00	0.10	0.00	0.00	0.12	0.00	0.00	0.14	0.16	0.00	0.38
TACO1	0.00	0.00	0.00	0.00	0.00	0.29	0.00	0.00	0.00	0.00	0.00	0.15	0.00	0.00	0.12	0.00	0.00	0.15	0.19	0.00	0.52
NDUFB4	0.00	0.00	0.00	0.00	0.00	0.26	0.00	0.00	0.00	0.00	0.00	0.22	0.00	0.00	0.13	0.00	0.00	0.21	0.23	0.00	0.57
TRNAL1	0.00	0.00	0.00	0.18	0.00	0.36	0.00	0.00	0.00	0.00	0.00	0.00	0.00	0.00	0.00	0.00	0.12	0.17	0.12	0.00	0.46

TRNL1	0.00	0.00	0.00	0.00	0.00	0.12	0.00	0.00	0.00	0.00	0.00	0.00	0.00	0.00	0.00	0.00	0.32	0.20	0.14	0.22	0.29
TRNQ	0.00	0.00	0.00	0.00	0.00	0.17	0.00	0.00	0.00	0.00	0.00	0.00	0.00	0.00	0.00	0.00	0.35	0.13	0.15	0.20	0.33
TRNE	0.00	0.00	0.00	0.00	0.00	0.23	0.00	0.00	0.00	0.00	0.00	0.00	0.00	0.00	0.00	0.00	0.41	0.20	0.14	0.29	0.33
TRNAL2	0.00	0.00	0.00	0.00	0.00	0.33	0.00	0.00	0.00	0.00	0.00	0.00	0.00	0.00	0.00	0.00	0.19	0.17	0.11	0.12	0.35
TRNL2	0.00	0.00	0.00	0.00	0.00	0.14	0.00	0.00	0.00	0.00	0.00	0.00	0.00	0.00	0.00	0.00	0.26	0.13	0.16	0.14	0.32
TRNI	0.00	0.00	0.00	0.00	0.00	0.24	0.00	0.00	0.00	0.00	0.00	0.00	0.00	0.00	0.00	0.00	0.24	0.25	0.22	0.18	0.44
TRNT	0.00	0.00	0.00	0.00	0.00	0.12	0.00	0.00	0.00	0.00	0.00	0.00	0.00	0.00	0.00	0.00	0.29	0.18	0.11	0.15	0.42
FICD	0.00	0.00	0.15	0.32	0.00	0.44	0.17	0.22	0.00	0.00	0.00	0.00	0.00	0.00	0.00	0.00	0.00	0.00	0.00	0.00	0.00
CXCR4	0.12	0.12	0.11	0.00	0.00	0.00	0.00	0.11	0.13	0.00	0.00	0.00	0.00	0.00	0.00	0.00	0.00	0.00	0.00	0.00	0.00
MIR369	0.00	0.00	0.00	0.18	0.00	0.21	0.11	0.30	0.14	0.00	0.00	0.00	0.00	0.00	0.00	0.00	0.00	0.00	0.00	0.00	0.00
MIR30E	0.00	0.00	0.00	0.20	0.00	0.28	0.11	0.45	0.33	0.00	0.00	0.00	0.00	0.00	0.00	0.00	0.00	0.00	0.00	0.00	0.00
MNG1	0.00	0.00	0.11	0.15	0.00	0.15	0.11	0.00	0.00	0.22	0.00	0.00	0.00	0.00	0.00	0.00	0.00	0.00	0.00	0.00	0.00
AP5B1	0.00	0.00	0.16	0.33	0.00	0.37	0.22	0.00	0.00	0.00	0.18	0.00	0.00	0.00	0.00	0.00	0.00	0.00	0.00	0.00	0.00
AP5S1	0.00	0.00	0.16	0.33	0.00	0.37	0.22	0.00	0.00	0.00	0.18	0.00	0.00	0.00	0.00	0.00	0.00	0.00	0.00	0.00	0.00
COQ6	0.00	0.00	0.00	0.11	0.00	0.25	0.00	0.15	0.11	0.00	0.10	0.00	0.00	0.00	0.00	0.00	0.00	0.00	0.00	0.00	0.00
DNAJB12	0.00	0.00	0.00	0.17	0.00	0.25	0.00	0.14	0.11	0.00	0.24	0.00	0.00	0.00	0.00	0.00	0.00	0.00	0.00	0.00	0.00
MRX18	0.00	0.00	0.13	0.21	0.00	0.20	0.15	0.00	0.00	0.00	0.00	0.14	0.00	0.00	0.00	0.00	0.00	0.00	0.00	0.00	0.00
MRX17	0.00	0.00	0.13	0.21	0.00	0.20	0.15	0.00	0.00	0.00	0.00	0.14	0.00	0.00	0.00	0.00	0.00	0.00	0.00	0.00	0.00
RAB9B	0.00	0.00	0.00	0.28	0.00	0.51	0.10	0.31	0.00	0.00	0.00	0.16	0.00	0.00	0.00	0.00	0.00	0.00	0.00	0.00	0.00
YWHAQP4	0.00	0.00	0.00	0.24	0.00	0.35	0.12	0.26	0.00	0.00	0.00	0.14	0.00	0.00	0.00	0.00	0.00	0.00	0.00	0.00	0.00
MIR133A2	0.00	0.00	0.00	0.19	0.00	0.32	0.00	0.39	0.19	0.00	0.00	0.10	0.00	0.00	0.00	0.00	0.00	0.00	0.00	0.00	0.00
C11ORF95	0.00	0.00	0.00	0.18	0.00	0.37	0.00	0.19	0.10	0.00	0.00	0.12	0.00	0.00	0.00	0.00	0.00	0.00	0.00	0.00	0.00
FRA6E	0.00	0.00	0.00	0.13	0.00	0.28	0.00	0.16	0.11	0.00	0.00	0.11	0.00	0.00	0.00	0.00	0.00	0.00	0.00	0.00	0.00
C15ORF63	0.00	0.00	0.00	0.25	0.00	0.44	0.00	0.28	0.11	0.00	0.00	0.13	0.00	0.00	0.00	0.00	0.00	0.00	0.00	0.00	0.00
LRRC26	0.00	0.00	0.00	0.28	0.00	0.48	0.00	0.21	0.00	0.00	0.18	0.18	0.00	0.00	0.00	0.00	0.00	0.00	0.00	0.00	0.00
SLC26A4	0.00	0.00	0.00	0.10	0.00	0.00	0.00	0.11	0.10	0.00	0.16	0.00	0.11	0.00	0.00	0.00	0.00	0.00	0.00	0.00	0.00
DDU	0.00	0.00	0.12	0.16	0.00	0.13	0.12	0.00	0.00	0.00	0.00	0.00	0.00	0.00	0.13	0.00	0.00	0.00	0.00	0.00	0.00
HSCRM2	0.00	0.00	0.13	0.20	0.00	0.24	0.14	0.00	0.00	0.00	0.00	0.00	0.00	0.00	0.12	0.00	0.00	0.00	0.00	0.00	0.00
ECB2	0.00	0.00	0.16	0.23	0.00	0.18	0.19	0.00	0.00	0.00	0.00	0.00	0.00	0.00	0.11	0.00	0.00	0.00	0.00	0.00	0.00
HSCRM1	0.00	0.00	0.10	0.18	0.00	0.23	0.11	0.00	0.00	0.00	0.00	0.00	0.00	0.00	0.13	0.00	0.00	0.00	0.00	0.00	0.00
NOP56	0.00	0.00	0.00	0.26	0.00	0.44	0.11	0.24	0.00	0.00	0.00	0.00	0.00	0.00	0.17	0.00	0.00	0.00	0.00	0.00	0.00
BFSP2	0.00	0.00	0.00	0.22	0.00	0.31	0.12	0.12	0.00	0.00	0.00	0.00	0.00	0.00	0.18	0.00	0.00	0.00	0.00	0.00	0.00
TBX6	0.00	0.00	0.00	0.21	0.00	0.21	0.14	0.25	0.00	0.00	0.00	0.00	0.00	0.00	0.22	0.00	0.00	0.00	0.00	0.00	0.00
IRF6	0.00	0.00	0.00	0.15	0.00	0.17	0.10	0.17	0.00	0.00	0.00	0.00	0.00	0.00	0.14	0.00	0.00	0.00	0.00	0.00	0.00
KRT12	0.00	0.00	0.00	0.20	0.00	0.24	0.12	0.15	0.00	0.00	0.00	0.00	0.00	0.00	0.17	0.00	0.00	0.00	0.00	0.00	0.00
EIF1AY	0.00	0.00	0.00	0.24	0.00	0.36	0.11	0.16	0.00	0.00	0.00	0.00	0.00	0.00	0.17	0.00	0.00	0.00	0.00	0.00	0.00
TPR	0.00	0.00	0.00	0.29	0.00	0.41	0.14	0.22	0.00	0.00	0.00	0.00	0.00	0.00	0.16	0.00	0.00	0.00	0.00	0.00	0.00
LEFTY1	0.00	0.00	0.00	0.16	0.00	0.12	0.11	0.17	0.00	0.00	0.00	0.00	0.00	0.00	0.14	0.00	0.00	0.00	0.00	0.00	0.00
HPFH	0.00	0.00	0.00	0.22	0.00	0.29	0.12	0.15	0.00	0.00	0.00	0.00	0.00	0.00	0.11	0.00	0.00	0.00	0.00	0.00	0.00
FBXW10	0.00	0.00	0.00	0.24	0.00	0.35	0.12	0.22	0.00	0.00	0.00	0.00	0.00	0.00	0.12	0.00	0.00	0.00	0.00	0.00	0.00

RP9	0.00	0.00	0.00	0.33	0.00	0.54	0.14	0.23	0.00	0.00	0.00	0.00	0.00	0.00	0.13	0.00	0.00	0.00	0.00	0.00	0.00
PAK1	0.00	0.00	0.11	0.13	0.00	0.00	0.00	0.14	0.14	0.00	0.00	0.00	0.00	0.00	0.12	0.00	0.00	0.00	0.00	0.00	0.00
LOC285299	0.00	0.00	0.00	0.21	0.00	0.38	0.00	0.20	0.13	0.00	0.00	0.00	0.00	0.00	0.11	0.00	0.00	0.00	0.00	0.00	0.00
MSLNL	0.00	0.00	0.00	0.17	0.00	0.29	0.00	0.16	0.24	0.00	0.00	0.00	0.00	0.00	0.11	0.00	0.00	0.00	0.00	0.00	0.00
AIPL1	0.00	0.00	0.00	0.17	0.00	0.23	0.00	0.10	0.14	0.00	0.00	0.00	0.00	0.00	0.15	0.00	0.00	0.00	0.00	0.00	0.00
KLHL7	0.00	0.00	0.00	0.17	0.00	0.31	0.00	0.13	0.14	0.00	0.00	0.00	0.00	0.00	0.18	0.00	0.00	0.00	0.00	0.00	0.00
ZEB2	0.00	0.00	0.00	0.16	0.00	0.18	0.00	0.38	0.25	0.00	0.00	0.00	0.00	0.00	0.16	0.00	0.00	0.00	0.00	0.00	0.00
SHOX	0.00	0.00	0.00	0.14	0.00	0.19	0.00	0.17	0.13	0.00	0.00	0.00	0.00	0.00	0.15	0.00	0.00	0.00	0.00	0.00	0.00
KRT81	0.00	0.00	0.00	0.23	0.00	0.38	0.00	0.26	0.13	0.00	0.00	0.00	0.00	0.00	0.15	0.00	0.00	0.00	0.00	0.00	0.00
PRPF8	0.00	0.00	0.00	0.20	0.00	0.38	0.00	0.15	0.10	0.00	0.00	0.00	0.00	0.00	0.15	0.00	0.00	0.00	0.00	0.00	0.00
FLCN	0.00	0.00	0.00	0.15	0.00	0.23	0.00	0.22	0.17	0.00	0.00	0.00	0.00	0.00	0.13	0.00	0.00	0.00	0.00	0.00	0.00
BAP1	0.00	0.00	0.00	0.17	0.00	0.33	0.00	0.39	0.15	0.00	0.00	0.00	0.00	0.00	0.14	0.00	0.00	0.00	0.00	0.00	0.00
GEMIN2	0.00	0.00	0.00	0.17	0.00	0.30	0.00	0.24	0.20	0.00	0.00	0.00	0.00	0.00	0.21	0.00	0.00	0.00	0.00	0.00	0.00
MAP2K2	0.00	0.00	0.00	0.15	0.00	0.13	0.00	0.17	0.19	0.00	0.00	0.00	0.00	0.00	0.12	0.00	0.00	0.00	0.00	0.00	0.00
SERPINC1	0.00	0.00	0.00	0.13	0.00	0.13	0.00	0.10	0.14	0.00	0.00	0.00	0.00	0.00	0.16	0.00	0.00	0.00	0.00	0.00	0.00
GEMIN5	0.00	0.00	0.00	0.24	0.00	0.43	0.00	0.25	0.12	0.00	0.00	0.00	0.00	0.00	0.18	0.00	0.00	0.00	0.00	0.00	0.00
SNRPD3	0.00	0.00	0.00	0.16	0.00	0.31	0.00	0.13	0.10	0.00	0.00	0.00	0.00	0.00	0.14	0.00	0.00	0.00	0.00	0.00	0.00
ANXA2	0.00	0.00	0.00	0.15	0.00	0.12	0.00	0.17	0.17	0.00	0.00	0.00	0.00	0.00	0.12	0.00	0.00	0.00	0.00	0.00	0.00
CCNT1	0.00	0.00	0.00	0.13	0.00	0.12	0.00	0.31	0.17	0.00	0.00	0.00	0.00	0.00	0.13	0.00	0.00	0.00	0.00	0.00	0.00
TNRC6A	0.00	0.00	0.00	0.21	0.00	0.40	0.00	0.44	0.15	0.00	0.00	0.00	0.00	0.00	0.15	0.00	0.00	0.00	0.00	0.00	0.00
FOXL2	0.00	0.00	0.00	0.16	0.00	0.17	0.00	0.24	0.12	0.00	0.00	0.00	0.00	0.00	0.14	0.00	0.00	0.00	0.00	0.00	0.00
PRPF3	0.00	0.00	0.00	0.22	0.00	0.39	0.00	0.14	0.11	0.00	0.00	0.00	0.00	0.00	0.16	0.00	0.00	0.00	0.00	0.00	0.00
KLK7	0.00	0.00	0.00	0.16	0.00	0.19	0.00	0.19	0.23	0.00	0.00	0.00	0.00	0.00	0.10	0.00	0.00	0.00	0.00	0.00	0.00
PIN1	0.00	0.00	0.00	0.14	0.00	0.12	0.00	0.28	0.27	0.00	0.00	0.00	0.00	0.00	0.11	0.00	0.00	0.00	0.00	0.00	0.00
COMP	0.00	0.00	0.00	0.16	0.00	0.18	0.00	0.12	0.16	0.00	0.00	0.00	0.00	0.00	0.13	0.00	0.00	0.00	0.00	0.00	0.00
HSPA4	0.00	0.00	0.00	0.13	0.00	0.11	0.00	0.13	0.15	0.00	0.00	0.00	0.00	0.00	0.12	0.00	0.00	0.00	0.00	0.00	0.00
IQCB1	0.00	0.00	0.00	0.17	0.00	0.29	0.00	0.13	0.13	0.00	0.00	0.00	0.00	0.00	0.15	0.00	0.00	0.00	0.00	0.00	0.00
FSHMD1A	0.00	0.00	0.00	0.17	0.00	0.30	0.00	0.17	0.17	0.00	0.00	0.00	0.00	0.00	0.13	0.00	0.00	0.00	0.00	0.00	0.00
EWSR1	0.00	0.00	0.00	0.17	0.00	0.20	0.00	0.33	0.25	0.00	0.00	0.00	0.00	0.00	0.17	0.00	0.00	0.00	0.00	0.00	0.00
CDAN1	0.00	0.00	0.00	0.19	0.00	0.33	0.00	0.20	0.11	0.00	0.00	0.00	0.00	0.00	0.13	0.00	0.00	0.00	0.00	0.00	0.00
ERVK-16	0.00	0.00	0.00	0.12	0.00	0.24	0.00	0.14	0.18	0.00	0.00	0.00	0.00	0.00	0.11	0.00	0.00	0.00	0.00	0.00	0.00
FLG	0.00	0.00	0.00	0.11	0.00	0.11	0.00	0.12	0.11	0.00	0.00	0.00	0.00	0.00	0.12	0.00	0.00	0.00	0.00	0.00	0.00
ERVK-12	0.00	0.00	0.00	0.12	0.00	0.24	0.00	0.14	0.18	0.00	0.00	0.00	0.00	0.00	0.11	0.00	0.00	0.00	0.00	0.00	0.00
MEN1	0.00	0.00	0.00	0.11	0.00	0.12	0.00	0.22	0.17	0.00	0.00	0.00	0.00	0.00	0.13	0.00	0.00	0.00	0.00	0.00	0.00
SPINK5	0.00	0.00	0.00	0.15	0.00	0.18	0.00	0.11	0.14	0.00	0.00	0.00	0.00	0.00	0.12	0.00	0.00	0.00	0.00	0.00	0.00
PALB2	0.00	0.00	0.00	0.11	0.00	0.16	0.00	0.13	0.11	0.00	0.00	0.00	0.00	0.00	0.10	0.00	0.00	0.00	0.00	0.00	0.00
FOXC1	0.00	0.00	0.00	0.13	0.00	0.17	0.00	0.22	0.11	0.00	0.00	0.00	0.00	0.00	0.16	0.00	0.00	0.00	0.00	0.00	0.00
FSCN2	0.00	0.00	0.00	0.13	0.00	0.24	0.00	0.12	0.16	0.00	0.00	0.00	0.00	0.00	0.11	0.00	0.00	0.00	0.00	0.00	0.00
HSF1	0.00	0.00	0.00	0.13	0.00	0.12	0.00	0.21	0.23	0.00	0.00	0.00	0.00	0.00	0.15	0.00	0.00	0.00	0.00	0.00	0.00
CDC73	0.00	0.00	0.00	0.15	0.00	0.25	0.00	0.28	0.18	0.00	0.00	0.00	0.00	0.00	0.10	0.00	0.00	0.00	0.00	0.00	0.00

KRT75	0.00	0.00	0.00	0.18	0.00	0.24	0.00	0.25	0.19	0.00	0.00	0.00	0.00	0.00	0.17	0.00	0.00	0.00	0.00	0.00	0.00
ERVK-2	0.00	0.00	0.00	0.12	0.00	0.24	0.00	0.14	0.18	0.00	0.00	0.00	0.00	0.00	0.11	0.00	0.00	0.00	0.00	0.00	0.00
FRG2	0.00	0.00	0.00	0.20	0.00	0.35	0.00	0.18	0.15	0.00	0.00	0.00	0.00	0.00	0.13	0.00	0.00	0.00	0.00	0.00	0.00
PDE6B	0.00	0.00	0.00	0.14	0.00	0.00	0.12	0.11	0.18	0.00	0.00	0.00	0.00	0.00	0.13	0.00	0.00	0.00	0.00	0.00	0.00
ENG	0.00	0.00	0.00	0.16	0.00	0.00	0.13	0.12	0.20	0.00	0.00	0.00	0.00	0.00	0.13	0.00	0.00	0.00	0.00	0.00	0.00
NFKB2	0.00	0.00	0.00	0.15	0.00	0.00	0.13	0.19	0.19	0.00	0.00	0.00	0.00	0.00	0.15	0.00	0.00	0.00	0.00	0.00	0.00
RELB	0.00	0.00	0.00	0.16	0.00	0.00	0.13	0.20	0.19	0.00	0.00	0.00	0.00	0.00	0.16	0.00	0.00	0.00	0.00	0.00	0.00
MYOT	0.00	0.00	0.00	0.21	0.00	0.28	0.12	0.00	0.00	0.00	0.11	0.00	0.00	0.00	0.16	0.00	0.00	0.00	0.00	0.00	0.00
AP5Z1	0.00	0.00	0.00	0.29	0.00	0.51	0.11	0.00	0.00	0.00	0.11	0.00	0.00	0.00	0.12	0.00	0.00	0.00	0.00	0.00	0.00
IGHG3	0.00	0.00	0.00	0.24	0.00	0.36	0.13	0.00	0.00	0.00	0.13	0.00	0.00	0.00	0.14	0.00	0.00	0.00	0.00	0.00	0.00
KRTAP1-5	0.00	0.00	0.00	0.22	0.00	0.33	0.10	0.00	0.00	0.00	0.12	0.00	0.00	0.00	0.17	0.00	0.00	0.00	0.00	0.00	0.00
KRTAP1-4	0.00	0.00	0.00	0.22	0.00	0.33	0.10	0.00	0.00	0.00	0.12	0.00	0.00	0.00	0.17	0.00	0.00	0.00	0.00	0.00	0.00
LOXHD1	0.00	0.00	0.00	0.15	0.00	0.29	0.00	0.11	0.00	0.00	0.12	0.00	0.00	0.00	0.15	0.00	0.00	0.00	0.00	0.00	0.00
PCDH15	0.00	0.00	0.00	0.13	0.00	0.15	0.00	0.10	0.00	0.00	0.12	0.00	0.00	0.00	0.15	0.00	0.00	0.00	0.00	0.00	0.00
C17ORF28	0.00	0.00	0.00	0.24	0.00	0.47	0.00	0.16	0.00	0.00	0.14	0.00	0.00	0.00	0.14	0.00	0.00	0.00	0.00	0.00	0.00
SUN1	0.00	0.00	0.00	0.20	0.00	0.35	0.00	0.14	0.00	0.00	0.13	0.00	0.00	0.00	0.14	0.00	0.00	0.00	0.00	0.00	0.00
DSG4	0.00	0.00	0.00	0.16	0.00	0.27	0.00	0.10	0.00	0.00	0.17	0.00	0.00	0.00	0.16	0.00	0.00	0.00	0.00	0.00	0.00
BSND	0.00	0.00	0.00	0.16	0.00	0.21	0.00	0.11	0.00	0.00	0.16	0.00	0.00	0.00	0.15	0.00	0.00	0.00	0.00	0.00	0.00
TECTA	0.00	0.00	0.00	0.17	0.00	0.21	0.00	0.10	0.00	0.00	0.16	0.00	0.00	0.00	0.16	0.00	0.00	0.00	0.00	0.00	0.00
EMD	0.00	0.00	0.00	0.17	0.00	0.21	0.00	0.14	0.00	0.00	0.13	0.00	0.00	0.00	0.12	0.00	0.00	0.00	0.00	0.00	0.00
OTOA	0.00	0.00	0.00	0.20	0.00	0.39	0.00	0.16	0.00	0.00	0.17	0.00	0.00	0.00	0.12	0.00	0.00	0.00	0.00	0.00	0.00
CHST6	0.00	0.00	0.00	0.12	0.00	0.22	0.00	0.00	0.12	0.00	0.10	0.00	0.00	0.00	0.12	0.00	0.00	0.00	0.00	0.00	0.00
SCN4A	0.00	0.00	0.00	0.12	0.00	0.12	0.00	0.00	0.16	0.00	0.17	0.00	0.00	0.00	0.15	0.00	0.00	0.00	0.00	0.00	0.00
USH2A	0.00	0.00	0.00	0.14	0.00	0.19	0.00	0.00	0.12	0.00	0.11	0.00	0.00	0.00	0.15	0.00	0.00	0.00	0.00	0.00	0.00
CRB1	0.00	0.00	0.00	0.16	0.00	0.23	0.00	0.00	0.12	0.00	0.11	0.00	0.00	0.00	0.19	0.00	0.00	0.00	0.00	0.00	0.00
ALG5	0.00	0.00	0.00	0.17	0.00	0.29	0.00	0.00	0.11	0.00	0.19	0.00	0.00	0.00	0.12	0.00	0.00	0.00	0.00	0.00	0.00
SGCB	0.00	0.00	0.00	0.14	0.00	0.18	0.00	0.00	0.18	0.00	0.14	0.00	0.00	0.00	0.16	0.00	0.00	0.00	0.00	0.00	0.00
SGCD	0.00	0.00	0.00	0.14	0.00	0.15	0.00	0.00	0.24	0.00	0.14	0.00	0.00	0.00	0.14	0.00	0.00	0.00	0.00	0.00	0.00
TMEM43	0.00	0.00	0.00	0.19	0.00	0.31	0.00	0.00	0.13	0.00	0.14	0.00	0.00	0.00	0.14	0.00	0.00	0.00	0.00	0.00	0.00
BEST1	0.00	0.00	0.00	0.14	0.00	0.17	0.00	0.00	0.14	0.00	0.16	0.00	0.00	0.00	0.15	0.00	0.00	0.00	0.00	0.00	0.00
DYX8	0.00	0.00	0.00	0.21	0.00	0.28	0.12	0.00	0.00	0.00	0.00	0.11	0.00	0.00	0.13	0.00	0.00	0.00	0.00	0.00	0.00
COL28A1	0.00	0.00	0.00	0.26	0.00	0.44	0.11	0.00	0.00	0.00	0.00	0.17	0.00	0.00	0.29	0.00	0.00	0.00	0.00	0.00	0.00
MYO1D	0.00	0.00	0.00	0.20	0.00	0.28	0.11	0.00	0.00	0.00	0.00	0.14	0.00	0.00	0.16	0.00	0.00	0.00	0.00	0.00	0.00
MYO7B	0.00	0.00	0.00	0.22	0.00	0.32	0.10	0.00	0.00	0.00	0.00	0.13	0.00	0.00	0.10	0.00	0.00	0.00	0.00	0.00	0.00
FAM13A	0.00	0.00	0.00	0.19	0.00	0.25	0.12	0.00	0.00	0.00	0.00	0.18	0.00	0.00	0.17	0.00	0.00	0.00	0.00	0.00	0.00
DENND4B	0.00	0.00	0.00	0.34	0.00	0.61	0.13	0.00	0.00	0.00	0.00	0.28	0.00	0.00	0.22	0.00	0.00	0.00	0.00	0.00	0.00
HEATR7B2	0.00	0.00	0.00	0.26	0.00	0.43	0.11	0.00	0.00	0.00	0.00	0.17	0.00	0.00	0.19	0.00	0.00	0.00	0.00	0.00	0.00
PQBP4	0.00	0.00	0.00	0.26	0.00	0.52	0.00	0.11	0.00	0.00	0.00	0.21	0.00	0.00	0.21	0.00	0.00	0.00	0.00	0.00	0.00
CAMKMT	0.00	0.00	0.00	0.22	0.00	0.50	0.00	0.23	0.00	0.00	0.00	0.17	0.00	0.00	0.18	0.00	0.00	0.00	0.00	0.00	0.00
TCOF1	0.00	0.00	0.00	0.22	0.00	0.35	0.00	0.17	0.00	0.00	0.00	0.14	0.00	0.00	0.21	0.00	0.00	0.00	0.00	0.00	0.00

EYA1	0.00	0.00	0.00	0.14	0.00	0.20	0.00	0.21	0.00	0.00	0.00	0.13	0.00	0.00	0.21	0.00	0.00	0.00	0.00	0.00	0.00
PMS2L2	0.00	0.00	0.00	0.17	0.00	0.36	0.00	0.10	0.00	0.00	0.00	0.11	0.00	0.00	0.11	0.00	0.00	0.00	0.00	0.00	0.00
KLHDC8B	0.00	0.00	0.00	0.24	0.00	0.46	0.00	0.21	0.00	0.00	0.00	0.14	0.00	0.00	0.13	0.00	0.00	0.00	0.00	0.00	0.00
SKP1P1	0.00	0.00	0.00	0.24	0.00	0.49	0.00	0.14	0.00	0.00	0.00	0.18	0.00	0.00	0.21	0.00	0.00	0.00	0.00	0.00	0.00
PHF8	0.00	0.00	0.00	0.22	0.00	0.34	0.00	0.30	0.00	0.00	0.00	0.16	0.00	0.00	0.18	0.00	0.00	0.00	0.00	0.00	0.00
ATRX	0.00	0.00	0.00	0.14	0.00	0.22	0.00	0.15	0.00	0.00	0.00	0.14	0.00	0.00	0.20	0.00	0.00	0.00	0.00	0.00	0.00
HPS5	0.00	0.00	0.00	0.18	0.00	0.24	0.00	0.11	0.00	0.00	0.00	0.16	0.00	0.00	0.15	0.00	0.00	0.00	0.00	0.00	0.00
BCL7B	0.00	0.00	0.00	0.26	0.00	0.48	0.00	0.22	0.00	0.00	0.00	0.21	0.00	0.00	0.19	0.00	0.00	0.00	0.00	0.00	0.00
SCAANT1	0.00	0.00	0.00	0.17	0.00	0.33	0.00	0.21	0.00	0.00	0.00	0.14	0.00	0.00	0.14	0.00	0.00	0.00	0.00	0.00	0.00
ZNF664	0.00	0.00	0.00	0.24	0.00	0.53	0.00	0.13	0.00	0.00	0.00	0.25	0.00	0.00	0.23	0.00	0.00	0.00	0.00	0.00	0.00
SEA	0.00	0.00	0.00	0.17	0.00	0.25	0.00	0.11	0.00	0.00	0.00	0.12	0.00	0.00	0.12	0.00	0.00	0.00	0.00	0.00	0.00
ZBED5	0.00	0.00	0.00	0.30	0.00	0.62	0.00	0.33	0.00	0.00	0.00	0.22	0.00	0.00	0.24	0.00	0.00	0.00	0.00	0.00	0.00
SMPX	0.00	0.00	0.00	0.19	0.00	0.38	0.00	0.16	0.00	0.00	0.00	0.14	0.00	0.00	0.19	0.00	0.00	0.00	0.00	0.00	0.00
VCX3B	0.00	0.00	0.00	0.21	0.00	0.44	0.00	0.11	0.00	0.00	0.00	0.24	0.00	0.00	0.22	0.00	0.00	0.00	0.00	0.00	0.00
ST8SIA3	0.00	0.00	0.00	0.20	0.00	0.37	0.00	0.10	0.00	0.00	0.00	0.24	0.00	0.00	0.19	0.00	0.00	0.00	0.00	0.00	0.00
IFT27	0.00	0.00	0.00	0.21	0.00	0.52	0.00	0.21	0.00	0.00	0.00	0.12	0.00	0.00	0.11	0.00	0.00	0.00	0.00	0.00	0.00
DNAH2	0.00	0.00	0.00	0.21	0.00	0.43	0.00	0.16	0.00	0.00	0.00	0.22	0.00	0.00	0.21	0.00	0.00	0.00	0.00	0.00	0.00
ACRV1	0.00	0.00	0.00	0.20	0.00	0.42	0.00	0.16	0.00	0.00	0.00	0.13	0.00	0.00	0.15	0.00	0.00	0.00	0.00	0.00	0.00
OFD1	0.00	0.00	0.00	0.20	0.00	0.31	0.00	0.12	0.00	0.00	0.00	0.17	0.00	0.00	0.20	0.00	0.00	0.00	0.00	0.00	0.00
WDPCP	0.00	0.00	0.00	0.26	0.00	0.47	0.00	0.25	0.00	0.00	0.00	0.14	0.00	0.00	0.21	0.00	0.00	0.00	0.00	0.00	0.00
DGCR5	0.00	0.00	0.00	0.27	0.00	0.48	0.00	0.39	0.00	0.00	0.00	0.23	0.00	0.00	0.29	0.00	0.00	0.00	0.00	0.00	0.00
RABGGTA	0.00	0.00	0.00	0.20	0.00	0.32	0.00	0.20	0.00	0.00	0.00	0.16	0.00	0.00	0.14	0.00	0.00	0.00	0.00	0.00	0.00
CEP152	0.00	0.00	0.00	0.20	0.00	0.41	0.00	0.16	0.00	0.00	0.00	0.12	0.00	0.00	0.16	0.00	0.00	0.00	0.00	0.00	0.00
LRRC6	0.00	0.00	0.00	0.26	0.00	0.52	0.00	0.15	0.00	0.00	0.00	0.22	0.00	0.00	0.22	0.00	0.00	0.00	0.00	0.00	0.00
PMS2P6	0.00	0.00	0.00	0.20	0.00	0.40	0.00	0.13	0.00	0.00	0.00	0.11	0.00	0.00	0.11	0.00	0.00	0.00	0.00	0.00	0.00
KDM5C	0.00	0.00	0.00	0.21	0.00	0.36	0.00	0.24	0.00	0.00	0.00	0.17	0.00	0.00	0.18	0.00	0.00	0.00	0.00	0.00	0.00
TRIM74	0.00	0.00	0.00	0.22	0.00	0.41	0.00	0.13	0.00	0.00	0.00	0.12	0.00	0.00	0.15	0.00	0.00	0.00	0.00	0.00	0.00
RPL21	0.00	0.00	0.00	0.21	0.00	0.40	0.00	0.11	0.00	0.00	0.00	0.17	0.00	0.00	0.16	0.00	0.00	0.00	0.00	0.00	0.00
TRIM50	0.00	0.00	0.00	0.22	0.00	0.41	0.00	0.13	0.00	0.00	0.00	0.12	0.00	0.00	0.15	0.00	0.00	0.00	0.00	0.00	0.00
TRIM73	0.00	0.00	0.00	0.22	0.00	0.41	0.00	0.13	0.00	0.00	0.00	0.12	0.00	0.00	0.15	0.00	0.00	0.00	0.00	0.00	0.00
SRSF10P2	0.00	0.00	0.00	0.26	0.00	0.56	0.00	0.16	0.00	0.00	0.00	0.14	0.00	0.00	0.12	0.00	0.00	0.00	0.00	0.00	0.00
ABHD11-AS1	0.00	0.00	0.00	0.22	0.00	0.41	0.00	0.13	0.00	0.00	0.00	0.12	0.00	0.00	0.15	0.00	0.00	0.00	0.00	0.00	0.00
PRPF40B	0.00	0.00	0.00	0.16	0.00	0.30	0.00	0.12	0.00	0.00	0.00	0.14	0.00	0.00	0.12	0.00	0.00	0.00	0.00	0.00	0.00
TRIOBP	0.00	0.00	0.00	0.24	0.00	0.41	0.00	0.30	0.00	0.00	0.00	0.12	0.00	0.00	0.16	0.00	0.00	0.00	0.00	0.00	0.00
KIF24	0.00	0.00	0.00	0.18	0.00	0.32	0.00	0.12	0.00	0.00	0.00	0.11	0.00	0.00	0.12	0.00	0.00	0.00	0.00	0.00	0.00
LMNB1	0.00	0.00	0.00	0.17	0.00	0.24	0.00	0.20	0.00	0.00	0.00	0.11	0.00	0.00	0.18	0.00	0.00	0.00	0.00	0.00	0.00
SALL1	0.00	0.00	0.00	0.18	0.00	0.28	0.00	0.26	0.00	0.00	0.00	0.14	0.00	0.00	0.25	0.00	0.00	0.00	0.00	0.00	0.00
MED9	0.00	0.00	0.00	0.21	0.00	0.35	0.00	0.12	0.00	0.00	0.00	0.16	0.00	0.00	0.23	0.00	0.00	0.00	0.00	0.00	0.00
GTF2IRD2	0.00	0.00	0.00	0.25	0.00	0.49	0.00	0.15	0.00	0.00	0.00	0.21	0.00	0.00	0.25	0.00	0.00	0.00	0.00	0.00	0.00
RNASEH2C	0.00	0.00	0.00	0.18	0.00	0.42	0.00	0.13	0.00	0.00	0.00	0.11	0.00	0.00	0.15	0.00	0.00	0.00	0.00	0.00	0.00

RNF32	0.00	0.00	0.00	0.23	0.00	0.45	0.00	0.21	0.00	0.00	0.00	0.10	0.00	0.00	0.16	0.00	0.00	0.00	0.00	0.00	0.00
ACTR10	0.00	0.00	0.00	0.20	0.00	0.36	0.00	0.12	0.00	0.00	0.00	0.13	0.00	0.00	0.13	0.00	0.00	0.00	0.00	0.00	0.00
ZNF723	0.00	0.00	0.00	0.23	0.00	0.42	0.00	0.22	0.00	0.00	0.00	0.13	0.00	0.00	0.16	0.00	0.00	0.00	0.00	0.00	0.00
GTF2IRD2B	0.00	0.00	0.00	0.27	0.00	0.51	0.00	0.18	0.00	0.00	0.00	0.20	0.00	0.00	0.24	0.00	0.00	0.00	0.00	0.00	0.00
KIAA1731	0.00	0.00	0.00	0.23	0.00	0.52	0.00	0.13	0.00	0.00	0.00	0.16	0.00	0.00	0.16	0.00	0.00	0.00	0.00	0.00	0.00
IFITM8P	0.00	0.00	0.00	0.23	0.00	0.42	0.00	0.22	0.00	0.00	0.00	0.13	0.00	0.00	0.16	0.00	0.00	0.00	0.00	0.00	0.00
NOL10	0.00	0.00	0.00	0.27	0.00	0.61	0.00	0.13	0.00	0.00	0.00	0.17	0.00	0.00	0.22	0.00	0.00	0.00	0.00	0.00	0.00
HSPB9	0.00	0.00	0.00	0.21	0.00	0.44	0.00	0.11	0.00	0.00	0.00	0.16	0.00	0.00	0.12	0.00	0.00	0.00	0.00	0.00	0.00
TWISTNB	0.00	0.00	0.00	0.23	0.00	0.52	0.00	0.14	0.00	0.00	0.00	0.18	0.00	0.00	0.16	0.00	0.00	0.00	0.00	0.00	0.00
MRPS26	0.00	0.00	0.00	0.23	0.00	0.40	0.00	0.19	0.00	0.00	0.00	0.11	0.00	0.00	0.13	0.00	0.00	0.00	0.00	0.00	0.00
CBX8	0.00	0.00	0.00	0.20	0.00	0.32	0.00	0.34	0.00	0.00	0.00	0.15	0.00	0.00	0.21	0.00	0.00	0.00	0.00	0.00	0.00
NOP14	0.00	0.00	0.00	0.30	0.00	0.63	0.00	0.15	0.00	0.00	0.00	0.15	0.00	0.00	0.16	0.00	0.00	0.00	0.00	0.00	0.00
EPM2AIP1	0.00	0.00	0.00	0.26	0.00	0.51	0.00	0.27	0.00	0.00	0.00	0.13	0.00	0.00	0.14	0.00	0.00	0.00	0.00	0.00	0.00
PPP2R5C	0.00	0.00	0.00	0.20	0.00	0.32	0.00	0.30	0.00	0.00	0.00	0.18	0.00	0.00	0.13	0.00	0.00	0.00	0.00	0.00	0.00
CCDC102A	0.00	0.00	0.00	0.16	0.00	0.32	0.00	0.12	0.00	0.00	0.00	0.18	0.00	0.00	0.21	0.00	0.00	0.00	0.00	0.00	0.00
IFT80	0.00	0.00	0.00	0.24	0.00	0.44	0.00	0.17	0.00	0.00	0.00	0.15	0.00	0.00	0.19	0.00	0.00	0.00	0.00	0.00	0.00
FOXE3	0.00	0.00	0.00	0.17	0.00	0.23	0.00	0.20	0.00	0.00	0.00	0.13	0.00	0.00	0.20	0.00	0.00	0.00	0.00	0.00	0.00
TUBGCP5	0.00	0.00	0.00	0.24	0.00	0.41	0.00	0.17	0.00	0.00	0.00	0.19	0.00	0.00	0.20	0.00	0.00	0.00	0.00	0.00	0.00
WBSCR27	0.00	0.00	0.00	0.23	0.00	0.46	0.00	0.12	0.00	0.00	0.00	0.13	0.00	0.00	0.16	0.00	0.00	0.00	0.00	0.00	0.00
CGNL1	0.00	0.00	0.00	0.18	0.00	0.34	0.00	0.15	0.00	0.00	0.00	0.15	0.00	0.00	0.15	0.00	0.00	0.00	0.00	0.00	0.00
MID1	0.00	0.00	0.00	0.21	0.00	0.38	0.00	0.23	0.00	0.00	0.00	0.18	0.00	0.00	0.22	0.00	0.00	0.00	0.00	0.00	0.00
C4ORF6	0.00	0.00	0.00	0.20	0.00	0.38	0.00	0.15	0.00	0.00	0.00	0.17	0.00	0.00	0.21	0.00	0.00	0.00	0.00	0.00	0.00
SH3BGR	0.00	0.00	0.00	0.23	0.00	0.45	0.00	0.12	0.00	0.00	0.00	0.22	0.00	0.00	0.21	0.00	0.00	0.00	0.00	0.00	0.00
ZNF192	0.00	0.00	0.00	0.28	0.00	0.57	0.00	0.10	0.00	0.00	0.00	0.13	0.00	0.00	0.16	0.00	0.00	0.00	0.00	0.00	0.00
RPS27P3	0.00	0.00	0.00	0.23	0.00	0.42	0.00	0.22	0.00	0.00	0.00	0.13	0.00	0.00	0.16	0.00	0.00	0.00	0.00	0.00	0.00
RPS24	0.00	0.00	0.00	0.24	0.00	0.47	0.00	0.19	0.00	0.00	0.00	0.10	0.00	0.00	0.15	0.00	0.00	0.00	0.00	0.00	0.00
ZNF726	0.00	0.00	0.00	0.23	0.00	0.42	0.00	0.22	0.00	0.00	0.00	0.13	0.00	0.00	0.16	0.00	0.00	0.00	0.00	0.00	0.00
EMG1	0.00	0.00	0.00	0.26	0.00	0.54	0.00	0.18	0.00	0.00	0.00	0.16	0.00	0.00	0.24	0.00	0.00	0.00	0.00	0.00	0.00
UBAP2	0.00	0.00	0.00	0.19	0.00	0.36	0.00	0.11	0.00	0.00	0.00	0.16	0.00	0.00	0.22	0.00	0.00	0.00	0.00	0.00	0.00
HIGD1AP1	0.00	0.00	0.00	0.23	0.00	0.42	0.00	0.22	0.00	0.00	0.00	0.13	0.00	0.00	0.16	0.00	0.00	0.00	0.00	0.00	0.00
EPG5	0.00	0.00	0.00	0.28	0.00	0.55	0.00	0.24	0.00	0.00	0.00	0.17	0.00	0.00	0.18	0.00	0.00	0.00	0.00	0.00	0.00
GTF2IRD2P1	0.00	0.00	0.00	0.26	0.00	0.52	0.00	0.13	0.00	0.00	0.00	0.20	0.00	0.00	0.24	0.00	0.00	0.00	0.00	0.00	0.00
HIGD1C	0.00	0.00	0.00	0.23	0.00	0.42	0.00	0.22	0.00	0.00	0.00	0.13	0.00	0.00	0.16	0.00	0.00	0.00	0.00	0.00	0.00
VENTXP7	0.00	0.00	0.00	0.25	0.00	0.50	0.00	0.19	0.00	0.00	0.00	0.13	0.00	0.00	0.18	0.00	0.00	0.00	0.00	0.00	0.00
FSIP1	0.00	0.00	0.00	0.20	0.00	0.37	0.00	0.11	0.00	0.00	0.00	0.14	0.00	0.00	0.12	0.00	0.00	0.00	0.00	0.00	0.00
DNAJB8	0.00	0.00	0.00	0.25	0.00	0.43	0.00	0.21	0.00	0.00	0.00	0.13	0.00	0.00	0.23	0.00	0.00	0.00	0.00	0.00	0.00
PBLD	0.00	0.00	0.00	0.23	0.00	0.45	0.00	0.16	0.00	0.00	0.00	0.20	0.00	0.00	0.26	0.00	0.00	0.00	0.00	0.00	0.00
MRPL54	0.00	0.00	0.00	0.28	0.00	0.56	0.00	0.15	0.00	0.00	0.00	0.15	0.00	0.00	0.18	0.00	0.00	0.00	0.00	0.00	0.00
C7ORF13	0.00	0.00	0.00	0.28	0.00	0.55	0.00	0.14	0.00	0.00	0.00	0.13	0.00	0.00	0.13	0.00	0.00	0.00	0.00	0.00	0.00
VSX1	0.00	0.00	0.00	0.19	0.00	0.28	0.00	0.17	0.00	0.00	0.00	0.12	0.00	0.00	0.24	0.00	0.00	0.00	0.00	0.00	0.00

TDRD9	0.00	0.00	0.00	0.20	0.00	0.41	0.00	0.13	0.00	0.00	0.00	0.14	0.00	0.00	0.12	0.00	0.00	0.00	0.00	0.00	0.00
PPP2R3B	0.00	0.00	0.00	0.23	0.00	0.50	0.00	0.20	0.00	0.00	0.00	0.20	0.00	0.00	0.13	0.00	0.00	0.00	0.00	0.00	0.00
PPHLN1	0.00	0.00	0.00	0.27	0.00	0.48	0.00	0.28	0.00	0.00	0.00	0.18	0.00	0.00	0.25	0.00	0.00	0.00	0.00	0.00	0.00
RPS4Y1	0.00	0.00	0.00	0.25	0.00	0.46	0.00	0.12	0.00	0.00	0.00	0.29	0.00	0.00	0.24	0.00	0.00	0.00	0.00	0.00	0.00
CHD7	0.00	0.00	0.00	0.16	0.00	0.20	0.00	0.18	0.00	0.00	0.00	0.13	0.00	0.00	0.24	0.00	0.00	0.00	0.00	0.00	0.00
TTC26	0.00	0.00	0.00	0.22	0.00	0.46	0.00	0.15	0.00	0.00	0.00	0.18	0.00	0.00	0.19	0.00	0.00	0.00	0.00	0.00	0.00
SUN3	0.00	0.00	0.00	0.20	0.00	0.42	0.00	0.11	0.00	0.00	0.00	0.14	0.00	0.00	0.18	0.00	0.00	0.00	0.00	0.00	0.00
TARBP2P	0.00	0.00	0.00	0.19	0.00	0.36	0.00	0.19	0.00	0.00	0.00	0.11	0.00	0.00	0.15	0.00	0.00	0.00	0.00	0.00	0.00
DNAH5	0.00	0.00	0.00	0.19	0.00	0.28	0.00	0.00	0.12	0.00	0.00	0.13	0.00	0.00	0.18	0.00	0.00	0.00	0.00	0.00	0.00
ATXN8OS	0.00	0.00	0.00	0.10	0.00	0.18	0.00	0.00	0.14	0.00	0.00	0.16	0.00	0.00	0.18	0.00	0.00	0.00	0.00	0.00	0.00
AASTH41	0.00	0.00	0.00	0.14	0.00	0.20	0.00	0.00	0.13	0.00	0.00	0.11	0.00	0.00	0.14	0.00	0.00	0.00	0.00	0.00	0.00
C20ORF194	0.00	0.00	0.00	0.11	0.00	0.26	0.00	0.00	0.15	0.00	0.00	0.13	0.00	0.00	0.16	0.00	0.00	0.00	0.00	0.00	0.00
EVR3	0.00	0.00	0.00	0.13	0.00	0.19	0.00	0.00	0.10	0.00	0.00	0.12	0.00	0.00	0.17	0.00	0.00	0.00	0.00	0.00	0.00
MDC1B	0.00	0.00	0.00	0.17	0.00	0.30	0.00	0.00	0.11	0.00	0.00	0.15	0.00	0.00	0.15	0.00	0.00	0.00	0.00	0.00	0.00
FLJ45872	0.00	0.00	0.00	0.14	0.00	0.16	0.00	0.00	0.19	0.00	0.00	0.11	0.00	0.00	0.11	0.00	0.00	0.00	0.00	0.00	0.00
BRCD1	0.00	0.00	0.00	0.00	0.00	0.16	0.00	0.27	0.21	0.00	0.00	0.15	0.00	0.00	0.11	0.00	0.00	0.00	0.00	0.00	0.00
WHAMMP3	0.00	0.00	0.00	0.25	0.00	0.52	0.00	0.00	0.00	0.00	0.13	0.25	0.00	0.00	0.17	0.00	0.00	0.00	0.00	0.00	0.00
GOLGA8F	0.00	0.00	0.00	0.25	0.00	0.52	0.00	0.00	0.00	0.00	0.13	0.25	0.00	0.00	0.17	0.00	0.00	0.00	0.00	0.00	0.00
EEGV1	0.00	0.00	0.00	0.21	0.00	0.35	0.00	0.00	0.00	0.00	0.10	0.11	0.00	0.00	0.12	0.00	0.00	0.00	0.00	0.00	0.00
SEC23B	0.00	0.00	0.00	0.21	0.00	0.38	0.00	0.00	0.00	0.00	0.17	0.10	0.00	0.00	0.14	0.00	0.00	0.00	0.00	0.00	0.00
GJD3	0.00	0.00	0.00	0.19	0.00	0.24	0.00	0.00	0.00	0.00	0.15	0.15	0.00	0.00	0.20	0.00	0.00	0.00	0.00	0.00	0.00
MMEDF	0.00	0.00	0.00	0.13	0.00	0.27	0.00	0.00	0.00	0.00	0.11	0.15	0.00	0.00	0.18	0.00	0.00	0.00	0.00	0.00	0.00
LHFPL3	0.00	0.00	0.00	0.22	0.00	0.36	0.00	0.00	0.00	0.00	0.22	0.10	0.00	0.00	0.14	0.00	0.00	0.00	0.00	0.00	0.00
GJB4	0.00	0.00	0.00	0.18	0.00	0.25	0.00	0.00	0.00	0.00	0.14	0.10	0.00	0.00	0.15	0.00	0.00	0.00	0.00	0.00	0.00
LHFP	0.00	0.00	0.00	0.22	0.00	0.36	0.00	0.00	0.00	0.00	0.22	0.10	0.00	0.00	0.14	0.00	0.00	0.00	0.00	0.00	0.00
ABCD1P1	0.00	0.00	0.00	0.17	0.00	0.37	0.00	0.00	0.00	0.00	0.11	0.15	0.00	0.00	0.13	0.00	0.00	0.00	0.00	0.00	0.00
LHFPL2	0.00	0.00	0.00	0.22	0.00	0.36	0.00	0.00	0.00	0.00	0.22	0.10	0.00	0.00	0.14	0.00	0.00	0.00	0.00	0.00	0.00
WHAMM	0.00	0.00	0.00	0.29	0.00	0.56	0.00	0.00	0.00	0.00	0.12	0.24	0.00	0.00	0.17	0.00	0.00	0.00	0.00	0.00	0.00
KCNV2	0.00	0.00	0.00	0.12	0.00	0.22	0.00	0.00	0.00	0.00	0.13	0.11	0.00	0.00	0.14	0.00	0.00	0.00	0.00	0.00	0.00
VMA21	0.00	0.00	0.00	0.16	0.00	0.26	0.00	0.00	0.00	0.00	0.12	0.11	0.00	0.00	0.12	0.00	0.00	0.00	0.00	0.00	0.00
CLRN3	0.00	0.00	0.00	0.22	0.00	0.42	0.00	0.00	0.00	0.00	0.14	0.14	0.00	0.00	0.17	0.00	0.00	0.00	0.00	0.00	0.00
KBTBD13	0.00	0.00	0.00	0.23	0.00	0.39	0.00	0.00	0.00	0.00	0.11	0.11	0.00	0.00	0.17	0.00	0.00	0.00	0.00	0.00	0.00
CATSPER2P1	0.00	0.00	0.00	0.13	0.00	0.30	0.00	0.00	0.00	0.00	0.14	0.10	0.00	0.00	0.12	0.00	0.00	0.00	0.00	0.00	0.00
LHFPL4	0.00	0.00	0.00	0.22	0.00	0.36	0.00	0.00	0.00	0.00	0.22	0.10	0.00	0.00	0.14	0.00	0.00	0.00	0.00	0.00	0.00
PMM2P1	0.00	0.00	0.00	0.16	0.00	0.37	0.00	0.00	0.00	0.00	0.11	0.11	0.00	0.00	0.12	0.00	0.00	0.00	0.00	0.00	0.00
LRRC52	0.00	0.00	0.00	0.16	0.00	0.27	0.00	0.00	0.00	0.00	0.13	0.15	0.00	0.00	0.11	0.00	0.00	0.00	0.00	0.00	0.00
IPW	0.00	0.00	0.00	0.19	0.00	0.31	0.00	0.00	0.00	0.00	0.10	0.25	0.00	0.00	0.21	0.00	0.00	0.00	0.00	0.00	0.00
NBEAL2	0.00	0.00	0.00	0.14	0.00	0.32	0.00	0.00	0.00	0.00	0.11	0.10	0.00	0.00	0.12	0.00	0.00	0.00	0.00	0.00	0.00
DSAP2	0.00	0.00	0.00	0.16	0.00	0.28	0.00	0.00	0.00	0.00	0.10	0.11	0.00	0.00	0.13	0.00	0.00	0.00	0.00	0.00	0.00
KCNS3	0.00	0.00	0.00	0.15	0.00	0.24	0.00	0.00	0.00	0.00	0.12	0.24	0.00	0.00	0.20	0.00	0.00	0.00	0.00	0.00	0.00

FASA	0.00	0.00	0.00	0.28	0.00	0.57	0.00	0.00	0.00	0.00	0.14	0.23	0.00	0.00	0.11	0.00	0.00	0.00	0.00	0.00	0.00
SPG16	0.00	0.00	0.00	0.13	0.00	0.30	0.00	0.00	0.00	0.00	0.10	0.14	0.00	0.00	0.13	0.00	0.00	0.00	0.00	0.00	0.00
SCNM1	0.00	0.00	0.00	0.18	0.00	0.31	0.00	0.00	0.00	0.00	0.11	0.17	0.00	0.00	0.24	0.00	0.00	0.00	0.00	0.00	0.00
CLRN2	0.00	0.00	0.00	0.19	0.00	0.36	0.00	0.00	0.00	0.00	0.12	0.13	0.00	0.00	0.17	0.00	0.00	0.00	0.00	0.00	0.00
ALDH7A1P4	0.00	0.00	0.00	0.26	0.00	0.50	0.00	0.00	0.00	0.00	0.14	0.19	0.00	0.00	0.16	0.00	0.00	0.00	0.00	0.00	0.00
TRAPPC10	0.00	0.00	0.00	0.24	0.00	0.56	0.00	0.00	0.00	0.00	0.11	0.21	0.00	0.00	0.18	0.00	0.00	0.00	0.00	0.00	0.00
ALDH7A1P2	0.00	0.00	0.00	0.26	0.00	0.50	0.00	0.00	0.00	0.00	0.14	0.19	0.00	0.00	0.16	0.00	0.00	0.00	0.00	0.00	0.00
ALDH7A1P3	0.00	0.00	0.00	0.26	0.00	0.50	0.00	0.00	0.00	0.00	0.14	0.19	0.00	0.00	0.16	0.00	0.00	0.00	0.00	0.00	0.00
PRRT2	0.00	0.00	0.00	0.13	0.00	0.27	0.00	0.00	0.00	0.00	0.15	0.16	0.00	0.00	0.16	0.00	0.00	0.00	0.00	0.00	0.00
FAM83H	0.00	0.00	0.00	0.18	0.00	0.31	0.00	0.00	0.00	0.00	0.12	0.12	0.00	0.00	0.13	0.00	0.00	0.00	0.00	0.00	0.00
GJA9	0.00	0.00	0.00	0.23	0.00	0.43	0.00	0.00	0.00	0.00	0.12	0.20	0.00	0.00	0.18	0.00	0.00	0.00	0.00	0.00	0.00
CAMSAP3	0.00	0.00	0.00	0.25	0.00	0.52	0.00	0.00	0.00	0.00	0.11	0.19	0.00	0.00	0.25	0.00	0.00	0.00	0.00	0.00	0.00
OFD1P18Y	0.00	0.00	0.00	0.28	0.00	0.58	0.00	0.00	0.00	0.00	0.13	0.17	0.00	0.00	0.15	0.00	0.00	0.00	0.00	0.00	0.00
CRYM-AS1	0.00	0.00	0.00	0.18	0.00	0.41	0.00	0.00	0.00	0.00	0.27	0.17	0.00	0.00	0.11	0.00	0.00	0.00	0.00	0.00	0.00
CC2D2A	0.00	0.00	0.00	0.17	0.00	0.29	0.00	0.00	0.00	0.00	0.10	0.17	0.00	0.00	0.17	0.00	0.00	0.00	0.00	0.00	0.00
ZNF791	0.00	0.00	0.00	0.16	0.00	0.26	0.00	0.00	0.00	0.00	0.10	0.12	0.00	0.00	0.13	0.00	0.00	0.00	0.00	0.00	0.00
TMC2	0.00	0.00	0.00	0.22	0.00	0.38	0.00	0.00	0.00	0.00	0.22	0.12	0.00	0.00	0.16	0.00	0.00	0.00	0.00	0.00	0.00
GJD4	0.00	0.00	0.00	0.19	0.00	0.28	0.00	0.00	0.00	0.00	0.12	0.14	0.00	0.00	0.17	0.00	0.00	0.00	0.00	0.00	0.00
KCNA10	0.00	0.00	0.00	0.18	0.00	0.32	0.00	0.00	0.00	0.00	0.18	0.20	0.00	0.00	0.16	0.00	0.00	0.00	0.00	0.00	0.00
C19ORF43	0.00	0.00	0.00	0.16	0.00	0.26	0.00	0.00	0.00	0.00	0.10	0.12	0.00	0.00	0.13	0.00	0.00	0.00	0.00	0.00	0.00
PSHK2	0.00	0.00	0.00	0.19	0.00	0.30	0.00	0.00	0.00	0.00	0.15	0.12	0.00	0.00	0.14	0.00	0.00	0.00	0.00	0.00	0.00
B3GALTL	0.00	0.00	0.00	0.19	0.00	0.39	0.00	0.00	0.00	0.00	0.17	0.16	0.00	0.00	0.18	0.00	0.00	0.00	0.00	0.00	0.00
CLRN1-AS1	0.00	0.00	0.00	0.20	0.00	0.40	0.00	0.00	0.00	0.00	0.16	0.13	0.00	0.00	0.17	0.00	0.00	0.00	0.00	0.00	0.00
TMEM2	0.00	0.00	0.00	0.22	0.00	0.41	0.00	0.00	0.00	0.00	0.14	0.16	0.00	0.00	0.20	0.00	0.00	0.00	0.00	0.00	0.00
ADAMTS10	0.00	0.00	0.00	0.21	0.00	0.35	0.00	0.00	0.00	0.00	0.15	0.12	0.00	0.00	0.14	0.00	0.00	0.00	0.00	0.00	0.00
KCNJ18	0.00	0.00	0.00	0.16	0.00	0.23	0.00	0.00	0.00	0.00	0.18	0.13	0.00	0.00	0.10	0.00	0.00	0.00	0.00	0.00	0.00
SPPM	0.00	0.00	0.00	0.12	0.00	0.23	0.00	0.00	0.00	0.00	0.12	0.17	0.00	0.00	0.20	0.00	0.00	0.00	0.00	0.00	0.00
KCNG4	0.00	0.00	0.00	0.15	0.00	0.24	0.00	0.00	0.00	0.00	0.23	0.14	0.00	0.00	0.14	0.00	0.00	0.00	0.00	0.00	0.00
OFD1P17	0.00	0.00	0.00	0.28	0.00	0.58	0.00	0.00	0.00	0.00	0.13	0.17	0.00	0.00	0.15	0.00	0.00	0.00	0.00	0.00	0.00
LAMB4	0.00	0.00	0.00	0.22	0.00	0.37	0.00	0.00	0.00	0.00	0.15	0.16	0.00	0.00	0.21	0.00	0.00	0.00	0.00	0.00	0.00
NHSL1	0.00	0.00	0.00	0.24	0.00	0.52	0.00	0.00	0.00	0.00	0.12	0.16	0.00	0.00	0.14	0.00	0.00	0.00	0.00	0.00	0.00
KCNG2	0.00	0.00	0.00	0.11	0.00	0.23	0.00	0.00	0.00	0.00	0.11	0.17	0.00	0.00	0.13	0.00	0.00	0.00	0.00	0.00	0.00
KCNE1L	0.00	0.00	0.00	0.15	0.00	0.25	0.00	0.00	0.00	0.00	0.12	0.17	0.00	0.00	0.18	0.00	0.00	0.00	0.00	0.00	0.00
PIGV	0.00	0.00	0.00	0.20	0.00	0.38	0.00	0.00	0.00	0.00	0.24	0.13	0.00	0.00	0.15	0.00	0.00	0.00	0.00	0.00	0.00
GJB7	0.00	0.00	0.00	0.25	0.00	0.45	0.00	0.00	0.00	0.00	0.15	0.16	0.00	0.00	0.16	0.00	0.00	0.00	0.00	0.00	0.00
B9D1	0.00	0.00	0.00	0.20	0.00	0.39	0.00	0.00	0.00	0.00	0.11	0.19	0.00	0.00	0.22	0.00	0.00	0.00	0.00	0.00	0.00
EDNRB	0.00	0.00	0.16	0.16	0.00	0.00	0.13	0.00	0.00	0.00	0.00	0.00	0.12	0.00	0.14	0.00	0.00	0.00	0.00	0.00	0.00
DRD4	0.00	0.00	0.12	0.13	0.00	0.00	0.12	0.00	0.00	0.00	0.00	0.00	0.13	0.00	0.12	0.00	0.00	0.00	0.00	0.00	0.00
CLCNKB	0.00	0.00	0.00	0.15	0.00	0.16	0.00	0.00	0.00	0.00	0.14	0.00	0.11	0.00	0.14	0.00	0.00	0.00	0.00	0.00	0.00
DYX3	0.00	0.00	0.00	0.19	0.00	0.37	0.00	0.00	0.00	0.00	0.00	0.23	0.12	0.00	0.18	0.00	0.00	0.00	0.00	0.00	0.00

SCZD2	0.00	0.00	0.00	0.12	0.00	0.13	0.00	0.00	0.00	0.00	0.00	0.11	0.13	0.00	0.13	0.00	0.00	0.00	0.00	0.00	0.00
DYX1	0.00	0.00	0.00	0.15	0.00	0.21	0.00	0.00	0.00	0.00	0.00	0.14	0.11	0.00	0.14	0.00	0.00	0.00	0.00	0.00	0.00
GTS	0.00	0.00	0.00	0.13	0.00	0.24	0.00	0.00	0.00	0.00	0.00	0.18	0.12	0.00	0.25	0.00	0.00	0.00	0.00	0.00	0.00
SCZD11	0.00	0.00	0.00	0.11	0.00	0.12	0.00	0.00	0.00	0.00	0.00	0.13	0.14	0.00	0.13	0.00	0.00	0.00	0.00	0.00	0.00
TCERG1L	0.00	0.00	0.00	0.12	0.00	0.20	0.00	0.00	0.00	0.00	0.00	0.13	0.10	0.00	0.11	0.00	0.00	0.00	0.00	0.00	0.00
FOPNL	0.00	0.00	0.00	0.14	0.00	0.28	0.00	0.00	0.00	0.00	0.00	0.16	0.10	0.00	0.13	0.00	0.00	0.00	0.00	0.00	0.00
PAND1	0.00	0.00	0.00	0.13	0.00	0.17	0.00	0.00	0.00	0.00	0.00	0.14	0.12	0.00	0.15	0.00	0.00	0.00	0.00	0.00	0.00
PRD	0.00	0.00	0.00	0.12	0.00	0.18	0.00	0.00	0.00	0.00	0.00	0.21	0.12	0.00	0.18	0.00	0.00	0.00	0.00	0.00	0.00
MRX3	0.00	0.00	0.00	0.12	0.00	0.33	0.00	0.00	0.00	0.00	0.00	0.23	0.13	0.00	0.11	0.00	0.00	0.00	0.00	0.00	0.00
SEL1L3	0.00	0.00	0.00	0.14	0.00	0.15	0.00	0.00	0.00	0.00	0.00	0.11	0.14	0.00	0.13	0.00	0.00	0.00	0.00	0.00	0.00
MRX81	0.00	0.00	0.00	0.16	0.00	0.35	0.00	0.00	0.00	0.00	0.00	0.25	0.13	0.00	0.21	0.00	0.00	0.00	0.00	0.00	0.00
MRX5	0.00	0.00	0.00	0.11	0.00	0.22	0.00	0.00	0.00	0.00	0.00	0.19	0.11	0.00	0.11	0.00	0.00	0.00	0.00	0.00	0.00
PRS	0.00	0.00	0.00	0.11	0.00	0.22	0.00	0.00	0.00	0.00	0.00	0.19	0.11	0.00	0.11	0.00	0.00	0.00	0.00	0.00	0.00
DCDC1	0.00	0.00	0.00	0.17	0.00	0.42	0.00	0.00	0.00	0.00	0.00	0.23	0.16	0.00	0.17	0.00	0.00	0.00	0.00	0.00	0.00
MRX84	0.00	0.00	0.00	0.11	0.00	0.23	0.00	0.00	0.00	0.00	0.00	0.18	0.11	0.00	0.11	0.00	0.00	0.00	0.00	0.00	0.00
CCDC149	0.00	0.00	0.00	0.14	0.00	0.15	0.00	0.00	0.00	0.00	0.00	0.11	0.14	0.00	0.13	0.00	0.00	0.00	0.00	0.00	0.00
SCZD1	0.00	0.00	0.00	0.10	0.00	0.14	0.00	0.00	0.00	0.00	0.00	0.12	0.13	0.00	0.14	0.00	0.00	0.00	0.00	0.00	0.00
LIM2	0.00	0.00	0.00	0.21	0.00	0.33	0.00	0.00	0.00	0.00	0.00	0.18	0.11	0.00	0.25	0.00	0.00	0.00	0.00	0.00	0.00
ZDHHC15	0.00	0.00	0.00	0.16	0.00	0.36	0.00	0.00	0.00	0.00	0.00	0.28	0.10	0.00	0.14	0.00	0.00	0.00	0.00	0.00	0.00
DYT7	0.00	0.00	0.00	0.10	0.00	0.19	0.00	0.00	0.00	0.00	0.00	0.15	0.13	0.00	0.13	0.00	0.00	0.00	0.00	0.00	0.00
MRX26	0.00	0.00	0.00	0.10	0.00	0.16	0.00	0.00	0.00	0.00	0.00	0.13	0.10	0.00	0.10	0.00	0.00	0.00	0.00	0.00	0.00
CAMK2A	0.00	0.00	0.00	0.11	0.00	0.00	0.00	0.13	0.00	0.00	0.00	0.00	0.14	0.20	0.11	0.00	0.00	0.00	0.00	0.00	0.00
MRSS	0.00	0.00	0.00	0.00	0.00	0.16	0.00	0.00	0.00	0.00	0.00	0.20	0.11	0.10	0.11	0.00	0.00	0.00	0.00	0.00	0.00
FPEVF	0.00	0.00	0.00	0.00	0.00	0.14	0.00	0.00	0.00	0.00	0.00	0.12	0.11	0.11	0.11	0.00	0.00	0.00	0.00	0.00	0.00
BEND2	0.00	0.00	0.00	0.00	0.00	0.15	0.00	0.00	0.00	0.00	0.00	0.18	0.11	0.12	0.12	0.00	0.00	0.00	0.00	0.00	0.00
MRX53	0.00	0.00	0.00	0.00	0.00	0.20	0.00	0.00	0.00	0.00	0.00	0.20	0.12	0.10	0.12	0.00	0.00	0.00	0.00	0.00	0.00
ZNF778	0.00	0.00	0.00	0.00	0.00	0.17	0.00	0.00	0.00	0.00	0.00	0.24	0.15	0.16	0.18	0.00	0.00	0.00	0.00	0.00	0.00
IBGC1	0.00	0.00	0.00	0.00	0.00	0.00	0.00	0.00	0.11	0.00	0.00	0.18	0.15	0.16	0.13	0.00	0.00	0.00	0.00	0.00	0.00
SOX10	0.00	0.00	0.00	0.14	0.00	0.11	0.00	0.21	0.00	0.00	0.00	0.00	0.00	0.00	0.30	0.19	0.00	0.00	0.00	0.00	0.00
HOXB3	0.00	0.00	0.00	0.12	0.00	0.16	0.00	0.25	0.00	0.00	0.00	0.00	0.00	0.00	0.26	0.14	0.00	0.00	0.00	0.00	0.00
HOXB1	0.00	0.00	0.00	0.13	0.00	0.00	0.10	0.20	0.00	0.00	0.00	0.00	0.00	0.00	0.26	0.17	0.00	0.00	0.00	0.00	0.00
NEFM	0.00	0.00	0.00	0.14	0.00	0.00	0.11	0.14	0.00	0.00	0.00	0.00	0.00	0.00	0.19	0.14	0.00	0.00	0.00	0.00	0.00
CCR2	0.00	0.00	0.12	0.12	0.00	0.00	0.00	0.00	0.13	0.00	0.00	0.00	0.00	0.00	0.13	0.13	0.00	0.00	0.00	0.00	0.00
CD1D	0.00	0.00	0.00	0.11	0.00	0.00	0.00	0.13	0.19	0.00	0.00	0.00	0.00	0.00	0.15	0.14	0.00	0.00	0.00	0.00	0.00
ITGAM	0.00	0.00	0.00	0.12	0.00	0.00	0.00	0.12	0.16	0.00	0.00	0.00	0.00	0.00	0.12	0.12	0.00	0.00	0.00	0.00	0.00
IFNAR1	0.00	0.00	0.00	0.12	0.00	0.00	0.00	0.12	0.16	0.00	0.00	0.00	0.00	0.00	0.13	0.14	0.00	0.00	0.00	0.00	0.00
ZBTB11	0.00	0.00	0.00	0.21	0.00	0.47	0.00	0.00	0.00	0.00	0.00	0.18	0.00	0.00	0.22	0.16	0.00	0.00	0.00	0.00	0.00
ANKRD46	0.00	0.00	0.00	0.17	0.00	0.23	0.00	0.00	0.00	0.00	0.00	0.13	0.00	0.00	0.24	0.11	0.00	0.00	0.00	0.00	0.00
HSN1B	0.00	0.00	0.00	0.11	0.00	0.20	0.00	0.00	0.00	0.00	0.00	0.11	0.00	0.00	0.27	0.13	0.00	0.00	0.00	0.00	0.00
HYDIN	0.00	0.00	0.00	0.19	0.00	0.36	0.00	0.00	0.00	0.00	0.00	0.22	0.00	0.00	0.28	0.14	0.00	0.00	0.00	0.00	0.00

PLP1	0.00	0.00	0.00	0.12	0.00	0.00	0.00	0.00	0.00	0.00	0.00	0.14	0.11	0.00	0.30	0.34	0.00	0.00	0.00	0.00	0.00
SAX2	0.00	0.00	0.00	0.00	0.00	0.15	0.00	0.00	0.00	0.00	0.00	0.18	0.14	0.00	0.21	0.16	0.00	0.00	0.00	0.00	0.00
SCAX1	0.00	0.00	0.00	0.00	0.00	0.16	0.00	0.00	0.00	0.00	0.00	0.22	0.16	0.00	0.20	0.12	0.00	0.00	0.00	0.00	0.00
SCA30	0.00	0.00	0.00	0.00	0.00	0.24	0.00	0.00	0.00	0.00	0.00	0.16	0.10	0.00	0.19	0.13	0.00	0.00	0.00	0.00	0.00
OTX2	0.00	0.00	0.00	0.00	0.00	0.00	0.00	0.14	0.00	0.00	0.00	0.20	0.21	0.00	0.30	0.22	0.00	0.00	0.00	0.00	0.00
PAX6	0.00	0.00	0.00	0.00	0.00	0.00	0.00	0.13	0.00	0.00	0.00	0.12	0.18	0.00	0.13	0.13	0.00	0.00	0.00	0.00	0.00
CACNA1A	0.00	0.00	0.00	0.00	0.00	0.00	0.00	0.11	0.00	0.00	0.00	0.00	0.11	0.14	0.12	0.12	0.00	0.00	0.00	0.00	0.00
NCAM1	0.00	0.00	0.00	0.00	0.00	0.00	0.00	0.10	0.00	0.00	0.00	0.00	0.10	0.15	0.12	0.11	0.00	0.00	0.00	0.00	0.00
ALS3	0.00	0.00	0.00	0.00	0.00	0.00	0.00	0.00	0.00	0.00	0.00	0.14	0.12	0.13	0.19	0.17	0.00	0.00	0.00	0.00	0.00
SCAX2	0.00	0.00	0.00	0.00	0.00	0.00	0.00	0.00	0.00	0.00	0.00	0.23	0.21	0.18	0.18	0.19	0.00	0.00	0.00	0.00	0.00
RCHTS	0.00	0.00	0.00	0.00	0.00	0.00	0.00	0.00	0.00	0.00	0.00	0.21	0.15	0.14	0.24	0.17	0.00	0.00	0.00	0.00	0.00
PSORS2	0.00	0.00	0.11	0.20	0.00	0.21	0.15	0.00	0.00	0.00	0.00	0.00	0.00	0.00	0.00	0.00	0.13	0.00	0.00	0.00	0.00
PRSS1	0.00	0.00	0.00	0.13	0.00	0.13	0.00	0.11	0.12	0.00	0.00	0.00	0.00	0.00	0.00	0.00	0.13	0.00	0.00	0.00	0.00
SPINK1	0.00	0.00	0.00	0.14	0.00	0.12	0.00	0.12	0.15	0.00	0.00	0.00	0.00	0.00	0.00	0.00	0.13	0.00	0.00	0.00	0.00
MIR624	0.00	0.00	0.00	0.14	0.00	0.27	0.00	0.27	0.23	0.00	0.00	0.00	0.00	0.00	0.00	0.00	0.12	0.00	0.00	0.00	0.00
ZNF510	0.00	0.00	0.00	0.12	0.00	0.24	0.00	0.10	0.14	0.00	0.00	0.00	0.00	0.00	0.00	0.00	0.11	0.00	0.00	0.00	0.00
IGAN	0.00	0.00	0.00	0.12	0.00	0.17	0.00	0.11	0.18	0.00	0.00	0.00	0.00	0.00	0.00	0.00	0.14	0.00	0.00	0.00	0.00
C10ORF112	0.00	0.00	0.00	0.18	0.00	0.18	0.13	0.00	0.00	0.00	0.00	0.14	0.00	0.00	0.00	0.00	0.13	0.00	0.00	0.00	0.00
METTL19	0.00	0.00	0.00	0.14	0.00	0.22	0.00	0.00	0.17	0.00	0.00	0.11	0.00	0.00	0.00	0.00	0.11	0.00	0.00	0.00	0.00
AD6	0.00	0.00	0.00	0.00	0.00	0.13	0.00	0.00	0.00	0.00	0.00	0.17	0.10	0.14	0.00	0.00	0.10	0.00	0.00	0.00	0.00
TRAJ10	0.00	0.00	0.16	0.19	0.00	0.00	0.20	0.00	0.00	0.00	0.00	0.00	0.00	0.00	0.11	0.00	0.17	0.00	0.00	0.00	0.00
SPG25	0.00	0.00	0.00	0.16	0.00	0.17	0.12	0.00	0.00	0.00	0.00	0.00	0.00	0.00	0.12	0.00	0.11	0.00	0.00	0.00	0.00
SRBD1	0.00	0.00	0.00	0.16	0.00	0.24	0.00	0.14	0.00	0.00	0.00	0.00	0.00	0.00	0.14	0.00	0.14	0.00	0.00	0.00	0.00
ZNF433	0.00	0.00	0.00	0.14	0.00	0.25	0.00	0.10	0.00	0.00	0.00	0.00	0.00	0.00	0.10	0.00	0.19	0.00	0.00	0.00	0.00
BP7	0.00	0.00	0.00	0.14	0.00	0.26	0.00	0.11	0.00	0.00	0.00	0.00	0.00	0.00	0.13	0.00	0.19	0.00	0.00	0.00	0.00
HYT4	0.00	0.00	0.00	0.14	0.00	0.26	0.00	0.11	0.00	0.00	0.00	0.00	0.00	0.00	0.13	0.00	0.19	0.00	0.00	0.00	0.00
PPH2	0.00	0.00	0.00	0.14	0.00	0.19	0.00	0.00	0.11	0.00	0.00	0.00	0.00	0.00	0.10	0.00	0.10	0.00	0.00	0.00	0.00
MOB3B	0.00	0.00	0.00	0.17	0.00	0.22	0.00	0.00	0.11	0.00	0.00	0.00	0.00	0.00	0.10	0.00	0.21	0.00	0.00	0.00	0.00
FGGY	0.00	0.00	0.00	0.10	0.00	0.13	0.00	0.00	0.12	0.00	0.00	0.00	0.00	0.00	0.13	0.00	0.12	0.00	0.00	0.00	0.00
NOX1	0.00	0.00	0.00	0.11	0.00	0.00	0.00	0.13	0.16	0.00	0.00	0.00	0.00	0.00	0.12	0.00	0.10	0.00	0.00	0.00	0.00
NCF1C	0.00	0.00	0.00	0.12	0.00	0.00	0.00	0.11	0.14	0.00	0.00	0.00	0.00	0.00	0.12	0.00	0.11	0.00	0.00	0.00	0.00
ST11	0.00	0.00	0.00	0.00	0.00	0.12	0.00	0.14	0.16	0.00	0.00	0.00	0.00	0.00	0.11	0.00	0.12	0.00	0.00	0.00	0.00
AITD2	0.00	0.00	0.00	0.14	0.00	0.20	0.00	0.00	0.00	0.11	0.00	0.00	0.00	0.00	0.10	0.00	0.16	0.00	0.00	0.00	0.00
AMCN	0.00	0.00	0.00	0.19	0.00	0.31	0.00	0.00	0.00	0.00	0.10	0.00	0.00	0.00	0.11	0.00	0.12	0.00	0.00	0.00	0.00
BP36	0.00	0.00	0.00	0.14	0.00	0.20	0.00	0.00	0.00	0.00	0.00	0.12	0.00	0.00	0.13	0.00	0.20	0.00	0.00	0.00	0.00
PSORS7	0.00	0.00	0.00	0.12	0.00	0.18	0.00	0.00	0.00	0.00	0.00	0.10	0.00	0.00	0.14	0.00	0.15	0.00	0.00	0.00	0.00
BP38	0.00	0.00	0.00	0.14	0.00	0.20	0.00	0.00	0.00	0.00	0.00	0.12	0.00	0.00	0.13	0.00	0.20	0.00	0.00	0.00	0.00
MYMY3	0.00	0.00	0.00	0.14	0.00	0.18	0.00	0.00	0.00	0.00	0.00	0.15	0.00	0.00	0.11	0.00	0.14	0.00	0.00	0.00	0.00
HYT3	0.00	0.00	0.00	0.13	0.00	0.20	0.00	0.00	0.00	0.00	0.00	0.10	0.00	0.00	0.15	0.00	0.15	0.00	0.00	0.00	0.00
ZNF429	0.00	0.00	0.00	0.22	0.00	0.36	0.00	0.00	0.00	0.00	0.00	0.11	0.00	0.00	0.15	0.00	0.12	0.00	0.00	0.00	0.00

COPD25	0.00	0.00	0.00	0.13	0.00	0.20	0.00	0.00	0.00	0.00	0.00	0.13	0.00	0.00	0.13	0.00	0.13	0.00	0.00	0.00	0.00
BP37	0.00	0.00	0.00	0.14	0.00	0.20	0.00	0.00	0.00	0.00	0.00	0.12	0.00	0.00	0.13	0.00	0.20	0.00	0.00	0.00	0.00
LOC400680	0.00	0.00	0.00	0.22	0.00	0.36	0.00	0.00	0.00	0.00	0.00	0.11	0.00	0.00	0.15	0.00	0.12	0.00	0.00	0.00	0.00
BBS10	0.00	0.00	0.00	0.17	0.00	0.28	0.00	0.00	0.00	0.00	0.00	0.13	0.00	0.00	0.16	0.00	0.14	0.00	0.00	0.00	0.00
COPD24	0.00	0.00	0.00	0.13	0.00	0.20	0.00	0.00	0.00	0.00	0.00	0.13	0.00	0.00	0.13	0.00	0.13	0.00	0.00	0.00	0.00
COPD26	0.00	0.00	0.00	0.13	0.00	0.20	0.00	0.00	0.00	0.00	0.00	0.13	0.00	0.00	0.13	0.00	0.13	0.00	0.00	0.00	0.00
MACROD2	0.00	0.00	0.00	0.12	0.00	0.17	0.00	0.00	0.00	0.00	0.00	0.14	0.00	0.00	0.14	0.00	0.14	0.00	0.00	0.00	0.00
BBS7	0.00	0.00	0.00	0.19	0.00	0.32	0.00	0.00	0.00	0.00	0.00	0.13	0.00	0.00	0.16	0.00	0.16	0.00	0.00	0.00	0.00
PEE1	0.00	0.00	0.00	0.12	0.00	0.24	0.00	0.00	0.00	0.00	0.00	0.10	0.00	0.00	0.12	0.00	0.13	0.00	0.00	0.00	0.00
GLM1	0.00	0.00	0.00	0.14	0.00	0.20	0.00	0.00	0.00	0.00	0.00	0.16	0.00	0.00	0.13	0.00	0.12	0.00	0.00	0.00	0.00
CHDS3	0.00	0.00	0.00	0.14	0.00	0.22	0.00	0.00	0.00	0.00	0.00	0.15	0.00	0.00	0.14	0.00	0.12	0.00	0.00	0.00	0.00
ANIB3	0.00	0.00	0.00	0.17	0.00	0.22	0.00	0.00	0.00	0.00	0.00	0.10	0.00	0.00	0.13	0.00	0.11	0.00	0.00	0.00	0.00
HYPLIP2	0.00	0.00	0.00	0.17	0.00	0.28	0.00	0.00	0.00	0.00	0.00	0.12	0.00	0.00	0.12	0.00	0.14	0.00	0.00	0.00	0.00
SLI1	0.00	0.00	0.00	0.13	0.00	0.20	0.00	0.00	0.00	0.00	0.00	0.12	0.00	0.00	0.15	0.00	0.11	0.00	0.00	0.00	0.00
BP39	0.00	0.00	0.00	0.14	0.00	0.20	0.00	0.00	0.00	0.00	0.00	0.12	0.00	0.00	0.13	0.00	0.20	0.00	0.00	0.00	0.00
PGBD1	0.00	0.00	0.00	0.14	0.00	0.24	0.00	0.00	0.00	0.00	0.00	0.12	0.00	0.00	0.14	0.00	0.15	0.00	0.00	0.00	0.00
LGV1	0.00	0.00	0.00	0.19	0.00	0.29	0.00	0.00	0.00	0.00	0.00	0.11	0.00	0.00	0.12	0.00	0.11	0.00	0.00	0.00	0.00
BP35	0.00	0.00	0.00	0.17	0.00	0.25	0.00	0.00	0.00	0.00	0.00	0.13	0.00	0.00	0.15	0.00	0.19	0.00	0.00	0.00	0.00
ARCC1	0.00	0.00	0.00	0.00	0.00	0.14	0.00	0.00	0.00	0.00	0.00	0.12	0.12	0.00	0.12	0.00	0.13	0.00	0.00	0.00	0.00
CCA1	0.00	0.00	0.00	0.00	0.00	0.20	0.00	0.00	0.00	0.00	0.00	0.11	0.12	0.00	0.10	0.00	0.10	0.00	0.00	0.00	0.00
MHW2	0.00	0.00	0.00	0.00	0.00	0.00	0.00	0.00	0.00	0.00	0.00	0.13	0.14	0.11	0.12	0.00	0.12	0.00	0.00	0.00	0.00
MHW1	0.00	0.00	0.00	0.00	0.00	0.00	0.00	0.00	0.00	0.00	0.00	0.13	0.14	0.11	0.12	0.00	0.12	0.00	0.00	0.00	0.00
CRYBA1	0.00	0.00	0.00	0.13	0.00	0.17	0.00	0.10	0.00	0.00	0.00	0.00	0.00	0.00	0.16	0.00	0.00	0.13	0.00	0.00	0.00
ANON	0.00	0.00	0.00	0.14	0.00	0.18	0.00	0.00	0.00	0.00	0.00	0.16	0.00	0.00	0.21	0.00	0.00	0.13	0.00	0.00	0.00
MT1A	0.00	0.00	0.00	0.00	0.00	0.00	0.00	0.00	0.00	0.00	0.00	0.00	0.13	0.14	0.13	0.14	0.00	0.10	0.00	0.00	0.00
R3HCC1	0.00	0.00	0.20	0.21	0.00	0.00	0.24	0.00	0.11	0.00	0.00	0.00	0.00	0.00	0.00	0.00	0.00	0.00	0.13	0.00	0.00
FEN1	0.00	0.00	0.00	0.11	0.00	0.16	0.00	0.13	0.10	0.00	0.00	0.00	0.00	0.00	0.00	0.00	0.00	0.00	0.11	0.00	0.00
LOX	0.00	0.00	0.00	0.13	0.00	0.14	0.00	0.17	0.21	0.00	0.00	0.00	0.00	0.00	0.00	0.00	0.00	0.00	0.12	0.00	0.00
ABCB4	0.00	0.00	0.00	0.14	0.00	0.12	0.00	0.15	0.17	0.00	0.00	0.00	0.00	0.00	0.00	0.00	0.00	0.00	0.18	0.00	0.00
HLN2	0.00	0.00	0.00	0.15	0.00	0.14	0.11	0.00	0.00	0.00	0.00	0.12	0.00	0.00	0.00	0.00	0.00	0.00	0.12	0.00	0.00
APOF	0.00	0.00	0.00	0.19	0.00	0.26	0.00	0.13	0.00	0.00	0.00	0.13	0.00	0.00	0.00	0.00	0.00	0.00	0.25	0.00	0.00
CEPT1	0.00	0.00	0.00	0.14	0.00	0.28	0.00	0.19	0.00	0.00	0.00	0.10	0.00	0.00	0.00	0.00	0.00	0.00	0.34	0.00	0.00
MTF1	0.00	0.00	0.00	0.19	0.00	0.24	0.00	0.28	0.00	0.00	0.00	0.00	0.00	0.00	0.17	0.00	0.00	0.00	0.14	0.00	0.00
DHX30	0.00	0.00	0.00	0.22	0.00	0.42	0.00	0.32	0.00	0.00	0.00	0.00	0.00	0.00	0.14	0.00	0.00	0.00	0.20	0.00	0.00
GAR1	0.00	0.00	0.00	0.16	0.00	0.36	0.00	0.11	0.00	0.00	0.00	0.00	0.00	0.00	0.14	0.00	0.00	0.00	0.12	0.00	0.00
SRSF4	0.00	0.00	0.00	0.23	0.00	0.38	0.00	0.22	0.00	0.00	0.00	0.00	0.00	0.00	0.13	0.00	0.00	0.00	0.11	0.00	0.00
ABCA4	0.00	0.00	0.00	0.12	0.00	0.14	0.00	0.00	0.17	0.00	0.00	0.00	0.00	0.00	0.11	0.00	0.00	0.00	0.12	0.00	0.00
HAMP	0.00	0.00	0.00	0.15	0.00	0.00	0.12	0.00	0.15	0.00	0.00	0.00	0.00	0.00	0.13	0.00	0.00	0.00	0.17	0.00	0.00
SRD5A2	0.00	0.00	0.00	0.12	0.00	0.00	0.00	0.12	0.12	0.00	0.00	0.00	0.00	0.00	0.13	0.00	0.00	0.00	0.18	0.00	0.00
FDPSP2	0.00	0.00	0.00	0.18	0.00	0.42	0.00	0.00	0.00	0.00	0.00	0.21	0.00	0.00	0.11	0.00	0.00	0.00	0.27	0.00	0.00

ARSC2	0.00	0.00	0.00	0.21	0.00	0.39	0.00	0.00	0.00	0.00	0.00	0.22	0.00	0.00	0.14	0.00	0.00	0.00	0.20	0.00	0.00
NUDT4P1	0.00	0.00	0.00	0.25	0.00	0.46	0.00	0.00	0.00	0.00	0.00	0.19	0.00	0.00	0.10	0.00	0.00	0.00	0.18	0.00	0.00
ACRC	0.00	0.00	0.00	0.24	0.00	0.50	0.00	0.00	0.00	0.00	0.00	0.25	0.00	0.00	0.16	0.00	0.00	0.00	0.16	0.00	0.00
MT4	0.00	0.00	0.00	0.18	0.00	0.27	0.00	0.00	0.00	0.00	0.00	0.12	0.00	0.00	0.16	0.00	0.00	0.00	0.11	0.00	0.00
HGD	0.00	0.00	0.00	0.17	0.00	0.28	0.00	0.00	0.00	0.00	0.00	0.13	0.00	0.00	0.15	0.00	0.00	0.00	0.18	0.00	0.00
MTX1P1	0.00	0.00	0.00	0.22	0.00	0.45	0.00	0.00	0.00	0.00	0.00	0.17	0.00	0.00	0.20	0.00	0.00	0.00	0.11	0.00	0.00
FDPSP3	0.00	0.00	0.00	0.18	0.00	0.42	0.00	0.00	0.00	0.00	0.00	0.21	0.00	0.00	0.11	0.00	0.00	0.00	0.27	0.00	0.00
FTH1P13	0.00	0.00	0.00	0.22	0.00	0.37	0.00	0.00	0.00	0.00	0.00	0.13	0.00	0.00	0.13	0.00	0.00	0.00	0.12	0.00	0.00
FDPSP1	0.00	0.00	0.00	0.18	0.00	0.42	0.00	0.00	0.00	0.00	0.00	0.21	0.00	0.00	0.11	0.00	0.00	0.00	0.27	0.00	0.00
AMMECR1	0.00	0.00	0.00	0.25	0.00	0.57	0.00	0.00	0.00	0.00	0.00	0.32	0.00	0.00	0.19	0.00	0.00	0.00	0.17	0.00	0.00
FDPSL2A	0.00	0.00	0.00	0.18	0.00	0.42	0.00	0.00	0.00	0.00	0.00	0.21	0.00	0.00	0.11	0.00	0.00	0.00	0.27	0.00	0.00
FDPSP4	0.00	0.00	0.00	0.18	0.00	0.42	0.00	0.00	0.00	0.00	0.00	0.21	0.00	0.00	0.11	0.00	0.00	0.00	0.27	0.00	0.00
FDPSP5	0.00	0.00	0.00	0.18	0.00	0.42	0.00	0.00	0.00	0.00	0.00	0.21	0.00	0.00	0.11	0.00	0.00	0.00	0.27	0.00	0.00
RAB7L1	0.00	0.00	0.00	0.23	0.00	0.39	0.00	0.00	0.00	0.00	0.00	0.20	0.00	0.00	0.14	0.00	0.00	0.00	0.15	0.00	0.00
ATXN3L	0.00	0.00	0.00	0.19	0.00	0.47	0.00	0.00	0.00	0.00	0.00	0.25	0.00	0.00	0.17	0.00	0.00	0.00	0.13	0.00	0.00
ALG11	0.00	0.00	0.00	0.18	0.00	0.42	0.00	0.00	0.00	0.00	0.00	0.18	0.00	0.00	0.15	0.00	0.00	0.00	0.14	0.00	0.00
DGCR7	0.00	0.00	0.00	0.20	0.00	0.42	0.00	0.00	0.00	0.00	0.00	0.23	0.00	0.00	0.14	0.00	0.00	0.00	0.17	0.00	0.00
ARSE	0.00	0.00	0.00	0.21	0.00	0.42	0.00	0.00	0.00	0.00	0.00	0.16	0.00	0.00	0.15	0.00	0.00	0.00	0.11	0.00	0.00
GALNS	0.00	0.00	0.00	0.00	0.00	0.18	0.00	0.00	0.17	0.00	0.00	0.11	0.00	0.00	0.15	0.00	0.00	0.00	0.16	0.00	0.00
MAOA	0.00	0.00	0.00	0.00	0.00	0.00	0.00	0.00	0.00	0.00	0.00	0.10	0.16	0.12	0.13	0.00	0.00	0.00	0.12	0.00	0.00
AASTH42	0.00	0.00	0.00	0.15	0.00	0.16	0.11	0.00	0.00	0.00	0.00	0.00	0.00	0.00	0.00	0.00	0.11	0.00	0.15	0.00	0.00
BP33	0.00	0.00	0.00	0.15	0.00	0.16	0.11	0.00	0.00	0.00	0.00	0.00	0.00	0.00	0.00	0.00	0.11	0.00	0.15	0.00	0.00
LOC100129278	0.00	0.00	0.00	0.15	0.00	0.15	0.00	0.12	0.00	0.00	0.00	0.00	0.00	0.00	0.00	0.00	0.19	0.00	0.21	0.00	0.00
CYP39A1	0.00	0.00	0.00	0.13	0.00	0.19	0.00	0.00	0.00	0.00	0.00	0.14	0.00	0.00	0.00	0.00	0.11	0.00	0.36	0.00	0.00
ALAD	0.00	0.00	0.00	0.00	0.00	0.00	0.00	0.00	0.12	0.00	0.00	0.11	0.00	0.00	0.11	0.00	0.12	0.00	0.22	0.00	0.00
GPX1	0.00	0.00	0.00	0.00	0.00	0.00	0.00	0.00	0.00	0.00	0.00	0.00	0.10	0.12	0.12	0.00	0.15	0.00	0.00	0.12	0.00
BW12	0.00	0.00	0.00	0.00	0.00	0.17	0.00	0.00	0.00	0.00	0.00	0.11	0.00	0.00	0.00	0.00	0.22	0.14	0.00	0.15	0.00
MRXS7	0.00	0.00	0.00	0.00	0.00	0.14	0.00	0.00	0.00	0.00	0.00	0.16	0.00	0.00	0.00	0.00	0.14	0.12	0.00	0.14	0.00
BW13	0.00	0.00	0.00	0.00	0.00	0.17	0.00	0.00	0.00	0.00	0.00	0.11	0.00	0.00	0.00	0.00	0.22	0.14	0.00	0.15	0.00
BW14	0.00	0.00	0.00	0.00	0.00	0.17	0.00	0.00	0.00	0.00	0.00	0.11	0.00	0.00	0.00	0.00	0.22	0.14	0.00	0.15	0.00
OSBPL10	0.00	0.00	0.00	0.14	0.00	0.16	0.00	0.00	0.00	0.00	0.00	0.00	0.00	0.00	0.00	0.00	0.22	0.00	0.26	0.10	0.00
GBD2	0.00	0.00	0.00	0.11	0.00	0.16	0.00	0.00	0.00	0.00	0.00	0.00	0.00	0.00	0.00	0.00	0.36	0.00	0.16	0.24	0.00
GBD3	0.00	0.00	0.00	0.11	0.00	0.16	0.00	0.00	0.00	0.00	0.00	0.00	0.00	0.00	0.00	0.00	0.36	0.00	0.16	0.24	0.00
PON2	0.00	0.00	0.00	0.11	0.00	0.11	0.00	0.00	0.00	0.00	0.00	0.00	0.00	0.00	0.00	0.00	0.15	0.00	0.14	0.10	0.00
TTC39B	0.00	0.00	0.00	0.11	0.00	0.00	0.00	0.00	0.11	0.00	0.00	0.00	0.00	0.00	0.00	0.00	0.22	0.00	0.29	0.11	0.00
PHKA1P1	0.00	0.00	0.00	0.00	0.00	0.28	0.00	0.00	0.00	0.00	0.00	0.14	0.00	0.00	0.00	0.00	0.00	0.13	0.14	0.15	0.00
PHKG1P3	0.00	0.00	0.00	0.00	0.00	0.28	0.00	0.00	0.00	0.00	0.00	0.14	0.00	0.00	0.00	0.00	0.00	0.13	0.14	0.15	0.00
PHKG1P1	0.00	0.00	0.00	0.00	0.00	0.28	0.00	0.00	0.00	0.00	0.00	0.14	0.00	0.00	0.00	0.00	0.00	0.13	0.14	0.15	0.00
PHKG1P2	0.00	0.00	0.00	0.00	0.00	0.28	0.00	0.00	0.00	0.00	0.00	0.14	0.00	0.00	0.00	0.00	0.00	0.13	0.14	0.15	0.00
POLR3B	0.00	0.00	0.00	0.22	0.00	0.39	0.00	0.20	0.00	0.00	0.00	0.00	0.00	0.00	0.18	0.00	0.00	0.00	0.00	0.00	0.14

XIAP	0.00	0.00	0.00	0.10	0.00	0.00	0.00	0.17	0.25	0.00	0.00	0.00	0.00	0.00	0.11	0.00	0.00	0.00	0.00	0.00	0.11
NDUFB3	0.00	0.00	0.00	0.20	0.00	0.43	0.00	0.00	0.00	0.00	0.00	0.18	0.00	0.00	0.16	0.00	0.00	0.00	0.00	0.00	0.26
DNALI1	0.00	0.00	0.00	0.24	0.00	0.44	0.00	0.00	0.00	0.00	0.00	0.16	0.00	0.00	0.12	0.00	0.00	0.00	0.00	0.00	0.12
DCTN5	0.00	0.00	0.00	0.15	0.00	0.28	0.00	0.00	0.00	0.00	0.00	0.11	0.00	0.00	0.12	0.00	0.00	0.00	0.00	0.00	0.10
DCTN3	0.00	0.00	0.00	0.22	0.00	0.43	0.00	0.00	0.00	0.00	0.00	0.18	0.00	0.00	0.16	0.00	0.00	0.00	0.00	0.00	0.14
DNAI2	0.00	0.00	0.00	0.20	0.00	0.39	0.00	0.00	0.00	0.00	0.00	0.12	0.00	0.00	0.12	0.00	0.00	0.00	0.00	0.00	0.10
MTO1	0.00	0.00	0.00	0.16	0.00	0.38	0.00	0.10	0.00	0.00	0.00	0.00	0.00	0.00	0.00	0.00	0.00	0.00	0.11	0.00	0.29
HOGA1	0.00	0.00	0.00	0.17	0.00	0.24	0.00	0.00	0.12	0.00	0.00	0.00	0.00	0.00	0.00	0.00	0.00	0.00	0.23	0.00	0.12
TMEM14C	0.00	0.00	0.00	0.11	0.00	0.32	0.00	0.00	0.00	0.00	0.00	0.14	0.00	0.00	0.00	0.00	0.00	0.00	0.24	0.00	0.47
NTHL1	0.00	0.00	0.00	0.00	0.00	0.21	0.00	0.10	0.00	0.00	0.00	0.11	0.00	0.00	0.00	0.00	0.00	0.00	0.11	0.00	0.17
SDHD	0.00	0.00	0.00	0.00	0.00	0.13	0.00	0.00	0.10	0.00	0.00	0.00	0.00	0.00	0.14	0.00	0.00	0.00	0.13	0.00	0.17
C12ORF65	0.00	0.00	0.00	0.12	0.00	0.36	0.00	0.00	0.00	0.00	0.00	0.00	0.00	0.00	0.00	0.00	0.00	0.17	0.22	0.00	0.49
COX16	0.00	0.00	0.00	0.10	0.00	0.37	0.00	0.00	0.00	0.00	0.00	0.00	0.00	0.00	0.00	0.00	0.00	0.15	0.19	0.00	0.50
HMGCLL1	0.00	0.00	0.00	0.00	0.00	0.17	0.00	0.00	0.00	0.00	0.00	0.13	0.00	0.00	0.00	0.00	0.00	0.20	0.35	0.00	0.29
RARS2	0.00	0.00	0.00	0.00	0.00	0.26	0.00	0.00	0.00	0.00	0.00	0.15	0.00	0.00	0.00	0.00	0.00	0.20	0.24	0.00	0.54
MTFMT	0.00	0.00	0.00	0.00	0.00	0.35	0.00	0.00	0.00	0.00	0.00	0.14	0.00	0.00	0.00	0.00	0.00	0.23	0.30	0.00	0.60
COX3	0.00	0.00	0.00	0.00	0.00	0.26	0.00	0.00	0.00	0.00	0.00	0.11	0.00	0.00	0.00	0.00	0.00	0.18	0.21	0.00	0.47
AARS2	0.00	0.00	0.00	0.00	0.00	0.23	0.00	0.00	0.00	0.00	0.00	0.00	0.00	0.00	0.11	0.00	0.00	0.17	0.18	0.00	0.42
TTC19	0.00	0.00	0.00	0.00	0.00	0.23	0.00	0.00	0.00	0.00	0.00	0.00	0.00	0.00	0.13	0.00	0.00	0.18	0.17	0.00	0.49
TRNW	0.00	0.00	0.00	0.00	0.00	0.25	0.00	0.00	0.00	0.00	0.00	0.00	0.00	0.00	0.00	0.00	0.11	0.18	0.11	0.00	0.42
TRNG	0.00	0.00	0.00	0.00	0.00	0.15	0.00	0.00	0.00	0.00	0.00	0.00	0.00	0.00	0.00	0.00	0.14	0.11	0.12	0.00	0.33
ND6	0.00	0.00	0.00	0.00	0.00	0.22	0.00	0.00	0.00	0.00	0.00	0.00	0.00	0.00	0.00	0.00	0.14	0.13	0.11	0.00	0.34
TRNK	0.00	0.00	0.00	0.00	0.00	0.15	0.00	0.00	0.00	0.00	0.00	0.00	0.00	0.00	0.00	0.00	0.12	0.19	0.14	0.00	0.40
TRNS2	0.00	0.00	0.00	0.00	0.00	0.00	0.00	0.00	0.00	0.00	0.00	0.00	0.00	0.00	0.00	0.00	0.59	0.27	0.24	0.45	0.40
CRSA	0.00	0.00	0.11	0.23	0.00	0.24	0.18	0.00	0.00	0.00	0.00	0.00	0.00	0.00	0.00	0.00	0.00	0.00	0.00	0.00	0.00
ODPF	0.00	0.00	0.13	0.21	0.00	0.21	0.15	0.00	0.00	0.00	0.00	0.00	0.00	0.00	0.00	0.00	0.00	0.00	0.00	0.00	0.00
TDRD3	0.00	0.00	0.00	0.26	0.00	0.43	0.11	0.36	0.00	0.00	0.00	0.00	0.00	0.00	0.00	0.00	0.00	0.00	0.00	0.00	0.00
CCR5	0.10	0.10	0.00	0.00	0.12	0.00	0.00	0.00	0.11	0.00	0.00	0.00	0.00	0.00	0.00	0.00	0.00	0.00	0.00	0.00	0.00
RAF1	0.00	0.00	0.11	0.13	0.00	0.00	0.00	0.11	0.15	0.00	0.00	0.00	0.00	0.00	0.00	0.00	0.00	0.00	0.00	0.00	0.00
HEPACAM2	0.00	0.00	0.00	0.11	0.00	0.25	0.00	0.16	0.11	0.00	0.00	0.00	0.00	0.00	0.00	0.00	0.00	0.00	0.00	0.00	0.00
HPC4	0.00	0.00	0.00	0.12	0.00	0.16	0.00	0.11	0.15	0.00	0.00	0.00	0.00	0.00	0.00	0.00	0.00	0.00	0.00	0.00	0.00
BRCA3	0.00	0.00	0.00	0.16	0.00	0.23	0.00	0.15	0.12	0.00	0.00	0.00	0.00	0.00	0.00	0.00	0.00	0.00	0.00	0.00	0.00
MIR297	0.00	0.00	0.00	0.13	0.00	0.19	0.00	0.40	0.16	0.00	0.00	0.00	0.00	0.00	0.00	0.00	0.00	0.00	0.00	0.00	0.00
BDMF	0.00	0.00	0.00	0.11	0.00	0.23	0.00	0.18	0.12	0.00	0.00	0.00	0.00	0.00	0.00	0.00	0.00	0.00	0.00	0.00	0.00
TGFBI	0.00	0.00	0.00	0.13	0.00	0.14	0.00	0.14	0.20	0.00	0.00	0.00	0.00	0.00	0.00	0.00	0.00	0.00	0.00	0.00	0.00
HPFH2	0.00	0.00	0.00	0.17	0.00	0.24	0.00	0.14	0.16	0.00	0.00	0.00	0.00	0.00	0.00	0.00	0.00	0.00	0.00	0.00	0.00
IGHJ@	0.00	0.00	0.00	0.10	0.00	0.24	0.00	0.14	0.12	0.00	0.00	0.00	0.00	0.00	0.00	0.00	0.00	0.00	0.00	0.00	0.00
TAPBPL	0.00	0.00	0.00	0.16	0.00	0.26	0.00	0.10	0.00	0.00	0.18	0.00	0.00	0.00	0.00	0.00	0.00	0.00	0.00	0.00	0.00
SAA3P	0.00	0.00	0.00	0.27	0.00	0.43	0.12	0.00	0.00	0.00	0.00	0.17	0.00	0.00	0.00	0.00	0.00	0.00	0.00	0.00	0.00
UBE2L1	0.00	0.00	0.00	0.18	0.00	0.41	0.00	0.28	0.00	0.00	0.00	0.13	0.00	0.00	0.00	0.00	0.00	0.00	0.00	0.00	0.00

FMR3	0.00	0.00	0.00	0.18	0.00	0.37	0.00	0.12	0.00	0.00	0.00	0.17	0.00	0.00	0.00	0.00	0.00	0.00	0.00	0.00	0.00
DIP2B	0.00	0.00	0.00	0.20	0.00	0.40	0.00	0.11	0.00	0.00	0.00	0.15	0.00	0.00	0.00	0.00	0.00	0.00	0.00	0.00	0.00
CGGBP1	0.00	0.00	0.00	0.20	0.00	0.38	0.00	0.21	0.00	0.00	0.00	0.10	0.00	0.00	0.00	0.00	0.00	0.00	0.00	0.00	0.00
HSPB1P2	0.00	0.00	0.00	0.19	0.00	0.34	0.00	0.11	0.00	0.00	0.00	0.10	0.00	0.00	0.00	0.00	0.00	0.00	0.00	0.00	0.00
ZNF189	0.00	0.00	0.00	0.20	0.00	0.52	0.00	0.17	0.00	0.00	0.00	0.12	0.00	0.00	0.00	0.00	0.00	0.00	0.00	0.00	0.00
THCYTX	0.00	0.00	0.00	0.18	0.00	0.30	0.00	0.20	0.00	0.00	0.00	0.12	0.00	0.00	0.00	0.00	0.00	0.00	0.00	0.00	0.00
DIS3L2	0.00	0.00	0.00	0.28	0.00	0.54	0.00	0.31	0.00	0.00	0.00	0.11	0.00	0.00	0.00	0.00	0.00	0.00	0.00	0.00	0.00
ZMYM5	0.00	0.00	0.00	0.21	0.00	0.41	0.00	0.26	0.00	0.00	0.00	0.12	0.00	0.00	0.00	0.00	0.00	0.00	0.00	0.00	0.00
AD9	0.00	0.00	0.00	0.12	0.00	0.22	0.00	0.00	0.13	0.00	0.00	0.16	0.00	0.00	0.00	0.00	0.00	0.00	0.00	0.00	0.00
PSNP2	0.00	0.00	0.00	0.11	0.00	0.20	0.00	0.00	0.13	0.00	0.00	0.10	0.00	0.00	0.00	0.00	0.00	0.00	0.00	0.00	0.00
EXTL2P1	0.00	0.00	0.00	0.24	0.00	0.52	0.00	0.00	0.00	0.00	0.15	0.12	0.00	0.00	0.00	0.00	0.00	0.00	0.00	0.00	0.00
ADORA2A	0.00	0.00	0.12	0.13	0.00	0.00	0.00	0.00	0.15	0.00	0.00	0.00	0.10	0.00	0.00	0.00	0.00	0.00	0.00	0.00	0.00
MRX37	0.00	0.00	0.00	0.14	0.00	0.33	0.00	0.00	0.00	0.00	0.00	0.23	0.11	0.00	0.00	0.00	0.00	0.00	0.00	0.00	0.00
MRX51	0.00	0.00	0.00	0.11	0.00	0.29	0.00	0.00	0.00	0.00	0.00	0.21	0.12	0.00	0.00	0.00	0.00	0.00	0.00	0.00	0.00
MRX23	0.00	0.00	0.00	0.12	0.00	0.28	0.00	0.00	0.00	0.00	0.00	0.22	0.12	0.00	0.00	0.00	0.00	0.00	0.00	0.00	0.00
MRX24	0.00	0.00	0.00	0.13	0.00	0.29	0.00	0.00	0.00	0.00	0.00	0.19	0.11	0.00	0.00	0.00	0.00	0.00	0.00	0.00	0.00
PSEN1	0.00	0.00	0.00	0.00	0.00	0.00	0.00	0.00	0.14	0.00	0.12	0.00	0.13	0.13	0.00	0.00	0.00	0.00	0.00	0.00	0.00
MRX25	0.00	0.00	0.00	0.00	0.00	0.19	0.00	0.00	0.00	0.00	0.00	0.21	0.12	0.10	0.00	0.00	0.00	0.00	0.00	0.00	0.00
EIM	0.00	0.00	0.00	0.00	0.00	0.11	0.00	0.00	0.00	0.00	0.00	0.11	0.10	0.14	0.00	0.00	0.00	0.00	0.00	0.00	0.00
MRX39	0.00	0.00	0.00	0.00	0.00	0.15	0.00	0.00	0.00	0.00	0.00	0.20	0.14	0.10	0.00	0.00	0.00	0.00	0.00	0.00	0.00
FCMTE1	0.00	0.00	0.00	0.00	0.00	0.17	0.00	0.00	0.00	0.00	0.00	0.12	0.10	0.14	0.00	0.00	0.00	0.00	0.00	0.00	0.00
MRX49	0.00	0.00	0.00	0.00	0.00	0.20	0.00	0.00	0.00	0.00	0.00	0.18	0.13	0.11	0.00	0.00	0.00	0.00	0.00	0.00	0.00
MRX40	0.00	0.00	0.00	0.00	0.00	0.23	0.00	0.00	0.00	0.00	0.00	0.22	0.11	0.11	0.00	0.00	0.00	0.00	0.00	0.00	0.00
FEB-07	0.00	0.00	0.00	0.00	0.00	0.19	0.00	0.00	0.00	0.00	0.00	0.16	0.10	0.17	0.00	0.00	0.00	0.00	0.00	0.00	0.00
TARM1	0.00	0.00	0.10	0.19	0.00	0.26	0.00	0.00	0.00	0.00	0.00	0.00	0.00	0.00	0.11	0.00	0.00	0.00	0.00	0.00	0.00
HPLH1	0.00	0.00	0.00	0.17	0.00	0.19	0.12	0.00	0.00	0.00	0.00	0.00	0.00	0.00	0.14	0.00	0.00	0.00	0.00	0.00	0.00
RD3	0.00	0.00	0.00	0.21	0.00	0.31	0.11	0.00	0.00	0.00	0.00	0.00	0.00	0.00	0.15	0.00	0.00	0.00	0.00	0.00	0.00
LMBR1	0.00	0.00	0.00	0.18	0.00	0.26	0.00	0.14	0.00	0.00	0.00	0.00	0.00	0.00	0.18	0.00	0.00	0.00	0.00	0.00	0.00
SUPT3H	0.00	0.00	0.00	0.25	0.00	0.45	0.00	0.34	0.00	0.00	0.00	0.00	0.00	0.00	0.14	0.00	0.00	0.00	0.00	0.00	0.00
KLHL9	0.00	0.00	0.00	0.16	0.00	0.34	0.00	0.16	0.00	0.00	0.00	0.00	0.00	0.00	0.17	0.00	0.00	0.00	0.00	0.00	0.00
BANF2	0.00	0.00	0.00	0.20	0.00	0.39	0.00	0.21	0.00	0.00	0.00	0.00	0.00	0.00	0.11	0.00	0.00	0.00	0.00	0.00	0.00
RRN3	0.00	0.00	0.00	0.24	0.00	0.39	0.00	0.36	0.00	0.00	0.00	0.00	0.00	0.00	0.17	0.00	0.00	0.00	0.00	0.00	0.00
COL4A4	0.00	0.00	0.00	0.12	0.00	0.12	0.00	0.11	0.00	0.00	0.00	0.00	0.00	0.00	0.10	0.00	0.00	0.00	0.00	0.00	0.00
RPS17	0.00	0.00	0.00	0.24	0.00	0.45	0.00	0.16	0.00	0.00	0.00	0.00	0.00	0.00	0.14	0.00	0.00	0.00	0.00	0.00	0.00
ESCO2	0.00	0.00	0.00	0.15	0.00	0.32	0.00	0.25	0.00	0.00	0.00	0.00	0.00	0.00	0.15	0.00	0.00	0.00	0.00	0.00	0.00
ZNF750	0.00	0.00	0.00	0.16	0.00	0.31	0.00	0.13	0.00	0.00	0.00	0.00	0.00	0.00	0.10	0.00	0.00	0.00	0.00	0.00	0.00
TSPYL1	0.00	0.00	0.00	0.21	0.00	0.35	0.00	0.15	0.00	0.00	0.00	0.00	0.00	0.00	0.12	0.00	0.00	0.00	0.00	0.00	0.00
L1RE4	0.00	0.00	0.00	0.11	0.00	0.32	0.00	0.11	0.00	0.00	0.00	0.00	0.00	0.00	0.11	0.00	0.00	0.00	0.00	0.00	0.00
TADA1	0.00	0.00	0.00	0.24	0.00	0.45	0.00	0.23	0.00	0.00	0.00	0.00	0.00	0.00	0.12	0.00	0.00	0.00	0.00	0.00	0.00
TAF12	0.00	0.00	0.00	0.18	0.00	0.29	0.00	0.23	0.00	0.00	0.00	0.00	0.00	0.00	0.13	0.00	0.00	0.00	0.00	0.00	0.00

PABPC1P2	0.00	0.00	0.00	0.18	0.00	0.41	0.00	0.11	0.00	0.00	0.00	0.00	0.00	0.00	0.12	0.00	0.00	0.00	0.00	0.00	0.00
INF2	0.00	0.00	0.00	0.23	0.00	0.41	0.00	0.19	0.00	0.00	0.00	0.00	0.00	0.00	0.17	0.00	0.00	0.00	0.00	0.00	0.00
ATXN7L1	0.00	0.00	0.00	0.18	0.00	0.40	0.00	0.18	0.00	0.00	0.00	0.00	0.00	0.00	0.11	0.00	0.00	0.00	0.00	0.00	0.00
SNRPB2	0.00	0.00	0.00	0.22	0.00	0.42	0.00	0.14	0.00	0.00	0.00	0.00	0.00	0.00	0.19	0.00	0.00	0.00	0.00	0.00	0.00
POLR3A	0.00	0.00	0.00	0.18	0.00	0.33	0.00	0.21	0.00	0.00	0.00	0.00	0.00	0.00	0.19	0.00	0.00	0.00	0.00	0.00	0.00
NCAPD2	0.00	0.00	0.00	0.23	0.00	0.41	0.00	0.21	0.00	0.00	0.00	0.00	0.00	0.00	0.12	0.00	0.00	0.00	0.00	0.00	0.00
HPX-2	0.00	0.00	0.00	0.22	0.00	0.41	0.00	0.20	0.00	0.00	0.00	0.00	0.00	0.00	0.17	0.00	0.00	0.00	0.00	0.00	0.00
MSX2P1	0.00	0.00	0.00	0.22	0.00	0.41	0.00	0.20	0.00	0.00	0.00	0.00	0.00	0.00	0.17	0.00	0.00	0.00	0.00	0.00	0.00
CIRH1A	0.00	0.00	0.00	0.21	0.00	0.41	0.00	0.15	0.00	0.00	0.00	0.00	0.00	0.00	0.13	0.00	0.00	0.00	0.00	0.00	0.00
GEMIN6	0.00	0.00	0.00	0.15	0.00	0.28	0.00	0.12	0.00	0.00	0.00	0.00	0.00	0.00	0.18	0.00	0.00	0.00	0.00	0.00	0.00
GEMIN8	0.00	0.00	0.00	0.17	0.00	0.33	0.00	0.14	0.00	0.00	0.00	0.00	0.00	0.00	0.14	0.00	0.00	0.00	0.00	0.00	0.00
PHF2	0.00	0.00	0.00	0.21	0.00	0.33	0.00	0.30	0.00	0.00	0.00	0.00	0.00	0.00	0.14	0.00	0.00	0.00	0.00	0.00	0.00
KANSL1	0.00	0.00	0.00	0.24	0.00	0.40	0.00	0.32	0.00	0.00	0.00	0.00	0.00	0.00	0.11	0.00	0.00	0.00	0.00	0.00	0.00
FBXW4	0.00	0.00	0.00	0.19	0.00	0.28	0.00	0.20	0.00	0.00	0.00	0.00	0.00	0.00	0.17	0.00	0.00	0.00	0.00	0.00	0.00
SNRPD1	0.00	0.00	0.00	0.18	0.00	0.34	0.00	0.14	0.00	0.00	0.00	0.00	0.00	0.00	0.14	0.00	0.00	0.00	0.00	0.00	0.00
MPLKIP	0.00	0.00	0.00	0.14	0.00	0.34	0.00	0.13	0.00	0.00	0.00	0.00	0.00	0.00	0.14	0.00	0.00	0.00	0.00	0.00	0.00
TAF1	0.00	0.00	0.00	0.15	0.00	0.17	0.00	0.22	0.00	0.00	0.00	0.00	0.00	0.00	0.13	0.00	0.00	0.00	0.00	0.00	0.00
BRIP1	0.00	0.00	0.00	0.12	0.00	0.19	0.00	0.16	0.00	0.00	0.00	0.00	0.00	0.00	0.10	0.00	0.00	0.00	0.00	0.00	0.00
LOC442131	0.00	0.00	0.00	0.22	0.00	0.40	0.00	0.15	0.00	0.00	0.00	0.00	0.00	0.00	0.14	0.00	0.00	0.00	0.00	0.00	0.00
SNRPD2	0.00	0.00	0.00	0.16	0.00	0.33	0.00	0.10	0.00	0.00	0.00	0.00	0.00	0.00	0.15	0.00	0.00	0.00	0.00	0.00	0.00
NIPBL	0.00	0.00	0.00	0.14	0.00	0.29	0.00	0.16	0.00	0.00	0.00	0.00	0.00	0.00	0.18	0.00	0.00	0.00	0.00	0.00	0.00
ATXN7L3	0.00	0.00	0.00	0.23	0.00	0.43	0.00	0.31	0.00	0.00	0.00	0.00	0.00	0.00	0.12	0.00	0.00	0.00	0.00	0.00	0.00
PMS1	0.00	0.00	0.00	0.15	0.00	0.22	0.00	0.15	0.00	0.00	0.00	0.00	0.00	0.00	0.11	0.00	0.00	0.00	0.00	0.00	0.00
SMNDC1	0.00	0.00	0.00	0.22	0.00	0.37	0.00	0.20	0.00	0.00	0.00	0.00	0.00	0.00	0.15	0.00	0.00	0.00	0.00	0.00	0.00
CHM	0.00	0.00	0.00	0.17	0.00	0.26	0.00	0.13	0.00	0.00	0.00	0.00	0.00	0.00	0.12	0.00	0.00	0.00	0.00	0.00	0.00
SNRPB	0.00	0.00	0.00	0.18	0.00	0.32	0.00	0.14	0.00	0.00	0.00	0.00	0.00	0.00	0.15	0.00	0.00	0.00	0.00	0.00	0.00
GEMIN7	0.00	0.00	0.00	0.16	0.00	0.29	0.00	0.15	0.00	0.00	0.00	0.00	0.00	0.00	0.16	0.00	0.00	0.00	0.00	0.00	0.00
HIRA	0.00	0.00	0.00	0.22	0.00	0.37	0.00	0.32	0.00	0.00	0.00	0.00	0.00	0.00	0.17	0.00	0.00	0.00	0.00	0.00	0.00
ERCC8	0.00	0.00	0.00	0.16	0.00	0.19	0.00	0.13	0.00	0.00	0.00	0.00	0.00	0.00	0.17	0.00	0.00	0.00	0.00	0.00	0.00
FAM160B2	0.00	0.00	0.00	0.17	0.00	0.35	0.00	0.13	0.00	0.00	0.00	0.00	0.00	0.00	0.15	0.00	0.00	0.00	0.00	0.00	0.00
TARBP1	0.00	0.00	0.00	0.20	0.00	0.36	0.00	0.34	0.00	0.00	0.00	0.00	0.00	0.00	0.13	0.00	0.00	0.00	0.00	0.00	0.00
MYH1	0.00	0.00	0.00	0.12	0.00	0.16	0.00	0.12	0.00	0.00	0.00	0.00	0.00	0.00	0.14	0.00	0.00	0.00	0.00	0.00	0.00
TAF13	0.00	0.00	0.00	0.14	0.00	0.29	0.00	0.14	0.00	0.00	0.00	0.00	0.00	0.00	0.10	0.00	0.00	0.00	0.00	0.00	0.00
SUPT5H	0.00	0.00	0.00	0.19	0.00	0.27	0.00	0.34	0.00	0.00	0.00	0.00	0.00	0.00	0.18	0.00	0.00	0.00	0.00	0.00	0.00
SNRPF	0.00	0.00	0.00	0.16	0.00	0.32	0.00	0.10	0.00	0.00	0.00	0.00	0.00	0.00	0.14	0.00	0.00	0.00	0.00	0.00	0.00
MLH3	0.00	0.00	0.00	0.12	0.00	0.21	0.00	0.14	0.00	0.00	0.00	0.00	0.00	0.00	0.11	0.00	0.00	0.00	0.00	0.00	0.00
MYO7A	0.00	0.00	0.00	0.12	0.00	0.11	0.00	0.15	0.00	0.00	0.00	0.00	0.00	0.00	0.15	0.00	0.00	0.00	0.00	0.00	0.00
EIF4A1	0.00	0.00	0.00	0.18	0.00	0.30	0.00	0.27	0.00	0.00	0.00	0.00	0.00	0.00	0.16	0.00	0.00	0.00	0.00	0.00	0.00
ELOF1	0.00	0.00	0.00	0.22	0.00	0.47	0.00	0.30	0.00	0.00	0.00	0.00	0.00	0.00	0.17	0.00	0.00	0.00	0.00	0.00	0.00
SMC1A	0.00	0.00	0.00	0.15	0.00	0.24	0.00	0.17	0.00	0.00	0.00	0.00	0.00	0.00	0.14	0.00	0.00	0.00	0.00	0.00	0.00

DUX4L9	0.00	0.00	0.00	0.21	0.00	0.38	0.00	0.15	0.00	0.00	0.00	0.00	0.00	0.00	0.11	0.00	0.00	0.00	0.00	0.00	0.00
COL7A1	0.00	0.00	0.00	0.13	0.00	0.14	0.00	0.00	0.15	0.00	0.00	0.00	0.00	0.00	0.11	0.00	0.00	0.00	0.00	0.00	0.00
CCDC28B	0.00	0.00	0.00	0.14	0.00	0.24	0.00	0.00	0.11	0.00	0.00	0.00	0.00	0.00	0.13	0.00	0.00	0.00	0.00	0.00	0.00
PRPH2	0.00	0.00	0.00	0.14	0.00	0.13	0.00	0.00	0.17	0.00	0.00	0.00	0.00	0.00	0.15	0.00	0.00	0.00	0.00	0.00	0.00
FECD3	0.00	0.00	0.00	0.11	0.00	0.16	0.00	0.00	0.13	0.00	0.00	0.00	0.00	0.00	0.13	0.00	0.00	0.00	0.00	0.00	0.00
COL6A1	0.00	0.00	0.00	0.15	0.00	0.18	0.00	0.00	0.12	0.00	0.00	0.00	0.00	0.00	0.16	0.00	0.00	0.00	0.00	0.00	0.00
MEFV	0.00	0.00	0.00	0.11	0.00	0.13	0.00	0.00	0.16	0.00	0.00	0.00	0.00	0.00	0.10	0.00	0.00	0.00	0.00	0.00	0.00
CNGB3	0.00	0.00	0.00	0.16	0.00	0.18	0.00	0.00	0.11	0.00	0.00	0.00	0.00	0.00	0.16	0.00	0.00	0.00	0.00	0.00	0.00
PRPF31	0.00	0.00	0.00	0.16	0.00	0.31	0.00	0.00	0.13	0.00	0.00	0.00	0.00	0.00	0.12	0.00	0.00	0.00	0.00	0.00	0.00
MFRP	0.00	0.00	0.00	0.18	0.00	0.27	0.00	0.00	0.10	0.00	0.00	0.00	0.00	0.00	0.16	0.00	0.00	0.00	0.00	0.00	0.00
HPS1	0.00	0.00	0.00	0.15	0.00	0.19	0.00	0.00	0.12	0.00	0.00	0.00	0.00	0.00	0.10	0.00	0.00	0.00	0.00	0.00	0.00
CCAL1	0.00	0.00	0.00	0.11	0.00	0.20	0.00	0.00	0.12	0.00	0.00	0.00	0.00	0.00	0.12	0.00	0.00	0.00	0.00	0.00	0.00
RPGR	0.00	0.00	0.00	0.12	0.00	0.21	0.00	0.00	0.11	0.00	0.00	0.00	0.00	0.00	0.11	0.00	0.00	0.00	0.00	0.00	0.00
HMGB1	0.00	0.00	0.00	0.12	0.00	0.00	0.00	0.14	0.19	0.00	0.00	0.00	0.00	0.00	0.12	0.00	0.00	0.00	0.00	0.00	0.00
MAP2K1	0.00	0.00	0.00	0.11	0.00	0.00	0.00	0.15	0.18	0.00	0.00	0.00	0.00	0.00	0.11	0.00	0.00	0.00	0.00	0.00	0.00
TRPV1	0.00	0.00	0.00	0.11	0.00	0.00	0.00	0.11	0.12	0.00	0.00	0.00	0.00	0.00	0.11	0.00	0.00	0.00	0.00	0.00	0.00
HSPB1	0.00	0.00	0.00	0.00	0.00	0.11	0.00	0.14	0.16	0.00	0.00	0.00	0.00	0.00	0.14	0.00	0.00	0.00	0.00	0.00	0.00
GJB3	0.00	0.00	0.00	0.13	0.00	0.15	0.00	0.00	0.00	0.00	0.11	0.00	0.00	0.00	0.13	0.00	0.00	0.00	0.00	0.00	0.00
ANKS4B	0.00	0.00	0.00	0.22	0.00	0.36	0.00	0.00	0.00	0.00	0.15	0.00	0.00	0.00	0.14	0.00	0.00	0.00	0.00	0.00	0.00
GLT1D1	0.00	0.00	0.00	0.14	0.00	0.18	0.00	0.00	0.00	0.00	0.20	0.00	0.00	0.00	0.11	0.00	0.00	0.00	0.00	0.00	0.00
PRB4	0.00	0.00	0.00	0.19	0.00	0.35	0.00	0.00	0.00	0.00	0.12	0.00	0.00	0.00	0.17	0.00	0.00	0.00	0.00	0.00	0.00
OTOP3	0.00	0.00	0.00	0.17	0.00	0.31	0.00	0.00	0.00	0.00	0.16	0.00	0.00	0.00	0.14	0.00	0.00	0.00	0.00	0.00	0.00
OTOP2	0.00	0.00	0.00	0.17	0.00	0.31	0.00	0.00	0.00	0.00	0.16	0.00	0.00	0.00	0.14	0.00	0.00	0.00	0.00	0.00	0.00
TMC6	0.00	0.00	0.00	0.16	0.00	0.27	0.00	0.00	0.00	0.00	0.13	0.00	0.00	0.00	0.12	0.00	0.00	0.00	0.00	0.00	0.00
TMC8	0.00	0.00	0.00	0.19	0.00	0.30	0.00	0.00	0.00	0.00	0.15	0.00	0.00	0.00	0.12	0.00	0.00	0.00	0.00	0.00	0.00
STRC	0.00	0.00	0.00	0.11	0.00	0.18	0.00	0.00	0.00	0.00	0.12	0.00	0.00	0.00	0.11	0.00	0.00	0.00	0.00	0.00	0.00
GLT8D1	0.00	0.00	0.00	0.14	0.00	0.18	0.00	0.00	0.00	0.00	0.20	0.00	0.00	0.00	0.11	0.00	0.00	0.00	0.00	0.00	0.00
USH1G	0.00	0.00	0.00	0.13	0.00	0.19	0.00	0.00	0.00	0.00	0.11	0.00	0.00	0.00	0.15	0.00	0.00	0.00	0.00	0.00	0.00
GJA3	0.00	0.00	0.00	0.16	0.00	0.18	0.00	0.00	0.00	0.00	0.14	0.00	0.00	0.00	0.14	0.00	0.00	0.00	0.00	0.00	0.00
IGKV1-39	0.00	0.00	0.00	0.19	0.00	0.30	0.00	0.00	0.00	0.00	0.10	0.00	0.00	0.00	0.11	0.00	0.00	0.00	0.00	0.00	0.00
CLRN1	0.00	0.00	0.00	0.18	0.00	0.27	0.00	0.00	0.00	0.00	0.15	0.00	0.00	0.00	0.17	0.00	0.00	0.00	0.00	0.00	0.00
LOC647264	0.00	0.00	0.00	0.13	0.00	0.20	0.00	0.00	0.00	0.00	0.11	0.00	0.00	0.00	0.18	0.00	0.00	0.00	0.00	0.00	0.00
GLT8D2	0.00	0.00	0.00	0.14	0.00	0.18	0.00	0.00	0.00	0.00	0.20	0.00	0.00	0.00	0.11	0.00	0.00	0.00	0.00	0.00	0.00
KRT74	0.00	0.00	0.00	0.21	0.00	0.39	0.00	0.00	0.00	0.00	0.13	0.00	0.00	0.00	0.16	0.00	0.00	0.00	0.00	0.00	0.00
LHFPL5	0.00	0.00	0.00	0.18	0.00	0.31	0.00	0.00	0.00	0.00	0.16	0.00	0.00	0.00	0.16	0.00	0.00	0.00	0.00	0.00	0.00
MED12L	0.00	0.00	0.00	0.15	0.00	0.30	0.00	0.00	0.00	0.00	0.18	0.00	0.00	0.00	0.14	0.00	0.00	0.00	0.00	0.00	0.00
CDH23	0.00	0.00	0.00	0.12	0.00	0.12	0.00	0.00	0.00	0.00	0.12	0.00	0.00	0.00	0.13	0.00	0.00	0.00	0.00	0.00	0.00
KRT25	0.00	0.00	0.00	0.24	0.00	0.40	0.00	0.00	0.00	0.00	0.11	0.00	0.00	0.00	0.20	0.00	0.00	0.00	0.00	0.00	0.00
USH1C	0.00	0.00	0.00	0.14	0.00	0.19	0.00	0.00	0.00	0.00	0.13	0.00	0.00	0.00	0.14	0.00	0.00	0.00	0.00	0.00	0.00
SLC4A11	0.00	0.00	0.00	0.13	0.00	0.22	0.00	0.00	0.00	0.00	0.16	0.00	0.00	0.00	0.14	0.00	0.00	0.00	0.00	0.00	0.00

IGKV1D-39	0.00	0.00	0.00	0.19	0.00	0.30	0.00	0.00	0.00	0.00	0.10	0.00	0.00	0.00	0.11	0.00	0.00	0.00	0.00	0.00	0.00
ROM1	0.00	0.00	0.00	0.16	0.00	0.27	0.00	0.00	0.00	0.00	0.11	0.00	0.00	0.00	0.15	0.00	0.00	0.00	0.00	0.00	0.00
GJA8	0.00	0.00	0.00	0.15	0.00	0.15	0.00	0.00	0.00	0.00	0.12	0.00	0.00	0.00	0.14	0.00	0.00	0.00	0.00	0.00	0.00
PDZD7	0.00	0.00	0.00	0.17	0.00	0.28	0.00	0.00	0.00	0.00	0.13	0.00	0.00	0.00	0.13	0.00	0.00	0.00	0.00	0.00	0.00
DFNA52	0.00	0.00	0.00	0.14	0.00	0.32	0.00	0.00	0.00	0.00	0.10	0.00	0.00	0.00	0.11	0.00	0.00	0.00	0.00	0.00	0.00
FADS6	0.00	0.00	0.00	0.16	0.00	0.32	0.00	0.00	0.00	0.00	0.12	0.00	0.00	0.00	0.14	0.00	0.00	0.00	0.00	0.00	0.00
DFNB59	0.00	0.00	0.00	0.14	0.00	0.24	0.00	0.00	0.00	0.00	0.10	0.00	0.00	0.00	0.17	0.00	0.00	0.00	0.00	0.00	0.00
CALM1P2	0.00	0.00	0.00	0.22	0.00	0.47	0.00	0.00	0.00	0.00	0.00	0.23	0.00	0.00	0.12	0.00	0.00	0.00	0.00	0.00	0.00
PRSS56	0.00	0.00	0.00	0.12	0.00	0.28	0.00	0.00	0.00	0.00	0.00	0.12	0.00	0.00	0.16	0.00	0.00	0.00	0.00	0.00	0.00
PRNT	0.00	0.00	0.00	0.19	0.00	0.35	0.00	0.00	0.00	0.00	0.00	0.12	0.00	0.00	0.15	0.00	0.00	0.00	0.00	0.00	0.00
HERC2P2	0.00	0.00	0.00	0.21	0.00	0.42	0.00	0.00	0.00	0.00	0.00	0.15	0.00	0.00	0.13	0.00	0.00	0.00	0.00	0.00	0.00
FRA13A	0.00	0.00	0.00	0.22	0.00	0.46	0.00	0.00	0.00	0.00	0.00	0.19	0.00	0.00	0.14	0.00	0.00	0.00	0.00	0.00	0.00
HERC2P3	0.00	0.00	0.00	0.21	0.00	0.42	0.00	0.00	0.00	0.00	0.00	0.15	0.00	0.00	0.13	0.00	0.00	0.00	0.00	0.00	0.00
PAR-SN	0.00	0.00	0.00	0.13	0.00	0.25	0.00	0.00	0.00	0.00	0.00	0.19	0.00	0.00	0.12	0.00	0.00	0.00	0.00	0.00	0.00
PLF	0.00	0.00	0.00	0.15	0.00	0.23	0.00	0.00	0.00	0.00	0.00	0.12	0.00	0.00	0.10	0.00	0.00	0.00	0.00	0.00	0.00
VCX	0.00	0.00	0.00	0.24	0.00	0.50	0.00	0.00	0.00	0.00	0.00	0.19	0.00	0.00	0.15	0.00	0.00	0.00	0.00	0.00	0.00
WBSCR22	0.00	0.00	0.00	0.19	0.00	0.40	0.00	0.00	0.00	0.00	0.00	0.19	0.00	0.00	0.14	0.00	0.00	0.00	0.00	0.00	0.00
RSPH6A	0.00	0.00	0.00	0.24	0.00	0.55	0.00	0.00	0.00	0.00	0.00	0.18	0.00	0.00	0.13	0.00	0.00	0.00	0.00	0.00	0.00
C15ORF2	0.00	0.00	0.00	0.13	0.00	0.29	0.00	0.00	0.00	0.00	0.00	0.18	0.00	0.00	0.14	0.00	0.00	0.00	0.00	0.00	0.00
DNAH9	0.00	0.00	0.00	0.18	0.00	0.40	0.00	0.00	0.00	0.00	0.00	0.18	0.00	0.00	0.15	0.00	0.00	0.00	0.00	0.00	0.00
NUP210L	0.00	0.00	0.00	0.18	0.00	0.28	0.00	0.00	0.00	0.00	0.00	0.13	0.00	0.00	0.16	0.00	0.00	0.00	0.00	0.00	0.00
NSUN5	0.00	0.00	0.00	0.19	0.00	0.40	0.00	0.00	0.00	0.00	0.00	0.19	0.00	0.00	0.14	0.00	0.00	0.00	0.00	0.00	0.00
NSUN5P1	0.00	0.00	0.00	0.19	0.00	0.40	0.00	0.00	0.00	0.00	0.00	0.19	0.00	0.00	0.14	0.00	0.00	0.00	0.00	0.00	0.00
NSUN5P2	0.00	0.00	0.00	0.19	0.00	0.40	0.00	0.00	0.00	0.00	0.00	0.19	0.00	0.00	0.14	0.00	0.00	0.00	0.00	0.00	0.00
KLHL4	0.00	0.00	0.00	0.18	0.00	0.44	0.00	0.00	0.00	0.00	0.00	0.19	0.00	0.00	0.17	0.00	0.00	0.00	0.00	0.00	0.00
LOC645355	0.00	0.00	0.00	0.12	0.00	0.28	0.00	0.00	0.00	0.00	0.00	0.12	0.00	0.00	0.13	0.00	0.00	0.00	0.00	0.00	0.00
GINGF2	0.00	0.00	0.00	0.17	0.00	0.34	0.00	0.00	0.00	0.00	0.00	0.15	0.00	0.00	0.11	0.00	0.00	0.00	0.00	0.00	0.00
FTH1P7	0.00	0.00	0.00	0.22	0.00	0.48	0.00	0.00	0.00	0.00	0.00	0.12	0.00	0.00	0.13	0.00	0.00	0.00	0.00	0.00	0.00
HNRNPA0	0.00	0.00	0.00	0.28	0.00	0.54	0.00	0.00	0.00	0.00	0.00	0.20	0.00	0.00	0.19	0.00	0.00	0.00	0.00	0.00	0.00
HERC2P7	0.00	0.00	0.00	0.24	0.00	0.46	0.00	0.00	0.00	0.00	0.00	0.15	0.00	0.00	0.16	0.00	0.00	0.00	0.00	0.00	0.00
LOH1CR1	0.00	0.00	0.00	0.18	0.00	0.32	0.00	0.00	0.00	0.00	0.00	0.16	0.00	0.00	0.18	0.00	0.00	0.00	0.00	0.00	0.00
ZNF711	0.00	0.00	0.00	0.22	0.00	0.39	0.00	0.00	0.00	0.00	0.00	0.21	0.00	0.00	0.17	0.00	0.00	0.00	0.00	0.00	0.00
LRRC48	0.00	0.00	0.00	0.20	0.00	0.45	0.00	0.00	0.00	0.00	0.00	0.15	0.00	0.00	0.11	0.00	0.00	0.00	0.00	0.00	0.00
ADAMTSL4	0.00	0.00	0.00	0.17	0.00	0.36	0.00	0.00	0.00	0.00	0.00	0.12	0.00	0.00	0.18	0.00	0.00	0.00	0.00	0.00	0.00
ALG9	0.00	0.00	0.00	0.16	0.00	0.30	0.00	0.00	0.00	0.00	0.00	0.15	0.00	0.00	0.19	0.00	0.00	0.00	0.00	0.00	0.00
GUSBP1	0.00	0.00	0.00	0.20	0.00	0.36	0.00	0.00	0.00	0.00	0.00	0.12	0.00	0.00	0.16	0.00	0.00	0.00	0.00	0.00	0.00
CCDC34	0.00	0.00	0.00	0.12	0.00	0.28	0.00	0.00	0.00	0.00	0.00	0.12	0.00	0.00	0.13	0.00	0.00	0.00	0.00	0.00	0.00
IGHV4-28	0.00	0.00	0.00	0.18	0.00	0.34	0.00	0.00	0.00	0.00	0.00	0.12	0.00	0.00	0.15	0.00	0.00	0.00	0.00	0.00	0.00
MDD1	0.00	0.00	0.00	0.12	0.00	0.20	0.00	0.00	0.00	0.00	0.00	0.12	0.00	0.00	0.13	0.00	0.00	0.00	0.00	0.00	0.00
USP53	0.00	0.00	0.00	0.18	0.00	0.34	0.00	0.00	0.00	0.00	0.00	0.20	0.00	0.00	0.14	0.00	0.00	0.00	0.00	0.00	0.00

DIH1	0.00	0.00	0.00	0.15	0.00	0.31	0.00	0.00	0.00	0.00	0.00	0.12	0.00	0.00	0.15	0.00	0.00	0.00	0.00	0.00	0.00
NOP14-AS1	0.00	0.00	0.00	0.22	0.00	0.46	0.00	0.00	0.00	0.00	0.00	0.18	0.00	0.00	0.10	0.00	0.00	0.00	0.00	0.00	0.00
FRMD7	0.00	0.00	0.00	0.17	0.00	0.27	0.00	0.00	0.00	0.00	0.00	0.18	0.00	0.00	0.22	0.00	0.00	0.00	0.00	0.00	0.00
GJE1	0.00	0.00	0.00	0.21	0.00	0.39	0.00	0.00	0.00	0.00	0.00	0.14	0.00	0.00	0.16	0.00	0.00	0.00	0.00	0.00	0.00
ADFN	0.00	0.00	0.00	0.16	0.00	0.30	0.00	0.00	0.00	0.00	0.00	0.17	0.00	0.00	0.16	0.00	0.00	0.00	0.00	0.00	0.00
TCTN2	0.00	0.00	0.00	0.16	0.00	0.26	0.00	0.00	0.00	0.00	0.00	0.14	0.00	0.00	0.18	0.00	0.00	0.00	0.00	0.00	0.00
GOLGA6B	0.00	0.00	0.00	0.23	0.00	0.39	0.00	0.00	0.00	0.00	0.00	0.13	0.00	0.00	0.12	0.00	0.00	0.00	0.00	0.00	0.00
GOLGA6A	0.00	0.00	0.00	0.23	0.00	0.39	0.00	0.00	0.00	0.00	0.00	0.13	0.00	0.00	0.12	0.00	0.00	0.00	0.00	0.00	0.00
RSS	0.00	0.00	0.00	0.14	0.00	0.24	0.00	0.00	0.00	0.00	0.00	0.14	0.00	0.00	0.11	0.00	0.00	0.00	0.00	0.00	0.00
OCLM	0.00	0.00	0.00	0.19	0.00	0.40	0.00	0.00	0.00	0.00	0.00	0.14	0.00	0.00	0.14	0.00	0.00	0.00	0.00	0.00	0.00
DIRC1	0.00	0.00	0.00	0.17	0.00	0.32	0.00	0.00	0.00	0.00	0.00	0.14	0.00	0.00	0.10	0.00	0.00	0.00	0.00	0.00	0.00
ZNF169	0.00	0.00	0.00	0.15	0.00	0.32	0.00	0.00	0.00	0.00	0.00	0.16	0.00	0.00	0.19	0.00	0.00	0.00	0.00	0.00	0.00
LOC100131434	0.00	0.00	0.00	0.16	0.00	0.39	0.00	0.00	0.00	0.00	0.00	0.14	0.00	0.00	0.14	0.00	0.00	0.00	0.00	0.00	0.00
SERPINA13	0.00	0.00	0.00	0.18	0.00	0.38	0.00	0.00	0.00	0.00	0.00	0.16	0.00	0.00	0.15	0.00	0.00	0.00	0.00	0.00	0.00
MKRN3-AS1	0.00	0.00	0.00	0.14	0.00	0.27	0.00	0.00	0.00	0.00	0.00	0.16	0.00	0.00	0.12	0.00	0.00	0.00	0.00	0.00	0.00
HFM	0.00	0.00	0.00	0.12	0.00	0.22	0.00	0.00	0.00	0.00	0.00	0.14	0.00	0.00	0.18	0.00	0.00	0.00	0.00	0.00	0.00
ANMA	0.00	0.00	0.00	0.12	0.00	0.20	0.00	0.00	0.00	0.00	0.00	0.16	0.00	0.00	0.14	0.00	0.00	0.00	0.00	0.00	0.00
VCX2	0.00	0.00	0.00	0.21	0.00	0.46	0.00	0.00	0.00	0.00	0.00	0.16	0.00	0.00	0.12	0.00	0.00	0.00	0.00	0.00	0.00
FGS2	0.00	0.00	0.00	0.14	0.00	0.24	0.00	0.00	0.00	0.00	0.00	0.16	0.00	0.00	0.12	0.00	0.00	0.00	0.00	0.00	0.00
IGHV4-39	0.00	0.00	0.00	0.19	0.00	0.38	0.00	0.00	0.00	0.00	0.00	0.13	0.00	0.00	0.16	0.00	0.00	0.00	0.00	0.00	0.00
VPS13B	0.00	0.00	0.00	0.12	0.00	0.27	0.00	0.00	0.00	0.00	0.00	0.13	0.00	0.00	0.14	0.00	0.00	0.00	0.00	0.00	0.00
F8A1	0.00	0.00	0.00	0.21	0.00	0.31	0.00	0.00	0.00	0.00	0.00	0.13	0.00	0.00	0.12	0.00	0.00	0.00	0.00	0.00	0.00
ZNF674	0.00	0.00	0.00	0.16	0.00	0.40	0.00	0.00	0.00	0.00	0.00	0.17	0.00	0.00	0.14	0.00	0.00	0.00	0.00	0.00	0.00
ZNF673	0.00	0.00	0.00	0.16	0.00	0.40	0.00	0.00	0.00	0.00	0.00	0.17	0.00	0.00	0.14	0.00	0.00	0.00	0.00	0.00	0.00
FGS3	0.00	0.00	0.00	0.15	0.00	0.33	0.00	0.00	0.00	0.00	0.00	0.17	0.00	0.00	0.11	0.00	0.00	0.00	0.00	0.00	0.00
BRP44L	0.00	0.00	0.00	0.20	0.00	0.50	0.00	0.00	0.00	0.00	0.00	0.18	0.00	0.00	0.17	0.00	0.00	0.00	0.00	0.00	0.00
ZNF41	0.00	0.00	0.00	0.20	0.00	0.47	0.00	0.00	0.00	0.00	0.00	0.18	0.00	0.00	0.13	0.00	0.00	0.00	0.00	0.00	0.00
CST2	0.00	0.00	0.00	0.21	0.00	0.34	0.00	0.00	0.00	0.00	0.00	0.18	0.00	0.00	0.16	0.00	0.00	0.00	0.00	0.00	0.00
FHDC1	0.00	0.00	0.00	0.19	0.00	0.36	0.00	0.00	0.00	0.00	0.00	0.14	0.00	0.00	0.15	0.00	0.00	0.00	0.00	0.00	0.00
CHML	0.00	0.00	0.00	0.19	0.00	0.38	0.00	0.00	0.00	0.00	0.00	0.14	0.00	0.00	0.14	0.00	0.00	0.00	0.00	0.00	0.00
MT1L	0.00	0.00	0.00	0.21	0.00	0.47	0.00	0.00	0.00	0.00	0.00	0.13	0.00	0.00	0.13	0.00	0.00	0.00	0.00	0.00	0.00
NHS	0.00	0.00	0.00	0.18	0.00	0.31	0.00	0.00	0.00	0.00	0.00	0.17	0.00	0.00	0.18	0.00	0.00	0.00	0.00	0.00	0.00
ZNF630	0.00	0.00	0.00	0.11	0.00	0.23	0.00	0.00	0.00	0.00	0.00	0.16	0.00	0.00	0.11	0.00	0.00	0.00	0.00	0.00	0.00
CXORF40B	0.00	0.00	0.00	0.13	0.00	0.34	0.00	0.00	0.00	0.00	0.00	0.13	0.00	0.00	0.13	0.00	0.00	0.00	0.00	0.00	0.00
TMEM216	0.00	0.00	0.00	0.14	0.00	0.30	0.00	0.00	0.00	0.00	0.00	0.17	0.00	0.00	0.17	0.00	0.00	0.00	0.00	0.00	0.00
RP1L1	0.00	0.00	0.00	0.22	0.00	0.38	0.00	0.00	0.00	0.00	0.00	0.17	0.00	0.00	0.19	0.00	0.00	0.00	0.00	0.00	0.00
SCA31	0.00	0.00	0.00	0.18	0.00	0.35	0.00	0.00	0.00	0.00	0.00	0.21	0.00	0.00	0.21	0.00	0.00	0.00	0.00	0.00	0.00
C17ORF39	0.00	0.00	0.00	0.20	0.00	0.45	0.00	0.00	0.00	0.00	0.00	0.15	0.00	0.00	0.11	0.00	0.00	0.00	0.00	0.00	0.00
HPS3	0.00	0.00	0.00	0.16	0.00	0.24	0.00	0.00	0.00	0.00	0.00	0.11	0.00	0.00	0.10	0.00	0.00	0.00	0.00	0.00	0.00
DOCK6	0.00	0.00	0.00	0.14	0.00	0.25	0.00	0.00	0.00	0.00	0.00	0.11	0.00	0.00	0.15	0.00	0.00	0.00	0.00	0.00	0.00

AASTH36	0.00	0.00	0.00	0.19	0.00	0.32	0.00	0.00	0.00	0.00	0.00	0.15	0.00	0.00	0.14	0.00	0.00	0.00	0.00	0.00	0.00
SPATA7	0.00	0.00	0.00	0.13	0.00	0.30	0.00	0.00	0.00	0.00	0.00	0.10	0.00	0.00	0.13	0.00	0.00	0.00	0.00	0.00	0.00
RPGRIP1	0.00	0.00	0.00	0.18	0.00	0.32	0.00	0.00	0.00	0.00	0.00	0.10	0.00	0.00	0.16	0.00	0.00	0.00	0.00	0.00	0.00
KIF21A	0.00	0.00	0.00	0.13	0.00	0.23	0.00	0.00	0.00	0.00	0.00	0.10	0.00	0.00	0.18	0.00	0.00	0.00	0.00	0.00	0.00
AASTH33	0.00	0.00	0.00	0.19	0.00	0.32	0.00	0.00	0.00	0.00	0.00	0.15	0.00	0.00	0.14	0.00	0.00	0.00	0.00	0.00	0.00
AASTH35	0.00	0.00	0.00	0.19	0.00	0.32	0.00	0.00	0.00	0.00	0.00	0.15	0.00	0.00	0.14	0.00	0.00	0.00	0.00	0.00	0.00
AASTH37	0.00	0.00	0.00	0.19	0.00	0.32	0.00	0.00	0.00	0.00	0.00	0.15	0.00	0.00	0.14	0.00	0.00	0.00	0.00	0.00	0.00
PMS2P1	0.00	0.00	0.00	0.15	0.00	0.33	0.00	0.00	0.00	0.00	0.00	0.10	0.00	0.00	0.10	0.00	0.00	0.00	0.00	0.00	0.00
ABCD1P4	0.00	0.00	0.00	0.14	0.00	0.28	0.00	0.00	0.00	0.00	0.00	0.11	0.00	0.00	0.14	0.00	0.00	0.00	0.00	0.00	0.00
AASTH34	0.00	0.00	0.00	0.19	0.00	0.32	0.00	0.00	0.00	0.00	0.00	0.15	0.00	0.00	0.14	0.00	0.00	0.00	0.00	0.00	0.00
LOC646652	0.00	0.00	0.00	0.18	0.00	0.35	0.00	0.00	0.00	0.00	0.00	0.11	0.00	0.00	0.14	0.00	0.00	0.00	0.00	0.00	0.00
HSPB1P1	0.00	0.00	0.00	0.15	0.00	0.32	0.00	0.00	0.00	0.00	0.00	0.11	0.00	0.00	0.10	0.00	0.00	0.00	0.00	0.00	0.00
HERC2P6	0.00	0.00	0.00	0.17	0.00	0.36	0.00	0.00	0.00	0.00	0.00	0.11	0.00	0.00	0.12	0.00	0.00	0.00	0.00	0.00	0.00
MRX65	0.00	0.00	0.00	0.10	0.00	0.23	0.00	0.00	0.00	0.00	0.00	0.15	0.00	0.00	0.11	0.00	0.00	0.00	0.00	0.00	0.00
NDUFA7	0.00	0.00	0.00	0.20	0.00	0.44	0.00	0.00	0.00	0.00	0.00	0.15	0.00	0.00	0.13	0.00	0.00	0.00	0.00	0.00	0.00
WBSCR28	0.00	0.00	0.00	0.17	0.00	0.43	0.00	0.00	0.00	0.00	0.00	0.15	0.00	0.00	0.13	0.00	0.00	0.00	0.00	0.00	0.00
FAM189A2	0.00	0.00	0.00	0.12	0.00	0.34	0.00	0.00	0.00	0.00	0.00	0.10	0.00	0.00	0.12	0.00	0.00	0.00	0.00	0.00	0.00
LOH19CR1	0.00	0.00	0.00	0.11	0.00	0.20	0.00	0.00	0.00	0.00	0.00	0.10	0.00	0.00	0.12	0.00	0.00	0.00	0.00	0.00	0.00
RPL36	0.00	0.00	0.00	0.22	0.00	0.46	0.00	0.00	0.00	0.00	0.00	0.10	0.00	0.00	0.10	0.00	0.00	0.00	0.00	0.00	0.00
EVC2	0.00	0.00	0.00	0.15	0.00	0.26	0.00	0.00	0.00	0.00	0.00	0.10	0.00	0.00	0.14	0.00	0.00	0.00	0.00	0.00	0.00
DPY19L2	0.00	0.00	0.00	0.21	0.00	0.44	0.00	0.00	0.00	0.00	0.00	0.10	0.00	0.00	0.14	0.00	0.00	0.00	0.00	0.00	0.00
WBSCR2	0.00	0.00	0.00	0.17	0.00	0.43	0.00	0.00	0.00	0.00	0.00	0.15	0.00	0.00	0.13	0.00	0.00	0.00	0.00	0.00	0.00
TTC12	0.00	0.00	0.00	0.13	0.00	0.15	0.00	0.00	0.00	0.00	0.00	0.10	0.00	0.00	0.11	0.00	0.00	0.00	0.00	0.00	0.00
FTSJ1	0.00	0.00	0.00	0.15	0.00	0.31	0.00	0.00	0.00	0.00	0.00	0.15	0.00	0.00	0.15	0.00	0.00	0.00	0.00	0.00	0.00
BASP1P1	0.00	0.00	0.00	0.14	0.00	0.29	0.00	0.00	0.00	0.00	0.00	0.15	0.00	0.00	0.20	0.00	0.00	0.00	0.00	0.00	0.00
BPESC1	0.00	0.00	0.00	0.16	0.00	0.39	0.00	0.00	0.00	0.00	0.00	0.15	0.00	0.00	0.11	0.00	0.00	0.00	0.00	0.00	0.00
COD2	0.00	0.00	0.00	0.11	0.00	0.30	0.00	0.00	0.00	0.00	0.00	0.10	0.00	0.00	0.10	0.00	0.00	0.00	0.00	0.00	0.00
BULN	0.00	0.00	0.00	0.13	0.00	0.16	0.00	0.00	0.00	0.00	0.00	0.15	0.00	0.00	0.16	0.00	0.00	0.00	0.00	0.00	0.00
FGS5	0.00	0.00	0.00	0.10	0.00	0.25	0.00	0.00	0.00	0.00	0.00	0.15	0.00	0.00	0.11	0.00	0.00	0.00	0.00	0.00	0.00
ABCD1P3	0.00	0.00	0.00	0.14	0.00	0.28	0.00	0.00	0.00	0.00	0.00	0.11	0.00	0.00	0.14	0.00	0.00	0.00	0.00	0.00	0.00
ABCD1P2	0.00	0.00	0.00	0.14	0.00	0.28	0.00	0.00	0.00	0.00	0.00	0.11	0.00	0.00	0.14	0.00	0.00	0.00	0.00	0.00	0.00
DEPDC7	0.00	0.00	0.00	0.23	0.00	0.53	0.00	0.00	0.00	0.00	0.00	0.22	0.00	0.00	0.14	0.00	0.00	0.00	0.00	0.00	0.00
ST7-OT3	0.00	0.00	0.00	0.22	0.00	0.40	0.00	0.00	0.00	0.00	0.00	0.22	0.00	0.00	0.20	0.00	0.00	0.00	0.00	0.00	0.00
RSPH4A	0.00	0.00	0.00	0.14	0.00	0.34	0.00	0.00	0.00	0.00	0.00	0.11	0.00	0.00	0.12	0.00	0.00	0.00	0.00	0.00	0.00
CRYBA4	0.00	0.00	0.00	0.14	0.00	0.24	0.00	0.00	0.00	0.00	0.00	0.11	0.00	0.00	0.15	0.00	0.00	0.00	0.00	0.00	0.00
BEAN1	0.00	0.00	0.00	0.22	0.00	0.37	0.00	0.00	0.00	0.00	0.00	0.23	0.00	0.00	0.25	0.00	0.00	0.00	0.00	0.00	0.00
SMA5	0.00	0.00	0.00	0.17	0.00	0.35	0.00	0.00	0.00	0.00	0.00	0.12	0.00	0.00	0.15	0.00	0.00	0.00	0.00	0.00	0.00
ST7-AS2	0.00	0.00	0.00	0.16	0.00	0.29	0.00	0.00	0.00	0.00	0.00	0.22	0.00	0.00	0.17	0.00	0.00	0.00	0.00	0.00	0.00
SPG45	0.00	0.00	0.00	0.10	0.00	0.27	0.00	0.00	0.00	0.00	0.00	0.12	0.00	0.00	0.13	0.00	0.00	0.00	0.00	0.00	0.00
LOC202181	0.00	0.00	0.00	0.15	0.00	0.30	0.00	0.00	0.00	0.00	0.00	0.22	0.00	0.00	0.13	0.00	0.00	0.00	0.00	0.00	0.00

ST7-OT4	0.00	0.00	0.00	0.16	0.00	0.29	0.00	0.00	0.00	0.00	0.00	0.22	0.00	0.00	0.17	0.00	0.00	0.00	0.00	0.00	0.00
ST7-AS1	0.00	0.00	0.00	0.16	0.00	0.29	0.00	0.00	0.00	0.00	0.00	0.22	0.00	0.00	0.17	0.00	0.00	0.00	0.00	0.00	0.00
GUSBP3	0.00	0.00	0.00	0.20	0.00	0.40	0.00	0.00	0.00	0.00	0.00	0.12	0.00	0.00	0.17	0.00	0.00	0.00	0.00	0.00	0.00
ZNF469	0.00	0.00	0.00	0.18	0.00	0.32	0.00	0.00	0.00	0.00	0.00	0.15	0.00	0.00	0.18	0.00	0.00	0.00	0.00	0.00	0.00
COL6A5	0.00	0.00	0.00	0.18	0.00	0.30	0.00	0.00	0.00	0.00	0.00	0.12	0.00	0.00	0.12	0.00	0.00	0.00	0.00	0.00	0.00
CEP41	0.00	0.00	0.00	0.17	0.00	0.35	0.00	0.00	0.00	0.00	0.00	0.25	0.00	0.00	0.16	0.00	0.00	0.00	0.00	0.00	0.00
SHFM2	0.00	0.00	0.00	0.14	0.00	0.27	0.00	0.00	0.00	0.00	0.00	0.11	0.00	0.00	0.11	0.00	0.00	0.00	0.00	0.00	0.00
RAI2	0.00	0.00	0.00	0.21	0.00	0.44	0.00	0.00	0.00	0.00	0.00	0.25	0.00	0.00	0.19	0.00	0.00	0.00	0.00	0.00	0.00
FAM161A	0.00	0.00	0.00	0.17	0.00	0.30	0.00	0.00	0.00	0.00	0.00	0.11	0.00	0.00	0.17	0.00	0.00	0.00	0.00	0.00	0.00
PPP4R4	0.00	0.00	0.00	0.18	0.00	0.36	0.00	0.00	0.00	0.00	0.00	0.12	0.00	0.00	0.12	0.00	0.00	0.00	0.00	0.00	0.00
C7ORF10	0.00	0.00	0.00	0.12	0.00	0.28	0.00	0.00	0.00	0.00	0.00	0.11	0.00	0.00	0.13	0.00	0.00	0.00	0.00	0.00	0.00
HERC2P1	0.00	0.00	0.00	0.22	0.00	0.43	0.00	0.00	0.00	0.00	0.00	0.15	0.00	0.00	0.14	0.00	0.00	0.00	0.00	0.00	0.00
PHF10P1	0.00	0.00	0.00	0.17	0.00	0.32	0.00	0.00	0.00	0.00	0.00	0.11	0.00	0.00	0.12	0.00	0.00	0.00	0.00	0.00	0.00
KCNMB3P1	0.00	0.00	0.00	0.16	0.00	0.36	0.00	0.00	0.00	0.00	0.00	0.24	0.00	0.00	0.19	0.00	0.00	0.00	0.00	0.00	0.00
FAM193A	0.00	0.00	0.00	0.24	0.00	0.47	0.00	0.00	0.00	0.00	0.00	0.24	0.00	0.00	0.20	0.00	0.00	0.00	0.00	0.00	0.00
CCT8L2	0.00	0.00	0.00	0.16	0.00	0.36	0.00	0.00	0.00	0.00	0.00	0.24	0.00	0.00	0.19	0.00	0.00	0.00	0.00	0.00	0.00
CMD1B	0.00	0.00	0.00	0.00	0.00	0.13	0.00	0.00	0.14	0.00	0.00	0.17	0.00	0.00	0.15	0.00	0.00	0.00	0.00	0.00	0.00
PARK4	0.00	0.00	0.00	0.00	0.00	0.11	0.00	0.00	0.10	0.00	0.00	0.16	0.00	0.00	0.14	0.00	0.00	0.00	0.00	0.00	0.00
WHCR	0.00	0.00	0.00	0.00	0.00	0.12	0.00	0.00	0.15	0.00	0.00	0.14	0.00	0.00	0.13	0.00	0.00	0.00	0.00	0.00	0.00
LOC728519	0.00	0.00	0.00	0.00	0.00	0.19	0.00	0.00	0.16	0.00	0.00	0.12	0.00	0.00	0.19	0.00	0.00	0.00	0.00	0.00	0.00
CNIH4	0.00	0.00	0.00	0.12	0.00	0.10	0.00	0.00	0.00	0.00	0.00	0.00	0.10	0.00	0.11	0.00	0.00	0.00	0.00	0.00	0.00
C3ORF70	0.00	0.00	0.00	0.12	0.00	0.10	0.00	0.00	0.00	0.00	0.00	0.00	0.10	0.00	0.11	0.00	0.00	0.00	0.00	0.00	0.00
FHA2	0.00	0.00	0.00	0.13	0.00	0.18	0.00	0.00	0.00	0.00	0.00	0.00	0.11	0.00	0.11	0.00	0.00	0.00	0.00	0.00	0.00
SCZD6	0.00	0.00	0.00	0.00	0.00	0.13	0.00	0.00	0.00	0.00	0.00	0.12	0.13	0.00	0.12	0.00	0.00	0.00	0.00	0.00	0.00
AUTS8	0.00	0.00	0.00	0.00	0.00	0.11	0.00	0.00	0.00	0.00	0.00	0.16	0.14	0.00	0.14	0.00	0.00	0.00	0.00	0.00	0.00
SCZD3	0.00	0.00	0.00	0.00	0.00	0.11	0.00	0.00	0.00	0.00	0.00	0.14	0.16	0.00	0.16	0.00	0.00	0.00	0.00	0.00	0.00
SCZD7	0.00	0.00	0.00	0.00	0.00	0.12	0.00	0.00	0.00	0.00	0.00	0.12	0.13	0.00	0.12	0.00	0.00	0.00	0.00	0.00	0.00
ASPG1	0.00	0.00	0.00	0.00	0.00	0.11	0.00	0.00	0.00	0.00	0.00	0.16	0.14	0.00	0.14	0.00	0.00	0.00	0.00	0.00	0.00
SCZD10	0.00	0.00	0.00	0.00	0.00	0.15	0.00	0.00	0.00	0.00	0.00	0.11	0.12	0.00	0.11	0.00	0.00	0.00	0.00	0.00	0.00
PLG	0.00	0.00	0.00	0.00	0.00	0.00	0.00	0.10	0.15	0.00	0.00	0.00	0.00	0.10	0.13	0.00	0.00	0.00	0.00	0.00	0.00
EIG2	0.00	0.00	0.00	0.00	0.00	0.19	0.00	0.00	0.00	0.00	0.00	0.15	0.00	0.13	0.14	0.00	0.00	0.00	0.00	0.00	0.00
EPPS	0.00	0.00	0.00	0.00	0.00	0.00	0.00	0.00	0.00	0.00	0.00	0.14	0.13	0.22	0.10	0.00	0.00	0.00	0.00	0.00	0.00
AUTS6	0.00	0.00	0.00	0.00	0.00	0.00	0.00	0.00	0.00	0.00	0.00	0.15	0.13	0.11	0.13	0.00	0.00	0.00	0.00	0.00	0.00
SCA29	0.00	0.00	0.00	0.00	0.00	0.00	0.00	0.00	0.00	0.00	0.00	0.16	0.15	0.11	0.16	0.00	0.00	0.00	0.00	0.00	0.00
NMSR	0.00	0.00	0.00	0.14	0.00	0.24	0.00	0.00	0.00	0.00	0.00	0.00	0.00	0.00	0.28	0.15	0.00	0.00	0.00	0.00	0.00
LOC644462	0.00	0.00	0.00	0.12	0.00	0.24	0.00	0.00	0.00	0.00	0.00	0.00	0.00	0.00	0.15	0.12	0.00	0.00	0.00	0.00	0.00
PSMA2P3	0.00	0.00	0.00	0.12	0.00	0.24	0.00	0.00	0.00	0.00	0.00	0.00	0.00	0.00	0.15	0.12	0.00	0.00	0.00	0.00	0.00
EGR2	0.00	0.00	0.00	0.11	0.00	0.00	0.00	0.21	0.00	0.00	0.00	0.00	0.00	0.00	0.28	0.22	0.00	0.00	0.00	0.00	0.00
CASP1	0.00	0.00	0.00	0.11	0.00	0.00	0.00	0.00	0.16	0.00	0.00	0.00	0.00	0.00	0.14	0.12	0.00	0.00	0.00	0.00	0.00
HMNJ	0.00	0.00	0.00	0.00	0.00	0.12	0.00	0.00	0.11	0.00	0.00	0.00	0.00	0.00	0.24	0.14	0.00	0.00	0.00	0.00	0.00

CMT2H	0.00	0.00	0.00	0.00	0.00	0.12	0.00	0.00	0.11	0.00	0.00	0.00	0.00	0.00	0.18	0.14	0.00	0.00	0.00	0.00	0.00
GJB1	0.00	0.00	0.00	0.11	0.00	0.00	0.00	0.00	0.00	0.00	0.11	0.00	0.00	0.00	0.21	0.19	0.00	0.00	0.00	0.00	0.00
SPG30	0.00	0.00	0.00	0.00	0.00	0.16	0.00	0.00	0.00	0.00	0.00	0.11	0.00	0.00	0.19	0.13	0.00	0.00	0.00	0.00	0.00
SPG32	0.00	0.00	0.00	0.00	0.00	0.16	0.00	0.00	0.00	0.00	0.00	0.15	0.00	0.00	0.16	0.11	0.00	0.00	0.00	0.00	0.00
SPOAN	0.00	0.00	0.00	0.00	0.00	0.16	0.00	0.00	0.00	0.00	0.00	0.11	0.00	0.00	0.23	0.18	0.00	0.00	0.00	0.00	0.00
CMT2G	0.00	0.00	0.00	0.00	0.00	0.00	0.00	0.00	0.11	0.00	0.00	0.12	0.00	0.00	0.39	0.32	0.00	0.00	0.00	0.00	0.00
SCASI	0.00	0.00	0.00	0.00	0.00	0.00	0.00	0.00	0.00	0.00	0.00	0.13	0.11	0.00	0.24	0.22	0.00	0.00	0.00	0.00	0.00
CALB1	0.00	0.00	0.00	0.00	0.00	0.00	0.00	0.00	0.00	0.00	0.00	0.00	0.18	0.14	0.14	0.13	0.00	0.00	0.00	0.00	0.00
UAE2	0.00	0.00	0.00	0.15	0.00	0.13	0.11	0.00	0.00	0.00	0.00	0.00	0.00	0.00	0.00	0.00	0.24	0.00	0.00	0.00	0.00
UAE1	0.00	0.00	0.00	0.15	0.00	0.13	0.11	0.00	0.00	0.00	0.00	0.00	0.00	0.00	0.00	0.00	0.24	0.00	0.00	0.00	0.00
SAMD3	0.00	0.00	0.00	0.15	0.00	0.19	0.00	0.12	0.00	0.00	0.00	0.00	0.00	0.00	0.00	0.00	0.10	0.00	0.00	0.00	0.00
CYP27C1	0.00	0.00	0.00	0.11	0.00	0.14	0.00	0.00	0.14	0.00	0.00	0.00	0.00	0.00	0.00	0.00	0.13	0.00	0.00	0.00	0.00
LINC00305	0.00	0.00	0.00	0.14	0.00	0.22	0.00	0.00	0.10	0.00	0.00	0.00	0.00	0.00	0.00	0.00	0.14	0.00	0.00	0.00	0.00
LOC401180	0.00	0.00	0.00	0.13	0.00	0.17	0.00	0.00	0.11	0.00	0.00	0.00	0.00	0.00	0.00	0.00	0.15	0.00	0.00	0.00	0.00
CCDC122	0.00	0.00	0.00	0.13	0.00	0.00	0.12	0.00	0.12	0.00	0.00	0.00	0.00	0.00	0.00	0.00	0.11	0.00	0.00	0.00	0.00
ALB	0.00	0.00	0.00	0.12	0.00	0.00	0.00	0.11	0.12	0.00	0.00	0.00	0.00	0.00	0.00	0.00	0.10	0.00	0.00	0.00	0.00
EXT3	0.00	0.00	0.00	0.14	0.00	0.32	0.00	0.00	0.00	0.00	0.13	0.00	0.00	0.00	0.00	0.00	0.11	0.00	0.00	0.00	0.00
CFM1	0.00	0.00	0.00	0.00	0.00	0.12	0.00	0.00	0.17	0.00	0.15	0.00	0.00	0.00	0.00	0.00	0.10	0.00	0.00	0.00	0.00
QSER1	0.00	0.00	0.00	0.12	0.00	0.16	0.00	0.00	0.00	0.00	0.00	0.13	0.00	0.00	0.00	0.00	0.15	0.00	0.00	0.00	0.00
GINGF4	0.00	0.00	0.00	0.18	0.00	0.30	0.00	0.00	0.00	0.00	0.00	0.12	0.00	0.00	0.00	0.00	0.12	0.00	0.00	0.00	0.00
CDCP2	0.00	0.00	0.00	0.15	0.00	0.21	0.00	0.00	0.00	0.00	0.00	0.15	0.00	0.00	0.00	0.00	0.14	0.00	0.00	0.00	0.00
HRTRT1	0.00	0.00	0.00	0.11	0.00	0.16	0.00	0.00	0.00	0.00	0.00	0.11	0.00	0.00	0.00	0.00	0.23	0.00	0.00	0.00	0.00
MS4A6A	0.00	0.00	0.00	0.14	0.00	0.22	0.00	0.00	0.00	0.00	0.00	0.17	0.00	0.00	0.00	0.00	0.12	0.00	0.00	0.00	0.00
STQTL7	0.00	0.00	0.00	0.14	0.00	0.22	0.00	0.00	0.00	0.00	0.00	0.11	0.00	0.00	0.00	0.00	0.10	0.00	0.00	0.00	0.00
MS4A4E	0.00	0.00	0.00	0.14	0.00	0.22	0.00	0.00	0.00	0.00	0.00	0.17	0.00	0.00	0.00	0.00	0.12	0.00	0.00	0.00	0.00
PPR1	0.00	0.00	0.00	0.00	0.00	0.11	0.00	0.00	0.00	0.00	0.00	0.11	0.00	0.10	0.00	0.00	0.11	0.00	0.00	0.00	0.00
CP20	0.00	0.00	0.00	0.19	0.00	0.32	0.00	0.00	0.00	0.00	0.00	0.00	0.00	0.00	0.12	0.00	0.12	0.00	0.00	0.00	0.00
PHOBS	0.00	0.00	0.00	0.11	0.00	0.14	0.00	0.00	0.00	0.00	0.00	0.00	0.00	0.00	0.10	0.00	0.10	0.00	0.00	0.00	0.00
DCAF17	0.00	0.00	0.00	0.14	0.00	0.27	0.00	0.00	0.00	0.00	0.00	0.00	0.00	0.00	0.14	0.00	0.17	0.00	0.00	0.00	0.00
STQTL3	0.00	0.00	0.00	0.11	0.00	0.17	0.00	0.00	0.00	0.00	0.00	0.00	0.00	0.00	0.13	0.00	0.14	0.00	0.00	0.00	0.00
AIS1	0.00	0.00	0.00	0.11	0.00	0.17	0.00	0.00	0.00	0.00	0.00	0.00	0.00	0.00	0.13	0.00	0.16	0.00	0.00	0.00	0.00
STQTL4	0.00	0.00	0.00	0.11	0.00	0.17	0.00	0.00	0.00	0.00	0.00	0.00	0.00	0.00	0.13	0.00	0.14	0.00	0.00	0.00	0.00
CCV	0.00	0.00	0.00	0.11	0.00	0.17	0.00	0.00	0.00	0.00	0.00	0.00	0.00	0.00	0.13	0.00	0.12	0.00	0.00	0.00	0.00
PTLAH	0.00	0.00	0.00	0.10	0.00	0.23	0.00	0.00	0.00	0.00	0.00	0.00	0.00	0.00	0.11	0.00	0.11	0.00	0.00	0.00	0.00
BP23	0.00	0.00	0.00	0.11	0.00	0.15	0.00	0.00	0.00	0.00	0.00	0.00	0.00	0.00	0.13	0.00	0.19	0.00	0.00	0.00	0.00
STQTL2	0.00	0.00	0.00	0.12	0.00	0.18	0.00	0.00	0.00	0.00	0.00	0.00	0.00	0.00	0.11	0.00	0.19	0.00	0.00	0.00	0.00
TBL2	0.00	0.00	0.00	0.16	0.00	0.32	0.00	0.00	0.00	0.00	0.00	0.00	0.00	0.00	0.11	0.00	0.16	0.00	0.00	0.00	0.00
BP22	0.00	0.00	0.00	0.16	0.00	0.32	0.00	0.00	0.00	0.00	0.00	0.00	0.00	0.00	0.12	0.00	0.18	0.00	0.00	0.00	0.00
PIH	0.00	0.00	0.00	0.12	0.00	0.21	0.00	0.00	0.00	0.00	0.00	0.00	0.00	0.00	0.13	0.00	0.14	0.00	0.00	0.00	0.00
SLEB3	0.00	0.00	0.00	0.16	0.00	0.20	0.00	0.00	0.00	0.00	0.00	0.00	0.00	0.00	0.11	0.00	0.12	0.00	0.00	0.00	0.00

GJA4	0.00	0.00	0.00	0.15	0.00	0.13	0.00	0.00	0.00	0.00	0.00	0.00	0.00	0.00	0.16	0.00	0.13	0.00	0.00	0.00	0.00
PARK3	0.00	0.00	0.00	0.00	0.00	0.14	0.00	0.00	0.00	0.00	0.00	0.14	0.00	0.00	0.14	0.00	0.10	0.00	0.00	0.00	0.00
SLI2	0.00	0.00	0.00	0.00	0.00	0.18	0.00	0.00	0.00	0.00	0.00	0.12	0.00	0.00	0.13	0.00	0.11	0.00	0.00	0.00	0.00
PARK10	0.00	0.00	0.00	0.00	0.00	0.13	0.00	0.00	0.00	0.00	0.00	0.11	0.00	0.00	0.11	0.00	0.11	0.00	0.00	0.00	0.00
C3ORF39	0.00	0.00	0.00	0.00	0.00	0.00	0.00	0.00	0.00	0.00	0.00	0.10	0.10	0.00	0.11	0.00	0.10	0.00	0.00	0.00	0.00
LRRIQ3	0.00	0.00	0.00	0.00	0.00	0.00	0.00	0.00	0.00	0.00	0.00	0.10	0.10	0.00	0.11	0.00	0.10	0.00	0.00	0.00	0.00
MS	0.00	0.00	0.00	0.00	0.00	0.16	0.00	0.00	0.00	0.00	0.00	0.00	0.00	0.00	0.14	0.13	0.31	0.00	0.00	0.00	0.00
CRYGC	0.00	0.00	0.00	0.13	0.00	0.18	0.00	0.00	0.00	0.00	0.00	0.00	0.00	0.00	0.11	0.00	0.00	0.11	0.00	0.00	0.00
CRYBB2	0.00	0.00	0.00	0.15	0.00	0.18	0.00	0.00	0.00	0.00	0.00	0.00	0.00	0.00	0.15	0.00	0.00	0.12	0.00	0.00	0.00
GH1	0.00	0.00	0.00	0.12	0.00	0.00	0.00	0.00	0.11	0.00	0.00	0.00	0.00	0.00	0.10	0.00	0.00	0.11	0.00	0.00	0.00
CRYAB	0.00	0.00	0.00	0.00	0.00	0.00	0.00	0.13	0.13	0.00	0.00	0.00	0.00	0.00	0.11	0.00	0.00	0.18	0.00	0.00	0.00
NPY	0.00	0.00	0.00	0.10	0.00	0.00	0.00	0.00	0.00	0.00	0.00	0.00	0.00	0.14	0.14	0.00	0.00	0.19	0.00	0.00	0.00
LPAL1	0.00	0.00	0.00	0.18	0.00	0.30	0.00	0.16	0.00	0.00	0.00	0.00	0.00	0.00	0.00	0.00	0.00	0.00	0.16	0.00	0.00
FTH1P5	0.00	0.00	0.00	0.18	0.00	0.36	0.00	0.00	0.00	0.00	0.00	0.11	0.00	0.00	0.00	0.00	0.00	0.00	0.11	0.00	0.00
PTS-P1	0.00	0.00	0.00	0.11	0.00	0.40	0.00	0.00	0.00	0.00	0.00	0.15	0.00	0.00	0.00	0.00	0.00	0.00	0.11	0.00	0.00
TCN1	0.00	0.00	0.00	0.13	0.00	0.12	0.00	0.00	0.00	0.00	0.00	0.00	0.00	0.00	0.11	0.00	0.00	0.00	0.24	0.00	0.00
CYP2G2P	0.00	0.00	0.00	0.14	0.00	0.30	0.00	0.00	0.00	0.00	0.00	0.00	0.00	0.00	0.10	0.00	0.00	0.00	0.18	0.00	0.00
GBAP1	0.00	0.00	0.00	0.15	0.00	0.32	0.00	0.00	0.00	0.00	0.00	0.00	0.00	0.00	0.12	0.00	0.00	0.00	0.11	0.00	0.00
CYP4V2	0.00	0.00	0.00	0.13	0.00	0.21	0.00	0.00	0.00	0.00	0.00	0.00	0.00	0.00	0.15	0.00	0.00	0.00	0.13	0.00	0.00
LOXL1	0.00	0.00	0.00	0.11	0.00	0.14	0.00	0.00	0.00	0.00	0.00	0.00	0.00	0.00	0.13	0.00	0.00	0.00	0.10	0.00	0.00
HRES1	0.00	0.00	0.00	0.15	0.00	0.19	0.00	0.00	0.00	0.00	0.00	0.00	0.00	0.00	0.11	0.00	0.00	0.00	0.13	0.00	0.00
TCN2	0.00	0.00	0.00	0.12	0.00	0.11	0.00	0.00	0.00	0.00	0.00	0.00	0.00	0.00	0.13	0.00	0.00	0.00	0.24	0.00	0.00
VHL	0.00	0.00	0.00	0.00	0.00	0.00	0.00	0.16	0.14	0.00	0.00	0.00	0.00	0.00	0.15	0.00	0.00	0.00	0.11	0.00	0.00
SLC19A1	0.00	0.00	0.00	0.00	0.00	0.11	0.00	0.00	0.00	0.00	0.11	0.00	0.00	0.00	0.12	0.00	0.00	0.00	0.23	0.00	0.00
CLF	0.00	0.00	0.00	0.13	0.00	0.16	0.00	0.00	0.00	0.00	0.00	0.00	0.00	0.00	0.00	0.00	0.13	0.00	0.16	0.00	0.00
PSMC1P9	0.00	0.00	0.00	0.11	0.00	0.12	0.00	0.00	0.00	0.00	0.00	0.00	0.00	0.00	0.00	0.00	0.18	0.00	0.16	0.00	0.00
C2ORF43	0.00	0.00	0.00	0.00	0.00	0.11	0.00	0.11	0.00	0.00	0.00	0.00	0.00	0.00	0.00	0.00	0.18	0.00	0.14	0.00	0.00
WDR72	0.00	0.00	0.00	0.15	0.00	0.20	0.00	0.00	0.00	0.00	0.00	0.00	0.00	0.00	0.00	0.00	0.37	0.00	0.00	0.25	0.00
IDDM17	0.00	0.00	0.00	0.11	0.00	0.17	0.00	0.00	0.00	0.00	0.00	0.00	0.00	0.00	0.00	0.00	0.34	0.00	0.00	0.14	0.00
HHEX	0.00	0.00	0.00	0.10	0.00	0.00	0.00	0.14	0.00	0.00	0.00	0.00	0.00	0.00	0.00	0.00	0.30	0.00	0.00	0.21	0.00
WTS	0.00	0.00	0.00	0.00	0.00	0.18	0.00	0.00	0.00	0.00	0.00	0.14	0.00	0.00	0.00	0.00	0.11	0.00	0.00	0.12	0.00
CHDS1	0.00	0.00	0.00	0.00	0.00	0.16	0.00	0.00	0.00	0.00	0.00	0.00	0.00	0.00	0.00	0.00	0.38	0.00	0.12	0.21	0.00
HDLCQ1	0.00	0.00	0.00	0.00	0.00	0.14	0.00	0.00	0.00	0.00	0.00	0.00	0.00	0.00	0.00	0.00	0.21	0.00	0.20	0.11	0.00
HNF1A	0.00	0.00	0.00	0.00	0.00	0.00	0.00	0.16	0.00	0.00	0.00	0.00	0.00	0.00	0.00	0.00	0.24	0.00	0.10	0.17	0.00
IFT43	0.00	0.00	0.00	0.15	0.00	0.40	0.00	0.00	0.00	0.00	0.00	0.11	0.00	0.00	0.00	0.00	0.00	0.00	0.00	0.00	0.12
HPT	0.00	0.00	0.00	0.13	0.00	0.29	0.00	0.00	0.00	0.00	0.00	0.12	0.00	0.00	0.00	0.00	0.00	0.00	0.00	0.00	0.12
DNAH10	0.00	0.00	0.00	0.14	0.00	0.35	0.00	0.00	0.00	0.00	0.00	0.15	0.00	0.00	0.00	0.00	0.00	0.00	0.00	0.00	0.15
DNAH6	0.00	0.00	0.00	0.14	0.00	0.35	0.00	0.00	0.00	0.00	0.00	0.15	0.00	0.00	0.00	0.00	0.00	0.00	0.00	0.00	0.15
GJA6P	0.00	0.00	0.00	0.22	0.00	0.42	0.00	0.00	0.00	0.00	0.00	0.14	0.00	0.00	0.00	0.00	0.00	0.00	0.00	0.00	0.21
C9ORF128	0.00	0.00	0.00	0.12	0.00	0.40	0.00	0.00	0.00	0.00	0.00	0.00	0.00	0.00	0.11	0.00	0.00	0.00	0.00	0.00	0.23

NSUN7	0.00	0.00	0.00	0.15	0.00	0.27	0.00	0.00	0.00	0.00	0.00	0.00	0.00	0.00	0.13	0.00	0.00	0.00	0.00	0.00	0.14
LGMD1H	0.00	0.00	0.00	0.00	0.00	0.12	0.00	0.00	0.16	0.00	0.00	0.00	0.00	0.00	0.00	0.00	0.11	0.00	0.00	0.00	0.17
ANE1	0.00	0.00	0.00	0.00	0.00	0.13	0.00	0.00	0.00	0.00	0.00	0.00	0.00	0.00	0.11	0.00	0.00	0.13	0.00	0.00	0.27
TRNV	0.00	0.00	0.00	0.00	0.00	0.15	0.00	0.00	0.00	0.00	0.00	0.00	0.00	0.00	0.00	0.00	0.00	0.20	0.14	0.00	0.42
TRNF	0.00	0.00	0.00	0.00	0.00	0.17	0.00	0.00	0.00	0.00	0.00	0.00	0.00	0.00	0.00	0.00	0.00	0.24	0.19	0.00	0.55
TRNP	0.00	0.00	0.00	0.00	0.00	0.00	0.00	0.00	0.00	0.00	0.00	0.00	0.00	0.00	0.00	0.00	0.17	0.14	0.16	0.00	0.32
SLC25A5P3	0.00	0.00	0.00	0.00	0.00	0.12	0.00	0.00	0.00	0.00	0.00	0.00	0.00	0.00	0.00	0.00	0.44	0.00	0.00	0.18	0.13
DYX7	0.00	0.00	0.17	0.18	0.00	0.00	0.17	0.00	0.00	0.00	0.00	0.00	0.00	0.00	0.00	0.00	0.00	0.00	0.00	0.00	0.00
SLC25A46	0.00	0.00	0.00	0.12	0.00	0.31	0.00	0.15	0.00	0.00	0.00	0.00	0.00	0.00	0.00	0.00	0.00	0.00	0.00	0.00	0.00
PRSS3P2	0.00	0.00	0.00	0.16	0.00	0.31	0.00	0.11	0.00	0.00	0.00	0.00	0.00	0.00	0.00	0.00	0.00	0.00	0.00	0.00	0.00
SBK2	0.00	0.00	0.00	0.12	0.00	0.37	0.00	0.26	0.00	0.00	0.00	0.00	0.00	0.00	0.00	0.00	0.00	0.00	0.00	0.00	0.00
FRA16B	0.00	0.00	0.00	0.18	0.00	0.29	0.00	0.13	0.00	0.00	0.00	0.00	0.00	0.00	0.00	0.00	0.00	0.00	0.00	0.00	0.00
SLX4	0.00	0.00	0.00	0.13	0.00	0.30	0.00	0.10	0.00	0.00	0.00	0.00	0.00	0.00	0.00	0.00	0.00	0.00	0.00	0.00	0.00
HPC5	0.00	0.00	0.00	0.18	0.00	0.27	0.00	0.12	0.00	0.00	0.00	0.00	0.00	0.00	0.00	0.00	0.00	0.00	0.00	0.00	0.00
MSH3	0.00	0.00	0.00	0.10	0.00	0.15	0.00	0.13	0.00	0.00	0.00	0.00	0.00	0.00	0.00	0.00	0.00	0.00	0.00	0.00	0.00
ERVW-5	0.00	0.00	0.00	0.15	0.00	0.29	0.00	0.13	0.00	0.00	0.00	0.00	0.00	0.00	0.00	0.00	0.00	0.00	0.00	0.00	0.00
PSMA8	0.00	0.00	0.00	0.13	0.00	0.25	0.00	0.18	0.00	0.00	0.00	0.00	0.00	0.00	0.00	0.00	0.00	0.00	0.00	0.00	0.00
ERVW-3	0.00	0.00	0.00	0.15	0.00	0.29	0.00	0.13	0.00	0.00	0.00	0.00	0.00	0.00	0.00	0.00	0.00	0.00	0.00	0.00	0.00
URB2	0.00	0.00	0.00	0.17	0.00	0.28	0.00	0.11	0.00	0.00	0.00	0.00	0.00	0.00	0.00	0.00	0.00	0.00	0.00	0.00	0.00
MASTL	0.00	0.00	0.00	0.20	0.00	0.31	0.00	0.16	0.00	0.00	0.00	0.00	0.00	0.00	0.00	0.00	0.00	0.00	0.00	0.00	0.00
ELAC2	0.00	0.00	0.00	0.14	0.00	0.21	0.00	0.14	0.00	0.00	0.00	0.00	0.00	0.00	0.00	0.00	0.00	0.00	0.00	0.00	0.00
RAB41	0.00	0.00	0.00	0.16	0.00	0.23	0.00	0.15	0.00	0.00	0.00	0.00	0.00	0.00	0.00	0.00	0.00	0.00	0.00	0.00	0.00
FRA6F	0.00	0.00	0.00	0.14	0.00	0.39	0.00	0.21	0.00	0.00	0.00	0.00	0.00	0.00	0.00	0.00	0.00	0.00	0.00	0.00	0.00
FMR1-AS1	0.00	0.00	0.00	0.15	0.00	0.29	0.00	0.20	0.00	0.00	0.00	0.00	0.00	0.00	0.00	0.00	0.00	0.00	0.00	0.00	0.00
TCO	0.00	0.00	0.00	0.14	0.00	0.24	0.00	0.17	0.00	0.00	0.00	0.00	0.00	0.00	0.00	0.00	0.00	0.00	0.00	0.00	0.00
C17ORF79	0.00	0.00	0.00	0.24	0.00	0.38	0.00	0.28	0.00	0.00	0.00	0.00	0.00	0.00	0.00	0.00	0.00	0.00	0.00	0.00	0.00
TNR1	0.00	0.00	0.00	0.16	0.00	0.33	0.00	0.00	0.12	0.00	0.00	0.00	0.00	0.00	0.00	0.00	0.00	0.00	0.00	0.00	0.00
ATFB1	0.00	0.00	0.00	0.10	0.00	0.22	0.00	0.00	0.13	0.00	0.00	0.00	0.00	0.00	0.00	0.00	0.00	0.00	0.00	0.00	0.00
TGM2	0.00	0.00	0.00	0.12	0.00	0.00	0.00	0.14	0.21	0.00	0.00	0.00	0.00	0.00	0.00	0.00	0.00	0.00	0.00	0.00	0.00
ERVK-22	0.00	0.00	0.00	0.00	0.00	0.16	0.00	0.13	0.22	0.00	0.00	0.00	0.00	0.00	0.00	0.00	0.00	0.00	0.00	0.00	0.00
ERVK-23	0.00	0.00	0.00	0.00	0.00	0.16	0.00	0.13	0.22	0.00	0.00	0.00	0.00	0.00	0.00	0.00	0.00	0.00	0.00	0.00	0.00
MIR659	0.00	0.00	0.00	0.00	0.00	0.16	0.00	0.19	0.20	0.00	0.00	0.00	0.00	0.00	0.00	0.00	0.00	0.00	0.00	0.00	0.00
ASTE1	0.00	0.00	0.00	0.00	0.00	0.11	0.00	0.23	0.18	0.00	0.00	0.00	0.00	0.00	0.00	0.00	0.00	0.00	0.00	0.00	0.00
BMND4	0.00	0.00	0.00	0.11	0.00	0.14	0.00	0.00	0.00	0.10	0.00	0.00	0.00	0.00	0.00	0.00	0.00	0.00	0.00	0.00	0.00
BMND5	0.00	0.00	0.00	0.11	0.00	0.14	0.00	0.00	0.00	0.10	0.00	0.00	0.00	0.00	0.00	0.00	0.00	0.00	0.00	0.00	0.00
ND4L	0.00	0.00	0.00	0.13	0.00	0.23	0.00	0.00	0.00	0.00	0.14	0.00	0.00	0.00	0.00	0.00	0.00	0.00	0.00	0.00	0.00
STRCP1	0.00	0.00	0.00	0.11	0.00	0.27	0.00	0.00	0.00	0.00	0.14	0.00	0.00	0.00	0.00	0.00	0.00	0.00	0.00	0.00	0.00
IGKV4-1	0.00	0.00	0.00	0.22	0.00	0.40	0.00	0.00	0.00	0.00	0.13	0.00	0.00	0.00	0.00	0.00	0.00	0.00	0.00	0.00	0.00
PIGFP1	0.00	0.00	0.00	0.20	0.00	0.40	0.00	0.00	0.00	0.00	0.21	0.00	0.00	0.00	0.00	0.00	0.00	0.00	0.00	0.00	0.00
DFNB17	0.00	0.00	0.00	0.11	0.00	0.26	0.00	0.00	0.00	0.00	0.10	0.00	0.00	0.00	0.00	0.00	0.00	0.00	0.00	0.00	0.00

PPIP5K1P1	0.00	0.00	0.00	0.11	0.00	0.27	0.00	0.00	0.00	0.00	0.14	0.00	0.00	0.00	0.00	0.00	0.00	0.00	0.00	0.00	0.00
PIGCP1	0.00	0.00	0.00	0.19	0.00	0.41	0.00	0.00	0.00	0.00	0.22	0.00	0.00	0.00	0.00	0.00	0.00	0.00	0.00	0.00	0.00
IGHV4-30-4	0.00	0.00	0.00	0.21	0.00	0.33	0.00	0.00	0.00	0.00	0.14	0.00	0.00	0.00	0.00	0.00	0.00	0.00	0.00	0.00	0.00
ACBD5	0.00	0.00	0.00	0.28	0.00	0.55	0.00	0.00	0.00	0.00	0.12	0.00	0.00	0.00	0.00	0.00	0.00	0.00	0.00	0.00	0.00
PIGAP1	0.00	0.00	0.00	0.17	0.00	0.43	0.00	0.00	0.00	0.00	0.12	0.00	0.00	0.00	0.00	0.00	0.00	0.00	0.00	0.00	0.00
OTOGL	0.00	0.00	0.00	0.19	0.00	0.29	0.00	0.00	0.00	0.00	0.14	0.00	0.00	0.00	0.00	0.00	0.00	0.00	0.00	0.00	0.00
DKBI	0.00	0.00	0.00	0.11	0.00	0.21	0.00	0.00	0.00	0.00	0.11	0.00	0.00	0.00	0.00	0.00	0.00	0.00	0.00	0.00	0.00
SPG29	0.00	0.00	0.00	0.13	0.00	0.28	0.00	0.00	0.00	0.00	0.13	0.00	0.00	0.00	0.00	0.00	0.00	0.00	0.00	0.00	0.00
VWSM	0.00	0.00	0.00	0.14	0.00	0.25	0.00	0.00	0.00	0.00	0.00	0.10	0.00	0.00	0.00	0.00	0.00	0.00	0.00	0.00	0.00
AA06	0.00	0.00	0.00	0.12	0.00	0.31	0.00	0.00	0.00	0.00	0.00	0.11	0.00	0.00	0.00	0.00	0.00	0.00	0.00	0.00	0.00
BP29	0.00	0.00	0.00	0.14	0.00	0.30	0.00	0.00	0.00	0.00	0.00	0.10	0.00	0.00	0.00	0.00	0.00	0.00	0.00	0.00	0.00
ANOP1	0.00	0.00	0.00	0.16	0.00	0.41	0.00	0.00	0.00	0.00	0.00	0.10	0.00	0.00	0.00	0.00	0.00	0.00	0.00	0.00	0.00
HERC2P5	0.00	0.00	0.00	0.17	0.00	0.33	0.00	0.00	0.00	0.00	0.00	0.12	0.00	0.00	0.00	0.00	0.00	0.00	0.00	0.00	0.00
LOC100500719	0.00	0.00	0.00	0.16	0.00	0.38	0.00	0.00	0.00	0.00	0.00	0.13	0.00	0.00	0.00	0.00	0.00	0.00	0.00	0.00	0.00
MRX28	0.00	0.00	0.00	0.12	0.00	0.29	0.00	0.00	0.00	0.00	0.00	0.17	0.00	0.00	0.00	0.00	0.00	0.00	0.00	0.00	0.00
FRA12A	0.00	0.00	0.00	0.19	0.00	0.33	0.00	0.00	0.00	0.00	0.00	0.13	0.00	0.00	0.00	0.00	0.00	0.00	0.00	0.00	0.00
MRX80	0.00	0.00	0.00	0.12	0.00	0.25	0.00	0.00	0.00	0.00	0.00	0.19	0.00	0.00	0.00	0.00	0.00	0.00	0.00	0.00	0.00
DYT4	0.00	0.00	0.00	0.12	0.00	0.27	0.00	0.00	0.00	0.00	0.00	0.13	0.00	0.00	0.00	0.00	0.00	0.00	0.00	0.00	0.00
TAPVR1	0.00	0.00	0.00	0.25	0.00	0.46	0.00	0.00	0.00	0.00	0.00	0.12	0.00	0.00	0.00	0.00	0.00	0.00	0.00	0.00	0.00
DURS1	0.00	0.00	0.00	0.11	0.00	0.31	0.00	0.00	0.00	0.00	0.00	0.13	0.00	0.00	0.00	0.00	0.00	0.00	0.00	0.00	0.00
CGF1	0.00	0.00	0.00	0.10	0.00	0.16	0.00	0.00	0.00	0.00	0.00	0.17	0.00	0.00	0.00	0.00	0.00	0.00	0.00	0.00	0.00
RPL3L	0.00	0.00	0.00	0.24	0.00	0.49	0.00	0.00	0.00	0.00	0.00	0.16	0.00	0.00	0.00	0.00	0.00	0.00	0.00	0.00	0.00
ZNF280D	0.00	0.00	0.00	0.11	0.00	0.24	0.00	0.00	0.00	0.00	0.00	0.14	0.00	0.00	0.00	0.00	0.00	0.00	0.00	0.00	0.00
LOC440786	0.00	0.00	0.00	0.21	0.00	0.38	0.00	0.00	0.00	0.00	0.00	0.15	0.00	0.00	0.00	0.00	0.00	0.00	0.00	0.00	0.00
HPE1	0.00	0.00	0.00	0.18	0.00	0.43	0.00	0.00	0.00	0.00	0.00	0.15	0.00	0.00	0.00	0.00	0.00	0.00	0.00	0.00	0.00
UBE3AP1	0.00	0.00	0.00	0.16	0.00	0.28	0.00	0.00	0.00	0.00	0.00	0.16	0.00	0.00	0.00	0.00	0.00	0.00	0.00	0.00	0.00
UBE3AP2	0.00	0.00	0.00	0.16	0.00	0.28	0.00	0.00	0.00	0.00	0.00	0.16	0.00	0.00	0.00	0.00	0.00	0.00	0.00	0.00	0.00
PAR5	0.00	0.00	0.00	0.12	0.00	0.20	0.00	0.00	0.00	0.00	0.00	0.14	0.00	0.00	0.00	0.00	0.00	0.00	0.00	0.00	0.00
FAUP1	0.00	0.00	0.00	0.18	0.00	0.41	0.00	0.00	0.00	0.00	0.00	0.16	0.00	0.00	0.00	0.00	0.00	0.00	0.00	0.00	0.00
GTF2IP1	0.00	0.00	0.00	0.21	0.00	0.38	0.00	0.00	0.00	0.00	0.00	0.12	0.00	0.00	0.00	0.00	0.00	0.00	0.00	0.00	0.00
LOC100093631	0.00	0.00	0.00	0.21	0.00	0.38	0.00	0.00	0.00	0.00	0.00	0.12	0.00	0.00	0.00	0.00	0.00	0.00	0.00	0.00	0.00
FRA10AC1	0.00	0.00	0.00	0.19	0.00	0.40	0.00	0.00	0.00	0.00	0.00	0.12	0.00	0.00	0.00	0.00	0.00	0.00	0.00	0.00	0.00
RAB9BP1	0.00	0.00	0.00	0.23	0.00	0.47	0.00	0.00	0.00	0.00	0.00	0.12	0.00	0.00	0.00	0.00	0.00	0.00	0.00	0.00	0.00
ZNF81	0.00	0.00	0.00	0.14	0.00	0.33	0.00	0.00	0.00	0.00	0.00	0.12	0.00	0.00	0.00	0.00	0.00	0.00	0.00	0.00	0.00
FRA10A	0.00	0.00	0.00	0.19	0.00	0.40	0.00	0.00	0.00	0.00	0.00	0.12	0.00	0.00	0.00	0.00	0.00	0.00	0.00	0.00	0.00
BDMR	0.00	0.00	0.00	0.11	0.00	0.20	0.00	0.00	0.00	0.00	0.00	0.12	0.00	0.00	0.00	0.00	0.00	0.00	0.00	0.00	0.00
LIPB	0.00	0.00	0.00	0.18	0.00	0.35	0.00	0.00	0.00	0.00	0.00	0.11	0.00	0.00	0.00	0.00	0.00	0.00	0.00	0.00	0.00
USP17	0.00	0.00	0.00	0.21	0.00	0.43	0.00	0.00	0.00	0.00	0.00	0.11	0.00	0.00	0.00	0.00	0.00	0.00	0.00	0.00	0.00
HERC2P8	0.00	0.00	0.00	0.17	0.00	0.33	0.00	0.00	0.00	0.00	0.00	0.12	0.00	0.00	0.00	0.00	0.00	0.00	0.00	0.00	0.00

HERC2P4	0.00	0.00	0.00	0.17	0.00	0.33	0.00	0.00	0.00	0.00	0.00	0.12	0.00	0.00	0.00	0.00	0.00	0.00	0.00	0.00	0.00
PKD1P2	0.00	0.00	0.00	0.17	0.00	0.35	0.00	0.00	0.00	0.00	0.00	0.12	0.00	0.00	0.00	0.00	0.00	0.00	0.00	0.00	0.00
PKD1P4	0.00	0.00	0.00	0.17	0.00	0.35	0.00	0.00	0.00	0.00	0.00	0.12	0.00	0.00	0.00	0.00	0.00	0.00	0.00	0.00	0.00
PKD1P6	0.00	0.00	0.00	0.17	0.00	0.35	0.00	0.00	0.00	0.00	0.00	0.12	0.00	0.00	0.00	0.00	0.00	0.00	0.00	0.00	0.00
PKD1P1	0.00	0.00	0.00	0.17	0.00	0.35	0.00	0.00	0.00	0.00	0.00	0.12	0.00	0.00	0.00	0.00	0.00	0.00	0.00	0.00	0.00
PKD1P3	0.00	0.00	0.00	0.17	0.00	0.35	0.00	0.00	0.00	0.00	0.00	0.12	0.00	0.00	0.00	0.00	0.00	0.00	0.00	0.00	0.00
LOC100128651	0.00	0.00	0.00	0.17	0.00	0.40	0.00	0.00	0.00	0.00	0.00	0.12	0.00	0.00	0.00	0.00	0.00	0.00	0.00	0.00	0.00
PKD1P5	0.00	0.00	0.00	0.17	0.00	0.35	0.00	0.00	0.00	0.00	0.00	0.12	0.00	0.00	0.00	0.00	0.00	0.00	0.00	0.00	0.00
SALL1P1	0.00	0.00	0.00	0.18	0.00	0.49	0.00	0.00	0.00	0.00	0.00	0.11	0.00	0.00	0.00	0.00	0.00	0.00	0.00	0.00	0.00
MYO1H	0.00	0.00	0.00	0.14	0.00	0.25	0.00	0.00	0.00	0.00	0.00	0.10	0.00	0.00	0.00	0.00	0.00	0.00	0.00	0.00	0.00
MRX15	0.00	0.00	0.00	0.00	0.00	0.19	0.00	0.00	0.00	0.00	0.00	0.18	0.11	0.00	0.00	0.00	0.00	0.00	0.00	0.00	0.00
MRX72	0.00	0.00	0.00	0.00	0.00	0.22	0.00	0.00	0.00	0.00	0.00	0.18	0.12	0.00	0.00	0.00	0.00	0.00	0.00	0.00	0.00
MRX14	0.00	0.00	0.00	0.00	0.00	0.26	0.00	0.00	0.00	0.00	0.00	0.21	0.13	0.00	0.00	0.00	0.00	0.00	0.00	0.00	0.00
ADHD2	0.00	0.00	0.00	0.00	0.00	0.12	0.00	0.00	0.00	0.00	0.00	0.10	0.13	0.00	0.00	0.00	0.00	0.00	0.00	0.00	0.00
ADHD3	0.00	0.00	0.00	0.00	0.00	0.12	0.00	0.00	0.00	0.00	0.00	0.10	0.13	0.00	0.00	0.00	0.00	0.00	0.00	0.00	0.00
MRX50	0.00	0.00	0.00	0.00	0.00	0.22	0.00	0.00	0.00	0.00	0.00	0.17	0.11	0.00	0.00	0.00	0.00	0.00	0.00	0.00	0.00
MRX1	0.00	0.00	0.00	0.00	0.00	0.18	0.00	0.00	0.00	0.00	0.00	0.17	0.10	0.00	0.00	0.00	0.00	0.00	0.00	0.00	0.00
MRX52	0.00	0.00	0.00	0.00	0.00	0.12	0.00	0.00	0.00	0.00	0.00	0.16	0.13	0.00	0.00	0.00	0.00	0.00	0.00	0.00	0.00
MRX42	0.00	0.00	0.00	0.00	0.00	0.15	0.00	0.00	0.00	0.00	0.00	0.16	0.11	0.00	0.00	0.00	0.00	0.00	0.00	0.00	0.00
MRX27	0.00	0.00	0.00	0.00	0.00	0.20	0.00	0.00	0.00	0.00	0.00	0.17	0.11	0.00	0.00	0.00	0.00	0.00	0.00	0.00	0.00
MRX31	0.00	0.00	0.00	0.00	0.00	0.17	0.00	0.00	0.00	0.00	0.00	0.17	0.11	0.00	0.00	0.00	0.00	0.00	0.00	0.00	0.00
ADHD4	0.00	0.00	0.00	0.00	0.00	0.12	0.00	0.00	0.00	0.00	0.00	0.10	0.13	0.00	0.00	0.00	0.00	0.00	0.00	0.00	0.00
MRX78	0.00	0.00	0.00	0.00	0.00	0.12	0.00	0.00	0.00	0.00	0.00	0.20	0.13	0.00	0.00	0.00	0.00	0.00	0.00	0.00	0.00
EIG3	0.00	0.00	0.00	0.11	0.00	0.11	0.00	0.00	0.00	0.00	0.00	0.00	0.00	0.11	0.00	0.00	0.00	0.00	0.00	0.00	0.00
CREB1	0.00	0.00	0.00	0.00	0.00	0.00	0.00	0.11	0.11	0.00	0.00	0.00	0.00	0.12	0.00	0.00	0.00	0.00	0.00	0.00	0.00
MRXSA	0.00	0.00	0.00	0.00	0.00	0.16	0.00	0.00	0.00	0.00	0.00	0.16	0.00	0.12	0.00	0.00	0.00	0.00	0.00	0.00	0.00
ETL2	0.00	0.00	0.00	0.00	0.00	0.00	0.00	0.00	0.00	0.00	0.00	0.15	0.20	0.31	0.00	0.00	0.00	0.00	0.00	0.00	0.00
RNF212	0.00	0.00	0.00	0.17	0.00	0.34	0.00	0.00	0.00	0.00	0.00	0.00	0.00	0.00	0.12	0.00	0.00	0.00	0.00	0.00	0.00
CDRT1	0.00	0.00	0.00	0.12	0.00	0.34	0.00	0.00	0.00	0.00	0.00	0.00	0.00	0.00	0.14	0.00	0.00	0.00	0.00	0.00	0.00
DPCD	0.00	0.00	0.00	0.16	0.00	0.28	0.00	0.00	0.00	0.00	0.00	0.00	0.00	0.00	0.13	0.00	0.00	0.00	0.00	0.00	0.00
COL4A5	0.00	0.00	0.00	0.10	0.00	0.11	0.00	0.00	0.00	0.00	0.00	0.00	0.00	0.00	0.11	0.00	0.00	0.00	0.00	0.00	0.00
AVSD1	0.00	0.00	0.00	0.13	0.00	0.25	0.00	0.00	0.00	0.00	0.00	0.00	0.00	0.00	0.12	0.00	0.00	0.00	0.00	0.00	0.00
EVC	0.00	0.00	0.00	0.18	0.00	0.28	0.00	0.00	0.00	0.00	0.00	0.00	0.00	0.00	0.14	0.00	0.00	0.00	0.00	0.00	0.00
C2ORF71	0.00	0.00	0.00	0.15	0.00	0.28	0.00	0.00	0.00	0.00	0.00	0.00	0.00	0.00	0.12	0.00	0.00	0.00	0.00	0.00	0.00
BDET	0.00	0.00	0.00	0.15	0.00	0.32	0.00	0.00	0.00	0.00	0.00	0.00	0.00	0.00	0.11	0.00	0.00	0.00	0.00	0.00	0.00
SPG12	0.00	0.00	0.00	0.11	0.00	0.25	0.00	0.00	0.00	0.00	0.00	0.00	0.00	0.00	0.14	0.00	0.00	0.00	0.00	0.00	0.00
SPTA1	0.00	0.00	0.00	0.15	0.00	0.16	0.00	0.00	0.00	0.00	0.00	0.00	0.00	0.00	0.13	0.00	0.00	0.00	0.00	0.00	0.00
RBM20	0.00	0.00	0.00	0.15	0.00	0.33	0.00	0.00	0.00	0.00	0.00	0.00	0.00	0.00	0.12	0.00	0.00	0.00	0.00	0.00	0.00
LCA5L	0.00	0.00	0.00	0.15	0.00	0.34	0.00	0.00	0.00	0.00	0.00	0.00	0.00	0.00	0.13	0.00	0.00	0.00	0.00	0.00	0.00
KLHL10	0.00	0.00	0.00	0.15	0.00	0.33	0.00	0.00	0.00	0.00	0.00	0.00	0.00	0.00	0.11	0.00	0.00	0.00	0.00	0.00	0.00

SMA4	0.00	0.00	0.00	0.17	0.00	0.33	0.00	0.00	0.00	0.00	0.00	0.00	0.00	0.00	0.17	0.00	0.00	0.00	0.00	0.00	0.00
LRTOMT	0.00	0.00	0.00	0.12	0.00	0.28	0.00	0.00	0.00	0.00	0.00	0.00	0.00	0.00	0.11	0.00	0.00	0.00	0.00	0.00	0.00
CMAL	0.00	0.00	0.00	0.10	0.00	0.16	0.00	0.00	0.00	0.00	0.00	0.00	0.00	0.00	0.14	0.00	0.00	0.00	0.00	0.00	0.00
ERCC6	0.00	0.00	0.00	0.10	0.00	0.11	0.00	0.00	0.00	0.00	0.00	0.00	0.00	0.00	0.12	0.00	0.00	0.00	0.00	0.00	0.00
CACD	0.00	0.00	0.00	0.14	0.00	0.27	0.00	0.00	0.00	0.00	0.00	0.00	0.00	0.00	0.12	0.00	0.00	0.00	0.00	0.00	0.00
ETM2	0.00	0.00	0.00	0.13	0.00	0.22	0.00	0.00	0.00	0.00	0.00	0.00	0.00	0.00	0.11	0.00	0.00	0.00	0.00	0.00	0.00
SDTY1	0.00	0.00	0.00	0.11	0.00	0.22	0.00	0.00	0.00	0.00	0.00	0.00	0.00	0.00	0.12	0.00	0.00	0.00	0.00	0.00	0.00
DBH-AS1	0.00	0.00	0.00	0.17	0.00	0.38	0.00	0.00	0.00	0.00	0.00	0.00	0.00	0.00	0.10	0.00	0.00	0.00	0.00	0.00	0.00
IDSP1	0.00	0.00	0.00	0.15	0.00	0.35	0.00	0.00	0.00	0.00	0.00	0.00	0.00	0.00	0.11	0.00	0.00	0.00	0.00	0.00	0.00
KRT6C	0.00	0.00	0.00	0.21	0.00	0.35	0.00	0.00	0.00	0.00	0.00	0.00	0.00	0.00	0.10	0.00	0.00	0.00	0.00	0.00	0.00
ENAM	0.00	0.00	0.00	0.17	0.00	0.25	0.00	0.00	0.00	0.00	0.00	0.00	0.00	0.00	0.14	0.00	0.00	0.00	0.00	0.00	0.00
LRRC37B	0.00	0.00	0.00	0.11	0.00	0.25	0.00	0.00	0.00	0.00	0.00	0.00	0.00	0.00	0.14	0.00	0.00	0.00	0.00	0.00	0.00
FEOM3	0.00	0.00	0.00	0.13	0.00	0.27	0.00	0.00	0.00	0.00	0.00	0.00	0.00	0.00	0.14	0.00	0.00	0.00	0.00	0.00	0.00
KRT38	0.00	0.00	0.00	0.19	0.00	0.35	0.00	0.00	0.00	0.00	0.00	0.00	0.00	0.00	0.12	0.00	0.00	0.00	0.00	0.00	0.00
LOC440792	0.00	0.00	0.00	0.13	0.00	0.22	0.00	0.00	0.00	0.00	0.00	0.00	0.00	0.00	0.11	0.00	0.00	0.00	0.00	0.00	0.00
RSPH9	0.00	0.00	0.00	0.12	0.00	0.28	0.00	0.00	0.00	0.00	0.00	0.00	0.00	0.00	0.11	0.00	0.00	0.00	0.00	0.00	0.00
DNAAF1	0.00	0.00	0.00	0.16	0.00	0.32	0.00	0.00	0.00	0.00	0.00	0.00	0.00	0.00	0.13	0.00	0.00	0.00	0.00	0.00	0.00
KERSMCR	0.00	0.00	0.00	0.10	0.00	0.25	0.00	0.00	0.00	0.00	0.00	0.00	0.00	0.00	0.13	0.00	0.00	0.00	0.00	0.00	0.00
KRT3	0.00	0.00	0.00	0.15	0.00	0.31	0.00	0.00	0.00	0.00	0.00	0.00	0.00	0.00	0.11	0.00	0.00	0.00	0.00	0.00	0.00
PRCD	0.00	0.00	0.00	0.14	0.00	0.30	0.00	0.00	0.00	0.00	0.00	0.00	0.00	0.00	0.11	0.00	0.00	0.00	0.00	0.00	0.00
LOC653080	0.00	0.00	0.00	0.12	0.00	0.25	0.00	0.00	0.00	0.00	0.00	0.00	0.00	0.00	0.14	0.00	0.00	0.00	0.00	0.00	0.00
OFC3	0.00	0.00	0.00	0.13	0.00	0.18	0.00	0.00	0.00	0.00	0.00	0.00	0.00	0.00	0.13	0.00	0.00	0.00	0.00	0.00	0.00
HMU	0.00	0.00	0.00	0.12	0.00	0.30	0.00	0.00	0.00	0.00	0.00	0.00	0.00	0.00	0.10	0.00	0.00	0.00	0.00	0.00	0.00
ARVD3	0.00	0.00	0.00	0.11	0.00	0.19	0.00	0.00	0.00	0.00	0.00	0.00	0.00	0.00	0.12	0.00	0.00	0.00	0.00	0.00	0.00
KRT86	0.00	0.00	0.00	0.17	0.00	0.31	0.00	0.00	0.00	0.00	0.00	0.00	0.00	0.00	0.17	0.00	0.00	0.00	0.00	0.00	0.00
KRT9	0.00	0.00	0.00	0.17	0.00	0.30	0.00	0.00	0.00	0.00	0.00	0.00	0.00	0.00	0.14	0.00	0.00	0.00	0.00	0.00	0.00
NDIC	0.00	0.00	0.00	0.16	0.00	0.30	0.00	0.00	0.00	0.00	0.00	0.00	0.00	0.00	0.13	0.00	0.00	0.00	0.00	0.00	0.00
COL9A1	0.00	0.00	0.00	0.12	0.00	0.18	0.00	0.00	0.00	0.00	0.00	0.00	0.00	0.00	0.15	0.00	0.00	0.00	0.00	0.00	0.00
CCPSO	0.00	0.00	0.00	0.11	0.00	0.18	0.00	0.00	0.00	0.00	0.00	0.00	0.00	0.00	0.11	0.00	0.00	0.00	0.00	0.00	0.00
ARSDP1	0.00	0.00	0.00	0.15	0.00	0.30	0.00	0.00	0.00	0.00	0.00	0.00	0.00	0.00	0.12	0.00	0.00	0.00	0.00	0.00	0.00
HTS	0.00	0.00	0.00	0.13	0.00	0.19	0.00	0.00	0.00	0.00	0.00	0.00	0.00	0.00	0.12	0.00	0.00	0.00	0.00	0.00	0.00
ZNF286A	0.00	0.00	0.00	0.20	0.00	0.31	0.00	0.00	0.00	0.00	0.00	0.00	0.00	0.00	0.15	0.00	0.00	0.00	0.00	0.00	0.00
LCA9	0.00	0.00	0.00	0.12	0.00	0.29	0.00	0.00	0.00	0.00	0.00	0.00	0.00	0.00	0.11	0.00	0.00	0.00	0.00	0.00	0.00
NT5C3P1	0.00	0.00	0.00	0.11	0.00	0.31	0.00	0.00	0.00	0.00	0.00	0.00	0.00	0.00	0.10	0.00	0.00	0.00	0.00	0.00	0.00
RNR2	0.00	0.00	0.00	0.13	0.00	0.32	0.00	0.00	0.00	0.00	0.00	0.00	0.00	0.00	0.11	0.00	0.00	0.00	0.00	0.00	0.00
BRCA1P1	0.00	0.00	0.00	0.18	0.00	0.29	0.00	0.00	0.00	0.00	0.00	0.00	0.00	0.00	0.13	0.00	0.00	0.00	0.00	0.00	0.00
COL9A3	0.00	0.00	0.00	0.13	0.00	0.21	0.00	0.00	0.00	0.00	0.00	0.00	0.00	0.00	0.13	0.00	0.00	0.00	0.00	0.00	0.00
EYCL1	0.00	0.00	0.00	0.14	0.00	0.22	0.00	0.00	0.00	0.00	0.00	0.00	0.00	0.00	0.13	0.00	0.00	0.00	0.00	0.00	0.00
FTH1P10	0.00	0.00	0.00	0.19	0.00	0.29	0.00	0.00	0.00	0.00	0.00	0.00	0.00	0.00	0.13	0.00	0.00	0.00	0.00	0.00	0.00
KIAA1468	0.00	0.00	0.00	0.16	0.00	0.20	0.00	0.00	0.00	0.00	0.00	0.00	0.00	0.00	0.11	0.00	0.00	0.00	0.00	0.00	0.00

WDR36	0.00	0.00	0.00	0.16	0.00	0.25	0.00	0.00	0.00	0.00	0.00	0.00	0.00	0.00	0.13	0.00	0.00	0.00	0.00	0.00	0.00
RPL9P2	0.00	0.00	0.00	0.20	0.00	0.31	0.00	0.00	0.00	0.00	0.00	0.00	0.00	0.00	0.15	0.00	0.00	0.00	0.00	0.00	0.00
COL9A2	0.00	0.00	0.00	0.11	0.00	0.17	0.00	0.00	0.00	0.00	0.00	0.00	0.00	0.00	0.13	0.00	0.00	0.00	0.00	0.00	0.00
FAM46A	0.00	0.00	0.00	0.20	0.00	0.31	0.00	0.00	0.00	0.00	0.00	0.00	0.00	0.00	0.12	0.00	0.00	0.00	0.00	0.00	0.00
PBCRA1	0.00	0.00	0.00	0.11	0.00	0.25	0.00	0.00	0.00	0.00	0.00	0.00	0.00	0.00	0.12	0.00	0.00	0.00	0.00	0.00	0.00
MYP2	0.00	0.00	0.00	0.12	0.00	0.29	0.00	0.00	0.00	0.00	0.00	0.00	0.00	0.00	0.11	0.00	0.00	0.00	0.00	0.00	0.00
RP2	0.00	0.00	0.00	0.15	0.00	0.28	0.00	0.00	0.00	0.00	0.00	0.00	0.00	0.00	0.11	0.00	0.00	0.00	0.00	0.00	0.00
MHS2	0.00	0.00	0.00	0.18	0.00	0.26	0.00	0.00	0.00	0.00	0.00	0.00	0.00	0.00	0.13	0.00	0.00	0.00	0.00	0.00	0.00
EEC2	0.00	0.00	0.00	0.11	0.00	0.15	0.00	0.00	0.00	0.00	0.00	0.00	0.00	0.00	0.16	0.00	0.00	0.00	0.00	0.00	0.00
BMS1	0.00	0.00	0.00	0.18	0.00	0.39	0.00	0.00	0.00	0.00	0.00	0.00	0.00	0.00	0.16	0.00	0.00	0.00	0.00	0.00	0.00
NF1P1	0.00	0.00	0.00	0.12	0.00	0.24	0.00	0.00	0.00	0.00	0.00	0.00	0.00	0.00	0.10	0.00	0.00	0.00	0.00	0.00	0.00
NYS4	0.00	0.00	0.00	0.15	0.00	0.27	0.00	0.00	0.00	0.00	0.00	0.00	0.00	0.00	0.14	0.00	0.00	0.00	0.00	0.00	0.00
KRT2	0.00	0.00	0.00	0.16	0.00	0.23	0.00	0.00	0.00	0.00	0.00	0.00	0.00	0.00	0.15	0.00	0.00	0.00	0.00	0.00	0.00
EYS	0.00	0.00	0.00	0.15	0.00	0.23	0.00	0.00	0.00	0.00	0.00	0.00	0.00	0.00	0.13	0.00	0.00	0.00	0.00	0.00	0.00
MYO15B	0.00	0.00	0.00	0.18	0.00	0.42	0.00	0.00	0.00	0.00	0.00	0.00	0.00	0.00	0.11	0.00	0.00	0.00	0.00	0.00	0.00
DFNB81	0.00	0.00	0.00	0.10	0.00	0.24	0.00	0.00	0.00	0.00	0.00	0.00	0.00	0.00	0.11	0.00	0.00	0.00	0.00	0.00	0.00
OHDS	0.00	0.00	0.00	0.11	0.00	0.14	0.00	0.00	0.00	0.00	0.00	0.00	0.00	0.00	0.16	0.00	0.00	0.00	0.00	0.00	0.00
MYP1	0.00	0.00	0.00	0.11	0.00	0.24	0.00	0.00	0.00	0.00	0.00	0.00	0.00	0.00	0.10	0.00	0.00	0.00	0.00	0.00	0.00
ZNF385B	0.00	0.00	0.00	0.12	0.00	0.13	0.00	0.00	0.00	0.00	0.00	0.00	0.00	0.00	0.11	0.00	0.00	0.00	0.00	0.00	0.00
LRRC37BP1	0.00	0.00	0.00	0.12	0.00	0.26	0.00	0.00	0.00	0.00	0.00	0.00	0.00	0.00	0.14	0.00	0.00	0.00	0.00	0.00	0.00
NNO3	0.00	0.00	0.00	0.11	0.00	0.23	0.00	0.00	0.00	0.00	0.00	0.00	0.00	0.00	0.11	0.00	0.00	0.00	0.00	0.00	0.00
LCA5	0.00	0.00	0.00	0.11	0.00	0.24	0.00	0.00	0.00	0.00	0.00	0.00	0.00	0.00	0.12	0.00	0.00	0.00	0.00	0.00	0.00
RP1	0.00	0.00	0.00	0.17	0.00	0.27	0.00	0.00	0.00	0.00	0.00	0.00	0.00	0.00	0.13	0.00	0.00	0.00	0.00	0.00	0.00
RP23	0.00	0.00	0.00	0.13	0.00	0.27	0.00	0.00	0.00	0.00	0.00	0.00	0.00	0.00	0.10	0.00	0.00	0.00	0.00	0.00	0.00
NF1P2	0.00	0.00	0.00	0.12	0.00	0.24	0.00	0.00	0.00	0.00	0.00	0.00	0.00	0.00	0.10	0.00	0.00	0.00	0.00	0.00	0.00
PMS2	0.00	0.00	0.00	0.00	0.00	0.11	0.00	0.12	0.00	0.00	0.00	0.00	0.00	0.00	0.10	0.00	0.00	0.00	0.00	0.00	0.00
CSF1	0.00	0.00	0.00	0.12	0.00	0.00	0.00	0.00	0.13	0.00	0.00	0.00	0.00	0.00	0.12	0.00	0.00	0.00	0.00	0.00	0.00
C22DDELS	0.00	0.00	0.00	0.00	0.00	0.11	0.00	0.00	0.13	0.00	0.00	0.00	0.00	0.00	0.12	0.00	0.00	0.00	0.00	0.00	0.00
C9ORF72	0.00	0.00	0.00	0.00	0.00	0.15	0.00	0.00	0.11	0.00	0.00	0.00	0.00	0.00	0.12	0.00	0.00	0.00	0.00	0.00	0.00
ATM	0.00	0.00	0.00	0.00	0.00	0.00	0.00	0.11	0.11	0.00	0.00	0.00	0.00	0.00	0.12	0.00	0.00	0.00	0.00	0.00	0.00
RET	0.00	0.00	0.00	0.00	0.00	0.00	0.00	0.11	0.10	0.00	0.00	0.00	0.00	0.00	0.15	0.00	0.00	0.00	0.00	0.00	0.00
MIF	0.00	0.00	0.00	0.00	0.00	0.00	0.00	0.11	0.18	0.00	0.00	0.00	0.00	0.00	0.11	0.00	0.00	0.00	0.00	0.00	0.00
FOXN1	0.00	0.00	0.00	0.00	0.00	0.00	0.00	0.13	0.17	0.00	0.00	0.00	0.00	0.00	0.11	0.00	0.00	0.00	0.00	0.00	0.00
HPP1	0.00	0.00	0.00	0.00	0.00	0.21	0.00	0.00	0.00	0.00	0.11	0.00	0.00	0.00	0.11	0.00	0.00	0.00	0.00	0.00	0.00
SCN5A	0.00	0.00	0.00	0.00	0.00	0.00	0.00	0.00	0.15	0.00	0.11	0.00	0.00	0.00	0.11	0.00	0.00	0.00	0.00	0.00	0.00
PTCHD1-AS	0.00	0.00	0.00	0.00	0.00	0.19	0.00	0.00	0.00	0.00	0.00	0.16	0.00	0.00	0.13	0.00	0.00	0.00	0.00	0.00	0.00
SCA20	0.00	0.00	0.00	0.00	0.00	0.20	0.00	0.00	0.00	0.00	0.00	0.11	0.00	0.00	0.13	0.00	0.00	0.00	0.00	0.00	0.00
COHEN1	0.00	0.00	0.00	0.00	0.00	0.24	0.00	0.00	0.00	0.00	0.00	0.17	0.00	0.00	0.13	0.00	0.00	0.00	0.00	0.00	0.00
ANCR	0.00	0.00	0.00	0.00	0.00	0.18	0.00	0.00	0.00	0.00	0.00	0.14	0.00	0.00	0.10	0.00	0.00	0.00	0.00	0.00	0.00
ICCA	0.00	0.00	0.00	0.00	0.00	0.19	0.00	0.00	0.00	0.00	0.00	0.12	0.00	0.00	0.12	0.00	0.00	0.00	0.00	0.00	0.00

SPG14	0.00	0.00	0.00	0.00	0.00	0.11	0.00	0.00	0.00	0.00	0.00	0.11	0.00	0.00	0.15	0.00	0.00	0.00	0.00	0.00	0.00
MBS2	0.00	0.00	0.00	0.00	0.00	0.19	0.00	0.00	0.00	0.00	0.00	0.12	0.00	0.00	0.18	0.00	0.00	0.00	0.00	0.00	0.00
SCA25	0.00	0.00	0.00	0.00	0.00	0.21	0.00	0.00	0.00	0.00	0.00	0.13	0.00	0.00	0.18	0.00	0.00	0.00	0.00	0.00	0.00
COHEN2	0.00	0.00	0.00	0.00	0.00	0.23	0.00	0.00	0.00	0.00	0.00	0.17	0.00	0.00	0.11	0.00	0.00	0.00	0.00	0.00	0.00
SCA26	0.00	0.00	0.00	0.00	0.00	0.21	0.00	0.00	0.00	0.00	0.00	0.17	0.00	0.00	0.17	0.00	0.00	0.00	0.00	0.00	0.00
MRX77	0.00	0.00	0.00	0.00	0.00	0.25	0.00	0.00	0.00	0.00	0.00	0.17	0.00	0.00	0.10	0.00	0.00	0.00	0.00	0.00	0.00
PARK12	0.00	0.00	0.00	0.00	0.00	0.11	0.00	0.00	0.00	0.00	0.00	0.13	0.00	0.00	0.12	0.00	0.00	0.00	0.00	0.00	0.00
MRX35	0.00	0.00	0.00	0.00	0.00	0.26	0.00	0.00	0.00	0.00	0.00	0.16	0.00	0.00	0.10	0.00	0.00	0.00	0.00	0.00	0.00
MRT5	0.00	0.00	0.00	0.00	0.00	0.17	0.00	0.00	0.00	0.00	0.00	0.15	0.00	0.00	0.12	0.00	0.00	0.00	0.00	0.00	0.00
PAND2	0.00	0.00	0.00	0.00	0.00	0.17	0.00	0.00	0.00	0.00	0.00	0.11	0.00	0.00	0.11	0.00	0.00	0.00	0.00	0.00	0.00
SCA19	0.00	0.00	0.00	0.00	0.00	0.20	0.00	0.00	0.00	0.00	0.00	0.14	0.00	0.00	0.15	0.00	0.00	0.00	0.00	0.00	0.00
MRT10	0.00	0.00	0.00	0.00	0.00	0.17	0.00	0.00	0.00	0.00	0.00	0.15	0.00	0.00	0.12	0.00	0.00	0.00	0.00	0.00	0.00
SCA21	0.00	0.00	0.00	0.00	0.00	0.12	0.00	0.00	0.00	0.00	0.00	0.11	0.00	0.00	0.11	0.00	0.00	0.00	0.00	0.00	0.00
MRT9	0.00	0.00	0.00	0.00	0.00	0.17	0.00	0.00	0.00	0.00	0.00	0.15	0.00	0.00	0.12	0.00	0.00	0.00	0.00	0.00	0.00
HNB1	0.00	0.00	0.00	0.00	0.00	0.19	0.00	0.00	0.00	0.00	0.00	0.10	0.00	0.00	0.16	0.00	0.00	0.00	0.00	0.00	0.00
RAB40AL	0.00	0.00	0.00	0.00	0.00	0.18	0.00	0.00	0.00	0.00	0.00	0.16	0.00	0.00	0.12	0.00	0.00	0.00	0.00	0.00	0.00
MRT8	0.00	0.00	0.00	0.00	0.00	0.17	0.00	0.00	0.00	0.00	0.00	0.15	0.00	0.00	0.12	0.00	0.00	0.00	0.00	0.00	0.00
WTRS	0.00	0.00	0.00	0.00	0.00	0.18	0.00	0.00	0.00	0.00	0.00	0.16	0.00	0.00	0.12	0.00	0.00	0.00	0.00	0.00	0.00
MBS3	0.00	0.00	0.00	0.00	0.00	0.16	0.00	0.00	0.00	0.00	0.00	0.12	0.00	0.00	0.19	0.00	0.00	0.00	0.00	0.00	0.00
TUKLS	0.00	0.00	0.00	0.00	0.00	0.16	0.00	0.00	0.00	0.00	0.00	0.11	0.00	0.00	0.16	0.00	0.00	0.00	0.00	0.00	0.00
MRT12	0.00	0.00	0.00	0.00	0.00	0.17	0.00	0.00	0.00	0.00	0.00	0.15	0.00	0.00	0.12	0.00	0.00	0.00	0.00	0.00	0.00
SPG38	0.00	0.00	0.00	0.00	0.00	0.19	0.00	0.00	0.00	0.00	0.00	0.11	0.00	0.00	0.14	0.00	0.00	0.00	0.00	0.00	0.00
MHAC	0.00	0.00	0.00	0.00	0.00	0.17	0.00	0.00	0.00	0.00	0.00	0.10	0.00	0.00	0.11	0.00	0.00	0.00	0.00	0.00	0.00
MRT11	0.00	0.00	0.00	0.00	0.00	0.17	0.00	0.00	0.00	0.00	0.00	0.15	0.00	0.00	0.12	0.00	0.00	0.00	0.00	0.00	0.00
GJB6	0.00	0.00	0.00	0.11	0.00	0.00	0.00	0.00	0.00	0.00	0.00	0.00	0.12	0.00	0.13	0.00	0.00	0.00	0.00	0.00	0.00
ZLS	0.00	0.00	0.00	0.00	0.00	0.00	0.00	0.00	0.00	0.00	0.00	0.17	0.11	0.00	0.13	0.00	0.00	0.00	0.00	0.00	0.00
CMTX3	0.00	0.00	0.00	0.00	0.00	0.12	0.00	0.00	0.00	0.00	0.00	0.00	0.00	0.00	0.22	0.13	0.00	0.00	0.00	0.00	0.00
HMN7A	0.00	0.00	0.00	0.00	0.00	0.17	0.00	0.00	0.00	0.00	0.00	0.00	0.00	0.00	0.24	0.12	0.00	0.00	0.00	0.00	0.00
CMTDI2	0.00	0.00	0.00	0.00	0.00	0.15	0.00	0.00	0.00	0.00	0.00	0.00	0.00	0.00	0.22	0.16	0.00	0.00	0.00	0.00	0.00
CSF2	0.00	0.00	0.00	0.00	0.00	0.00	0.00	0.00	0.14	0.00	0.00	0.00	0.00	0.00	0.11	0.11	0.00	0.00	0.00	0.00	0.00
PRNP	0.00	0.00	0.00	0.00	0.00	0.00	0.00	0.00	0.10	0.00	0.00	0.00	0.00	0.00	0.12	0.11	0.00	0.00	0.00	0.00	0.00
C6ORF10	0.00	0.00	0.00	0.13	0.00	0.19	0.00	0.00	0.00	0.00	0.00	0.00	0.00	0.00	0.00	0.00	0.19	0.00	0.00	0.00	0.00
MYP17	0.00	0.00	0.00	0.11	0.00	0.20	0.00	0.00	0.00	0.00	0.00	0.00	0.00	0.00	0.00	0.00	0.14	0.00	0.00	0.00	0.00
ANIB1	0.00	0.00	0.00	0.11	0.00	0.18	0.00	0.00	0.00	0.00	0.00	0.00	0.00	0.00	0.00	0.00	0.11	0.00	0.00	0.00	0.00
FRTS	0.00	0.00	0.00	0.12	0.00	0.24	0.00	0.00	0.00	0.00	0.00	0.00	0.00	0.00	0.00	0.00	0.11	0.00	0.00	0.00	0.00
ANIB2	0.00	0.00	0.00	0.11	0.00	0.18	0.00	0.00	0.00	0.00	0.00	0.00	0.00	0.00	0.00	0.00	0.13	0.00	0.00	0.00	0.00
PSORS3	0.00	0.00	0.00	0.12	0.00	0.19	0.00	0.00	0.00	0.00	0.00	0.00	0.00	0.00	0.00	0.00	0.13	0.00	0.00	0.00	0.00
SLSN3	0.00	0.00	0.00	0.11	0.00	0.24	0.00	0.00	0.00	0.00	0.00	0.00	0.00	0.00	0.00	0.00	0.11	0.00	0.00	0.00	0.00
GRD2	0.00	0.00	0.00	0.13	0.00	0.19	0.00	0.00	0.00	0.00	0.00	0.00	0.00	0.00	0.00	0.00	0.15	0.00	0.00	0.00	0.00
ZNF816	0.00	0.00	0.00	0.12	0.00	0.14	0.00	0.00	0.00	0.00	0.00	0.00	0.00	0.00	0.00	0.00	0.18	0.00	0.00	0.00	0.00

ZNF782	0.00	0.00	0.00	0.14	0.00	0.24	0.00	0.00	0.00	0.00	0.00	0.00	0.00	0.00	0.00	0.00	0.13	0.00	0.00	0.00	0.00
LOC100132626	0.00	0.00	0.00	0.17	0.00	0.27	0.00	0.00	0.00	0.00	0.00	0.00	0.00	0.00	0.00	0.00	0.12	0.00	0.00	0.00	0.00
CELIAC5	0.00	0.00	0.00	0.14	0.00	0.23	0.00	0.00	0.00	0.00	0.00	0.00	0.00	0.00	0.00	0.00	0.16	0.00	0.00	0.00	0.00
RPL23AP28	0.00	0.00	0.00	0.11	0.00	0.15	0.00	0.00	0.00	0.00	0.00	0.00	0.00	0.00	0.00	0.00	0.20	0.00	0.00	0.00	0.00
ANIB4	0.00	0.00	0.00	0.12	0.00	0.23	0.00	0.00	0.00	0.00	0.00	0.00	0.00	0.00	0.00	0.00	0.11	0.00	0.00	0.00	0.00
MYP15	0.00	0.00	0.00	0.13	0.00	0.22	0.00	0.00	0.00	0.00	0.00	0.00	0.00	0.00	0.00	0.00	0.13	0.00	0.00	0.00	0.00
ZNF385D	0.00	0.00	0.00	0.13	0.00	0.16	0.00	0.00	0.00	0.00	0.00	0.00	0.00	0.00	0.00	0.00	0.15	0.00	0.00	0.00	0.00
VUR	0.00	0.00	0.00	0.11	0.00	0.15	0.00	0.00	0.00	0.00	0.00	0.00	0.00	0.00	0.00	0.00	0.14	0.00	0.00	0.00	0.00
MYP6	0.00	0.00	0.00	0.11	0.00	0.18	0.00	0.00	0.00	0.00	0.00	0.00	0.00	0.00	0.00	0.00	0.10	0.00	0.00	0.00	0.00
CLEC16A	0.00	0.00	0.00	0.13	0.00	0.13	0.00	0.00	0.00	0.00	0.00	0.00	0.00	0.00	0.00	0.00	0.27	0.00	0.00	0.00	0.00
CCNP	0.00	0.00	0.00	0.11	0.00	0.23	0.00	0.00	0.00	0.00	0.00	0.00	0.00	0.00	0.00	0.00	0.11	0.00	0.00	0.00	0.00
CELIAC2	0.00	0.00	0.00	0.13	0.00	0.15	0.00	0.00	0.00	0.00	0.00	0.00	0.00	0.00	0.00	0.00	0.17	0.00	0.00	0.00	0.00
PSORS4	0.00	0.00	0.00	0.14	0.00	0.20	0.00	0.00	0.00	0.00	0.00	0.00	0.00	0.00	0.00	0.00	0.16	0.00	0.00	0.00	0.00
PSNP3	0.00	0.00	0.00	0.10	0.00	0.14	0.00	0.00	0.00	0.00	0.00	0.00	0.00	0.00	0.00	0.00	0.14	0.00	0.00	0.00	0.00
CHDS5	0.00	0.00	0.00	0.00	0.00	0.18	0.00	0.19	0.00	0.00	0.00	0.00	0.00	0.00	0.00	0.00	0.14	0.00	0.00	0.00	0.00
SLEN3	0.00	0.00	0.00	0.00	0.00	0.10	0.00	0.00	0.15	0.00	0.00	0.00	0.00	0.00	0.00	0.00	0.12	0.00	0.00	0.00	0.00
SLEN2	0.00	0.00	0.00	0.00	0.00	0.10	0.00	0.00	0.15	0.00	0.00	0.00	0.00	0.00	0.00	0.00	0.12	0.00	0.00	0.00	0.00
SLEN1	0.00	0.00	0.00	0.00	0.00	0.10	0.00	0.00	0.15	0.00	0.00	0.00	0.00	0.00	0.00	0.00	0.12	0.00	0.00	0.00	0.00
SLEH1	0.00	0.00	0.00	0.00	0.00	0.10	0.00	0.00	0.11	0.00	0.00	0.00	0.00	0.00	0.00	0.00	0.10	0.00	0.00	0.00	0.00
CHDS2	0.00	0.00	0.00	0.00	0.00	0.20	0.00	0.00	0.00	0.00	0.00	0.10	0.00	0.00	0.00	0.00	0.13	0.00	0.00	0.00	0.00
FAM113B	0.00	0.00	0.00	0.00	0.00	0.13	0.00	0.00	0.00	0.00	0.00	0.11	0.00	0.00	0.00	0.00	0.14	0.00	0.00	0.00	0.00
C15ORF53	0.00	0.00	0.00	0.00	0.00	0.11	0.00	0.00	0.00	0.00	0.00	0.00	0.11	0.00	0.00	0.00	0.13	0.00	0.00	0.00	0.00
CCDC60	0.00	0.00	0.00	0.00	0.00	0.15	0.00	0.00	0.00	0.00	0.00	0.00	0.10	0.00	0.00	0.00	0.10	0.00	0.00	0.00	0.00
AD7	0.00	0.00	0.00	0.00	0.00	0.00	0.00	0.00	0.00	0.00	0.00	0.13	0.00	0.13	0.00	0.00	0.11	0.00	0.00	0.00	0.00
CYBA	0.00	0.00	0.00	0.10	0.00	0.00	0.00	0.00	0.00	0.00	0.00	0.00	0.00	0.00	0.10	0.00	0.15	0.00	0.00	0.00	0.00
C9ORF135	0.00	0.00	0.00	0.12	0.00	0.00	0.00	0.00	0.00	0.00	0.00	0.00	0.00	0.00	0.13	0.00	0.12	0.00	0.00	0.00	0.00
BTBD9	0.00	0.00	0.00	0.00	0.00	0.12	0.00	0.00	0.00	0.00	0.00	0.00	0.00	0.00	0.13	0.00	0.15	0.00	0.00	0.00	0.00
AIS4	0.00	0.00	0.00	0.00	0.00	0.10	0.00	0.00	0.00	0.00	0.00	0.00	0.00	0.00	0.14	0.00	0.15	0.00	0.00	0.00	0.00
AIS	0.00	0.00	0.00	0.00	0.00	0.13	0.00	0.00	0.00	0.00	0.00	0.00	0.00	0.00	0.10	0.00	0.14	0.00	0.00	0.00	0.00
RRIS	0.00	0.00	0.00	0.00	0.00	0.13	0.00	0.00	0.00	0.00	0.00	0.00	0.00	0.00	0.10	0.00	0.11	0.00	0.00	0.00	0.00
RMD1	0.00	0.00	0.00	0.00	0.00	0.15	0.00	0.00	0.00	0.00	0.00	0.00	0.00	0.00	0.13	0.00	0.00	0.12	0.00	0.00	0.00
CRYGD	0.00	0.00	0.00	0.00	0.00	0.14	0.00	0.00	0.00	0.00	0.00	0.00	0.00	0.00	0.10	0.00	0.00	0.14	0.00	0.00	0.00
LIPN	0.00	0.00	0.00	0.17	0.00	0.27	0.00	0.00	0.00	0.00	0.00	0.00	0.00	0.00	0.00	0.00	0.00	0.00	0.26	0.00	0.00
GUSBP4	0.00	0.00	0.00	0.14	0.00	0.30	0.00	0.00	0.00	0.00	0.00	0.00	0.00	0.00	0.00	0.00	0.00	0.00	0.14	0.00	0.00
GUSBP2	0.00	0.00	0.00	0.14	0.00	0.30	0.00	0.00	0.00	0.00	0.00	0.00	0.00	0.00	0.00	0.00	0.00	0.00	0.14	0.00	0.00
APOC1P1	0.00	0.00	0.00	0.21	0.00	0.35	0.00	0.00	0.00	0.00	0.00	0.00	0.00	0.00	0.00	0.00	0.00	0.00	0.13	0.00	0.00
C12ORF4	0.00	0.00	0.00	0.10	0.00	0.18	0.00	0.00	0.00	0.00	0.00	0.00	0.00	0.00	0.00	0.00	0.00	0.00	0.14	0.00	0.00
CYP4F22	0.00	0.00	0.00	0.10	0.00	0.18	0.00	0.00	0.00	0.00	0.00	0.00	0.00	0.00	0.00	0.00	0.00	0.00	0.25	0.00	0.00
HTC1	0.00	0.00	0.00	0.15	0.00	0.20	0.00	0.00	0.00	0.00	0.00	0.00	0.00	0.00	0.00	0.00	0.00	0.00	0.11	0.00	0.00
HCHOLA4	0.00	0.00	0.00	0.16	0.00	0.26	0.00	0.00	0.00	0.00	0.00	0.00	0.00	0.00	0.00	0.00	0.00	0.00	0.10	0.00	0.00

PHEX	0.00	0.00	0.00	0.11	0.00	0.00	0.00	0.00	0.11	0.00	0.00	0.00	0.00	0.00	0.00	0.00	0.00	0.00	0.23	0.00	0.00
ABCC2	0.00	0.00	0.00	0.00	0.00	0.00	0.00	0.11	0.14	0.00	0.00	0.00	0.00	0.00	0.00	0.00	0.00	0.00	0.11	0.00	0.00
CYP2C9	0.00	0.00	0.00	0.00	0.00	0.00	0.00	0.13	0.21	0.00	0.00	0.00	0.00	0.00	0.00	0.00	0.00	0.00	0.14	0.00	0.00
RFC1	0.00	0.00	0.00	0.00	0.00	0.12	0.00	0.00	0.00	0.00	0.00	0.00	0.00	0.00	0.11	0.00	0.00	0.00	0.20	0.00	0.00
PTGS1	0.00	0.00	0.00	0.00	0.00	0.00	0.00	0.00	0.14	0.00	0.00	0.00	0.00	0.00	0.11	0.00	0.00	0.00	0.11	0.00	0.00
ABCA1	0.00	0.00	0.00	0.00	0.00	0.00	0.00	0.10	0.00	0.00	0.00	0.00	0.00	0.00	0.00	0.10	0.00	0.00	0.11	0.00	0.00
CETP	0.00	0.00	0.00	0.00	0.00	0.00	0.00	0.00	0.12	0.00	0.00	0.00	0.00	0.00	0.00	0.00	0.14	0.00	0.16	0.00	0.00
CYP51P2	0.00	0.00	0.00	0.17	0.00	0.35	0.00	0.00	0.00	0.00	0.00	0.00	0.00	0.00	0.00	0.00	0.00	0.00	0.00	0.00	0.10
DNAAF2	0.00	0.00	0.00	0.17	0.00	0.33	0.00	0.00	0.00	0.00	0.00	0.00	0.00	0.00	0.00	0.00	0.00	0.00	0.00	0.00	0.11
DNAAF3	0.00	0.00	0.00	0.13	0.00	0.30	0.00	0.00	0.00	0.00	0.00	0.00	0.00	0.00	0.00	0.00	0.00	0.00	0.00	0.00	0.12
DFNM2	0.00	0.00	0.00	0.15	0.00	0.35	0.00	0.00	0.00	0.00	0.00	0.00	0.00	0.00	0.00	0.00	0.00	0.00	0.00	0.00	0.28
OPA6	0.00	0.00	0.00	0.00	0.00	0.18	0.00	0.00	0.00	0.00	0.00	0.00	0.00	0.00	0.12	0.00	0.00	0.00	0.00	0.00	0.11
SDHAF2	0.00	0.00	0.00	0.00	0.00	0.20	0.00	0.00	0.00	0.00	0.00	0.00	0.00	0.00	0.11	0.00	0.00	0.00	0.00	0.00	0.18
OPA4	0.00	0.00	0.00	0.00	0.00	0.12	0.00	0.00	0.00	0.00	0.00	0.00	0.00	0.00	0.10	0.00	0.00	0.00	0.00	0.00	0.17
SLC25A38	0.00	0.00	0.00	0.00	0.00	0.23	0.00	0.00	0.00	0.00	0.00	0.00	0.00	0.00	0.00	0.00	0.00	0.00	0.20	0.00	0.30
TRNR	0.00	0.00	0.00	0.00	0.00	0.12	0.00	0.00	0.00	0.00	0.00	0.00	0.00	0.00	0.00	0.00	0.00	0.00	0.18	0.00	0.28
PAFC	0.00	0.00	0.00	0.10	0.00	0.18	0.00	0.00	0.00	0.00	0.00	0.00	0.00	0.00	0.00	0.00	0.00	0.00	0.00	0.00	0.00
AASTH27	0.00	0.00	0.00	0.13	0.00	0.18	0.00	0.00	0.00	0.00	0.00	0.00	0.00	0.00	0.00	0.00	0.00	0.00	0.00	0.00	0.00
HSR	0.00	0.00	0.00	0.12	0.00	0.15	0.00	0.00	0.00	0.00	0.00	0.00	0.00	0.00	0.00	0.00	0.00	0.00	0.00	0.00	0.00
CANDN1	0.00	0.00	0.00	0.11	0.00	0.11	0.00	0.00	0.00	0.00	0.00	0.00	0.00	0.00	0.00	0.00	0.00	0.00	0.00	0.00	0.00
LCE5A	0.00	0.00	0.00	0.12	0.00	0.16	0.00	0.00	0.00	0.00	0.00	0.00	0.00	0.00	0.00	0.00	0.00	0.00	0.00	0.00	0.00
OR51A1P	0.00	0.00	0.00	0.11	0.00	0.16	0.00	0.00	0.00	0.00	0.00	0.00	0.00	0.00	0.00	0.00	0.00	0.00	0.00	0.00	0.00
KATNAL2	0.00	0.00	0.00	0.11	0.00	0.15	0.00	0.00	0.00	0.00	0.00	0.00	0.00	0.00	0.00	0.00	0.00	0.00	0.00	0.00	0.00
RSL24D1P7	0.00	0.00	0.00	0.12	0.00	0.16	0.00	0.00	0.00	0.00	0.00	0.00	0.00	0.00	0.00	0.00	0.00	0.00	0.00	0.00	0.00
NMTC1	0.00	0.00	0.00	0.11	0.00	0.16	0.00	0.00	0.00	0.00	0.00	0.00	0.00	0.00	0.00	0.00	0.00	0.00	0.00	0.00	0.00
ZNF275	0.00	0.00	0.00	0.15	0.00	0.18	0.00	0.00	0.00	0.00	0.00	0.00	0.00	0.00	0.00	0.00	0.00	0.00	0.00	0.00	0.00
CMDR	0.00	0.00	0.00	0.13	0.00	0.12	0.00	0.00	0.00	0.00	0.00	0.00	0.00	0.00	0.00	0.00	0.00	0.00	0.00	0.00	0.00
EKV3	0.00	0.00	0.00	0.10	0.00	0.17	0.00	0.00	0.00	0.00	0.00	0.00	0.00	0.00	0.00	0.00	0.00	0.00	0.00	0.00	0.00
CANDF1	0.00	0.00	0.00	0.10	0.00	0.14	0.00	0.00	0.00	0.00	0.00	0.00	0.00	0.00	0.00	0.00	0.00	0.00	0.00	0.00	0.00
MGR2	0.00	0.00	0.00	0.12	0.00	0.18	0.00	0.00	0.00	0.00	0.00	0.00	0.00	0.00	0.00	0.00	0.00	0.00	0.00	0.00	0.00
TRNAQ1	0.00	0.00	0.00	0.15	0.00	0.42	0.00	0.00	0.00	0.00	0.00	0.00	0.00	0.00	0.00	0.00	0.00	0.00	0.00	0.00	0.00
RPS19P1	0.00	0.00	0.00	0.18	0.00	0.38	0.00	0.00	0.00	0.00	0.00	0.00	0.00	0.00	0.00	0.00	0.00	0.00	0.00	0.00	0.00
IGKV1D-17	0.00	0.00	0.00	0.13	0.00	0.21	0.00	0.00	0.00	0.00	0.00	0.00	0.00	0.00	0.00	0.00	0.00	0.00	0.00	0.00	0.00
SYM2	0.00	0.00	0.00	0.11	0.00	0.21	0.00	0.00	0.00	0.00	0.00	0.00	0.00	0.00	0.00	0.00	0.00	0.00	0.00	0.00	0.00
OTSC4	0.00	0.00	0.00	0.15	0.00	0.30	0.00	0.00	0.00	0.00	0.00	0.00	0.00	0.00	0.00	0.00	0.00	0.00	0.00	0.00	0.00
GINGF3	0.00	0.00	0.00	0.11	0.00	0.24	0.00	0.00	0.00	0.00	0.00	0.00	0.00	0.00	0.00	0.00	0.00	0.00	0.00	0.00	0.00
AMCX5	0.00	0.00	0.00	0.11	0.00	0.27	0.00	0.00	0.00	0.00	0.00	0.00	0.00	0.00	0.00	0.00	0.00	0.00	0.00	0.00	0.00
OR6L1P	0.00	0.00	0.00	0.11	0.00	0.27	0.00	0.00	0.00	0.00	0.00	0.00	0.00	0.00	0.00	0.00	0.00	0.00	0.00	0.00	0.00
KRTAP5-1	0.00	0.00	0.00	0.11	0.00	0.21	0.00	0.00	0.00	0.00	0.00	0.00	0.00	0.00	0.00	0.00	0.00	0.00	0.00	0.00	0.00
SPRNP1	0.00	0.00	0.00	0.13	0.00	0.29	0.00	0.00	0.00	0.00	0.00	0.00	0.00	0.00	0.00	0.00	0.00	0.00	0.00	0.00	0.00

IGLV@	0.00	0.00	0.00	0.15	0.00	0.21	0.00	0.00	0.00	0.00	0.00	0.00	0.00	0.00	0.00	0.00	0.00	0.00	0.00	0.00	0.00
TEC	0.00	0.00	0.00	0.16	0.00	0.38	0.00	0.00	0.00	0.00	0.00	0.00	0.00	0.00	0.00	0.00	0.00	0.00	0.00	0.00	0.00
PTCPRN	0.00	0.00	0.00	0.12	0.00	0.21	0.00	0.00	0.00	0.00	0.00	0.00	0.00	0.00	0.00	0.00	0.00	0.00	0.00	0.00	0.00
FRA11A	0.00	0.00	0.00	0.15	0.00	0.34	0.00	0.00	0.00	0.00	0.00	0.00	0.00	0.00	0.00	0.00	0.00	0.00	0.00	0.00	0.00
MMDK	0.00	0.00	0.00	0.14	0.00	0.24	0.00	0.00	0.00	0.00	0.00	0.00	0.00	0.00	0.00	0.00	0.00	0.00	0.00	0.00	0.00
DAZ3	0.00	0.00	0.00	0.13	0.00	0.33	0.00	0.00	0.00	0.00	0.00	0.00	0.00	0.00	0.00	0.00	0.00	0.00	0.00	0.00	0.00
FEB-06	0.00	0.00	0.00	0.15	0.00	0.32	0.00	0.00	0.00	0.00	0.00	0.00	0.00	0.00	0.00	0.00	0.00	0.00	0.00	0.00	0.00
CNTNAP3B	0.00	0.00	0.00	0.10	0.00	0.26	0.00	0.00	0.00	0.00	0.00	0.00	0.00	0.00	0.00	0.00	0.00	0.00	0.00	0.00	0.00
CMD1C	0.00	0.00	0.00	0.10	0.00	0.20	0.00	0.00	0.00	0.00	0.00	0.00	0.00	0.00	0.00	0.00	0.00	0.00	0.00	0.00	0.00
PMS2P4	0.00	0.00	0.00	0.13	0.00	0.31	0.00	0.00	0.00	0.00	0.00	0.00	0.00	0.00	0.00	0.00	0.00	0.00	0.00	0.00	0.00
EBM	0.00	0.00	0.00	0.10	0.00	0.20	0.00	0.00	0.00	0.00	0.00	0.00	0.00	0.00	0.00	0.00	0.00	0.00	0.00	0.00	0.00
LVNC2	0.00	0.00	0.00	0.11	0.00	0.28	0.00	0.00	0.00	0.00	0.00	0.00	0.00	0.00	0.00	0.00	0.00	0.00	0.00	0.00	0.00
DAZ4	0.00	0.00	0.00	0.13	0.00	0.32	0.00	0.00	0.00	0.00	0.00	0.00	0.00	0.00	0.00	0.00	0.00	0.00	0.00	0.00	0.00
GLC1C	0.00	0.00	0.00	0.14	0.00	0.32	0.00	0.00	0.00	0.00	0.00	0.00	0.00	0.00	0.00	0.00	0.00	0.00	0.00	0.00	0.00
CMD1Q	0.00	0.00	0.00	0.13	0.00	0.24	0.00	0.00	0.00	0.00	0.00	0.00	0.00	0.00	0.00	0.00	0.00	0.00	0.00	0.00	0.00
RPS19P2	0.00	0.00	0.00	0.18	0.00	0.38	0.00	0.00	0.00	0.00	0.00	0.00	0.00	0.00	0.00	0.00	0.00	0.00	0.00	0.00	0.00
PMS2P5	0.00	0.00	0.00	0.12	0.00	0.31	0.00	0.00	0.00	0.00	0.00	0.00	0.00	0.00	0.00	0.00	0.00	0.00	0.00	0.00	0.00
USP26	0.00	0.00	0.00	0.18	0.00	0.24	0.00	0.00	0.00	0.00	0.00	0.00	0.00	0.00	0.00	0.00	0.00	0.00	0.00	0.00	0.00
CDY1B	0.00	0.00	0.00	0.15	0.00	0.38	0.00	0.00	0.00	0.00	0.00	0.00	0.00	0.00	0.00	0.00	0.00	0.00	0.00	0.00	0.00
C8ORF37	0.00	0.00	0.00	0.11	0.00	0.22	0.00	0.00	0.00	0.00	0.00	0.00	0.00	0.00	0.00	0.00	0.00	0.00	0.00	0.00	0.00
TW	0.00	0.00	0.00	0.13	0.00	0.30	0.00	0.00	0.00	0.00	0.00	0.00	0.00	0.00	0.00	0.00	0.00	0.00	0.00	0.00	0.00
XS	0.00	0.00	0.00	0.12	0.00	0.23	0.00	0.00	0.00	0.00	0.00	0.00	0.00	0.00	0.00	0.00	0.00	0.00	0.00	0.00	0.00
DYT17	0.00	0.00	0.00	0.11	0.00	0.23	0.00	0.00	0.00	0.00	0.00	0.00	0.00	0.00	0.00	0.00	0.00	0.00	0.00	0.00	0.00
DUX4L7	0.00	0.00	0.00	0.11	0.00	0.29	0.00	0.00	0.00	0.00	0.00	0.00	0.00	0.00	0.00	0.00	0.00	0.00	0.00	0.00	0.00
TPRN	0.00	0.00	0.00	0.13	0.00	0.31	0.00	0.00	0.00	0.00	0.00	0.00	0.00	0.00	0.00	0.00	0.00	0.00	0.00	0.00	0.00
MSSE	0.00	0.00	0.00	0.19	0.00	0.26	0.00	0.00	0.00	0.00	0.00	0.00	0.00	0.00	0.00	0.00	0.00	0.00	0.00	0.00	0.00
IGKV2-40	0.00	0.00	0.00	0.18	0.00	0.34	0.00	0.00	0.00	0.00	0.00	0.00	0.00	0.00	0.00	0.00	0.00	0.00	0.00	0.00	0.00
DUXB	0.00	0.00	0.00	0.13	0.00	0.31	0.00	0.00	0.00	0.00	0.00	0.00	0.00	0.00	0.00	0.00	0.00	0.00	0.00	0.00	0.00
GJA1P1	0.00	0.00	0.00	0.12	0.00	0.23	0.00	0.00	0.00	0.00	0.00	0.00	0.00	0.00	0.00	0.00	0.00	0.00	0.00	0.00	0.00
ENUR2	0.00	0.00	0.00	0.12	0.00	0.24	0.00	0.00	0.00	0.00	0.00	0.00	0.00	0.00	0.00	0.00	0.00	0.00	0.00	0.00	0.00
PLGLB1	0.00	0.00	0.00	0.24	0.00	0.43	0.00	0.00	0.00	0.00	0.00	0.00	0.00	0.00	0.00	0.00	0.00	0.00	0.00	0.00	0.00
NEFLL1	0.00	0.00	0.00	0.19	0.00	0.44	0.00	0.00	0.00	0.00	0.00	0.00	0.00	0.00	0.00	0.00	0.00	0.00	0.00	0.00	0.00
DUH	0.00	0.00	0.00	0.11	0.00	0.26	0.00	0.00	0.00	0.00	0.00	0.00	0.00	0.00	0.00	0.00	0.00	0.00	0.00	0.00	0.00
DAZ2	0.00	0.00	0.00	0.13	0.00	0.31	0.00	0.00	0.00	0.00	0.00	0.00	0.00	0.00	0.00	0.00	0.00	0.00	0.00	0.00	0.00
ZNF213	0.00	0.00	0.00	0.16	0.00	0.41	0.00	0.00	0.00	0.00	0.00	0.00	0.00	0.00	0.00	0.00	0.00	0.00	0.00	0.00	0.00
PMS2LP2	0.00	0.00	0.00	0.18	0.00	0.39	0.00	0.00	0.00	0.00	0.00	0.00	0.00	0.00	0.00	0.00	0.00	0.00	0.00	0.00	0.00
IGKV1-37	0.00	0.00	0.00	0.14	0.00	0.27	0.00	0.00	0.00	0.00	0.00	0.00	0.00	0.00	0.00	0.00	0.00	0.00	0.00	0.00	0.00
DYX4	0.00	0.00	0.00	0.12	0.00	0.24	0.00	0.00	0.00	0.00	0.00	0.00	0.00	0.00	0.00	0.00	0.00	0.00	0.00	0.00	0.00
PRY2	0.00	0.00	0.00	0.17	0.00	0.30	0.00	0.00	0.00	0.00	0.00	0.00	0.00	0.00	0.00	0.00	0.00	0.00	0.00	0.00	0.00

DTX2P1-UPK3BP1-PMS2P11	0.00	0.00	0.00	0.14	0.00	0.33	0.00	0.00	0.00	0.00	0.00	0.00	0.00	0.00	0.00	0.00	0.00	0.00	0.00	0.00	0.00
ATF4P3	0.00	0.00	0.00	0.10	0.00	0.29	0.00	0.00	0.00	0.00	0.00	0.00	0.00	0.00	0.00	0.00	0.00	0.00	0.00	0.00	0.00
CNC	0.00	0.00	0.00	0.11	0.00	0.27	0.00	0.00	0.00	0.00	0.00	0.00	0.00	0.00	0.00	0.00	0.00	0.00	0.00	0.00	0.00
ATF4P4	0.00	0.00	0.00	0.10	0.00	0.29	0.00	0.00	0.00	0.00	0.00	0.00	0.00	0.00	0.00	0.00	0.00	0.00	0.00	0.00	0.00
IGKV2D-40	0.00	0.00	0.00	0.14	0.00	0.27	0.00	0.00	0.00	0.00	0.00	0.00	0.00	0.00	0.00	0.00	0.00	0.00	0.00	0.00	0.00
FRA11B	0.00	0.00	0.00	0.13	0.00	0.29	0.00	0.00	0.00	0.00	0.00	0.00	0.00	0.00	0.00	0.00	0.00	0.00	0.00	0.00	0.00
ARVD6	0.00	0.00	0.00	0.12	0.00	0.22	0.00	0.00	0.00	0.00	0.00	0.00	0.00	0.00	0.00	0.00	0.00	0.00	0.00	0.00	0.00
CHDM	0.00	0.00	0.00	0.10	0.00	0.22	0.00	0.00	0.00	0.00	0.00	0.00	0.00	0.00	0.00	0.00	0.00	0.00	0.00	0.00	0.00
WS2B	0.00	0.00	0.00	0.10	0.00	0.24	0.00	0.00	0.00	0.00	0.00	0.00	0.00	0.00	0.00	0.00	0.00	0.00	0.00	0.00	0.00
TEX28	0.00	0.00	0.00	0.16	0.00	0.33	0.00	0.00	0.00	0.00	0.00	0.00	0.00	0.00	0.00	0.00	0.00	0.00	0.00	0.00	0.00
OR1F1	0.00	0.00	0.00	0.15	0.00	0.38	0.00	0.00	0.00	0.00	0.00	0.00	0.00	0.00	0.00	0.00	0.00	0.00	0.00	0.00	0.00
CDY1	0.00	0.00	0.00	0.12	0.00	0.27	0.00	0.00	0.00	0.00	0.00	0.00	0.00	0.00	0.00	0.00	0.00	0.00	0.00	0.00	0.00
COX7B2	0.00	0.00	0.00	0.12	0.00	0.25	0.00	0.00	0.00	0.00	0.00	0.00	0.00	0.00	0.00	0.00	0.00	0.00	0.00	0.00	0.00
DBA2	0.00	0.00	0.00	0.14	0.00	0.28	0.00	0.00	0.00	0.00	0.00	0.00	0.00	0.00	0.00	0.00	0.00	0.00	0.00	0.00	0.00
HERC2P9	0.00	0.00	0.00	0.16	0.00	0.28	0.00	0.00	0.00	0.00	0.00	0.00	0.00	0.00	0.00	0.00	0.00	0.00	0.00	0.00	0.00
TMEM87A	0.00	0.00	0.00	0.16	0.00	0.28	0.00	0.00	0.00	0.00	0.00	0.00	0.00	0.00	0.00	0.00	0.00	0.00	0.00	0.00	0.00
LI5	0.00	0.00	0.00	0.10	0.00	0.20	0.00	0.00	0.00	0.00	0.00	0.00	0.00	0.00	0.00	0.00	0.00	0.00	0.00	0.00	0.00
OR1F2P	0.00	0.00	0.00	0.14	0.00	0.36	0.00	0.00	0.00	0.00	0.00	0.00	0.00	0.00	0.00	0.00	0.00	0.00	0.00	0.00	0.00
SERAC1	0.00	0.00	0.00	0.10	0.00	0.19	0.00	0.00	0.00	0.00	0.00	0.00	0.00	0.00	0.00	0.00	0.00	0.00	0.00	0.00	0.00
MDRV	0.00	0.00	0.00	0.12	0.00	0.25	0.00	0.00	0.00	0.00	0.00	0.00	0.00	0.00	0.00	0.00	0.00	0.00	0.00	0.00	0.00
TRBV7-1	0.00	0.00	0.00	0.19	0.00	0.35	0.00	0.00	0.00	0.00	0.00	0.00	0.00	0.00	0.00	0.00	0.00	0.00	0.00	0.00	0.00
MMVP2	0.00	0.00	0.00	0.10	0.00	0.19	0.00	0.00	0.00	0.00	0.00	0.00	0.00	0.00	0.00	0.00	0.00	0.00	0.00	0.00	0.00
OTSC8	0.00	0.00	0.00	0.12	0.00	0.25	0.00	0.00	0.00	0.00	0.00	0.00	0.00	0.00	0.00	0.00	0.00	0.00	0.00	0.00	0.00
BPY2	0.00	0.00	0.00	0.18	0.00	0.35	0.00	0.00	0.00	0.00	0.00	0.00	0.00	0.00	0.00	0.00	0.00	0.00	0.00	0.00	0.00
WHAMMP2	0.00	0.00	0.00	0.16	0.00	0.28	0.00	0.00	0.00	0.00	0.00	0.00	0.00	0.00	0.00	0.00	0.00	0.00	0.00	0.00	0.00
CAPZA1P	0.00	0.00	0.00	0.10	0.00	0.26	0.00	0.00	0.00	0.00	0.00	0.00	0.00	0.00	0.00	0.00	0.00	0.00	0.00	0.00	0.00
C11ORF80	0.00	0.00	0.00	0.15	0.00	0.34	0.00	0.00	0.00	0.00	0.00	0.00	0.00	0.00	0.00	0.00	0.00	0.00	0.00	0.00	0.00
LOC653541	0.00	0.00	0.00	0.11	0.00	0.29	0.00	0.00	0.00	0.00	0.00	0.00	0.00	0.00	0.00	0.00	0.00	0.00	0.00	0.00	0.00
PMS2P3	0.00	0.00	0.00	0.12	0.00	0.31	0.00	0.00	0.00	0.00	0.00	0.00	0.00	0.00	0.00	0.00	0.00	0.00	0.00	0.00	0.00
RP24	0.00	0.00	0.00	0.14	0.00	0.18	0.00	0.00	0.00	0.00	0.00	0.00	0.00	0.00	0.00	0.00	0.00	0.00	0.00	0.00	0.00
DUX4L3	0.00	0.00	0.00	0.11	0.00	0.29	0.00	0.00	0.00	0.00	0.00	0.00	0.00	0.00	0.00	0.00	0.00	0.00	0.00	0.00	0.00
RPL21P4	0.00	0.00	0.00	0.12	0.00	0.26	0.00	0.00	0.00	0.00	0.00	0.00	0.00	0.00	0.00	0.00	0.00	0.00	0.00	0.00	0.00
CPXCR1	0.00	0.00	0.00	0.10	0.00	0.26	0.00	0.00	0.00	0.00	0.00	0.00	0.00	0.00	0.00	0.00	0.00	0.00	0.00	0.00	0.00
DAZ1	0.00	0.00	0.00	0.12	0.00	0.25	0.00	0.00	0.00	0.00	0.00	0.00	0.00	0.00	0.00	0.00	0.00	0.00	0.00	0.00	0.00
MYP18	0.00	0.00	0.00	0.12	0.00	0.26	0.00	0.00	0.00	0.00	0.00	0.00	0.00	0.00	0.00	0.00	0.00	0.00	0.00	0.00	0.00
PTPN11	0.00	0.00	0.00	0.11	0.00	0.00	0.00	0.12	0.00	0.00	0.00	0.00	0.00	0.00	0.00	0.00	0.00	0.00	0.00	0.00	0.00
LFS3	0.00	0.00	0.00	0.00	0.00	0.23	0.00	0.13	0.00	0.00	0.00	0.00	0.00	0.00	0.00	0.00	0.00	0.00	0.00	0.00	0.00
PCNXL2	0.00	0.00	0.00	0.00	0.00	0.25	0.00	0.14	0.00	0.00	0.00	0.00	0.00	0.00	0.00	0.00	0.00	0.00	0.00	0.00	0.00
BCPR	0.00	0.00	0.00	0.00	0.00	0.22	0.00	0.20	0.00	0.00	0.00	0.00	0.00	0.00	0.00	0.00	0.00	0.00	0.00	0.00	0.00

TYS	0.00	0.00	0.00	0.00	0.00	0.25	0.00	0.10	0.00	0.00	0.00	0.00	0.00	0.00	0.00	0.00	0.00	0.00	0.00	0.00	0.00
ELN	0.00	0.00	0.00	0.11	0.00	0.00	0.00	0.00	0.11	0.00	0.00	0.00	0.00	0.00	0.00	0.00	0.00	0.00	0.00	0.00	0.00
HSPA1A	0.00	0.00	0.00	0.10	0.00	0.00	0.00	0.00	0.13	0.00	0.00	0.00	0.00	0.00	0.00	0.00	0.00	0.00	0.00	0.00	0.00
LOC392787	0.00	0.00	0.00	0.00	0.00	0.19	0.00	0.00	0.11	0.00	0.00	0.00	0.00	0.00	0.00	0.00	0.00	0.00	0.00	0.00	0.00
MYMY1	0.00	0.00	0.00	0.00	0.00	0.20	0.00	0.00	0.13	0.00	0.00	0.00	0.00	0.00	0.00	0.00	0.00	0.00	0.00	0.00	0.00
GHS	0.00	0.00	0.00	0.00	0.00	0.14	0.00	0.00	0.16	0.00	0.00	0.00	0.00	0.00	0.00	0.00	0.00	0.00	0.00	0.00	0.00
OTSC5	0.00	0.00	0.00	0.00	0.00	0.19	0.00	0.00	0.14	0.00	0.00	0.00	0.00	0.00	0.00	0.00	0.00	0.00	0.00	0.00	0.00
PDR	0.00	0.00	0.00	0.00	0.00	0.14	0.00	0.00	0.12	0.00	0.00	0.00	0.00	0.00	0.00	0.00	0.00	0.00	0.00	0.00	0.00
OTSC7	0.00	0.00	0.00	0.00	0.00	0.20	0.00	0.00	0.13	0.00	0.00	0.00	0.00	0.00	0.00	0.00	0.00	0.00	0.00	0.00	0.00
LGMD1G	0.00	0.00	0.00	0.00	0.00	0.11	0.00	0.00	0.14	0.00	0.00	0.00	0.00	0.00	0.00	0.00	0.00	0.00	0.00	0.00	0.00
LOC619409	0.00	0.00	0.00	0.00	0.00	0.12	0.00	0.00	0.12	0.00	0.00	0.00	0.00	0.00	0.00	0.00	0.00	0.00	0.00	0.00	0.00
WDM	0.00	0.00	0.00	0.00	0.00	0.11	0.00	0.00	0.11	0.00	0.00	0.00	0.00	0.00	0.00	0.00	0.00	0.00	0.00	0.00	0.00
CDKL4	0.00	0.00	0.00	0.00	0.00	0.14	0.00	0.00	0.10	0.00	0.00	0.00	0.00	0.00	0.00	0.00	0.00	0.00	0.00	0.00	0.00
LGMD1D	0.00	0.00	0.00	0.00	0.00	0.13	0.00	0.00	0.11	0.00	0.00	0.00	0.00	0.00	0.00	0.00	0.00	0.00	0.00	0.00	0.00
LGMD1F	0.00	0.00	0.00	0.00	0.00	0.14	0.00	0.00	0.14	0.00	0.00	0.00	0.00	0.00	0.00	0.00	0.00	0.00	0.00	0.00	0.00
PCAP	0.00	0.00	0.00	0.00	0.00	0.19	0.00	0.00	0.12	0.00	0.00	0.00	0.00	0.00	0.00	0.00	0.00	0.00	0.00	0.00	0.00
APEX1	0.00	0.00	0.00	0.00	0.00	0.00	0.00	0.12	0.15	0.00	0.00	0.00	0.00	0.00	0.00	0.00	0.00	0.00	0.00	0.00	0.00
TERC	0.00	0.00	0.00	0.00	0.00	0.00	0.00	0.13	0.17	0.00	0.00	0.00	0.00	0.00	0.00	0.00	0.00	0.00	0.00	0.00	0.00
BRAF	0.00	0.00	0.00	0.00	0.00	0.00	0.00	0.11	0.16	0.00	0.00	0.00	0.00	0.00	0.00	0.00	0.00	0.00	0.00	0.00	0.00
SERPINA1	0.00	0.00	0.00	0.00	0.00	0.00	0.00	0.12	0.11	0.00	0.00	0.00	0.00	0.00	0.00	0.00	0.00	0.00	0.00	0.00	0.00
GSK3B	0.00	0.00	0.00	0.00	0.00	0.00	0.00	0.10	0.14	0.00	0.00	0.00	0.00	0.00	0.00	0.00	0.00	0.00	0.00	0.00	0.00
SLC6A3	0.00	0.00	0.00	0.00	0.00	0.00	0.00	0.10	0.11	0.00	0.00	0.00	0.00	0.00	0.00	0.00	0.00	0.00	0.00	0.00	0.00
NFE2L2	0.00	0.00	0.00	0.00	0.00	0.00	0.00	0.12	0.14	0.00	0.00	0.00	0.00	0.00	0.00	0.00	0.00	0.00	0.00	0.00	0.00
MNG3	0.00	0.00	0.00	0.00	0.00	0.11	0.00	0.00	0.00	0.21	0.00	0.00	0.00	0.00	0.00	0.00	0.00	0.00	0.00	0.00	0.00
DHS	0.00	0.00	0.00	0.00	0.00	0.19	0.00	0.00	0.00	0.00	0.11	0.00	0.00	0.00	0.00	0.00	0.00	0.00	0.00	0.00	0.00
CSE	0.00	0.00	0.00	0.00	0.00	0.22	0.00	0.00	0.00	0.00	0.10	0.00	0.00	0.00	0.00	0.00	0.00	0.00	0.00	0.00	0.00
DFNB14	0.00	0.00	0.00	0.00	0.00	0.18	0.00	0.00	0.00	0.00	0.10	0.00	0.00	0.00	0.00	0.00	0.00	0.00	0.00	0.00	0.00
NYS3	0.00	0.00	0.00	0.00	0.00	0.19	0.00	0.00	0.00	0.00	0.10	0.00	0.00	0.00	0.00	0.00	0.00	0.00	0.00	0.00	0.00
MA	0.00	0.00	0.00	0.00	0.00	0.21	0.00	0.00	0.00	0.00	0.11	0.00	0.00	0.00	0.00	0.00	0.00	0.00	0.00	0.00	0.00
DFNA16	0.00	0.00	0.00	0.00	0.00	0.16	0.00	0.00	0.00	0.00	0.10	0.00	0.00	0.00	0.00	0.00	0.00	0.00	0.00	0.00	0.00
C10ORF11	0.00	0.00	0.00	0.00	0.00	0.19	0.00	0.00	0.00	0.00	0.00	0.16	0.00	0.00	0.00	0.00	0.00	0.00	0.00	0.00	0.00
MRX82	0.00	0.00	0.00	0.00	0.00	0.23	0.00	0.00	0.00	0.00	0.00	0.18	0.00	0.00	0.00	0.00	0.00	0.00	0.00	0.00	0.00
MS4A4A	0.00	0.00	0.00	0.00	0.00	0.12	0.00	0.00	0.00	0.00	0.00	0.17	0.00	0.00	0.00	0.00	0.00	0.00	0.00	0.00	0.00
MNG2	0.00	0.00	0.00	0.00	0.00	0.16	0.00	0.00	0.00	0.00	0.00	0.12	0.00	0.00	0.00	0.00	0.00	0.00	0.00	0.00	0.00
MRXS11	0.00	0.00	0.00	0.00	0.00	0.22	0.00	0.00	0.00	0.00	0.00	0.15	0.00	0.00	0.00	0.00	0.00	0.00	0.00	0.00	0.00
MRX8	0.00	0.00	0.00	0.00	0.00	0.12	0.00	0.00	0.00	0.00	0.00	0.13	0.00	0.00	0.00	0.00	0.00	0.00	0.00	0.00	0.00
VWS2	0.00	0.00	0.00	0.00	0.00	0.14	0.00	0.00	0.00	0.00	0.00	0.10	0.00	0.00	0.00	0.00	0.00	0.00	0.00	0.00	0.00
FEB-01	0.00	0.00	0.00	0.00	0.00	0.25	0.00	0.00	0.00	0.00	0.00	0.11	0.00	0.00	0.00	0.00	0.00	0.00	0.00	0.00	0.00
AIC	0.00	0.00	0.00	0.00	0.00	0.16	0.00	0.00	0.00	0.00	0.00	0.11	0.00	0.00	0.00	0.00	0.00	0.00	0.00	0.00	0.00
MRX4	0.00	0.00	0.00	0.00	0.00	0.18	0.00	0.00	0.00	0.00	0.00	0.11	0.00	0.00	0.00	0.00	0.00	0.00	0.00	0.00	0.00

FRAXE	0.00	0.00	0.00	0.00	0.00	0.13	0.00	0.00	0.00	0.00	0.00	0.11	0.00	0.00	0.00	0.00	0.00	0.00	0.00	0.00	0.00
GUST	0.00	0.00	0.00	0.00	0.00	0.20	0.00	0.00	0.00	0.00	0.00	0.14	0.00	0.00	0.00	0.00	0.00	0.00	0.00	0.00	0.00
MRX64	0.00	0.00	0.00	0.00	0.00	0.16	0.00	0.00	0.00	0.00	0.00	0.14	0.00	0.00	0.00	0.00	0.00	0.00	0.00	0.00	0.00
CLA3	0.00	0.00	0.00	0.00	0.00	0.21	0.00	0.00	0.00	0.00	0.00	0.11	0.00	0.00	0.00	0.00	0.00	0.00	0.00	0.00	0.00
EPRPDC	0.00	0.00	0.00	0.00	0.00	0.20	0.00	0.00	0.00	0.00	0.00	0.13	0.00	0.00	0.00	0.00	0.00	0.00	0.00	0.00	0.00
ATOD3	0.00	0.00	0.00	0.00	0.00	0.14	0.00	0.00	0.00	0.00	0.00	0.11	0.00	0.00	0.00	0.00	0.00	0.00	0.00	0.00	0.00
MPDMRS	0.00	0.00	0.00	0.00	0.00	0.13	0.00	0.00	0.00	0.00	0.00	0.11	0.00	0.00	0.00	0.00	0.00	0.00	0.00	0.00	0.00
MRX2	0.00	0.00	0.00	0.00	0.00	0.18	0.00	0.00	0.00	0.00	0.00	0.11	0.00	0.00	0.00	0.00	0.00	0.00	0.00	0.00	0.00
MS4A6E	0.00	0.00	0.00	0.00	0.00	0.12	0.00	0.00	0.00	0.00	0.00	0.17	0.00	0.00	0.00	0.00	0.00	0.00	0.00	0.00	0.00
SCKL2	0.00	0.00	0.00	0.00	0.00	0.12	0.00	0.00	0.00	0.00	0.00	0.12	0.00	0.00	0.00	0.00	0.00	0.00	0.00	0.00	0.00
MRX73	0.00	0.00	0.00	0.00	0.00	0.22	0.00	0.00	0.00	0.00	0.00	0.14	0.00	0.00	0.00	0.00	0.00	0.00	0.00	0.00	0.00
AD5	0.00	0.00	0.00	0.00	0.00	0.19	0.00	0.00	0.00	0.00	0.00	0.15	0.00	0.00	0.00	0.00	0.00	0.00	0.00	0.00	0.00
MRX75	0.00	0.00	0.00	0.00	0.00	0.28	0.00	0.00	0.00	0.00	0.00	0.21	0.00	0.00	0.00	0.00	0.00	0.00	0.00	0.00	0.00
ASPG3	0.00	0.00	0.00	0.00	0.00	0.00	0.00	0.00	0.00	0.00	0.00	0.15	0.13	0.00	0.00	0.00	0.00	0.00	0.00	0.00	0.00
MSD	0.00	0.00	0.00	0.00	0.00	0.00	0.00	0.00	0.00	0.00	0.00	0.16	0.14	0.00	0.00	0.00	0.00	0.00	0.00	0.00	0.00
NR3C1	0.00	0.00	0.00	0.10	0.00	0.00	0.00	0.00	0.00	0.00	0.00	0.00	0.00	0.12	0.00	0.00	0.00	0.00	0.00	0.00	0.00
CREBBP	0.00	0.00	0.00	0.00	0.00	0.00	0.00	0.10	0.00	0.00	0.00	0.00	0.00	0.10	0.00	0.00	0.00	0.00	0.00	0.00	0.00
CYP19A1	0.00	0.00	0.00	0.00	0.00	0.00	0.00	0.00	0.11	0.00	0.00	0.00	0.00	0.10	0.00	0.00	0.00	0.00	0.00	0.00	0.00
BDNF	0.00	0.00	0.00	0.00	0.00	0.00	0.00	0.00	0.11	0.00	0.00	0.00	0.00	0.11	0.00	0.00	0.00	0.00	0.00	0.00	0.00
MAPT	0.00	0.00	0.00	0.00	0.00	0.00	0.00	0.00	0.12	0.00	0.00	0.00	0.00	0.11	0.00	0.00	0.00	0.00	0.00	0.00	0.00
FGG	0.00	0.00	0.00	0.13	0.00	0.00	0.00	0.00	0.00	0.00	0.00	0.00	0.00	0.00	0.10	0.00	0.00	0.00	0.00	0.00	0.00
MYOC	0.00	0.00	0.00	0.00	0.00	0.14	0.00	0.00	0.00	0.00	0.00	0.00	0.00	0.00	0.14	0.00	0.00	0.00	0.00	0.00	0.00
AUNX1	0.00	0.00	0.00	0.00	0.00	0.14	0.00	0.00	0.00	0.00	0.00	0.00	0.00	0.00	0.13	0.00	0.00	0.00	0.00	0.00	0.00
BOS2	0.00	0.00	0.00	0.00	0.00	0.12	0.00	0.00	0.00	0.00	0.00	0.00	0.00	0.00	0.11	0.00	0.00	0.00	0.00	0.00	0.00
DFN8	0.00	0.00	0.00	0.00	0.00	0.14	0.00	0.00	0.00	0.00	0.00	0.00	0.00	0.00	0.10	0.00	0.00	0.00	0.00	0.00	0.00
SPG28	0.00	0.00	0.00	0.00	0.00	0.21	0.00	0.00	0.00	0.00	0.00	0.00	0.00	0.00	0.12	0.00	0.00	0.00	0.00	0.00	0.00
SPG27	0.00	0.00	0.00	0.00	0.00	0.16	0.00	0.00	0.00	0.00	0.00	0.00	0.00	0.00	0.13	0.00	0.00	0.00	0.00	0.00	0.00
TRESMCR	0.00	0.00	0.00	0.00	0.00	0.16	0.00	0.00	0.00	0.00	0.00	0.00	0.00	0.00	0.13	0.00	0.00	0.00	0.00	0.00	0.00
SHFM1D	0.00	0.00	0.00	0.00	0.00	0.20	0.00	0.00	0.00	0.00	0.00	0.00	0.00	0.00	0.11	0.00	0.00	0.00	0.00	0.00	0.00
COPD22	0.00	0.00	0.00	0.00	0.00	0.12	0.00	0.00	0.00	0.00	0.00	0.00	0.00	0.00	0.11	0.00	0.00	0.00	0.00	0.00	0.00
GLC1B	0.00	0.00	0.00	0.00	0.00	0.21	0.00	0.00	0.00	0.00	0.00	0.00	0.00	0.00	0.12	0.00	0.00	0.00	0.00	0.00	0.00
SRP68P1	0.00	0.00	0.00	0.00	0.00	0.16	0.00	0.00	0.00	0.00	0.00	0.00	0.00	0.00	0.13	0.00	0.00	0.00	0.00	0.00	0.00
PDB4	0.00	0.00	0.00	0.00	0.00	0.17	0.00	0.00	0.00	0.00	0.00	0.00	0.00	0.00	0.11	0.00	0.00	0.00	0.00	0.00	0.00
COPD19	0.00	0.00	0.00	0.00	0.00	0.12	0.00	0.00	0.00	0.00	0.00	0.00	0.00	0.00	0.11	0.00	0.00	0.00	0.00	0.00	0.00
COPD18	0.00	0.00	0.00	0.00	0.00	0.12	0.00	0.00	0.00	0.00	0.00	0.00	0.00	0.00	0.11	0.00	0.00	0.00	0.00	0.00	0.00
COPD17	0.00	0.00	0.00	0.00	0.00	0.12	0.00	0.00	0.00	0.00	0.00	0.00	0.00	0.00	0.11	0.00	0.00	0.00	0.00	0.00	0.00
COPD23	0.00	0.00	0.00	0.00	0.00	0.12	0.00	0.00	0.00	0.00	0.00	0.00	0.00	0.00	0.11	0.00	0.00	0.00	0.00	0.00	0.00
GER	0.00	0.00	0.00	0.00	0.00	0.19	0.00	0.00	0.00	0.00	0.00	0.00	0.00	0.00	0.12	0.00	0.00	0.00	0.00	0.00	0.00
KWE	0.00	0.00	0.00	0.00	0.00	0.16	0.00	0.00	0.00	0.00	0.00	0.00	0.00	0.00	0.11	0.00	0.00	0.00	0.00	0.00	0.00
LOC619540	0.00	0.00	0.00	0.00	0.00	0.21	0.00	0.00	0.00	0.00	0.00	0.00	0.00	0.00	0.11	0.00	0.00	0.00	0.00	0.00	0.00

DFNA47	0.00	0.00	0.00	0.00	0.00	0.16	0.00	0.00	0.00	0.00	0.00	0.00	0.00	0.00	0.11	0.00	0.00	0.00	0.00	0.00	0.00
COPD21	0.00	0.00	0.00	0.00	0.00	0.12	0.00	0.00	0.00	0.00	0.00	0.00	0.00	0.00	0.11	0.00	0.00	0.00	0.00	0.00	0.00
SPG46	0.00	0.00	0.00	0.00	0.00	0.18	0.00	0.00	0.00	0.00	0.00	0.00	0.00	0.00	0.12	0.00	0.00	0.00	0.00	0.00	0.00
RLS2	0.00	0.00	0.00	0.00	0.00	0.11	0.00	0.00	0.00	0.00	0.00	0.00	0.00	0.00	0.13	0.00	0.00	0.00	0.00	0.00	0.00
AAT2	0.00	0.00	0.00	0.00	0.00	0.18	0.00	0.00	0.00	0.00	0.00	0.00	0.00	0.00	0.10	0.00	0.00	0.00	0.00	0.00	0.00
NYS2	0.00	0.00	0.00	0.00	0.00	0.15	0.00	0.00	0.00	0.00	0.00	0.00	0.00	0.00	0.14	0.00	0.00	0.00	0.00	0.00	0.00
SCAR3	0.00	0.00	0.00	0.00	0.00	0.13	0.00	0.00	0.00	0.00	0.00	0.00	0.00	0.00	0.10	0.00	0.00	0.00	0.00	0.00	0.00
GLC1I	0.00	0.00	0.00	0.00	0.00	0.15	0.00	0.00	0.00	0.00	0.00	0.00	0.00	0.00	0.13	0.00	0.00	0.00	0.00	0.00	0.00
MAGEA9B	0.00	0.00	0.00	0.00	0.00	0.22	0.00	0.00	0.00	0.00	0.00	0.00	0.00	0.00	0.11	0.00	0.00	0.00	0.00	0.00	0.00
LOC100272228	0.00	0.00	0.00	0.00	0.00	0.22	0.00	0.00	0.00	0.00	0.00	0.00	0.00	0.00	0.11	0.00	0.00	0.00	0.00	0.00	0.00
COPD20	0.00	0.00	0.00	0.00	0.00	0.12	0.00	0.00	0.00	0.00	0.00	0.00	0.00	0.00	0.11	0.00	0.00	0.00	0.00	0.00	0.00
TBP	0.00	0.00	0.00	0.00	0.00	0.00	0.00	0.16	0.00	0.00	0.00	0.00	0.00	0.00	0.14	0.00	0.00	0.00	0.00	0.00	0.00
MYH9	0.00	0.00	0.00	0.00	0.00	0.00	0.00	0.15	0.00	0.00	0.00	0.00	0.00	0.00	0.15	0.00	0.00	0.00	0.00	0.00	0.00
SNCA	0.00	0.00	0.00	0.00	0.00	0.00	0.00	0.00	0.14	0.00	0.00	0.00	0.00	0.00	0.11	0.00	0.00	0.00	0.00	0.00	0.00
MROS	0.00	0.00	0.00	0.00	0.00	0.00	0.00	0.00	0.15	0.00	0.00	0.00	0.00	0.00	0.16	0.00	0.00	0.00	0.00	0.00	0.00
MMP3	0.00	0.00	0.00	0.00	0.00	0.00	0.00	0.00	0.11	0.00	0.00	0.00	0.00	0.00	0.10	0.00	0.00	0.00	0.00	0.00	0.00
TNFRSF1B	0.00	0.00	0.00	0.00	0.00	0.00	0.00	0.00	0.13	0.00	0.00	0.00	0.00	0.00	0.11	0.00	0.00	0.00	0.00	0.00	0.00
HSPA1B	0.00	0.00	0.00	0.00	0.00	0.00	0.00	0.00	0.13	0.00	0.00	0.00	0.00	0.00	0.10	0.00	0.00	0.00	0.00	0.00	0.00
MRST	0.00	0.00	0.00	0.00	0.00	0.00	0.00	0.00	0.00	0.00	0.00	0.15	0.00	0.00	0.15	0.00	0.00	0.00	0.00	0.00	0.00
XCE	0.00	0.00	0.00	0.00	0.00	0.00	0.00	0.00	0.00	0.00	0.00	0.13	0.00	0.00	0.11	0.00	0.00	0.00	0.00	0.00	0.00
NPPB	0.00	0.00	0.00	0.00	0.00	0.00	0.00	0.00	0.00	0.00	0.00	0.11	0.00	0.00	0.10	0.00	0.00	0.00	0.00	0.00	0.00
FGB	0.00	0.00	0.00	0.11	0.00	0.00	0.00	0.00	0.00	0.00	0.00	0.00	0.00	0.00	0.00	0.00	0.11	0.00	0.00	0.00	0.00
CIMT	0.00	0.00	0.00	0.00	0.00	0.13	0.00	0.00	0.00	0.00	0.00	0.00	0.00	0.00	0.00	0.00	0.18	0.00	0.00	0.00	0.00
LRRC63	0.00	0.00	0.00	0.00	0.00	0.13	0.00	0.00	0.00	0.00	0.00	0.00	0.00	0.00	0.00	0.00	0.18	0.00	0.00	0.00	0.00
3.8-1.2	0.00	0.00	0.00	0.00	0.00	0.11	0.00	0.00	0.00	0.00	0.00	0.00	0.00	0.00	0.00	0.00	0.11	0.00	0.00	0.00	0.00
EJM3	0.00	0.00	0.00	0.00	0.00	0.12	0.00	0.00	0.00	0.00	0.00	0.00	0.00	0.00	0.00	0.00	0.15	0.00	0.00	0.00	0.00
MGR3	0.00	0.00	0.00	0.00	0.00	0.12	0.00	0.00	0.00	0.00	0.00	0.00	0.00	0.00	0.00	0.00	0.12	0.00	0.00	0.00	0.00
TRNAA-UGC	0.00	0.00	0.00	0.00	0.00	0.12	0.00	0.00	0.00	0.00	0.00	0.00	0.00	0.00	0.00	0.00	0.11	0.00	0.00	0.00	0.00
IBD4	0.00	0.00	0.00	0.00	0.00	0.13	0.00	0.00	0.00	0.00	0.00	0.00	0.00	0.00	0.00	0.00	0.12	0.00	0.00	0.00	0.00
DPY19L3	0.00	0.00	0.00	0.00	0.00	0.11	0.00	0.00	0.00	0.00	0.00	0.00	0.00	0.00	0.00	0.00	0.13	0.00	0.00	0.00	0.00
BTBD16	0.00	0.00	0.00	0.00	0.00	0.11	0.00	0.00	0.00	0.00	0.00	0.00	0.00	0.00	0.00	0.00	0.13	0.00	0.00	0.00	0.00
KIAA0564	0.00	0.00	0.00	0.00	0.00	0.11	0.00	0.00	0.00	0.00	0.00	0.00	0.00	0.00	0.00	0.00	0.12	0.00	0.00	0.00	0.00
MYP16	0.00	0.00	0.00	0.00	0.00	0.13	0.00	0.00	0.00	0.00	0.00	0.00	0.00	0.00	0.00	0.00	0.13	0.00	0.00	0.00	0.00
OMS	0.00	0.00	0.00	0.00	0.00	0.11	0.00	0.00	0.00	0.00	0.00	0.00	0.00	0.00	0.00	0.00	0.15	0.00	0.00	0.00	0.00
PAPA3	0.00	0.00	0.00	0.00	0.00	0.13	0.00	0.00	0.00	0.00	0.00	0.00	0.00	0.00	0.00	0.00	0.10	0.00	0.00	0.00	0.00
ATFB3	0.00	0.00	0.00	0.00	0.00	0.15	0.00	0.00	0.00	0.00	0.00	0.00	0.00	0.00	0.00	0.00	0.13	0.00	0.00	0.00	0.00
DFNA41	0.00	0.00	0.00	0.00	0.00	0.17	0.00	0.00	0.00	0.00	0.00	0.00	0.00	0.00	0.00	0.00	0.11	0.00	0.00	0.00	0.00
C9ORF53	0.00	0.00	0.00	0.00	0.00	0.17	0.00	0.00	0.00	0.00	0.00	0.00	0.00	0.00	0.00	0.00	0.19	0.00	0.00	0.00	0.00
GPDS1	0.00	0.00	0.00	0.00	0.00	0.17	0.00	0.00	0.00	0.00	0.00	0.00	0.00	0.00	0.00	0.00	0.12	0.00	0.00	0.00	0.00
NEDE	0.00	0.00	0.00	0.00	0.00	0.17	0.00	0.00	0.00	0.00	0.00	0.00	0.00	0.00	0.00	0.00	0.14	0.00	0.00	0.00	0.00

PAOD1	0.00	0.00	0.00	0.00	0.00	0.16	0.00	0.00	0.00	0.00	0.00	0.00	0.00	0.00	0.00	0.00	0.28	0.00	0.00	0.00	0.00
MGR	0.00	0.00	0.00	0.00	0.00	0.16	0.00	0.00	0.00	0.00	0.00	0.00	0.00	0.00	0.00	0.00	0.10	0.00	0.00	0.00	0.00
OA10	0.00	0.00	0.00	0.00	0.00	0.14	0.00	0.00	0.00	0.00	0.00	0.00	0.00	0.00	0.00	0.00	0.10	0.00	0.00	0.00	0.00
RFH1	0.00	0.00	0.00	0.00	0.00	0.21	0.00	0.00	0.00	0.00	0.00	0.00	0.00	0.00	0.00	0.00	0.18	0.00	0.00	0.00	0.00
DFNA21	0.00	0.00	0.00	0.00	0.00	0.20	0.00	0.00	0.00	0.00	0.00	0.00	0.00	0.00	0.00	0.00	0.11	0.00	0.00	0.00	0.00
HYALP	0.00	0.00	0.00	0.00	0.00	0.18	0.00	0.00	0.00	0.00	0.00	0.00	0.00	0.00	0.00	0.00	0.11	0.00	0.00	0.00	0.00
MYP7	0.00	0.00	0.00	0.00	0.00	0.19	0.00	0.00	0.00	0.00	0.00	0.00	0.00	0.00	0.00	0.00	0.13	0.00	0.00	0.00	0.00
MYP8	0.00	0.00	0.00	0.00	0.00	0.19	0.00	0.00	0.00	0.00	0.00	0.00	0.00	0.00	0.00	0.00	0.13	0.00	0.00	0.00	0.00
RLS	0.00	0.00	0.00	0.00	0.00	0.18	0.00	0.00	0.00	0.00	0.00	0.00	0.00	0.00	0.00	0.00	0.10	0.00	0.00	0.00	0.00
MYP9	0.00	0.00	0.00	0.00	0.00	0.19	0.00	0.00	0.00	0.00	0.00	0.00	0.00	0.00	0.00	0.00	0.13	0.00	0.00	0.00	0.00
FIMG1	0.00	0.00	0.00	0.00	0.00	0.18	0.00	0.00	0.00	0.00	0.00	0.00	0.00	0.00	0.00	0.00	0.10	0.00	0.00	0.00	0.00
OTSC3	0.00	0.00	0.00	0.00	0.00	0.19	0.00	0.00	0.00	0.00	0.00	0.00	0.00	0.00	0.00	0.00	0.11	0.00	0.00	0.00	0.00
MYP10	0.00	0.00	0.00	0.00	0.00	0.19	0.00	0.00	0.00	0.00	0.00	0.00	0.00	0.00	0.00	0.00	0.13	0.00	0.00	0.00	0.00
PSORS9	0.00	0.00	0.00	0.00	0.00	0.16	0.00	0.00	0.00	0.00	0.00	0.00	0.00	0.00	0.00	0.00	0.16	0.00	0.00	0.00	0.00
HDLC2	0.00	0.00	0.00	0.00	0.00	0.16	0.00	0.00	0.00	0.00	0.00	0.00	0.00	0.00	0.00	0.00	0.24	0.00	0.00	0.00	0.00
SLC25A43	0.00	0.00	0.00	0.00	0.00	0.11	0.00	0.00	0.00	0.00	0.00	0.00	0.00	0.00	0.00	0.00	0.12	0.00	0.00	0.00	0.00
MYP12	0.00	0.00	0.00	0.00	0.00	0.14	0.00	0.00	0.00	0.00	0.00	0.00	0.00	0.00	0.00	0.00	0.11	0.00	0.00	0.00	0.00
OA25	0.00	0.00	0.00	0.00	0.00	0.12	0.00	0.00	0.00	0.00	0.00	0.00	0.00	0.00	0.00	0.00	0.11	0.00	0.00	0.00	0.00
CMM4	0.00	0.00	0.00	0.00	0.00	0.14	0.00	0.00	0.00	0.00	0.00	0.00	0.00	0.00	0.00	0.00	0.15	0.00	0.00	0.00	0.00
BP6	0.00	0.00	0.00	0.00	0.00	0.12	0.00	0.00	0.00	0.00	0.00	0.00	0.00	0.00	0.00	0.00	0.19	0.00	0.00	0.00	0.00
MCKD1	0.00	0.00	0.00	0.00	0.00	0.15	0.00	0.00	0.00	0.00	0.00	0.00	0.00	0.00	0.00	0.00	0.11	0.00	0.00	0.00	0.00
DFNA18	0.00	0.00	0.00	0.00	0.00	0.15	0.00	0.00	0.00	0.00	0.00	0.00	0.00	0.00	0.00	0.00	0.10	0.00	0.00	0.00	0.00
CHE2	0.00	0.00	0.00	0.00	0.00	0.10	0.00	0.00	0.00	0.00	0.00	0.00	0.00	0.00	0.00	0.00	0.10	0.00	0.00	0.00	0.00
OA15	0.00	0.00	0.00	0.00	0.00	0.14	0.00	0.00	0.00	0.00	0.00	0.00	0.00	0.00	0.00	0.00	0.10	0.00	0.00	0.00	0.00
OA14	0.00	0.00	0.00	0.00	0.00	0.14	0.00	0.00	0.00	0.00	0.00	0.00	0.00	0.00	0.00	0.00	0.10	0.00	0.00	0.00	0.00
FAM151A	0.00	0.00	0.00	0.00	0.00	0.15	0.00	0.00	0.00	0.00	0.00	0.00	0.00	0.00	0.00	0.00	0.11	0.00	0.00	0.00	0.00
OA11	0.00	0.00	0.00	0.00	0.00	0.14	0.00	0.00	0.00	0.00	0.00	0.00	0.00	0.00	0.00	0.00	0.10	0.00	0.00	0.00	0.00
OA13	0.00	0.00	0.00	0.00	0.00	0.14	0.00	0.00	0.00	0.00	0.00	0.00	0.00	0.00	0.00	0.00	0.10	0.00	0.00	0.00	0.00
OA12	0.00	0.00	0.00	0.00	0.00	0.14	0.00	0.00	0.00	0.00	0.00	0.00	0.00	0.00	0.00	0.00	0.10	0.00	0.00	0.00	0.00
OR12D3	0.00	0.00	0.00	0.00	0.00	0.00	0.00	0.00	0.00	0.00	0.00	0.00	0.00	0.00	0.11	0.00	0.17	0.00	0.00	0.00	0.00
OR2H1	0.00	0.00	0.00	0.00	0.00	0.00	0.00	0.00	0.00	0.00	0.00	0.00	0.00	0.00	0.11	0.00	0.17	0.00	0.00	0.00	0.00
C7ORF57	0.00	0.00	0.00	0.00	0.00	0.00	0.00	0.00	0.00	0.00	0.00	0.00	0.00	0.00	0.12	0.00	0.12	0.00	0.00	0.00	0.00
SCA9	0.00	0.00	0.00	0.00	0.00	0.00	0.00	0.00	0.00	0.00	0.00	0.00	0.00	0.00	0.13	0.00	0.11	0.00	0.00	0.00	0.00
CRYAA	0.00	0.00	0.00	0.00	0.00	0.00	0.00	0.00	0.00	0.00	0.00	0.00	0.00	0.00	0.10	0.00	0.00	0.17	0.00	0.00	0.00
GSTO2	0.00	0.00	0.00	0.00	0.00	0.13	0.00	0.00	0.00	0.00	0.00	0.00	0.00	0.00	0.00	0.00	0.00	0.00	0.18	0.00	0.00
TLCD2	0.00	0.00	0.00	0.00	0.00	0.16	0.00	0.00	0.00	0.00	0.00	0.00	0.00	0.00	0.00	0.00	0.00	0.00	0.20	0.00	0.00
NAT2	0.00	0.00	0.00	0.00	0.00	0.00	0.00	0.00	0.11	0.00	0.00	0.00	0.00	0.00	0.00	0.00	0.00	0.00	0.12	0.00	0.00
LOC647275	0.00	0.00	0.00	0.00	0.00	0.00	0.00	0.00	0.17	0.00	0.00	0.00	0.00	0.00	0.00	0.00	0.00	0.00	0.17	0.00	0.00
CYP2C19	0.00	0.00	0.00	0.00	0.00	0.00	0.00	0.00	0.15	0.00	0.00	0.00	0.00	0.00	0.00	0.00	0.00	0.00	0.13	0.00	0.00
CYP2D6	0.00	0.00	0.00	0.00	0.00	0.00	0.00	0.00	0.15	0.00	0.00	0.00	0.00	0.00	0.00	0.00	0.00	0.00	0.13	0.00	0.00

NQO1	0.00	0.00	0.00	0.00	0.00	0.00	0.00	0.00	0.00	0.00	0.00	0.00	0.00	0.10	0.00	0.00	0.00	0.00	0.11	0.00	0.00
MTHFD1	0.00	0.00	0.00	0.00	0.00	0.00	0.00	0.00	0.00	0.00	0.00	0.00	0.00	0.00	0.11	0.00	0.00	0.00	0.26	0.00	0.00
CBS	0.00	0.00	0.00	0.00	0.00	0.00	0.00	0.00	0.00	0.00	0.00	0.00	0.00	0.00	0.12	0.00	0.00	0.00	0.25	0.00	0.00
IGFBP3	0.00	0.00	0.00	0.00	0.00	0.00	0.00	0.00	0.11	0.00	0.00	0.00	0.00	0.00	0.00	0.00	0.00	0.00	0.00	0.11	0.00
IDDM6	0.00	0.00	0.00	0.00	0.00	0.00	0.00	0.00	0.00	0.00	0.00	0.00	0.00	0.00	0.00	0.00	0.35	0.00	0.00	0.16	0.00
CCDC39	0.00	0.00	0.00	0.00	0.00	0.22	0.00	0.00	0.00	0.00	0.00	0.00	0.00	0.00	0.00	0.00	0.00	0.00	0.00	0.00	0.12
RNR1	0.00	0.00	0.00	0.00	0.00	0.12	0.00	0.00	0.00	0.00	0.00	0.00	0.00	0.00	0.00	0.00	0.00	0.00	0.00	0.00	0.14
MRPS18CP2	0.00	0.00	0.00	0.00	0.00	0.27	0.00	0.00	0.00	0.00	0.00	0.00	0.00	0.00	0.00	0.00	0.00	0.00	0.00	0.00	0.22
TRNS1	0.00	0.00	0.00	0.00	0.00	0.12	0.00	0.00	0.00	0.00	0.00	0.00	0.00	0.00	0.00	0.00	0.00	0.00	0.00	0.00	0.21
MUTYH	0.00	0.00	0.00	0.00	0.00	0.10	0.00	0.00	0.00	0.00	0.00	0.00	0.00	0.00	0.00	0.00	0.00	0.00	0.00	0.00	0.11
OPA5	0.00	0.00	0.00	0.00	0.00	0.13	0.00	0.00	0.00	0.00	0.00	0.00	0.00	0.00	0.00	0.00	0.00	0.00	0.00	0.00	0.34
